# IDP–CRF: Intrinsically Disordered Protein/Region Identification Based on Conditional Random Fields

**DOI:** 10.3390/ijms19092483

**Published:** 2018-08-22

**Authors:** Yumeng Liu, Xiaolong Wang, Bin Liu

**Affiliations:** School of Computer Science and Technology, Harbin Institute of Technology Shenzhen Graduate School, Shenzhen 518055, Guangdong, China; ymliu.hitsz@gmail.com (Y.L.); wangxl@insun.hit.edu.cn (X.W.)

**Keywords:** intrinsically disordered proteins/regions, conditional random fields (CRFs), PSSMs, kmer, secondary structure, relative solvent accessibility

## Abstract

Accurate prediction of intrinsically disordered proteins/regions is one of the most important tasks in bioinformatics, and some computational predictors have been proposed to solve this problem. How to efficiently incorporate the sequence-order effect is critical for constructing an accurate predictor because disordered region distributions show global sequence patterns. In order to capture these sequence patterns, several sequence labelling models have been applied to this field, such as conditional random fields (CRFs). However, these methods suffer from certain disadvantages. In this study, we proposed a new computational predictor called IDP–CRF, which is trained on an updated benchmark dataset based on the MobiDB database and the DisProt database, and incorporates more comprehensive sequence-based features, including PSSMs (position-specific scoring matrices), kmer, predicted secondary structures, and relative solvent accessibilities. Experimental results on the benchmark dataset and two independent datasets show that IDP–CRF outperforms 25 existing state-of-the-art methods in this field, demonstrating that IDP–CRF is a very useful tool for identifying IDPs/IDRs (intrinsically disordered proteins/regions). We anticipate that IDP–CRF will facilitate the development of protein sequence analysis.

## 1. Introduction

Intrinsically disordered proteins/regions (IDPs/IDRs) refer to the proteins/regions without a stable three-dimensional structure in their native state [1]. IDPs/IDRs are widely distributed in nature, and are correlated with many biological functions [2,3] and a broad range of human diseases, such as genetic diseases [4], cancer [3] and neurodegenerative diseases [5,6]. Therefore, accurately identifying IDPs/IDRs is crucial for understanding the mechanism of biological functions and exploring the relationship between IDPs/IDRs and diseases.

There are several databases containing experimentally determined IDPs/IDRs. For example, PDB [7] contains a large number of IDPs/IDRs annotated by X-ray crystallography (X-ray), and these IDPs/IDRs are organized by the MobiDB database [8,9]. DisProt [2] archives experimentally certified IDPs/IDRs by different techniques, such as X-ray crystallography, nuclear magnetic resonance (NMR) and circular dichroism (CD) spectroscopy. However, identifying IDPs/IDRs by using experimental methods is time consuming and expensive. Therefore, fast and efficient computational methods are urgently needed.

Existing computational predictors can be divided into four categories according to different strategies [1]: (1) physicochemical-based methods that directly utilize the physical principles to discriminate IDPs/IDRs [10,11]; (2) machine learning-based methods that are constructed based on machine learning algorithms, including classification models [12] and sequence labeling models [13,14]; (3) template-based methods that search for homologous proteins with known structures; (4) meta-methods that integrate various predictors into one prediction model [15]. For more information of these methods, please refer to the recent review paper [1].

In machine learning-based methods, different from the sequence labeling models, the classification models treat each amino acid residue as a separate sample, ignoring the interdependency between labels of sequence-adjacent residues [16,17]. However, sequence-adjacent residues may have similar characters in forming IDPs/IDRs [18], and the disordered residues tend to be neighbors in the sequence of a protein. In order to incorporate this information, several sequence labelling methods have been proposed. For example, OnD–CRF [14] is based on conditional random fields (CRFs) [19], and SPOT-disorder [13] is based on a bidirectional long short-term memory (BLSTM) model incorporating long-range interactions between amino acid residues. Both the two methods have made important contributions to the development of this very important field. However, they have several shortcomings: (1) inaccurate representation of proteins. The OnD–CRF is only based on several sequence-based features, which fail to capture the characteristics of disordered regions; (2) high computational cost. The computational cost of the SPOT-disorder model is high, preventing its applications to analyze large-scale datasets; (3) un-catching up. Both the two methods were trained and tested on small benchmark datasets. As a result, their generalization ability and performance are limited, and an updated benchmark dataset is highly required.

In order to overcome these disadvantages, in this study, we combine CRFs and various sequence-based features [20,21] to further improve the predictive performance, including PSSMs (position-specific scoring matrices), kmer, predicted secondary structure and relative solvent accessibility, and a predictor called IDP–CRF is proposed. Furthermore, the IDP–CRF is trained on a comprehensive and updated benchmark dataset constructed based on the MobiDB database [8,9,22] and the latest version of the DisProt_v7.0 database [2,23]. Tested on two widely used independent datasets, experimental results show that IDP–CRF achieves better or at least comparable predictive performance with 25 currently existing state-of-the-art methods in this field. IDP–CRF would be a useful tool for protein sequence analysis.

## 2. Results and Discussion

### 2.1. The Influence of Different Ratios of Positive and Negative Samples on the Performance of Various Predictors

**Figure 1:**
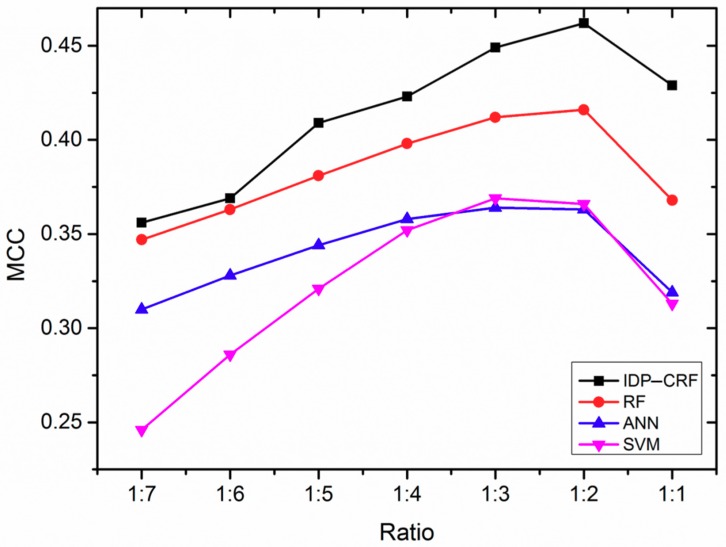
The performance of IDP–CRF (intrinsically disordered protein–conditional random field) and three classification-based predictors trained with different ratios of disordered residues and ordered residues. These three classification-based predictors include a RF (random forest) predictor, an ANN (artificial neural network) predictor and an SVM (support vector machine) predictor. MCC represents Matthew’s correlation coefficient performance metrics.

In a training dataset, the imbalanced number of ordered residues and disordered residues would impact the performance of the computational predictors [24,25]. Therefore, we analyze the effect of different ratios of positive and negative samples on the performance of IDP–CRF. For comparison purposes, three classification-based predictors are constructed as well, which are based on support vector machine (SVM), artificial neural network (ANN) and random forest (RF) models. A series of training datasets are constructed by randomly removing a different number of ordered residues. By using five-fold cross-validation, the Matthew’s correlation coefficient (MCC) changing curves of IDP–CRF and these three classification-based predictors at different ratios of disordered residues and ordered residues in training are shown in Figure 1. From Figure 1, we can see that IDP–CRF outperforms other predictors, and different predictors can achieve the best performance when the ratio of positive and negative samples is around 1:2. The reason is that IDP–CRF can capture the interdependency between labels of sequence-adjacent residues, and therefore, the global sequence patterns of disordered regions can be incorporated into IDP–CRF.

### 2.2. IDP–CRF (Intrinsically Disordered Protein–Conditional Random Field) Outperforms Classification-Based Predictors

Sequential adjacent residues may have similar characteristics in the formation of IDPs/IDRs [18]. However, traditional classification-based predictors treat each target residue as an independent sample, ignoring the global sequence patterns of disordered regions. To address this problem, IDP–CRF, proposed in this study, can take the relationship between labels of sequential adjacent residues into account. The performance of IDP–CRF and several classification-based predictors (cf. Section 3.1) is compared by using five-fold cross-validation, and is shown in Table 1. From Table 1, we can see that IDP–CRF obtains the highest accuracy (ACC). When the positive and negative samples are extremely unbalanced, although ACC favors “greedy” predictions (i.e., predicting more residues as disordered), IDP–CRF obtains the highest sensitivity (Sn) and specificity (Sp), indicating that IDP–CRF can achieve better trade-off between Sn and Sp automatically. Besides, the highest MCC of IDP–CRF also fully illustrates that it is an efficient predictor for identifying IDPs/IDRs. This is because IDP–CRF can obtain more information of global sequence patterns of disordered regions compared with classification-based predictors.

### 2.3. Several Examples Predicted by IDP–CRF and Three Classification-Based Predictors

In this section, three examples are used to visualize the prediction of the four predictors listed in Table 1, including IDP–CRF, RF, SVM and ANN. These proteins are 3H2YA, 2ODKA and 4AD4A, and their structure information is acquired from the PDB database [7]. To visualize the 3D structures of these proteins, PyMOL [26] software is adopted to generate 3D structures of ordered regions. For those disordered regions, their 3D structure is drawn manually.

**Figure 2:**
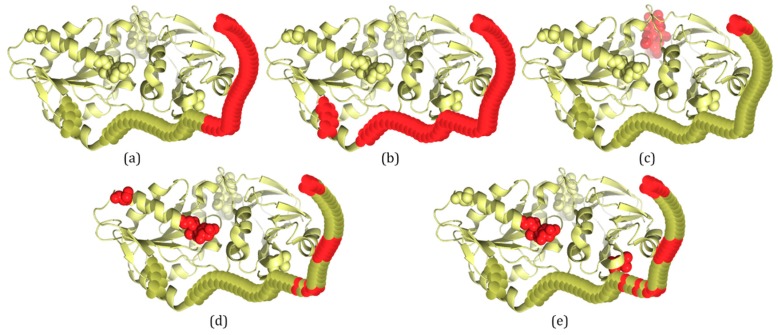
A schematic view of protein 3H2YA with IDRs predicted by IDP–CRF and three classification-based predictors, where the residues with red color represent disordered residues and those with yellow color represent ordered residues. (**a**) IDRs predicted by IDP–CRF are: (1, 32). (**b**) Actual IDRs are: (1, 55) and (199, 202). (**c**) IDRs predicted by the RF predictor are: (1, 3) and (365, 368). (**d**) IDRs predicted by the SVM predictors are: (1, 3), (16, 21), (28, 29), (31, 31), (170, 172) and (185, 185). (**e**) IDRs predicted by the ANN predictor are: (1, 4), (16, 21), (28, 29), (31, 31), (34, 34), (170, 171) and (314, 314). These curly braces represent the position intervals of the IDRs in the protein.

For PDB proteins/regions, structures are solved by X-ray crystallography, and missing electron densities are IDPs/IDRs. According to the definition of IDRs, 3H2YA has two IDRs with a total of 59 disordered residues (Figure 2b). For 3H2YA, 32 disordered residues are correctly predicted by IDP–CRF (TP (true positive): 32, FP (false positive): 0, TN (true negative): 309 and FN (false negative): 27) (Figure 2a), three disordered residues are correctly predicted by the RF predictor (TP: 3, FP: 4, TN: 305 and FN: 56) (Figure 2c), 12 disordered residues are correctly predicted by the SVM predictor (TP: 12, FP: 4, TN: 305 and FN: 47) (Figure 2d), and 14 disordered residues are correctly predicted by the ANN predictor (TP: 14, FP: 3, TN: 306 and FN: 45) (Figure 2e).

**Figure 3:**
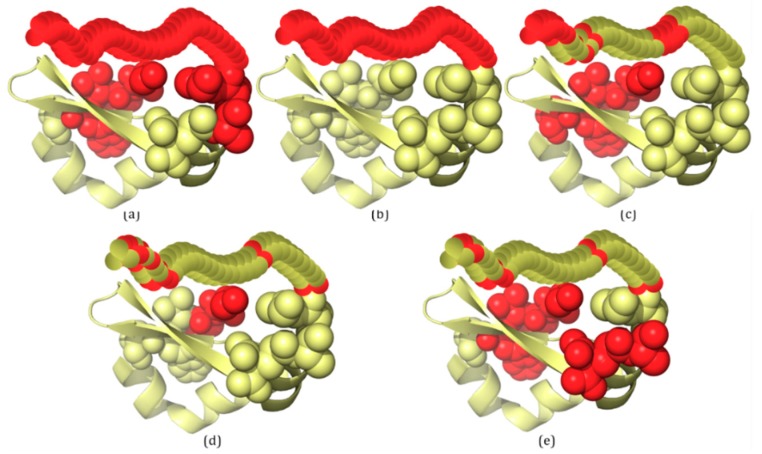
A schematic view of protein 2ODKA with IDRs predicted by IDP–CRF and three classification-based predictors, where the residues with red color represent disordered residues and those with yellow color represent ordered residues. (**a**) IDRs predicted by IDP–CRF are: (1, 4) and (49, 85). (**b**) Actual IDR is: (52, 85). (**c**) IDRs predicted by the RF predictor are: (1, 5), (62, 65), (75, 75), (77, 77) and (81, 85). (**d**) IDRs predicted by the SVM predictors are: (1, 2), (52, 52), (62, 62), (77, 78), (81, 81) and (83, 84). (**e**) IDRs predicted by the ANN predictor are: (1, 4), (48, 49), (52, 52), (62, 62), (77, 78) and (83, 84). These curly braces represent the position intervals of the IDRs in the protein.

The second example, 2ODKA, contains one IDR with 34 disordered residues (Figure 3b). For 2ODKA, 34 disordered residues are correctly predicted by IDP–CRF (TP: 34, FP: 7, TN: 44 and FN: 0) (Figure 3a), 11 disordered residues are correctly predicted by the RF predictor (TP: 11, FP: 5, TN: 46 and FN: 23) (Figure 3c), seven disordered residues are correctly predicted by the SVM predictor (TP: 7, FP: 2, TN: 49 and FN: 27) (Figure 3d), and six disordered residues are correctly predicted by the ANN predictor (TP: 6, FP: 6, TN: 45 and FN: 28) (Figure 3e).

**Figure 4:**
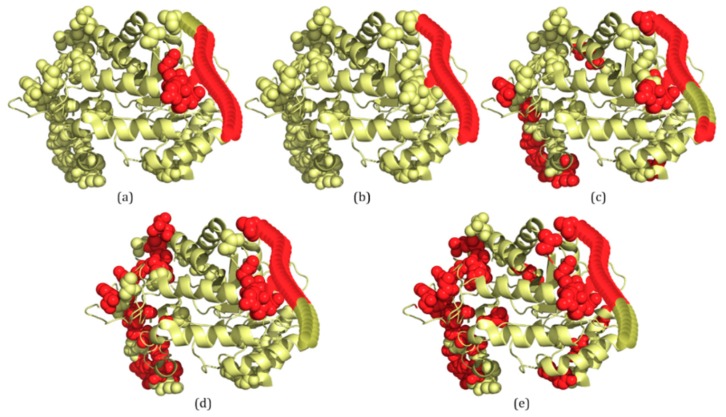
A schematic view of protein 4AD4A with IDRs predicted by IDP–CRF and three classification-based predictors, where the residues with red color represent disordered residues and those with yellow color represent ordered residues. (**a**) IDRs predicted by IDP–CRF are: (1, 26) and (376, 380). (**b**) Actual IDRs are: (1, 30), (380, 380). (**c**) IDRs predicted by the RF predictor are: (1, 3), (13, 31), (69, 81), (132, 133), (236, 236), (346, 346) and (377, 380). (**d**) IDRs predicted by the SVM predictors are: (11, 31), (54, 55), (65, 69), (72, 75), (78, 82), (88, 88), (97, 99), (104, 104), (337, 337), (346, 346) and (376, 380). (**e**) IDRs predicted by the ANN predictor are: (10, 31), (33, 33), (53, 54), (65, 66), (68, 68), (72, 76), (78, 82), (86, 86), (88, 88), (97, 99), (129, 129), (201, 201), (207, 207), (260, 260), (337, 338), (342, 342), (344, 344), (346, 347) and (376, 380). These curly braces represent the position intervals of the IDRs in the protein.

The last example is 4AD4A, which contains two IDRs with a total of 31 disordered residues (Figure 4b). For 4AD4A, IDP–CRF predicts 27 disordered residues correctly (TP: 27, FP: 4, TN: 345 and FN: 4) (Figure 4a), the RF predictor predicts 22 disordered residues correctly (TP: 22, FP: 22, TN: 327 and FN: 9) (Figure 4c), the SVM predictor predicts 21 disordered residues correctly (TP: 21, FP: 28, TN: 321 and FN: 10) (Figure 4d), and the ANN predictor predicts 22 disordered residues correctly (TP: 22, FP: 36, TN: 313 and FN: 9) (Figure 4e).

From these figures, we can see that within the scope of actual IDRs, disordered residues predicted by IDP–CRF are continuous, while those predicted by the classification-based predictors are discontinuous; and within the scope of ordered regions, the number of FPs predicted by IDP–CRF is obviously less than that predicted by the classification-based predictors.

### 2.4. Comparison with Other Related Predictors

Two widely used independent datasets (MxD494 and SL329) are used to further evaluate the performance of the proposed method and other related predictors. The performance of these predictors is shown in Table 2 and Table 3 respectively. From these two tables, we can see that IDP–CRF shows better or at least comparable predictive performance with 25 currently existing state-of-the-art methods in this field. In particular, IDP–CRF outperforms the existing CRF-based predictor OnD–CRF [14] because IDP–CRF adopts more comprehensive sequence-based features to represent proteins. Besides, according to Table 2, IDP–CRF shows comparable performance with the state-of-the-art meta-predictor MFDp [15], and outperforms all the other related methods. According to Table 3, the performance of IDP–CRF is highly comparable with that of SPOT-disorder [13], and outperforms all the other related methods. The predictive results show that IDP–CRF achieves state-of-the-art performance.

## 3. Materials and Method

### 3.1. Benchmark Dataset

As discussed in previous studies [45,46,47,48,49], a reliable benchmark dataset is crucial to the construction of an accurate predictor [50]. In this study, we construct a comprehensive and updated benchmark dataset S based on the MobiDB database [8,9,22] and the DisProt_v7.0 database [2,23]. S can be represented as

(1) $$S=S_{1}\cup S_{2},$$

where $S_{1}$ contains 4590 proteins from the MobiDB database, whose structures are solved by X-ray crystallography, and those proteins/regions with missing electron densities are IDPs/IDRs; and $S_{2}$ contains 683 proteins from the DisProt database. The proteins in $S_{1}$ are selected from 24,669 proteins by the following criteria: (a) resolution ≤2Å, (b) length ≥30 residues, (c) contains at least one IDR. DisProt_v7.0 includes both confident and ambiguous annotations for IDPs/IDRs. In this study, all the proteins with confident annotations are selected, and then merged with the selected proteins from the MobiDB database. Furthermore, the redundant proteins in the merged dataset are removed by using the Blastclust algorithm [51] with similarity 25%. Finally, 5273 proteins are left and used for 5-fold cross-validation. The detailed sequences in the benchmark dataset S are given in Supplementary Materials.

### 3.2. Benchmark Independent Datasets

To make a comprehensive comparison with more different methods, two benchmark independent datasets MxD494 [15,40] and SL329 [13,52] are selected as independent test datasets. In order to fairly test our method on these two independent datasets, two training datasets are constructed by removing the overlaps between our constructed benchmark dataset and these two independent test datasets by using the Blastclust algorithm [51] with 25% sequence identity cutoff.

### 3.3. Features

Feature extraction is a key step for constructing a predictor [53,54,55,56,57]. The construction of IDP–CRF is based on transition and state features. In this study, four different state features are used, including PSSMs, kmer, secondary structure and relative solvent accessibility. In addition, all the classification-based predictors shown in this article are based on these four features.

#### 3.3.1. Transition Feature

The transition feature depends on the current position and the previous position of the label sequence. Suppose the label set for residues is ∅={O, D}, where O represents ordered residue and D represents disordered residue. Transition feature is defined as [19]:

(2) $$t_{y,y^{\prime }\;}\left(y_{i-1},y_{i},x,i\right)=\{\begin{array}{l} 1\;\;\mathrm{if}\;y_{i-1}=y\;\mathrm{and}\;y_{i}=y^{\prime }\\ 0\;\;\mathrm{otherwise} \end{array}$$

where $y_{i-1}$ and $y_{i}$ ($y,\;y^{\prime }\epsilon \;\emptyset$) represent the labels of residues at position *i* − 1 and *i* in the protein sequence x, respectively.

#### 3.3.2. PSSMs (Position-Specific Scoring Matrices)

Due to its ability to capture the important evolutionary information, PSSM features are considered as one of the most important and essential features in a number of previous bioinformatics studies [58,59,60,61,62,63,64,65]. In this study, the PSSMs are obtained by running three iterations of PSI-BLAST [51] searching against the nrdb90 database [66] under condition E-value = 0.001, and the other parameters of PSI-BLAST are set as default. Then, PSSMs are normalized to [0, 1] by the following equation [67]:

(3) $$\mathrm{norm}\left(x\right)=\{\begin{array}{ll} 0.0 & \mathrm{if}\;x\leq -5\\ 0.5+0.1x & \mathrm{if}-5<x<5\\ 1.0 & \mathrm{if}\;x\geq 5 \end{array}$$

For each target residue, its PSSM feature is constructed based on the 11 nearest sequential adjacent residues centered on the target residue. Therefore, for each residue, the dimension of the PSSM feature is 20×11=220.

#### 3.3.3. Kmer

Kmer [68,69] is the most direct representation of protein sequence, which is defined as the occurrence frequencies of *k* neighboring amino acids. In this study, for each target residue, the kmer (*k* is set to 1) feature is calculated in the window of 11 nearest sequential adjacent residues centered to the target residue. Therefore, for each residue, the dimension of the kmer feature is 20.

#### 3.3.4. Secondary Structure

Secondary structure features are effective in protein structure prediction [70,71]. The PSIPRED version 4.01 package [72] includes two approaches to predict secondary structure of proteins; one is a profile-based method and the other is a sequence-based method. In this study, the profile-based PSIPRED is adopted to predict secondary structure for each target residue among three types of structures (i.e., helix, beta strand and coil). However, when a protein has no homologous sequences after searching against the nrdb90 database [66], the sequence-based PSIPRED is adopted. For each target residue, the dimension of secondary structure feature is one.

#### 3.3.5. Relative Solvent Accessibility

Previous studies have indicated that incorporating the predicted solvent accessibility information is useful for improving the prediction of protein functional sites [73,74,75,76]. In this study, Sable version 2 package [77,78] is adopted to generate relative solvent accessibility information for each target residue, and the dimension of this feature is one for each target residue. The parameters of Sable are set as: SA_ACTION = SVR, SA_OUT = RELATIVE and other parameters are set as default.

### 3.4. Conditional Random Fields

Conditional random fields (CRFs) were proposed by Lafferty et al. [19], and compose a probabilistic model for labeling sequence data. Due to their advantages, CRFs have been widely applied to solve a number of prediction tasks in the field of bioinformatics and computational biology, including protein-protein interaction prediction [79,80], phosphorylation site prediction [81], transcription factor binding site prediction [82], and protein-RNA residue-based contact prediction [83].

In this study, the identification of IDPs/IDRs is solved as a sequence labeling task by using CRFs, in which proteins are observation sequences, then each amino acid residue is annotated as disordered or ordered. Given some protein sequences represented as X and their label sequences represented as Y, then these data are used to train a conditional probability model P(Y|X), which is finally used to label unlabeled protein sequences. In general, CRFs employ the simplest first-order chain structure. Therefore, given an unlabeled observation sequence *x*, the conditional probability of its label sequence *y* has the following form [19]:

(4) $$P\left(y|x\right)=\frac{1}{Z\left(x\right)}\exp\left(\sum_{i,k}\lambda_{k}t_{k}\left(y_{i-1},y_{i},x,i\right)+\sum_{i,l}\mu_{l}s_{l}\left(y_{i},x,i\right)\right)$$

where Z(x) is a normalization factor, $t_{k}\left(y_{i-1},y_{i},x,i\right)$ is a transition feature function of the observation sequence *x* and the labels at position *i* − 1 and *i*, and the transition feature is defined as Equation (2) in this study. $s_{l}\left(y_{i},x,i\right)$ is a state feature function of observation sequence *x* and the label at position *i*. In this study, state features include PSSMs, kmer, predicted secondary structures and relative solvent accessibility. The index *k* of $t_{k}$ and the index *l* of $s_{l}$ is the number of different features. $\lambda_{k}$ and $\mu_{l}$ are the weights of $t_{k}\left(y_{i-1},y_{i},x,i\right)$ and $s_{l}\left(y_{i},x,i\right)$, respectively.

### 3.5. Implementations

FlexCRF [84] is an implementation of CRF, which was modified to be able to handle real value features as described by Li et al. [85]. In this study, the modified FlexCRF is adopted, and the first-order Markov CRF is used. The parameter num_iterations is optimized from 30 to 60 with an increment of 10, and the optimal value is 50. The parameter init_lambda_val is optimized from 0.05 to 0.1 with an increment of 0.05, and the optimal value is 0.05. Scikit-learn [86] version 0.19.1 is used for the implementations of random forest (RF) and artificial neural network (ANN). For the RF predictor, the parameter n_estimators is optimized from 100 to 1000 with an increment of 100 and the optimal value is 500. For the ANN predictor, its structure includes an input layer, a hidden layer, and an output layer. The parameter hidden_layer_sizes is optimized from 20 to 80 with an increment of 10, and the optimal value is 40. In order to handle large-scale datasets, LIBLINEAR [87] is adopted for the implementation of support vector machine (SVM). For the SVM predictor, the parameter *c* is optimized in the range of $2^{i}$, where *i* is an integer and i∈[−5, 5], and the optimal value is $2^{-4}$. The other parameters of each algorithm are set as default.

### 3.6. Criteria for Performance Evaluation

In this study, sensitivity (Sn) and specificity (Sp) are adopted, which measure the performance of each class in binary prediction. In the datasets of IDPs/IDRs, the positive and negative samples are unbalanced, and the number of ordered residues is far more than that of disordered residues. Therefore, we choose another two metrics, balanced accuracy (ACC) and Matthew’s correlation coefficient (MCC) [88,89], to measure the performance of different methods. These metrics are defined as follows:

(5) $$\left\{ \begin{array}{c} \mathrm{Sn}=\frac{\mathrm{TP}}{\mathrm{TP}+\mathrm{FN}}\\ \mathrm{Sp}=\frac{\mathrm{TN}}{\mathrm{TN}+\mathrm{FP}}\\ \mathrm{ACC}=\frac{1}{2}\left(\frac{\mathrm{TP}}{\mathrm{TP}+\mathrm{FN}}+\frac{\mathrm{TN}}{\mathrm{TN}+\mathrm{FP}}\right)\\ \mathrm{MCC}=\frac{\left(\mathrm{TP}\times \mathrm{TN}\right)-\left(\mathrm{FP}\times \mathrm{FN}\right)}{\sqrt{\left(\mathrm{TP}+\mathrm{FP}\right)\left(\mathrm{TP}+\mathrm{FN}\right)\left(\mathrm{TN}+\mathrm{FP}\right)\left(\mathrm{TN}+\mathrm{FN}\right)}} \end{array}\right.$$

where TP (true positive) and FP (false positive) represent the number of corrected and misclassified predicted disordered residues, respectively; TN (true negative) and FN (false negative) represent the number of corrected and misclassified predicted ordered residues, respectively.

## 4. Conclusions

In this study, we propose a new computational method called IDP–CRF combining various sequence-based features and conditional random fields (CRFs) to predict IDPs/IDRs. Furthermore, this predictor is trained on an updated benchmark dataset. Experimental results show that IDP–CRF performs better than, or at least highly comparable to, 25 existing state-of-the-art methods in this field. The good performance of IDP–CRF can be attributed to its following three advantages. (1) IDP–CRF is trained on a more reliable benchmark dataset, which is the currently most comprehensive benchmark dataset constructed in this paper; (2) combining CRFs enables IDP–CRF to contain the relationship between labels of sequential adjacent residues, and therefore, the global sequence patterns of disordered region distributions are incorporated; (3) IDP–CRF improves the previous CRF-based predictor by incorporating more comprehensive sequence-based features. In our future studies, we will focus on exploring new machine learning algorithms to further improve the accuracy of prediction of IDPs/IDRs [90,91,92,93].

## Figures and Tables

**Table 1:** Performance comparison of IDP–CRF (intrinsically disordered protein–conditional random field) and three classification-based predictors by using 5-fold cross-validation.

Methods	Ratio ^a^	Sn ^b^	Sp ^c^	ACC ^d^	MCC ^e^
IDP–CRF	1:2	0.637	0.910	0.774	0.462
RF	1:2	0.524	0.928	0.726	0.416
SVM	1:2	0.543	0.896	0.720	0.366
ANN	1:2	0.537	0.897	0.717	0.363

^a^ Represents the ratio of disordered residues and ordered residues in training dataset. ^b^ Represents sensitivity. ^c^ Represents specificity. ^d^ Represents balanced accuracy. ^e^ Represents Matthew’s correlation coefficient.

**Table 2:** The performance comparison of different predictors on independent dataset MxD494.

Predictor ^a^	Sn	Sp	ACC	MCC	Rank	
					ACC	MCC
IDP–CRF	0.680	0.821	0.750	0.460	2	1
MFDp [15]	0.746	0.768	0.757	0.451	1	2
MD [27]	0.673	0.813	0.743	0.444	3	3
PONDR-FIT [28]	0.631	0.821	0.726	0.419	6	4
DISOPRED2 [29]	0.647	0.800	0.724	0.406	7	5
IUPred-long [30]	0.581	0.841	0.711	0.405	8	6
PONDR VSL2B [31]	0.774	0.698	0.736	0.401	4	7
OnD–CRF ^b^ [14]	0.752	0.711	0.732	0.396	5	8
IUPred-short [30]	0.522	0.866	0.694	0.389	10	9
RONN [32]	0.664	0.754	0.709	0.368	9	10
NORSnet [33]	0.532	0.829	0.681	0.347	11	11
DisEMBL-R [34]	0.316	0.936	0.626	0.323	15	12
DISpro [35,36]	0.303	0.940	0.622	0.318	16	13
Ucon [37]	0.554	0.787	0.671	0.313	12	14
Spritz [38]	0.494	0.812	0.653	0.293	14	15
FoldIndex [10]	0.602	0.717	0.660	0.278	13	16
DisEMBL-H [34]	0.435	0.792	0.614	0.216	17	17
PROFbval [39]	0.835	0.387	0.611	0.196	18	18
GlobPlot [11]	0.353	0.826	0.590	0.182	19	19
DisEMBL-C [34]	0.760	0.414	0.587	0.150	20	20

^a^ The results of the 18 compared predictors (MFDp, MD, PONDR-FIT, DISOPRED2, IUPred-long, PONDR VSL2B, IUPred-short, RONN, NORSnet, DisEMBL-R, DISpro, Ucon, Spritz, FoldIndex, DisEMBL-H, PROFbval, GlobPlot, DisEMBL-C) are obtained from [40]. ^b^ The results of OnD–CRF are acquired from web-server.

**Table 3:** The performance comparison of different predictors on independent dataset SL329.

Predictor ^a^	Sn	Sp	ACC	MCC	Rank	
					ACC	MCC
IDP–CRF	0.75	0.88	0.817	0.64	1	2
SPOT-disorder [13]	0.67	0.96	0.815	0.67	2	1
SPINE-D [12]	0.78	0.85	0.815	0.63	2	3
DISOPRED3 [41]	-	-	0.795	0.61	4	4
DISOPRED2 [29]	0.69	0.90	0.795	0.59	4	5
OnD–CRF ^b^ [14]	0.79	0.80	0.793	0.58	6	6
MD [27]	0.66	0.89	0.775	0.58	7	6
PONDR-FIT [28]	0.61	0.91	0.760	0.55	8	8
IUPred-long [30]	0.60	0.92	0.760	0.55	8	8
MFDp [15]	0.88	0.62	0.750	0.51	11	10
DISOClust [42]	0.81	0.70	0.755	0.51	10	10
NORSnet [33]	0.54	0.92	0.730	0.51	12	10
IUPred-short [30]	0.50	0.94	0.720	0.50	13	13
Ucon [37]	0.59	0.81	0.700	0.42	14	14
DisEMBL [34]	-	-	0.660	0.40	16	15
Dispro [35,36]	0.28	0.99	0.635	0.40	18	15
PONDR VL-XT [43]	0.59	0.78	0.685	0.38	15	17
Espritz [44]	-	-	0.605	0.35	19	18
PROFbval [39]	-	-	0.648	0.30	17	19

^a^ The results of the 17 compared predictors (SPOT-disorder, SPINE-D, DISOPRED3, DISOPRED2, MD, PONDR-FIT, IUPred-long, MFDp, DISOClust, NORSnet, IUPred-short, Ucon, DisEMBL, Dispro, PONDR VL-XT, Espritz, PROFbval) are obtained from [12,13]. ^b^ The results of OnD–CRF are acquired from web-server.

## Supplementary Materials

Supplementary Materials are available online at http://www.mdpi.com/1422-0067/19/9/2483/s1.

## Abbreviations

IDP	intrinsically disordered protein
IDR	intrinsically disordered region
CRFs	conditional random fields
SVM	support vector machine
ANN	artificial neural network
RF	random forest
BLSTM	bidirectional long short-term memory
PSSM	position-specific scoring matrix
X-Ray	X-ray crystallography
NMR	nuclear magnetic resonance
CD	circular dichroism
TP	true positive
FP	false positive
TN	true negative
FN	false negative
Sn	sensitivity
Sp	specificity
ACC	balanced accuracy
MCC	Matthew’s correlation coefficient
