# Supplementary material for: IDP–CRF: Intrinsically Disordered Protein/Region Identification Based on Conditional Random Fields
Source: Int J Mol Sci. 2018 Aug 22;19(9):2483. doi: 10.3390/ijms19092483 (PMC6164615; doi:10.3390/ijms19092483)
Supplement: Supplementary file 1 [file ijms-339037-SI.pdf › ijms-339037-SI.pdf]

Online Supporting Information S1. The benchmark dataset $\mathbb{S}$ contains 5273 protein sequences, in which 4590 proteins are acquired from MobiDB database and 683 sequences are acquired from DisProt_v7.0 database. The label sequences are composed of '1' and '0', where '1' represents disordered residue and '0' represents ordered residue.

>A7IGZ0_4dyv_0_249
MSKTGKKIAIVTGAGSGVGRAVAVALAGAGYGVALAGRRLDALQETAAEIGDDALCVPTD
VTDPDSVRALFTATVEKFGRVDVLFNNAGTGAPAIPMEDLTFAQWKQVVDTNLTGPFLCT
QEAFRVMKAQEPRGGRIINNGSISATSPRPYSAPYTATKHAITGLTKSTSLDGRVHDIACGQI
DIGNADTPMAQKMKAGVPQADLSIKVEPVMDVAHVASAVVYMASLPLDANVQFMTIMA
TKMPLIGRG
11111000000000000000000000000000000000000000000000000000000000000000000000000000000
00000000000000000000000000000000000000000000000000000000000000000000000000000000000
00000000000000000000001111111111111111111111110000000000000000000000000000001111111
1
>Q39KL8_3nxl_1_464
SESRRDGTPRVTRMQVIPVAGRDSMLLNLCGAHAPYFTRNLVILDDSSGHTGVGEVPGGEG
IRHALERMTDLVVGQSIGRYQATLNAVRAALSGAGPGAGRTIRHEVTSAGEAAVLRQPHEI
NLRLDNVITAIEAALLDLLGQHLDVPVAALLGEGQQRDAVPMLAYLFYIGDRGRTDLPYRD
EAQARTPWFRLRNEEALTPAAIARQAEAAVDRYGFADFKLKGGVMAGADEMEAIAAIKA
CFPDARATLDPNGAWSLDEAVALCRGQGHLLAYAEDPCGPEGGYSGREVMAEFRRATGIP
TATNMIATDWRQMDHAVRLQAVDIPLADPHFWTMQGSVRLAQLCRDWGLTWGSHSNN
HFDVSLAMFTHAAAAAPGTITAIDTHWIWQEGDARLTREPLKIVGGQVAVPERPGLGIELD
MAQVEAAHALYKEVGGTARDDAVAMRYLVPGWTYDPKRPSFGRG
11111100000000000000000000000000000000000000000000000000000000000000000000000000000
00000000001111111111111111111111111111111000000000000000000000000000000000000000000
00000000000000000000000000000000000000000000000000000000000000000000000000000000000
00000000000000000000000000000000000000000000000000000000000000000000000000000000000
00000000000000000000000000000000000000000000000000000000000000000000000000000000000
0000000000000000000000000000000000000000000000011
>Q9ZVQ3_1e6b_0_220
MANSGEEKLKLYSYWRSSCAHRVRIALALKGLDYEYIPVNLLKGDQFDSDFKKINPMGTVP
ALVDGDVVINDSFAIIMYLDEKYPEPPLLPRDLHKRAVNYQAMSIVLSGIQPHQNLAVIRYIE
EKINVEEKTAWVNNAITKGFTALEKLLVNCAGKHATGDEIYLADLFLAPQIHGAINRFQIN
MEPYPTLAKCYESYNELPAFQNALPEKQPDAPSSTI
11111110000000000000000000000000000000000000000000000000000000000000000000000000000
00000000000000000000000000000011111111111111111110000000000000000000000000000000000
0000000000000000000000000000000000000000000000000000001
>O06414_1q52_0_313
MVAPAGEQGRSSTALSDNPFDAKAWRLVDGFDDLTDITYHRHVDDATVRVAFNRPEVRN
AFRPHTVDELYRVLDHARMSPDVGVVLLTGNGPSPKDGGWAFCSGGDQRIRGRSGYQYAS
GDTADTVDVARAGRLHILEVQRLIRFMPKVVICLVNGWAAGGGHSLHVVCDLTLASREYA
RFKQTDADVGSFDGGYGSAYLARQVGQKFAREIFFLGRTYTAEQMHQMGAVNAVAEHAE
LETVGLQWAAEINAKSPQAQRMLKFAFNLLDDGLVGQQLFAGEATRLAYMTDEAVEGRD
AFLQKRPPDWSPFPRYF
11111111111111111000000000000000000000000000000000000000000000000000000000000000000
00000000000000000000000111111111111111111111111111100000000000000000000000000000000
00000000000000000000000000000000000000000000000000000000000000000000000000000000000
00000000000000000000000000000000000000000000000000000000000000000
>Q9M7R2_1unf_7_244
NVAGINLLFKEGPKVNAKFELKPPPYPLNGLEPVMSQQTLEFHWGKHHRTYVENLKKQV
VGTELDGKSLEEIIVTAYNKGDILPAFNNAAQVWNHDFFWECMKPGGGGKPSGELLELIER

DFGSFEKFLDEFKAAAATQFGSGWAWLAYKASKLDGENAANPPSADEDNKLVVIKSPNAV
NPLVWGGYYPLLTIDVWEHAYYLDFQNRRPDYISVFMDKLVSWDAVSSRLEQAKALSA
11111111111110000000000000000000000000000000000000000000001100000000000000000000000
00000000000000000000000000000000000000000000000000000000000000000000000011111111100
000000000000000000000000000000000000000000000000000000000000000000000000
>Q3L181_3v0s_0_336
MPRVKLGTQGLEVSKLGFGCMGLSGDYNDALPEEQGIAVIKEAFNCGITFFDTSDIYGENGS
NEELLGKALKQLPREKIQVGTKFGIHEIGFSGVKAKGTPDYVRSCCEASLKRLDVDYIDLFYI
HRIDTTVPIEITMGELKKLVEEGKIKYVGLSEASPDTIRRAHAVHPVTALQIEYSLWTRDIEDE
IVPLCRQLGIGIVPYSPIGRGLFAGKAIKESLPENSVLTSHPRFVGENLEKNKQIYYRIEALSQK
HGCTPVQLALAWVLHQGEDVVPIPGTTKIKNLHNNVGALKVKLTKEDLKEISDAVPLDEV
AGESIHEVIAVTNWKFANTPPLK
00000000000000000000000011111110000000000000000000000000000000000000000000000000000
00000000000000000000000000000000000000000000000000000000000000000000000000000000000
00000000000000000000000000000000000000000000000000000111111111111111111111111100000
00000000000000000000000000000000000000000000000000000000000001111111111111111100000
00001
>Q4WHY9_2c3b_0_204
MSQVFFDVEYAPVGTAETKVGRIVFNLFDKDVPKTAKNFRELCKRPAGEGYRESTFHRIIPN
FMIQGGDFTRGNGTGGRSIYGDKFADENFSRKHDKKGILSMANAGPNTNGSQFFITTAVTS
WLDGKHVVFGEVADEKSYSVVKEIEALGSSSGSVRSNTRPKIVNCGEL
00000000000000000000000000000000000000000000000000000000000000000000001111111111111
11111110000000000000011111110000000000000000000000000000000000000000000000000000000
00000
>P01751_1a6v_19_138
QVQLQQPGAELVKPGASVKLSCKASGYTFTSYWMHWVKQRPGRGLEWIGRIDPNSGGTK
YNEKFKSKATLTVDKPSSTAYMQLSSLTSEDSAVYYCARYDYYGSSYFDYWGQGTTLTVSS
00000000000000000000000000000000000000000000000000000000000000000000000000000000000
0000000000000000000000000000000000001
>Q9RZ32_3i4j_0_429
MSNVFYRSSKPYPVAVRGEGVFLYDDAGRRYLDGSSGALVANIGHGRAEVGERMAAQAAR
LPFVHGSQFSSDVLEEYAGRLARFVGLPTFRFWAVSGGSEATESAVKLARQYHVERGEPGRF
KVITRVPSYHGASLGSLAASGMGARRELYTPLMRPEAWPKLPKPDPARNGAEDAEGLRAL
LEREGPETVAAFMAEPVVGASDAALAPAPGYYERVRDICDEAGIIFIADEVMSGMGRCGSP
LALSRWSGVTPDIAVLGKGLAAGYAPLAGLLAAPQVYETVMGGSGAFMHGFTYAGHPVS
VAAGLSVLDIVEREDLTGAAKERGAQLLAGLQALQARFPQMMQVRGTGLLLGVVLGDLA
TGQAFETPGIASRIGAAALKRGLITYPGSGAEPNGRGDHLLLGPPLSITAAEVDELLALLAG
ALEDVLG
11000000000000000000000000000000000000000000000000000000000000000000000000000000000
00000000000000000000000000000000000000000000000111111111111111110000000000000000000
00000000000000000000000000000000000000000000000000000000000000000000000000000000000
00000000000000000000000000000000000000000000000000000000000000000000000000000000000
00000000000000000000000000001111111111000000000000000001111111111000000000000000000
000000000000001
>P00593_4bp2_15_144
QAGLNSRALWQFNGMIKCKIPSSEPLLDFNNYGCYCGLGGSGTPVDDLDRCCQTHDNCY
KQAKKLDSCKVLVDNPYTNNYSYSCSNNEITCSSENNACEAFICNCDRNAAICFSKVPYNK
EHKNLDKKNC
11111100000000000000000000000000000000000000000000000000000000000000000111111000000
00000000000000000000000000000000000000000000000
>P0A616_2i1u_0_115

MTDSEKSATIKVTDASFATDVLSSNKPVLVDFWATWCGPCKMVAPVLEEIATERATDLTVAK
LDVDTNPETARNFQVVSIPTLILFKDGQPVKRIVGAKGKAALLRELSDVVPNLN
11111100000000000000000000000000000000000000000000000000000000000000000000000000000
000000000000000000000000000000011
>P00808_1w7f_0_306
MKLWFSTLKLKKAAAVLLFSCVALAGCANNQTNASQPAEKNEKTEMKDDFAKLEEQFDA
KLGIFALDTGTNRTVAYRPDERFAFASTIKALTVGVLLQQKSIEDLNQRITYTRDDLVNYNPI
TEKHVDTGMTLKELADASLRYSDNAAQNLILKQIGGPESLKKELRKIGDEVTNPERFEPEL
NEVNPGETQDTSTARALVTSLRAFALEDKLPSEKRELLIDWMKRNTTGDALIRAGVPDGWE
VADKTGAASYGTRNDIAIIWPPKGDPVVLAVLSSRDKKDAKYDDKLIAEATKVVMKALNM
NGK
11111111111111111111111111111111111111111111111000000000000000000000000000000000000
00000000000000000000000000000000000000000000000000000000000000000000000000000000000
00000000000000000000000000000000000000000000000000000000000000000000000000000000000
0000000000000000000000000000000000000000000000000000001111
>Q06141_1uv0_26_174
EEPQRELPSARIRCPKGSKAYGSHCYALFLSPKSWTDADLACQKRPSGNLVSVLSGAEGSFV
SSLVKSIGNSYSYVWIGLHDPTQGTEPNGEGWEWSSSDVMNYFAWERNPSTISSPGHCASLS
RSTAFLRWKDYNCNVRLPYVCKFTD
11111111100000000000000000000000000000000000000000000000000000000000000000000000000
000001100000000000000000000000000000000000000000000000000000000000
>Q7D0E8_2r8w_0_301
MATRFKGLSAFPITPADEAGRVDIEAFSALIARLDAAEVDSVGILGSTGIYMYLTREERRRAIE
AAATILRGRRTLMAGIGALRTDEAVALAKDAEAAGADALLLAPVSYTPLTQEEAYHHFAA
VAGATALPLAIYNNPTTTRFTFSDELLVRLAYIPNIRAIKMPLPADADYAGELARLRPKLSDD
FAIGYSGDWGCTDATLAGGDTWYSVVAGLLPVPALQLMRAAQAGNAEEAKRLDATFQPL
WALFKEFGSIRVIYAAANILSLTVSEPPRPILPLTSAERQRVEEALEALSALETAP
00000000000000000000000000000000000000000000000000000000000000000000000000000000000
00000000000000000000000000000000000000000000000000000000000000000000000000000000000
00000000000000000000000000000000000000000000000000000000000000000000000000000000000
00000000000000000000000000000000000000000000000111111
>A0PZS2_3g14_0_191
MEFYEVIKKRKSIKKFEQTAIDRDKLLKIIDMAMRAPSWKNKTPYKFIVVESDKLKLDIANAI
ENKTSAASEAVLNSPMTIVAVANPEESGDVSGKEIYLIDTAIAMEHIVLGATDEGYGTCWIA
AFNENKIKEALKIPDNLRVVALTPLGVPKDSAEDEPHHPKKDMDEYLYIDKWGTSFMESNV
KILEKN
00000000000000000000000000000000000000000000000000000000000000000000000000000000000
00000000000000000000000000000000000000000000000000000000000000000000000001111111000
00000000000000000000111111
>O57946_3ath_0_427
MHEDVQLNIYVHSQEGIGMEENIKSMLGDVERFFSKKALEMRASEVRELLKLVETSDIISLA
GGLPNPKTFPKEIIRDILVEIMEKYADKALQYGTTKGFTPLRETLMKWLGKRYGISQDNDIMI
TSGSQQALDLIGRVFLNPGDIVVVEAPTYLAALQAFNFYEPQYIQIPLDDEGMKVEILEEKL
KELKSQGKKVKVVYTVPTFQNPAGVTMNEDRRKYLLELASEYDFIVVEDDPYGELRYSGNP
EKKIKALDNEGRVIYLGTFSKILAPGFRIGWMVGDPGIIRKMEIAKQSTDLCTNVFGQVVAW
RYVDGGYLEKHIPEIRKFYKPRRDAMLEALEEFMPEGVKWTKPEGGMFIWVTLPDGIDSKK
MLERAIKKGVAYVPGEAFYAHRDVKNTMRLNFTYVDEDKIMEGIKRLAETIKEELKA
11111111111111111111111100000000000000000000000000000000000000000000000000000000000
00000000000000000000000000000000000000000000000000000000000000000000000000000000000
00000000000000000000000000000000000000000000000000000000000000000000000000000000000
00000000000000000000000000000000000000000000000000000000000000000000000000000000000

0000000000000000000000000000000000000000000000000000000000000000000000000000000000
0000000000000
>O87605_2cd8_0_415
MRRTQQGTTASPPVLDLGALGQDFAADPYPTYARLRAEGPAHRVRTPEGDEVWLVVGYD
RARAVLADPRFSKDWRNSTTPLTEAEAALNHNMLESDPPRHTRLRKLVAREFTMRRVELLR
PRVQEIVDGLVDAMLAAPDGRADLMESLAWPLPITVISELLGVPEPDRAAFRVWTDAFVFP
DDPAQAQTAMAEMSGYLSRLIDSKRGQDGEDLLSALVRTSDEDGSRLTSEELLGMAHILLV
AGHETTVNLIANGMYALLSHPDQLAALRADMTLLDGAVEEMLRYEGPVESATYRFPVEPV
DLDGTVIPAGDTVLVVLADAHRTPERFPDPHRFDIRRDTAGHLAFGHGIHFCIGAPLARLE
ARIAVRALLERCPDLALDVSPGELVWYPNPMIRGLKALPIRWRRGREAGRRTG
1111111111111000000000000000000000000000000000000000000000000000000000000000000000
0000000000000000000000000000000000000000000000000000000000000000000000000000000000
0000000000000000000000000000000000000000000000000000000000000000000000000000000000
0000000000000000000000000000000000000000000000000000000000000000000000000000000000
0000000000000000000000000000000000000000000000000000000000000000000000000111111111
1
>Q97YM2_2h6e_5_348
MVKSKAALLKKFSEPLSIEDVNIPEPQGEEVLIRIGGAGVCRTDLRVWKGVEAKQGFRLPIIL
GHENAGTIVEVGELAKVKKGDNVVVYATWGDLTCRYCREGKFNICKNQIIPGQTTNGGFS
EYMLVKSSRWLVKLNSLSPVEAAPLADAGTTSMGAIRQALPFISKFAEPVVIVNGIGGLAVY
TIQILKALMKNITIVGISRSKKHRDFALELGADYVSEMKDAESLINKLTDGLGASIAIDLVGTE
ETTYNLGKLLAQEGAIILVGMEGKRVSLEAFDTAVWNKKLLGSNYGSLNDLEDVVRLSESG
KIKPYIIKVPLDDINKAFTNLDEGRVDGRQVITP
0000000000000111110000000000000000000000000000000000000000000000000000000000000000
0000000000000000000000000000000000000000000000000000000000000000000000000000000000
0000000000000000000000000000000000000000000000000000000000000000000000000000000000
0000000000000000000000000000000000000000000000000000000000000000000000011111111111
111110000000
>O75970_2fcf_1147_1242
QPRRVELWREPSKSLGISIVGGRGMGSRLSNGEVMRGIFIKHVLEDSPAGKNGTLKPGDRIV
EVDGMDLRDASHEQAVEAIRKAGNPVVFMVQSII
0000000000010000000000011111111111110000000000000000000000000000000000000000000000
0000000000000
>P13792_1mvo_0_134
MNKKILVVDDEESIVTLLQYNLERSGYDVITASDGEEALKKAETEKPDLIVLDVMLPKLDGI
EVCKQLRQQKLMFPILMLTAKDEEFDKVLGLELGADDYMTKPFSPREVNARVKAILRRSEI
AAPSSEMKNDEM
0000000000000000000000000000000000000000000000000000000000000000000000000000000000
0000000000000000000000000000000000000011111111111111
>Q2IR47_3sg0_0_382
MQQTKTLIVALATMLAGVTAAQAEIKIGITMSASGPGAALGQPQSKTVAALPKEIGGEKVT
YFALDDESDPTKAAQNARKLLSEEKVDVLIGSSLTPVSLPLIDIAAEAKTPLMTMAAAAILV
APMDERRKWVYKVVPNDDIMAEAIGKYIAKTGAKKVGYIGFSDAYGEGYYKVLAAAAPK
LGFELTTHEVYARSDASVTGQVLKIIATKPDAVFIASAGTPAVLPQKALRERGFKGAIYQTHG
VATEEFIKLGGKDVEGAIFAGEAFSGAEDMPADSPFRKVKARFVDAYKAANGGAAPTIFGV
HLWDSMTLVENAIPAALKAAKPGTPEFRAAIRDQIEKSKDLALNNGLSNMTPDNHNGYD
ERSAFLIEIRDGAFRLKQ
1111111111111111111110000000000000000000000000000000000000000000000000000000000000
0000000000000000000000000000000000000000000000000000000000000000000000000000000000
0000000000000000000000000000000000000000000000000000000000000000000000000000000000
0000000000000000000000000000000000000000000000000000000000000000000000000000000000

00000000000000000000000000000000000000000000000000001
>O59346_2hoq_0_240
MVKVIFFDLDDTLVDTSKLAEIARKNAIENMIRHGLPVDFETAYSELIELIKEYGSNFPYHFD
YLLRRLDLPYNPKWISAGVIAYHNTKFAYLREVPGARKVLIRLKELGYELGIITDGNPVKQW
EKILRLELDDFFEHVIISDFEGVKKPHPKIFKKALKAFNVKPEEALMVGDRLYSDIYGAKRVG
MKTVWFRYGKHSERELEYRKYADYEIDNLESLLEVLARESSSNKKVHPPRQQI
00000000000000000000000000000000000000000000000000000000000000000000000000000000000
00000000000000000000000000000000000000000000000000000000000000000000000000000000000
000000000000000000000000000000000000000000000000000000000000000000000001111
>P07998_1dza_28_155
KESRAKKFQRQHMDSDSSPSSSSTYCNQMMRRRNMTQGRCKPVNTFVHEPLVDVQNVCF
QEKVTCKNGQGNCYKSNSSMHITDCRLTNGSRYPNCAYRTSPKERHIIVACEGSPYVPVHF
DASVEDST
00000000000000000011110000000000000000000000000000000000000000000000000000000000000
0000000000000000000000000000000000000000011
>Q56R04_4i9b_1_503
ANRNVPIPRRQLYIGGEWREPVKKNRIPIINPATEEIIGDIPAATAEDVDIAVEAARKAIARDD
WGSTTGAQRAKYLRAIAAKVLEKKSVLATLESLDSGKTLYESAADMDDVAGCFEYYAGLA
EALDSRRMTPVNLNSDSYKSYVLREPLGVVGLITPWNYPLLMAIWKVAPALAAGCAAILKP
SELASITCLELGEICREIGLPSGALNILTGLGPEAGGPLASHPHVDKISFTGSGPTGSKIMTAA
AQLVKPVSLELGGKSPIVVFDDIDNLDIAAEWTLFGIFANTGQVCSATSRLIVQENIASAFMD
RLLKWTKNIKISDPLEEDCKLGPVVSAGQYEKVLKFISNAKSEGATILCGGERPQHLKKGYY
VQPTIITDVNTSMEIWKEEVFGPVLCVKTFKTEEQAIELANDTKYGLGAAVMSKDVKRCER
FTKAFQTGIIWINCSQPTFNELPWGGKKRSGFGRDLGKWGLENFLNIKQVTEYTSAEPLAFY
KSPSKN
11110000000000000000000000000000000000000000000000000000000000000000000000000000000
00000000000000000000000000000000000000000000000000000000000000000000000000000000000
00000000000000000000000000000000000000000000000000000000000000000000000000000000000
00000000000000000000000000000000000000000000000000000000000000000000000000000000000
00000000000000000000000000000000000000000000000000000000000000000000000000000000000
00000000000000000000000000000000000000000000000000000000000000000000000000000000000
00111
>C6CRV0_3ro8_517_850
APLKDVYKNDFLIGNAISAEDLEGTRLELLKMHHDVVTAGNAMKPDALQPTKGNFTFTA
ADAMIDKVLAEGMKMHGHVLVWHQQSPAWLNTKKDDNNNTVPLGRDEALDNLRTHI
QTVMKHFGNKVISWDVVNEAMNDNPSNPADYKASLRQTPWYQAIGSDYVEQAFLAARE
VLDENPSWNIKLYYNDYNEDNQNKATAIYNMVKDINDRYAAAHNGKLLIDGVGMQGHY
NINTNPDNVKLSLEKFISLGVEVSVSELDVTAGNNYTLPENLAVGQAYLYAQLFKLYKEHA
DHIARVTFWGMDDNTSWRAENNPLLFDKNLQAKPAYYGVIDP
00000000000000000000000000000000000000000000000000000000000000000000000000000000000
00000000000000000000000000000000000000000000000000000000000000000000000000000000000
00000000000000000000000000000000000000000000000000000000000000000000000000000000000
00000000000000000000000000000000000000000000000000001111111111111111111111111111111
11
>O87989_2q1t_0_356
MPVIMNASKLANTNVMVVGGAGFVGSNLVKRLLELGVNQVHVVDNLLSAEKINVPDHP
AVRFSETSITDDALLASLQDEYDYVFHLATYHGNQSSIHDPLADHENNTLTTLKLYERLKHF
KRLKKVVYSAAGCSIAEKTFDDAKATEETDIVSLHNNDSPYSMSKIFGEFYSVYYHKQHQLP
TVRARFQNVYGPGEILGAGRWRGTPATVWRNVTPTFIYKALKGMPLPLENGGVATRDFIFV
EDVANGLIACAADGTPGGVYNIASGKETSIADLATKINEITGNNTELDRLPKRPWDNSGKR
FGSPEKARRELGFSADVSIDDGLRKTIEWTKANLAVIEQIMRKHDSALATYGK

11111000000000000000000000000000000000000000000000000000000000000000000000000000
00000000000000000000000000000000000000000000000111111111111000000000000000000000
00000000000000000000000000000000000000000000000000000000000000000000000000000000
00000000000000000000000000000000000000000000000001111000000000000000000000000000
000000000000000000000011
>P55144_3qup_484_799
LDSLGISDELKEKLEDVLIPEQQFTLGRMLGKGEFGSVREAQLKQEDGSFVKVAVKMLKADI
IASSDIEEFLREAACMKEFDHPHVAKLVGVSLRSRAKGRLPIPMVILPFMKHGDLHAFLLAS
RIGENPFNLPLQTLVRFMVDIACGMEYLSSRNFIHRDLAARNCMLAEDMTVCVADFGLSR
KIYSGDYYRQGCASKLPVKWLALESLADNLYTVHSDVWAFGVTMWEIMTRGQTPYAGIEN
AEIYNYLIGGNRLKQPPECMEEVYDLMYQCWSADPKQRPSFTCLRMELENILGHLSVLSTS
QDPLYINIERA
11111111111111000000111000000000000000000001111110000000011111100000000000000000000
00000000000001111110000000000000000000000011100000000000000000000000000000000000
00000000000000001111111111111111000000000000000000000000000000000000000000000000
0000000000000000000000000000000000000000000000011111111111111111
>Q963I9_2rh7_0_232
MDLAKLGLKEVMPTKINLEGLVGDHAFSMEGVGEGNILEGTQEVKISVTKGAPLPFAFDIV
SVAFSYGNRAYTGYPEEISDYFLQSFPEGFTYERNIRYQDGGTAIVKSDISLEDGKFIVNVDFK
AKDLRRMGPVMQQDIVGMQPSYESMYTNVTSVIGECIIAFKLQTGKHFTYHMRTVYKSKK
PVETMPLYHFIQHRLVKTNVDTASGYVVQHETAIAAHSTIKKIEGSLP
11111100000000000000000000000000000000000000000000000000000000000000000000000000
00000000000000000000000000000010000000000000000000000000000000000000000000000000
0000000000000000000000000000000000000111110000000000000001111111
>Q59452_1z9n_22_198
HGDHMHNHDTKMDTMSKDMMSMEKIVVPVQQLDPQNGNKDVGTVEITESAYGLVFTPK
LHDLAHGLHGFHIHEKPSCEPKEKDGKLVAGLGAGGHWDPKQTQKHGYPWSDDAHMG
DLPALFVMHDGSATTPVLAPRLKKLAEVKGHSLMIHAGGDNHSDHPAPLGGGGPRMAC
GVIK
11111111111111111111110000000000000000000000000000000000000000000000000000000000
00000000000000000000000000000000000000000000000000000000000000000000000000000000
00000000000
>Q59976_1gnx_0_478
MVPAAQQTATAPDAALTFPEGFLWGSATASYQIEGAAAEDGRTPSIWDTYARTPGRVRNG
DTGDVATDHYHRWREDVALMAELGLGAYRFSLAWPRIQPTGRGPALQKGLDFYRRLADEL
LAKGIQPVATLYHWDLPQELENPGGWPERPTAERFAEYAAIAADALGDRVKTWTTLNEPW
CSAFLGYGSGVHAPGRTDPVAALRAAHHLNLGHGLAVQALRDRLPADAQCSVTLNIHHV
RPLTDSEADADAVRRIDALANRVFTGPMLQGAYPEDLVKDTAGLTDWSFVRDGDLRLAHQ
KLDFLGVNYYSPTLVSEADGSGTHNSDGHGRSAHSPWPGADRVAFHQPPGETTAMGWAV
DPSGLYELLRRLSSDFPALPLVITENGAAFHDYADPEGNVNDPERIAYVRDHLAAVHRAIKD
GSDVRGYFLWSLLDNFEWAHGYSKRFGAVYVDYPTGTRIPKASARWYAEVARTGVLPTA
11111111111111000000000000000000000000000000000000000000000000000000000000000000
00000000000000000000000000000000000000000000000000000000000000000000000000000000
00000000000000000000000000000000000000000000000000000000000000000000000000000000
00000000000000000000000000000000000000000000000000000000000000001111111111111110
00000000000000000000000000000000000000000000000000000000000000000000000000000000
00000000000000000000000000000000000000000000000000000000000000
>Q45670_1dbi_121_400
WTPNDTYYQGYQYGPQNTYTDYAWDVTKGSSGQEIAVIDTGVDYTHPDLDGKVIKGYDF
VDNDYDPMDLNNHGTHVAGIAAAETNNATGIAGMAPNTRILAVRALDRNGSGTLSDIAD
AIIYAADSGAEVINLSLGCDCHTTTLENAVNYAWNKGSVVVAAAGNNGSSTTFEPASYENV

IAVGAVDQYDRLASFSNYGTWVDVVAPGVDIVSTITGNRYAYMSGTSMASPHVAGLAALL
ASQGRNNIEIRQAIEQTADKISGTGTYFKYGRINSYNAVTY
00000000000000000000000000000000000000000000000000000000000000000000000000000000000
00000000000000000000000000000000000000000000000000000000000000000000000000000000001
11111111000000000000000000000000000000000000000000000000000000000000000000000000000
0000000000000000000000000000000
>Q00499_2bgv_20_153
QEGDAAKGEKEFNKCKACHMVQAPDGTDIVKGGKTGPNLYGVVGRKIASVEGFKYGDGI
LEVAEKNPDMVWSEADLIEYVTDPKPWLVEKTGDSAAKTKMTFKLGKNQADVVAFLAQH
SPDAGAEAAPAEGAAN
10000000000000000000000000000000000000000000000000000000000000000000000000000000000
000000000000000000000000000000000000001111111111111
>A4JL30_4f32_0_429
MTLRVVVTGIGIVSPLGCGKELVWQRLIGGGSGLRRLGDDIAGELSAKVGGTVQDVAEDPE
GGFDPERSVPHKELRKMDRFIQMAMVAADEALAEAGWAPEAEQQRERTATVVASGIGGFP
GLAEAVRIGETRGVRRLSPFTIPFFLSNLAAGQISIKHRFRGPLGCPVTACAASVQAIGDAMR
MIRTGEADVVLAGGAEAAFDKVSLGGFAAARALSTGFSEEPVRASRPFDRDRDGFVMGEG
AAMVVVESLDHALARGARPIAEIIGYGTTADAYHMTAGPDDGSGAMRAMKLALRMGDV
APEQVDYVNAHATSTPVGDAGEIEALKTVFGVGAGPAISSTKSATGHLLGAAGAIEAAFSIL
ALRDGVLPGTLNLEHPDPAADGLDLIGPAARHVPVEIALSNGFGFGGVNASVLFRRYPSDR
SAEAH
00000000000000000000000000000000000000000000000000000000000000000000000000000000000
00000000000000000000000000000000000000000000000000000000000000000000000000000000000
00000000000000000000000000000000000000000000000000000000000000000000000000000000000
00000000000000000000000000000000000000000000000000000000000000000000000000000000000
00000000000000000000000000000000000000000000000000000000000000000000000000000000000
000000011111111
>Q84II3_1j1i_0_283
MLNKAEQISEKSERAFVERFVNAGGVETRYLEAGKGQPVILIHGGGAGAESEGNWRNVIPI
LARHYRVIAMDMLGFGKTAKPDIEYTQDRRIRHLHDFIKAMNFDGKVSIVGNSMGGATGL
GVSVLHSELVNALVLMGSAGLVVEIHEDLRPIINYDFTREGMVHLVKALTNDGFKIDDAMI
NSRYTYATDEATRKAYVATMQWIREQGGLFYDPEFIRKVQVPTLVVQGKDDKVVPVETAYK
FLDLIDDSWGYIIPHCGHWAMIEHPEDFANATLSFLSLRVD
11111111111111000000000000000000000000000000000000000000000000000000000000000000000
00000000000000000000000000000000000000000000000000000000000000111111111100000000000
00000000000000000000000000000000000000000000000000000000000000000000000000000000000
00000000000000000000000000000000011
>Q6N089_4hpy_19_246
EVQLVESGGGLVQPGRSLRLSCAASGFTFDDYAMHWVRQAPGKGLEWVSGISWNSGSIAY
ADSVKGRFTISRDNGKNSLYLQMNSLRAEDTALYYCAKAHNFYYYGMDVWGQGTTVTVS
SASTKGPSVFPLAPSSKSTSGGTAALGCLVKDYFPEPVTVSWNSGALTSGVHTFPAVLQSSGL
YSLSSVVTVPSSSLGTQTYICNVNHKPSNTKVDKRVEPKSCDK
10000000000000000000000000000000000000000000000000000000000000000000000000000000000
00000000000000000000000000000000000000000000000000000000000000000000000000000000000
00000000000000000000000000000000000000000000000000000011111
>P00306_3brp_0_161
MKTPITEAIAAADNQGRFLSNTELQAVNGRYQRAAASLEAARSLTSNAERLINGAAQAVYS
KFPYTSQMPGPQYASSAVGKAKCARDIGYYLRMVTYCLVVGGTGPMDEYLIAGLEEINRTF
DLSPSWYVEALNYIKANHGLSGQAANEANTYIDYAINALS
10000000000000000000000000000000000000000000000000000000000000000000000000000000000
0000000000000000000000000000000000000000000000000000000000000000000000000000011

>P52759_1qah_1_135
SSIIRKVISTSKAPAAIGAYSQAVLVDRTIYVSGQIGMDPSSGQLVPGGVAEEAKQALKNLGEI
LKAAGCDFTNVVKTTVLLADINDFGTVNEIYKTYFQGNLPARAAYQVAALPKGSRIEIEAIA
VQGPFTTAG
00000000000000000000000000000000000000000000000000000000000000000000000000000000000
0000000000000000000000000000000000000000000000000111
>Q9FEW9_2hs8_0_395
MASSAQDGNNPLFSPYKMGKFNLSHRVVLAPMTRCRALNNIPQAALGEYYEQRATAGGF
LITEGTMISPTSAGFPHVPGIFTKEQVREWKKIVDVVHAKGAVIFCQLWHVGRASHEVYQP
AGAAPISSTEKPISNRWRILMPDGTHGIYPKPRAIGTYEISQVVEDYRRSALNAIEAGFDGIEI
HGAHGYLIDQFLKDGINDRTDEYGGSLANRCKFITQVVQAVVSAIGADRVGVRVSPAIDHL
DAMDSNPLSLGLAVVERLNKIQLHSGSKLAYLHVTQPRYVAYGQTEAGRLGSEEEEARLMR
TLRNAYQGTFICSGGYTRELGIEAVAQGDADLVSYGRLFISNPDLVMRIKLNAPLNKYNRKT
FYTQDPVVGYTDYPFLQGNGSNGPLSRL
11111100000000000000000000000000000000000000000000000000000000000000000000000000000
00000000000000000000000000000000000000000000000000000000000000000000000000000000000
00000000000000000000000000000000000000000000000000000000000000000000000000000000000
00000000000000000000000000000000001111111111100000000000000000000000000000000000000
000000000000000000000000000000000000000000000000000111111111111
>Q10399_3ht5_0_367
MTSGSLQFTVLRAVNPATDAQRESMLREPGFGKYHTDHMVSIDYAEGRGWHNARVIPYGP
IELDPSAIVLHYAQEVFEGLKAYRWADGSIVSFRADANAARLRSSARRLAIPELPDAVFIESLR
QLIAVDKAWVPGAGGEEALYLRPFIFATEPGLGVRPATQYRYLLIASPAGAYFKGGIAPVSV
WVSTEYVRACPGGTGAAKFGGNYAASLLAQAEAAENGCDQVVWLDAVERRYIEEMGGM
NIFFVLGSGGSARLVTPELSGSLLPGITRDSLLQLAIDAGFAVEERRIDIDEWQKKAAAGEITE
VFACGTAAVITPVARVRHGASEFRIADGQPGEVTMALRDTLTGIQRGTFADTHGWMARLG
11111111111111111111111111111111100000000000000000000000000000000000000000000000000
00000000000000000000000000000000000000000000000000000000000000000000000000000000000
00000000000000000000000000000000000000000000000000000000000000000000000000000000000
00000000000000000000000000000000000000000000000000000000000000000000000000000000000
00000000000000000000000000000000000
>B2RM04_3fi9_1_332
SYLTEEKLTIVGAAGMIGSNMAQTAAMMRLTPNLCLYDPFAVGLEGVAEEIRHCGFEGLNL
TFTSDIKEALTDAKYIVSSGGAPRKEGMTREDLLKGNAEIAAQLGKDIKSYCPDCKHVIIIFN
PADITGLVTLIYSGLKPSQVTTLAGLDSTRLQSELAKHFGIKQSLVTNTRTYGGHGEQMAVF
ASTAKVNGTPLTDLIGTDKLTNEQWAELKQRVVKGGANIIKLRGRSSFQSPSYVSIEMIRAA
MGGEAFRWPAGCYVNVPGFEHIMMAMETTITKDGVKHSDINQLGNEAERAALKESYSHL
AKLRDEVIAMGIIPAIADWKTVNPN
00000000000000000000000000000000000000000000000000000000000000000000000000000000001
11111100000000000000000000000000000000000000000000000000000000000000000000000000000
00000000000000000000000000000000000000000000000000000000000000000000000000000000000
00000000000000000000000000000000000000000000000000000000000000000000000000000111111
>Q2QEH4_3hiw_21_279
VIIYELNLQGTTKAQYSTFLKQLRDDIKDPNLHYGGTNLPVIKRPVGPPKFLRVNLKASTGT
VSLAVQRSNLYVAAYLAKNNNKQFRAYYFKGFQITTNQLNNLFPEATGVSNQQELGYGESY
PQIQNAAGVTRQQAGLGIKKLAESMTKVNGVARVEKDEALFLLIVVQMVGEAARFKYIEN
LVLNNFDTAKEVEPVPDRVIILENNWGLLSRAAKTANNGVFQTPLVLTSYAVPGVEWRVTT
VAEVEIGIFLNVDNN
00000000000000000000000000000000000000000000000000000000000000000000000000000000000
00000000000000000000000000000000000000000000000000000000000000000000000000000000000
00000000000000000000000000000000000000000000000000000000000000000000000000000000000

0000000011
>A4GRE3_2os5_0_118
MPMVRVATNLPDKDVPANFEERLTDLLAESMNKPRNRIAIEVLAGQRITHGASRNPVAVIK
VESIGALSADDNIRHTQKITQFCQDTLKLPKDKVIITYFDLQPIHVGFNGTTVAAATM
10000000000000000000000000000000000000000000000000000000000000000000000000000000000
000000000000000000000000000000000000
>O66513_2eg2_0_111
MKKIEAIIKPFKLDEVKDALVEIGIGGMTVTEVKGFGQQKGHTEIYRGTEYVIDFLPKVKIEV
VVRDEDVEKVVETIVKTAQTGRVGDGKIFIIPVEDVIRIRTGERGEQAI
00000000000000000000000000000000000001111111111111111000000000000000000000000000000
00000000000000000000000000000
>Q93VA3_2v07_70_174
QTLDIQRGATLFNRACIGCHDTGGNIIQPGATLFTKDLERNGVDTEEEIYRVTYFGKGRMPG
FGEKCTPRGQCTFGPRLQDEEIKLLAEFVKFQADQGWPTVSTD
11100000000000000000000000000000000000000000000000000000000000000000000000000000000
0000000000000000000111
>Q9GK11_4aa9_61_380
AREPLTSYLDSQYFGKIYIGTPPQEFTVVFDTGSSDLWVPSIYCKSNVCKNHHRFDPRKSSTF
RNLGKPLSIHYGTGSMEGFLGYDTVTVSNIVDPNQTVGLSTEQPGEVFTYSEFDGILGLAYPS
LASEYSVPVFDNMMDRHLVARDLFSVYMDRNGQGSMLTLGAIDPSYYTGSLHWVPVTLQ
QYWQFTVDSVTINGVAVACVGGCQAILDTGTSVLFGPSSDILKIQMAIGATENRYGEFDVN
CGNLRSMPTVVFEINGRDYPLSPSAYTSKDQGFCTSGFQGDNNSELWILGDVFIREYYSVFD
RANNRVGLAKAI
11111110000000000000000000000000000000000000000000000000000000000000000000000000000
00000000000000000000000000000000000000000000000000000000000000000000000000000000000
00000000000000000000000000000000000000000000000000000000000000000000000000000000000
00000000000000000000000000000000000000000000000000000000000000000000000
>O43175_2g76_3_313
ANLRKVLISDSLDPCCRKILQDGGLQVVEKQNLSKEELIAELQDCEGLIVRSATKVTADVIN
AAEKLQVVGRAGTGVDNVDLEAATRKGILVMNTPNGNSLSAAELTCGMIMCLARQIPQA
TASMKDGKWERKKFMGTELNGKTLGILGLGRIGREVATRMQSFGMKTIGYDPIISPEVSASF
GVQQLPLEEIWPLCDFITVHTPLLPSTTGLLNDNTFAQCKKGVRVVNCARGGIVDEGALLR
ALQSGQCAGAALDVFTEEPPRDRALVDHENVISCPHLGASTKEAQSRCGEEIAVQFVDMV
KGKSLTG
11000000000000000000000000000000000000000000000000000000000000000000000000000000000
00000000000000000000000000000000000000000000000000000000000000000000000000000000000
00000000000000000000000000000000000000000000000000000000000000000000000000000000000
00000000000000000000000000000000000000000000000000000001111111
>Q5SJ34_2cwd_0_160
MDRPVRVLFVCLGNICRSPMAEGIFRKLLKERGLEDRFEVDSAGTGAWHVGEPMDPRARR
VLEEEGAYFPHVARRLTREDVLAYDHILVMDRENLEEVLRRFPEARGKVRLVLEELGGGEV
QDPYYGDLEDFREVYWTLEAALQAFLDRHGSPSPAAEGRA
10000000000000000000000000000000000000000000000000000000000000000000000000000000000
000000000000000000000000000000000000000000000000000000000000000000001111111111
>Q6W6X6_2wua_16_448
PSSTSSSLENLSVSVCAAGDSAAYQRNSVFGDDVVIVAAYRSPLCKAKRGGLKDTYPDDILA
PVLKALIEKTNINPAEVGDIVVGSVLGAGSQRASECRMAAFYAGFPETVPVRTVNRQCSSGL
QAVADVAAAIKAGFYDIGIGAGLESMTANPMAWEGSVNPKVKTMAQAQDCLLPMGITSE
NVAQKFSITRQEQDQAAVGSHRKAAAATAAGRFKDEIIPIKTKIVDPKTGDEKPVTISVDDG
IRPGTSLADLAKLKPVFRKDGSTTAGTSSQVSDGAGAVLLMKRSIALQKGLPILGVFRTFAA
VGVPPSIMGIGPAVAIPAVKAAGLQIDDIDLFEINEAFASQFVYCQKKLEIDPQKINVNGGA

MAIGHPLGATGARCVATLLHEMKRRGRDCRFGVVSMCIGTGMGAAAVFERGDACDELC
NAKAK
11111111111111111111111111100000000000000000000000000000000000000000000000000000
00000000000000000000000000000000000000000000000000000000000000000000000000000000
00000000000000000000000000000000000000000000000000000000000000000000000000000000
00000000000000000000000000000000000000000000000000000000000000000000000000000000
00000000000000000000000000000000000000000000000000000000000000000000000000000000
00000001111111111
>Q3JV82_3dms_0_418
MPYQHIKVPEGGDKITVNKDFSLNVSDQPIIPYIEGDGTGFDITPVMIKVVDAAVEKAYGGK
KKIHWMEIYAGEKATKVYGPDVWLPEETLQVLKEYVVSIKGPLTTPVGGGIRSLNVALRQEL
DLYVCLRPIQYFKGVPSPVREPEKTNMVIFRENSEDIYAGIEWAAESEQAKKVIKFLQEEMG
VKKIRFPQTSGIGIKPVSKEGTERLVRKAIQYAIDNDRKSVTLVHKGNIMKFTEGAFRDAGYA
LAQKEFGAELIDGGPWMKFKNPKTGNEIVVKDSIADAFLQQILLRPAEYDVIATLNLNGDY
ISDALAAQVGGIGIAPGANLSDSVAMFEATHGTAPKYAGKDYVNPGSEILSAEMMLRHLG
WTEAADVIISAMEKSIKQKRVTYDFARLMEGATQVSCSGFGQVLIENME
11000000000000000000000000000000000000000000000000000000000000000000000000000000
00000000000000000000000001111000000000000000000000000000000000000000000000000000
00000000000000000000000000000000000000000000000000000000000000000000000000000000
00000000000000000000000000000000000000000000000000000000000000000000000000000000
00000000000000000000000000000000000000000000000000000000000000000000000000000000
0000
>Q5L3S8_2egu_0_307
MARTVNSITELIGDTPAVKLNRIVDEDSADVYLKLEFMNPGSSVKDRIALAMIEAAEKAGK
LKPGDTIVEPTSGNTGIGLAMVAAAKGYKAVLVMPDTMSLERRNLLRAYGAELVLTPGAQ
GMRGAIAKAEELVREHGYFMPQQFKNEANPEIHRLTTGKEIVEQMGDQLDAFVAGVGTG
GTITGAGKVLREAYPNIKIYAVEPADSPVLSGGKPGPHKIQGIGAGFVPDILDTSIYDGVITVT
TEEAFAAARRAAREEGILGGISSGAAIHAALKVAKELGKGKKVLAIIPSNGERYLSTPLYQFE
D
00000000000000000000000000000000000000000000000000000000000000000000000000000000
00000000000000000000000000000000000000000000000000000000011111111111111000000000
00000000000000000000000000000000000000011111111111111111111110000000000000000000
000000000000000000000000000000000000000000000000000000000
>P49347_1cnv_25_323
DISSTEIAVYWGQREDGLLRDTCKTNNYKIVFISFLDKFGCEIRKPELELEGVCGPSVGNPCSF
LESQIKECQRMGVKVFLALGGPKGTYSACSADYAKDLAEYLHTYFLSERREGPLGKVALDG
IHFDIQKPVDELNWDNLLEELYQIKDVYQSTFLLSAAPGCLSPDEYLDNAIQTRHFDYIFVRF
YNDRSCQYSTGNIQRIRNAWLSWTKSVYPRDKNLFLELPASQATAPGGGYIPPSALIGQVLP
YLPDLQTRYAGIALWNRQADKETGYSTNIIRYLNATAMPFTSNLLKYPS
00000000000000000000000000000000000000000000000000000000000000000000000000000000
00000000000000000000000000000000000000000000000000000000000000000000000000000000
00000000000000000000000000000000000000000000000000000000000000000000000000000000
000000000000000000000000000000000111111111111111
>B1YL86_3l6u_29_310
TSPKRNIVGFTIVNDKHEFAQRLINAFKAEAKANKYEALVATSQNSRISEREQILEFVHLKVD
AIFITTLDDVYIGSAIEEAKKAGIPVFAIDRMIRSDAVVSSITSNNQMIGEQLASYIKNELIKQT
GRSTGRIVEITGTANVYTTNERHRGFLKGIENEPTLSIVDSVSGNYDPVTSERVMRQVIDSGIP
FDAVYCHNDDIAMGVLEALKKAKISGKIVVGIDGNRAILEAVDMKSMDATVVQSAEEMM
KVAFSALKLHTKNKKIPDRFYTYSYLYDGSR
11111000000000000000000000000000000000000000000000000000000000000000000000000000
00000000000000000000000000000000000000000000000000000000000000000000000000000000

00000000000000000000000000000000000000000000000000000000000000000000000000000000000
000000000000000000000000000000111
>P40409_2xuz_20_316
GSKNESTASKASGTASEKKKIEYLDKTYEVTVPTDKIAITGSVESMEDAKLLDVHPQGAISFS
GKFPDMFKDITDKAEPTGEKMEPNIEKILEMKPDVILASTKFPEKTLQKISTAGTTIPVSHISS
NWKENMMLLAQLTGKEKKAKKIIADYEQDLKEIKTKINDKAKDSKALVIRIRQGNIYIYPE
QVYFNSTLYGDLGLKAPNEVKAAKAQELSSLEKLSEMNPDHIFVQFSDDENADKPDALKD
LEKNPIWKSLKAVKEDHVYVNSVDPLAQGGTAWSKVRFLKAAAEKLTQN
11111111111111111000000000000000000000000000000000000000000000000000000000000000000
00000000000000000000000000000000000000000000000000000000000000000000000000000000000
00000000000000000000000000000000000000000000000000000000000000000000000000000000000
000000000000000000000000000000000000000000000000
>P23280_3fe4_20_289
SDWTYSEGALDEAHWPQHYPACGGQRQSPINLQRTKVRYNPSLKGLNMTGYETQAGEFP
MVNNGHTVQISLPSTMRMTVADGTVYIAQQMHFHWGGASSEISGSEHTVDGIRHVIEIHIV
HYNSKYKSYDIAQDAPDGLAVLAAFVEVKNYPENTYYSNFISHLANIKYPGQRTTLTGLDV
QDMLPRNLQHYYTYHGSLTTPPCTENVHWFVLADFVKLSRTQVWKLENSLLDHRNKTIH
NDYRRTQPLNHRVVESNFPNQEYTLGSEFQ
11111111111000000000000000000000000000000000000000000000000000000000000000000000000
00000000000001110000000000000000000000000000000000000000000000000000000000000000000
00000000000000000000000000000000000000000000000000000000000000000000000000000000000
000000000001111111111
>P18465_2bvp_24_299
GSHSMRYFYTAMSRPGRGEPRFIAVGYVDDTQFVRFDSDAASPRMAPRAPWIEQEGPEYW
DGETRNMKASAQTYRENLRIALRYYNQSEAGSHIIQVMYGCDVGPDGRLLRGHDQSAYD
GKDYIALNEDLSSWTAADTAAQITQRKWEAARVAEQLRAYLEGLCVEWLRRYLENGKETL
QRADPPKTHVTHHPISDHEATLRCWALGFYPAEITLTWQRDGEDQTQDTELVETRPAGDRT
FQKWAAVVVPSGEEQRYTCHVQHEGLPKPLTLRWEP
00000000000000000000000000000000000000000000000000000000000000000000000000000000000
00000000000000000000000000000000000000000000000000000000000000000000000000000000000
00000000000000000000000000000000000000000000000000000000000000000000000000000000000
000000000000000000000000011
>P94556_1zuw_0_271
MLEQPIGVIDSGVGGLTVAKEIMRQLPKENIIYVGDTKRCPYGPRPEEEVLQYTWELTNYLL
ENHHIKMLVIACNTATAIALDDIQRSVGIPVVGVIQPGARAAIKVTDNQHIGVIGTENTIKS
NAYEEALLALNPDLKVENLACPLLVPFVESGKFLDKTADEIVKTSLYPLKDTSIDSLILGCTH
YPILKEAIQRYMGEHVNIISSGDETAREVSTILSYKGLLNQSPIAPDHQFLTTGARDQFAKIAD
DWFGHEVGHVECISLQEPIKR
10000000000000000000000000000000000000000000000000000000000000000000000000000000000
00000000000000000000000000000000000000000000000000000000000000000000000000000000000
00000000000000000000000000000000000000000000000000000000000000000000000000000000000
00000111110000000111111
>P0A7Z0_1o8b_0_218
MTQDELKKAVGWAALQYVQPGTIVGVGTGSTAAHFIDALGTMKGQIEGAVSSSDASTEKL
KSLGIHVFDLNEVDSLGIYVDGADEINGHMQMIKGGGAALTREKIIASVAEKFICIADASKQ
VDILGKFPLPVEVIPMARSAVARQLVKLGGRPEYRQGVVTDNGNVILDVHGMEILDPIAME
NAINAIPGVVTVGLFANRGADVALIGTPDGVKTIVK
10000000000000000111100000000000000000000000000000000000000000000000000000000000000
00000000000000100000000000000000000000000000000000000000000000000000000000000000000
00000000000000000000000000000000000000000000000000001
>P00785_2act_126_345

LPSYVDWRSAGAVVDIKSQGECGGCWAFSAIATVEGINKIVTGVLISLSEQELIDCGRTQNT
RGCNGGYITDGFQFIINNGGINTEENYPYTAQDGECNVDLQNEKYVTIDTYENVPYNNEW
ALQTAVTYQPVSVALDAAGDAFKQYSSGIFTGPCGTAVDHAVTIVGYGTEGGIDYWIVKNS
WDTTWGEEGYMRILRNVGGAGTCGIATMPSYPVKYNN
00000000000000000000000000000000000000000000000000000000000000000000000000000000000
00000000000000000000000000000000000000000000000000000000000000000000000000000000000
000000000000000000000000000000000000000000000000000011
>Q9L9E5_2c0z_0_206
MRLRPLGIEGVWEITPEQRADPRGVFLDWYHVDRFAEAIGRPLRLAQANLSVSVRGVVRGI
HFVDVPPGQAKYVTCVRGAVFDVVVDLRVGSPTYGCWEGTRLDDVSRRAVYLSEGIGHGF
CAISDEATLCYLSSGTYDPATEHGVHPLDPELAIDWPTGTPLLSPRDQDALLLAEARDAGLL
PTYATCQAVTVPSPAPGSVGDPGP
00000000000000000000000000000000000000000000000000000000000000000000000000000000000
00000000000000000000000000000000000000000000000000000000000000000000000000000000000
00000000000000000000000011111111111111111
>A2RQE2_2ea3_198_386
FDVIGGNAYTIGGRSRCSIGFAVNGGFITAGHCGRTGATTANPTGTFAGSSFPGNDYAFVRT
GAGVNLLAQVNNYSGGRVQVAGHTAAPVGSAVCRSGSTTGWHCGTITALNSSVTYPEGT
VRGLIRTTVCAEPGDSGGSLLAGNQAQGVTSGGSGNCRTGGTTFFQPVNPILQAYGLRMIT
TDSGSSP
00000000000000000000000000000000000000000000000000000000000000000000000000000000000
00000000000000000000000000000000000000000000000000000000000000000000000000000000000
00000000000000000111111
>Q2QJL3_1u11_0_181
MSETAPLPSASSALEDKAASAPVVGIIMGSQSDWETMRHADALLTELEIPHETLIVSAHRTP
DRLADYARTAAERGLNVIIAGAGGAAHLPGMCAAWTRLPVLGVPVESRALKGMDSLLSIV
QMPGGVPVGTLAIGASGAKNAALLAASILALYNPALAARLETWRALQTASVPNSPITEDK
11111111111111111000000000000000000000000000000000000000000000000000000000000000000
00000000000000000000000000000000000000000000000000000000000000000000000000000000000
0000000000000011
>B3YEF4_3ak9_0_166
MSTAKLVKTKASNLLYTRNDVSESDKKATVELLNRQVIQFIDLSLITKQAHWNMRGANFIA
VHEMLDGFRTALTDHLDTMAERAVQLGGVALGTTQVINSKTPLKSYPLDIHNVQDHLKEL
ADRYAVVANDVRKAIGEAKDEDTADIFTAASRDLDKFLWFIESNIE
11111111111100000000000000000000000000000000000000000000000000000000000000000000000
00000000000000000000000000000000000000000000000000000000000000000000000000000000000
0
>Q6H9H7_1v10_0_520
MPSFASLKSLVVLSLTSLSLAATVALDLHILNANLDPDGTGARSAVTAEGTTIAPLITGNIDD
RFQINVIDQLTDANMRRATSIHWHGFFQAGTTEMDGPAFVNQCPIIPNESFVYDFVVPGQA
GTYWYHSHLSTQYCDGLRGAFVVYDPNDPHLSLYDVDDASTVITIADWYHSLSTVLFPNP
NKAPPAPDTTLINGLGRNSANPSAGQLAVVSVQSGKRYRFRIVSTSCFPNYAFSIDGHRMTV
IEVDGVSHQPLTVDSLTIFAGQRYSVVVEANQAVGNYWIRANPSNGRNGFTGGINSAIFRYE
GAAVAEPTTSQNSGTALNEANLIPLINPGAPGNPVPGGADINLNLRIGRNATTADFTINGA
PFIPPTVPVLLQILSGVTNPNDLLPGGAVISLPANQVIEISIPGGGNHPFHLHGHNFDVVRTP
GSSVYNYVNPVRRDVVSIGGGGDNVTFRFVTDNPGPWFLHCHIDWHLEAGLAVVFAEDIP
NIPIANAISPAWDDLCPKYNANNPDSGLA
11111111111111111111100000000000000000000000000000000000000000000000000000000000000
00000000000000000000000000000000000000000000000000000000000000000000000000000000000
00000000000011111110000000000000000000000000000000000000000000000000000000000000000
00000000000000000000000000000000000000000000000000000000000000000000000000000000000

00000000000000000000000000000000000000000000000000000000000000000000000000000000000
00000000000000000000000000000000000000000000000000000000000000000000000000000000000
0000000000000000111111
>P00392_1zx9_95_560
EPPVQVAVIGSGGAAMAAALKAVEQGAQVTLIERGTIGGTCVNVGCVPSKIMIRAAHIAHL
RRESPFDGGIAATVPTIDRSKLLAQQQARVDELRHAKYEGILGGNPAITVVHGEARFKDDQ
SLTVRLNEGGERVVMFDRCLVATGASPAVPPIPGLKESPYWTSTEALASDTIPERLAVIGSSVV
ALELAQAFARLGSKVTVLARNTLFFREDPAIGEAVTAAFRAEGIEVLEHTQASQVAHMDGE
FVLTTTHGELRADKLLVATGRTPNTRSLALDAAGVTVNAQGAIVIDQGMRTSNPNIYAAG
DCTDQPQFVYVAAAAGTRAAINMTGGDAALDLTAMPAVVFTDPQVATVGYSEAEAHHD
GIETDSRTLTLDNVPRALANFDTRGFIKLVIEEGSHRLIGVQAVAPEAGELIQTAALAIRNRM
TVQELADQLFPYLTMVEGLKLAAQTFNKDVKQLSCCAG
11000000000000000000000000000000000000000000000000000000000000000000000000000000000
00000000000000000000000000000000000000000000000000000000000000000000000000000000000
00000000000000000000000000000000000000000000000000000000000000000000000000000000000
00000000000000000000000000000000000000000000000000000000000000000000000000000000000
00000000000000000000000000000000000000000000000000000000000000000000000000000000000
000000000000000000000000000000000000000001111111111
>Q9R4E4_2gga_0_454
MSHGASSRPATARKSSGLSGTVRIPGDKSISHRSFMFGGLASGETRITGLLEGEDVINTGKAM
QAMGARIRKEGDTWIIDGVGNGGLLAPEAPLDFGNAATGCRLTMGLVGVYDFDSTFIGDA
SLTKRPMGRVLNPLREMGVQVKSEDGDRLPVTLRGPKTPTPITYRVPMASAQVKSAVLLAG
LNTPGITTVIEPIMTRDHTEKMLQGFGANLTVETDADGVRTIRLEGRGKLTGQVIDVPGDPS
STAFPLVAALLVPGSDVTILNVLMNPTRTGLILTLQEMGADIEVINPRLAGGEDVADLRVRSS
TLKGVTVPEDRAPSMIDEYPILAVAAAFAEGATVMNGLEELRVKESDRLSAVANGLKLNGV
DCDEGETSLVVRGRPDGKGLGNASGAAVATHLDHRIAMSFLVMGLVSENPVTVDDATMIA
TSFPEFMDLMAGLGAKIELSDTKAA
11111000000000000000000000000000000000000000000000000000000000000000000000000000000
00000000000000000000000000000000000000000000000000000000000000000000000000000000000
00000000000000000000000000000000000000000000000000000000000000000000000000000000000
00000000000000000000000000000000000000000000000000000000000000000000000000000000000
00000000000000000000000000000000000000000000000000000000000000000000000000000000000
0000000000000000000000000000000000011111
>P13254_1gc0_0_397
MHGSNKLPGFATRAIHHGYDPQDHGGALVPPVYQTATFTFPTVEYGAACFAGEQAGHFYS
RISNPTLNLLEARMASLEGGEAGLALASGMGAITSTLWTLLRPGDEVLLGNTLYGCTFAFL
HHGIGEFGVKLRHVDMADLQALEAAMTPATRVIYFESPANPNMHMADIAGVAKIARKHG
ATVVVDNTYCTPYLQRPLELGADLVVHSATKYLSGHGDITAGIVVGSQALVDRIRLQGLKD
MTGAVLSPHDAALLMRGIKTLNLRMDRHCANAQVLAEFLARQPQVELIHYPGLASFPQYT
LARQQMSQPGGMIAFELKGGIGAGRRFMNALQLFSRAVSLGDAESLAQHPASMTHSSYTP
EERAHYGISEGLVRLSVGLEDIDDLLADVQQALKASA
11111100000000000000000000000000000000111111111111111111111100000000000000000000
00000000000000000000000000000000000000000000000000000000000000000000000000000000000
00000000000000000000000000000000000000000000000000000000000000000000000000000000000
00000000000000000000000000000000000000000000111111111111111000000000000000000000000
000000000000000000000000000000000000000000000000000000000000000000
>Q3LSM4_2huf_0_392
MEYKVTPPAVLREPLVTPNKLLMGPGPSNAPQRVLDAMSRPILGHLHPETLKIMDDIKEGV
RYLFQTNNIATFCLSASGHGGMEATLCNLLEDGDVILIGHTGHWGDRSADMATRYGADV
RVVKSKVGQSLSLDEIRDALLIHKPSVLFLTQGDSSTGVLQGLEGVGALCHQHNCLLIVDTV
ASLGGAPMFMDRWEIDAMYTGSQKVLGAPPGITPVSFSHRAVERYKRRNTKVKVYYWDM

SLVGDYWGCFGRPRIYHHTISSTLLYGLREAIAMACEEGLPALIARHEDCAKRLYRGLQDAG
FELYADPKDRLSTVTTIKVPQGVDWLKAAQYAMKTYLVEISGGLGPTAGQVFRIGLMGQN
ATTERVDRVLQVFQEAVAAVKPDVQVKGKM
00000000000000000000000000000000000000000000000000000000000000000000000000000000000
00000000000000000000000000000000000000000000000000000000000000000000000000000000000
00000000000000000000000000000000000000000000000000000000000000000000000000000000000
00000000000000000000000000000000000000000000000000000000000000000000000000000000000
0000000000000000000000000000000000000000000000000000011111111
>A1B7S1_3fa5_0_280
MSDPAGKPCIICVAITGSLPTKENNPAVPITLAEQVESTHEAFEAGASIAHCHVRDDEGRPTS
DPDRFAALKEGLERHCPGMIVQLSTGGRSGAGQARGAMLPLCPDMASLSVGSNNFPTRVY
ENPPDLVDWLAAEMLKYDIKPEIEAFDLSHILQAKRMAGDGRLAGTPYVQFVMGVRNAM
PADRDVFDYYIHTVRRLFGEDAPWCAAGIGPSQIVLNEWAISSGGHARTGLEDNVRLDRD
RLAPSNAALVGRAVELCEKYERPVATWRQARQILGLRMV
11111000000000000000000000000000000000000000000000000000000000000000000000000000000
00000000000000000000000000000000000000000000000000000000000000000000000000000000000
00000000000000000000000000000000000000000000000000000000000000000000000000000000000
00000000000000000000000000000000
>P26045_2b49_627_912
DTLEGSMAQLKKGLESGTVLIQFEQLYRKKPGLAITFAKLPQNLDKNRYKDVLPYDTTRVL
LQGNEDYINASYVNMEIPAANLVNKYIATQGPLPHTCAQFWQVVWDQKLSLIVMLTTLTE
RGRTKCHQYWPDPPDVMNHGGFHIQCQSEDCTIAYVSREMLVTNTQTGEEHTVTHLQYV
AWPDHGVPDDSSDFLEFVNYVRSLRVDSEPVLVHCSAGIGRTGVLVTMETAMCLTERNLPI
YPLDIVRKMRDQRAMMVQTSSQYKFVCEAILRVYEEGLVQMLDPS
11111111111111111100000000000000000000000000000000000000000000000000000000000000000
00000000000000000000000000000000000000000000000000000000000000000000000000000000000
00000000000000000000000000000000000000000000000000000000000000000000000000000000000
0000000000000000000000000000111111111
>O07539_3dnp_0_286
MSKQLLALNIDGALLRSNGKIHQATKDAIEYVKKKGIYVTLVTNRHFRSAQKIAKSLKLDA
KLITHSGAYIAEKIDAPFFEKRISDDHTFNIVQVLESYQCNIRLLHEKYSIGNKKKVNSNLLG
KALIHPSDPIFYPVQFVESLSDLLMDEPVSAPVIEVYTEHDIQHDITETITKAFPAVDVIRVND
EKLNIVPKGVSKEAGLALVASELGLSMDDVVAIGHQYDDLPMIELAGLGVAMGNAVPEIKR
KADWVTRSNDEQGVAYMMKEYFRMQQRKGFLDKFHMKR
11000000000000000000000000000000000000000000000000000000000000000000000000000000000
00000000000000000000000000000000000000000000000000000000000000000000000000000000000
00000000000000000000000000000000000000000000000000000000000000000000000000000000000
00000000000000000000000000001111111111
>Q5SH92_2d2e_0_249
MSQLEIRDLWASIDGETILKGVNLVVPKGEVHALMGPNGAGKSTLGKILAGDPEYTVERGE
ILLDGENILELSPDERARKGLFLAFQYPVEVPGVTIANFLRLALQAKLGREVGVAEFWTKVK
KALELLDWDESYLSRYLNEGFSGGEKKRNEILQLLVLEPTYAVLDETDSGLDIDALKVVARG
VNAMRGPNFGALVITHYQRILNYIQPDKVHVMMDGRVVATGGPELALELEAKGYEWLKE
KVKEGA
10000000000000000000000000000000000000000000000000000000000000000000000000000000000
00000001000000000000000000000000000000000000000000000000001111000000000000000000000
00000000000000000000000000000000000000000000000000000000000000000000000000000001111
1
>Q1QTX1_3lwd_0_224
MMNTTQEGRQRLAERLADTVAQALEADLAKRERALLVVSGGSTPKPFFTSLAAKALPWAR
VDVTLADERWVTADDADSNARLVRETLLVGPAAEACFHPLTTDDDTPEAGVETVAERLES

LPWPASAVILGMGGDGHTASLFPDSEQLATALETTSAAVVVHAPSVPQARITLSASRLADA
GLHVLHITGNDKRRVLAEALAGDDVRQLPIRAFLSQPIATYWAP
11000000000000000000000000000000000000000000000000000000000000000000000000000000000
00000000000000000000000000000000000000000000000000000000000000000000000000000000000
00000000000000000000000000000000000000000000000000000000000
>A4YI54_3ve9_0_214
MNRVILSLDSPIPEETLRKLNGKVAGIKVGWPLLLNLGKEKVKELVGLVDGIKILDLKLADID
NTMILIVDELKDITNSFIAHAFVGVEGSLASLSQRVDLFLVLSMSHPGWNDAFYPYLREVAR
RVNPKGFVAPATRPSMISRVKGDFPDKLVISPGVGTQGAKPGIALCHGADYEIVGRSVYQSA
DPVRKLEEIVRSQEEVLSSCEGAKDRGS
00000000000000000000000000000000000000000000000000000000000000000000000000000000000
00000000000000000000000000000000000000000000000000000000000000000000000000000000000
0000000000000000000000000000000000000001111111111
>P15700_1ukz_1_203
TAATTSQPAFSPDQVSVIFVLGGPGAGKGTQCEKLVKDYSFVHLSAGDLLRAEQGRAGSQY
GELIKNCIKEGQIVPQEITLALLRNAISDNVKANKHKFLIDGFPRKMDQAISFERDIVESKFIL
FFDCPEDIMLERLLERGKTSGRSDDNIESIKKRFNTFKETSMPVIEYFETKSKVVRVRCDRSVE
DVYKDVQDAIRDSL
11111110000000000000000000000000000000000000000000000000000000000000000000000000000
00000000000000000000000000000000000000000000000000000000000000000000000000000000000
0000000000000000000000000000000000000
>Q9KQI2_3lv8_0_211
MNAKFIVIEGLEGAGKSTAIQVVVETLQQNGIDHITRTREPGGTLLAEKLRALVKEEHPGEE
LQDITELLLVYAARVQLVENVIKPALARGEWVVGDRHDMSSQAYQGGGRQIAPSTMQSLK
QTALGDFKPDLTLYLDIDPKLGLERARGRGELDRIEKMDISFFERARERYLELANSDDSVVMI
DAAQSIEQVTADIRRALQDWLSQVNRV
10000000000000000000000000000000000000000000000000000000000000000000000000000000000
00000000000000000000000000000000000000000000000000000000000000001111100000000000000
0000000000000000000000000000000000000000000011
>Q5RZ98_3zse_42_235
AVTSNETGYHDGYFYSFWTDAPGTVSMELGPGGNYSTSWRNTGNFVAGKGWATGGRRTV
TYSASFNPSGNAYLTLYGWTRNPLVEYYIVESWGTYRPTGTYMGTVTTDGGTYDIYKTTRYN
APSIEGTRTFDQYWSVRQSKRTSGTITAGNHFDAWARHGMHLGTHDYMIMATEGYQSSGS
SNVTLGTSGGDNP
00000000000000000000000000000000000000000000000000000000000000000000000000000000000
00000000000000000000000000000000000000000000000000000000000000000000000000000000000
0000000000000000000000111111
>Q2GI30_3u04_0_187
MSVLSIVTVPDKRLSLCSEEVEKVDQSIRKLVDDMFETMHANQGLGLAAVQVGVHKRILV
MNVPEEFEDSEDIENVEDKIEGYELYGGPYCIINPKIVDISQEKVKLKEGCLSVPGYFDYIVRP
QRIAVQYLDYNGNECIIKAQGWLARCLQHEIDHLNGTVFLKYLSKFKRDFAIEKVKKKERT
DLI
00000000000000000000000000000000000000000000000000000000000000000011111111111110000
00000000000000000000000000000000000000000000000000000000000000000000000000000000000
0000000000000000000111
>Q9KCH6_2ah6_0_195
MRLYTRTGDKGKTSVIGGRLAKDDTRVVAYGTTDELNSFVGLAITQLDENTFADIRGELFKI
QHELFDCGGDLAMLKVKEDRPYKAKQEIVDFLEQRIDAYIKEAPELERFILPGGSEAAASLH
VCRTIARRAERYVVRLQQEGEINPIVLKYLNRLSDYFFAVARVVNSRLQVPDVEYERSAIVFR
EGKRKEDKK
11111111111111111111100000000000000000000000000000000000000000000000000000000000000

00000000000000000000000000000000000000000000000000000000000000000000000000000000
00000000000000111111111111111
>P53673_3lwk_7_195
SAGPWKMVVWDEDGFQGRRHEFTAECPSVLELGFETVRSLKVLSGAWVGFEHAGFQGQQ
YILERGEYPSWDAWGGNTAYPAERLTSFRPAACANHRDSRLTIFEQENFLGKKGELSDDYPS
LQAMGWEGNEVGSFHVHSGAWVCSQFPGYRGFQYVLECDHHSGDYKHFREWGSHAPTF
QVQSIRRIQQ
00000000000000000000000000000000000000000000000000000000000000000000000011111000
00000000000000000000000000000000000000000000000000000000000000000000000000000000
0000001111000000000000
>Q2GFI3_3lo0_0_171
MNLDNVTGGDNVPKEINVIIEISQNSCPVKYEFDKEKNLFCVDRFLPTSMYYPCNYGFIPHT
CAGDGDPVDVLVASRFPVMSGAVIRARPVGVLVMHDESGEDVKILAVPTHKVDQYYNNIK
DYSDFPVSFLNSISHFFTFYKKLEEDKFVSVEGWKDVTVAEKLILSALIK
11100000000000000000000000000000000000000000000000000000000000000000000000000000
00000000000000011100000000000000000000000000000000000000001111111000000000000000
000011
>Q29147_2ra6_15_179
LQPECSRSEEDLSDEKERKWEQLSRHWHTVVLASSDRSLIEEEGPFRNFIQNITVESGNLNGF
FLTRKNGQCIPLYLTAFKTEEARQFKLNYYGTNDVYYGSSKPNEYAKFIFYNYHDGKVNVV
ANLFGRTPNLSNEIKKRFEEDFMNRGFRRENILDISEVDHC
11111111111111111111110000000000000000000000000000000000000000000000000000000000
0000000000000000000000000000000000000000000000000000000000000000000000000000000
>P03366_1w5w_489_598
ADRQGTVSFNFPQITLWQRPLVTIKIGGQLKEALLDTGADDTVLEEMSLPGRWKPKMIGGI
GGFIKVRQYDQILIEICGHKAIGTVLVGPTPVNIIGRNLLTQIGCTLNF
11111111111000000000000000000000000000000000000000000000000000000000000000000000
000000000000000000000000000
>P13377_2o2c_0_606
MSSYLDDLRIDLAASPASGGSASIAVGSFNIPYEVTRRLKGVGADADTTLTSCASWTQLQKL
YEQYGDEPIKKHFEADSERGQRYSVKVSLGSKDENFLFLDYSKSHINDEIKCALLRLAEERGI
RQFVQSVFRGERVNTTENRPVLHIALRNRSNRPIYVDGKDVMPAVNKVLDQMRSFSEKVR
TGEWKGHTGKAIRHVVNIGIGGSDLGPVMATEALKPFSQRDLSLHFVSNVDGTHIAEVLKS
IDIEATLFIVASKTFTTQETITNALSARRALLDYLRSRGIDEKGSVAKHFVALSTNNQKVKEFG
IDEENMFQFWDWVGGRYSMWSAIGLPIMISIGYENFVELLTGAHVIDEHFANAPPEQNVPL
LLALVGVWYINFFGAVTHAILPYDQYLWRLPAYLQQLDMESNGKYVTRSGKTVSTLTGPIIF
GEAGTNGQHAFYQLIHQGTNLIPCDFIGAIQSQNKIGDHHKIFMSNFFAQTEALMIGKSPSE
VRRELEAAGERSAEKINALLPHKTFIGGRPSNTLLIKSLTPRALGAIIAMYEHKVLVQGAIW
GIDSYDQWGVELGKVLAKSILPQLRPGMRVNNHDSSTNGLINMFNELSHL
11111111111111111111111111111111111111111100000000000000000000000000000000000000
00000000000000000000000000000000000000000000000000000000000000000000000000000000
00000000000000000000000000000000000000000000000000000000000000000000000000000000
00000000000000000000000000000000000000000000000000000000000000000000000000000000
00000000000000000000000000000000000000000000000000000000000000000000000000000000
00000000000000000000000000000000000000000000000000000000000000000000000000000000
00000000000000000000000000000000000000000000000000000000000000000000000000000000
00000000000000000000000001
>P0A6P9_3h8a_0_431
MSKIVKIIGREIIDSRGNPTVEAEVHLEGGFVGMAAAPSGASTGSREALELRDGDKSRFLGK
GVTKAVAAVNGPIAQALIGKDAKDQAGIDKIMIDLDGTENKSKFGANAILAVSLANAKAA
AAAKGMPLYEHIAELNGTPGKYSMPVPMMNIINGGEHADNNVDIQEFMIQPVGAKTVKE

AIRMGSEVFHHLAKVLKAKGMNTAVGDEGGYAPNLGSNAEALAVIAEAVKAAGYELGKD
ITLAMDCAASEFYKDGKYVLAGEGNKAFTSEEFTHFLEELTKQYPIVSIEDGLDESDWDGFA
YQTKVLGDKIQLVGDDLFVTNTKILKEGIEKGIANSILIKFNQIGSLTETLAAIKMAKDAGYT
AVISHRSGETEDATIADLAVGTAAGQIKTGSMSRSDRVAKYNQLIRIEEALGEKAPYNGRKEI
KGQA
10000000000000000000000000000000000000000000000000000000000000000000000000000000000
00000000000000000000000000000000000000000000000000000000000000000000000000000000000
00000000000000000000000000000000000000000000000000000000000000000000000000000000000
00000000000000000000000000000000000000000000000000000000000000000000000000000000000
00000000000000000000000000000000000000000000000000000000000000000000000000000000000
0000000000000001
>Q3JGP5_3ecd_0_423
MSNANPFFSQSLAERDASVRGAILKELERQQSQVELIASENIVSRAVLDAQGSVLTNKYAEG
YPGKRYYGGCEFADEVEALAIERVKRLFNAGHANVQPHSGAQANGAVMLALAKPGDTVL
GMSLDAGGHLTHGAKPALSGKWFNALQYGVSRDTMLIDYDQVEALAQQHKPSLIIAGFS
AYPRKLDFARFRAIADSVGAKLMVDMAHIAGVIAAGRHANPVEHAHVVTSTTHKTLRGP
RGGFVLTNDEEIAKKINSAVFPGLQGGPLMHVIAGKAVAFGEALTDDFKTYIDRVLANAQA
LGDVLKAGGVDLVTGGTDNHLLLVDLRPKGLKGAQVEQALERAGITCNKNGIPFDPEKPT
ITSGIRLGTPAGTTRGFGAAEFREVGRLILEVFEALRTNPEGDHATEQRVRREIFALCERFPIY
11000000000000000000000000000000000000000000000000000000000000011111111110000000000
00000000000000000000000000000000000000000011111111111111111000000000000000000000000
00000000000000000000000000000000000000000000000000000000000000000000000000000000000
00000000000111110000000000000000000000000000000000000000000000000000000000000000000
00000000000000000000000000000000000000000000000000000000000000000000000000000000000
000000000
>P19576_3jyr_0_395
MKIKTGVGILALSALTTMMISAPALAKIEEGKLVIWINGDKGYNGLAEVGKKFEQDTGIKVT
VEHPDKLEEKFPQVAATGDGPDIIFWAHDRFGGYAQSGLLAEVTPDKAFQDKLYPFTWDA
VRYNGKLIAYPIAVEALSLIYNKDLVPNPPKTWEEIPALDKELKVKGKSAIMFNLQEPYFTW
PLIAADGGYAFKFENGKYDVKDVGVDNAGAKAGLTFLIDMIKNKNMSADTDYSIAEAAF
NKGETAMTINGPWAWSNIDKSKVNYGVTLLPTFKGKPSKPFVGVLSAGINAASPNKELAK
EFLENYLLTDQGLEAVNKDKPLGAVALKSFQEQLAKDPRIAATMDNAQKGEIMPNIPQMS
AFWYAVRTAVINAASGRQTVDAALKDAQSRITK
11111111111111111111111110000000000000000000000000000000000000000000000000000000000
00000000000000000000000000000000000000000000000000000000000000000000000000000000000
00000000000000000000000000000000000000000000000000000000000000000000000000000000000
00000000000000000000000000000000000000000000000000000000000000000000000000000000000
0000000000000000000000000000000000000000000000000000000000000000
>B7X9Z2_3abx_19_402
APSASFERRQGSVNPYIGRSPLVIKSYAEKLEETIAYFEAQGDELNAARTRTVQGIPTFAWISD
SATIDTIQPLIADAVAHQEASGEQVLVQLVIYNLPDRDCAAKASDGEFHLDDDGANKYRAY
VDRIVAELSTADADKLHFSIVLEPDSLGNMVTNMHVPKCQGAATAYKEGIAYTIASLQKPNI
DLYIDAAHGGWLGWNDNLRPSAEIFKETLDLARQITPNATVRGLAINVSNYNPYKTRARE
DYTEWNNAYDEWNYVKTLTPHLQAVGFPAQFIVDQGRSGREGIRTEWGQWCNIRNAGFG
IRPTTDQAIVDSANVDAIVWVKPGGESDGTSDVNAVRFDENCRSPASHVPAPEAGEWFNE
FVVNLVINANPPLEPTYA
11111111111100000000000000000000000000000000000000000000000000000000000000000000000
00000000000000000000000000000000000000000000000000000000000000000000000000000000000
00000000000000000000000000000000000000000000000000000000000000000000000000000000000
00000000000000000000000000000000000000000000000000000000000000000000000000000000000
0000000000000000000000000000000000000000000000000000

>O94753_3fmu_30_360
ATCDDGRTTANAACCILFPILDDIQENLFDGAQCGEEVHESLRLTFHDAIGFSPTLGGGGA
DGSIIAFDTIETNFPANAGIDEIVSAQKPFVAKHNISAGDFIQFAGAVGVSNCPGGVRIPFFLG
RPDAVAASPDHLVPEPFDSVDSILARMGDAGFSPVEVVWLLASHSIAAADKVDPSIPGTPFD
STPGVFDSQFFIETQLKGRLFPGTADNKGEAQSPLQGEIRLQSDHLLARDPQTACEWQSMV
NNQPKIQNRFAATMSKMALLGQDKTKLIDCSDVIPTPPALVGAAHLPAGFSLSDVEQACA
ATPFPALTADPGPVTSVPPVPGS
00000000000000000000000000000000000000000000000000000000000000000000000000000000000
00000000000000000000000000000000000000000000000000000000000000000000000000000000000
00000000000000000000000000000000000000000000000000000000000000000000000000000000000
0000000000000000000000000000000000000000000000000000000000000000000000111111111111
>Q8X236_3f8d_0_323
MSLLPRTTSVKPGEKFDVIIVGLGPAAYGAALYSARYMLKTLVIGETPGGQLTEAGIVDDYL
GLIEIQASDMIKVFNKHIEKYEVPVLLDIVEKIENRGDEFVVKTKRKGEFKADSVILGIGVKR
RKLGVPGEQEFAGRGISYCSVCDAPLFKNRVVAVIGGGDSALEGAEILSSYSTKVYLIHRRDT
FKQQPIYVETVKKKPNVEFVLNSVVKEIKGDKVVKQVVVENLKTGEIKELNVNGVFIEIGFD
PPTDFAKSNGIETDTNGYIKVDEWMRTSVPGVFAAGDCTSAWLGFRQVITAVAQGAVAATS
AYRYVTEKKGKK
11111111100000000000000000000000000000000000000000000000000000000000000000000000000
00000000000000010000000000000000000000000000000000000000000000000000000000000000000
00000000000000000000000000000000000000000000000000000000000000000000000000000000000
00000000000000000000000000000000000000000000000000000000000000000000001111
>Q9KQH6_3hjv_0_311
MKERFMSKFAIVFPGQGSQAVGMLADLAEQYAVVKQTFAEASEVLGYDLWALVQDGPVE
DLNQTFRTQPALLAASVAIWRVWQQLGLEQPAVLAGHSLGEYSALVCAGVIDFKQAIKLVE
LRGQLMQQAVPAGTGAMYAIIGLEDEAIAKACADAAQGEVVSPVNFNSPGQVVIAGQKD
AVERAGVLCKEAGAKRALPLPVSVPSHCALMKPAADELAKTLAELEFNAPQIPVINNVDV
VAETDPVKIKDALIRQLYSPVRWTECVEQMSAQGVEKLIEMGPGKVLTGLTKRIVKTLEGVA
VNDVASLDAVK
11111000000000000000000000000000000000000000000000000000000000000000000000000000000
00000000000000000000000000000000000000000000000000000000000000000000000000000000000
00000000000000000000000000000000000000000000000000000000000000000000000000000000000
00000000000000000000000000000000000000000000000000000000000000
>P32718_3htv_0_308
MQKQHNVVAGVDMGATHIRFCLRTAEGETLHCEKKRTAEVIAPGLVSGIGEMIDEQLRRF
NARCHGLVMGFPALVSKDKRTIISTPNLPLTAADLYDLADKLENTLNCPVEFSRDVNLQLS
WDVVENRLTQQLVLAAYLGTGMGFAVWMNGAPWTGAHGVAGELGHIPLGDMTQHCAC
GNPGCLETNCSGMALRRWYEQQPRNYPLRDLFVHAENAPFVQSLLENAARAIATSINLFD
PDAVILGGGVMDMPAFPRETLVAMTQKYLRRPLPHQVVRFIAASSSDFNGAQGAAILAHQ
RFLPQFCAKAP
11000000000000000000000000000000000000000000000000000000000000000000000000000000000
00000000000000000000000000000000000000000000000000000000000000000000000000000000111
11111111111111111100000000000000000000000000000000000000000000000000000000000000000
00000000000000000000000000000000000000000000000000001111111
>Q5LX22_3l12_0_311
MNGFSQLEGLRGHPSVVRVIGHRGARGVMPENTLEGFAFTLAAGVRALEFDVVMTADGV
PVVTHNHHLANAMTRDGQGHWLTGAERQVAEMTYAEIRALDVGGLDGRTVYGRRFPDQ
AFLTGIHVPRLGELLDLCAGYGDQAPYLLLELKSDPALMHDHAARAEMVAAVLADVRRY
RMEPRTVMHSFDWALLGECRRQAPDLPTSYLSQLPENADDPGEDSAKPVGPDYDRMTESL
PQAVASAGGQLWCPYFLDVTPELVAEAHDLGLIVLTWTVNEPEDIRRMATTGVDGIVTDYP
GRTQRILIDMGLSWT

11000000000000000000000000000000000000000000000000000000000000000000000000000000000
00000000000000000000000000000000000000000000000000000000000000000000000000000000000
00000000000000000000000000000000000000000000001111111111111000000000000000000000000
000000000000000000000000000000000000000000000000000000000000000
>P59337_3a2n_0_309
MSKPIEIEIRRAPVLGSSMAYRETGAQDAPVVLFLHGNPTSSHIWRNILPLVSPVAHCIAPDLI
GFGQSGKPDIAYRFFDHVRYLDAFIEQRGVTSAYLVAQDWGTALAFHLAARRPDFVRGLAF
MEFIRPMPTWQDFHHTEVAEEQDHAEAARAVFRKFRTPGEGEAMILEANAFVERVLPGGI
VRKLGDEEMAPYRTPFPTPESRRPVLAFPRELPIAGEPADVYEALQSAHAALAASSYPKLLF
TGEPGALVSPEFAERFAASLTRCALIRLGAGLHYLQEDHADAIGRSVAGWIAGIEAVRPQLA
A
11111111100000000000000000000000000000000000000000000000000000000000000000000000000
00000000000000000000000000000000000000000000000000000000000000000000000000000000000
00000000000000000000000000000000000000000000000000000000000000000000000000000000000
0000000000000000000000000000000000000000000000000000000000111
>C4M7X7_3ipw_0_301
MAQQFIDIGANLTDDNYFGNYHGKHYHEEDIDVVLQRAERNGLSHIIITSGCLNDFKKAIEI
INKYQNLTNIKLVTTIGVHPTRTNELKQEGYLDELLLLCEKNIDKVVAIGEIGLDYERLQFSD
KETQLSGYRTLSILHQKYPYLPFFFHCRKSWSDLCQLNKELGYNGCKGVVHCFDGTEEEM
NQILNEGWDIGVTGNSLQSIELLNVMKQIPIERLHIETDCPYCGIKKTSAGFKYLKEKDFGV
KVEKYQRNKYVQRRNEPSNIIDIAIIMSSIKHISLFEFVNKVYSNSMNMYFPTMN
00000000000000000000000000000000000000000000000000000000000000000000000000000000000
00000000000000000000000000000000000000000000000000000000000000000000000000000000000
00000000000000000000000000000000000000000000000000000000000000000000000000000000000
00000000000000000000000000000000000000000000000000011
>Q8Y9N5_3tnl_0_290
MTNKITERITGHTELIGLIATPIRHSLSPTMHNEAFAKLGLDYVYLAFEVGDKELKDVVQGF
RAMNLRGWNVSMPNKTNIHKYLDKLSPAAELVGAVNTVVNDDGVLTGHITDGTGYMRA
LKEAGHDIIGKKMTICGAGGAATAICIQAALDGVKEISIFNRKDDFYANAEKTVEKINSKTD
CKAQLFDIEDHEQLRKEIAESVIFTNATGVGMKPFEGETLLPSADMLRPELIVSDVVYKPTKT
RLLEIAEEQGCQTLNGLGMMLWQGAKAFEIWTHKEMPVDYIKEILF
11000000000000000000000000000000000000000000000000000000000000000000000000000000000
00000000000000000000000000000000000000000000000000000000000000000000000000000000000
00000000000000000000000000000000000000000000000000000000000000000000000000000000000
000000000000000000000000000000000000000000
>B4EDA2_3tml_0_283
MKLCDFEVGLDQPFFLIAGTCVVESEQMTIDTAGRLKEICEKLNVPFIYKSSYDKANRSSGKS
FRGLGMDEGLRILSEVKRQLGLPVLTDVHSIDEIEQVASVVDVLQTPAFLCRQTDFIHACAR
SGKPVNIKKGQFLAPHDMKNVIDKARDAAREAGLSEDRFMACERGVSFGYNNLVSDMRS
LAIMRETNAPVVFDATHSVQLPGGQGTSSGGQREFVPVLARAAVATGVAGLFMETHPNPA
EAKSDGPNAVPLNRMGALLETLVTLDQAVKRNPFLENDFN
00000000000000000000000000000000000000000000000000000111111111111100000000000000000
00000000000000000000000000000000000000000000000000000000000000000000000000000000000
00000000000000000000000000000000000000000111111100000000000000000000000000000000000
00000000000000000000000000000000001
>G1ED17_3atg_52_307
APGDLLWSDEFDGAAGSAPNPAVWNHETGAHGWGNAELQNYTASRANSALDGQGNLVI
TARREGDGSYTSARMTTQGKYQPQYGRIEARIQIPRGQGIWPAFWMLGGSFPGTPWPSSGEI
DIMENVGFEPHRVHGTVHGPGYSGGSGITGMYQHPQGWSFADTFHTFAVDWKPGEITWF
VDGQQFHRVTRASVGANAWVFDQPFFLILNVAVGGQWPGYPDGTTQLPQQMKVDYVRV
YDNGSGSSNPGNPGTGLPT

00000000000000000000000000000000000000000000000000000000000000000000000000000000000
00000000000000000000000000000000000000000000000000000000000000000000000000000000000
00000000000000000000000000000000000000000000000000000000000000000000000000001111111
1111111
>P02873_1dhk_23_245
ATETSFIIDAFNKTNLILQGDATVSSNGNLQLSYNSYDSMSRAFYSAPIQIRDSTTGNVASFDT
NFTMNIRTHRQANSAVGLDFVLVPVQPESKGDTVTVEFDTFLSRISIDVNNNDIKSVPWDV
HDYDGQNAEVRITYNSSTKVFSVSLSNPSTGKSNNVSTTVELEKEVYDWVSVGFSATSGAY
QWSYETHDVLSWSFSSKFINLKDQKSERSNIVLNKIL
00000000000000000000000000000000000000000000000000000000000000000000000000111000000
00000011111100000000000000000000000000000000000000000000000000000000000000000000000
000000000000000000000000000000000000001111111111111111111
>Q54331_2nlr_40_273
DTTICEPFGTTTIQGRYVVQNNRWGSTAPQCVTATDTGFRVTQADGSAPTNGAPKSYPSVF
NGCHYTNCSPGTDLPVRLDTVSAAPSSISYGFVDGAVYNASYDIWLDPTARTDGVNQTEIM
IWFNRVGPIQPIGSPVGTASVGGRTWEVWSGGNGSNDVLSFVAPSAISGWSFDVMDFVRAT
VARGLAENDWYLTSVQAGFEPWQNGAGLAVNSFSSTVETGTPGGTDPGDPG
00000000000000000000000000000000000000000000000000000000000000000000000000000000000
00000000000000000000000000000000000000000000000000000000000000000000000000000000000
00000000000000000000000000000000000000000000000000000000111111111111
>Q8L3E1_2j4x_26_237
DAVLVNSELKNIYMKDVINKTNMKITKKIGTQLIFNTNEKTRVWDDDNYNKVISSNVSPAQ
ERRFKEEEEVDIYALIKSYSVICKEQYNYVDGGLIKTSDREKLDSTIYMNIFGEQIPLKEQSKY
KITFQNKFVTFQEIDVRLRKSLMSDNRIKLYEHNSICKKGYWGIHYKDNTTKFTDLFTHPNY
TDNETIDMSKVSHFDVYLNEEFSKN
00000000000000000000000000000000000011111111111111000000000000000000000000000000000
00000000000000000000000000000000000000000000000000000000000000000000000000000000000
0000000000000000000000000000000000000000000011
>Q48J46_3irv_30_251
AEVNPMSKPLVRWPINPLRTAVIVVDMQKVFCEPTGALYVKSTADIVQPIQKLLQAARAAQ
VMVIYLRHIVRGDGSDTGRMRDLYPNVDQILARHDPDVEVIEALAPQSDDVIVDKLFYSGF
HNTDLDTVLRARDVDTIIVCGTVTNVCCETTIRDGVHREYKVIALSDANAAMDYPDVGFG
AVSAADVQRISLTTIAYEFGEVTTTAEVIRRIESAYPHGR
11111000000000000000000000000000000000000000000000000000000000000000000000000000000
00000000000000000000000000000000000000000000000000000000000000000000000000000000000
00000000000000000000000000000000000000000000000000001111
>P0A2H9_3l9s_18_206
AAQISDGKQYITLDKPVAGEPQVLEFFSFYCPHCYQFEEVLHVSDNVKKKLPEGTKMTKYH
VEFLGPLGKELTQAWAVAMALGVEDKVTVPLFEAVQKTQTVQSAADIRKVFVDAGVKGE
DYDAAWNSFVVKSLVAQQEKAAADLQLQGVPAMFVNGKYQINPQGMDTSSMDVFVQQY
ADTVKYLVDKK
11100000000000000000000000000000000000000000000000000000000000000000000000000000000
00000000000000000000000000000000000000000000000000000000000000000000000000000000000
00000000000000000000001
>P66952_1xvq_0_164
MAQITLRGNAINTVGELPAVGSPAPAFTLTGGDLGVISSDQFRGKSVLLNIFPSVDTPVCATS
VRTFDERAAASGATVLCVSKDLPFAQKRFCGAEGTENVMPASAFRDSFGEDYGVTIADGP
MAGLLARAIVVIGADGNVAYTELVPEIAQEPNYEAALAALGA
10000000000000000000000000000000000000000000000000000000000000000000000000000000000
0000000000111111000000000000000000000000000000000000000000000000000000000000000000
>P93330_4jhg_0_162

MGVITSESEYVSSLSAEKLYRGIVEDGNIIYPKALPRFIEKAETLEGDGGPGTIKKLTFVGDFGS
TKQHIDMVDRENCAYTYSVYEGIALSDQPLEKIVFEFKLVPTPEEGCIVKSTTKYYTKGDDIE
LSKDYLEAGIERFEGFTKAVESFLLANPDYNKDSN
00000000000000000000000000000000000000000000000000000000000000000000000000000000000
00000000000000000000000000000000000000000000000000000000000000000000000000011111
>Q99L83_2d6m_0_156
MALFSAQSPYINPIIPFTGPIQGGLQEGLQVTLQGTTKSFAQRFVVNFQNSFNGNDIAFHFN
PRFEEGGYVVCNTKQNGQWGPEERKMQMPFQKGMPFELCFLVQRSEFKVMVNKKFFVQY
QHRVPYHLVDTIAVSGCLKLSFITFQTQDFRPAHQA
00000000000000000000000000000000000000000000000000000000000000000000000000000000000
00000000000000000000000000000000000000000000000000000000000000000011111111
>O31302_3nfd_0_152
MLDNREAMTVGVDLVHIPGFAEQLSRPGSTFEQVFSPLERRHAQTRRDAAADATNSSLAG
SRTEHLAGRWAAKEAFIKAWSQAIYGKPPVIEPDLVNFAEIEVLPDRWGRVALQLKGEVAA
KLQESIGDVELALSISHDGDYATAQCLLRYQR
11111000000000000000000000000000000000000000001111111111110000000000000000000000000
0000000000000000000000000000000000000000000000000000000000000000000000
>P00138_1cgo_1_126
FAKPEDAVKYRQSALTLMASHFGRMTPVVKGQAPYDAAQIKANVEVLKTLSALPWAAFG
PGTEGGDARPEIWSDAASFKQKQQAFQDNIVKLSAAADAGDLDKLRAAFGDVGASCKAC
HDAYRKKK
00000000000000000000000000000000000000000000000000000000000000000000000000000000000
0000000000000000000000000000000000000000011
>P33764_1kso_0_100
MARPLEQAVAAIVCTFQEYAGRCGDKYKLCQAELKELLQKELATWTPTEFRECDYNKFMS
VLDTNKDCEVDFVEYVRSLACLCLYCHEYFKDCPSEPPCSQ
10000000000000000000000000000000000000000000000000000000000000000000000000000000000
000000000001111111
>P05205_1q3l_16_75
AEEEEEEYAVEKIIDRRVRKGKVEYYLKWKGYPETENTWEPENNLDCQDLIQQYEASRKD
111111000000000000000000000000000000000000000000000000000011
>P21852_1h2a_0_566
MSGCRAQNAPGGIPVTPKSSYSGPIVVDPVTRIEGHLRIEVEVENGKVKNAYSSSTLFRGLEII
LKGRDPRDAQHFTQRTCGVCTYTHALASTRCVDNAVGVHIPKNATYIRNLVLGAQYLHD
HIVHFYHLHALDFVDVTAALKADPAKAAKVASSISPRKTTAADLKAVQDKLKTFVESGQL
GPFTNAYFLGGHPAYYLDPETNLIATAHYLEALRLQVKAARAMAVFGAKNPHTQFTVVGG
VTCYDALTPQRIAEFEALWKETKAFVDEVYIPDLLVVAAAYKDWTQYGGTDNFITFGEFPK
DEYDLNSRFFKPGVVFKRDFKNIKPFDKMQIEEHVRHSWYEGAEARHPWKGQTQPKYTDL
HGDDRYSWMKAPRYMGEPMETGPLAQVLIAYSQGHPKVKAVTDAVLAKLGVGPEALFST
LGRTAARGIETAVIAEYVGVMLQEYKDNIAKGDNVICAPWEMPKQAEGVGFVNAPRGGLS
HWIRIEDGKIGNFQLVVPSTWTLGPRCDKNKLSPVEASLIGTPVADAKRPVEILRTVHSFDP
CIACGVHVIDGHTNEVHKFRIL
11111111111111111100000000000000000000000000000000000000000000000000000000000000000
00000000000000000000000000000000000000000000000000000000000000000000000000000000000
00000000000000000000000000000000000000000000000000000000000000000000000000000000000
00000000000000000000000000000000000000000000000000000000000000000000000000000000000
00000000000000000000000000000000000000000000000000000000000000000000000000000000000
00000000000000000000000000000000000000000000000000000000000000000000000000000000000
000000000000000000000000000000000000000000000000000000111111111111111
>A6KZB0_3lew_22_515
LDTIPTTYVDAGSVFGKTGDAEKVLNGGWNYLMETFNSYANPGYGAMLRANDAMGSDV

VLNSKYGFRTHNEFSAIYGKGGTNTLSWLLAYRVINDCNGVLDNIDAAEGTQADRNRIKG
QALALRGFLYLHLASCYSFAIDKDPDAVCAPIYTQSTDETIAAEGKPASSVSEVYAQSINDLE
EALELIPETYVRDAKHKIDNEVVLGILSRACLYARQWEKAKTYSDKLLAKDNYLMTESEYK
AGFNSVDNKEWIWGHAQTNDQSNASYQFHYLDTTTKGSYYYSFNVDPYFRDLFEDGDYR
KEMLFWATDPGADVESAAYVWMRNSKFRFRDIENQLGDIVLMRVAEIYLINAEAKAHLND
PDAINKLNDLKTARGAKTIHTNLSQQDLLETIWLERRKELWGEGFSLIDIIRNQQTVVRNAY
PEGPIDYIYTDENGQTHTLKKKTQGHRFFNFPDKSAFCPNSKYYLYRITDSEELANKNLYKD
HPKLSIYTK
11111111111111000000000000000000000000000000000000000000000000000000000000000000000
00000000000000000000000000000000000000000000000000000000000000000000000010000000000
00000000000000000000000000000000000000000000000000000000000000000000000000000000000
00000000000000000000000000000000000000000000000000000000000000000000000000000000000
00000000000000000000000000000000000000000000000000000000000000000000000000000000000
0000000000000000000111000000000000000000000000000000000000000000000000000000000
>P56680_1ojk_1_401
KPGETKEVHPQLTTFRCTKRGGCKPATNFIVLDSLSHPIHRAEGLGPGGCGDWGNPPPKDV
CPDVESCAKNCIMEGIPDYSQYGVTTNGTSLRLQHILPDGRVPSPRVYLLDKTKRRYEMLHL
TGFEFTFDVDATKLPCGMNSALYLSEMHPTGAKSKYNPGGAYYGTGYCDAQCFVTPFING
LGNIEGKGSCCNEMDIWEANSRASHVAPHTCNKKGLYLCEGEECAFEGVCDKNGCGWN
NYRVNVTDYYGRGEEFKVNTLKPFTVVTQFLANRRGKLEKIHRFYVQDGKVIESFYTNKEG
VPYTNMIDDEFCEATGSRKYMELGATQGMGEALTRGMVLAMSIWWDQGGNMEWLDHG
EAGPCAKGEGAPSNIVQVEPFPEVTYTNLRWGEIGSTYQELQ
00000000000000000000000000000000000000000000000000000000000000000000000000000000000
00000000000000000000000000000000000000000000000000000000000000000000000000000000000
00000000000000000000000000000000000000000000000000000000000000000000000000000000000
00000000000000000000000000000000000000000000000000000000000000000000000000000000000
000000000000000000000000000000000000000000000000000000000000000000111
>Q4FX34_4h51_0_411
MSTQAAMTTAERWQKIQAQAPDVIFDLAKRAAAAKGPKANLVIGAYRDEQGRPYPLRVV
RKAEQLLLDMNLDYEYLPISGYQPFIDEAVKIIYGNTVELENLVAVQTLSGTGAVSLGAKLLT
RVFDAETTPIYLSDPTWPNHYGVVKAAGWKNICTYAYYDPKTVSLNFEGMKKDILAAPDG
SVFILHQCAHNPTGVDPSQEQWNEIASLMLAKHHQVFFDSAYQGYASGSLDTDAYAARLF
ARRGIEVLLAQSFSKNMGLYSERAGTLSLLLKDKTKRADVKSVMDSLIREEYTCPPAHGARL
AHLILSNNELRKEWEAELSAMAERIRTMRRTVYDELLRLQTPGSWEHVINQIGMFSFLGLS
KAQCEYCQNHNIFITVSGRANMAGLTHETALMLAQTINDAVRNVNRE
11111100000000000000000000000000000000000000000000000000000000000000000000000000000
00000000000000000000000000000000000000000000000000000000000000000000000000000000000
00000000000000000000000000000000000000000000000000000000000000000000000000000000000
00000000000000000000000000000000000000000000000000000000000000000000000000000000000
0000000000000000000000000000000000000000000000000000000000000000000000000000111
>O06961_2e1z_1_401
NEFPVVLVINCGSSSIKFSVLDVATCDVLMAGIADGMNTENAFLSINGDKPINLAHSNYED
ALKAIAFELEKRDLTDSVALIGHRIAHGGELFTQSVIITDEIIDNIRRVSPLAPLHNYANLSGID
AARHLFPAVRQVAVFDTSFHQTLAPEAYLYGLPWEYFSSLGVRRYGFHGTSHRYVSRRAYEL
LDLDEKDSGLIVAHLGNGASICAVRNGQSVDTSMGMTPLEGLMMGTRSGDVDFGAMAWI
AKETGQTLSDLERVVNKESGLLGISGLSSDLRVLEKAWHEGHERARLAIKTFVHRIARHIAG
HAASLHRLDGIIFTGGIGENSVLIRQLVIEHLGVLGLTLDVEMNKQPNSHGERIISANPSQVI
CAVIPTNEEKMIALDAIHLGNVKAPVEFA
11000000000000000000000000000000000000000000000000000000000000000000000000000000000
00000000000000000000000000000000000000000000000000000000000000000000000000000000000
00000000000000000000000000000000000000000000000000000000000000000000000000000000000

00000000000000000000000000000000000000000000000000000000000000000000000000000000000
000000000000000000000000000000000000000000000000000000000000000011111
>P26214_1czf_0_361
MHSFASLLAYGLVAGATFASASPIEARDSCTFTTAAAAKAGKAKCSTITLNNIEVPAGTTLDL
TGLTSGTKVIFEGTTTFQYEEWAGPLISMSGEHITVTGASGHLINCDGARWWDGKGTSGKK
KPKFFYAHGLDSSSITGLNIKNTPLMAFSVQANDITFTDVTINNADGDTQGGHNTDAFDV
GNSVGVNIIKPWVHNQDDCLAVNSGENIWFTGGTCIGGHGLSIGSVGDRSNNVVKNVTIE
HSTVSNSENAVRIKTISGATGSVSEITYSNIVMSGISDYGVVIQQDYEDGKPTGKPTNGVTIQ
DVKLESVTGSVDSGATEIYLLCGSGSCSDWTWDDVKVTGGKKSTACKNFPSVASC
11111111111111111111111111100000000000000000000000000000000000000000000000000000000
00000000000000000000000000000000000000000000000000000000000000000000000000000000000
00000000000000000000000000000000000000000000000000000000000000000000000000000000000
00000000000000000000000000000000000000000000000000000000000000000000000000000000000
00000000000000000000000000000
>Q2M1P5_4a14_7_346
LPGAEEAPVRVALRVRPLLPKELLHGHQSCLQVEPGLGRVTLGRDRHFGFHVVLAEDAGQ
EAVYQACVQPLLEAFFEGFNATVFAYGQTGSGKTYTMGEASVASLLEDEQGIVPRAMAEAF
KLIDENDLLDCLVHVSYLEVYKEEFRDLLEVGTASRDIQLREDERGNVVLCGVKEVDVEGL
DEVLSLLEMGNAARHTGATHLNHLSSRSHTVFTVTLEQRGRAPSRLPRPAPGQLLVSKFHF
VDLAGSERVLKTGSTGERLKESIQINSSLLALGNVISALGDPQRRGSHIPYRDSKITRILKDSL
GGNAKTVMIACVSPSSSDFDETLNTLNYASRAQ
11110000000000000000000000000000000000000000000000000000000000000000000000000000000
00000000000000001111111100000000000000000000000000000000000000000000000000000000000
00000000000000000000000000000000111111000000000000000000111111111111000000000000000
00011111111111110000000000000000000000000000000000000000000000000000000000000000000
00000000
>P20582_3h78_1_336
GNPILAGLGFSLPKRQVSNHDLVGRINTSDEFIVERTGVRTRYHVEPEQAVSALMVPAARQ
AIEAAGLLPEDIDLLLVNTLSPDHHDPSQACLIQPLLGLRHIPVLDIRAQCSGLLYGLQMAR
GQILAGLARHVLVVCGEVLSKRMDCSDRGRNLSILLGDGAGAVVVSAGESLEDGLLDLRL
GADGNYFDLLMTAAPGSASPTFLDENVLREGGGEFLMRGRPMFEHASQTLVRIAGEMLAA
HELTLDDIDHVICHQPNLRILDAVQEQLGIPQHKFAVTVDRLGNMASASTPVTLAMFWPDI
QPGQRVLVLTYGSGATWGAALYRKPEEVNRPC
00000000000000000000000000000000000000000000000000000000000000000000000000000000000
00000000000000000000000000000000000000000000000000000000000000000000000000000000000
00000000000000000000000000000000000000000000000000000000000000000000000000000000000
00000000000000000000000000000000000000000000000000000000000000000000000000000000111
1111
>Q8NT37_3lmd_1_349
SSGRTVPTRSHGLGKEGVSTTGASQVEFGDPELTARINDAMVQVEELLHTELSSGEDFLVDI
VMHLTRAGGKRFRPMFALLASEFGEKPLSENVIKAAVVVEITHLATLYHDDVMDEASMRR
GVPSANARWDNSVAILAGDILLAHASGLMSQLGTDTVAHFAETFGELVTGQMRETVGPRD
TDPIEHYTNVIREKTGVLIASAGYLGAMHAGAAPEHIDALKNFGAAVGMIFQIVDDIIDIFS
ETHESGKTPGTDLREGVFTLPVLYALREDTPVGAELRDILTGPLEDDETVNHVLELLSQSGG
RQAALDEVYRYMDIANAELDRLPDSTVKEALRNLATFTVKRVG
11111111111111110000000000000000000000000000000000000000000000000000000000000000000
00000000000000000000000000000000011111111110000000000000000000000000000000000000000
00000000000000000000000000000000000000000000000000000000000000000000000000000011111
11100000000000000000000000000000000000000000000000000000000000000000000000000000000
00000000000000000
>Q8P8J2_3kzo_0_338

MSLKHFLNTQDWSRAELDALLTQAALFKRNKLGSELKGKSIALVFFNPSMRTRTSFELGAF
QLGGHAVVLQPGKDAWPIEFNLGTVMDGDTEEHIAEVARVLGRYVDLIGVRAFPKFVDWS
KDREDQVLKSFAKYSPVPVINMETITHPCQELAHALALQEHFGTPDLRGKKYVLTWTYHP
KPLNTAVANSALTIATRMGMDVTLLCPTPDYILDERYMDWAAQNVAESGGSLQVSHDIDS
AYAGADVVYAKSWGALPFFGNWEPEKPIRDQYQHFIVDERKMALTNNGVFSHCLPLRRN
VKATDAVMDSPNCIAIDEAENRLHVQKAIMAALVGQSRP
11000000000000000000000000000000000000000000000000000000000000000000000000000000000
00000000000000000000000000000000000000000000000000000000000000000000000000000000000
00000000000000000000000000000000000000000000000000000000000000000000000000000000000
00000000000000000000000000000000000000000000000000000000000000000000000000000000000
0011111
>Q8Y8T4_4gl0_27_356
SEGYAGSNTLTIYNWGDYIDPSLITKFEKETGIKVIYQTFDSNEAMMTKIEQGGTTFDIAVPS
DYAISKMKEENLLIPLDHSKLPNEKYLDPRFMDLSFDDDNKYSMPYFWGTLGIIYNKEMFP
DKNFDTWNALFDPELKNQILLIDGAREVMGLGLNSLGYSLNDTNKAHLQAARDKLETMT
PNVKAIVGDEIKLLMADNEAGVAVTFSGEAAEMLSENEDLEYVIPKDGSNLWFDNMVIPK
TAKNVDGAHKFINFMLKPENAAINAEYVGYATPNAKAVELLPKEISSDERFYPDMDELNN
LEVYDNLGKRMLSYYNELFLEFKMYRK
11111100000000000000000000000000000000000000000000000000000000000000000000000000000
00000000000000000000000000000000000000000000000000000000000000000000000000000000000
00000000000000000000000000000000000100000000000000000000000000000000000000000000000
000000000000000000000000000000000000000000000000000000000000000000000000000000001
>Q8TTZ5_3k6v_26_351
DNQPEPGNTSAGEGEVLTVFHAGSLSVPFEELEAEFEAQHPGVDVQREAAGSAQSVRKITE
LGKKADVLASADYALIPSLMVPEYADWYAAFARNQMILAYTNESKYGDEINTDNWYEILR
RPDVRYGFSNPNDDPAGYRSQMVTQLAESYYNDDMIYDDLMLANTGMTLTTEENGTALI
HVPASEEISPNTSKIMLRSMEVELSSALETGEIDYLYIYRSVAEQHGFEYVALPPAIDLSSLEYA
DNYSKVQVEMVNGEVVTGSPIVYGVTIPNNAENSELATEFVALLLGETGQQIFIENGQPPIV
PAIAEGKDSMPEELQALVV
11111111111111000000000000000000000000000000000000000000000000000000000000000000000
00000000000000000000000000000000000000000000000000000000000000000000000000000000000
00000000000000000000000000000000000000000000000000000000000000000000000000000000000
00000000000000000000000000000000000000000000000000000000000000000000000000000
>P83749_1rj9_0_303
MGFFDRLKAGLAKTRERLLKAIPWGGNLEEVLEELEMALLAADVGLSATEEILQEVRASGR
KDLKEAVKEKLVGMLEPDERRATLRKLGFNPQKPKPVEPKGRVVLVVGVNGVGKTTTIAK
LGRYYQNLGKKVMFCAGDTFRAAGGTQLSEWGKRLSIPVIQGPEGTDPAALAYDAVQAM
KARGYDLLFVDTAGRLHTKHNLMEELKKVKRAIAKADPEEPKEVWLVLDAVTGQNGLEQ
AKKFHEAVGLTGVIVTKLDGTAKGGVLIPIVRTLKVPIKFVGVGEGPDDLQPFDPEAFVEAL
LED
11111111111111111111111111000000000000000000000000000000000000000000000000000000000
00000000000000000000000000000000000000000000000000000000000000000000000000000000000
00000000000000000000000000000000000000000000000000000000000000000000000000000000000
0000000000000000000000000000000000000000000000000000001
>Q9Z4N6_1q35_22_341
ANEVNVYSYRQPYLIEPMLKNFEKDTGIKVNIIFADKGLVDRVKQEGELSPADVLLTVDISR
VMEIVNADLAQKIDSKVLEKNIPAQFRDSNDQWFGLTTRARVIYTSKDRVGKLPAGFDYLD
LAKPEYKGKVCVRSGKNSYNVSLFAAMIEHYGIEKTKAFLEGLKANLARKPQGGDRDQVK
AIKEGICDYSIGNSYYYGKMLDDEKQKSWAEAAIINFPSGEHGTHKNISGVVIAKHSPNKA
NAVKLIEYLSGEKAQGLYAELNHEYPVKEGIEPSAIVKGWGTFKSDTIKLEDIAKNYEAALK
LVDEVKFDDFSEKK

0000000000000000000000000000000000001000000000000000000000000000000000000000000000000
00000000000000000000000000000000000000000000000000000000000000000000000000000000000
00000000000000000000000000000000000000000000000000000000000000000000000000000000000
00000000000000000000000000000000000000000000000000000000000000000000011
>O80358_3twl_0_303
MPELPEVEAARRAIEENCLGKKIKRVIIADDNKVIHGISPSDFQTSILGKTIISARRKGKNLWL
ELDSPPFPSFQFGMAGAIYIKGVAVTKYKRSAVKDSEEWPSKYSKFFVELDDGLELSFTDKRR
FAKVRLLANPTSVSPISELGPDALLEPMTVDEFAESLAKKKITIKPLLLDQGYISGIGNWIADE
VLYQARIHPLQTASSLSKEQCEALHTSIKEVIEKAVEVDADSSQFPSYWIFHNREKKPGKAFV
DGKKIDFITAGGRTTAYVPELQKLYGKDAEKAAKVRPAKRGVKPKEDDGD
10000000000000000000000000000000000000000000000000000000000000000000000000000000000
00111111111111111110000000000000000000000000000000000000000000000000000000000000000
00000000000000000000000000000000000000000000000000000000000000000000000000000000000
0000000000000111111000000000000000000011111111111111111
>Q1JU72_1y37_0_303
MFEGFERRLVDVGDVTINCVVGGSGPALLLLHGFPQNLHMWARVAPLLANEYTVVCADL
RGYGGSSKPVGAPDHANYSFRAMASDQRELMRTLGFERFHLVGHDRGGRTGHRMALDH
PDSVLSLAVLDIIPTYVMFEEVDRFVARAYWHWYFLQQPAPYPEKVIGADPDTFYEGCLFG
WGATGADGFDPEQLEEYRKQWRDPAAIHGSCCDYRAGGTIDFELDHGDLGRQVQCPALV
FSGSAGLMHSLFEMQVVWAPRLANMRFASLPGGHFFVDRFPDDTARILREFLSDARSGIHQ
TERRES
00000000000000000000000000000000000000000000000000000000000000000000000000000000000
00000000000000000000000000000000000000000000000000000000000000000000000000000000000
00000000000000000000000000000000000000000000000000000000000000000000000000000000000
0000000000000000000000000000000000000000000001111111111
>Q7CWE5_2h8o_0_303
MDAQMTNFETRLRENAAKTEALLGHLLSGEARADEITRPQNLLEAMRHGVLNGGKRLRP
FLVIESVALLGGDAEAGLHVGAALECLHCYSLVHDDLPAMDDDDLRRGQPTVHRKFDEAT
AILAGDSLLTLAFDIIASDDNPLAAERKAALVISLARAAGIGGMAGGQALDLAAEKKAPDE
DGIITLQAMKTGALLRFACEAGAIIAGSNQAERQRLRLFGEKIGLSFQLADDLLDLTADAAT
MGKATGKDAARGKGTLVALRGEAWAREKLQEQVAEASELLAPYGEKAAILIAAARFIAER
KS
10000000000000000000000000000000000000000000000000000000000000000000000000000000000
00000000000000000000000000000000000000000000000000000000000000000000000000000000000
00000000000000000000000000000000000000000000000000000000000000000000000111111111111
1111100000000000000000000000000000000000000000000000111
>Q62645_3oen_423_826
DDTQHLTVATLEERPFVIVEPADPISGTCIRDSVPCRSQLNRTHSPPPDAPRPEKRCCKGFCID
ILKRLAHTIGFSYDLYLVTNGKHGKKIDGVWNGMIGEVFYQRADMAIGSLTINEERSEIVDF
SVPFVETGISVMVARVDTVSGLSDRKFQRPQEQYPPLKFGTVPNGSTEKNIRSNYPDMHSY
MVRYNQPRVEEALTQLKAGKLDAFIYDAAVLNYMARKDEGCKLVTIGSGKVFATTGYGIA
LHKGSRWKRPIDLALLQFLGDDEIEMLERLWLSGICHN
11000000000000000000000000000000000000001111111111000000000000000000000000000000000
00000000000000000000000000000000000000000000000000000000001111111111111111111100000
00000000000000000000000000000000000000000000000000000000000000000000000000000000000
000000000000000000000000000000000001
>P31663_1iho_0_282
MLIIETLPLLRQQIRRLRMEGKRVALVPTMGNLHDGHMKLVDEAKARADVVVVSIFVNPM
QFDRPEDLARYPRTLQEDCEKLNKRKVDLVFAPSVKEIYPNGTETHTYVDVPGLSTMLEGA
SRPGHFRGVSTIVSKLFNLVQPDIACFGEKDFQQLALIRKMVADMGFDIEIVGVPIMRAKDG
LALSSRNGYLTAEQRKIAPGLYKVLSSIADKLQAGERDLDEIITIAGQELNEKGFRADDIQIR

DADTLLEVSETSKRAVILVAAWLGDARLIDNKMVELA
00000000000000000000000000000000000000000000000000000000000000000000000000000000
00000000000000000000000000000000000000000000000000000000000000000000000000000000
00000000000000000000000000000000000000000000000000000000000000000000000000000000
000000000000000000000000000000001
>D0VWZ1_3hkx_0_262
MRIALMQHTARPLDPQHNLDLIDDAAARASEQGAQLLLTPELFGFGYVPSQICAQVSAEQ
VDAARSRLRGIARDRGIALVWSLPGPEGPEQRGITAELADEHGEVLASYQKVQLYGPEEKA
AFVPGEQPPPVLSWGGRQLSLLVCYDVEFPEMVRAAAARGAQLVLVPTALAGDETSVPGIL
LPARAVENGITLAYANHCGPEGGLVFDGGSVVVGPAGQPLGELGVEPGLLVVDLPDQSQD
AGSDSADYLQDRRAELHRNWL
00000000000000000000000000000000000000000000000000000000000000000000000000000000
00000000000000000000000000000000000000000000000000000000000000000000000000000000
00000000000000000000000000000000000000000000000000000000000000000000001111111110
00000000000000
>O32125_3pdw_1_255
KTYKGYLIDLDGTMYNGTEKIEEACEFVRTLKDRGVPYLFVTNNSSRTPKQVADKLVSFDIP
ATEEQVFTTSMATAQHIAQQKKDASVYVIGEEGIRQAIEENGLTFGGENADFVVVGIDRSIT
YEKFAVGCLAIRNGARFISTNGDIAIPTERGLLPGNGSLTSVLTVSTGVQPVFIGKPESIIMEQ
AMRVLGTDVSETLMVGDNYATDIMAGINAGMDTLLVHTGVTKREHMTDDMEKPTHAID
SLTEWIPYI
00000000000000000000000000000000000000000000000000000000000000000000000000000000
00000000000000000000000000000000000000000000000000000000000000000000000000000000
00000000000000000000000000000000000000000000000000000000000111111000000000000000
000000
>Q7RMC9_2a4a_0_261
MANYTEKFAAWSVICLTDHTFLDENGTEDDIRELCNESVKTCPFAAAVCVYPKFVKFINEKI
KQEINPFKPKIACVINFPYGTDSMEKVLNDTEKALDDGADEIDLVINYKKIIENTDEGLKEA
TKLTQSVKKLLTNKILKVIIEVGELKTEDLIIKTTLAVLNGNADFIKTSTGKVQINATPSSVEYII
KAIKEYIKNNPEKNNKIGLKVSGGISDLNTASHYILLARRFLSSLACHPDNFRIGSSSLVIKLR
KVISQCPL
11000000000000000000000000000000000000000000000000000000000000000000000000000000
00000000000000000000000000000000000000000000000000000000000000000000000000000000
00000000000000000000000000000000000000000000000000000000000000011111100000000000
0000000001111
>P63590_2ocz_0_227
MRIVAPVMPRHFDEAQAIDISKYEDVNLIEWRADFLPKDEIVAVAPAIFEKFAGKEIIFTLRTV
QEGGNITLSSQEYVDIIKEINAIYNPDYIDFEYFTHKSVFQEMLDFPNLILSYHNFEETPENLM
EAFSEMTKLAPRVVKIAVMPQSEQDVLDLMNYTRGFKTLNPEQEFATISMGKLGRLSRFAG
DVIGSSWTYVSLDHVSGPGQVTLNDMKRIIEVLEMDISN
00000000000000000000000000000000000000000000000000000000000000000000000000000000
00000000000000000000000000000000000000000000000000000000000000000000000000000000
00000000000000000000000000000000011111100000000000000011111
>C7B6Z3_3m19_0_249
KTCETVTGCTCNEGKKEVDCQGKSLDSVPSGIPADTEKLDLQSTGLATLSDATFRGLTKLTW
LNLDYNQLQTLSAGVFDDLTELGTLGLANNQLASLPLGVFDHLTQLDKLYLGGNQLKSLP
SGVFDRLTKLKELRLNTNQLQSIPAGAFDKLTNLQTLSLSTNQLQSVPHGAFDRLGKLQTIT
LFGNQFDCSRCEILYLSQWIRENSNKVKDGTGQNLHESPDGVTCSDGKVVRTVTNETLKYE
CPFVE
11000000000000000000000000000000000000000000000000000000000000000000000000000000
00000000000000000000000000000000000000000000000000000000000000000000000000000000

00000000000000000000000000000000000000000000000111111100000000000000000000000000111
1
>Q56691_1bkj_0_239
MNNTIETILAHRSIRKFTAVPITDEQRQTIIQAGLAASSSSMLQVVSIVRVTDSEKRNELAQFA
GNQAYVESAAEFLVFCIDYQRHATINPDVQADFTELTLIGAVDSGIMAQNCLLAAESMGLG
GVYIGGLRNSAAQVDELLGLPENSAVLFGMCLGHPDQNPEVKPRLPAHVVVHENQYQEL
NLDDIQSYDQTMQAYYASRTSNQKLSTWSQEVTGKLAGESRPHILPYLNSKGLAKR
10000000000000000000000000000000000000000000000000000000000000000000000000000000000
00000000000000000000000000000000000000000000000000000000000000000000000000000000000
00000000000000000000000000000000001111111100000000000000000000000000000000
>O84385_3del_23_256
CLKEGGDSNSEKFIVGTNATYPPFEFVDKRGEVVGFDIDLAREISNKLGKTLDVREFSFDALI
LNLKQHRIDAVITGMSITPSRLKEILMIPYYGEEIKHLVLVFKGENKHPLPLTQYRSVAVQTGT
YQEAYLQSLSEVHIRSFDSTLEVLMEVMHGKSPVAVLEPSIAQVVLKDFPALSTATIDLPEDQ
WVLGYGIGVASDRPALALKIEAAVQEIRKEGVLAELEQKWGLNN
11111111110000000000000000000000000000000000000000000000000000000000000000000000000
00000000000000000000000000000000000000000000000000000000000000000000000000000000000
00000000000000000000000000000000000000000000000000000000000000000000
>P46107_3ro0_0_214
MEKKVLLTGFDPFGGETVNPSWEAVKRLNGAAEGPASIVSEQVPTVFYKSLAVLREAIKKH
QPDIIICVGQAGGRMQITPERVAINLNEARIPDNEGNQPVGEDISQGGPAAYWTGLPIKRIV
EEIKKEGIPAAVSYTAGTFVCNHLFYGLMDEISRHHPHIRGGFIHIPYIPEQTLQKSAPSLSLD
HITKALKIAAVTAAVHEDDIETGGGELH
00000000000000000000000000000000000000000000000000000000000000000000000000000000000
00000000000000000000000000000000000000000000000000000000000000000000000000000000000
0000000000000000000000000000000000000000001111111
>Q8ZE60_4ese_0_200
MSKVLVLKSSILATSSQSNQLADFFVEQWQAAHAGDQITVRDLAAQPIPVLDGELVGALRP
SGTALTPRQQEALALSDELIAELQANDVIVIAAPMYNFNIPTQLKNYFDMIARAGVTFRYTE
KGPEGLVTGKRAIILTSRGGIHKDTPTDLVVPYLRLFLGFIGITDVEFVFAEGIAYGPEVATKA
QADAKTLLAQVVAA
00000000000000000000000000000000000000000000000000000000000000000000000000000000000
00000000000000000000000000000000000000000000000000000000000111111110000000000000000
00000000000000000000000000000000001
>P37528_2nv0_0_195
MLTIGVLGLQGAVREHIHAIEACGAAGLVVKRPEQLNEVDGLILPGGESTTMRRLIDTYQF
MEPLREFAAQGKPMFGTCAGLIILAKEIAGSDNPHLGLLNVVVERNSFGRQVDSFEADLTI
KGLDEPFTGVFIRAPHILEAGENVEVLSEHNGRIVAAKQGQFLGCSFHPELTEDHRVTQLFV
EMVEEYKQKALV
00000000000000000000000000000000000000000000000000000000000000000000000000000000000
00000001110000000000000000000000000000000000000000000000000000000000000000000000000
000000000000000000000000000001
>Q72G28_3hu5_1_193
TRNRTVALAIIDMQNDFVLPGAPACVEGAMGTVPVIAGLLAKARAEGWMVLHVVRAHR
ADGSDAEKSREHLFLEGGGLCVAGTPGAEIVAGLEPASGETVLVKTRFSAFMGTECDMLLR
RRGVDTLLVSGTQYPNCIRGTAVDAFALDYDVVVVTDACSARTPGVAESNINDMRAMGIT
CVPLTALDDVLARR
11000000000000000000000000000000000000000000000000000000000000000000000000000000000
00000000000000000000000000000000000000000000000000000000000000000000000000000000000
000000000000000000000000001
>Q9SSV1_1vcz_23_235

AQDFDFFYFVQQWPASYCDTRRSCCYPTTGKPDEDFSIHGLWPNYENGKWPQNCDRESSL
DESEISDLISTMEKNWPSLACPSSDGVRFWSHEWLKHGTCSALGERAYFQAALDFRKKSNL
LENLKNAEITPRNGEHYTLESIKKAIEEGVGHSPYIECNVDTQGNHQIYQVYLCVDKTATDF
IDCPIFPHGRGCGSKIEFPPFSSESDHDEF
00000000000000000000000000000000000000000000000000000000000000000000000000000000000
00000000000000000000000000000000000000000000000000000000000000000000000000000000000
00000000000000000000000000000000000001111111111
>O57413_1kuf_189_391
EQQRFPQRYIKLAIVVDHGMYTKYSSNFKKIRKRVHQMVSNINEMCRPLNIAITLALLDVW
SEKDFITVQADAPTTAGLFGDWRERVLLKKKNHDHAQLLTDTNFARNTIGWAYVGRMCD
EKYSVAVVKDHSSKVFMVAVTMTHELGHNLGMEHDDKDKCKCDTCIMSAVISDKQSKLFS
DCSKDYYQTFLTNDNPQCILNAP
11000000000000000000000000000000000000000000000000000000000000000000000000000000000
00000000000000000000000000000000000000000000000000000000000000000000000000000000000
0000000000000000000000000000000000000
>Q9WY06_1vp2_0_195
MKKLTVYLATTNPHKVEEIKMIAPEWMEILPSPEKIEVVEDGETFLENSVKKAVVYGKKLK
HPVMADDSGLVIYSLGGFPGVMSARFMEEHSYKEKMRTILKMLEGKDRRAAFVCSATFFD
PVENTLISVEDRVEGRIANEIRGTGGFGYDPFFIPDGYDKTFGEIPHLKEKISHRSKAFRKLFS
VLEKILESENR
11000000000000000000000000000000000000000000000000000000000000000000000000000000000
00000000000000000000000000000000000000000000000000000000000000000000000000000000000
000000000000000000000000011111
>Q869A5_3dwv_1_175
LRSSRKKMSAASSIFDFEVLDADHKPYNLVQHKGSPLLIYNVASKCGYTKGGYETATALYNK
YKSQGFTVLAFPCNQFGGQEPGTEEEIKEFVCTKFKAEFPIMAKINVNGENAHPLYEYMKK
TKPGILATKAIKWNFTSFLIDRDGVPVERFSPGASVKDIEEKLIPLLGSARL
11111110000000000000000000000000000000000000000000000000000000000000000000000000000
00000000000000000000000000000000000000000000000000000000000000000000000000000000000
000011111
>P09237_2y6d_94_266
YSLFPNSPKWTSKVVTYRIVSYTRDLPHITVDRLVSKALNMWGKEIPLHFRKVVWGTADIMI
GFARGAHGDSYPFDGPGNTLAHAFAPGTGLGGDAHFDEDERWTDGSSLGINFLYAATHEL
GHSLGMGHSSDPNAVMYPTYGNGDPQNFKLSQDDIKGIQKLYGKRSNSRKK
00000000000000000000000000000000000000000000000000000000000000000000000000000000000
00000000000000000000000000000000000000000000000000000000000111111000000000000000001
1111111
>Q16763_1zdn_0_155
MNSNVENLPPHIIRLVYKEVTTLTADPPDGIKVFPNEEDLTDLQVTIEGPEGTPYAGGLFRM
KLLLGKDFPASPPKGYFLTKIFHPNVGANGEICVNVLKRDWTAELGIRHVLLTIKCLLIHPN
PESALNEEAGRLLLENYEEYAARARLLTEIHG
11111000000000000000000000000000000000000000000000000000000000000000000000000000000
0000000000000000000000000000000000000000000000000000000000000000000000000
>Q381H3_4fky_0_152
MPSERTFIAVKPDGVQRNLVGEIIKRFENKGYKLVGLKLLQPTEEQAKQHYIDLASKPFYSG
LVSYFSSGPIVGMVWEGLGVVKGGRVLLGATNPADSLPGTIRGDFAVDVGRNVCHGSDSVE
SAKREIAFWFKAEELVSWTSHSVKQIYERA
00000000000000000000000000000000000000000000000000000000000000000000000000000000000
0000000000000000000000000000000000000000000000000000000000000000000001
>A0R291_3o0m_0_144
MSCVFCAIVSGDAPAIRIYEDENFLGILDIRPFTRGHTLVIPKTHTVDLTDTPPETVAGMAAV

GQRIARAARESGLHADGNNIAINDGKAAFQTVFHIHLHVVPRRNGDKLSFAKGMVMRRD
PDREESGRLLRAALAQLDSAEQD
00000000000000000000000000000000000000000000000000000000000000000000000000000000000
00000000000000000000000000000011111100000000000000000000000011
>P0A6T9_3a8k_0_128
MSNVPAELKYSKEHEWLRKEADGTYTVGITEHAQELLGDMVFVDLPEVGATVSAGDDCA
VAESVKAASDIYAPVSGEIVAVNDALSDSPELVNSEPYAGGWIFKIKASDESELESLLDATAYE
ALLEDE
11000000000000000000000000000000000000000000000000000000000000000000000000000000000
0000000000000000000000000000000000000000000001
>A2W1I5_2vnv_0_128
MADSQTSSNRAGEFSIPPNTDFRAIFFANAAEQQHIKLFIGDSQEPAAYHKLTTRDGPREAT
LNSGNGKIRFEVSVNGKPSATDARLAPINGKKSDGSPFTVNFGIVVSEDGHDSDYNDGIVV
LQWPIG
10000000000000000000000000000000000000000000000000000000000000000000000000000000000
0000000000000000000000000000000000000000000000
>Q9GZQ8_2zjd_0_124
MPSEKTFKQRRTFEQRVEDVRLIREQHPTKIPVIIERYKGEKQLPVLDKTKFLVPDHVNMSEL
IKIIRRRLQLNANQAFFLLVNGHSMVSVSTPISEVYESEKDEDGFLYMVYASQETFGMKLSV
00000000000000000000000000000000000000000000000000000000000000000000000000000000000
000000000000000000000000000000000000000111
>Q15599_2ocs_8_90
PRLCRLVRGEQGYGFHLHGEKGRRGQFIRRVEPGSPAEAAALRAGDRLVEVNGVNVEGET
HHQVVQRIKAVEGQTRLLVVDQE
00000000000000000001110000000000000000000000000000000000000000000000000000000000000
>Q74ED8_3h4n_20_91
HDKVVVLEAKNGNVTFDHKKHAGVKGECKACHETEAGGKIAGMGKDWAHKTCTGCH
KEMGKGPTKCGECHKK
110000000000000000000000000000000000000000000000000000000000000000000001
>Q9Y5K6_3u23_108_167
KKRQCKVLFEYIPQNEDELELKVGDIIDINEEVEEGWWSGTLNNKLGLFPSNFVKELEVT
110000000000000000000000000000000000000000000000000000000011
>Q5NF74_3kom_0_662
MSLSIPREFSNAIRFLSIDATLKAKSGHPGMPMGMADIATVLWTKFLKHNPNNPHWINRDR
FVLSNGHGSMLLYSLLHLTGYDLSIEDIKNFRQLHSKTPGHPEYGYTPGVETTTGPLGQGVA
NAVGMALGEKLLSDRYNTPDLKVIDHHTYVFLGDGCLMEGVSHEACSLAGTLGLNKLVAF
WDDNNISIDGDTKGWFSDNTPERFRAYGWHVIENVDGHDFVAIEKAINEAHSQQQKPTLI
CCKTVIGFGSPEKAGTASVHGSPLSDQERASAAKELNWDYQAFEIPQDVYKYWDAREKGQ
ALEANWQGQWNLFKDSPKFDEFERVLSKELPVGLESAINDYIASQLSNPVKVATRKASQM
VLEVLCKNMPEMFGGSADLTGSNNTNWSGSVWLNNTQEGANYLSYGVREFGMAAIMNG
LSLYGGIKPYGGTFLVFSDYSRNAIRMSALMKQPVVHVMSHDSIGLGEDGPTHQPIEHVPSL
RLIPNLSVWRPADTIETMIAWKEAVKSKDTPSVMVLTRQNLMPVVQTQHQVANIARGGYL
VKDNPDAKLTIVATGSEVELAVKVANEFEKKGIKLNVASIPCVEVFATQAHEYKKTVIKDDI
PAVFVEMAQPDMWYKYMPKAGGEVKGIYSFGESAPAEDLFKRFGFTVENISNIVAKYV
11000000000000000000000000000000000000000000000000000000000000000000000000000000000
00000000000000000000000000000000000000000000000000000000000000000000000000000000000
00000000000000000000001111100000000000000000000000000000000000000000000000000000000
00000000000000000000000000000000000000000000000000000000000000000000000000000000000
00000000000000000000000000000000000000000000000000010000000000000000000000000000000
00000000000000000000000000000000000000000000000000000000000000000000000000000000000
00000000000000000000000000000000000000000000000000000000000000000000000000000000000

000000000000000000000000000000000000000000000000000000000000000000000000000000000000000000
>Q8GCC7_2ogt_0_497
MERTVVETRYGRLRGEMNEGVFVWKGIPYAKAPVGERRFLPPEPPDAWDGVREATSFGPV
VMQPSDPIFSGLLGRMSEAPSEDGLYLNIWSPAADGKKRPVLFWIHGGAFLFGSGSSPWYD
GTAFAKHGDVVVVTINYRMNVFGFLHLGDSFGEAYAQAGNLGILDQVAALRWVKENIAA
FGGDPDNITIFGESAGAASVGVLLSLPEASGLFRRAMLQSGSGSLLLRSPETAMAMTERILD
KAGIRPGDRERLLSIPAEELLRAALSLGPGVMYGPVVDGRVLRRHPIEALRYGAASGIPILIG
VTKDEYNLFTLTDPSWTKLGEKELLDRINREVGPVPEEAIRYYKETAEPSAPTWQTWLRIMT
YRVFVEGMLRTADAQAAQGADVYMYRFDYETPVFGGQLKACHALELPFVFHNLHQPGV
ANFVGNRPEREAIANEMHYAWLSFARTGDPNGAHLPEAWPAYTNERKAAFVFSAASHVE
DDPFGRERAAWQGR
1100000000000000000000000000000000000000000000000000000000000000000000000111111111111110000
0000000000000000000000000000000000000000000000000000000000000000000000000000000000000000000
0000000000000000000000000000000000000000000000000000000000000000000000000000000000000000000
0000000000000000000000000000000000000000000000000000000000000000000000000000000000000000000
0000000000000000111111111100000000000000000000000000000000000000000000000000111111100000000
0000000000000000000000000000000000000000000000000000000000000000000000000000000000000000011
>Q13WK3_2v7b_0_528
MEALLEKAANPPAATVEAPPALFNFAAYLFRLNETRAGKTAYIDDTGSTTYGELEERARRFA
SALRTLGVHPEERILLVMLDTVALPVAFLGALYAGVVPVVANTLLTPADYVYMLTHSHARA
VIASGALVQNVTQALESAEHDGCQLIVSQPRESEPRLAPLFEELIDAAAPAAKAAATGCDDI
AFWLYSSGSTGKPKGTVHTHANLYWTAELYAKPILGIAENDVVFSAAKLFFAYGLGNGLTFP
LSVGATAILMAERPTADAIFARLVEHRPTVFYGVPTLYANMLVSPNLPARADVAIRICTSAGE
ALPREIGERFTAHFGCEILDGIGSTEMLHIFLSNRAGAVEYGTTGRPVPGYEIELRDEAGHAV
PDGEVGDLYIKGPSAAVMYWNNREKSRATFLGEWIRSGDKYCRLPNGCYVYAGRSDDML
KVSGQYVSPVEVEMVLVQHDAVLEAAVVGVDHGGLVKTRAFVVLKREFAPSEILAEELKAF
VKDRLAPHKYPRDIVFVDDLPKTATGKIQRFKLREQ
1111111111111111111111100000000000000000000000000000000000000000000000000000000000000000000
0000000000000000000000000000000000000000000000000000000000111000000000111111000000000000000
0000000000000000000000000011111000000000000000000000000000000000000000000000000000000000000
0000000000000000000000000000000000000000000000000000000000000000000000000000000000000000000
0000000000000000000000000000000000000000000000000000000000000000000000000000000000000000000
0000000000000000011110000000000000000000000000000000000000000000000000000000000000000000000
0000000000000000000000000000000001
>P77872_1qwm_0_504
MVNKDVKQTTAFGAPVWDDNNVITAGPRGPVLLQSTWFLEKLAAFDRERIPERVVHAKG
SGAYGTFTVTKDITKYTKAKIFSKVGKKTECFFRFSTVAGERGSADAVRDPRGFAMKYYTEE
GNWDLVGNNTPVFFIRDAIKFPDFIHTQKRDPQTNLPNHDMVWDFWSNVPESLYQVTWV
MSDRGIPKSFRHMDGFGSHTFSLINAKGERFWVKFHFHTMQGVKHLTNEEAAEVRKYDP
DSNQRDLFNAIARGDFPKWKLSIQVMPEEDAKKYRFHPFDVTKIWYLQDYPLMEVGIVEL
NKNPENYFAEVEQAAFSPANVVPGIGYSPDRMLQGRLFSYGDTHRYRLGVNYPQIPVNKP
RCPFHSSSRDGYMQNGYYGSLQNYTPSSLPGYKEDKSARDPKFNLAHIEKEFEVWNWDYR
ADDSDYYTQPGDYYRSLPADEKERLHDTIGESLAHVTHKEIVDKQLEHFKKADPKYAEGV
KKALEKHQKMMKDMHGKDMHHTKKKK
0000000000000000000000000000000000000000000000000000000000000000000000000000000000000000000
0000000000000000000000000000000000000000000000000000000000000000000000000000000000000000000
0000000000000000000000000000000000000000000000000000000000000000000000000000000000000000000
0000000000000000000000000000000000000000000000000000000000000000000000000000000000000000000
0000000000000000000000000000000000000000000000000000000000000000000000000000000000000000000
0000000000000000000000000000000000000000000000000000000000000000000000000000000000011111111
1111111

>Q9ZGI4_3qp9_919_1422
GSVQDSWRYRIDWKRLAVADASERAGLSGRWLVVVPEDRSAEAAPVLAALSGAGADPVQ
LDVSPLGDRQRLAATLGEALAAAGGAVDGVLSLLAWDESAHPGHPAPFTRGTGATLTLVQ
ALEDAGVAAPLWCVTHGAVSVGRADHVTSPAQAMVWGMGRVAALEHPERWGGLIDLPS
DADRAALDRMTTVLAGGTGEDQVAVRASGLLARRLVRASLPAHGTASPWWQADGTVLV
TGAEEPAAAEAARRLARDGAGHLLLHTTPSGSEGAEGTSGAAEDSGLAGLVAELADLGAT
ATVVTCDLTDAEAAARLLAGVSDAHPLSAVLHLPPTVDSEPLAATDADALARVVTAKATA
ALHLDRLLREAAAAGGRPPVLVLFSSVAAIWGGAGQGAYAAGTAFLDALAGQHRADGPT
VTSVAWSPWEGSRVTEGATGERLRRLGLRPLAPATALTALDTALGHGDTAVTIADVDWSSF
APGFTTARPGTLLADLPEARRALDEQQST
11000000000000000011111100000000000000000000000000000000000000000000000000000000000
00000000000000000000000000000000000000000000000000000000000000000000000000000000000
00000000000000000000000000000000000000000000000000001100000000000000000000000000000
00000000000000001111111111111100000000000000000000000000000000000000000000000000000
00000000000000000000000000000000000111100000000000000000000000000000000000000000000
00000000000000000000000000000000000000000000000000000000000000000000000000000000111
111111
>Q0PQZ5_3dc8_0_483
MSTVIKGGTIVTADLTYKADVKVEGGRIVEIGPNLSGAETLDATGCYVMPGGIDPHTHLEM
PFMGTYSSDDFESGTRAALAGGTTMVVDFALPSPGQSLLEALTMWDNKSTRANCDYSFHM
AITWWGEQVFNEMETIVKDKGINTFKHFMAYKGALMVDDDEMFSSFQRCAALGALPLVH
AENGDVVAQLQAKLLAEGNSGPEAHAYSRPAEVEGEAANRAIMIADMAGCPVYIVHTSC
EQAHEAIRRARAKGMRVFGEPLIQHLTLDETEYFDKDWDHAARRVMSPPFRNKLHQDSL
WAGLASGSLQVVATDHCAFTTEQKRFGVGDFTRIPNGTGGLEDRMPMLWTYGVATGRITM
NEFVAVTSTNIAKILNIYPKKGAILVGADADLVVWDPKRSKTISAKTQQSAIDYNVFEGKTV
TGLPRFTLTRGVVSIEEGTVKTQEGHGEFVRRDPFPAVSTALSTWKEVTAPRAVQRSGIPASG
V
10000000000000000000000000000000000000000000000000000000000000000000000000000000000
00000000000100000000000000000000000000000000000000000000000000000000000000000000000
00000000000000000000000000000000000000000000000000000000000000000000000000000000000
00000000000000000000000000000000000000000000000000000000000000000000000000000000000
00000000000000000000000000000000000000000000000000000000000000000000000000000000000
000000000000000000000000000000000000000000000000000000000000000000000
>Q8Z9S7_4fce_0_455
MSNSSMSVVILAAGKGTRMYSDLPKVLHPLAGKPMVQHVIDAAMKLGAQHVHLVYGHG
GELLKKTLADPSLNWVLQAEQLGTGHAMQQAAPHFADDEDILMLYGDVPLISVDTLQRL
LAAKPEGGIGLLTVKLDNPSGYGRIVRENGDVVGIVEHKDASDAQREINEINTGILVANGR
DLKRWLSLLDNNNAQGEFYITDIIALAHADGKKIATVHPTRLSEVEGVNNRLQLSALERVF
QTEQAEKLLLAGVMLLDPSRFDLRGELTHGRDITIDTNVIIEGHVILGDRVRIGTGCVLKNC
VIGDDSEISPYTVLEDARLDANCTVGPFARLRPGAELAEGAHVGNFVEIKKARLGKGSKAG
HLSYLGDAEIGAGVNIGAGTITCNYDGANKFKTIIGDDVFVGSDTQLVAPVTVANGATIGA
GTTVTRDVAENELVISRVKQVHIQGWKRPVKKK
11100000000000000000000000000000000000000000000000000011111000000000000000000000000
00000000000000000000000000000000000000000000000000000000000000000000000000000000000
00000000000000000000000000000000000000000000000000000000000000000000000000000000000
00000000000000000000000000000000000000000000000000000000000000000000000000000000000
00000000000000000000000000000000000000000000000000000000000000000000000000000000000
00000000000000000000000000000000001111111
>Q8P8H1_4dzh_1_448
DPLMTNAPPEPCDLLIEAGYVVPIEPHAVVLEDHAVAVSNGVIVAVLPTADARVRFAPARTV
SRPDAALMPGLVNAHTHNPMTLLRGVADDLPLMVWLQQHIWPVEAAVIGPEFVADGTTL

AIAEMLRGGTTCVNENYFFADVQAAVYKQHGFRALVGAVIIDFPTAWASSDDEYFARAGEL
HDQWRDDPLISTAFAPHAPYTVNDANFERVRMLADQLDMPVHLHTHETAQEVADSVAQ
YGQRPLARLDRLGLVNDRLIAVHMTQLTEAEIHLCAERGVSVVHCPESNLKLASGFCPACA
LQRASVNLAIGTDGCASNNDLDMFSENRTAAILAKAVANDATALDAATTLRAATLGGAR
ALGFGDRIGSIEVGKQADLVCVDLSALETQPLHHVLSQLIYAAGRHQVTDVWIAGKPKLVQ
RELIDMDTAALVANARQWRDRIRTVRA
11111110000000000000000000000000000000000000000000000000000000000000000000000000000
00000000000000000000000000000000000000000000000000000000000000000000000000000000000
00000000000000000000000000000000000000000000000000000000000000000000000000000000000
00000000000000000000000000000000000000000000000000000000000000000000000000000000000
00000000000000000000000000000000000000000000000000000000000000000000000000000000000
000000000000000000000000000000011
>Q2L4H3_2j66_0_427
MKKGGDLRMNLDQAEITALTKRFETPFYLYDGDFIEAHYRQLRSRTNPAIQFYLSLKANNN
IHLAKLFRQWGLGVEVASAGELALARHAGFSAENIIFSGPGKKRSELEIAVQSGIYCIIAESVE
ELFYIEELAEKENKTARVAIRINPDKSFGSTAIKMGGVPRQFGMDESMLDAVMDAVRSLQFT
KFIGIHVYTGTQNLNTDSIIESMKYTVDLGRNIYERYGIVCECINLGGGFGVPYFSHEKALDI
GKITRTVSDYVQEARDTRFPQTTFIIESGRYLLAQAAVYVTEVLYRKASKGEVFVIVDGGMH
HHAASTFRGRSMRSNYPMEYIPVREDSGRRELEKVTIAGPLCTPEDCLGKDVHVPALYPGD
LVCVLNSGAYGLSFSPVHFLGHPTPIEILKRNGSYELIRRKGTADDIVATQLQTE
11100000000000000000000000000000000000000000000000000000000000000000000000000000000
00000000000000000000000000000000000000000000000000000000000011000000000000000000000
00000000000000000000000000000000000000000000000000000000000000000000110000000000000
00000000000000000000000000000000000000000000000000000000000011111111110000001111111
11100000000000000000000000000000000000000000000000000000000000000000000000000000000
0001111111111
>Q9WYC6_2p2w_0_366
MIQKGLEGVKICESSICYLDGINGRLYYRGIPVEELAEKSTFEETAYFLWYGKLPTKSELEEFK
RKMADYRELPAEALGILYHLPKNLHYIDVLKIFLSIHGSMDGNDEDLREKAIRVASVFPTILA
YYYRYSKGKELIRPRKDLSHVENFYYMMFGERNEKIRLLESAFILLMEQDINASTFAALVIAS
TLSDLYSCIVGALGALKGPLHGGASEKVPPMLEEIGSEDRVEEFVQKCLKEKRKIMGFGHRV
YKTYDPRAVFLKRVLQEHFPDSKLFRIASKLEEYIVSNKIKNIYPNVDLYSSVLFEELGFPRNM
FTALFATARVVGWTAHVIEYVSDNKLIRPTSEYVGPMDVEYIPIERRDENG
00000000000000000000000000000000000000000000000000000000000000000000000000000000000
00000000000000000011111110000000000000000000000000000000000000000000000000000000000
00000000000000000000000000000000000000000000000000000000000000000000000000000000000
00000000000000000000000000000000000000000000000000000000000000000000000000000000000
0000000000000000000000000000000011
>G0RZX9_4gni_0_402
MAESASKAAPGERVVIGITFGNSNSSIAHTVDDKAEVIANEDGDRQIPTILSYVDGDEYYGQ
QAKNFLVRNPKNTVAYFRDILGQDFKSVDPTHNHASAHPQEAGDNVVFTIKDKAEEDAEP
STLTVSEIATRYLRRLVGAASEYLGKKVTSAVITIPTNFTEKQKAALIAAAAAADLEVLQLISE
PAAAVLAYDARPEATISDKIIVVADLGGSRSDVTVLASRSGMYTILATVHDYEYHGIALDKV
LIDHFSKEFLKKNPGAKDPRENPRSLAKLRLEAESTKRALSRSTNASFSVESLIDGLDFASTIN
RLRYETIARTVFEGFNRLVESAVKKAGLDPLDVDEVIMSGGTSNTPRIAANFRYIFPESTRILA
PSTDPSALNPSELQARGAALQASLIQE
11111111111000000000000000000000000000000000000000000000000000000000000000000000000
00000000000000000000000000000000000000000000000000000000000000000000000000000000000
00000000000000000000000000000011111100000000000000000000000000000000000000000000000
00000000000000000000000000000000000000000000000000000000000000000000000000000000000
0000000000000000000000000000000000000000000000000000000000000000000000

>Q94FV7_3a5r_0_383
MATEEMKKLATVMAIGTANPPNCYYQADFPDFYFRVTNSDHLINLKQKFKRLCENSRIEKR
YLHVTEEILKENPNIAAYEATSLNVRHKMQVKGVAELGKEAALKAIKEWGQPKSKITHLIV
CCLAGVDMPGADYQLTKLLDLDPSVKRFMFYHLGCYAGGTVLRLAKDIAENNKGARVLI
VCSEMTTTCFRGPSETHLDSMIGQAILGDGAAAVIVGADPDLTVERPIFELVSTAQTIVPESH
GAIEGHLLESGLSFHLYKTVPTLISNNIKTCLSDAFTPLNISDWNSLFWIAHPGGPAILDQVT
AKVGLEKEKLKVTRQVLKDYGNMSSATVFFIMDEMRKKSLENGQATTGEGLEWGVLFGFG
PGITVETVVLRSVPVIS
11111110000000000000000000000000000000000000000000000000000000000000000000000000000
00000000000000000000000000000000000000000000000000000000000000000000000000000000000
00000000000000000000000000000000000000000000000000000000000000000000000000000000000
00000000000000000000000000000000000000000000000000000000000000000000000000000000000
0000000000000000000000000000000000000000000000000001
>A0QV68_3sf6_0_398
MTNSAVAQPRSQRRGADDLIGINAVLSAEEREIRDTVRSVVQRRIKPHIASWYEDGELPARE
LAVELGELGLLGMHLKGYGCAGMSAVAYGLACLELEAGDSGIRSLVSVQGSLAMYAIHAF
GSDEQKDQWLPDMASGHRIGCFGLTEPDHGSDPAGMRTRATRSGDDWILTGTKMWITNG
SVADVAVVWARTDEGIRGFVVPTDTPGFTANTIKSKMSLRASVTSELVLDGVRLPDSARLPG
ATSLGAPLRCLNEARFGIVFGALGAARDCLETALAYACSREQFDRPIGGFQLTQQKLADMT
LEYGKGFLLALHLGRQKDAGELAPEQVSLGKLNNVREAIEIARTARTVLGASGITGEYPVM
RHANNLESVLTYEGTSEMHTLIIGQALTGVGAFR
11111111111100000000000000000000000000000000000000000000000000000000000000000000000
00000000000000000000000000000000000000000000000000000000000000000000000000000000000
00000000000000000000000000000000000000000000000000000000000000000000000000000000000
00000000000000000000000000000000000000000000000000000000000000000000000000000000000
0000000000000000000000000000000000000000000000000000000000000000000
>Q6G2E9_3gir_0_371
MGTSQEFETSSLKTLPLYELHEKAGAKFGAFAGWRMPLTYPLGVLKEHLHTRAHAGLFDIS
HMKLIAVEGPKAVEFLSYALPVDAALLKIGQSRYSYLLNERAGILDDLILTRLAECRFMLVA
NAGNAQADFAELEKRAFGFECQVIALERVLLALQGPQAAAVLADAGLPGNELLFMQGFE
PQQDWFITRSGYTGEDGFEIALPIGCARALAEKLLGDSRVEWVGLAARDSLRLEAGLCLHG
NDITPDTTPIDAALTWAVPKNVREKAQFYGAKAFLESLQKGPSRCRVGLKPQTRQPIRAGA
VLFDNEGNRIGVVTSGGFGPSFDGPVAMGYVPVAWKVEGTEVFTELRGKKIALSVHSLPFV
EQRYFKG
11111111110000000000000000000000000000000000000000000000000000000000000000000000000
00000000000000000000000000000000000000000000000000000000000000000000000000000000000
00000000000000000000000000000000000000000000000000000000000000000000000000000000000
00000000000000000000000000000000000000000000000000000000000000000000000000000000000
0000000000000000000000000000000000000001
>P0A4X2_1xfc_2_385
MTPISQTPGLLAEAMVDLGAIEHNVRVLREHAGHAQLMAVVKADGYGHGATRVAQTAL
GAGAAELGVATVDEALALRADGITAPVLAWLHPPGIDFGPALLADVQVAVSSLRQLDELL
HAVRRTGRTATVTVKVDTGLNRNGVGPAQFPAMLTALRQAMAEDAVRLRGLMSHMVYA
DKPDDSINDVQAQRFTAFLAQAREQGVRFEVAHLSNSSATMARPDLTFDLVRPGIAVYGLS
PVPALGDMGLVPAMTVKCAVALVKSIRAGEGVSYGHTWIAPRDTNLALLPIGYADGVFRSL
GGRLEVLINGRRCPGVGRICMDQFMVDLGPGPLDVAEGDEAILFGPGIRGEPTAQDWADL
VGTIHYEVVTSPRGRITRTYREAENR
11111111111000000000000000000000000000000000000000000000000000000000000000000000000
00000000000000000000000000000000000000000000000000000000000000000000000000000000000
00000000000000000000000000000000000000000000000000000000000000000000000000000000000
00000000000000000000000000000000000000000000000000000000000000000000000000000000000

0000000000000000000000000000000000000000000000000111
>I4IY26_4awe_0_384
KVPKGFVTTEGDHFKLDGKDFYFAGSNAYYFPFNDQPDIEKGMTAARAAGLTVFRTWGFN
DKNRTYIPTGLPQYGNEGAGDPTNTVFQWFEADGTQTIDVSPFDKVVDSATKTGIKLIVAL
TNNWADYGGMDVYTVNLGGKYHDDFYTVPKIKEAFKRYVKAMVTRYRDSEAILAWELA
NEARCGADGTRNLPRSEKGCTTETVTGWIEEMSAYVKSLDGNHLVTWGGEGGFNRGEDE
EDGFYNGADGGDFDRELGLRNVDFGTMHLYPDWWSKSIEWSNQWIHDHAASGRAANK
PVVLEEYGWMTDKGRLDQLGQVKNETRLEVVGGWQKIAIQEKLAGDMYWQFGYGGYSY
GRNHDDSFTIYLEDDEAKELVYKHAKKVQKLN
11110000000000000000000000000000000000000000000000000000000000000000000000000000000
00000000000000000000000000000000000000000000000000000000000000000000000000000000000
00000000000000000000000000000000000000000000000000000000000000000000011000000000000
00000000000000000000000000000000000000000000000000000000000000000000000000000000000
00000000000000000000000000000000000000000000000000000
>Q9A9H3_3dr7_0_370
MSDLPRISVAAPRLDGNERDYVLECMDTTWISSVGRFIVEFEKAFADYCGVKHAIACNNGT
TALHLALVAMGIGPGDEVIVPSLTYIASANSVTYCGATPVLVDNDPRTFNLDAAKLEALITP
RTKAIMPVHLYGQICDMDPILEVARRHNLLVIEDAAEAVGATYRGKKSGSLGDCATFSFFG
NKIITTGEGGMITTNDDDLAAKMRLLRGQGMDPNRRYWFPIVGFNYRMTNIQAAIGLAQL
ERVDEHLAARERVVGWYEQKLARLGNRVTKPHVALTGRHVFWMYTVRLGEGLSTTRDQV
IKDLDALGIESRPVFHPMHIMPPYAHLATDDLKIAEACGVDGLNLPTHAGLTEADIDRVIA
ALDQVLV
11110000000000000000000000000000000000000000000000000000000000000000000000000000000
00000000000000000000000000000000000000000000000000000000000000000000000000000000000
00000000000000000000000000000000000000000000000000000000000000000000000000000000000
00000000000000000000000000000000000000000000000000000000000000000000000000000000000
000000000000000000000000000000000000000
>Q9X273_3amc_0_316
MGVDPFERNKILGRGINIGNALEAPNEGDWGVVIKDEFFDIIKEAGFSHVRIPIRWSTHAYA
FPPYKIMDRFFKRVDEVINGALKRGLAVVINIHHYEELMNDPEEHKERFLALWKQIADRYK
DYPETLFFEILNEPHGNLTPEKWNELLEEALKVIRSIDKKHTIIIGTAEWGGISALEKLSVPKW
EKNSIVTIHYYNPFEFTHQGAEWVEGSEKWLGRKWGSPDDQKHLIEEFNFIEEWSKKNKRP
IYIGEFGAYRKADLESRIKWTSFVVREMEKRRWSWAYWEFCSGFGVYDTLRKTWNKDLLEA
LIGGDSIE
11100000000000000000000000000000000000000000000000000000000000000000000000000000000
00000000000000000000000000000000000000000000000000000000000000000000000000000000000
00000000000000000000000000000000000000000000000000000000000000000000000000000000000
00000000000000000000000000000000000000000000000000000000000000111111
>P24735_2wzz_26_396
GEAPADRLKALVDAAVQPVMKANDIPGLAVAISLKGEPHYFSYGLASKEDGRRVTPETLFEI
GSVSKTFTATLAGYALTQDKMRLDDRASQHWPALQGSRFDGISLLDLATYTAGGLPLQFPD
SVQKDQAQIRDYYRQWQPTYAPGSQRLYSNPSIGLFGYLAARSLGQPFERLMEQQVFPALG
LEQTHLDVPEAALAQYAQGYGKDDRPLRVGPGPLDAEGYGVKTSAADLLRFVDANLHPE
RLDRPWAQALDATHRGYYKVGDMTQGLGWEAYDWPISLKRLQAGNSTPMALQPHRIAR
LPAPQALEGQRLLNKTGSTNGFGAYVAFVPGRDLGLVILANRNYPNAERVKIAYAILSGLEQ
QGKVPLKR
11000000000000000000000000000000000000000000000000000000000000000000000000000000000
00000000000000000000000000000000000000000000000000000000000000000000000000000000000
00000000000000000000000000000000000000000000000000000000000000000000000000000000000
00000000000000000000000000000000000000000000000000000000000000000000000000000000000
000000000000000000000000000000111111111

>P53704_2poc_346_712
MKGPYKHFMQKEIFEQPDSAFNTMRGRIDFENCVVTLGGLKSWLSTIRRCRRIIMIACGTSY
HSCLATRSIFEELTEIPVSVELASDFLDRRSPVFRDDTCVFVSQSGETADSILALQYCLERGALT
VGIVNSVGSSMSRQTHCGVHINAGPEIGVASTKAYTSQYIALVMFALSLSNDSISRKGRHEEII
KGLQKIPEQIKQVLKLENKIKDLCNSSLNDQKSLLLLGRGYQFATALEGALKIKEISYMHSEG
VLAGELKHGILALVDEDLPIIAFATRDSLFPKVMSAIEQVTARDGRPIVICNEGDAIISNDKV
HTTLEVPETVDCLQGLLNVIPLQLISYWLAVNRGIDVDFPRNLAKSVTVE
11100000000000000000000000000000000000000000000000000000000000000000000000000000000
00000000000000000000000000000000000000000000000000000000000000000000000000000000000
00000000000000000000000000000000000000000000000000000000000000000000000000000000000
00000000000000000000000000000000000000000000000000000000000000000000000000000000000
00000000000000000000000111111111111
>O96507_2j1q_0_356
MASAEVVSKLEAAFAKLQNASDCHSLLKKYLTKEVFDQLKGKQTKMGATLMDVIQSGVE
NLDSGIGVYAPDAESYTLFAALFDPIIEDYHKGFKPSDKQPPKDFGDLNTFIDVDPDKKYVIS
TRVRCGRSLEGYPFNPCLKKQQYEEMESRVKGQLESMSGELRGKYYPLTGMTKETQKQLID
DHFLFKEGDRFLQAAHACKFWPTGRGIYHNDAKTFLVWVNEEDHLRIISMQKGGNLKEV
FGRLVTAVGVIEEKVKFSRDDRLGFLTFCPTNLGTTIRASVHIKLPKLGADRKKLEEVAAKY
NLQVRGTAGEHSDSPDGVYDISNKRRLGLSEYEAVKEMQDGILELIKAEESAR
00000000000000000000000000000000000000000000000000000000000000000000000000000000000
00000000000000000000000000000000000000000000000000000000000000000000000000000000000
00000000000000000000000000000000000000000000000000000000000000000000000000000000000
00000000000000000000000000000000000000111110000000000000000011111111111000000000000
0000000000000000000000011
>O59196_1y0r_0_352
MEVRNMVDYELLKKVVEAPGVSGYEFLGIRDVVIEEIKDYVDEVKVDKLGNVIAHKKGEG
PKVMIAAHMDQIGLMVTHIEKNGFLRVAPIGGVDPKTLIAQRFKVWIDKGKFIYGVGASVP
PHIQKPEDRKKAPDWDQIFIDIGAESKEEAEDMGVKIGTVITWDGRLERLGKHRFVSIAFDD
RIAVYTILEVAKQLKDAKADVYFVATVQEEVGLRGARTSAFGIEPDYGFAIDVTIAADIPGTP
EHKQVTHLGKGTAIKIMDRSVICHPTIVRWLEELAKKHEIPYQLEILLGGGTDAGAIHLTKA
GVPTGALSVPARYIHSNTEVVDERDVDATVELMTKALENIHELKI
11111000000000000000000000000000000000000000000000000000000000000000000000000000000
00000000000000000000000000000000000011111111111100000000000000000000000000000000000
00000000000000000000000000000000000000000000000000000000000000000000000000000000000
00000000000000000000000000000000000000000000000000000000000000000000000000000000000
000000000000000000011
>Q0PAS0_3qm3_0_353
MGVLDIVKAGVISGDELNKIYDYAKAEGFAIPAVNVVGTDSINAVLEAAKKVNSPVIIQFSN
GGAKFYAGKNCPNGEVLGAISGAKHVHLLAKAYGVPVILHTDHAARKLLPWIDGLIEAN
AQYKKTHGQALFSSHMLDLSEESLEENLSTCEVYLQKLDALGVALEIELGCTGGEEDGVDN
TGIDNSKLYTQPEDVALAYERLGKISDKFSIAASFGNVHGVYKPGNVSLQPEILKNSQKFVK
DKFALNSDKPINFVFHGGSGSELKDIKNAVSYGVIKMNIDTDTQWAFWDGVREYELKNRA
YLQGQIGNPEGDDKPNKKYYDPRVWLRSGEESMIKRLEIAFEDLNCINKN
00000000000000000000000000000000000000000000000000000000000000000000000000000000000
00000000000000000000000000000000000000000000000000000000000000000000000000000000000
00000000011111000000000000000000000000000000000000000000001111100000000000000000000
00000000000000000000000000000000000000000000000000000000000000000000000000000000000
0000000000000000000000
>P07355_1w7b_0_338
MSTVHEILCKLSLEGDHSTPPSAYGSVKAYTNFDAERDALNIETAIKTKGVDEVTIVNILTNR
SNAQRQDIAFAYQRRTKKELASALKSALSGHLETVILGLLKTPAQYDASELKASMKGLGTD

EDSLIEIICSRTNQELQEINRVYKEMYKTDLEKDIISDTSGDFRKLMVALAKGRRAEDGSVID
YELIDQDARDLYDAGVKRKGTDVPKWISIMTERSVPHLQKVFDRYKSYSPYDMLESIRKEVK
GDLENAFLNLVQCIQNKPLYFADRLYDSMKGKGTRDKVLIRIMVSRSEVDMLKIRSEFKRKY
GKSLYYYIQQDTKGDYQKALLYLCGGDD
11111111111111111111000000000000000000000000000000000000000000000000000000000000
00000000000000000000000000000000000000000000000000000000000000000000000000000000
00000000000000000000000000000000000000000000000000000000000000000000000000000000
00000000000000000000000000000000000000000000000000000000000000000000000000000000
0000000
>Q91H09_3pvd_221_536
SKTKAFTIPVLKISEMTNSRFPVPVDQMYTSRSEGIVVQPQNGRATIDGELLGTTLVSPVSVC
NFKGNLQAEVPGQHQLYQLQLTNLDGSPIDPTDDTPGPLGCPDFTGLLYGVASQRGPGDA
TRAHEARIDTGSDTFAPKIGQVRFYSTSSDFETNQPTHFTPIGIYIEGNSSDFNQWQLPRYGG
HLANNNHLAPAVSPLFPGEQILFFRSFIPGASGHTNGEMDCLLPQEFVQHFYQEAATARSE
VALLRFVNPDTGRALFESKLHKQGFMTIASSGDHPIIMPTNGYFRFEAWVNQFYSLAPVGT
GSGRRRIQ
11110000000000000000000000000000000000000000000000000000000000000000000000000000
00000000000000000000000000000000000000000000000000000000000000000000000000000000
00000000000000000000000000000000000000000000000000000000000000000000000000000000
0000000000000000000000000000000000000000000000000000001111111111
>Q4QEW7_4ef8_0_319
MSLQVNLLNNTFANPFMNAAGVMCTTTEELVAMTESASGSLVSKSCTPALREGNPTPRYQ
ALPLGSINSMGLPNNGFDFYLAYAAEQHDYGKKPLFLSMSGLSMRENVEMCKRLAAVATE
KGVILELNLSCPNVPGKPQVAYDFDAMRQCLTAVSEVYPHSFGVKMPPYFDFAHFDAAAEI
LNEFPKVQFITCINSIGNGLVIDAETESVVIKPKQGFGGLGGRYVLPTALANINAFYRRCPGK
LIFGCGGVYTGEDAFLHVLAGASMVQVGTALQEEGPSIFERLTSELLGVMAKKRYQTLDEF
RGKVRTLDGTAESTR
00000000000000000000000000000000000000000000000000000000000000000000000000000000
00000000000000000000000000000000000000000000000000000000000000000000000000000000
00000000000000000000000000000000000000000000000000000000000000000000000000000000
00000000000000000000000000000000000000000000000000000000000011111111
>P08176_1xkg_18_319
RPSSIKTFEEYKKAFNKSYATFEDEEAARKNFLESVKYVQSNGGAINHLSDLSLDEFKNRFL
MSAEAFEHLKTQFDLNAETNACSINGNAPAEIDLRQMRTVTPIRMQGGCGSCWAFSGVAA
TESAYLAYRNQSLDLAEQELVDCASQHGCHGDTIPRGIEYIQHNGVVQESYYRYVAREQSC
RRPNAQRFGISNYCQIYPPNVNKIREALAQTHSAIAVIIGIKDLDAFRHYDGRTIIQRDNGYQ
PNYHAVNIVGYSNAQGVDYWIVRNSWDTNWGDNGYGYFAANIDLMMIEEYPYVVIL
11100000000000000000000000000000000000000000000000000000000000000000000000111110
00000000000000000000000000000000000000000000000000000000000000000000000000000000
00000000000000000000000000000000000000000000000000000000000000000000000000000000
000000000000000000000000000000000000000000000000000
>Q9NQU5_2c30_382_680
GVVTHEQFKAALRMVVDQGDPRLLLDSYVKIGEGSTGIVCLAREKHSGRQVAVKMMDLR
KQQRRELLFNEVVIMRDYQHFNVVEMYKSYLVGEELWVLMEFLQGGALTDIVSQVRLNEE
QIATVCEAVLQALAYLHAQGVIHRDIKSDSILLTLDGRVKLSDFGFCAQISKDVPKRKSLVGT
PYWMAPEVISRSLYATEVDIWSLGIMVIEMVDGEPPYFSDSPVQAMKRLRDSPPPKLKNSHK
VSPVLRDFLERMLVRDPQERATAQELLDHPFLLQTGLPECLVPLIQLYRKQTSTC
11000000000000000000000000000000000000000000000000000000000000000000000000000000
00000000000000000000000000000000000000000000000000000000000000000000000000000000
00000000000000000000000000000000000000000000000000000000000000000000000000000000
000000000000000000000000000000000000000001111111

>Q8ZMG4_3g0o_2_293
TGTDFHVGIVGLGSMGMGAARSCLRAGLSTWGADLNPQACANLLAEGACGAAASAREF
AGVVDALVILVVNAAQVRQVLFGEDGVAHLMKPGSAVMVSSTISSADAQEIAAALTALNL
NMLDAPVSGGAVKAAQGEMTVMASGSEAAFTRLKPVLDAVASNVYRISDTPGAGSTVKII
HQLLAGVHIAAAAEAMALAARAGIPLDVMYDVVTHAAGNSWMFENRMQHVVDGDYT
PRSAVDIFVKDLGLVADTAKALRFPLPLASTALNMFTSASNAGYGKEDDSAVIKIFSG
11100000000000000000000000000000000000000000000000000000000000000000000000000000110
00000000000000000000000000000000000000000000000000000000000000000000000000000000000
00000000000000000000000000000000000000000000000000000000000000000000000000000000000
00000000000000000000000000000000000000000011
>Q66D48_3odg_0_286
MTTVNSNINVDADFNELPFQAVKYIQKIKPGFKPQIAFILGSGLGDLVDQITNDTTISYADIP
GFPVSSVHGHAGELVLGDLCGVPVMCMKGRGHFYEGKGMSIMTNPVRTFKLMGCEFLFC
TNAAGSLRPEVLPGSVVMLKDHINTMPGTPLVGPNDDRFGPRFFSLANAYDKDLRADMA
KIAQQLDIPLTEGVFVSYPGPCFETPAEIRMMQIIGGDVVGMSVVPEVLSAAHCGLKVIALT
AITNLAEGLSDVVLSHEQTLKFAKVASVNFTKLIEAFLKSKALR
11111111111100000000000000000000000000000000000000000000000000000000000000000000000
00000000000000000000000000000000000000000000000000000000000000000000000000000000000
00000000000000000000000000000000000000000000000000000000000000000000000000000000000
00000000000000000000000000000000000011
>O33253_1i9g_0_279
MSATGPFSIGERVQLTDAKGRRYTMSLTPGAEFHTHRGSIAHDAVIGLEQGSVVKSSNGALF
LVLRPLLVDYVMSMPRGPQVIYPKDAAQIVHEGDIFPGARVLEAGAGSGALTLSLLRAVGP
AGQVISYEQRADHAEHARRNVSGCYGQPPDNWRLVVSDLADSELPDGSVDRAVLDMLAP
WEVLDAVSRLLVAGGVLMVYVATVTQLSRIVEALRAKQCWTEPRAWETLQRGWNVVGLA
VRPQHSMRGHTAFLVATRRLAPGAVAPAPLGRKREGRDG
11100000000000000000000000000000000000000000000000000000000000000000000000000000000
00000000000000000000000000000000000000000000000000000000000000000000000000000000000
00000000000000000000000000000000000000000000000000000000000000000000000000000000000
0000000000000000001111111111111
>Q63X76_3uzu_0_274
MSNSRQHQGHFARKRFGQNFLVDHGVIDAIVAAIRPERGERMVEIGPGLGALTGPVIARLA
TPGSPLHAVELDRDLIGRLEQRFGELLELHAGDALTFDFGSIARPGDEPSLRIIGNLPYNISSP
LLFHLMSFAPVVIDQHFMLQNEVVERMVAEPGTKAFSRLSVMLQYRYVMDKLIDVPPESFQ
PPPKVDSAIVRMIPHAPHELPAVDPAVLGEVVTAAFSQRRKMLRNTLGGYRDLVDFDALGF
DLARRAEDIGVDEYVRVAQAVASARASG
11111111111110000000000000000000000000000000000000000000000000000000000000000000000
00000000000000000000000000000000000000000000000000000000000000000000000000000000000
00000000000000000000000000000000000000000000000000000000000000000000000000000000000
00000000000000000000000000
>Q63226_2v3u_439_812
GVVLRVVTVLEEPFVMVSENVLGKPKKYQGFSIDVLDALSNYLGFNYEIYVAPDHKYGSPQ
EDGTWNGLVGELVFKRADIGISALTITPDRENVVDFTTRYMDYSVGVLLRRESSIQSLQDLSK
QTDIPYGTVLDSAVYQHVRMKGLNPFERDSMYSQMWRMINRSNGSENNVLESQAGIQKV
KYGNYAFVWDAAVLEYVAINDPDCSFYTVGNTVADRGYGIALQHGSPYRDVFSQRILELQ
QSGDMDILKHKWWPKNGQCDL
00000000000000000000000000000000000000000000000000000000000000000000000000000000000
00000000000000000000000000000000000000000000000000000000000000000000000000000000001
11110000000000000000000000000000000000000000011100000000000000000000000000000000000
000000000001111
>Q6XEC0_3hbr_0_264

MRVLALSAVFLVASIIGMPAVAKEWQENKSWNAHFTEHKSQGVVVLWNENKQQGFTNNL
KRANQAFLPASTFKIPNSLIALDLGVVKDEHQVFKWDGQTRDIATWNRDHNLITAMKYSV
VPVYQEFARQIGEARMSKMLHAFDYGNEDISGNVDSFWLDGGIRISATEQISFLRKLYHNKL
HVSERSQRIVKQAMLTEANGDYIIRAKTGYSTRIEPKIGWWVGWVELDDNVWFFAMNMD
MPTSDGLGLRQAITKEVLKQEKIIP
11111111111111111111111100000000000000000000000000000000000000000000000000000000000
00000000000000000000000000000000000000000000000000000000000000000000000000000000000
00000000000000000000000000000000000000000000000000000000000000000000000000000000000
0000000000000000
>P0A078_1qxz_0_251
MIVKTEEELQALKEIGYICAKVRNTMQAATKPGITTKELDNIAKELFEEYGAISAPIHDENFP
GQTCISVNEEVAHGIPSKRVIREGDLVNIDVSALKNGYYADTGISFVVGESDDPMKQKVCD
VATMAFENAIAKVKPGTKLSNIGKAVHNTARQNDLKVIKNLTGHGVGLSLHEAPAHVLN
YFDPKDKTLLTEGMVLAIEPFISSNASFVTEGKNEWAFETSDKSFVAQIEHTVIVTKDGPILTT
KIEEE
00000000000000000000000000000000000000000000000000000000000000000000000000000000000
00000000000000000000000000000000000000000000000000000000000000000000000000000000000
00000000000000000000000000000000000000000000000000000000000000000000000000000000000
111
>Q99WD9_1p99_18_279
GSNNDKKVTIGVASNDTKAWEKVKELAKKDDIDVEIKHFSDYNLPNKALNDGDIDMNAF
QHFAFLDQYKKAHKGTKISALSTTVLAPLGIYSDKIKDVKKVKDGAKVVIPNDVSNQARAL
KLLEAAGLIKLKKDFGLAGTVKDITSNPKHLKITAVDAQQTARALSDVDIAVINNGVATKA
GKDPKNDPIFLEKSNSDAVKPYINIVAVNDKDLDNKTYAKIVELYHSKEAQKALQEDVKDG
EKPVNLSKDEIKAIETSLAK
11111100000000000000000000000000000000000000000000000000000000000000000000000000000
00000000000000000000000000000000000000000000000000000000000000000000000000000000000
00000000000000000000000000000000000000000000000000000000000000000000000000000000000
0000000000001
>Q83E52_3tqd_0_248
MEFRVIIPARFDSTRLPGKALVDIAGKPMIQHVYESAIKSGAEEVVIATDDKRIRQVAEDFGA
VVCMTSSDHQSGTERIAEAAVALGFEDDEIIVCLQGDEPLIPPDAIRKLAEDLDEHDNVKVA
SLCTPITEVDELFNPHSTKVVLNRRNYALYFSHAPIPWGRDTFSDKENLQLNGSHYRHVGIY
AYRVGFLEEYLSWDACPAEKMEALEQLRILWHGGRIHMVVAKSKCPPGVDTEEDLERVRA
YF
10000000000011100000000000000000000000000000000000000000000000000000000000000000000
00000000000000000000000000000000000001100000000000000000000000000000000000000000000
00000000000000000000000000000000000000000000000000000000000000000000000000000000000
>D0VWV7_2b94_0_244
MEEEMQRHIKLTPSQTTPVVLVVGDPGRVDKVKMLCDSYVDLAYNREYKSVECTYKGQKF
LCVSHGVGSAGCAICFEELMNNGAKVIIRAGSCGSLQPTQMKRGDICICNAAVREDRVSHL
MIYSDFPAVADFEVYDTLNKVAQELEVPVFNGISLSSDLYYPHKIIPTRLEDYSKANVAVVEM
EVATLMVMGTLRKVKTGGIFIVDGCPLKWDEGDFDNNLVPEKLENMIKISLETCARLAKKY
00000000000000000000000000000000000000000001110000000000000000000000000000000000000
00000000000000000000000000000000000000000000000000000000000000000000000000000000000
0000000000000000000000000000000000000000000000001111110000000000000000000000000
>P04190_2uyx_29_256
ASQKVEKTVIKNETGTISISQLNKNVWVHTELGSFNGEAVPSNGLVLNTSKGLVLVDSSWD
DKLTKELIEMVEKKFQKRVTDVIITHAHADRIGGIKTLKERGIKAHSTALTAELAKKNGYEE
PLGDLQTVTNLKFGNMKVETFYPGKGHTEDNIVVWLPQYNILVGGCLVKSTSAKDLGNVA
DAYVNEWSTSIENVLKRYRNINAVVPGHGEVGDKGLLLHTLDLLK

11111100000000000000000000000000001111100000000000000000000000000000000000000000000
00000000000000000000000000000000000000000000000000000000000000000000000000000000000
0000000000000000000000000000000000000000000000000000000000000
>O57978_3u55_0_237
MVKLMEVYEGKAKKMIPIDDDKLIMEFKDDATAFDGTKKARFKGKGWLNAQLSVIFFKLL
EEHGIKTHFIGVAGGNRLIVEKLDMYPLEVVVRNVVAGSLKKRLPLPEGYELPEPIVELYYK
NDELHDPMINYYHAKVLGISLDEIKKIEEIALKVNEILKDYLAKKGIILVDFKLEFGKDKNG
DIVLADEISPDTCRFWDAKTKRSLDKDVFRFDKGDLIEAYKEIYERITGEKPEF
11111111111000000000000000000000000000000000000000000000000000000000000000000000000
00000000000000000000000000000000000000000000000000000000000000000000000000000000000
00000000000000000000000000000000000000000000000000000000000000000000000
>P65522_1yxy_0_233
MPDKPTKEKLMEQLKGGIIVSCQALPGEPLYSETGGIMPLMAKAAQEAGAVGIRANSVRDI
KEIQAITDLPIIGIIKKDYPPQEPFITATMTEVDQLAALNIAVIAMDCTKRDRHDGLDIASFIR
QVKEKYPNQLLMADISTFDEGLVAHQAGIDFVGTTLSGYTPYSRQEAGPDVALIEALCKAGI
AVIAEGKIHSPEEAKKINDLGVAGIVVGGAITRPKEIAERFIEALKS
11100000000000000000000000000000000000000000000000000000000000000000000000000000000
00000000000000000000000000000000000000000000000000000000000000000000000000000000000
0000000000000000000000000000000000000000000000000000000000000000001
>Q97R43_3mms_0_229
MKIGIIAAMPEELAYLVQHLDNAQEQVVFGNTYHTGTIASHEVVLVESGIGKVMSAMSVAI
LADHFQVDALINTGSAGAVAEGIAVGDVVIADKLAYHDVDVTAFGYAYGQMAQQPLYFES
DKTFVAQIQKSLSQLDQNWHLGLIATGDSFVAGNDKIEAIKSHFPEVLAVEMEGAAIAQAA
HALNLPVLVIRAMSDNANHEANIFFDEFIIEAGRRSAQVLLTFLKALD
00000000000000000000000000000000000000000000000000000000000000000000000000000000000
00000000000000000000000000000000000000000000000000111100000000000000000000000000000
000000000000000000000000000000000000000000000000000000000000001
>P12295_2eug_0_228
MANELTWHDVLAEEKQQPYFLNTLQTVASERQSGVTIYPPQKDVFNAFRFTELGDVKVVIL
GQDPYHGPGQAHGLAFSVRPGIAIPPSLLNMYKELENTIPGFTRPNHGYLESWARQGVLLL
NTVLTVRAGQAHSHASLGWETFTDKVISLINQHREGVVFLLWGSHAQKKGAIIDKQRHHV
LKAPHPSPLSAHRGFFGCNHFVLANQWLEQRGETPIDWMPVLPAESE
11110000000000000000000000000000000000000000000000000000000000000000000000000000000
00000000000000000000000000000000000000000000000000000000000000000000000000000000000
00000000000000000000000000000000000000000000000000000000000000
>Q8E0S6_4g1h_18_232
ASHQNINQFKREVAKIDTNTVERRIALANAYNETLSRNPLLIDPFTSKQKEGLREYARMLEV
HEQIGHVAIPSIGVDIPIYAGTSETVLQKGSGHLEGTSLPVGGLSTHSVLTAHRGLPTARLFTD
LNKVKKGQIFYVTNIKETLAYKVVSIKVVDPTALSEVKIVNGKDYITLLTCTPYMINSHRLLV
KGERIPYDSTEAEKHKEQTVQDYRLS
11111111111111100000000000000000000000000000000011110000000000000000000000000000000
00000000000000000000000000000000000000000000000000000000000000000000000000000000000
000000000000000000000000000001111111111111111111
>Q5L3C9_3av3_0_209
MKRLAVFASGSGTNFQAIVDAAKRGDLPARVALLVCDRPGAKVIERAARENVPAFVFSPKD
YPSKAAFESEILRELKGRQIDWIALAGYMRLIGPTLLSAYEGKIVNIHPSLLPAFPGKDAIGQA
YRAGVSETGVTVHYVDEGMDTGPVIAQRVVPIVPGEPIEALEERIHQVEHELYPTVLRMLLG
EKEQQEERIENDGSETSIDQRVQ
00000000000000000000000000000000000000000000000000000000000000000000000000000000000
00000000000000000000000000000000000000000000000000000000000000000000000000000000000
0000000000000000000001111111111111111111111

>Q9KPQ8_3ot1_0_204
MEQGMSKRILVPVAHGSEEMETVIIVDTLVRAGFQVTMAAVGDKLQVQGSRGVWLTAEQT
LEACSAEAFDALALPGGVGGAQAFADSTALLALIDAFSQQGKLVAAICATPALVFAKQQKF
VGARMTCHPNFFDHIPSERLSRQRVCYYATQHLLTSQGPGTALEFALAMIALLAGVELAQH
VAAPMVLHPQQLTELSGFIDAQS
11110000000000000000000000000000000000000000000000000000000000000000000000000000
00000000000000000000000000000000000000000000000000000000000000000000000000000000
0000000000000000000000000000000000000001
>Q8IEL9_2plw_69_269
NYRSRAAYKLIELDNKYLFLKKNKIILDIGCYPGSWCQVILERTKNYKNKIIGIDKKIMDPIP
NVYFIQGEIGKDNMNNIKNINYIDNMNNNSVDYKLKEILQDKKIDIILSDAAVPCIGNKIDD
HLNSCELTLSITHFMEQYINIGGTYIVKMYLGSQTNNLKTYLKGMFQLVHTTKPKASRNESR
EIYLVCKNFLGRKK
10000000000000000000000000000000000000000000000000000000000000000000000000000111
11111110000000000000000000000000000000000000000000000000000000000000000000000000
0000000000000011111000000000000011
>Q8CTG7_3bwv_0_178
MTRQRIAIDMDEVLADTLGAVVKAVNERADLNIKMESLNGKKLKHMIPEHEGLVMDILKE
PGFFRNLDVMPHAQEVVKQLNEHYDIYIATAAMDVPTSFHDKYEWLLEYFPFLDPQHFVF
CGRKNIILADYLIDDNPKQLEIFEGKSIMFTASHNVYEHRFERVSGWRDVKNYFNSIEK
10000000000000000000000000000000000000000111111110000000000000000000000000000000
00000000000000000000000000000000000000000000000000000000000000000000000000000000
0000000000001
>P76268_1ysp_87_262
DLIRSADIQMRELSRLTKETIHLGALDEDSIVYIHKIDSMYNLRMYSRIGRRNPLYSTAIGKVL
LAWRDRDEVKQILEGVEYKRSTERTITSTEALLPVLDQVREQGYGEDNEEQEEGLRCIAVPV
FDRFGVVIAGLSISFPTLRFSEERLQEYVAMLHTAARKISAQMGYHDYPF
00000000000000000000000000000000000000111111110000000000000000000000000000000000
00000000000000000000000000000000000000000000000000000000000000000000000000000000
0000011111
>Q9KP52_3n07_1_184
SSTVSTLYGEVEPSLLEIAKQIKLLICDVDGVFSDGLIYMGNQGEELKTFHTRDGYGVKALM
NAGIEIAIITGRRSQIVENRMKALGISLIYQGQDDKVQAYYDICQKLAIAPEQTGYIGDDLID
WPVMEKVALRVCVADGHPLLAQRANYVTHIKGGHGAVREVCDLILQARNELDVHKGLSL
00000000000000000000000000000000000000000000000000000000000000000000000000000000
00000000000000000000000000000000000000000000000000000000000000000000000000000000
000000000000111111
>Q2GIS1_3ixc_0_169
MREVLVPYAGVSPSVDSTAFIAGNARIIGDVCIGKNASIWYGTVLRGDVDKIEVGEGTNIQD
NTVVHTDSMHGDTVIGKFVTIGHSCILHACTLGNNAFVGMGSIVMDRAVMEEGSMLAAG
SLLTRGKIVKSGELWAGRPAKFLRMMTEEEILYLQKSAENYIALSRGYL
00000000000000000000000000000000000000000000000000000000000000000000111110000000
00000000000000000000000000000000000000000000000000000000000000000000000000000000
0000
>Q81KC7_3o7m_0_182
MNIEIKDTLISEEQLQEKVKELALQIERDFEGEEIVVIAVLKGSFVFAADLIRHIKNDVTIDFIS
ASSYGNQTETTGKVKLLKDIDVNITGKNVIVVEDIIDSGLTLHFLKDHFFMHKPKALKFCTL
LDKPERRKVDLTAEYVGFQIPDEFIVGYGIDCAEKYRNLPFIASVVTEESYNLKSR
10000000000000000000000000000000000000000000000000000000000000000001111111000000
00000000000000000000000000000000000000000000000000000000000000000000000000000000
00000001111111111

>Q1AN23_2wfc_27_185
MPIKEGDKLPAVTVFGATPNDKVNMAELFAGKKGVLFAVPGAFTPGCSKTHLPGYVEQAA
AIHGKGVDIIACMAVNDSFVMDAWGKAHGADDKVQMLADPGGAFTKAVDMELDLSAV
LGNVRSKRYSLVIEDGVVTKVNVEPDGKGLTCSLAPNILSQL
10000000000000000000000000000000000000000000000000000000000000000000000000000000000
0000000000000000000000000000000000000000000000000000000000000000000000000000
>P66034_2c94_0_159
MKGGAGVPDLPSLDASGVRLAIVASSWHGKICDALLDGARKVAAGCGLDDPTVVRVLGAI
EIPVVAQELARNHDAVVALGVVIRGQTPHFDYVCDAVTQGLTRVSLDSSTPIANGVLTTNT
EEQALDRAGLPTSAEDKGAQATVAALATALTLRELRAHS
11111111111111000000000000000000000000000000000000000000000000000000000000000000000
00000000000000000000000000000000000000000000000000000000000000000000000000000
>P12473_1kqr_61_223
EPVLDGPYQPTSFNPPVDYWMLLAPTAAGVVVEGTNNTDRWLATILVEPNVTSETRSYTLF
GTQEQITIANASQTQWKFIDVVKTTQNGSYSQYGPLQSTPKLYAVMKHNGKIYTYNGETPN
VTTKYYSTTNYDSVNMTAFCDFYIIPREEESTCTEYINNGL
11100000000000000000000000000000000000000000000000000000000000000000000000000000000
00000000000000000000000000000000000000000000000000000000000000000000000000000000
>Q6NJA6_2g2c_0_163
MHIKSAIIVVSDRISTGTRENKALPLLQRLMSDELQDYSYELISEVVVPEGYDTVVEAIATALK
QGARFIITAGGTGIRAKNQTPEATASFIHTRCEGLEQQILIHGSTHTHLAGLSRGIVGVTGRD
DHAALIVNAPSSSGGITDTWAVISPVIPNIFEGLDAS
00000000000000000000000000000000011110000000000000000000000000000000000000000000000
000000000000000000000001111111100000000000000000000000000000000000000000000000001
>Q9HII6_1rlh_0_152
MVIPAEANIIVGYSHFIKTVEDLNEIIRTHVPGSKYGIGFSEASGDRLIRYDGNDDDLVKACIE
NIRRISAGHTFVILIRNAYPINILNAVKMCQEVGSIFAATANPLQIIVYKGERGNGVLGVIDGY
SPVGVESDADIEKRRQFLRRIGYKE
00000000000000000000000000000000000000000000000000000000000000000000000000000000000
0000000000000000000000000000000000000000000000000000111111111111111111
>Q9I2W7_3c8o_0_161
MHYVTPDLCDAYPELVQVVEPMFSNFGGRDSFGGEIVTIKCFEDNSLVKEQVDKDGKGKVL
VVDGGGSLRRALLGDMLAEKAAKNGWEGIVVYGCIRDVDVIAQTDLGVQALASHPLKTD
KRGIGDLNVAVTFGGVTFRPGEFVYADNNGIIVSPQALKMPE
00000000000000000000000000000000000000000000000000000000000000000000000000000000000
0000000000000000000000000000000000000000000000000000000000000000000000000000001
>O51614_3hzh_1_160
RIDYIEPFLDAASSVLRDMLLVENIEMGKPGLKSINQKIKGVSVIVGLAGSVEGSIIIDMDIET
ALFVASKLNFEEYDDFDDEETKEMVAATLTEVGNIIAGNFVTTLHAKGFVFDITPPAFIYGE
NMKISNKGSEALIVPFSLPDGKIIEVNIAIRERV
00000000000000000000000000000000001111000000000000000000000000000000000000000000000
00000000000000000000000000000000000000000000000000000000000000000000000000011
>Q5SJS9_1od6_0_159
MHVVYPGSFDPLTNGHLDVIQRASRLFEKVTVAVLENPSKRGQYLFSAEERLAIIREATAHL
ANVEAATFSGLLVDFVRRVGAQAIVKGLRAVSDYEYELQMAHLNRQLYPGLETLFILAATR
YSFVSSTMVKEIARYGGDVSKLVPPATLRALKAKLGQ
00000000000000000000000000000000000111110000000000000000000000000000000000000000000
00000000000000000000000000000000000000000000000000000000000000000000000000000
>B7GNK7_3mvk_0_147
MLKGIPKIIPPELLKVLCEMGHGDQLVIADGNFPAESIGKNAIVVRMDGHGGGEILKAILTV
FPLDTYVDKPATLMEKVPGDTVATPIWDVYAGLIKEHDERGADAIGSLERFAFYEQAKNAY

CVIASGESAQYANLILQKGVVFNAE
11110000000000000000000000000000000000000000000000000000000000000000000000000000000
00000000000000000000000000000000000000000000000000000000000000111
>A6L2G9_4grf_251_391
ATGSVAPAITGIDLKGNSVSLNDFKGKYVLVDFWFAGCSWCRKETPYLLKTYNAFKDKGFT
IYGVSTDRREEDWKKAIEEDKSYWNQVLLQKDDVKDVLESYCIVGFPHIILVDPEGKIVAKE
LRGDDLYNTVEKFVNGAK
00000000000000000000000000000000000000000000000000000000000000000000000000000000000
0000000000000000000000000000000000000000000000000000000111
>P02746_2wnv_117_252
ATQKIAFSATRTINVPLRRDQTIRFDHVITNMNNNYEPRSGKFTCKVPGLYYFTYHASSRGN
LCVNLMRGRERAQKVVTFCDYAYNTFQVTTGGMVLKLEQGENVFLQATDKNSLLGMEGA
NSIFSGFLLFPDMEA
10000000000001000000000000000000000000000000000000000000000000000000000000000000000
00000000000000000000000000000000000000000000000000011
>Q5M567_3peb_0_133
MSKLDRIRHFLNENKSGLAIVSDPVTVNYLTGFDCDPHERQMFLFVYENREPALFVPALEV
ARASSVLDFPVFGYVDSENPWQKIKAGLASTDIPIIYAEFDNLNVTKFQGLQTVFEGRFENL
TPFIHKMRVIK
00000000000000000000000000000000000000000000000000000000000000000000000000000000000
000000000000000000000000000000000000000000000000111
>B2HDU6_4f3w_0_130
MPDIDWKQLRDKATQVAAGAYAPYSRFPVGAAALVDDGRVVTGCNVENVSYGLALCAEC
GVVCALHATGGGRLVALACVDGRGAPLMPCGRCRQLLFEHGGPELLVDHLAGPRRLGDL
LPEPFHADLTGEP
11000000000000000000000000000000000000000000000000000000000000000000000000000000000
000000000000000000000000000000000000000111111111
>A7YYL1_2uz2_18_147
TEAQKCNLQGQWRNKLGSNLIIESVSQNGEFTGTYFTSVSLTNSTIRISPLTGYQKLTEKPTF
GFTVHWAFSDSITVWTGQCFLNEKGEEILHTMWLLRSSQEKEQDNWTGTRVGGNTFTRLS
KKKIRKE
11100000000000000000000000000000000000000000000000000000000000000000000000000000000
00000000000000000000000000000000000000001111111
>A7M5Y8_4dsd_20_147
KDVITKDMNQLPLPARNFINSNFTKPQVAHIKIDKDMMESTKYEVVLMDGTEIDFDSKGN
WEEVSAKKGQTVPVSIVPGFAVNYLKAHNFVNEGVTKVERDRKGYEIELSTGLSFKFDKKG
KFIKTDD
00000000000000000000000000000000000000000000000000000000000000000000000000000000000
000000000000000000000000000000000000000000011
>B2C3H7_3suj_19_144
AVKLSYDEAYDNPSSSLLSVTCSDGENGLYPKYRTFGDLPGFPCIGGSSDIAGYNSPNCGSCY
QLTYSSAHTTPKSIYMVAIDRSAEGFTASKQAMDDLTNKRAEELGTVNVDVRKVDFSRCER
KS
00000000000000000000000000000000000000000000000000000000000000000000000000000000000
0000000000000000000000000000000000000000111
>O74017_2d3k_0_120
MIKMFKYKQVIVARADLKLSKGKLAAQVAHGAVTAAFEAYKKKREWFEAWFREGQKKVV
VKVESEEELFKLKAEAEKLGLPNALIRDAGLTEIPPGTVTVLAVGPAPEEIVDKVTGNLKLL
11100000000000000000000000000000000000000000000000000000000000000000000000000000000
00000000000000000000000000000000000000
>Q9R638_1gyo_0_108

LDVPCKVVITAPEGEDPHPRFGKVEMSHAKHRNVSCVSCHHMFDGCGDFQKCADCHIDR
DDRSYERGFYKAWHSESEISCRGCHKAMKAKNEQTGPIGCLQGCHEAAQK
00000000000000000000000000000000000000000000000000000000000000000000000000000000000
00000000000000000000000111
>P07515_1ptf_0_87
MEKKEFHIVAETGIHARPATLLVQTASKFNSDINLEYKGKSVNLKSIMGVMSLGVGQGSDV
TITVDGADEAEGMAAIVETLQKEGLAE
00000000000000000000000000000000000000000000000000000000000000000000000000000000000
00001
>Q805F6_3c05_418_481
DAPANPCCDAATCKLTTGSQCADGLCCDQCKFMKEGTVCRRARGDDLDDYCNGISAGCP
RNPFH
1110000000000000000000000000000000000000000000000000000000000011
>P12111_1knt_3107_3164
ETDICKLPKDEGTCRDFILKWYYDPNTKSCARFWYGGCGGNENKFGSQKECEKVCAPV
1000000000000000000000000000000000000000000000000000000011
>O33819_1rm6_0_768
MSPKLPQHGTVGVRTPLVDGVEKVTGKAKYTADIAAPDALVGRILRSPHAHARILAIDTSA
AEALEGVIAVCTGAETPVPFGVLPIAENEYPLARDKVRYRGDPVAAVAAIDEVTAEKALALI
KVDYEVLPAYMTPKAAMKAGAIALHDDKPNNILREVHAEFGDVAAAFAEADLIREKTYTF
AEVNHVHMELNATLAEYDPVRDMLTLNTTTQVPYYVHLKVAACLQMDSARIRVIKPFLG
GGFGARTEGLHFEIIAGLLARKAKGTVRLLQTREETFIAHRGRPWTEVKMKIGLKKDGKIA
ALALEATQAGGAYAGYGIITILYTGALMHGLYHIPAIKHDAWRVYTNTPPCGAMRGHGTV
DTRAAFEALLTEMGEELGIDSLKIRQINMLPQIPYVTMYAQRVMSYGVPECLEKVKAASGW
EERKGKLPKGRGLGIALSHFVSGTSTPKHWTGEPHATVNLKLDFDGGITLLTGAADIGQGS
NTMASQVAAEVLGVRLSRIRVISADSALTPKDNGSYSSRVTFMVGNASISAAEELKGVLVKA
AAKKLDAREEDIEVIDEMFMVSGSQDPGLSFQEVVKAAMVDSGTITVKGTYTCPTEFQGDK
KIRGSAIGATMGFCYAAQVVEASVDEITGKVTAHKVWVAVDVGKALNPLAVEGQTQGGV
WMGMGQALSEETVYDNGRMVHGNILDYRVPTIVESPDIEVIIVESMDPNGPFGAKEASEG
MLAGFLPAIHEAVYEAVGVRATDFPLSPDRITELLDAKEAAA
11111111000000000000000000000000000000000000000000000000000000000000000000000000000
00000000000000000000000000000000000000000000000000000000000000000000000000000000000
00000000000000000000000000000000000000000000000000000000000000000000000000000000000
00000000000000000000000000000000000000000000000000000000000000000000000000000000000
00000000000000000000000000000000000000000000000000000000000000000000000000000000000
00000000000000000000000000000000000000000000000000000000000000000000000000000000000
00000000000000000000000000000000000000000000000000000000000000000000000000000000000
00000000000000000000000000000000000000000000000000000000000000000000000000000000000
00000000000000000000000000000000000000000000000000000000000000000000000000000000000
0000000000000000000000
>P21179_3pq6_0_752
MSQHNEKNPHQHQSPLHDSSEAKPGMDSLAPEDGSHRPAAEPTPPGAQPTAPGSLKAPD
TRNEKLNSLEDVRKGSENYALTTNQGVRIADDQNSLRAGSRGPTLLEDFILREKITHFDHER
IPERIVHARGSAAHGYFQPYKSLSDITKADFLSDPNKITPVFVRFSTVQGGAGSADTVRDIRG
FATKFYTEEGIFDLVGNNTPIFFIQDAHKFPDFVHAVKPEPHWAIPQGQSAHDTFWDYVSL
QPETLHNVMWAMSDRGIPRSYRTMEGFGIHTFRLINAEGKATFVRFHWKPLAGKASLVWD
EAQKLTGRDPDFHRRELWEAIEAGDFPEYELGFQLIPEEDEFKFDFDLLDPTKLIPEELVPVQ
RVGKMVLNRNPDNFFAENEQAAFHPGHIVPGLDFTNDPLLQGRLFSYTDTQISRLGGPNF
HEIPINRPTCPYHNFQRDGMHRMGIDTNPANYEPNSINDNWPRETPPGPKRGGFESYQER
VEGNKVRERSPSFGEYYSHPRLFWLSQTPFEQRHIVDGFSFELSKVVRPYIRERVVDQLAHID
LTLAQAVAKNLGIELTDDQLNITPPPDVNGLKKDPSLSLYAIPDGDVKGRVVAILLNDEVRS

ADLLAILKALKAKGVHAKLLYSRMGEVTADDGTVLPIAATFAGAPSLTVDAVIVPCGNIADI
ADNGDANYYLMEAYKHLKPIALAGDARKFKATIKIADQGEEGIVEADSADGSFMDELLTL
MAAHRVWSRIPKIDKIPA
11111111111111111111111111000000000000000000000000000000000000000000000000000000
00000000000000000000000000000000000000000000000000000000000000000000000000000000
00000000000000000000000000000000000000000000000000000000000000000000000000000000
00000000000000000000000000000000000000000000000000000000000000000000000000000000
00000000000000000000000000000000000000000000000000000000000000000000000000000000
00000000000000000000000000000000000000000000000000000000000000000000000000000000
00000000000000000000000000000000000000000000000000000000000000000000000000000000
00000000000000000000000000000000000000000000000000000000000000000000000000000000
00000000000000000000000000000000000000000000000000000000000000000000000000000000
000000
>Q9ZNM8_1yr2_0_722
MKNRLWLAMAAPLALATPVAFAQTPPTLAKDQAMPSLPPYPASPQVPLVEDHFGEKVSDP
WRWLEADVRTDAKVAAWVQAQSAYTAAYLKQLPERAALEKRMKALIDYERFGLPQRRGA
SVFYSWNSGLMNQSQLLVRPADAPVGTKGRVLLDPNTWAKDGATALDAWAASDDGRLL
AYSVQDGGSDWRTVKFVGVADGKPLADELKWVKFSGLAWLGNDALLYSRFAEPKEGQAF
QALNYNQTVWLHRLGTPQSADQPVFATPELPKRGHGASVSSDGRWVVITSSEGTDPVNTV
HVARVTNGKIGPVTALIPDLKAQWDFVDGVGDQLWFVSGDGAPLKKIVRVDLSGSTPRFD
TVVPESKDNLESVGIAGNRLFASYIHDAKSQVLAFDLDGKPAGAVSLPGIGSASGLSGRPGD
RHAYLSFSSFTQPATVLALDPATAKTTPWEPVHLTFDPADFRVEQVFYPSKDGTKVPMFIVR
RKDAKGPLPTLLYGYGGFNVALTPWFSAGFMTWIDSGGAFALANLRGGGEYGDAWHDA
GRRDKKQNVFDDFIAAGEWLIANGVTPRHGLAIEGGSNGGLLIGAVTNQRPDLFAAASPA
VGVMDMLRFDQFTAGRYWVDDYGYPEKEADWRVLRRYSPYHNVRSGVDYPAILVTTADT
DDRVVPGHSLKYTAALQTAAIGPKPHLIRIETRAGHGSGKPIDKQIEETADVQAFLAHFTGL
TPRP
11111111111111111111111111111111111100000000000000000000000000000000000000000000
00000000000000000000000000000000000000000000000000000000000000000000000111100000
00000000000000000000000000000000000000000000000000000000000000111111110000000000
00000000000000000000000000000000000000000000000000000000000000000000000000000000
00000000000000000000000000000000000000000000000000000000000000000000000000000000
00000000000000000000000000000000000000000000000000000000000000000000000000000000
00000000000000000000000000000000000000000000000000000000000000000000000000000000
00000000000000000000000000000000000000000000000000000000000000000000000000000000
00000000000000000000000000000000000000000000000000000000000000000000000000000000
000000000000000000000001111111100000000000000000000000000
>Q2PS28_4gin_0_583
MLMKRLFAASLMLAFSSVSSVRAEEAVKPGAPWWKSAVFYQVYPRSFKDTNGDGIGDFKG
LTEKLDYLKGLGIDAIWINPHYASPNTDNGYDISDYREVMKEYGTMEDFDRLMAELKKRG
MRLMVDVVINHSSDQHEWFKSSRASKDNPYRDYYFWRDGKDGHEPNNYPSFFGGSAWE
KDPVTGQYYLHYFGRQQPDLNWDTPKLREELYAMLRFWLDKGVSGMRFDTVATYSKTPGF
PDLTPEQMKNFAEAYTQGPNLHRYLQEMHEKVFDHYDAVTAGEIFGAPLNQVPLFIDSRR
KELDMAFTFDLIRYDRALDRWHTIPRTLADFRQTIDKVDAIAGEYGWNTFFLGNHDNPRA
VSHFGDDRPQWREASAKALATVTLTQRGTPFIFQGDELGMTNYPFKTLQDFDDIEVKGFFQ
DYVETGKATAEELLTNVALTSRDNARTPFQWDDSANAGFTTGKPWLKVNPNYTEINAARE
IGDPKSVYSFYRNLISIRHETPALSTGSYRDIDPSNADVYAYTRSQDGETYLVVVNFKAEPRSF
TLPDGMHIAETLIESSSPAAPAAGAASLELQPWQSGIYKVK
11111111111111111111111111110000000000000000000000000000000000000000000000000000
00000000000000000000000000000000000000000000000000000000000000000000000000000000
00000000000000000000000000000000000000000000000000000000000000000000000000000000
00000000000000000000000000000000000000000000000000000000000000000000000000000000

00000000000000000000000000000000000000000000000000000000000000000000000000000000
00000000000000000000000000000000000000000000000000000000000000000000000000000000
00000000000000000000000000000000000000000000000000000000000000000000000000000000
000
>P51852_2q5l_0_544
MKLAEALLRALKDRGAQAMFGIPGDFALPFFKVAEETQILPLHTLSHEPAVGFAADAAARY
SSTLGVAAVTYGAGAFNMVNAVAGAYAEKSPVVVISGAPGTTEGNAGLLLHHQGRTLDTQ
FQVFKEITVAQARLDDPAKAPAEIARVLGAARALSRPVYLEIPRNMVNAEVEPVGDDPAWP
VDRDALAACADEVLAAMRSATSPVLMVCVEVRRYGLEAKVAELAQRLGVPVVTTFMGRG
LLADAPTPPLGTYIGVAGDAEITRLVEESDGLFLLGAILSDTNFAVSQRKIDLRKTIHAFDRAV
TLGYHTYADIPLAGLVDALLEGLPPSDRTTRGKEPHAYPTGLQADGEPIAPMDIARAVNDR
VRAGQEPLLIAADMGDCLFTAMDMIDAGLMAPGYYAGMGFGVPAGIGAQCVSGGKRILT
VVGDGAFQMTGWELGNCRRLGIDPIVILFNNASWEMLRTFQPESAFNDLDDWRFADMAA
GMGGDGVRVRTRAELKAALDKAFATRGRFQLIEAMIPRGVLSDTLARFVQGQKRLHAAPR
E
00000000000000000000000000000000000000000000000000000000000000000000000000000000
00000000000000000000000000001111100000000000000000000000000000000000000000000000
00000000000000000000000000000000000000000000000000000000000000000000000000000000
00000000000000000000000000000000000000000000000000000000000000000000000000000000
00000000000000000000000000000000000000000000000000000000000000000000000000000000
00000000000000000000000000000000000000000000000000000000000000000000000000000000
000000000000000000000000000000000000011111111
>Q3M5Z3_2nyn_0_566
MKTLSQAQSKTSSQQFSFTGNSSANVIIGNQKLTINDVARVARNGTLVSLTNNTDILQGIQA
SCDYINNAVESGEPIYGVTSGFGGMANVAISREQASELQTNLVWFLKTGAGNKLPLADVRA
AMLLRANSHMRGASGIRLELIKRMEIFLNAGVTPYVYEFGSIGASGDLVPLSYITGSLIGLDPS
FKVDFNGKEMDAPTALRQLNLSPLTLLPKEGLAMMNGTSVMTGIAANCVYDTQILTAIAM
GVHALDIQALNGTNQSFHPFIHNSKPHPGQLWAADQMISLLANSQLVRDELDGKHDYRD
HELIQDRYSLRCLPQYLGPIVDGISQIAKQIEIEINSVTDNPLIDVDNQASYHGGNFLGQYVG
MGMDHLRYYIGLLAKHLDVQIALLASPEFSNGLPPSLLGNRERKVNMGLKGLQICGNSIM
PLLTFYGNSIADRFPTHAEQFNQNINSQGYTSATLARRSVDIFQNYVAIALMFGVQAVDLRT
YKKTGHYDARACLSPATERLYSAVRHVVGQKPTSDRPYIWNDNEQGLDEHIARISADIAAG
GVIVQAVQDILPCLH
11111111111111111111111100000000000000000000000000000000000000000000000111111111
11111111000000000000000000000000000000000000000000000000000000000000000000000000
00000000000000000000000000000000000000000000000000000000000000000000000000000000
00000000000000000000000000000000000000000000000000111111100000000000000000000000
00000000000000000000000000000000000000000000000000000000000000000000000000000000
00000000000000000000000000000000000000000000000000000000000000000000000000000000
000000000000000000000000000000000000000000000000000000000000001111
>Q5SKJ3_1tuo_0_456
MSAPIRFGTEGFRGVIAREFTFATLHRLAEAYGRHLLERGGGLVVVGHDTRFLADAFARALS
GHLAGMGLKVVLLKGPVPTPLLSFAVRHLKAAGGAMLTASHNPPQYLGVKFKDATGGPI
AQEEAKAIEALVPEEARALEGAYETLDLREAYFEALKAHLDLKALSGFSGVLYHDSMGGAG
AGFLKGFLRHVGLEIPVRPIREEPHPLFHGVNPEPIPKNLGVTLAVLGPETPPSFAVATDGDA
DRVGVVLPGGVFFNPHQVLTTLALYRFRKGHRGRAVKNFAVTWLLDRLGERLGFGVTTTP
VGFKWIKEEFLKGDCFIGGEESGGVGYPEHLPERDGILTSLLLLESVAATGKDLAEQFKEVEA
LTGLTHAYDRLDLPLKAPLDLTPFREPRPLAGLTPKGVDTLDGVKWLYEEAWVLFRASGTE
PVVRIYVEAQSPELVRALLEEARKLVEG
00000000000000000000000000000000000000000000000000000000000000000000000000000000
00000000000000000000000000000000000000000000000000000000000000000000000000000000

00000000000000000000000000000000000000000000000000000000000000000000000000000000000
00000000000000000000000000000000000000000000000000000000000000000000000000000000000
00000000000000000000000000000000000000000000000011111111111111100000000000000000000
000000000001111100000000000000000000000000
>P32906_1g6i_0_548
MKNSVGISIATIVAIIAAIYYVPWYEHFERKSPGAGEMRDRIESMFLESWRDYSKHGWGYDV
YGPIEHTSHNMPRGNQPLGWIIVDSVDTLMLMYNSSTLYKSEFEAEIQRSEHWINDVLDFDI
DAEVNVFETTIRMLGGLLSAYHLSDVLEVGNKTVYLNKAIDLGDRLALAFLSTQTGIPYSSI
NLHSGQAVKNHADGGASSTAEFTTLQMEFKYLAYLTGNRTYWELVERVYEPLYKNNDLLN
TYDGLVPIYTFPDTGKFGASTIRFGSRGDSFYEYLLKQYLLTHETLYYDLYRKSMEGMKKHLL
AQSKPSSLWYIGEREQGLHGQLSPKMDHLVCFMGGLLASGSTEGLSIHEARRRPFFSKSDW
DLAKGITDTCYQMYKQSSSGLAPEIVVFNDGNIKQDGWWRSSVGDFFVKPLDRHNLQRPE
TVESIMFMYHLSHDHKYREWGAEIATSFFENTCVDCNDPKLRRFTSLSDCITLPTKKSNNM
ESFWLAETLKYLYILFLDEFDLTKVVFNTEAHPFPVLDEEILKSQSLTTGWSL
11111111111111111111111111111111110000000000000000000000000000000000000000000000000
00000000000000000000000000000000000000000000000000000000000000000000000000000000000
00000000000000000000000000000000000000000000000000000000000000000000000000000000000
00000000000000000000000000000000000000000000000000000000000000000000000000000000000
00000000000000000000000000000000000000000000000000000000000000000000000010000000000
00000000000000000000000000000000000000000000000000000000000000000000000000000000000
0000000000000000000000000000000000000000000000
>O52575_3c2u_0_537
MNIQNPVLKGFNPDPSIVRAGDDYYIATSTFEWFPGVQIHHSKDLVHWHLVAHPLSTTEFL
DMKGNPDSGGIWAPDLSYADGKFWLIYTDVKVVDGMWKDCHNYLTTAEDIKGPWSKPIL
LNGAGFDASLFHDPSGKKYLVNMYWDQRVYHHNFYGIALQEYSVAEEKLIGKPEIIYKGTD
IAYTEGPHLYYINDMYYLMTAEGGTTYQHSETIARSKTIHGPYEIQPDYPLLSAWKEVHNPL
QKCGHASLVETQNGQWYLAHLTGRPLPAPAGFPSREREQHAFCPLGRETAIQKIEWQDGW
PVVVGGQQGSLEVEAPDLPQQEWAPTYEERDDFDKDTLNINFQTLRIPFSEHLGSLTARPGF
LRLYGRESLQSKFTQAHIARRWQSFNFDAGTSVEFSPNSFQQMAGLTCYYNTENWSSIHVT
WNEEKGRIIDLVTADNGTFSMPLAGAEIPIPDEVKTVHFKVSVRGRIYQYAYSFDGETFHTLP
IELPSWKLSDDYVRGGGFFTGAFVGINAIDITGTALPADFDYFTYKELD
00000000000000000000000000000000000000000000000000000000000000000000000000000000000
00000000000000000000000000000000000000000000000000000000000000000000000000000000000
00000000000000000000000000000000000000000000000000000000000000000000000000000000000
00000000000000000000000000000000000000000000000000000000000000000000000000000000000
00000000000000000000000000000000000000000000000000000000000000000000000000000000000
00000000000000000000000000000000000000000000000000000000000000000000000000000000000
0000000000000000000000000000000000000001
>P95480_2apg_0_537
MNKPIKNIVIVGGGTAGWMAASYLVRALQQQANITLIESAAIPRIGVGEATIPSLQKVFFDF
LGIPEREWMPQVNGAFKAAIKFVNWRKSPDPSRDDHFYHLFGNVPNCDGVPLTHYWLRK
REQGFQQPMEYACYPQPGALDGKLAPCLSDGTRQMSHAWHFDAHLVADFLKRWAVERG
VNRVVDEVVDVRLNNRGYISNLLTKEGRTLEADLFIDCSGMRGLLINQALKEPFIDMSDYL
LCDSAVASAVPNDDARDGVEPYTSSIAMNSGWTWKIPMLGRFGSGYVFSSHFTSRDQATAD
FLKLWGLSDNQPLNQIKFRVGRNKRAWVNNCVSIGLSSCFLEPLESTGIYFIYAALYQLVKH
FPDTSFDPRLSDAFNAEIVHMFDDCRDFVQAHYFTTSRDDTPFWLANRHDLRLSDAIKEKV
QRYKAGLPLTTTSFDDSTYYETFDYEFKNFWLNGNYYCIFAGLGMLPDRSLPLLQHRPESIE
KAEAMFASIRREAERLRTSLPTNYDYLRSLRDGDAGLSRGQRGPKLAAQESL
10000000000000000000000000000000000000000000000000000000000000000000000000000000000
00000000000000000000000000000000000000000000000000000000000000000000000000000000000
00000000000000000000000000000000000000000000000000000000000000000000000000000000000

00000000000000000000000000000000000000000000000000000000000000000000000000000000000
00000000000000000000000000000000000000000000000000000000000000000000000000000000000
00000000000000000000000000000000000000000000000000000000000000000000000000000000000
0000000000000000000011111111111111111111
>Q19905_2o3j_0_480
MTDQVFGKVSKVVCVGAGYVGGPTCAMIAHKCPHITVTVVDMNTAKIAEWNSDKLPIYE
PGLDEIVFAARGRNLFFSSDIPKAIAEADLIFISVNTPTKMYGRGKGMAPDLKYVESVSRTIA
QYAGGPKIVVEKSTVPVKAAESIGCILREAQKNNENLKFQVLSNPEFLAEGTAMKDLANPD
RVLIGGESSPEGLQAVAELVRIYENWVPRNRIITTNTWSSELSKLVANAFLAQRISSINSISAVC
EATGAEISEVAHAVGYDTRIGSKFLQASVGFGGSCFQKDVLSLVYLCESLNLPQVADYWQG
VININNWQRRRFADKIIAELFNTVTDKKIAIFGFAFKKNTGDTRESSAIHVIKHLMEEHAKLS
VYDPKVQKSQMLNDLASVTSAQDVERLITVESDPYAAARGAHAIVVLTEWDEFVELNYSQI
HNDMQHPAAIFDGRLILDQKALREIGFRTFAIGTSPDQAYNLFGTAGY
11111000000000000000000000000000000000000000000000000000000000000000000000000000000
00000000000000000000000000000000000000000000000000000000000000000000000111100000000
00000000000000000000000000000000000000000000000000000000000000000000000000000000000
00000000000000000000000000000000000000000000000000000000000000000000000000000000000
00000000000000000000000000000000000000000000000000000000000000000000000000000000000
000000000000000000000000000000000000000000000000000000001111111111
>B2HT54_3qbp_0_473
MAHSAHDDADNDTEYRIEHDTMGEVRVPAKALWRAQTQRAVENFPISGRGLERAQIRAL
GLLKGACAQVNMDLGLLAPEKAEAIIAAAAEIADGQHDDQFPIDVFQTGSGTSSNMNTN
EVIASIAGANGVAVHPNDDVNMSQSSNDTFPTATHIAATEAAVSHLIPALEILQDALATKAL
EWQSVVKSGRTHLMDAVPVTLGQEFSGYARQIEAGIERVRATLPRLGELAIGGTAVGTGLN
APEGFGVKVVSVLVSQTGLPQLRTAANSFEAQAARDGLVEASGALRTIAVSLTKIANDIRW
MGSGPLTGLAEIQLPDLQPGSSIMPGKVNPVLPEAVTQVAAQVIGNDAAVAWGGANGAFE
LNVYIPMMARNILESFTLLTNVSKLFAQRCIAGLTANAEHLRELAESSPSIVTPLNSAIGYEEA
AAVAKQALKERKTIRQTVIDRGLIGDKLSLEELDRRLDVLAMAKVEAT
11111111111111111111111110000000000000000000000000000000000000000000000000000000000
00000000000000000000000000000000000000000000000000000000000000000000000000000000000
00000000000000000000000000000000000000000000000000000000000000000000000000000000000
00000000000000000000000000000000000000000000000000000000000000000000001111100000000
00000000000000000000000000000000000000000000000000000000000000000000000000000000000
00000000000000000000000000000000000000000000000000000000011
>Q02934_2xfg_53_515
ENKNGGYLPEEEIPDQPPATGAFNYGEALQKAIFFYECQRSGKLDPSTLRLNWRGDSGLDD
GKDAGIDLTGGWYDAGDHVKFNLPMSYSAAMLGWAVYEYEDAFKQSGQYNHILNNIKW
ACDYFIKCHPEKDVYYYQVGDGHADHAWWGPAEVMPMERPSYKVDRSSPGSTVVAETSA
ALAIASIIFKKVDGEYSKECLKHAKELFEFADTTKSDDGYTAANGFYNSWSGFYDELSWAA
VWLYLATNDSSYLDKAESYSDKWGYEPQTNIPKYKWAQCWDDVTYGTYLLLARIKNDNG
KYKEAIERHLDWWTTGYNGERITYTPKGLAWLDQWGSLRYATTTAFLACVYSDWENGDK
EKAKTYLEFARSQADYALGSTGRSFVVGFGENPPKRPHHRTAHGSWADSQMEPPEHRHVL
YGALVGGPDSTDNYTDDISNYTCNEVACDYNAGFVGLLAKMYKLYG
11111111111111111110000000000000000000000000000000000000000000000000000000000000000
00000000000000000000000000000000000000000000000000000000000000000000000000000000000
00000000000000000000000000000000000000000000000000000000000000000000000000000000000
00000000000000000000000000000000000000000000000000000000000000000000000000000000000
00000000000000000000000000000000000000000000000000000000000000000000000000000000000
000000000000000000000000000000000000000000000000
>Q65AT1_2ckf_0_453
MSGDTTLVDTVNASQSRQVFWDRDVYDLEIERIFSRAWLMLGHKSLLPKPGDFITTYMAED

KIILSHQSDGTFRAFINSCTHRGNQICHADSGNAKAFVCNYHGWVYGQDGSLVDVPLESR
CYHNKLDKQELAAKSVRVETYKGFIFGCHDPEAPSLEDYLGEFRFYLDTIWEGGGAGLELL
GPPMKSLLHCNWKVPVENFVGDGYHVGWTHAAALGQIGGPLAGLAGNRADIPFDDLGL
QFTTRHGHGFGVIDNAAAAIHRKGDGWNKYLEDTRGEVRRKFGADRERLYVGHWNGAI
FPNCSFLYGTNTFKIWHPRGPHEIEVWTYTMVPSDADPATKSAIQREATRTFGTAGTLESDD
GENMSSATYVNRGVITRDGMMNSTMGVGYEGPHPVYPGIVGISFIGETSYRGFYRFWKEMI
DAPDWASVKANDDNWDSVFTNRNFWNEKLNAAE
00000000000000000000000000000000000000000000000000000000000000000000000000000000
00000000000000000000000000000000000000000000000000000000000000000000000000000000
00000000000000000000000000000000000000000000000000111111111111111111100000000000
00000000000000000000000000000000000000000000000000000000000000000000000000000000
00000000000000000000000000000000000000000000000000000000000000000000000000000000
00000000000000000000000000000000000011
>Q8I501_3p1w_2_458
EHYDVIILGTGLKECILSGLLSHYGKKILVLDRNPYYGGETASLNLTNLYNTFKPKENIPSKYG
ENRHWNVDLIPKFILVGGNLVKILKKTRVTNYLEWLVVEGSYVYQHQKKGFLTSEKFIHKV
PATDMEALVSPLLSLMEKNRCKNFYQYVSEWDANKRNTWDNLDPYKLTMLEIYKHFNLC
QLTIDFLGHAVALYLNDDYLKQPAYLTLERIKLYMQSISAFGKSPFIYPLYGLGGIPEGFSRMC
AINGGTFMLNKNVVDFVFDDDNKVCGIKSSDGEIAYCDKVICDPSYVMHLKNKIKKIGQVI
RCICILSNPIPETNQTNSCQIIIPQNQLNRKSDIYINLVSFQHGVTLKGKYIAIVSATVETNNPI
KEIEKPLELLGTIEEKFVKISDLYVSTSKKPADNIFVTSSYDATSHFETATNDLLQIWENLWGQ
KLNFDDLNTNADGEAPDFN
00000000000000000000000000000000000000000000000000000000000000000000000000000000
00000000000000000000000000000000000000000000000000000000000000000000000000000000
00000000000000000000000000000000000000000000000000000000000000000000000000000000
00110000000000000000000000000000000000000000000000000000000000000000000000000000
00000000000000000000000000000000000000000000000000000000000000000000000000000000
0000000000000000000000000011111111111111
>Q2SWD3_3ue9_0_447
MSASAVNVTPGRNVVVVGTQWGDEGKGKIVDWLTDHAQGVVRFQGGHNAGHTLIIGGK
KTILRLIPSGIMREGVACYIGNGVVLSPEALFKEIGELEEAGLSVRERLFISEATTLILPYHIAID
QAREARKGAGKIGTTGRGIGPAYEDKVGRRALRVQDLFDARTFADRLRENLDFHNFVLTQ
YLGGAAVDFQATLDTMLGYADRLRPMVADVSRRLYEENHAGRNLLFEGAQGTLLDIDHG
TYPFVTSSNCVAGAAAAGAGVGPQKLNYILGITKAYCTRVGSGPFPSELYDADNPSRQDQI
GITLANVGKEFGSVTGRPRRTGWLDAAALRRSIQINGVSGLCMTKLDVLDGLDEVKLCVG
YKIDGEDADLLPRGAAEVARCEPVYETFGGWKESTVGINSWDALPANARAYLTRVQEVAG
VPIDMVSTGPDRDETILLRHPFKV
11111110000000000000000000000000000000000000000000011111100000000000000000000000
00000000000000000000000000000000000000000000011111111110000000000000000000000000
00000000000000000000000000000000000000000000000000000000000000000000000000000000
00000000000000000000000000000000000000000000000000000000000000000000000000000000
00000000000000000000000000000000000000000000000000000000000000000000000000000000
00000000000000000000000000000000
>P30996_2a97_0_438
MPVAINSFNYNDPVNDDTILYMQIPYEEKSKKYYKAFEIMRNVWIIPERNTIGTNPSDFDPP
ASLKNGSSAYYDPNYLTTDAEKDRYLKTTIKLFKRINSNPAGKVLLQEISYAKPYLGNDHTPI
DEFSPVTRTTSVNIKLSTNVESSMLLNLLVLGAGPDIFESCCYPVRKLIDPDVVYDPSNYGFG
SINIVTFSPEYEYTFNDISGGHNSSTESFIADPAISLAHELIHALHGLYGARGVTYEETIEVKQA
PLMIAEKPIRLEEFLTFGGQDLNIITSAMKEKIYNNLLANYEKIATRLSEVNSAPPEYDINEYK
DYFQWKYGLDKNADGSYTVNENKFNEIYKKLYSFTESDLANKFKVKCRNTYFIKYEFLKVP
NLLDDDIYTVSEGFNIGNLAVNNRGQSIKLNPKIIDSIPDKGLVEKIVKFCKSVIPRKGTK

10000000000000000000000000000000000000000000000000000000000000000000000000000000000
00000000000000000000000000000000000000000000000000000000000000000000000000000000000
00000000000000000000000000000000000000111111111100000000000000000000000000000000001
11111111111000000000000000000000000000000000000000000000000000000000000000000000000
00000000000000000000000000000000000000000000000000000000000000000000000000000000000
111111111111111111111111
>A9X6P7_3ubm_0_435
MGTTSENSKPLDGIKVIDFGGVQSVPSAAQLLAWYGADVIKIERVGVGDITRNQLRDIPDA
DALYFTMLNCNKRSVELNTKTPEGKAVFEKCIKWADILLENFRPGAMERMGFTWEYLQQL
NPRLIYGTVKGFGENSPWAGVSAYENVAQCAGGATSTTGYWNGAPLVDGQAPGNNNGPL
VSAAALGDSNTGNHLLIGVLAALFGRERTGKGQKISVSMQDAVLNLCRVKLRDQQRLERV
GYLEEYPQYPNGKFGDTVPRGGNAGGGGQPGWILKCKGWETDDNAYIYCTVQEQDWGP
TCEAIGKPEWATDPKYNTAKARETHMFEIFAAIEKAIADKTKYEAVAHLAKYRVPCSPVLS
MKEIAEAPDLRESGTIVEVQQPKRGTFLTINPIKFSGFTPEIKAAPLLGQHTDEVLAELGYSA
EEIKSLRDKKITCA
11111100000000000000000000000000000000000000000000000000000000000000000000000000000
00000000000000000000000000000000000000000000000000000000000000000000000000000000000
00000000000000000000000000000000000000000000000000000000000000000000000000000000000
00000000000000000000000000000000000000000000000000000000000000000000000000000000000
00000000000000000000000000000000000000000000000000000000000000000000000000000000000
000000000000000000000
>C4XAX5_3plr_0_387
MKITISGTGYVGLSNGVLIAQNHEVVALDIVQAKVDMLNQKISPIVDKEIQEYLAEKPLNFR
ATTDKHDAYRNADYVIIATPTDYDPKTNYFNTSTVEAVIRDVTEINPNAVMIIKSTIPVGFTR
DIKERLGIDNVIFSPEFLREGRALYDNLHPSRIVIGERSARAERFADLLKEGAIKQDIPTLFTDS
TEAEAIKLFANTYLALRVAYFNELDSYAESQGLNSKQIIEGVCLDPRIGNHYNNPSFGYGGY
CLPKDTKQLLANYESVPNNIIAAIVDANRTRKDFIADSILARKPKVVGVYRLIMKSGSDNFR
ASSIQGIMKRIKAKGIPVIIYEPVMQEDEFFNSRVVRDLNAFKQEADVIISNRMAEELADVAD
KVYTRDLFGND
00000000000000000000000000000000000000000000000000000000000000000000000000000000000
00000000000000000000000000000000000000000000000000000000000000000000000000000000000
00000000000000000000000000000000000000000000000000000000000000000000000000000000000
00000000000000000000000000000000000000000000000000000001111111111100000000000000000
00000000000000000000000000000000000000000000000000000011
>P39683_1vlp_0_428
MSEPVIKSLLDTDMYKITMHAAVFTNFPDVTVTYKYTNRSSQLTFNKEAINWLKEQFSYLG
NLRFTEEEIEYLKQEIPYLPSAYIKYISSSNYKLHPEEQISFTSEEIEGKPTHYKLKILVSGSWKD
TILYEIPLLSLISEAYFKFVDIDWDYENQLEQAEKKAETLFDNGIRFSEFGTRRRRSLKAQDLI
MQGIMKAVNGNPDRNKSLLLGTSNILFAKKYGVKPIGTVAHEWVMGVASISEDYLHANK
NAMDCWINTFGAKNAGLALTDTFGTDDFLKSFRPPYSDAYVGVRQDSGDPVEYTKKISHH
YHDVLKLPKFSKIICYSDSLNVEKAITYSHAAKENGMLATFGIGTNFTNDFRKKSEPQVKSE
PLNIVIKLLEVNGNHAIKISDNLGKNMGDPATVKRVKEELGYTERSWSGDNEAHRWT
00000000000000000000000000000000000000000000000000000000000000000000000000000000000
00000000000000000000000000000000000000000000000000000000000000000000000000000000000
00000000000000000000000000000000000000000000000000000000000000000000000000000000000
00000000000000000000000000000000000000000000000000000000000000000000000000000000000
00000000000000000000000000000000000000000000000000000000000000000000000000000000000
01111111111111
>Q53685_2iyf_0_429
MTTQTTPAHIAMFSIAAHGHVNPSLEVIRELVARGHRVTYAIPPVFADKVAATGPRPVLYHS
TLPGPDADPEAWGSTLLDNRRTFLNDAIQALPQLADAYADDIPDLVLHDITSYPARVLARR

WGVPAVSLSPNLVAWKGYEEEVAEPMWREPRQTERGRAYYARFEAWLKENGITEHPDTFA
SHPPRSLVLIPKALQPHADRVDEDVYTFVGACQGDRAEEGGWQRPAGAEKVVLVSLGSAF
TKQPAFYRECVRAFGNLPGWHLVLQIGRKVTPAELGELPDNVEVHDWVPQLAILRQADLF
VTHAGAGGSQEGLATATPMIAVPQAVDQFGNADMLQGLGVARKLATEEATADLLRETAL
ALVDDPEVARRLRRIQAEMAQEGGTRRAADLIEAELPARHERQEPVGDRPNVGDRPAGVR
SDRQRSAL
11111000000000000000000000000000000000000000000000000000000000000000000000000000000
00000000000000000000000000000000000000000000000000000000000000000000000000000000000
00000000000000000000000000000000000000000000000000000000000000000000000000000000000
00000000000000000000000000000000000000000000000000000000000000000000000000000000000
00000000000000000000000000000000000000000000000000000000000000000000111111111111111
111111111111111
>P09955_3glj_15_415
HHSGEHFEGEKVFRVNVEDENDISLLHELASTRQIDFWKPDSVTQIKPHSTVDFRVKAEDIL
AVEDFLEQNELQYEVLINNLRSVLEAQFDSRVRTTGHSYEKYNNWETIEAWTKQVTSENPD
LISRTAIGTTFLGNNIYLLKVGKPGPNKPAIFMDCGFHAREWISHAFCQWFVREAVLTYGYE
SHMTEFLNKLDFYVLPVLNIDGYIYTWTKNRMWRKTRSTNAGTTCIGTDPNRNFDAGWC
TTGASTDPCDETYCGSAAESEKETKALADFIRNNLSSIKAYLTIHSYSQMILYPYSYDYKLPEN
NAELNNLAKAAVKELATLYGTKYTYGPGATTIYPAAGGSDDWAYDQGIKYSFTFELRDKGR
YGFILPESQIQATCEETMLAIKYVTNYVLGHL
11100000000000000000000000000000000000000000000000000000000000000000000000000000000
00000000000000000000000000000000000000000000000000000000000000000000000000000000000
00000000000000000000000000000000000000000000000000000000000000000000000000000000000
00000000000000000000000000000000000000000000000000000000000000000000000000000000000
000000000000000000000000000000000000000000000000000000000000000000000
>Q2T0S5_4izo_0_397
MTALPNPNSPILPGAWLGMVGGGQLGRMFCFAAQAMGYRVAVLDPDPTSPAGAVADKH
LRAAYDDEAALAELAQLCDAVSTEFENVPAASLDFLAQSTFVAPAGRCVAIAQDRIAEKRFI
AASGVPVAPHVVIESAAQLAALADADLAAVLPGILKTARLGYDGKGQVRVATAQEARDAY
GSLGGVPCVLEKRLPLKYEVSALIARGANGASAVFPLAQNTHHGGILSLSVVPAPAASDAL
VRDAQQAAARIADSLDYVGVLCVEFFVLEDGSLVANEMAPRPHNSGHYTVDACETSQFEQ
QVRAMTRLPLGSTRQHSPAAMLNVLGDVWFASGASGEPVTPPWDQVAAMPTARLHLYG
KEEARVGRKMGHVNFTAATLDEAVAGATACARLLRIPLD
11111111000000000000000000000000000000000000000000000000000000000000000000000000001
11000000000000000000000000000000000000000000000000000000000000000000000000001111100
00000000000000000000000000000000000000000000000000000000000000000000000000000000000
00000000000000000000000000000000000000000000000000000000000000000000000000000000001
111100000000000000000000000000000000000000000000000000000000000001
>Q81X75_3uwd_0_393
MNKKSIRDVDLKGKRVFCRVDFNVPMKEGKITDETRIRAALPTIQYLVEQGAKVILASHLG
RPKGQAVEELRLTPVAARLGELLGKDVKKADEAFGPVAQEMVAAMNEGDVLVLENVRFY
AGEEKNDAELAKEFAALADIFVNDAFGAAHRAHASTAGIADYLPAVSGLLMEKELEVLGK
ALSNPERPFTAIIGGAKVKDKIGLIRHLLDKVDNLIIGGGLAYTFVKALGHEIGLSLCEDDKIE
LAKEFMQLAKEKGVNFYMPVDVVITEEFSETATTKIVGIDSIPSNWEGVDIGPKTREIYADVI
KNSKLVVWNGPMGVFEMTPFAEGTKAVGQALADAEGTYSVIGGGDSAAAVEKFGMADK
MSHISTGGGASLEFMEGKELPGVVCLNDK
00000000000000000000000000110000000000000000000000000000000000000000000000000000000
00000000000000000000000000000000000000000000000000000000000000000000000000000000000
00000000000000000000000000000000000000000000000000000000000000000000000000000000000
00000000000000000000000000000000000000000000000000000000000000000000000000000000000
00000000000000000000000000000000000000000000000000000000000000

>Q71YC9_3k0b_0_381
MKSFQLVATAASGLEAIVGKEVARLGYDPKVENGKVYFEGDLSAIARANLWLRVADRVKIV
VGVFKATTFDELFEKTKALPWEDYLPLDAQFPVAGKSVKSTLYSVPDCQAIVKKAIVNRVSE
KYRRSGRLMETGALFKLEVSILKDEVTLTIDTSGAGLHKRGYRLAQGSAPIKETMAAALVLL
TSWHPDRPFYDPVCGSGTIPIEAALIGQNIAPGFNREFVSETWDWMPKQVWADARQEAED
LANYDQPLNIIGGDIDARLIEIAKQNAVEAGLGDLITFRQLQVADFQTEDEYGVVVANPPY
GERLEDEEAVRQLYREMGIVYKRMPTWSVYVLTSYELFEEVYGKKATKKRKLYNGYLRTDL
YQYWGPRKPRPKKED
00000000000000000000000000000000000000000000000000000000000000000000000000000000
00000000000000000000000000000000000000000000000000000000000000000000000000000000
00000000000000000000000000000000000000000000000000000000000000000000000000000000
00000000000000000000000000000000000000000000000000000000000000000000000000000000
000000000000000000000000001111000000000111111111
>Q8GWW7_1vkp_0_382
MEESRESPAEHGYYMPAEWDSHAQTWIGWPERQDNWRHNALPAQRVFADVAKAISKFEP
VTVCASPAQWENARKQLPEDIRVVEMSMNDSWFRDSGPTFIVRKRPVKLSSLNRNIAGID
WNFNAWGGANDGCYNDWSHDLLVSRKILALERIPRFQHSMILEGGSIHVDGEGTCLVTEE
CLLNKNRNPHMSKEQIEEELKKYLGVQSFIWLPRGLYGDEDTNGHIDNMCCFARPGVVLL
SWTDDETDPQYERSVEALSVLSNSIDARGRKIQVIKLYIPEPLYMTEEESSGITQDGEAIPRLA
GTRLAASYVNFYIANGGIIAPQFGDPIRDKEAIRVLSDTFPHHSVVGIENAREIVLAGGNIHC
ITQQQPAEPTSVAENGH
11110000000000000000000000000000000000000000000000000000000000000000000000000000
00000000000000000000011111100000000000000000000000000000000000000000000000000000
00000000000000000000000000000000000000000000000000000000000000000000000000000000
00000000000000000000000000000000000000000000000000000000000000000000000000000000
0000000000000000000000000000000000000000001111111
>D0VWY2_2q9t_0_378
MDINGGGATLPQALYQTSGVLTAGFAQYIGVGSGNGKAAFLNNDYTKFQAGVTNKNVH
WAGSDSKLSATELSTYASAKQPTWGKLIQVPSVGTSVAIPFNKSGSAAVDLSVQELCGVFSG
RINTWDGISGSGRTGPIVVVYRSESSGTTELFTRFLNAKCNAETGNFAVTTTFGTSFSGGLPA
GAVAATGSQGVMTALAAGDGRITYMSPDFAAPTLAGLDDATKVARVGKNVATNTQGVSP
AAANVSAAIGAVPVPAAADRSNPDAWVPVFGPDNTAGVQPYPTSGYPILGFTNLIFSQCYA
DATQTTQVRDFFTKHYGASNNNDAAITANAFVPLPTAWKATVRASFLTASNALSIGNTNV
CNGIGRPLLEHHHHHH
00000000000000000000000000000000000000000000000000000000000000000000000000000000
00000000000000000000000000000000000000000000000000000000000000000000000000000000
00000000000000000000000000000000000000000000000000000000000000000000000000000000
00000000000000000000000000000000000000000000000000000000000000000000000000000000
000000000000000000000000000000000000000011111
>P0A542_3tz6_1_344
GLSIGIVGATGQVGQVMRTLLDERDFPASAVRFFASARSQGRKLAFRGQEIEVEDAETADPS
GLDIALFSAGSAMSKVQAPRFAAAGVTVIDNSSAWRKDPDVPLVVSEVNFERDAHRRPKGI
IANPNCTTMAAMPVLKVLHDEARLVRLVVSSYQAVSGSGLAGVAELAEQARAVIGGAEQL
VYDGGALEFPPPNTYVAPIAFNVVPLAGSLVDDGSGETDEDQKLRFESRKILGIPDLLVSGTC
VRVPVFTGHSLSINAEFAQPLSPERARELLDGATGVQLVDVPTPLAAAGVDESLVGRIRRDP
GVPDGRGLALFVSGDNLRKGAALNTIQIAELLTADL
00000000000000000000000000000000000000000000000000000000000000000000000000000000
00000000000000000000000000000000000000000000000000000000000000000000000000000000
00000000000000000000000000000000000000000000000000000000000000000000000000000000
00000000000000000000000000000000000000000000000000000000000000000000000000000000
000000000011

>P07752_1f2j_1_370
SKRVEVLLTQLPAYNRLKTPYEAELIETAKKMTAPGKGLLAADESTGSCSKRFAGIGLSNTAE
HRRQYRALMLECEGFEQYISGVILHDETVYQKAKTGETFPQYLRRRGVVPGIKTDCGLEPLV
EGAKGEQMTAGLDGYIKRAKKYYAMGCRFCKWRNVYKIQNGTVSEAVVRFNAETLARYA
ILSQLCGLVPIVEPEVMIDGTHDIETCQRVSQHVWSEVVSALHRHGVVWEGCLLKPNMVV
PGAESGLKGHAEQVAEYTVKTLARVIPPALPGVTFLSGGLSEVMASEYLNAMNNCPLPRP
WKLTFSYARALQSSAIKRWGGKESGVEAGRRAFMHRAKMNSLAQLGKYNRADDDKDSQ
SLYVAGNT
00000000000000000000000000000000000000000000000000000000000000000000000000000000000
00000000000000000000000000000000000000000000000000000000000000000000000000000000000
00000000000000000000000000000000000000000000000000000000000000000000000000000000000
00000000000000000000000000000000000000000000000000000000000000000000000000000000000
00000000000000000000000001111111111111
>F2YGR7_3q60_174_540
EAGDSFMRDLLKREEELIGYCREEALKEPAAMVEAVTATVWPQNAETTVDSLLSQGERKLK
LVEPLRVGDRSVVFLVRDVERLEDFALKVFTMGAENSRSELERLHEATFAAARLLGESPEEA
RDRRRLLLPSDAVAVQSQPPFAQLSPGQDDYAVANYLLLMPAASVDLELLFSTLDFVYVFRG
DEGILALHILTAQLIRLAANLQSKGLVHGHFTPDNLFIMPDGRLMLGDVSALWKVGTRGPA
SSVPVTYAPREFLNASTATFTHALNAWQLGLSIYRVWCLFLPFGLVTPGIKGSWKRPSLRVPG
TDSLAFGSCTPLPDFVKTLIGRFLNFDRRRRLLPLEAMETPEFLQLQNEISSSLSTGQ
10000000000000000000000000000000000000000000000000000000000000000000000000000000000
00000000000000000000000000000000001111111111000000000000000000000001110000000000000
00000000000000000000000000000000000000000000000000000000000000000000000000000000000
00000000000000000000000000000000000000000000000000000000000000000000000000000000000
00000000000000000000000000000000111
>Q6D4Z0_3ly1_41_393
ETQPESAAFTAPSTDNPIRINFNENPLGMSPKAQAAARDAVVKANRYAKNEILMLGNKLA
AHHQVEAPSILLTAGSSEGIRAAIEAYASLEAQLVIPELTYGDGEHFAKIAGMKVTKVKMLD
NWAFDIEGLKAAVAAYSGPSIVYLVNPNNPTGTITPADVIEPWIASKPANTMFIVDEAYAEFV
NDPRFRSISPMITQGAENIILLKTFSKIHAMAGMRVGYAVAHPTVIALMGRYVAGEKINFSG
VDAALASMNDSAFITYSKKSNDVSRQILLKALEDLKLPYLPSEGNFVFHQLVVPLKDYQTH
MADAGVLIGRAFPPADNWCRISLGTPQEMQWVADTMREFRKKSWI
11111100000000000000000000000000000000000000000000000000000000000000000000000000000
00000000000000000000000000000000000000000000000000000000000000000000000000000000000
00000000000000000000000000000000000000000000000000000000000000000000000000000000000
00000000000000000000000000000000000000000000000000000000000000000000000000000000000
000000000000000000000
>Q8YXQ9_1vqu_0_361
MTSSPTSTQESSTSWYLLLQQLIDGESLSRSQAAELMQGWLSEAVPPELSGAILTALNFKGVS
ADELTGMAEVLQSQSKMGTGENYSQLPITNSPFSIIDTCGTGGDGSSTFNISTAVAFVAAAYG
VPVAKHGNRSASSLTGSADVLEALGVNLGASPEKVQAALQEVGITFLFAPGWHPALKAVA
TLRRTLRIRTVFNLLGPLVNPLRPTGQVVGLFTPKLLTTVAQALDNLGKQKAIVLHGRERLD
EAGLGDLTDLAVLSDGELQLTTINPQEVGVTPAPIGALRGGDVQENAEILKAVLQGKGTQA
QQDAVALNAALALQVAGAVPLLDHAQGVSVAKEILQTGTAWAKLAQLVYFLGN
11111111111100000000000000000000000000000000000000000000000000000000000000000000111
11111111000000000000011100000000000000000000000000000000000000000000000000000000000
00000000000000000000000000000000000000000000000000000000000000000000000000000000000
00000000000000000000000000000000000000000000000000000000000000000000000000000000000
000000000000000000000000000000
>D0VP31_3vmw_36_361
SNGPQGYASMNGGTTGGAGGRVEYASTGAQIQQLIDNRSRSNNPDEPLTIYVNGTITQGNS

PQSLIDVKNHRGKAHEIKNISIIGVGTNGEFDGIGIRLSNAHNIIIQNVSIHHVREGEGTAIEV
TDDSKNVWIDHNEFYSEFPGNGDSDYYDGLVDMKRNAEYITVSWNKFENHWKTMLVGH
TDNASLAPDKITYHHNYFNNLNSRVPLIRYADVHMFNNYFKDINDTAINSRVGARVFVEN
NYFDNVGSGQADPTTGFIKGPVGWFYGSPSTGYWNLRGNVFVNTPNSHLNSTTNFTPPYS
YQVQSATQAKSSVEQHSGVGVIN
11000000000000000000000000000000000000000000000000000000000000000000000000000000000
00000000000000000000000000000000000000000000000000000000000000000000000000000000000
00000000000000000000000000000000000000000000000000000000000000000000000000000000000
00000000000000000000000000000000000000000000000000000000000000000000000000000
>Q5SKS3_2ism_0_351
MDRRRFLVGLLGLGLARGQGLRVEEVVGGLEVPWALAFLPDGGMLIAERPGRIRLFREGRL
STYAELSVYHRGESGLLGLALHPRFPQEPYVYAYRTVAEGGLRNQVVRLRHLGERGVLDRV
VLDGIPARPHGLHSGGRIAFGPDGMLYVTTGEVYERELAQDLASLGGKILRLTPEGEPAPGN
PFLGRRGARPEVYSLGHRNPQGLAWHPKTGELFSSEHGPSGEQGYGHDEVNLIVPGGNYG
WPRVVGRGNDPRYRDPLYFWPQGFPPGNLAFFRGDLYVAGLRGQALLRLVLEGERGRWRV
LRVETALSGFGRLREVQVGPDGALYVTTSNRDGRGQVRPGDDRVLRLL
11111111111111111110000000000000000000000000000000000000000000000000000000000000000
00000000000000000000000000000000000000000000000000000000000000000000000000000000000
00000000000000000000000000000000000000000000000000000000000000000000000000000000000
00000000000000000000000000000000000000000000000000000000000000000000000000000000000
00000000000000000000
>Q5LCX7_3fhl_1_351
EIIKTGLAAFGMSGQVFHAPFISTNPHFELYKIVERSKELSKERYPQASIVRSFKELTEDPEIDLI
VVNTPDNTHYEYAGMALEAGKNVVVEKPFTSTTKQGEELIALAKKKGLMLSVYQNRRWD
ADFLTVRDILAKSLLGRLVEYESTFARYRNFIKPNTWKETGESGGGLTYNLGSHLIDQAIQLF
GMPEAVFADLGILREGGKVDDYFIIHLLHPSLAPNVKITLKASYLMREAEPRFALHGTLGSY
VKYGVDKQEAALLAGEIPERPNWGEESEQEWGLLHTEINGKEICRKYPGIAGNYGGFYQNI
YEHLCLGQPLETHAQDILNVIRIIEAAYQSHRENKIVNLK
00000000000000000000000000000000000000000000000000000000000000000000000000000000000
00000000000000000000000000000000000000000000000000000000000000000000000000111111111
11100000000000000000000000000000000000000000000000000000000000000000000000000000000
00000000000000000000000000000000000000000000000000000000000000000000000000000000000
0000000000000000001
>B1XGB5_4fju_0_348
MKISRETLHQLIENKLCQAGLKREHAATVAEVLVYADARGIHSHGAVRVEYYAERISKGGT
NREPEFRLEETGPCSAILHADNAAGQVAAKMGMEHAIKTAQQNGVAVVGISRMGHSGAIS
YFVQQAARAGFIGISMCQSDPMVVPFGGAEIYYGTNPLAFAAPGEGDEILTFDMATTVQA
WGKVLDARSRNMSIPDTWAVDKNGVPTTDPFAVHALLPAAGPKGYGLMMMIDVLSGVLL
GLPFGRQVSSMYDDLHAGRNLGQLHIVINPNFFSSSELFRQHLSQTMRELNAITPAPGFNQ
VYYPGQDQDIKQRKAAVEGIEIVDDIYQYLISDALYNTSYETKNPFAQ
00000000000000000000000000000000000000000000000000000000000000000000000000000000000
00000000000000000000000000000000000000000000000000000000000000000000000000000000000
00000000000000000000000000000000000000000000000000000000000000000000000000000000000
00000000000000000000000000000000000000000000000000000000000000000000000000000000000
00000011111111111
>P96253_3c8n_0_335
MAELKLGYKASAEQFAPRELVELAVAAEAHGMDSATVSDHFQPWRHQGGHAPFSLSWM
TAVGERTNRLLLGTSVLTPTFRYNPAVIAQAFATMGCLYPNRVFLGVGTGEALNEIATGYEG
AWPEFKERFARLRESVGLMRQLWSGDRVDFDGDYYRLKGASIYDVPDGGVPVYIAAGGPA
VAKYAGRAGDGFICTSGKGEELYTEKLMPAVREGAAAADRSVDGIDKMIEIKISYDPDPELA
LNNTRFWAPLSLTAEQKHSIDDPIEMEKAADALPIEQIAKRWIVASDPDEAVEKVGQYVTW

GLNHLVFHAPGHDQRRFLELFQSDLAPRLRRLG
01000000000000000000000000000000000000000000000000000000000000000000000000000000000
00000000000000000000000000000000000000000000000000000000000000000000000000000000000
00000000000000000000000000000000000000000000000000000000000000000000000000000000000
00000000000000000000000000000000000000000000000000000000000000000000000000000000000
0011
>P37146_3n38_0_318
MKLSRISAINWNKISDDKDLEVWNRLTSNFWLPEKVPLSNDIPAWQTLTVVEQQLTMRVFT
GLTLLDTLQNVIGAPSLMPDALTPHEEAVLSNISFMEAVHARSYSSIFSTLCQTKDVDAAYA
WSEENAPLQRKAQIIQQHYRGDDPLKKKIASVFLESFLFYSGFWLPMYFSSRGKLTNTADLI
RLIIRDEAVHGYYIGYKYQKNMEKISLGQREELKSFAFDLLLELYDNELQYTDELYAETPWA
DDVKAFLCYNANKALMNLGYEPLFPAEMAEVNPAILAALSPNADENHDFFSGSGSSYVM
GKAVETEDEDWNF
11111000000000000000000000000000000000000000000000000000000000000000000000000000000
00000000000000000000000000000000000000000000000000000000000000000000000000000000000
00000000000000000000000000000000000000000000000000000000000000000000000000000000000
000000000000000000000000000000000000001111111111111111111111111111111
>P06608_1hfw_21_347
ADKLPNIVILATGGTIAGSAATGTQTTGYKAGALGVDTLINAVPEVKKLANVKGEQFSNM
ASENMTGDVVLKLSQRVNELLARDDVDGVVITHGTDTVEESAYFLHLTVKSDKPVVFVAA
MRPATAISADGPMNLLEAVRVAGDKQSRGRGVMVVLNDRIGSARYITKTNASTLDTFKAN
EEGYLGVIIGNRIYYQNRIDKLHTTRSVFDVRGLTSLPKVDILYGYQDDPEYLYDAAIQHGV
KGIVYAGMGAGSVSVRGIAGMRKAMEKGVVVIRSTRTGNGIVPPDEELPGLVSDSLNPAHA
RILLMLALTRTSDPKVIQEYFHTY
11100000000000000111111111111111110000000000000000000000000000000000000000000000000
00000000000000000000000000000000000000000000000000000000000000000000000000000000000
00000000000000000000000000000000000000000000000000000000000000000000000000000000000
000000000000000000000000000000000000000000000000000000000000000000000000000000
>A8BZ95_3gbz_0_307
MSVSAAPSATSIDRYRRITKLGEGTYGEVYKAIDTVTNETVAIKRIRLEHEEEGVPGTAIREVS
LLKELQHRNIIELKSVIHHNHRLHLIFEYAENDLKKYMDKNPDVSMRVIKSFLYQLINGVNF
CHSRRCLHRDLKPQNLLLSVSDASETPVLKIGDFGLARAFGIPIRQFTHEIITLWYRPPEILLG
SRHYSTSVDIWSIACIWAEMLMKTPLFPGDSEIDQLFKIFEVLGLPDDTTWPGVTALPDWKQ
SFPKFRGKTLKRVLGALLDDEGLDLLTAMLEMDPVKRISAKNALEHPYFSHNDFDP
11111111110000000000000000000000000000000000000000111111100000000000000000000000000
00000000000000000000000000000000000000000000000000000000000000011111000000000000000
01111111111100000000000000000000000000000000000000000000000000000000000000000000000
0000000000000000000000000000000000000000000000000000000000
>P0A5J2_3pkc_0_284
MPSRTALSPGVLSPTRPVPNWIARPEYVGKPAAQEGSEPWVQTPEVIEKMRVAGRIAAGAL
AEAGKAVAPGVTTDELDRIAHEYLVDNGAYPSTLGYKGFPKSCCTSLNEVICHGIPDSTVIT
DGDIVNIDVTAYIGGVHGDTNATFPAGDVADEHRLLVDRTREATMRAINTVKPGRALSVIG
RVIESYANRFGYNVVRDFTGHGIGTTFHNGLVVLHYDQPAVETIMQPGMTFTIEPMINLGA
LDYEIWDDGWTVVTKDRKWTAQFEHTLLVTDTGVEILTCL
11100000000000000000000000000000000000000000000000000000000000000000000000000000000
00000000000000000000000000000000000000000000000000000000000000000000000000000000000
00000000000000000000000000000000000000000000000000000000000000000000000000000000000
00000000000000000000000000000000001
>D0VWS9_3c1a_0_314
GMSIPANNSPVRLALIGAGRWGKNYIRTIAGLPGAALVRLASSNPDNLALVPPGCVIESDW
RSVVSAPEVEAVIIATPPATHAEITLAAIASGKAVLVEKPLTLDLAEAEAVAAAAKATGVMV

WVEHTQLFNPAWEALKADLTSIGPILAVRSEAGNHGPYRPGGVPMLWDWGAHDVSMVL
DLMGRDPDSTSASWAARGEKDGGEAGDVTLTLAFSTVEAHIRLCNTMDKCRRLAVFGEA
GTLVMDDRATDKLTLHPPQPDGNWPVGQGHALTVTDEMPLTRAVRLFAGAVRQPEPGPS
PLELGLRVVRVLGACS
11111111000000000000000000000000000000000000000000000000000000000000000000000000000
00000000000000000000000000000000000000000000000000000000000000000000000000000000000
00000000000000000000000000000000000000000000000000000000000000000000000000000000000
000000000000000000000000000000000000000000000000000000000000000000
>P53228_3cq0_0_332
MSEPSEKKQKVATSSLEQLKKAGTHVVADSGDFEAISKYEPQDSTTNPSLILAASKLEKYARF
IDAAVEYGRKHGKTDHEKIENAMDKILVEFGTQILKVVPGRVSTEVDARLSFDKKATVKKA
LHIIKLYKDAGVPKERVLIKIASTWEGIQAARELEVKHGIHCNMTLLFSFTQAVACAEANVT
LISPFVGRIMDFYKALSGKDYTAETDPGVLSVKKIYSYYKRHGYATEVMAASFRNLDELKAL
AGIDNMTLPLNLLEQLYESTDPIENKLNSESAKEEGVEKVSFINDEPHFRYVLNEDQMATEK
LSDGIRKFSADIEALYKLVEEKM
11111111111000000000000000000000000000000000000000000000000000000000000000000000000
00000000000000000000000000000000000000000000000000000000000000000000000000000000000
00000000000000000000000000000000000111000000000000000000000000000000000000000000000
00000000000000000000000000000000000000000000000000000000000000000000000000000000000
0
>Q8NTZ3_3pl2_21_338
MTNLTSTHEVLAIGRLGVDIYPLQSGVGLADVQSFGKYLGGSAANVSVAAARHGHNSALL
SRVGNDPFGEYLLAELERLGVDNQYVATDQTFKTPVTFCEIFPPDDFPLYFYREPKAPDLNIE
SADVSLDDVREADILWFTLTGFSEEPSRGTHREILTTRANRRHTIFDLDYRPMFWESPEEATK
QAEWALQHSTVAVGNKEECEIAVGETEPERAGRALLERGVELAIVKQGPKGVMAMTKDET
VEVPPFFVDVINGLGAGDAFGGALCHGLLSEWPLEKVLRFANTAGALVASRLECSTAMPTT
DEVEASLNQKV
11111000000000000000000000000000000000000000000000000000000000000000000000000000000
00000000000000000000000000000000000000000000000000000000000000000000000000000000000
00000000000000000000000000000000000000000000000000000000000000000000000000000000000
000000000000000000000000000000000000000000000000000000000000000000111
>Q837V6_1ta8_0_324
MEQQPLTLTAATTRAQELRKQLNQYSHEYYVKDQPSVEDYVYDRLYKELVDIETEFPDLITP
DSPTQRVGGKVLSGFEKAPHDIPMYSLNDGFSKEDIFAFDERVRKAIGKPVAYCCELKIDGL
AISLRYENGVFVRGATRGDGTVGENITENLRTVRSVPMRLTEPISVEVRGECYMPKQSFVAL
NEEREENGQDIFANPRNAAAGSLRQLDTKIVAKRNLNTFLYTVADFGPMKAKTQFEALEEL
SAIGFRTNPERQLCQSIDEVWAYIEEYHEKRSTLPYEIDGIVIKVNEFALQDELGFTVKAPRW
AIAYKFPPEEAETVV
11110000000000000000000000000000000000000000000000000000000000000000000000000000000
00000000000000000000000000000000000000000000000000000000000000000000000000000000000
00000000000000000000000000000000000000000000000000000000000000000000000000000000000
0000000000000000000000000000000000000000000000000000000000000000000011111111
>Q9HJT3_3bf5_0_286
MRFLAYFGHLNIDVLISVDSIPREGSVNVKDLRPRFGGTAGNFAIVAQKFRIPFDLYSAVGMK
THREYLAMIESMGINTGHVEKFEDESGPICYIATDGKKQVSFMHQGAMEKWKPQLADEYE
YVHFSTGPNYLDMAKSIRSKIIFDPSQEIHKYSKDELKKFHEISYMSIFNDHEYRVFREMTGLS
SPKVTTIVTNGERGSSLFMDGKKYDFPAIPSSGDTVGAGDSFRAGLYLALYNRRSIEKGMIYG
TIIAHHVIDDGIENFSLNMEDLERETENYRRMFTKRS
00000000000000000000000000000000000000000000000000000000000000000000000000000000000
00000000000000000000000000000000000000000000000000000000000000000000000000000000000
00000000000000000000000000000000000000000000000000000000000000000000000000000000000

00000000000000000000000000000000001111
>Q8EF24_3o5c_22_332
AEPIEVITPAKITEPEKVELGKMLFFEPRLSKSGFISCNSCHNLSTGGVDALPTSIGHHWQEG
PINSPTVLNADFMLAQFWDGRASNLKEQAAGPIANPKEMGFTHELATETIASMPAYRARF
AKVYGDEKVDIDRLTDAIAAFEKTLVTPNSPFDQYLLGKQDAISGDAKAGYQLFKDKGCVS
CHNGPAVGGTMFMKMGLIKPFHTNNPAEGRKGVTGKDADKFVFKVPTLRNIELTYPYFH
DGSVWTLEEAVNTMADIQLGQKLTEKETKEMVAFLNSLTGEQPQISLPILPPSNKETPRPVP
FATGAK
10000000000000000000000000000000000000000000000000000000000000000000000000000000000
00000000000000000000000000000000000000000000000000000000000000000000000000000000000
00000000000000000000000000000000000000000000000000000000000000000000000000000000000
0000000000000000000000000000000000000000000000000000000000111
>P00433_1w4w_30_352
QLTPTFYDNSCPNVSNIVRDTIVNELRSDPRIAASILRLHFHDCFVNGCDASILLDNTTSFRT
EKDAFGNANSARGFPVIDRMKAAVESACPRTVSCADLLTIAAQQSVTLAGGPSWRVPLGR
RDSLQAFLDLANANLPAPFFTLPQLKDSFRNVGLNRSSDLVALSGGHTFGKNQCRFIMDRL
YNFSNTGLPDPTLNTTYLQTLRGLCPLNGNLSALVDFDLRTPTIFDNKYYVNLEEQKGLIQS
DQELFSSPNATDTIPLVRSFANSTQTFFNAFVEAMDRMGNITPLTGTQGQIRLNCRVVNSNS
LLHDMVEVVDFVSSM
00000000000000000000000000000000000000000000000000000000000000000000000000000000000
00000000000000000000000000000000000000000000000000000000000000000000000000000000000
00000000000000000000000000000000000000000000000000000000000000000000000000000000000
0000000000000000000000000000000000000000000000000000000011111111111111111
>P94522_1uv4_30_322
EAAFWGASNELLHDPTMIKEGSSWYALGTGLTEERGLRVLKSSDAKNWTVQKSIFTTPLSW
WSNYVPNYGQNQWAPDIQYYNGKYWLYYSVSSFGSNTSAIGLASSTSISSGGWKDEGLVIRS
TSSNNYNAIDPELTFDKDGNPWLAFGSFWSGIKLTKLDKSTMKPTGSLYSIAARPNNGGAL
EAPTLTYQNGYYYLMVSFDKCCDGVNSTYKIAYGRSKSITGPYLDKSGKSMLEGGGTILDSG
NDQWKGPGGQDIVNGNILVRHAYDANDNGIPKLLINDLNWSSGWPSY
11000000000000000000000000000000000000000000000000000000000000000000000000000000000
00000000000000000000000000000000000000000000000000000000000000000000000000000000000
00000000000000000000000000000000000000000000000000000000000000000000000000000000000
0000000000000000000000000000000000000000000
>A6KZZ2_3gux_21_333
GNSKSRNDRTETVDKEVIKAPEFDADSAYQYIQVQADFGPRVPNTQAHKECGEYLAGQLE
KFGAKVYNQYADLIAYDGTILKSRNIIGAYKPESKKRILLCAHWDSRPYADNDPDPKNHHT
PILGVNDGASGVGVLLEIARQIQKEQPALGIDIVFFDSEDYGIPEFYDGKYKQDTWCLGSQY
WARTPHVQNYNARYGILLDMVGGKDATFYYEGYSARTARSEMKKIWKKAHELGYGKYFV
KEDGGETVDDHIYVNKLARIPCVDIINYDAGNPQSSFGSFWHTVNDTMENIDRNTLKAVG
QTVMDVIYNEK
11111111111111100000000000000000000000000000000000000000000000000000000000000000000
00000000000000000000000001111111111111110000000000000000000000000000000000000000111
11111110000000000000000000000000000000000000000000000000000000000000000000000000000
000000000000000000000011111111111111100000000000000000000000000
>A1JJJ9_3nzk_0_310
MRDKTMIKQRTLKRIVQATGVGLHTGKKVTLTMRPAPANTGVIYRRTDLNPPVDFPADAK
SVRDTMLCTCLVNEHDVRISTVEHLNAALAGLGIDNIIIEVNAPEVPIMDGSASPFVYLLLD
AGIEELNSAKKFLRLKETVRVEDGDKWAELSPFNGFRLDFTIDFNHPAIDSSTQRYRLDFSA
DSFVRQISRARTFGFMRDIEYLQSRGLCLGGSFDCAIVVDDYRVLNEDGLRFEDEFVRHKML
DAIGDLFMCGHNIIGAFTAYKSGHALNNKLLQAVLAKQEAWEFVTFQDEAEMPLAFKAPS
TVLAY

11111000000000000000000000000000000000000000000000000000000000000000000000000000000
00000000000000000000000000000000000000000000000000000000000000000000000000000000000
00000000000000000000000000000000000000000000000000000000000000000000000000000000000
00000000000000000000000000000000000000000000000000000000111111
>Q1QU06_4jci_0_310
MKRVHVIDSHTAGEPTRLVMEGMPALSGRTIAEKCDDFRDNHDAWRRAIMLEPRGHDVL
VGALYCAPESSDASCGVIFFNNSGYLGMCGHGTIGLVASLHHLGQLTPGCHKIDTPAGPVS
ATLHDDGAVTVRNVLSYRHRRRVPVEVPGYGTVHGDIAWGGNWFFLVSDHDMTLELDN
VEALTDYTWAIRQALEAQSITGENGGVIDHIELFCDDREADSRNFVLCPGKAYDRSPCGTGT
SAKLACLAADGKLAPGQVWTQASICGSRFEAFYEREGDGIRPSIKGRAYLSADATLLIDERD
PFAWGIASP
00000000000000000000000000000000000000000000000000000000000000000000000000000000000
00000000000000000000000000000000000000000000000000000000000000000000000000000000000
00000000000000000000000000000000000000000000000000000000000000000000000000000000000
00000000000000000000000000000000000000000000000000000000000011
>Q5SJI0_1uar_0_284
MGYAHPEVLVSTDWVQEHLEDPKVRVLEVDEDILLYDTGHIPGAQKIDWQRDFWDPVVR
DFISEEEFAKLMERLGISNDTTVVLYGDKNNWWAAYAFWFFKYNGHKDVRLMNGGRQK
WVEEGRPLTTEVPSYPPGRYEVPYRDESIRAYRDDVLEHIIKVKEGKGALVDVRSPQEYRGEL
THMPDYPQEGALRAGHIPGAKNIPWAKAVNPDGTFKSAEELRALYEPLGITKDKDIVVYCR
IAERSSHSWFVLKYLLGYPHVKNYDGSWTEWGNLVGVPIAKGEE
10000000000000000000000000000000000000000000000000000000000000000000000000000000000
00000000000000000000000000000000000000000000000000000000000000000000000000000000000
00000000000000111111110000000000000000000000000000000000000000000000000000000000000
000000000000000000000000000000000000
>Q8IXQ5_3ii7_282_585
GTRPRRKKHDYRIALFGGSQPQSCRYFNPKDYSWTDIRCPFEKRRDAACVFWDNVVYILG
GSQLFPIKRMDCYNVVKDSWYSKLGPPTPRDSLAACAAEGKIYTSGGSEVGNSALYLFECY
DTRTESWHTKPSMLTQRCSHGMVEANGLIYVCGGSLGNNVSGRVLNSCEVYDPATETWTE
LCPMIEARKNHGLVFVKDKIFAVGGQNGLGGLDNVEYYDIKLNEWKMVSPMPWKGVTV
KCAAVGSIVYVLAGFQGVGRLGHILEYNTETDKWVANSKVRAFPVTSCLICVVDTCGANE
ETLET
11111110000000000000000000000000000000000000000000000000000000000000000000000000000
00000000000000000000000000000000000000000000000000000000000000000000000000000000000
00000000000000000000000000000000000000000000000000000000000000000000000000000000000
0000000000000000000000000000000000000000000000111111111
>Q186X0_4dun_0_259
MEYYIVDSFATKLFKGNPAGVCVLDRRIPLELMQKIAEENNLPETAFVVKGKGNYELRWFT
PKAEIDLCGHATLAAAYVISNFIDVNVKKIDFFTQSGKLEVTRNGNLYEMIFPEIMPIEIELSP
QQANLIGCVPSDVYSSRDLILLLNSEQEVINYKPNYAQLRKLTDWLGIIITAQGSNTDFVSRY
FCPELDSEDPVTGSSHCNLIPYWSEKLGKHKMVAAQLSNRGGIIQCEVLKDNTVKISGEAVL
FMQGTIKIDI
00000000000000000000000000000000000000000000000000000000000000000000000000000000000
00000000000000000000000000000000000000000000000000000000000000000000000000000000000
00000000000000000000000000000000000000000000000000000000000000000000000000000000000
00000000011
>Q5SLV2_2dqw_0_293
MGAPPLGPTIIPPVRTLWLRDRALDLDRVRLLGVLNLTPDSFSDGGRYLDPERALERAREM
VAEGADILDLGAESTRPGAAPVPVEEEKRRLLPVLEAVLSLGVPVSVDTRKPEVAEEALKLG
AHLLNDVTGLRDERMVALAARHGVAAVVMHMPVPDPATMMAHARYRDVVAEVKAFLE
AQARRALSAGVPQVVLDPGFGFGKLLEHNLALLRRLDEIVALGHPVLVGLSRKRTIGELSG

VEDPAQRVHGSVAAHLFAVMKGVRLLRVHDVRAHREALGVWEALYGGDRPSRA
11111111111110000000000000000000000000011111111111000000000000000000000000011111111
00000000000000000000000000000000000000000000000000000000000000000000000000000000000
00000000000000000000000000000000000000000000000000000000000000000000000000000000000
000000000000000000000000000000000000001111111
>P37744_1h5t_0_292
MKMRKGIILAGGSGTRLYPVTMAVSKQLLPIYDKPMIYYPLSTLMLAGIRDILIISTPQDTPRF
QQLLGDGSQWGLNLQYKVQPSPDGLAQAFIIGEEFIGGDDCALVLGDNIFYGHDLPKLME
AAVNKESGATVFAYHVNDPERYGVVEFDKNGTAISLEEKPLEPKSNYAVTGLYFYDNDVVQ
MAKNLKPSARGELEITDINRIYLEQGRLSVAMMGRGYAWLDTGTHQSLIEASNFIATIEERQ
GLKVSCPEEIAFRKGFIDVEQVRKLAVPLIKNNYGQYLYKMTKDSN
10000000000000000000000000000000000000000000000000000000000000000000000000000000000
00000000000000000000000000000000000000000000000000000000000000000000000000000000000
00000000000000000000000000000000000000000000000000000000000000000000000000000000000
00000000000000000000000000000000000000000011
>O86309_1gx3_0_274
MAMDLGGYLTRIGLDGRPRPDLGTLHAIVAAHNRSIPFENLDPLLGIPVADLSAEALFAKLV
DRRRGGYCYEHNGLLGYVLEELGFEVERLSGRVVWMRADDAPLPAQTHNVLSVAVPGAD
GRYLVDVGFGGQTLTSPIRLEAGPVQQTRHEPYRLTRHGDDHTLAAQVRGEWQPLYTFTTE
PRPRIDLEVGSWYVSTHPGSHFVTGLTVAVVTDDARYNLRGRNLAVHRSGATEHIRFDSAA
QVLDAIVNRFGIDLGDLAGRDVQARVAEVLDT
10000000000000000000000000000000000000000000000000000000000000000000000000000000000
00000000000000000000000000000000000000000000000000000000000000000000000000000000000
00000000000000000000000000000000000000000000000000000000000000000000000000000000000
00000000000000000000000000
>D0VWZ4_3i6y_0_279
GMSIENLSSNKSFGGWHKQYSHVSNTLNCAMRFAIYLPPQASTGAKVPVLYWLSGLTCSDE
NFMQKAGAQRLAAELGIAIVAPDTSPRGEGVADDEGYDLGQGAGFYVNATQAPWNRHY
QMYDYVVNELPELIESMFPVSDKRAIAGHSMGGHGALTIALRNPERYQSVSAFSPINNPVN
CPWGQKAFTAYLGKDTDTWREYDASLLMRAAKQYVPALVDQGEADNFLAEQLKPEVLEA
AASSNNYPLELRSHEGYDHSYYFIASFIEDHLRFHSNYLNA
11000000000000000000000000000000000000000000000000000000000000000000000000000000000
00000000000000000000000000000000000000000000000000000000000000000000000000000000000
00000000000000000000000000000000000000000000000000000000000000000000000000000000000
0000000000000000000000000000000
>Q9KLD9_1twy_1_277
IRMALAAVCALLFSITTMTPFVQASEITISGSTSVARIMDVLAEKYNQQHPETYVAVQGVGST
AGISLLKKGVADIAMTSRYLTESEAQNTLHTFTLAFDGLAIVVNQANPVTNLTREQLYGIYK
GQITNWKQVGGNDQKIAVVTREASSGTRYSFESLMGLTKTVKDREVSDVAPTALVVNSNS
MMKTLVNHNTQAVGFISIGSVDKSVKAIQFEKADPTSDNIAKHTYQLSRPFLILHYSDNADE
QTKEFIAFLKSESAKKLIVEYGYIMPSDVE
11111111111111111111111100000000000000000000000000000000000000000000000000000000000
00000000000000000000000000000000000000000000000000000000000000000000000000000000000
00000000000000000000000000000000000000000000000000000000000000000000000000000000000
0000000000000000000000001111
>G0L322_1o4y_19_294
AQDWNGIPVPANPGNGMTWQLQDNVSDSFNYTSSEGNRPTAFTSKWKPSYINGWTGPGS
TIFNAPQAWTNGSQLAIQAQPAGNGKSYNGIITSKNKIQYPVYMEIKAKIMDQVLANAFW
TLTDDETQEIDIMEGYGSDRGGTWFAQRMHLSHHTFIRNPFTDYQPMGDATWYYNGGTP
WRSAYHRYGCYWKDPFTLEYYIDGVKVRTVTRAEIDPNNHLGGTGLNQATNIIIDCENQT
DWRPAATQEELADDSKNIFWVDWIRVYKPVAVSGGGNN

00000000000000000000000000000000000000000000000000000000000000000000000000000000000
00000000000000000000000000000000000000000000000000000000000000000000000000000000000
00000000000000000000000000000000000000000000000000000000000000000000000000000000000
000000000000000000000111111
>D0VX01_3eld_0_274
MLGKQEFERYKVSDITEVDRTAARRYLKEGRTDVGISVSRGAAKIRWLHERGYLRITGRVLD
LGCGRGGWSYYAAAQKEVMSVKGYTLGIEGHEKPIHMQTLGWNIVKFKDKSNVFTMPTE
PSDTLLCDIGESSSNPLVERDRTMKVLENFERWKHVNTENFCVKVLAPYHPDVIEKLERLQ
LRFGGGIVRVPFSRNSTHEMYYISGARNNITHMVNTTSRSLLRRMTRPSGKAIIEGDVFLPTG
TRSVASEAGTIDHEALKLRVDQIKAEYSKT
00000000000000000000000000000000000000000000000000000000000000000000000000000000000
00000000000000000000000000000000000000000000000000000000000000000000000000000000000
00000000000000000000000000000000000000000000000000000000000000000000000000000000000
11100000000000000001111111
>P18843_1wxi_0_274
MTLQQQIIKALGAKPQINAEEEIRRSVDFLKSYLQTYPFIKSLVLGISGGQDSTLAGKLCQMA
INELRLETGNESLQFIAVRLPYGVQADEQDCQDAIAFIQPDRVLTVNIKGAVLASEQALREA
GIELSDFVRGNEKARERMKAQYSIAGMTSGVVVGTDHAAEAITGFFTKYGDGGTDINPLYR
LNKRQGKQLLAALACPEHLYKKAPTADLEDDRPSLPDEVALGVTYDNIDDYLEGKNVPQQ
VARTIENWYLKTEHKRRPPITVFDDFWKK
10000000000000000000000000000000000000000000000000000000000000000000000000000000000
00000000000000000000000000000000000000000000000000000000000000000000000000000000000
00000000000000000000000000000000000000000001111111111111000000000000000000000000000
00000000000000000000000000
>A1A3G9_3cpg_1_271
TAYMDHKNLANEVIDDARAREITDGVHRVLDRIAAAEEQAGREAGSVRLLAATKTRDIGEI
MAAIDAGVRMIGENRPQEVTAKAEGLARRCAERGFSLGVAGAAPDAAAEHIPFHLIGQLQ
SNKIGKVLPVVDTIESVDSIDLAEKISRRAVARGITVGVLLEVNESGEESKSGCDPAHAIRIAQ
KIGTLDGIELQGLMTIGAHVHDETVIRRGFSHLRKTRDLILASGEPGTDRCRELSMGMTGD
MELAIAEGSTIVRVGTAIFGERAFI
11111100000000000000000000000000000000000000000000000000000000000000000000000000000
00000000000000000111111000000000000000000000000000000000000000000000000000000000000
00000000000000000000000000000000000000000000000000000000000000000000000000000000000
0000000000000000111111
>A6LFX0_3h3l_30_269
AGTQTAQTQALDSDGIPTGGEWITMFDGKTLNGWRGYCRQDVPLGWVVEDGSITYKGSD
NKADTGFGDLIYDKKFKNFVFEIEWKIDKAGNSGIFYTAQEIEGTPIYYSSPEYQLLDNENM
PDAWEGCDGNRQAGAVYDMIMPDPQPVKPYGNWNKTRIVVYNQRVIHYMNDVKILEFQ
FGTPVWRALVDHSKFSKFSTSPEKCPEAYDLMLQCGKQPGYIGMQDHGYGVCFRNIRIKEL
11111111100000000000000000000000000000000000000000000111110000000000000000
00000000000000000000000000000000000000000000000000000000000000000000000000000000000
00000000000000000000000000000000000000000000000000000000000000000000000000
>Q13KT2_2xua_0_262
MPYAAVNGTELHYRIDGERHGNAPWIVLSNSLGTDLSMWAPQVAALSKHFRVLRYDTRG
HGHSEAPKGPYTIEQLTGDVLGLMDTLKIARANFCGLSMGGLTGVALAARHADRIERVAL
CNTAARIGSPEVWVPRAVKARTEGMHALADAVLPRWFTADYMEREPVVLAMIRDVFVHT
DKEGYASNCEAIDAADLRPEAPGIKVPALVISGTHDLAATPAQGRELAQAIAGARYVELDA
SHISNIERADAFTKTVVDFLTEQK
00000000000000000000000000000000000000000000000000000000000000000000000000000000000
00000000000000000000000000000000000000000000000000000000000000000000000000000000000
00000000000000000000000000000000000000000000000000000000000000000000000000000000000

00000000000011
>Q56008_1jfr_48_309
AANPYERGPAPTNASIEASRGPYATSQTSVSSLVASGFGGGTIYYPTSTADGTFGAVVISPGFT
AYQSSIAWLGPRLASQGFVVFTIDTNTTLDQPDSRGRQLLSALDYLTQRSSVRTRVDATRLG
VMGHSMGGGGSLEAAKSRTSLKAAIPLTGWNTDKTWPELRTPTLVVGADGDTVAPVATH
SKPFYESLPGSLDKAYLELRGASHFTPNTSDTTIAKYSISWLKRFIDSDTRYEQFLCPIPRPSLTI
AEYRGTCPHTS
11000000000000000000000000000000000000000000000000000000000000000000000000000000000
00000000000000000000000000000000000000000000000000000000000000000000000000000000000
00000000000000000000000000000000000000000000000000000000000000000000000000000000000
0000000000000
>P82597_3rli_0_249
MSEQYPVLSGAEPFYAENGPVGVLLVHGFTGTPHSMRPLAEAYAKAGYTVCLPRLKGHGT
HYEDMERTTFHDWVASVEEGYGWLKQRCQTIFVTGLSMGGTLTLYLAEHHPDICGIVPINA
AVDIPAIAAGMTGGGELPRYLDSIGSDLKNPDVKELAYEKTPTASLLQLARLMAQTKAKLD
RIVCPALIFVSDEDHVVPPGNADIIFQGISSTEKEIVRLRNSYHVATLDYDQPMIIERSLEFFAK
HAG
00000000000000000000000000000000000000000000000000000000000000000000000000000000000
00000000000000000000000000000000000000000000000011111100000000000000000000000000000
00000000000000000000000000000000000000000000000000000000000000000000000000000000000
1
>Q63XU7_3gw8_0_248
MYKLVLIRHGESTWNKENRFTGWVDVDLTEQGNREARQAGQLLKEAGYTFDIAYTSVLKR
AIRTLWHVQDQMDLMYVPVVHSWRLNERHYGALSGLNKAETAAKYGDEQVLVWRRSYD
TPPPALEPGDERAPYADPRYAKVPREQLPLTECLKDTVARVLPLWNESIAPAVKAGKQVLIA
AHGNSLRALIKYLDGISDADIVGLNIPNGVPLVYELDESLTPIRHYYLGDQEAIAKAQAAVA
QQGKSAA
00000000000000000000000000000000000000000000000000000000000000000000000000000000000
00000000000000000000000000000000000000000000000000000000000000000000000000000000000
00000000000000000000000000000000000000000000000000000000000000001111111111111111111
>P31947_3lw1_0_247
MERASLIQKAKLAEQAERYEDMAAFMKGAVEKGEELSCEERNLLSVAYKNVVGGQRAAW
RVLSSIEQKSNEEGSEEKGPEVREYREKVETELQGVCDTVLGLLDSHLIKEAGDAESRVFYLK
MKGDYYRYLAEVATGDDKKRIIDSARSAYQEAMDISKKEMPPTNPIRLGLALNFSVFHYEIA
NSPEEAISLAKTTFDEAMADLHTLSEDSYKDSTLIMQLLRDNLTLWTADNAGEEGGEAPQE
PQS
00000000000000000000000000000000000000000000000000000000000000000000000000000000000
00000000000000000000000000000000000000000000000000000010000000000000000000000000000
0000000000000000000000000000000000000000000000000000000000000000011111111111111111
>P24468_3cjw_174_413
HSYLSGYISLLLRAEPYPTSRFGSQCMQPNNIMGIENICELAARMLFSAVEWARNIPFFPDLQ
ITDQVALLRLTWSELFVLNAAQCSMPLHVAPLLAAAGLHASPMSADRVVAFMDHIRIFQE
QVEKLKALHVDSAEYSCLKAIVLFTSDACGLSDVAHVESLQEKSQCALEEYVRSQYPNQPT
RFGKLLLRLPSLRTVSSSVIEQLFFVRLVGKTPIETLIRDMLLSGSSFNWPYMAIQ
00000000000000000001111111111111000000000000000000000000000000000000000000000000000
00000000000111111111111111100000000000000000000000000000000000000000000000000000000
00000000000000000000000000000000000000000000000000000000000000000001111111
>O00499_2fic_0_271
MAEMGSKGVTAGKIASNVQKKLTRAQEKVLQKLGKADETKDEQFEQCVQNFNKQLTEGT
RLQKDLRTYLASVKAMHEASKKLNECLQEVYEPDWPGRDEANKIAENNDLLWMDYHQK
LVDQALLTMDTYLGQFPDIKSRIAKRGRKLVDYDSARHHYESLQTAKKKDEAKIAKAEEELI

KAQKVFEEMNVDLQEELPSLWNSRVGFYVNTFQSIAGLEENFHKEMSKLNQNLNDVLVGL
EK
1111111111111111111111111111111111111111100000000000000000000000000000000000000000000000000000
000000000000000000000000000000000000000000000000000000000000000000000000000000000000000000000
00000000000000000000000000000000000000000000000000000000000000000000000000000000000001
>Q46893_1inj_0_235
MATTHLDVCAVVPAAGFGRRMQTECPKQYLSIGNQTILEHSVHALLAHPRVKRVVIAISPG
DSRFAQLPLANHPQITVVDGGDERADSVLAGLKAAGDAQWVLVHDAARPCLHQDDLAR
LLALSETSRTGGILAAPVRDTMKRAEPGKNAIAHTVDRNGLWHALTPQFFPRELLHDCLTR
ALNEGATITDEASALEYCGFHPQLVEGRADNIKVTRPEDLALAEFYLTRTIHQENT
111100000000000111111111110000000000000000000000000000000000000000000000000000000000000000000
000000000000000000000000000000000000000000000000000000000000000000000000000000000000000000000
000000000000000000000000000000000000000000000000000000000000000000000011111111
>Q8PHA1_2h5y_24_257
QTAPVTVFAAASLKESMDEAATAYEKATGTPVRVSYAASSALARQIEQGAPADVFFSADLE
WMDYLQQHGLVLPAQRHNLLGNTLVLVAPASSKLRVDPRAPGAIAKALGENGRLAVGQT
ASVPAGKYAAAALRKLGQWDSVSNRLAESESVRAALMLVSRGEAPLGIVYGSDARADAKV
RVVATFPDDSHDAIVYPVAALKNSNNPATAAFVSWLGSKPAKAIFARRGFSLKD
100000000000000000000000000000000000000000000000000000000000000000000000000000000000000000000
000000000000000000000000000000000000000000000000000000000000000000000000000000000000000000000
0000000000000000000000000000000000000000000000000000000000000000000000000001
>P0A5Z6_1ys6_0_232
MDTGVTSPRVLVVDDDSDVLASLERGLRLSGFEVATAVDGAEALRSATENRPDAIVLDINM
PVLDGVSVVTALRAMDNDVPVCVLSARSSVDDRVAGLEAGADDYLVKPFVLAELVARVKA
LLRRRGSTATSSSETITVGPLEVDIPGRRARVNGVDVDLTKREFDLLAVLAEHKTAVLSRAQL
LELVWGYDFAADTNVVDVFIGYLRRKLEAGGGPRLLHTVRGVGFVLRMQ
111111000000000000000000000000000000000000000000000000000000000000000000000000000000000000000
000000000000000000000000000000000000000000000000000000000000000000000000000000000000000000000
000000000000000000000000000000000000000000000000000000000000000000000000000
>P22087_2ipx_82_314
GKNVMVEPHRHEGVFICRGKEDALVTKNLVPGESVYGEKRVSISEGDDKIEYRAWNPFRSK
LAAAILGGVDQIHIKPGAKVLYLGAASGTTVSHVSDIVGPDGLVYAVEFSHRSGRDLINLAK
KRTNIIPVIEDARHPHKYRMLIAMVDVIFADVAQPDQTRIVALNAHTFLRNGGHFVISIKAN
CIDSTASAEAVFASEVKKMQQENMKPQEQLTLEPYERDHAVVVGVYRP
111100000000000000111110000000000000000001111000000000000000000000000000000000000000000000000
000000000000000000000000000000000000000000000000000000000000000000000000000000000000000000000
000000000000000000000000000000000000000000000000000000000000000000000000000
>Q8TG90_1k4p_0_232
MPSTDSIPKSNFDAIPDVIQAFKNGEFVVVLDDPSRENEADLIIAAESVTTEQMAFMVRHSS
GLICAPLTPERTTALDLPQMVTHNADPRGTAYTVSVDAEHPSTTTGISAHDRALACRMLAA
PDAQPSHFRRPGHVFPLRAVAGGVRARRGHTEAGVELCRLAGKRPVAVISEIVDDGQEVEG
RAVRAAPGMLRGDECVAFARRWGLKVCTIEDMIAHVEKTEGKLETNGSG
111111111110000000000000000000000000000000000000000000000000000000000000000000000000000000000
000000000000000000000000000000000000000000000000000000000000000000000000000000000000000000000
000000000000000000000000000000000000000000000000000000000000000000000111111
>Q53464_1zrn_0_231
MDYIKGIAFDLYGTLFDVHSVVGRCDEAFPGRGREISALWRQKQLEYTWLRSLMNRYVNF
QQATEDALRFTCRHLGLDLDARTRSTLCDAYLRLAPFSEVPDSLRELKRRGLKLAILSNGSP
QSIDAVVSHAGLRDGFDHLLSVDPVQVYKPDNRVYELAEQALGLDRSAILFVSSNAWDAT
GARYFGFPTCWINRTGNVFEEMGQTPDWEVTSLRAVVELFETAAGKAEKG
110000000000000000000000000000000000000000000000000000000000000000000000000000000000000000000

00000000000000000000000000000000000000000000000000000000000000000000000000000000000
000000000000000000000000000000000000000000000000000000001111111111
>P74061_1tqj_0_229
MSKNIVVAPSILSADFSRLGEEIKAVDEAGADWIHVDVMDGRFVPNITIGPLIVDAIRPLTKK
TLDVHLMIVEPEKYVEDFAKAGADIISVHVEHNASPHLHRTLCQIRELGKKAGAVLNPSTP
LDFLEYVLPVCDLILIMSVNPGFGGQSFIPEVLPKIRALRQMCDERGLDPWIEVDGGLKPNN
TWQVLEAGANAIVAGSAVFNAPNYAEAIAGVRNSKRPEPQLATV
11000000000000000000000000000000000000000000000000000000000000000000000000000000000
00000000000000000000000000000000000000000000000000000000000001111100000000000000000
0000000000000000000000000000000000000000000000000000000001111111
>G6TGY9_4emv_0_225
MSKKEININNYNDDAIQVLEGLDAVRKRPGMYIGSTDGAGLHHLVWEIVDNAVDEALSGF
GDRIDVTINKDGSLTVQDHGRGMPTGMHAMGIPTVEVIFTILHAGGKFGQGGYKTSGGLH
GVGSSVVNALSSWLEVEITRDGAVYKQRFENGGKPVTTLKKIGTALKSKTGTKVTFMPDATI
FSTTDFKYNTISERLNESAFLLKNVTLSLTDKRTDEAIEFHYEN
11111111111111110000000000000000000000000000000000000000000000000000000000000000000
00000000000000000000111111111111100000000000000000000000000000000000000000000000000
000000000000000000000000000000000000000000000000000000000000
>Q0IFG2_4fd4_0_216
MVAPESIVLRVARLDELEQVREILHRIYYPEEGITISYVHGKSHTLDDERFSLSFVEQGTVVVA
EDSAAKKFIGVSIAGPIQPGDPDAMVEEAATTETKKWGDILKLLALLERTADVCGRYGLEK
AYHVHILAVDPTYRGHSLGQRLLQFQMDLSKKLGFKAISGDFTSVFSVKLAEKLGMECISQL
ALGDYRDEKGEKLFEPLDVHQVIKTCVKLL
11111000000000000000000000000000000000000000000000000000000000000000000000000000000
00000000000000000000000000000000000000000000000000000000000000000000000000000000000
000000000000000000000000000000000000000000000000000
>Q9KDE1_2gpy_1_222
IEERLKHYLEKQIPARDQYIEQMEREAHEQQVPIMDLLGMESLLHLLKMAAPARILEIGTAI
GYSAIRMAQALPEATIVSIERDERRYEEAHKHVKALGLESRIELLFGDALQLGEKLELYPLFD
VLFIDAAKGQYRRFFDMYSPMVRPGGLILSDNVLFRGLVAETDIEHKRHKQLATKIDTYNQ
WLLEHPQYDTRIFPVGDGIAISIKRETKGDTDDEKA
10000000000000000000000000000000000000000000000000000000000000000000000000000000000
00000000000000000000000000000000000000000000000000000000000000000000000000000111111
11111111111111110000000000000000000000000000001111111111
>D2YW47_3vcr_0_216
GMTQLDTWLANTKPLIPVIVIDDLVHAIPMAKALVAGGVHLLEVTLRTEAGLAAISAIKKA
VPEAIVGAGTVCTADDFQKAIDAGAQFIVSPGLTPELIEKAKQVKLDGQWQGVFLPGVATA
SEVMIAAQAGITQLKCFPASAIGGAKLLKAWSGPFPDIQFCPTGGISKDNYKEYLGLPNVIC
AGGSWLTESKLLIEGDWNEVTRRASEIVKLSDI
10000000000000000000000000000000000000000000000000000000000000000000000000000000000
00000000000000000000000000000000000000000000000000000000000000000000000000000000000
000000000000000000000000000000000000000000000000000
>P0C6D3_3tb4_0_214
MAIPKIASYPLPVSLPTNKVDWRIDASRAVLLIHDMQEYFVHYFDSQAEPIPSLIKHIQQLKA
HAKQAGIPVVYTAQPANQDPAERALLSDFWGPGLSEETAIIAPLAPESGDVQLTKWRYSAF
KKSPLLDWLRETGRDQLIITGVYAHIGILSTALDAFMFDIQPFVIGDGVADFSLSDHEFSLRYI
SGRTGAVKSTQQACLEIAAQHSKLTGL
11000000000000000000000000000000000000000000000000000000000000000000000000000000000
00000000000000000000000000000000000000000000000000000000000000000000000000000000000
0000000000000000000000000000000000000000011111111
>Q2FXC3_2vid_36_239

ENNVTKVKDTNIFPYTGVVAFKSATGFVVGKNTILTNKHVSKNYKVGDRITAHPNSDKGN
GGIYSIKKIINYPGKEDVSVIQVEERAIERGPKGFNFNDNVTPFKYAAGAKAGERIKVIGYPH
PYKNKYVLYESTGPVMSVEGSSIVYSAHTESGNSGSPVLNSNNELVGIHFASDVKNDDNRN
AYGVYFTPEIKKFIAENIDK
00000000000000000000000000000000000000000000000000000000000000000000000000000000000
00000000000000000000000000000000000000000000000000000000000000000000000000000000000
00000000000011000000000000000000000000
>Q8AA96_4hf7_28_235
QKHEFANYKRYATENAALAQPVKKEKRVVFMGNSITEGWVRTHPDFFKTNGYIGRGISGQ
TSYQFLLRFREDVINLSPALVVINAGTNDVAENTGAYNEDYTFGNIASMAELAKANKIKVIL
TSVLPAAEFPWRREIKDAPQKIQSLNARIEAYAKANKIPFVNYYQPMVVGENKALNPQYTK
DGVHPTGEGYDIMEALIKQAIEKAL
11100000000000000000000000000000000000000000000000000000000000000000000000000000000
00000000000000000000000000000000000000000000000000000000000000000000000000000000000
000000000000000000000000000000000000000000
>Q5HGM3_2j41_0_206
MDNEKGLLIVLSGPSGVGKGTVRKRIFEDPSTSYKYSISMTTRQMREGEVDGVDYFFKTRDA
FEALIKDDQFIEYAEYVGNYYGTPVQYVKDTMDEGHDVFLEIEVEGAKQVRKKFPDALFIFL
APPSLEHLRERLVGRGTESDEKIQSRINEARKEVEMMNLYDYVVVNDEVELAKNRIQCIVE
AEHLKRERVEAKYRKMILEAKK
11110000000000000000000000000000000000000000000000000000000000000000000000000000000
00000000000000000000000000000000000000000000111111111111111111111111000000000000000
00000000000000000000000000000000000000011
>P15141_3exw_134_342
GLTFNSNNICIDDNINTLWTGVNPTTANCQIMASSESNDCKLILTLVKTGGLVTAFVYVIGVS
NDFNMLTTHKNINFTAELFFDSTGNLLTSLSSLKTPLNHKSGQNMATGALTNAKGFMPSTT
AYPFNVNSREKENYIYGTCYYTASDHTAFPIDISVMLNQRALNNETSYCIRVTWSWNTGVA
PEVQTSATTLVTSPFTFYYIREDD
11111111111000000000000000000000000000000000000000000000000000000000000000000000000
00000000000000000000000000000000000000000000000000000000000000000000000000000000000
0000000000000000000000000000000000000000000
>Q9ZFS6_1m4v_30_233
SEHESKYENVTKDIFDLRDYYSRASKELKNVTGYRYSKGGKHYLIFDKNRKFTRIQIFGKDIE
RIKKRKNPGLDIFVVKEAENRNGTVYSYGGVTKKNQGAYYDYLSAPRFVIKKEVGAGVSV
HVKRYYIYKEEISLKELDFKLRQYLIQDFDLYKKFPKASKIKVTMKDGGYYTFELNKKLQTN
RMSDVIDGRNIEKIEANIR
11110000000000000000000000000000000000000000000000000000000000000000000000000000000
11100000000000000000000000000000000000000000000000000000000000000000000000000000000
00000000000000000000000000000000000000
>Q27709_1qv0_0_194
MSSKYAVKLKTDFDNPRWIKRHKHMFDFLDINGNGKITLDEIVSKASDDICAKLEATPEQT
KRHQVCVEAFFRGCGMEYGKEIAFPQFLDGWKQLATSELKKWARNEPTLIREWGDAVFDI
FDKDGSGTITLDEWKAYGKISGISPSQEDCEATFRHCDLDNSGDLDVDEMTRQHLGFWYTL
DPEADGLYGNGVP
11111100000000000000000000000000000000000000000000000000000000000000000000000000000
00000000000000000000000000000000000000001111000000000000000000000000000000000000000
00000000000000000000000000000
>A6NI73_2d3v_41_236
GNLSKATLWAEPGSVISRGNSVTIRCQGTLEAQEYRLVKEGSPEPWDTQNPLEPKNKARFSI
PSMTEHHAGRYRCYYYSPAGWSEPSDPLELVVTGFYNKPTLSALPSPVVTSGENVTLQCGSR
LRFDRFILTEEGDHKLSWTLDSQLTPSGQFQALFPVGPVTPSHRWMLRCYGSRRHILQVWS

EPSDLLEIPVS
11000000000000000000000000000000000000000000000000000000000000000000000000000000000
00000000000000000000001111111111111000000000000111000000000000000000000000000000000
000000000000000000000000000001
>Q9YDV3_2dvk_0_187
MGSIEEVLLEERLIGYLDPGAEKVLARINRPSKIVSTSSCTGRITLIEGEAHWLRNGARVAYKT
HHPISRSEVERVLRRGFTNLWLKVTGPILHLRVEGWQCAKSLLEAARRNGFKHSGVISIAED
SRLVIEIMSSQSMSVPLVMEGARIVGDDALDMLIEKANTILVESRIGLDTFSREVEELVECF
11111111111111111000000000000000000000000000011110000000000000000000000000000000000
00000000000000000000000000000000000000000000000000000000000000000000000000000000000
0000000000000000000001
>Q97JL1_3ccg_0_188
MWSYDKITDYLMNNLGEKRYKHSLGVMDTAVRLAGIYNEDTEKARIAGLVHDCAKKLPG
EKIIEICTNEGYELGDEDIRNSYLLHGLAGRILAKKVIGIDDEDVLNAIEFHTTGRPNMSLLE
KIIYIADYIEPGREFKGVDELRKAADEDLNKALLMSFDNTIKFVIDKGGFLHHNTIEARNYLI
SRKG
00000000000000000000000000000000000000000000000000000000000000000000000000000000000
00000000000000000000000000000000000000000000000000000000000000000000000000000000000
00000000000000000000001
>P00905_1i1q_1_191
ADILLLDNIDSFTWNLADQLRTNGHNVVIYRNHIPAQTLIDRLATMKNPVLMLSPGPGVPS
EAGCMPELLTRLRGKLPIIGICLGHQAIVEAYGGYVGQAGEILHGKASSIEHDGQAMFAGL
ANPLPVARYHSLVGSNVPAGLTINAHFNGMVMAVRHDADRVCGFQFHPESILTTQGARLL
EQTLAWAQQ
00000000000000000000000000000000000000000000000000000000000000000000000000000000000
00000000000000011100000000000000000000000000000000111000000000000000000000000000000
0000000000000000000000000
>P0AF03_1di7_2_194
TLRIGLVSISDRASSGVYQDKGIPALEEWLTSALTTPFELETRLIPDEQAIIEQTLCELVDEMSC
HLVLTTGGTGPARRDVTPDATLAVADREMPGFGEQMRQISLHFVPTAILSRQVGVIRKQALI
LNLPGQPKSIKETLEGVKDAEGNVVVHGIFASVPYCIQLLEGPYVETAPEVVAAFRPKSARR
DVSE
00000000000011111000000000000000000000000000000000000000000000000000000000000000000
00000000000000000000000000000000000000000000000000000000000000000000000000000000000
000000000000000000000001111
>P65941_1w30_0_192
MGAAGDAAIGRESRELMSAADVGRTISRIAHQIIEKTALDDPVGPDAPRVVLLGIPTRGVTL
ANRLAGNITEYSGIHVGHGALDITLYRDDLMIKPPRPLASTSIPAGGIDDALVILVDDVLYSG
RSVRSALDALRDVGRPRAVQLAVLVDRGHRELPLRADYVGKNVPTSRSESVHVRLREHDG
RDGVVISR
11111111111000000000000000000000000000000000000000000000000000000000000000000000000
00000001111100000000000000000000000000000000000000000000000000000000000000000000000
000000000000000000000000000
>Q62191_2vok_290_469
VHITLDRNTANSWLIISKDRRQVRMGDTHQNVSDNKERFSNYPMVLGAQRFSSGKMYWE
VDVTQKEAWDLGVCRDSVQRKGQFSLSPENGFWTIWLWQDSYEAGTSPQTTLHIQVPPCQ
IGIFVDYEAGVVSFYNITDHGSLIYTFSECVFAGPLRPFFNVGFNYSGGNAAPLKLCPLKM
00000000000000000000000000000000000000000000000000000000000000000000000000000000000
00000000000000000000000000000000000000000000000000000000000000000000000000000000000
00000000000011
>B9TU22_3gxr_30_216

VGYGDITQVETSGASSKTSRQDKLEYDGVRASHTMAQTDAGRMEKYKSFINNVAKKHVV
DPAVIAAIISRESRAGNVIFNTTPPGWGDNYNGFGLMQVDKRYHEPRGAWNSEEHIDQAT
GILVNFIQLIQKKFPSWSTEQQLKGGIAAYNTGDGRVESYESVDSRTTGKDYSNDVVARAQ
WYKKNGF
10000000000000000000000000000000000000000000000000000000000000000000000000000000
00000000000000000000000000000000000000000000000000000000000000000000000000000000
00000000000000000000
>P07229_1bg7_0_175
MDSQVRQNFHRDCEAAINRMVNMELYASYTYLSMAFYFDRDDIALHNVAKFFKEQSHEE
REHAEKLMKDQNKRGGRIVLQDVKKPERDEWGNTLEAMQAALQLEKTVNQALLDLHK
VGSDKVDPHLCDFLETEYLEEQVKSIKQLGDYITNLKRLGLPQNGMGEYLFDKHTMGESS
10000000000000000000000000000000000000000000000000000000000000000000000000000000
00000000000000000000000000000011111111111111111000000000000000000000000000000000
0000000011
>P11672_3s26_20_199
QDSTQNLIPAPSLLTVPLQPDFRSDQFRGRWYVVGLAGNAVQKKTEGSFTMYSTIYELQEN
NSYNVTSILVRDQDQGCRYWIRTFVPSSRAGQFTLGNMHRYPQVQSYNVQVATTDYNQFA
MVFFRKTSENKQYFKITLYGRTKELSPELKERFTRFAKSLGLKDDNIIFSVPTDQCIDN
11111000000000000000000000000000000000000011100000000000000000000000111100000000
00000000000000000000000000000000000000000000000000000000000000000000000000000000
00000000000000
>Q8SXQ7_2xz4_51_225
SPNKGLHILDRSEWLGEPPSGKYPHLKLPVSNIIIHHTATEGCEQEDVCIYRMKTIQAFHMK
SFGWVDIGYNFLVGGDGQIYVGRGWHIQGQHVNGYGAISVSIAFIGTFVNMEPPARQIEAA
KRLMDEGVRLHRLQPDYHIYAHRQLSPTESPGQKLFELMQNWPRFTQDPTSL
11111100000000000000000000000000000000000000000000000000000000000000000000000000
00000000000000000000000000000000000000000000000000000000000000000000000000000000
000001111
>Q8ZPD3_3dr6_0_170
MTIRFADKADCAAITEIYNHAVLHTAAIWNDRTVDTDNRLAWYEARQLLGYPVLVSEENG
VVTGYASFGDWRSFDGFRYTVEHSVYVHPAHQGKGLGRKLLSRLIDEARRCGKHVMVAGI
ESQNAASIRLHHSLGFTVTAQMPQVGVKFGRWLDLTFMQLQLDEHAAPDAC
00000000000000000000000000000000000000000000000000000000000000000000000000000000
00000000000000000000000000000000000000000000000000000000000000000000000000000000
00001
>B7AGU1_4ici_20_189
QQKQKMNSKHSNSKILVAYFSATGTTARAAEKLGAAVGGDLYPIAPAQPYTSADLDWNNK
RSRSSVEMNDPKMRPAIKSKKENIGTYDVVFIGYPIWWDLAPRIINTFIEGHSLKGKTVVPFA
TSGGSSIGNSATVLKKTYPDLNWKEGRLLNRTDEKAIRAWLDVIAVK
11111111111100000000000000000000000000000000000000000000000000000000000000000000
00000000000000000000000000000000000000000000000000000000000000000000000000000000
0000
>Q8P559_3qyy_127_290
MRDLKRHALFDPLTEALNRRGCEQAMRDSVTAAQREGWPFVLFVLDMDNLKPINDRFGH
LAGDRVLVRLVESAYGWLGAQDWIGRWGGDEFLIGVHASEDEATLKLNQWLSMLEREDA
EEAPLHVSAGSAVCEVGIDATELYRRADAAMYRAKFSGGRRLVRDG
11000000000000000000000000000000000000000000000000000000000000000000000000000000
000000000000000000000000000000001111000000000000000000000000000000000000000000001
>Q8XAB8_3eye_1_157
SSPNILLTRIDNRLVHGQVGVTWTSTIGANLLVVVDDVVANDDIQQKLMGITAETYGFGIRF
FTIEKTINVIGKAAPHQKIFLICRTPQTVRKLVEGGIDLKDVNVGNMHFSEGKKQISSKVYV

DDQDLTDLRFIKQRGVNVFIQDVPGDQKEQIPD
11100000000000000000000000000000000000000000000000000000000000000000000000000000000
00000000000000000000000000000000000000000000000000000000000000000000000001
>O64527_3emm_0_165
MNQLQQLQNPGESPPVHPFVAPLSYLLGTWRGQGEGEYPTIPSFRYGEEIRFSHSGKPVIAYT
QKTWKLESGAPMHAESGYFRPRPDGSIEVVIAQSTGLVEVQKGTYNVDEQSIKLKSDLVGN
ASKVKEISREFELVDGKLSYVVRMSTTTNPLQPHLKAILDKL
11111111111110000000000000000000000000000000000000000000000000000000000000000000000
00000000000000000000000000000000000000000000000000000000000000000000000000000000000
>Q99KK2_3ewi_266_431
KEKLKEIKLLVCNIDGCLTNGHIYVSGDQKEIISYDVKDAIGISLLKKSGIEVRLISERACSKQT
LSALKLDCKTEVSVSDKLATVDEWRKEMGLCWKEVAYLGNEVSDEECLKRVGLSAVPADA
CSGAQKAVGYICKCSGGRGAIREFAEHIFLLIEKVNNSCQK
11110000000000000000000000000000000000000000000000000000000000000000000000000000000
00000000000000000000000000000000000000000000000000000000000000000000000000000000111
>Q9V3I1_3ecy_0_159
MPSTDFADIPAAKKMKIDTCVLRFAKLTENALEPVRGSAKAAGVDLRSAYDVVVPARGKAI
VKTDLQVQVPEGSYGRVAPRSGLAVKNFIDVGAGVVDEDYRGNLGVVLFNHSDVDFEVK
HGDRIAQFICERIFYPQLVMVDKLEDTERGEAGFGSTGVK
11111111111111111100000000000000000000000000000000000000000000000000000000000000011
11111000000000000000000000000000000000000000000000000000001111111111111111
>Q06848_3ul4_29_174
QTNTIEIIIGNVKARPGDRIEVPVSLKNVPDKGIVSSDFVIEYDSKLFKVIELKAGDIVENPSES
FSYNVVEKDEIIAVLYLEETGLGIEAIRTDGVFFTIVMEVSKDVKPGISPIKFESFGATADNDM
NEMTPKLVEGKVEIIEA
11000000000000000000000000000000000000000000000000000000000000000000000000000000000
000000000000000000000000000000000000000000000000000000000000011
>Q9WZU8_1o6d_1_150
RVRIAVIGKLDGFIKEGIKHYEKFLRRFCKPEVLEIKRVHRGSIEEIVRKETEDLTNRILPGSFV
MVMDKRGEEVSSEEFADFLKDLEMKGKDITILIGGPYGLNEEIFAKAHRVFSLSKMTFTHG
MTVLIVLEQIFRAFKIIHGENYHY
00000000000000000000000000000000000000000000000000000000000000000000000000000000000
0000000000000000000000000000000000000000000000000000000000000001111
>P43577_1i12_0_158
MSLPDGFYIRRMEEGDLEQVTETLKVLTTVGTITPESFSKLIKYWNEATVWNDNEDKKIMQ
YNPMVIVDKRTETVAATGNIIIERKIIHELGLCGHIEDIAVNSKYQGQGLGKLLIDQLVTIGFD
YGCYKIILDCDEKNVKFYEKCGFSNAGVEMQIRK
00000000000000000000000000000000000000000000000000000111100000000000000000000000000
0000000000000000000000000000000000000000000000000000000000000000000000000000
>Q1MFM4_3otl_0_157
MTIRSAEHATLVIERHLKAPISRVFRAWSTPESKRQWFACHGEWVPLDYGLDFRPGGTERN
YVADTDGLLHAYDAHYIDIVPDARIIYAYEMKLGESRISASLTTVAFEAAPGGTKMVFTEQV
VFLDGYADNGARLQGTEIGLDNLELFLEREESPIH
10000000000000000000000000000000000000000000000000000000000000000000000000000000000
000000000000000000000000000000000000000000000000000000000000000000000001111
>D6ZTY6_3u80_0_147
MTKVIVVNGPNLGRLGVRQPDVYGRQDLDTLRKLCAEWGKDLGLEVEVRQTDDEAEMV
RWMHQAADEKTPVVMNPAAFTHYSYALADAAHMVIDENLPLMEVHISNPSARDEFRKRS
VISPVATGTITGMGFYGYKLALDAVAHLLSE
00000000000011111111111100000000000000000000000000000000000000000000000000000000000
00000000000000000000000000111111000000000000000000000000000000000

>Q2PHL4_3lei_37_188
EQGNRPVETENIARGKQASQSSTAYGGAAARAVDGNVDSDYGHHSVTHTNFEDNAWWQ
VDLGKTENVGKVKLYNRGDGNVANRLSNFDVVLLNEAKQEVARQHFDSLNGKAELEVFF
TAKAARYVKVELKTKNTPLSLAEVEVFRSATTQVG
11111000000000000000000000000000000000000000000000000000000000000000000000000000000
000000000000000000000000000000000000000000000000000000000000000001111
>Q8WUJ0_2r0b_25_176
RREMQEILPGLFLGPYSSAMKSKLPVLQKHGITHIICIRQNIEANFIKPNFQQLFRYLVLDIAD
NPVENIIRFFPMTKEFIDGSLQMGGKVLVHGNAGISRSAAFVIAYIMETFGMKYRDAFAYVQ
ERRFCINPNAGFVHQLQEYEAIYLAK
00000000000000000000000000000000000000000000000000000000000000000000000000000000000
000000000000000000000000000000000000000000000000000000000000000000001
>Q5SJV7_2z09_0_136
MFKTILLAYDGSEHARRAAEVAKAEAEAHGARLIVVHAYEPVPDYLGEPFFEEALRRRLER
AEGVLEEARALTGVPKEDALLLEGVPAEAILQAARAEKADLIVMGTRGLGALGSLFLGSQS
QRVVAEAPCPVLLVR
00000000000000000000000000000000000000000111111111111100000000000000000000000000000
000000000000000000000000000000000000000000000000000000
>P02213_4hbi_0_145
PSVYDAAAQLTADVKKDLRDSWKVIGSDKKGNGVALMTTLFADNQETIGYFKRLGDVSQ
GMANDKLRGHSITLMYALQNFIDQLDNPDDLVCVVEKFAVNHITRKISAAEFGKINGPIKK
VLASKNFGDKYANAWAKLVAVVQAAL
10000000000000000000000000000000000000000000000000000000000000000000000000000000000
000000000000000000000000000000000000000000000000000000000000000
>P04252_1vhb_0_145
MLDQQTINIIKATVPVLKEHGVTITTTFYKNLFAKHPEVRPLFDMGRQESLEQPKALAMTV
LAAAQNIENLPAILPAVKKIAVKHCQAGVAAAHYPIVGQELLGAIKEVLGDAATDDILDA
WGKAYGVIADVFIQVEADLYAQAVE
10000000000000000000000000000000000000000001111111110000000000000000000000000000000
000000000000000000000000000000000000000000000000000000000000001
>A7LRD6_4gzv_22_162
QKKTKFKAADLKGIWQLCHYVSESPDVPGALKPSNTFKVLSDDGRIVNFTIIPGADAIITGY
GTYKQLTDDSYKESIEKNIHLPMLDNQDNILEFEIKDNDYLHLKYFIKSDLNGNELNTWYY
ETWKRVEMPAKFPEDIVR
11111000000000000000000000000000000000000000000000000000000000000000000000000000000
0000000000000000000000000000000000000000000000000000000000
>Q89BM6_3i4s_34_171
SEPAWSLHSRLKEDTIDIGDLPLSKVLVIKDANYPWLLLVPRRPDAVEIIDLDEVQQAQLMTE
ISRVSRALKEITKCDKLNIAALGNLVPQLHVHIIARRTGDAAWPRPVWGVMQPLAHDATE
VQNFISALRRKIWLG
11000000000000000000000000000000000000000000000000000000000000000000000000000000000
0000000000000000000000000000000000000000000000000000001
>O83028_2xqc_0_139
MTYVILPLEMKKGRGYVYQLEYHLIWCVKYRHQVLVGEVADGLKDILRDIAAQNGLEVIT
MEVMPDHVHLLLSATPQQAIPDFVKALKGASARRMFVAYPQLKEKLWGGNLWNPSYCILT
VSENTRAQIQKYIESQHDKE
11111111000000000000000000000000000000000000000000000000000000000000000000000000000
000000000000000000000000000000000000000000000000000000000
>Q838I6_3bed_0_138
MKPKLILMSHGRMAEETLASTQMIVGELADAAIVSMTAEDGLSGTQAKLAAILKEAGNVP
TLVLADLKGGTPCNVAMMAMGTYPQLRVVAGLNLAMAIEAAVSPVENVDELAAYLTQIG

QSAVTTIDLPELTDEEEFEE
00000000000000000000000000000000000000000000000000000000000000000000000000000000
000000000000000000000000000000000000000000000001111111
>Q9KG76_1w9s_789_924
DLKNPYERIQAEAYDAMSGIQTEGTDDDGGGDNIGWINDGDWVKYERVHFERDASSIEVR
VASDTPGGRIEIRTGSPTGTLLGDVQVPNTGGWQQWQTVTGNVQIQPGTYDVYLVFKGSPE
YDLMNVNWFVFRANG
00000000000000000000000000000000000000000000000000000000000000000000000000000000
000000000000000000000000000000000000000000000000011
>P09382_3t2t_0_134
MACGLVASNLNLKPGECLRVRGEVAPDAKSFVLNLGKDSNNLCLHFNPRFNAHGDANTI
VCNSKDGGAWGTEQREAVFPFQPGSVAEVCITFDQANLTVKLPDGYEFKFPNRLNLEAINY
MAADGDFKIKCVAFD
10000000000000000000000000000000000000000000000000000000000000000000000000000000
00000000000000000000000000000000000000000000000000
>Q7YYQ3_2aif_1_133
SQNEASEDTGFNPKAFPLASPDLNNKIINLVQQACNYKQLRKGANEATKALNRGIAEIVLL
AADAEPLEILLHLPLVCEDKNTPYVFVRSKVALGRACGVSRPVIAAAITSKDGSSLSSQITEL
KDQIEQILV
11111111111111100000000000000000000000000000000000000000000000000000000000000000
000000000000000000000000000000000000000000000111
>Q9WZ82_1o3u_0_122
MDAAKDDLEHAKHDLEHGFYNWACFSSQQAAEKAVKAVFQRMGAQAWGYSVPDFLGE
LSSRFEIPEELMDHALELDKACIPTRYPDALPSGSPRNRYSRIEAERLVNYAEKIIRFCEDLLSR
I
00000000000000000000000000000000000000000000000000000000000000000000000000001111
110000000000000000000000000000000000000
>Q7BSV8_3g20_24_143
ASLVVPNLMGNKDKADRQKVMSDLVALESTLDMYRLDNNRYPTTEQGLRALVSKPTVQP
EPRNYRQDGYIRRLPQDPWGGDYQLLNPGQYSDIDIFSPGPDGVPNTEDDIGNWTLGNAQ
P
10000000000000000000000000000000000000000000000000000000000000000000000000000000
000000000000000000000000000000000000
>Q9P1W8_2jjw_28_146
EEELQMIQPEKLLLVTVGKTATLHCTVTSLLPVGPVLWFRGVGPGRELIYNQKEGHFPRVTT
VSDLTKRNNMDFSIRISSITPADVGTYYCVKFRKGSPENVEFKSGPGTEMALGAKPS
11100000000000000000000000000000000000000000000000000000000000000000000000000000
00000000000001111100000000000000001
>P0ACE7_3n1t_0_118
MAEETIFSKIIRREIPSDIVYQDDLVTAFRDISPQAPTHILIIPNILIPTVNDVSAEHEQALGRMI
TVAAKIAEQEGIAEDGYRLIMNTNRHGGQEVYHIHMHLLGGRPLGPMLAHKGL
11000000000000000000000000000000000000000000000000000000000000000000000000000000
00000000000000000000000000000000011
>P0A1D1_3cgi_0_115
MERQPTTDRMIQEYVPGKQVTLAHLIANPGKDLFKKLGLQDAVSAIGILTITPSEASIIACDI
ATKSGAVEIGFLDRFTGAVVLTGDVSAVEYALKQVTRTLGEMMQFTTCSITRT
11111000000000000000000000000000000000000000000000000000000000000000000000000000
00000000000000000000000000000000
>Q03330_1e6i_323_438
AQRPKRGPHDAAIQNILTELQNHAAAWPFLQPVNKEEVPDYYDFIKEPMDLSTMEIKLESN
KYQKMEDFIYDARLVFNNCRMYNGENTSYYKYANRLEKFFNNKVKEIPEYSHLID

11111000000000000000000000000000000000000000000000000000000000000000000000000000000
000000000000000000000000000000000
>Q06110_1j48_33_142
APAFSVSPASGLSDGQSVSVSVSVSGAAAGETYYIAQCAPVGGQDACNPATATSFTTDASGAA
SFSFVVRKSYTGSTPEGTPVGSVDCATAACNLGAGNSGLDLGHVALTFG
00000000000000000000000000000000000000000000000000000000000000000000000000000000000
000000000000000000000000001
>Q52440_2e4q_0_108
MTFTKACSVDEVPPGEALQVSHDAQKVAIFNVDGEFFATQDQCTHGEWSLSEGGYLDGD
VVECSLHMGKFCVRTGKVKSPPPCEPLKVYPIRIEGRDVLVDFSRAALHA
10000000000000000000000000000000000000000000000000000000000000000000000000000000000
00000000000000000000000000
>P78352_3i4w_301_402
LGEEDIPREPRRIVIHRGSTGLGFNIVGGEDGEGIFISFILAGGPADLSGELRKGDQILSVNGV
DLRNASHEQAAIALKNAGQTVTIIAQYKPEEYSRFEAK
11100000000000000000000000000000000000000000000000000000000000000000000000000000000
0000000000000000001
>Q9PIK3_3plx_25_125
ISIDEKLLQASGILEYEKVQVVNVNNGARFETYTIATQEEGVVCLNGAAARLAEVGDKVIIM
SYADFNEEEAKTFKPKVVFVDENNTATKITNYEKHGAIF
00000000000000000000000000000000000000000000000000000000000000000000000000000000000
000000000000000001
>Q82WP3_2omo_0_99
MYVTIVYASVKTDKTEAFKEATRMNHEQSIREPGNMRFDILQSADDPTRFVLYEAYKTRKD
AAAHKETAHYLTWRDTVADWMAEPRKGVIYGGLYPTGDD
00000000000000000000000000000000000000000000000000000000000000000000000000000000000
00000000000000111
>A6LX94_3sny_117_212
PTKAVTFYEDINYGGASVSLQPGNYTLSQLNTAKIPNDWMTSLKVPSGWTVDVYENDNFT
GTKWTYTSDTPWVGNDANDKMTSVKIYSTTNTGGDT
11000000000000000000000000000000000000000000000000000000000000000000000000000000000
0000001111111
>O15021_2w7r_1142_1233
QPIVIHSSGKNYGFTIRAIRVYVGDSDIYTVHHIVWNVEEGSPACQAGLKAGDLITHINGEP
VHGLVHTEVIELLLKSGNKVSITTTPFENT
00000000100000000000000000000000000000000000000000000000000000000000000000000000000
000000000
>P13479_2vlo_0_85
MELKHSISDYTEAEFLQLVTTICNADTSSEEELVKLVTHFEEMTEHPSGSDLIYYPKEGDDDS
PSGIVNTVKQWRAANGKSGFKQG
11100000000000000000000000000000000000000000000000000000000000000000000000000000000
000
>O67611_2ehs_1_77
SLEERVKEIIAEQLGVEKEKITPEAKFVEDLGADSLDVVELIMAFEEEFGIEIPDEDAEKIQTV
GDVINYLKEKVGG
00000000000000000000000000000000000000000000000000000000000000000000000000011
>Q06ZW0_2h5f_34_110
QAVGLPHGFCIQCNRKTWSNCSIGHRCLPYHMTCYTLYKPDENGEMKWAVKGCARMCPT
AKSGERVKCCTGASCNSD
11000000000000000000000000000000000000000000000000000000000000000000000000000
>O76041_4f14_954_1013

NLRTYRAMYDYSAQDEDEVSFRDGDYIVNVQPIDDGWMYGTVQRTGRTGMLPANYIEFV
N
111000000000000000000000000000000000000000000000000000000001
>P07811_2fh8_19_1086
CDNSSSSSTSGSPGSPGNPGNPGTPGTPDPQDVVVRLPDVAVPGEAVQASARQAVIHLVDIA
GITSSTPADYATKNLYLWNNETCDALSAPVADWNDVSTTPTGSDKYGPYWVIPLTKESGSIN
VIVRDGTNKLIDSGRVSFSDFTDRTVSVIAGNSAVYDSRADAFRAAFGVALADAHWVDKTT
LLWPGGENKPIVRLYYSHSSKVAADSNGEFSDKYVKLTPTTVNQQVSMRFPHLASYPAFKLP
DDVNVDELLQGDDGGIAESDGILSLSHPGADRRRAGRYLCRRAEALSYGAQLTDSGVTFRV
WAPTAQQVELVIYSADKKVIASHPMTRDSASGAWSWQGGSDLKGAFYRYAMTVYHPQSR
KVEQYEVTDPYAHSLSTNSEYSQVVDLNDSALKPEGWDGLTMPHAQKTKADLAKMTIHE
SHIRDLSAWDQTVPAELRGKYLALTAQESNMVQHLKQLSASGVTHIELLPVFDLATVNEFS
DKVADIQQPFSRLCEVNSAVKSSEFAGYCDSGSTVEEVLTQLKQNDSKDNPQVQALNTLVA
QTDSYNWGYDPFHYTVPEGSYATDPEGTARIKEFRTMIQAIKQDLGMNVIMDVVYNHTNA
AGPTDRTSVLDKIVPWYYQRLNETTGSVESATCCSDSAPEHRMFAKLIADSLAVWTTDYKI
DGFRFDLMGYHPKAQILSAWERIKALNPDIYFFGEGWDSNQSDRFEIASQINLKGTGIGTFS
DRLRDAVRGGGPFDSGDALRQNQGVGSGAGVLPNELTTLSDDQARHLADLTRLGMAGN
LADFVLIDKDGAVKRGSEIDYNGAPGGYAADPTEVVNYVSKHDNQTLWDMISYKAAQEA
DLDTRVRMQAVSLATVMLGQGIAFDQQGSELLRSKSFTRDSYDSGDWFNRVDYSLQDNNY
NVGMPRSSDDGSNYDIIARVKDAVATPGETELKQMTAFYQELTALRKSSPLFTLGDGATVM
KRVDFRNTGADQQTGLLVMTIDDGMQAGVQVAADGSVTLPAWSVAVLELPQ
111111111111111111111111111111100000000111111111111111111111111111111111111111111111111
111111111111111111111111111111111111111111111111111111111111111111111111111111111111111
110000000000000000000000000000000000000000000000000000000000000000000000000000000000000
000000000000000000000000000000000000000000000000000000000000000000000000000000000000000
000000000000000000000000000000000000000000000000000000000000000000000000000000000000000
000000000000000000000000000000000000000000000000000000000000000000000000000000000000000
000000000000000000000000000000000000000000000000000000000000000000000000000000000000000
000000000000000000000000000000000000000000000000000000000000000000000000000000000000000
000000000000000000000000000000000000000000000000000000000000000000000000000000000000000
000000000000000000000000000000000000000000000000000000000000000000000000000000000000000
000000000000000000000000000000000000000000000000000000000000000000000000000000000000000
000000000000000000000000000000000000000000000000000000000000000000000000000000000000000
000000000000000000000000
>P24183_1kqf_0_1014
MDVSRRQFFKICAGGMAGTTVAALGFAPKQALAQARNYKLLRAKEIRNTCTYCSVGCGLL
MYSLGDGAKNAREAIYHIEGDPDHPVSRGALCPKGAGLLDYVNSENRLRYPEYRAPGSDK
WQRISWEEAFSRIAKLMKADRDANFIEKNEQGVTVNRWLSTGMLCASGASNETGMLTQK
FARSLGMLAVDNQARVHGPTVASLAPTFGRGAMTNHWVDIKNANVVMVMGGNAAEA
HPVGFRWAMEAKNNNDATLIVVDPRFTRTASVADIYAPIRSGTDITFLSGVLRYLIENNKIN
AEYVKHYTNASLLVRDDFAFEDGLFSGYDAEKRQYDKSSWNYQLDENGYAKRDETLTHPR
CVWNLLKEHVSRYTPDVVENICGTPKADFLKVCEVLASTSAPDRTTTFLYALGWTQHTVG
AQNIRTMAMIQLLLGNMGMAGGGVNALRGHSNIQGLTDLGLLSTSLPGYLTLPSEKQVDL
QSYLEANTPKATLADQVNYWSNYPKFFVSLMKSFYGDAAQKENNWGYDWLPKWDQTY
DVIKYFNMMDEGKVTGYFCQGFNPVASFPDKNKVVSCLSKLKYMVVIDPLVTETSTFWQN
HGESNDVDPASIQTEVFRLPSTCFAEEDGSIANSGRWLQWHWKGQDAPGEARNDGEILAG
IYHHLRELYQSEGGKGVEPLMKMSWNYKQPHEPQSDEVAKENNGYALEDLYDANGVLIA
KKGQLLSSFAHLRDDGTTASSCWIYTGSWTEQGNQMANRDNSDPSGLGNTLGWAWAWP
LNRRVLYNRASADINGKPWDPKRMLIQWNGSKWTGNDIPDFGNAAPGTPTGPFIMQPEG
MGRLFAINKMAEGPFPEHYEPIETPLGTNPLHPNVVSNPVVRLYEQDALRMGKKEQFPYV
GTTYRLTEHFHTWTKHALLNAIAQPEQFVEISETLAAAKGINNGDRVTVSSKRGFIRAVAVV

TRRLKPLNVNGQQVETVGIPIHWGFEGVARKGYIANTLTPNVGDANSQTPEYKAFLVNIEK
A
11111111111111111111111111111111100000000000000000000000000000000000000000000000000
00000000000000000000000000000000000000000000000000000000000000000000000000000000000
00000000000000000000000000000000000000000000000000000000000000000000000000000000000
00000000000000000000000000000000000000000000000000000000000000000000000000000000000
00000000000000000000000000000000000000000000000000000000000000000000000000000000000
00000000000000000000000000000000000000000000000000000000000000000000000000000000000
00000000000000000000000000000000000000000000000000000000000000000000000000000000000
00000000000000000000000000000000000000000000000000000000000000000000000000000000000
00000000000000000000000000000000000000000000000000000000000000000000000000000000000
00000000000000000000000000000000000000000000000000000000000000000000000000000000000
00000000000000000000000000000000000000000000000000000000000000000000000000000000000
00000000000000000000000000000000000000000000000000000000000000000000000000000000000
000000000000000000
>P04825_2dqm_0_869
MTQQPQAKYRHDYRAPDYQITDIDLTFDLDAQKTVVTAVSQAVRHGASDAPLRLNGEDL
KLVSVHINDEPWTAWKEEEGALVISNLPERFTLKIINEISPAANTALEGLYQSGDALCTQCEA
EGFRHITYYLDRPDVLARFTTKIIADKIKYPFLLSNGNRVAQGELENGRHWVQWQDPFPKP
CYLFALVAGDFDVLRDTFTTRSGREVALELYVDRGNLDRAPWAMTSLKNSMKWDEERFGL
EYDLDIYMIVAVDFFNMGAMENKGLNIFNSKYVLARTDTATDKDYLDIERVIGHEYFHNW
TGNRVTCRDWFQLSLKEGLTVFRDQEFSSDLGSRAVNRINNVRTMRGLQFAEDASPMAHPI
RPDMVIEMNNFYTLTVYEKGAEVIRMIHTLLGEENFQKGMQLYFERHDGSAATCDDFVQA
MEDASNVDLSHFRRWYSQSGTPIVTVKDDYNPETEQYTLTISQRTPATPDQAEKQPLHIPFA
IELYDNEGKVIPLQKGGHPVNSVLNVTQAEQTFVFDNVYFQPVPALLCEFSAPVKLEYKWS
DQQLTFLMRHARNDFSRWDAAQSLLATYIKLNVARHQQGQPLSLPVHVADAFRAVLLDE
KIDPALAAEILTLPSVNEMAELFDIIDPIAIAEVREALTRTLATELADELLAIYNANYQSEYRV
EHEDIAKRTLRNACLRFLAFGETHLADVLVSKQFHEANNMTDALAALSAAVAAQLPCRD
ALMQEYDDKWHQNGLVMDKWFILQATSPAANVLETVRGLLQHRSFTMSNPNRIRSLIGAF
AGSNPAAFHAEDGSGYLFLVEMLTDLNSRNPQVASRLIEPLIRLKRYDAKRQEKMRAALEQ
LKGLENLSGDLYEKITKALA
11100000000000000000000000000000000000000000000000000000000000000000000000000000000
00000000000000000000000000000000000000000000000000000000000000000000000000000000000
00000000000000000000000000000000000000000000000000000000000000000000000000000000000
00000000000000000000000000000000000000000000000000000000000000000000000000000000000
00000000000000000000000000000000000000000000000000000000000000000000000000000000000
00000000000000000000000000000000000000000000000000000000000000000000000000000000000
00000000000000000000000000000000000000000000000000000000000000000000000000000000000
00000000000000000000000000000000000000000000000000000000000000000000000000000000000
00000000000000000000000000000000000000000000000000000000000000000000000000000000000
00000000000000000000000000000000000000000000000000000000000000000000000000000000000
0000000000000000000000000000000000000000
>P00489_3l7a_0_842
MSRPLSDQEKRKQISVRGLAGVENVTELKKNFNRHLHFTLVKDRNVATPRDYYFALAHTV
RDHLVGRWIRTQQHYYEKDPKRIYYLSLEFYMGRTLQNTMVNLALENACDEATYQLGLD
MEELEEIEEDAGLGNGGLGRLAACFLDSMATLGLAAYGYGIRYEFGIFNQKICGGWQMEE
ADDWLRYGNPWEKARPEFTLPVHFYGRVEHTSQGAKWVDTQVVLAMPYDTPVPGYRNN
VVNTMRLWSAKAPNDFNLKDFNVGGYIQAVLDRNLAENISRVLYPNDNFFEGKELRLKQE
YFVVAATLQDIIRRFKSSKFGCRDPVRTNFDAFPDKVAIQLNDTHPSLAIPELMRVLVDLERL
DWDKAWEVTVKTCAYTNHTVLPEALERWPVHLLETLLPRHLQIIYEINQRFLNRVAAAFPG
DVDRLRRMSLVEEGAVKRINMAHLCIAGSHAVNGVARIHSEILKKTIFKDFYELEPHKFQN

KTNGITPRRWLVLCNPGLAEIIAERIGEEYISDLDQLRKLLSYVDDEAFIRDVAKVKQENKLK
FAAYLEREYKVHINPNSLFDVQVKRIHEYKRQLLNCLHVITLYNRIKKEPNKFVVPRTVMIG
GKAAPGYHMAKMIIKLITAIGDVVNHDPVVGDRLRVIFLENYRVSLAEKVIPAADLSEQIST
AGTEASGTGNMKFMLNGALTIGTMDGANVEMAEEAGEENFFIFGMRVEDVDRLDQRGY
NAQEYYDRIPELRQIIEQLSSGFFSPKQPDLFKDIVNMLMHHDRFKVFADYEEYVKCQERVS
ALYKNPREWTRMVIRNIATSGKFSSDRTIAQYAREIWGVEPSRQRLPAPDEKIP
11111111111100000000000000000000000000000000000000000000000000000000000000000000000
00000000000000000000000000000000000000000000000000000000000000000000000000000000000
00000000000000000000000000000000000000000000000000000000000000000000000000000000000
00000011111100000000000000000000000000000000000000000000000000001111111100000000
00000000000000000000000000000000000000000000000000000000000000000000000000000000000
00000000000000000000000000000000000000000000000000000000000000000000000000000000000
00000000000000000000000000000000000000000000000000000000000000000000000000000000000
00000000000000000000000000000000000000000000000000000000000000000000000000000000000
00000000000000000000000000000000000000000000000000000000000000000000000000000000000
00000000000000000000000000000000000000000000000000000000000000000000000000000000000
0000000111111
>O81086_3sdt_0_816
MAGVSAVSKVSSLVCDLSSTSGLIRRTANPHPNVWGYDLVHSLKSPYIDSSYRERAEVLVSEI
KAMLNPAITGDGESMITPSAYDTAWVARVPAIDGSARPQFPQTVDWILKNQLKDGSWGIQS
HFLLSDRLLATLSCVLVLLKWNVGDLQVEQGIEFIKSNLELVKDETDQDSLVTDFEIIFPSLLR
EAQSLRLGLPYDLPYIHLLQTKRQERLAKLSREEIYAVPSPLLYSLEGIQDIVEWERIMEVQSQ
DGSFLSSPASTACVFMHTGDAKCLEFLNSVMIKFGNFVPCLYPVDLLERLLIVDNIVRLGIYR
HFEKEIKEALDYVYRHWNERGIGWGRLNPIADLETTALGFRLLRLHRYNVSPAIFDNFKDA
NGKFICSTGQFNKDVASMLNLYRASQLAFPGENILDEAKSFATKYLREALEKSETSSAWNN
KQNLSQEIKYALKTSWHASVPRVEAKRYCQVYRPDYARIAKCVYKLPYVNNEKFLELGKLD
FNIIQSIHQEEMKNVTSWFRDSGLPLFTFARERPLEFYFLVAAGTYEPQYAKCRFLFTKVACL
QTVLDDMYDTYGTLDELKLFTEAVRRWDLSFTENLPDYMKLCYQIYYDIVHEVAWEAEKE
QGRELVSFFRKGWEDYLLGYYEEAEWLAAEYVPTLDEYIKNGITSIGQRILLLSGVLIMDGQ
LLSQEALEKVDYPGRRVLTELNSLISRLADDTKTYKAEKARGELASSIECYMKDHPECTEEE
ALDHIYSILEPAVKELTREFLKPDDVPFACKKMLFEETRVTMVIFKDGDGFGVSKLEVKDHIK
ECLIEPLPL
11111111111111111111111111111111100000000000000000000000000000000000000000000000000
00000000000000000000000000000000000000000000000000000000000000000000000000000000000
00000000000000000000000000000000000000000000000000000000000000000000000000000000000
00000000000000000000000000000000000000000000000000000000000000000000000000000000000
00000000000000000000000000000000000000000000110000000000000000000000000000000000000
00000000000000000000000000000000000000000000000000000000000000000000000000000000000
00000000000000000000000000000000000000000000000000000000000000000000000000000000000
00000000000000000000000000000000000000000000000000000000000000000000000000000000000
00000000000000000000000000000000000000000000000000000000111111000000000000000000000
0000000000000000000000000000000000000000000000000000000000000000000000
>Q9NY33_3fvy_0_725
MADTQYILPNDIGVSSLDCREAFRLLSPTERLYAYHLSRAAWYGGLAVLLQTSPEAPYIYALL
SRLFRAQDPDQLRQHALAEGLTEEEYQAFLVYAAGVYSNMGNYKSFGDTKFVPNLPKEKL
ERVILGSEAAQQHPEEVRGLWQTCGELMFSLEPRLRHLGLGKEGITTYFSGNCTMEDAKLA
QDFLDSQNLSAYNTRLFKEVDGEGKPYYEVRLASVLGSEPSLDSEVTSKLKSYEFRGSPFQVT
RGDYAPILQKVVEQLEKAKAYAANSHQGQMLAQYIESFTQGSIEAHKRGSRFWIQDKGPIV
ESYIGFIESYRDPFGSRGEFEGFVAVVNKAMSAKFERLVASAEQLLKELPWPPTFEKDKFLTP
DFTSLDVLTFAGSGIPAGINIPNYDDLRQTEGFKNVSLGNVLAVAYATQREKLTFLEEDDKDL
YILWKGPSFDVQVGLHELLGHGSGKLFVQDEKGAFNFDQETVINPETGEQIQSWYRSGET

WDSKFSTIASSYEECRAESVGLYLCLHPQVLEIFGFEGADAEDVIYVNWLNMVRAGLLALEF
YTPEAFNWRQAHMQARFVILRVLLEAGEGLVTITPTTGSDGRPDARVRLDRSKIRSVGKPAL
ERFLRRLQVLKSTGDVAGGRALYEGYATVTDAPPECFLTLRDTVLLRKESRKLIVQPNTRLE
GSDVQLLEYEASAAGLIRSFSERFPEDGPELEEILTQLATADARFW
11110000000000000000000000000000000000000000000000000000000000000000000000000000000
00000000000000000000000000000000000000000000000000000000000000000000000000000000000
00000000000000000000000000000000000000000000000000000000011110000000000000000000000
00000000000000000000000000000000000000000000000000000000000000000000000000000000000
00000000000000000000000000000000000000000000000000000000000000000000000000000000000
00000000000000000000000000000000000000000000000000000000000000000000000000000000000
00000000000000000000000000000000000000000000000000000000000000000000000000000000000
00000000000000000000000000000000000000000000000000000000000000000000000000000000000
00000000000000000000000000000000000000000000000000000000000000
>Q9Y3Q0_3ff3_35_739
ETTTSVRYHQSIRWKLVSEMKAENIKSFLRSFTKLPHLAGTEQNFLLAKKIQTQWKKFGLDS
AKLVHYDVLLSYPNETNANYISIVDEHETEIFKTSYLEPPPDGYENVTNIVPPYNAFSAQGM
PEGDLVYVNYARTEDFFKLEREMGINCTGKIVIARYGKIFRGNKVKNAMLAGAIGIILYSDP
ADYFAPEVQPYPKGWNLPGTAAQRGNVLNLNGAGDPLTPGYPAKEYTFRLDVEEGVGIPR
IPVHPIGYNDAEILLRYLGGIAPPDKSWKGALNVSYSIGPGFTGSDSFRKVRMHVYNINKITR
IYNVVGTIRGSVEPDRYVILGGHRDSWVFGAIDPTSGVAVLQEIARSFGKLMSKGWRPRRTII
FASWDAEEFGLLGSTEWAEENVKILQERSIAYINSDSSIEGNYTLRVDCTPLLYQLVYKLTKEI
PSPDDGFESKSLYESWLEKDPSPENKNLPRINKLGSGSDFEAYFQRLGIASGRARYTKNKKT
DKYSSYPVYHTIYETFELVEKFYDPTFKKQLSVAQLRGALVYELVDSKIIPFNIQDYAEALKNY
AASIYNLSKKHDQQLTDHGVSFDSLFSAVKNFSEAASDFHKRLIQVDLNNPIAVRMMNDQ
LMLLERAFIDPLGLPGKLFYRHIIFAPSSHNKYAGESFPGIYDAIFDIENKANSRLAWKEVKK
HISIAAFTIQAAAGTLKEVL
11111111110000000000000000000000000000000000000000000000000000000000000000000000000
00000000000000111100000000000000000000000000000000000000000000000000000000000000000
00000000000000000000000000000000000000000000000000000000000000000000000000000000000
00000000000000000000000000000000000000000010000000000000000000000000000000000000000
00000000000000000000000000000000000000000000000000000000000000000000000000000000000
00000000000000000000000000000000000000000000000000000000000000000000000000000000000
00000000000000000000000000000000000000000000000000000000000000000000000000000000000
00000000000000000000000000000000000000000000000000000000000000000000000000000000000
00000000000000000000000000000000000000001
>O44249_3hhs_1_684
TDAKNNLLYFFDRPNEPCFMQKGEDKVVFEIPDHYYPDKYKSLSNTLSNRFGNEATKRIPIR
NITLPNLEVPMQLPYNDQFSLFVPKHRTMAAKLIDIFMGMRDVEDLQSVCSYCQLRINPY
MFNYCLSVAILHRPDTKGLSIPTFAETFPDKFMDSKVFLRAREVSNVVISGSRMPVNVPINYT
ANTTEPEQRVAYFREDIGINLHHWHWHLVYPFDSADRSIVNKDRRGELFYYMHQQIIGRYN
VERMCNGLPQVKPFSDFSAPIEEGYFPKLDSQVASRTWPPRFAGSVFRNLDRTVDQVKIDVR
KLFTWRDQFLEAIQKMAIKMPNGRELPLDEVTGIDMLGNLMESSIISPNRGYYGDLHNMG
HVFAAYTHDPDHRHLEQFGVMGDSATAMRDPFFYRWHRFVDDVFNIYKEKLTPYTNERL
DFPGVRVSSVGIEGARPNTLRTLWQQSTVELGRGLDFTPRGSVLARFTHLQHDEFQYVIEVN
NTTGGNLMGTVRIFMAPKVDDNGQPMSFNKQRRLMIELDKFSQALRPGTNTIRRRSVDSS
VTIPYERTFRNQSERPGDPGTAGAAEFDFCGCGWPHHMLIPKGTAQGYPVVLFVMISNWN
NDRIEQDLVGSCNDAASYCGIRDRKYPDKQAMGYPFDRKMANDAATLSDFLRPNMAVRD
CSIQFSDTTVERGQQG
10000000000000000000000000000000000000000000000000000000000000000000000000000000000
00000000000000000000000000000000000000000000000000000000000000000000000000000000000
00000000000000000000000000000000000000000000000000000000000000000000000000000000000

00000000000000000000000000000000000000000000000000000000000000000000000000000000
00000000000000000000000000000000000000000000000000000000000000000000000000000000
00000000000000000000000001000000000000000000000000000000000000000000000000000000
00000000000000000000000000000000000000000000000000000000011111111111111111100000
00000000000000000000000000000000001100000000000000000000000000000000000000000000
0000000000000011111
>Q46444_1kb0_31_707
TGPAAQAAAAVQRVDGDFIRANAARTPDWPTIGVDYAETRYSRLDQINAANVKDLGLAW
SYNLESTRGVEATPVVVDGIMYVSASWSVVHAIDTRTGNRIWTYDPQIDRSTGFKGCCDVV
NRGVALWKGKVYVGAWDGRLIALDAATGKEVWHQNTFEGQKGSLTITGAPRVFKGKVII
GKRGAEYGVRGYITAYDAETGERKWRWFSVPGDPSKPFEDESMKRAARTWDPSGKWWEA
GGGGTMWDSMTFDAELNTMYVGTGNGSPWSHKVRSPKGGDNLYLASIVALDPDTGKYK
WHYQETPGDNWDYTSTQPMILADIKIAGKPRKVILHAPKNGFFFVLDRTNGKFISAKNFVP
VNWASGYDKHGKPIGIAAARDGSKPQDAVPGPYGAHNWHPMSFNPQTGLVYLPAQNVP
VNLMDDKKWEFNQAGPGKPQSGTGWNTAKFFNAEPPKSKPFGRLLAWDPVAQKAAWSV
EHVSPWNGGTLTTAGNVVFQGTADGRLVAYHAATGEKLWEAPTGTGVVAAPSTYMVDGR
QYVSVAVGWGGVYGLAARATERQGPGTVYTFVVAGKARMPEFVAQRTGQLLQGVKYDPA
KVEAGTMLYVANCVFCHGVPGVDRGGNIPNLGYMDASYIENLPNFVFKGPAMVRGMPDF
TGKLSGDDVESLKAFIQGTADAIRPKP
00000000000000000000000000000000000000000000000000000000000000000000000000000000
00000000000000000000000000000000000000000000000000000000000000000000000000000000
00000000000000000000000000000000000000000000000000000000000000000000000000000000
00000000000000000000000000000000000000000000000000000000000000000000000000000000
00000000000000000000000000000000000000000000000000000000000000000000000000000000
00000000000000000000000000000000000000000000000000000000000000000000000000000000
00000000000000000000000000000000000000000000000000000000000000000000000011111000
00000000000000000000000000000000000000000000000000000000000000000000000000000000
000000000011
>P16278_3thc_23_676
LRNATQRMFEIDYSRDSFLKDGQPFRYISGSIHYSRVPRFYWKDRLLKMKMAGLNAIQTYV
PWNFHEPWPGQYQFSEDHDVEYFLRLAHELGLLVILRPGPYICAEWEMGGLPAWLLEKESI
LLRSSDPDYLAAVDKWLGVLLPKMKPLLYQNGGPVITVQVENEYGSYFACDFDYLRFLQKR
FRHHLGDDVVLFTTDGAHKTFLKCGALQGLYTTVDFGTGSNITDAFLSQRKCEPKGPLINS
EFYTGWLDHWGQPHSTIKTEAVASSLYDILARGASVNLYMFIGGTNFAYWNGANSPYAAQ
PTSYDYDAPLSEAGDLTEKYFALRNIIQKFEKVPEGPIPPSTPKFAYGKVTLEKLKTVGAALDI
LCPSGPIKSLYPLTFIQVKQHYGFVLYRTTLPQDCSNPAPLSSPLNGVHDRAYVAVDGIPQGV
LERNNVITLNITGKAGATLDLLVENMGRVNYGAYINDFKGLVSNLTLSSNILTDWTIFPLDT
EDAVRSHLGGWGHRDSGHHDEAWAHNSSNYTLPAFYMGNFSIPSGIPDLPQDTFIQFPGW
TKGQVWINGFNLGRYWPARGPQLTLFVPQHILMTSAPNTITVLELEWAPCSSDDPELCAVT
FVDRPVIGSSVTYDHPSKPVEKRLMPPPPQKNKDSWLDHV
11111000000000000000000000000000000000000000000000000000000000000000000000000000
00000000000000000000000000000000000000000000000000000000000000000000000000000000
00000000000000000000000000000000000000000000000000000000000000000000000000000000
00000000000000000000000000000000000000000000000000000000000000000000000000000000
00000000000000000000000000000000000000000000000000000000000000000000000000000000
00000000000000000000000000000000000000000000000000000000000000000000000000000000
00000000011111111111111000000000000000000000000000000000000000000000000000000000
00000000000000000000000000000000000000000011111111111111111111111111111
>P94127_2iwf_0_641
MESKEHKGLSRRALFSATAGSAILAGTVGPAALSLGAAGLATPARAATGADGSVAPGKLD
DYYGFWSSGQTGEMRILGIPSMRELMRVPVFNRCSATGWGQTNESIRIHQRTMTEKTKKQL

AANGKKIHDNGDLHHVHMSFTDGKYDGRYLFMNDKANTRVARVRCDVMKTDAILEIPN
AKGIHGMRPQKWPRSNYVFCNGEDEAPLVNDGSTMTDVATYVNIFTAVDADKWEVAWQ
VKVSGNLDNCDADYEGKWAFSTSYNSEMGMTLEEMTKSEMDHVVVFNIAEIEKAIKAGQ
YEEINGVKVVDGRKEAKSLFTRYIPIANNPHGCNMAPDRKHLCVAGKLSPTVTVLDVTKF
DALFYDNAEPRSAVVAEPELGLGPLHTAFDGRGNAYTSLFLDSQVVKWNIDEAIRAYAGEK
INPIKDKLDVQYQPGHLKTVMGETLDAANDWLVCLCKFSKDRFLNVGPLKPENDQLIDIS
GDKMVLVHDGPTFAEPHDAIAVSPSILPNIRSVWDRKDPLWAETRKQAEADEVDIDEWTE
AVIRDGNKVRVYMTSVAPSFSQPSFTVKEGDEVTVIVTNLDEIDDLTHGFTMGNHGVAMEV
GPQQTSSVTFVAANPGVYWYYCQWFCHALHMEMRGRMFVEPKGA
11111111111111111111111111111111111111111111111000000000000000000000000000000000
00000000000000000000000000000000000000000000000000000000000000000000000000000000
00000000000000000000000000000000000000000000000000000000000000000000000000000000
00000000000000000000000000000000000000000000000000000000000000000000000000000000
00000000000000000000000000000000000000000000000000000000000000000000000000000000
00000000000000000000000000000000000000000000000000000000000000000000000000000000
00000000000000000000000000000000000000000000000000000000000000000000000000000000
00000000000000000000000000000000000000000000000000000000111
>Q26966_1ms9_1_634
LAPGSSRVELFKRQSSKVPFEKDGKVTERVVHSFRLPALVNVDGVMVAIADARYETSNDNS
LIDTVAKYSVDDGETWETQIAIKNSRASSVSRVVDPTVIVKGNKLYVLVGSYNSSRSYWTSH
GDARDWDILLAVGEVTKSTAGGKITASIKWGSPVSLKEFFPAEMEGMHTNQFLGGAGVAIV
ASNGNLVYPVQVTNKKKQVFSKIFYSEDEGKTWKFGKGRSAFGCSEPVALEWEGKLIINTR
VDYRRRLVYESSDMGNSWLEAVGTLSRVWGPSPKSNQPGSQSSFTAVTIEGMRVMLFTHPL
NFKGRWLRDRLNLWLTDNQRIYNVGQVSIGDENSAYSSVLYKDDKLYCLHEINSNEVYSLV
FARLVGELRIIKSVLQSWKNWDSHLSSICTPADPAASSSERGCGPAVTTVGLVGFLSHSATKT
EWEDAYRCVNASTANAERVPNGLKFAGVGGGALWPVSQQGQNQRYRFANHAFTVVASV
TIHEVPSVASPLLGASLDSSGGKKLLGLSYDERHQWQPIYGSTPVTPTGSWEMGKRYHVVLT
MANKIGSEYIDGEPLEGSGQTVVPDERTPDISHFYVGGYKRSDMPTISHVTVNNVLLYNRQ
LNAEEIRTLFLSQDLIGTEAHMD
10000000000000000000000000000000000000000000000000000000000000000000000000000000
00000000000000000000000000000000000000000000000000000000000000000000000000000000
00000000000000000000000000000000000000000000000000000000000000000000000000000000
00000000000000000000000000000000000000000000000000000000000000000000000000000000
00000000000000000000000000000000000000000000000000000000000000001111111110000000
00000000000000000000000000000000000000000000000000000000000000000000000000000000
00000000000000000000000000000000000000000000000000000000000000000000000000000000
000000000000000000000000000000000000000000000000001
>Q9RBY1_2z8z_0_616
MGVYDYKNFGTADSKALFSDAMAITLYSYHNLDNGFAAGYQHNGFGLGLPATLVTALLG
GTDSQGVIPGIPWNPDSEKLALDAVKKAGWTPITASQLGYDGKTDARGTFFGEKAGYTTA
QVEILGKYDAQGHLTEIGIAFRGTSGPRENLILDSIGDVINDLLAAFGPKDYAKNYVGEAFG
NLLNDVVAFAKANGLSGKDVLVSGHSLGGLAVNSMADLSGGKWGGFFADSNYIAYASPT
QSSTDKVLNVGYENDPVFRALDGSTFTGASVGVHDAPKESATDNIVSFNDHYASTAWNLL
PFSILNIPTWISHLPTAYGDGMNRIIESKFYDLTSKDSTIIVANLSDPARANTWVQDLNRNAE
THKGSTFIIGSDSNDLIQGGSGNDYLEGRAGNDTFRDGGGYNVILGGAGNNTLDLQKSVN
TFDFANDGAGNLYVRDANGGISITRDIGSIVTKEPGFLWGLFKDDVTHSVTASGLKVGSNV
TQYDASVKGTNGADTLKAHAGGDWLFGLDGNDHLIGGVGNDVFVGGAGNDLMESGGG
ADTFLFNGAFGQDRVVGFTSNDKLVFLGVQGVLPNDDFRAHASMVGQDTVLKFGGDSVT
LVGVALNSLSADGIVIA
10000000000000000000000000000000000000000000000000000000000000000000000000000000
00000000000000000000000000000000000000000000000000000000000000000000000000000000

00000000000000000000000000000000000000000000000000000000000000000000000000000000000
00000000000000000000000000000000000000000000000000000000000000000000000000000000000
00000000000000000000000000000000000000000000000000000000000000000000000000000000000
00000000000000000000000000000000000000000000000000000000000000000000000000000000000
00000000000000000000000000000000000000000000000000000000000000000000000000000000000
000000000000000000000000000000000000
>Q9L9D7_3pui_0_573
MVDGNYSVASNVMVPMRDGVRLAVDLYRPDADGPVPVLLVRNPYDKFDVFAWSTQSTN
WLEFVRDGYAVVIQDTRGLFASEGEFVPHVDDEADAEDTLSWILEQAWCDGNVGMFGVS
YLGVTQWQAAVSGVGGLKAIAPSMASADLYRAPWYGPGGALSVEALLGWSALIGTGLITS
RSDARPEDAADFVQLAAILNDVAGAASVTPLAEQPLLGRLIPWVIDQVVDHPDNDESWQS
ISLFERLGGLATPALITAGWYDGFVGESLRTFVAVKDNADARLVVGPWSHSNLTGRNADRK
FGIAATYPIQEATTMHKAFFDRHLRGETDALAGVPKVRLFVMGIDEWRDETDWPLPDTAY
TPFYLGGSGAANTSTGGGTLSTSISGTESADTYLYDPADPVPSLGGTLLFHNGDNGPADQRP
IHDRDDVLCYSTEVLTDPVEVTGTVSARLFVSSSAVDTDFTAKLVDVFPDGRAIALCDGIVR
MRYRETLVNPTLIEAGEIYEVAIDMLATSNVFLPGHRIMVQVSSSNFPKYDRNSNTGGVIAR
EQLEEMCTAVNRIHRGPEHPSHIVLPIIKR
11000000000000000000000000000000000000000000000000000000000000000000000000000000000
00000000000000000000000000000000000000000000000000000000000000000000000000000000000
00000000000000000000000000000000000000000000000000000000000000000000000000000000000
00000000000000000000000000000000000000000000000000000000000000000000000000000000000
00000000000000000000000000000000000000000000000000000000000000000000000000000000000
00000000000000000000000000000000000000000000000000000000000000000000000000000000000
0000000000000000000000000000000000000000000000000000000000000000000000000000
>P37700_1kfg_35_648
AGTYNYGEALQKSIMFYEFQRSGDLPADKRDNWRDDSGMKDGSDVGVDLTGGWYDAGD
HVKFNLPMSYTSAMLAWSLYEDKDAYDKSGQTKYIMDGIKWANDYFIKCNPTPGVYYYQV
GDGGKDHSWWGPAEVMQMERPSFKVDASKPGSAVCASTAASLASAAVVFKSSDPTYAEK
CISHAKNLFDMADKAKSDAGYTAASGYYSSSSFYDDLSWAAVWLYLATNDSTYLDKAESY
VPNWGKEQQTDIIAYKWGQCWDDVHYGAELLLAKLTNKQLYKDSIEMNLDFWTTGVNG
TRVSYTPKGLAWLFQWGSLRHATTQAFLAGVYAEWEGCTPSKVSVYKDFLKSQIDYALGST
GRSFVVGYGVNPPQHPHHRTAHGSWTDQMTSPTYHRHTIYGALVGGPDNADGYTDEINN
YVNNEIACDYNAGFTGALAKMYKHSGGDPIPNFKAIEKITNDEVIIKAGLNSTGPNYTEIK
AVVYNQTGWPARVTDKISFKYFMDLSEIVAAGIDPLSLVTSSNYSEGKNTKVSGVLPWDVS
NNVYYYVNVDLTGENIYPGGQSACRREVQFRIAAPQGTTYWNPKNDFSYDGLPTTSTVNTV
TNIPVYDNGVKVFGNEP
10000000000000000000000000000000000000000000000000000000000000000000000000000000000
00000000000000000000000000000000000000000000000000000000000000000000000000000000000
00000000000000000000000000000000000000000000000000000000000000000000000000000000000
00000000000000000000000000000000000000000000000000000000000000000000000000000000000
00000000000000000000000000000000000000000000000000000000000000000000000000000000000
00000000000000000000000000000000000000000000000000000000000000000000000000000000000
00000000000000000000000000000000000000000000000000000000000000000000000000000000000
000000000000000000000000000000000
>O31527_2zuy_0_611
MKPKKRQMEYLTRGLIAVQTEQGVFVSWRFLGTDHETTAFHLYRDGKRITRDPIAESTNFL
DQNGTADSVYQVAAVNKGREEKLSKKARVWQENVLEVPLAKPEGGVTPDGKPYTYSAND
ASVGDIDGDGEYEMILKWDPSNSKDNAHDGYTGEVLIDAYKLDGTFLWRINLGRNIRAGA
HYTQFMVYDLDGDGKAEIAMKTADGTTDGKGHIIGDEQADFRNEQGRILSGPEYLTVFKG
ETGEALTTVEYEPPRGKLEDWGDGYGNRMDRFLAGTAYLDGERPSLVMARGYYTRTVLVA
YDFRNGRLKKRWVFDSNQPGHEAYAGQGNHSLSVADVDGDGKDEIIYGAMAVDHDGTG

LYSTGLGHGDAMHVGDLDPSRKGLEVFQVHEDATKPYGLSLRDAGTGEILWGVHAGTDV
GRGMAAHIDPSYKGSLVWGIDPPGNDGMSYGLFTSKGEKISDKAPSSANFAIWWDGDLVR
ELLDHDWDGTIGRPKIEKWDAENGCLKTIFQPAGVLSNNGTKGNPVLQANLFGDWREEVI
WRTEDSSALRIYTTTHLTRHCFYTLMHDPVYRLGIAWQNTAYNQPPHTSFYLGTGMKKPPK
PALYIAGSKAEAPL
11000000000000000000000000000000000000000000000000000000000000000000000000000000000
00000000000000000000000000000000000000000000000000000000000000000000000000000000000
00000000000000000000000000000000000000000000000000000000000000000000000000000000000
00000000000000000000000000000000000000000000000000000000000000000000000000000000000
00000000000000000000000000000000000000000000000000000000000000000000000000000000000
00000000000000000000000000000000000000000000000000000000000000000000000000000000000
00000000000000000000000000000000000000000000000000000000000000000000000000000000000
0000000000000000000000000111111
>P16027_1w6s_27_625
NDKLVELSKSDDNWVMPGKNYDSNNFSDLKQINKGNVKQLRPAWTFSTGLLNGHEGAPL
VVDGKMYIHTSFPNNTFALGLDDPGTILWQDKPKQNPAARAVACCDLVNRGLAYWPGDG
KTPALILKTQLDGNVAALNAETGETVWKVENSDIKVGSTLTIAPYVVKDKVIIGSSGAELGV
RGYLTAYDVKTGEQVWRAYATGPDKDLLLASDFNIKNPHYGQKGLGTGTWEGDAWKIGG
GTNWGWYAYDPGTNLIYFGTGNPAPWNETMRPGDNKWTMTIFGRDADTGEAKFGYQKT
PHDEWDYAGVNVMMLSEQKDKDGKARKLLTHPDRNGIVYTLDRTDGALVSANKLDDTV
NVFKSVDLKTGQPVRDPEYGTRMDHLAKDICPSAMGYHNQGHDSYDPKRELFFMGINHI
CMDWEPFMLPYRAGQFFVGATLNMYPGPKGDRQNYEGLGQIKAYNAITGDYKWEKMER
FAVWGGTMATAGDLVFYGTLDGYLKARDSDTGDLLWKFKIPSGAIGYPMTYTHKGTQYVA
IYYGVGGWPGVGLVFDLADPTAGLGAVGAFKKLANYTQMGGGVVVFSLDGKGPYDDPN
VGEWKSAAK
00000000000000000000000000000000000000000000000000000000000000000000000000000000000
00000000000000000000000000000000000000000000000000000000000000000000000000000000000
00000000000000000000000000000000000000000000000000000000000000000000000000000000000
00000000000000000000000000000000000000000000000000000000000000000000000000000000000
00000000000000000000000000000000000000000000000000000000000000000000000000000000000
00000000000000000000000000000000000000000000000000000000000000000000000000000000000
00000000000000000000000000000000000000000000000000000000000000000000000000000000000
000000000000000011
>Q5KWC1_3px6_286_877
MESPSSEEEKPLAKMAFTLADRVTEEMLADKAALVVEVVEENYHDAPIVGIAVVNEHGRFF
LRPETALADPQFVAWLGDETKKKSMFDSKRAAVALKWKGIELCGVSFDLLLAAYLLDPAQ
GVDDVAAAAKMKQYEAVRPDEAVYGKGAKRAVPDEPVLAEHLVRKAAAIWELERPFLDE
LRRNEQDRLLVELEQPLSSILAEMEFAGVKVDTKRLEQMGKELAEQLGTVEQRIYELAGQE
FNINSPKQLGVILFEKLQLPVLKKTKTGYSTSADVLEKLAPYHEIVENILHYRQLGKLQSTYI
EGLLKVVRPDTKKVHTIFNQALTQTGRLSSTEPNLQNIPIRLEEGRKIRQAFVPSESDWLIFA
ADYSQIELRVLAHIAEDDNLMEAFRRDLDIHTKTAMDIFQVSEDEVTPNMRRQAKAVNFGI
VYGISDYGLAQNLNISRKEAAEFIERYFESFPGVKRYMENIVQEAKQKGYVTTLLHRRRYLP
DITSRNFNVRSFAERMAMNTPIQGSAADIIKKAMIDLNARLKEERLQARLLLQVHDELILEA
PKEEMERLCRLVPEVMEQAVTLRVPLKVDYHYGSTWYDAK
11111111111100000000000000000000000000000000000000000000000000000000000000000000000
00000000000000000000000000000000000000000000000000000000000000000000000000000000000
00000000000000000000000000000000000000000000000000000000000000000000000000000000000
00000000000000000000000000000000000000000000000000000000000000000000000000000000000
00000000000000000000000000000000000000000000000000000000000000000000000000000000000
00000000000000000000000000000000000000000000000000000000000000000000000000000000000
00000000000000000000000000000000000000000000000000000000000000000000000000000000000

00000000000
>A5LBQ0_2wmi_421_1004
SEVDKRREINNEHPLLMMPLYANGEEFNQGKYTFWGGDTLTGKWENIPDDLKPYTVIQLH
PDDLPKRDGAARDFYEHMLEEAAKYVNPKTGKNEPIPVILTVYTAGNMPYYTSAHWLSTS
WIDKMYQKYPNLHGIFSTENYWIWANDIENKAADYLKVSAKNGGYFIWAEQNNGSAIEK
AFGKNGKIAFQKSVDKYWKNLIFMFKNTPAAEGNDSTTESYMKGLWLSNHTYQWGGLM
DTWKWYETGKWKLFASGNIGKSQGDRQWLTEPESMLGEEALGVYLNGGVVYNFEHPAYT
YGVNNKESLLFSEVIKEFFRYVIAHPAPSKEKVLEDTKVFIHGDYSNKGNGKFFVNVNTDRE
QTPLYMTGRYNVIPAIPGVLKTDKLKESVSSSRIQIKEITSPEFSSTQARKEYLNKLYPMNYEG
DIFAQKLDNRWFVYNYKVNENVKQTGKLKFNSLEMNVEFEPHTYGIFERISNGLKVNLNN
FRTNKDSLWSNAQDANQAKKLPQLTKKGAIKWIEEHYIKDTQFGEKRVTKIVLRGIDKLPTI
HSLSGTNNSYDQPSLNFDQKNHMVTITINSNGNLEFELHF
11000000000000000000000000000000000000000000000000000000000000000000000000000000
00000000000000000000000000000000000000000000000000000000000000000000000000000000
00000000000000000000000000000000000000000000000000000000000000000000000000000000
00000000000000000000000000000000000000000000000000000000000000000000000000000000
00000000000000000000000000000000000000000000000111111111100000000000000000000000
00000000000000000000000000000000000000000000000000000000000000000000000000000000
00000000000000000000000000000000000000000000000000000000000000000000000000000000
000
>P07254_1x6l_0_562
MRKFNKPLLALLIGSTLCSAAQAAAPGKPTIAWGNTKFAIVEVDQAATAYNNLVKVKNAA
DVSVSWNLWNGDAGTTAKILLNGKEAWSGPSTGSSGTANFKVNKGGRYQMQVALCNAD
GCTASDATEIVVADTDGSHLAPLKEPLLEKNKPYKQNSGKVVGSYFVEWGVYGRNFTVDKI
PAQNLTHLLYGFIPICGGNGINDSLKEIEGSFQALQRSCQGREDFKVSIHDPFAALQKAQKG
VTAWDDPYKGNFGQLMALKQAHPDLKILPSIGGWTLSDPFFFMGDKVKRDRFVGSVKEFL
QTWKFFDGVDIDWEFPGGKGANPNLGSPQDGETYVLLMKELRAMLDQLSAETGRKYELT
SAISAGKDKIDKVAYNVAQNSMDHIFLMSYDFYGPFDLKNLGHQTALNAPAWKPDTAYTT
VNGVNALLAQGVKPGKVVVGTAMYGRGWTGVNGYQNNIPFTGTATGPVKGTWKNGIV
DYRQIAGQFMSGEWQYTYDATAEAPYVFKPSTGDLITFDDARSVQAKGKYVLDKQLGGLF
SWEIDADNGDILNSMNASLGNSAGVQ
11111111111111111111110000000000000000000000000000000000000000000000000000000000
00000000000000000000000000000000000000000000000000000000000000000000000000000000
00000000000000000000000000000000000000000000000000000000000000000000000000000000
00000000000000000000000000000000000000000000000000000000000000000000000000000000
00000000000000000000000000000000000000000000000000000000000000000000000000000000
00000000000000000000000000000000000000000000000000000000000000000000000000000000
00000000000000000000000000000000000000000000000000000000000001
>P11558_3pot_0_549
MADKLFINALKKKFEESPEEKKTTFYTLGGWKQSERKTEFVNAGKEVAAKRGIPQYNPDIG
TPLGQRVLMPYQVSTTDTYVEGDDLHFVNNAAMQQMWDDIRRTVIVGLNHAHAVIEKR
LGKEVTPETITHYLETVNHAMPGAAVVQEHMVETHPALVADSYVKVFTGNDEIADEIDPAF
VIDINKQFPEDQAETLKAEVGDGIWQVVRIPTIVSRTCDGATTSRWSAMQIGMSMISAYKQ
AAGEAATGDFAYAAKHAEVIHMGTYLPVRRARGENEPGGVPFGYLADICQSSRVNYEDPV
RVSLDVVATGAMLYDQIWLGSYMSGGVGFTQYATAAYTDNILDDFTYFGKEYVEDKYGLCE
APNNMDTVLDVATEVTFYGLEQYEEYPALLEDQFGGSQRAAVVAAAAGCSTAFATGNAQT
GLSGWYLSMYLHKEQHSRLGFYGYDLQDQCGASNVFSIRGDEGLPLELRGPNYPNYAMN
VGHQGEYAGISQAPHAARGDAFVFNPLVKIAFADDNLVFDFTNVRGEFAKGALREFEPAG
ERALITPAK
10000000000000000000000000000000000000000000000000000000000000000000000000000000
00000000000000000000000000000000000000000000000000000000000000000000000000000000

00000000000000000000000000000000000000000000000000000000000000000000000000000000000
00000000000000000000000000000000000000000000000000000000000000000000000000000000000
00000000000000000000000000000000000000000000000000000000000000000000000000000000000
00000000000000000000000000000000000000000000000000000000000000000000000000000000000
0000000000000000000000000000000000000000000000000001
>Q5JDJ7_3n98_0_561
MKGYLTFVLHTHIPYVRKHGKWPFGEEWVFEAISETYIPLLMEFERLRDSGVKFGIVINVTP
VLAEQLTDEYMKKAFEEYMERKLKAMEEDLKSGKYDEKAVSYMLNYFRKVYDYWKAING
DIIGKLRELQDQGYVEVITSAATHGYLPLLGRDEAIRAQIANGVATYEKHFGMKPKGIWLPE
CAYRPAGEWELPGGRKVKRQGIEKFLEEFGLRYFFVESRLIDEGPASNVYGEVLIADTEKTTL
RPYWIKGSNVAVFARNRETGHQVWSAHYGYPGDFWYREFHKKAPKSGGQYWRITSKEVG
LGEKEFYDPDKAMERVEEHARHFVSLVERLLREHEEKFGEKGIIVAPYDTELFGHWWFEGV
KWLGRVLELLYQRGVETPTLSRFLEEYSGEKHEIELPEGSWGANSDHSTWWNEETEWTWP
HIYRAEDRMVAIVSRFRGRDELTNRVIEQLARELLILEASDWQFLITTGQAKEYAKRRVLIHS
RDFHRLANELVRYVKIGEFDVKLLEELEERDNPFRPVVVGPYVSENPPELEEYVEPPEVPPEK
EETEEKPKVL
00000000000000000000000000000000000000000000000000000000000000000000000000000000000
00000000000000000000000000000000000000000000000000000000000000000000000000000000000
00000000000000000000000000000000000000000000000000000000000000000000000000000000000
00000000000000000000000000000000000000000000000000000000000000000000000000000000000
00000000000000000000000000000000000000000000000000000000000000000000000000000000000
00000000000000000000000000000000000000000000000000000000000000000000000000000000000
0000000000000000000000000000000000000000000000000000111111111111
>Q7SID9_2i0k_0_560
STGPVAPLPTPPNFPNDIALFQQAYQNWSKEIMLDATWVCSPKTPQDVVRLANWAHEHD
YKIRPRGAMHGWTPLTVEKGANVEKVILADTMTHLNGITVNTGGPVATVTAGAGASIEAI
VTELQKHDLGWANLPAPGVLSIGGALAVNAHGAALPAVGQTTLPGHTYGSLSNLVTELTA
VVWNGTTYALETYQRNDPRITPLLTNLGRCFLTSVTMQAGPNFRQRCQSYTDIPWRELFAP
KGADGRTFEKFVAESGGAEAIWYPFTEKPWMKVWTVSPTKPDSSNEVGSLGSAGSLVGKPP
QAREVSGPYNYIFSDNLPEPITDMIGAINAGNPGIAPLFGPAMYEITKLGLAATNANDIWG
WSKDVQFYIKATTLRLTEGGGAVVTSRANIATVINDFTEWFHERIEFYRAKGEFPLNGPVEIR
CCGLDQAADVKVPSVGPPTISATRPRPDHPDWDVAIWLNVLGVPGTPGMFEFYREMEQW
MRSHYNNDDATFRPEWSKGWAFGPDPYTDNDIVTNKMRATYIEGVPTTENWDTARARYN
QIDPHRVFTNGFMDKLLP
11111000000000000000000000000000000000000000000000000000000000000000000000000000000
00000000000000000000000000000000000000000000000000000000000000000000000000000000000
00000000000000000000000000000000000000000000000000000000000000000000000000000000000
00000000000000000000000000001111111111111111111100000000000000000000000000000000000
00000000000000000000000000000000000000000000000000000000000000000000000000000000000
00000000000000000000000000000000000000000000000000000000000000000000000000000000000
000000000000000000000000000000000000000000000000000000000000000
>Q5SIP1_2z1a_0_551
MKRREVLQAGMALGGLAGLGRALAQGGFTLTLVHTNDTHAHLEPVELTLSGEKTPVGGV
ARRVALFDRVWARAKNPLFLDAGDVFQGTLYFNQYRGLADRYFMHRLRYRAMALGNHE
FDLGPGPLADFLKGARFKVVSANVDASREPRLKGLFAPYAVVVVGGERVGIIGLTTPDTREIS
NPGPTVAFLDPYESAQKAVYELLAKGVNKIVVLSHLGYGEDLKLARRLVGVQVIVGGHSH
TLLGSFPHKELSPAGPYPTVVKNPEGKDVLVVQAWEWGKVVGLLEVTFDAKGELLAYKGE
ALLMTPEAAPEDFFAKEALLAYAQPVMALMQQVIAEAKVDLVGERAVVRRRESNLGNLIT
DGMLWKTRNAGTQIALQNGGGIRASIPKGPITVGKVYEVLPFGNTLVVMDLKGKEILAALE
NGVSQWENTAGRFLQVSGLRYAFDLSRPAGSRVVRVEVKTEKGYVPLDLEATYRVVVNNFI
ANGGDGFTVLKEAQGYRVDTGFSDAESFMDYLKELKVVEAGLEGRIEVLNEPKGERPAYFA

YRVPGLVGV
11111111111111111111111111000000000000000000000000000000000000000000000000000000
00000000000000000000000000000000000000000000000000000000000000000000000000000000
00000000000000000000000000000000000000000000000000000000000000000000000000000000
00000000000000000000000000000000000000000000000000000000000000000000000000000000
00000000000000000000000000000000000000000000000000000000000000000000000000000000
00000000000000000000000000000000000000000000000000000000000000000000000000000000
0000000000000000000000000000000000011111111111111111
>B6A876_3oa5_0_541
MEKEEKSNLIYDKDPGYVWDNKNECEGAAEETYQELNYEPSISADKLTWTPTRLAKTVFN
TYEDDDDFNVLCYFTDWSQYDPRIINKEIRDTGGRSADILRLNTPDGRPFKRLIYSFGGLIGD
KKYSADGNASIAVRLGVATDPDDAIANHKGKTIPVDPDGAVLASINCGFTKWEAGDANER
YNQEKAKGLLGGFRLLHEADKELEFSLSIGGWSMSGLFSEIAKDEILRTNFVEGIKDFFQRFP
MFSHLDIDWEYPGSIGAGNPNSPDDGANFAILIQQITDAKISNLKGISIASSADPAKIDAANI
PALMDAGVTGINLMTYDFFTLGDGKLSHHTNIYRDPSDVYSKYSIDDAVTHLIDEKKVDPK
AIFIGYAGYTRNAKNATITTSIPSEEALKGTYTDANQTLGSFEYSVLEWTDIICHYMDFEKGE
GRNGYKLVHDKVAKADYLYSEATKVFISLDTPRSVRDKGRYVKDKGLGGLFIWSGDQDNGI
LTNAAHEGLKRRIKNKVIDMTPFYLDSDEELPTYTEPAEPQCEACNIK
11111000000000000000000000000000000000000000000000000000000000000000000000000000
00000000000000000000000000000000000000000000000000000000000000000000000000000000
00000000000000000000000000000000000000000000000000000000000000000000000000000000
00000000000000000000000000000000000000000000000000000000000000000000000000000000
00000000000000000000000000000000000000000000000000000000000000000000000000000000
00000000000000000000000000000000000000000000000000000000000000000000000000000000
000000000000000000000111111111111111111111
>A6VKV4_1ylh_0_537
MTDLNKLVKELNDLGLTDVKEIVYNPSYEQLFEEETKPGLEGFDKGTLTTLGAVAVDTGIFT
GRSPKDKYIVCDETTKDTVWWNSEAAKNDNKPMTQETWKSLRELVAKQLSGKRLFVVEG
YCGASEKHRIGVRMVTEVAWQAHFVKNMFIRPTDEELKNFKADFTVLNGAKCTNPNWKE
QGLNSENFVAFNITEGIQLIGGTWYGGEMKKGMFSMMNYFLPLKGVASMHCSANVGKDG
DVAIFFGLSGTGKTTLSTDPKRQLIGDDEHGWDESGVFNFEGGCYAKTINLSQENEPDIYGA
IRRDALLENVVVRADGSVDFDDGSKTENTRVSYPIYHIDNIVRPVSKAGHATKVIFLTADAF
GVLPPVSKLTPEQTEYYFLSGFTAKLAGTERGVTEPTPTFSACFGAAFLSLHPIQYADVLVER
MKASGAEAYLVNTGWNGTGKRISIKDTRGIIDAILDGSIEKAEMGELPIFNLAIPKALPGVDP
AILDPRDTYADKAQWQVKAEDLANRFVKNFVKYTANPEAAKLVGAGPKA
11000000000000000000000000000000000000000000000000000000000000000000000000000000
00000000000000000000000000000000000000000000000000000000000000000000000000000000
00000000000000000000000000000000000000000000000000000000000000000000000000000000
00000000000000000000000000000000000000000000000000000000000000000000000000000000
00000000000000000000000000000000000000000000000000000111111111100000000000000000
00000000000000000000000000000000000000000000000000000000000000000000000000000000
000000000000000000000000000000000000001
>Q9T0N8_1w1q_0_533
MAVVYYLLLAGLIACSHALAAGTPALGDDRGRPWPASLAALALDGKLRTDSNATAAAST
DFGNITSALPAAVLYPSSTGDLVALLSAANSTPGWPYTIAFRGRGHSLMGQAFAPGGVVVN
MASLGDAAAPPRINVSADGRYVDAGGEQVWIDVLRASLARGVAPRSWNDYLYLTVGGTL
SNAGISGQAFRHGPQISNVLEMDVITGHGEMVTCSKQLNADLFDAVLGGLGQFGVITRARI
AVEPAPARARWVRFVYTDFAAFSADQERLTAPRPGGGGASFGPMSYVEGSVFVNQSLATDL
ANTGFFTDADVARIVALAGERNATTVYSIEATLNYDNATAAAAAVDQELASVLGTLSYVEG
FAFQRDVAYAAFLDRVHGEEVALNKLGLWRVPHPWLNMFVPRSRIADFDRGVFKGILQGT
DIVGPLIVYPLNKSMWDDGMSAATPSEDVFYAVSLLFSSVAPNDLARLQEQNRRILRFCDLA

GIQYKTYLARHTDRSDWVRHFGAAKWNRFVEMKNKYDPKRLLSPGQDIFN
11111111111111111111111111111111111111100000000000000000000000000000000000000000
00000000000000000000000000000000000000000000000000000000000000000000000000000000
00000000000000000000000000000000000000000000000000000000000000000000000000000000
00000000000000000000000011111100000000000000000000000000000000000000000000000000
00001111111000000000000000000000000000000000000000000000000000000000000000000000
00000000000000000000000000000000000000000000111000000000000000000000000000000000
00000000000000000000000000000000000
>B0Q556_3cux_1_528
STQTSRVTLVGEMLPAYNEILTPEALSFLKELHENFNERRIELLQKRMKKQQKIDAGEFPKFL
EETKRIREADWTIAKLPKDLEDRRVEITGPVDRKMVINALNSGAHLFMADFEDSNSPTWEN
AIEGQINLRDAVKGTISHKNENGKEYRLNSKTAVLIVRPRGWHLEEKHMQVDGKNMSGSL
VDFGLYFFHNAKALLEKGSGPYFYLPKMESYLEARLWNDVFVFAQKYIGIPNGTIKATVLLE
TIHASFEMDEILYELKDHSAGLNCGRWDYIFSFLKAFRNHNEFLLPDRAQVTMTAPFMRAY
SLKVIQTCHRRNAPAIGGMAAQIPIKNNPEANEAAFEKVRADKEREALDGHDGTWVAHP
GLVPVAMEVFNHIMKTPNQIFRKREEIHVTEKDLLEVPVGTITEEGLRMNISVGIQYIASWLS
GRGAAPIYNLMEDAATAEISRAQVWQWIRHEGGKLNDGRNITLELMEELKEEELAKIEREI
GKEAFKKGRFQEATTLFTNLVRNDEFVPFLTLPGYEIL
11111000000001100000000000000000000000000000000000000000000000000000000000000000
00000000000000000000000000000000000000000000000000000000000000000000000000000000
00000000000000000000000000000000000000000000000000000000000000000000000000000000
00000000000000000000000000000000000000000000000000000000000000000000000011111111
11111111110000000000000000000000000000000000000000000000000000000000000000000000
00000000000000000000000000000000000000000000000000000000000000000000000000001000
00000000000000000000000000000
>Q65XK0_3fr7_53_577
MVAAPPAVGAAMPSLDFDTSVFNKEKVSLAGHEEYIVRGGRNLFPLLPEAFKGIKQIGVIG
WGSQGPAQAQNLRDSLAEAKSDIVVKIGLRKGSKSFDEARAAGFTEESGTLGDIWETVSGS
DLVLLLISDAAQADNYEKIFSHMKPNSILGLSHGFLLGHLQSAGLDFPKNISVIAVCPKGMG
PSVRRLYVQGKEINGAGINSSFAVHQDVDGRATDVALGWSVALGSPFTFATTLEQEYKSDIF
GERGILLGAVHGIVEALFRRYTEQGMDEEMAYKNTVEGITGIISKTISKKGMLEVYNSLTEEG
KKEFNKAYSASFYPCMDILYECYEDVASGSEIRSVVLAGRRFYEKEGLPAFPMGNIDQTRMW
KVGEKVRSTRPENDLGPLHPFTAGVYVALMMAQIEVLRKKGHSYSEIINESVIESVDSLNPF
MHARGVAFMVDNCSTTARLGSRKWAPRFDYILTQQAFVTVDKDAPINQDLISNFMSDPVH
GAIEVCAELRPTVDISVPANADFVRPELRQSS
11111111111111000000000000000000000000000000000000000000000000000000000000000000
00000000000000000000000000000000000000000000000000000000000000000000000000000000
00000000000000000000000000000000000000000000000000000000000000000000000000000000
00000000000000000000000000000000000000000000000000000000000000000000000000000000
00000000000000000000000000000000000000000000000000000000000000000000000000000000
00000000000000000000000000000000000000000000000000000000000000000000000000000000
00000000000000000000000000000000000000000000000000000000000000000000000000000000
000000000001111111111111111
>P00898_1i1q_0_519
MQTPKPTLELLTCDAAYRENPTALFHQVCGDRPATLLLESADIDSKDDLKSLLLVDSALRIT
ALGDTVTIQALSDNGASLLPLLDTALPAGVENDVLPAGRVLRFPPVSPLLDEDARLCSLSVF
DAFRLLQGVVNIPTQEREAMFFGGLFAYDLVAGFEALPHLEAGNNCPDYCFYLAETLMVID
HQKKSTRIQASLFTASDREKQRLNARLAYLSQQLTQPAPPLPVTPVPDMRCECNQSDDAFG
AVVRQLQKAIRAGEIFQVVPSRRFSLPCPSPLAAYYVLKKSNPSPYMFFMQDNDFTLFGASP
ESSLKYDAASRQIEIYPIAGTRPRGRRADGTLDRDLDSRIELDMRTDHKELSEHLMLVDLAR
NDLARICTPGSRYVADLTKVDRYSYVMHLVSRVVGELRHDLDALHAYRACMNMGTLSGA
PKVRAMQLIADAEGQRRGSYGGAVGYFTAHGDLDTCIVIRSALVENGIATVQAGAGIVLDS

VPQSEADETRNKARAVLRAIATAHHAQETF
11110000000000000000000000000000000000000000000000000000000000000000000000000000000
00000000000000000000000000000000000000000000000000000000000000000000000000000000000
00000000000000000000000000000000000000000000000000000000000000000000000000000000000
00000000000000000000000000000000000000000000000000000000000000000000000000000000000
00000000000000000000000000000000000000000000000000000000000000000000000000000000000
00000000000000000000000000000000000000000000000000000000000000000000000000000000000
0000000000000000001111
>P42061_1xoc_24_542
SGSKSSNSSAKKSAGKPQQGGDLVVGSIGEPTLFNSLYSTDDASTDIENMLYSFLTKTDEKLN
VKLSLAESIKELDGGLAYDVKIKKGVKFHDGKELTADDVVFTYSVPLSKDYKGERGSTYEML
KSVEKKGDYEVLFKLKYKDGNFYNNALDSTAILPKHILGNVPIADLEENEFNRKKPIGSGPF
KFKEWKQGQYIKLEANDDYFEGRPYLDTVTYKVIPDANAAVAQLQAGDINFFNVPATDYK
TAEKFNNLKIVTDLALSYVYIGWNEKNELFKDKKVRQALTTALDRESIVSQVLDGDGEVAYI
PESPLSWNYPKDIDVPKFEYNEKKAKQMLAEAGWKDTNGDGILDKDGKKFSFTLKTNQG
NKVREDIAVVVQEQLKKIGIEVKTQIVEWSALVEQMNPPNWDFDAMVMGWSLSTFPDQY
DIFHSSQIKKGLNYVWYKNAEADKLMKDAKSISDRKQYSKEYEQIYQKIAEDQPYTFLYYP
NNHMAMPENLEGYKYHPKRDLYNIEKWWLAK
11111111111111100000000000000000000000000000000000000000000000000000000000000000000
00000000000000000000000000000000000000000000000000000000000000000000000000000000000
00000000000000000000000000000000000000000000000000000000000000000000000000000000000
00000000000000000000000000000000000000000000000000000000000000000000000000000000000
00000000000000000000000000000000000000000000000000000000000000000000000000000000000
00000000000000000000000000000000000000000000000000000000000000000000000000000000000
000000000000000000000
>O94220_3sc7_0_515
MLNPKVAYMVWMTCLGLTLPSQAQSNDYRPSYHFTPDQYWMNEPNGLIKIGSTWHLFFQ
HNPTANVWGNICWGHATSTDLMHWAHKPTAIADENGVEAFTGTAYYDPNNTSGLGDSA
NPPYLAWFTGYTTSSQTQDQRLAFSVDNGATWTKFQGNPIISTSQEAPHDITGGLESRDPKV
FFHRQSGNWIMVLAHGGQDKLSFWTSADTINWTWQSDLKSTSINGLSSDITGWEVPDMFE
LPVEGTEETTWVVMMTPAEGSPAGGNGVLAITGSFDGKSFTADPVDASTMWLDNGRDFD
GALSWVNVPASDGRRIIAAVMNSYGSNPPTTTWKGMLSFPRTLSLKKVGTQQHFVQQPITE
LDTISTSLQILANQTITPGQTLLSSIRGTALDVRVAFYPDAGSVLSLAVRKGASEQTVIKYTQS
DATLSVDRTESGDISYDPAAGGVHTAKLEEDGTGLVSIRVLVDTCSVEVFGGQGEAVISDLIF
PSDSSDGLALEVTGGNAVLQSVDVRSVSLE
11111111111111111111111000000000000000000000000000000000000000000000000000000000000
00000000000000000000000000000000000000000000000000000000000000000000000000000000000
00000000000000000000000000000000000000000000000000000000000000000000000000000000000
00000000000000000000000000000000000000000000000000000000000000000000000000000000000
00000000000000000000000000000000000000000000000000000000000000000000000000000000000
00000000000000000000000000000000000000000000000000000000000000000000000000000000000
000000000000000000
>Q8A1B4_3sgh_23_520
FEDFNTNPYQPSKVPASNLLSTMFNVYACPQQNACQEINCMWASFSGQVTATANWSFGK
NIFAYYNASEGHNDSSWGRLYGYIYPSFFLVENSTEKKGVIYAMAQLTRVYGMQLLASLQGP
IPYTQMKAGETEAPYDNEQTVWHAMFDDLDNAITILKSAATFGVNQDLAVVDQFYKGDC
SKWLKFANTLKLRMAIRISGVEPEYAQTKAQEAVLGGVMESVGDSSYDTTNGGINENGYAI
VSGWPEVRANACLVSYMNGYNDPRRPAYFTPQTQTAAGGYVGVRSGSAEIPEPTVYANYS
KLFIATDKTLPQPVMYAAEAAFLRAEGALKGWNMGGDAKTFYEKGVRLSFEEFGVSGAD
DYLADATSIPGNYVDNLIAGHTGNNYTNQSSITIKWEDGADDAKKLERVLTQKWIACYPD
PMNGWADFRRTGYPRIFPATESMNADCNTGRGQRRLRFTRSEYNNNKANVEAAVSMLSN

GKDSNGTDLWWAMKENGTY
11111111111111000000000000000000000000000000000000000000000000000000000000000000000
00000000000000000000000000000000000000000000000000000000000000000000000000000000000
00000000000000000000000000000000000000000000000000000000000000000000000000000000000
00000000000000000000000000000000000000000000000000000000000000000000000000000000000
00000000000000000000000000000000000000000000000000000000000000000000000000000000000
00000000000000000000000000000000000000000000000000000000000000000000000000000000000
>P05187_1ew2_22_534
IIPVEEENPDFWNREAAEALGAAKKLQPAQTAAKNLIIFLGDGMGVSTVTAARILKGQKKD
KLGPEIPLAMDRFPYVALSKTYNVDKHVPDSGATATAYLCGVKGNFQTIGLSAAARFNQC
NTTRGNEVISVMNRAKKAGKSVGVVTTTRVQHASPAGTYAHTVNRNWYSDADVPASAR
QEGCQDIATQLISNMDIDVILGGGRKYMFRMGTPDPEYPDDYSQGGTRLDGKNLVQEWLA
KRQGARYVWNRTELMQASLDPSVTHLMGLFEPGDMKYEIHRDSTLDPSLMEMTEAALRL
LSRNPRGFFLFVEGGRIDHGHHESRAYRALTETIMFDDAIERAGQLTSEEDTLSLVTADHSH
VFSFGGYPLRGSSIFGLAPGKARDRKAYTVLLYGNGPGYVLKDGARPDVTESESGSPEYRQQ
SAVPLDEETHAGEDVAVFARGPQAHLVHGVQEQTFIAHVMAFAACLEPYTACDLAPPAGT
TDAAHPGRSVVPALLPLLAGTLLLLETATAP
00000000000000000000000000000000000000000000000000000000000000000000000000000000000
00000000000000000000000000000000000000000000000000000000000000000000000000000000000
00000000000000000000000000000000000000000000000000000000000000000000000000000000000
00000000000000000000000000000000000000000000000000000000000000000000000000000000000
00000000000000000000000000000000000000000000000000000000000000000000000000000000000
00000000000000000000000000000000000000000000000000000000000000001111111111111111111
111111111111111
>Q5LEF0_3nqp_25_537
DKLDLSPIDYYGSGSYWKTEAQATAYIDGIHKHLRDAAWQHTITFGELRGGRFITGASSDG
MGVSNGDIILQNFDETHTGVSKFGDLFGRITNLNLFIARVTDATYLSDEMKNFYLGEVYGL
RAFYYFDLYRIYGGVPLRLTADVVEGVIDPNKLYMARSTPKEVMTQIKSDLNKSMEYFGNM
NDFDPYKRGKKVYWSKAATECLMGEVYLWTSKVTTGDDVANPADLTIAKTHLESVLNNY
NLKMLDDFSQVFNAKNKANDEIIFAIRFLEGEATNSNGTFTYNVGTGSTKNRYQANGEVF
GDALDIQNTGNQTYEYNKAVYQNFDDADTRKEATFIASYNKDGKTGELSLYGTHVRKNIG
YVNAQGARVYCGDYIFYRLPWVYLTLAEIANMEGDNAAVAKYINLVRKRAYGNAWDETL
YAYPETADFTTNELAILHEKDKEFIQEGQRWWDLRRMTLTKGGTPLVFCKEGSLLGDAPILN
KSTEAHKLLWPIEKTMLNKDPALEQTPGYK
11111111111111100000000000000000000000000000000000000000000000000000000000000000000
00000000000000000000000000000000000000000000000000000000000111111111110000000000000
00000000000000000000000000000000000000000000000000000000000000000000000000000000000
00000000000000000000000000000000000000000000000000000000000000000000000000000000000
00000000000000000000000000000000000000000000000000000000000000000000000000000000000
00000000000000000000000000000000000000000000000000000000000000000000000000000000000
000000000000000
>Q84AQ2_3u52_0_510
MVSKNKKLNLKDKYQYLTRDMAWEPTYQDKKDIFPEEDFEGIKITDWSQWEDPFRLTMD
AYWKYQAEKEKKLYAIFDAFAQNNGHQNISDARYVNALKLFISGISPLEHAAFQGYSKVGR
QFSGAGARVACQMQAIDELRHSQTQQHAMSHYNKHFNGLHDGPHMHDRVWYLSVPKS
FFDDARSAGPFEFLTAISFSFEYVLTNLLFVPFMSGAAYNGDMATVTFGFSAQSDEARHMTL
GLEVIKFILEQHEDNVPIVQRWIDKWFWRGFRLLSLVSMMMDYMLPNKVMSWSEAWEVY
YEQNGGALFKDLERYGIRPPKYQDVANDAKHHLSHQLWTTFYQYCQATNFHTWIPEKEE
MDWMSEKYPDTFDKYYRPRYEYLAKEAAAGRRFYNNTLPQLCQVCQIPTIFTEKDAPTML
SHRQIEHEGERYHFCSDGCCDIFKHEPEKYIQAWLPVHQIYQGNCEGGDLETVVQKYYHIN
IGEDNFDYVGSPDQKHWLSIKGRKPADKNQDAA

11111000000000000000000000000000000000000000000000000000000000000000000000000000000
00000000000000000000000000000000000000000000000000000000000000000000000000000000000
00000000000000000000000000000000000000000000000000000000000000000000000000000000000
00000000000000000000000000000000000000000000000000000000000000000000000000000000000
00000000000000000000000000000000000000000000000000000000000000000000000000000000000
00000000000000000000000000000000000000000000000000000000000000000000000000000000000
0111111111111
>P22637_1coy_45_551
APSRTLADGDRVPALVIGSGYGGAVAALRLTQAGIPTQIVEMGRSWDTPGSDGKIFCGMLN
PDKRSMWLADKTDQPVSNFMGFGINKSIDRYVGVLDSERFSGIKVYQGRGVGGGSLVNGG
MAVTPKRNYFEEILPSVDSNEMYNKYFPRANTGLGVNNIDQAWFESTEWYKFARTGRKTA
QRSGFTTAFVPNVYDFEYMKKEAAGQVTKSGLGGEVIYGNNAGKKSLDKTYLAQAAATG
KLTITTLHRVTKVAPATGSGYSVTMEQIDEQGNVVATKVVTADRVFFAAGSVGTSKLLVSMK
AQGHLPNLSSQVGEGWGNNGNIMVGRANHMWDATGSKQATIPTMGIDNWADPTAPIFA
EIAPLPAGLETYVSLYLAITKNPERARFQFNSGTGKVDLTWAQSQNQKGIDMAKKVFDKIN
QKEGTIYRTDLFGVYFKTWGDDFTYHPLGGVLLNKATDNFGRLPEYPGLYVVDGSLVPGN
VGVNPFVTITRLAERNMDKIISSDIQ
11100000000000000000000000000000000000000000000000000000000000000000000000000000000
00000000000000000000000000000000000000000000000000000000000000000000000000000000000
00000000000000000000000000000000000000000000000000000000000000000000000000000000000
00000000000000000000000000000000000000000000000000000000000000000000000000000000000
00000000000000000000000000000000000000000000000000000000000000000000000000000000000
00000000000000000011000000000000000000000000000000000000000000000000000000000000000
000000001
>D0VX21_3gh5_0_504
MASMMSFIPESASASTSQPSILPKPVSYTVGSGQFVLTKNASIFVAGNNVGETDELFNIGQAL
AKKLNASTGYTISVVKSNQPTAGSIYLTTVGGNAALGNEGYDLITTSNQVTLTANKPEGVFR
GNQTLLQLLPAGIEKNTVVSGVQWVIPHSNISDKPEYEYRGLMLDVARHFFTVDEVKRQID
LASQYKINKFHMHLSDDQGWRIEIKSWPDLIEIGSKGQVGGGPGGYYTQEQFKDIVSYAAE
RYIEVIPEIDMPGHTNAALASYGELNPDGKRKAMRTDTAVGYSTLMPRAEITYQFVEDVISE
LAAISPSPYIHLGGDESNATSAADYDYFFGRVTAIANSYGKKVVGWDPSDTSSGATSDSVLQ
NWTCSASTGTAAKAKGMKVIVSPANAYLDMKYYSDSPIGLQWRGFVNTNRAYNWDPTDC
IKGANIYGVESTLWTETFVTQDHLDYMLYPKLLSNAEVGWTARGDRNWDDFKERLIEHTP
RLQNKGIKFFADPIV
00001111111111110000000000000000000000000000000000000000000000000000000000000000000
00000000000000000000000000000000000000000000000000000000000000000000000000000000000
00000000000000000000000000000000000000000000000000000000000000000000000000000000000
00000000000000000000000000000000000000000000000000000000000000000000000000000000000
00000000000000000000000000000000000000000000000000000000000000000000000000000000000
00000000000000000000000000000000000000000000000000000000000000000000000000000000000
0000000
>A0FCI1_2yp4_16_518
QKLPGNDNSTATLCLGHHAVPNGTIVKTITNDQIEVTNATELVQSSSTGGICDSPHQILDGE
NCTLIDALLGDPQCDGFQNKKWDLFVERSKAYSNCYPYDVPDYASLRSLVASSGTLEFNNE
SFNWTGVTQNGTSSACKRRSNNSFFSRLNWLTHLKFKYPALNVTMPNNEKFDKLYIWGVH
HPGTDNDQISLYAQASGRITVSTKRSQQTVIPNIGSRPRVRDIPSRISIYWTIVKPGDILLINSTG
NLIAPRGYFKIRSGKSSIMRSDAPIGKCNSECITPNGSIPNDKPFQNVNRITYGACPRYVKQN
TLKLATGMRNVPEKQTRGIFGAIAGFIENGWEGMVDGWYGFRHQNSEGIGQAADLKSTQ
AAINQINGKLNRLIGKTNEKFHQIEKEFSEVEGRIQDLEKYVEDTKIDLWSYNAELLVALEN
QHTIDLTDSEMNKLFERTKKQLRENAEDMGNGCFKIYHKCDNACIGSIRNGTYDHDVYRD
EALNNRFQIK

11111110000000000000000000000000000000000000000000000000000000000000000000000000000
00000000000000000000000000000000000000000000000000000000000000000000000000000000000
00000000000000000000000000000000000000000000000000000000000000000000000000000000000
00000000000000000000000000000000000000000000000000000000000000000000000000000001111
10000000000000000000000000000000000000000000000000000000000000000000000000000000000
00000000000000000000000000000000000000000000000000000000000000000000000000000000000
00000
>Q9X0Z9_2gi3_0_474
MIDLDFRKLTIEECLKLSEEEREKLPQLSLETIKRLDPHVKAFISVRENVSVEKKGKFWGIPVA
IKDNILTLGMRTTCASRILENYESVFDATVVKKMKEAGFVVVGKANLDEFAMGSSTERSAFF
PTRNPWDLERVPGGSSGGSAAAVSAGMVVAALGSDTGGSVRQPASLCGVVGYKPTYGLVS
RYGLVAFASSLDQIGPITKTVRDAAILMEIISGRDENDATTVNRKVDFLSEIEEGVSGMKFAVP
EEIYEHDIEEGVSERFEEALKLLERLGAKVERVKIPHIKYSVATYYVIAPAEASSNLARFDGVK
YGLRIKEKGLREMYMKTRNVGFGEEVRRRIMIGTFTLSAAYYEAYFNKAMKVRRKISDELN
EVLSQYDAILTPTSPVTAFKIGEIKDPLTYYLMDIFTIPANLAGLPAISVPFGFSNNLPVGVQVI
GRRFADGKVFRIARAIEKNSPYNENGMFPLPEVKA
00000000000000000000000000000000000000000000000000000000000000000000000000000000000
00000000000000000000000000000000000000000000000000000000000000000000000000000000000
00000000000000000000000000000000000000000000000000000000000000000000000000000000000
00000000000000000000000000000000000000000000000000000000011111111111111111111111100
00000000000000000000000000000000000000000000000000000000000000000000000000000000000
000000000000000000000000000000000000000000000000000000000000
>Q0PCD7_2ipi_43_544
DGGACGARTALVKVDRVDRRYQDLVTRGFNGRFRGRPDVVYVVHTADQVVDAVNQAM
AAGQRIAVRSGGHCFEGFVDDPAVRAVIDMSQMRQVFYDSGKRAFAVEPGATLGETYRAL
YLDWGVTIPAGVCPQVGVGGHVLGGGYGPLSRRDGVVADHLYAVEVVVVDASGRARKVV
ATSAADDPNRELWWAHTGGGGGNFGIVTRYWFRTPGATGTDPSQLLPKAPTSTLRHIVTW
DWSALTEEAFTRIIDNHGAWHQSNSAAGTPYASMHSVFYLNSRAAGQILLDIQIDGGLDG
AEALLNDFVAAVNEGTGVEPAVQRSTEPWLRATLANKFDTGGFDRTKSKGAYLRKPWTAA
QAATLYRHLSADSQVWGEVSLYSYGGKVNSVPETATATAQRDSIIKVWMSATWMDPAHDD
ANLAWIREIYREIFATTGGVPVPDDRTEGTFINYPDVDLVDERWNTSGVPWYTLYYKGNYP
RLQKVKARWDPRDVFRHALSVRPPG
11111111100000000000000000000000000000000000000000000000000000000000000000000000000
00000000000000000000000000000000000000000000000000000000000000000000000000000000000
00000000000000000000000000000000000000000000000000000000000000000000000000000000000
00000000000000000000000000000000000000000000000000000000000000000000000000000000000
00000000000000000000000000000000000000000000000000000000000000000000000000000000000
00000000000000000000000000000000000000000000000000000000000000000000000000000000000
0001
>Q8U2I8_3icj_1_501
ASLPISNFFTFNHQSTLFTKVKNFMGVKHIGDCMKALINGTIYTSFSPVKKVSGLVISNERVL
YAGDSSTALRIAELAGGEIIDLKGKFVMPAFFDSHLHLDELGMSLEMVDLRGVKSMEELVER
VKKGRGRIIFGFGWDQDELGRWPTREDLDVIDRPVFLYRRCFHVAVMNSKMIDLLNLKPSK
DFDESTGIVRERALEESRKIINEKILTVKDYKHYIESAQEHLLSLGVHSVGFMSVGEKALKAL
FELEREGRLKMNVFAYLSPELLDKLEELNLGKFEGRRLRIWGVKLFVDGSLGARTALLSEPY
TDNPTTSGELVMNKDEIVEVIERAKPLGLDVAVHAIGDKAVDVALDAFEEAEFSGRIEHASL
VRDDQLERIKELKVRISAQPHFIVSDWWIVNRVGEERAKWAYRLKTLSSITKLGFSTDSPIEP
ADPWVSIDAAVNRYVVDPGERVSREEALHLYTHGSAQVTLAEDLGKLERGFRAEYIILDRD
PLKE
11111111111111111111111111111111000000000000000000000000000000000000000000000000000
00000000000000000000000000000000000000000000000000000000000000000000000000000000000

00000000000000000000000000000000000000000000000000000000000000000000000000000000000
00000000000000000000000000000000000000000000000000000000000000000000000000000000000
00000000000000000000000000000000000000000000000000000000000000000000000000000000000
00000000000000000000000000000000000000000000000000000000000000000000000000000000000
001
>Q8A204_3kya_23_517
KDDDNVETGAFDPSKPVAISDFTPKEGGAYQKLLIYGENFGTDVSKVKVKIGGKDAIVINVK
STYVYCFVPSGAFSGEIEITVGEGENAVTTTASTTFSYEKKMVVGTLCGYRNNRDDQGWRD
GPFDGPEGVKCCGFSDNGRLAFDPLNKDHLYICYDGHKAIQLIDLKNRMLSSPLNINTIPT
NRIRSIAFNKKIEGYADEAEYMIVAIDYDGKGDESPSVYIIKRNADGTFDDRSDIQLIAAYKQ
CNGATIHPINGELYFNSYEKGQVFRLDLVDYFKTIKNGGSWDPIVKNNPNTFKQLFTIADPS
WEFQIFIHPTGKYAYFGVINNHYFMRSDYDEIKKEFITPYNFVGGYKQSGYRDDVGTEARM
NNPCQGVFVKNPDYTGEEEYDFYFVDRLNFCVRKVTPEGIVSTYAGRGASTSLADGNQWG
TDDGDLREVARFRDVSGLVYDDVKEMFYVHDQVGHTIRTISMEQEENVAGDENIPEDESTV
ESNE
11111000000000000000000000000000000000000000000000000000000000000000000000000000000
00000000000000000000000000000000000000000000000000000000000000000000000000000000000
00000000000000000000000000000000000000000000000000000000000000000000000000000000000
00000000000000000000000000000000000000000000000000000000000000000000000000000000000
00000000000000000000000000000000000000000000000000000000000000000000000000000000000
00000000000000000000000000000000000000000000000000000000000001111111111111111111
>Q8FHD0_4im7_0_485
MGNHLLSAKATLPVYDRNNLAPRIVHLGFGAFHRAHQGVYADILATEHFSDWGYYEVNLI
GGEQQIADLQQQDNLYTVAEMSADAWTARVVGVVKKALHVQIDGLETVLAAMCEPQIAI
VSLTITEKGYFHSPATGQLMLDHPMVVADVQNPHQPKTATGVIVEALARRKAAGLPAFTV
MSCDNMPENGHVMRDVVTSYAQAIDVKLAQWIEDNVTFPSTMVDRIVPAVTEDTLAKIEQ
LTGVRDAAGVACEPFRQWVIEDNFVAGRPEWEKAGAELVSDVLPYEEMKLRMLNGSHSFL
AYLGYLAGYQHINDCMEDEHYRHAAYTLMLQEQAPTLKVQGVDLQDYANRLIERYSNPA
LRHRTWQIAMDGSQKLPQRMLDSVRWHLAHDSKFDLLALGVAGWMRYVGGVDEQGNP
IEISDPLLPVIQKAVQSSAEGTARVQSLLAIKAIFGDDLPGNSLFTTKVTEAYLSLLAHGAKAT
VAKYSVK
11100000000000000000000000000000000000000000000000000000000000000000000000000000000
00000000000000000000000000000000000000000000000000000000000000000000000000000000000
00000000000000000000000000000000000000000000000000000000000000000000000000000000000
00000000000000000000000000000000000000000000000000000000000000000000000000000000000
00000000000000000000000000000000000000000000000000000000000000000000000000000000000
00000000000000000000000000000000000000000000000000000000000000000000000
>Q8FLC3_3ed4_18_508
ASLIGLAVCTGNAFSPALAAEAKQPNLVIIMADDLGYGDLATYGHQIVKTPNIDRLAQEGV
KFTDYYAPAPLSSPSRAGLLTGRMPFRTGIRSWIPSGKDVALGRNELTIANLLKAQGYDTAM
MGKLHLNAGGDRTDQPQAQDMGFDYSLANTAGFVTDATLDNAKERPRYGMVYPTGWL
RNGQPTPRADKMSGEYVSSEVVNWLDNKKDSKPFFLYVAFTEVHSPLASPKKYLDMYSQY
MSAYQKQHPDLFYGDWADKPWRGVGEYYANISYLDAQVGKVLDKIKAMGEEDNTIVIFTS
DNGPVTREARKVYELNLAGETDGLRGRKDNLWEGGIRVPAIIKYGKHLPQGMVSDTPVYG
LDWMPTLAKMMNFKLPTDRTFDGESLVPVLEQKALKREKPLIFGIDMPFQDDPTDEWAIR
DGDWKMIIDRNNKPKYLYNLKSDRYETLNLIGKKPDIEKQMYGKFLKYKTDIDNDSLMKA
RGDKPEAVTWG
11111111111000001111111000000000000000000000000000000000000000000000000000000000000
00000000000000000000000000000000000000000000000000000000000000000000000000000000000
00000000000000000000000000000000000000000000000000000000000000000000000000000000000
00000000000000000000000000000000000000000000000000000000000000000000000000000000000

0000000000000000000000000000000000000000000000000000000000000000000000000000000000000000000
00000000000000000000000000000000000000000000000000000000000000000000000000000000000
>Q5SJP8_2yyl_0_480
MARTGAEYIEALKTRPPNLWYKGEKVEDPTTHPVFRGIVRTMAALYDLQHDPRYREVLTYE
EEGKRHGMSFLIPKTKEDLKRRGQAYKLWADQNLGMMGRSPDYLNAVVMAYAASADYF
GEFAENVRNYYRYLRDQDLATTHALTNPQVNRARPPSGQPDPYIPVGVVKQTEKGIVVRG
ARMTATFPLADEVLIFPSTLLQAGSEKYALAFALPTSTPGLHFVCREALVGGDSPFDHPLSSR
VEEMDCLVIFDDVLVPWERVFILGNVELCNNAYAATGALNHMAHQVVALKTAKTEAFLG
VAALMAEGIGADVYGHVQEKIAEIIVYLEAMRAFWTRAEEEAKENAYGLLVPDRGALDGA
RNLYPRLYPRIREILEQIGASGLITLPSEKDFKGPLGPFLEKFLQGAALEAKERVALFRLAWD
MTLSGFGARQELYERFFFGDPVRMYQTLYNVYNKEPYKERIRAFLKESLKVFEEVQA
1000000000000000000000000000000000000000000000000000000000000000000000000000000000000000000
0000000000000000000000000000000000000000000000000000000000000000000000000000000000000000000
0000000000000000000000000000000000000000000000000000000000000000000000000000000000000000000
0000000000000000000000000000000000000000000000000000000000000000000000000000000000000000000
0000000000000000000000000000000000000000000000000000000000000000000000000000000000000000000
000000000000000000000000000000000000000000000000000000000000000000001111
>P05177_2hi4_26_513
VLKGLRPRVPKGLKSPPEPWGWPLLGHVLTLGKNPHLALSRMSQRYGDVLQIRIGSTPVLV
LSRLDTIRQALVRQGDDFKGRPDLYTSTLITDGQSLTFSTDSGPVWAARRRLAQNALNTFSI
ASDPASSSSCYLEEHVSKEAKALISRLQELMAGPGHFDPYNQVVVSVANVIGAMCFGQHFP
ESSDEMLSLVKNTHEFVETASSGNPLDFFPILRYLPNPALQRFKAFNQRFLWFLQKTVQEHY
QDFDKNSVRDITGALFKHSKKGPRASGNLIPQEKIVNLVNDIFGAGFDTVTTAISWSLMYLV
TKPEIQRKIQKELDTVIGRERRPRLSDRPQLPYLEAFILETFRHSSFLPFTIPHSTTRDTTLNGFY
IPKKCCVFVNQWQVNHDPELWEDPSEFRPERFLTADGTAINKPLSEKMMLFGMGKRRCIG
EVLAKWEIFLFLAILLQQLEFSVPPGVKVDLTPIYGLTMKHARCEHVQARRFSI
1111111000000000000000000000000000000000000000000000000000000000000000000000000000000000000
0000000000000000000000000000000000000000000000000000000000000000000000000000000000000000000
0000000000000000000000000000000000000000000000000000000000000000000000000000000000000000000
0000000000000000000000000000000000000000000000000000000000000000000000000000000000000000000
0000000000000000000000000000000000000000000000000000000000000000000000000000000000000000000
00000000000000000000000000000000000000000000000000000000000000000000000000000001
>P35557_4dch_0_464
MLDDRARMEAAKKEKVEQILAEFQLQEEDLKKVMRRMQKEMDRGLRLETHEEASVKML
PTYVRSTPEGSEVGDFLSLDLGGTNFRVMLVKVGEGEEGQWSVKTKHQMYSIPEDAMTGT
AEMLFDYISECISDFLDKHQMKHKKLPLGFTFSFPVRHEDIDKGILLNWTKGFKASGAEGN
NVVGLLRDAIKRRGDFEMDVVAMVNDTVATMISCYYEDHQCEVGMIVGTGCNACYMEE
MQNVELVEGDEGRMCVNTEWGAFGDSGELDEFLLEYDRLVDESSANPGQQLYEKLIGGKY
MGELVRLVLLRLVDENLLFHGEASEQLRTRGAFETRFVSQVESDTGDRKQIYNILSTLGLRPS
TTDCDIVRRACESVSTRAAHMCSAGLAGVINRMRESRSEDVMRITVGVDGSVYKLHPSFKE
RFHASVRRLTPSCEITFIESEEGSGRGAALVSAVACKKACMLGQ
1111111100000000000000000000000000000000000000000000000000000000000000000000000000000000000
0000000000011000000000000000000000000000000000000000000000000000000000000000001111111111111
1111111111110000000000000000000000000000000000000000000000000000000000000000000000000000000
0000000000000000000000000000000000000000000000000000000000000000000000000000000000000000000
0000000000000000000000000000000000000000000000000000000000000000000000000000000000000000000
0000000000000000000000000000000000000000000000000000000
>Q9AGH8_1rjq_0_483
MSQPDATPFDYILSGGTVIDGTNAPGRLADVGVRGDRIAAVGDLSASSARRRIDVAGKVVS
PGFIDSHTHDDNYLLKHRDMTPKISQGVTTVVTGNCGISLAPLAHANPPAPLDLLDEGGSF
RFARFSDYLEALRAAPPAVNAACMVGHSTLRAAVMPDLRREATADEIQAMQALADDALA

SGAIGISTGAFYPPAAHASTEEIIEVCRPLITHGGVYATHMRDEGEHIVQALEETFRIGRELDV
PVVISHHKVMGKLNFGRSKETLALIEAAMASQDVSLDAYPYVAGSTMLKQDRVLLAGRTLI
TWCKPYPELSGRDLEEIAAERGKSKYDVVPELQPAGAIYFMMDEPDVQRILAFGPTMIGSD
GLPHDERPHPRLWGTFPRVLGHYSRDLGLFPLETAVWKMTGLTAAKFGLAERGQVQPGYY
ADLVVFDPATVADSATFEHPTERAAGIHSVYVNGAAVWEDQSFTGQHAGRVLNRAGA
11111110000000000000000000000000000000000000000000000000000000000000000000000000000
00000000000000000000000000000000000000000000000000000000000000000000000000000000000
00000000000000000000000000000000000000000000000000000000000000000000000000000000000
00000000000000000000000000000000000000000000000000000000000000000000000000000000000
00000000000000000000000000000000000000000000000000000000000000000000000000000000000
000000000000000000000000000000000000000000000000000000000000000000111
>P06278_1vjs_29_511
ANLNGTLMQYFEWYMPNDGQHWKRLQNDSAYLAEHGITAVWIPPAYKGTSQADVGYGA
YDLYDLGEFHQKGTVRTKYGTKGELQSAIKSLHSRDINVYGDVVINHKGGADATEDVTAV
EVDPADRNRVISGEHRIKAWTHFHFPGRGSTYSDFKWHWYHFDGTDWDESRKLNRIYKFQ
GKAWDWEVSNENGNYDYLMYADIDYDHPDVAAEIKRWGTWYANELQLDGFRLDAVKHI
KFSFLRDWVNHVREKTGKEMFTVAEYWQNDLGALENYLNKTNFNHSVFDVPLHYQFHA
ASTQGGGYDMRKLLNSTVVSKHPLKAVTFVDNHDTQPGQSLESTVQTWFKPLAYAFILTRE
SGYPQVFYGDMYGTKGDSQREIPALKHKIEPILKARKQYAYGAQHDYFDHHDIVGWTREG
DSSVANSGLAALITDGPGGAKRMYVGRQNAGETWHDITGNRSEPVVINSEGWGEFHVNG
GSVSIYVQR
11000000000000000000000000000000000000000000000000000000000000000000000000000000000
00000000000000000000000000000000000000000000000000000000000000000000000000000000000
00000000000000011111111111000000000000000000000000000000000000000000000000000000000
00000000000000000000000000000000000000000000000000000000000000000000000000000000000
00000000000000000000000000000000000000000000000000000000000000000000000000000000000
00000000000000000000000000000000000000000000000000000000000000000001
>Q2SWH9_3v7n_0_482
MNYISTRGAGIGERHTFSDILLGGLAKDGGLYLPSEYPQVSADELARWRTLPYADLAFEILSK
FCDDIAAADLRAITRRTYTADVYRHARRGGNAADITPLTTLGTENGAPVSLLELSNGPTLA
FKDMAMQLLGNLFEYTLAKHGETLNILGATSGDTGSAAEYAMRGKEGVRVFMLSPHKKM
SAFQTAQMYSLQDPNIFNLAVNGVFDDCQDIVKAVSNDHAFKAQQKIGTVNSINWARVV
AQVVYYFKGYFAATRSNDERVSFTVPSGNFGNVCAGHIARMMGLPIEKLVVATNENDVLD
EFFRTGAYRVRSAQDTYHTSSPSMDISKASNFERFVFDLLGRDPARVVQLFRDVEQKGGFDL
AASGDFARVAEFGFVSGRSTHADRIATIRDVFERYRTMIDTHTADGLKVAREHLRPGVPMV
VLETAQPIKFGESIREALGQEPSRPAAFDGLEALPQRFEVVDANAQQVKDFIAAHTGA
10000000000000000000000000000000000000000000000000000000000000000000000000000000000
00000000000000000000000000000000000000000000000000000000000000000000000000000000000
00000000000000000000000000000000000000000000000000000000000000000000000000000000000
00000000000000000000000000000000000000000000000000000000000000011111111111111111100
00000000000000000000000000000000000000000000000000000000000000000000000000000000000
00000000000000000000000000000000000000000000000000000000000000000000
>Q8AB42_3hdx_35_511
ASETQQWKTLEDTRSALMGVYGLTRAALADNNTHWICGDLRKGDFTVYKRSDLQAVSDN
ELNKPYDLLKKVSNWRRFYAVINAASVFMEKAPRTVELDRSYSEQNLKYDIAQVRALRAFA
YFYMVRIWGDVPLVTYSYDNGTFPSMPRTDAQTVLSYAKAELLTAIEDLPYQYGTQTNLYY
GSYGAQWQGKLFNKLSAYSVLAHICAWQGNYAEAETYSAFIIDHASEINAKYTSIADLTSET
GLFYSNASVKGSRILGFNFAHNDNEATQSGHLEQLTLAYPLVQKSYPEIYISKDSLFSIFTNFD
DLRFGIIDTIKYSSYYVQNLNEETPVFSKIKIIQDGSAKDNDFGVFGSSIVFTRLEDITLLRAEA
LCALNRSTEAVSYLNMIRTNRGLREVSFKKDFGNNRESLIAEIFEERRRELMGEGWRWYDL
VRRQKLMKDNEAFLRLISSGGIYWPVSEDIITANSQIEQNEFWK

11100000000000000000000000000000000000000000000000000000000000000000000000000000000
00000000000000000000000000000000000000000000000000000000000000000000000000000000000
00000000000000000000000000000000000000000000000000000000000000000000000000000000000
00000000000000011111111100000000000000000000000000000000000000000000000000000000000
00000000011111111100000000000000000000000000000000000000000000000000000000000000000
00000000000000000000000000000000000000000000000000000000000000
>P51094_2c1x_0_455
MSQTTTNPHVAVLAFPFSTHAAPLLAVVRRLAAAAPHAVFSFFSTSQSNASIFHDSMHTMQ
CNIKSYDISDGVPEGYVFAGRPQEDIELFTRAAPESFRQGMVMAVAETGRPVSCLVADAFIW
FAADMAAEMGLAWLPFWTAGPNSLSTHVYIDEIREKIGVSGIQGREDELLNFIPGMSKVRF
RDLQEGIVFGNLNSLFSRMLHRMGQVLPKATAVFINSFEELDDSLTNDLKSKLKTYLNIGPF
NLITPPPVVPNTTGCLQWLKERKPTSVVYISFGTVTTPPPAEVVALSEALEASRVPFIWSLRD
KARVHLPEGFLEKTRGYGMVVPWAPQAEVLAHEAVGAFVTHCGWNSLWESVAGGVPLIC
RPFFGDQRLNGRMVEDVLEIGVRIEGGVFTKSGLMSCFDQILSQEKGKKLRENLRALRETA
DRAVGPKGSSTENFITLVDLVSKPKDV
11111100000000000000000000000000000000000000000000000111111100000000000000000000000
00000000000000000000000000000000000000000000000000000000000000000000000000000000000
00000000000000000000000000000000000000000000000000000000000000000000000000000000000
01111111110000000000000000000000000000000000000000000000000000000000000000000000000
00000000000000000000000000000000000000000000000000000000000000000000000000000000000
0000000000000000000000000000000000000000
>P16317_1k7q_0_478
MGKNLSLRQDDAQHALSANTSSAYNSVYDFLRYHDRGDGLTVNGKTSYSIDQAAAQITRE
NVSWNGTNVFGKSANLTFKFLQSVSSIPSGDTGFVKFNAEQIEQAKLSLQSWSDVANLTFTE
VTGNKSANITFGNYTRDASGNLDYGTQAYAYYPGNYQGAGSSWYNYNQSNIRNPGSEEYG
RQTFTHEIGHALGLAHPGEYNAGEGDPSYNDAVYAEDSYQFSIMSYWGENETGADYNGH
YGGAPMIDDIAAIQRLYGANMTTRTGDSVYGFNSNTDRDFYTATDSSKALIFSVWDAGGTD
TFDFSGYSNNQRINLNEGSFSDVGGLKGNVSIAHGVTIENAIGGSGNDILVGNSADNILQG
GAGNDVLYGGAGADTLYGGAGRDTFVYGSGQDSTVAAYDWIADFQKGIDKIDLSAFRNE
GQLSFVQDQFTGKGQEVMLQWDAANSITNLWLHEAGHSSVDFLVRIVGQAAQSDIIV
11111111111111111000000000000000000000000000000000000000000000000000000000000000000
00000000000000000000000000000000000000000000000000000000000000000000000000000000000
00000000000000000000000000000000000000000000000000000000000000000000000000000000000
00000000000000000000000000000000000000000000000000000000000000000000000000000000000
00000000000000000000000000000000000000000000000000000000000000000000000000000000000
000000000000000000000000000000000000000000000000000000000000000
>P05031_3k40_35_509
MEAPEFKDFAKTMVDFIAEYLENIRERRVLPEVKPGYLKPLIPDAAPEKPEKWQDVMQDIE
RVIMPGVTHWHSPKFHAYFPTANSYPAIVADMLSGAIACIGFTWIASPACTELEVVMMDWL
GKMLELPAEFLACSGGKGGGVIQGTASESTLVALLGAKAKKLKEVKELHPEWDEHTILGKL
VGYCSDQAHSSVERAGLLGGVKLRSVQSENHRMRGAALEKAIEQDVAEGLIPFYAVVTLGT
TNSCAFDYLDECGPVGNKHNLWIHVDAAYAGSAFICPEYRHLMKGIESADSFNFNPHKW
MLVNFDCSAMWLKDPSWVVNAFNVDPLYLKHDMQGSAPDYRHWQIPLGRRFRALKLWF
VLRLYGVENLQAHIRRHCNFAKQFGDLCVADSRFELAAEINMGLVCFRLKGSNERNEALL
KRINGRGHIHLVPAKIKDVYFLRMAICSRFTQSEDMEYSWKEVSAAADEMEQEQ
00000000000000000000000000000000000000000000000000000000000000000000000000000000000
00000000000000000000000000000000000000000000000000000000000000000000000000000000000
00000000000000000000000000000000000000000000000000000000000000000000000000000000000
00000000000000000000000000000000000000000000000000000000000000000000000011111111111
11111111111111110000000000000000000000000000000000000000000000000000000000000000000
00000000000000000000000000000000000000000000000000000000000

>B3FRL6_2x8t_0_468
MFNRLFRVCFLAALIMAFTLPNSVYAQKPIFKEVSVHDPSIIETNGTFYVFGSHLASAKSNDL
MQWQQLTTSVSNDNPLIPNVYEELKETFEWAQSDTLWAADVTQLADGKYYMYYNACRG
DSPRSAMGVAVADNIEGPYKNKGIFLKSGMEGTSSDGTPYDATKHPNVVDPHTFFDKDGK
LWMVYGSYSGGIFILEMNPKTGFPLPGQGYGKKLLGGNHSRIEGPYVLYNPDTQYYYLYLSY
GGLDATGGYNIRVARSKKPDGPYYDAEGNPMLDVRGKGGTFFDDRSIEPYGVKLMGSYTF
ETENEKGTGYVSPGHNSAYYDEKTGRSYLIFHTRFPGRGEEHEVRVHQLFMNKDGWPVAA
PYRYAGETLKEVKQKDITGTYKLIQHGKDISADIKQTINIQLNKNHTISGEMTGTWRKTGK
NTADITLAGKKYNGVFLRQWDSVREKNVMTFSVLNTSGEAVWGSK
11111111111111111111111111100000000000000000000000000000000000000000000000000000000
00000000000000000000000000000000000000000000000000000000000000000000000000000000000
00000000000000000000000000000000000000000000000000000000000000000000000000000000000
00000000000000000000000000000000000000000000000000000000000000000000000000000000000
00000000000000000000000000000000000000000000000000000000000000000000000000000000000
00000000000000000000000000000000000000000000000000000
>E2STD2_3ziy_31_498
ASAKLPGDFGPPRGEPIHAVLTSPPLVPPPVNRTYPAKVIVELEVVEKEMQISEGVSYTFWTF
GGTVPGSFIRVRQGDTVEFHLKNHPSSKMPHNIDLHGVTGPGGGAASSFTAPGHESQFTFK
ALNEGIYVYHCATAPVGMHIANGMYGLILVEPPEGLPKVDHEYYVMQGDFYTAGKYREK
GLQPFDMEKAIDERPSYVLFNGAEGALTGDKALHAKVGETVRIFVGNGGPNLVSSFHVIGA
IFDQVRYEGGTNVQKNVQTTLIPAGGAAVVKFTARVPGSYVLVDHSIFRAFNKGAMAILKI
DGAENKLVYSGKELDSVYLGDRAAPNMSAVTKATQASVSGTLTVQDQVQAGRALFAGTC
SVCHQGNGAGLPGVFPPLAKSDFLAADPKRAMNIVLHGLNGKIKVNGQEYDSVMPPMT
QLNDDEVANILTYVLNSWDNPGGRVSAEDVKKVRAQPAPAKAVAEH
11100000000000000000000000000000000000000000000000000000000000000000000000000000000
00000000000000000000000000000000000000000000000000000000000000000000000000000000000
00000000000000000000000000000000000000000000000000000000000000000000000000000000000
00000000000000000000000000000000000000000000000000000000000000000000000000000000000
00000000000000000000000000000000000000000000000000000000000000000000000000000000000
00000000000000000000000000000000000000000000011111111
>Q9KH42_2wn4_0_462
MKKFRKHKRISNCISILLILYLTLGGLLPNNIYAQDLQSYSEKVCNTTYKAPIERPEDFLKDKE
KAKEWERKEAERIEQKLERSEKEALESYKKDSVEISKYSQTRNYFYDYQIEANSREKEYKELR
NAISKNKIDKPMYVYYFESPEKFAFNKVIRTENQNEISLEKFNEFKETIQNKLFKQDGFKDIS
LYEPGKGDEKPTPLLMHLKLPRNTGMLPYTNTNNVSTLIEQGYSIKIDKIVRIVIDGKHYIKA
EASVVSSLDFKDDVSKGDSWGKANYNDWSNKLTPNELADVNDYMRGGYTAINNYLISNG
PVNNPNPELDSKITNIENALKREPIPTNLTVYRRSGPQEFGLTLTSPEYDFNKLENIDAFKSK
WEGQALSYPNFISTSIGSVNMSAFAKRKIVLRITIPKGSPGAYLSAIPGYAGEYEVLLNHGSKF
KINKIDSYKDGTITKLIVDATLIP
11111111111111111111111111111111111111111111111111111111111111111111110000000000000
00000000000000000000000000000000000000000000000000000000000000000000000000000000000
00000000000000000000000000000000000000000000000000000000110000000000000000000000000
00000000000000000000000000000000000000000000000000000000000000000000000000000000000
00000000000000000000000000000000000000000000000000000000000000000000000000000000000
00000000000000000000000000000000000000000000000
>Q8A169_3gza_18_459
AQQQELPVPKPHQLKWHEAEMGAVFHYDLHVFDGIRYGQGNNRINPIEDYNIFNPTELNT
DQWVQAAKAAGCKFAVLTATHETGFGLWQSDVNPYCLKAVKWRDGKGDIVRDFVNSCR
KYGLQPGIYIGIRWNSLLGIHNFKAEGEGAFARNRQAWYKRLCEKMVTELCTRYGDLYMI
WFDGGADDPRADGPDVEPIVNKYQPNCLFYHNIDRADFRWGGSETGTVEYPCWSTFPVP
CSHHKRIESSIDQLELLKHGDKNGRYWVPAMADTPLRGANGRHEWFWEPDDENNIYPLN

TLMDKYEKSVGRNATLILGLTPDPTGLIPAGDAQRLKEMGDEINRRFSSPIARISGQKKSLTL
KLGKEQSVNYCIIQENIKNGERIRQYQIEAKVNGKWQTVCKGESVGHKRIEKFEPVEATALR
LTVSESIALPDIINFSAYSVK
11110000000000000000000000000000000000000000000000000000000000000000000000000000000
00000000000000000000000000000000000000000000000000000000000000000000000000000000000
00000000000000000000000000000000000000000000000000000000000000000000000000000000000
00000000000000000000000000000000000000000000000000000000000000000000000000000000000
00000000000000000000000000000000000000000000000000000000000000000000000000000000000
000000000000000000000000000
>P21827_3of7_26_481
SHIINAQEDYKHMYLSVQPLDIFCWGTGSMCELGLGPLAKNKEVKRPRLNPFLPRDEAKIIS
FAVGGMHTLALDEESNVWSWGCNDVGALGRDTSNAKEQLKDMDADDSSDDEDGDLNE
LESTPAKIPRESFPPLAEGHKVVQLAATDNMSCALFSNGEVYAWGTFRCNEGILGFYQDKIK
IQKTPWKVPTFSKYNIVQLAPGKDHILFLDEEGMVFAWGNGQQNQLGRKVMERFRLKTLD
PRPFGLRHVKYIASGENHCFALTKDNKLVSWGLNQFGQCGVSEDVEDGALVTKPKRLALP
DNVVIRSIAAGEHHSLILSQDGDLYSCGRLDMFEVGIPKDNLPEYTYKDVHGKARAVPLPT
KLNNVPKFKSVAAGSHHSVAVAQNGIAYSWGFGETYAVGLGPFEDDTEVPTRIKNTATQD
HNIILVGCGGQFSVSGGVKLSDEDAEKRADEMDD
00000000000000000000000000000000000000000000000000000000000000000000000000000000000
00000000000011111111111111111111100000000000000000000000000000000000000000000000000
00000000000000000000000000000000000000000000000000000000000000000000000000000000000
00000000000000000000000000000000000000000000000000000000000000000000000000000000000
00000000000000000000000000000000000000000000000000000000000000000000000000000000000
00000000000000000000000000000000000000000
>O53665_3v1t_0_453
MAPKRSSDLFSQVVNSGPGSFLARQLGVPQPETLRRYRAGEPPLTGSLLIGGAGRVVEPLRA
ALEKDYDLVGNNLGGRWADSFGGLVFDATGITEPAGLKGLHEFFTPVLRNLGRCGRVVVV
GGTPEAAASTNERIAQRALEGFTRSLGKELRRGATTALVYLSPDAKPAATGLESTMRFLLSA
KSAYVDGQVFSVGADDSTPPADWEKPLDGKVAIVTGAARGIGATIAEVFARDGAHVVAID
VESAAENLAETASKVGGTALWLDVTADDAVDKISEHLRDHHGGKADILVNNAGITRDKLL
ANMDDARWDAVLAVNLLAPLRLTEGLVGNGSIGEGGRVIGLSSIAGIAGNRGQTNYATTK
AGMIGITQALAPGLAAKGITINAVAPGFIETQMTAAIPLATREVGRRLNSLLQGGQPVDVAE
AIAYFASPASNAVTGNVIRVCGQAMIGA
11111111111111111111111111110000000000000000000000000000000000001111111111111111000
00000000000000000000000000000000000000000000000000000000000000000000000000000000000
00000000000000000000000000000000000000000000000000000000000000000000000000000000000
00000000000000000000000000000000000000000000000000000000000000000000000000000000000
00000000000000000000000000000000000000000000000000000000000000001111111100000000000
00000000000000000000000000000000000000
>Q9KTK3_3gh1_6_456
IIQVSPAGSMDLLSQLEVERLKKTASSDLYQLYRNCSLAVLNSGSHTDNSKELLDKYKNFDIT
VMRRERGIKLELANPPEHAFVDGQIIKGIQEHLFSVLRDIVYVNMHLADSQRLNLTNATHIT
NLVFGILRNAGALIPGATPNLVVCWGGHSINEVEYQYTREVGHELGLRELNICTGCGPGAM
EGPMKGAAVGHAKQRYSEYRYLGLTEPSIIAAEPPNPIVNELVIMPDIEKRLEAFVRMAHGII
IFPGGPGTAEELLYILGIMMHPENADQPMPIVLTGPKQSEAYFRSLDKFITDTLGEAARKHYS
IAIDNPAEAARIMSNAMPLVRQHRKDKEDAYSFNWSLKIEPEFQLPFEPNHESMANLDLHL
NQRPEVLAANLRRAFSGVVAGNVKAEGIREIERHGPFEMHGDPVLMKKMDQLLNDFVAQ
NRMKLPGGSAYEPCYKIVT
00000000000000000000000000000000000000000000000000000000000000000000000000000000000
00000000000000000000000000001111111000000000000000000000000000000000000000000000000
00000000000000000000000000000000000000000000000000000000000000000000000000000000000

00000000000000000000000000000000000000000000000000000000000000000000000000000000000
00000000000000000000000000000000000000000000000000000000000000000000000000000000000
000000000000000000000000000000000000
>P11560_3pot_0_442
MAKFEDKVDLYDDRGNLVEEQVPLEALSPLRNPAIKSIVQGIKRTVAVNLEGIENALKTAKV
GGPACKIMGRELDLDIVGNAESIAAAAKEMIQVTEDDDTNVELLGGGKRALVQVPSARFD
VAAEYSAAPLVTATAFVQAIINEFDVSMYDANMVKAAVLGRYPQSVEYMGANIATMLDIP
QKLEGPGYALRNIMVNHVVAATLKNTLQAAALSTILEQTAMFEMGDAVGAFERMHLLGL
AYQGMNADNLVFDLVKANGKEGTVGSVIADLVERALEDGVIKVEKELTDYKVYGTDDLA
MWNAYAAAGLMAATMVNQGAARAAQGVSSTLLYYNDLIEFETGLPSVDFGKVEGTAVGF
SFFSHSIYGGGGPGIFNGNHIVTRHSKGFAIPCVAAAMALDAGTQMFSPEATSGLIKEVFSQ
VDEFREPLKYVVEAAAEIKNEI
10000000000000000000000000000000000000000000000000000000000000000000000000000000000
00000000000000000000000000000000000000000000000000000000000000000000000000000000000
00000000000000000000000000000000000000000000000000000000000000000000000000000000000
00000000000000000000000000000000000000000000000000000000000000000000000000000000000
00000000000000000000000000000000000000000000000000000000000000000000000000000000000
0000000000000000000000000000
>P00926_3ss9_0_441
MENAKMNSLIAQYPLVKDLVALKETTWFNPGTTSLAEGLPYVGLTEQDVQDAHARLSRFA
PYLAKAFPETAATGGIIESELVAIPAMQKRLEKEYQQPISGQLLLKKDSHLPISGSIKARGGIYE
VLAHAEKLALEAGLLTLDDDYSKLLSPEFKQFFSQYSIAVGSTGNLGLSIGIMSARIGFKVTV
HMSADARAWKKAKLRSHGVTVVEYEQDYGVAVEEGRKAAQSDPNCFFIDDENSRTLFLG
YSVAGQRLKAQFAQQGRIVDADNPLFVYLPCGVGGGPGGVAFGLKLAFGDHVHCFFAEPT
HSPCMLLGVHTGLHDQISVQDIGIDNLTAADGLAVGRASGFVGRAMERLLDGFYTLSDQT
MYDMLGWLAQEEGIRLEPSALAGMAGPQRVCASVSYQQMHGFSAEQLRNTTHLVWATG
GGMVPEEEMNQYLAKGR
11111111111000000000000000000000000000000000000000000000000000000000000000000000000
00000000000000000000000000000000000000000000000000000000000000000000000000000000000
00000000000000000000000000000000000000000000000000000000000000000000000000000000000
00000000000000000000000000000000000000000000000000000000000000000000000000000000000
00000000000000000000000000000000000000000000000000000000000000000000000000000000000
000000000000000000000000000
>A8IW39_3qgu_0_440
MQLNVRSTASGARSSTRSRRMTAVVQAVAQRAGTIDVQRNENFGKLRAGYLFPEIARRRK
AHQEKNPDAKIISLGIGDTTEPLPKYIADAMAKAAAGLATREGYSGYGAEQGQGALREAV
ASTFYGHAGRAADEIFISDGSKCDIARIQMMFGSKPTVAVQDPSYPVYVDTSVMMGMTGD
HNGTGFDGIEYMVCNPDNHFFPDLSKAKRTDIIFFCSPNNPTGAAATRAQLTELVNFARKN
GSILVYDAAYALYISNPDCPKTIYEIPGADEVAIETCSFSKYAGFTGVRLGWTVVPKALKYAN
GEPVHADWNRVMTTCFNGASNIVQAGGLACLQPEGLKEMNAMIKFYKENAQILKTTFTE
MGFSVYGGDDAPYIWVGFPGKPSWDVFAEILERCNIVTTPGSGFGPAGEGFVRASAFGSRE
NILEAVRRFKEAYGKRN
11111111111111111111111110000000000000000000000000000000000000000000000000000000000
00000000000000000000000000000000000000000000000000000000000000000000000000000000000
00000000000000000000000000000000000000000000000000000000000000000000000000000000000
00000000000000000000000000000000000000000000000000000000000000000000000000000000000
00000000000000000000000000000000000000000000000000000000000000000000000000000000000
00000000000000000000000111
>Q8P3K2_2hxt_0_440
MRTIIALETHDVRFPTSRELDGSDAMNPDPDYSAAYVVLRTDGAEDLAGYGLVFTIGRGND
VQTAAVAALAEHVVGLSVDKVIADLGAFARRLTNDSQLRWLGPEKGVMHMAIGAVINAA

WDLAARAANKPLWRFIAELTPEQLVDTIDFRYLSDALTRDEALAILRDAQPQRAARTATLIE
QGYPAYTTSPGWLGYSDEKLVRLAKEAVADGFRTIKLKVGANVQDDIRRCRLARAAIGPDI
AMAVDANQRWDVGPAIDWMRQLAEFDIAWIEEPTSPDDVLGHAAIRQGITPVPVSTGEHT
QNRVVFKQLLQAGAVDLIQIDAARVGGVNENLAILLLAAKFGVRVFPHAGGVGLCELVQ
HLAMADFVAITGKMEDRAIEFVDHLHQHFLDPVRIQHGRYLAPEVPGFSAEMHPASIAEFS
YPDGRFWVEDLAASKAKA
10000000000000000000000000000000000000000000000000000000000000000000000000000000000
00000000000000000000000000000000000000000000000000000000000000000000000000000000000
00000000000000000000000000000000000000000000000000000000000000000000000000000000000
00000000000000000000000000000000000000000000000000000000000000000000000000000000000
00000000000000000000000000000000000000000000000000000000000000000000000000000000000
00000000000000000000111111
>B4EL89_2y4o_0_439
MTHPTHPAAALEPIETASRDELQALQLERLKWSLRHAYDNVPHYRRTFDAAGVHPDDLKS
LADLAKFPFSTKNDLRDNYPFGLFAVPREQVVRVHASSGTTGKPTVVGYTARDIDTWANVT
ARSIRAAGGRPGDTLHNAFGYGLFTGGLGIHYGAERLGCMVVPMSGGQTEKQVQLIRDFE
PKIILVTPSYMLNLIDEMVRQGMDPAESSLKIGIFGAEPWTQALRNEVETRVGIDALDIYGLS
EVMGPGVACECVETKDGPVIWEDHFYPEIIDPVTGEVLPDGSQGELVFTSLTKEAMPVIRYR
TRDLTALLPPTARAMRRLAKITGRSDDMLIVRGVNVFPSQIEEIVVALPLLSGQFQITLSRDG
HMDRLDLAVELRSEAAASVTDGERAALARELQHRIKTMVGVSSGVTVLAAGGIPATATGK
ARRVIDRRQAA
11111000000000000000000000000000000000000000000000000000000000000000000000000000000
00000000000000000000000000000000000000000000000000000000000000000000000000000000000
00000000000000000000000000000000000000000000000000000000000000000000000000000000000
00000000000000000000000000000000000000000000000000000000000000000000000000000000000
00000000000000000000000000000000000000000000000000000000000000000000000000000000000
0000000000000000000000011
>P27395_2z83_1684_2122
PNMLRKRQMTVLDLHPGSGKTRKILPQIIKDAIQQRLRTAVLAPTRVVAAEMAEALRGLPV
RYQTSAVQREHQGNEIVDVMCHATLTHRLMSPNRVPNYNLFVMDEAHFTDPASIAARGYI
ATKVELGEAAAIFMTATPPGTTDPFPDSNAPIHDLQDEIPDRAWSSGYEWITEYAGKTVWF
VASVKMGNEIAMCLQRAGKKVIQLNRKSYDTEYPKCKNGDWDFVITTDISEMGANFGASR
VIDCRKSVKPTILEEGEGRVILGNPSPITSASAAQRRGRVGRNPNQVGDEYHYGGATSEDDS
NLAHWTEAKIMLDNIHMPNGLVAQLYGPEREKAFTMDGEYRLRGEEKKNFLELLRTADLP
VWLAYKVASNGIQYTDRKWCFDGPRTNAILEDNTEVEIVTRMGERKILKPRWLDARVYAD
HQALKWFKDFAAGKR
00000000000000000000000000000000000000000000000000000000000000000111111110000000000
00000000001000000000000000000000000000000000000000000000000000000000000000000000000
00000000000000000000000000000000000000000000000000000000000000011100000000000000000
00000000000000000000000000000000000000000000000000000000000000000000000000000000000
00000000000000000000000000000000000000000000000000000000000000000000000000000000000
000000000000000000000001
>Q9WZI6_1vl4_0_434
MTFEEFKDRLFALAKKNGVEVQISFLETREFSLRLANGDLDQYTDAGKFNVEIKVLKDGKT
GTFRTQVLENPEKCFEEALSNLQVKDSEEKEYFFEGGKEYREMETYVGRFEKLSVKEKMDM
AKKAHESAAKDERVVMVPTVMYKDMVIKKIITNTLGLDVESQMDGGFLFAMAIARDANP
RSGSWYELARTPEDLNPEEIGKRAAEEAISLIGSKTIPSGKYPVLMRNTALLDLMEMFIPMIS
AENVQKNLSPLKGKLGEQVGNPAVSIKDLPYHPKGLSSTPFDDEGVPTTEKFVLENGVLKT
FLHNLKTARKEGVEPTGNGFVGGIRPVNLMLMPGEKSFEELLKEMDRGVVITEVEGMHAG
ANSISGEFSLFAKGYWVENGEIAHGVEDITISGNFLDLLRKIVLVGNDVKVSQHTIAPSVLVE
VLDVAGK

00000000000000000000000000000000000000000000000000000000000000000000000000000000000
00011110000000000000000000000000000000000000000000000000000000000000000000000000000
00000000000000000000000000000000000000000000000000000000000000000000000000000000000
00000000000000000000000000000000000000000000000000000000000000000000000000000000000
00000000000000000000000000000000000000000000000000000000000000000000000000000000000
00000000000000000011
>O57980_1x56_0_433
MIEKVYCQEVKPELDGKKVRLAGWVYTNMRVGKKIFLWIRDSTGIVQAVVAKNVVGEETF
EKAKKLGRESSVIVEGIVKADERAPGGAEVHVEKLEVIQAVSEFPIPENPEQASPELLLDYRH
LHIRTPKASAIMKVKETLIMAAREWLLKDGWHEVFPPILVTGAVEGGATLFKLKYFDKYAY
LSQSAQLYLEAAIFGLEKVWSLTPSFRAEKSRTRRHLTEFWHLELEAAWMDLWDIMKVEEE
LVSYMVQRTLELRKKEIEMFRDDLTTLKNTEPPFPRISYDEAIDILQSKGVNVEWGDDLGAD
EERVLTEEFDRPFFVYGYPKHIKAFYMKEDPNDPRKVLASDMLAPEGYGEIIGGSQREDDY
DKLLNRILEEGMDPKDYEWYLDLRRYGSVPHSGFGLGVERLVAWVLKLDHIRWAALFPRT
PARLYP
00000000000000000000000000000000000000000000000000000000000000000000000000000000000
00000000000000000000000000000000000000000000000000000000000000000000000000000000011
11111100000000000000000000000000000000000000000111111000000000000000000000000000000
00000000000000000000000000000000000000000000000000000000000000000000000000000000000
00000000000000000000000000000000000000000000000000000000000000000000000000000000000
0000000000000000000
>P20901_2h12_0_435
MSASQKEGKLSTATISVDGKSAEMPVLSGTLGPDVIDIRKLPAQLGVFTFDPGYGETAACNS
KITFIDGDKGVLLHRGYPIAQLDENASYEEVIYLLLNGELPNKVQYDTFTNTLTNHTLLHEQ
IRNFFNGFRRDAHPMAILCGTVGALSAFYPDANDIAIPANRDLAAMRLIAKIPTIAAWAYK
YTQGEAFIYPRNDLNYAENFLSMMFARMSEPYKVNPVLARAMNRILILHADHEQNASTST
VRLAGSTGANPFACIAAGIAALWGPAHGGANEAVLKMLARIGKKENIPAFIAQVKDKNSG
VKLMGFGHRVYKNFDPRAKIMQQTCHEVLTELGIKDDPLLDLAVELEKIALSDDYFVQRKL
YPNVDFYSGIILKAMGIPTSMFTVLFAVARTTGWVSQWKEMIEEPGQRISRPRQLYIGAPQR
DYVPLAKR
11111111110000000000000000000000000000000000000000000000000000000000000000000000000
00000000000000000000000000000000000000000000000000000000000000000000000000000000000
00000000000000000000000000000000000000000000000000000000000000000000000000000000000
00000000000000000000000000000000000000000000000000000000000000000000000000000000000
00000000000000000000000000000000000000000000000000000000000000000000000000000000000
000000000000000000000
>Q7CLE1_3lg3_0_434
MTISRTQQIQQLEQEWTSPRWKNITRPYSAEDVIKLRGSVNPECTFAQNGAKKLWELLHGG
SRKGYINCLGALTGGQALQQAKAGVEAIYMSGWQVAADANTASSMYPDQSLYPVDSVPA
VVKRINNSFRRADQIQWSNNIEPGSKGYTDYFLPIVADAEAGFGGVLNAFELMKAMIEAG
AAGVHFEDQLAAVKKCGHMGGKVLVPTQEAIQKLVAARLAADVLGVPTLLIARTDADAA
DLLTSDCDPYDREFITGDRTAEGFFRTRAGIEQAISRGLAYAPYADLVWCETSTPDLALAKRF
ADAVHAQFPGKLLAYNCSPSFNWKKNLTDQQIASFQDELSAMGYKYQFITLAGIHSMWFN
MFDLAHAYAQGEGMKHYVEKVQQPEFASVDRGYTFASHQQEVGTGYFDKVTNIIQGGAS
SVTALTGSTEEQQF
00000000000000000000000000000000000000000000000000000000000000000000000000000000000
00000000000000000000000000000000000000000000000000000000000000000000000000000000000
00000000000000000000000000000000000000000000000000000000000000000000000000000000000
00000000000000000000000000000000000000000000000000000000000000000000000000000000000
00000000000000000000000000000000000000000000000000000000000000000000000000000000000
00001111111111111111

>P15640_1gso_0_428
MKVLVIGNGGREHALAWKAAQSPLVETVFVAPGNAGTALEPALQNVAIGVTDIPALLDFA
QNEKIDLTIVGPEAPLVKGVVDTFRAAGLKIFGPTAGAAQLEGSKAFTKDFLARHKIPTAEY
QNFTEVEPALAYLREKGAPIVIKADGLAAGKGVIVAMTLEEAEAAVHDMLAGNAFGDAG
HRIVIEEFLDGEEASFIVMVDGEHVLPMATSQDHKRVGDKDTGPNTGGMGAYSPAPVVTD
DVHQRTMERIIWPTVKGMAAEGNTYTGFLYAGLMIDKQGNPKVIEFNCRFGDPETQPIML
RMKSDLVELCLAACESKLDEKTSEWDERASLGVVMAAGGYPGDYRTGDVIHGLPLEEVAG
GKVFHAGTKLADDEQVVTNGGRVLCVTALGHTVAEAQKRAYALMTDIHWDDCFCRKDI
GWRAIEREQN
00000000000000000000000000000000000000000000000000000000000000000000000000000000000
00000000000000000000000000000000000000000000000000000000000000011111100000000000000
00000000000000000000000000000000000000000000000000000000000000000000000000000000000
00000000000000000000000000000000000000000000000000000000000000000000000000000000000
00000000000000000000000000000000000000001110000000000000000000000000000000000000000
00000000000111
>P73452_2g29_29_445
TSPTTTSTGTGTGSSTDQAISPLVEGENAPEVTTAKLGFIALTDAAPLIIAKEKGFYAKYGMP
DVEVLKQASWGTTRDNLVLGSASGGIDGAHILTPMPYLITMGTVTDGKPTPMYILARLNV
NGQGIQLGNNYKDLKVGTDAAPLKEAFAKVTDPKVAMTFPGGTHDMWIRYWLAAGGM
EPGKDFSTIVVPPAQMVANVKVNAMESFCVGEPWPLQTVNQGVGYQALTTGQLWKDHPE
KAFGMRADWVDQNPKAAKALLMAVMEAQQWCDQAENKEEMCQILSKREWFKVPFEDI
IDRSKGIYNFGNGQETFEDQEIMQKYWVDNASYPYKSHDQWFLTENIRWGYLPASTDTKAI
VDKVNREDLWREAAQALEVPADQIPSSPSRGIETFFDGITFDPENPQAYLDSLKIKSIKA
11111111111111111111111111100000000000000000000000000000000000000000000000000000000
00000000000000000000000000000000000000000000000000000000000000000000000000000000000
00000000000000000000000000000000000000000000000000000000000000000000000000000000000
00000000000000000000000000000000000000000000000000000000000000000000000000000000000
00000000000000000000000000000000000000000000000000000000000000000000000000000000111
11
>D0AB09_2wuu_0_420
MSRNPSNSDAAHAFWSTQPVPQTEDETEKIVFAGPMDEPKTVADIPEEPYPIASTFEWWTP
NMEAADDIHAIYELLRDNYVEDDDSMFRFNYSEEFLQWALCPPSYIPDWHVAVRRKADKK
LLAFIAGVPVTLRMGTPKYMKVKAQEKGQEEEAAKYDAPRHICEINFLCVHKQLREKRLA
PILIKEVTRRVNRTNVWQAVYTAGVLLPTPYASGQYFHRSLNPEKLVEIRFSGIPAQYQKFQN
PMAMLKRNYQLPNAPKNSGLREMKPSDVPQVRRILMNYLDNFDVGPVFSDAEISHYLLPR
DGVVFTYVVENDKKVTDFFSFYRIPSTVIGNSNYNILNAAYVHYYAATSMPLHQLILDLLIV
AHSRGFDVCNMVEILDNRSFVEQLKFGAGDGHLRYYFYNWAYPKIKPSQVALVML
11111111110000000000000000000000000000000000000000000000000000000000000000000000001
11000000000000000000000000000000000000000000000000000000000000000000000000000000000
00000000000000000000000000000000000000000000000000000000000000000000000000000000000
00000000000000000000000000000000000000000000000000000000000000000000000000000000000
01111110000000000000000000000000000000000000000000000000000000000000000000000000000
000000
>P39116_2bsp_0_419
MKKVMLATALFLGLTPAGANAADLGHQTLGSNDGWGAYSTGTTGGSKASSSNVYTVSNR
NQLVSALGKETNTTPKIIYIKGTIDMNVDDNLKPLGLNDYKDPEYDLDKYLKAYDPSTWGK
KEPSGTQEEARARSQKNQKARVMVDIPANTTIVGSGTNAKVVGGNFQIKSDNVIIRNIEFQ
DAYDYFPQWDPTDGSSGNWNSQYDNITINGGTHIWIDHCTFNDGSRPDSTSPKYYGRKYQ
HHDGQTDASNGANYITMSYNYYHDHDKSSIFGSSDSKTSDDGKLKITLHHNRYKNIVQRA
PRVRFGQVHVYNNYYEGSTSSSSYPFSYAWGIGKSSKIYAQNNVIDVPGLSAAKTISVFSGGT
ALYDSGTLLNGTQINASAANGLSSSVGWTPSLHGSIDASANVKSNVINQAGAGKLN

11111111111111111111100000000000000000000000000000000000000000000000000000000000000
00000000000000000000000000000000000000000000000000000000000000000000000000000000000
00000000000000000000000000000000000000000000000000000000000000000000000000000000000
00000000000000000000000000000000000000000000000000000000000000000000000000000000000
00000000000000000000000000000000000000000000000000000000000000000000000000000000000
00000
>Q8U8Z6_2puz_0_418
MPGNNSAKGTATGNATALWRNAQLATLNPAMDGIGAVENAVIAVRNGRIAFAGPESDLP
DDLSTADETTDCGGRWITPALIDCHTHLVFGGNRAMEFEMRLNGATYEEIAKAGGGIVSSV
RDTRALSDEVLVAQALPRLDTLLSEGVSTIEIKSGYGLDIETELKMLRVARRLETLRPVRIVTS
YLAAHATPADYKGRNADYITDVVLPGLEKAHAEGLADAVDGFCEGIAFSVKEIDRVFAAA
QQRGLPVKLHAEQLSNLGGAELAASYNALSADHLEYLDETGAKALAKAGTVAVLLPGAF
YALREKQLPPVQALRDAGAEIALATDCNPGTSPLTSLLLTMNMGATLFRMTVEECLTATTR
NAAKALGLLAETGTLEAGKSADFAIWDIERPAELVYRIGFNPLHARIFKGQKVSP
11111111111111000000000000000000000000000000000000000000000000000000000000000000000
00000000000000000000000000000000000000000000000000000000000000000000000000000000000
00000000000000000000000000000000000000000000000000000000000000000000000000000000000
00000000000000000000000000000000000000000000000000000000000000000000000000000000000
00000000000000000000000000000000000000000000000000000000000000000000000000000000000
0001
>Q92ZP9_3vca_0_411
MTANPTSIHQRLDRRLSGFSLEQPFYTSPEVYALDLQHIFYKQWLYAVPVCQLAKAGSYTTL
RVGAYEVVIVRSRDGEVRAFHNSCRHRGSLICKARQGQVAKLVCPYHQWTYELDGKLIWA
NDMGPDFDASKYGLKPVNLRNLDGLIYICLSDTPPDFQTFAQLARPYLEVHDLKDAKVAFT
STIIEKGNWKLVWENNRECYHCSSNHPALCRSFPLDPEVAGVQADGGVSKKLQAHFDRCE
AAGTPAQFVLAGDGQYRLARMPLQEKALSYTMDGKAAVSRHLGRVAPPDAGTLLMFHYP
STWNHFLPDHSLTFRVMPISPTETEVTTTWLVHKDAVEGVDYDLKRLTEVWIATNDEDREI
VETNQQGILSPAYVPGPYSPGQESGVMQFVDWYAASLERALAPRQVAAE
11111000000000000000000000000000000000000000000000000000000000000000000000000000000
00000000000000000000000000000000000000000000000000000000000000000000000000000000000
00000000000000000000000000000000000000000000000000000000000111100000000000000000000
00000000000000000000000000000000000000000000000000000000000000000000000000000000000
00000000000000000000000000000000000000000000000000000000000000000000000000111111
>P29717_2pbo_38_437
GGGHNVAWDYDNNVIRGVNLGGWFVLEPYMTPSLFEPFQNGNDQSGVPVDEYHWTQTL
GKEAASRILQKHWSTWITEQDFKQISNLGLNFVRIPIGYWAFQLLDNDPYVQGQVQYLEKA
LGWARKNNIRVWIDLHGAPGSQNGFDNSGLRDSYNFQNGDNTQVTLNVLNTIFKKYGG
NEYSDVVIGIELLNEPLGPVLNMDKLKQFFLDGYNSLRQTGSVTPVIIHDAFQVFGYWNNF
LTVAEGQWNVVVDHHHYQVFSGGELSRNINDHISVACNWGWDAKKESHWNVAGEWSA
ALTDCAKWLNGVNRGARYEGAYDNAPYIGSCQPLLDISQWSDEHKTDTRRYIEAQLDAFE
YTGGWVFWSWKTENAPEWSFQTLTYNGLFPQPVTDRQFPNQCGFH
11111100000000000000000000000000000000000000000000000000000000000000000000000000000
00000000000000000000000000000000000000000000000000000000000000000000000000000000000
00000000000000000000000000000000000000000000000000000000000000000000000000000000000
00000000000000000000000000000000000000000000000000000000000000000000000000000000000
00000000000000000000000000000000000000000000000000000000000000000000
>P13917_3aup_24_426
VTPTKPINLVVLPVQNDGSTGLHWANLQKRTPLMQVPVLVDLNGNHLWVNCEQQYSSKT
YQAPFCHSTQCSRANTHQCLSCPAASRPGCHKNTCGLMSTNPITQQTGLGELGEDVLAIH
ATQGSTQQLGPLVTVPQFLFSCAPSFLVQKGLPRNTQGVAGLGHAPISLPNQLASHFGLQR
QFTTCLSRYPTSKGAIIFGDAPNNMRQFQNQDIFHDLAFTPLTITLQGEYNVRVNSIRINQHS

VFPLNKISSTIVGSTSGGTMISTSTPHMVLQQSVYQAFTQVFAQQLPKQAQVKSVAPFGLCF
NSNKINAYPSVDLVMDKPNGPVWRISGEDLMVQAQPGVTCLGVMNGGMQPRAEITLGA
RQLEENLVVFDLARSRVGFSTSSLHSHGVKCADLFNFANA
11111000000000000000000000000000000000000000000000000000000000000000000000000000
00000000000000000000000000000000000011111100000000000000000000000000000000000000
00000000000000000000000000000000000001111110000000000000000000000000000000000011
11000000000000000000000000000000000000000000000000000000000000000000000000000000
00000000000000000000000000000000000000000000000000000000000000000011
>F9VMT6_3r1m_0_383
MKTTISVIKADIGSLAGHHIVHPDTMAAANKVLASAKEQGIILDYYITHVGDDLQLIMTHT
RGELDTKVHETAWNAFKEAAKVAKDLGLYAAGQDLLSDSFSGNVRGLGPGVAEMEIEERA
SEPIAIFMADKTEPGAYNLPLYKMFADPFNTPGLVIDPTMHGGFKFEVLDVYQGEAVMLSA
PQEIYDLLALIGTPARYVIRRVYRNEDNLLAAVVSIERLNLIAGKYVGKDDPVMIVRLQHGL
PALGEALEAFAFPHLVPGWMRGSHYGPLMPVSQRDAKATRFDGPPRLLGLGFNVKNGRLV
GPTDLFDDPAFDETRRLANIVADYMRRHGPFMPHRLEPTEMEYTTLPLILEKLKDRFKKESD
VYKAKESIYAKEESQGHD
00000000000000000000000000000000000000000000000000000000000000000000000000000000
00000000000000000000000000000000000000000000000000000000000000000000000000000000
00000000000000000000000000000000000000000000000000000000000000000000000000000000
00000000000000000000000000000000000000000000000000000000000000000000000000000000
0000000000000000000011111110000111111111111111111111
>Q8E372_3anj_0_397
MMKIKPVKVESIENPKRFLNSRLLTKIEVEEAIEKALKQLYINIDYFGEEYPTPATFNNIYKVM
DNTEWTNGFWTGCLWLAYEYNQDKKLKNIAHKNVLSFLNRINNRIALDHHDLGFLYTPS
CTAEYRINGDVKALEATIKAADKLMERYQEKGGFIQAWGELGYKEHYRLIIDCLLNIQLLFF
AYEQTGDEKYRQVAVNHFYASANNVVRDDSSAFHTFYFDPETGEPLKGVTRQGYSDESSW
ARGQAWGIYGIPLSYRKMKDYQQIILFKGMTNYFLNRLPEDKVSYWDLIFTDGSGQPRDTS
ATATAVCGIHEMLKYLPEVDPDKETYKYAMHTMLRSLIEQYSNNELIAGRPLLLHGVYSWH
SGKGVDEGNIWGDYYYLEALIRFYKDWELYW
10000000000000000000000000000000000000000000000000000000000000000000000000000000
00000000000000000000000000000000000000000000000000000000000000001111111111111111
11111000000000000000000000000000000000000000000000000000000000000000000000000000
00000000000000000000000000000000000000000000000000000000000000000000000000000000
0000000000000000000000000000000000000000000000000000000000000000
>Q8EJW4_2pvz_0_396
MSNKLFPPQIKVAATYMRGGTSKGVFFRLQDLPEAAQVPGPARDALLLRVIGSPDPYAKQI
DGMGGATSSTSKTVILSHSSKANHDVDYLFGQVSIDKPFVDWSGNCGNLTAAVGAFAISNG
LIDAARIPRNGVCTVRIWQANIGKTIIAHVPITDGAVQETGDFELDGVTFPAAEVQIEFMNP
AADDDGEGGCMFPTGNLVDVLEVPGIGRFNATMINAGIPTIFINAEDLGYTGTELQDDINS
DNAALAKFETIRAHGALRMGLIKHIDEAASRQHTPKIAFVAPPKSYASSSGKTVAAEDVDL
LVRALSMGKLHHAMMGTAAVAIGTAAAIPGTLVNLAAGGGEKEAVRFGHPSGTLRVGAQ
AVQENGEWTVIKAIMSRSARVLMEGFVRVPKP
11110000000000000000000000000000000000000000000000000000000000000000000000000000
00000000000000000000000000000000000000000000000000000000000000000000000000000000
00000000000000000000011111100000000000000000000000000000000000000000000000000000
00000000000000000000000000000000000000000000000000000000000000000000000000000000
000000000000000000000000000000000000000000000000000000000000000
>Q99JF5_3f0n_5_400
PQDLMVTCTAPVNIAVIKYWGKRDEALILPINSSLSVTLHQDQLKTTTTVAISKDFTEDRIWL
NGREEDVGQPRLQACLREIRRLARKRRSTEDGDTLPLSLSYKVHVASVNNFPTAAGLASSA
AGYACLAYTLAQVYGVEGDLSEVARRGSGSACRSLYGGFVEWQMGEQADGKDSIARQIAP

EWHWPQLRILILVVSADKKQTGSTVGMQTSVETSTLLKFRAESVVPERMKEMTRCIQEQDF
QGFAQLTMKDSNQFHATCLDTFPPISYLNDTSRRIIQLVHRFNTHQGQTKVAYTFDAGPNA
VIFTLEDTVAEFVAAVRHSFPPAANGDKFLKGLQVAPVLLSDELKAALAVEPSPGGVQYIIAT
QVGPGPQVLDDTHDHLLGQDGLPQRDL
1100000000000000000000000000000000000000000000000000000000000000000000000000000000
0000000111110000000000000000000000000000000000000000000000000000000000000000000000
00000000000000000000000000000000000000000000000000000000000000000000000000000000000
00000000000000000000000000000000000000000000000000000000000000000000000000000000001
1000000000000000000000000000000000000000000000000000000000000111
>Q9IGQ6_3cye_82_468
VILTGNSSLCPISGWAIYSKDNGIRIGSKGDVFVIREPFISCSHLECRTFFLTQGALLNDKHSN
GTVKDRSPYRTLMSCPVGEAPSPYNSRFESVAWSASACHDGMGWLTIGISGPDNGAVAVLK
YNGIITDTIKSWRNNILRTQESECACVNGSCFTIMTDGPSNGQASYKILKIEKGKVTKSIELN
APNYHYEECSCYPDTGKVMCVCRDNWHGSNRPWVSFDQNLDYQIGYICSGVFGDNPRPN
DGTGSCGPVSSNGANGIKGFSFRYDNGVWIGRTKSTSSRSGFEMIWDPNGWTETDSSFSVR
QDIVAITDWSGYSGSFVQHPELTGLDCMRPCFWVELIRGQPKENTIWTSGSSISFCGVNSDT
VGWSWPDGAELPFSIDK
00000000000000000000000000000000000000000000000000000000000000000000000000000000000
00000000000000000000000000000000000000000000000000000000000000000000000000000000000
00000000000000000000000000000000000000000000000000000000000000000000000000000000000
00000000000000000000000000000000000000000000000000000000000000000000000000000000000
0000000000000000000000000000000000000000000000000000011
>A6LDR8_3mdo_0_387
MSDQRYNLRGVSASKEDVHNAIKNIDKGIFPKAFCKIIPDILGGDPEYCNIMHADGAGTKS
SLAYMYWKETGDLSVWKGIAQDALIMNIDDLLCVGAVDNILVSSTIGRNKLLVPGEVISAII
NGTDELLAELREMGVGCYATGGETADVGDLVRTIIVDSTVTCRMKRSDVIDNKNIQGGDVI
VGLASSGQATYEKEYNGGMGSNGLTSARHDVFSKYLAKKYPESYDAAVPKELVYSGGLKLT
DKIEELGIDAGKMVLSPTRTYAPVIKVLLDKLRSQIHGMVHCSGGAQTKVMHFVENKRVT
KDNLFPIPPLFRTIQEQSGTDWSEMYKVFNMGHRMEIYIAPEHAEEVIGISKSFGIDAQIVGF
VEEADKNELIIESEKGRFTY
11111111111111111111111000000000000000000000000000000000000000000000000000000000000
00000000000000000000000000000000000000000000000000000000000000000000000000000000000
00000000000000000000000000000000000000000000000000000000000000000000000000000000000
00000000000000000000000000000000000000000000000000000000000000000000000000000000000
00000000000000000000000000000000000000000000000000000000
>P07062_1wyb_5_391
TGSHPARYPSAAAGEPTLDSWQEPPHNRWAFAHLGEMVPSAAVSRRPVNAPGHALARLG
AIAAQLPDLEQRLEQTYTDAFLVLRGTEVVAEYYRAGFAPDDRHLLMSVSKSLCGTVVGAL
VDEGRIDPAQPVTEYVPELAGSVYDGPSVLQVLDMQISIDYNEDYVDPASEVQTHGRSAGW
RTRATGDPADTYEFLTTLRGDGSTGEFQYCSANTDVLAWIVERVTGLRYVEALSTYLWAKL
DADRDATITVDTTGFGFAHGGVSCTARDLARVGRMMLDGGVAPGGRVVSEDWVRRVLA
GGSHEAMTDKGFTNTFPDGSYTRQWWCTGNERGNVSGIGIHGQNLWLDPLTDSVIVKLSS
WPDPDTEHWHRLQNGILLDVSRALDAV
00000000000000000000000000000000000000000000000111100000000000000000000000000000000
00000000000000000000000000000000000000000000000000000000000000000000000000000000111
11100000000000000000000000000000000000000000000000000000000000000000000000000000000
00000000000000000000000000000000000000000000000000000000000000000000000000000000000
0000000000000000000000000000000000000000000000000000000
>Q13838_1xti_43_427
SSGFRDFLLKPELLRAIVDCGFEHPSEVQHECIPQAILGMDVLCQAKSGMGKTAVFVLATLQ
QLEPVTGQVSVLVMCHTRELAFQISKEYERFSKYMPNVKVAVFFGGLSIKKDEEVLKKNCP

HIVVGTPGRILALARNKSLNLKHIKHFILDECDKMLEQLDMRRDVQEIFRMTPHEKQVMM
FSATLSKEIRPVCRKFMQDPMEIFVDDETKLTLHGLQQYYVKLKDNEKNRKLFDLLDVLEF
NQVVIFVKSVQRCIALAQLLVEQNFPAIAIHRGMPQEERLSRYQQFKDFQRRILVATNLFGR
GMDIERVNIAFNYDMPEDSDTYLHRVARAGRFGTKGLAITFVSDENDAKILNDVQDRFEV
NISELPDEIDISSYIEQTR
1100000000000000000000000000000000000000000000000000000000000000000000000000000000
00000000000000000000000000000000000000000000000000000000000000000000000000000000000
00000000000000000000000000000000000000000000000000000000000000000000000000000000000
00000000000000000000000000000000000000000000000000000000000000000000000000000000000
00000000000000000000000000000000000000000000000000011
>A8LS88_3sqs_0_390
MTKIKSVRTRVWNWTGPTVPPQGNFCTNASDALWIQGDAMASFRFHQWLTCEVETEDGT
IGIGNAALAPNVVKQAIDEWYAPLVIGEDPFDYAYLWEKMYRRTHAWGRKGIGMTAISAID
IAIWDLMGKLVGKPVFKLLGGRTKEKIPVYYSKLYADSIPAMQAEAEEAQKHGYQGYKTRF
GYGPKDGPAGMRENLKRVEALREVLGYDVDLMLECYMGWNLDYTKRMLPKLERFEPRW
LEEPVIADDVAGYAELNAMGIVPISGGEHEFSVMGCAELINRKAVSVLQYDTNRVGGITAA
QKINAIAEAAQIIVIPHAGQMHNYHLTMANMNCPISEYFPVFDVEVGNELFYYIFDGDPEA
VDGYLQLDDDTPGLGITISDAHLKHFEITE
00000000000000000000000000000000000111110000000000000000000000000000000000000000000
00000000000000000000000000000000000000000000000000000000000000000000000000000000000
00000000000000000000000000000000000000000000000000000000000000000000000000000000000
00000000000000000000000000000000000000000000000000000000000000000000000000000000000
00000000000000000000000000000000000000000000000000000000000
>Q8DUI5_3sqz_0_390
MRIGIDKIGFTSSQYVLNMKDLAEARGEDPQKFSKGLLLNALSIAPITDDVVTLAAGSANEI
LTAEDKEKIDMVILATESSVDQSKAGAVYVHSLLGIQPFARSFEMKEACYSATAALNYAKLH
VEKHPDTRVLVLASDIAKYGIGTPGESTQGAGSIAMLVKKDPRILILHDETLAQTRDIMDFW
RPNYTTTPYVNGMYSTKQYLDMLKTTWAEYQKRFDVSLTDFAAFCFHLPFPKLALKGFNKI
MDKQVPSDLQEKLKVNFEASILYSKQIGNIYTGSLFLGLLSLLENSQNLVAGDKIALFSYGSG
AVAEIFTGTLVKGFKEQLQTNRLDKLKRRTPLSVENYEKIFFEEAQLDDKGNASFKEYQTGP
FALKEILEHQRIYGKVNES
00000000000000000000000000000000000000000000000000000000000000000000000000000000000
00000000000000000000000000000000000000000000000000000000000000000000000000000000000
00000000000000000000000000000000000000000000000000000000000000000000000000000000000
00000000000000000000000000000000000000000000000000000000000000000000000000000000000
00000000000000000000000000000000000000000000000000000000111
>Q9ALM8_3tsa_0_389
MRVLVVPLPYPTHLMAMVPLCWALQASGHEVLIAAPPELQATAHGAGLTTAGIRGNDRT
GDTGGTTQLRFPNPAFGQRDTEAGRQLWEQTASNVAQSSLDQLPEYLRLAEAWRPSVLLV
DVCALIGRVLGGLLDLPVVLHRWGVDPTAGPFSDRAHELLDPVCRHHGLTGLPTPELILDP
CPPSLQASDAPQGAPVQYVPYNGSGAFPAWGAARTSARRVCICMGRMVLNATGPAPLLR
AVAAATELPGVEAVIAVPPEHRALLTDLPDNARIAESVPLNLFLRTCELVICAGGSGTAFTAT
RLGIPQLVLPQYFDQFDYARNLAAAGAGICLPDEQAQSDHEQFTDSIATVLGDTGFASAAI
KLSDEITAMPHPAALVRTLENTAAIRA
00000000000000000000000000000000000000000000000000000001111111111110000000000000000
00000000000000000000000000000000000000000000000000000000000000000000000000000000000
00000000000000000000000000000000000000000000000000000000000000000000000000000000000
00000000000000000000000000000000000000000000000000000000000000000000000000000000000
0000000000000000000000000000000000000000000000000000011111
>P42907_3c3j_0_383
MPENYTPAAAATGTWTEEEIRHQPRAWIRSLTNIDALRSALNNFLEPLLRKENLRIILTGAG

TSAFIGDIIAPWLASHTGKNFSAVPTTDLVTNPMDYLNPAHPLLLISFGRSGNSPESVAAVEL
ANQFVPECYHLPITCNEAGALYQNAINSDNAFALLMPAETHDRGFAMTSSITTMMASCLA
VFAPETINSQTFRDVADRCQAILTSLGDFSEGVFGYAPWKRIVYLGSGGLQGAARESALKVL
ELTAGKLAAFYDSPTGFRHGPKSLVDDETLVVVFVSSHPYTRQYDLDLLAELRRDNQAMRV
IAIAAESSDIVAAGPHIILPPSRHFIDVEQAFCFLMYAQTFALMQSLHMGNTPDTPSASGTVN
RVVQGVIIHPWQA
11110000000000000000000000000000000000000000000000000000000000000000000000000000000
00000000000000000000000000000000000000000000000000000000000000000000000000000000000
00000000000000000000000000000000000000000000000000000000000000000000000000000000000
00000000000000000000000000000000000000000000000000000000000000000000000000000000000
0000000000000000000000000000000011111111111000000000
>P28720_4hvx_0_385
MVEATAQETDRPRFSFSIAAREGKARTGTIEMKRGVIRTPAFMPVGTAATVKALKPETVRAT
GADIILGNTYHLMLRPGAERIAKLGGLHSFMGWDRPILTDSGGYQVMSLSSLTKQSEEGVT
FKSHLDGSRHMLSPERSIEIQHLLGSDIVMAFDECTPYPATPSRAASSMERSMRWAKRSRDA
FDSRKEQAENAALFGIQQGSVFENLRQQSADALAEIGFDGYAVGGLAVGEGQDEMFRVLD
FSVPMLPDDKPHYLMGVGKPDDIVGAVERGIDMFDCVLPTRSGRNGQAFTWDGPINIRNA
RFSEDLTPLDSECHCAVCQKWSRAYIHHLIRAGEILGAMLMTEHNIAFYQQLMQKIRDSISE
GRFSQFAQDFRARYFARNS
11111111100000000000000000000000000000000000000000000000000000000000000000000000000
00000000000000000000000000111111100000000111111110000000000000000000000000000000000
00000000000000000000000000000000000000000000000000000000000000000000000000000000000
00000000000000000000000000000000000000000000000000000000000000000000000000000000000
00000000000000000000000000000000000000000000000000111
>P71703_1gr0_0_366
MSEHQSLPAPEASTEVRVAIVGVGNCASSLVQGVEYYYNADDTSTVPGLMHVRFGPYHVR
DVKFVAAFDVDAKKVGFDLSDAIFASENNTIKIADVAPTNVIVQRGPTLDGIGKYYADTIEL
SDAEPVDVVQALKEAKVDVLVSYLPVGSEEADKFYAQCAIDAGVAFVNALPVFIASDPVW
AKKFTDARVPIVGDDIKSQVGATITHRVLAKLFEDRGVQLDRTMQLNVGGNMDFLNMLE
RERLESKKISKTQAVTSNLKREFKTKDVHIGPSDHVGWLDDRKWAYVRLEGRAFGDVPLNL
EYKLEVWDSPNSAGVIIDAVRAAKIAKDRGIGGPVIPASAYLMKSPPEQLPDDIARAQLEEFI
IG
11111111111110000000000000000000000000000000000000000000000000000000000000000000000
00000000000000000000000000000000000000000000000000000000000000000000000000000000000
00000000000000000000000000000000000000000000000000000000000000000000000000011111111
11111111111111111100000000000000000000000000000000000000000000000000000000000000000
0000000000000000000000000000000000
>O87198_2ztj_0_375
MREWKIIDSTLREGEQFEKANFSTQDKVEIAKALDEFGIEYIEVTTPVASPQSRKDAEVLASL
GLKAKVVTHIQCRLDAAKVAVETGVQGIDLLFGTSKYLRAAHGRDIPRIIEEAKEVIAYIREA
APHVEVRFSAEDTFRSEEQDLLAVYEAVAPYVDRVGLADTVGVATPRQVYALVREVRRVVG
PRVDIEFHGHNDTGCAIANAYEAIEAGATHVDTTILGIGERNGITPLGGFLARMYTLQPEYV
RRKYKLEMLPELDRMVARMVGVEIPFNNYITGETAFSHKAGMHLKAIYINPEAYEPYPPEV
FGVKRKLIIASRLTGRHAIKARAEELGLHYGEEELHRVTQHIKALADRGQLTLEELDRILRE
WITA
00000000000000000000000000000000000000000000000000000000000000000000000000000000000
00000000000000111111110000000000000000000000000000000000000000000000000000000000000
00000000000000000000000000000000000000000000000000000000000000000000000000000000000
00000000000000000000000000000000000000000000000000000000000000000000000111111111111
1111111111111111111111111111111111111111111
>P83824_1v4v_0_375

MEGGMKRVVLAFGTRPEATKMAPVYLALRGIPGLKPLVLLTGQHREQLRQALSLFGIQEDR
NLDVMQERQALPDLAARILPQAARALKEMGADYVLVHGDTLTTFAVAWAAFLEGIPVGH
VEAGLRSGNLKEPFPEEANRRLTDVLTDLDFAPTPLAKANLLKEGKREEGILVTGQTGVDA
VLLAAKLGRLPEGLPEGPYVTVTMHRRENWPLLSDLAQALKRVAEAFPHLTFVYPVHLNP
VVREAVFPVLKGVRNFVLLDPLEYGSMAALMRASLLLVTDSGGLQEEGAALGVPVVVLRN
VTERPEGLKAGILKLAGTDPEGVYRVVKGLLENPEELSRMRKAKNPYGDGKAGLMVARG
VAWRLGLGPRPEDWLP
11100000000000000000000000000000000000000000000000000000000000000000000000000000000
00000000000000000000000000000000000000000000000000000000000000000000000000000000000
00000000000000000000000000000000000000000000000000000000000000000000000000000000000
00000000000000000000000000000000000000000000000000000000000000000000000000000000000
00000000000000000000000000000000000000000000
>Q59632_1b65_0_374
MTSQTPTRKPRARDLGLPFTGVTGPYNAITDVDGVGVGFQTIIENEPRPGRKRPARSGVTAI
LPHMQSETPVPVYAGVHRFNGNGEMTGTHWIEDGGYFLGPVVITNTHGIGMAHHATVR
WMVDRYASTYQTDDFLWIMPVVAETYDGALNDINGFPVTEADVRKALDNVASGPVQEGN
CGGGTGMITYGFKGGTGTASRVVEFGGRSFTIGALVQANHGQRDWLTIAGVPVGQHMRD
GTPQSQLQERGSIIVVLATDLPLMPHQLKRLARRASIGIGRNGTPGGNNSGDIFIAFSTANQR
PMQHRSAPFLDVEMVNDEPLDTVYLAAVDSVEEAVVNAMIAAEDMGGTPFDRLLVQAID
HERLRAVLRQYGRLA
11111111000000000000000000000000000000000000000000000000000000000000000000000000000
00000000000000000000000000000000000000000000000000000000000000000000000000000000000
00000000000000000000000000000000000000000000000000000000000000000000000000000001111
00000000000000000000000000000000000000000000000000000000000000000000000000000000000
0000000000000000000000000000000000000000000
>D2Z028_3vvm_0_373
MREFIPPASRFIELPDGFAMRRGGALYGARIAYETFGSLNAARDNAVLVLTGLSPDAHAASR
PDDPTPGWWEAMVGPGKPVDTDLWHVICVNSLGSCKGSTGPASTDPRTGEPYRLSFPELSI
EDIADAAAHTVRALGISRLACVVGASMGGMSALALLARHPELARTHISLSGAVHALPFSIA
VRSLQREAIRSDPGWLQGHYDEGEGPRRGMLTARKLGMMTYRSAQEWDCRFGRTRIGER
RRADQGRFGPEFEVESYLDFHAQRFADRFDPNSYLYLSHAMDQFDLGDGGGGGGAPGA
LSRMRVERALVMGARTDILFPLSQQQEIADGLSAGGADVSFLPVDTPAGHDAFLVDIERFGP
PVAKFLAIVA
00000000000000000000000000000000000000000000000000000000000000000000000000000000000
00000000000000000000000000000000000000000000000000000000000000000000000000000000000
00000000000000000000000000000000000000000000000000000000000000000000000000000111110
00000000000000000000000000000000000000000000000000000000000000000000000000000000000
000000000000000000000000000000000000000000
>Q9UJM8_2rdt_0_369
MLPRLICINDYEQHAKSVLPKSIYDYYRSGANDEETLADNIAAFSRWKLYPRMLRNVAETD
LSTSVLGQRVSMPICVGATAMQRMAHVDGELATVRACQSLGTGMMLSSWATSSIEEVAEA
GPEALRWLQLYIYKDREVTKKLVRQAEKMGYKAIFVTVDTPYLGNRLDDVRNRFKLPPQL
RMKNFETSTLSFSPEENFGDDSGLAAYVAKAIDPSISWEDIKWLRRLTSLPIVAKGILRGDDA
REAVKHGLNGILVSNHGARQLDGVPATIDVLPEIVEAVEGKVEVFLDGGVRKGTDVLKAL
ALGAKAVFVGRPIVWGLAFQGEKGVQDVLEILKEEFRLAMALSGCQNVKVIDKTLVRKNP
LAVSKI
00000000000000000000000000000000000000000000000000000000000000000000000000000000000
00000000000000000000000000000000000000000000000000000000000000000000000000000000000
00000000011111111111111111111111111110000000000000000000000000000000000000000000000
00000000000000000000000000000000000000000000000000000000000000000000000000000000000
00000000000000000000000000000001111111

>P32816_1jpu_0_369
MAAERVFISPAKYVQGKNVITKIANYLEGIGNKTVVIADEIVWKIAGHTIVNELKKGNIAAE
EVVFSGEASRNEVERIANIARKAEAAIVIGVGGGKTLDTAKAVADELDAYIVIVPTAASTDA
PTSALSVIYSDDGVFESYRFYKKNPDLVLVDTKIIANAPPRLLASGIADALATWVEARSVIKS
GGKTMAGGIPTIAAEAIAEKCEQTLFKYGKLAYESVKAKVVTPALEAVVEANTLLSGLGFES
GGLAAAHAIHNGFTALEGEIHHLTHGEKVAFGTLVQLALEEHSQQEIERYIELYLSLDLPVT
LEDIKLKDASREDILKVAKAATAEGETIHNAFNVTADDVADAIFAADQYAKAYKEKHRK
11000000000000000000000000000000000000000000000000000000000000000000000000000000000
00000000000000000000000000000000000000000000000011111000000000000000000000000000000
00000000000000000000000000000000000000000000000000000000000000000000000000000000000
00000000000000000000000000000000000000000000000000000000000000000000000000000000000
0000000000000000000000000000000000011
>Q7YXJ9_2bjr_0_367
MPNPPAKEDTWAFGPIGSPFPDNPVKALGQQNMYVALWYKNGRPMHGRAWNNGGVIEC
SFPYNKSELTGVKDLGGQIQVLQYKGNHLSLGYWYNWIKYSDRFDKMDKGAEMLRCGDS
FPILWSERPGGALLGYVDNKTEIARFSHDGKVDEVSGSALANMLIIVRELKGGPPYCECEEC
KSEPPKPIVRVTLNEWADFRCGDPWPTSGTPVRALGRSLDTLPGENPDQYVALWYQSGEPV
MGRIWNDGGKIAACFGWGGHEYRQKIGSIQVLYELPEAIRGFDYDWKPFPEAAQFGAKE
WIPVHVDHHKGNISPAVLIVDGKEILGKADIRNERATIGYGGTEKVLVGPAVHSCMVLCRK
AKPGCTID
11111000000000000000000000000000000000000000000000000000000000000000000000000000000
00000000000000000000000000000000000000000000000000000000000000000000000000000000000
00000000000000000000000000000000000000000000000000000000000000000000000000000000000
00000000000000000000000000000000000000000000000000000000000000000000000000000000000
00000000000000000000000000000000000
>B8ZY56_2ydp_0_364
KPAQKGKTFSNVEIFDPPTNYRDPQVLYARPLELSDGTLLGTWENYSPEPPNVWFPIVKSKD
GGKTWKEISKVKDTQNNWGLRYQPQLYELPRAFGKYPKGTVLCSGSSIPSDLSETLIEVYAS
RDKGYTWEFVSHVALGGEALPNPGLTPVWEPFLMTYKEKLILYYSDQRDNATHSQKLVHQ
TTTDLKKWSKVVDDTKYANYYARPGMPTVAKLPNNEYIYVYEYGGGPNPPAGSDYWFPV
YYRLSKDPQKFLNKAHHQIVSNDGTTPAGSPYVVWTPYGGKNGTIVVSCGTRSEIFTNQAL
GDASAWKKWDVPQPTAYTRSLLTFQKDPDLLMIMGAGILPPAGGKNTVSASVVRLSEVMK
S
11111000000000000000000000000000000000000000000000000000000000000000000000000000000
00000000000000000000000000000000000000000000000000000000000000000000000000000000000
00000000000000000000000000000000000000000000000000000000000000000000000000000000000
00000000000000000000000000000000000000000000000000000000000000000000000000000000000
00000000000000000000000000000001
>O13437_2j6i_1_363
KIVLVLYDAGKHAADEEKLYGCTENKLGIANWLKDQGHELITTSDKEGETSELDKHIPDAD
IIITTPFHPAYITKERLDKAKNLKLVVVAGVGSDHIDLDYINQTGKKISVLEVTGSNVVSVAE
HVVMTMLVLVRNFVPAHEQIINHDWEVAAIAKDAYDIEGKTIATIGAGRIGYRVLERLLPFN
PKELLYYDYQALPKEAEEKVGARRVENIEELVAQADIVTVNAPLHAGTKGLINKELLSKFKK
GAWLVNTARGAICVAEDVAAALESGQLRGYGGDVWFPQPAPKDHPWRDMRNKYGAGN
AMTPHYSGTTLDAQTRYAEGTKNILESFFTGKFDYRPQDIILLNGEYVTKAYGKHDKK
00000000000000000000000000000000000000000000000000000000000000000000000000000000000
00000000000000000000000000000000000000000000000000000000000000000000000000000000000
00000000000000000000000000000000000000000000000000000000000000000000000000000000000
00000000000000000000000000000000000000000000000000000000000000000000000000000000000
0000000000000000000011111111111
>A5KE01_3ewd_0_362

MNILQEPIDFLKKEELKNIDLSQMSKKERYKIWKRIPKCELHCHLDLCFSADFFVSCIRKYNL
QPNLSDEEVLDYYLFAKGGKSLGEFVEKAIKVADIFHDYEVIEDLAKHAVFNKYKEGVVLM
EFRYSPTFVAFKYNLDIELIHQAIVKGIKEVVELLDHKIHVALMCIGTGHEAANIKASADFCL
KHKADFVGFDHGGHEVDLKEYKEIFDYVRESGVPLSVHAGEDVTLPNLNTLYSAIQVLKVE
RIGHGIRVAESQELIDMVKEKNILLEVCPISNVLLKNAKSMDTHPIRQLYDAGVKVSVNSDD
PGMFLTNINDDYEELYTHLNFTLEDFMKMNEWALEKSFMDSNIKDKIKNLYF
11111100000000000000000000000000000000000000000000000000000000000000000000000000000
00000000000000000000000000000000000000000000000000000000000000000000000000000000000
00000000000000000000000000000000000000000000000000000000000000000000000000000000000
00000000000000000000000000000000000000000000000000000000000000000000000000000000000
000000000000000000000000000000
>Q9RME2_2big_0_360
MVKQVFNFNAGPSALPKPALERAQKELLNFNDTQMSVMELSHRSQSYEEVHEQAQNLLR
ELLQIPNDYQILFLQGGASLQFTMLPMNLLTKGTIGNYVLTGSWSEKALKEAKLLGETHIA
ASTKANSYQSIPDFSEFQLNENDAYLHITSNNTIYGTQYQNFPEINHAPLIADMSSDILSRPL
KVNQFGMIYAGAQKNLGPSGVTVVIVKKDLLNTKVEQVPTMLQYATHIKSDSLYNTPPTFSI
YMLRNVLDWIKDLGGAEAIAKQNEEKAKIIYDTIDESNGFYVGHAEKGSRSLMNVTFNLR
NEELNQQFLAKAKEQGFVGLNGHRSVGGCRASIYNAVPIDACIALRELMIQFKENA
11100000000000000000000000000000000000000000000000000000000000000000000000000000000
00000000000000000000000000000000000000000000000000000000000000000000000000000000000
00000000000000000000000000000000000000000000000000000000000000000000000000000000000
00000000000000000000000000000000000000000000000000000000000000000000000000000000000
00000000000000000000000000000
>D0CB74_3zpg_0_360
MSELKQDQYWMQQAIELAKRGLYSTKPNPNVGCVIVKDDQLIGEGFHPKAGQPHAEVFA
LRQAGEQAQGATAYVTLEPCAHYGRTPPCAEALVKAQVKKVVVACPDPNPLVAGKGVQIL
KNAGIEVEIGICEDLAAKLNQGFLKAMSTGMPYVRLKVASSLDGRTAMASGESKWITGSAA
RQDVQHWRAISGAVITGIDTVIADDCQLNVRSLHNIDIETVAQPKRVILDRRGRLPLTAKILE
NPETVMVMGPYRQELADLGVIQLEIQPLKTLLQTLSKQYQIYDVLIEAGATLSSAFLQEGLID
EMISYVAPTLLGQSARAMFNADFEYMAQQLRFKLLDVIQLDQDIRLRLIPTQEKV
10000000000000000000000000000000000000000000000000000000000000000000000000000000000
00000000000000000000000000000000000000000000000000000000000000000000000000000000000
00000011000000000000000000000000000000000000000000000000000000000000000000000000000
00000000000000000000000000000000000000000000000000000000000000000000000000000000000
00000000000000000000000001111
>Q5LGG4_3scy_24_383
AQPTDPSTTDSELTMLVGTYTSGNSKGIYTFRFNEETGESLPLSDAEVANPSYLIPSADGKFV
YSVNEFSKDQAAVSAFAFDKEKGTLHLLNTQKTMGADPCYLTTNGKNIVTANYSGGSITVF
PIGQDGALLPASDVIEFKGSGPDKERQTMPHLHCVRITPDGKYLLADDLGTDQIHKFNINP
NANADNKEKFLTKGTPEAFKVAPGSGPRHLIFNSDGKFAYLINEIGGTVIAFRYADGMLDEI
QTVAADTVNAQGSGDIHLSPDGKYLYASNRLKADGVAIFKVDETNGTLTKVGYQLTGIHPR
NFIITPNGKYLLVACRDTNVIQIFERDQATGLLTDIKKDIKVDKPVCLKFVD
11110000000000000000000000000000000000000000000000000000000000000000000000000000000
00000000000000000000000000000000000000000000000000000000000000000000000000000000000
00000000000000000000000000000000000000000000000000000000000000000000000000000000000
00000000000000000000000000000000000000000000000000000000000000000000000000000000000
0000000000000000000000000000
>Q6S996_2r8q_581_939
QRNSITAQRAPKSVKVIAVTPEEREAVMSIDFGGAYDFTSPGFNLFEVREKYSEPMDAAAGV
VYNLLWNSGLPEKFGCREQTLLNFILQCRRRYRRVPYHNFYHVVDVCQTLHTYLYTGKASE
LLTELECYVLLVTALVHDLDHMGVNNSFYLKTDSPLGILSSASGNNSVLEVHHCSLAIEILSD

PAADVFEGLSGQDVAYAYRALIDCVLATDMAKHADALSRFTELATSGFEKDNDTHRRLVM
ETLIKAGDVSNVTKPFETSRMWAMAVTEEFYRQGDMEKEKGVEVLPMFDRSKNNELARG
QIGFIDFVAGKFFRDIVGNLFHGMQWCVDTVNSNRAKWQEILDGRRDSIRSSIV
11111111111111100000000000000000000000000000000000000000000000000000000000000000000
00000000000000000000000000000000000000000000000000000000000000000000000000000000000
00000000000000000000000000000000000000000000000000000000000000000000000000000000000
00000000000000000000000000000000000000000000000000000000000000000000000000000000000
000000000000000000111111111
>O24529_1fpx_0_351
MASSINGRKPSEIFKAQALLYKHIYAFIDSMSLKWAVEMNIPNIIQNHGKPISLSNLVSILQVP
SSKIGNVRRLMRYLAHNGFFEIITKEEESYALTVASELLVRGSDLCLAPMVECVLDPTLSGSY
HELKKWIYEEDLTLFGVTLGSGFWDFLDKNPEYNTSFNDAMASDSKLINLALRDCDFVFD
GLESIVDVGGGTGTTAKIICETFPKLKCIVFDRPQVVENLSGSNNLTYVGGDMFTSIPNADA
VLLKYILHNWTDKDCLRILKKCKEAVTNDGKRGKVTIIDMVIDKKKDENQVTQIKLLMDV
NMACLNGKERNEEEWKKLFIEAGFQHYKISPLTGFLSLIEIYP
11111110000000000000000000000000000000000000000000000000000000000000000000000000000
00000000000000000000000000000000000000000000000000000000000000000000000000000000000
00000000000000000000000000000000000000000000000000000000000000000000000000000000000
00000000000000000000000000000000000000000000000000000000000000000000000000000000000
00000000000000000000
>P76015_1oi2_0_355
MKKLINDVQDVLDEQLAGLAKAHPSLTLHQDPVYVTRADAPVAGKVALLSGGGSGHEPM
HCGYIGQGMLSGACPGEIFTSPTPDKIFECAMQVDGGEGVLLIIKNYTGDILNFETATELLH
DSGVKVTTVVIDDDVAVKDSLYTAGRRGVANTVLIEKLVGAAAERGDSLDACAELGRKLN
NQGHSIGIALGACTVPAAGKPSFTLADNEMEFGVGIHGEPGIDRRPFSSLDQTVDEMFDTL
LVNGSYHRTLRFWDYQQGSWQEEQQTKQPLQSGDRVIALVNNLGATPLSELYGVYNRLTT
RCQQAGLTIERNLIGAYCTSLDMTGFSITLLKVDDETLALWDAPVHTPALNWGK
11111111100000000000000000000000000000000000000000000000000000000000000000000000000
00000000000000000000000000000000000000000000000000000000000000000000000000000000000
00000000000000000000000000001111111111100000000000000000000000000000000000000000000
00000000000000000000000000000000000000000000000000000000000000000000000000000000000
000000000000000000000000
>G7CC99_4hdt_1_349
TAKNEDVLVNVEGGVGLLTLNRPKAINSLTHGMVTTMAERLAAWENDDSVRAVLLTGAG
ERGLCAGGDVVAIYHSAKADGAEARRFWFDEYRLNAHIGRYPKPYVSIMDGIVMGGGVG
VGAHGNVRVVTDTTKMAMPEVGIGFIPDVGGTYLLSRAPGKLGLHAALTGAPFSGADAIV
MGFADHYVPHDKIDEFTRAVIADGVDAALAAHAQEPPASPLAEQRSWIDECYTGDTVADII
AALRAHDAPAAGEAADLIATRSPIALSVTLESVRRAAKLQSLEDTLRQEYRVSCASLKSHDL
VEGIRAQLVDKDRNPKWRPATLAEVTEADVEAYFAPVDPELTFEGETQ
11100000000000000000000000000000000000000000000000000000000000000000000000000000000
00000000000000000000000000000000000000000000000000000000000000000000000000000000000
00000000000000000000000000000000000000000000000000000000000000000000000000000000000
00000000000000000000000000000000000000000000000000000000000001111000000000000000000
00000000000000011
>Q7WSJ4_3w08_0_351
MESAIDTHLKCPRTLSRRVPEEYQPPFPMWVARADEQLQQVVMGYLGVQYRGEAQREAA
LQAMRHIVSSFSLPDGPQTHDLTHHTDSSGFDNLMVVGYWKDPAAHCRWLRSAEVNDW
WTSQDRLGEGLGYFREISAPRAEQFETLYAFQDNLPGVGAVMDSTSGEIEEHGYWGSMRDR
FPISQTDWMKPTNELQVVAGDPAKGGRVVIMGHDNIALIRSGQDWADAEAEERSLYLDEIL
PTLQDGMDFLRDNGQPLGCYSNRFVRNIDLDGNFLDVSYNIGHWRSLEKLERWAESHPTH
LRIFVTFFRVAAGLKKLRLYHEVSVSDAKSQVFEYINCHPHTGMLRDAVVAPT

00000000000000000000000000000000000000000000000000000000000000000000000000000000000
00000000000000000000000000000000000000000000000000000000000000000000000000000000000
00000000000000000000000000000000000000000000000000000000000000000000000000000000000
00000000000000000000000000000000000000000000000000000000000000000000000000000000000
0000000000000000001
>P63562_2i3g_0_351
MQNRQVANATKVAVAGASGYAGGEILRLLLGHPAYADGRLRIGALTAATSAGSTLGEHHP
HLTPLAHRVVEPTEAAVLGGHDAVFLALPHGHSAVLAQQLSPETLIIDCGADFRLTDAAV
WERFYGSSHAGSWPYGLPELPGARDQLRGTRRIAVPGCYPTAALLALFPALAADLIEPAVTV
VAVSGTSGAGRAATTDLLGAEVIGSARAYNIAGVHRHTPEIAQGLRAVTDRDVSVSFTPVLI
PASRGILATCTARTRSPLSQLRAAYEKAYHAEPFIYLMPEGQLPRTGAVIGSNAAHIAVAVDE
DAQTFVAIAAIDNLVKGTAGAAVQSMNLALGWPETDGLSVVGVAP
11111000000000000000000000000000000000000000000000000000000000000000000000000000000
00000000000000000000000000000000000000000000000000000000000000000000000000000000000
00000000000000000000000000000000000000000000000000000000000000000000000000000000000
00000000000000000000000000000000000000000000000000000000000000000000000000000000000
0000000000000000000
>Q46E78_3t7v_0_349
MIQKMALDEFDSLGDKVIEGYQLTDNDLRTLLSLESKEGLERLYSAARKVRDHYFGNRVFL
NCFIYFSTYCKNQCSFCYYNCRNEINRYRLTMEEIKETCKTLKGAGFHMVDLTMGEDPYYY
EDPNRFVELVQIVKEELGLPIMISPGLMDNATLLKAREKGANFLALYQETYDTELYRKLRVG
QSFDGRVNARRFAKQQGYCVEDGILTGVGNDIESTILSLRGMSTNDPDMVRVMTFLPQEGT
PLEGFRDKSNLSELKIISVLRLMFPKRLIPASLDLEGIDGMVLRLNAGANIVTSILPPDSQLEG
VANYDRDLEERDRDIKSVVRRLEIMGMKPARQADFEAVLGC
11111111111000000000000000000000000000000000000000000000000000000000000000000000000
00000000000000000000000000000000000000000000000000000000000000000000000000000000000
00000000000000000000000000000000000000000000000000000000000000000000000000000000000
00000000000000000000000000000000000000000000000000000000000000000000000000000000000
00000000000000011
>Q13RP4_4aty_0_348
MSRTRDRAMTVALAIGDPNGIGPEIAVKAAAQMARDERSEHGGHAVHSQPRIVLFGDAFV
IRRYARQCCPELMLRLVQLDDLPSAEPNAIDMVDVASLPAEAFVPGEVAAAAGTATLAYVS
AALRAARAGQVDAVIACPHSETAINASGVRFAGYPGFVAHEMGMPAEDVYLLLIGGGLRI
VHATLHEGIASALARLDQRHVERAARAAVQALQLMGIAHPVVGLMGINPHAGEGGLFGR
DDIDITEPVARKLRDDGMTVIGPQGADLLLTNPDIDVFVAMYHDQGHIPVKLRAGRHSAA
LSIGAGVLFSSVGHGSGFDIAGTLLADPAPLLGAIRLVTTGTVLAPLAE
11111100000000000000000000000000011111111111111100100000000000000000110000000111110
00111100000000000000000000000000000000000000000000000000000000000011100000000000000
00000000000000000000000000000000000000000000000000000000000000000000000000000000000
00000000000000000000000000000000000000000000000000000000000000000000000000000000000
0000000000001111
>Q9L543_2plq_0_347
MRHGDISSSHDTVGIAVVNYKMPRLHTKAEVIENAKKIADMVVGMKQGLPGMDLVVFPE
YSTMGIMYDQDEMFATAASIPGEETAIFAEACKKADTWGVFSLTGEKHEDHPNKAPYNTL
VLINNKGEIVQKYRKIIPWCPIEGWYPGDTTYVTEGPKGLKISLIVCDDGNYPEIWRDCAMK
GAELIVRCQGYMYPAKEQQIMMAKAMAWANNTYVAVANATGFDGVYSYFGHSAIIGFDG
RTLGECGTEENGIQYAEVSISQIRDFRKNAQSQNHLFKLLHRGYTGLINSGEGDRGVAECPF
DFYRTWVLDAEKARENVEKITRSTVGTAECPIQGIPNEGKTKEIGV
00000000000000000000000000000000000000000000000000000000000000000000000000000000000
00000000000000000000000000000000000000000000000000000000000000000000000000000000000
00000000000000000000000000000000000000000000000000000000000000000000000000000000000

00000000000000000000000000000000000000000000000000000000000000000000000000000000000
0000000011111111
>P18654_3g51_43_366
EGSIKEIAITHHVKEGHEKADPSQFELLKVLGQGSFGKVFLVKKISGSDARQLYAMKVLKKA
TLKVRDRVRTKMERDILVEVNHPFIVKLHYAFQTEGKLYLILDFLRGGDLFTRLSKEVMFTEE
DVKFYLAELALALDHLHSLGIIYRDLKPENILLDEEGHIKLTDFGLSKESIDHEKKAYSFCGT
VEYMAPEVVNRRGHTQSADWWSFGVLMFEMLTGTLPFQGKDRKETMTMILKAKLGMPQ
FLSPEAQSLLRMLFKRNPANRLGAGPDGVEEIKRHSFFSTIDWNKLYRREIHPPFKPATGRPE
DTFYFDPEFTAKTPK
11100000000000000000000000000000000000000000000000000000000000000001111111110000000
00000000000000000000000000000000000000000000000000000000000000000000000000000000000
00000000001111111111100000000000000000000000000000000000000000000000000000000000000
000000000000000000000000000000000000000000000000000000111111111111111111111
>Q99SY4_2qx2_58_398
PFKESQARGLLQDNMANSYNGGDFEDGLLNLSKEVFPTDKYLYQDGQFLDKKTINAYLNP
KYTKREIDKMSEKDKKDKKANENLGLNPSHEGETNPEKIAEKSPAYLSNILEQDFYGGGDT
KGKNIKGMTIGLAMNSVYYYKKEKDGPTFSKKLDDSEVKKQGKQMASEILSRLRENDDLK
DIPIHFAIYKQSSEDSITPGEFITQATAEKSQTKLNEWHNINEKSALLPSSTAADYDENLNNN
FKQFNDNLQSYFSNFTQAVGKVKFVDKKPQRLVVDLPIDYYGQAETIGITQYVTEQANKYF
DKIDNYEIRIKDGNQPRALISKTKDDKEPQVHIYSN
11111111100000000000000000000000000000000000000000000000000000000000000000000000000
00000000000000000000000000000000011111100000000000000000000000000000000000000000000
00000000000000000000000000000000000000000000000001000000000000000000000000000000000
00001111111000000000000000000000000000000000000000000000000000000000000000000000000
000000000
>Q9KPY6_3p4e_0_345
MSGNNPSLSYKDAGVDIDAGNALVERIKGAVKRTRRPEVMGGLGGFGALCELPTKYKHPV
LVSGTDGVGTKLRLALDMKKHDTIGIDLVAMCVNDLIVQGAEPLFFLDYYATGKLDVDTA
AEVISGIADGCLQAGCALIGGETAEMPGMYEGEDYDVAGFCVGVVEKEEIIDGSKVQVGD
ALIAVGSSGPHSNGYSLVRKILEVSKADKNERLAGKTIGEHLLAPTKIYIKSGLKLIAEHDIH
AISHITGGGFWENIPRVLPEGTKAVIDGKSWEWPVIFQWLQEKGNVTTHEMYRTFNCGVG
LIIALPKDQANAAVALLQAEGETAWVIGEIAAANSNEAQVEIN
11111111111111111000000000000000000000011111100000000000000000000000000000000000000
00000000000000000000000000000000000000000000000000000000000000000000000000000000000
00000000000000000000000000000000000000000000000000000000000000000000000000000000000
00000000000000000000000000000000000000000000000000000000000000000000000000000000000
00000000000000
>Q47511_3tlc_1_343
MIQSHPLLAAPLAVGDTIGFFSSSAPATVTAKNRFFRGVEFLQRKGFKLVSGKLTGKTDFYRS
GTIKERAQEFNELVYNPDITCIMSTIGGDNSNSLLPFLDYDAIIANPKIIIGYSDTTALLAGIYA
KTGLITFYGPALIPSFGEHPPLVDITYESFIKILTRKQSGIYTYTLPEKWSDESINWNENKILRPK
KLYKNNCAFYGSGKVEGRVIGGNLNTLTGIWGSEWMPEILNGDILFIEDSRKSIATIERLFSM
LKLNRVFDKVSAIILGKHELFDCAGSKRRPYEVLTEVLDGKQIPVLDGFDCSHTHPMLTLPL
GVKLAIDFDNKNISITEQYLSTEK
11111000000000000000000000000000000000000000000000000000000000000000000000000000000
00000000000000000000000000000000000000000000000000000000000000000000000000000000000
00000000000000000000000000000000000000000000000000000000000000000000000000000000000
00000000000000000000000000000000000000000000000000000000000000000000000000000000000
00000000111
>Q8ZJF2_3n9i_0_341
MVLSKPTVSSKPIVFSGAQPSGELTIGNYMGALRQWVQMQDDYDCIYCIVDLHAITARQDP

ALLRKRTLDTLALYLACGIDPKKSTIFVQSHVPEHSQLSWALNCYTYFGELSRMTQFKDKSA
RYAENINAGLFDYPVLMAADILLYQTNQVPVGEDQKQHLELSRDIASRFNNLYGDIFKIPEP
FIPKAGARVMSLQDPTKKMSKSDDNRNNVIELLEDPKSVVKKIKRAMTDSDEPALIRYDVE
KKAGVSNLLDILSGVTGQSIPELEAQFTGQMYGHLKGAVADAVSGMLSELQERYRTYREDE
ALLQDVMREGAAKARARAQVTLAKVYEAIGFVAQP
11111111110000000000000000000000000000000000000000000000000000000000000000000000000
00000000000000000000000000000000000000000000000000000000000000000000000000000000000
00000000000000000000000000000000000000000000000000000000000000000000000000000000000
00000000000000000000000000000000000000000000000000000000000000000000000000000000000
0000000000
>Q2JZD9_4dvj_0_340
MKAVGYNKPAPITDDASLLDIELPKPAPAGHDILVEVKAVSVNPVDYKVRRSTPPDGTDWK
VIGYDAAGIVSAVGPDVTLFRPGDEVFYAGSIIRPGTNAEFHLVDERIVGRKPKTLDWAEAA
ALPLTSITAWEAFFDRLDVNKPVPGAAPAILIVGGAGGVGSIAVQIARQRTDLTVIATASRPE
TQEWVKSLGAHHVIDHSKPLAAEVAALGLGAPAFVFSTTHTDKHAAEIADLIAPQGRFCLI
DDPSAFDIMLFKRKAVSIHHELMFTRPMFGTPDMSEQGRLLNDVSRLVDEGRLRTTLTNRL
SPINAANLKQAHALVESGTARGKVVIEGFGLQR
00000000000000000000000000000000000000000000000000000000100000000000000000000000000
00000000000000000000000000000000000000000000000000000000000000000000000000000000000
00000000000000000000000000000000000000000000000000000000000000000000000000000000000
00000000000000000000000000000000000000000000000000000000000000000000000000000000000
000000011
>P39840_3mwx_0_324
MANFIEKITYLGTPAIKAGNEHLEMIVVPEWGSNVISLVDKTTNVQLLREPETAESFHDTPT
LYGIPILFPPNRISDGTFSFRGRTYHFDINEKDKHNHLHGFLYHEKWNVVTTKQTDEGVIVE
TEIDLSELPHVQKQFPHHAVVRMTYTIKENTLFKHATVMNKGKEAFPWGIGYHTTFIFPAES
SLFSLTADQQWELDERLLPTGKLMDVPYKEALHEGMDLRHKQLDDVFLSSYQKRGGENQ
AVIYHQHAHISIIYKADEQFKHWVVYNADGKQGYLCPEPYTWVTNAVNLDLPSSLTGLQV
LEPGEETTAKSSITIELNHQ
10000000000000000000000000000000000000000000000000000000000000000000000000000000000
00000000000000000000000000000000000000000000000000000000000000000000000000000000000
00000000000000000000000000000000000000000000000000000000000000000000000000000000000
0000000000000000000000000000000000000000000000000000000000000000000000000011
>Q05583_2hes_0_329
MASINLIKSLKLYKEKIWSFDFSQGILATGSTDRKIKLVSVKYDDFTLIDVLDETAHKKAIRSV
AWRPHTSLLAAGSFDSTVSIWAKEESADRTFEMDLLAIIEGHENEVKGVAWSNDGYYLATC
SRDKSVWIWETDESGEEYECISVLQEHSQDVKHVIWHPSEALLASSSYDDTVRIWKDYDDD
WECVAVLNGHEGTVWSSDFDKTEGVFRLCSGSDDSTVRVWKYMGDDEDDQQEWVCEAI
LPDVHKRQVYNVAWGFNGLIASVGADGVLAVYEEVDGEWKVFAKRALCHGVYEINVVK
WLELNGKTILATGGDDGIVNFWSLEKAA
11000000000000000000000000000000000000000000000000000000000000000000000000000000000
00001111111000000000111100000000000000000000000000000000000000000000000000000000000
00000000000000000000000000000000000000000000000000000000000000000000000000000000000
000000000000000000000000000000000000000000000000000000001111100000000000000001111
>Q53653_1n67_220_558
VAADAPAAGTDITNQLTNVTVGIDSGTTVYPHQAGYVKLNYGFSVPNSAVKGDTFKITVPK
ELNLNGVTSTAKVPPIMAGDQVLANGVIDSDGNVIYTFTDYVNTKDDVKATLTMPAYIDPE
NVKKTGNVTLATGIGSTTANKTVLVDYEKYGKFYNLSIKGTIDQIDKTNNTYRQTIYVNPSG
DNVIAPVLTGNLKPNTDSNALIDQQNTSIKVYKVDNAADLSESYFVNPENFEDVTNSVNIT
FPNPNQYKVEFNTPDDQITTPYIVVVNGHIDPNSKGDLALRSTLYGYNSNIIWRSMSWDNE
VAFNNGSGSGDGIDKPVVPEQPDEPGEIEPIPE

11111111000000000000000000000000000000000000000000000000000000000000000000000000000
00000000000000000000000000000000000000000000000000000000000000000000000000000000000
00000000000000000000000000000000000000000000000000000000000000000000000000000000000
00000000000000000000000000000000000000000000000000000000000000000000000000000000000
0000000
>P10477_2wao_484_813
PDEDNPGILYNGRFDFSDPNGPKCAWSGSNVELNFYGTEASVTIKSGGENWFQAIVDGNPL
PPFSVNATTSTVKLVSGLAEGAHHLVLWKRTEASLGEVQFLGFDFGSGKLLAAPKPLERKIE
FIGDSITCAYGNEGTSKEQSFTPKNENSYMSYAAITARNLNASANMIAWSGIGLTMNYGGA
PGPLIMDRYPYTLPYSGVRWDFSKYVPQVVVINLGTNDFSTSFADKTKFVTAYKNLISEVRR
NYPDAHIFCCVGPMLWGTGLDLCRSYVTEVVNDCNRSGDLKVYFVEFPQQDGSTGYGED
WHPSIATHQLMAERLTAEIKNKLGW
11111100000000000000000000000000000000000000000000000000000000000000000000000000000
00000000000000000000000000000000000000000000000000000000000000000000000000000000000
00000000000000000000000000000000000000000000000000000000000000000000000000000000000
000000000000000000000000000000000000000000000000000000000000000000000000000000000
>O00757_3ifa_1_338
TDRSPFETDMLTLTRYVMEKGRQAKGTGELTQLLNSMLTAIKAISSAVRKAGLAHLYGIAGS
VNVTGDEVKKLDVLSNSLVINMVQSSYSTCVLVSEENKDAIITAKEKRGKYVVCFDPLDGSS
NIDCLASIGTIFAIYRKTSEDEPSEKDALQCGRNIVAAGYALYGSATLVALSTGQGVDLFMLD
PALGEFVLVEKDVKIKKKGKIYSLNEGYAKYFDAATTEYVQKKKFPEDGSAPYGARYVGSM
VADVHRTLVYGGIFLYPANQKSPKGKLRLLYECNPVAYIIEQAGGLATTGTQPVLDVKPEAI
HQRVPLILGSPEDVQEYLTCVQKNQAGS
11111110000000000000000000000000000000000000000000000111000000000000000000000
00000000000000000000000000000000000000000000000000000000000010000000000000000000000
00000000000000000000000000000000000000000000000000000000000000000000000000000000000
00000000000000000000000000000000000000000000000000000000000000000000000000000000000
000011
>P0A962_2p2d_0_337
MQKKSIYVAYTGGTIGMQRSEQGYIPVSGHLQRQLALMPEFHRPEMPDFTIHEYTPLMDSS
DMTPEDWQHIAEDIKAHYDDYDGFVILHGTDTMAYTASALSFMLENLGKPVIVTGSQIPLA
ELRSDGQINLLNALYVAANYPINEVTLFFNNRLYRGNRTTKAHADGFDAFASPNLPPLLEA
GIHIRRLNTPPAPHGEGELIVHPITPQPIGVVTIYPGISADVVRNFLRQPVKALILRSYGVGNA
PQNKAFLQELQEASDRGIVVVNLTQCMSGKVNMGGYATGNALAHAGVIGGADMTVEAT
LTKLHYLLSQELDTETIRKAMSQNLRGELTPDD
10000000000000000000000000000000000000000000000000000000000000000000000000000000000
00000000000000000000000000000000000000000000000000000000000000000000000000000000000
00000000000000000000000000000000000000000000000000000000000000000000000000000000000
00000000000000000000000000000001111110000000000000000000000000000000000000000000000
000001
>Q9DAR7_1vlr_0_337
MADTAPQLKRKREQEAEEAETPSTEEKEAGVGNGTSAPVRLPFSGFRVQKVLRESARDKIIF
LHGKVNEDSGDTHGEDAVVILEKTPFQVEHVAQLLTGSPELKLQFSNDIYSTYNLFPPRHLS
DIKTTVVYPATEKHLQKYMRQDLRLIRETGDDYRTITLPYLESQSLSIQWVYNILDKKAEAD
RIVFENPDPSDGFVLIPDLKWNQQQLDDLYLIAICHRRGIRSLRDLTPEHLPLLRNILREGQE
AILKRYQVTGDRLRVYLHYLPSYYHLHVHFTALGFEAPGSGVERAHLLAQVIENLECDPKH
YQQRTLTFALRTDDPLLQLLQKAQQERN
11111111111111111111111111111111111110000000000000000000000000000000011111100000000
00000000000000000000000000000000000000000000000000000000000000000000000000000000000
00000000000000000000000000000000000000000000000000000000000000000000000000000000000
00000000000000000000000000000000000000000000000000000000000000000000000000000000000

000001
>Q9GZT4_3l6b_2_339
AQYCISFADVEKAHINIRDSIHLTPVLTSSILNQLTGRNLFFKCELFQKTGSFKIRGALNAVRS
LVPDALERKPKAVVTHSSGNHGQALTYAAKLEGIPAYIVVPQTAPDCKKLAIQAYGASIVYC
EPSDESRENVAKRVTEETEGIMVHPNQEPAVIAGQGTIALEVLNQVPLVDALVVPVGGGGM
LAGIAITVKALKPSVKVYAAEPSNADDCYQSKLKGKLMPNLYPPETIADGVKSSIGLNTWPI
IRDLVDDIFTVTEDEIKCATQLVWERMKLLIEPTAGVGVAAVLSQHFQTVSPEVKNICIVLSG
GNVDLTSSITWVKQAERPASYQSVSV
00000000000000000000000000000000000000000000000000000000000000011111000000000000
00000000000000000000000000000000000000000000000000000000000000000000000000000000
00000000000000000000000000000000000000000000000000000000000000000000000000000000
00000000000000000000000000000000000000000000000000000000000000000000000000011111
111111
>P68400_3pe1_0_336
MSGPVPSRARVYTDVNTHRPREYWDYESHVVEWGNQDDYQLVRKLGRGKYSEVFEAINIT
NNEKVVVKILKPVKKKKIKREIKILENLRGGPNIITLADIVKDPVSRTPALVFEHVNNTDFKQ
LYQTLTDYDIRFYMYEILKALDYCHSMGIMHRDVKPHNVMIDHEHRKLRLIDWGLAEFYH
PGQEYNVRVASRYFKGPELLVDYQMYDYSLDMWSLGCMLASMIFRKEPFFHGHDNYDQL
VRIAKVLGTEDLYDYIDKYNIELDPRFNDILGRHSRKRWERFVHSENQHLVSPEALDFLDKL
LRYDHQSRLTAREAMEHPYFYTVVKDQARMGSS
11000000000000000000000000000000000000000000000000000000000000000000000000000000
00000000000000000000000000000000000000000000000000000000000000000000000000000000
00000000000000000000000000000000000000000000000000000000000000000000000000000000
00000000000000000000000000000000000000000000000000000000000000000000000000000111
11111
>Q59643_1gzg_0_336
MSFTPANRAYPYTRLRRNRRDDFSRRLVRENVLTVDDLILPVFVLDGVNQRESIPSMPGVER
LSIDQLLIEAEEWVALGIPALALFPVTPVEKKSLDAAEAYNPEGIAQRATRALRERFPELGIIT
DVALDPFTTHGQDGILDDDGYVLNDVSIDVLVRQALSHAEAGAQVVAPSDMMDGRIGAI
REALESAGHTNVRIMAYSAKYASAYYGPFRDAVGSASNLGKGNKATYQMDPANSDEALHE
VAADLAEGADMVMVKPGMPYLDIVRRVKDEFRAPTFVYQVSGEYAMHMGAIQNGWLAE
SVILESLTAFKRAGADGILTYFAKQAAEQLRRGR
11110000000000000000000000000000000000000000000000000000000000000000000000000000
00000000000000000000000000000000000000000000000000000000000000000000000000000000
00000000000000000000000000000000000000000000000000000000000000000000000000000000
00000000000000000000000000000000000000000000000000000000000000000000000000000000
00011
>P04406_1u8f_0_334
MGKVKVGVNGFGRIGRLVTRAAFNSGKVDIVAINDPFIDLNYMVYMFQYDSTHGKFHGTV
KAENGKLVINGNPITIFQERDPSKIKWGDAGAEYVVESTGVFTTMEKAGAHLQGGAKRVII
SAPSADAPMFVMGVNHEKYDNSLKIISNASCTTNCLAPLAKVIHDNFGIVEGLMTTVHAIT
ATQKTVDGPSGKLWRDGRGALQNIIPASTGAAKAVGKVIPELNGKLTGMAFRVPTANVSV
VDLTCRLEKPAKYDDIKKVVKQASEGPLKGILGYTEHQVVSSDFNSDTHSSTFDAGAGIAL
NDHFVKLISWYDNEFGYSNRVVDLMAHMASKE
11000000000000000000000000000000000000000000000000000000000000000000000000000000
00000000000000000000000000000000000000000000000000000000000000000000000000000000
00000000000000000000000000000000000000000000000000000000000000000000000000000000
00000000000000000000000000000000000000000000000000000000000000000000000000000000
000
>P17584_1dxy_0_332
MKIIAYGARVDEIQYFKQWAKDTGNTLEYHTEFLDENTVEWAKGFDGINSLQTTPYAAGVF

EKMHAYGIKFLTIRNVGTDNIDMTAMKQYGIRLSNVPAYSPAAIAEFALTDTLYLLRNMGK
VQAQLQAGDYEKAGTFIGKELGQQTVGVMGTGHIGQVAIKLFKGFGAKVIAYDPYPMKG
DHPDFDYVSLEDLFKQSDVIDLHVPGIEQNTHIINEAAFNLMKPGAIVINTARPNLIDTQA
MLSNLKSGKLAGVGIDTYEYETEDLLNLAKHGSFKDPLWDELLGMPNVVLSPHIAYYTETA
VHNMVYFSLQHLVDFLTKGETSTEVTGPAK
00000000000000000000000000000000000000000000000000000000000000000000000000000000
00000000000000000000000000000000000000000000000000000000000000000000000000000000
00000000000000000000000000000000000000000000000000000000000000000000000000000000
0000000000000000000000000000000000000000000000000000000000000000000000000000000011
1
>Q4Z4C3_2fds_1_333
HFKTKLKNRRNEVNTCLCIGLDPDEDDIKNFMRNEEKNGYKNVKNNMNSNNNRIENVIK
IGKEILLTDEENIENLSEEDKFFYFFNHFCFYIINNTKEYALIYKMNFAFYIPYGSVGINALKN
VFDYLNSMNIPTMLDMKINDIGNTVKNYRKFIFEYLKSDSCTINVYMGTSMLKDICFDYEK
NKYYSAYVLIKTTNKDSFIFQNELSINDKQAYIVMAEETQKMATDLKIDQNNEFIGFVVGSN
AFEEMKIIRNKFPDSYILSPGIGAQNGDLYKTLKNGYNKDYEKLLINVGRAITKSPNPKKSSE
SYYNQIIQIFKDIENGGNIEQVYL
00000000000000000000000000000000000000000000000000000000000000000000000000000000
00000000000000000000000000000000000000000000000000000000000000000000000000000000
00000000000000000000000000000000000000000000000000000000000000000000000000000000
000000000000000000111110000000000000000000000000000000000000000000000000011111111
1
>Q9CF33_2o20_0_331
MVESTTTIYDVARVAGVSMATVSRVVNGNANVKEKTRQKVLEAIAELDYRPNAVARGLAS
KRTTTVGVILPTITSTYFAAITRGVDDIDSMYKYNMILANSDNDVEKEEKVLETFLSKQVDGI
VYMGSSLDEKIRTSLKNSRTPVVLVGTIDGDKEIPSVNIDYHLAAYQSTKKLIDSGNKKIAYI
MGSLKDVENTERMVGYQEALLEANIEFDENLVFEGNYSYEQGKALAERLLERGATSAVVS
HDTVAVGLLSAMMDKGVKVPEDFEIISGANSPITQYTYPTLTSVNQPLYDLGAVAMRLLTKL
MLKEDVEQNQLVLDHEIFSRRSTK
11111111111111111111111111111111111111111111111111111111111000000000000000000000
00000000000000000000000000000000000000000000000000000000000000000000000000000000
00000000000000000000000000000000000000000000000000000000000000000000000000000000
00000000000000000000000000000000000000000000000000000000000000000000000000000000
>P27254_2p67_1_330
INEATLAESIRRLRQGERATLAQAMTLVESRHPRHQALSTQLLDAIMPYCGNTLRLGVTGTP
GAGKSTFLEAFGMLLIREGLKVAVIAVDPSSPVTGGSILGDKTRMNDLARAEAAFIRPVPSSG
HLGGASQRARELMLLCEAAGYDVVIVETVGVGQSETEVARMVDCFISLQIAGGGDDLQGI
KKGLMEVADLIVINKDDGDNHTNVAIARHMYESALHILRRKYDEWQPRVLTCSALEKRGI
DEIWHAIIDFKTALTASGRLQQVRQQQSVEWLRKQTEEEVLNHLFANEDFDRYYRQTLLAV
KNNTLSPRTGLRQLSEFIQTQYFD
00000000000000000000000000000000000000000000000000000000000000000000000000000000
00000000011111111111111100000000000000111110000000000000000000000000000000000000
00000000001111110000000000000000000000000000000000000000000000000000000000000000
000000000000000000000000000000000000000000000000000000000000000000000000001111
>Q4EFN5_3pnz_0_329
MSFIRTFYGDIAPEQLGFTYSHEHIVCVPAYWQERDADDLLLDDKEKSQLDVQDFADLGGK
TIVDATAVDYGRRVLDVAQISKETGIQIVGTAGFNKSFLWDGKIKPELKPIIGDFETYYEWIEN
TTTDKLTEFVVNEVENGLEGTPYKAGQVKFGTGYNMITPLEEKTIRAVARAHHETKAPIHS
HTEAGTMALEQIEILKQENIPLEYLSIGHMDRNLDPYYHKQVAKTGAFMSFDGIAKIKYAPE
SARIAAILYLVSEGFEDQILVSGDTARKTYYKHYGHGPGLEYIAKKWVPRFIDEANEKGFDG
EKLVKKFFVDNPARCFTFKK

10000000000000000000000000000000000000000000000000000000000000000000000000000000000
00000000000000000000000000000000000000000000000000000000000000000000000000000000000
00000000000000000000000000000000000000000000000000000000000000000000000000000000000
00000000000000000000000000000000000000000000000000000000000000000000000000000001
>Q9NXS2_3pb4_52_381
SGWHRRTEELPLGRELRVPLIGSLPEARLRRVVGQLDPQRLWSTYLRPLLVVRTPGSPGNLQ
VRKFLEATLRSLTAGWHVELDPFTASTPLGPVDFGNVVATLDPRAARHLTLACHYDSKLFP
PGSTPFVGATDSAVPCALLLELAQALDLELSRAKKQAAPVTLQLLFLDGEEALKEWGPKDS
LYGSRHLAQLMESIPHSPGPTRIQAIELFMLLDLLGAPNPTFYSHFPRTVRWFHRLRSIEKRL
HRLNLLQSHPQEVMYFQPGEPFGSVEDDHIPFLRRGVPVLHLISTPFPAVWHTPADTEVNL
HPPTVHNLCRILAVFLAEYLGL
11111111111111111110000000000000000000000000000000000000000000000000000000000000000
00000000000000000000000000000000000000000000000000000000000000000000000000000000000
00000000000000000000000000000000000000000000000000000000000000000000000000000000000
00000000000000000000000000000000000000000000000000000000000000000000000000000000
>P63705_3mhf_1_326
TITLTENKRKSMEKLSVDGVISALAFDQRGALKRMMAQHQTKEPTVEQIEELKSLVSEELTP
FASSILLDPEYGLPASRVRSEEAGLLLAYEKTGYDATTSRLPDCLDVWSAKRIKEAGAEAVK
FLLYYDIDGDQDVNEQKKAYIERIGSECRAEDIPFYLEILTYDEKIADNASPEFAKVKAHKVN
EAMKVFSKERFGVDVLKVEVPVNMKFVEGFADGEVLFTKEEAAQAFRDQEASTDLPYIYLS
AGVSAKLFQDTLVFAAESGAKFNGVLCGRATWAGSVKVYIEEGPQAAREWLRTEGFKNID
ELNKVLDKTASPWTEKM
10000000000000000000000000000000000000000000000000000000000000000000000000000000000
00000000000000000000000000000000000000000000000000000000000000000000000000000000000
00000000000000000000000000000000000000000000000000000000000000000000000000000000000
00000000000000000000000000000000000000000000000000000000000000000000000011
>Q9HIZ7_3ing_0_323
MKEIRIILMGTGNVGLNVLRIIDASNRRRSAFSIKVVGVSDSRSYASGRNLDISSIISNKEKTGR
ISDRAFSGPEDLMGEAADLLVDCTPASRDGVREYSLYRMAFESGMNVVTANKSGLANKW
HDIMDSANQNSKYIRYEATVAGGVPLFSVLDYSILPSKVKRFRGIVSSTINYVIRNMANGRSL
RDVVDDAIKKGIAESNPQDDLNGLDAARKSVILVNHIFGTEYTLNDVEYSGVDERSYNAN
DRLVTEVYVDDRRPVAVSRIISLNKDDFLMSIGMDGLGYQIETDSNGTVNVSDIYDGPYETA
GAVVNDILLLSKVQK
10000000000000000000000000000110000000000000000000000000000000000000000000000000000
00000000000000000000000000000000000000000000000000000000000000000000000000000000000
00000000000000000000000000000000000000000000000000000000000000000000000000000000000
00000000000000000000000000000000000000000000000000000000000000000000000011
>P69739_3use_45_371
LENKPRIPVVWIHGLECTCCTESFIRSAHPLAKDVILSLISLDYDDTLMAAAGTQAEEVFEDI
ITQYNGKYILAVEGNPPLGEQGMFCISSGRPFIEKLKRAAAGASAIIAWGTCASWGCVQAA
RPNPTQATPIDKVITDKPIIKVPGCPPIPDVMSAIITYMVTFDRLPDVDRMGRPLMFYGQRIH
DKCYRRAHFDAGEFVQSWDDDAARKGYCLYKMGCKGPTTYNACSSTRWNDGVSFPIQSG
HGCLGCAENGFWDRGSFYSRVVDIPQMGTHSTADTVGLTALGVVAAAVGVHAVASAVDQ
RRRHNQQPTETEHQPGNEDKQA
11100000000000000000000000000000000000000000000000000000000000000000000000000000000
00000000000000000000000000000000000000000000000000000000000000000000000000000000000
00000000000000000000000000000000000000000000000000000000000000000000000000000000000
00000000000000000000001111111111111111111111111111111111111111111111111111111
>Q60GU1_2dvu_0_326
MQGKVALEEHFAIPETLQDSAGFVPGDYWKELQHRLLDIQDTRLKLMDAHGIETMILSLN
APAVQAIPDRRKAIEIARRANDVLAEECAKRPDRFLAFAALPLQDPDAATEELQRCVNDLG

FVGALVNGFSQEGDGQTPLYYDLPQYRPFWGEVEKLDVPFYLHPRNPLPQDSRIYDGHPW
LLGPTWAFAQETAVHALRLMASGLFDEHPRLNIILGHMGEGLPYMMWRIDHRNAWVKLP
PRYPAKRRFMDYFNENFHITTSGNFRTQTLIDAILEIGADRILFSTDWPFENIDHASDWFNAT
SIAEADRVKIGRTNARRLFKLDGA
00000000000000000000000000000000000000000000000000000000000000000000000000000000000
00000000000000000000000000000000000000000000000000000000000000000000000000000000000
00000000000000000000000000000000000000000000000000000000000000000000000000000000000
0000000000000000000000000000000000000000000000000000000000000000000000000000000011
>C0STU6_4e9v_34_358
AEREFDMTIEEVTIKVAPGLDYKVFGFNGQVPGPLIHVQEGDDVIVNVTNNTSLPHTIHWH
GVHQKGTWRSDGVPGVTQQPIEAGDSYTYKFKADRIGTLWYHCHVNVNEHVGVRGMW
GPLIVDPKQPLPIEKRVTKDVIMMMSTWESAVADKYGEGGTPMNVADYFSVNAKSFPLTQP
LRVKKGDVVKIRFFGAGGGIHAMHSHGHDMLVTHKDGLPLDSPYYADTVLVSPGERYDVII
EADNPGRFIFHDHVDTHVTAGGKHPGGPITVIEYDGVPVDDWYVWKDKYDPNFFYSESL
KQGYGMFDHDGFKGEFEQRQRRPGR
00000000000000000000000000000000000000000000000000000000000000000000000000000000000
00000000000000000000000000000000000000000000000000000000000000000000000000000000000
00000000000000000000000000000000000000000000000000000000000000000000000000000000000
0000000000000000000000000000000000000000000000000000000000000000000111111111
>Q07702_3uc1_513_837
EDVVVTITETGYAKRTKTDLYRSQKRGGKGVQGAGLKQDDIVAHFFVCSTHDLILFFTTQG
RVYRAKAYDLPEASRTARGQHVANLLAFQPEERIAQVIQIRGYTDAPYLVLATRNGLVKKS
KLTDFDSNRSGGIVAVNLRDNDELVGAVLCSAGDDLLLVSANGQSIRFSATDEALRPMGRA
TSGVQGMRFNIDDRLVSLNVVREGTYLLVATSGGYAKRTAIEEYPVQGRGGKGVLTVMYDR
RRGRLVGALIVDDDSELYAVTSGGGVIRTAARQVRKAGRQTKGVRLMNLGEGDTLLAIAR
NAEESGDDNAVDANGADQTGN
00000000000000000000000001111111100000000000000000000000000000000000000000000000000
00000000000000000000000000000000000000000000000000000000000000000000000000000000000
00000000000000000000000000000000000000000000000000000000000000000000000000000000000
0000000000000000000000000000000000000000000000000000000001111111111111111111
>Q59N78_2g9z_4_329
SEDSELIEQVIEQPDSLIISPPSDSSSSSYNHIQPFVYLESTATTQTNHNVLLILNQKITIDLISLW
KKCEIIVCADGGANSLYEYFNDNNHHHHHENLQRSDYIPDYIVGDFDSISPDVKTYYESHG
SKIIRQSSQYYNDFTKSIHCIQLHYQLNHTKENWFESIDEVDGLAKLWNGLNNSSDVVVDI
DITIYVLNAIGGRFDQTVQSINQLYIMNEDYPKVTVFFITTNDIIFLLKKGVNYISYKNRLMFH
KDNGSSPTPTCGLLPLSNKTPIILNSYGLKYDMRNWKTEMLGQVSSSNRISGETGFIVECSD
DIVMNIEIDV
11100000000000000000001111110000000000111111111100000000000000000000000000000000000
00000111111111100000000000000000000000000000000000000000000000000000000000000000000
00000000000000000000000000000000000000000000000000000000000000000000000000000000000
0000000000000000000000000000000000000000000000000000000000000000000000000000
>Q99UY5_4h0a_23_344
SPRLKFDVLENPNKGNKVNRSEQVNKSNNHAENPKPKEGVGTWVGKDIKVLTSKFGQAD
RVYPFRDGYKNYVFKDKNSYYIVSTKREEIVSVYATGEKVNVSPLKIGQHSAEIFNHTSINPE
PSFKVDGKKYEFELSDEDLKTQTLIKYGDIYAQVYSDQQSKKVLSVRFLTKEMLADIEPYQL
NSNSTSEEHNKRPVEQNPNQLISLYEVTNEMRKLKGLKPLKINSDLAHIASNNLYEATSNGS
DSVEFTEDALRGQLDKNHVTYKTTAQNVGYAFNDVPTLIHSWMNSDIHRSRLLNSKYDEM
GGDVMRDYYSLIFLEK
10000000000000111111111111111100000000000000000000000000000000000000000000000000000
00000000000000000000000000000000000000000000000000000000000000000000000000000000000
00000000000000000000000000000000000000000000000000000000000000000000000000000111100

000000000000000000000000000000000000000000000000000000000000000000000000
>D0VWT0_3c1o_0_320
GSHMEKIIIYGGTGYIGKFMVRASLSFSHPTFIYARPLTPDSTPSSVQLREEFRSMGVTIIEGEM
EEHEKMVSVLKQVDIVISALPFPMISSQIHIINAIKAAGNIKRFLPSDFGCEEDRIKPLPPFESV
LEKKRIIRRAIEAAALPYTYVSANCFGAYFVNYLLHPSPHPNRNDDIVIYGTGETKFVLNYEE
DIAKYTIKVACDPRCCNRIVIYRPPKNIISQNELISLWEAKSGLSFKKVHMPDEQLVRLSQELP
QPQNIPVSILHSIFVKGDLMSYEMRKDDIEASNLYPELEFTSIDGLLDLFISGRAPPPTLAEFE
1110000000000000000000000000000000000000000000000000000000000000000000000000000000
0000000000000000000000000000000000000000000000000000000000000000000000000000000000
0000000000000000000000000000000000000000000000000000000000000000000000000000000000
00000000000000000000000000000000000000000000000000000000000000000001111
>Q6N5G3_4e04_0_317
MTEGSVARQPDLSTCDDEPIHIPGAIQPHGLLLALAADMTIVAGSDNLPELTGLAIGALIGR
SAADVFDSETHNRLTIALAEPGAAVGAPIAVGFTMRKDAGFVGSWHRHDQLVFLELEPPQ
RDVAEPQAFFRRTNSAIRRLQAAETLESACAAAAQEVREITGFDRVMIYRFASDFSGEVIAE
DRCAEVESYLGLHFPASDIPAQARRLYTINPVRIIPDINYRPVPVTPDLNPVTGRPIDLSFAILR
SVSPVHLEYMRNIGMHGTMSISILRGERLWGLIACHHRKPNYVDLDGRQACELVAQVLAW
QIGVMEEQA
1111111111000000000000000000000000000000000000000000000000000000000000000000000000
0000000000000000000000000000000000000000000000000000000000000000000000000000000000
0000000000000000000000000000000000000000000000000000000000000000000000000000000000
00000000000000000000000000000000000000000000000000000000000000000000
>P29208_1r6w_0_319
MRSAQVYRWQIPMDAGVVLRDRRLKTRDGLYVCLREGEREGWGEISPLPGFSQETWEEAQ
SVLLAWVNNWLAGDCELPQMPSVAFGVSCALAELTDTLPQAANYRAAPLCNGDPDDLIL
KLADMPGEKVAKVKVGLYEAVRDGMVVNLLLEAIPDLHLRLDANRAWTPLKGQQFAKY
VNPDYRDRIAFLEEPCKTRDDSRAFARETGIAIAWDESLREPDFAFVAEEGVRAVVIKPTLTG
SLEKVREQVQAAHALGLTAVISSSIESSLGLTQLARIAAWLTPDTIPGLDTLDLMQAQQVRR
WPGSTLPVVEVDALERLL
0000000000000000000100000000000000000000000000000000000000000000000000000000000000
0000000000000000000000000000000000000000000000000000000000000000000000000000000000
0000000000000000000000000000000000000000000000000000000000000000000000000000000000
0000000000000000000000000000000000000000000000000000000000000000000000
>P41052_1ltm_41_360
EPQHNVMQMGGDFANNPNAQQFIDKMVNKHGFDRQQLQEILSQAKRLDSVLRLMDNQ
APTTSVKPPSGPNGAWLRYRKKFITPDNVQNGVVFWNQYEDALNRAWQVYGVPPEIIVGII
GVETRWGRVMGKTRILDALATLSFNYPRRAEYFSGELETFLLMARDEQDDPLNLKGSFAGA
MGYGQFMPSSYKQYAVDFSGDGHINLWDPVDAIGSVANYFKAHGWVKGDQVAVMANG
QAPGLPNGFKTKYSISQLAAAGLTPQQPLGNHQQASLLRLDVGTGYQYWYGLPNFYTITR
YNHSTHYAMAVWQLGQAVALARVQ
0000000000000000000000000000000000000000000000000000000111111111110000000000000000
0000000000000000000000000000000000000000000000000000000000000000000000000000000000
0000000000000000000000000000000000000000000000000000000000000000000000000000000000
0000000000000000000000000000000000000000000000000000000000000000000000
>Q9WYF8_2hpg_16_335
SAVFGAKYTLRFGHVLAPGEPYHQAFLKWAKAVEEKTNGDVRIEVFPSSQLGVEEDIIEQIR
MGAPVGWNTDSARLGMYVKDIGVMNLAYFIDFMGAKTPEEAIEVLKKIKQSPTMQKWLK
ELEQRFGIKVLSFYWVQGYRHFVTNKPIRKPEDLNGLRIRTPGAPAWQESIRSLGAIPVAVNF
GEIYTAVQTRAVDGAELTYANVNYNGGLYEVLKYMSETGHFLLINFEIVSADWFNSLPKEYQ
KIIEEEMDKAGIEVSLKIMKELEEEYKQKCIEKGMAVIPASEIDKEAFMEKAKQAYKNLGLE
NALNQLIKEVKGE

11100000000000000000000000000000000000000000000000000000000000000000000000000000000
00000000000000000000000000000000000000000000000000000000000000000000000000000000000
00000000000000000000000000000000000000000000000000000000000000000000000000000000000
0000000000000000000000000000000000000000000000000000000000000000000000
>Q05489_1cvl_39_357
ADTYAATRYPVILVHGLAGTDKFANVVDYWYGIQSDLQSHGAKVYVANLSGFQSDDGPN
GRGEQLLAYVKQVLAATGATKVNLIGHSQGGLTSRYVAAVAPQLVASVTTIGTPHRGSEFA
DFVQDVLKTDPTGLSSTVIAAFVNVFGTLVSSSHNTDQDALAALRTLTTAQTATYNRNFPS
AGLGAPGSCQTGAATETVGGSQHLLYSWGGTAIQPTSTVLGVTGATDTSTGTLDVANVTD
PSTLALLATGAVMINRASGQNDGLVSRCSSLFGQVISTSYHWNHLDEINQLLGVRGANAED
PVAVIRTHVNRLKLQGV
00000000000000000000000000000000000000000000000000000000000000000000000000000000000
00000000000000000000000000000000000000000000000000000000000000000000000000000000000
00000000000000000000000000000000000000000000000000000111000000000000000000000000000
0000000000000000000000000000000000000000000000000000000000000000000000
>O68212_3qdh_198_487
NEAISVEKSIQEQKLNGYGVGSLIKFPVSSTAPTLDAKSFYKYFQLRDTLDDRLTAVTATEVSL
EGTTLDPTDYKVDTKGQTVTVTFTAEGLKKIKAAPGKKVSAVFQGKVTEARNGAITNRAQ
VISDTVYAEQPPTPEEPPANPENPPTSNEVTSRWGDLLIKKVDNHQQGQDKAGLQGAQFQ
LYKAKNAYAGTCTKDKEGDPIAINGETTLTTDAQGAINVKGLFISDSIDGANRDNQKDATA
RCYVLVETKAPAGYVLPAGDGAVTPVKIEVGAVTTDNVTIENTKQ
11100000000000000000000000000000000000000000000000000000000000000000000000000000000
00000000000000000000000000000000000000000000000000000000000000000000000000000000000
11111111110000000000000000000000000000000000000000000000000000000000000000000000000
00000000000000000000000000111111000000000
>P0AEL6_3tlk_0_317
MRLAPLYRNALLLTGLLLSGIAAVQAADWPRQITDSRGTHTLESQPQRIVSTSVTLTGSLLAI
DAPVIASGATTPNNRVADDQGFLRQWSKVAKERKLQRLYIGEPSAEAVAAQMPDLILISAT
GGDSALALYDQLSTIAPTLIINYDDKSWQSLLTQLGEITGHEKQAAERIAQFDKQLAAAKE
QIKLPPQPVTAIVYTAAAHSANLWTPESAQGQMLEQLGFTLAKLPAGLNASQSQGKRHDII
QLGGENLAAGLNGESLFLFAGDQKDADAIYANPLLAHLPAVQNKQVYALGTETFRLDYYS
AMQVLDRLKALF
11111111111111111111111111100000000000000000000000000000000000000000000000000000000
00000000000000000000000000000000000000000000000000000000000000000000000000000000000
00000000000000000000000000000000000000000000000000000000000000000000000000000000000
000000000000000000000000000000000000000000000000000000000000000000000
>Q1H3U4_3mcq_0_317
MASEFDLIQRYFRRAHPSAVLGVGDDAALIQPSPGMELAVSADMLVANTHFYPNIDPWLIG
WKSLAVNISDMAAMGAQPRWATLTIALPEADEDWISKFAAGFFACAAQFDIALIGGDTTR
GPLTISVQIMGETPPGASLLRSTARADDDIWVSGPLGDAALALAAIQGRYPLSDTELAACGK
ALHQPQPRVVLGQALRGLAHSALDISDGLLADLGHILEHSQVGAEVWLKAIPKSEVVSAH
SQEVAIQKMILSGGDDYELCFTASTQHRQQIADIGRQLSLDMAVIGRITDTQQLVIHGLDDA
PLTLKEHGFDHFA
11111100000000000000000000000000000000000000000000000000000000000000000000000000000
00000000000000000000000000000000000000000000000000000000000000000000000000000000000
00000000000000000000000000000000000000000000000000000000000000000000000000000000000
000000000000000000000000000000000000000000000000000000000001111111111
>Q63T51_3s6o_0_316
MAFDPNYPRDLIGYGRHPVQANWPGRARVAVQFVLNYEEGGENCVLHGDPASEQFLSEIV
GAAAYPARHMSMESIYEYGSRAGVWRILREFDKRGLPLTVFGVGMAIERHPELARAFVELG
HEIACHGWRWIHYQDMTPEREAEHMRLGMEAIERVTGVRPLGWYTGRDSPNTHRLVAEY

GGFLYDSDHYGDDLPFWMDVEVSGGASVPQLIVPYTLDANDMRFATPQGFNTADHFFHY
LRDAFDVLYEEGDEAPKMMSIGMHCRLLGRPGRFRALQRFLDHIERHDRVWVARRVEIAR
HWREHHPYRPHARREGDA
11000000000000000000000000000000000000000000000000000000000000000000000000000000
00000000000000000000000000000000000000000000000000000000000000000000000000000000
00000000000000000000000000000000000000000000000000000000000000000000000000000000
0000000000000000000000000000000000000000000000000000000001111111111
>G3XD23_3oa2_0_315
MKNFALIGAAGYIAPRHMRAIKDTGNCLVSAYDINDSVGIIDSISPQSEFFTEFEFFLDHASN
LKRDSATALDYVSICSPNYLHYPHIAAGLRLGCDVICEKPLVPTPEMLDQLAVIERETDKRLY
NILQLRHHQAIIALKDKVAREKSPHKYEVDLTYITSRGNWYLKSWKGDPRKSFGVATNIGV
HFYDMLHFIFGKLQRNVVHFTSEYKAAGYLEYEQARVRWFLSVDANDLPESVKGKKPTYR
SITVNGEEMEFSEGFTDLHTTSYEEILAGRGYGIDDARHCVETVNTIRSAVIVPASDNEGHPF
VAALAR
00000000000000000000000000000000000000000000000000000000000000000000000000000000
00000000000000000000000000000000000000000000000000000000000000000000000000000000
00000000000000000000000000000000000000000000000000000000000000000000000000000000
000000000111110000000000000000000000000000000000000000000000001111
>P62623_3kef_0_315
MQILLANPRGFCAGVDRAISIVENALAIYGAPIYVRHEVVHNRYVVDSLRERGAIFIEQISEV
PDGAILIFSAHGVSQAVRNEAKSRDLTVFDATCPLVTKVHMEVARASRRGEESILIGHAGHP
EVEGTMGQYSNPEGGMYLVESPDDVWKLTVKNEEKLSFMTQTTLSVDDTSDVIDALRKRF
PKIVGPRKDDICYATTNRQEAVRALAEQAEVVLVVGSKNSSNSNRLAELAQRMGKRAFLID
DAKDIQEEWVKEVKCVGVTAGASAPDILVQNVVARLQQLGGGEAIPLEGREENIVFEVPKE
LRVDIREVD
00000000000000000000000000000000000000000000000000000000000000000000000000000000
00000000000000000000000000000000000000000000000000000000000000000000000000000000
00000000000000000000000000000000000000000000000000000000000000000000000000000000
000000000000000000000000000000000000000000000000000000000001111111
>Q9YAH1_2e9y_0_315
MDSGRLAVIALGGNAIAGPGMDVSVESQTAAVKRASSIIADVLADGWRSVITHGNGPQVG
YLSEAFEALPPERPRQPLYIATAMTQAWIGLLLKHSLEEELRRRGLNVLVPVVISRVLVDVSD
PSFNNPSKPVGPIYGREEAEELSRRYGWVFKRDPRGGFRRVVPSPRPVSIVDRDLIAEASAES
PAVVALGGGGVPVVERPGGVLEPVEAVVDKDLASSLLATQLNADLLVILTDVPGVAVNYGR
EGERWLRRAAASELKKYLREGHFPPGSMGPKVEAAISFVERTGKPAVIGSLEEARQVLSLQA
GTVVMLG
11100000000000000000000000000000000000000000000000000000000000000000000000000000
00000000000000000000000000000000000000000000000000000000000000000000000000000000
00000000000000000000000000000000000000000000000000000000000000000000000000000000
000000000000000000000000000000000000000000000000000000000000000000
>P83817_1us5_0_313
MRKPILAALTLAGLGLAQEFITIGSGSTTGVYFPVATGIAKLVNDANVGIRANARSTGGSVA
NINAINAGEFEMALAQNDIAYYAYQGCCIPAFEGKPVKTIRALAALYPEVVHVVARKDAGI
RTVADLKGKRVVVGDVGSGTEQNARQILEAYGLTFDDLGQAIRVSASQGIQLMQDKRADA
LFYTVGLGASAIQQLALTTPIALVAVDLNRIQAIAKKYPFYVGFNIPGGTYKGVDVTTPTVAV
QAMLIASERLSEETVYKFMKAVFGNLEAFKKIHPNLERFFGLEKAVKGLPIPLHPGAERFYK
EAGVLK
11111111111111000000000000000000000000000000000000000000000000000000000000000000
00000000000000000000000000000000000000000000000000000000000000000000000000000000
00000000000000000000000000000000000000000000000000000000000000000000000000000000
0000000000000000000000000000000000000000000000000000000000000000

>P96693_3oaj_0_311
MAKKTMGIHHITAIVGHPQENTDFYAGVLGLRLVKQTVNFDDPGTYHLYFGNEGGKPGTII
TFFPWAGARQGVIGDGQVGVTSYVVPKGAMAFWEKRLEKFNVPYTKIERFGEQYVEFDDP
HGLHLEIVEREEGEANTWTFGEVTPDVAIKGFGGATLLSEQPDKTADLLENIMGLERVGKE
GDFVRYRSAGDIGNVIDLKLTPIGRGQMGAGTVHHIAWRANDDEDQLDWQRYIASHGYG
VTPVRDRNYFNAIYFREHGEILFEIATDPPGFAHDETQETMGEKLMLPVQYEPHRTQIEQGL
LPFEVRELD
10000000000000000000000000000000000000000000000000000000000000000000000000000000000
00000000000000000000000000000000000000000000000000000000000000000000000000000000000
00000000000000000000000000000000000000000000000000000000000000000000000000000000000
00000000000000000000000000000000000000000000000000000000000001
>P07773_1dlt_0_310
MEVKIFNTQDVQDFLRVASGLEQEGGNPRVKQIIHRVLSDLYKAIEDLNITSDEYWAGVAYL
NQLGANQEAGLLSPGLGFDHYLDMRMDAEDAALGIENATPRTIEGPLYVAGAPESVGYAR
MDDGSDPNGHTLILHGTIFDADGKPLPNAKVEIWHANTKGFYSHFDPTGEQQAFNMRRSI
ITDENGQYRVRTILPAGYGCPPEGPTQQLLNQLGRHGNRPAHIHYFVSADGHRKLTTQINV
AGDPYTYDDFAYATREGLVVDAVEHTDPEAIKANDVEGPFAEMVFDLKLTRLVDGVDNQV
VDRPRLAV
11000000000000000000000000000000000000000000000000000000000000000000000000000000000
00000000000000000000000000000000000000000000000000000000000000000000000000000000000
00000000000000000000000000000000000000000000000000000000000000000000000000000000000
00000000000000000000000000000000000000000000000000000000000000
>B3PD60_3qee_27_333
QAENPIFTDVFTADPAALVHKGRVYLYAGRDEAPDNTTFFVMNEWLVYSSDDMANWEAH
GPGLRAKDFTWAKGDAWASQVIERNGKFYWYVTVRHDDTKPGFAIGVAVGDSPIGPFKDA
LGKALITNDMTTDTPIDWDDIDPSVFIDDDGQAYLFWGNTRPRYAKLKKNMVELDGPIRAI
EGLPEFTEAIWVHKYQDNYYLSYAMGFPEKIGYAMGKSIKGPWVYKGILNEVAGNTPTNH
QAIIEFNNKHYFIYHTGAGRPDGGQYRRSVSIDELFYNPDGTIKRIVMTTEGVAPNKSPERV
KKAAK
00000000000000000000000000000000000000000000000000000000000000000000000000000000000
00000000000000000000000000000000000000000000000000000000000000000000000000000000000
00000000000000000000000000000000000000000000000000000000000000000000000000000000000
0000000000000000000000000000000000000000000000000011111111
>Q2CIP5_4eum_0_304
MHILSIGECMAELAPADLPGTYRLGFAGDTFNTAWYLARLRPESRISYFSAIGDDALSQQMR
AAMSAAGIDGGGLRVIPGRTVGLYLITLEQGERSFAYWRGQSAARELAGDADALAAAMAR
ADVVYFSGITLAILDQCGRATLLRALAQARATGRTIAFDPNLRPRLWAGTGEMTETIMQGA
AVSDIALPSFEDEAAWFGDAGPDATADRYARAGVRSVVVKNGPHAVHFLQDGRRGRVPV
PPVAQVVDTTAAGDSFNAGLLDSVLAGQPLETAIAAAAALAGQVVQGKGALVEVPSLRPH
ADA
00000000000000000000000000000000000000000000000000000000000000000000000000000000000
00000001110000000000000000000000000000000000000000000000000000000000000000000000000
00000000000000000000000000000000000000000000000000000000000000000000000000000000000
00000000000000000000000000000000000000000000000111111111
>E5KWG9_4ee6_0_301
MSESADLTELYSIIEKTAQVVDVTASHDKVWPILNAFQDVIADSVISFRASTGSSADDLDCRF
TMLPKGLDPYARALEHGLTPKTDHPVGSLLKEVHENLPITSCGVDFGVAGGFTKTWSFPSA
EKLGKVSELVKLPSIPDAVAANRDFFEKWGIADMVSTVGIDYSKRTMNLYFGGGVGDRVPA
GVFEEKGVRAILGELGLAAPSEELLKFCERSFVIYVTLSWDSPKINRFTYSVMTPEPLGLPVDL
APTFERLIKSAPYDTEGRNYVYGIASTPKGEYHKIASYYQWQKRVEKLLRSDG
00000000000000000000000000000000000000000000000000000000000000000000000000000000000

00000000000000000000000000000000000000000000000000000000000000000000000000000000000
00000000000000000000000000000000000000000000000000000000000000000000000000000000000
00000000000000000000000000000000000000000001111111111
>P0CE80_3rlh_0_305
MLPYIVLVLGCWSVLSQAAQTDDEERAGNRRPIWIMGHMVNAIGQIDEFVNLGANSIETD
VSFDDNANPEYTYHGIPCDCGRNCKKYENFNDFLKGLRSATTPGNSKYQEKLVLVVFDLKT
GSLYDNQANDAGKKLAKNLLQHYWNNGNNGGRAYIVLSIPDLNHYPLIKGFKDQLTKDG
HPELMDKVGHDFSGNDDIGDVGKAYKKAGITGHIWQSDGITNCLPRGLSRVNAAVANRD
SANGFINKVYYWTVDKRSTTRDALDAGVDGIMTNYPDVITDVLNEAAYKKKFRVATYDEN
PWVTFKK
11111111111111111111111111100000000000000000000000000000000000000000000000000000000
00000000000000000000000000000000000000000000000000000000000000000000000000000000000
00000000000000000000000000000000000000000000000000000000000000000000000000000000000
00000000000000000000000000000000000000000000000000000001
>P38913_2wsi_0_305
MQLSKAAEMCYEITNSYLHIDQKSQIIASTQEAIRLTRKYLLSEIFVRWSPLNGEISFSYNGGK
DCQVLLLLYLSCLWEYFFIKAQNSQFDFEFQSFPMQRLPTVFIDQEETFPTLENFVLETSERYC
LSLYESQRQSGASVNMADAFRDFIKIYPETEAIVIGIRHTDPFGEALKPIQRTDSNWPDFMRL
QPLLHWDLTNIWSFLLYSNEPICGLYGKGFTSIGGINNSLPNPHLRKDSNNPALHFEWEIIH
AFGKDAEGERSSAINTSPISVVDKERFSKYHDNYYPGWYLVDDTLERAGRIKN
00000000000000000000000000000000000000000000000000000000000000000000000000000000000
00000111111110000000000000000000000000000000000000000011111000000000000000000000000
00000000000000000000000000000000000000000000000000000000000000000000000000000000000
000000000111100000000000000000000000011000000000000000011
>P96762_3tdw_0_305
MKQNKLHYTTMIMTQFPDISIQSVESLGEGFRNYAILVNGDWVFRFPKSQQGADELNKEIQ
LLPLLVGCVKVNIPQYVYIGKRSDGNPFVGYRKVQGQILGEDGMAVFPDDAKDRLALQLA
EFMNELSAFPVETAISAGVPVTNLKNKILLLSEAVEDQVFPLLDESLRDYLTLRFQSYMTHPV
YTRYTPRLIHGDLSPDHFLTNLNSRQTPLTGIIDFGDAAISDPDYDYVYLLEDCGELFTRQVM
AYRGEVDLDTHIRKVSLFVTFDQVSYLLEGLRARDQDWISEGLLELLEEDKANNFGANSA
11100000000000000000000000000000000000000000000000000000000000000000000000000000000
00000000000000000000000000000000000000000000000000000000000000000000000000000000000
00000000000000000000000000000000000000000000000000000000000000000000000000000000000
00000000000000000000000000000000000000000000000000000001
>P0A108_2ei1_0_301
MSKQAAVIELGYMGISVKDPDAWKSFATDMLGLQVLDEGEKDRFYLRMDYWHHRIVVHH
NGQDDLEYLGWRVAGKPEFEALGQKLIDAGYKIRICDKVEAQERMVLGLMKTEDPGGNP
TEIFWGPRIDMSNPFHPGRPLHGKFVTGDQGLGHCIVRQTDVAEAHKFYSLLGFRGDVEYR
IPLPNGMTAELSFMHCNARDHSIAFGAMPAAKRLNHLMLEYTHMEDLGYTHQQFVKNEI
DIALQLGIHANDKALTFYGATPSGWLIEPGWRGATAIDEAEYYVGDIFGHGVEATGYGLDV
KLS
11110000000000000000000000000000000000000000000000000000000000000000000000000000000
00000000000000000000000000000000000000000000000000000000000000000000000000000000000
00000000000000011111000000000000000000000000000000000000000000000000000000000000000
00000000000000000000000000000000000000000000000000000
>A6LDL3_3l23_32_333
ALGVVDEVHAGKEIGLQIYSLSQELYKGDVAANLRKVKDMGYSKLELAGYGKGAIGGVPM
MDFKKMAEDAGLKIISSHVNPVDTSISDPFKAMIFKYSKEVTPKIMEYWKATAADHAKLGC
KYLIQPMMPTITTHDEAKLVCDIFNQASDVIKAEGIATGFGYHNHNMEFNRVATKEQQEK
VKGNPFAAFMKVGDQIYDLMLKDTDPSKVYFEMDVYWTVMGQNDPVEYMQKHPDRIKV
LHIKDRAVFGQSGMMNFEMIFKQMYANGIKDYFVELEQMPDGRTQFAGVKDCADYLIKA

PFVK
11111111110000000000000000000000000000000000000000000000000000000000000000000000
00000000000000000000000000000000000000000000000000000000000000000000000000000000
00000000000001111111111000000000000000000000000000000000000000000000000000000000
000000000000000000000000000000000000000000000000000
>B5DCA0_4awy_0_302
MKRRFTLLGSVVALALSSTALASDAPASRGCADDAGWNDPAMPLKVYGNTWYVGTCGIS
ALLVTSDAGHILVDAATPQAGPQILANIRALGFRPEDVRAIVFSHEHFDHAGSLAELQKAT
GAPVYARAPAIDTLKRGLPDRTDPQFEVAEPVAPVANIVTLADDGVVSVGPLALTAVASPG
HTPGGTSWTWRSCEGDDCRQMVYADSLTAISDDVFRYSDDAAHPGYLAAFRNTLARVAA
LDCDILVTPHPSASGLWNRIGPRAAAPLMDTTACRRYAQGARQRLEKRLAEEAATSPSSGA
RP
11111111111111111111111111000000000000000000000000000000000000000000000000000000
00000000000000000000000000000000000000000000000000000000000000000000000000000000
00000000000000000000000000000000000000000000000000000000000000000000000000000000
0000000000000000000000000000000000000000000001111111
>P0A9J4_1yon_0_302
MKITVLGCGALGQLWLTALCKQGHEVQGWLRVPQPYCSVNLVETDGSIFNESLTANDPDF
LATSDLLLVTLKAWQVSDAVKSLASTLPVTTPILLIHNGMGTIEELQNIQQPLLMGTTTHAA
RRDGNVIIHVANGITHIGPARQQDGDYSYLADILQTVLPDVAWHNNIRAELWRKLAVNCVI
NPLTAIWNCPNGELRHHPQEIMQICEEVAAVIEREGHHTSAEDLRDYVMQVIDATAENISS
MLQDIRALRHTEIDYINGFLLRRARAHGIAVPENTRLFEMVKRKESEYERIGTGLPRPW
00000000000000000000000000000000000000000000000000000000000000000000000000000000
00000000000000000000000000000000000000000000000000000000000000000000000000000000
00000000000000000000000000000000000000000000000000000000000000000000000000000000
0000000000000000000000000000000000000000011111111111
>P0C6U6_3tlo_2939_3241
SGLKKMAQPSGCVERCVVRVCYGSTVLNGVWLGDTVTCPRHVIAPSTTVLIDYDHAYSTM
RLHNFSVSHNGVFLGVVGVTMHGSVLRIKVSQSNVHTPKHVFKTLKPGDSFNILACYEGIA
SGVFGVNLRTNFTIKGSFINGACGSPGYNVRNDGTVEFCYLHQIELGSGAHVGSDFTGSVY
GNFDDQPSLQVESANLMLSDNVVAFLYAALLNGCRWWLCSTRVNVDGFNEWAMANGYT
SVSSVECYSILAAKTGVSVEQLLASIQHLHEGFGGKNILGYSSLCDEFTLAEVVKQMYGVNL
Q
00000000000000000000000000000000000000000000000000000000000000000000000000000000
00000000000000000000000000000000000000000000000000000000000000000000000000000000
00000000000000000000000000000000000000000000000000000000000000000000000000000000
0000000000000000000000000000000000000000000000000001
>P71913_3go6_1_303
ANASETNVGPMAPRVCVVGSVNMDLTFVVDALPRPGETVLAASLTRTPGGKGANQAVAA
ARAGAQVQFSGAFGDDPAAAQLRAHLRANAVGLDRTVTVPGPSGTAIIVVDASAENTVLV
APGANAHLTPVPSAVANCDVLLTQLEIPVATALAAARAAQSADAVVMVNASPAGQDRSSL
QDLAAIADVVIANEHEANDWPSPPTHFVITLGVRGARYVGADGVFEVPAPTVTPVDTAGA
GDVFAGVLAANWPRNPGSPAERLRALRRACAAGALATLVSGVGDCAPAAAAIDAALRA
NRHNGS
11111111111000000000000000000000000000000000000000000000000000000000000000000000
00000000000000000000000000000000000000000000000000000000000000000000000000000000
00000000000000000000000000000000000000000000000000000000000000000000000000000000
0000000000000000000000000000000000000000000000011111
>P18153_3dzt_19_320
GPFDPEEMLFTFTRCMEDNLEDGPNRLPMLAKWKEWINEPVDSPATQCFGKCVLVRTGLY
DPVAQKFDASVIQEQFKAYPSLGEKSKVEAYANAVQQLPSTNNDCAAVFKAYDPVHKAHK

DTSKNLFHGNKELTKGLYEKLGKDIRQKKQSYFEFCENKYYPAGSDKRQQLCKIRQYTVLD
DALFKEHTDCVMKGIRYITKNNELDAEEVKRDFMQVNKDTKALEKVLNDCKSKEPSNAG
EKSWHYYKCLVESSVKDDFKEAFDYREVRSQIYAFNLPKKQVYSKPAVQSQVMEIDGKQCP
Q
00000000000000000000000000000000000000000000000000000000000000000000000000000000000
00000000000000000000000000000000000000000000000000000000000000000000000000000000000
00000000000000000000000000000000000000000000000000000000000000000000000000000000000
00000000000000000000000000000000000000011110000000001
>Q8NBK3_1y1f_72_373
EANAPGPVPGERQLAHSKMVPIPAGVFTMGTDDPQIKQDGEAPARRVTIDAFYMDAYEVS
NTEFEKFVNSTGYLTEAEKFGDSFVFEGMLSEQVKTNIQQAVAAAPWWLPVKGANWRHP
EGPDSTILHRPDHPVLHVSWNDAVAYCTWAGKRLPTEAEWEYSCRGGLHNRLFPWGNKL
QPKGQHYANIWQGEFPVTNTGEDGFQGTAPVDAFPPNGYGLYNIVGNAWEWTSDWWTV
HHSVEETLNPKGPPSGKDRVKKGGSYMCHRSYCYRYRCAARSQNTPDSSASNLGFRCAAD
RLPTMD
11111111111110000000000000000000000000000000000000000000000000000000000000000000000
00000001111111111111100000000000000000000000000000000000000000000000000000000000000
00000000000000000000000000000000000000000000000000000000000000000000000000000000000
00000000000000000000000000000000000000000000000000111
>Q7W019_2qpq_29_329
ATGDFPNKPLDIIVTFPPGGGTDMLARLIGNYLTESLGQTAVVENRPGASGNVGARLVADR
APDGYSLLMVNSSFAVNPGVFRNLPFDPKKDFAAVINVAYVPSVFVVPAGSKYKTLGELMA
AAKQTNTQVTYGSCGNGTPQHLAGELLNVSAKTHMVHVPYKGCGPALNDVLGSQIGLA
VVTASSAIPFIKAGKLQALAVTSKERSALLPEVPTVAEQGVAGYELNQWHGLLVPGATPMA
VRQKLYDGIAKVMQRDDVQKKLADLGYSTASDGPEVFQKMVETDIDRFSALTKQIGLKVD
11111000000000000000000000000000000000000000000000000000000000000000000000000000000
00000000000000000000000000000000000000000000000000000000000000000000000000000000000
00000000000000000000000000000000000000000000000000000000000000000000000000000000000
0000000000000000000000000000000000000000000000000000
>Q9KGN3_2ii1_0_299
MIRLSNENTIFFMDKENVPIASCQSGDTVIFETKDCFSDQITNEEQALTSIDFNRVNPATGPLY
VEGARRGDMLEIEILDIKVGKQGVMTAAPGLGALGESLNSPTTKLFPIEGDDVVYSTGLRLP
LQPMIGVIGTAPPGEPINNGTPGPHGGNLDTKDIKPGTTVYLPVEVDGALLALGDLHAAM
GDGEILICGVEIAGTVTLKVNVKKERMFPLPALKTDTHFMTIASAETLDAAAVQATKNMAT
FLANRTALSIEEAGMLLSGAGDLYVSQIVNPLKTARFSLALHYFEKLGVDLCN
00000000000000000000000000000000000000000000000000000000000000000000000000000000000
00000000000000000000000000000000000000000000000000000000000000000000000000000000000
00000000000000000000000000000000000000000000000000000000000000000000000000000000000
000000000000000000000000000000000000000000000000111
>Q5SLF5_2cw5_0_254
MRPVYFLSDFGLEDPYVAVVKAVLAERAPGPAVVDLAHALPPQDLRRAAYALFEALPYLPE
GAVVLAVVDPGVGTARRAVAALGRWTYVGPDNGLFTLAWLLDPPRRAFLLEPPRPRPKAA
LPGWAPGEATFHGRDVFAPAAAHLALGLPPEGLGPEVPVETLARLPLALTEGPEGEVLTFD
RFGNAITTLLRAPVGGFVEVGGRRVPVRRTFGEVPEGAPVAYLGSAGLLEVAVNRGSAREA
LGLKEGMPVRLL
00000000000000000000000001000000000000000000000000000000000000000000111110000000000
00000000000000000000000000000001111111111111111111000000000000000000000000000000000
00000000000000000000000000000000000000000000000011100000000000000000000000000000000
000000
>O75688_2p8e_1_296
GAFLDKPKTEKHNAHGAGNGLRYGLSSMQGWRVEMEDAHTAVVGIPHGLEDWSFFAVY

DGHAGSRVANYCSTHLLEHITTNEDFRAAGKSGSALELSVENVKNGIRTGFLKIDEYMRNF
SDLRNGMDRSGSTAVGVMISPKHIYFINCGDSRAVLYRNGQVCFSTQDHKPCNPREKERIQ
NAGGSVMIQRVNGSLAVSRALGDYDYKCVDGKGPTEQLVSPEPEVYEILRAEEDEFIILACD
GIWDVMSNEELCEYVKSRLEVSDDLENVCNWVVDTCLHKGSRDNMSIVLVCFSN
11000000000000000000000000000000000000000000000000000000000000000000000000000000000
00111111111110000000000000000000000000000000000000000000000000000000000000000000000
00000000000000000000000000000000000000000000000000000000000000000000000000000000000
00000000000000000000000000000000000000000000011
>Q8U4G1_1mjf_0_280
MERAFIEWYPRGYGVAFKIKKKIYEKLSKYQKIEVYETEGFGRLLALDGTVQLVTLGERSYH
EPLVHPAMLAHPKPKRVLVIGGGDGGTVREVLQHDVDEVIMVEIDEDVIMVSKDLIKIDNG
LLEAMLNGKHEKAKLTIGDGFEFIKNNRGFDVIIADSTDPVGPAKVLFSEEFYRYVVYDALN
NPGIYVTQAGSVYLFTDELISAYKEMKKVFDRVYYYSFPVIGYASPWAFLVGVKGDIDFTKID
RERAKKLQLEYYDPLMHETLFQMPKYIRETLQRL
11100000000000000000000000000000000000000000000000000000000000000000000000000000000
00000000000000000000000000000000000000000000000000000000000000000000000000000000111
11000000000000000000000000000000000000000000000000000000000000000000000000000000000
00000000000000000000000000000011
>P31007_3tvt_617_969
TQKRSLYVRALFDYDPNRDDGLPSRGLPFKHGDILHVTNASDDEWWQARRVLGDNEDEQ
IGIVPSKRRWERKMRARDRSVKSEENVLSYEAVQRLSINYTRPVIILGPLKDRINDDLISEYPD
KFGSCVPHTTRPKREYEVDGRDYHFVSSREQMERDIQNHLFIEAGQYNDNLYGTSVASVRE
VAEKGKHCILDVSGNAIKRLQVAQLYPVAVFIKPKSVDSVMEMNRRMTEEQAKKTYERAIK
MEQEFGEYFTGVVQGDTIEEIYSKVKSMIWSQSGPTIWVPSKESL
11110000000000001111111110000000000000000000000000001111111100000000000000011111111
11000000000000000000000000000000000000000000000000000000000000000000000000000000000
00000000000000000000000000000000000000000000000000000000000000000000000000000000000
00000000000000000000000000000000000001111
>Q9C8L4_2gcu_50_293
KLLFRQLFENESSTFTYLLADVSHPDKPALLIDPVDKTVDRDLKLIDELGLKLIYAMNTHVH
ADHVTGTGLLKTKLPGVKSVISKASGSKADLFLEPGDKVSIGDIYLEVRATPGHTAGCVTYV
TGEGADQPQPRMAFTGDAVLIRGCGRTDFQEGSSDQLYESVHSQIFTLPKDTLIYPAHDYK
GFEVSTVGEEMQHNPRLTKDKETFKTIMSNLNLSYPKMIDVAVPANMVCGLQDVPSQAN
00000000000000000000000000000000000000000000000000000000000000000000000000000000000
00000000000000000000000000000000000000000000000000000000000000000000000000000000000
000000000000000000000000000000000000000000000000000000000000000000000000000001
>Q58643_2yv1_0_293
MVLRDKMILLDENTKAIVQGITGRQGSFHTKKMLECGTKIVGGVTPGKGGQNVHGVPVF
DTVKEAVKETDANASVIFVPAPFAKDAVFEAIDAGIELIVVITEHIPVHDTMEFVNYAEDVG
VKIIGPNTPGIASPKVGKLGIIPMEVLKEGSVGMVSRSGTLTYEIAHQIKKAGFGVSTCVGIG
GDPIVGLRYKEVLDLFEKDDETEAIVMIGEIGGGAEEEAAKFIEKMKKPVIGYIAGQSAPEG
KRMGHAGAIVEKGKGTAESKMKALEEAGYVAKNISDIPKLLAGILGK
11111100000000000000000000000000000000000000000000000000000000000000000000000000000
00000000000000000000000000000000000000000000000000000000000000000000000000000000000
00000000000000000000000000000000000000000000000000000000000000000000000000011111111
111111111110000000000000000000000000000000001
>Q8TZN6_1nnh_0_293
MNAVEIISREISPTLDIQTKILEYMTDFFVKEGFKWLLPVIISPITDPLWPDPAGEGMEPAEVEI
YGVKMRLTHSMILHKQLAIAMGLKKIFVLSPNIRLESRQKDDGRHAYEFTQLDFEVERAKM
EDIMRLIERLVYGLFRKAEEWTGREFPKTKRFEVFEYSEVLEEFGSDEKASQEMEEPFWIINIP
REFYDREVDGFWRNYDLILPYGYGEVASGGEREWEYEKIVAKIRKAGLNEDSFRPYLEIAKA

GKLKPSAGAGIGVERLVRFIVGAKHIAEVQPFPRIPGIPAVI
10000000000000000000000000000000000000000000000000000000000000000000000000000000000
00000000000000000000000000000000000000000000000000000000000000000000000000000000000
00000000000000000000000000000000000000000000000000000000000000000000000000000000000
000000000000000000000000000000000000000000000
>Q75WT9_3phz_0_285
MSFQGHGIYYIASAYVANTRLALSEDSSANKSPDVIISSDAVDPLNNLWLIEPVGEADTYTVR
NAFAGSYMDLAGHAATDGTAIIGYRPTGGDNQKWIISQINDVWKIKSKETGTFVTLLNGD
GGGTGTVVGWQNITNNTSQNWTFQKLSQTGANVHATLLACPALRQDFKSYLSDGLYLVL
TRDQISSIWQASGLGSTPWRSEIFDCDDFATVFKGAVAKWGNENFKANGFALLCGLMFGSK
SSGAHAYNWFVERGNFSTVTFFEPQNGTYSANAWDYKAYFGLF
10000000000000000000000001111000000000000000000000000000000000000000000000000000000
00000000000000000000000000000000000000011100000000000000000000000000000000000000000
00000000000000000000000000000000000000000000000000000000000000000000000000000000000
0000000000000000000000000000000000000
>Q62DH4_3lou_0_290
MSVPQRPHQFVLTLSCPSAAGQVAAVVGLLDRHRCYVDELTVFDDDLSARFFVRCVFHAT
DDADALRVDALRREFEPIAERFRMQWAIHDVAARPKVLIMVSKLEHCLADLLFRWKMGEL
KMDIVGIVSNHPDFAPLAAQHGLPFRHFPITADTKAQQEAQWLDVFETSGAELVILARYM
QVLSPEASARLANRAINIHHSFLPGFKGAKPYHQAHARGVKLIGATAHFVTDDLDEGPIIE
QVVERVDHSYRPEQLLAVGRDVECITLARAVKAFIERRVFLNGDRTVVFQ
11111100000000000000000000000000000000000000000000000000111100000000000000000000000
00000000000000000000000000000000000000000000000000000000000000000000000000000000000
00000000000000000000000000000000000000000000000000000000000000000000000000000000000
000000000000000000000000000000000000000000
>Q833Y3_2r3b_0_290
MRYLSKDILEEVITQRPSDSYKSNFGRVVLIGGNRQYGGAIIMSTEACINSGAGLTTVITDVK
NHGPLHARCPEAMVVGFEETVLLTNVVEQADVILIGPGLGLDATAQQILKMVLAQHQKQ
QWLIIDGSAITLFSQGNFSLTYPEKVVFTPHQMEWQRLSHLPIEQQTLANNQRQQAKLGSTI
VLKSHRTTIFHAGEPFQNTGGNPGMATGGTGDTLAGIIAGFLAQFKPTIETIAGAVYLHSLI
GDDLAKTDYVVLPTKISQALPTYMKKYAQPHTAPDSELLEQKRSR
00000000000000000000000000000000000000000000000000000000000000000000000000000000000
00000000000000000000000000000000000000000000000000000000000000000000000000000000000
00000000000000000000000000000000000000000000000000000000000000000000000000000000000
000000000000000000000000000111111111111111
>Q2K0Z9_3miz_49_338
AARLIRSSRSNTFGIITDYVSTTPYSVDIVRGIQDWANANGKTILIANTGGSSEREVEIWKMF
QSHRIDGVLYVTMYRRIVDPESGDVSIPTVMINCRPQTRELLPSIEPDDYQGARDLTRYLLER
GHRRIGYIRLNPILLGAELRLDAFRRTTSEFGLTENDLSISLGMDGPVGAENNYVFAAATEM
LKQDDRPTAIMSGNDEMAIQIYIAAMALGLRIPQDVSIVGFDDFRTVTMALKPELTTAALPY
YDLGREGAKWLNDLIAGEKIYPGSRVVSCKLVERSSAAFG
11111111000000000000000000000000000000000000000000000000000000000000000000000000000
00000000000000000000000000000000000000000000000000000000000000000000000000000000000
00000000000000000000000000000000000000000000000000000000000000000000000000000000000
00000000000000000000000000000000000000000
>Q8A183_3mpr_20_309
SYQPTSLTVASYNLRNANGSDSARGDGWGQRYPVIAQMVQYHDFDIFGTQECFLHQLKD
MKEALPGYDYIGVGRDDGKDKGEHSAIFYRTDKFDIVEKGDFWLSETPDVPSKGWDAVLP
RICSWGHFKCKDTGFEFLFFNLHMDHIGKKARVESAFLVQEKMKELGRGKNLPAILTGDFN
VDQTHQSYDAFVSKGVLCDSYEKCDYRYALNGTFNNFDPNSFTESRIDHIFVSPSFHVKRYG
VLTDTYRSVRENSKKEDVRDCPEEITIKAYEARTPSDHFPVKVELVFD

00000000000000000000000000000000000000000000000000000000000000000000000000000000000
00000000000000000000000000000000000000000000000000000000000000000000000000000000000
00000000000000000000000000000000000000000000000000000000000000000000000000000000000
00001111111111111111000000000000000000000
>Q9VY78_2yv7_0_259
MSEVESQQSQETNGSSKFDVPEIELIIKASTIDGRRKGACLFCQEYFMDLYLLAELKTISLKVT
TVDMQKPPPDFRTNFEATHPPILIDNGLAILENEKIERHIMKNIPGGYNLFVQDKEVATLIEN
LYVKLKLMLVKKDEAKNNALLSHLRKINDHLSARNTRFLTGDTMCCFDCELMPRLQHIRV
AGKYFVDFEIPTHLTALWRYMYHMYQLDAFTQSCPADQDIINHYKLQQSLKMKKHEELET
PTFTTYIPIDISE
11111111111110000000000000000000000000000000000000000000000000000000000011111000000
00000000000000000000000000000000000000000000000000000000000000000000000000000000000
00000000000000000000000000000000000000000000000000000000000000000000000011111000000
00000000011
>P96116_1toa_22_307
FGSKDAAADGKPLVVTTIGMIADAVKNIAQGDVHLKGLMGPGVDPHLYTATAGDVEWLG
NADLILYNGLHLETKMGEVFSKLRGSRLVVAVSETIPVSQRLSLEEAEFDPHVWFDVKLWSY
SVKAVYESLCKLLPGKTREFTQRYQAYQQQLDKLDAYVRRKAQSLPAERRVLVTAHDAFGY
FSRAYGFEVKGLQGVSTASEASAHDMQELAAFIAQRKLPAIFIESSIPHKNVEALRDAVQAR
GHVVQIGGELFSDAMGDAGTSEGTYVGMVTHNIDTIVAALAR
11111111100000000000000000000000000000000000000000000000000000000000000000000000000
00000000000000000000000000000000000000000000000000000000000000000000000000000000000
00000000000000000000000000000000000000000000000000000000000000000000000000000000000
0000000000000000000000000000000000000
>Q9H9B1_2rfi_981_1265
NSQVWSALQMSKALQDSAPDRPSPVERIVSRDIARGYERIPIPCVNAVDSEPCPSNYKYVSQ
NCVTSPMNIDRNITHLQYCVCIDDCSSSNCMCGQLSMRCWYDKDGRLLPEFNMAEPPLIF
ECNHACSCWRNCRNRVVQNGLRARLQLYRTRDMGWGVRSLQDIPPGTFVCEYVGELISDS
EADVREEDSYLFDLDNKDGEVYCIDARFYGNVSRFINHHCEPNLVPVRVFMAHQDLRFPRI
AFFSTRLIEAGEQLGFDYGERFWDIKGKLFSCRCGSPKCRHS
11111111111111111111111100000000000000000000000000000000000000000000000000000000000
00000000000000000000000000000000000000000000000000000000000000000000000000000000000
00000000000000000000000000000000000000000000000000000000000000000000000000000000000
000000000000000000000000000000000000
>P72222_1t3q_0_287
MKFPAFSYRAPASLQEVIQVLADDPDARIIAGGQSLLPLLAFRLVYPSCLVDLRNVSELFEISQ
SAGILSVGAMVTHFRNKTDPTVAKCVPILPKVLAHVAHQAVRNRGTLGGSLAHADAGAE
MPFLMATLGATMYIASSAGVRSVSATDFMKGHYFTDLEAGEVLVRVEIPIPALHWEFDEYA
RRKGDYALVMAAAGLSMQGGRCVAARIALGAVEERAHQAIRANDFLVGKVIDESTAATA
AELATEGLEPRSDIHGSRDLRLSLAKAITQRVILKAAQGAMYAGA
00000000000000000000000000000000000000000000000000000000000000000000000000000000000
00000000000000000000000000000000000000000000000000000000000000000000000000000000000
00000000000000000000000000000000000000000000000000000000000000000000000000000000000
000000000000000000000000000000000000111
>Q45871_1ybi_5_292
VIQNSLNDKIVTISCKADTNLFFYQVAGNVSLFQQTRNYLERWRLIYDSNKAAYKIKSMDIH
NTNLVLTWNAPTHNISTQQDSNADNQYWLLLKDIGNNSFIIASYKNPNLVLYADTVARNL
KLSTLNNSNYIKFIIEDYIISDLNNFTCKISPILDLNKVVQQVDVTNLNVNLYTWDYGRNQK
WTIRYNEEKAAYQFFNTILSNGVLTWIFSNGNTVRVSSSNDQNNDAQYWLINPVSDTDETY
TITNLRDTTKALDLYGGQTANGTAIQVFNYHGDDNQKWNIRNP
11110000000000000000000000000000000000000000000000000000000000000000000000000000000

00000000000000000000000000000000000000000000000000000000000000000000000000000000000
00000000000000000000000000000000000000000000000000000000000000000000000000000000000
000000000000000000000000000000000000000
>Q4WEQ6_2y8v_0_287
MPPVPGRPEHRRVICYHQTLCPNRGDYVSVLPLVKNNTGVTHIIIAAFHLNEDPGHITLND
DPPDHEMYNPLWAEVPVLKRSGVKVMGMLGGAAQGSYRCLDGDQEKFERYYQPLLAMV
RRHQLDGLDLDVEEEMSLPGIIRLIDRLKLDLGDDFIITLAPVAAALLGIGNLSGFDYRQLEQ
QRGSKISWYNAQFYNGWGLAEDPRMYAAIVAQGWSPQRVVYGLLTNPGNGSQGYVPRER
IGPVLAVLVEQFPNFGGVMGWEYFNSIPGEQQSPWQWAAEMSLSMHM
11111110000000000000000000000000000000000000000000000000000000000000000000000000000
00000000000000000000000000000000000000000000000000000000000000000000000000000000000
00000000000000000000000000000000000000000000000000000000000000000000000000000000000
000000000000000000000000000000000000001
>A0QFP4_3rd7_0_283
MISTLEDVLSLLDLQQIDDAAFVGTQPDTPNHHIIGSQVAAQALMAAGRTTPGRLAHSMH
MYFLRRGDARQPIQYDVTPLRDGGTISSRRVTASQSGVVLFEALASFTIIADDVDWQQRMP
DVAGPSAVHGLEDLLAPYAEEFGDWWPQQRPFTMRYLDAPPRVALDLSDPPPPRLRIWLR
ANGEVTDDPLVNSCVVAYLSALTLLECVMTTMRTTPVGPRLSALVDHTIWFHRAADFTDW
LLFDQFSPSIVGRRGLATGTLYNRSGELVCIATQEGYFAEQRQ
00000000000000000000000000000000000000000000000000000000000000000000000000000000000
00000000000000000000000000000000000000000000000000000000000011111100000000000000000
00000000000000000000000000000000000000000000000000000000000000000000000000000000000
00000000000000000000000000000000011
>P0A4V6_1r88_26_298
AEPTAKAAPYENLMVPSPSMGRDIPVAFLAGGPHAVYLLDAFNAGPDVSNWVTAGNAM
NTLAGKGISVVAPAGGAYSMYTNWEQDGSKQWDTFLSAELPDWLAANRGLAPGGHAAV
GAAQGGYGAMALAAFHPDRFGFAGSMSGFLYPSNTTTNGAIAAGMQQFGGVDTNGMW
GAPQLGRWKWHDPWVHASLLAQNNTRVWVWSPTNPGASDPAAMIGQAAEAMGNSRM
FYNQYRSVGGHNGHFDFPASGDNGWGSWAPQLGAMSGDIVGAIR
11111100000000000000000000000000000000000000000000000000000000000000000000000000000
00000000000000000000000000000000000000000000000000000000000000000000000000000000000
00000000000000000000000000000000000000000000000000000000000000000000000000000000000
000000000000000000000000
>Q45462_3r6u_22_305
CSLPGLSAAADQTIKIGAQSMSESEIIASMLGQLIEHHTDLKTTTIKNLGSNAVQQQALMNG
EIDIAATRYTGDALTGTLRMEPEKDPDKALALTQREFKKRYDLKWYDSYGFDNTYAFTVSK
ELADQYHLETVSDVKKWAPQLKLGVDNYWMKLKGNGYQDFTKTYGMTFGGTYPMQIGL
VYDAVKSGKMDIVLAYSTDGRIKSYGLKMLKDDKQFFPPYDCSPVVPEKVLKEHPELEGIIK
KMLGKIDTATMQELNYEVDGNLKEPSVVAKEYLEKHRYFES
11111111111000000000000000000000000000000000000000000000000000000000000000000000000
00000000000000000000000000000000000000000000000000000000000000000000000000000000000
00000000000000000000000000000000000000000000000000000000000000000000000000000000000
00000000000000000000000000000000001
>Q9RUZ0_1sgj_0_283
MNAPPALLRSVLFAPGNRADLIAKLPRSAPDAVVIDLEDAVPGTAEAKAAARPVAHDAAR
DLIAAAPHLAVFVRVNALHSPYFEDDLSVLTPELSGVVVPKLEMGAEARQVAQMLQERSLP
LPILAGLETGAGVWNAREIMEVPEVAWAYFGAEDYTTDLGGKRTPGGLEVLYARSQVALA
ARLTGVAALDIVVTALNDPETFRADAEQGRALGYSGKLCIHPAQVALAHEYFGPTEADRA
RARALLDAAAAAAQRGHGAFSFEGQMVDEPMLAKARTLLSHEA
11100000000000000000000000000000000000000000000000000000000000000000000000000000000
00000000000000000000000000000000000000000000000000000000000000000000000000000000000

00000000000000000000000000000000000000000000000000000000000000000000111111111111111
11111111111111111111111111111111111
>F2Z6J5_3ayx_40_322
NKIAHAMETKPRTPVIWLHGLECTCCSESFIRSAHPLAKDVVLSMISLDYDDTLMAASGHA
AEAILDEIKEKYKGNYILAVEGNPPLNQDGMSCIIGGRPFSEQLKRMADDAKAIISWGSCAS
WGCVQAAKPNPTQATPVHKFLGGGYDKPIIKVPGCPPIAEVMTGVITYMLTFDRIPELDRQ
GRPKMFYSQRIHDKCYRRPHFDAGQFVEEWDDEGARKGYCLYKVGCKGPTTYNACSTVR
WNGGTSFPIQSGHGCIGCSEDGFWDKGSFYSRDTEMNAFG
11111111110000000000000000000000000000000000000000000000000000000000000000000000000
00000000000000000000000000000000000000000000000000000000000000000000000000000000000
00000000000000000000000000000000000000000000000000000000000000000000000000000000000
0000000000000000000000000000000000
>Q1GZM5_3gpi_1_275
SKILIAGCGDLGLELARRLTAQGHEVTGLRRSAQPMPAGVQTLIADVTRPDTLASIVHLRPE
ILVYCVAASEYSDEHYRLSYVEGLRNTLSALEGAPLQHVFFVSSTGVYGQEVEEWLDEDTPP
IAKDFSGKRMLEAEALLAAYSSTILRFSGIYGPGRLRMIRQAQTPEQWPARNAWTNRIHRD
DGAAFIAYLIQQRSHAVPERLYIVTDNQPLPVHDLLRWLADRQGIAYPAGATPPVQGNKKL
SNARLLASGYQLIYPDYVSGYGALLAAMR
00000000000000000000000000000000000000000000000000000000000000000000000000111110000
00000000000000000000000000000000000000000000000000000000000000000000000000000000000
00000000000000000000000000000000000000000000000000000000000000000000000000000000000
00000000000000000000000000
>Q9UNQ2_1zq9_30_312
NTGIGQHILKNPLIINSIIDKAALRPTDVVLEVGPGTGNMTVKLLEKAKKVVACELDPRLVA
ELHKRVQGTPVASKLQVLVGDVLKTDLPFFDTCVANLPYQISSPFVFKLLLHRPFFRCAILMF
QREFALRLVAKPGDKLYCRLSINTQLLARVDHLMKVGKNNFRPPPKVESSVVRIEPKNPPPP
INFQEWDGLVRITFVRKNKTLSAAFKSSAVQQLLEKNYRIHCSVHNIIIPEDFSIADKIQQILTS
TGFSDKRARSMDIDDFIRLLHGFNAEGIHFS
11111000000000000000000000000000000000000000000000000000000000000000000000000000000
00000000000000000000000000000000000000000000000000000000000000000000000000000000000
00000000000000000000000000000000000000000000000000000000000000000000000000000000000
0000000000000000000000000000000000
>P0ADG4_2qfl_0_266
MHPMLNIAVRAARKAGNLIAKNYETPDAVEASQKGSNDFVTNVDKAAEAVIIDTIRKSYP
QHTIITEESGELEGTDQDVQWVIDPLDGTTNFIKRLPHFAVSIAVRIKGRTEVAVVYDPMRNE
LFTATRGQGAQLNGYRLRGSTARDLDGTILATGFPFKAKQYATTYINIVGKLFNECADFRRT
GSAALDLAYVAAGRVDGFFEIGLRPWDFAAGELLVREAGGIVSDFTGGHNYMLTGNIVAG
NPRVVKAMLANMRDELSDALKR
00000000000000000000000000000000000000000000000000000000000000000000000000000000000
00000000000000000000000000000000000000000000000000000000000000000000000000000000000
00000000000000000000000000000000000000000000000000000000000000000000000000000000000
000000000000011111
>Q9K0A8_1rv9_0_258
MKTITETLNLAPKGKNFLTADWPAPANVKTLITTRNGGVSQGAYQSLNLGTHVGDNPEAV
RRNREIVQQQVGLPVAYLNQIHSTVVVNAAEALGGTPDADASVDDTGKVACAVMTADCL
PVLFCDRAGTAVAAAHAGWRGLAGGVLQNTIAAMKVPPVEMMAYLGPAISADAFEVGQ
DVFDAFCTPMPEAATAFEGIGSGKFLADLYALARLILKREGVGGVYGGTHCTVLERDTFFSY
RRDGATGRMASLIWLDGNAV
11111111111111000000000000000000000000000000000000000000000000000000000000000000000
00000000000000000000000000000000000000000000000000000000000000000000000000000000000
00000000000000000000000000000000000000000000000000000000000000000000000000000000000

0000000111
>O29777_3a1d_397_672
KGAELGILIKNADALEVAEKVTAVIFDKTGTLTKGKPEVTDLVPLNGDERELLRLAAIAERRS
EHPIAEAIVKKALEHGIELGEPEKVEVIAGEGVVADGILVGNKRLMEDFGVAVSNEVELALE
KLEREAKTAVIVARNGRVEGIIAVSDTLKESAKPAVQELKRMGIKVGMITGDNWRSAEAISR
ELNLDLVIAEVLPHQKSEEVKKLQAKEVVAFVGDGINDAPALAQADLGIAVGSGSDVAVES
GDIVLIRDDLRDVVAAIQLSRKTMSKIK
10000000000000000000000000000000000000000000000000000000000000000000000000000000000
00000000000000000000000000000000000000000000000000000000000000000000000000000000000
00000000000000000000000000000000000000000000000000000000000000000000000000000000000
000000000000000000000000111
>P16152_3bhm_1_276
SSGIHVALVTGGNKGIGLAIVRDLCRLFSGDVVLTARDVTRGQAAVQQLQAEGLSPRFHQL
DIDDLQSIRALRDFLRKEYGGLDVLVNNAGIAFKVADPTPFHIQAEVTMKTNFFGTRDVCT
ELLPLIKPQGRVVNVSSIMSVRALKSCSPELQQKFRSETITEEELVGLMNKFVEDTKKGVHQ
KEGWPSSAYGVTKIGVTVLSRIHARKLSEQRKGDKILLNACCPGWVRTDMAGPKATKSPEE
GAETPVYLALLPPDAEGPHGQFVSEKRVEQW
11000000000000000000000000000000000000000000000000000000000000000000000000000000000
00000000000000000000000000000000000000000000000000000000000000000000000000000000000
00000000000000000000000000000000000000000000000000000000000000000000000000000000000
000000000000000000000000000
>P00387_1umk_26_300
FQRSTPAITLESPDIKYPLRLIDREIISHDTRRFRFALPSPQHILGLPVGQHIYLSARIDGNLVVR
PYTPISSDDDKGFVDLVIKVYFKDTHPKFPAGGKMSQYLESMQIGDTIEFRGPSGLLVYQGK
GKFAIRPDKKSNPIIRTVKSVGMIAGGTGITPMLQVIRAIMKDPDDHTVCHLLFANQTEKDI
LLRPELEELRNKHSARFKLWYTLDRAPEAWDYGQGFVNEEMIRDHLPPPEEEPLVLMCGPP
PMIQYACLPNLDHVGHPTERCFVF
11110000000000000000000000000000000000000000000000000000000000000000000000000000000
00000000000000000000000000000000000000000000000000000000000000000000000000000000000
00000000000000000000000000000000000000000000000000000000000000000000000000000000000
00000000000000000000000000
>Q99WQ5_3h04_0_274
MTEIKYKVITKDAFALPYTIIKAKNQPTKGVIVYIHGGGLMFGKANDLSPQYIDILTEHYDLI
QLSYRLLPEVSLDCIIEDVYASFDAIQSQYSNCPIFTFGRSSGAYLSLLIARDRDIDGVIDFYGYS
RINTEPFKTTNSYYAKIAQSINETMIAQLTSPTPVVQDQIAQRFLIYVYARGTGKWINMINIA
DYTDSKYNIAPDELKTLPPVFIAHCNGDYDVPVEESEHIMNHVPHSTFERVNKNEHDFDRR
PNDEAITIYRKVVDFLNAITMV
11000000000000000000000000000000000000000000000000000000000000000000000000000000000
00000000000000000000000000000000000000000000000000000000000000000000000000000000000
00000000000000000000000000000000000000000000000000000000000000000000000000000000000
00000000000000000000000001
>Q9YAQ8_1wta_0_274
MFEITRPPGVRAHVGVIGGSGLYDPGIVENPVEVKVSTPYGNPSDFIVVGDVAGVKVAFLPR
HGRGHRIPPHAINYRANIWALKALGVKWVISVSAVGSLREDYRPGDFVVPDQFIDMTKNR
RHYTFYDGPVTVHVSMADPFCEDLRQRLIDSGRRLGYTVHERGTYVCIEGPRFSTRAESRV
WKDVFKADIIGMTLVPEINLACEAQLCYATLAMVTDYDVWADRPVTAEEVERVMISNVER
ARRMLYDVIPKLAGEPELERCSCCRALDTAAI
11000000000000000000000000000000000000000000000000000000000000000000000000000000000
00000000000000000000000000000000000000000000000000000000000000000000000000000000000
00000000000000000000000000000000000000000000000000000000000000000000000000000000000
00000000000000000000000000

>P84308_2ogx_1_274
TDTTNSIKHVISPLARQTLQDRDLTRPVAGKRPIRLLPWLQVVKIGGRVMDRGADAILPLVE
ELRKLLPEHRLLILTGAGVRARHVFSVGLDLGLPVGSLAPLAASEAGQNGHILAAMLASEG
VSYVEHPTVADQLAIHLSATRAVVGSAFPPYHHHEFPGSRIPPHRADTGAFLLADAFGAAG
LTIVENVDGIYTADPNGPDRGQARFLPETSATDLAKSEGPLPVDRALLDVMATARHIERVQ
VVNGLVPGRLTAALRGEHVGTLIRTGVRP
11111111111111111111111111111000000000000000000000000000000000000000000000000000000
00000000000000000000000000000000000000000000000000000000000000000000000000000000000
00000000000000000000000000000000000000000000000000000000000000000000000000000000000
0000000000000000000000000
>Q4FZB7_3s8p_62_334
QSRYVPSSGMSAKELCENDDLATSLVLDPYLGFQTHKMNTSAFPSRSSRHFSKSDSFSHNNP
VRFRPIKGRQEELKEVIERFKKDEHLEKAFKCLTSGEWARHYFLNKNKMQEKLFKEHVFIYL
RMFATDSGFEILPCNRYSSEQNGAKIVATKEWKRNDKIELLVGCIAELSEIEENMLLRHGEN
DFSVMYSTRKNCAQLWLGPAAFINHDCRPNCKFVSTGRDTACVKALRDIEPGEEISCYYGD
GFFGENNEFCECYTCERRGTGAFKSR
11111111000000000000000000000000000000001111111111111111111111111000000000000000000
00000000000000000000000000000000000000000000000000000000000000000000000000000000000
00000000000000000000000000000000000000000000000000000000000000000000000000000000000
000000000000000000000001
>P0A632_3qja_0_271
MSPATVLDSILEGVRADVAAREASVSLSEIKAAAAAAPPPLDVMAALREPGIGVIAEVKRAS
PSAGALATIADPAKLAQAYQDGGARIVSVVTEQRRFQGSLDDLDAVRASVSIPVLRKDFVV
QPYQIHEARAHGADMLLLIVAALEQSVLVSMLDRTESLGMTALVEVHTEQEADRALKAGA
KVIGVNARDLMTLDVDRDCFARIAPGLPSSVIRIAESGVRGTADLLAYAGAGADAVLVGEG
LVTSGDPRAAVADLVTAGTHPSCPKPAR
11100000000000000000000000000000000000000000000000000000000111111111111100000000000
00000000000000000000000000000000000000000000000000000000000000000000000000000000000
00000000000000000000000000000000000000000000000000000000000000000000000000000000000
00000000000000000000011
>Q2SYZ1_3vav_0_270
MTYLQESSRPAVTVPKLQAMREAGEKIAMLTCYDASFAALLDRANVDVQLIGDSLGNVLQ
GQTTTLPVTLDDIAYHTACVARAQPRALIVADLPFGTYGTPADAFASAVKLMRAGAQMVK
FEGGEWLAETVRFLVERAVPVCAHVGLTPQSVHAFGGFKVQGKTEAGAAQLLRDARAVE
EAGAQLIVLEAVPTLVAAEVTRELSIPTIGIGAGAECSGQVLVLHDMLGVFPGKRPRFVKDF
MQGQPSIFAAVEAYVRAVKDGSFPGPEHSF
11111111000000000000000000000000000000000000000000000000000000000000000000000000000
00000000000000000000000000000000000000000000000000000000000000000000000001110000000
00000000000000000000000000000000000000000000000000000000000000000000000000000000000
0000000000000000000000
>Q5SJC0_1wxj_0_270
MTTLEAFAKARSEGRAALIPYLTAGFPSREGFLQAVEEVLPYADLLEIGLPYSDPLGDGPVIQ
RASELALRKGMSVQGALELVREVRALTEKPLFLMTYLNPVLAWGPERFFGLFKQAGATGVI
LPDLPPDEDPGLVRLAQEIGLETVFLLAPTSTDARIATVVRHATGFVYAVSVTGVTGMRERL
PEEVKDLVRRIKARTALPVAVGFGVSGKATAAQAAVADGVVVGSALVRALEEGRSLAPLLQ
EIRQGLQRLEANPGLKESSKKPLP
00000000000000000000000000000000000000000000000000000000000001111100000000000000000
00000000000000000000000000000000000000000000000000000000000000000000000000000000000
00000000011111111111110000000000000000000000000000000000000000000000000000000000000
0000000011111111111111
>Q10QA5_4ih9_50_317

PSGAKLLQILNVRVVGSGERVVVLSHGFGTDQSAWSRVLPYLTRDHRVVLYDLVCAGSVNP
DHFDFRRYDNLDAYVDDLLAILDALRIPRCAFVGHSVSAMIGILASIRRPDLFAKLVLIGASP
RFLNDSDYHGGFELEEIQQVFDAMGANYSAWATGYAPLAVGADVPAAVQEFSRTLFNMRP
DISLHVCQTVFKTDLRGVLGMVRAPCVVVQTTRDVSVPASVAAYLKAHLGGRTTVEFLQTE
GHLPHLSAPSLLAQVLRRALARY
11100000000000000000000000000000000000000000000000000000000000000000000000000000000
00000000000000000000000000000000000000000000000000000000000000000000000000000000000
00000000000000000000000000000000000000000000000000000000000000000000000000000000000
0000000000000000000
>P52700_2fu8_21_289
AEVPLPQLRAYTVDASWLQPMAPLQIADHTWQIGTEDLTALLVQTPDGAVLLDGGMPQM
ASHLLDNMKARGVTPRDLRLILLSHAHADHAGPVAELKRRTGAKVAANAESAVLLARGG
SDDLHFGDGITYPPANADRIVMDGEVITVGGIVFTAHFMAGHTPGSTAWTWTDTRNGKPV
RIAYADSLSAPGYQLQGNPRYPHLIEDYRRSFATVRALPCDVLLTPHPGASNWDYAAGARA
GAKALTCKAYADAAEQKFDGQLAKETAGAR
10000000000000000000000000000000000000000000000000000000000000000000000000000000000
00000000000000000000000000000000000000000000000000000000000000000000000000000000000
00000000000000000000000000000000000000000000000000000000000000000000000000000000000
00000000000000000011
>Q4Q3K2_3riv_34_302
EEPPFDIRALRADIEDMISEKLELGPSLIRLAWHEAASYDCFKKDGSPNSASMRFKPECLYAG
NKGLDIPRKALETLKKKYPQISYADLWVLAAYVAIEYMGGPTIPFCWGRVDAKDGSVCGPD
GRLPDGSKTQSHVREVFRRLGFNDQETVALIGAHTCGECHIEFSGYHGPWTHDKNGFDNS
FFTQLLDEDWVLNPKVEQMQLMDRATTKLMMLPSDVCLLLDPSYRKYVELYAKDNDRFN
KDFANAFKKLTELGTRNLHKAPASES
00000000000000000000000000000000000000000000000000000000000000000000000000000000000
00000000000000000000000000000000000000000000000000000000000000000000000000000000000
00000000000000000000000000000000000000000000000000000000000000000000000000000000000
00000000000000000111
>Q72EC8_2xvx_28_296
GHGAPKAQKTGILLVAFGTSVEEARPALDKMGDRVRAAHPDIPVRWAYTAKMIRAKLRAE
GIAAPSPAEALAGMAEEGFTHVAVQSLHTIPGEEFHGLLETAHAFQGLPKGLTRVSVGLPLI
GTTADAEAVAEALVASLPADRKPGEPVVFMGHGTPHPADICYPGLQYYLWRLDPDLLVGT
VEGSPSFDNVMAELDVRKAKRVWLMPLMAVAGDHARNDMAGDEDDSWTSQLARRGIE
AKPVLHGTAESDAVAAIWLRHLDDALARLN
11111110000000000000000000000000000000000000000000000000000000000000000000000000000
00000000000000000000000000000000000000000000000000000000000000000000000000000000000
00000000000000000000000000000000000000000000000000000000000000000000000000000000000
00000000000000000000
>Q02150_4gyf_1_268
KKLDYHFHSHFSADSEELPRKHVTEAIAHGLEEICFTEHRDFYFPGMDFSLNLPEYFQEINR
LQAEFKDKIKIKIGLEMGIDLRFKSEINQFIDSAPFDFVIASVHEIGDIEVYDGTEFYLQKIKEE
AQREYLLACLDVVQNFENYNSFGHLDYVARYGPYTDKSIKFAENREILFEILRALASKEKAL
EINTRLFDDPKTEQFYSDLLINFKKLGGKFITLGTDSHIAKRDWLSIHKARTLIKKAGFHELA
TFSGMKIDKNKKSIKE
00000000000000000000000000000000000000000000000000000000000000000000000000000000000
00000000000000000000000000000000000000000000000000000000000000000000000000000000000
00000000000000000000000000000000000000000000000000000000000000000000000000000000000
0000000000001111111
>Q8AAQ3_3b8b_0_267
MAAIDAALKAGEKILSIYEDPKSDFEIERKADNSPLTIADRKAHEAIVAILNETPFPVLSEEGK

HMDYAVRRGWDTLWIVDPLDGTKEFIKRNGEFTVNIALVQNAVPVMGVIYVPVKKELYFA
VEGTGAYKCSGIVGLEDEGVTLQQMIEKSERMPLADARDHFIAVASRSHLTPETETYIADLK
KKHGNVELISSGSSIKICLVAEGKADVYPRFAPTMEWDTAAGHAIARAAGMEVYQAGKEE
PLRYNKEDLLNPWFIVEAKRER
00000000000000000000000001110000000000000000000000000000000000100000000000000000000
00000000000000000000000000000000000000000000000000000000000000000000000000000000000
00000000000000000000000000000000000000000000000000000000000000000000000000000000000
0000000000000000001
>Q8NQY2_4dbf_0_267
MRFGRIATPDGMCFCSIEGEGDDVANLTAREIEGTPFTEPKFTGREWPLKDVRLLAPMLPSK
VVAIGRNYADHVAEVFKKSAESLPPTLFLKPPTAVTGPESPIRIPSFATKVEFEGELAVVIGKPC
KNVKADDWKSVVLGFTIINDVSSRDLQFADGQWARAKGIDTFGPIGPWIETDINSIDLDNL
PIKARLTHDGETQLKQDSNSNQMIMKMGEIIEFITASMTLLPGDVIATGSPAGTEAMVDGD
YIEIEIPGIGKLGNPVVDA
00000000000000000000000000000000000000000000000000000000000000000000000011111111111
00000000000000000000000000000000000000000000000000000000000000000000000000000000000
00000000000000000000000000000000000000000000000000000000000000000000000000000000000
0000000000000000000
>P76469_2vws_0_266
MNALLSNPFKERLRKGEVQIGLWLSSTTAYMAEIAATSGYDWLLIDGEHAPNTIQDLYHQL
QAVAPYASQPVIRPVEGSKPLIKQVLDIGAQTLLIPMVDTAEQARQVVSATRYPPYGERGVG
ASVARAARWGRIENYMAQVNDSLCLLVQVESKTALDNLDEILDVEGIDGVFIGPADLSASL
GYPDNAGHPEVQRIIETSIRRIRAAGKAAGFLAVAPDMAQQCLAWGANFVAVGVDTMLYS
DALDQRLAMFKSGKNGPRIKGSY
00000000000000000000000000000000000000000000000000000000000000000000000000000000000
00000000000000000000000000000000000000000000000000000000000000000000000000000000000
00000000000000000000000000000000000000000000000000000000000000000000000000000000000
000000011111111111
>P10275_2ama_653_918
ETTQKLTVSHIEGYECQPIFLNVLEAIEPGVVCAGHDNNQPDSFAALLSSLNELGERQLVHV
VKWAKALPGFRNLHVDDQMAVIQYSWMGLMVFAMGWRSFTNVNSRMLYFAPDLVFNEY
RMHKSRMYSQCVRMRHLSQEFGWLQITPQEFLCMKALLLFSIIPVDGLKNQKFFDELRMN
YIKELDRIIACKRKNPTSCSRRFYQLTKLLDSVQPIARELHQFTFDLLIKSHMVSVDFPEMMA
EIISVQVPKILSGKVKPIYFHTQ
11111111111111111000000000000000000000000000000000000000000000000000000000000000000
00000000000000000000000000000000000000000000000000000000000000000000000000000000000
00000000000000000000000011111100000000000000000000000000000000000000000000000000000
00000000000000000
>O95050_2a14_0_262
MKGGFTGGDEYQKHFLPRDYLATYYSFDGSPSPEAEMLKFNLECLHKTFGPGGLQGDTLID
IGSGPTIYQVLAACDSFQDITLSDFTDRNREELEKWLKKEPGAYDWTPAVKFACELEGNSGR
WEEKEEKLRAAVKRVLKCDVHLGNPLAPAVLPLADCVLTLLAMECACCSLDAYRAALCN
LASLLKPGGHLVTTVTLRLPSYMVGKREFSCVALEKEEVEQAVLDAGFDIEQLLHSPQSYSV
TNAANNGVCFIVARKKPGP
11110000000000000000000000000000000000000000000000000000000000000000000000000000000
00000000000000000000000000000000000000000000000000000000000000000000000000000000000
00000000000000000000000000000000000000000000000000000000000000000000000000000000000
00000000000011
>Q5E0H6_3kv1_62_325
FHPVYSVQLEQKLVEKFNLKRALISLDQPNTNEQRKQVAALVSSYLNNNLQEGMAVAVGQ
GQNVAAVADHAGIVTQRNARFVSAIGGTHRSGDIINADHICRRLAKKYGGSSETLYAPAYV

NDPSLRSAFMEHATIKETLSQARKAEFALVGIGDMDENSHMVKLGFFTPKEFVEARLNDGI
VGDIGGFDFFKLDGTDADTLMRGRVIGLEMEDLRQIPNVVAMASESRKALSIMGALRTGVI
DVLATSVSCAMALLNLAENEE
1111100000000000000000000000000000000000000000000000000000000000000000000000000000
0000001111000000000000000000000000000000000000000000000000000000000000000000000000
0000000000000000000000000000000000000000000000000000000000000000000000000000000000
000000000000011
>Q9KH06_2fxq_0_263
MARGLNQVFLIGTLTARPDMRYTPGGLAILDLNLAGQDAFTDESGQEREVPWYHRVRLLG
RQAEMWGDLLEKGQLIFVEGRLEYRQWEKDGEKKSEVQVRAEFIDPLEGRGRETLEDARG
QPRLRRALNQVILMGNLTRDPDLRYTPQGTAVVRLGLAVNERRRGQEEERTHFLEVQAWR
ELAEWASELRKGDGLLVIGRLVNDSWTSSSGERRFQTRVEALRLERPTRGPAQAGGSRPPTV
QTGGVDIDEGLEDFPPEEDLPF
1100000000000000000000000000000000000000000000000000000000000000000000000000000000
0011111111100000000000000001111111111111100000000000000000000000000000000000001111
1110000000000000000000000000000000000000000000000000000000000011111111111111111111
111111111111111
>F0QHB3_4e6t_1_261
SNHDLIHSTAIIDPSAVIASDVQIGPYCIIGPQVTIGAGTKLHSHVVVGGFTRIGQNNEIFQFA
SVGEVCQDLKYKGEETWLEIGNNNLIREHCSLHRGTVQDNALTKIGSHNLLMVNTHIAHD
CIVGDHNIFANNVGVAGHVHIGDHVIVGGNSGIHQFCKIDSYSMIGGASLILKDVPAYVMA
SGNPAHAFGINIEGMRRKGWSKNTIQGLREAYKLIFKSGLTSVQAIDQIKSEILPSVPEAQLLI
DSLEQSERGIVR
1110000000000000000000000000000000000000000000000000000000000000000000000000000000
0000000000000000000000000000000000000000000000000000000000000000000000000000000000
0000000000000000000000000000000000000000000000000000000000000000000000000000000000
000000000000
>Q2YLA2_3ngf_0_260
MPRFAANLSTMFNEVPFLERFRLAAEAGFGGVEFLFPYDFDADVIARELKQHNLTQVLFN
MPPGDWAAGERGMAAISGREQEFRDNVDIALHYALALDCRTLHAMSGITEGLDRKACEE
TFIENFRYAADKLAPHGITVLVEPLNTRNMPGYFIVHQLEAVGLVKRVNRPNVAVQLDLYH
AQIMDGDLTRLIEKMNGAFSHVQIASVPDRHEPDEGELNYPYLFSVLESVGYRGWVGCEY
NPRGKTESGLAWFAPYRDQSA
0000000000000000000000000000000000000000000000000000000000000000000000000000000000
0000000000000000000000000000000000000000000000000000000000000000000000000000000000
0000000000000000000000000000000000000000000000000000000000000000000000000000000000
000000000111
>Q7N439_3gqj_52_312
KNSINTIKLIDDIIALHNDPKGNKLLWNDNWQDKIINRDLANIFEKIDESVSELGGLEMYQE
MVGVNPYDPTEPVCGLSAQNIFKLMTEGEHAVDPVEMAQTGKIDGNEFAESVDQLSSAKN
YVALVNDRRLGHMFLIDIPSNDQETVGYIYQSDLGQGALPPLKIADWLNSRGKDAVSLNKL
KKLLSREFNLLSDDEKRALISETLDIHKDVSNVELDRIKRDRGVDIYLTEYDVNNFYENIETL
KSKLSNYDKKLSKPK
1100000000000000000000000000000000000000000000000000000000000000000000000000000000
0000000000000000000000000000000000000000000000000000000000000000000000000000000000
0000000000000000000000000000000000000000000000000000000000000000000000000000000000
011111111111
>Q15233_3sde_52_311
EGLTIDLKNFRKPGEKTFTQRSRLFVGNLPPDITEEEMRKLFEKYGKAGEVFIHKDKGFGFIR
LETRTLAEIAKVELDNMPLRGKQLRVRFACHSASLTVRNLPQYVSNELLEEAFSVFGQVER
AVVIVDDRGRPSGKGIVEFSGKPAARKALDRCSEGSFLLTTFPRPVTVEPMDQLDDEEGLPE

KLVIKNQQFHKEREQPPRFAQPGSFEYEYAMRWKALIEMEKQQQDQVDRNIKEAREKLEM
EMEAARHEHQVMLM
11111111111110000000000000000000000000000000000000000000000000000000000000000000000
00000000000000000000000000000000000000000000000000000000000000000000000000000000000
00000000000000000000000000000000000000000000000000000000000000000000000000000000000
00011111111
>Q62902_1gv9_24_283
SQFVGSDGMGGDAAAPGAAGTQAELPHRRFEYKYSFKGPHLVQSDGTVPFWAHAGNAIP
SADQIRIAPSLKSQRGSVWTKTKAAFENWEVEVTFRVTGRGRIGADGLAIWYTENQGLDGP
VFGSADMWNGVGIFFDSFDNDGKKNNPAIVVVGNNGQINYDHQNDGATQALASCQRDF
RNKPYPVRAKITYYQKTLTVMINNGFTPDKNDYEFCAKVENMVIPTQGHFGISAATGGLA
DDHDVLSFLTFQLTEPGKEPPT
11111111111111111111111110000000000000000000000000000000000000000000000000000000000
00000000000000000000000000000000000000000000000000000000011111000000000000000000000
00000000000000000000000000000000000000000000000000000000000000000000000000000000000
00001111111
>Q9KI21_3ty2_0_257
MKKTATPKLRLLLSNDDGVYAKGLAILAKTLADLGEVDVVAPDRNRSGASNSLTLNAPLHI
KNLENGMISVEGTPTDCVHLAITGVLPEMPDMVVAGINAGPNLGDDVWYSGTVAAAME
GRFLGLPALAVSLGGELFRYYETAAKVVYQLIQRIEKDPLPPSTILNINVPDLPYEELKGFEVT
RLGTRHRAEPTIRQIDPRGHPIYWVGAAGPEQDSGPGTDFFAMNHHCVSITPLRVDLTHYE
AFDQLASWVKRLEM
11111110000000000000000000000000000000000000000000000000000000000000000000000000000
00000000000000000000000000000000000000000000000000000000000000000000000000000000000
00000000000000000000000000000000000000000000000000000000000000000000000000000000000
000000000
>D0VWX8_2pvu_0_251
MGGGRSTETSSSSGGDGGATKKKVVVGTDAAFAPFEYMQKGKIVGFDVDLLDAVMKAAG
LDYELKNIGWDPLFASLQSKEVDMGISGITITDERKQSYDFSDPYFEATQVILVKQGSPVKNA
LDLKGKTIGVQNATTGQEAAEKLFGKGPHIKKFETTVVAIMELLNGGVDAVITDNAVANE
YVKNNPNKKLQVIEDPKNFASEYYGMIFPKNSELKAKVDEALKNVINSGKYTEIYKKWFGK
EPKLDRLKQ
11111111111111111000000000000000000000000000000000000000000000000000000000000000000
00000000000000000000000000000000000000000000000000000000000000000000000000000000000
00000000000000000000000000000000000000000000000000000000000000000000000000000000000
000
>P0A794_1m5w_0_242
MAELLLGVNIDHIATLRNARGTAYPDPVQAAFIAEQAGADGITVHLREDRRHITDRDVRIL
RQTLDTRMNLEMAVTEEMLAIAVETKPHFCCLVPEKRQEVTTEGGLDVAGQRDKMRDAC
KRLADAGIQVSLFIDADEEQIKAAAEVGAPFIEIHTGCYADAKTDAEQAQELARIAKAATF
AASLGLKVNAGHGLTYHNVKAIAAIPEMHELNIGHAIIGRAVMTGLKDAVAEMKRLMLE
ARG
10000000000000000000000000000000000000000000000000000000000000000000000000000000000
00000000000000000000000000000000000000000000000000000000000000000000000000000000000
00000000000000000000000000000000000000000000000000000000000000000000000000000
>Q6NIF5_2npn_0_249
MRTIYVIGIGTGSPEFLTLQAISGLRHAQAIVALDKGEQKSDLLALRQKIVDTHAPGTPIYAV
TDPERDRNPDNYEEEVRRWHAERAHLLASTIRERTPDDGAVAFLVWGDPSLYDSTLRIIEH
MRNLEDLHADVKVIPGITAVQVLTAEHGILINRIGEAIHITTGRNLPETSAKDRRNCVVMLD
GKTAWQDVATEHTYMWWGAFLGTEQQVLRKGYVHEIGAQVAELKQQLRTEHGWIMDTY
LLRELD

00000000000000000000000000000000000111000000000000000000000000000111111111100000000
00000000000000000000000000000000000000000000000000000000000000000000000000000000000
00000000000000000000000000000000000000000000000000000000000000000000000000000000000
0
>O58714_1v6t_0_254
MRVDLNSDLGESFGRYKLGLDEEVMKYITSANVACGWHAGDPLVMRKTVRLAKENDVQ
VGAHPGYPDLMGFGRRYMKLTPEEARNYILYQVGALYAFAKAEGLELQHVKPHGALYNA
MVKEEDLARAVIEGILDFDKDLILVTLSNSRVADIAEEMGLKVAHEVFADRAYNPDGTLVPR
GRPGAVIEDKEEIAERVISMVKDGGIRAINGEWVDLKVDTICVHGDNPKAVEITSYIRKVLEE
EGVKIVPMKEFIR
00000000000000000000000000000000000000000000000000000000000000000000000000000000000
00000000000000000000000000000000000000000000000000000000000000000000000000000000000
00000000000001111100000000000000000000000000000000000000000000000000000000000000000
000001
>Q92R45_2q62_0_240
MSDDDSSHDLPAANLQQLRLPDSASLRPAFSTHRPRILILYGSLRTVSYSRLLAEEARRLLEFF
GAEVKVFDPSGLPLPDAAPVSHPKVQELRELSIWSEGQVWVSPERHGAMTGIMKAQIDWI
PLSTGSIRPTQGKTLAVMQVSGGSQSFNAVNQMRILGRWMRMITIPNQSSVAKAFQEFDAN
GRMKPSSYYDRVVDVMEELVKFTLLTRDCSAYLTDRYSERKESAAELEHRVTLKSV
11111110000000000000000000000000000000000000000000000000000000000000000000000000000
00000000000000000000000000000000000000000000000000000000000000000000000000000000000
000000000000000000000000000000000000000000000000000000000000000111111111111
>P26093_3sf0_21_273
GSHQMKSEGHANMQLQQQAVLGLNWMQDSGEYKALAYQAYNAAKVAFDHAKVAKGK
KKAVVADLDETMLDNSPYAGWQVQNNKPFDGKDWTRWVDARQSRAVPGAVEFNNYVN
SHNGKVFYVTNRKDSTEKSGTIDDMKRLGFNGVEESAFYLKKDKSAKAARFAEIEKQGYEI
VLYVGDNLDDFGNTVYGKLNADRRAFVDQNQGKFGKTFIMLPNANYGGWEGGLAEGYF
KKDTQGQIKARLDAVQAWDGK
11111110000000000000000000000000000000000000000000000000000000000000000000000000000
00000000000000000000000000000000000000000000000000000000000000000000000000000000000
00000000000000000000000000000000000000000000000000000000000000000000000000000000000
0000
>P35398_1n83_303_555
AELEHLAQNISKSHLETCQYLREELQQITWQTFLQEEIENYQNKQREVMWQLCAIKITEAIQ
YVVEFAKRIDGFMELCQNDQIVLLKAGSLEVVFIRMCRAFDSQNNTVYFDGKYASPDVFKS
LGCEDFISFVFEFGKSLCSMHLTEDEIALFSAFVLMSADRSWLQEKVKIEKLQQKIQLALQH
VLQKNHREDGILTKLICKVSTLRALCGRHTEKLMAFKAIYPDIVRLHFPPLYKELFTSEFEPA
MQIDG
00000000000000000000000000000000000000000000000000000000000000000000000000000000000
00000000000000000000000000000000000000000000000000000000000000000000000000000000000
00000000000000000000000000000000000000000000000000000000000000000000000000011111111
1111
>P36438_1hcz_35_286
YPIFAQQNYENPREATGRIVCANCHLASKPVDIEVPQAVLPDTVFEAVVKIPYDMQLKQVL
ANGKKGALNVGAVLILPEGFELAPPDRISPEMKEKIGNLSFQNYRPNKKNILVIGPVPGQKY
SEITFPILAPDPATNKDVHFLKYPIYVGGNRGRGQIYPDGSKSNNTVYNATAGGIISKILRKE
KGGYEITIVDASNERQVIDIIPRGLELLVSEGESIKLDQPLTSNPNVGGFGQGDAEIVLQDPLR
VQ
00000000000000000000000000000000000000000000000000000000000000000000000000000000000
00000000000000000000000000000000000000000000000000000000000000000000000000000000000
00000000000000000000000000000000000000000000000000000000000000000000000000000000000

011
>P11562_3pot_0_248
MAQYYPGTTKVAQNRRNFCNPEYELEKLREISDEDVVKILGHRAPGEEYPSVHPPLEEMDE
PEDAIREMVEPIDGAKAGDRVRYIQFTDSMYFAPAQPYVRSRAYLCRYRGADAGTLSGRQII
ETRERDLEKISKELLETEFFDPARSGVRGKSVHGHSLRLDEDGMMFDMLRRQIYNKDTGRV
EMVKNQIGDELDEPVDLGEPLDEETLMEKTTIYRVDGEAYRDDVEAVEIMQRIHVLRSQGG
FNLE
10000000000000000000000000000000000000000000000000000000000000000000000000000000000
00000000000000000000000000000000000000000000000000000000000000000000000000000000000
00000000000000000000000000000000000000000000000000000000000000000000000000000000011
>Q7Z9M7_2vtc_0_248
MKSCAILAALGCLAGSVLGHGQVQNFTINGQYNQGFILDYYYQKQNTGHFPNVAGWYAE
DLDLGFISPDQYTTPDIVCHKNAAPGAISATAAAGSNIVFQWGPGVWPHPYGPIVTYVVEC
SGSCTTVNKNNLRWVKIQEAGINYNTQVWAQQDLINQGNKWTVKIPSSLRPGNYVFRHE
LLAAHGASSANGMQNYPQCVNIAVTGSGTKALPAGTPATQLYKPTDPGILFNPYTTITSYTI
PGPALWQG
11111111111111111110000000000000000000000000000000000000000000000000000000000000000
00000000000000000000000000000000000000000000000000000000000000000000000000000000000
00000000000000000000000000000000000000000000000000000000000000000000000000000000011
>P28861_1fdr_0_247
MADWVTGKVTKVQNWTDALFSLTVHAPVLPFTAGQFTKLGLEIDGERVQRAYSYVNSPD
NPDLEFYLVTVPDGKLSPRLAALKPGDEVQVVSEAAGFFVLDEVPHCETLWMLATGTAIGP
YLSILQLGKDLDRFKNLVLVHAARYAADLSYLPLMQELEKRYEGKLRIQTVVSRETAAGSLT
GRIPALIESGELESTIGLPMNKETSHVMLCGNPQMVRDTQQLLKETRQMTKHLRRRPGHM
TAEHYW
10000000000000000000000000000000000000011100000000000000000000000000000000000000000
00000000000000000000000000000000000000000000000000000000000000000000000000000000000
0000000000000000000000000000000000000000000000000000000000000000000000000000000000
>Q6DWK3_2iw0_0_247
MHFSTLFGAAATAALAGSTNASPLARRQVPVGTPILQCTQPGLVALTYDDGPFTFTPQLLDI
LKQNDVRATFFVNGNNWANIEAGSNPDTIRRMRADGHLVGSHTYAHPDLNTLSSADRISQ
MRHVEEATRRIDGFAPKYMRAPYLSCDAGCQGDLGGLGYHIIDTNLDTKDYENNKPETTH
LSAEKFNNELSADVGANSYIVLSHDVHEQTVVSLTQKLIDTLKSKGYRAVTVGECLGDAPE
NWYKA
11111111111111111111111111110000000000000000000000000000000000000000000000000000000
00000000000000000000000000000000000000000000000000000000000000000000000000000000000
0000000000000000000000000000000000000000000000000000000000000000000000000000000000
>O15247_2r4v_0_246
MSGLRPGTQVDPEIELFVKAGSDGESIGNCPFCQRLFMILWLKGVKFNVTTVDMTRKPEEL
KDLAPGTNPPFLVYNKELKTDFIKIEEFLEQTLAPPRYPHLSPKYKESFDVGCNLFAKFSAYIK
NTQKEANKNFEKSLLKEFKRLDDYLNTPLLDEIDPDSAEEPPVSRRLFLDGDQLTLADCSLL
PKLNIIKVAAKKYRDFDIPAEFSGVWRYLHNAYAREEFTHTCPEDKEIENTYANVAKQKS
11111111100000000000000000000000000000000001111111110000000000000000000000000000000
00000000000000000000000000000000000000000000000000000000000000000000000000000000000
00000000000000000000000000000000000000000000000000000000000000000000000000000000011
>P75960_1s5p_2_236
KPRVLVLTGAGISAESGIRTFRAADGLWEEHRVEDVATPEGFDRDPELVQAFYNARRRQLQ
QPEIQPNAAHLALAKLQDALGDRFLLVTQNIDNLHERAGNTNVIHMHGELLKVRCSQSG
QVLDWTGDVTPEDKCHCCQFPAPLRPHVVWFGEMPLGMDEIYMALSMADIFIAIGTSGH
VYPAAGFVHEAKLHGAHTVELNLEPSQVGNEFAEKYYGPASQVVPEFVEKLLKGLK
00000000000000000000000000000000000000000000000000000000000000000000000000000000000

0000000000000000000000000000111111000000000000000000000000000000000000000000000000000
000000000000000000000000000000000000000111000000000000000000000000000
>P50597_1r6l_0_238
MNRPSGRAADQLRPIRITRHYTKHAEGSVLVEFGDTKVICTVSAESGVPRFLKGQGQGWLT
AEYGMLPRSTGERNQREASRGKQGGRTLEIQRLIGRSLRAALDLSKLGENTLYIDCDVIQAD
GGTRTASITGATVALIDALAVLKKRGALKGNPLKQMVAAVSVGIYQGVPVLDLDYLEDSAA
ETDLNVVMTDAGGFIEVQGTAEGAPFRPAELNAMLELAQQGMQELFELQRAALAE
0000000000000000000000000000000000000000111000000000000000000000000000000000000000000
00000000000000000000000000000000000000000000000000000000000000000000000000000000000
0000000000000000000000000000000000000000000000000000000000000000000000000
>F8DUF3_3nol_39_282
SHAATPSIPIYDYQIVHSYPHDTKAFTEGFFYRNGYFYESTGLNGRSSIRKVDIESGKTLQQIE
LGKRYFGEGISDWKDKIVGLTWKNGLGFVWNIRNLRQVRSFNYDGEGWGLTHNDQYLIM
SDGTPVLRFLDPESLTPVRTITVTAHGEELPELNELEWVDGEIFANVWQTNKIVRIDPETGKV
TGIIDLNGILAEAGPLPSPIDVLNGIAWDKEHHRLFVTGKLWPKVFEITLTQRVGGHK
11111100000000000000000000000000000000000000000000000000000000000000000000000000000
00000000000000000000000000000000000000000000000000000000000000000000000000000000000
000000000000000000000000000000000000000000000000000000000000000000000000001111
>Q8ZJZ9_3s81_0_243
MKHTIGILGGMGPAATADMLEKFVELRHASCDQQHIPLIVSSIPDIPDRTACLLSGGPSPYRY
LERYLHMLEDAGAECIVIPCNTAHYWFDDLQNVAKARMISILDATLGDIPPSARHVGLLAT
NATLATGLYQKKALARGLTLIQPEDAGQALVMQAIYTLKRGDKTAAQALLLPQIDSLIARG
AQAIIMGCTEIPLIVAGHERAIACPMIDSTASLVRAAIRWYESWPDTRASLTGEQRLTA
00000000000000000000000000000000000000000000000000000000000000000000000000000000000
00000000000000000000000000000000000000000000000000000000000000000000000000000000000
000000000000000000000000000000000000000000000000000000000000000000011111111111
>Q8Y4T3_4gvo_28_268
AANQKVQTITVGTGTQFPNVCFLDENGKLTGYDVELVKEIDKRLPGYKFKFKTMDFSNLLV
SLGAGKVDIVAHQMEKSKEREKKFLFNDVAYNNFPLQLTVLDSNNSINSTKDLAGKRVITS
ATSNGALVLKKINEEQGNNFEIAYEGQGSNDTANQLKTGRADATISTPFAVDFQNKTSAIKE
KVVGDVLSNAKVYFMLGKDETKLSKKVDEALQSIIDDGTLKKLSEKWLGADYSKEQY
11111000000000000000000000000000000000000000000000000000000000000000000000000000000
00000000000000000000000000000000000000000000000000000000000000000000000000000000000
000000000000000000000000000000000000000000000000000000000000000000000000000
>Q9H3S4_3s4y_14_242
GNLKYCLVILNQPLDNYFRHLWNKALLRACADGGANRLYDITEGERESFLPEFINGDFDSIR
PEVREYYATKGCELISTPDQDHTDFTKCLKMLQKKIEEKDLKVDVIVTLGGLAGRFDQIMAS
VNTLFQATHITPFPIIIIQEESLIYLLQPGKHRLHVDTGMEGDWCGLIPVGQPCMQVTTTGLK
WNLTNDVLAFGTLVSTSNTYDGSGVVTVETDHPLLWTMAIKS
10000000000000000000000000000000000000000000000000000000000000000000000000000000000
00000000000000000000000000000000000000000000000000000000000000000000000000000111000
000000000000000000000000000000000000000000000000000000000000001
>Q9X1X6_1j5p_0_240
MTVLIIGMGNIGKKLVELGNFEKIYAYDRISKDIPGVVRLDEFQVPSDVSTVVECASPEAVKE
YSLQILKNPVNYIIISTSAFADEVFRERFFSELKNSPARVFFPSGAIGGLDVLSSIKDFVKNVRIE
TIKPPKSLGLDLKGKTVVFEGSVEEASKLFPRNINVASTIGLIVGFEKVKVTIVADPAMDHNI
HIVRISSAIGNYEFKIENIPSPENPKTSMLTVYSILRTLRNLESKIIFG
00000000000000000000000000000000000000000000000000000000000000000000000000000000000
00000000000000000000000000000000000000000000000000000000000000000000000000000000000
000000000000000000000000000000000000000000000111111110000000000000000000000
>Q81MQ2_3r8y_0_239

MKMMDANEIISFIQKSEKKTPVKVYIKGDLKEVTFPETVQAFVNKKSGVLFGEWSEIKTILD
ENSKYIVDYVVENDRRNSAIPMLDLKGIKARIEPGAIIRDHVEIGDNAVIMMNATINIGAVIG
EGSMIDMNAVLGGRATVGKNCHVGAGAVLAGVIEPPSAKPVIVEDDVVIGANVVVLEGVT
VGKGAVVAAGAVVTEDVPPYTVVAGTPARVIKEIDEKTKAKTEIKQELRQLNPEK
11111111111111110000000000000000000000000000000000000000000000000000000000000000000
00000000000000000000000000000000000000000000000000000000000000000000000000000000000
0000000000000000000000000000000000000000000000000001111111111111111111111
>Q64UP6_2p1g_20_257
QESSLVLSNGLGFVDTPYKAGTLEVDDTEDLIINCDEVDCTTFVEYALAMALCPQQGDEMQ
EGDFARNLQRIRYRDGKIDGYTSRLHYISDWINNAVRQGLLEDVTAAYSPFKQKLSLSYMST
HPELYKSLKNSPENVAQMAKYEKALSGKEVHYLPKDKLEPDGLPWIKNGDIIALTTNTPGL
DVSHMGIAIYIKGQLHLLHASSKEGKVVVGKTALSQMLKDRKSLTGIRVLRMKK
11111000000000000000000000000000000000000000000000000000000000000000000000000000000
00000000000000000000000000000000000000000000000000000000000000000000000000000000000
00000000000000000000000000000000000000000000000000000000000000000000011
>A1Z1Q3_4iqy_6_242
KKKVWREEKERLLKMTLEERRKEYLRDYIPLNSILSWKEEMKGKGQNDEENTQETSQVKKS
LTEKVSLYRGDITLLEVDAIVNAANASLLGGGGVDGCIHRAAGPCLLAECRNLNGCDTGH
AKITCGYDLPAKYVIHTVGPIARGHINGSHKEDLANCYKSSLKLVKENNIRSVAFPCISTGIY
GFPNEPAAVIALNTIKEWLAKNHHEVDRIIFCVFLEVDFKIYKKKMNEFFSVD
11100000000000000000000000000000000000000001111110000000000000000000000000000000000
00000000000000000000000000000000000000000000000000000000000000000000000000000000000
00000000000000000000000000000000000000000000000000000000000000000011
>P81460_4i30_0_236
ADTIVAVELDTYPNTDIGDPSYPHIGIDIKSVRSKKTAKWNMQNGKVGTAHIIYNSVGKRLS
AVVSYPNGDSATVSYDVDLDNVLPEWVRVGLSASTGLYKETNTILSWSFTSKLKSNSTHETN
ALHFVFNQFSKDQKDLILQGDATTGTDGNLELTRVSSNGSPQGNSVGRALFYAPVHIWESS
AVVASFDATFTFLIKSSDSHPADGIAFFISNIDSSIPSGSTGRLLGLFPDAN
00000000000000000000000000000000000000000000000000000000000000000000000000000000000
00000000000000000000000000000000001111100000000000000000000000000000000000000000000
0000000000000000000000000000000000000000000000000000000000000000000000
>Q04432_1qvw_0_236
MAPKKVLLALTSYNDVFYSDGAKTGVFVVEALHPFNTFRKEGFEVDFVSETGKFGWDEHS
LAKDFLNGQDETDFKNKDSDFNKTLAKIKTPKEVNADDYQIFFASAGHGTLFDYPKAKDL
QDIASEIYANGGVVAAVCHGPAIFDGLTDKKTGRPLIEGKSITGFTDVGETILGVDSILKAKN
LATVEDVAKKYGAKYLAPVGPWDDYSITDGRLVTGVNPASAHSTAVRSIDALKN
10000000000000000000000000000000000000000000000000000000000000000000000000000000000
00000000000000000000000000000000000000000000000000000000000000000000000000000000000
0000000000000000000000000000000000000000000000000000000000000000000000
>Q5VWZ2_3u0v_0_236
MAAASGSVLQRCIVSPAGRHSASLIFLHGSGDSGQGLRMWIKQVLNQDLTFQHIKIIYPTAP
PRSYTPMKGGISNVWFDRFKITNDCPEHLESIDVMCQVLTDLIDEEVKSGIKKNRILIGGFSM
GGCMAIHLAYRNHQDVAGVFALSSFLNKASAVYQALQKSNGVLPELFQCHGTADELVLHS
WAEETNSMLKSLGVTTKFHSFPNVYHELSKTELDILKLWILTKLPGEMEKQK
11111111000000000000000000000000000000000000000000000000000000000000000000000000000
00000000000000000000000000000000000000000000000000000000000000000000000000000000000
0000000000000000000000000000000000000000000000000000000000000001111111
>Q6YI95_3c3y_0_236
MDFAVMKQVKNTGLLQSEELCQYILRTSVYPREAGFLKELREANESHPDSYMSTSPLAGQL
MSFVLKLVNAKKTIEVGVFTGYSLLLTALSIPDDGKITAIDFDREAYEIGLPFIRKAGVEHKIN
FIESDAMLALDNLLQGQESEGSYDFGFVDADKPNYIKYHERLMKLVKVGGIVAYDNTLWG

GTVAQPESEVPDFMKENREAVIELNKLLAADPRIEIVHLPLGDGITFCRRLY
11111111111100000000000000000000000000000000000000000000000000000000000000000000000
00000000000000000000000000000000000000000000000000000000000000000000000000000000000
00000000000000000000000000000000000000000000000000000000000000000000000
>Q8DIJ1_1z3x_0_234
MVTTEPALADLQEQLYNGNEKSQLAAMSTLSTAGTEGYHLLQEFLKDSATFSPPPAPWIRG
QAYRLLFHSPEASVQAFLQQHYPQGVIPLRSDRGVDYQELAKLLVAEKFEAADRLTTQKLC
ELAGPLAQKRRWLYFTEVEQLPIPDLQTIDQLWLAFSLGRFGYSVQRQLWLGCGQNWDRL
WEKIGWRQGKRWPRYPNEFIWDLSAPRGHLPLTNQLRGVQVLNALLNHPAWTA
00011000000000000000000000000000000000000000000000000000000000000000000000000000000
00000000000000000000000000000000000000000000000000000000000000000000000000000000000
000000000000000000000000000000000000000000000000000000000000000000000
>A0R6D0_2wzv_0_233
MSVPTLPTGPTVDLAQAAERLIKGRRAVRAFRPDEVPEETMRAVFELAGHAPSNSNTQPW
HVEVVSGAARDRLAEALVTAHAEERVTVDFPYREGLFQGVLQERRADFGSRLYAALGIARD
QTDLLQGYNTESLRFYGAPHVAMLFAPNNTEARIAGDMGIYAQTLMLAMTAHGIASCPQ
ALLSFYADTVRAELGVENRKLLMGISFGYADDTAAVNGVRIPRAGLSETTRFSR
11111111111000000000000000000000000000000000000000000000000000000000000000000000000
00000000000000000000000000000000000000000000000000000000000000000000000000000000000
00000000000000000000000000000000000000000000000000000000000000000000
>Q8DZA6_3lec_0_226
MDLQLSKRLQKVANYVPKGARLLDVGSDHAYLPIFLLQMGYCDFAIAGEVVNGPYQSALK
NVSEHGLTSKIDVRLANGLSAFEEADNIDTITICGMGGRLIADILNNDIDKLQHVKTLVLQP
NNREDDLRKWLAANDFEIVAEDILTENDKRYEILVVKHGHMNLTAKELRFGPFLLSNNTTV
FKEKWQNELNKLTFALNSIPNSKMEERAILEDKIQDIKEVLDES
00000000000000000000000000000000000000000000000000000000000000000000000000000000000
00000000000000000000000000000000000000000000000000000000000000000110000000000000000
0000000000000000000000000000000000000000000000000000000000011
>P42624_2vec_1_232
ITTRTARQCGQADYGWLQARYTFSFGHYFDPKLLGYASLRVLNQEVLAPGAAFQPRTYPK
VDILNVILDGEAEYRDSEGNHVQASAGEALLLSTQPGVSYSEHNLSKDKPLTRMQLWLDA
CPQRENPLIQKLALNMGKQQLIASPEGAMGSLQLRQQVWLHHIVLDKGESANFQLHGPR
AYLQSIHGKFHALTHHEEKAALTCGDGAFIRDEANITLVADSPLRALLIDLPV
11111000000000000000000000000000000000000000000000000000000000000000000000000000000
00000000000000000000000000000000000000000000000000000000000000000000000000000000000
000000000000000000000000000000000000000000000000000000000000000000
>P08038_2vnd_0_230
MMIFRFVTTLAASLPLLTFAAPISFSHAKNEAVKIYRDHPVSFYCGCEIRWQGKKGIPDLESC
GYQVRKNENRASRIEWEHVVPAWQFGHQLQCWQQGGRKNCTRTSPEFNQMEADLHNL
TPAIGEVNGDRSNFSFSQWNGVDGVTYGQCEMQVNFKERTAMPPERARGAIARTYLYMSE
QYGLRLSKAQSQLMQAWNNQYPVSEWECVRDQRIEKVQGNSNRFVREQCPN
11111111111111111111111100000000000000000000000000000000000000000000000000000000000
00000000000000000000000000000000000000000000000000000000000000000000000000000000000
00000000000000000000000000000000000000000000000000000000000000000
>Q9NRG1_2jbh_0_224
MAGSSEEAPDYGRGVVIMDDWPGYDLNLFTYPQHYYGDLEYVLIPHGIIVDRIERLAKDIM
KDIGYSDIMVLCVLKGGYKFCADLVEHLKNISRNSDRFVSMKVDFIRLKSYRNDQSMGEM
QIIGGDDLSTLAGKNVLIVEDVVGTGRTMKALLSNIEKYKPNMIKVASLLVKRTSRSDGFRP
DYAGFEIPNLFVVGYALDYNEYFRDLNHICVINEHGKEKYRV
11111100000000000000000000000000000000000000000000000000000000000000000000000000000
00000000000000000000000000000000000000000000000000000000000000000000000000000000000

0000000001000000000000000000000000000000000000000000000000
>Q75WP3_2zac_80_307
PAAAPGKNFDLSHWKLQLPDANTTEISSANLGLYTSQYFYTDTDGAMTFWAPTTGGTTA
NSSYPRSELREMLDPSNSKVNWGWQGTHTMKLSGKTVQLPSSGKIIVAQIHGIMDDGTNA
PPLVKAVFQDGQLDMQVKQNSDGTGSDVHNYFTGIKLGDLYNMEIRVTDGVAYVTMNGD
TRSVDFVGKDAGWKNLKYYFKAGNYVQDNTSTGGSAIAKLYSLSVSHSN
00000000000000000000000000000000000000000000000000000000000000000000000000000000000
00000000000000000000000000000000000000000000000000000000000000000000000000000000000
00000000000000000000000000000000000000000000000000000000000001
>Q9UHI6_2oxc_40_267
RTAQDLSSPRTRTGDVLLAEPADFESLLLSRPVLEGLRAAGFERPSPVQLKAIPLGRCGLDLI
VQAKSGTGKTCVFSTIALDSLVLENLSTQILILAPTREIAVQIHSVITAIGIKMEGLECHVFIGG
TPLSQDKTRLKKCHIAVGSPGRIKQLIELDYLNPGSIRLFILDEADKLLEEGSFQEQINWIYSSL
PASKQMLAVSATYPEFLANALTKYMRDPTFVRLNS
11111111111111111111100000000000000000000000000000000000000000000000000000000000000
00000000000000000000000000000000000000000000000000000000000000000000000000000000000
00000000000000000000000000000000000000000000000000000000000011
>P0A6I0_2fem_0_226
MTAIAPVITIDGPSGAGKGTLCKAMAEALQWHLLDSGAIYRVLALAALHHHVDVASEDAL
VPLASHLDVRFVSTNGNLEVILEGEDVSGEIRTQEVANAASQVAAFPRVREALLRRQRAFRE
LPGLIADGRDMGTVVFPDAPVKIFLDASSEERAHRRMLQLQEKGFSVNFERLLAEIKERDD
RDRNRAVAPLVPAADALVLDSTTLSIEQVIEKALQYARQKLALA
11000000000000000000000000000000000000000000000000000000000000000000000000000000000
00000000000000000000000000000000000000000000000000000000000000000000000000000000000
0000000000000111111111111100000000000000000000000000000000000
>Q13907_2dho_0_226
MPEINTNHLDKQQVQLLAEMCILIDENDNKIGAETKKNCHLNENIEKGLLHRAFSVFLFN
TENKLLLQQRSDAKITFPGCFTNTCCSHPLSNPAELEESDALGVRRAAQRRLKAELGIPLEE
VPPEEINYLTRIHYKAQSDGIWGEHEIDYILLVRKNVTLNPDPNEIKSYCYVSKEELKELLKK
AASGEIKITPWFKIIAATFLFKWWDNLNHLNQFVDHEKIYRM
11111111111100000000000000000000000000000000000000000000000000000000000000000000000
00000000000000000000000000000000000000000000000000000000000000000000000000000000000
0000000000000000000000000000000000000000000000000000000000000
>Q5NHL5_3mjd_0_207
MFIEFALKNQVLKFGEFTLKSGRISPYFFNAGLFNTGAQLATLADYYAQLIIKSDVKYDILFG
PAYKGIPLVAAISTVLALKYNIDMPYAFDRKEAKDHGEGGVFVGADMTNKKVLLIDDVMT
AGTAFYESYNKLKIINAKIAGVVLSIDRQEKAKDSDISATKKISQDFNIPVLAVTNFESIFEYV
KENLDETMIDKFKQYRQKYGS
00000000000000000000000000000000000000000000000000000000000000000000000000000000000
00000000000011111110000000000000000000000000000000000000000000000000000000000000000
000000000000000000000000000000000000000000
>A1Z6E0_2fnj_110_334
RELQADFVKPARIDILLDMPPASRDLQLKHSWNSEDRSLNIFVKEDDKLTFHRHPVAQSTD
CIRGKVGLTKGLHIWEIYWPTRQRGTHAVVGVCTADAPLHSVGYQSLVGSTEQSWGWDLG
RNKLYHDSKNCAGVTYPAILKNDEAFLVPDKFLVALDMDEGTLSFIVDQQYLGIAFRGLRG
KKLYPIVSAVWGHCEITMRYIGGLDPEPLPLMDLCRRTIRQKI
11111100000000000000000000000000000000000000000000000000000000000000000000000000000
00000000000000000000000000000000000000000000000000000000000000000000000000000000000
00000000000000000000000000000000000000000000000000000000011
>A9CIK7_2i3d_0_224
MPEVIFNGPAGRLEGRYQPSKEKSAPIAIILHPHPQFGGTMNNQIVYQLFYLFQKRGFTTLRF

NFRSIGRSQGEFDHGAGELSDAASALDWVQSLHPDSKSCWVAGYSFGAWIGMQLLMRRP
EIEGFMSIAPQPNTYDFSFLAPCPSSGLIINGDADKVAPEKDVNGLVEKLKTQKGILITHRTLP
GANHFFNGKVDELMGECEDYLDRRLNGELVPEPAAKRIR
00000000000000000000000000000000000000000000000000000000000000000000000000000000000
00000000000000000000000000000000000000000000000000000000000000000000000000000000000
00000000000000000000000000000000000000000000000000000011111
>A6T8J6_3tfw_0_223
MGKRHMQQQWSAVDNYLIKALIPGDPVLDRVLENNHRAGLPAHDVAANQGQFLALLVR
LTQAKRILEIGTLGGYSTIWMARELPADGQLLTLEADAHHAQVARENLQLAGVDQRVTLR
EGPALQSLESLGECPAFDLIFIDADKPNNPHYLRWALRYSRPGTLIIGDNVVRDGEVVNPQS
ADERVQGVRQFIEMMGAEPRLTATALQTVGTKGWDGFTLAWVNA
11110000000000000000000000000000000000000000000000000000000000000000000000000000000
00000000000000000000000000000000000000000000000000000000000000000000000000000000000
0000000000000000000000000000000000000000000000000000000000
>Q8A5V9_3qu2_0_223
MRKKLKAVLFDMDGVLFNSMPYHSEAWHQVMKTHGLDLSREEAYMHEGRTGASTINIVF
QRELGKEATQEEIESIYHEKSILFNSYPEAERMPGAWELLQKVKSEGLTPMVVTGSGQLSLLE
RLEHNFPGMFHKELMVTAFDVKYGKPNPEPYLMALKKGGLKADEAVVIENAPLGVEAGH
KAGIFTIAVNTGPLDGQVLLDAGADLLFPSMQTLCDSWDTIML
11100000000000000000000000000000000000000000000000000000000000000000000000000000000
00000000000000000000000000000000000000000000000000000000000000000000000000000000000
0000000000000000000000000000000000000000000000000000000000
>P70202_1wnh_0_221
MEIPPTHYAASRAASVAENCINYQQGTPHKLFLVQTVQQASKEDIPGRGHKYHLKFSVEEII
QKQVTVNCTAEVLYPQMGQGSAPEVNFTFEGEIGKNPDEEDNTFYQSLMSLKRPLEAQDIP
DNFGNVSPQMKPVQHLAWVACGYVMWQNSTEDTWYKMLKIQTVKQVQRNDDFIELDY
TILLHDIASQEIIPWQMQVLWHPQYGTKVKHNSRLPKEGQAE
00000000000000000000000000000000000000000000000000000000000000000000000000000000000
00000000000000000000000000000000000000000000000000000000000000000000000000000000000
00000000000000000000000000000000000000000000000000011111
>Q51793_1ty9_0_221
MNGSIQGKPLLGKGMSESLTGTLDAPFPEYQTLPADPMSVLHNWLERARRVGIREPRALAL
ATADSQGRPSTRIVVISEISDAGVVFSTHAGSQKGRELLHNPWASGVLYWRETSQQIILNGQ
AVRLPNAKADDAWLKRPYATHPMSSVSRQSEELQDVQAMRNAARQLAELQGPLPRPEGY
CVFELRLESLEFWGNGQERLHERLRYDRSDTGWNVRRLQP
11111111111111100000000000000000000000000000000000000000000000000000000000000000000
00000000000000000000000000000000000000000000000000000000000000000000000000000000000
00000000000000000000000000000000000000000000000000000000
>P26926_4ay0_23_257
AQPDIKFASKEYGVTIGESRIIYPLDAAGVMVSVKNTQDYPVLIQSRIYDENKEKESEDPFVV
TPPLFRLDAKQQNSLRIAQAGGVFPRDKESLKWLCVKGIPPKDEDIWVDVQFAINNCIKLL
VRPNELKGTPIQFAENLSWKVDGGKLIAENPSPFYMNIGELTFGGKSIPSHYIPPKSTWAFDL
PKGLAGARNVSWRIINDQGGLDRLYSKNVTL
11111111111100000000000000000000000000000000000000000000000000000000000000000000000
00000000000000000000011111111111110000000000000000000000000000000000000000000000000
0000000000000000000000000000000000000000000000000000
>O75636_2j60_80_298
VNLLRCQEGPRNCRELLSQGATLSGWYHLCLPEGRALPVFCDMDTEGGGWLVFQRRQDG
SVDFFRSWSSYRAGFGNQESEFWLGNENLHQLTLQGNWELRVELEDFNGNRTFAHYATFR
LLGEVDHYQLALGKFSEGTAGDSLSLHSGRPFTTYDADHDSSNSNCAVIVHGAWWYASCY
RSNLNGRYAVSEAAAHKYGIDWASGRGVGHPYRRVRMMLR

11111100000000000000000000000000000000000000000000000000000000000000000000000000000
00000000000000000000000000000000000000000000000000000000000000000000000000000000000
00000000000000000000000000000000000000000000000000000
>P83699_1q0u_1_218
AETQFTRFPFQPFIIEAIKTLRFYKPTEIQERIIPGALRGESVGQSQTGTGKTHAYLLPIEKIKPE
RAEVQAVITAPTRELATQIYHETLKITKFCPKDRIVARCLIGGTDKQKALEKLNVQPHIVIGT
PGRINDFIREQALDVHTAHILVVDEADLLDGFITDVDQIAARPKDLQLVFSATIPEKLKPFLK
KYENPTFVHVLEHHHHHH
00000000000000000000000000000000000000000000000000000000000000000000000000000000000
00000000000000000000000000000000000000000000000000000000000000000000000000000000000
00000000000000000000000000000000000001111111
>P0A744_1ff3_1_211
SLFDKKHLVSPADALPGRNTPMPVATLHAVNGHSMTNVPDGMEIAIFAMGCFWGVERLF
WQLPGVYSTAAGYTGGYTPNPTYREVCSGDTGHAEAVRIVYDPSVISYEQLLQVFWENHD
PAQGMRQGNDHGTQYRSAIYPLTPEQDAAARASLERFQAAMLAADDDRHITTEIANATPF
YYAEDDHQQYLHKNPYGYCGIGGIGVCLPPEA
00000000000000000000000000000000000000000000000000000000000000000000000000000000000
00000000000000000000000000000000000000000000000000000000000000000000000000000000000
000000000000000000000000000011111111111111111
>Q7BGI8_2h0u_0_216
MDREQVVALQHQRFAAKKYDPNRRISQKDWEALVEVGRLAPSSIGLEPWKMLLLKNERM
KEDLKPMAWGALFGLEGASHFVIYLARKGVTYDSDYVKKVMHEVKKRDYDTNSRFAQIIK
NFQENDMKLNSERSLFDWASKQTYIQMANMMMAAAMLGIDSCPIEGYDQEKVEAYLEEK
GYLNTAEFGVSVMACFGYRNQEITPKTRWKTEVIYEVIE
10000000000000000000000000000000000000000000000000000000111111111111111111110000000
00000000000000000000000000000000000000000000000000000000000000000000000000000000000
000000000000000000000000000000000000000000000000000
>P00590_1ffa_16_229
LPTSNPAQELEARQLGRTTRDDLINGNSASCRDVIFIYARGSTETGNLGTLGPSIASNLESAF
GKDGVWIQGVGGAYRATLGDNALPRGTSSAAIREMLGLFQQANTKCPDATLIAGGYSQG
AALAAASIEDLDSAIRDKIAGTVLFGYTKNLQNRGRIPNYPADRTKVFCNTGDLVCTGSLIV
AAPHLAYGPDARGPAPEFLIEKVRAVRGSA
11111111111110000000000000000000000000000000000000000000000000000000000000000000000
00000000000000000000000000000000000000000000000000000000000000000000000000000000000
000000000000000000000000000000000000000000000001
>P77544_3bby_0_213
MSKPAITLWSDAHFFSPYVLSAWVALQEKGLSFHIKTIDLDSGEHLQPTWQGYGQTRRVPL
LQIDDFELSESSAIAEYLEDRFAPPTWERIYPLDLENRARARQIQAWLRSDLMPIREERPTDV
VFAGAKKAPLTAEGKASAEKLFAMAEHLLVLGQPNLFGEWCIADTDLALMINRLVLHGDE
VPERLVDYATFQWQRASVQRFIALSAKQSG
11000000000000000000000000000000000000011111111111111111000000000000000000000000000
00000000000000000000000000000000000000000000000000000000000000000000000000000000000
000000000000000000000000000000000000000000000111
>A5H7H0_3st1_17_229
IAGTVMDHDKIAKLPASGSPLETKFQPQLHIGNGCHSYPAVDAQGNWSGGLKPTGAPSAA
CKDTSKAQTYVRSATFQGKTALVYAWYMPKDEISTGIGHRHDWEGAVVFLNSDTQQIDGV
AASAHGKWRKYPNPGGANIDDTHVKLQYSAEPVINSHALDLTDKGGDLPTLASWEGMG
ADARAAINERSHWGDANPPIADSLIGSSLSGAWMW
10000000000000000000000000000000000000000000000000000000000000000000000000000000000
00000000000000000000000000000000000000000000000000000000000000000000000000000000000
00000000000000000000000000000000000000000000000

>O53169_3pbi_29_240
DAEPGQWDPTLPALVSAGAPGDPLAVANASLQATAQATQTTLDLGRQFLGGLGINLGGPA
ASAPSAATTGASRIPRANARQAVEYVIRRAGSQMGVPYSWGGGSLQGPSKGVDSGANTVG
FDCSGLVRYAFAGVGVLIPRFSGDQYNAGRHVPPAEAKRGDLIFYGPGGGQHVTLYLGNG
QMLEASGSAGKVTVSPVRKAGMTPFVTRIIEY
111100000000000000000000000000000000000000000000000000000000111111110000000000000000
000000000000000000000000000000000000000000000000000000000000000000000000000000000000
0000000000000000000000000000000000000000000000
>Q5T1C6_4ae8_36_239
SSEEVILKDCSVPNPSWNKDLRLLFDQFMKKCEDGSWKRLPSYKRTPTEWIQDFKTHFLDP
KLMKEEQMSQAQLFTRSFDDGLGFEYVMFYNDIEKRMVCLFQGGPYLEGPPGFIHGGAIAT
MIDATVGMCAMMAGGIVMTANLNINYKRPIPLCSVVMINSQLDKVEGRKFFVSCNVQSV
DEKTLYSEATSLFIKLNPAKSLT
111111000000000000000000000000000000000000001111111111111111111111111000000000000000
000000000000000000000000000000000000000000000000000000000000000000000000000000000000
00000000000000000000000000000000111111
>A4XEL3_3c5y_0_211
MKIALIIENSQAAKNAVVHEALTTVAEPLGHKVFNYGMYTAEDKASLTYVMNGLLAGILL
NSGAADFVVTGCGTGMGSMLAANAMPGVFCGLVIDPTDAFLFGQINDGNAISMPYSKGF
GWAAELNLQDVYRKLFDGERGLGYPRERAEIMRKNRGILRELKDASCRDMLTVLKTVDQ
DLLRAAIAGEKFAELFYPNCKDDAIANYLRSLDA
000000000000000000000000000000000000000000000000000000000000000000000000000000000000
000000000000000000000000000000000000000000000000000000000000000000000000000000000000
0000000000000000000000000000000000000000000001
>Q5HKE8_4fuo_2016_2222
VDGDSITSTEEIPFDKKREFDPNLAPGTEKVVQKGEPGTKTITTPTTKNPLTGEKVGEGEPTE
KITKQPVDEIVHYGGEQIPQGHKDEFDPNAPVDSKTEVPGKPGVKNPDTGEVVTPPVDDV
TKYGPVDGDSITSTEEIPFDKKREFDPNLAPGTEKVVQKGEPGTKTITTPTTKNPLTGEKVGE
GKSTEKVTKQPVDEIVEYGPT
000000000000000000000000000000000000000000000000000000000100000000000000000000000000
000000000000000000000000000000000000000000000000000000000000000000000000000000000000
00000000000000000000000000000000000000000
>Q8PVV4_2nr4_1_211
IGFLSDRQGFEKQYHDIDLSSFGIREGISEIIASTGFEHPNAAPIGIVMKGERPFVRLFKGSHT
WENVLKEKCLASNVVYDPILFVRSTFSDLVPSEFEYVDAGEFKFPVLKEAIAWVVFECINLR
NTDQSLVADLVPLNAGFNERNIKELPVPNRGFNAVLEATVHATRYQLTGEEKYLELIRHYES
LASKCGGDAEKKAMKLIYEALGL
111111111111111110000000000000000000000000000000000000000000000000000000000000000000
000000000000000000000000000000000000000000000000000000000000000000000000000000000000
000000000000000000000000000000000000000000011
>Q50EL4_3boe_196_405
SISPAQIAEALQGRGWDAEIVTDASMAGQLVDVRPEGILKCVDGRGSDNTRMGGPKMPGG
IYAIAHNRGVTSIEGLKQITKEVASKGHLPSVHGDHSSDMLGCGFFKLWVTGRFDDMGYPR
PQFDADQGANAVKDAGGIIEMHHGSHTEKVVYINLLANKTLEPNENDQRFIVDGWAADK
FGLDVPKFLIAAAATVEMLGGPKNAKIVVP
100000000000000000000000000000000000000000000000000000000000000000000000000000000000
000000000000000000000000000000000000000000000000000000000000000000000000000000000000
00000000000000000000000000000000000000000000
>Q81WF6_3m3h_0_209
MKKEIASHLLEIGAVFLQPNDPFTWSSGMKSPIYCDNRLTLSYPKVRQTIAAGLEELIKEHFP
TVEVIAGTATAGIAHAAWVSDRMDLPMCYVRSKAKGHGKGNQIEGKAEKGQKVVVVED

LISTGGSAITCVEALREAGCEVLGIVSIFTYELEAGKEKLEAANVASYSLSDYSALTEVAAEKG
IIGQAETKKLQEWRKNPADEAWITA
00000000000000000000000000000000000000000000000000000000000000000000000111000000000
00000000000111111111000000000000000000000000000000000000000000000000000000000000000
00000000000000000000000000000000000000000000
>Q2YRF5_3e7d_0_207
MTDYIRDGQAIYDRSFAIIRAEADLRHIPADLEKLAVRVIHACGMVDVANDLAFSEGAGKA
GRNALLAGAPILCDARMVAEGITRSRLPADNRVIYTLSDPSVPELAKKIGNTRSAAALDLWL
PHIEGSIVAIGNAPTALFRLFELLDAGAPKPALIIGMPVGFVGAAESKDELAANSRGVPYVIV
RGRRGGSAMTAAAVNALASERE
11100000000000000000000000000000000000000000000000000000000000000000000000000000000
00000000000000000000000000000000000000000000000000000000000000000000000000000000000
000000000000000000000000000000000000000001
>E9Q401_4etv_2698_2903
NFNPQPVDTSNITIPEKLEYFINKYAEHSHDKWSMDKLANGWIYGEIYSDSSKIQPLMKPYK
LLSEKEKEIYRWPIKESLKTMLAWGWRIERTREGDSMALYNRTRRISQTSQVSIDAAHGYSP
RAIDMSNVTLSRDLHAMAEMMAENYHNIWAKKKKLELESKGGGNHPLLVPYDTLTAKEK
AKDREKAQDIFKFLQISGYVVSR
00000000000000000000000000000000000000000000000000000000000000000000000000000000000
00000000000000000000111111111111110000000000000000000000000000000000000000000000000
0000000000000000000000000000000000000000
>O32393_1wc1_997_1201
GDLTLDLRPEPRLITILFSDIVGFTRMSNALQSQGVAELLNEYLGEMTRAVFENQGTVDKFV
GDAIMALYGAPEEMSPSEQVRRAIATARQMLVALEKLNQGWQERGLVGRNEVPPVRFRCG
IHQGMAVVGLFGSQERSDFTAIGPSVNIAARLQEATAPNSIMVSAMVAQYVPDEEIIKREFLE
LKGIDEPVMTCVINPNMLNQ
11111100000000000000000000000000000000000000000000000000000000000000000000000000000
00000000000000000000000000000000000000000000000000000000000000000000000000000000000
000000000000000000000000000000000000011
>Q00901_1r4b_40_243
SYADTFTEFTNVEEAKKWGNAQYKKYGLSKPEQEAIKFYTRDASKINGPLRANQGNENGL
PADILQKVKLIDQSFSKMKMPQNIILFRGDDPAYLGPEFQDKILNKDGTINKTVFEQVKAKF
LKKDRTEYGYISTSLMSAQFGGRPIVTKFKVTNGSKGGYIDPISYFPGQLEVLLPRNNSYYISD
MQISPNNRQIMITAMIFK
11110000000000000000000000000000000000000000000000000000000000000000000000000000000
00000000000000000000000000000000000000000000000000000000000000000000000000000000000
00000000000000000000000000000000000000
>Q59034_3aia_0_204
MREFIFKANKTITSSDINLKDLPGSCGRLDLLCRCVSDAFFLSHDIRRDVVFYAVLYGQPNPP
VCIKFVGSELKKVSPDERNIAIFIKKALKKFEELDEEQRKDWNQSTPGIYVRRLGFRNLVLEK
LEEGKNIYYLHMNGEDVENVDIENPVFIIGDHIGIGEEDERFLDEIKAKRISLSPLELHANHC
ITIIHNVLDKKRICEI
00000000000000000000000000000000000000000000000000000000000000000000000000000000000
00000000000000000000000000000000000000000000000000000000000000000000000000000000000
000000000000000000000000000000000011111
>Q8A8Z5_3g6i_18_221
GAVDLNKENRDPKYVESIVNRSQKIVDKLGLTDAKVAEDVCNVIANRYFELNDIYEIRDAK
VKAVKESGLTGDAKNEALKAAENEKDAALYRSHFAFPASLSLFLNEEQIEAVKDGMTYGV
VKVTYEATLDMIPSLKEEEKVQIYAWLVEAREFAMDAENSNKKHAAFGKYKGRINNYLAK
RGYNLTKEREEWAKRVKARGGTL
10000000000000000000000000000000000000000000000000000000000000000000000000000000000

00000000000000000000000000000000000000000000000000000000000000000000000000000000000
00000000000000000000000000000000000111
>P0A7C2_1jhf_0_201
MKALTARQQEVFDLIRDHISQTGMPPTRAEIAQRLGFRSPNAAEEHLKALARKGVIEIVSGA
SRGIRLLQEEEEGLPLVGRVAAGEPLLAQQHIEGHYQVDPSLFKPNADFLLRVSGMSMKDIG
IMDGDLLAVHKTQDVRNGQVVVARIDDEVTVKRLKKQGNKVELLPENSEFKPIVVDLRQQ
SFTIEGLAVGVIRNGDWL
10000000000000000000000000000000000000000000000000000000000000000000000000000000000
00000000000000000000000000000000000000000000000000000000000000000000000000000000000
000000000000000000000000000000000111
>Q11R54_3eup_0_201
MKELSKSDRTRQFIIESTAPVFNVKGLAGTSLTDLTEATNLTKGSIYGNFENKEAVAIAAFDY
NWGHVKSVLTAKVQACNTYKEMLLVYSSMYNDADGSLFPVGGCPLLNTTIEADDTHDAL
RKKAGEAILSWKKNLVTIIKKGIQAKEFRPDTDVTKIAFSMIALVEGAILIHRATKNRAYSDY
VFESLEDLIAGIEVKKK
00000000000000000000000000000000000000000000000000000000000000000000000000000000000
00000000000000000000000000000000000000000000000000000000000000000000000000000000000
000000000000000000000000000000000001
>O43897_3edi_147_347
AATSRTERIWPGGVIPYVIGGNFTGSQRAMFKQAMRHWEKHTCVTFIERSDEESYIVFTYRP
CGCCSYVGRRGNGPQAISIGKNCDKFGIVVHELGHVIGFWHEHTRPDRDNHVTIIRENIQP
GQEYNFLKMEPGEVNSLGERYDFDSIMHYARNTFSRGMFLDTILPSRDDNGIRPAIGQRTRL
SKGDIAQARKLYRCPA
00000000000000000000000000000000000000000000000000000000000001111000000000000000011
00000000000000000000000000000000000000000000000000000000000000000000000000000000000
00000000000000000000000000000000000
>P21457_1rec_1_201
GNSKSGALSKEILEELQLNTKFTEEELSSWYQSFLKECPSGRITRQEFQTIYSKFFPEADPKAY
AQHVFRSFDANSDGTLDFKEYVIALHMTSAGKTNQKLEWAFSLYDVDGNGTISKNEVLEIV
TAIFKMISPEDTKHLPEDENTPEKRAEKIWGFFGKKDDDKLTEKEFIEGTLANKEILRLIQFEP
QKVKEKLKEKKL
11111110000000000000000000000000000000000000000000000000000000000011111000000
00000000000000000000000000000000000000000000000000000000000000000000000000000000000
00000000000000000000000000000001111
>Q9M5P4_1rym_56_255
VEYTPWLIAGLGNPGNKYYGTRHNVGFEMVDRIAAEEGITMNTIQSKSLLGIGSIGEVPVLV
VKPQSYMNYSGEAIGPLAAYYQVPLRHILLIYDDTSLPNGVLRLQKKGGHGRHNGLQNVIE
HLDGRREFPRLSIGIGSPPGKMDPRAFLLQKFSSEERVQIDTALEQGVDAVRTLVLKGFSGST
ERFNLVQKYKFHRV
11000000000000000000000000000000000000000000000000000000000000000000000000000000000
00000000000000000000000000011111100000000000000000000000000000000000000000000000000
0000000000000000000000000001111111
>Q723E8_3fxa_0_199
MDKQAILDNIHQTWQEEANAISRLPEVTSEEALVKTVEKIAECTGKIVVAGCGTSGVAAKK
LVHSFNCIERPAVFLTPSDAVHGTLGVLQKEDILILISKGGNTGELLNLIPACKTKGSTLIGVT
ENPDSVIAKEADIFFPVSVSKEPDPFNMLATASTMAVIASFDAVIVCLMTYMNYTKEQFSVIH
PGGAVGNKLLNK
00000000000000000000000000000000000000000000000000000000000000000000000000000000000
00000000000000000000000000000000000000000000000000000000000000000000000000000000000
0000000000000000000000000111111111
>Q99593_2x6u_50_250

MEGIKVFLHERELWLKFHEVGTEMIITKAGRRMFPSYKVKVTGLNPKTKYILLMDIVPADD
HRYKFADNKWSVTGKAEPAMPGRLYVHPDSPATGAHWMRQLVSFQKLKLTNNHLDPFG
HIILNSMHKYQPRLHIVKADENNGFGSKNTAFCTHVFPETAFIAVTSYQNHKITQLKIENNP
FAKGFRGSDDMELHRMSRMQ
10000000000000000000000000000000000000000000000000000000000000000000000000000000111
00000000000000000000000000000000000000000000000000000000000000000000000000000000000
00000000000000111111111111111111111
>P02340_2ioi_91_288
PSQKTYQGNYGFHLGFLQSGTAKSVMCTYSPPLNKLFCQLAKTCPVQLWVSATPPAGSRVR
AMAIYKKSQHMTEVVRRCPHHERCSDGDGLAPPQHLIRVEGNLYPEYLEDRQTFRHSVVV
PYEPPEAGSEYTTIHYKYMCNSSCMGGMNRRPILTIITLEDSSGNLLGRDSFEVRVCACPGR
DRRTEEENFRKKEVL
11000000000000000000000000000000000000000000000000000000000000000000000000000000000
00000000000000000000000000000000000000000000000000000000000000000000000000000000000
00000000000000000000000111111111
>Q7RP52_2pwq_7_201
SKELLRLQKELKDIENENVQEIDAHIKDSNFFEWVGFIKGPEGTPYEGGHFTLAITIPNDYPY
NPPKIKFVTKIWHPNISSQTGAICLDVLKNEWSPALTIRTALLSIQALLSDPQPDDPQDAEVA
KMYKENHALFVKTASVWTKTFATGPKEEPREVIIKKITEMGFSEDQAKNALIKANWNETLA
LNTLLENS
00000000000000000000000000000000000000000000000000000000000000000000000000000000000
00000000000000000000000000000000000000000000000000000000000000000000111111111111111
11111111111111111111111111111
>O53706_2yes_0_196
MTATQITGVVLAAGRSNRLGTPKQLLPYRDTTVLGATLDVARQAGFDQLILTLGGAASAVR
AAMALDGTDVVVVEDVERGCAASLRVALARVHPRATGIVLMLGDQPQVAPATLRRIIDVG
PATEIMVCRYADGVGHPFWFSRTVFGELARLHGDKGVWKLVHSGRHPVRELAVDGCVPL
DVDTWDDYRRLLESVPS
10000000000000001111000000000000000000000000000000000000000000000000000000000100000
00000000000000000000000000000000000000000000000000000000000000000000000000000000000
0000000000000000000000000000011
>Q5HCZ5_3ftt_0_198
MTEKEKMLAEKWYDANFDQYLINERARAKDICFELNHTRPSATNKRKELIDQLFQTTTDN
VSISIPFDTDYGWNVKLGKNVYVNTNCYFMDGGQITIGDNVFIGPNCGFYTATHPLNFHH
RNEGFEKAGPIHIGSNTWFGGHVAVLPGVTIGEGSVIGAGSVVTKDIPPHSLAVGNPCKVVR
KIDNDLPSETLNDETIK
00000000000000000000000000000000000000000000000000000000000000000000000000000000000
00000000000000000000000000000000000000000000000000000000000000000000000000000000000
000000000000000000000011111111111
>Q9ADD9_2rek_0_198
MSWENPGPPKRADARRNYDRIIEAAAAEVARHGADASLEEIARRAGVGSATLHRHFPSRW
GLLQAVFQERVAQLCDEARSLAAEHPPATALTRWLTSLAVFGAVTRGAARSLLPATGTNTG
AALDSRCEQLLTEAGADLLARAQEDGTVRDDVTALELLSLANAVSLAAEHTPDAAHHAT
RLMGIALGGLGAPGPRPQG
11111111110000000000000000000000000000000000000000000000000000000000000000000000000
00000000000000000000000000000011111110000000000000000000000000000000000000000000000
000000000000000000000000000111111
>Q9I509_2a5l_0_197
MSSPYILVLYYSRHGATAEMARQIARGVEQGGFEARVRTVPAVSTECEAVAPDIPAEGALYAT
LEDLKNCAGLALGSPTRFGNMASPLKYFLDGTSSLWLTGSLVGKPAAVFTSTASLHGGQET
TQLSMLLPLLHHGMLVLGIPYSEPALLETRGGGTPYGASHFAGADGKRSLDEHELTLCRAL

GKRLAETAGKLGS
110000000000000000000000000000000000000000000001111111111110000000000000000000000000
000000000000000000000000000000000000000000000000000000000000000000111111111000000000
000000000000000000000000000000
>Q93UJ2_2xbl_0_196
MENRELTYITNSIAEAQRVMAAMLADERLLATVRKVADACIASIAQGGKVLLAGNGGSAA
DAQHIAGEFVSRFAFDRPGLPAVALTTDTSILTAIGNDYGYEKLFSRQVQALGNEGDVLIGYS
TSGKSPNILAAFREAKAKGMTCVGFTGNRGGEMRELCDLLLEVPSADTPKIQEGHLVLGHI
VCGLVEHSIFGKQ
110000000000000000000000000000000000000000000000000000000000000000000000000000000000
000000000000000000000000000000000000000000000000000000000000000000000000000000000000
00000000000000000000000000001
>Q1IYE3_2oyo_0_194
MTTTQPEAKDRISSLPVPDATQVPEGVRKLWAKAEANIGFVPNVFRAQAVNGEQFLAWW
NYFNLLLNKEGYLTNAERELVAVVVSGVNRCLYCAVSHGAALREFLGDPQKADAVAVNW
RHADLTEREQALAAYAEKLTRHPAEVTAADLEPLRAVGLDDHQIMELVQVIGMFNLTNRV
SSALGFVPNPEYYRQAR
111111111000000000000000000000000000000000000000000000000000000000000000000000000000
000000000000000000000000000000000000000000000000000000000000000000000000000000000000
000000000000000000000000000
>O01479_1pgv_198_391
GTTFNGIMQSYVPRIVPDEPDNDTDVESCINRLREDDTDLKEVNINNMKRVSKERIRSLIEA
ACNSKHIEKFSLANTAISDSEARGLIELIETSPSLRVLNVESNFLTPELLARLLRSTLVTQSIVEF
KADNQRQSVLGNQVEMDMMMAIEENESLLRVGISFASMEARHRVSEALERNYERVRLRRL
GKDPNV
111111111111111111111000000000000000000000000000000000000000000000000000000000000000
000000000000000000000000000000000000000000000000000000000000000000000000000000000000
00000000000000000000001111
>P49643_3l9q_271_463
SLDQIDLLSTKSFPPCMRQLHKALRENHHLRHGGRMQYGLFLKGIGLTLEQALQFWKQEFI
KGKMDPDKFDKGYSYNIRHSFGKEGKRTDYTPFSCLKIILSNPPSQGDYHGCPFRHSDPELL
KQKLQSYKISPGGISQILDLVKGTHYQVACQKYFEMIHNVDDCGFSLNHPNQFFCESQRILN
GGKDIKKE
000000000000000000000000000000000000000000000000000000000000011111111100000000000001
111100000000000000000000000000000000000000000000000000000000000000000000000000000000
0000000000000000001111111
>P63284_1jbk_158_350
QALKKYTIDLTERAEQGKLDPVIGRDEEIRRTIQVLQRRTKNNPVLIGEPGVGKTAIVEGLA
QRIINGEVPEGLKGRRVLALDMGALVAGAKYRGEFEERLKGVLNDLAKQEGNVILFIDELH
TMVGAGKADGAMDAGNMLKPALARGELHCVGATTLDEYRQYIEKDAALERRFQKVFVA
EPSVEDTIAILR
000000000000000000000000000000000000000000000000000000000000000000000000000000000000
000000000000000000000000000000000000000000000011111100000000000000000000000000000000
0000000000000000000000000
>P06564_1uww_570_760
GTEVEIPVVHDPKGEAVLPSVFEDGTRQGWDWAGESGVKTALTIEEANGSNALSWEFGYPE
VKPSDNWATAPRLDFWKSDLVRGENDYVTFDFYLDPVRATEGAMNINLVFQPPTNGYWV
QAPKTYTINFDELEEPNQVNGLYHYEVKINVRDITNIQDDTLLRNMMIIFADVESDFAGRVF
VDNVRFEGA
111110000000000000000000000000000000000000000000000000000000000000000000000000000000
000000000000000000000000000000000000000000000000000000000000000000000000000000000000

0000000000000000000000000
>Q9LS02_1z8k_61_252
TASRALSQNGNIENPRPSKVQELSVYEINELDRHSPKILKNAFSLMFGLGDLVPFTNKLYTG
DLKKRVGITAGLCVVIEHVPEKKGERFEATYSFYFGDYGHLSVQGPYLTYEDSFLAITGGAGI
FEGAYGQVKLQQLVYPTKLFYTFYLKGLANDLPLELTGTPVPPSKDIEPAPEAKALEPSGVIS
NYTN
11111111111111111100000000000000000000000000000000000000000000000000000000000000000
00000000000000000000000000000000000000000000000000000000000000000000000000000000000
00000000000000000000000000
>Q5ZU41_3on4_0_187
MPMNISNTKERILAVAEALIQKDGYNAFSFKDIATAINIKTASIHYHFPSKEDLGVAVISWHT
DKIAAVLSDISNNSSLSAKEKIQKFFDAILTLTYNSENKMCLGGMFASDFQSLPVSIQNQAKK
FFELIIEWLKGVLETNGYDNESSLSLAKQIISLVEGGLLLARLYGDETFLEGVRHFIDQTIK
10000000000000000000000000000000000000000000000000000000000000000000000000000000000
00000000000000000000000000000000000000000000000000000000000000000000000000000000000
0000000000000000000000
>A5K000_2gzq_0_187
MGGPPTIEELKREKIIPHVFPDENVDLTVDMYISFKSGKEVNHGNILDLAGTGSVPRNIKFSE
EPPEDYCYILFMIDPDFPSRRRPDGRDYVHWAVSGIKSKELVKGTDKNCITLLPYVGPSIKKG
TGLHRISFILSLVKEENKGNVTGVPLYRGEHYITRVKFNNCQSAYNVIQMNDMKIVGFNWC
Q
10000000000000000000000000000000000000000000000000000000000000000000000000000000000
00000000000000000000000000000000000000000000000000000000000000000000000000000000000
0000000000000000000000
>P30086_1beh_0_186
MPVDLSKWSGPLSLQEVDEQPQHPLHVTYAGAAVDELGKVLTPTQVKNRPTSISWDGLDS
GKLYTLVLTDPDAPSRKDPKYREWHHFLVVNMKGNDISSGTVLSDYVGSGPPKGTGLHRY
VWLVYEQDRPLKCDEPILSNRSGDHRGKFKVASFRKKYELRAPVAGTCYQAEWDDYVPKL
YEQLSGK
11000000000000000000000000000000000000000000000000000000000000000000000000000000000
00000000000000000000000000000000000000000000000000000000000000000000000000000000000
000000000000000000001
>Q8S380_3tc2_28_220
LPSDATPVLDVTGKELDPRLSYRIISTFWGALGGDVYLGKSPNSDAPCANGVFRYNSDVGPS
GTPVRFIGSSSHFGQGIFEDELLNIQFAISTSKMCVSYTIWKVGDYDASLGTMLLETGGTIGQ
ADSSWFKIVKSSQFGYNLLYCPVTTSSDDQFCLKVGVVHQNGKRRLALVKDNPLDVSFKQ
VQ
11100000000000000000000000000000000000000000000000000000000000000000000000000000000
00000000000000000000000000000000000000000000000000000000000000000111110000000000000
000000000000000000000
>P61316_1ua8_21_202
DAASDLKSRLDKVSSFHASFTQKVTDGSGAAVQEGQGDLWVKRPNLFNWHMTQPDESIL
VSDGKTLWFYNPFVEQATATWLKDATGNTPFMLIARNQSSDWQQYNIKQNGDDFVLTPK
ASNGNLKQFTINVGRDGTIHQFSAVEQDDQRSSYQLKSQQNGAVDAAKFTFTPPQGVTVD
DQRK
00000000000000000000000000011111000000000000000000000000000000000000000000000000000
00000000000000000000000000000000000011100000000000000000000000000000000000000000000
0000000000000000
>P12302_2vu4_81_266
AYGEAANVFGKPKKNTEFMPYNGDGFKLLVPSKWNPSKEKEFPGQVLRYEDNFDATSNLS
VLVQPTDKKSITDFGSPEDFLSQVDYLLGKQAYFGKTDSEGGFDSGVVASANVLESSTPVVD

GKQYYSITVLTRTADGDEGGKHQVIAATVKDGKLYICKAQAGDKRWFKGAKKFVESATSSF
SVA
11111111111111100000000000000000000000000000000000000000000000000000000000000000000
00000011111111111111111100000000000000000000000000011111000000000000000000000000000
00000000000000000000
>Q65AT0_2ckf_0_173
MSTEQVPVTPDVHYAVEAHYRAEVRLLQTGQYREWLHGMVAEDIHYWMPIYEQRFVRDR
RPDPTPDDAAIYNDDFEELKQRVERLYSGQVWMEDPPSKIRYFVSNVEAFEAENGELDVLS
NILVYRNRRQTEVTVHTLGREDKLRQDGNGFKVFRRKLILDARVTQDKNLYFFC
11110000000000000000000000000000000000000000000000000000000000000000000000000000000
00000000000000000000000000000000000000000000000000000000000000000000000000000000000
00000000
>Q5HKQ1_2zcm_0_184
MKDKIIDNAITLFSEKGYDGTTLDDISKSVNIKKASLYYHYDNKEEIYRKSVENCFNYFIDFLL
RNHDDNYSIDGLYQFLFKFIFDVDERYIKLYVQLSSAPEALNSEIKHHLQEINTTLHDELIKY
YDPTHIALDKEDFINLILLFLETWYFRASFSQKFGIIEDSKNRFKDQVYSLLNVFLKK
00000000000000000000000000000000000000000000000000000000000000000011110000000000000
00000000000000000000000000000000000000000000000000000000000000000000000000000000000
0000000000000000001
>Q9YBF1_3ajv_0_185
MGDRCAPIKASGVLIGDSVLVTDVEQARSLYSCGYYGQPLDVEKPRGADFEGPLRLSLIESLY
LAEKGVLEVAKPDGSSVGVEDLRTAVRGNPRFSMLYNIYRDLRERGFVVRSGLKFGSDFAVY
RLGPGIDHAPFIVHAYSPEDNIDPVEIVRAGRLSHSVRKKFVFAVTRGGDVSYLMIDWFRP
11111100000000000000000000000000000000000000000000000000000000000000000000000000000
00000000000000000000000000000000000000000000000000000000000000000000000000000000000
00000000000000000000
>P49435_1g2q_2_186
IASYAQELKLALHQYPNFPSEGILFEDFLPIFRNPGLFQKLIDAFKLHLEEAFPEVKIDYIVGLE
SRGFLFGPTLALALGVGFVPVRKAGKLPGECFKATYEKEYGSDLFEIQKNAIPAGSNVIIVDD
IIATGGSAAAAGELVEQLEANLLEYNFVMELDFLKGRSKLNAPVFTLLNAQKEALKK
00000000000000000000000000000000000000000000000000000000000000000000000000000000000
00000000000000000000000000000000000000000000000000000000000000000000000000000000000
0000000000111111111
>Q37875_1xjt_0_184
MKGKTAAGGGAICAIAVMITIVMGNGNVRTNQAGLELIGNAEGCRRDPYMCPAGVWTD
GIGNTHGVTPGVRKTDQQIAADWEKNILIAERCINQHFRGKDMPDNAFSAMTSAAFNMG
CNSLRTYYSKARGMRVETSIHKWAQKGEWVNMCNHLPDFVNSNGVPLRGLKIRREKERQ
LCLTGLVNE
11111111000000000000000000000000000000000000000000000000000000000000000000000000000
00000000000000000000000000000000000000000000000000000000000000000000000000000000000
0000000000000000000
>P0ADA1_1ivn_26_207
ADTLLILGDSLSAGYRMSASAAWPALLNDKWQSKTSVVNASISGDTSQQGLARLPALLKQ
HQPRWVLVELGGNDGLRGFQPQQTEQTLRQILQDVKAANAEPLLMQIRLPANYGRRYNE
AFSAIYPKLAKEFDVPLLPFFMEEVYLKPQWMQDDGIHPNRDAQPFIADWMAKQLQPLV
NHDS
00000000000000000000000000000001000000000000000000000000000000000000000000000000000
00000000000000000000000000000000000000000000000000000000000000000000000000000000000
0000000000000011
>Q5XAY9_2esr_12_178
LKTLDGKITRPTSDKVRGAIFNMIGPYFNGGRVLDLFAGSGGLAIEAVSRGMSAAVLVEKNR

KAQAIIQDNIIMTKAENRFTLLKMEAERAIDCLTGRFDLVFLDPPYAKETIVATIEALAAKNL
LSEQVMVVCETDKTVLLPKEIATLGIWKEKIYGISKVTVYVN
11111111111111100000000000000000000000000000000000000000000000000000000000000000
00000000000000000000000000000000000000000000000000000000000000000000000000000000
0
>Q94734_4hpb_21_204
ACTKNAIAQTGFNKDKYFNGDVWYVTDYLDLEPDDVPKRYCAALAAGTASGKLKEALYH
YDPKTQDTFYDVSELQVESLGKYTANFKKVDKNGNVKVAVTAGNYYTFTVMYADDSSALI
HTCLHKGNKDLGDLYAVLNRNKDAAAGDKVKSAVSAATLEFSKFISTKENNCAYDNDSLK
SLLTK
00000000000000000000000000000000111110000000000000000000000000000000000000000000
00000000000000000000000000000000000000000000000000000000000000000000000000000000
000000000000000000
>O59483_2een_0_182
MYEVELKGYANDEIFEKVRETFEFMRKEIHEDIYYQHPCRDFSKTDEALRIRIKRFNGHNEV
FLTYKGPKIDEKSKTRLEIEVEIQEDVDKYFELLDRLGFKEVLKVVKTREKYYVEKGVTITLDE
VEGLGKFIEIETLVKEKDEIPEAVEKLEKILRELGVEKFERRSYLELLLEKRTELNI
10000000000000000000000000000000000000000000000000000000000000000000000000000000
00000000000000000000000000000000000000000000000000000000000000000000000000000000
00000000000000001
>Q9I4D4_1rtt_1_180
SDDIKVLGISGSLRSGSYNSAALQEAIGLVPPGMSIELADISGIPLYNEDVYALGFPPAVERFRE
QIRAADALLFATPEYNYSMAGVLKNAIDWASRPPEQPFSGKPAAILGASAGRFGTARAQYH
LRQTLVFLDVHPLNKPEVMISSAQNAFDAQGRLLDDKARELIQQQLQALQLWVR
11100000000000000000000000000000000000000000000000000000000000000000000000000000
00000000000000000000000000000000000000000000000000000000000000000000000000000000
00000000000111
>P0A5L0_1nwa_0_181
MTSNQKAILAGGCFWGLQDLIRNQPGVVSTRVGYSGGNIPNATYRNHGTHAEAVEIIFDPT
VTDYRTLLEFFFQIHDPTTKDRQGNDRGTSYRSAIFYFDEQQKRIALDTIADVEASGLWPGK
VVTEVSPAGDFWEAEPEHQDYLQRYPNGYTCHFVRPGWRLPRRTAESALRASLSPELGT
00000000000000000000000000000000000000000000000000000000000000000000000000000000
00000000000000000000000000000000000000000000000000000000000000000000000000000000
1111111111111111
>Q8ZM82_3hyq_0_180
MTEEHVVLLDEQDKPSGTLEKYAAHTLNTPLHLAFSCWLFNEDGQLLVTRRSLSKKAWPG
VWTNSVCGHPQQGETTEEAIIRRCRFELGVEITDLTPVYPHFSYRATDPNGIVENEVCPVFA
ARATSVLQVNSEEVMDYQWSEFKSVWKSLLATPWAFSPWMVMQASDEQARERLLNYCQR
11111111111111111111111111111000000000000000000000000000000000000000000000000000
00000000000000000000000000000000000000000000000000000000000000000000000000000000
000000000000000
>Q8PJY1_2g3w_0_181
MALTATVRRAELQISDMDRGYYANHSLTLAQHPSETDERLMVRLLAFALFADDRLEFGRGL
SNDDEPDLWRRDYTGDPDLWIDLGQPDESRVRKACNRSREAVVIGYGGQATETWWKKHA
NAMGRYRNLRVIELDSQATEALGALIQRGMRFDVIIQDGEVQMLADHGSVTLTPMVRQAP
AE
11100000000000000000000000000000000000000000000000000000000000000000000000000000
00000000000000000000000000000000000000000000000000000000000000000000000000000000
000000000000000
>Q41261_1qou_0_180
MAAKVSSDPLVIGRVIGDVVDHFTSTVKMSVIYNSNNSIKHVYNGHELFPSAVTSTPRVEVH

GGDMRSFFTLIMTDPDVPGPSDPYLREHLHWIVTDIPGTTDSSFGKEVVSYEMPRPNIGIHRF
VFLLFKQKKRGQAMLSPPVVCRDGFNTRKFTQENELGLPVAAVFFNCQRETAARRR
111100000000000000000000000000001100000000000000000000000000000000000000000000000000
000000000000000000000000000000000000000000000000000000111111100000000000000000000000
000000000011111
>P59915_3km5_1136_1313
ADFTETFESSTHGEAPAEWTTIDADGDGQGWLCLSSGQLDWLTAHGGTNVVASFSWNGM
ALNPDNYLISKDVTGATKVKYYYAVNDGFPGDHYAVMISKTGTNAGDFTVVFEETPNGIN
KGGARFGLSTEANGAKPQSVWIERTVDLPAGTKYVAFRHYNCSDLNYILLDDIQFTMGG
000000000000000000000000000000000000000000000000000000000000000000000000000000000000
000000000000000000000000000000000000000000000000000000000000000000000000000000000000
000000000001
>Q96Y52_3iwt_0_177
MSHAHKKHKENAPKSLNFYVITISTSRYEKLLKKEPIVDESGDIIKQLLIENGHKIIGYSLVPD
DKIKILKAFTDALSIDEVDVIISTGGTGYSPTDITVETIRKLFDREIEGFSDVFRLVSFNDPEVKA
AAYLTKASAGIIGKKIVYLLPGSPDAVKLALKELILPEVGHLVYLVRS
111111111111000000000000000000000000000000000000000000000000000000000000000000000000
000000000000000000000000000000000000000000000000000000000000000000000000000000000000
000000000000
>Q13526_3kai_0_162
MADEEKLPPGWEKRMSRSSGRVYYFNHITNASQWERPSGNSSSGGKNGQGEPARVRCSHL
LVKHSQSRRPSSWRQEKITRTKEEALELINGYIQKIKSGEEDFESLASQFSDCSSAKARGDLG
AFSRGQMQKPFEDASFALRTGEMSGPVFTDSGIHIILRTE
111111000000000000000000000000000000001111111111111000000000000000000000000000000000
000000000000000000000000000000000000000000000000000000000000000000000000000000000
>O58465_3d79_1_174
FRMELKIKHPLSKKDVKEIIAQLSQMFGEEIARKMLNKKDEVKVAEFDKTTEIILVNDKPMFI
RRKDLIFPLVIALYNLSDEEDLRKWPRRVVVDEGAVPHILNGADVMAPGIVDADEGIKEGD
FVFVVEEKYGRPLAIGIALMSGKVMKEKNRGKAVKVIHHARDKIWEVTAR
111111000000000000000000000000000000000000000000000000000000000000000000000000000000
000000000000000000000000000000000000000000000000000000000000000000000000000000000000
00000001
>P0A4Z2_2g1k_0_175
MAPKAVLVGLPGSGKSTIGRRLAKALGVGLLDTDVAIEQRTGRSIADIFATDGEQEFRRIEED
VVRAALADHDGVLSLGGGAVTSPGVRAALAGHTVVYLEISAAEGVRRTGGNTVRPLLAG
PDRAEKYRALMAKRAPLYRRVATMRVDTNRRNPGAVVRHILSRLQVPSPSEAAT
100000000000000000000000000000000000000000000000000000000000000000000000000000000000
000000000000000000000000000000000000000000000000000000000000000000000000000000000000
0001111111
>P35247_3ikn_200_374
PGLKGDKGIPGDKGAKGESGLPDVASLRQQVEALQGQVQHLQAAFSQYKKVELFPNGQS
VGEKIFKTAGFVKPFTEAQLLCTQAGGQLASPRSAAENAALQQLVVAKNEAAFLSMTDSK
TEGKFTYPTGESLVYSNWAPGEPNDDGGSEDCVEIFTNGKWNDRACGEKRLVVCEF
111111111111111111111110000000000000000000000000000000000000000000000000000000000000
000000000000000000000000000000000000000000000000000000000000000000000000000000000000
000000000
>Q14623_3k7j_27_201
CGPGRVVGSRRRPPRKLVPLAYKQFSPNVPEKTLGASGRYEGKIARSSERFKELTPNYNPDIIF
KDEENTGADRLMTQRCKDRLNSLAISVMNQWPGVKLRVTEGWDEDGHHSEESLHYEGR
AVDITTSDRDRNKYGLLARLAVEAGFDWVYYESKAHVHCSVKSEHSAAAKTGG
111111111111100000000000000000000000000000000000000000000000000001111110000000000000

00000000000000000000000000000000000000000000000000000000000000000000000000000000001
111111111
>A3DF10_3kcp_26_199
RADKASSIELKFDRNKGEVGDILIGTVRINNIKNFAGFQVNIVYDPKVLMAVDPETGKEFTS
STFPPGRTVLKNNAYGPIQIADNDPEKGILNFALAYSYIAGYKETGVAESSGIIAKIGFKILQK
KSTAVKFQDTLSMPGAISGTQLFDWDGEVITGYEVIQPDVLSLGDEPY
11111000000000000000000000000000000000000000000000000000000000000000000000000000000
00000000000000000000000000000000000000000000000000000000000000000000000000000000000
00111111
>P09464_1kxo_16_188
VYHDGACPEVKPVDNFDWSNYHGKWWEVAKYPNSVEKYGKCGWAEYTPEGKSVKVSN
YHVIHGKEYFIEGTAYPVGDSKIGKIYHKLTYGGVTKENVFNVLSTDNKNYIIGYYCKYDED
KKGHQDFVWVLSRSKVLTGEAKTAVENYLIGSPVVDSQKLVYSDFSEAACKVNN
00000000000000000000000000000000000000000000000000000000000000000000000000000000000
00000110000000000000000000110000000000000000000000000000000000000000000000000000000
1100111
>P14774_2c8s_25_196
QPQSGPQTGVVFRNTVTGEALDVSQGKEGGRDTPAVKKFLETGENLYIDDKSCLRNGESLF
ATSCSGCHGHLAEGKLGPGLNDNYWTYPSNTTDVGLFATIFGGANGMMGPHNENLTPDE
MLQTIAWIRHLYTGPKQDAVWLNDEQKKAYTPYKQGEVIPKDAKGQCKPLDE
11111111111111111111111000000000000000000000000000000000000000000000000000000000000
00000000000000000000000000000000000000000000000000000000000000000000000000000000000
000000
>O14593_3uxg_89_259
DSLSIHQLAAQGELDQLKEHLRKGDNLVNKPDERGFTPLIWASAFGEIETVRFLLEWGADP
HILAKERESALSLASTGGYTDIVGLLLERDVDINIYDWNGGTPLLYAVRGNHVKCVEALLA
RGADLTTEADSGYTPMDLAVALGYRKVQQVIENHILKLFQSNLVPADPE
10000000000000000000000000000000000000000000000000000000000000000000000000000000000
00000000000000000000000000000000000000000000000000000000000000000000000000000000011
11111
>P16559_2rer_0_169
MAARTDNSIVVNAPFELVWDVTNDIEAWPELFSEYAEAEILRQDGDGFDFRLKTRPDANG
RVWEWVSHRVPDKGSRTVRAHRVETGPFAYMNLHWTYRAVAGGTEMRWVQEFDMKPG
APFDNAHMTAHLNTTTRANMERIKKIIEDRHREGQRTPASVLPTELHAQQLLL
00000000000000000000000000000000000000000000000000000000000000000000000000000000000
00000000000000000000000000000000000000000000000000000000000000000000000011111111111
1111
>P86294_2jkg_0_169
MAEEYSWDSYLNDRLLATNQVSGAGLASEEDGVVYACVAQGEESDPNFDKWSLFYKEDY
DIEVEDNGTKTTKTINEGQTILVVFNEGYAPDGVWLGGTKYQFINIERDLEFEGYNFDVATC
AKLKGGLHLVKVPGGNILVVLYDEEKEQDRGNSKIAALTFAKELAESSQ
11110000000000000000000000000000000000000000000000000000000000000000000000000000000
00000000000000000000000000000000000000000000000000000000000000000000000000000000000
0011
>A9CFJ8_1vki_0_168
MTENSRKTATELFEFLDGLGISHTTKQHEPVFTVAESQSLRDLIPGGHTKNLFVKDKKDQYF
VLTVEENAVVDLKSVHKTIGAASRVSFGRPEKMLEYLGVVPGSVTVFGAINDTARQVTFVL
DSDLLENELVNGHPLSNDQTTTIASKDLIRFLEATGHAPLVLKVSE
11100000000000000000000000000000000000000000000000000000000000000000000000000000000
00000000000000000000000000000000000000000000000000000000000000000000000000000000000
000

>P72223_1t3q_0_167
MQAHEESQLMRISATINGKPRVFYVEPRMHLADALREVVGLTGTKIGCEQGVCGSCTILID
GAPMRSCLTLAVQAEGCSIETVEGLSQGEKLNALQDSFRRHHALQCGFCTAGMLATARSIL
AENPAPSRDEVREVMSGNLCRCTGYETIIDAITDPAVAEAARRGEV
11111100000000000000000000000000000000000000000000000000000000000000000000000000000
00000000000000000000000000000000000000000000000000000000000000000000000000000000000
00
>Q83BF9_3trc_7_174
MNMLKILRQITQEVNAAPNLEQALKLVVVRLCEALPADACSLFICDDVHGEYVLMATQGL
NSKQVGKLRLKFGEGLIGLVGEREEPINLADAPLHPAYKHRPELGEEDYHGFLGIPIIEQGEL
LGILVIQQLESHHFAEEEEAFCVTLAIHLAAEIAHARAKGALEKL
00000000000000000000000000000000000000000000000000000000000000000000000000000000000
00000000000000000000000000000000000000000000000000000000000000000000000000000000001
11
>Q8FHW2_3ogh_0_167
MNRIEHYHDWLRDAHAMEKQAESMLESMASRIDNYPELRARIEQHLSETKNQIVQLETIL
DRNDISRSVIKDSMSKMAALGQSIGGIFPSDEIVKGSISGYVFEQFEIACYTSLLAAAKNAGD
TASIPTIEAILNEEKHMADWLIQHIPQTTEKFLIRSETDGVEAKK
00000000000000000000000000000000000000000000000000000000000000000000000000000011111
11111110000000000000000000000000000000000000000000000000000000000000000000000001111
11
>P77731_1xsq_0_159
MKLQVLPLSQEAFSAYGDVIETQQRDFFHINNGLVERYHDLALVEILEQDCTLISINRAQPA
NLPLTIHELERHPLGTQAFIPMKGEVFVVVVALGDDKPDLSTLRAFITNGEQGVNYHRNV
WHHPLFAWQRVTDFLTIDRGGSDNCDVESIPEQELCFA
00000000000000000000000000000111100000000000000000000000000000000000000000000000000
00000000000000000000000000000000000000000000000000000000000110000000000000000
>Q92830_1z4r_496_661
GIIEFHVIGNSLTPKANRRVLLWLVGLQNVFSHQLPRMPKEYIARLVFDPKHKTLALIKDGR
VIGGICFRMFPTQGFTEIVFCAVTSNEQVKGYGTHLMNHLKEYHIKHNILYFLTYADEYAIG
YFKKQGFSKDIKVPKSRYLGYIKDYEGATLMECELNPRIPYT
00000000000000000000000000000000000000000000000000000000000000000000000000000000000
00000000000000000000000000000000000000000000000000000000000000000000000000000001111
>Q396C9_3juq_0_164
MSDVESLENTSENRAQVAARQHNRKIVEQYMHTRGEARLKRHLLFTEDGVGGLWTTDSG
QPIAIRGREKLGEHAVWSLQCFPDWVWTDIQIFETQDPNWFWVECRGEGAIVFPGYPRGQ
YRNHFLHSFRFENGLIKEQREFMNPCEQFRSLGIEVPEVRRDGLPS
11111110000000000000000000000000000000000000000000000000000000000000000000000000000
0000000000000000000000000000000000000000000000000000000000000000000000000000000001
>Q86V88_2wm8_0_164
MARLPKLAVFDLDYTLWPFWVDTHVDPPFHKSSDGTVRDRRGQDVRLYPEVPEVLKRLQS
LGVPGAAASRTSEIEGANQLLELFDLFRYFVHREIYPGSKITHFERLQQKTGIPFSQMIFFDDE
RRNIVDVSKLGVTCIHIQNGMNLQTLSQGLETFAKAQTGPL
00000000000000000000000000000000000000000000000000000000000000000000000000000000000
0000000000000000000000000000000000000000000000000000000000000000000000000000001111
>P83051_2gzb_0_163
SVILDTKGEPVSNAADAYYLVPVSHGEGGLALAKVGNEAEPKAVVLDPHHRPGLTVRFETP
LAIAIITESFFLNIKFVPSSSDSEVWDVSKQYPIGLAVKVTDTKSFVGPFRVEKEGEGYKIVYYP
DRGQTGLDIGLVHRNDKYYLAATEGEPFVFKIRKATYE
00000000000000000000000000000000000000000000000000000000000000000000000000000000000
000000000000000000000000000000000000000000000000000000000000000000000000000000011

>O30195_3vu0_432_592
EMTMDWKEALNWMKENLEAQDYLKAYEKPDYAVLSWWDYGNWILYVAKKAVVCNNF
QAGADDAAKFFTAQSEEEAMKIVEKRKVRYVVTVEELTVKPETNKTKFIPIMQIAGYSPEY
MKNKEIIDFFNKTMLYKLHVENATNLTHFRLLKNFGTVKIFEVK
0000000000000000000011111111000000000000000000000000000000000000000000000000000000000
000000000000000000000000000000000000000000000000000000000000000000000000000000
>P35555_1uzp_1485_1646
TDVNECLDPTTCISGNCVNTPGSYICDCPPDFELNPTRVGCVDTRSGNCYLDIRPRGDNGD
TACSNEIGVGVSKASCCCSLGKAWGTPCEMCPAVNTSEYKILCPGGEGFRPNPITVILEDIDE
CQELPGLCQGGKCINTFGSFQCRCPTGYYLNEDTRVCD
0000000000000000000000000000000000000000000000000000011111111110000000000000000000000
0000000000000000000000000000000000000000000000000000000000000000000000000000000
>Q5SHE1_3jqj_0_156
MDLTHFQDGRPRMVDVTEKPETFRTATAEAFVELTEEALSALEKGGVGKGDPLVVAQLAGI
LAAKKTADLIPLCHPLPLTGVEVRVELLKAEKRVRIEATVKTKAETGVEMEAMTACAVAAL
TVYDMLKAASKGLVISQVRLLHKAGGKSGEWRREQ
11111111000000000000000000000000000000000000000000000000000000000000000000000000000
00000000000000000000000000000000000000000000000000000000000000000000000001
>Q8P9V9_3gkk_0_159
MTDAVLELPAATFDLPLSLSGGTQTTLRAHAGHWLVIYFYPKDSTPGCTTEGLDFNALLPEF
DKAGAKILGVSRDSVKSHDNFCAKQGFAFPLVSDGDEALCRAFDVIKEKNMYGKQVLGIE
RSTFLLSPEGQVVQAWRKVKVAGHADAVLAALKAHAKQ
00000000000000000000000000000000000000000000000000000000000000000000000000000011100
0000000000000000000000000000000000000000000000000000000000000000000000000011
>P45202_1dbu_0_157
MTPAIDLLKKQKIPFILHTYDHDPNNQHFGDEAAEKLGIDPNRSFKTLLVAENGDQKKLAC
FVLATANMLNLKKAAKSIGVKKVEMADKDAAQKSTGYLVGGISPLGQKKRVKTVINSTAL
EFETIYVSGGKRGLSVEIAPQDLAKVLGAEFTDIVDE
10000000000000000000000001111100000000000000000000000000000000000000000000000000000
000000000000000000000000000000000000000000000000000000000000000000000000000
>P26281_3ude_1_158
TVAYIAIGSNLASPLEQVNAALKALGDIPESHILTVSSFYRTPPLGPQDQPDYLNAAVALETS
LAPEELLNHTQRIELQQGRVRKAERWGPRTLDLDIMLFGNEVINTERLTVPHYDMKNRGF
MLWPLFEIAPELVFPDGEMLRQILHTRAFDKLNKW
00000000000000000000000000000000000000000000000000000000000000000000000000000000001
111000000000000000000000000000000000000000000000000000000000000000000000000
>Q8RQP5_1iv1_0_151
MRIGYGEDSHRLEEGRPLYLCGLLIPSPVGALAHSDGDAALHALTDALLSAYGLGDIGLLFP
DTDPRWRGERSEVFLREALRLVEARGAKLLQASLVLTLDRPKLGPHRKALVDSLSRLLRLPQ
DRIGLTFKTSEGLAPSHVQARAVVLLDG
10000000000000000000000000000000000000000000000000000000000000000000000000000000000
000000000000000000000000000000000000000000000000000000000000000000001
>Q5SHN1_2cx5_0_157
MSLSPSARRVQGALETRGFGHLKVVELPASTRTAKEAAQAVGAEVGQIVKSLVFVGEKGAY
LFLVSGKNRLDLGKATRLVGGPLRQATPEEVRELTGFAIGGVPPVGHNTPLPAYLDEDLLGY
PEVWAAGGTPRALFRATPKELLALTGAQVADLKEG
10000000000000000000000000000000000000000000000000000000000000000000000000000000000
000000000000000000000000000000000000000000000000000000000000000000000000000
>Q99ZZ7_2wh7_214_371
NAVNIVMRQPTTPNFSSALNITSANEGGSAMQIRGVEKALGTLKITHENPSVDKEYDKNA
AALSIDIVKKQKGGKGTAAQGIYINSTSGTTGKLLRIRNLNDDKFYVKPDGGFYAKETSQID

GNLKLKDPIANDHAATKAYVDGEVEKLKALLAAKQM
00000000000000000000000000000000000000000000000000000000000000000000000000000000000
000000000000000000000000000000000000000000000000000000000000000000000111111
>A9CJ63_3d01_0_155
MSDVIEGRLKELGFTLPVAAAPAANYVPFTISGNLLYVSGQLPMESGKIAVTGLVGRDVDVA
SAQRAAELCAVNILAQVKAALNGDLSKIRRVIKLNGFVASVPEFVEQHLVINGASNLIATVL
GEPGRHARAAVGMASLPFNASVEIDAIVEIDV
00000000000000000011111000000000000000000000000000000000000000000000000000000000000
0000000000000000000000000000000000000000000000000000000000000000000000001
>P14147_3cgz_331_486
VLLSRELHPVAPLLDNLISALNKVYQRKGVNISMDISPEISFVGEQNDFVEVMGNVLDNAC
KYCLEFVEISARQTDDHLHIFVEDDGPGIPHSKRSLVFDRGQRADTLRPGQGVGLAVAREIT
EQYAGQIIASDSLLGGARMEVVFGRQHPTQKEE
11110000000000000000000000000000000000000000000000000000000000000000000000000000000
0000000001111111111111100000000000000000000000000000000000000000000111111
>P30960_1kng_38_193
DPSRIPSALIGRPAPQTALPPLEGLQADNVQVPGLDPAAFKGKVSLVNVWASWCVPCHDE
APLLTELGKDKRFQLVGINYKDAADNARRFLGRYGNPFGRVGVDANGRASIEWGVYGVPE
TFVVGREGTIVYKLVGPITPDNLRSVLLPQMEKALK
11111111111100000000000000000000000000000000000000000000000000000000000000000000000
0000000000000000000000000000000000000000000000000000000000000000000000001
>Q8D0Z7_3qra_38_193
EGESSISIGYAQSRVKEDGYKLDKNPRGFNLKYRYEFNNDWGVIGSFAQTRRGFEESVDGFK
LIDGDFKYYSVTAGPVFRINEYVSLYGLLGAGHGKAKFSSIFGQSESRSKTSLAYGAGLQFNP
HPNFVIDASYEYSKLDDVKVGTWMLGAGYRF
00000000000000000000000000000000000000000000000000000000111100000000000000000000000
0000000000000000000100000000000000000000000000000000000000000000000000000
>Q93SU7_3h08_0_145
MEKTITIYTDGACSGNPGKGGWGALLMYGSSRKEISGYDPATTNNRMELMAAIKGLEALK
EPCRVQLYSDSAYLVNAMNEGWLKRWVKNGWKTAAKKPVENIDLWQEILKLTTLHRVTF
HKVKGHSDNPYNSRCDELARLAIKENS
10000000000000000000000000000000000000000000000000000000000000000000000000000000000
000000000010000000000000000000000000000001000000000000000000001
>B7FGU6_3us6_0_152
MEVGQMRRQWVDYIKSMFMEGFLDGQFLQLQQLQDENNPEFVFEVVSLFFDDSERILKDL
SFAVDQQSIDFKKVDAHVHQFKGSSASIGAQRVKNSCVAFRNFCEEQNIDACRRCLQQVK
QEYLLVKNKLETLLRLEQQIVAAGGSIPMMELN
10000000000000000000000000000000000000000000000000000000000000000000000000000000000
0000000000000000000000000000000000000000000000000000000000000000001111
>Q12KE2_3lza_17_156
KVGDRPIEQQLALGYIKALTEHDYQTLSKYYNRDSVFYDKTADTKYIGTRSIIAFLQRSHEGV
LEFDFNIEHMFNTGPLVVMIGNYHLRGPGEQFGKPGKIIDIAIPGVTTLKFDPNTQRLTEQV
DLMDYQTMSDQLQSQ
11100000000000000000000000000000000000000000000000000000000000000000000000000000000
000000000000000000000000000000000000000000000000000000000
>Q7D1C9_2gmy_0_152
MKTRINYAKASPEAFKAVMALENYVQSSGLEHRFIHLIKLRASIINGCAFCVDMHVKESRH
DGLSEQWINLMSVWRESPVYTEQERALLGWVDAVTKIAETGAPDDAFETLRAHFSDEEIVK
ITVAIGAINTWNRIAVGFRSQHPVEAAAKAA
10000000000000000000000000000000000000000000000000000000000000000000000000000000000
0000000000000000000000000000000000000000000000000000000000000000111111

>Q9BVM4_3juc_0_152
MALVFVYGTLKRGQPNHRVLRDGAHGSAAFRARGRTLEPYPLVIAGEHNIPWLLHLPGSG
RLVEGEVYAVDERMLRFLDDFESCPALYQRTVLRVQLLEDRAPGAEEPPAPTAVQCFVYSRA
TFPPEWAQLPHHDSYDSEGPHGLRYNPRENR
00000000000000000000000000000000000000000000000000000000000000000000000000000000000
0000000000000000000001100000000000000000000000000000000000000000000011
>A9CK16_2a8n_0_143
MAERTHFMELALVEARSAGERDEVPIGAVLVLDGRVIARSGNRTRELNDVTAHAEIAVIRM
ACEALGQERLPGADLYVTLEPCTMCAAAISFARIRRLYYGAQDPKGGAVESGVRFFSQPTC
HHAPDVYSGLAESESAEILRQF
10000000000000000000000000000000000000000000000000000000000000000000000000000000000
0000000000000000000000000000000000000000000000000000000000111
>Q9RMX2_3pmc_0_135
MEEIKCLLCRYLKERQEKFISDWKKKVIIRERDPYKEEIIKNGEHLLSAFIMYLKEEISLQEIEIT
SKKIARERIDAKVNIAEFIHNTNVAKIEIMNILTLLNPDLQQYQALVKKINQFFDHLIYYTVH
SYYEQKA
11110000000000000000000000000000000000000000000000000000000000000000000000000000000
00000000000000000000000000000000000000000000000000000
>Q8A8Q1_3htn_37_184
AQNEKNMYSYKKIGNKYIVSINNHTEIVKALNAFCKEKGILSGSINGIGAIGELTLRFFNPKT
KAYDDKTFREQMEISNLTGNISSMNEQVYLHLHITVGRSDYSALAGHLLSAIQNGAGEFVV
EDYSERISRTYNPDLGLNIYDFER
11111000000000000000000000000000000000000000000000000000000000000000000000000000000
00000000000000000000000000000000000000000000000000000000000000000
>Q09JX9_3bu1_16_162
QQQCDTVSAWQSLRGPGTGGYYLFKTTEGGKTDCTYVKGSNFNDAAQTATYTYGNLGSG
NQLTQQTASASISGNAIVVGTDHSEVLYSDGSTCDVVRLNGQIELWIHSSATSNTGNLNSCC
TDKFNQEKGSRPEHVVYRSTCPNLPQ
11100000000000000000000000000000000000000000000000000000000000000000000000000000000
0000000000000000000000000000000000000000000000000000000000000000
>O96438_1qk8_0_145
MSGLDKYLPGIEKLRRGDGEVEVKSLAGKLVFFYFSASWCPPCRGFTPQLIEFYDKFHESKN
FEVVFCTWDEEEDGFAGYFAKMPWLAVPFAQSEAVQKLSKHFNVESIPTLIGVDADSGDVV
TTRARATLVKDPEGEQFPWKDAP
11000000000000000000000000000000000000000000000000000000000000000000000000000000000
000000000000000000000000000000000000000000000000000000000000001
>Q5YUF0_3r5y_0_145
MRMSSTGEYVPSPSEWIGNQVAQYEASDGAEAGEFDGRPLVILTTVGRKTGALRKTPVMRV
EHDGRYAVVASQGGAPTHPAWYFNLVADPRAQLRDKDAVLSVVARELAGPERAEWWERA
VRAYPTYQEYQDNTRRLIPVLLLEPG
11111000000000000000000000000000000000000000000000000000000000000000000000000000000
000000000000000000000000000000000000000000000000000000000000000
>Q63HR2_3hqc_1263_1408
STAADLLRQGAACSVLYLTSVETESLTGPQAVARASSAALSCSPRPTPAVVHFKVSAQGITLT
DNQRKLFFRRHYPVNSITFSSTDPQDRRWTNPDGTTSKIFGFVAKKPGSPWENVCHLFAEL
DPDQPAGAIVTFITKVLLGQRK
00000000000000000000000000000000000000000001110000000000000000000000000000000000000
000000000000000000000000000000000000000000000000000000000000111
>Q15819_1j7d_0_144
MAVSTGVKVPRNFRLLEELEEGQKGVGDGTVSWGLEDDEDMTLTRWTGMIIGPPRTNYEN
RIYSLKVECGPKYPEAPPSVRFVTKINMNGINNSSGMVDARSIPVLAKWQNSYSIKVVLQEL

RRLMMSKENMKLPQPPEGQTYNN
11111000000000000000000000000000000000000000000000000000000000000000000000000000000
00000000000000000000000000000000000000000000000000000000000000
>Q9RTA8_1usp_0_138
MANVYTAEATATGGRAGTTRSSDDRLNLDLSVPAEMGGDGGPGTNPEQLFAAGYAACFQ
GALGVVSRRQKIDVPADSTITARVGLQKAGLAFALDVELEGHFPGLSREQAEGLMHAAHE
VCPYSAATRNNVDVRLKVRE
10000000000000000000000000000000000000000000000000000000000000000000000000000000000
00000000000000000000000000000000000000000000000000000000
>F9VPE5_1j30_0_143
MKDLKGTKTAENLKQGFIGESMANRRYLYFAKRADEEGYPEIAGLLRSIAEGETAHAFGHL
DFIRQGGLTDPATDKPIGTLEQMIESAIAGETYEWTQMYPGFAKVAREEGFPEVAEWFETLA
RAEKSHAEKFQNVLKQLKGGT
11000000000000000000000000000000000000000000000000000000000000000000000000000000000
0000000000000000000000000000000000000000000000000000000000001
>Q9ZA17_2zez_755_898
VSNLIVNGTAENGMDGWPDWGYPVSAVPEAAYGGTKGFKLSGGKQAGMGQKVALKPNT
TYILGAWGKFTAKPGTYCDVIVQYHLKDANNTYVQNILRFTETDWTYKQVVFTTPDAFGS
DPEFVLWKDDASNADFYADNITLVEV
10000000000000000000000000000000000000000000000000000000000000000000000000000000000
0000000000000000000000000000000000000000000000000000000000001
>O52646_2f99_1_143
SEQIAAVRRMVEAYNTGKTDDVADYIHPEYMNPGTLEFTSLRGPELFAINVAWVKKTFSEE
ARLEEVGIEERADWVRARLVLYGRHVGEMVGMAPTGRLFSGEQIHLLHFVDGKIHHHRD
WPDYQGTYRQLGEPWPETEHRRP
00000000000000000000000000000000000000000000000000000000000000000000000000000000000
000000000000000000000000000000000000000000000000000000000111
>Q5LG06_4fr9_23_165
GDDDDTGYLPPSQAIQDALKKLYPNATAIKWEQKGVYYVADCQADGREKEVWFDANAN
WLMTETELNSINNLPPAVLTAFMESSYNNWVVDDVVILEYPNEPSTEFVVTVEQGKKVDLY
FSEGGGLLHEKDVTNGDDTHWPRV
11000000000000000000000000000000000000000000000000000000000000000000000000000000000
000000000000000000000000000000000000000000000000000000000000
>P83876_1qgv_0_141
MSYMLPHLHNGWQVDQAILSEEDRVVVIRFGHDWDPTCMKMDEVLYSIAEKVKNFAVIY
LVDITEVPDFNKMYELYDPCTVMFFFRNKHIMIDLGTGNNNKINWAMEDKQEMVDIIETV
YRGARKGRGLVVSPKDYSTKYRY
10000000000000000000000000000000000000000000000000000000000000000000000000000000000
00000000000011111100000000000000000000000000000000000001111
>Q91YR1_1m4j_0_141
MSHQTGIQASEDVKEIFARARNGKYRLLKISIENEQLVVGSCSPPSDSWEQDYDSFVLPLLED
KQPCYVLFRLDSQNAQGYEWIFIAWSPDHSHVRQKMLYAATRATLKKEFGGGHIKDEVFG
TVKEDVSLHGYKKYLLSQS
11111100000000000000000000000000000000000000000000000000000000000000000000000000000
00000000000000000000000000000000000000000000000000000000111
>Q8EGL1_2ooj_0_139
MEMTKVTGKFDVKLTPENAYATGVGGVNLGRMALDKTFYGELEARSQGEMLSAMTAVKG
SAGYVAIEQVVGKLCGRQGSFVLQHFGIMTDGQNRLHLEVVPHSGAGELTGLYGTMAISIE
NGQHFYEFSFCFEPASEVEG
00000000000000000000000000000000000000000000000000000000000000000000000000000000000
000000000000000000000000000000000000000000000000000011111

>Q9UKK6_1jkg_0_139
MASVDFKTYVDQACRAAEEFVNVYYTTMDKRRRLLSRLYMGTATLVWNGNAVSGQESLS
EFFEMLPSSEFQISVVDCQPVHDEATPSQTTVLVVICGSVKFEGNKQRDFNQNFILTAQASPS
NTVWKIASDCFRFQDWAS
100000000000000000000000000000000000000000000000000000000000000000000000000000000000
00000000000000000000000000000000000000000000000000000000
>Q9ULD4_3pfs_1055_1194
DYNGSGRSLLLPFEDRGDLEPLELVWAKCRGYPSYPALIIDPKMPREGLLHNGVPIPVPPLD
VLKLGEQKQAEAGEKLFLVLFFDNKRTWQWLPRDKVLPLGVEDTVDKLKMLEGRKTSIRK
SVQVAYDRAMIHLSRVRG
111110000000000000000000000000000000000000000000000000000000000000000000000000000000
00000000000000000000000000000000000000000000000000000001
>P45947_1jl3_0_138
MENKIIYFLCTGNSCRSQMAEGWAKQYLGDEWKVYSAGIEAHGLNPNAVKAMKEVGIDIS
NQTSDIIDSDILNNADLVVTLCGDAADKCPMTPPHVKREHWGFDDPARAQGTEEEKWAF
FQRVRDEIGNRLKEFAETGK
110000000000000000000000000000000000000000000000000000000000000000000000000000000000
0000000000000000000000000000000000000000000000000000000
>Q2T2I4_3ec9_0_138
MRNPSEDHMMRTPYQIVADHYAASDRHDPAAMMADIAPAIEWTEMAGFPCAGTYRSAD
EIVRNVFRRLGEEWDGYTFKLDALHDAGDTVIGVGRYSGTYRRTGKSFECRVAHVWRVDA
GKIVHFEQFTDTLLVAQAMQP
111111110000000000000000000000000000000000000000000000000000000000000000000000000000
0000000000000000000000000000000000000000000000000000000
>Q8PDF7_3i0y_0_138
MSESNRQRATGLVQAYYEAFNRGDWDAMLAFLAEDVAHDLNQGPREIGRAAFASFLQRM
NDSYREQLRDIVVTANDEGTRVGAEYVVHGVYHTTDEGLPDANGQTYVLPGGAFFDVRD
GQITRVTNYYNLQEWIAQVSR
110000000000000000000000000000000000000000000000000000000000000000000000000000000000
0000000000000000000000000000000000000000000000000000000
>P24723_2fk9_0_137
MSSGTMKFNGYLRVRIGEAVGLQPTRWSLRHSLFKKGHQLLDPYLTVSVDQVRVGQTSTK
QKTNKPTYNEEFCANVTDGGHLELAVFHETPLGYDHFVANCTLQFQELLRTTGASDTFEG
WVDLEPEGKVFVVITLTG
111100000000000000000000000000000000000000000000000000000000000000000000000000000000
000000000000000000000000000000000000000000000000000001
>Q939U4_1h32_22_159
AEVAPGDVAIDGQGHVARPLTDAPGDPVEGRRLMTDRSVGNCIACHEVTEMADAQFPGT
VGPSLDGVAARYPEAMIRGILVNSKNVFPETVMPAYYRVEGFNRPGIAFTSKPIEGEIRPLMT
AGQIEDVVAYLMTLTQ
000000000000000000000000000000000000000000000000000011100000000000000000000000000000
000000000000000000000000000000000000000000000000000000
>Q9KCV1_3oa4_0_137
MAEKSNKLDHIGIAVTSIKDVLPFYVGSLKLKLLGMEDLPSQGVKIAFLEIGESKIELLEPLSE
ESPIAKFIQKRGEGIHHIAIGVKSIEERIQEVKENGVQMINDEPVPGARGAQVAFLHPRSARG
VLYEFCEKKEQ
111000000000000000000000000000000000000000000000000000000000000000000000000000000000
000000000000000000000000000000000000000000000000000000
>Q82T10_2pn0_0_136
MSIKPKIMISSLDAERLEILLETLSQNAFPGRDDLEAELARAEVVDPEEIPPTVVTMNSTVRFR
VESSAEEFCLTLVYPKDVDTSGEKISILAPVGSALLGLAQGDEIEWPKPGGGVLRVRIVEVTY

QPERSGEYYR
11100000000000000000000000000000000000000000000000000000000000000000000000000000000
000000000000000000000000000000000000000000001111111111
>Q01381_3lyn_18_153
RRWTFVRYHYINKAYEVTMKIQIISGFDRQLTAWLRVHGRRLTNNQKKTLFFVNRRYMQT
HWQNYMLWVKRKIKALGRPAAVGDYTRLGAEIGRRVDMVFFYNFLSGRKMIPPYSAYMA
KLNALRPADVPVKNHGK
11111111110000000000000000000000000000000000000000000000000000000000000000000000000
00000000000000000000000000000000000000000000000000011
>Q58625_2eif_0_131
MPGTKQVNVGSLKVGQYVMIDGVPCEIVDISVSKPGKHGGAKARVVGIGIFEKVKKEFVAP
TSSKVEVPIIDRRKGQVLAIMGDMVQIMDLQTYETLELPIPEGIEGLEPGGEVEYIEAVGQYKI
TRVIGGK
00000000000000000000000000000000000000000000000000000000000000000000000000000000000
0000000000000000000000000000000000000000000000111
>Q81DD6_3oms_0_134
MSNANQKITTFLMFEGKAEEAMNFYTSLFDQSEIVSISRYDENGPGKEGTVIHATFTLNGQE
FMCIDSYVNHNFTFTPAMSLYVTCETEEEIDTVFHKLAQDGAILMPLGSYPFSKKFGWLND
KYGVSWQLTLAE
11111000000000000000000000000000000000000000000000000000000000000000000000000000000
0000000000000000000000000000000000000000000000000001
>Q9BWU0_4h87_148_275
ARAPPYQEPPWGGPATAPYSLETLKGGTILGTRSLKGTSYCLFGRLSGCDVCLEHPSVSRYH
AVLQHRASGPDGECDSNGPGFYLYDLGSTHGTFLNKTRIPPRTYCRVHVGHVVRFGGSTRL
FILQG
00000000000000000000000000000000000000000000000000000000000000000000000001111110000
000000000000000000000000000000000000000000000
>Q8DU42_3l7t_0_133
MKLKAVHHVALIVSDYDKSYEFYVNQLGFEVIRENHRPKRHDYKLDLKCGDIELEIFGNKL
TDSNYCAPPERISWPREACGLRHLAFYVEDVEASRQELIALGIRVEEVRYDDYTGKKMAFFF
DPDGLPLELHE
10000000000000000000000000000000000000000000000000000000000000000000000000000000000
000000000000000000000000000000000000000000000000000
>Q8PBH4_2fuj_0_133
MSEHKILARVPISVRWRDMDSMGHVNNAKYISYLEEARVRWMLGVEGVAMTDRIAPVVA
ATNVNYKRPLVWPNDILVELFVERLGSSSVTIGHRILDQKDEGVLYSDGNVVVVWIDTQTG
KSASLPDAVRAASS
11110000000000000000000000000000000000000000000000000000000000000000000000000000000
000000000000000000000000000000000000000011111111111
>O67466_2egr_0_127
MPFIYRRRVQFYETDAQGIVHHSNYFRYFEEARGEFLRSKGFPYSKMRDMGLEVVLLNAYC
EYKKPLFYDDVFEVHLNLEELSRFTFTFSYIVFKEDIAVAKANTKHCMVKNGKIVSIPKEVLE
VLKD
10000000000000000000000000000000000000000000000000000000000000000000000000000000000
000000000000000000000000000000000000000000001
>Q9NQG5_4fu3_1_134
SSFSESALEKKLSELSNSQQSVQTLSLWLIHHRKHAGPIVSVWHRELRKAKSNRKLTFLYLA
NDVIQNSKRKGPEFTREFESVLVDAFSHVAREADEGCKKPLERLLNIWQERSVYGGEFIQQL
KLSMEDSKSP
00000000000000000000000000000000000000000000000000000000000000000000000000000000000
000000000000000000000000000000000000000000000111111

>P0A772_3n3a_0_132
MSQLVYFSSSSENTQRFIERLGLPAVRIPLNERERIQVDEPYILIVPSYGGGGTAGAVPRQVIRF
LNDEHNRALLRGVIASGNRNFGEAYGRAGDVIARKCGVPWLYRFELMGTQSDIENVRKGV
TEFWQRQP
1100000000000000000000000000000000000000000000000000000000000000000000000000000000
00000000000000000000000000000000000000000000000000
>Q2RRQ9_3fsd_0_132
MSNALATLASPADDIAFYEERLRAAMLTGDLKGLETLLADDLAFVDHTGCVKTKQTHLEP
YRAGLLKLSRLDLSDAVVRAAGEDGRVVVVRAVTAGVYDGEAFTETLRFTRIWRRTQGPA
GWKLVAGHCSVIL
1111111111110000000000000000000000000000000000000000000000000000000000000000000000
00000000000000000000000000000000000000000000000000
>Q3KJ04_4fbo_0_132
MSKYAVANQWGGSSAPWHPGGTWVLGARDNQNVVAIEIKSGDGGKSFTGTMTYAGEGPI
GFKAQRTGQNQYNVENQWGGNDAPWHPGGKWVIGGRDNQNVIALSVTSSDGGKNLSG
TNTYANEGPIGFRGQIE
1000000000000000000000000000000000000000000000000000000000000000000000000000000000
00000000000000000000000000000000000000000000000000
>B3VH91_2xw6_0_131
MRALALIAHDAKKEEMVAFCQRHREVLARFPLVATGTTGRRIEEATGLTVEKLLSGPLGGD
QQMGARVAEGRILAVIFFRDPLTAQPHEPDVQALLRVCDVHGVPLATNPMAAEALIPWLQ
SLVGYQTPQGQ
0000000000000000000000000000000000000000000000000000000000000000000000000000000000
0000000000000000000000000000000000000000001111111
>B9UIZ4_4iag_0_131
MAVLLSGVPVLAALDVSTTQKFWIEVLGFTEEFLTEDFGGVSRDGVELFICSVEDQVVPDNT
QAWLRVRDIDALHAEWSARVSSDYADASHPAMTAIREVPWGREFGLRDPAGNLVHFSELS
EAAETTRTVR
1000000000000000000000000000000000000000000000000000000000000000000000000000000000
0000000000000000000000000000000000000001111111111
>O31602_1z3e_0_130
MVTLYTSPSCTSCRKARAWLEEHEIPFVERNIFSEPLSIDEIKQILRMTEDGTDEIISTRSKVFQ
KLNVNVESMPLQDLYRLINEHPGLLRRPIIIDEKRLQVGYNEDEIRRFLPRKVRSFQLREAQR
LAN
0000000000000000000000000000000000000000000000000000000000000000000000000000000000
000000000000000000000000000000000001111111111111
>P07445_3rgr_0_130
MNLPTAQEVQGLMARYIELVDVGDIEAIVQMYADDATVEDPFGQPPIHGREQIAAFYRQG
LGGGKVRACLTGPVRASHNGCGAMPFRVEMVWNGQPCALDVIDVMRFDEHGRIQTMQ
AYWSEVNLSVREPQ
0000000000000000000000000000000000000000000000000000000000000000000000000000000000
000000000000000000000000000000000000000000000111
>Q7TS55_2q8o_42_172
SLKPTAIESCMVKFELSSSKWHMTSPKPHCVNTTSDGKLKILQSGTYLIYGQVIPVDKKYIKD
NAPFVVQIYKKNDVLQTLMNDFQILPIGGVYELHAGDNIYLKFNSKDHIQKTNTYWGIILM
PDLPFIS
1111110000000000000000000000000000000000000000000000000000000000000000000000000000
000000000000000000000000000000000000000000000000
>P31948_1elr_222_351
KQALKEKELGNDAYKKKDFDTALKHYDKAKELDPTNMTYITNQAAVYFEKGDYNKCREL
CEKAIEVGRENREDYRQIAKAYARIGNSYFKEEKYKDAIHFYNKSLAEHRTPDVLKKCQQA

EKILKEQERL
00000000000000000000000000000000000000000000000000000000000000000000000000000000000
00000000000000000000000000000000000000000000111
>Q8DHA2_3zsu_24_151
GGPSATTPPPPTYSELQITRIQDYLRDIEKNAERFADLEVSVAKGDWQEARNIMRGPLGEML
MDMRALNRNLLAKDQPTPTALTRALTDDFLKIDQGADLDSVTVAQEGFREAEADFKAYL
NSLPELS
11111111100000000000000000000000000000000000000000000000000000000000000000000000000
000000000000000000000000000000000000000000001
>Q5CQI4_1zo2_0_128
MDQSINLNPQFDQIGKQFVQHYYQTFQTNRPALGGLYGPQSMLTWEDTQFQGQANIVNK
FNSLNFQRVQFEITRVDCQPSPNNGSIVFVTGDVRIDDGQPLKFSQVFNLMPSGNGGFMIFN
DLFRLNLG
11100000000000000000000000000000000000000000000000000000000000000000000000000000000
0000000000000000000000000000000000000000000011
>O60885_2ouo_332_459
KDVPDSQQHPAPEKSSKVSEQLKCCSGILKEMFAKKHAAYAWPFYKPVDVEALGLHDYCD
IIKHPMDMSTIKSKLEAREYRDAQEFGADVRLMFSNCYKYNPPDHEVVAMARKLQDVFEM
RFAKMPDE
11111111111111110000000000000000000000000000000000000000000000000000000000000000000
000000000000000000000000000000000000000000001
>P0A595_2qrw_0_127
MPKSFYDAVGGAKTFDAIVSRFYAQVAEDEVLRRVYPEDDLAGAEERLRMFLEQYWGGPR
TYSEQRGHPRLRMRHAPFRISLIERDAWLRCMHTAVASIDSETLDDEHRRELLDYLEMAAH
SLVNSPF
10000000000000000000000000000000000000000000000000000000000000000000000000000000000
000000000000000000000000000000000000000000000
>Q0INY7_1wdd_47_174
MQVWPIEGIKKFETLSYLPPLTVEDLLKQIEYLLRSKWVPCLEFSKVGFVYRENHRSPGYYD
GRYWTMWKLPMFGCTDATQVLKELEEAKKAYPDAFVRIIGFDNVRQVQLISFIAYKPPGCE
ESGGN
00000000000000000000000000000000000000000000000000000000000000000000000000000000000
000000000000000000000000000000000000000111111
>P19080_1fnj_0_126
MMIRGIRGATTVERDTEEEILQKTKQLLEKIIEENHTKPEDVVQMLLSATPDLHAVFPAKAV
RELSGWQYVPVTCMQEMDVTGGLKKCIRVMMTVQTDVPQDQIRHVYLEKVVVLRPDLSL
TKNTEL
10000000000000000000000000000000000000000000000000000000000000000000000000000000000
00000000000000000000000000000000001111111111
>Q46495_1vzi_0_125
MPERLQVYKCEVCGNIVEVLNGGIGELVCCNQDMKLMSENTVDAAKEKHVPVIEKIDGG
YKVKVGAVAHPMEEKHYIQWIELLADDKCYTQFLKPGQAPEAVFLIEAAKVVAREYCNIH
GHWKAEN
10000000000000000000000000000000000000000000000000000000000000000000000000000000000
0000000000000000000000000000000000000000000
>Q931P4_2z6f_538_663
PTNDQLTDLQEAHFVVFESEENSESVMDGFVEHPFYTATLNGQKYVVMKTKDDSYWKDLI
VEGKRVTTVSKDPKNNSRTLIFPYIPDKAVYNAIVKVVVANIGYEGQYHVRIINQDINTKDD
DTSQ
11111000000000000000000000000000000000000000000000000000000000000000000000000000000
0000000000000000000000000000000000111111111

>A5K893_2fo3_65_189
KYNMGNANYRIQKELHNFLNNPPINCTLDVHPNNIRIWIVKYVGLENTIYANEVYKLKIIF
PDDYPLKPPIVYFLQKPPKHTHVYSNGDICLSLLGDDYNPSLSISGLVLSIISMLSSAKEKKLP
11111111000000000000000000000000000000000000000000000000000000000000000000000000000
000000000000000000000000000000000011111111
>P41182_1r29_4_128
ADSCIQFTRHASDVLLNLNRLRSRDILTDVVIVVSREQFRAHKTVLMACSGLFYSIFTDQLK
CNLSVINLDPEINPEGFCILLDFMYTSRLNLREGNIMAVMATAMYLQMEHVVDTCRKFIKA
SE
11000000000000000000000000000000000000000000000000000000000000000000000000000000000
000000000000000000000000000000000000000001
>P06909_2yby_321_443
LKPCEFPQFKYGRLYYEESLRPNFPVSIGNKYSYKCDNGFSPPSGYSWDYLRCTAQGWEPEV
PCVRKCVFHYVENGDSAYWEKVYVQGQSLKVQCYNGYSLQNGQDTMTCTENGWSPPPK
CIR
00000000000000000000000000000000000000000000000000000000000000000000000000000000000
0000000000000000000000000000000000000001
>Q63042_1oqc_73_197
MRTQQKRDIKFREDCPQDREELGRNTWAFLHTLAAYYPDMPTPEQQQDMAQFIHIFSKFY
PCEECAEDIRKRIDRSQPDTSTRVSFSQWLCRLHNEVNRKLGKPDFDCSRVDERWRDGWK
DGSCD
11111111111100000000000000000000000000000000000000000000000000000000000000000000000
000000000000000000000000000000000000000001
>Q8WRW5_3bjh_25_143
APDWVPPEVFDLVAEDKARCMSEHGTTQAQIDDVDKGNLVNEPSITCYMYCLLEAFSLVD
DEANVDEDIMLGLLPDQLQERAQSVMGKCLPTSGSDNCNKIYNLAKCVQESAPDVWFVI
11000000000000000000000000000000000000000000000000000000000000000000000000000000000
000000000000000000000000000000000000
>O16259_4gco_130_252
RLAYINPELAQEEKNKGNEYFKKGDYPTAMRHYNEAVKRDPENAILYSNRAACLTKLMEF
QRALDDCDTCIRLDSKFIKGYIRKAACLVAMREWSKAQRAYEDALQVDPSNEEAREGVRN
CLR
11100000000000000000000000000000000000000000000000000000000000000000000000000000000
0000000000000000000000000000000000000000
>Q6ZUM4_3pp2_490_612
AVRTKTLDKAGVLHRTKTADKGKRLRKKHWSASWTVLEGGVLTFFKDSKTSAAGGLRQPS
KFSTPEYTVELRGATLSWAPKDKSSRKNVLELRSRDGSEYLIQHDSEAIISTWHKAIAQGIQE
11111100000000000000000000000000000000000000000011100000000000000000000000000000000
0000000000000000000000000000000000000001
>Q035U5_2i8d_0_121
MGSLAEWYQRIPTPDDLTRVESLFANMQAQFPQLKLEFKWNQPMFTDHGTFIMGFNPSKK
HLAVAIEPQTMTRFIPQIDKAGYDHSQIIRFPWHKPLDEQLIHDLIAYTIDQKKDATTFWQR
10000000000000000000000000000000000000000000000000000000000000000000000000000000000
000000000000000000000000000000000000000
>P17724_3aq6_0_120
MNKPQTIYEKLGGENAMKAAVPLFYKKVLADERVKHFFKNTDMDHQTKQQTDFLTMLL
GGPNHYKGKNMTEAHKGMNLQNLHFDAIIENLAATLKELGVTDAVINEAAKVIEHTRKD
MLGK
11110000000000000000000000000000000000000000000000000000000000000000000000000000000
00000000000000000000000000000000000000
>Q02784_3gx8_29_149

LSTEIRKAIEDAIESAPVVLFMKGTPEFPKCGFSRATIGLLGNQGVDPAKFAAYNVLEDPELR
EGIKEFSEWPTIPQLYVNKEFIGGCDVITSMARSGELADLLEEAQALVPEEEEETKDR
1000000000000000000000000000000000000000000000000000000000000000000000000000000000000000000000
00000000000000000000000000000111111111
>A1RHE3_3myf_804_922
FDLHTLNWDLCLTQANHKSNLALEMLKMLLDSLPETVEKIQTALGQNDQATMLSTIHKL
HGASCYCGVPTTQRLCQEIESALKRQTPVEDLEPEILELLDELTKVESAVKQVLSQLSAE
1000000000000000000000000000000000000000000000000000000000000000000000000000000000000000000000
000000000000000000000000000000000011
>Q5PSV9_3va1_28_138
EPIGQLRLFSGTHGPERDFPLYLGKNVVGRSPDCSVALPFPSISKQHAVIEISAWNKAPILQD
CGSLNGTQIVKPPRVLPPGVSHRLRDQELILFADFPCQYHRLDVPPPL
0000000000000000000000000000000000000000000000000000000000000000000000000000000000000000000000
000000000000000000000001111
>Q9JZJ4_3kjj_0_119
MDIRYFGTTPRYSEAVGANGLIFLSGMVPENGETAAEQTADVLAQIDRWLAECGSDKAHV
LDAVIYLRDMGDYAEMNGVWDAWVAAGRTPARACVEARLARPEWRVEIKITAVKRDAAT
A
0000000000000000000000000000000000000000000000000000000000000000000000000000000000000000000000
0000000000000000000000000000000000111
>Q9NP99_1smo_20_138
ATKLTEEKYELKEGQTLDVKCDYTLEKFASSQKAWQIIRDGEMPKTLACTERPSKNSHPVQ
VGRIILEDYHDHGLLRVRMVNLQVEDSGLYQCVIYQPPKEPHMLFDRIRLVVTKGFSG
0000000000000000000000000000000000000000000000000000000000000000000000000000000000000000000000
000000000000000000000000000000001111
>Q7XZK3_2vm1_0_117
MAAEEGAVIACHTKQEFDTHMANGKDTGKLVIIDFTASWCGPCRVIAPVFAEYAKKFPGAI
FLKVDVDELKDVAEAYNVEAMPTFLFIKDGEKVDSVVGGRKDDIHTKIVALMGSAST
1111000000000000000000000000000000000000000000000000000000000000000000000000000000000000000000
00000000000000000000000000000000111
>Q9HCM2_4e74_1487_1602
DKLIRQQIDYKTLVLSCVSPDNANSPEVPVKILNCDTITQVKEKILDAIFKNVPCSHRPKAA
DMDLEWRQGSGARMILQDEDITTKIENDWKRLNTLAHYQVPDGSVVALVSKQVT
1111111100000000000011110000000000000000000000000000000000000000000000000000000000000000000000
000011100000000000000000000000000
>P12643_2h64_282_395
QAKHKQRKRLKSSCKRHPLYVDFSDVGWNDWIVAPPGYHAFYCHGECPFPLADHLNSTN
HAIVQTLVNSVNSKIPKACCVPTELSAISMLYLDENEKVVLKNYQDMVVEGCGCR
1111111110000000000000000000000000000000000000000000000000000000000000000000000000000000000000
0000000000000000000000000000000
>A6T926_3qva_0_107
MSTLSTHILDISTGTPAEGVTVSLSREGETLANLVTNAQGRIATFSAAPLPAGRYCLTAETGA
WFARAGRESVFTRAQIDFVIGEAAEDHFHLPFLIAPGGWSTYRGS
0000000000000000000000000000000000000000000000000000000000000000000000000000000000000000000000
0111000000000000000000000
>Q97QU3_2idl_0_113
MIQAVFERAEDGELRSAEITGHAESGEYGLDVVCASVSTLAINFINSIEKFAGYEPILELNEDE
GGYLMVEIPKDLPSHQREMTQLFFESFFLGMANLSENYSEFVQTRVITEN
0000000000000000000000000000000000000000000000000000000000000000000000000000000000000000000000
0000000000000000000000000000001
>A3DHB8_1ybx_0_112

MAKGGFPGFGGNINNLVKQAQKMQRDMERVQEELKEKTVEASAGGGAVTVVATGRKDI
KEITIKPEVVDPDDVEMLQDLILAAVNEALRKADEMVTAEISKITGGLGGIPGLF
1111111111100000000000000000000000000000000000000000000000000000000000000000000000
000000000000000000001111111111
>A9CI62_2q2i_0_112
MSGEISYADFEKVDIRVGTIVEAVPFPEARKPAIKVKIDFGPEIGIKKSSAQITVHYTPESLVGR
QVLGVVNFPPRQIGPFRSEVLTLGFADANGDIVLAAVERPVPNGEKMC
1000000000000000000000000000000000000000000000000000000000000000000000000000000000
000000000000000000000000000000
>O04350_3mxz_0_112
MATIRNLKIKTSTCKRIVKELHSYEKEVEREAAKTADMKDKGADPYDLKQQENVLGESRM
MIPDCHKRLESALADLKSTLAELEETDEKEGPEIEDAKKTVADVEKQFPTEDA
0000000000000000000000000000000000000000000000000000000000000000000000000000000000
000100000000000000000000111111
>Q3M7V9_2nwv_0_112
MDNVAEYRKLIKQVLTEYDNLSRQSPETNYETCLVFDENHDNYLWLAVDWQGSKRIKYTY
VHIRIKNEKIYIEEDYTEEGIATELMRLGVTNNDIVLAFHPPDVRKFTDFATA
1000000000000000000000000000000000000000000000000000000000000000000000000000000000
000000000000000000000000000000
>Q76CS9_3ah7_0_112
MPLVTFLPHEKFCPEGLTVEVKPGTNILELAHDHHIEMESACGGVKACTTCHCIVRKGFDS
LEEADELEEDMLDKAWGLEAQSRLGCQVFVADEDLTIEIPKYSLNHAAEAPH
0000000000000000000000000000000000000000000000000000000000000000000000000000000000
000000000000000000000000001111
>Q16665_4h6j_237_347
DSKTFLSRHSLDMKFSYCDERITELMGYEPEELLGRSIYEYYHALDSDHLTKTHHDMFTKGQ
VTTGQYRMLAKRGGYVWVETQATVIYNTKNSQPQCIVCVNYVVSGIIQH
0000000000000000000000000000000000000000000000000000000000000000000000000000000000
0000000000000000000000011111
>Q8ZMZ1_4dy5_22_132
TGKNVNVEFDKGQNSARYSGVIKGYDYDTYNFQARKGQKVHVSISNEGADTYLFGPGISD
SVDLSRYSSELDGNGQYTLPASGKYELKVLQTRNEARKNKAKKYSVNIQIK
1000000000110000000000000000000000000000000000000000000000000000000000000000000000
0000000000000000000000000000
>Q8NX62_1xbw_0_106
MKFMAENRLTLTKGTAKDIIERFYTRHGIETLEGFDGMFVTQTLEQEDFDEVKILTVWKSKQ
AFTDWLKSDVFKAAHKHVRSKNEDESSPIINNKVITYDIGYSYMK
0000000000000000000000000000000000000000000000000000000000000000000000000000010111
111110000000000000000000
>Q9NHA8_3ie4_25_131
YEVPKAKIDVFYPKGFEVSIPDEEGITLFAFHGKLNEEMEGLEAGTWARDIVKAKNGRWTF
RDRITALKPGDTLYYWTYVIYNGLGYREDDGSFVVNGYSGNNASPH
0000000000000000000000000000000000000000000000000000000000000000000000000000000000
000000000000000000111111
>O06156_3hx9_0_104
MPVVKINAIEVPAGAGPELEKRFAHRAHAVENSPGFLGFQLLRPVKGEERYFVVTHWESDE
AFQAWANGPAIAAHAGHRANPVATGASLLEFEVVLDVGGTGKTA
1000000000000000000000000000000000000000000000000000000000000000000000000000000000
0000000000000000000111
>P71012_2r4q_170_272
KILAVTACPTGIAHTFMAADALKEKAKELGVEIKVETNGSSGIKHKLTAQEIEDAPAIIVAAD

KQVEMERFKGKRVLQVPVTAGIRRPQELIEKAMNQDAPIY
00000000000110000000000000000000000000000000000000000000000000000000000000000000000
00000000000000000000
>Q9KFX8_4es3_0_95
MLVLITYDVQTSSMGGTKRLRKVAKACQNYGQRVQNSVFECIVDSTQLTSLKLELTSLIDEE
KDSLRIYRLGNNYKTKVEHIGAKPSIDLEDPLIF
00000000000000000000000000000000000000000000000000000000000000000000001100110000000
0001111111111
>Q9KA76_3on1_0_100
MSEAKWLSLLGLAARARQLLTGEEQVVKAVQNGQVTLVILSSDAGIHTKKKLLDKCGSYQI
PVKVVGNRQMLGRAIGKHERVVIGVKDAGFSRKLAALIDE
11000000000000000000000000000000000000000000000000000000000000000000000000000000000
000000000000000000
>P0A676_2fvh_0_99
MRLTPHEQERLLLSYAAELARRRRARGLRLNHPEAIAVIADHILEGARDGRTVAELMASGR
EVLGRDDVMEGVPEMLAEVQEATFPDGTKLVTVHQPIA
10000000000000000000000000000000000000000000000000000000000000000000000000000000000
00000000000000000
>Q836C2_2hwv_134_233
TIGDLTIHPDAYMVSKRGEKIELTHREFELLYYLAKHIGQVMTREHLLQTVWGYDYFGDVR
TVDVTVRRLREKIEDSPSHPTYLVTRRGVGYYLRNPEQE
00000000000000000000000000000000000000000000000000000000000000000000000000000000000
00000000000000011
>P07311_2vh7_0_98
MAEGNTLISVDYEIFGKVQGVFFRKHTQAEGKKLGLVGWVQNTDRGTVQGQLQGPISKVR
HMQEWLETRGSPKSHIDKANFNNEKVILKLDYSDFQIVK
11111000000000000000000000000000000000000000000000000000000000000000000000000000000
0000000000000000
>P71376_1jo0_1_98
TTLSTKQKQFLKGLAHHLNPVVMLGGNGLTEGVLAEIENALNHHELIKVKVAGADRETK
QLIINAIVRETKAAQVQTIGHILVLYRPSEEAKIQLPRK
00000000000000000000000000000000000000000000000000000000000000000000000000000000000
000000000000001
>A4IKE4_3zbh_0_96
MAGVIRLTPEELRGVARQYNVESSNVTELIARLDQMSHTLQGIWEGASSEAFIQQYQELRPS
FEKMAVLLNEVGQQLHNSATILEDTDQQIASQIRG
11100000000000000000000000000000000000000000000000000000000000000000000000000000000
00000000000001
>Q53752_3dgy_66_162
ADPALADVCRTKLPSQAQDTLALIAKNGPYPYNRDGVVFENRESRLPKKGNGYYHEFTVV
TPGSNDRGTRRVVTGGYGEQYWSPDHYATFQEIDPRC
11100000000000000000000000000000000000000000000000000000000000011000000000000000000
00000000000000
>P43214_2vxq_26_121
VPKVTFTVEKGSNEKHLAVLVKYEGDTMAEVELREHGSDEWVAMTKGEGGVWTFDSEEP
LQGPFNFRFLTEKGMKNVFDDVVPEKYTIGATYAPEE
11000000000000000000000000000000000000000000000000000000000000000000000000000000000
0000000000011
>P36206_1riy_2_89
KKELIDRVAKKAGAKKKDVKLILDTILETITEALAKGEKVQIVGFGSFEVRKAAARKGVNP
QTRKPITIPERKVPKFKPGKALKEKVK

00000000000000000000000000000000000000000000000000001111111111111111111000000000000
00000
>P00171_1cyo_5_97
SKAVKYYTLEEIQKHNNSKSTWLILHYKVYDLTKFLEEHPGGEEVLREQAGGDATENFEDV
GHSTDARELSKTFIIGELHPDDRSKITKPSES
00000000000000000000000000000000000000000000000000000000000000000000000000000000000
0000011111
>P02751_3cal_92_181
AEETCFDKYTGNTYRVGDTYERPKDSMIWDCTCIGAGRGRISCTIANRCHEGGQSYKIGDT
WRRPHETGGYMLECVCLGNGKGEWTCKPI
10000000000000000000000000000000000000000000000000000000000000000001000000000000000
0000000
>P25208_1n1j_50_142
SFREQDIYLPIANVARIMKNAIPQTGKIAKDAKECVQECVSEFISFITSEASERCHQEKRKTIN
GEDILFAMSTLGFDSYVEPLKLYLQKFRE
11111100000000000000000000000000000000000000000000000000000000000000000000000000000
0000000000
>P49791_3gj7_657_749
AGSSWQCDTCLLQNKVTDNKCIACQAAKLPLKETAKQTGIGTPSKSDKPASTSGTGFGDKF
KPAIGTWDCDTCLVQNKPEAVKCVACETPKPG
11111111111111111111111111111111111111111111111111111111111111111000000000000000000
0000000001
>Q72LN8_2d8d_0_89
MDERIQALRKEVDRVNREILRLLSERGRLVQEIGRLQTELGLPHYDPKREEEMLAYLTAENP
GPFPDETIRKLFKEIFKASLDLEERQDQ
11000000000000000000000000000000000000000000000000000000000000000000000000000000000
0011111
>Q8XXK6_2bt9_1_90
SSVQTAATSWGTVPSIRVYTANNGKITERCWDGKGWYTGAFNEPGDNVSVTSWLVGSAIHI
RVYASSGTTTTEWCWDGNGWTKGAYTSTN
00000000000000000000000000000000000000000000000000000000000000000000000000000000000
0000001
>P08046_1a1g_332_420
ERPYACPVESCDRRFSRSDELTRHIRIHTGQKPFQCRICMRNFSRSDHLTTHIRTHTGEKPFA
CDICGRKFARSDERKRHTKIHLRQKD
10000000000000000000000000000000000000000000000000000000000000000000000000000000000
001111
>Q6ZN17_4a4i_23_110
ESQVLRGTGHCKWFNVRMGFGFISMINREGSPLDIPVDVFVHQSKLFMEGFRSLKEGEPVE
FTFKKSSKGLESIRVTGPGGSPCLGSE
11100000000000000000000000000000000000000000000000000000000000000000000000000000000
00000
>Q975W5_2zbc_0_82
MASAIVLINTDAGGEDEVFERLKSMSEVTEVHVVYGVYDIVVKVEADSMDKLKDFVTNTIR
KLPKVRSTLTMIIVEGKSLVKK
10000000000000000000000000000000000000000000000000000000000000000000000000000000001
>P31483_3bs9_104_185
HFHVFVGDLSPEITTEDIKAAFAPFGRISDARVVKDMATGKSKGYGFVSFFNKWDAENAIQ
QMGGQWLGGRQIRTNWATRKP
0000000000000000000000000000000000000000000000000000000000000000000000000000000111
>P00208_3eun_1_82

ALMITDECINCDVCEPECPNGAISQGDETYVIEPSLCTECVGHYETSQCVEVCPVDCIIKDPS
HEETEDELRAKYERITGEG
0000000000000000000000000000000000000000000000000000000000000000000000000000000001
>P0AC65_1h75_0_80
MRITIYTRNDCVQCHATKRAMENRGFDFEMINVDRVPEAAEALRAQGFRQLPVVIAGDLS
WSGFRPDMINRLHPAPHAASA
000000000000000000000000000000000000000000000000000000000000000000000000000011111
>Q15843_4f8c_0_80
MLIKVKTLTGKEIEIDIEPTDKVERIKERVEEKEGIPPQQQRLIYSGKQMNDEKTAADYKILG
GSVLHLVLALRGGGGLRQ
000000000000000000000000000000000000000000000000000000000000000000000000001111111
>Q8EF93_3d3r_0_79
MCLSIPSQVVAVDNERQSVTVDTLGVRRDVSSHLMTEPLAIGDYVLIHIGFVMNKIDRNDA
LQSLELYQEIVSKLENETT
00000000000000000000000000000000000000000000000000000000000000000000000000001111
>P38540_1g72_22_90
YDGQNCKEPGNCWENKPGYPEKIAGSKYDPKHDPVELNKQEESIKAMDARNAKRIANAK
SSGNFVFDVK
000000000000000000000000000000000000000000000000000000001111111111111
>Q9X6J6_2zcz_0_73
MYTNSDFVVIKALEDGVNVIGLTRGADTRFHHSEKLDKGEVLIAQFTEHTSAIKVRGKAYIQ
TRHGVIESEGKK
11110000000000000000000000000000000000000000000000000000000000000000000011
>P21859_1j2l_0_69
GEECDCGSPSNPCCDAATCKLRPGAQCADGLCCDQCRFKKKRTICRIARGDFPDDRCTGQ
SADCPRWNGL
0000000000000000000000000000000000000000000000000000000000000000000011
>Q9S9F3_3rdy_0_68
LRQCSGKQEWPELVGERGSKAAKIIENENEDVRAIVLPEGSAVPRDLRCDRVWVFVDERGV
VVDTPVVM
110000000000000000000000000000000000000000000000000000000000000000000
>O35964_3c0c_302_367
MPPLDQPSCKALYDFEPENDGELGFREGDLITLTNQIDENWYEGMLHGQSGFFPLSYVQVL
VPLPQ
110000000000000000000000000000000000000000000000000000000000000000
>P13123_1wtv_0_65
MVKVKFKYKGEEKEVDTSKIKKVWRVGKMVSFTYDDNGKTGRGAVSEKDAPKELLDMLA
RAEREKK
100000000000000000000000000000000000000000000000000000000000000001
>O43763_3a03_161_215
TSFSRSQVLELERRFLRQKYLASAERAALAKALRMTDAQVKTWFQNRRTKWRRQT
1100000000000000000000000000000000000000000000000000000
>O89100_1oeb_264_321
VRWARALYDFEALEEDELGFRSGEVVEVLDSSNPSWWTGRLHNKLGLFPANYVAPMMR
0000000000000000000000000000000000000000000000000000000001
>P09152_1q16_0_1246
MSKFLDRFRYFKQKGETFADGHGQLLNTNRDWEDGYRQRWQHDKIVRSTHGVNCTGSCS
WKIYVKNGLVTWETQQTDYPRTRPDLPNHEPRGCPRGASYSWYLYSANRLKYPMMRKRL
MKMWREAKALHSDPVEAWASIIEDADKAKSFKQARGRGGFVRSSWQEVNELIAASNVYTI
KNYGPDRVAGFSPIPAMSMVSYASGARYLSLIGGTCLSFYDWYCDLPPASPQTWGEQTDVP
ESADWYNSSYIIAWGSNVPQTRTPDAHFFTEVRYKGTKTVAVTPDYAEIAKLCDLWLAPKQ

GTDAAMALAMGHVMLREFHLDNPSQYFTDYVRRYTDMPMLVMLEERDGYYAAGRMLR
AADLVDALGQENNPEWKTVAFNTNGEMVAPNGSIGFRWGEKGKWNLEQRDGKTGEETE
LQLSLLGSQDEIAEVGFPYFGGDGTEHFNKVELENVLLHKLPVKRLQLADGSTALVTTVYD
LTLANYGLERGLNDVNCATSYDDVKAYTPAWAEQITGVSRSQIIRIAREFADNADKTHGRS
MIIVGAGLNHWYHLDMNYRGLINMLIFCGCVGQSGGGWAHYVGQEKLRPQTGWQPLAF
ALDWQRPARHMNSTSYFYNHSSQWRYETVTAEELLSPMADKSRYTGHLIDFNVRAERMG
WLPSAPQLGTNPLTIAGEAEKAGMNPVDYTVKSLKEGSIRFAAEQPENGKNHPRNLFIWRS
NLLGSSGKGHEFMLKYLLGTEHGIQGKDLGQQGGVKPEEVDWQDNGLEGKLDLVVTLDF
RLSSTCLYSDIILPTATWYEKDDMNTSDMHPFIHPLSAAVDPAWEAKSDWEIYKAIAKKFSE
VCVGHLGKETDIVTLPIQHDSAAELAQPLDVKDWKKGECDLIPGKTAPHIMVVERDYPAT
YERFTSIGPLMEKIGNGGKGIAWNTQSEMDLLRKLNYTKAEGPAKGQPMLNTAIDAAEMI
LTLAPETNGQVAVKAWAALSEFTGRDHTHLALNKEDEKIRFRDIQAQPRKIISSPTWSGLED
EHVSYNAGYTNVHELIPWRTLSGRQQLYQDHQWMRDFGESLLVYRPPIDTRSVKEVIGQKS
NGNQEKALNFLTPHQKWGIHSTYSDNLLMLTLGRGGPVVWLSEADAKDLGIADNDWIEV
FNSNGALTARAVVSQRVPAGMTMMYHAQERIVNLPGSEITQQRGGIHNSVTRITPKPTHMI
GGYAHLAYGFNYYGTVGSNRDEFVVVRKMKNIDWLDGEGNDQVQESVK
10000000000000000000000000000000000000000000000000000000000000000000000000000000
00000000000000000000000000000000000000000000000000000000000000000000000000000000
00000000000000000000000000000000000000000000000000000000000000000000000000000000
00000000000000000000000000000000000000000000000000000000000000000000000000000000
00000000000000000000000000000000000000000000000000000000000000000000000000000000
00000000000000000000000000000000000000000000000000000000000000000000000000000000
00000000000000000000000000000000000000000000000000000000000000000000000000000000
00000000000000000000000000000000000000000000000000000000000000000000000000000000
00000000000000000000000000000000000000000000000000000000000000000000000000000000
00000000000000000000000000000000000000000000000000000000000000000000000000000000
00000000000000000000000000000000000000000000000000000000000000000000000000000000
00000000000000000000000000000000000000000000000000000000000000000000000000000000
00000000000000000000000000000000000000000000000000000000000000000000000000000000
00000000000000000000000000000000000000000000000000000000000000000000000000000000
00000000000000000000000000000000000000000000000000000000000000000000000000000000
11
>Q76NL8_3s5m_0_1192
MNLTKLMKVIGYINIITNCVQSFTNRADKKRYNVFAKSFINTINTNLYTFKAVMSKTPEWIH
EKSPKHNSYDIIEKRYNEEFKMTYTVVYQHKKAKTQVISLGTNDPLDVEQAFAFYVKTLTHS
GKGIPHILEHSVLSGSKNYNYKNSIGLLEKGTLHTHLNAYTFNDRTVYMAGSMNNKDFFNI
MGVYMDSVFQPNVLENKYIFETEGWTYEVEKLKEDEKGKAEIPQMKDYKVSFNGIVYNEM
KGALSSPLEDLYHEEMKYMFPDNVHSNNSGGDPKEITNLTYEEFKEFYYKNYNPKKVKVFF
FSKNNPTELLNFVDQYLGQLDYSKYRDDAVESVEYQTYKKGPFYIKKKYGDHSEEKENLVS
VAWLLNPKVDKTNNHNNNHSNNQSSENNGYSNGSHSSDLSLENPTDYFVLLIINNLLIHT
PESVLYKALTDCGLGNNVIDRGLNDSLVQYIFSIGLKGIKRNNEKIKNFDKVHYEVEDVIMN
ALKKVVKEGFNKSAVEASINNIEFILKEANLKTSKSIDFVFEMTSKLNYNRDPLLIFEFEKYL
NIVKNKIKNEPMYLEKFVEKHFINNAHRSVILLEGDENYAQEQENLEKQELKKRIENFNEQ
EKEQVIKNFEELSKYKNAEESPEHLNKFPIISISDLNKKTLEVPVNVYFTNINENNNIMETYN
KLKTNEHMLKDNMDVFLKKYVLKNDKHNTNNNNNNNNNMDYSFTETKYEGNVPILVY
EMPTTGIVYLQFVFSLDHLTVDELAYLNLFKTLILENKTNKRSSEDFVILREKNIGSMSANVA
LYSKDDHLNVTDKYNAQALFNLEMHVLSHKCNDALNIALEAVKESDFSNKKKVIDILKRK
INGMKTTFSEKGYAILMKYVKAHLNSKHYAHNIIYGYENYLKLQEQLELAENDFKTLENIL
VRIRNKIFNKKNLMVSVTSDYGALKHLFVNSNESLKNLVSYFEENDKYINDMQNKVNDPT
VMGWNEEIKSKKLFDEEKVKKEFFVLPTFVNSVSMSGILFKPGEYLDPSFTVIVAALKNSYL
WDTVRGLNGAYGVFADIEYDGSVVFLSARDPNLEKTLATFRESAKGLRKMADTMTENDLL

RYIINTIGTIDKPRRGIELSKLSFLRLISNESEQDRVEFRKRIMNTKKEDFYKFADLLESKVNEF
EKNIVIITTKEKANEYIANVDGEFKKVLIE
11111111111111111111111111111111111111111111111111111111000000000000000000000000000
00000000000000000000000000000000000000000000000000000000000000000000000000000000000
00000000000000000000000000000000000000000000000000000000000000000000000000000000000
00000000000000000000000000000000000000000000000000000000000000000000000000000000000
00000000000000000000000000000000000000000000111111111111111111111100000000000000000
00000000000000000000000000000000000000000000000000000000000000000000000000000000000
00000000000000000000000000000000000000000000000000000000000000000000000000000000000
00000000000000000000000000000000000000000000000000000000000000000000000000000000000
00000000000000000000000000000000001111111111111111111110000000000000000000000000000
00000000000000000000000000000000000000000000000000000000000000000000000000000000000
00000000000000000000000000000000000000000000000000000000000000000000000000000000000
00000000000000000000000000000000000000000000000000000000000000000000000000000000000
00000000000000000000000000000000000000000000000000000000000000000000000000000000000
00000000000000000000000000000000000000000000000000000000000000000000000000000000000
0000000000000000000000000000000
>Q8G9Q2_3ttq_1758_2834
QAGHYITKNGNDWQYDTNGELAKGLRQDSNGKLRYFDLTTGIQAKGQFVTIGQETYYFSK
DHGDAQLLPMVTEGHYGTITLKQGQDTKTAWVYRDQNNTILKGLQNINGTLQFFDPYTG
EQLKGGVAKYDDKLFYFESGKGNLVSTVAGDYQDGHYISQDGQTRYADKQNQLVKGLVT
VNGALQYFDNATGNQIKNQQVIVDGKTYYFDDKGNGEYLFTNTLDMSTNAFSTKNVAFN
HDSSSFDHTVDGFLTADTWYRPKSILANGTTWRDSTDKDMRPLITVWWPNKNVQVNYLN
FMKANGLLTTAAQYTLHSDQYDLNQAAQDVQVAIERRIASEHGTDWLQKLLFESQNNNP
SFVKQQFIWNKDSEYHGGGDAWFQGGYLKYGNNPLTPTTNSDYRQPGNAFDFLLANDVD
NSNPVVQAENLNWLHYLMNFGTITAGQDDANFDSIRIDAVDFIHNDTIQRTYDYLRDAYQ
VQQSEAKANQHISLVEAGLDAGTSTIHNDALIESNLREAATLSLTNEPGKNKPLTNMLQDV
DGGTLITDHTQNSTENQATPNYSIIHAHDKGVQEKVGAAITDATGADWTNFTDEQLKAGL
ELFYKDQRATNKKYNSYNIPSIYALMLTNKDTVPRMYYGDMYQDDGQYMANKSIYYDAL
VSLMTARKSYVSGGQTMSVDNHGLLKSVRFGKDAMTANDLGTSATRTEGLGVIIGNDPKL
QLNDSDKVTLDMGAAHKNQKYRAVILTTRDGLATFNSDQAPTAWTNDQGTLTFSNQEIN
GQDNTQIRGVANPQVSGYLAVWVPVGASDNQDARTAATTTENHDGKVLHSNAALDSNL
IYEGFSNFQPKATTHDELTNVVIAKNADVFNNWGITSFEMAPQYRSSGDHTFLDSTIDNGY
AFTDRYDLGFNTPTKYGTDGDLRATIQALHHANMQVMADVVDNQVYNLPGKEVVSATR
AGVYGNDDATGFGTQLYVTNSVGGGQYQEKYAGQYLEALKAKYPDLFEGKAYDYWYKN
YANDGSNPYYTLSHGDRESIPADVAIKQWSAKYMNGTNVLGNGMGYVLKDWHNGQYFK
LDGDKSTLPQI
11111111111111111111111000000000000000000000000000000000000000000000000000000000111
11111000000000000000000000000000000000000000000000000000000000000000000000000000000
00000000000000000000000000000000000000000000000000000000000000000000000000000000000
00000000000000000000000000000000000000000000000000000000000000000000000000000000000
00000000000000000000000000000000000000000000000000000000000000000000000000000000000
00000000000000000000000000000000000000000000000000000000000000000000000000000000000
00000000000000000000000000000000000000000000000000000000000000000000000000000000000
00000000000000000000000000000000000000000000000000000000000000000000000000000000000
00000000000000000000000000000000000000000000000000000000000000000000000000000000000
00000000000000000000000000000000000000000000000000000000000000000000000000000000000
00000000000000000000000000000000000000000000000000000000000000000000000000000000000
00000000000000000000000000000000000000000000000000000000000000000000000000000000000
000000000000000000000000000000000000000000000000000000000000000000000001111111111
>Q91062_1lsh_17_1071

FQPGKVYRYSYDAFSISGLPEPGVNRAGLSGEMKIEIHGHTHNQATLKITQVNLKYFLGPW
PSDSFYPLTAGYDHFIQQLEVPVRFDYSAGRIGDIYAPPQVTDTAVNIVRGILNLFQLSLKKN
QQTFELQETGVEGICQTTYVVQEGYRTNEMAVVKTKDLNNCDHKVYKTMGTAYAERCPT
CQKMNKNLRSTAVYNYAIFDEPSGYIIKSAHSEEIQQLSVFDIKEGNVVIESRQKLILEGIQSA
PAASQAASLQNRGGLMYKFPSSAITKMSSLFVTKGKNLESEIHTVLKHLVENNQLSVHEDA
PAKFLRLTAFLRNVDAGVLQSIWHKLHQQKDYRRWILDAVPAMATSEALLFLKRTLASEQL
TSAEATQIVYSTLSNQQATRESLSYARELLHTSFIRNRPILRKTAVLGYGSLVFRYCANTVSCP
DELLQPLHDLLSQSSDRADEEEIVLALKALGNAGQPNSIKKIQRFLPGQGKSLDEYSTRVQA
EAIMALRNIAKRDPRKVQEIVLPIFLNVAIKSELRIRSCIVFFESKPSVALVSMVAVRLRREPNL
QVASFVYSQMRSLSRSSNPEFRDVAAACSVAIKMLGSKLDRLGCRYSKAVHVDTFNARTMA
GVSADYFRINSPSGPLPRAVAAKIRGQGMGYASDIVEFGLRAEGLQELLYRGSQEQDAYGTA
LDRQTLLRSGQARSHVSSIHDTLRKLSDWKSVPEERPLASGYVKVHGQEVVFAELDKKMM
QRISQLWHSARSHHAAAQEQIRAVVSKLEQGMDVLLTKGYVVSEVRYMQPVCIGIPMDLN
LLVSGVTTNRANLHASFSSLPADMKLADLLATNIELRVAATTSMSQHAVAIMGLTTDLAKA
GMQTHYKTSAGLGVNGKIEMNARESNFKASLKPFQQKTVVVLSTMESIVFVRDPSGSRILP
VLPPKMTLDKGLISQQQQQPHHQQQPHQHGQDQARAAYQRPWASHEFSPAEQKQIHDI
MTARPVMRRKQHCSKSAALSSKVCFSARLRNAAFIRNALLYKITGDYVSKVYVQPTSSKAQ
IQKVELELQA
00000000000000000000000000000000000000000000000000000000000000000000000000000000000
00000000000000000000000000000000000000000000000000000000000000000000000000000000000
00000000000000000000000000000000000000000000000000000000000000000000000000000000000
00000000000000000000000000000000000000000000000000000000000000000000000000000000000
00000000000000000000000000000000000000000000000000000000000000000000000000000000000
00000000000000000000000000000000000000000000000000000000000000000000000000000000000
00000000000000000000000000000000000000000000000000000000000000000000000000000000000
00000000000000000000000000000000000000000000000000000000000000000000000000000000000
00000001111111111111111111111111111111111111110000000000000000000000000000000011111
11111111111110000000000000000000000000000000000000000000000000000000000000000000000
00000000000000000000000000000000000000000000000000000000000000000000000000000000000
00000000000000000001111111111111111111111111111111111111111111100000000000000000000
0000000000000000000000000000000000000000000000000000000000
>Q24451_1ps3_63_1107
NLRARLAETSNRDDPIRPPLKVARSPRPGQCQDVVQDVPNVDVQMLELYDRMSFKDIDGG
VWKQGWNIKYDPLKYNAHHKLKVFVVPHSHNDPGWIQTFEEYYQHDTKHILSNALRHL
HDNPEMKFIWAEISYFARFYHDLGENKKLQMKSIVKNGQLEFVTGGWVMPDEANSHWR
NVLLQLTEGQTWLKQFMNVTPTASWAIDPFGHSPTMPYILQKSGFKNMLIQRTHYSVKKEL
AQQRQLEFLWRQIWDNKGDTALFTHMMPFYSYDIPHTCGPDPKVCCQFDFKRMGSFGLS
CPWKVPPRTISDQNVAARSDLLVDQWKKKAELYRTNVLLIPLGDDFRFKQNTEWDVQRV
NYERLFEHINSQAHFNVQAQFGTLQEYFDAVHQAERAGQAEFPTLSGDFFTYADRSDNYW
SGYYTSRPYHKRMDRVLMHYVRAAEMLSAWHSWDGMARIEERLEQARRELSLFQHHDGI
TGTAKTHVVVDYEQRMQEALKACQMVMQQSVYRLLTKPSIYSPDFSFSYFTLDDSRWPGS
GVEDSRTTIILGEDILPSKHVVMHNTLPHWREQLVDFYVSSPFVSVTDLANNPVEAQVSPV
WSWHHDTLTKTIHPQGSTTKYRIIFKARVPPMGLATYVLTISDSKPEHTSYASNLLLRKNPTS
LPLGQYPEDVKFGDPREISLRVGNGPTLAFSEQGLLKSIQLTQDSPHVPVHFKFLKYGVRSH
GDRSGAYLFLPNGPASPVELGQPVVLVTKGKLESSVSVGLPSVVHQTIMRGGAPEIRNLVDI
GSLDNTEIVMRLETHIDSGDIFYTDLNGLQFIKRRRLDKLPLQANYYPIPSGMFIEDANTRLT
LLTGQPLGGSSLASGELEIMQDRRLASDDERGLGQGVLDNKPVLHIYRLVLEKVNNCVRPS
ELHPAGYLTSAAHKASQSLLDPLDKFIFAENEWIGAQGQFGGDHPSAREDLDVSVMRRLT
KSSAKTQRVGYVLHRTNLMQCGTPEEHTQKLDVCHLLPNVARCERTTLTFLQNLEHLDG
MVAPEVCPMETAAYVSSHSS
11111111111111111111111111111100000000000000000000000000000000000000000000000000000

00000000000000000000000000000000000000000000000000000000000000000000000000000000000000
00000000000000000000000000000000000000000000000000000000000000000000000000000000000000
00000000000000000000000000000000000000000000000000000000000000000000000000000000000000
00000000000000000000000000000000000000000000000000000000000000000000000000000000000000
00000000000000000000000000000000000000000000000000000000000000000000000000000000000000
00000000000000000000000000000000000000000000000000000000000000000000000000000000000000
00000000000000000000000000000000000000000000000000000000000000000000000000000000000000
00000000000000000000000000000000000000000000000000000000000000000000000000000000000000
00000000000000000000000000000000000000000000000000000000000000000000000000000000000000
00000000000000000000000000000000000000000000000000000000000000000000000000000000000000
00000000000000000000000000000000000000000000000000000000000000000000000000000000000000
00000000000000000000000000000000000000000000000001
>Q56F26_2vzs_1_1031
SFRQKRTRIPLLAMTVTALAAAVCGVTTAPAATGAEVAVPLSVGAAAGNATPIPGYVIQSSA
QVSDDSAVSKPGFPTSGWYPVSSRSTVYAGLLQNGKYADPFYSTNMQNVPAAQFSVPWWY
RTDLNVDDTSSRTYLDFSGVLSKADVWVNGTKVATKDQVNGAYTRHDLDITAQVHTGVN
SVAFKVYPNDPNRDLSMGWIDWAQTPPDQNMGIVRDVLVRRSGAVALRSAHVIQKLNSA
LDHADLTVKADVRNDSANAVQTTVAGTVAGKPISQTVSLAAKERKTVTFPLVGLDRPNVW
WPAGMGGQHRYDLDLTASVGGTPSDAAKSKFGVRDVKATLNSSGGRQYSVNGKPLLIRG
GGYTPDLFLRWNETAAADKLKYVLNLGLNTVRLEGHIEPDEFFDIADDLGVLTMPGWECC
DKWEGQVNGEEKGEPWVESDYPIAKASMFSEAERLRDHPSVISFHIGSDFAPDRRIEQGYL
DAMKAADFLLPVIPAASARPSPITGASGMKMNGPYDYVPPVYWYDKSQKDRGGAWSFNS
ETSAGVDIPTMDTLKRMMSASELDTMWKNPSAKQYHRSSSDTFGNLKLFGDALTKRYGAS
ANLNDFVRKAQLSQYENVRAEFESHSRNYTDSTNPSTGLIYWMLNSPWTSLHWQLFDAY
MDQNGAYYGAKKANEPLHIQYSHDNRSVVVINQTSNAVSGLTATTKLYNLDGTEKYSNTK
TGLSVGALGAKATAVTVPAVSGLSTTYLAKWVLTDSSGKEVSRNVYWLSTKADTLNWGGS
DWYYTPQSAFADLSGLNNLGQSAVGATANSVAGADGTTTTTVTLKNTSGGRLPAFYVDSK
VVDSAGKPVLPVEWNDNAVSLWPGETTTLTAKYRTADLKGSKPSVRISGWNTGTQTVPAD
GSGPGPSDPVDYQAEDATIVQGAVESNHAGYTGTGFVNYDNVAGSSVEWTVTVPSAGTYD
VVVRYANGTTTSRPLDFSVNGSISASGVAFGSTGTWPAWTTKTVRVTLAAGVNKIKAVATT
ANGGPNVDKITL
1111111111111111111111111111111111111111000000000000000000000000000000000000000000000000
00000000000000000000000000000000000000000000000000000000000000000000000000000000000000
00000000000000000000000000000000000000000000000000000000000000000000000000000000000000
00000000000000000000000000000000000000000000000000000000000000000000000000000000000000
00000000000000000000000000000000000000000000000000000000000000000000000000000000000000
00000000000000000000000000000000000000000000000000000000000000000000000000000000000000
00000000000000000000000000000000000000000000000000000000000000000000000000000000000000
00000000000000000000000000000000000000000000000000000000000000000000000000000000000000
00000000000000000000000000000000000000000000000000000000000000000000000000000000000000
00000000000000000000000000000000000000000000000000000000000000000000000000000000000000
00000000000000000000000000000000000000000000000000000000000000000000000000000000000000
00000000000000000000000000000000000000000000000000000000000000000000000111111111111111
11111111111111111111111111111111111111111111111111111111111111111111111111111111111111
111111111111111111111111111111111111
>Q82PP4_3w5m_0_1029
MSALRVTSPSVEYVQRPLGLDAAHPRLSWPMASAAPGRRQSAYQVRVASSAAGLSHPDV
WDSGKVVSDDSVLVPYAGPPLKPRTRYFWSVRVWDADGGASEWSAPSWWETGLMGASQ
WSAKWISAPAPLTEAPSLEGSSWIWFPEGEPANSAPAATRWFRRTVDLPDDITGATLAISAD
NVYAVSVDGAEVARTDLEADNEGWRRPAVIDVLDHVHSGNNTLAVSASNASVGPAGWIC
VLVLTTASGEKKIFSDASWKSTDHEPADGWREPDFDDSGWPAAKVAAAWGAGPWGRVAP
VASAANQLRHEFRLPHKKVSRARLYATALGLYEAHLNGRRVGRDQLAPGWTDYRKRVQY

QTYDVTSSVRPGANALAAYVAPGWYAGNVGMFGPHQYGERPALLAQLEVEYADGTSERIT
SGPDWRAASGPIVSADLLSGETYDARKETAGWTSPGFDDRAWLAVRGADNDVPEQIVAQV
DGPVRIAKELPARKVTEPKPGVFVLDLGQNMVGSVRLRVSGDAGTTVRLRHAEVLNPDGT
IYTANLRSAAATDTYTLKGQGEETYEPRFTFHGFRYVEVTGFPGKPSTTSVTGRVMHTSAPF
TFEFETNVPMLNKLHSNITWGQRGNFLSVPTDTPARDERLGWTGDINVFAPTAAYTMESA
RFLTKWLVDLRDAQTSDGAFTDVAPAVGNLGNGVAGWGDAGVTVPWALYQAYGDRQVL
ADALPSVHAWLRYLEKHSDGLLRPADGYGDWLNVSDETPKDVIATAYFAHSADLAARMA
TELGKDAAPYTDLFTRIRKAFQTAYVASDGKVKGDTQSAYVLTLSMNLVPDALRKAAADRL
VALIEAKDWHLSTGFLGTPRLLPVLTDTGHTDVAYRLLHQRTFPSWGYPIDKGSTTMWERW
DSIQPDGGFQTPEMNSFNHYAYGSVGEWMYANIAGIAPGRAGYRQVVIRPRPGGEVTSAR
ATFASLHGPVSTRWQQRSGGFVLTCSVPPNTTAEVWIPADHPDRVQHTHGTFVRAEDGCA
VFEVGSGSHRFTV
11000000000000000000000000000000000000000000000000000000000000000000000000000000
00000000000000000000000000000000000000000000000000000000000000000000000000000000
00000000000000000000000000000000000000000000000000000000000000000000000000000000
00000000000000000000000000000000000000000000000000000000000000000000000000000000
00000000000000000000000000000000000000000000000000000000000000000000000000000000
00000000000000000000000000000000000000000000000000000000000000000000000000000000
00000000000000000000000000000000000000000000000000000000000000000000000000000000
00000000000000000000000000000000000000000000000000000000000000000000000000000000
00000000000000000000000000000000000000000000000000000000000000000000000000000000
00000000000000000000000000000000000000000000000000000000000000000000000000000000
00000000000000000000000000000000000000000000000000000000000000000000000000000000
00000000000000000000000000000000000000000000000000000000000000000000000000000000
000000000000000000000000000000000
>Q8KRF6_1yq2_0_1022
MTTADVSYLTDQGPGSGRRVPARSWLHSDAPALSLNGDWRFRLLPAAPGTAGAGSVLPSG
ETVEGVAAESYDDAAWDTLPVPSHWVMGQDGKYGRPIYTNVQYPFPIDPPHVPDANPTG
DFRRRFDVPAQWFESTTAALTLRFDGVESRYKVWVNGQEIGVGSGSRLAQEFDVSDALRA
GSNLLVVRVHQWSAASYLEDQDQWWLPGIFRDVTLQARPAGGITDAWLRTGWSARSGAG
TGTIDPEITADATAFPVTLSVPELGVNVTWKSAEEVAPLALENVEPWSAEVPRLYEASVSSA
AESISVRLGFRTVRIVGDQFLVNGRRVVFHGVNRHETHPDRGRVFDEAGAREDLALMKRF
NVNAIRTSHYPPHPRLLDLADEMGFWVILECDLETHGFEAGGWVENPSDVPAWRDALVD
RMERTVERDKNHPSIVMWSLGNESGTGSNLAAMAAWAHARDSSRPVHYEGDYTGAYTD
VYSRMYSSIPETDSIGRNDSHALLLGCDSAESARQRTKPFILCEYVHAMGNGPGAMDQYEA
LVDKYPRLHGGFVWEWRDHGIRTRTAEGMEFFAYGGDFGEVVHDSNFVMDGMVLSDSTP
TPGLYEFKQIVSPIRLGLSLPAGGKPTLAVANLRHTADASDVVLRWRVEHDGAVAASGEVA
AEGSDGPLRAGESATIALPAMPAAPLGETWLTVEAVLRDATGWAPAGHPLGAVQLDLSAP
AVPTRSPRPATPLDGALPVSLGPATFDAGTLVSLAGQPVSGPRLELWRAPTDNDRGAGFGA
YGPGDPWLNSGRGVPAPSSEAVWKQAGLDRLTRRVEDVAALPDGIRVRTRYAAADSTHSV
AVEENWQLDGGELCLRIDITPSAGWNLVWPRIGVRWDLPTDVDGAAWFGAGPRESYPDS
MHATVVARHAASLEELNVPYARPQETGHRSDVRWLELDRAGAPWLRIDAEPDAAGRRPG
FSLARHTAQEIAAAGHPHELPTPSHSYLYVDAAQHGLGSRACGPDVWPDFALRPEARTLK
LRISPA
11100000000000000000000000000000000000000000000000000000000000000000000000000000
00000000000000000000000000000000000000000000000000000000000000000000000000000000
00000000000000000000000000000000000000000000000000000000000000000000000000000000
00000000000000000000000000000000000000000000000000000000000000000000000000000000
00000000000000000000000000000000000000000000000000000000000000000000000000000000
00000000000000000000000000000000000000000000000000000000000000000000000000000000
00000000000000000000000000000000000000000000000000000000000000000000000000000000

00000000000000000000000000000000000000000000000000000000000000000000000000000000000
00000000000000000000000000000000000000000000000000000000000000000000000000000000000
00000000000000000000000000000000000000000000000000000000000000000000000000000000000
00000000000000000000000000000000000000000000000000000000000000000000000000000000000
00000000000000000000000000000000000000000000000000000000000000000000000000000000000
000000000000000000000000000
>P59807_1hn0_0_1020
MPIFRFTALAMTLGLLSAPYNAMAATSNPAFDPKNLMQSEIYHFAQNNPLADFSSDKNSIL
TLSDKRSIMGNQSLLWKWKGGSSFTLHKKLIVPTDKEASKAWGRSSTPVFSFWLYNEKPIDG
YLTIDFGEKLISTSEAQAGFKVKLDFTGWRAVGVSLNNDLENREMTLNATNTSSDGTQDSI
GRSLGAKVDSIRFKAPSNVSQGEIYIDRIMFSVDDARYQWSDYQVKTRLSEPEIQFHNVKPQ
LPVTPENLAAIDLIRQRLINEFVGGEKETNLALEENISKLKSDFDALNIHTLANGGTQGRHLI
TDKQIIIYQPENLNSQDKQLFDNYVILGNYTTLMFNISRAYVLEKDPTQKAQLKQMYLLMT
KHLLDQGFVKGSALVTTHHWGYSSRWWYISTLLMSDALKEANLQTQVYDSLLWYSREFKS
SFDMKVSADSSDLDYFNTLSRQHLALLLLEPDDQKRINLVNTFSHYITGALTQVPPGGKDG
LRPDGTAWRHEGNYPGYSFPAFKNASQLIYLLRDTPFSVGESGWNNLKKAMVSAWIYSNP
EVGLPLAGRHPFNSPSLKSVAQGYYWLAMSAKSSPDKTLASIYLAISDKTQNESTAIFGETIT
PASLPQGFYAFNGGAFGIHRWQDKMVTLKAYNTNVWSSEIYNKDNRYGRYQSHGVAQIVS
NGSQLSQGYQQEGWDWNRMQGATTIHLPLKDLDSPKPHTLMQRGERGFSGTSSLEGQYG
MMAFDLIYPANLERFDPNFTAKKSVLAADNHLIFIGSNINSSDKNKNVETTLFQHAITPTLN
TLWINGQKIENMPYQTTLQQGDWLIDSNGNGYLITQAEKVNVSRQHQVSAENKNRQPTE
GNFSSAWIDHSTRPKDASYEYMVFLDATPEKMGEMAQKFRENNGLYQVLRKDKDVHIILD
KLSNVTGYAFYQPASIEDKWIKKVNKPAIVMTHRQKDTLIVSAVTPDLNMTRQKAATPVTI
NVTINGKWQSADKNSEVKYQVSGDNTELTFTSYFGIPQEIKLSPLP
11111111111111111111111100000000000000000000000000000000000000000000000000000000000
00000000000000000000000000000000000000000000000000000000000000000000000000000000001
11111111111111111111111110000000000000000000000000000000000000000000000000000000000
00000000000000000000000000000000000000000000000000000000000000000000000000000000000
00000000000000000000000000000000000000000000000000000000000000000000000000000000000
00000000000000000000000000000000000000000000000000000000000000000000000000000000000
00000000000000000000000000000000000000000000000000000000000000000000000000000000000
00000000000000000000000000000000000000000000000000000000000000000000000000000000000
00000000000000000000000000000000000000000000000000000000000000000000000000000000000
00000000000000000000000000000000000000000000000000000000000000000000000000000000000
00000000000000000000000000000000000000000000000000000000000000000000000000000000000
00000000000000000000000000000000000000000000000000000000000000000000000000000000000
0000000000000000000000000
>Q70SY0_3og2_20_1022
TSIGLHGGRPRDIILDDAKGPLQNIVTWDEHSLFVHGERVVIFSGEVHPFRLPVPSLYLDVFH
KIKALGFNTVSFYVDWALLEGKPGRFRADGIFSLEPFFEAATKAGIYLLARPGPYINAEVSGG
GFPGWLQRVKGKLRTDAPDYLHATDNYVAHIASIIAKAQITNGGPVILYQPENEYSGAAEG
VLFPNKPYMQYVIDQARNAGIIVPLINNDAFPGGTGAPGTGLGSVDIYGHDGYPLGFDCA
HPSAWPDNGLPTTWRQDHLNISPSTPFSLVEFQGGAFDPFGGWGFEQCSALVNHEFERVFY
KNNMAAGVTIFNIYMTFGGTNWGNLGHPGGYTSYDYGASIREDRRIDREKYSELKLQGQF
LKVSPGYITATPENATQGVYSDSQNIVITPLLAKESGDFFVVRHANYSSTDTASYTVKLPTSA
GDLTIPQLGGSLTLTGRDSKIHVTDYPVGKFTLLYSTAEIFTWNEFAEKTVLVLYGGAQELHE
FAVKNPFGSSKTAKAKKIEGSNVTIHTTSNLTVVLQWTASSARQVVQLGSLVIYMVDRNSAY
NYWVPTLPGSGKQSAYGSSLMNPDSVIINGGYLIRSVAIKGNALSVQADFNVTTPLEIIGIPK
GISKLAVNGKELGYSVSELGDWIAHPAIEIPHVQVPELTKLKWYKVDSLPEIRSNYDDSRWP
LANLRTSNNTYAPLKTPVSLYGSDYGFHAGTLLFRGRFTARTARQQLFLSTQGGSAFASSVW
LNDRFIGSFTGFDAASAANSSYTLDRLVRGRRYILTVVVDSTGLDENWTTGDDSMKAPRGIL

DYALTSSSGANVSISWKLTGNLGGEDYRDVFRGPLNEGGLFFERQGFHLPSPPLSDFTHGPS
SSSSSSSPLDGIAHAGIAFYAAKLPLHLPAQEYDIPLSFVFDNATAAAPYRALLYVNGFQYGK
YVSNIGPQTEFPVPEGILDYNGDNWIGVALWALESRGAKVPGLALKSKSPILTGRERVEVVK
GPHFKKRHGAY
1111111111111111100000000000000000000000000000000000000000000000000000000000000000000
0000000000000000000000000000000000000000000000000000000000000000000000000000000000000
0000000000000000000000000000000000000000000000000000000000000000000000000000000000000
0000000000000000000000000000000000000000000000000000000000000000000000000000000000000
0000000000000000000000000000000000000000000000000000000000000000000000000000000000000
0000000000000000000000000000000000000000000000000000000000000000000000000000000000000
0000000000000000000000000000000000000000000000000000000000000000000000000000000000000
0000000000000000000000000000000000000000000000000000000000000000000000000000000000000
0000000000000000000000000000000000000000000000000000000000000000000000000000000000000
0000000000000000000000000000000000000000000000000000000000000000000000000000000000000
0000000000000000000000000000000000000000000000000000000000000000000000000000000000000
0000000000000000000000000000000000000000000000000000000000000000000000000000000000000
0000000
>P44969_3h09_25_1005
ALVRDDVDYQIFRDFAENKGRFSVGATNVEVRDKNNHSLGNVLPNGIPMIDFSVVDVDKR
IATLINPQYVVGVKHVSNGVSELHFGNLNGNMNNGNAKSHRDVSSEENRYFSVEKNEYPT
KLNGKAVTTEDQTQKRREDYYMPRLDKFVTEVAPIEASTASSDAGTYNDQNKYPAFVRLGS
GSQFIYKKGDNYSLILNNHEVGGNNLKLVGDAYTYGIAGTPYKVNHENNGLIGFGNSKEE
HSDPKGILSQDPLTNYAVLGDSGSPLFVYDREKGKWLFLGSYDFWAGYNKKSWQEWNIYK
PEFAKTVLDKDTAGSLTGSNTQYNWNPTGKTSVISNGSESLNVDLFDSSQDTDSKKNNHG
KSVTLRGSGTLTLNNNIDQGAGGLFFEGDYEVKGTSDSTTWKGAGVSVADGKTVTWKVH
NPKSDRLAKIGKGTLIVEGKGENKGSLKVGDGTVILKQQADANNKVKAFSQVGIVSGRST
VVLNDDKQVDPNSIYFGFRGGRLDANGNNLTFEHIRNIDDGARLVNHNTSKTSTVTITGES
LITDPNTITPYNIDAPDEDNPYAFRRIKDGGQLYLNLENYTYYALRKGASTRSELPKNSGESN
ENWLYMGKTSDEAKRNVMNHINNERMNGFNGYFGEEEGKNNGNLNVTFKGKSEQNRF
LLTGGTNLNGDLKVEKGTLFLSGRPTPHARDIAGISSTKKDQHFAENNEVVVEDDWINRN
FKATNINVTNNATLYSGRNVANITSNITASDNAKVHIGYKAGDTVCVRSDYTGYVTCTTDK
LSDKALNSFNATNVSGNVNLSGNANFVLGKANLFGTISGTGNSQVRLTENSHWHLTGDS
NVNQLNLDKGHIHLNAQNDANKVTTYNTLTVNSLSGNGSFYYLTDLSNKQGDKVVVTKS
ATGNFTLQVADKTGEPTKNELTLFDASNATRNNLNVSLVGNTVDLGAWKYKLRNVNGRY
DLYNPEVEKRNQTVDTTNITTP
0000000000000000000000000000000000000000000000000000000000000000000000000000000000000
0000000000000000000000000000000000000000000000000000000000000000000000000000000000000
0000000000000000000000000000000000000000000000000000000000000000000000000000000000000
0000000000000000000000000000000000000000000000000000000000000000000000000000000000000
0000000000000000000000000000000000000000000000000000000000000000000000000000000000000
0000000000000000000000000000000000000000000000000000000000000000000000000000000000000
0000000000000000000000000000000000000000000000000000000000000000000000000000000000000
0000000000000000000000000000000000000000000000000000000000000000000000000000000000000
0000000000000000000000000000000000000000000000000000000000000000000000000000000000000
0000000000000000000000000000000000000000000000000000000000000000000000000000000000000
0000000000000000000000000000000000000000000000000000000000000000000000000000000000000
0000000000000000000000000000000000000000000000000000011111111111111111
>Q5P5I0_2ivf_0_975
MTRDEMISVEPEAAELQDQHRRDFLKRSGAAVLSLSLSSLATGVVPGFLKDAQAGTKAPGY
ASWEDIYRKEWKWDKVNWGSHLNICWPQGSCKFYVYVRNGIVWREEQAAQTPACNVDY
VDYNPLGCQKGSAFNNNLYGDERVKYPLKRVGKRGEGKWKRVSWDEAAGDIADSIIDSFE

AQGSDGFILDAPHVHAGSIAWGAGFRMTYLMDGVSPDINVDIGDTYMGAFHTFGKMHM
GYSADNLLDAELIFMTCSNWSYTYPSSYHFLSEARYKGAEVVVIAPDFNPTTPAADLHVPV
RVGSDAAFWLGLSQVMIDEKLFDRQFVCEQTDLPLLVRMDTGKFLSAEDVDGGEAKQFYF
FDEKAGSVRKASRGTLKLDFMPALEGTFSARLKNGKTIQVRTVFEGLREHLKDYTPEKASA
KCGVPVSLIRELGRKVAKKRTCSYIGFSSAKSYHGDLMERSLFLAMALSGNWGKPGTGAFA
WAYSDDNMVYLGVMSKPTAQGGMDELHQMAEGFNKRTLEADPTSTDEMGNIEFMKVVT
SAVGLVPPAMWLYYHVGYDQLWNNKAWTDPALKKSFGAYLDEAKEKGWWTNDHIRPAP
DKTPQVYMLLSQNPMRRKRSGAKMFPDVLFPKLKMIFALETRMSSSAMYADIVLPCAWYY
EKHEMTTPCSGNPFFTFVDRSVAPPGECREEWDAIALILKKVGERAAARGLTEFNDHNGRK
RRYDELYKKFTMDGHLLTNEDCLKEMVDINRAVGVFAKDYTYEKFKKEGQTRFLSMGTGV
SRYAHANEVDVTKPIYPMRWHFDDKKVFPTHTRRAQFYLDHDWYLEAGESLPTHKDTPM
VGGDHPFKITGGHPRVSIHSTHLTNSHLSRLHRGQPVVHMNSKDAAELGIKDGDMAKLF
NDFADCEIMVRTAPNVQPKQCIVYFWDAHQYKGWKPYDILLIGMPKPLHLAGGYEQFRY
YFMNGSPAPVTDRGVRVSIKKA
1111111111111111111111111111111111111111111111111111111111111000000000000000000
00000000000000000000000000000000000000000000000000000000000000000000000000000000
00000000000000000000000000000000000000000000000000000000000000000000000000000000
00000000000000000000000000000000000000000000000000000000000000000000000000000000
00000000000000000000000000000000000000000000000000000000000000000000000000000000
00000000000000000000000000000000000000000000000000000000000000000000000000000000
00000000000000000000000000000000000000000000000000000000000000000000000000000000
00000000000000000000000000000000000000000000000000000000000000000000000000000000
00000000000000000000000000000000000000000000000000000000000000000000000000000000
00000000000000000000000000000000000000000000000000000000000000000000000000000000
00000000000000000000000000000000000000000000000000000000000000000000000000000000
00000000000000000000000000000000000000000000000000000000000000000000000000000000
0000000000000000000000000000000000000000000000000000000000000
>P32504_4aco_0_955
MRSSILFLLKLMKIMDVQQQQEAMSSEDRFQELVDSLKPRTAHQYKTYYTKYIQWCQLNQI
IPTPEDNSVNSVPYKDLPISAELIHWFLLDTLITDDKPGEKREETEDLDEEEENSFKIATLKKII
GSLNFLSKLCKVHENPNANIDTKYLESVTKLHTHWIDSQKAITTNETNNTNTQVLCPPLLK
VSLNLWNPETNHLSEKFFKTCSEKLRFLVDFQLRSYLNLSFEERSKIRFGSLKLGKRDRDAIIY
HKVTHSAEKKDTPGHHQLLALLPQDCPFICPQTTLAAYLYLRFYGIPSVSKGDGFPNLNAD
ENGSLLQDIPILRGKSLTTYPREETFSNYYTTVFRYCHLPYKRREYFNKCNLVYPTWDEDTFR
TFFNEENHGNWLEQPEAFAFPDKIPFDFKKIMNFKSPYTSYSTNAKKDPFPPPKDLLVQIFPE
IDEYKRHDYEGLSQNSRDFLDLMEVLRERFLSNLPWIYKFFPNHDIFQDPIFGNSDFQSYFN
DKTIHSKGSPILSFDILPGFNKIYKNKTNFYSLLIERPSQLTFASSHNPDTHPTQKQESEGPLQ
MSQLDTTQLNELLKQQSFEYVQFQTLSNFQILLSVFNKIFEKLEMKKSSRGYILHQLNLFKIT
LDERIKKSKIDDADKFIRDNQPIKKEENIVNEDGPNTSRRTKRPKQIRLLSIADSSDESSTEDS
NVFKKDGESIEDGAYGENEDENDSEMQEQLKSMINELINSKISTFLRDQMDQFELKINALL
DKILEEKVTRIIEQKLGSHTGKFSTLKRPQLYMTEEHNVGFDMEVPKKLRTSGKYAETVKDN
DDHQAMSTTASPSPEQDQEAKSYTDEQEFMLDKSIDSIEGIILEWFTPNAKYANQCVHSMN
KSGNKSWRANCEALYKERKSIVEFYIYLVNHESLDRYKAVDICEKLRDQNEGSFSRLAKFLR
KWRHDHQNSFDGLLVYLSN
11111111111111111111111111111111111111110000000000000000000000111111000000000000
00000000000000111111111000000000000000000000000000000000000000000000000000000000
00111111111111100000000000000000000000000000000000000000000000000000000000000000
00000000111111000000000000000000000000000000000000000000000000000000000000000000
00000000000000000000000000000000000000000000000000000000000000000000000000000001
11111110000000000000000000000000000000000000000000000000000000000000000000000000
00000000000000000000000000000000000000011111111111111111111111111111111111111111
11111111111111111111111111111111111111111111111111111111111111111111111111111111

1111111111111111111111111111111111111111111111111111111111111111111111111111
1111111111111111111111111111111111111111111111111111111111111111111111111111
1111111111111111111111111111111111111111111111111111111111111111111111111111
1111111111111111111111111111111111111111111
>Q93RE7_2okx_0_955
MAGRNWNASWIWGGQEESPRNEWRCFRGSFDAPASVEGPAMLHITADSRYVLFVNGEQV
GRGPVRSWPKEQFYDSYDIGGQLRPGVRNTIAVLVLHFGVSNFYYLRGRGGLIAEIEADGRT
LAATDAAWRTERLGGQRSNSPRMACQQGFGEVIDARELAEDWALPAFDDGGWAQARSIG
PAGTAPWTSLVPRDIPFLTEEKLYPASIQSLSRVKAPKYAAALDLRNQMVPESVNHANPVSY
CGYVATILTLETSGVVTLGFPTGVRGSGVWVDGVLQTEWTGVQPERYYSLNLAAGEHLVLV
DITSSDHGGSSHFAIDSEAAFTLRSPAGDNGVPLATIGTFDQSEYIDHRPGRRMQTDHPDYR
ALPEAAPTAAALEAFASWVKPFEPSLYTEENVFGSNVWRTLAERRAVPRSVLNAILPVPEPG
VLPVFEDGDCELVIDLGAERSGFIGFELEAPAGTIIDAYGVEYMREGYTQHTYGLDNTFRYIC
REGRQSYVSPVRRGFRYLFLTVRGNSAPVKLHEIYIRQSTYPVAEQGSFRCSDALLNATWEIS
RHTTRLCMEDTFVDCPSYEQVFWVGDSRNEALVNYYVFGETEIVERCLNLVPGSADETPLY
LDQVPSAWSSVIPNWTFFWILACREYAAHTGNEAFAARIWPAVKHTLTHYLEHIDDSGLLN
MAGWNLLDWAPIDQPNEGIVTHQNLFLVKALRDSRALAAAAGATEEADAFAARADLLA
ETINAVLWDEEKRAYIDCIHADGRRSDVYSMQTQVVAYLCGVAQGEREAVIEGYLSSPPPAF
VQIGSPFMSFFYYEALEKAGRQTLMLDDIRRNYGQMLRYDATTCWEMYPNFAENRSNPD
MLTRSHCHAWSAAPGYFLGSSILGVKRGADGWRTVDIAPQPCDLTWAEGVVPLPQGGHIA
VSWEFVSAGKLKLRIEAPEDIEVNVTLPEGIEGEVTQVKYMS
1100000000000000000000000000000000000000000000000000000000000000000000000000
0000000000000000000000000000000000000000000000000000000000000000000000000000
0000000000000000000000000000000000000000000000000000000000000000000000000000
0000000000000000000000000000000000000000000000000000000000000000000000000000
0000000000000000000000000000000000000000000000000000000000000000000000000000
0000000000000000000000000000000000000000000000000000000000000000000000000000
0000000000000000000000000000000000000000000000000000000000000000000000000000
0000000000000000000000000000000000000000000000000000000000000000000000000000
0000000000000000000000000000000000000000000000000000000000000000000000000000
0000000000000000000000000000000000000000000000000000000000000000000000000000
0000000000000000000000000000000000000000000000000000000000000000000000000000
0000000000000000000000000000000000000000000000000000000000000000000000000000
0000000000000000000000000000000000000000000
>Q8CZ52_3gdb_37_952
EETAVPENSGANTELVSGESEHSTNEADKQNEGEHARENKLEKAEGVATASETASPASNEA
ATTETAEAASAAKPEEKASEVVAETPSAEAKPKSDKETEAKPEATNQGDESKPAAEANKTE
KEVQPDVPKNTEKTLKPKEIKFNSWEELLKWEPGAREDDAINRGSVVLASRRTGHLVNEK
ASKEAKVQALSNTNSKAKDHASVGGEEFKAYAFDYWQYLDSMVFWEGLVPTPDVIDAGH
RNGVPVYGTLFFNWSNSIADQERFAEALKQDADGSFPIARKLVDMAKYYGYDGYFINQET
TGDLVKPLGEKMRQFMLYSKEYAAKVNHPIKYSWYDAMTYNYGRYHQDGLGEYNYQFM
QPEGDKVPADNFFANFNWDKAKNDYTIATANWIGRNPYDVFAGLELQQGGSYKTKVKW
NDILDENGKLRLSLGLFAPDTITSLGKTGEDYHKNEDIFFTGYQGDPTGQKPGDKDWYGIA
NLVADRTPAVGNTFTTSFNTGHGKKWFVDGKVSKDSEWNYRSVSGVLPTWRWWQTSTGE
KLRAEYDFTDAYNGGNSLKFSGDVAGKTDQDVRLYSTKLEVTEKTKLRVAHKGGKGSKVY
MAFSTTPDYKFDDADAWKELTLSDNWTNEEFDLSSLAGKTIYAVKLFFEHEGAVKDYQFNL
GQLTISDNHQEPQSPTSFSVVKQSLKNAQEAEAVVQFKGNKDADFYEVYEKDGDSWKLLT
GSSSTTIYLPKVSRSASAQGTTQELKVVAVGKNGVRSEAATTTFDWGMTVKDTSLPKPLAE
NIVPGATVIDSTFPKTEGGEGIEGMLNGTITSLSDKWSSAQLSGSVDIRLTKSRTVVRWVMD
HAGAGGESVNDGLMNTKDFDLYYKDADGEWKLAKEVRGNKAHVTDITLDKPITAQDWR
LNVVTSDNGTPWKAIRI
1111111111111111111111111111111111111111111111111111111111111111111111111111

11111111111111111111111111111111111111111111111111000000000000000000000000000000
00000000000000000000000000000000000000000000000000000000000000000000000000000000
00000000000000000000000000000000000000000000000000000000000000000000000000000000
00000000000000000000000000000000000000000000000000000000000000000000000000000000
00000000000000000000000000000000000000000000000000000000000000000000000000000000
00000000000000000000000000000000000000000000000000000000000000000000000000000000
00000000000000000000000000000000000000000000000000000000000000000000000000000000
00000000000000000000000000000000000000000000000000000000000000000000000000000000
00000000000000000000011111111111111111111111111111111111111111111111111111111111
11111111111111111111111111111111111111111111111111111111111111111111111111111111
111
>Q6JV24_2ead_576_1473
VIASVEDGGDGDTSKDDWLWYKQPASQTDATATAGGNYGNPDNNRWQQTTLPFGNGKI
GGTVWGEVSRERVTFNEETLWTGGPGSSTSYNGGNNETKGQNGATLRALNKQLANGAET
VNPGNLTGGENAAEQGNYLNWGDIYLDYGFNDTTVTEYRRDLNLSKGKADVTFKHDGV
TYTREYFASNPDNVMVARLTASKAGKLNFNVSMPTNTNYSKTGETTTVKGDTLTVKGALG
NNGLLYNSQIKVVLDNGEGTLSEGSDGASLKVSDAKAVTLYIAAATDYKQKYPSYRTGETA
AEVNTRVAKVVQDAANKGYTAVKKAHIDDHSAIYDRVKIDLGQSGHSSDGAVATDALLK
AYQRGSATTAQKRELETLVYKYGRYLTIGSSRENSQLPSNLQGIWSVTAGDNAHGNTPWGS
DFHMNVNLQMNYWPTYSANMGELAEPLIEYVEGLVKPGRVTAKVYAGAETTNPETTPIGE
GEGYMAHTENTAYGWTAPGQSFSWGWSPAAVPWILQNVYEAYEYSGDPALLDRVYALLK
EESHFYVNYMLHKAGSSSGDRLTTGVAYSPEQGPLGTDGNTYESSLVWQMLNDAIEAAKA
KGDPDGLVGNTTDCSADNWAKNDSGNFTDANANRSWSCAKSLLKPIEVGDSGQIKEWYF
EGALGKKKDGSTISGYQADNQHRHMSHLLGLFPGDLITIDNSEYMDAAKTSLRYRCFKGN
VLQSNTGWAIGQRINSWARTGDGNTTYQLVELQLKNAMYANLFDYHAPFQIDGNFGNTS
GVDEMLLQSNSTFTDTAGKKYVNYTNILPALPDAWAGGSVSGLVARGNFTVGTTWKNGK
ATEVRLTSNKGKQAAVKITAGGAQNYEVKNGDTAVNAKVVTNADGASLLVFDTTAGTTY
TITKKAS
11111111110000000000000000000000000000000000000000000000000000000000000000000000
00000000000000000000000000000000000000000000000000000000000000000000000000000000
00000000000000000000000000000000000000000000000000000000000000000000000000000000
00000000000000000000000000000000000000000000000000000000000000000000000000000000
00000000000000000000000000000000000000000000000000000000000000000000000000000000
00000000000000000000000000000000000000000000000000000000000000000000000000000000
00000000000000000000000000000000000000000000000000000000000000000000000000000000
00000000000000000000000000000000000000000000000000000000000000000000000000000000
00000000000000000000000000000000000000000000000000000000000000000000000000000000
00000000000000000000000000000000000000000000000000000000000000000000000000000000
00000000000000000000000000000000000000000000000000000000000000011
>Q38HX4_2yaj_0_896
MNVKETKLEDVLKSRGIDMKDAYNISEADIPEAKESTQKLMDIYYTLKVTADMEAAYWYN
RTWWENDGEVIEVRRAKAVAASLSHMTPTILPYEKLVMNKTKNVRGAFPFPWVCASFFNA
QAEALMNEVDAPAENEADSVSVVGAGGGNVTESYGNVISIAKKFGMRKEEIPVLVKTSKP
WEGISVEELSNKYSKMTPGYDQFKNIMESVICMFDSFAIPQGREVINYYMPLQYGFDGIIKLC
DEKIAEVMGEAGDDGDFGMSRGYYYAAMKEITKGLSAWCENYSKRAKYLASIETDSEIKA
NYEKIEEVMGNIAHKKPANFWEAIQMTLCCHFGVVNEDPQSGLSIGRLGQVLQPFYEKDV
EDGIMTDEEVIELLELYRIKITCIECFASAGVSGGVLSGNTFNNLSLGGQNYDGLSAVTPLEYL
IVEAGMRNQTPQPTLSVLYDEKTPEDFLMKAASCTKLGLGYPAWMNNQTGMNFMMRNY
GPEGMDLHDARAWCLGGCLESAPGCFLPLEYNGKVTMIPGGASPTCGTGVHFIGMPKVLE
LVLTNGLDKRTGKQVYPPHNKKLDSYETMVNQWKEYMELTTDVVNRCNNIQMDIWRKY
NMPAVNSLLKPDCFKKGKHIGTMGARYNSCINFESCGTITFVNSLSSIKKNVFDDSKFTIEE

MTDAMLNNFGFKTAYETEVFSPDFRESTDKSTKYEKIFAACVNAPKYGNADKYADEIFKAY
HYYIYDMTHKFRSYYGKPLYLCQISVSTHGPQGFVTLATADGRLAGTTYSDGSVSAAAGTD
KNGIYAIFESATVYDHSMHQNAQMNLKLHPTAVKGINGTRKLLDLVRAYMRKGGFHVQF
NVVDSKTLRDAQLTPEKYRELMVRVAGFTQYWCEIGKPIQDEVIYRTEYDK
11111111111111111111111111000000000000000000000000000000000000000000000000000000000
00000000000000000000000000000000000000000000000000000000000000000000000000000000000
00000000000000000000000000000000000000000000000000000000000000000000000000000000000
00000000000000000000000000000000000000000000000000000000000000000000000000000000000
00000000000000000000000000000000000000000000000000000000000000000000000000000000000
00000000000000000000000000000000000000000000000000000000000000000000000000000000000
00000000000000000000000000000000000000000000000000000000000000000000000000000000000
00000000000000000000000000000000000000000000000000000000000000000000000000000000000
00000000000000000000000000000000000000000000000000000000000000000000000000000000000
00000000000000000000000000000000000000000000000000000000000000000000000000000000000
0000000000000000000000000000000000000000000000000000000000000000000
>Q97SC3_2ww8_0_892
MLNRETHMKKVRKIFQKAVAGLCCISQLTAFSSIVALAETPETSPAIGKVVIKETGEGGALLG
DAVFELKNNTDGTTVSQRTEAQTGEAIFSNIKPGTYTLTEAQPPVGYKPSTKQWTVEVEKN
GRTTVQGEQVENREEALSDQYPQTGTYPDVQTPYQIIKVDGSEKNGQHKALNPNPYERVIP
EGTLSKRIYQVNNLDDNQYGIELTVSGKTVYEQKDKSVPLDVVILLDNSNSMSNIRNKNAR
RAERAGEATRSLIDKITSDSENRVALVTYASTIFDGTEFTVEKGVADKNGKRLNDSLFWNYD
QTSFTTNTKDYSYLKLTNDKNDIVELKNKVPTEAEDHDGNRLMYQFGATFTQKALMKAD
EILTQQARQNSQKVIFHITDGVPTMSYPINFNHATFAPSYQNQLNAFFSKSPNKDGILLSDFI
TQATSGEHTIVRGDGQSYQMFTDKTVYEKGAPAAFPVKPEKYSEMKAAGYAVIGDPINGG
YIWLNWRESILAYPFNSNTAKITNHGDPTRWYYNGNIAPDGYDVFTVGIGINGDPGTDEAT
ATSFMQSISSKPENYTNVTDTTKILEQLNRYFHTIVTEKKSIENGTITDPMGELIDLQLGTDGR
FDPADYTLTANDGSRLENGQAVGGPQNDGGLLKNAKVLYDTTEKRIRVTGLYLGTDEKVT
LTYNVRLNDEFVSNKFYDTNGRTTLHPKEVEQNTVRDFPIPKIRDVRKYPEITISKEKKLGDI
EFIKVNKNDKKPLRGAVFSLQKQHPDYPDIYGAIDQNGTYQNVRTGEDGKLTFKNLSDGK
YRLFENSEPAGYKPVQNKPIVAFQIVNGEVRDVTSIVPQDIPAGYEFTNDKHYITNEPIPPKR
EYPRTGGIGMLPFYLIGCMMMGGVLLYTRKHP
11111111111111111111111111111111111111111111100000000000000000000000000000000000000
00000000000000000000000000000000000000000000000000000000000000000000000000000000000
00000000000000000000000000000000000000000000000000000000000000000000000000000000000
00000000000000000000000000000000000000000000000000000000000000000000000000000000000
00000000000000000000000000000000000000000000000000000000000000000000000000000000000
00000000000000000000000000000000000000000000000000000000000000000000000000000000000
00000000000000000000000000000000000000000000000000000000000000000000000000000000000
00000000000000000000000000000000000000000000000000000000000000000000000000000000000
00000000000000000000000000000000000000000000000000000000000000000000000000000000000
00000000000000000000000000000000000000000000000000000000000000000000000000000000000
000000000000000000000000000000111111111111111111111111111111111
>O43451_3l4x_86_953
SAECPVVNELERINCIPDQPPTKATCDQRGCCWNPQGAVSVPWCYYSKNHSYHVEGNLV
NTNAGFTARLKNLPSSPVFGSNVDNVLLTAEYQTSNRFHFKLTDQTNNRFEVPHEHVQSFS
GNAAASLTYQVEISRQPFSIKVTRRSNNRVLFDSSIGPLLFADQFLQLSTRLPSTNVYGLGEH
VHQQYRHDMNWKTWPIFNRDTTPNGNGTNLYGAQTFFLCLEDASGLSFGVFLMNSNAM
EVVLQPAPAITYRTIGGILDFYVFLGNTPEQVVQEYLELIGRPALPSYWALGFHLSRYEYGTL
DNMREVVERNRAAQLPYDVQHADIDYMDERRDFTYDSVDFKGFPEFVNELHNNGQKLVI
IVDPAISNNSSSSKPYGPYDRGSDMKIWVNSSDGVTPLIGEVWPGQTVFPDYTNPNCAVWW
TKEFELFHNQVEFDGIWIDMNEVSNFVDGSVSGCSTNNLNNPPFTPRILDGYLFCKTLCMD

AVQHWGKQYDIHNLYGYSMAVATAEAAKTVFPNKRSFILTRSTFAGSGKFAAHWLGDNTA
TWDDLRWSIPGVLEFNLFGIPMVGPDICGFALDTPEELCRRWMQLGAFYPFSRNHNGQGY
KDQDPASFGADSLLLNSSRHYLNIRYTLLPYLYTLFFRAHSRGDTVARPLLHEFYEDNSTWD
VHQQFLWGPGLLITPVLDEGAEKVMAYVPDAVWYDYETGSQVRWRKQKVEMELPGDKIG
LHLRGGYIFPTQQPNTTTLASRKNPLGLIIALDENKEAKGELFWDNGETKDTVANKVYLLC
EFSVTQNRLEVNISQSTYKDPNNLAFNEIKILGTEEPSNVTVKHNGVPSQTSPTVTYDSNLK
VAIITDIDLLLGEAYTVEW
11111100000000000000000000000000000000000000000000000000000000000000000000000000000
00000000000000000000000000000000000000000000000000000000000000000000000000000000000
00000000000000000000000000000000000000000000000000000000000000000000000000000000000
00000000000000000000000000000000000000000000000000000000000000000000000000000000000
00000000000000000000000000000000000000000000000000000000000000000000000000000000000
00000000000000000000000000000000000000000000000000000000000000000000000000000000000
00000000000000000000000000000000000000000000000000000000000000000000000000000000000
00000000000000000000000000000000000000000000000000000000000000000000000000000000000
00000000000000000000000000000000000000000000000000000000000000000000000000000000000
00000000000000000000000000000000000000000000000000000000000000000000000000000000000
00000000000000000000000000000000000000000000000000000000000000000000000000000000000
00000010000000000000000000000000000000
>Q06142_2bpt_0_860
MSTAEFAQLLENSILSPDQNIRLTSETQLKKLSNDNFLQFAGLSSQVLIDENTKLEGRILAALT
LKNELVSKDSVKTQQFAQRWITQVSPEAKNQIKTNALTALVSIEPRIANAAAQLIAAIADIEL
PHGAWPELMKIMVDNTGAEQPENVKRASLLALGYMCESADPQSQALVSSSNNILIAIVQG
AQSTETSKAVRLAALNALADSLIFIKNNMEREGERNYLMQVVCEATQAEDIEVQAAAFGC
LCKIMSLYYTFMKPYMEQALYALTIATMKSPNDKVASMTVEFWSTICEEEIDIAYELAQFPQS
PLQSYNFALSSIKDVVPNLLNLLTRQNEDPEDDDWNVSMSAGACLQLFAQNCGNHILEPV
LEFVEQNITADNWRNREAAVMAFGSIMDGPDKVQRTYYVHQALPSILNLMNDQSLQVKE
TTAWCIGRIADSVAESIDPQQHLPGVVQACLIGLQDHPKVATNCSWTIINLVEQLAEATPSPI
YNFYPALVDGLIGAANRIDNEFNARASAFSALTTMVEYATDTVAETSASISTFVMDKLGQT
MSVDENQLTLEDAQSLQELQSNILTVLAAVIRKSPSSVEPVADMLMGLFFRLLEKKDSAFIE
DDVFYAISALAASLGKGFEKYLETFSPYLLKALNQVDSPVSITAVGFIADISNSLEEDFRRYSD
AMMNVLAQMISNPNARRELKPAVLSVFGDIASNIGADFIPYLNDIMALCVAAQNTKPENG
TLEALDYQIKVLEAVLDAYVGIVAGLHDKPEALFPYVGTIFQFIAQVAEDPQLYSEDATSRAA
VGLIGDIAAMFPDGSIKQFYGQDWVIDYIKRTRSGQLFSQATKDTARWAREQQKRQLSL
00000000000000000000000000000000000000000000000000000000000000000000000000000000000
00000000000000000000000000000000000000000000000000000000000000000000000000000000000
00000000000000000000000000000000000000000000000000000000000000000000000000000000000
00000000000000000000000000000000000000000000000000000000000000000000000000000000000
00000001000000000000000000000000000000000000000000000000000000000000000000000000000
00000000000000000000000000000000000000000000000000000000000000000000000000000000000
00000000000000000000000000000000000000000000000000000000000000000000000000000000000
00000000000000000000000000000000000000000000000000000000000000000000000000000000000
00000000000000000000000000000000000000000000000000000000000000000000000000000000000
00000000000000000000000000000000000000000000000000000000000000000000000000000000000
0000000000000000000000000000000
>P07374_4gy7_0_839
MKLSPREVEKLGLHNAGYLAQKRLARGVRLNYTEAVALIASQIMEYARDGEKTVAQLMCL
GQHLLGRRQVLPAVPHLLNAVQVEATFPDGTKLVTVHDPISRENGELQEALFGSLLPVPSL
DKFAETKEDNRIPGEILCEDECLTLNIGRKAVILKVTSKGDRPIQVGSHYHFIEVNPYLTFDRR
KAYGMRLNIAAGTAVRFEPGDCKSVTLVSIEGNKVIRGGNAIADGPVNETNLEAAMHAVR
SKGFGHEEEKDASEGFTKEDPNCPFNTFIHRKEYANKYGPTTGDKIRLGDTNLLAEIEKDYA
LYGDECVFGGGKVIRDGMGQSCGHPPAISLDTVITNAVIIDYTGIIKADIGIKDGLIASIGKAG

NPDIMNGVFSNMIIGANTEVIAGEGLIVTAGAIDCHVHYICPQLVYEAISSGITTLVGGGTGP
AAGTRATTCTPSPTQMRLMLQSTDDLPLNFGFTGKGSSSKPDELHEIIKAGAMGLKLHED
WGSTPAAIDNCLTIAEHHDIQINIHTDTLNEAGFVEHSIAAFKGRTIHTYHSEGAGGGHAP
DIIKVCGIKNVLPSSTNPTRPLTSNTIDEHLDMLMVCHHLDREIPEDLAFAHSRIRKKTIAAE
DVLNDIGAISIISSDSQAMGRVGEVISRTWQTADKMKAQTGPLKCDSSDNDNFRIRRYIAKY
TINPAIANGFSQYVGSVEVGKLADLVMWKPSFFGTKPEMVIKGGMVAWADIGDPNASIPTP
EPVKMRPMYGTLGKAGGALSIAFVSKAALDQRVNVLYGLNKRVEAVSNVRKLTKLDMKL
NDALPEITVDPESYTVKADGKLLCVSEATTVPLSRNYFLF
00000000000000000000000000000000000000000000000000000000000000000000000000000000000
00000000000000000000000000000000000000000001111000000000000000000000000000000000000
00000000000000000000000000000000000000000000000000000000000000000000000000000000000
00000000000000000000000000000000000000000000000000000000000000000000000000000000000
00000000000000000000000000000000000000000000000000000000000000000000000000000000000
00000000000000000000000000000000000000000000000000000000000000000000000000000000000
00000000000000000000000000000000000000000000000000000000000000000000000000000000000
00000000000000000000000000000000000000000000000000000000000000000000000000000000000
00000000000000000000000000000000000000000000000000000000000000000000000000000000000
00000000000000000000000000000000000000000000000000000000000000000000000000000000000
0000000000
>P08170_1fgq_0_838
MFSAGHKIKGTVVLMPKNELEVNPDGSAVDNLNAFLGRSVSLQLISATKADAHGKGKVG
KDTFLEGINTSLPTLGAGESAFNIHFEWDGSMGIPGAFYIKNYMQVEFFLKSLTLEAISNQGT
IRFVCNSWVYNTKLYKSVRIFFANHTYVPSETPAPLVSYREEELKSLRGNGTGERKEYDRIYD
YDVYNDLGNPDKSEKLARPVLGGSSTFPYPRRGRTGRGPTVTDPNTEKQGEVFYVPRDENL
GHLKSKDALEIGTKSLSQIVQPAFESAFDLKSTPIEFHSFQDVHDLYEGGIKLPRDVISTIIPLP
VIKELYRTDGQHILKFPQPHVVQVSQSAWMTDEEFAREMIAGVNPCVIRGLEEFPPKSNLDP
AIYGDQSSKITADSLDLDGYTMDEALGSRRLFMLDYHDIFMPYVRQINQLNSAKTYATRTIL
FLREDGTLKPVAIELSLPHSAGDLSAAVSQVVLPAKEGVESTIWLLAKAYVIVNDSCYHQLM
SHWLNTHAAMEPFVIATHRHLSVLHPIYKLLTPHYRNNMNINALARQSLINANGIIETTFL
PSKYSVEMSSAVYKNWVFTDQALPADLIKRGVAIKDPSTPHGVRLLIEDYPYAADGLEIWAA
IKTWVQEYVPLYYARDDDVKNDSELQHWWKEAVEKGHGDLKDKPWWPKLQTLEDLVEV
CLIIIWIASALHAAVNFGQYPYGGLIMNRPTASRRLLPEKGTPEYEEMINNHEKAYLRTITSK
LPTLISLSVIEILSTHASDEVYLGQRDNPHWTSDSKALQAFQKFGNKLKEIEEKLVRRNNDPS
LQGNRLGPVQLPYTLLYPSSEEGLTFRGIPNSISI
11111000000000000000111111111100000000000000000000000000000000000000000000000000000
00000000000000000000000000000000011110000000000000000000000000000000000000000000000
00000000000000000000000000000000000000000000000000000000000000000000000000000000000
00000000000000000000000000000000000000000000000000000000000000000000000000000000000
00000000000000000000000000000000000000000000000000000000000000000000000000000000000
00000000000000000000000000000000000000000000000000000000000000000000000000000000000
00000000000000000000000000000000000000000000000000000000000000000000000000000000000
00000000000000000000000000000000000000000000000000000000000000000000000000000000000
00000000000000000000000000000000000000000000000000000000000000000000000000000000000
00000000000000000000000000000000000000000000000000000000000000000000000000000000000
000000000
>Q8AAK6_2vjx_25_863
QGNDTSEVMLLDTGWEFSQSGTEKWMPATVPGTVHQDLISHELLPNPFYGMNEKKIQWV
ENEDWEYRTSFIVSEEQLNRDGIQLIFEGLDTYADVYLNGSLLLKADNMFVGYTLPVKSVLR
KGENHLYIYFHSPIRQTLPQYASNGFNYPADNDHHEKHLSVFSRKAPYSYGWDWGIRMVT
SGVWRPVTLRFYDIATISDYYVRQLSLTDENARLSNELIVNQIVPQKIPAEVRVNVSLNGTTV
TEVKQQVTLQPGINHITLPAEVTNPVRWMPNGWGTPTLYDFSAQIACGDRIVAEQSHRIGL

RTIRVVNEKDKDGESFYFEVNGIPMFAKGANYIPQDALLPNVTTERYQTLFRDMKEANMN
MVRIWGGGTYENNLFYDLADENGILVWQDFMFACTPYPSDPTFLKRVEAEAVYNIRRLRN
HASLAMWCGNNEILEALKYWGFEKKFTPEVYQGLMHGYDKLFRELLPSTVKEFDSDRFYV
HSSPYLANWGRPESWGTGDSHNWGVWYGKKPFESLDTDLPRFMSEFGFQSFPEMKTIAAF
AAPEDYQIESEVMNAHQKSSIGNSLIRTYMERDYIIPESFEDFVYVGLVLQGQGMRHGLEA
HRRNRPYCMGTLYWQLNDSWPVVSWSSIDYYGNWKALHYQAKRAFAPVLINPIQQNDSL
SVYLISDRLDTMEQMTLEMKVVDFDGKTLGKKIQVHSLEVPANTSKCVYRAKLDGWLTPE
DCRRSFLKLILKDKSGHQVAESVHFFRKTKDLQLPPTSVSYQMKQTDGKCELTLFSSMLAK
DIFIETPLQGARYSDNFFDLLPGERKKVIITSPRIKKGEELPVNIKHIRETYK
11000000000000000000000000000000000000000000000000000000000000000000000000000000
00000000000000000000000000000000000000000000000000000000000000000000000000000000
00000000000000000000000000000000000000000000000000000000000000000000000000000000
00000000000000000000000000000000000000000000000000000000000000000000000000000000
00000000000000000000000000000000000000000000000000000000000000000000000000000000
00000000000000000000000000000000000000000000000000000000000000000000000000000000
00000000000000000000000000000000000000000000000000000000000000000000000000000000
00000000000000000000000000000000000000000000000000000000000000000000000000000000
00000000000000000000000000000000000000000000000000000000000000000000000000000000
00000000000000000000000000000000000000000000000000000000000000000000000000000000
000000000
>Q9AGP8_1pj5_0_829
MASTPRIVIIGAGIVGTNLADELVTRGWNNITVLDQGPLNMPGGSTSHAPGLVFQTNPSKT
MASFAKYTVEKLLSLTEDGVSCFNQVGGLEVATTETRLADLKRKLGYAAAWGIEGRLLSPA
ECQELYPLLDGENILGGLHVPSDGLASAARAVQLLIKRTESAGVTYRGSTTVTGIEQSGGRV
TGVQTADGVIPADIVVSCAGFWGAKIGAMIGMAVPLLPLAHQYVKTTPVPAQQGRNDQP
NGARLPILRHQDQDLYYREHGDRYGIGSYAHRPMPVDVDTLGAYAPETVSEHHMPSRLDF
TLEDFLPAWEATKQLLPALADSEIEDGFNGIFSFTPDGGPLLGESKELDGFYVAEAVWVTHS
AGVAKAMAELLTTGRSETDLGECDITRFEDVQLTPEYVSETSQQNFVEIYDVLHPLQPRLSP
RNLRVSPFHARHKELGAFFLEAGGWERPYWFEANAALLKEMPAEWLPPARDAWSGMFSS
PIAAAEAWKTRTAVAMYDMTPLKRLEVSGPGALKLLQELTTADLAKKPGAVTYTLLLDHA
GGVRSDITVARLSEDTFQLGANGNIDTAYFERAARHQTQSGSATDWVQVRDTTGGTCCIGL
WGPLARDLVSKVSDDDFTNDGLKYFRAKNVVIGGIPVTAMRLSYVGELGWELYTSADNGQ
RLWDALWQAGQPFGVIAAGRAAFSSLRLEKGYRSWGTDMTTEHDPFEAGLGFAVKMAKE
SFIGKGALEGRTEEASARRLRCLTIDDGRSIVLGKEPVFYKEQAVGYVTSAAYGYTVAKPIAY
SYLPGTVSVGDSVDIEYFGRRITATVTEDPLYDPKMTRLRG
11100000000000000000000000000000000000000000000000000000000000000000000000000000
00000000000000000000000000000000000000000000000000000000000000000000000000000000
00000000000000000000000000000000000000000000000000000000000000000000000000000000
00000000000000000000000000000000000000000000000000000000000000000000000000000000
00000000000000000000000000000000000000000000000000000000000000000000000000000000
00000000000000000000000000000000000000000000000000000000000000000000000000000000
00000000000000000000000000000000000000000000000000000000000000000000000000000000
00000000000000000000000000000000000000000000000000000000000000000000000000000000
00000000000000000000000000000000000000000000000000000000000000000000000000000000
00000000000000000000000000000000000000000000000000000000000000000000000000000000
>C5IXN7_4i3g_1_827
TGKTRIPRVRRGRTTPRAFTLAVVGTLLAGTTVAAAAPGAADTANVQYTSRAAELVAQMT
LDEKISFVHWALDPDRQNVGYLPGVPRLGIPELRAADGPNGIRLVGQTATALPAPVALASTF
DDTMADSYGKVMGRDGRALNQDMVLGPMMNNIRVPHGGRNYETFSEDPLVSSRTAVAQI
KGIQGAGLMTTAKHFAANNQENNRFSVNANVDEQTLREIEFPAFEASSKAGAASFMCAY
NGLNGKPSCGNDELLNNVLRTQWGFQGWVMSDWLATPGTDAITKGLDQEMGVELPGD

VPKGEPSPPAKFFGEALKTAVLNGTVPEAAVTRSAERIVGQMEKFGLLLATPAPRPERDKAG
AQAVSRKVAENGAVLLRNEGQALPLAGDAGKSIAVIGPTAVDPKVTGLGSAHVVPDSAAA
PLDTIKARAGAGATVTYETGEETFGTQIPAGNLSPAFNQGHQLEPGKAGALYDGTLTVPAD
GEYRIAVRATGGYATVQLGSHTIEAGQVYGKVSSPLLKLTKGTHKLTISGFAMSATPLSLELG
WVTPAAADATIAEAVESARKARTAVVFAYDDGTEGVDRPNLSLPGTQDKLISAVADANPN
TIVVLNTGSSVLMPWLSKTRAVLDMWYPGQAGAEATAALLYGDVNPSGKLTQSFPAAENQ
HAVAGDPTSYPGVDNQQTYREGIHVGYRWFDKENVKPLFPFGHGLSYTSFTQSAPTVVRTS
AGGLKVTVTVRNSGKRAGQEVVQAYLGASPNVTAPQAKKKLVGYTKVSLAAGEAKTVTV
NVDRRQLQFWDAATDNWKTGTGNRLLQTGSSSADLRGSATVNVW
11111111111111111111111111111111111111111111100000000000000000000000000000000000
00000000000000000000000000000000000000000000000000000000000000000000000000000000
00000000000000000000000000000000000000000000000000000000000000000000000000000000
00000000000000000000000000000000000000000000000000000000000000000000000000000000
00000000000000000000000000000000000000000000000000000000000000000000000000000000
00000000000000000000000000000000000000000000000000000000000000000000000000000000
00000000000000000000000000000000000000000000000000000000000000000000000000000000
00000000000000000000000000000000000000000000000000000000000000000000000000000000
00000000000000000000000000000000000000000000000000000000000000000000000000000000
00000000000000000000000000000000000000000000000000000000000000000000000000000
>D6PAH1_3ahc_0_824
MTNPVIGTPWQKLDRPVSEEAIEGMDKYWRVTNYMSIGQIYLRSNPLMKEPFTRDDVKHR
LVGHWGTTPGLNFLLAHINRLIADHQQNTVFIMGPGHGGPAGTSQSYVDGTYTEYYPNIT
KDEAGLQKFFRQFSYPGGIPSHFAPETPGSIHEGGELGYALSHAYGAVMNNPSLFVPCIIGD
GEAETGPLATGWQSNKLVNPRTDGIVLPILHLNGYKIANPTILARISDEELHDFFRGMGYHP
YEFVAGFDNEDHMSIHRRFAELFETIFDEICDIKAAAQTDDMTRPFYPMLIFRTPKGWTCPK
FIDGKKTEGSWRAHQVPLASARDTEEHFEVLKGWMESYKPEELFNADGSIKDDVTAFMPK
GELRIGANPNANGGVIREDLKLPELDQYEVTGVKEYGHGWGQVEAPRALGAYCRDIIKNN
PDSFRIFGPDETASNRLNATYEVTDKQWDNGYLSGLVDEHMAVTGQVTEQLSEHQCEGFL
EAYLLTGRHGIWSSYESFVHVIDSMLNQHAKWLEATVREIPWRKPISSVNLLVSSHVWRQD
HNGFSHQDPGVTSLLINKTFNNDHVTNIYFATDANMLLAISEKCFKSTNKINAIFAGKQPA
PTWVTLDEARAELEAGAAEWKWASNAENNDEVQVVLASAGDVPTQELMAASDALNKM
GIKFKVVNVVDLLKLQSRENNDEALTDEEFTELFTADKPVLFAYHSYAQDVRGLIYDRPNH
DNFHVVGYKEQGSTTTPFDMVRVNDMDRYALQAAALKLIDADKYADKIDELNAFRKKAF
QFAVDNGYDIPEFTDWVYPDVKVDETQMLSATAATAGDNE
11110000000000000000000000000000000000000000000000000000000000000000000000000000
00000000000000000000000000000000000000000000000000000000000000000000000000000000
00000000000000000000000000000000000000000000000000000000000000000000000000000000
00000000000000000000000000000000000000000000000000000000000000000000000000000000
00000000000000000000000000000000000000000000000000000000000000000000000000000000
00000000000000000000000000000000000000000000000000000000000000000000000000000000
00000000000000000000000000000000000000000000000000000000000000000000000000000000
00000000000000000000000000000000000000000000000000000000000000000000000000000000
00000000000000000000000000000000000000000000000000000000000000000000000000000000
000000000000000000000000000000000000000000000000000000000111111111111111111
>Q9R1E6_3nkr_35_861
AEWDEGPPTVLSDSPWTNTSGSCKGRCFELQEVGPPDCRCDNLCKSYSSCCHDFDELCLKT
ARGWECTKDRCGEVRNEENACHCSEDCLSRGDCCTNYQVVCKGESHWVDDDCEEIRVPE
CPAGFVRPPLIIFSVDGFRASYMKKGSKVMPNIEKLRSCGTHAPYMRPVYPTKTFPNLYTLA
TGLYPESHGIVGNSMYDPVFDATFHLRGREKFNHRWWGGQPLWITATKQGVRAGTFFWS
VSIPHERRILTILQWLSLPDNERPSVYAFYSEQPDFSGHKYGPFGPEMTNPLREIDKTVGQLM
DGLKQLKLHRCVNVIFVGDHGMEDVTCDRTEFLSNYLTNVDDITLVPGTLGRIRPKIPNNL

KYDPKAIIANLTCKKPDQHFKPYMKQHLPKRLHYANNRRIEDLHLLVERRWHVARKPLDV
YKKPSGKCFFQGDHGFDNKVNSMQTVFVGYGPTFKYRTKVPPFENIELYNVMCDLLGLKP
APNNGTHGSLNHLLRTNTFRPTLPEEVSRPNYPGIMYLQSDFDLGCTCDDKNKLEELNKRL
HTKGSTEERHLLYGRPAVLYRTSYDILYHTDFESGYSEIFLMPLWTSYTISKQAEVSSIPEHLTN
CVRPDVRVSPGFSQNCLAYKNDKQMSYGFLFPPYLSSSPEAKYDAFLVTNMVPMYPAFKRV
WTYFQRVLVKKYASERNGVNVISGPIFDYNYNGLRDIEDEIKQYVEGSSIPVPTHYYSIITSCL
DFTQPADKCDGPLSVSSFILPHRPDNDESCNSSEDESKWVEELMKMHTARVRDIEHLTGLD
FYRKTSRSYSEILTLKTYLHTYESEI
11111111111111100000000000000000000000000000000000000000000000000000000000000000000
00000000000000000000000000000000000000000000000000000000000000000000000000000000000
00000000000000000000000000000000000000000000000000000000000000000000000000000000000
00000000000000000000000000000000000000000000000000000000000000000000000000000000000
00000000000000000000000000000000000000000000000000000000000000000000000000000000000
00000000111111111000000000000000000000000000000000000000000000000000000000000000000
00000000000000000000000000000000000011111111111111111000000000000000000000000000000
00000000000000000000000000000000000000000000000000000000000000000000000000000000000
00000000000000000000000000000000000000000000000000000000000000000000000000000000000
0000000000000000000000000000000000000000000000000000000000000000000000000111
>Q7SIF4_1g8k_3_824
PNDRITLPPANAQRTNMTCHFCIVGCGYHVYKWPELQEGGRAPEQNALGLDFRKQLPPL
AVTLTPAMTNVVTEHNGRRYNIMVVPDKACVVNSGLSSTRGGKMASYMYTPTGDGKQRL
KAPRLYAADQWVDTTWDHAMALYAGLIKKTLDKDGPQGVFFSCFDHGGAGGGFENTWG
TGKLMFSAIQTPMVRIHNRPAYNSECHATREMGIGELNNAYEDAQLADVIWSIGNNPYESQ
TNYFLNHWLPNLQGATTSKKKERFPNENFPQARIIFVDPRETPSVAIARHVAGNDRVLHLA
IEPGTDTALFNGLFTYVVEQGWIDKPFIEAHTKGFDDAVKTNRLSLDECSNITGVPVDMLKR
AAEWSYKPKASGQAPRTMHAYEKGIIWGNDNYVIQSALLDLVIATHNVGRRGTGCVRMG
GHQEGYTRPPYPGDKKIYIDQELIKGKGRIMTWWGCNNFQTSNNAQALREAILQRSAIVK
QAMQKARGATTEEMVDVIYEATQNGGLFVTSINLYPTKLAEAAHLMLPAAHPGEMNLTS
MNGERRIRLSEKFMDPPGTAMADCLIAARIANALRDMYQKDGKAEMAAQFEGFDWKTE
EDAFNDGFRRAGQPGAPAIDSQGGSTGHLVTYDRLRKSGNNGVQLPVVSWDESKGLVGTE
MLYTEGKFDTDDGKAHFKPAPWNGLPATVQQQKDKYRFWLNNGRNNEVWQTAYHDQY
NSLMQERYPMAYIEMNPDDCKQLDVTGGDIVEVYNDFGSTFAMVYPVAEIKRGQTFMLFG
YVNGIQGDVTTDWTDRNIIPYYKGTWGDIRKVGSMEEFKRTVSFKSRRF
10000000000000000000000000000000000000000000000000000000000000000000000000000000000
00000000000000000000000000000000000000000000000000000000000000000000000000000000000
00000000000000000000000000000000000000000000000000000000000000000000000000000000000
00000000000000000000000000000000000000000000000000000000000000000000000000000000000
00000000000000000000000000000000000000000000000000000000000000000000000000000000000
00000000000000000000000000000000000000000000000000000000000000000000000000000000000
00000000000000000000000000000000000000000000000000000000000000000000000000000000000
00000000000000000000000000000000000000000000000000000000000000000000000000000000000
00000000000000000000000000000000000000000000000000000000000000000000000000000000000
000000000000000000000000000000000000000000000000000000000000000000000000000
>B3PEE6_4ba0_0_815
MFRRIAGFSPIFLMLFGSSLPTMGNPVKREIHPDAVFYKEHKLRNDGLVITTNQGNIRLQFKS
EAAIEVLYRADSKQLPSFALAQPESAIKAQLTETENHLQFSGGTLTARIQKRPFAISYYRDSEL
LLAEESGFQVNTDKINFRFYLSPGEKILGGGQRILGMDRRGQRFPLYNRAHYGYSDHSGQM
YFGLPAIMSSKQYILVFDNSASGAMDIGKTESDILQLEAKSGRSAYILVAGNSYPSLIENFTQV
TGRQPLPPRWALGSFASRFGYRSEAETRATVQKYKTEDFPLDTIVLDLYWFGKDIKGHMGN
LDWDKENFPTPLDMMADFKQQGVKTVLITEPFVLTSSKRWDDAVKAKALAKDPQGQPKA
FELYFGNGGIIDVFSKEGSRWFSSIYKDLSKQGVAGWWGDLGEPEMHPEDTQHAIGDADTV

HNAYGHRWAEMLYQQQLDQFPELRPFIMMRAGFVGSQRYGMIPWTGDVSRTWGGLASQ
VELALQMSLLGFGYIHSDLGGFADGETLDKEMYIRWLQYGVFQPVYRPHGQDHIPSEPVFQ
DEETKAILRPLVKLRYRMLPYIYTAAYQNTLTGMPLMRPLFFSDEKNPALIDNKTSYFWGDS
LLVTPITQAGVESVSIPAPKGVWFDFWKDTRYQTDGAPLTLPTDLHTIPVLVKAGAFMPYVP
AVSTTEDYRSDSLEIHYYADASVPLAQGEIFEDDGKDPNSIKRNQFDLLTLQATHTDNQLHF
QLARTGKGYRGMPERRATTLVIHNASDQYQHLDINGKTIAIAQADCASTPALACYDQERR
QLQLVFTWGREALNLRLH
1111111111111111111111111111111111000000000000000000000000000000000000000000000000000
0000000000000000000000000000000000000000000000000000000110000000000000000000000000
00000000000000000000000000000000000000000000000000000000000000000000000000000000000
00000000000000000000000000000000000000000000000000000000000000000000000000000000000
00000000000000000000000000000000000000000000000000000000000000000000000000000000000
00000000000000000000000000000000000000000000000000000000000000000000000000000000000
00000000000000000000000000000000000000000000000000000000000000000000000000000000000
00000000000000000000000000000000000000000000000000000000000000000000000000000000000
00000000000000000000000000000000000000000000000000000000000000000000000000000000000
000000000000000000000000000000000000000000000000000000000000000000000
>F8UWD6_3ucp_27_842
GGSDGKDGEDGKPGPVGLDISQATTLKATLEDVKIDNGTVSVDIVLTNANGVPVTGLEQYA
QINAIGLGIAKLTPESGKGYKTPQWVSYINSVKAADPARSLANYSYTDGKDSAGNPITKEVK
FTPGDAIQANIESSCKTTCLTVVDSGVVYRYTFQTNLSTLPAIEGLDLTYDPTLIHRITLELQTD
GSKDAKLVNSHIDFLPSDNFRVAKETETRTVVDLEANCIKCHSTNYSDTSSTAKPLALHGGR
RIGIANCQVCHTSYSKDPETGSPLDMGAMVHAIHKGTYAMVGYSGTAYDFSGTMAKAAA
ESGYPQYREGKDVSERVTLPVSIGNCQSCHSTDDKGPVDAASFKHHKGLACASCHMSGFN
PVDNSEWLTPPEGQKDRGFVGNYFHYYATPEIDGIPGVNLVHVFQNGGCASCHAEQGEEG
SAKYHLAKANATKLLRTEYAYKLENGTFDVAKGELTFTVNWHSDVAPHQDPKVKEFWVS
LTAFNGTEYTMGPRPSNGTLGRSENRISVNLAKVETNANLTAVPNGSKVTYTLTGIKAVIGT
SSVPYKQIVSIGKGFMDGKLLICANSAELDPTMDAAIDCSNTEAPIYEVIVGSNKASFSADAS
NVTARSIVISEAKCANCHGEKADFSASHALTHAADKPDNSCGTCHSAVPNTAVALADGSC
VACHNGAPAHSKKPFERGFDFKVMIHQIHADTRSVRRLTTDAATFPENPANCAACHDKG
QLSLATLGNKPAFLASTGEYSPTVAACASCHATTATDSAVIGHFETNGGVYNAAAGTYTPG
SETCATCHGEGKSFGVDKVHPVKY
11111111111111110000000000000000000000000000000000000000000000000000000000000000000
00000000000000000000000000000000000000000000000000000000000000000000000000000000000
00000000000000000000000000000000000000000000000000000000000000000000000000000000000
00000000000000000000000000000000000000000000000000000000000000000000000000000000000
00000000000000000000000000000000000000000000000000000000000000000000000000000000000
00000000000000000000000000000000000000000000000000000000000000000000000000000000000
00000000000000000000000000000000000000000000000000000000000000000000000000000000000
00000000000000000000000000000000000000000000000000000000000000000000000000000000000
00000000000000000000000000000000000000000000000000000000000000000000000000000000000
000000000000000000000000000000000000000000000000000000000000000000000
>P39185_3o5a_29_830
ANFVTDSEVTKLKWSKAPCRFCGTGCGVTVAVKDNKVVATQGDPQAEVNKGLNCVKGYF
LSKIMYGQDRLTRPLMRMKNGKYDKNGDFAPVTWDQAFDEMERQFKRVLKEKGPTAVG
MFGSGQWTVWEGYAAAKLYKAGFRSNNIDPNARHCMASAAAGFMRTFGMDEPMGCYD
DFEAADAFVLWGSNMAEMHPILWTRVTDRRLSHPKTRVVVLSTFTHRCFDLADIGIIFKPQ
TDLAMLNYIANYIIRNNKVNKDFVNKHTVFKEGVTDIGYGLRPDHPLQKAAKNASDPGA
AKVITFDEFAKFVSKYDADYVSKLSAVPKAKLDQLAELYADPNIKVMSLWTMGFNQHTRG
TWANNMVYNLHLLTGKIATPGNSPFSLTGQPSACGTAREVGTFSHRLPADMVVTNPKHRE
EAERIWKLPPGTIPDKPGYDAVLQNRMLKDGKLNAYWVQVNNNMQAAANLMEEGLPG

YRNPANFIVVSDAYPTVTALAADLVLPSAMWVEKEGAYGNAERRTQFWHQLVDAPGEAR
SDLWQLVEFAKRFKVEEVWPPELIAKKPEYKGKTLYDVLYRNGQVDKFPLKDVNAEYHNA
EAKAFGFYLQKGLFEEYATFGRGHGHDLAPFDAYHEARGLRWPVVNGKETRWRYREGSD
PYVKAGTGFQFYGNPDGKAVIFALPYEPPAESPDKEYPYWLVTGRVLEHWHSGSMTRRVPE
LYRSFPNAVVFMHPEDAKALGLRRGVEVEVVSRRGRMRSRIETRGRDAPPRGLVFVPWFDA
SQLINKVTLDATCPISLQTDFKKCAVKIVKV
1111111111000000000000000000000000000000000000000000000000000000000000000000000000
0000000000000000000000000000000000000000000000000000000000000000000000000000000000
0000000000000000000000000000000000000000000000000000000000000000000000000000000000
0000000000000000000000000000000000000000000000000000000000000000000000000000000000
0000000000000000000000000000000000000000000000000000000000000000000000000000000000
0000000000000000000000000000000000000000000000000000000000000000000000000000000000
0000000000000000000000000000000000000000000000000000000000000000000000000000000000
0000000000000000000000000000000000000000000000000000000000000000000000000000000000
0000000000000000000000000000000000000000000000000000000000000000000000000000000000
000000000000000000000000000000000000000000000000000000
>Q96X16_1n9e_0_786
MRLTNLLSLTTLVALAVAVPDFYQKREAVSSKEAALLRRDASAECVSNENVEIEAPKTNIWT
SLAKEEVQEVLDLLHSTYNITEVTKADFFSNYVLWIETLKPNKTEALTYLDEDGDLPPRNAR
TVVYFGEGEEGYFEELKVGPLPVSDETTIEPLSFYNTNGKSKLPFEVGHLDRIKSAAKSSFLN
KNLNTTIMRDVLEGLIGVPYEDMGCHSAAPQLHDPATGATVDYGTCNINTENDAENLVPT
GFFFKFDMTGRDVSQWKMLEYIYNNKVYTSAEELYEAMQKDDFVTLPKIDVDNLDWTVIQ
RNDSAPIRHLDDRKSPRLVEPEGRRWAYDGEEEYFSWMDWGFYTSWSRDTGISFYDITFKGE
RIVYELSLQELIAEYGSDDPFNQHTFYSDISYGVGNRFSLVPGYDCPATAGYFTTDTFEYDEFY
NRTLSYCVFENQEDYSLLRHTGASYSAITQNPTLNVRFISTIGNYDYNFLYKFFLDGTLEVSV
RAAGYIQAGYWNPETSAPYGLKIHDVLSGSFHDHVLNYKVDLDVGGTKNRASKYVMKDV
DVEYPWAPGTVYNTKQIAREVLEKEDFNGINWPENGQGILLIESAEETNSFGNPRAYNIMP
GGGGVHRIVKNSRSGPETQNWARSNLFLTKHKDEELRSSTALNTNALYDPPVNFNAFLDD
ESLDGEDIVAWVNLGLHHLPNSNDLPNTIFSTAHASFMLTPFNYFDSENSRDTTQQVFYTY
DDETEESNWEFYGNDWSSCGLEVPEPNFEDYTYGRGTRINKKMTNSDEVY
1111111111111111111111111111111111111111000000000000000000000000000000000000000000
0000000000000000000000000000000000000000000000000000000000000000000000000000000000
0000000000000000000000000000000000000000000000000000000000000000000000000000000000
0000000000000000000000000000000000000000000000000000000000000000000000000000000000
0000000000000000000000000000000000000000000000000000000000000000000000000000000000
0000000000000000000000000000000000000000000000000000000000000000000000000000000000
0000000000000000000000000000000000000000000000000000000000000000000000000000000000
0000000000000000000000000000000000000000000000000000000000000000000000000000000000
0000000000000000000000000000000000000000000000000000000000000000000000000000000000
0000000000000000000000000000111111111111
>P0C6E9_1w0o_0_780
MRFKNVKKTALMLAMFGMATSSNAALFDYNATGDTEFDSPAKQGWMQDNTNNGSGVL
TNADGMPAWLVQGIGGRAQWTYSLSTNQHAQASSFGWRMTTEMKVLSGGMITNYYANG
TQRVLPIISLDSSGNLVVEFEGQTGRTVLATGTAATEYHKFELVFLPGSNPSASFYFDGKLIRD
NIQPTASKQNMIVWGNGSSNTDGVAAYRDIKFEIQGDVIFRGPDRIPSIVASSVTPGVVTAFA
EKRVGGGDPGALSNTNDIITRTSRDGGITWDTELNLTEQINVSDEFDFSDPRPIYDPSSNTVL
VSYARWPTDAAQNGDRIKPWMPNGIFYSVYDVASGNWQAPIDVTDQVKERSFQIAGWGG
SELYRRNTSLNSQQDWQSNAKIRIVDGAANQIQVADGSRKYVVTLSIDESGGLVANLNGVS
APIILQSEHAKVHSFHDYELQYSALNHTTTLFVDGQQITTWAGEVSQENNIQFGNADAQID
GRLHVQKIVLTQQGHNLVEFDAFYLAQQTPEVEKDLEKLGWTKIKTGNTMSLYGNASVNP
GPGHGITLTRQQNISGSQNGRLIYPAIVLDRFFLNVMSIYSDDGGSNWQTGSTLPIPFRWKSS

SILETLEPSEADMVELQNGDLLLTARLDFNQIVNGVNYSPRQQFLSKDGGITWSLLEANNA
NVFSNISTGTVDASITRFEQSDGSHFLLFTNPQGNPAGTNGRQNLGLWFSFDEGVTWKGPI
QLVNGASAYSDIYQLDSENAIVIVETDNSNMRILRMPITLLKQKLTLSQN
11111111111111111111111000000000000000000000000000000000000000000000000000000000
00000000000000000000000000000000000000000000000000000000000000000000000000000000
00000000000000000000000000000000000000000000000000000000000000000000000000000000
00000000000000000000000000000000000000000000000000000000000000000000000000000000
00000000000000000000000000000000000000000000000000000000000000000000000000000000
00000000000000000000000000000000000000000000000000000000000000000000000000000000
00000000000000000000000000000000000000000000000000000000000000000000000000000000
00000000000000000000000000000000000000000000000000000000000000000000000000000000
00000000000000000000000000000000000000000000000000000000000000000000000000000000
0000000000000000000000000000001111
>Q57366_1eu1_42_821
EGLANGEVMSGCHWGVFKARVENGRAVAFEPWDKDPAPSHQLPGVLDSIYSPTRIKYPMV
RREFLEKGVNADRSTRGNGDFVRVTWDEALDLVARELKRVQESYGPTGTFGGSYGWKSPG
RLHNCQVLMRRALNLAGGFVNSSGDYSTAAAQIIMPHVMGTLEVYEQQTAWPVVVENTD
LMVFWAADPMKTNEIGWVIPDHGAYAGMKALKEKGTRVICINPVRTETADYFGADVVSPR
PQTDVALMLGMAHTLYSEDLHDKDFLENCTTGFDLFAAYLTGESDGTPKTAEWAAEICGL
PAEQIRELARSFVAGRTMLAAGWSIQRMHHGEQAHWMLVTLASMIGQIGLPGGGFGLSY
HYSNGGSPTSDGPALGGISDGGKAVEGAAWLSESGATSIPCARVVDMLLNPGGEFQFNGAT
ATYPDVKLAYWAGGNPFAHHQDRNRMLKAWEKLETFIVQDFQWTATARHADIVLPATTS
YERNDIESVGDYSNRAILAMKKVVDPLYEARSDYDIFAALAERLGKGAEFTEGRDEMGWIS
SFYEAAVKQAEFKNVAMPSFEDFWSEGIVEFPITEGANFVRYADFREDPLFNPLGTPSGLIEIY
SKNIEKMGYDDCPAHPTWMEPAERLGGAGAKYPLHVVASHPKSRLHSQLNGTSLRDLYAV
AGHEPCLINPADAAARGIADGDVLRVFNDRGQILVGAKVSDAVMPGAIQIYEGGWYDPLD
PSEEGTLDKYGDVNVLSLDVGTSKLAQGNCGQTILADVEKYAGAPVTVTVFDTPKGA
11100000000000000000000000000000000000000000000000000000000000000000000000000000
00000000000000000000000000000000000000000000000000000000000000000000000000000000
00000000000000000000000000000000000000000000000000000000000000000000000000000000
00000000000000000000000000000000000000000000000000000000000000000000000000000000
00000000000000000000000000000000000000000000001110011111110000000000000000000000
00000000000000000000000000000000000000000000000000000000000000000000000000000000
00000000000000000000000000000000000000000000000000000000000000000000000000000000
00000000000000000000000000000000000000000000000000000000000000000000000000000000
00000000000000000000000000000000000000000000000000000000000000000000000000000000
000000000000000000000000000000000
>A4QUT2_3ut2_23_785
QGCPFAKRDGTVDSSLPQKRADAPETTTFGRCAVKSNQAGGGTRSHDWWPCQLRLDVLR
QFQPSQNPLGGDFDYAEAFQSLDYEAVKKDIAALMTESQDWWPADFGNYGGLFVRMAW
HSAGTYRAMDGRGGGGMGQQRFAPLNSWPDNQNLDKARRLIWPIKQKYGNKISWADL
MLLTGNVALENMGFKTLGFGGGRADTWQSDEAVYWGAETTFVPQGNDVRYNNSVDINA
RADKLEKPLAATHMGLIYVNPEGPNGTPDPAASAKDIREAFGRMGMNDTETVALIAGGH
AFGKTHGAVKGSNIGPAPEAADLGMQGLGWHNSVGDGNGPNQMTSGLEVIWTKTPTK
WSNGYLESLINNNWTLVESPAGAHQWEAVNGTVDYPDPFDKTKFRKATMLTSDLALINDP
EYLKISQRWLEHPEELADAFAKAWFKLLHRDLGPTTRYLGPEVPKESFIWQDPLPAREGDLI
DDADVDKLKAAILSTDGLDVSKLASTAMACATTYRNSDKRGGCNGARIALEPQRNWVSN
NPTQLSAVLDALKKVQSDFNGSNGNKKVSLADLIVLGGTAAVEKAAKDAGVDIKVPFSAG
RVDATQEQTDVTQFSYLEPQADGFRNYGRGTARARTEEIMVDKASQLTLTPPELTVLVGGM
RALGANYDGSDVGVFTANKGKLTPDFFVNLVDMNIAWTASGADGESWVGTDRKSRSEKY
KGSRADLVFGSHAELRAIAEVYAENGNQEKFVKDFVAAWTKVMNLDRFDLKVKK

1111111111111111111111111100000000000000000000000000000000000000000000000000000000000
00000000000000000000000000000000000000000000000000000000000000000000000000000000000
00000000000000000000000000000000000000000000000000000000000000000000000000000000000
00000000000000000000000000000000000000000000000000000000000000000000000000000000000
00000000000000000000000000000000000000000000000000000000000000000000000000000000000
00000000000000000000000000000000000000000000000000000000000000000000000000000000000
00000000000000000000000000000000000000000000000000000000000000000000000000000000000
00000000000000000000000000000000000000000000000000000000000000000000000000000000000
00000000000000000000000000000000000000000000000000000000000000000000000000000000000
0000000000000011
>P84141_1rwg_0_756
EAEPGAAEFAALRNRWVDQITGRNVIQAGDPDFAKAITALNNKAADSLAKLDVKKGRTS
VFTDLSLAKDAEMVTTYTRLSQLATAWATPTAAVFGDAAVLAAIKAGLADANTLCYNDRK
EEVGNWWSWEIGVPRALADAMVLLHAELSAAERTAYCAAIDHFVPDPWLQFPPKRGKITS
VGANRVDLCQGIIIRSLAGEDPTKLNHAVAGLSQVWQYVTSGDGIFRDGSFIQHSTTPYTGS
YGVVLLTGLSKLFSLLGGTAFEVSDPTRSIFFDAVEGSFAPVMINGAMADAVRGRSISREANT
GYDLGASAIEAILLLARAMDPATAARWRGLCAGWIARDTYRPILNSASVPRTALVKQLEAT
GVAPVAEATGHKLFPAMDRTMHRGPGWALSLALSSNRIAWYECGNGENNRGYHTGSGM
TYFYTSDLGQYDDAFWATANYNRLPGITVDTTPLPDKVEGQWGAAVPADEWSGATALGE
VAAVGQHLVGPGRTGLTARKSWFVSGDVTVCLGADISTASGAKVETIVDHRNLHQGSNTL
TTAAGTIAGTAGTVEVLGDGRWVHLEGFGGYAMLDDSPLHVLRETRSGSWSGVNINGSAT
VQQRNFATLYVNHGVGPVAGSYAYMVAPGASVDLTRKLLEGNKYSVIRNDATAQSVEFKT
AKTTAATFWKPGMAGDLGASGPACVVFSRHGNELSLAVSEPTQKAAGLTLTLPEGTWSSV
LEGAGTLGTDADGRSTLTLDTTGLSGKTKLIKLKR
11100000000000000000000000000000000000000000000000000000000000000000000000000000000
00000000000000000000000000000000000000000000000000000000000000000000000000000000000
00000000000000000000000000000000000000000000000000000000000000000000000000000000000
00000000000000000000000000000000000000000000000000000000000000000000000000000000000
00000000000000000000000000000000000000000000000000000000000000000000000000000000000
00000000000000000000000000000000000000000000000000000000000000000000000000000000000
00000000000000000000000000000000000000000000000000000000000000000000000000000000000
00000000000000000000000000000000000000000000000000000000000000000000000000000000000
00000000000000000000000000000000000000000000000000000000000000000000000000000000000
0000000000
>Q01401_3aml_65_819
MVTVVEEVDHLPIYDLDPKLEEFKDHFNYRIKRYLDQKCLIEKHEGGLEEFSKGYLKFGINT
VDGATIYREWAPAAQEAQLIGEFNNWNGAKHKMEKDKFGIWSIKISHVNGKPAIPHNSKV
KFRFRHGGGAWVDRIPAWIRYATFDASKFGAPYDGVHWDPPACERYVFKHPRPPKPDAPR
IYEAHVGMSGEEPEVSTYREFADNVLPRIRANNYNTVQLMAIMEHSYYASFGYHVTNFFAV
SSRSGTPEDLKYLVDKAHSLGLRVLMDVVHSHASNNVTDGLNGYDVGQNTHESYFHTGD
RGYHKLWDSRLFNYANWEVLRFLLSNLRYWMDEFMFDGFRFDGVTSMLYHHHGINKGFT
GNYKEYFSLDTDVDAIVYMMLANHLMHKLLPEATIVAEDVSGMPVLCRPVDEGGVGFDF
RLAMAIPDRWIDYLKNKEDRKWSMSEIVQTLTNRRYTEKCIAYAESHDQSIVGDKTIAFLLM
DKEMYTGMSDLQPASPTINRGIALQKMIHFITMALGGDGYLNFMGNEFGHPEWIDFPREG
NNWSYDKCRRQWSLVDTDHLRYKYMNAFDQAMNALEEEFSFLSSSKQIVSDMNEKDKVI
VFERGDLVFVFNFHPNKTYKGYKVGCDLPGKYRVALDSDALVFGGHGRVGHDVDHFTSPE
GMPGVPETNFNNRPNSFKVLSPPRTCVAYYRVDEDREELRRGGAVASGKIVTEYIDVEATSG
ETISGGWKGSEKDDCGKKGMKFVFRSSDEDCK
11111100000000000000000000000000000000000000000000000000000000000000000000000000000
00000000000000000000000000000000000000000000000000000000000000000000000000000000000
00000000000000000000000000000000000000000000000000000000000000000000000000000000000

00000000000000000000000000000000000000000000000000000000000000000000000000000000000
00000000000000000000000000000000000000000000000000000000000000000000000000000000000
00000000000000000000000000000000000000000000000000000000000000000000000000000000000
00000000000000000000000000000000000000000000000000000000000000000000000000000000000
00000000000000000000000000000000000000000000000000000000000000000000000000000000000
00000000000000000000000000000000000111111111111111111111111111111111111111111111111
11111111
>O95487_3eh1_517_1267
QPESLRPVNLTQERNILPMTPVWAPVPNLNADLKKLNCSPDSFRCTLTNIPQTQALLNKAK
LPLGLLLHPFRDLTQLPVITSNTIVRCRSCRTYINPFVSFIDQRRWKCNLCYRVNDVPEEFMY
NPLTRSYGEPHKRPEVQNSTVEFIASSDYMLRPPQPAVYLFVLDVSHNAVEAGYLTILCQSLL
ENLDKLPGDSRTRIGFMTFDSTIHFYNLQEGLSQPQMLIVSDIDDVFLPTPDSLLVNLYESKE
LIKDLLNALPNMFTNTRETHSALGPALQAAFKLMSPTGGRVSVFQTQLPSLGAGLLQSRED
PNQRSSTKVVQHLGPATDFYKKLALDCSGQQTAVDLFLLSSQYSDLASLACMSKYSAGCIY
YYPSFHYTHNPSQAEKLQKDLKRYLTRKIGFEAVMRIRCTKGLSMHTFHGNFFVRSTDLLSL
ANINPDAGFAVQLSIEESLTDTSLVCFQTALLYTSSKGERRIRVHTLCLPVVSSLADVYAGVD
VQAAICLLANMAVDRSVSSSLSDARDALVNAVVDSLSAYGSTVSNLQHSALMAPSSLKLFP
LYVLALLKQKAFRTGTSTRLDDRVYAMCQIKSQPLVHLMKMIHPNLYRIDRLTDEGAVHVN
DRIVPQPPLQKLSAEKLTREGAFLMDCGSVFYIWVGKGCDNNFIEDVLGYTNFASIPQKMT
HLPELDTLSSERARSFITWLRDSRPLSPILHIVKDESPAKAEFFQHLIEDRTEAAFSYYEFLLHV
QQQICK
00000000000000000000000000000000000000000000000000000000000000000000000000000000000
00000000000000000000000000000000000000001111111110000000000000000000000000000000000
00000000000000000000000000000000000000000000000000000000000000000000000000000000000
00000000000000000000000000000000000000000000000000000000000000000000000000000000000
00000000000000000000000000000000000000000000000000000000000000000000000000000000000
00000000000000000000000000000000000000000000000000000000000000000000000000000000000
00000000000000000000000000000000000000000011111000000000000000000000000000000000000
00000000000000000000000000000000000000000000000000000000000000000000000000000000000
00000000000000000000000000000000000000000000000000000000000000000000000000000000000
0000
>P0A5J4_3s9z_0_740
MTDRVSVGNLRIARVLYDFVNNEALPGTDIDPDSFWAGVDKVVADLTPQNQALLNARDEL
QAQIDKWHRRRVIEPIDMDAYRQFLTEIGYLLPEPDDFTITTSGVDAEITTTAGPQLVVPVLN
ARFALNAANARWGSLYDALYGTDVIPETDGAEKGPTYNKVRGDKVIAYARKFLDDSVPLSS
GSFGDATGFTVQDGQLVVALPDKSTGLANPGQFAGYTGAAESPTSVLLINHGLHIEILIDPES
QVGTTDRAGVKDVILESAITTIMDFEDSVAAVDAADKVLGYRNWLGLNKGDLAAAVDKD
GTAFLRVLNRDRNYTAPGGGQFTLPGRSLMFVRNVGHLMTNDAIVDTDGSEVFEGIMDAL
FTGLIAIHGLKASDVNGPLINSRTGSIYIVKPKMHGPAEVAFTCELFSRVEDVLGLPQNTMKI
GIMDEERRTTVNLKACIKAADRVVFINTGFLDRTGDEIHTSMEAGPMVRKGTMKSQPWI
LAYEDHNVDAGLAAGFSGRAQVGKGMWTMTELMADMVETKIAQPRAGASTAWVPSPTA
ATLHALHYHQVDVAAVQQGLAGKRRATIEQLLTIPLAKELAWAPDEIREEVDNNCQSILGY
VVRWVDQGVGCSKVPDIHDVALMEDRATLRISSQLLANWLRHGVITSADVRASLERMAPL
VDRQNAGDVAYRPMAPNFDDSIAFLAAQELILSGAQQPNGYTEPILHRRRREFKARAAEKP
APSDRAGDDAAR
10000000000000000000000000000000000000000000000000000000000000000000000000000000000
00000000000000000000000000000000000000000000000000000000000000000000000000000000000
00000000000000000000000000000000000000000000000000000000000000000000000000000000000
00000000000000000000000000000000000000000000000000000111111100000000000000000000000
00000000000000000000000000000000000000000000000011000000000000000000000000000000000
00000000000000000000000000000000000000000000000000000000000000000000000000000000000

00000000000000000000000000000000000000000000000000000000000000000000000000000000000
00000000000000000000000000000000000000000000000000000000000000000000000000000000000
00000000001000000000000000000000000000000000000000000000000000011111111111111
>P22033_2xij_11_749
SPHYLRQVKESSGSRLIQQRLLHQQQPLHPEWAALAKKQLKGKNPEDLIWHTPEGISIKPLY
SKRDTMDLPEELPGVKPFTRGPYPTMYTFRPWTIRQYAGFSTVEESNKFYKDNIKAGQQGLS
VAFDLATHRGYDSDNPRVRGDVGMAGVAIDTVEDTKILFDGIPLEKMSVSMTMNGAVIPV
LANFIVTGEEQGVPKEKLTGTIQNDILKEFMVRNTYIFPPEPSMKIIADIFEYTAKHMPKFNSI
SISGYHMQEAGADAILELAYTLADGLEYSRTGLQAGLTIDEFAPRLSFFWGIGMNFYMEIAK
MRAGRRLWAHLIEKMFQPKNSKSLLLRAHCQTSGWSLTEQDPYNNIVRTAIEAMAAVFGG
TQSLHTNSFDEALGLPTVKSARIARNTQIIIQEESGIPKVADPWGGSYMMECLTNDVYDAAL
KLINEIEEMGGMAKAVAEGIPKLRIEECAARRQARIDSGSEVIVGVNKYQLEKEDAVEVLAI
DNTSVRNRQIEKLKKIKSSRDQALAERCLAALTECAASGDGNILALAVDASRARCTVGEIT
DALKKVFGEHKANDRMVSGAYRQEFGESKEITSAIKRVHKFMEREGRRPRLLVAKMGQDG
HDRGAKVIATGFADLGFDVDIGPLFQTPREVAQQAVDADVHAVGISTLAAGHKTLVPELIK
ELNSLGRPDILVMCGGVIPPQDYEFLFEVGVSNVFGPGTRIPKAAVQVLDDIEKCLEKKQQS
V
11111111111111111111111100000000000000000000000000000000000000000000000000000000000
00000000000000000000000000000000000000000000000000000000000000000000000000000000000
00000000000000000000000000000000000000000000000000000000000000000000000000000000000
00000000000000000000000000000000000000000000000000000000000000000000000000000000000
00000000000000000000000000000000000000000000000000000000000000000000000000000000000
00000000000000000000000000000000000000000000000000000000000000000000000000000000000
00000000000000000000000000000000000000000000000000000000000000000000000000000000000
00000000000000000000000000000000000000000000000000000000000000000000000000000000000
000000000000000000000000000000000000000000000000000000000000000000000011111
>Q59490_1l1l_0_738
MSEEISLSAEFIDRVKASVKPHWGKLGWVTYKRTYARWLPEKGRSENWDETVKRVVEGNI
NLDPRLQDSPSLELKQSLTEEAERLYKLIYGLGATPSGRNLWISGTDYQRRTGDSLNNCWFV
AIRPQKYGDSKIVPSYLGKQEKAVSMPFSFLFDELMKGGGVGFSVARSNISQIPRVDFAIDLQ
LVVDETSESYDASVKVGAVGKNELVQDADSIYYRLPDTREGWVLANALLIDLHFAQTNPD
RKQKLILDLSDIRPYGAEIHGFGGTASGPMPLISMLLDVNEVLNNKAGGRLTAVDAADICN
LIGKAVVAGNVRRSAELALGSNDDQDFISMKQDQEKLMHHRWASNNSVAVDSAFSGYQPI
AAGIRENGEPGIVNLDLSKNYGRIVDGYQAGIDGDVEGTNPCGEISLANGEPCNLFEVFPLI
AEEQGWDLQEVFALAARYAKRVTFSPYDWEISREIIQKNRRIGISMSGIQDWLLTRLGNRVV
TGFKDDFDPETHEAIKVPVYDKRAIKMVDQLYKAVVKADQDYSKTLGCNESIKHTTVKPSG
TVAKLAGASEGMHFHYGAYLIQRIRFQDSDPLLPALKACGYRTEADIYTENTTCVEFPIKAV
GADNPNFASAGTVSIAEQFATQAFLQTYWSDNAVSCTITFQDSEGDQVESLLRQYRFITKST
SLLPYFGGSLQQAPKEPIDKETYEKRSQEITGNVEEVFSQLNSDVKDLELVDQTDCEGGACPI
K
11100000000000000000000000000000000000000000000000000000000000000000000000000000000
00000000000000000000000000000000000000000000000000000000000000000000000000000000000
00000000000000000000000000000000000000000000000000000000000000000000000000000000000
00000000000000000000000000000000000000000000000000000000000000000001111000000000000
00000000000000000000000000000000000000000000000000000000000000000000000000000000000
00000000000000000000000000000000000000000000000000000000000000000000000000000000000
00000000000000000000000000000000000000000000000000000000000000000000000000000000000
00000000000000000000000000000000000000000000000000000000000000000000000000000000000
000000000000000000000000000000000000000000000000000000000000111111111111111
>P50216_3mbc_0_737
MAKIIWTRTDEAPLLATYSLKPVVEAFAATAGIEVETRDISLAGRILAQFPERLTEDQKVGNA

LAELGELAKTPEANIIKLPNISASVPQLKAAIKELQDQGYDIPELPDNATTDEEKDILARYNA
VKGSAVNPVLREGNSDRRAPIAVKNFVKKFPHRMGEWSADSKTNVATMDANDFRHNEKS
IILDAADEVQIKHIAADGTETILKDSLKLLEGEVLDGTVLSAKALDAFLLEQVARAKAEGILF
SAHLKATMMKVSDPIIFGHVVRAYFADVFAQYGEQLLAAGLNGENGLAAILSGLESLDNG
EEIKAAFEKGLEDGPDLAMVNSARGITNLHVPSDVIVDASMPAMIRTSGHMWNKDDQEQ
DTLAIIPDSSYAGVYQTVIEDCRKNGAFDPTTMGTVPNVGLMAQKAEEYGSHDKTFRIEAD
GVVQVVSSNGDVLIEHDVEANDIWRACQVKDAPIQDWVKLAVTRSRLSGMPAVFWLDPE
RAHDRNLASLVEKYLADHDTEGLDIQILSPVEATQLSIDRIRRGEDTISVTGNVLRDYNTDLF
PILELGTSAKMLSVVPLMAGGGLFETGAGGSAPKHVQQVQEENHLRWDSLGEFLALAESF
RHELNNNGNTKAGVLADALDKATEKLLNEEKSPSRKVGEIDNRGSHFWLTKFWADELAA
QTEDADLAATFAPVAEALNTGAADIDAALLAVQGGATDLGGYYSPNEEKLTNIMRPVAQF
NEIVDALKK
100000000000000000000000000000000000000000000000000000000000000000000000000000000000000000
000000000000000000000000000000000000000000000000000000000000000000000000000000000000000000
000000000000000000000000000000000000000000000000000000000000000000000000000000000000000000
000000000000000000000000000000000000000000000000000000000000000000000000000000000000000000
000000000000000000000000000000000000000000000000000000000000000000000000000000000000000000
000000000000000000000000000000000000000000000000000000000000000000000000000000000000000000
000000000000000000000000000000000000000000000000000000000000000000000000000000000000000000
000000000000000000000000000000000000000000000000000000000000000000000000000000000000000000
000000000000000000000000000000000000000000000000000000000000000000000000000000011
>Q4QHU7_2xe4_0_730
MSSDSSVAASAQPPIAAKKPHRVTFGYVEGEDRGPNPMNPPRYREDPYFWMRDDDRKDP
AVIEHLNKEKVYFQARSADIAQLRDDIYAEHISHINEDDMSAPYVYGKYRYYTREVKGKPY
KIYCRVFTDKEPGDVAAEEVIIDVNQVAEGKAFCDVMEVKPAPPEHDLVAFSVDMSGNEVY
TIEFKRISDPSQTIADKVSGTNGEIVWGPDHTSLFYVTKDETLRENKVWRHVMGKLQSEDV
CLYEEHNPLFSAFMYKAADTNTLCIGSQSPETAEVHLLDLRKGNAHNTLEIVRPREKGVRY
DVQMHGTSHLVILTNEGGAVNHKLLIAPRGQPSDWSHVLVDHSEDVFMESIAVRSNYLVV
AGRRAGLTRIWTMMADSQDGVFKAGTGLREVVMEEPIFTVHLVESQMLEYEEPTFRMEYSS
LATPNTWFDVSPQDHSRTAVKVREVGGGFDAANYKVERRFATAPDQTKIPLSVVYHKDLD
MSQPQPCMLYGYGSYGLSMDPQFSIQHLPYCDRGMIFAIAHIRGGSELGRAWYEIGAKYLT
KRNTFSDFIAAAEFLVNAKLTTPSQLACEGRSAGGLLMGAVLNMRPDLFKVALAGVPFVD
VMTTMCDPSIPLTTGEWEEWGNPNEYKYYDYMLSYSPMDNVRAQEYPNIMVQCGLHDPR
VAYWEPAKWVSKLRECKTDNNEILLNIDMESGHFSAKDRYKFWKESAIQQAFVCKHLKST
VRLLVRR
111111111000000000000000000000000000000000000000000000000000000000000000000000000000000000
000000000000000000000000000000000000000000000000000000000000000000000000000000000000000000
000000000000000000000000000000000000000000000000000000000000000000000000000000000000000000
000000000000000000000000000000000000000000000000000000000000000000000000000000000000000000
000000000000000000000000000000000000000000000000000000000000000000000000000000000000000000
000000000000000000000000000000000000000000000000000000000000000000000000000000000000000000
000000000000000000000000000000000000000000000000000000000000000000000000000000000000000000
000000000000000000000000000000000000000000000000000000000000000000000000000000000000000000
0000000000000000000000000000000000000000000000000000000000000000000000001
>Q54873_1ojo_284_1008
ASVKDAYTDRLDDWNGIIAGNQYYDSKNEQMAKLNQELEGKVADSLSSISSQADRTYLWE
KFSNYKTSANLTATYRKLEEMAKQVTNPSSRYYQDETVVRTVRDSMEWMHKHVYNSEKSI
VGNWWDYEIGTPRAINNTLSLMKEYFSDEEIKKYTDVIEKFVPDPEHFRKTTDNPFKALGG
NLVDMGRVKVIAGLLRKDDQEISSTIRSIEQVFKLVDQGEGFYQDGSYIDHTNVAYTGAYGN
VLIDGLSQLLPVIQKTKNPIDKDKMQTMYHWIDKSFAPLLVNGELMDMSRGRSISRANSEG
HVAAVEVLRGIHRIADMSEGETKQCLQSLVKTIVQSDSYYDVFKNLKTYKDISLMQSLLSDA

GVASVPRPSYLSAFNKMDKTAMYNAEKGFGFGLSLFSSRTLNYEHMNKENKRGWYTSDG
MFYLYNGDLSHYSDGYWPTVNPYKMPGTTETDAKRADSDTGKVLPSAFVGTSKLDDANA
TATMDFTNWNQTLTAHKSWFMLKDKIAFLGSNIQNTSTDTAATTIDQRKLESGNPYKVYV
NDKEASLTEQEKDYPETQSVFLESFDSKKNIGYFFFKKSSISMSKALQKGAWKDINEGQSDK
EVENEFLTISQAHKQNRDSYGYMLIPNVDRATFNQMIKELESSLIENNETLQSVYDAKQGV
WGIVKYDDSVSTISNQFQVLKRGVYTIRKEGDEYKIAYYNPETQESAPDQEVFKKLEQ
11000000000000000000000000000000000000000000000000000000000000000000000000000000000
00000000000000000000000000000000000000000000000000000000000000000000000000000000000
00000000000000000000000000000000000000000000000000000000000000000000000000000000000
00000000000000000000000000000000000000000000000000000000000000000000000000000000000
00000000000000000000000000000000000000000000000000000000000000000000000000000000000
00000000000000000000000000000000000000000000000000000000000000000000000000000000000
00000000000000000000000000000000000000000000000000000000000000000000000000000000000
00000000000000000000000000000000000000000000000000000000000000000000000000000000000
0000000000000000000000000000000000000000000000000000000000001
>P81186_2nap_32_754
ADNRPEKWVKGVCRYCGTGCGVLVGVKDGKAVAIQGDPNNHNAGLLCLKGSLLIPVLNS
KERVTQPLVRRHKGGKLEPVSWDEALDLMASRFRSSIDMYGPNSVAWYGSGQCLTEESYVA
NKIFKGGFGTNNVDGNPRLCMASAVGGYVTSFGKDEPMGTYADIDQATCFFIIGSNTSEAH
PVLFRRIARRKQVEPGVKIIVADPRRTNTSRIADMHVAFRPGTDLAFMHSMAWVIINEELD
NPRFWQRYVNFMDAEGKPSDFEGYKAFLENYRPEKVAEICRVPVEQIYGAARAFAESAAT
MSLWCMGINQRVQGVFANNLIHNLHLITGQICRPGATSFSLTGQPNACGGVRDGGALSHL
LPAGRAIPNAKHRAEMEKLWGLPEGRIAPEPGYHTVALFEALGRGDVKCMIICETNPAHTL
PNLNKVHKAMSHPESFIVCIEAFPDAVTLEYADLVLPPAFWCERDGVYGCGERRYSLTEKA
VDPPGQCRPTVNTLVEFARRAGVDPQLVNFRNAEDVWNEWRMVSKGTTYDFWGMTRER
LRKESGLIWPCPSEDHPGTSLRYVRGQDPCVPADHPDRFFFYGKPDGRAVIWMRPAKGAA
EEPDAEYPLYLTSMRVIDHWHTATMTGKVPELQKANPIAFVEINEEDAARTGIKHGDSVIVE
TRRDAMELPARVSDVCRPGLIAVPFFDPKKLVNKLFLDATDPVSREPEYKICAARVRKA
11100000000000000000000000000000000000000000000000000000000000000000000000000000000
00000000000000000000000000000000000000000000000000000000000000000000000000000000000
00000000000000000000000000000000000000000000000000000000000000000000000000000000000
00000000000000000000000000000000000000000000000000000000000000000000000000000000000
00000000000000000000000000000000000000000000000000000000000000000000000000000000000
00000000000000000000000000000000000000000000000000000000000000000000000000000000000
00000000000000000000000000000000000000000000000000000000000000000000000000000000000
00000000000000000000000000000000000000000000000000000000000000000000000000000000000
00000000000000000000000000000000000000000000000000000000000
>O15294_3pe4_322_1040
CPTHADSLNNLANIKREQGNIEEAVRLYRKALEVFPEFAAAHSNLASVLQQQGKLQEALM
HYKEAIRISPTFADAYSNMGNTLKEMQDVQGALQCYTRAIQINPAFADAHSNLASIHKDSG
NIPEAIASYRTALKLKPDFPDAYCNLAHCLQIVCDWTDYDERMKKLVSIVADQLEKNRLPS
VHPHHSMLYPLSHGFRKAIAERHGNLCLDKINVLHKPPYEHPKDLKLSDGRLRVGYVSSDF
GNHPTSHLMQSIPGMHNPDKFEVFCYALSPDDGTNFRVKVMAEANHFIDLSQIPCNGKA
ADRIHQDGIHILVNMNGYTKGARNELFALRPAPIQAMWLGYPGTSGALFMDYIITDQETSP
AEVAEQYSEKLAYMPHTFFIGDHANMFPHLKKKAVIDFKSNGHIYDNRIVLNGIDLKAFLD
SLPDVKIVKMKCPDGGDNADSSNTALNMPVIPMNTIAEAVIEMINRGQIQITINGFSISNGL
ATTQINNKAATGEEVPRTIIVTTRSQYGLPEDAIVYCNFNQLYKIDPSTLQMWANILKRVPN
SVLWLLRFPAVGEPNIQQYAQNMGLPQNRIIFSPVAPKEEHVRRGQLADVCLDTPLCNGHT
TGMDVLWAGTPMVTMPGETLASRVAASQLTCLGCLELIAKNRQEYEDIAVKLGTDLEYLK
KVRGKVWKQRISSPLFNTKQYTMELERLYLQMWEHYAAGNKPDHMIKPVE
11000000000000000000000000000000000000000000000000000000000000000000000000000000000

00000000000000000000000000000000000000000000000000000000000000000000000000000000000
00000000000000000000000000000000000000000000000000000000000000000000000000000000000
00000000000000000000000000000000000000000000000000000000000000000000000000000000000
00000000000000000000000000000000000000000000000000000000000000000000001111000000000
00000000000000000001111111111111110000000000000000000000000000000000000000000000000
00000000000000000000000000000000000000000000000000000000000000000000000000000000000
00000000000000000000000000000000000000000000000000000000000000000000000000000000000
0000000000000000000000000000000000000000000000000000111
>P71094_2jkp_21_737
QQKLTSPDNNLVMTFQVDSKGAPTYELTYKNKVVIKPSTLGLELKKEDNTRTDFDWVDRR
DLTKLDSKTNLYDGFEVKDTQTATFDETWQPVWGEEKEIRNHYNELAVTLYQPMNDRSIVI
RFRLFNDGLGFRYEFPQQKSLNYFVIKEEHSQFGMNGDHIAFWIPGDYDTQEYDYTISRLSEI
RGLMKEAITPNSSQTPFSQTGVQTALMMKTDDGLYINLHEAALVDYSCMHLNLDDKNMV
FESWLTPDAKGDKGYMQTPCNTPWRTIIVSDDARNILASRITLNLNEPCKIADAASWVKPV
KYIGVWWDMITGKGSWAYTDELTSVKLGETDYSKTKPNGKHSANTANVKRYIDFAAAHG
FDAVLVEGWNEGWEDWFGNSKDYVFDFVTPYPDFDVKEIHRYAARKGIKMMMHHETSAS
VRNYERHMDKAYQFMADNGYNSVKSGYVGNIIPRGEHHYGQWMNNHYLYAVKKAADY
KIMVNAHEATRPTGICRTYPNLIGNESARGTEYESFGGNKVYHTTILPFTRLVGGPMDYTPG
IFETHCNKMNPANNSQVRSTIARQLALYVTMYSPLQMAADIPENYERFMDAFQFIKDVAL
DWDETNYLEAEPGEYITIARKAKDTDDWYVGCTAGENGHTSKLVFDFLTPGKQYIATVYA
DAKDADWKENPQAYTIKKGILTNKSKLNLHAANGGGYAISIKEVKDKSEAKGLKRL
00000000000000000000000000000000000000000000000111111111111111111100000000000000000
00000000000000000000000000000000000000000000000000000000000000000000000000000000000
00000000000000000000000000000000000000000000000000000000000000000000000000000000000
00000000000000000000000000000000000000000000000000000000000000000000000000000000000
00000000000000000000000000000000000000000000000000000000000000000000000000000000000
00000000000000000000000000000000000000000000000000000000000000000000000000000000000
00000000000000000000000000000000000000000000000000000000000000000000000000000000000
00000000000000000000000000000000000000000000000000000000000000000000000000000000000
00000000000000000000000000000000000000000001111111111
>P13423_3tew_29_763
EVKQENRLLNESESSSQGLLGYYFSDLNFQAPMVVTSSTTGDLSIPSSELENIPSENQYFQSAI
WSGFIKVKKSDEYTFATSADNHVTMWVDDQEVINKASNSNKIRLEKGRLYQIKIQYQRENP
TEKGLDFKLYWTDSQNKKEVISSDNLQLPELKQKSSNSRKKRSTSAGPTVPDRDNDGIPDSL
EVEGYTVDVKNKRTFLSPWISNIHEKKGLTKYKSSPEKWSTASDPYSDFEKVTGRIDKNVSP
EARHPLVAAYPIVHVDMENIILSKNEDQSTQNTDSQTRTISKNTSTSRTHTSEGFSNSNSSTV
AIDHSLSLAGERTWAETMGLNTADTARLNANIRYVNTGTAPIYNVLPTTSLVLGKNQTLATI
KAKENQLSQILAPNNYYPSKNLAPIALNAQDDFSSTPITMNYNQFLELEKTKQLRLDTDQV
YGNIATYNFENGRVRVDTGSNWSEVLPQIQETTARIIFNGKDLNLVERRIAAVNPSDPLETT
KPDMTLKEALKIAFGFNEPNGNLQYQGKDITEFDFNFDQQTSQNIKNQLAELNATNIYTVL
DKIKLNAKMNILIRDKRFHYDRNNIAVGADESVVKEAHREVINSSTEGLLLNIDKDIRKILS
GYIVEIEDTEGLKEVINDRYDMLNISSLRQDGKTFIDFKKYNDKLPLYISNPNYKVNVYAVTK
ENTIINPSENGDTSTNGIKKILIFSKKGYEIG
11111111111111000000000000000000000000000000000000000000000000000000000000000000000
00000000000000000000000000000000000000000000000000000000000000000000000000000000000
00000000000000000000000000000000000000000000000000000000000000000000000000000000000
00000000000000000000000000000000000000000000000000000000000000000000000000000000000
00000000000000000000000000000000000000000000000000000000000000000000000000000000000
00000000000000000000000000000000000000000000000000000000000000000000000000000000000
00000000000000000000000000000000000000000000000000000000000000000000000000000000000
00000000000000000000000000000000000000000000000000000000000000000000000000000000000

000000000000000000000000000000000000000000000000000
>P24481_1w27_0_715
MENGNGATTNGHVNGNGMDFCMKTEDPLYWGIAAEAMTGSHLDEVKKMVAEYRKPVV
KLGGETLTISQVAAISARDGSGVTVELSEAARAGVKASSDWVMDSMNKGTDSYGVTTGFG
ATSHRRTKQGGALQKELIRFLNAGIFGNGSDNTLPHSATRAAMLVRINTLLQGYSGIRFEILE
AITKFLNQNITPCLPLRGTITADLVPLSYIAGLLTGRPNSKAVGPTGVILSPEEAFKLAGVEGG
FFELQPKEGLALVNGTAVGSGMASMVLFEANILAVLAEVMSAIFAEVMQGKPEFTDHLTH
KLKHHPGQIEAAAIMEHILDGSAYVKAAQKLHEMDPLQKPKQDRYALRTSPQWLGPQIEV
IRSSTKMIEREINSVNDNPLIDVSRNKAIHGGNFQGTPIGVSMDNTRLAIAAIGKLMFAQFSE
LVNDFYNNGLPSNLSGGRNPSLDYGFKGAEIAMASYCSELQFLANPVTNHVQSAEQHNQ
DVNSLGLISSRKTSEAVEILKLMSTTFLVGLCQAIDLRHLEENLKSTVKNTVSSVAKRVLTMG
VNGELHPSRFCEKDLLRVVDREYIFAYIDDPCSATYPLMQKLRQTLVEHALKNGDNERNLS
TSIFQKIATFEDELKALLPKEVESARAALESGNPAIPNRIEECRSYPLYKFVRKELGTEYLTGEK
VTSPGEEFEKVFIAMSKGEIIDPLLECLESWNGAPLPIC
11111111111111111111111100000000000000000000000000000000000000000000000000000000000
00000000000000000000000000000000000000000000000000000000000000000000000000000000000
00000000000000000000000000000000000000000000000000000000000000000000000000000000000
00000000000000000000000000000000000000000000000000000000000000000000000000000000000
00000000000000000000000000000000000000000000000000000000000000000000000000000000000
00000000000000000000000000000000000000000000000000000000000000000000000000000000000
00000000000000000000000000000000000000000000000000000000000000000000000000000000000
00000000000000000000000000000000000000000000000000000000000000000000000000000000000
000000000000000000000000000000000000000000000000000
>Q5W9R9_2yr6_1_713
GVTVIPRLLGLKDEKKIATTVGEARLSGINYRHPDSALVSYPVAAAAPLGRLPAGNYRIAIVG
GGAGGIAALYELGRLAATLPAGSGIDVQIYEADPDSFLHDRPGIKAIKVRGLKAGRVSAALV
HNGDPASGDTIYEVGAMRFPEIAGLTWHYASAAFGDAAPIKVFPNPGKVPTEFVFGNRVD
RYVGSDPKDWEDPDSPTLKVLGVVAGGLVGNPQGENVAMYPIANVDPAKIAAILNAATPP
ADALERIQTKYWPEFIAQYDGLTLGAAVREIVTVAFEKGTLPPVDGVLDVDESISYYVELFGR
FGFGTGGFKPLYNISLVEMMRLILWDYSNEYTLPVTENVEFIRNLFLKAQNVGAGKLVVQV
RQERVANACHSGTASARAQLLSYDSHNAVHSEAYDFVILAVPHDQLTPIVSRSGFEHAASQ
NLGDAGLGLETHTYNQVYPPLLLSDSSPAANARIVTAIGQLHMARSSKVFATVKTAALDQP
WVPQWRGEPIKAVVSDSGLAASYVVPSPIVEDGQAPEYSSLLASYTWEDDSTRLRHDFGLY
PQNPATETGTADGMYRTMVNRAYRYVKYAGASNAQPWWFYQLLAEARTADRFVFDWTT
NKTAGGFKLDMTGDHHQSNLCFRYHTHALAASLDNRFFIASDSYSHLGGWLEGAFMSAL
NAVAGLIVRANRGDVSALSTEARPLVIGLRPVVKVPAAELATSQ
11111111111111100000000000000000000000000000000000000000000000000000000000000000000
00000000000000000000011110000000000000000000000000000000000000000000000000000000000
00000000000000000000000000000000000000000000000000000000000000000000000000000000000
00000000000000000000000000000000000000000000000000000000000000000000000000000000000
00000000000000000000000000000000000000000000000000000000000000000000000000000000000
00000000000000000000000000000000000000000000000000000000000000000000000000000000000
00000000000000000000000111100000000000000000000000000000000000000000000000000000000
00000000000000000000000000000000000000000000000000000000000000000000000000000000000
0000000000000000000000000000000000000000000111111
>Q8VP74_1gql_24_731
EDGYDMWLRYQPIADQTLLKTYQKQIRHLHVAGDSPTINAAAAELQRGLSGLLNKPIVAR
DEKLKDYSLVIGTPDNSPLIASLNLGERLQALGAEGYLLEQTRINKRHVVIVAANSDVGVLY
GSFHLLRLIQTQHALEKLSLSSAPRLQHRVVNHWDNLNRVVERGYAGLSLWDWGSLPNYL
APRYTDYARINASLGINGTVINNVNADPRVLSDQFLQKIAALADAFRPYGIKMYLSINFNSP
RAFGDVDTADPLDPRVQQWWKTRAQKIYSYIPDFGGFLVKADSEGQPGPQGYGRDHAEG

ANMLAAALKPFGGVVFWRAFVYHPDIEDRFRGAYDEFMPLDGKFADNVILQIKNGPIDFQ
PREPFSALFAGMSRTNMMMEFQITQEYFGFATHLAYQGPLFEESLKTETHARGEGSTIGNIL
EGKVFKTRHTGMAGVINPGTDRNWTGHPFVQSSWYAFGRMAWDHQISAATAADEWLR
MTFSNQPAFIEPVKQMMLVSREAGVNYRSPLGLTHLYSQGDHYGPAPWTDDLPRADWTA
VYYHRASKTGIGFNRTKTGSNALAQYPEPIAKAWGDLNSVPEDLILWFHHLSWDHRMQSG
RNLWQELVHKYYQGVEQVRAMQRTWDQQEAYVDAARFAQVKALLQVQEREAVRWRNS
CVLYFQSVAGRPIPANYEQPEHDLEYYKMLARTTYVPEPWHPASSSRVLK
00000000000000000000000000000000000000000000000000000000000000000000000000000000000
00000000000000000000000000000000000000000000000000000000000000000000000000000000000
00000000000000000000000000000000000000000000000000000000000000000000000000000000000
00000000000000000000000000000000000000000000000000000000000000000000000000000000000
00000000000000000000000000000000000000000000000000000000000000000000000000000000000
00000000000000000000000000000000000000000000000000000000000000000000000000000000000
00000000000000000000000000000000000000000000000000000000000000000000000000000000000
00000000000000000000000000000000000000000000000000000000000000000000000000000000000
00000000000000000000000000000000000001111111
>Q68XJ3_4hvt_18_719
AMEDNNKQIFNPKETKFLEEAEGVEALEWAKERTSKTEKALQAMQEYKQIKKEIETIFYDQ
RKTPYGVIRKGYVYNFWMDDKNPQGLWRRTLVDNYSKDKPNWEVLIDFDKLSKKIGKKV
AYRGVSNCFQNPNRYLISMSFGGKDEMFFREWDLEKKDFVKNGFEPITNSGKLLEGKFTYP
TWINKDTIIFNLVLHKNEITSSLYPNSLYIWKRGESIEKAKKLFEVPKEYIYVSAGKLLSDTISSS
LIFISANKDFYNYDNYILDTKYKNLKLQKINMPSDATLQGSFKEYVFWLLRSDWKFKSHNIK
AGSLVALHFTDLLKTESDKTSLKILFTPTANEVFNFISTTKDRVFLATYDNVVAKVVTFTLEN
EQWTKPVVLKLPYQNAIFGMSSYEEEEEALITIENSIVPPTIYLWVKTHELKIIRKALYSFDSEN
YVLEQKEATSFDGVKIPYFLVYKKGIKFDGKNPTLLEAYGGFQVINAPYFSRIKNEVWVKNA
GVSVLANIRGGGEFGPEWHKSAQGIKRQTAFNDFFAVSEELIKQNITSPEYLGIKGGSNGGL
LVSVAMTQRPELFGAVACEVPILDMIRYKEFGAGHSWVTEYGDPEIPNDLLHIKKYAPLENL
SLTQKYPTVLITDSVLDQRVHPWHGRIFEYVLAQNPNTKTYFLESKDSGHGSGSDLKESAN
YFINLYTFFANALKLKIN
11111111110000000000000000000000000000000000000000000000000000000000000000000000000
00000000000000000000000000000000000000000000000000000000000000000000000000000000000
00000000000000000000000000000000000000000000000000000000000000000000000000000000000
00000000000000000000000000000000000000000000000000000000000000000000000000000000000
00000000000000000000000000000000000000000000000000000000000000000000000000000000000
00000000000000000000000000000000000000000000000000000000000000000000000000000000000
00000000000000000000000000000000000000000000000000000000000000000000000000000000000
00000000000000000000000000000000000000000000000000000000000000000000000000000000000
00000000000000000000000000000000000001
>Q89YR9_4fnv_0_701
MKNIFFICFCALFAFSGCADDDDDLLTGGNVDIDLLPDAKPNDVVDPQVFEAINLNYPGLE
KVKEFYEAGEHYYAANALLEYYRTRTNVTNPNLSLINVTISEAEQAKADYALVDYRFHVN
NFYEDKETLKPYSVKQDGGINWEYSPKDASDEYQKQLHRHQWFIPQAKAYRVSGDEKYIQ
SWIEVYKNWIENNPKPTTGPNTTSWWQLQVSTRIGDQVQLLEYFKNSVNFTPEWLSTFLVE
FAEQADFLVDYPYESGGNILISQANALATAGTLMPEFKNAEKWMNTGYQILSEEVQNQIMS
DGWHKEMSLHYHIGIVADFYEAMKLAEANQLSSKLPSDFTEPLRKAAEVVMYFTYPNYFIK
GSDNVVPMFNDSWSRTRNVLKNTNFKQYVEMFPDSEELKYMQTAGNGGTAQGRTPNND
MKLFDQAGYYVLRNGWTPASTVMILSNNKSNDASNSLSAYSHNQPDNGTFELYHNGRNF
FPDSGVCTYYTSGGDNDLRYWFRGIDKHNTLSIGKQNIKKAAGKLLKSEEGATELVVFENQ
GYDNLKHRRAVFYVNKKFFVLVDEGIGNAEGTINLSFNLCEGTASEVVMDTDKNGVHTAF
SNNNNIIVRTFANKAVTCSPFTGRIAYLVDGAYNTRQSYTIDMNKSADETARYITVILPVNGS
TDTSSISAKFIDSGYSENSASVEVSVNGETHTLSYTL

11111111111111111111111111111111111111111110000000000000000000000000000000000000000
00000000000000000000000000000000000000000000000000000000000000000000000000000000000
00000000000000000000000000000000000000000000000000000000000000000000000000000000000
00000000000000000000000000000000000000000000000000000000000000000000000000000000000
00000000000000000000000000000000000000000000000000000000000000000000000000000000000
00000000000000000000000000000000000000000000000000000000000000000000000000000000000
00000000000000000000000000000000000000000000000000000000000000000000000000000000000
00000000000000000000000000000000000000000000000000000000000000000000000000000000000
00000000000000000000000000000000000000
>P04397_1z45_0_698
MTAQLQSESTSKIVLVTGGAGYIGSHTVVELIENGYDCVVADNLSNSTYDSVARLEVLTKHH
IPFYEVDLCDRKGLEKVFKEYKIDSVIHFAGLKAVGESTQIPLRYYHNNILGTVVLLELMQQY
NVSKFVFSSSATVYGDATRFPNMIPIPEECPLGPTNPYGHTKYAIENILNDLYNSDKKSWKFA
ILRYFNPIGAHPSGLIGEDPLGIPNNLLPYMAQVAVGRREKLYIFGDDYDSRDGTPIRDYIHV
VDLAKGHIAALQYLEAYNENEGLCREWNLGSGKGSTVFEVYHAFCKASGIDLPYKVTGRR
AGDVLNLTAKPDRAKRELKWQTELQVEDSCKDLWKWTTENPFGYQLRGVEARFSAEDMR
YDARFVTIGAGTRFQATFANLGASIVDLKVNGQSVVLGYENEEGYLNPDSAYIGATIGRYA
NRISKGKFSLCNKDYQLTVNNGVNANHSSIGSFHRKRFLGPIIQNPSKDVFTAEYMLIDNEK
DTEFPGDLLVTIQYTVNVAQKSLEMVYKGKLTAGEATPINLTNHSYFNLNKPYGDTIEGTEI
MVRSKKSVDVDKNMIPTGNIVDREIATFNSTKPTVLGPKNPQFDCCFVVDENAKPSQINTL
NNELTLIVKAFHPDSNITLEVLSTEPTYQFYTGDFLSAGYEARQGFAIEPGRYIDAINQENWK
DCVTLKNGETYGSKIVYRFS
11111111110000000000000000000000000000000000000000000000000000000000000000000000000
00000000000000000000000000000000000000000000000000000000000000000000000000000000000
00000000000000000000000000000000000000000000000000000000000000000001111110000000000
00000000000000000000000000000000000000000000000000000000111111111000000000000000000
00000000000000000000000000000000000000000000000000000000000000000000000000000000000
00000000000000000000000000000000000000000000000000000000000000000000000000000000000
00000000000000000000000000000000000000000000000000000000000000000000000000000000000
00000000000000000000000000000000000000000000000000000000000000000000000000000000000
00000000000000000000000000000000000
>Q89YS0_4ak1_28_725
SDTETTDSTKFTIYYTGMTDIGPSMTGVISSPTYKGGTPYDFAITRITLDGEPFSDSIFAIDSETG
KITLNSTSNTPVGLYKLSVACYSNNNRYEYTDIVEINMMKPVPDGIKTDPEKLQVEYADIIDT
ESSNELPTSQIRTEGNHISISNYTIASAMWNGVAVESPEDYFAVSDKGEISIIKGNQNIQPGKYI
LSFKLTTAATGEDPEKGIFENALEINVTSRPLSLIYTPDEGKIEEEGERSPETTFQSNIPALKGSA
EGLVYSISSVSPNTDKITIDPTTGVLSVAAHHGFKDGEKYQISVKAINEFSPEGVVFENVFTLN
TVEFIEPIANFGYADVNDVQAVEIDINKNENFKGDEVKYEFVNLPTDLQGELALDLDGNIAI
KKGNKIPVGQYTVQVMATNTKGSETATFTLTITANPNYFTYFRYGNNLGLTPIENYADQFRI
EAGGKLNSVKPVPTATDAKDGLSSLKWEVELKHNPNNTKATINESTGQITITGLKQGQCG
MVMVTATAGEGKTAVSVKQPVFFHFSMISDSNVQLEYTPFVFQVNPARGGESIAPSLGAGID
KSTFRLDYRRDFFYYNIAGPDSHISGALAQKVDNFLSEMWNSYDATAGTSRKPMSYFENTT
NLSKALGYIDQTDFKVHINPNLWRNKDGYANGAMIGQITYDVTGKDPQAATSGARVSPIFI
WFDTKF
11111111110000000000000000000000000000000000000000000000000000000000000000000000000
00000000000000000000000000000001111111111111111111111111110000000000000000011111111
11111111111111111111000000000000000000000000000000000001100110000000000000000000000
00000011111111111111111000000000000000000000000000111000011111111100000000000000000
00000000000000000000000000000000000000000000000000000000000000000000000000000000000
00000000000000000000000000000000000000000000000000000000000000000000000000000000000
00000000000000000000000000000000000000000000000000000000000000000000000000000000000

00000000000000000000000000000000000000000000000000000000000000000000000000000000000
0000000000000000000000000000000000
>Q08282_2zwa_0_694
MKNLTTIKQTNKNVKQERRKKYADLAIQGTNNSSIASKRSVELLYLPKLSSANNFQMDKN
NKLLEYFKFFVPKKIKRSPCINRGYWLRLFAIRSRLNSIIEQTPQDKKIVVVNLGCGYDPLPFQ
LLDTNNIQSQQYHDRVSFIDIDYSDLLKIKIELIKTIPELSKIIGLSEDKDYVDDSNVDFLTTPK
YLARPCDLNDSKMFSTLLNECQLYDPNVVKVFVAEVSLAYMKPERSDSIIEATSKMENSHFII
LEQLIPKGPFEPFSKQMLAHFKRNDSPLQSVLKYNTIESQVQRFNKLGFAYVNVGDMFQLW
ESADEATKKELLKVEPFDELEEFHLFCHHYVLCHATNYKEFAFTQGFLFDRSISEINLTVDED
YQLLECECPINRKFGDVDVAGNDVFYMGGSNPYRVNEILQLSIHYDKIDMKNIEVSSSEVPV
ARMCHTFTTISRNNQLLLIGGRKAPHQGLSDNWIFDMKTREWSMIKSLSHTRFRHSACSLP
DGNVLILGGVTEGPAMLLYNVTEEIFKDVTPKDEFFQNSLVSAGLEFDPVSKQGIILGGGFM
DQTTVSDKAIIFKYDAENATEPITVIKKLQHPLFQRYGSQIKYITPRKLLIVGGTSPSGLFDRT
NSIISLDPLSETLTSIPISRRIWEDHSLMLAGFSLVSTSMGTIHIIGGGATCYGFGSVTNVGLKLI
AIAK
11100000000000000000000000000000000000000000000000000000000000000000000000000000000
00000000000000000000000000000000000000000000000000000000000000000000000000000000000
00000000000000000000000000000000000000000000000000000000000000000000000000000000000
00000000000000000000000000000000000000000000000000000000000000000000000000000000000
00000000000000000000000000000000111100000000000000000000000000000000000000000000000
00000000000000000000000000000000000000000000000000000000000000000000000000000000000
00000000000000000000000000000000000000000000000000000000000000000000000000000000000
00000000000000000000000000000000000000000000000000000000000000000000000000000000000
000000000000000000000000000001
>Q8NBP7_2p4e_0_691
MGTVSSRRSWWPLPLLLLLLLLLGPAGARAQEDEDGDYEELVLALRSEEDGLAEAPEHGTT
ATFHRCAKDPWRLPGTYVVVLKEETHLSQSERTARRLQAQAARRGYLTKILHVFHGLLPGF
LVKMSGDLLELALKLPHVDYIEEDSSVFAQSIPWNLERITPPRYRADEYQPPDGGSLVEVYLL
DTSIQSDHREIEGRVMVTDFENVPEEDGTRFHRQASKCDSHGTHLAGVVSGRDAGVAKGA
SMRSLRVLNCQGKGTVSGTLIGLEFIRKSQLVQPVGPLVVLLPLAGGYSRVLNAACQRLAR
AGVVLVTAAGNFRDDACLYSPASAPEVITVGATNAQDQPVTLGTLGTNFGRCVDLFAPGE
DIIGASSDCSTCFVSQSGTSQAAAHVAGIAAMMLSAEPELTLAELRQRLIHFSAKDVINEAW
FPEDQRVLTPNLVAALPPSTHGAGWQLFCRTVWSAHSGPTRMATAVARCAPDEELLSCSSF
SRSGKRRGERMEAQGGKLVCRAHNAFGGEGVYAIARCCLLPQANCSVHTAPPAEASMGT
RVHCHQQGHVLTGCSSHWEVEDLGTHKPPVLRPRGQPNQCVGHREASIHASCCHAPGLE
CKVKEHGIPAPQEQVTVACEEGWTLTGCSALPGTSHVLGAYAVDNTCVVRSRDVSTTGSTS
EGAVTAVAICCRSRHLAQASQELQ
11111111111111111111111111111111111111111111111111111111111100000000000000000000000
00000000000000000000000000000000000000000000000000000000000000000000000000000000000
00111111110000000000000000000000000000000000001111110000000000000000000000000000000
00000000000000000000000000000000000000000000000000000000000000000000000000000000000
00000000000000000000000000000000000000000000000000000000000000000000000000000000000
00000000000000000000000000000000001110000000000000000000000000000000000000000000000
00000000000000000000000000000000000000000000000000000000000000000000000000111111111
11100000000000000000000000000000000000000000000000000000000000000000000000000011111
1110000000000000001111111111
>O34208_3tu3_0_686
MHIQSLGATASSLNQEPVETPSQAAHKSASLRQEPSGQGLGVALKSTPGILSGKLPESVSDV
RFSSPQGQGESRTLTDSAGPRQITLRQFENGVTELQLSRPPLTSLVLSGGGAKGAAYPGAML
ALEEKGMLDGIRSMSGSSAGGITAALLASGMSPAAFKTLSDKMDLISLLDSSNKKLKLFQHI
SSEIGASLKKGLGNKIGGFSELLLNVLPRIDSRAEPLERLLRDETRKAVLGQIATHPEVARQPT

VAAIASRLQSGSGVTFGDLDRLSAYIPQIKTLNITGTAMFEGRPQLVVFNASHTPDLEVAQA
AHISGSFPGVFQKVSLSDQPYQAGVEWTEFQDGGVMINVPVPEMIDKNFDSGPLRRNDNLI
LEFEGEAGEVAPDRGTRGGALKGWVVGVPALQAREMLQLEGLEELREQTVVVPLKSERGD
FSGMLGGTLNFTMPDEIKAHLQERLQERVGEHLEKRLQASERHTFASLDEALLALDDSMLT
SVAQQNPEITDGAVAFRQKARDAFTELTVAIVSANGLAGRLKLDEAMRSALQRLDALADT
PERLAWLAAELNHADNVDHQQLLDAMRGQTVQSPVLAAALAEAQRRKVAVIAENIRKE
VIFPSLYRPGQPDSNVALLRRAEEQLRHATSPAEINQALNDIVDNYSARGFLRFGKPLSSTTV
EMAKAWRNKEFT
11111111111111111111111111111111111111111111111111100000000000000000000000000000
00000000000000000000000000000000000000000000000000000000000000000000000000000000
00000000000000000000001111111111111110000000000000000000000000000000000000000000
00000000000000000000000000000000000000000000000000000000000000000000111111111111
11111111111111110000000000000000000000000011111111111111111000000001111111111000
00000000000111111111111111111110000000000000000000000000000000000000000000000000
00000000000000000000000000000000000000000000000000000000000000000000000000000000
00000000000000000000000000000000000000000000000000000000000000000000000000000000
00000000000000000000000000000000000000000000000000000000000000000000000000011111
11111111000000000001111
>P52888_2o36_15_687
SPCSVVNDLRWDLSAQQIEERTRELIEQTKRVYDQVGTQEFEDVSYESTLKALADVEVTYTV
QRNILDFPQHVSPSKDIRTASTEADKKLSEFDVEMSMREDVYQRIVWLQEKVQKDSLRPEA
ARYLERLIKLGRRNGLHLPRETQENIKRIKKKLSLLCIDFNKNLNEDTTFLPFTLQELGGLPE
DFLNSLEKMEDGKLKVTLKYPHYFPLLKKCHVPETRRKVEEAFNCRCKEENCAILKELVTL
RAQKSRLLGFHTHADYVLEMNMAKTSQTVATFLDELAQKLKPLGEQERAVILELKRAECE
RRGLPFDGRIRAWDMRYYMNQVEETRYCVDQNLLKEYFPVQVVTHGLLGIYQELLGLAFH
HEEGASAWHEDVRLYTARDAASGEVVGKFYLDLYPREGKYGHAACFGLQPGCLRQDGSR
QIAIAAMVANFTKPTADAPSLLQHDEVETYFHEFGHVMHQLCSQAEFAMFSGTHVERDFV
EAPSQMLENWVWEQEPLLRMSRHYRTGSAVPRELLEKLIESRQANTGLFNLRQIVLAKVD
QALHTQTDADPAEEYARLCQEILGVPATPGTNMPATFGHLAGGYDAQYYGYLWSEVYSM
DMFHTRFKQEGVLNSKVGMDYRSCILRPGGSEDASAMLRRFLGRDPKQDAFLLSKGLQVG
GCEPEPQV
11111111000000000000000000000000000000000000000000000000000000000000000000000000
00000000000000000000000000000000000000000000000000000000000000000000000000000000
00000000000000000000000000000000000000000000000000000000000000000000000000000000
00000000000000000000000000000000000000000000000000000000000000000000000000000000
00000000000000000000000000000000000000000000000000000000000000000000000000000000
00000000000000000000000000000000000000000000000000000000000000000000000000000000
00000000000000000000000000000000000000000000000000000000000000000000000000000000
00000000000000000000000000000000000000000000000000000000000000000000000000000011
111111111
>Q2SJA2_4fus_32_703
SAEEYNERFMEMWNKIHDPANGYFSADGGPYHSVETLIVEAPDHGHESTSEAYSYFLLLEA
YYGKVTGDWSKLRNAWAKMEEHIIPTQEMQPTNNFYNPSKPASYAAEHAQPSGYPSQLEF
GVPVGEDPISAKLAQTYGSWDVYGMHWLLDMDNIYGYGNLGDGVSTPSYINTFQRGEQE
SVWETVTHPSWESFKWGGPNGFLPLFTKDNNYSRQWRYTNAPDADARAVQVMYWAYQ
WIKEQGKDPEQEVPGLMAKAAKMGDYLRLAMFDKYFKKMGTQDKNAQGGKGYESAHY
LMSWYYAWGGAADANAGWAFRIGSSHVHFGYQNPIAAMALSEFDPLKPRTPGATEDWAT
GLKRSMEFYTWLQSAEGGIAGGATNSWDGSYKPHPQDRADATFYGMVYDENPVYHDPGS
GTWFGWQAWSMQRVAEYYYLKGDAQAKQLMDKWAPWVLSNINWLEDGSFEIPATLEWT
GKPEKWDPANPKANTNLHVSVVDHGQDLGIAAGVAKALMFYAAAAEKYDTPQNEAKE
ASKKLLDAMWTHFKTPKGLAAPEKRGDYARFFDKVYVPGEFNGSMANGDAINSESTFLS
MRSFYLDDPMFKQVEDALNSGEDPVFTYHRFWAQTEAATAYANYAALFEGDNPCDEGCA

PTAQPLSVSTRVNKAVSITLKGTDSDG
00000000000000000000000000000000000000000000000000000000000000000000000000000000000
00000000000000000000000000000000000000000000000000000000000000000000000000000000000
00000000000000000000000000000000000000000000000000000000000000000000000000000000000
00000000000000000000000000000000000000000000000000000000000000000000000000000000000
00000000000000000000000000000000000000000000000000000000000000000000000000000000000
00000000000000000000000000000000000000000000000000000000000000000000000000000000000
00000000000000000000000000000000000000000000000000000000000000000000000000000000000
00000000000000000000000000000000000000000000000000000011111111111111111111111111111
11111111
>P12528_2xc1_1_666
TDITANVVVSNPRPIFTESRSFKAVANGKIYIGQIDTDPVNPANQIPVYIENEDGSHVQITQPL
IINAAGKIVYNGQLVKIVTVQGHSMAIYDANGSQVDYIANVLKYDPDQYSIEADKKFKYSV
KLSDYPTLQDAASAAVDGLLIDRDYNFYGGETVDFGGKVLTIECKAKFIGDGNLIFTKLGK
GSRIAGVFMESTTTPWVIKPWTDDNQWLTDAAAVVATLKQSKTDGYQPTVSDYVKFPGIET
LLPPNAKGQNITSTLEIRECIGVEVHRASGLMAGFLFRGCHFCKMVDANNPSGGKDGIITF
ENLSGDWGKGNYVIGGRTSYGSVSSAQFLRNNGGFERDGGVIGFTSYRAGESGVKTWQGT
VGSTTSRNYNLQFRDSVVIYPVWDGFDLGADTDMNPELDRPGDYPITQYPLHQLPLNHLI
DNLLVRGALGVGFGMDGKGMYVSNITVEDCAGSGAYLLTHESVFTNIAIIDTNTKDFQAN
QIYISGACRVNGLRLIGIRSTDGQGLTIDAPNSTVSGITGMVDPSRINVANLAEEGLGNIRAN
SFGYDSAAIKLRIHKLSKTLDSGALYSHINGGAGSGSAYTQLTAISGSTPDAVSLKVNHKDCR
GAEIPFVPDIASDDFIKDSSCFLPYWENNSTSLKALVKKPNGELVRLTLATL
11111000000000000000000000000000000000000000000000000000000000000000000000000000000
00000000000000000000000000000000000000000000000000000000000000000000000000000000000
00000000000000000000000000000000000000000000000000000000000000000000000000000000000
00000000000000000000000000000000000000000000000000000000000000000000000000000000000
00000000000000000000000000000000000000000000000000000000000000000000000000000000000
00000000000000000000000000000000000000000000000000000000000000000000000000000000000
00000000000000000000000000000000000000000000000000000000000000000000000000000000000
00000000000000000000000000000000000000000000000000000000000000000000000000000000000
00
>P15245_1pn0_0_664
MTKYSESYCDVLIVGAGPAGLMAARVLSEYVRQKPDLKVRIIDKRSTKVYNGQADGLQCR
TLESLKNLGLADKILSEANDMSTIALYNPDENGHIRRTDRIPDTLPGISRYHQVVLHQGRIER
HILDSIAEISDTRIKVERPLIPEKMEIDSSKAEDPEAYPVTMTLRYMSDHESTPLQFGHKTENS
LFHSNLQTQEEEDANYRLPEGKEAGEIETVHCKYVIGCDGGHSWVRRTLGFEMIGEQTDYI
WGVLDAVPASNFPDIRSPCAIHSAESGSIMIIPRENNLVRFYVQLQARAEKGGRVDRTKFTP
EVVIANAKKIFHPYTFDVQQLDWFTAYHIGQRVTEKFSKDERVFIAGDACHTHSPKAGQG
MNTSMMDTYNLGWKLGLVLTGRAKRDILKTYEEERHAFAQALIDFDHQFSRLFSGRPAKD
VADEMGVSMDVFKEAFVKGNEFASGTAINYDENLVTDKKSSKQELAKNCVVGTRFKSQPV
VRHSEGLWMHFGDRLVTDGRFRIIVFAGKATDATQMSRIKKFSAYLDSENSVISLYTPKVSDR
NSRIDVITIHSCHRDDIEMHDFPAPALHPKWQYDFIYADCDSWHHPHPKSYQAWGVDETK
GAVVVVRPDGYTSLVTDLEGTAEIDRYFSGILVEPKEKSGAQTEADWTKSTA
10000000000000000000000000000000000000000000000000000000000000000000000000000000000
00000000000000000000000000000000000000000000000000000000000000000000000000000000000
00000000000000000000000000000000000000000000000000000000000000000000000000000000000
00000000000000000000000000000000000000000000001111110000000000000000000000000000000
00000000000000000000000000000000000000000000000000000000000000000000000000000000000
00000000000000000000000000000000000000000000000000000000000000000000000000000000000
00000000000000000000000000000000000000000000000000000000000000000000000000000000000
00000000000000000000000000000000000000000000000000000000000000000000000000000000001

1
>Q56243_1d2m_0_664
MTFRYRGPSPKGDQPKAIAGLVEALRDGERFVTLLGATGTGKTVTMAKVIEALGRPALVLA
PNKILAAQLAAEFRELFPENAVEYFISYYDYYQPEAYVPGKDLYIEKDASINPEIERLRHSTTR
SLLTRRDVIVVASVSAIYGLGDPREYRARNLVVERGKPYPREVLLERLLELGYQRNDIDLSPG
RFRAKGEVLEIFPAYETEPIRVELFGDEVERISQVHPVTGERLRELPGFVLFPATHYLSPEGLEE
ILKEIEKELWERVRYFEERGEVLYAQRLKERTLYDLEMLRVMGTCPGVENYARYFTGKAPGE
PPYTLLDYFPEDFLVFLDESHVTVPQLQGMYRGDYARKKTLVDYGFRLPSALDNRPLRFEEF
LERVSQVVFVSATPGPFELAHSGRVVEQIIRPTGLLDPLVRVKPTENQILDLMEGIRERAARG
ERTLVTVLTVRMAEELTSFLVEHGIRARYLHHELDAFERQALIRDLRLGHYDCLVGINLLRE
GLDIPEVSLVAILDADKEGFLRSERSLIQTIGRAARNARGEVWLYADRVSEAMQRAIEETNR
RRALQEAYNLEHGITPETVRKEVRAVIRPEGYEEAPLEADLSGEDLRERIAELELAMWQAAE
ALDFERAARLRDEIRALEARLQGVRAPEPVPGGRKRKRR
10000000000000000000000000000000000000000000000000000000000000000000000000000000000
00000000000000000000000000000000000000000000000000000000000000000000000000011111110
00000000000000000000000000000000000000000000001111111111111111111111100000000000000
00000000000000000000000000000000000000000000000000000000000000000000000000000000000
00000000000000000000000000000000000000000000000000000000000000000000000000000000000
00000000000000000000000000000000000000000000000000000000000000000000000000000000000
00000000000000000000000000000000000000000000000000000000000000000000000000000000000
00111111111111111111111111111111111111111111111111111111111111111111111111111111111
1
>P97275_4bby_0_657
MAEAAAAAAAAAAAGETSASSGSAAERDPDQDRAGRRLRVLSGHLLGRPQEALSTNECK
ARRAASAATAAPTATPAAPESGIIPKKRQELMKWNGWGYNDSKFFLNKKGQLELTGKRYP
LSGVALPTFKDWIQNTFGINLDHKTTSKASLNPSDTPPSIVNEDFLHELKKTNISYSQEADDR
VFRAHGHCLHEIFLLREGMFERIPDIVLWPTCHDDVVKIVNLACKYNLCIIPIGGGTSVSYGL
MCPADETRTIISLDTSQMNRILWVDENNLTAHVEAGITGQELERQLKESGYCTGHEPDSLEF
STVGGWISTRASGMKKNIYGNIEDLVVHMKVVTPRGVIEKSCQGPRMSTGPDIHHFIMGSE
GTLGVITEATIKIRPTPEYQKYGSVAFPNFEQGVACLREIAKQRCAPASIRLMDNQQFQFGH
ALKPQVSSIFTSFLDGLKKFYITKFKGFDPNQLSVATLLFEGDREKVLQHEKQVYDIAAKFGG
LAAGEDNGQRGYLLTYVIAYMRDLGLEYYIIGESFETSAPWDRVVDLCRNVKERIRRECKEK
GVQFPPLSTCRVTQTYDAGACIYFYFAFNYRGISDPLAVFEQTEAAAREEILANGGSLSHHH
GVGKLRKQWLKESISDVGFGMLKSVKDYVDPTNIFGNRNLL
11111111111111111111111111111111111111111111111111111111111111111111111111111111000
00000000000000000000000000000000000000000000000000000000000000000000000000000000000
00000000000000000000000000000000000000000000000000000000000000000000000000000000000
00000000000000000000000000000000000000000000000000000000000000000000000000000000000
00000000000000000000000000000000000000000000000000000000000000000000000000000000000
00000000000000000000111111111111111111110000000000000000000000000000000000000000000
00000000000000000000000000000000000000000000000000000000000000000000000000000000000
0000000000000000000000000000000000000000000000000000000000000000000000000000
>Q9I5I9_2cfu_0_657
MSRLLALLALAPLLAGAAETTAPKPPSAFTVEAQRRVEAELPFADRADFERADRGLIRRPER
LLIRNPDGSVAWQLGGYDFLLDGKPRDSINPSLQRQALLNLKYGLFEVAEGIYQVRGFDLA
NITFIRGDSGWIVVDTLTTPATARAAYELVSRELGERPIRTVIYSHAHADHFGGVRGLVEPQQ
VASGAVQIIAPAGFMEAAIKENVLAGNAMMRRATYQYGTQLPKGPQGQVDMAIGKGLAR
GPLSLLAPTRLIEGEGEDLVLDGVPFTFQNTPGTESPAEMNIWLPRQKALLMAENVVGTLH
NLYTLRGAEVRDALGWSKYINQALHRFGRQAEVMFAVHNWPRWGNAEIVEVLEKQRDLY
GYLHDQTLHLANQGVTIGQVHNRLRLPPSLDQEWYDRGYHGSVSHNARAVLNRYLGYY
DGNPATLDPLSPEDSAGRYVEYMGGAERLLEQARASYARGEYRWVVEVVNRLVFAEPDNR

AARELQADALEQLGYQAENAGWRNSYLSAAYELRHGVPRDQPTMKAGSADALAAMDT
GLLFDYLGVRLDAGAAEGKALSINLRLPDIGENYLLELKNSHLNNLRGVQSEDAGQTVSID
RADLNRLLLKEVSAVRLVFEGKLKSSGNPLLLGQLFGMLGDFDFWFDIVTPAAKSEG
11111111111111111110000000000000000000000000000000000000000000000000000000000000000
00000000000000000000000000000000000000000000000000000000000000000000000000000000000
00000000000000000000000000000000000000011100000000000000000000000000000000000000000
00000000000000000000000000000000000000000000000000000000000000000000000000000000000
00000000000000000000000000000000000000000000000000000000000000000000000000000000000
00000000000000000000000000000000000000000000000000000000000000000000000000000000000
00000000000000000000000000111110000000000000000000000000000000000000000000000000000
00000000000000000000000000000000000000000000000000000000000000000000000000111
>P0CS93_1k3i_24_679
AVGTGIPEGSLQFLSLRASAPIGSAISRNNWAVTCDSAQSGNECNKAIDGNKDTFWHTFYG
ANGDPKPPHTYTIDMKTTQNVNGLSMLPRQDGNQNGWIGRHEVYLSSDGTNWGSPVAS
GSWFADSTTKYSNFETRPARYVRLVAITEANGQPWTSIAEINVFQASSYTAPQPGLGRWGPT
IDLPIVPAAAAIEPTSGRVLMWSSYRNDAFGGSPGGITLTSSWDPSTGIVSDRTVTVTKHDMF
CPGISMDGNGQIVVTGGNDAKKTSLYDSSSDSWIPGPDMQVARGYQSSATMSDGRVFTIGG
SWSGGVFEKNGEVYSPSSKTWTSLPNAKVNPMLTADKQGLYRSDNHAWLFGWKKGSVFQ
AGPSTAMNWYYTSGSGDVKSAGKRQSNRGVAPDAMCGNAVMYDAVKGKILTFGGSPDY
QDSDATTNAHIITLGEPGTSPNTVFASNGLYFARTFHTSVVLPDGSTFITGGQRRGIPFEDSTP
VFTPEIYVPEQDTFYKQNPNSIVRVYHSISLLLPDGRVFNGGGGLCGDCTTNHFDAQIFTPN
YLYNSNGNLATRPKITRTSTQSVKVGGRITISTDSSISKASLIRYGTATHTVNTDQRRIPLTLTN
NGGNSYSFQVPSDSGVALPGYWMLFVMNSAGVPSVASTIRVTQ
11111000000000000000000000000000000000000000000000000000000000000000000000000000000
00000000000000000000000000000000000000000000000000000000000000000000000000000000000
00000000000000000000000000000000000000000000000000000000000000000000000000000000000
00000000000000000000000000000000000000000000000000000000000000000000000000000000000
00000000000000000000000000000000000000000000000000000000000000000000000000000000000
00000000000000000000000000000000000000000000000000000000000000000000000000000000000
00000000000000000000000000000000000000000000000000000000000000000000000000000000000
000000000000000000000000000000000000000000000000000000000000000000000000000
>O33839_3o0n_0_643
MKLSDLISRWIDVEPSKNAQIILRDRYFMKDLDGNYLETKWEDVARRVARVVATAELLNPS
YKKNEKLDRIKEWEDIFFRVLKARLFIPNSPTLFNAGLGVKHDLLWKPIDQMTLEDYEEIYR
SRNHLHMLSACFVVPVGDSIEEIFEAVKEYALITKVGGGVGSNFSELRPKGSFVAGTHGKAS
GPVSFMHVFNSAISVVKQGYRRRGALMGILNINHPDIEEFIDAKKENTGEAVLNFFNLSVGF
PMDKKEILKLYEEDGELELSHPRSTIRKKVKIRELFRKIATNAWKSGDPGLAFLGEMNKYYP
LYPHRKINSTNPCGEIGLSDYEACNLGSIDVAKFYNNGFVDLEALQELVQIAVRFLDNVIDV
NVFPIDKITKAVKESRRLGLGIMGFADLLYKLEIPYNSQEARDFAANLMAFIALHAHRTSYE
LGKEKGNFPLLEISRYRTEDNFVPFAMGMSNYDDEIREVMKMTKEFRRNVALLTIAPTGSIS
NIADTSSGLEPNFLLAYTRFVTKEDGTKEPLLYVNQVLREKLNPEILKRIEKELIEKGSLKDIP
DVPEKIKKVFVVALDIDPMDHLLMQDAFQRYVDNNISKTINMPQSATVDDVLNVYLEALR
TNVRGITVYRDGSLQTQVLTKALKT
00000000000000000000000000000000000000000000000000000000000000000000000000000000000
00000000000000000000000000000000000000000000000000000000000000000000000000000000000
00000000000000000000000000000000000011111110000000000000000000001111110000000000000
00000000000000000000000000000000000000000000000000000000000000000000000000000000000
00000000000000000000000000000000000000000000000000000000000000000000000000000000000
00000000000000000000000000000000000000000000000000000000000000000000000000000000000
00000000000000000111111111000000000000000000000000000000000000000000000000000000000
000000000000000000000000000000000000000000000000000011111111111

>P40406_3nvd_0_641
MRPVFPLILSAVLFLSCFFGARQTEASASKRAIDANQIVNRMSLDEKLGQMLMPDFRNWQ
KEGESSPQALTKMNDEVASLVKKYQFGGIILFAENVKTTKQTVQLTDDYQKASPKIPLMLSI
DQEGGIVTRLGEGTNFPGNMALGAARSRINAYQTGSIIGKELSALGINTDFSPVVDINNNPD
NPVIGVRSFSSNRELTSRLGLYTMKGLQRQDIASALKHFPGHGDTDVDSHYGLPLVSHGQE
RLREVELYPFQKAIDAGADMVMTAHVQFPAFDDTTYKSKLDGSDILVPATLSKKVMTGLLR
QEMGFNGVIVTDALNMKAIADHFGQEEAVVMAVKAGVDIALMPASVTSLKEEQKFARVI
QALKEAVKNGDIPEQQINNSVERIISLKIKRGMYPARNSDSTKEKIAKAKKIVGSKQHLKAE
KKLAEKAVTVLKNEQHTLPFKPKKGSRILIVAPYEEQTASIEQTIHDLIKRKKIKPVSLSKMNF
ASQVFKTEHEKQVKEADYIITGSYVVKNDPVVNDGVIDDTISDSSKWATVFPRAVMKAALQ
HNKPFVLMSLRNPYDAANFEEAKALIAVYGFKGYANGRYLQPNIPAGVMAIFGQAKPKGT
LPVDIPSVTKPGNTLYPLGYGLNIKTGRPL
11111111111111111111111110000000000000000000000000000000000000000000000000000000000
00000000000000000000000000000000000000000000000000000000000000000000000000000000000
00000000000000000000000000000000000000000000000000000000000000000000000000000000000
00000000000000000000000000000000000000000000000000000000000000000000000000000000000
00000000000000000000000000000000000000000000000000000000000000000000000000000000000
00000000000000000000000000000000000000000000000000000000000000000000000000000000000
00000000000000000000000000000000000000000000000000000000000000000000000000000000000
0000000000000000000000000000000000000000000000000000000000000
>Q05KQ5_2dkh_0_638
MQFHLNGFRPGNPLIAPASPLAPAHTEAVPSQVDVLIVGCGPAGLTLAAQLAAFPDIRTCIV
EQKEGPMELGQADGIACRTMEMFEAFEFADSILKEACWINDVTFWKPDPGQPGRIARHGR
VQDTEDGLSEFPHVILNQARVHDHYLERMRNSPSRLEPHYARRVLDVKVDHGAADYPVT
VTLERCDAAHAGQIETVQARYVVGCDGARSNVRRAIGRQLVGDSANQAWGVMDVLAVT
DFPDVRYKVAIQSEQGNVLIIPREGGHLVRFYVEMDKLDADERVASRNITVEQLIATAQRVL
HPYKLEVKNVPWWSVYEIGQRICAKYDDVVDAVATPDSPLPRVFIAGDACHTHSPKAGQG
MNFSMQDSFNLGWKLAAVLRKQCAPELLHTYSSERQVVAQQLIDFDREWAKMFSDPAKE
GGQGGVDPKEFQKYFEQHGRFTAGVGTHYAPSLLTGQAKHQALASGFTVGMRFHSAPVV
RVCDAKPVQLGHCGKADGRWRLYAFAAQNDLAQPESGLLALCRFLEGDAASPLRRFTPA
GQDIDSIFDLRAVFPQAYTEVALETLPALLLPPKGQLGMIDYEKVFSPDLKNAGQDIFELRGI
DRQQGALVVVRPDQYVAQVLPLGDHAALSAYFESFMRA
00000000000000000000000000000000000000000000000000000000000000000000000000000000000
00000000000000000000000000000000000000000000000000000000000000000000000000000000000
00000000000000000000000000000000000000000000000000000000000000000000000000000000000
00000000000000000000000000111111111110000000000000000000000000000000000000000000000
00000000000000000000000000000000000000000000000000000000000000000000000000000000111
11111111111000000000000000000000000000000000000000000000000000000000000000000000000
00000000000000000000000000000000000000000000000000000000000000000000000000000000000
0000000000000000000000000000000000000000000000000000000000
>Q8P5I2_2wpg_0_636
MIASSPIDAAALRASVAAALDPSTAVATLARFDTHAPRLLDALSTLYGDHADYVTWLPQWL
SALGVVAQARPAALRHLDDSRAPGWFGQQDMLGYSAYVDRFAGTLRGVAERVPYLQELG
VRYLHLLPFLRARAGDNDGGFAVSDYGQVEPALGSNDDLVALTARLRAANISLCADFVLN
HTADDHAWAQAARAGDTRYLDYYHHFADRNAPDQYDTTLVQVFPQTAPGNFTWVDETR
QWMWTTFYPYQWDLNWSNPAVFGEMALAMLELANLGVEAFRLDSTAYLWKRPGTNCM
NQPEAHTILVALRAVADIVAPSVVMKAEAIVPMAELPPYFGSGVQRGHECHLAYHSTLMA
AGWSALALQRGDILQDVIAHSPPLPPNCAWLSYVRCHDDIGWNVLQHEAAGTAAQPPFS
LREVAQFYANAVPGSYARGESFQSSGDGVHGTNGMSAALVGVQAAHEHADAAAAARAV
DRLVLLYAVSLAMPGVPLIYMGDELALPNDTAYLDDAQRRHEGRWLHRPAMAWELAAQR
HDASTLAGTVYTRLRALIRLRAGLPALAATQSLGSVALGDARLFALTRGDSFLAVHNFSDVP

LPVDLTQTGHALWAVLDTDGTGDAPEPHTELLLPAYGVRWLQRR
11110000000000000000000000000000000000000000000000000000000000000000000000000000000
00000000000000000000000000000000000000000000000000000000000000000000000000000000000
00000000000000000000000000000000000000000000000000000000001110000000000000000000000
00000000000000000000000000000000000000000000000000000000000000000000000000000000000
00000000000000000000000000000000000000000000000000000000000000000000000000000000000
00000000000000000000001111100000000000000000000000000000000000000000000000000000000
00000000000000000000000000000000000000000000000000000000000000000000000000000000000
00000000000000000000000000000000000000000000000000000000
>P43405_4fl3_0_634
MASSGMADSANHLPFFFGNITREEAEDYLVQGGMSDGLYLLRQSRNYLGGFALSVAHGRK
AHHYTIERELNGTYAIAGGRTHASPADLCHYHSQESDGLVCLLKKPFNRPQGVQPKTGPFE
DLKENLIREYVKQTWNLQGQALEQAIISQKPQLEKLIATTAHEKMPWFHGKISREESEQIVL
IGSKTNGKFLIRARDNNGSYALCLLHEGKVLHYRIDKDKTGKLSIPEGKKFDTLWQLVEHYS
YKADGLLRVLTVPCQKIGTQGNVNFGGRPQLPGSHPATWSAGGIISRIKSYSFPKPGHRKSS
PAQGNRQESTVSFNPYEPELAPWAADKGPQREALPMDTEVYESPYADPEEIRPKEVYLDRK
LLTLEDKELGSGNFGTVKKGYYQMKKVVKTVAVKILKNEANDPALKDELLAEANVMQQL
DNPYIVRMIGICEAESWMLVMEMAELGPLNKYLQQNRHVKDKNIIELVHQVSMGMKYLE
ESNFVHRDLAARNVLLVTQHYAKISDFGLSKALRADENYYKAQTHGKWPVKWYAPECIN
YYKFSSKSDVWSFGVLMWEAFSYGQKPYRGMKGSEVTAMLEKGERMGCPAGCPREMYDL
MNLCWTYDVENRPGFAAVELRLRNYYYDVVN
11111110000000000000000000000000000000000000000000000000000000000000000000000000000
00000000000000000000000000000000000000000000000000000000000000000000000000000000000
00000000000000000000000000000000000000000000000000000000000000000000000000000000000
00000000000011111111111111111111111111111111111111111111111111111111111111111111111
11111000000000000000000000000000000000000000000000000000000000000000000000000000000
00000000000000000000000000000000000000000000000000000000000000000000000000000000000
00000000000000000000000111111111111100000000000000000000000000000000000000000000000
000000000000000000000000000000000000000000000000000000
>Q54443_3vmp_99_731
QKNGNMINLTTDKAVYQAGEAVHLNLTLNNTTSLAQNITATAEVYSLENKLKTLQYTKYL
LPNESYTTQKGEFVIPANSLANNRGYLLKVNISDSQNNILEQGNRAIAVEDDWRTFPRYAAI
GGSQKDNNSVLTKNLPDYYRELEQMKNMNINSYFFYDVYKSATNPFPNVPKFDQSWNW
WSHSQVETDAVKALVNRVHQTGAVAMLYNMILAQNANETAVLPDTEYIYNYETGGYGQN
GQVMTYSIDDKPLQYYYNPLSKSWQNYISNAMAQAMKNGGFDGWQGDTIGDNRVLSHN
QKDSRDIAHSFMLSDVYAEFLNKMKEKLPQYYLTLNDVNGENISKLANSKQDVIYNELWPF
GTSALGNRPQESYGDLKARVDQVRQATGKSLIVGAYMEEPKFDDNRVPLNGAARDVLAS
ATYQTDAVLLTTAAIAAAGGYHMSLAALANPNDGGGVGVLETAYYPTQSLKVSKELNRK
NYHYQQFITAYENLLRDKVENDSAEPQTFTANGRQLSQDALGINGDQVWTYAKKGNDFR
TIQLLNLMGITSDWKNEDGYENNKTPDEQTNLLVTYPLTGVSMAEADRIAKQVYLTSPDD
WLQSSMISLATQIKTNENGDPVLYIQVPRLTLWDMIYI
11100000000000000000000000000000000000000000000000000000000000000000000000000000000
00000000000000000000000000000000000000000000000000000000000000000000000000000000000
00000000000000000000000000000000000000000000000000000000000000000000000000000000000
00000000000000000000000000000000000000000000000000000000000000000000000000000000000
00000000000000000000000000000000000000000000000000000000000000000000000000000000000
00000000000000000000000000000000000000000000000000000000000000000000000000000000000
00000000000000000000000000000000000000000000000000000000000000000000000000000000000
0000000000000000000000000000000000000000000000000000
>B5QNF0_4iuw_0_631
MTLPRIQDDLYLAVNGEWQAKTPIPPDKSVVSADSNLTDDIRQKLVADLSTMTKTAKTLPL

QYAARLFAKANDQTRRQQLGIEPVRDRISFLMALTTLDQFRSAMPKLVADQYVLPISPYVD
ADMHDAEHNILNLGGPDTILPDAAMYQHEDAENAADLAAWSQMAAAMLAAVGFSQT
DQTAYVEAAKRFDRRLADYVPANVDLAVDSTYDNPLSWQAFEDAAGYLGIPQAFATYMP
QTPAKVNAVVPAYLPHLSKLLTPDNYSEWHAWMVINELLTCATYLSDDLRQLAGQYDRFL
AGQPEASSWTKHAFGIANEYFDDVIGQYYGQTYFGADAKADVTAMVKQILAQYRVQLEN
NTWLSPATKQKAMRKLATMQVKMGYPERLFSLYDHLSVDVDDDLLTAILKLSAQTQAFWF
KQLGQTVDRNQWNMPGHLVNASYDPLKNDITFPAGILQPPYYSLKWTRAENLGGTGATI
GHEISHSFDNNGALYDEYGNLHNWWTPADKQAFDQLVKAMAAQFDGRDYEGVKVNGT
LTVSENMADNAGMDVALALLGDQPDVKDLQAFFITYARSWATKMRPERAKTVLRQDVH
APATLRVNVPVQNFPAWYQAFNVQPQDGMYRQPQKRLTIWHQ
11000000000000000000000000000000000000000000000000000000000000000000000000000000000
00000000000000000000000000000000000000000000000000000000000000000000000000000000000
00000000000000000000000000000000000000000000000000000000000000000000000000000000000
00000000000000000000000000000000000000000000000000000000000000000000000000000000000
00000000000000000000000000000000000000000000000000000000000000000000000000000000000
00000000000000000000000000000000000000000000000000000000000000000000000000000000000
00000000000000000000000000000000000000000000000000000000000000000000000000000000000
000000000000000000000000000000000000000000000000000
>P46799_2gaj_2_632
KKVKKYIVVESPAKAKTIKSILGNEYEVFASMGHIIDLPKSKFGVDLEKDFEPEFAVIKGKEKV
VEKLKDLAKKGELLIASDMDREGEAIAWHIARVTNTLGRKNRIVFSEITPRVIREAVKNPREI
DMKKVRAQLARRILDRIVGYSLSPVLWRNFKSNLSAGRVQSATLKLVCDREREILRFVPKKY
HRITVNFDGLTAEIDVKEKKFFDAETLKEIQSIDELVVEEKKVSVKKFAPPEPFKTSTLQQEAY
SKLGFSVSKTMMIAQQLYEGVETKDGHIAFITYMRTDSTRVSDYAKEEARNLITEVFGEEYV
GSKRERRKSNAKIQDAHEAIRPTNVFMTPEEAGKYLNSDQKKLYELIWKRFLASQMKPSQY
EETRFVLRTKDGKYRFKGTVLKKIFDGYEKVWKTERNTGEFPFEEGESVKPVVVKIEEQETK
PKPRYTEGSLVKEMERLGIGRPSTYASTIKLLLNRGYIKKIRGYLYPTIVGSVVMDYLEKKYSD
VVSVSFTAEMEKDLDEVEQGKKTDKIVLREFYESFSSVFDRNDRIVVDFPTNQKCSCGKEMR
LSFGKYGFYLKCECGKTRSVKNDEIAVIDDGKIFLGRKDSESGSPDGRSVEGKGNLSEKRRK
GKKGS
11110000000000000000000000000000000000000000000000000000000000000000000000000000000
00000000000000000000000000000000000000000000000000000000000000000000000000000000000
00000000000000000000000000000000000000000000000000000000000000000000000000000000000
00000000000000000000000000000000000000000000000000000000000000000001111111111111100
00000000000000000000000000000000000000000000000000000000000000000000000000000000000
00000000000000000000000000000000000000000000000000000000000000000000000000000000000
00000000000000000000000000000000000000000000000000000000000000000000000000000000000
00000000000000000011111111111111111111111111111111
>Q6DLV0_4hhj_2761_3389
NMDVIGERIKRIKEEHNSTWHYDDENPYKTWAYHGSYEVKATGSASSMINGVVKLLTKPW
DVVPMVTQMAMTDTTPFGQQRVFKEKVDTRTPRPLPGTRKVMGITAEWLWRTLGRNKRP
RLCTREEFTKKVRTNAAMGAVFTEENQWDSAKAAVEDEEFWKLVDRERELHKLGKCGSC
VYNMMGKREKKLGEFGKAKGSRAIWYMWLGVRYLEFEALGFLNEDHWFSRENSYSGVEG
EGLHKLGYILRDISKIPGGAMYADDTAGWDTRITEDDLHNEEKIIQQMDPEHRQLANAIFK
LTYQNKVVKVQRPTPTGTVMDIISRKDQRGSGQVGTYGLNTFTNMEAQLVRQMEGEGVLT
KADLENPHLLEKKITQWLETKGVERLKRMAISGDDCVVKPIDDRFANALLALNDMGKVR
KDIPQWQPSKGWHDWQQVPFCSHHFHELIMKDGRKLVVPCRPQDELIGRARISQGAGWS
LRETACLGKAYAQMWSLMYFHRRDLRLASNAICSAVPVHWVPTSRTTWSIHAHHQWMTT
EDMLTVWNRVWIEENPWMEDKTPVTTWENVPYLGKREDQWCGSLIGLTSRATWAQNIPT
AIQQVRSLIGNEEFLDYMPSMKRFRKEEESEGAIW
00000000000000000000000000000000000000000000000000000000000000000000000000000000000

00000000000000000000000000000000000000000000000000001111111111111000000000000000000
00000000000000000000111111111110000000000000000000000000000000000000000000000000000
00000000000000000000000000000000000000000000000000000000000000000000000000000000000
00000000000000000000000000000000000000000000000000000000000000000000000000000000000
00000000000000000000000000000000000000000000000000000000000000000000000000000000000
00000000000000000000000000000000000000000000000000000000000000000000000000000000000
000000000000000000000000000000011111111111111111
>P12821_3nxq_29_656
LDPGLQPGNFSADEAGAQLFAQSYNSSAEQVLFQSVAASWAHDTNITAENARRQEEAALL
SQEFAEAWGQKAKELYEPIWQNFTDPQLRRIIGAVRTLGSANLPLAKRQQYNALLSNMSRI
YSTAKVCLPNKTATCWSLDPDLTNILASSRSYAMLLFAWEGWHNAAGIPLKPLYEDFTALS
NEAYKQDGFTDTGAYWRSWYNSPTFEDDLEHLYQQLEPLYLNLHAFVRRALHRRYGDRYI
NLRGPIPAHLLGDMWAQSWENIYDMVVPFPDKPNLDVTSTMLQQGWNATHMFRVAEEF
FTSLELSPMPPEFWEGSMLEKPADGREVVCHASAWDFYNRKDFRIKQCTRVTMDQLSTVH
HEMGHIQYYLQYKDLPVSLRRGANPGFHEAIGDVLALSVSTPEHLHKIGLLDRVTNDTESD
INYLLKMALEKIAFLPFGYLVDQWRWGVFSGRTPPSRYNFDWWYLRTKYQGICPPVTRNET
HFDAGAKFHVPNVTPYIRYFVSFVLQFQFHEALCKEAGYEGPLHQCDIYRSTKAGAKLRKV
LQAGSSRPWQEVLKDMVGLDALDAQPLLKYFQPVTQWLQEQNQQNGEVLGWPEYQWH
PPLPDNYPEGIDLVTDEAEASKFVEEYD
00000000000000000000000000000000000000000000000000000000000000000000000000000000000
00000000000000000000000000000000000000000000000000000000000000000000000000000000000
00000000000000000000000000000000000000000000000000000000000000000000000000000000000
00000000000000000000000000000000000000000000000000000000000000000000000000000000000
00000000000000000000000000000000000000000000000000000000000000000000000000000000000
00000000000000000000000000000000000000000000000000000000000000000000000000000000000
00000000000000000000000000000000000000000000000000000000000000000000000000000000000
00000000000000000000000000000111111111111111111
>Q6ST21_2epk_0_626
MATFLGLSSKQEKALVRLDKYLNLGEIAVSLVTDSATSIKVEGRQGYYQVSYKQPHQLYRAL
ALLSAALRSGQDEVQIEEEAAYEDLAYMADCSRNAVLNLSSAKKMIEVLALMGYSTFELYM
EDTYEIENQPYFGYFRGRYTVAELQEIEDYAADFDMSFVPCIQTLAHLSAFVKWGIKEVQEL
RDVEDILLIGEEKVYDLIEGMFQTMAHLHTRKINIGMDEAHLVGLGRYLIKHGFQNRSLLM
CQHLERVLDIADKYGFNCQMWSDMFFKLMSADGQYDRDVEIPEETRVYLDRLKERVTLVY
WDYYQDSEEKYNRNFQNHHKISQDIAFAGGAWKWIGFTPHNHFSRLVAIEANKACRKNQ
VKEVIVTGWGDNGGETSQFSVLPALQIWAELAYRNDLKKVSEHFLVSTGLDFDDFMKIDLA
NLLPDLPDNLSGINPNRYVLYQDVLCPLLEQHIRPEKDKQHFASSAQQLGEISKRAGEYAYI
FETQAQLNALLALKISITSGIQKAYRNGDKEHLSALAEKDFPQLYQMVEDFSDQFSRQWQQ
ENKIFGLDTIDIRFGGLLKRIKRAQERLEQFISGQIDCVEELEQEILPFNDFYKDQGLTATTAN
QWHLIATASTIYTT
10000000000000000000000000000000000000000000000000000000000000000000000000000000000
00000000000000000000000000000000000000000000000000000000000000000000000000000000000
00000000000000000000000000000000000000000000000000000000000000000000000000000000000
00000000000000000000000000111111111110000000000000000000000000000000000000000000000
00000000000000000000000000000000000000000000000000000000000000000000000000000000000
00000000000000000000000000000000000000000000000000000000000000000000000000000000000
00000000000000000000000000000000000000000000000000000000000000000000000000000000000
0000000000000000000000000000000000000000000001
>P70208_3ig3_1246_1871
TQDADRTLKRLQLQMDNLESRVALECKEAFAELQTDINELTNHMDGVQIPFLDYRTYAVR
VLFPGIEAHPVLKELDTPPNVEKALRLFGQLLHSRAFLLTFIHTLEAQSSFSMRDRGTVASLT
MVALQSRLDYATGLLKQLLADLIEKNLESKNHPKLLLRRTESVAEKMLTNWFTFLLHKFLK

ECAGEPLFLLYCAIKQQMEKGPIDAITGEARYSLSEDKLIRQQIDYKTLTLHCVCPESEGSAQ
VPVKVLNCDSITQAKDKLLDTVYKGIPYSQRPKAEDMDLEWRQGRMARIILQDEDITTKIE
CDWKRVNSLAHYQVTDGSLVALVPKQVSAYNMANSFTFTRSLSRYESLLRAASSPDSLRSRA
PMLTPDQEAGTKLWHLVRNHDHTDHREGDRGSKMVSEIYLTRLLATKGTLQKFVDDLFET
VFSTAHRGSALPLAIKYMFDFLDEQADQRQISDPDVRHTWKSNCLPLRFWVNVIKNPQFVF
DIHKNSITDACLSVVAQTFMDSCSTSEHRLGKDSPSNKLLYAKDIPNYKSWVERYYRDIAKM
ASISDQDMDAYLVEQSRLHANDFNVLSALSELYFYVTKYRQEILTSLDRDASCRKHKLRQKL
EQIITLVSSSS
00000000000000000000000000000000000000000111000000000000000000000000000000000000
00000000000000000000000000000000000000000000000000000000000000000000000000000000
00000000000000000000000000000000000000000000000000000000000000000000000000110000
00000000000000000000000000000000000000000000000000000000000000000000000000000000
01111111111111111111111111111111111111111111000000000001111111111111000000000000
00000000000000000000000000000000000000000000000000000000000000000000000000000000
00000000000000000000000000000000000000000000000000000000000000000000000000000000
0000000000000000000000000000000000000000001
>P0C2S4_1clc_0_624
SLTGVFPSGLIETKVSAAKITENYQFDSRIRLNSIGFIPNHSKKATIAANCSTFYVVKEDGTIVY
TGTATSMFDNDTKETVYIADFSSVNEEGTYYLAVPGVGKSVNFKIAMNVYEDAFKTAMLG
MYLLRCGTSVSATYNGIHYSHGPCHTNDAYLDYINGQHTKKDSTKGWHDAGDYNKYVV
NAGITVGSMFLAWEHFKDQLEPVALEIPEKNNSIPDFLDELKYEIDWILTMQYPDGSGRVA
HKVSTRNFGGFIMPENEHDERFFVPWSSAATADFVAMTAMAARIFRPYDPQYAEKCINAA
KVSYEFLKNNPANVFANQSGFSTGEYATVSDADDRLWAAAEMWETLGDEEYLRDFENRA
AQFSKKIEADFDWDNVANLGMFTYLLSERPGKNPALVQSIKDSLLSTADSIVRTSQNHGYG
RTLGTTYYWGCNGTVVRQTMILQVANKISPNNDYVNAALDAISHVFGRNYYNRSYVTGL
GINPPMNPHDRRSGADGIWEPWPGYLVGGGWPGPKDWVDIQDSYQTNEIAINWNAALIY
ALAGFVNYNSPQNEVLYGDVNDDGKVNSTDLTLLKRYVLKAVSTLPSSKAEKNADVNRD
GRVNSSDVTILSRYLIRVIEKLPI
11111111110000000000000000000000000000000000000000000000000000000000000000000000
00000000000000000000000000000000000000000000000000000000000000000000000000000000
00000000000000000000000000000000000000000000000000000000000000000000000000000000
00000000000000000000000000000000000000000000000000000000000000000000000000000000
00000000000000000000000000000000000000000000000000000000000000000000000000000000
00000000000000000000000000000000000000000000000000000000000000000000000000000000
00000000000000000000000000000000000000000000000000011111111111111111111111111111
111111111111111111111111111111111111111111
>O43252_1x6v_0_623
MEIPGSLCKKVKLSNNAQNWGMQRATNVTYQAHHVSRNKRGQVVGTRGGFRGCTVWL
TGLSGAGKTTVSMALEEYLVCHGIPCYTLDGDNIRQGLNKNLGFSPEDREENVRRIAEVAK
LFADAGLVCITSFISPYTQDRNNARQIHEGASLPFFEVFVDAPLHVCEQRDVKGLYKKARA
GEIKGFTGIDSEYEKPEAPELVLKTDSCDVNDCVQQVVELLQERDIVPVDASYEVKELYVPE
NKLHLAKTDAETLPALKINKVDMQWVQVLAEGWATPLNGFMREREYLQCLHFDCLLDG
GVINLSVPIVLTATHEDKERLDGCTAFALMYEGRRVAILRNPEFFEHRKEERCARQWGTTCK
NHPYIKMVMEQGDWLIGGDLQVLDRVYWNDGLDQYRLTPTELKQKFKDMNADAVFAFQ
LRNPVHNGHALLMQDTHKQLLERGYRRPVLLLHPLGGWTKDDDVPLMWRMKQHAAVL
EEGVLNPETTVVAIFPSPMMYAGPTEVQWHCRARMVAGANFYIVGRDPAGMPHPETGKD
LYEPSHGAKVLTMAPGLITLEIVPFRVAAYNKKKKRMDYYDSEHHEDFEFISGTRMRKLARE
GQKPPEGFMAPKAWTVLTEYYKSLEKA
11111111111111111111111111111111000000000000000000000000000000000000000000000000
00000000000000000000000000000000000000000000000000000000000000000000000000000000
00000000000000111100000000000000000000000000000000000000000000000000000000000000

00000000000000000000000000000000000000000000000000000000000000000000000000000000000
00000000000000000000000000000000000000000000000000000000000000000000000000000000000
00000000000000000000000000000000000000000000000000000000000000000000000000000000000
00000000000000000000000000000000000000000000000000000000000000000000000000000000000
0000000000000000000000000000000000000000000
>B3TLD6_4jaw_40_662
GYSATAPVNLTRPATVPSMDGWTDGTGAWTLGEGTRVVSSDALAARAQSLASELTKFTDV
DIKAATGSATGKDISLTLDASKKAELGDEGFKLNIGSKGLEVIGATDIGVFYGTRSVSQMLRQ
GQLTLPAGTVATKPKYKERGATLCACQINISTDWIDRFLSDMADLRLNYVLLEMKLKPEED
NTKKAATWSYYTRDDVKKFVKKANNYGIDVIPEINSPGHMNVWLENYPEYQLADNSGRK
DPNKLDISNPEAVKFYKTLIDEYDGVFTTKYWHMGADEYMIGTSFDNYSKLKTFAEKQYG
AGATPNDAFTGFINDIDKYVKAKGKQLRIWNDGIVNTKNVSLNKDIVIEYWYGAGRKPQE
LVQDGYTLMNATQALYWSRSAQVYKVNAARLYNNNWNVGTFDGGRQIDKNYDKLTGA
KVSIWPDSSYFQTENEVEKEIFDGMRFISQMTWSDSRPWATWNDMKADIDKIGYPLDIREY
DYTPVDAGIYDIPQLKSISKGPWELITTPDGYYQMKDTVSGKCLALFTGSKHLDVVTQVGA
RPELRNCADVSVGQDQRNTANERNTQKWQIRADKDGKYTISPALTQQRLAIATGNEQNID
LETHRPAAGTVAQFPADLVSD
00000000000000000000000000000000000000000000000000000000000000000000000000000000000
00000000000000000000000000000000000000000000000000000000000000000000000000000000000
00000000000000000000000000000000000000000000000000000000000000000000000000000000000
00000000000000000000000000000000000000000000000000000000000000000000000000000000000
00000000000000000000000000000000000000000000000000000000000000000000000000000000000
00000000000000000000000000000000000000000000000000000000000000000000000000000000000
00000000000000000000000000000000000000000000000000000000000000000000000000000000000
000000000000000000000000000000000000000001
>Q11SE1_3ilv_1_623
STIRIGGAAVNQTPIDWENNVKNILDAIEEAKNANVEILCLPELCITGYGCEDLFLTDWVAE
TAIEYCFEIAASCTDITVSLGLPMRIAGITYNCVCLVENGIVKGFSAKQFLANEGVHYETRWF
TAWPRNHTTTFLYNDVKYPFGDVLYNVKDARIGFEICEDAWRTDRVGIRHYEKGATLVLNP
SASHFAFGKSAIRYDLVIGGSERFDCTYVYANLLGNEAGRMIYDGEVLIAHKGKLIQRNDRL
SFKNVNLIYADIATDSAETPETVLTQDDLEKEFEFWEATSLGLFDYMRKSRSKGFVLSLSGGA
DSSACAIMVAEMIRKGLKELGLTAFLQKSNMETLFDLPALQHLPFEEQAKKITAVFLTTAYQ
STRNSGDETYTSAKTLAESIGATFYNWSVDEEIEQYKATIENVIERPLTWEKDDITLQNIQAR
GRAPIIWMLTNVKQALLITTSNRSEGDVGYATMDGDTAGGIAPIAGVDKDFIRSWLRWAEK
NRNQHGLHIVNKLAPTAELRPSEYTQTDERDLMPYDVLARIERKAIKERLSPVQVYTALLTE
GPYTKNEFKYWVKKFFRLWSINQWKRERLAPSFHMDDFNIDPRSWYRFPILSSGFAKELND
LDQ
00000000000000000000000000000000000000000000000000000000000000000000000000000000000
00000000000000000000000000000000000000000000000000000000000000000000000000000000011
11111111000000000000000000000000000000000000000000000000000000000000000000000000000
00000000000001111111111111100000000000000000000000000000000000000000000000000000000
00000000000000000000000000000000000000000000000000000000000000000000000000000000000
00000000000000000000000000000000000000000000000000000000000000000000000000000000000
00000000001111111111111111110000000000000000000000000000000000000000000000000000000
000000000000000000000000000000000000000000
>Q9NQW7_3ctz_0_622
MPPKVTSELLRQLRQAMRNSEYVTEPIQAYIIPSGDAHQSEYIAPCDCRRAFVSGFDGSAGT
AIITEEHAAMWTDGRYFLQAAKQMDSNWTLMKMGLKDTPTQEDWLVSVLPEGSRVGVD
PLIIPTDYWKKMAKVLRSAGHHLIPVKENLVDKIWTDRPERPCKPLLTLGLDYTGISWKDK
VADLRLKMAERNVMWFVVTALDEIAWLFNLRGSDVEHNPVFFSYAIIGLETIMLFIDGDRID
APSVKEHLLLDLGLEAEYRIQVHPYKSILSELKALCADLSPREKVWVSDKASYAVSETIPKDH

RCCMPYTPICIAKAVKNSAESEGMRRAHIKDAVALCELFNWLEKEVPKGGVTEISAADKAE
EFRRQQADFVDLSFPTISSTGPNGAIIHYAPVPETNRTLSLDEVYLIDSGAQYKDGTTDVTRT
MHFGTPTAYEKECFTYVLKGHIAVSAAVFPTGTKGHLLDSFARSALWDSGLDYLHGTGHG
VGSFLNVHEGPCGISYKTFSDEPLEAGMIVTDEPGYYEDGAFGIRIENVVLVVPVKTKYNFN
NRGSLTFEPLTLVPIQTKMIDVDSLTDKECDWLNNYHLTCRDVIGKELQKQGRQEALEWLI
RETQPISKQH
11000000000000000000000000000000000000000000000000000000000000000000000000000000000
00000000000000000000000000000000000000000000000000000000000000000000000000000000000
00000000000000000000000000000000000000000000000000000000000000000000000000000000000
00000000000000000000000000000000000000000000000000000000000000000000000000000000000
00000000000000000000000000000000000000000000000000000000000000000000000000000000000
00000000000000000000000000000000000000000000000000000000000000000000000000000000000
00000000000000000000000000000000000000000000000000000000000000000000000000000000000
000000000000000000000000000000000000001111
>Q8U1K3_1b25_8_626
MYGWWGRILRVNLTTGEVKVQEYPEEVAKKFIGGRGLAAWILWNEARGVEPLSPENKLIF
AAGPFNGLPTPSGGKLVVAAKSPLTGGYGDGNLGTMASVHLRRAGYDALVVEGKAKKPV
YIYIEDDNVSILSAEGLWGKTTFETERELKEIHGKNVGVLTIGPAGENLVKYAVVISQEGRAA
GRPGMGAVMGSKKLKAVVIRGTKEIPVADKEELKKLSQEAYNEILNSPGYPFWKRQGTMA
AVEWCNTNYALPTRNFSDGYFEFARSIDGYTMEGMKVQQRGCPYCNMPCGNVVLDAEG
QESELDYENVALLGSNLGIGKLNEVSVLNRIADEMGMDTISLGVSIAHVMEAVERGILKEGP
TFGDFKGAKQLALDIAYRKGELGNLAAEGVKAMAEKLGTHDFAMHVKGLEVSGYNCYIY
PAMALAYGTSAIGAHHKEAWVIAWEIGTAPIEGEKAEKVEYKISYDPIKAQKVVELQRLRG
GLFEMLTACRLPWVEVGLSLDYYPKLLKAITGVTYTWDDLYKAADRVYSLIRAYWVREFNG
KWDRKMDYPPKRWFTEGLKSGPHKGEHLDEKKYDELLSEYYRIRGWDERGIPKKETLKEL
DLDFVIPELEKVTNLE
00000000000000000000000000000000000000000000000000000000000000000000000000000000000
00000000000000000000000000000000000000000000000000000000000000000000000000000000000
00000000000000000000000000000000000000000000000000000000000000000000000000000000000
00000000000000000000000000000000000000000000000000000000000000000000000000000000000
00000000000000000000000000000000000000000000000000000000000000000000000000000000000
00000000000000000000000000000000000011111110000000000000000000000000000000000000000
00000000000000000000000000000000000000000000000000000000000000000000000000000000000
00000000000000000000000000000000000000
>P0CE12_3g06_165_782
PAEYDAVWSAWRRAAPAEESRGRAAVVQKMRACLNNGNAVLNVGESGLTTLPDCLPAHI
TTLVIPDNNLTSLPALPPELRTLEVSGNQLTSLPVLPPGLLELSIFSNPLTHLPALPSGLCKLWI
FGNQLTSLPVLPPGLQELSVSDNQLASLPALPSELCKLWAYNNQLTSLPMLPSGLQELSVSD
NQLASLPTLPSELYKLWAYNNRLTSLPALPSGLKELIVSGNRLTSLPVLPSELKELMVSGNRLT
SLPMLPSGLLSLSVYRNQLTRLPESLIHLSSETTVNLEGNPLSERTLQALREITSAPGYSGPIIRF
DMAGASAPRETRALHLAAADWLVPAREGEPAPADRWHMFGQEDNADAFSLFLDRLSETE
NFIKDAGFKAQISSWLAQLAEDEALRANTFAMATEATSSCEDRVTFFLHQMKNVQLVHNA
EKGQYDNDLAALVATGREMFRLGKLEQIAREKVRTLALVDEIEVWLAYQNKLKKSLGLTSV
TSEMRFFDVSGVTVTDLQDAELQVKAAEKSEFREWILQWGPLHRVLERKAPERVNALREK
QISDYEETYRMLSDTELRPSGLVGNTDAERTIGARAMESAKKTFLDGLRPLVEEMLGSYLNV
11111000000000000000000000000000000000000000000000000000000000000000000000000000000
00000000000000000000000000000000000000000000000000000000000000000000000000000000000
00000000000000000000000000000000000000000000000000000000000000000000000000000000000
00000000000000000000000000000000000000000000000000000000000000000000000011110000000
00000000111111110000000000000000000000000000000000000000000000000000000000000000000
00000000000000000000000000000000000000000000000000000000000000000000000000000000000

00000000000000000000000000000000000000000000000000000000000000000000000000000000000
0000000000000000000000000000000000000
>P94255_2x0q_0_617
MSRTTPPHPAEIVAHLQPEIWNKVNRLLVRKAISEYAHEWLLEPQRLGPGETPGFERFRLTL
ADGAQYDFDAQVMAMRHWRIPPESIVKTVAGVPAPLDALQFVIEIRDKLGLPVDRLPIYM
DEITSTLHGSAYKHGRTTLGAAALARADYQTIETSMIEGHPSFVANNGRLGFDAEDYHGYA
PEAATPVRLMWLAVHKDNAHFSCLSDMDYDSLMSEELGESAVTDFAARLREQGLHPADY
YFMPAHPWQWFNKLSLAFAPYVAQRKIVCLGYGEEQYLAQQSIRTFFNISRPGKRYVKTSLS
ILNMGFMRGLSPYYMAGTPAINEYIHDLISADPWLRANGFRILREVASMGFRNYYYEAAID
TDTPYKKMFSALWRENPLTLIAPGQNLMTMAALLHVDPQGRALLPELIQASGLDAGTWLE
RYVDAYLTPLIHCFYAHDLVFMPHGENVILVIQDGVPVRAFMKDIAEESSILNPQVRLPQAA
QRLAADVPEAYKLLTIFVDVFEGYFRHLTQILVETELMPEHDFWRLVAGRIAAYQQAHPQR
LDKYRRYDLFAPDMIHSCLNRLQLANNLQMVNLADPIGSFQMAPNLPNPIACFRPSWLGS
GEALQTLTAA
11111110000000000000000000000000000000000000000000000000000000000000000000000000000
00000000000000000000000000000000000000000000000000000000000000000000000000000000000
00000000000000000000000000000000000000000000000000000000000000000000000000000000000
00000000000000000000000000000000000000000000000000000000000000000000000000000000000
00000000000000000000000000000000000000000000000000000000000000000000000000000000000
00000000000000000000000000000000000000000000000000000000000000000000000000000000000
00000000000000000000000000000000000000000000000000000000000000000000000000001111111
1111111111000000000000000111111111111
>P75906_4f9d_41_654
QPWPHNGFVAISWHNVEDEAADQRFMSVRTSALREQFAWLRENGYQPVSIAQIREAHRG
GKPLPEKAVVLTFDDGYQSFYTRVFPILQAFQWPAVWAPVGSWVDTPADKQVKFGDELVD
REYFATWQQVREVARSRLVELASHTWNSHYGIQANATGSLLPVYVNRAYFTDHARYETAA
EYRERIRLDAVKMTEYLRTKVEVNPHVFVWPYGEANGIAIEELKKLGYDMFFTLESGLANA
SQLDSIPRVLIANNPSLKEFAQQIITVQEKSPQRIMHIDLDYVYDENLQQMDRNIDVLIQRVK
DMQISTVYLQAFADPDGDGLVKEVWFPNRLLPMKADIFSRVAWQLRTRSGVNIYAWMPVL
SWDLDPTLTRVKYLPTGEKKAQIHPEQYHRLSPFDDRVRAQVGMLYEDLAGHAAFDGILF
HDDALLSDYEDASAPAITAYQQAGFSGSLSEIRQNPEQFKQWARFKSRALTDFTLELSARVK
AIRGPHIKTARNIFALPVIQPESEAWFAQNYADFLKSYDWTAIMAMPYLEGVAEKSADQWL
IQLTNQIKNIPQAKDKSILELQAQNWQKNGQHQAISSQQLAHWMSLLQLNGVKNYGYYP
DNFLHNQPE
10000000000000000000100000000000000000000000000000000000000000000000000000000000000
00000000000000000000000000000000000000000000000000000000000000000000000000000000000
00000000000000000000000000000000000000000000000000000000000000000000000000000000000
00000000000000000000000000000000000000000000000000000000000000000000000000000000000
00000000000000000000000000000000000000000000000000000000000000000000000000000000000
00000000000000000000000000000000000000000000000000000000000000000000000000000000000
00000000000000000000000000000000000000000000000000000000000000000000001111111111100
00000000000000000000000000111111111
>P11439_1ikp_25_637
AEEAFDLWNECAKACVLDLKDGVRSSRMSVDPAIADTNGQGVLHYSMVLEGGNDALKL
AIDNALSITSDGLTIRLEGGVEPNKPVRYSYTRQARGSWSLNWLVPIGHEKPSNIKVFIHELN
AGNQLSHMSPIYTIEMGDELLAKLARDATFFVRAHESNEMQPTLAISHAGVSVVMAQAQP
RREKRWSEWASGKVLCLLDPLDGVYNYLAQQRCNLDDTWEGKIYRVLAGNPAKHDLDIK
PTVISHRLHFPEGGSLAALTAHQACHLPLETFTRHRQPRGWEQLEQCGYPVQRLVALYLAA
RLSWNQVDQVIRNALASPGSGGDLGEAIREQPEQARLALTLAAAESERFVRQGTGNDEAG
AASADVVSLTCPVAAGECAGPADSGDALLERNYPTGAEFLGDGGDISFSTRGTQNWTVER
LLQAHRQLEERGYVFVGYHGTFLEAAQSIVFGGVRARSQDLDAIWRGFYIAGDPALAYGY

AQDQEPDARGRIRNGALLRVYVPRSSLPGFYRTGLTLAAPEAAGEVERLIGHPLPLRLDAIT
GPEEEGGRLETILGWPLAERTVVIPSAIPTDPRNVGGDLDPSSIPDKEQAISALPDYASQPGK
PPREDLK
10000000000000000000000000000000000000000000000000000000000000000000000000000000000
00000000000000000000000000000000000000000000000000000000000000000000000000000000000
00000000000011111100000000000000000000000000000000000000000000000000000000000000000
00000000000000000000000000000000000000000000000000000000000000000000000000000000000
00000000000000000000000000000000000000000000000000000000000000000000000000000000000
00000000000000000000000000000000000000000000000000000000000000000000000000000000000
00000000000000000000000000000000000000000000000000000000000000000000000000000000000
00000000000000000000000001111111
>P09960_2vj8_0_610
MPEIVDTCSLASPASVCRTKHLHLRCSVDFTRRTLTGTAALTVQSQEDNLRSLVLDTKDLTIE
KVVINGQEVKYALGERQSYKGSPMEISLPIALSKNQEIVIEISFETSPKSSALQWLTPEQTSGKE
HPYLFSQCQAIHCRAILPCQDTPSVKLTYTAEVSVPKELVALMSAIRDGETPDPEDPSRKIYK
FIQKVPIPCYLIALVVGALESRQIGPRTLVWSEKEQVEKSAYEFSETESMLKIAEDLGGPYVW
GQYDLLVLPPSFPYGGMENPCLTFVTPTLLAGDKSLSNVIAHEISHSWTGNLVTNKTWDHF
WLNEGHTVYLERHICGRLFGEKFRHFNALGGWGELQNSVKTFGETHPFTKLVVDLTDIDP
DVAYSSVPYEKGFALLFYLEQLLGGPEIFLGFLKAYVEKFSYKSITTDDWKDFLYSYFKDKVD
VLNQVDWNAWLYSPGLPPIKPNYDMTLTNACIALSQRWITAKEDDLNSFNATDLKDLSSH
QLNEFLAQTLQRAPLPLGHIKRMQEVYNFNAINNSEIRFRWLRLCIQSKWEDAIPLALKMA
TEQGRMKFTRPLFKDLAAFDKSHDQAVRTYQEHKASMHPVTAMLVGKDLKVD
10000000000000000000000000000000000000000000000000000000000000000000000000000000000
00000000000000000000000000000000000000000000000000000000000000000000000000000000000
00000000000000000000000000000000000000000000000000000000000000000000000000000000000
00000000000000000000000000000000000000000000000000000000000000000000000000000000000
00000000000000000000000000000000000000000000000000000000000000000000000000000000000
00000000000000000000000000000000000000000000000000000000000000000000000000000000000
00000000000000000000000000000000000000000000000000000000000000000000000000000000000
000000000000000000000000000000
>P49053_3bb0_0_608
MGSVTPIPLPKIDEPEEYNTNYILFWNHVGLELNRVTHTVGGPLTGPPLSARALGMLHLAI
HDAYFSICPPTDFTTFLSPDTENAAYRLPSPNGANDARQAVAGAALKMLSSLYMKPVEQPN
PNPGANISDNAYAQLGLVLDRSVLEAPGGVDRESASFMFGEDVADVFFALLNDPRGASQE
GYHPTPGRYKFDDEPTHPVVLIPVDPNNPNGPKMPFRQYHAPFYGKTTKRFATQSEHFLA
DPPGLRSNADETAEYDDAVRVAIAMGGAQALNSTKRSPWQTAQGLYWAYDGSNLIGTPPR
FYNQIVRRIAVTYKKEEDLANSEVNNADFARLFALVDVACTDAGIFSWKEKWEFEFWRPLS
GVRDDGRPDHGDPFWLTLGAPATNTNDIPFKPPFPAYPSGHATFGGAVFQMVRRYYNGRV
GTWKDDEPDNIAIDMMISEELNGVNRDLRQPYDPTAPIEDQPGIVRTRIVRHFDSAWELMF
ENAISRIFLGVHWRFDAAAARDILIPTTTKDVYAVDNNGATVFQNVEDIRYTTRGTREDPEG
LFPIGGVPLGIEIADEIFNNGLKPTPPEIQPMPQETPVQKPVGQQPVKGMWEEEQAPVVKE
AP
11100000000000000000000000000000000000000000000000000000000000000000000000000000000
00000000000000000000000000000000000000000000000000000000000000000000000000000000000
00000000000000000000000000000000000000000000000000000000000000000000000000000000000
00000000000000000000000000000000000000000000000000000000000000000000000000000000000
00000000000000000000000000000000000000000000000000000000000000000000000000000000000
00000000000000000000000000000000000000000000000000000000000000000000000000000000000
00000000000000000000000000000000000000000000000000000000000000000000000000000000011
1111111111111111111111111111
>Q47Z72_3o0y_23_631

NENNTVVAETKTSTASETSEPKQVISSKQQLASLYLQAKQSLFKQRALSATMYGLSQKDIGQ
VISSDMEFYSPENEKQLRAELLSISNTIAGIKLDDADITTKNNQQVMAGLTRYFAGEPNFNIG
YIDTWMGLSPFIVNQINGPLIDIPRVMQNDQPITTEKEALDYIVRLGQFDKLAATIIEKQTAD
AAQNWLPSKVTLQGAIKYLKGFTSGSAEQHPFVNVFREKIEKVDSLTTEQKQSLITQVIAKV
SQVVYPAYQSVEKASEQLLSEARSESGIWAQPKGSVYYQDAIKQLGDSELSPTQIHQIGLDEV
ARISGVMNEILLAQGYTKGTVGERMVALNEEPRFLYEDSIAGREELLSDINGYITEVTAKMA
PVFRTTPSYQVEVKSFPVEVQDGAPGGQYTSPAVDGSKPGIYWINLRDMKANPKFGLKTLT
YHEANPGHHWQIALNLDQAELPFLRRIAPYNAYTEGWALYSEQVAYELGMYENDPFGDL
GRLQAELFRAVRLVVDTGLHDKRWTREQAISYMSEQTGTAESDVVAEIERYMAWPGQALG
YKLGMLKILSLREQAKARLGDKFDLAEFHDVVLLNGAVPMAVLSRNVNHWLDNK
111111111111111111111111000000000000000000000000000000000000000000000000000000000000
000000000000000000000000000000000000000000000000000000000000000000000000000000000000
000000000000000000000000000000000000000000000000000000000000000000000000000000000000
000000000000000000000000000000000000000000000000000000000000000000000000000000000000
000000000000000000000000000000000000000000000000000000000000000000000000000000000000
000000000000000000000000000000000000000000000000000000000000000000000000000000000000
000000000000000000000000000000000000000000000000000000000000000000000000000000000000
0000000000000000000000000000
>Q62192_3t6q_20_625
TTSSDQKCIEKEVNKTYNCENLGLNEIPGTLPNSTECLEFSFNVLPTIQNTTFSRLINLTFLDL
TRCQIYWIHEDTFQSQHRLDTLVLTANPLIFMAETALSGPKALKHLFFIQTGISSIDFIPLHNQ
KTLESLYLGSNHISSIKLPKGFPTEKLKVLDFQNNAIHYLSKEDMSSLQQATNLSLNLNGNDI
AGIELGAFDSAVFQSLNFGGTQNLLVIFKGLKNSTIQSLWLGTFEDMDDEDISPAVFEGLCE
MSVESINLQKHYFFNISSNTFHCFSGLQELDLTATHLSELPSGLVGLSTLKKLVLSANKFENL
CQISASNFPSLTHLSIKGNTKRLELGTGCLENLENLRELDLSHDDIETSDCCNLQLRNLSHL
QSLNLSYNEPLSLKTEAFKECPQLELLDLAFTRLKVKDAQSPFQNLHLLKVLNLSHSLLDISS
EQLFDGLPALQHLNLQGNHFPKGNIQKTNSLQTLGRLEILVLSFCDLSSIDQHAFTSLKMM
NHVDLSHNRLTSSSIEALSHLKGIYLNLASNRISIILPSLLPILSQQRTINLRQNPLDCTCSNIYF
LEWYKENMQKLEDTEDTLCENPPLLRGVRLSDVTLSCS
111110000000000000000000000000000000000000000000000000000000000000000000000000000000
000000000000000000000000000000000000000000000000000000000000000000000000000000000000
000000000000000000000000000000000000000000000000000000000000000000000000000000000000
000000000000000000000000000000000000000000000000000000000000000000000000000000000000
000000000000000000000000000000000000000000000000000000000000000000000000000000000000
000000000000000000000000000000000000000000000000000000000000000000000000000000000000
000000000000000000000000000000000000000000000000000000000000000000000000000000000000
0000000000000000000000000
>Q8GCA3_2ewf_0_603
MAKKVNWYVSCSPRSPEKIQPELKVLANFEGSYWKGVKGYKAQEAFAKELAALPQFLGTT
YKKEAAFSTRDRVAPMKTYGFVFVDEEGYLRITEAGKMLANNRRPKDVFLKQLVKWQYPS
FQHKGKEYPEEEWSINPLVFVLSLLKKVGGLSKLDIAMFCLTATNNNQVDEIAEEIMQFRNE
REKIKGQNKKLEFTENYFFKRFEKIYGNVGKIREGKSDSSHKSKIETKMRNARDVADATTRY
FRYTGLFVARGNQLVLNPEKSDLIDEIISSSKVVKNYTRVEEFHEYYGNPSLPQFSFETKEQLL
DLAHRIRDENTRLAEQLVEHFPNVKVEIQVLEDIYNSLNKKVDVETLKDVIYHAKELQLEL
KKKKLQADFNDPRQLEEVIDLLEVYHEKKNVIEEKIKARFIANKNTVFEWLTWNGFIILGN
ALEYKNNFVIDEELQPVTHAAGNQPDMEIIYEDFIVLGEVTTSKGATQFKMESEPVTRHYL
NKKKELEKQGVEKELYCLFIAPEINKNTFEEFMKYNIVQNTRIIPLSLKQFNMLLMVQKKLI
EKGRRLSSYDIKNLMVSLYRTTIECERKYTQIKAGLEETLNNWVVDKEVRF
000000000000000000000000000000000000000000000000000000000111111100000000000000000000
000000000000000000000000000000000000000000000000000000000000000000000000000000000000
000000000000000000000000000000000000000000001111111111000000000000000000000000000000

00000000000000000000000000000000000000000000000000000000000000000000000000000000000
00000000000000000000000000000000000000000000000000000000000000000000000000000000000
00000000000000000000000000000000000000000000000000000000000000000000000000000000000
00000000000000000000000000000000000000000000000000000000000000000000000000000000000
00000000000000000000000
>Q5SHU6_2d5w_20_621
GPQDNSLVIGASQEPRVLAGDFLRVISNQAIKSEIEQYLFAPFIGFNADSQNFPVLATEVPTLE
NGRLRVTDIGGGKKRLEMDITIRPDAKWSDGRPITTEDVAFYFEVGKAKGMPVLNPDFWE
RVNVRIKDARNFTLIFEPAYYYDTYGPINTYAPKHIMGPEWERVKAAARGLDPDKDAEKLN
ELYRNFFLKFATPQALNRGAMVYSGPFKLKRWVPGNSIEMERNPNFPIKPEGGESKYVQKV
VYRFIQNTNSLLVAVIGGSIDATSSVSLTFDQGRSPQLVRRAPGRFDIWFVPGAIWEHIDINKF
ENCQVVKDLGLNDKRTRQAILHALNREGLVKAFFDGLQPVAHTWIAPVNPLFNPNVKKY
EFDLKKAEALLAEMGWRKGPDGILQRTVNGRTVRFEIEYVTTAGNVVRERTQQFFAEDLK
KIGIAVKINNAPSAVVFADEFIQRASECKWTGMFEFAWVSNLQEDGSLFQYKNLNTGAIMV
PTKENNYQGQNIGGWRNDEFDRLTSQAVLEFDPERRKQLFWRAQEIWAEELPALPLYFRA
NPYVVRKGLVNYVASAYSGGYGYPGWNAWEIGWESRGAVKKWDQAKYALSTR
00000000000000000000000000000000000000000000000000000000000000000000000000000000000
00000000000000000000000000000000000000000000000000000000000000000000000000000000000
00000000000000000000000000000000000000000000000000000000000000000000000000000000000
00000000000000000000000000000000000000000000000000000000000000000000000000000000000
00000000000000000000000000000000000000000000000000000000000000000000000000000000000
00000000000000000000000000000000000000000000000000000000000000000000000000000000000
00000000000000000000000000000000000000000000000000000000000000000000000000000000000
000000000000000000001
>Q8KKG0_3edj_18_618
AAPTAIEHMEPPFWWAGMQHKGLQLMVHGRDIGRMEAALDYPGVRLVSTTRVPNANYL
FVDLEIGPEAQPGSFDIVFKGDGRSERYRYRLLAREQGSAQRQGFGPGDAIYQIMPDRFANG
DPSNDNVAGMREQADRRHGGGRHGGDIRGTIDHLDYIAGLGFTQLWPTPLVENDAAAYS
YHGYAATDHYRIDPRYGSNEDFVRLSTEARKRGMGLIQDVVLSHIGKHHWWMKDLPTPD
WINYGGKFVPTQHHRVAVQDPYAAQADSENFTKGWFVEGMPDLNQTNPLVANYLIQNNI
WWIEYAGLSGLRIDTYGYSDGAFLTEYTRRLMAEYPRLNMVGEEWSTRVPVVARWQRGKA
NFDGYTSHLPSLMDFPLVDAMRNALSKTGEENGLNEVYETLSLDYLYPEPQNLVLFGGNH
DMARMFSAAGEDFDRWRMNLVFLMTMPRIPQFYSGDEILMTSTVKGRDDASYRRDFPGG
WAGDKANAFSGAGLTSQQRAAQDLVRKLANWRKNQPVIHNGRLMHFGPEENTWVYFR
YNKDKRIMVAMNNNDKPMTLPTARFQEMLKGAPSGVDFLSGKTVGLGRELRLAPKSVVVI
ELPGLPEA
11000000000000000000000000000000000000000000000000000000000000000000000000000000000
00000000000000000000000000000000000000000000000000000000000000000000000000000000000
00000000000000000000000000000000000000000000000000000000000000000000000000000000000
00000000000000000000000000000000000000000000000000000000000000000000000000000000000
00000000000000000000000000000000000000000000000000000000000000000000000000000000000
00000000000000000000000000000000000000000000000000000000000000000000000000000000000
00000000000000000000000000000000000000000000000000000000000000000000000000000000000
00000000000000000011
>Q9AYY6_2x6x_110_709
DPDQFRAIIESPEGAGHVGYQYRRNTGSTMRMVSDVLDERVSLWDFHCDPSGNVIQPGPN
VDSRQYLQAAIDYVSSNGGGTITIPAGYTWYLGSYGVGGIAGHSGIIQLRSNVNLNIEGRIHL
SPFFDLKPFQVFVGFDNGDPASSGNLENCHIYGHGVVDFGGYEFGASSQLRNGVAFGRSYN
CSVTGITFQNGDVTWAITLGWNGYGSNCYVRKCRFINLVNSSVNADHSTVYVNCPYSGVE
SCYFSMSSSFARNIACSVELHQHDTFYRGSTVNGYCRGAYVVMHAAEAAGAGSYAYNMQ
VENNIAVIYGQFVILGSDVTATVSGHLNDVIVSGNIVSIGERAAFSAPFGAFIDIGPDNSGAS

NVQDIQRVLVTGNSFYAPANITDSAAITLRANLNGCTFIANNFDCRYMVYNAPGTTSPVVQ
NLVWDKSNVIGGTHANQRAGQNLFDMQFASVVNSTIEVQLSCEDLSMFSCILFPASCQLSY
SKITVDSAWTKSMSNTAVFEGNQQAGANVYVSYPATVNLTSYNTQGAVPFFSTDTNYAWV
TSAYSLSINENLDFSPPATYTNKANGQLVGVGYNEIGGVRSVSVRLMLQRQV
11000000000000000000000000000000000000000000000000000000000000000000000000000000000
00000000000000000000000000000000000000000000000000000000000000000000000000000000000
00000000000000000000000000000000000000000000000000000000000000000000000000000000000
00000000000000000000000000000000000000000000000000000000000000000000000000000000000
00000000000000000000000000000000000000000000000000000000000000000000000000000000000
00000000000000000000000000000000000000000000000000000000000000000000000000000000000
00000000000000000000000000000000000000000000000000000000000000000000000000000000000
0000000000000000000
>Q9L5A4_3hjr_24_623
NEGCAPLTGKESGMDIGRSSTERCLPGANPLQDQQWYLLNSGQDGFSARGGIAGNDLNL
WWAHRTGVLGQGVNVAVVDDGLAIAHPDLADNVRPGSKNVVTGSDDPTPTDPDTAHGT
SVSGIIAAVDNAIGTKGIAPRAQLQGFNLLDDNSQQLQKDWLYALGDSNASRDNRVFNQS
YGMSVVDPRSANSLDQSQLDRLFEQQTLKAQGAAYIKAAGNGFNKIAAGGYVLNRTGNG
PKLPFENSNLDPSNSNFWNLVVSALNADGVRSSYSSVGSNIFLSATGGEYGTDTPAMVTTDL
PGCDMGYNRTDDPSTNRLHGNSQLDASCDYNGVMNGTSSATPSTSGAMALLMSAYPDLS
VRDLRDLLARSATRVDAKHQPVMVSYTSSTGKVRDVKGLEGWERNAAGMWFSPTYGFGL
IDVNKALELAANHQPLPPLVQLPWQKINVTGSAAAIADVGNSPTSSTTRIATPLTVEAVQV
MVSLDHQRLPDLLIELVSPAGTRSILLSPFNSLVGQSLDQQQLGFVRTKGLRDMRMLSNKFY
GESAQGTWRLEVTDVANGTRQVSLLNRETRERTTLTERNNRQPGKLISWSLRVLGHDANR
S
11000000000000000000000000000000000000000000000000000000000000000000000000000000000
00000000000000000000000000000000000000000000000000000000000000000000000000000000000
00000000000000000000000000000000000000000000000000000000000000000000000000000000000
00000000000000000000000000000000000000000000000000000000000000000000000000000000000
00000000000000000000000000000000000000000000000000000000000000000000000000000000000
00000000000000000000000000000000000000000000000000000000000000000000000000000000000
00000000000000000000000000000000000000000000000000000000000000000000000000000000000
0000000000000011111
>Q9RX51_2bhy_0_599
MTQTQPVTPTPPASFQTQHDPRTRLGATPLPGGAGTRFRLWTSTARTVAVRVNGTEHVMTS
LGGGIYELELPVGPGARYLFVLDGVPTPDPYARFLPDGVHGEAEVVDFGTFDWTDADWHG
IKLADCVFYEVHVGTFTPEGTYRAAAEKLPYLKELGVTAIQVMPLAAFDGQRGWGYDGA
AFYAPYAPYGRPEDLMALVDAAHRLGLGVFLDVVYNHFGPSGNYLSSYAPSYFTDRFSSAW
GMGLDYAEPHMRRYVTGNARMWLRDYHFDGLRLDATPYMTDDSETHILTELAQEIHELG
GTHLLLAEDHRNLPDLVTVNHLDGIWTDDFHHETRVTLTGEQEGYYAGYRGGAEALAYTI
RRGWRYEGQFWAVKGEEHERGHPSDALEAPNFVYCIQNHDQIGNRPLGERLHQSDGVTL
HEYRGAAALLLPMTPLLFQGQEWAASTPFQFFSDHAGELGQAVSEGRKKEFGGFSGFSGED
VPDPQAEQTFLNSKLNWAEREGGEHARTLRLYRDLLRLRREDPVLHNRQRENLTTGHDG
DVLWVRTVTGAGERVLLWNLGQDTRAVAEVKLPFTVPRRLLLHTEGREDLTLGAGEAVLV
G
11111111111110000000000000000000000000000000000000000000000000000000000000000000000
00000000000000000000000000000000000000000000000000000000000000000000000000000000000
00000000000000000000000000000000000000000000000000000000000000000000000000000000000
00000000000000000000000000000000000000000000000000000000000000000000000000000000000
00000000000000000000000000000000000000000000000000000000000000000000000000000000000
00000000000000000000000000000000000000000000000000000001111111110000000000000000000
00000000000000000000000000000000000000000000000000000000000000000000000000000000000

0000000000000000000
>B8CZ54_3bc9_24_622
GCSNISEDVNNPNRSLFLIESEPSTGASVSKNLTEIILIFSNDINKVSQLALTDLITDSDIQGIDY
NIEGNKVIINNFSLEPTCNYRLSYEVIDIYDNHLQGYIEFLVNQSNYPQIPDQEVNHTILQAF
YWEMNTGEYATEHPEEANLWNLLAERAPELAEAGFTAVWLPPANKGMAGIHDVGYGTY
DLWDLGEFDQKGTVRTKYGTKGELENAIDALHNNDIKVYFDAVLNHRMGADYAETVLLD
ENSRDKPGQYIKAWTGFNFPGRNGEYSNFTWNGQCFDGTDWDDYSKESGKYLFDEKSWD
WTYNWDEDYLMGADVDYENEAVQNDVIDWGQWIINNIDFDGFRLDAVKHIDYRFIDKW
MSAVQNSSNRDVFFVGEAWVEDVDDLKGFLDTVGNPDLRVFDFPLRSFFVDMLNGAYMA
DLRNAGLVNSPGYENRAVTFVDNHDTDRDEGSYTVSIYSRKYQAYAYILTRAEGVPTVYWK
DYYIWEMKEGLDKLLTARRYYAYGPGYEVDNNDADIYSYVRSGFPDVAGDGLVLMISDGTS
GNVAGKWINSRQPDTEFYDLTGHIKEHVTTDSEGYGNFKVIKSEDKGWSIWVPVE
11111111111111000000000000000000000000000000000000000000000000000000000000000000000
00000000000000000000000000000000000000000000000000000000000000000000000000000000000
00000000000000000000000000000000000000000000000000000000000000000000000000000000000
00000000000000000000000000000000000000000000000000000000000000000000000000000000000
00000000000000000000000000000000000000000000000000000000000000000000000000000000000
00000000000000000000000000000000000000000000000000000000000000000000000000000000000
00000000000000000000000000000000000000000000000000000000000000000000000000000000000
000000000000000000
>Q962Y6_2x8h_0_595
MPPADGTSQWLRKTVDSAAVILFSKTTCPYCKKVKDVLAEAKIKHATIELDQLSNGSAIQK
CLASFSKIETVPQMFVRGKFIGDSQTVLKYYSNDELAGIVNESKYDYDLIVIGGGSGGLAAG
KEAAKYGAKTAVLDYVEPTPIGTTWGLGGTCVNVGCIPKKLMHQAGLLSHALEDAEHFG
WSLDRSKISHNWSTMVEGVQSHIGSLNWGYKVALRDNQVTYLNAKGRLISPHEVQITDKN
QKVSTITGNKIILATGERPKYPEIPGAVEYGITSDDLFSLPYFPGKTLVIGASYVALECAGFLAS
LGGDVTVMVRSILLRGFDQQMAEKVGDYMENHGVKFAKLCVPDEIKQLKVVDTENNKP
GLLLVKGHYTDGKKFEEEFETVIFAVGREPQLSKVLCETVGVKLDKNGRVVCTDDEQTTVS
NVYAIGDINAGKPQLTPVAIQAGRYLARRLFAGATELTDYSNVATTVFTPLEYGACGLSEED
AIEKYGDKDIEVYHSNFKPLEWTVAHREDNVCYMKLVCRKSDNMRVLGLHVLGPNAGEIT
QGYAVAIKMGATKADFDRTIGIHPTCSETFTTLHVTKKSGVSPIVSGC
11111000000000000000000000000000000000000000000000000000000000000000000000000000000
00000000000000000000000000000000000000000000000000000000000000000000000000000000000
00000000000000000000000000000000000000000000000000000000000000000000000000000000000
00000000000000000000000000000000000000000000000000000000000000000000000000000000000
00000000000000000000000000000000000000000000000000000000000000000000000000000000000
00000000000000000000000000000000000000000000000000000000000000000000000000000000000
00000000000000000000000000000000000000000000000000000000000000000000000000000000000
000000000000111
>C0SSE7_3a9s_0_594
MAKDPRYVGNLPKIGIRPTIDGRRKGVRESLEETTMNMAKAVAKLLEENVFYYNGQPVEC
VIADTCIGGVKEAAEAAEKFAREGVGVSITVTPCWCYGTETMDMDPHIPKAVWGFNGTER
PGAVYLAAVLAGYNQKGLPAFGIYGKDVQDAGDTNIPEDVKEKLIRFAKAGLAVAMMKG
KSYLSIGSVSMGIAGSVVQEDFFQNYLGMRNEYVDMSEFVRRIELEIYDKEEYERALKWVKE
NCKVGPDNNRDGFKRTEEQKEKDWEISVKMALIARDLMVGNKKLEEMGYGEEALGRNAI
VAGFQGQRQWTDYFPNGDFMETILNSSFDWNGKRAPYIFATENDNLNGISMLFGYLLTNT
AQIFADVRTYWSPEAVKRVTGYTLEGRAANGIIHLINSGAAALDGTGEQTKDGKPVIKPYY
ELTDEDIKKCLEATQFRPASTEYFRGGGYSTDFLTKGGMPVTISRLNIVKGLGPVLQIAEGYT
VDLPEEVHDVLDKRTDPTWPTTWFVPNLTGEGAFKDVYSVMNNWGANHCSISYGHIGAD
LITLASILRIPVNMHNVPEEKIFRPDAWSMFGTKDLEGADYRACKNFGPIYK
10000000000000000000000000000000000000000000000000000000000000000000000000000000000

00000000000000000000000000000000000000000000000000000000000000000000000000000000000
00000000000000000000000000000000000000000000000000000000000000000000000000000000000
00000000000000000000000000000000000000000000000000000000000000000000000000000000000
00000000000000000000000000000000000000000000000000000000000000000000000000000000000
00000000000000000000000000000000000000000000000000000000000000000000000000000000000
00000000000000000000000000000000000000000000000000000000000000000000000000000000000
00000000011111
>Q81XJ1_3owa_0_593
MEKTVGNAVKGGSFLVDEITIDQVFTPEDFSSEHKMIAKTTEDFIVNEVLPELEYLEQHEFDR
SVRLLKEAGELGLLGADVPEEYGGIGLDKVSSALIAEKFSRAGGFAITHGAHVGIGSLPIVLF
GNEEQKKKYLPLLATGEKLAAYALTEPGSGSDALGAKTTARLNAEGTHYVLNGEKQWITN
SAFADVFIVYAKIDGEHFSAFIVEKDYAGVSTSPEEKKMGIKCSSTRTLILEDALVPKENLLGE
IGKGHIIAFNILNIGRYKLGVGTVGSAKRAVEISAQYANQRQQFKQPIARFPLIQEKLANMA
AKTYAAESSVYRTVGLFESRMSTLSEEEVKDGKAVAASIAEYAIECSLNKVFGSEVLDYTVDE
GVQIHGGYGFMAEYEIERMYRDSRINRIFEGTNEINRLIVPGTFLRKAMKGELPLLQKAQKL
QEELMMMMPEEVGDEPLALQKYLVNNAKKIGLMVAGLAAQKYGKALDKEQEILVNIADI
VSNLYAMESAVLRTEKAIKTTGLEKNKQKVLYTEVFCQEAFNEIEAHAKETLIAVENGDML
RMMLSSLRKLTRHTPLNVIPKKREIAAKILEDERYTV
11111110000000000000000000000000000000000000000000000000000000000000000000000000000
00000000000000000000000000000000000000000000000000000000000000000000000000000000000
00000000000000000000000000000000000000000000000000000000000000000000000000000000000
00000000000000000000000000000000000000000000000000000000000000000000000000000000000
00000000000000000000000000000000000000000000000000000000000000000000000000000000000
00000000000000000000000000000000000000000000000000000000000000000000000000000000000
00000000000000000000000000000000000000000000000000000000000000000000000000000000000
0000000000000
>Q8ZPF0_3m07_0_593
MSSKIFCKSWGAEYIAADVVRFRLWATGQQKVMLRLAGKDQEMQANGDGWFTLDVAGV
TPGTEYNFVLSDGMVVPDPASRAQKTDVNGPSYVVDPGSYTWRNTGWKGSRWEQAVVYE
MHTGTFTPEGTFRAAIAKLPYLAELGVTVIEVMPVAQFGGERGWGYDGVLLYAPHSAYGTP
DDFKAFIDAAHGYGLSVVLDIVLNHFGPEGNYLPLLAPAFFHKERMTPWGNGIAYDVDAV
RRYIIEAPLYWLTEYHLDGLRFDAIDQIEDSSARHVLVEIAQRIREDITDRPIHLTTEDSRNIISL
HPRDQDGNAPLFTAEWNDDFHNAVHVFATGETQAYYNDFADAPEKHLARALAEGFAYQ
GEISPQTGEPRGVKSTGQPPVAFVDFIQNHDQVGNRAQGDRLITLAGAERTKVLLATLLLSP
HIPLLFMGEEYGESRPFLFFTDFHGDLARAVREGRAKEFADHAGENVPDPNAPETFQRSKL
NWKQQHSEEGKAWLAFTRELLLLRQKHIVPLLSAARESSGTVLQTAPGFIAVSWRFPGGTL
SLALNISATTVLLPDLPGKTLFAWPNESTGSLSQHSLIVRLAQGESAS
11111000000000000000000000000000000000000000000000000000000000000000000000000000000
00000000000000000000000000000000000000000000000000000000000000000000000000000000000
00000000000000000000000000000000000000000000000000000000000000000000000000000000000
00000000000000000000000000000000000000000000000000000000000000000000000000000000000
00000000000000000000000000000000000000000000000000000000000000000000000000000000000
00000000000000000000000000000000000000000000000000000000000000000000000000000000000
00000000000000000000000000000000000000000000000000000000000000000000000000000000000
0000000011111
>P32455_1dg3_0_591
MASEIHMTGPMCLIENTNGRLMANPEALKILSAITQPMVVVAIVGLYRTGKSYLMNKLAG
KKKGFSLGSTVQSHTKGIWMWCVPHPKKPGHILVLLDTEGLGDVEKGDNQNDSWIFALA
VLLSSTFVYNSIGTINQQAMDQLYYVTELTHRIRSKSSPDENENEVEDSADFVSFFPDFVWTL
RDFSLDLEADGQPLTPDEYLTYSLKLKKGTSQKDETFNLPRLCIRKFFPKKKCFVFDRPVHR
RKLAQLEKLQDEELDPEFVQQVADFCSYIFSNSKTKTLSGGIQVNGPRLESLVLTYVNAISSG

DLPCMENAVLALAQIENSAAVQKAIAHYEQQMGQKVQLPTETLQELLDLHRDSEREAIEV
FIRSSFKDVDHLFQKELAAQLEKKRDDFCKQNQEASSDRCSALLQVIFSPLEEEVKAGIYSKP
GGYRLFVQKLQDLKKKYYEEPRKGIQAEEILQTYLKSKESMTDAILQTDQTLTEKEKEIEVER
VKAESAQASAKMLQEMQRKNEQMMEQKERSYQEHLKQLTEKMENDRVQLLKEQERTLA
LKLQEQEQLLKEGFQKESRIMKNEIQDLQTKMRRRKACTIS
11111000000000000000000000000000000000000000000000000000001111111111100000000000
00000000000000000000000000000000000000000000000000000000000000000000000001111111111
00000000000000000000000111100000000000000000000000000000000000000000000000000111111
11111110000000000000000000000000000000000000000000000000000000000000000000000000000
00000000000000000000000000000000000000000000000000000000000000000000000000000000000
00000000000000000000000000000000000000000000000000000000000000000000000000000000000
00000000000000000000000000000000000000000000000000000000000000000000000000000000000
00111111111
>Q16820_4gwm_24_613
ENFDVDGGMDQDIFDINEGLGLDLFEGDIRLDRAQIRNSIIGEKYRWPHTIPYVLEDSLEMN
AKGVILNAFERYRLKTCIDFKPWAGETNYISVFKGSGCWSSVGNRRVGKQELSIGANCDRIA
TVQHEFLHALGFWHEQSRSDRDDYVRIMWDRILSGREHNFNTYSDDISDSLNVPYDYTSV
MHYSKTAFQNGTEPTIVTRISDFEDVIGQRMDFSDSDLLKLNQLYNCSSSLSFMDSCSFELEN
VCGMIQSSGDNADWQRVSQVPRGPESDHSNMGQCQGSGFFMHFDSSSVNVGATAVLESR
TLYPKRGFQCLQFYLYNSGSESDQLNIYIREYSADNVDGNLTLVEEIKEIPTGSWQLYHVTLK
VTKKFRVVFEGRKGSGASLGGLSIDDINLSETRCPHHIWHIRNFTQFIGSPNGTLYSPPFYSSK
GYAFQIYLNLAHVTNAGIYFHLISGANDDQLQWPCPWQQATMTLLDQNPDIRQRMSNQR
SITTDPFMTTDNGNYFWDRPSKVGTVALFSNGTQFRRGGGYGTSAFITHERLKSRDFIKGDD
VYILLTVEDISHLNSTQIQLTPAPSVQDLCSKTTCK
00000000000000000000000000000000000000000000000000000000000000000000000000000000000
00000000000000000000000000000000000000000000000000000000000000000000000000000000000
00011111000000000000000000000000000000000000000000000000000000000000000000000000000
00000000000000000000000000001111100000000000000000000000000000000000000000000000000
00000000000000000000000000000000000000000000000000000000000000000000000000000000000
00000000000000000000000000000000000000000000000000000000000000000000000000000000000
00000000000000000000000000000000000000000000000000000000000000000000000000011111111
111111111
>B7TVP1_3gf3_0_587
MNMYSMPGYFQNMPTIGKELVNPNPENEQEIKAVESDIHESIKKALDAGITSEEKLNERGQ
LSAMQRINALIDPGTWCPLNSLFNPENNKFGTTNIVNGLGRVDGKWVYIVASDNKKMAG
AWVPGQAENLIRCSDAAKMMHLPLIYLLNCSGVEFPNQDKVYPNRRGGGTPFFRNSELN
QLGIPVIVGIYGTNPAGGGYHSISPTILIAHQDANMAVGGAGILSGMNPKGYIDDEAAEQII
AAQIENSKLKVPAPGSVPIHYDETGFFREVYQNDLGVIDGIKKYISYLPAYNLEFFRVDTPKA
PQLPAEDLYSIIPMNQKRPYDIYEVIARLFDNSEFSEYKKGYGPEMVTGLAKVNGLLVGVIA
NVQGLLMNYPEYKQNSVGIGGKLYRQGLIKMNEFVTLCARDRIPLIWLQDTTGIDVGDEA
EKAELLGLGQSLIYSIENSKLPSLEITIRKASAAAHYVLGGPQGNNTNVFSIGTGACEYYVMP
GETAANAMYSRKLVKAKKAGEDLQPIIGKMNDMIQMYTDKSRPKYCTEKGMVDEIVDMT
EVRPYIQAFTEAAYQNPQSICPMHQMLTPRSTREFETFGK
11000000000000000000000000000000000000000000000000000000000000000000000000000000000
00000000000000000000000000000000000000000000000000000000000000000000000000000000000
00000000000000000000000000000000000000000000000000000001111111111111110000000000000
00000000000000000000000000000000000000000000000000000000000000000000000000000000000
00000000000000000000000000000000000000000000000000000000000000000000000000000000000
00000000000000000000000000000000000000000000000000000000000000000000000000000000000
00000001111111000000000000000000000000000000000000000000000000000000000000000000000
0000001

>Q9QYM2_3uek_384_971
NSSSLNVDGRSSKQHGKRDSKITDHFVRIPKSEDKRKEQCEVRHQRAERKIPKYVPPNLPPD
KKWLGTPIEEMRKMPRCGVRLPLLRPSASHTVTVRVDLLRAGEVPKPFPTHYKDLWDNKH
VKMPCSEQNLYPVEDENGERTAGSRWELIQTALLNKFTRPQNLKDAILKYNVAYSKKWDF
TALVDFWDKVLEEAEAQHLYQSILPDMVKIALCLPNICTQPIPLLKQKMNHSVTMSQEQIA
SLLANAFFCTFPRRNAKMKSEYSSYPDINFNRLFEGRSSRKPEKLKTLFCYFRRVTEKKPTGL
VTFTRQSLEDFPEWERCDKPLTRLHVTYEGTIEGNGRGMLQVDFANRFVGGGVTGAGLVQ
EEIRFLINPELIVSRLFTEVLDHNECLIITGTEQYSEYTGYAETYRWARSHEDGSEKDDWQRC
CTEIVAIDALHFRRYLDQFVPEKVRRELNKAYCGFLRPGVPPENLSAVATGNWGCGAFGGD
ARLKALIQLLAAAAAERDVVYFTFGDSELMRDIYSMHTFLTERKLNVGKVYRLLLRYYREE
CRDCSSPGPDTKLYPFIYHAAESSAETSDQPGQRTGT
11111111111111111111111111111111111111111111111111111000000000000000000000000000000
00000000000000000000000000000000000000000000000000000000000000000000000000000000000
00000000000000000000000000000000000000000000000000000000000000000000000000000000000
00000000000000000000000000000000000000000000000000000000000000000000000000000000000
00000000000000000000000000000000000000000000000000000000000000000000000000000000000
00000000000000000000000000000000000000000000000000000000000000000000000000000000000
00000000000000000000000000000000000000000000000000000000000000000000000000001111111
1111111
>P49748_3b96_68_654
ESKSFAVGMFKGQLTTDQVFPYPSVLNEEQTQFLKELVEPVSRFFEEVNDPAKNDALEMVEE
TTWQGLKELGAFGLQVPSELGGVGLCNTQYARLVEIVGMHDLGVGITLGAHQSIGFKGILL
FGTKAQKEKYLPKLASGETVAAFCLTEPSSGSDAASIRTSAVPSPCGKYYTLNGSKLWISNGG
LADIFTVFAKTPVTDPATGAVKEKITAFVVERGFGGITHGPPEKKMGIKASNTAEVFFDGVR
VPSENVLGEVGSGFKVAMHILNNGRFGMAAALAGTMRGIIAKAVDHATNRTQFGEKIHN
FGLIQEKLARMVMLQYVTESMAYMVSANMDQGATDFQIEAAISKIFGSEAAWKVTDECIQI
MGGMGFMKEPGVERVLRDLRIFRIFEGTNDILRLFVALQGCMDKGKELSGLGSALKNPFG
NAGLLLGEAGKQLRRRAGLGSGLSLSGLVHPELSRSGELAVRALEQFATVVEAKLIKHKKGI
VNEQFLLQRLADGAIDLYAMVVVLSRASRSLSEGHPTAQHEKMLCDTWCIEAAARIREGM
AALQSDPWQQELYRNFKSISKALVERGGVVTSNPLGF
00000000000000000000000000000000000000000000000000000000000000000000000000000000000
00000000000000000000000000000000000000000000000000000000000000000000000000000000000
00000000000000000000000000000000000000000000000000000000000000000000000000000000000
00000000000000000000000000000000000000000000000000000000000000000000000000000000000
00000000000000000000000000000000000000000000000000000000000000000000000000000000000
00111111111111111111111111111111111000000000000000000000000000000000000000000000000
00000000000000000000000000000000000000000000000000000000000000000000000000000000000
000000
>Q3K5N8_4a9x_47_632
ASVSAGNSRLLGFDSIPAATTDTISLPKGYKSSVLISWGQPLHKNGPAFDPSGNGTAAAQEV
QFGDNNDGMSLFEFPGEKNRALMAINNEYTNYRYLYPHGGMPQSAEDVRKALACEGVSV
IEVQRKNGQWQFVQGSRYNRRIHGNSPLRISGPAAGHELMKTSADKHGKKVLGTFQNCA
NGKTPWGTYLTCEENFTDCFGSSNAQQQFDPAQKRYGVSAASREINWHPFDPRFDMAKN
PNELNRHGWVVEIDPFDPQSTPVKRTALGRFKHENAALAETDDGRAVVYMGDDERGEFIY
KFVSRDKINHRNAKANRDILDHGTLYVARFDAGDGNPDHPKGQGQWIELTHGKNGIDAS
SGFADQAEVLIHARLAASVVGATRMDRPEWIVVSPKDGQVYCTLTNNAKRGEDGQPVGG
PNPREKNVYGQILRWRTDRDDHASKTFAWDLFVVAGNPSVHAGTPKGGSSNITPQNMFN
SPDGLGFDKAGRLWILTDGDSSNAGDFAGMGNNQMLCADPATGEIRRFMVGPIGCEVTGI
SFSPDQKTLFVGIQHPGENGGSTFPEHLPNGKPRSSVMAITREDGGIVGA
11111110000000000000000000000000000000000000000000000000000000000000000000000000000
00000000000000000000000000000000000000000000000000000000000000000000000000000000000

00000000000000000000000000000000000000000000000000000000000000000000000000000000
00000000000000000000000000000000000000000000000000000000000000000000000000000000
00000000000000000000000000000000000000000000000000000000000000000000000000000000
00000000000000000000000000000000000000000000000000000000000000000000000000000000
00000000000000000000000000000000000000000000000000000000000000000000000000000000
00000
>Q9WXN8_2o7i_21_605
QVSLPREDTVYIGGALWGPATTWNLYAPQSTWGTDQFMYLPAFQYDLGRDAWIPVIAERY
EFVDDKTLRIYIRPEARWSDGVPITADDFVYALELTKELGIGPGGGWDTYIEYVKAVDTKVV
EFKAKEENLNYFQFLSYSLGAQPMPKHVYERIRAQMNIKDWINDKPEEQVVSGPYKLYYY
DPNIVVYQRVDDWWGKDIFGLPRPKYLAHVIYKDNPSASLAFERGDIDWNGLFIPSVWEL
WEKKGLPVGTWYKKEPYFIPDGVGFVYVNNTKPGLSDPAVRKAIAYAIPYNEMLKKAYFGY
GSQAHPSMVIDLFEPYKQYIDYELAKKTFGTEDGRIPFDLDMANKILDEAGYKKGPDGVRV
GPDGTKLGPYTISVPYGWTDWMMMCEMIAKNLRSIGIDVKTEFPDFSVWADRMTKGTFDL
IISWSVGPSFDHPFNIYRFVLDKRLSKPVGEVTWAGDWERYDNDEVVELLDKAVSTLDPEV
RKQAYFRIQQIIYRDMPSIPAFYTAHWYEYSTKYWINWPSEDNPAWFRPSPWHADAWPTLF
IISKKSDPQPVPSWLGTVDEGGIEIPTAKIFEDLQKATM
11000000000000000000000000000000000000000000000000000000000000000000000000000000
00000000000000000000000000000000000000000000000000000000000000000000000000000000
00000000000000000000000000000000000000000000000000000000000000000000000000000000
00000000000000000000000000000000000000000000000000000000000000000000000000000000
00000000000000000000000000000000000000000000000000000000000000000000000000000000
00000000000000000000000000000000000000000000000000000000000000000000000000000000
00000000000000000000000000000000000000000000000000000000000000000000000000000000
0001
>H8ZRU2_3sqr_0_579
MKYFTVFTALTALFAQASASAIPAVRSTLTPRQNTTASCANSATSRSCWGEYSIDTNWYDVT
PTGVTREYWLSVENSTITPDGYTRSAMTFNGTVPGPAIIADWGDNLIIHVTNNLEHNGTSIH
WHGIRQLGSLEYDGVPGVTQCPIAPGDTLTYKFQVTQYGTTWYHSHFSLQYGDGLFGPLII
NGPATADYDEDVGVIFLQDWAHESVFEIWDTARLGAPPALENTLMNGTNTFDCSASTDPN
CVGGGKKFELTFVEGTKYRLRLINVGIDSHFEFAIDNHTLTVIANDLVPIVPYTTDTLLIGIGQ
RYDVIVEANAAADNYWIRGNWGTTCSTNNEAANATGILRYDSSSIANPTSVGTTPRGTCE
DEPVASLVPHLALDVGGYSLVDEQVSSAFTNYFTWTINSSSLLLDWSSPTTLKIFNNETIFPTE
YNVVALEQTNANEEWVVYVIEDLTGFGIWHPIHLHGHDFFIVAQETDVFNSDESPAKFNLV
NPPRRDVAALPGNGYLAIAFKLDNPGSWLLHCHIAWHASEGLAMQFVESQSSIAVKMTDT
AIFEDTCANWNAYTPTQLFAEDDSGI
11111111111111111111111111111111111100000000000000000000000000000000000000000000
00000000000000000000000000000000000000000000000000000000000000000000000000000000
00000000000000000000000000000000000000000000000000000000000000000000000000000000
00000000000000000000000000000000000000000000000000000000000000000000000000000000
00000000000000000000000000000000000000000000000000000000000000000000000000000000
00000000000000000000000000000011110000000000000000000000000000000000000000000000
0000000000000000000000000000000000000000000000000000000000000000000000000000000
>Q5ZTD3_3kxw_1_580
KKEYLQCQSLVDVVRLRALHSPNKKSCTFLNKELEETMTYEQLDQHAKAIAATLQAEGAK
PGDRVLLLFAPGLPLIQAFLGCLYAGCIAVPIYPPAQEKLLDKAQRIVTNSKPVIVLMIADHIK
KFTADELNTNPKFLKIPAIALESIELNRSSSWQPTSIKSNDIAFLQYTSGSTMHPKGVMVSHH
NLLDNLNKIFTSFHMNDETIIFSWLPPHHDMGLIGCILTPIYGGIQAIMMSPFSFLQNPLSWL
KHITKYKATISGSPNFAYDYCVKRIREEKKEGLDLSSWVTAFNGAEPVREETMEHFYQAFKE
FGFRKEAFYPCYGLAEATLLVTGGTPGSSYKTLTLAKEQFQDHRVHFADDNSPGSYKLVSSG
NPIQEVKIIDPDTLIPCDFDQVGEIWVQSNSVAKGYWNQPEETRHAFAGKIKDDERSAIYLR

TGDLGFLHENELYVTGRIKDLIIIYGKNHYPQDIEFSLMHSPLHHVLGKCAAFVIQEEHEYK
LTVMCEVKNRFMDDVAQDNLFNEIFELVYENHQLEVHTIVLIPLKAMPHTTSGKIRRNFCR
KHLLDKTLPIVATWQLNKIEE
00000000000000000000000000000000000000000000000000000000000000000000000000000000000
00000000000000000000000000000000000000000000011111000000000000000000000000000000000
00000000000000000000000000000000000000000000000000000000000000000000000000000000000
00000000000000000000000000000000000000000000000000000000000000000000000000000000000
00000000000000000000000000000000000000000000000000000000000000000000000000000000000
00000000000000111000000000000000000000000000000000000000000000000000000000000000000
0000000000000000000000000000000000000000000000000000000000000000000000000000000011
>O96759_2uuv_8_586
PKEHIDLYQQIKWNGWGDTRKFLHQLKPSGTIAMTTPEVSSVPLPSLRGFIKKELTLPGEED
KPFVLDETPALQIENIHVDPPKQYPEFVRELKAFFLPDQLKDDKLARITHTFGKSLRDLIRVRI
GQVKNAPDLIVLPHSHEEVERLVQLAHKYNVVIIPMGGGSNIVGAIEPVSNERFTVSIDMRR
MNKVLWVDRREMTACIQVGIMGPELEKQLHKQGVSLGHDPDSFEFSTLGGWLATCSSGH
QSDKYGDIEDMAVSFRTVTPTGTLELRNGARSGAGINYKHIILGSEGTLGIITEAVMKVHAV
PQAVEYYGFLFPTFAHAVSALQQIRSSEVIPTMIRVYDPEETQLSFAWKPSKGAVSEFTSAMV
KKYLHYIRSFDFKNVCLSIIGFEGPKKVVDFHRTSVFDILSKNAAFGLGSAPGKTWAEKRYD
LPYIRDFLLDHNMWVDVAETTVSYANLQTLWKDAKQTFVKHFKDQGIPAWICAHISHTYT
NGVCLYFIFASKQNENKDMAQYIEAKKLMTDIIFKYGGSLSHHHGVGYEHVPWMTRYATR
GWINVYRSLKETIDPKDICNPRKLI
11100000000000000000000000000000000000000000000000000001111111100000000000000000000
00000000000000000000000000000000000000000000000000000000000000000000000000000000000
00000000000000000000000000000000000000000000000000000000000000000000000000000000000
00000000000000000000000001111110000000000000000000000000000000000000000000000000000
00000000000000000000000000000001111100000000000000000000000000000000000000000000000
00000000000000000000000000000000000000000000000000000000000000000000000000000000000
000000000011111111100000000000000111111111111111111111000000000000000000000000111
>A2RJ53_3ryb_23_599
GSNQSSSTSTKKLKAGNFDVAYQNPDKAIKGGNLKVAYQSDSPMKAQWLSGLSNDATFAT
MSGPGGGQDGLFFTDSGFKFIKGGAADVALDKESKTATITLRKDLKWSDGSEVTAKDYEFT
YETIANPAYGSDRWTDSLANIVGLSDYHTGKAKTISGITFPDGENGKVIKVQFKEMKPGMT
QSGNGYFLETVAPYQYLKDVAPKDLASSPKTTTKPLVTGPFKPENVVAGESIKYVPNPYYW
GEKPKLNSITYEVVSTAKSVAALSSSKYDIINGMVSSQYKQVKNLKGYKVLGQQAMYISLM
YYNLGHYDAKNSINVQDRKTPLQDQNVRQAIGYARNVAEVDNKFSNGLSTPANSLIPPIFK
QFTSSSVKGYEKQDLDKANKLLDEDGWKLNKSTGYREKDGKELSLVYAARVGDANAETIA
QNYIQQWKKIGVKVSLYNGKLMEFNSWVDHMTTPPGANDWDITDGSWSLASEPSQQDLF
SAAAPYNFGHFNDSEITKDLNDIDSAKSENPTYRKAAFVKYQEDMNKKAYVIPTNFMLNY
TPVNKRVVGMTLDYGAMNTWSEIGVSSAKLATK
11111111111100000000000000000000000000000000000000000000000000000000000000000000000
00000000000000000000000000000000000000000000000000000000000000000000000000000000000
00000000000000000000000000000000000000000000000000000000000000000000000000000000000
00000000000000000000000000000000000000000000000000000000000000000000000000000000000
00000000000000000000000000000000000000000000000000000000000000000000000000000000000
00000000000000000000000000000000000000000000000000000000000000000000000000000000000
0000000000000000000000000000000000000000000000000000000000000000000000000000111
>P15424_3sqw_87_663
SKLIHVPKEDNSKEVTLDSLLEEGVLDKEIHKAITRMEFPGLTPVQQKTIKPILSSEDHDVIAR
AKTGTGKTFAFLIPIFQHLINTKFDSQYMVKAVIVAPTRDLALQIEAEVKKIHDMNYGLKKY
ACVSLVGGTDFRAAMNKMNKLRPNIVIATPGRLIDVLEKYSNKFFRFVDYKVLDEADRLLE
IGFRDDLETISGILNEKNSKSADNIKTLLFSATLDDKVQKLANNIMNKKECLFLDTVDKNEP

EAHERIDQSVVISEKFANSIFAAVEHIKKQIKERDSNYKAIIFAPTVKFTSFLCSILKNEFKKDL
PILEFHGKITQNKRTSLVKRFKKDESGILVCTDVGARGMDFPNVHEVLQIGVPSELANYIHRI
GRTARSGKEGSSVLFICKDELPFVRELEDAKNIVIAKQEKYEPSEEIKSEVLEAVTEEPEDISDI
VISLISSYRSCIKEYRFSERRILPEIASTYGVLLNDPQLKIPVSRRFLDKLGLSRSPIGKAMFEIRD
YSSRDGNNKSYDYDDDSEISFRGNKNYNNRSQNRDYDDEPFRRSNNNRRSFSRSNDKNNY
SSRNSNIY
00000000000000000000000000000000000000000000000000000000000000000000000000000000000
00000000000000000000000000000000000000000000000000000000000000000000000000000000000
00000000000000000000000000000000000000000000000000000000000000000000000000000000000
00000000000000000000000000000000000000000000000000000000000000000000000000000000000
00000000000000000000000000000000000000000000000000000000000000000000000000000000000
00000000000000000000000000000000000000000000000000000000000000000000000000000000000
0000000000111111111111111111111111111111111111111111111111111111111111111111111
>P48845_1ogm_35_607
GTTNNTHCGADFCTWWHDSGEINTQTPVQPGNVRQSHKYSVQVSLAGTNNFHDSFVYES
IPRNGNGRIYAPTDPPNSNTLDSSVDDGISIEPSIGLNMAWSQFEYSHDVDVKILATDGSSLG
SPSDVVIRPVSISYAISQSDDGGIVIRVPADANGRKFSVEFKTDLYTFLSDGNEYVTSGGSVVG
VEPTNALVIFASPFLPSGMIPHMTPDNTQTMTPGPINNGDWGAKSILYFPPGVYWMNQDQ
SGNSGKLGSNHIRLNSNTYWVYLAPGAYVKGAIEYFTKQNFYATGHGILSGENYVYQANA
GDNYIAVKSDSTSLRMWWHNNLGGGQTWYCVGPTINAPPFNTMDFNGNSGISSQISDYK
QVGAFFFQTDGPEIYPNSVVHDVFWHVNDDAIKIYYSGASVSRATIWKCHNDPIIQMGWT
SRDISGVTIDTLNVIHTRYIKSETVVPSAIIGASPFYASGMSPDSRKSISMTVSNVVCEGLCPSL
FRITPLQNYKNFVVKNVAFPDGLQTNSIGTGESIIPAASGLTMGLNISNWTVGGQKVTMEN
FQANSLGQFNIDGSYWGEWQIS
10000000000000000000000000000000000000000000000000000000000000000000000000000000000
00000000000000000000000000000000000000000000000000000000000000000000000000000000000
00000000000000000000000000000000000000000000000000000000000000000000000000000000000
00000000000000000000000000000000000000000000000000000000000000000000000000000000000
00000000000000000000000000000000000000000000000000000000000000000000000000000000000
00000000000000000000000000000000000000000000000000000000000000000000000000000000000
000000000000000000000000000000000000000000000000000000000000000000000000000
>P06149_1f0x_0_570
MSSMTTTDNKAFLNELARLVGSSHLLTDPAKTARYRKGFRSGQGDALAVVFPGSLLELWRV
LKACVTADKIILMQAANTGLTEGSTPNGNDYDRDVVIISTLRLDKLHVLGKGEQVLAYPGT
TLYSLEKALKPLGREPHSVIGSSCIGASVIGGICNNSGGSLVQRGPAYTEMSLFARINEDGKLT
LVNHLGIDLGETPEQILSKLDDDRIKDDDVRHDGRHAHDYDYVHRVRDIEADTPARYNAD
PDRLFESSGCAGKLAVFAVRLDTFEAEKNQQVFYIGTNQPEVLTEIRRHILANFENLPVAGE
YMHRDIYDIAEKYGKDTFLMIDKLGTDKMPFFFNLKGRTDAMLEKVKFFRPHFTDRAMQK
FGHLFPSHLPPRMKNWRDKYEHHLLLKMAGDGVGEAKSWLVDYFKQAEGDFFVCTPEEG
SKAFLHRFAAAGAAIRYQAVHSDEVEDILALDIALRRNDTEWYEHLPPEIDSQLVHKLYYG
HFMCYVFHQDYIVKKGVDVHALKEQMLELLQQRGAQYPAEHNVGHLYKAPETLQKFYR
ENDPTNSMNPGIGKTSKRKNWQEVE
11111111000000000000000000000000000000000000000000000000000000000000000000000000000
00000000000000000000000000000000000000000000000000000000000000000000000000000000000
00000000000000000000000000000000000000000000000000000000000000000000000000000000000
00000000000000000000000000000000000000000000000000000000000000000000001111111111111
11111111111111111111111111111111111111111111000000000000000000000000000000000000000
00000000000000000000000000000000000000000000000000000000000000000000000000000000000
0000000000000000000000000000000000000000000000000000000000000000000001111
>Q99IB8_4aep_2442_3012
SMSYSWTGALITPCSPEEEKLPINPLSNSLLRYHNKVYCTTSKSASQRAKKVTFDRTQVLDA

HYDSVLKDIKLAASKVSARLLTLEEACQLTPPHSARSKYGFGAKEVRSLSGRAVNHIKSVW
KDLLEDPQTPIPTTIMAKNEVFCVDPAKGGKKPARLIVYPDLGVRVCEKMALYDITQKLPQ
AVMGASYGFQYSPAQRVEYLLKAWAEKKDPMGFSYDTRCFDSTVTERDIRTEESIYQACSLP
EEARTAIHSLTERLYVGGPMFNSKGQTCGYRRCRASGVLTTSMGNTITCYVKALAACKAAG
IVAPTMLVCGDDLVVISESQGTEEDERNLRAFTEAMTRYSAPPGDPPRPEYDLELITSCSSNV
SVALGPRGRRRYYLTRDPTTPLARAAWETVRHSPINSWLGNIIQYAPTIWVRMVLMTHFFSI
LMVQDTLDQNLNFEMYGSVYSVNPLDLPAIIERLHGLDAFSMHTYSHHELTRVASALRKLG
APPLRVWKSRARAVRASLISRGGKAAVCGRYLFNWAVKTKLKLTPLPEARLLDLSSWFTVG
AGGGDIFHSVSRARPRS
00000000000000000000000000000000000000000000000000000000000000000000000000000000000
00000000000000000000000000000000000000000000000000000000000000000000000000000000000
00000000000000000000000000000000000000000000000000000000000000000000000000000000000
00000000000000000000000000000000000000000000000000000000000000000000000000000000000
00000000000000000000000000000000000000000000000000000000000000000000000000000000000
00000000000000000000000000000000000000000000000000000000000000000000000000000000000
0000000000000000000000000000000000000000000000000000000000000000011111111
>P17846_4g38_0_569
MSEKHPGPLVVEGKLTDAERMKHESNYLRGTIAEDLNDGLTGGFKGDNFLLIRFHGMYQQ
DDRDIRAERAEQKLEPRHAMLLRCRLPGGVITTKQWQAIDKFAGENTIYGSIRLTNRQTFQ
FHGILKKNVKPVHQMLHSVGLDALATANDMNRNVLCTSNPYESQLHAEAYEWAKKISEH
LLPRTRAYAEIWLDQEKVATTDEEPILGQTYLPRKFKTTVVIPPQNDIDLHANDMNFVAIAE
NGKLVGFNLLVGGGLSIEHGNKKTYARTASEFGYLPLEHTLAVAEAVVTTQRDWGNRTDR
KNAKTKYTLERVGVETFKAEVERRAGIKFEPIRPYEFTGRGDRIGWVKGIDDNWHLTLFIEN
GRILDYPARPLKTGLLEIAKIHKGDFRITANQNLIIAGVPESEKAKIEKIAKESGLMNAVTPQ
RENSMACVSFPTCPLAMAEAERFLPSFIDNIDNLMAKHGVSDEHIVMRVTGCPNGCGRA
MLAEVGLVGKAPGRYNLHLGGNRIGTRIPRMYKENITEPEILASLDELIGRWAKEREAGEGF
GDFTVRAGIIRPVLDPARDLWD
11111111111111111111111111111111111111111111111111111111111111111111111111111111000
00000000000000000000000000000000000000000001111100000000000000000000000000000000000
00000000000000000111111111111111111111111111111100000000000000000000000000000000000
00000000000000000000000000000000000000000000000001111100000000000000000000000000000
00000000000000000000000000000000000000000000000000000000000000000000000000000000000
00000000000000000000000000000000000000000000000000000000000000000000000000000000000
000000000000000000000000000000000000000000000000000000000000000000000000
>P13699_3mx5_0_568
MSASKEIKSFLWTQSLRRELSGYCSNIKLQVVKDAQALLHGLDFSEVSNVQRLMRKERRDD
NDLKRLRDLNQAVNNLVELKSTQQKSILRVGTLTSDDLLILAADLEKLKSKVIRTERPLSAG
VYMGNLSSQQLDQRRALLNMIGMSGGNQGARAGRDGVVRVWDVKNAELLNNQFGTMP
SLTLACLTKQGQVDLNDAVQALTDLGLIYTAKYPNTSDLDRLTQSHPILNMIDTKKSSLNIS
GYNFSLGAAVKAGACMLDGGNMLETIKVSPQTMDGILKSILKVKKALGMFISDTPGERNP
YENILYKICLSGDGWPYIASRTSITGRAWENTVVDLESDGKPQKADSNNSSKSLQSAGFTAG
LTYSQLMTLKDAMLQLDPNAKTWMDIEGRPEDPVEIALYQPSSGCYIHFFREPTDLKQFKQ
DAKYSHGIDVTDLFATQPGLTSAVIDALPRNMVITCQGSDDIRKLLESQGRKDIKLIDIALSK
TDSRKYENAVWDQYKDLCHMHTGVVVEKKKRGGKEEITPHCALMDCIMFDAAVSGGLN
TSVLRAVLPRDMVFRTSTPRVVL
11111110000000000000000000000000000000000000000000000000000000000000000000000000000
00000000000000000000000000000000000000000000000000000000000000011111111111000000000
00000000000000000000000000000000000000000000000000000000000000000000000000000000000
00000000000000000000000000000000000000000000000000000000000000000000000000000000000
00000011111111111111111111111110000000000000000000000000000000000000000000000000000
00000000000000000000000000000000000000000000000000000000000000000000000000000000000

00000000000000000111100000000000000000000000000000000000000011111111
>Q76KB0_3vkt_18_586
FSKNAVKLHATPPSVAAPPTGAPEVAAERLEPRVEEKDGYWILKEQFRKGINPQEKVKIEKE
PMKLFMENGIEELAKIPIEEIDQSKLTKDDIDVRLKWLGLFHRRKNQYGRFMMRLKLPNGV
TTSAQTRYLASVIRKYGKEGCADITTRQNWQIRGVVLPDVPEILKGLAEVGLTSLQSGMDN
VRNPVGNPLAGIDPEEIVDTRPYTNLLSQFITGNSRGNPAVSNLPRKWNPCVVGSHDLYEH
PHINDLAYMPATKDGRFGFNLLVGGFFSAKRCDEAIPLDAWVPADDVVPVCKAILEAFRDL
GFRGNRQKCRMMWLIDELGVEGFRAEVEKRMPQQQLERASPEDLVQKQWERRDYLGVH
PQKQEGYSFIGLHIPVGRVQADDMDELARLADEYGSGEIRLTVEQNIIIPNIETSKIEALLKEP
VLSTFSPDPPILMKGLVACTGNQFCGQAIIETKARSLKITEEVQRQVSLTKPVRMHWTGCPN
TCAQVQVADIGFMGCLTRDKNGKTVEGADVFLGGRIGSDSHLGEVYKKAVPCDDLVPLVV
DLLVNNFGAVPREREETED
11111111111111111111111000000000000000000000000000000000000000000000000000000000
00000000000000000000000000000000000000000000000000000000000000000000000000000000
00000000000000000000000000000000000000000000000000000000000000000000000000000000
00000000000000000000000000000000000000000000000000000000000000000000000000000000
00000000000000000000000000000000000000000000000000000000000000000000000000000000
00000000000000000000000000000000000000000000000000000000000000000000000000000000
00000000000000000000000000000000000000000000000000000000000001111111
>B5CY96_4aw7_29_597
VDYNTRRFLSGVSELDRSKYFNIHSTSDDDKDVGKFLADYQVGLGRKFWGPYSYAYNKTH
EVGKYPQMKPYSGNISVKRYIATEHPYVQHIQGGIDVQAAGAWSAEYYSNSELVPEFFEPL
NEPFVHANDAGFTVQGQAMRELMVDFYASIGKHIHNNPRLNGKMKVIGYAAAYPAWED
GNFNYWNTRMKMFIDRAGAYMDGFSVHLYDGIVTGTDTKRSGSNSEAVLDMVEAYSYIKF
GHVKPLAISEFGGIDNSKPDDSYDDISSVRSVSSFNHFLFNLMERQDNLFISIPFVSDKAEWH
ITAANNYTSYSAALFIPDNPQNLKNTTWRLNDKKYFFELWKNVKGERVDITSSNPDIQVQA
FKDGGRLYIALDNLDDNPQTVYLNNKNSWKDVSNVTKRSLYVNYNAGIEYTEQNVPSMP
ESISIVPNQTIVLVADVSSSAFTNSIIRNKYYSSEYLKPISAGSSLSFPFTGIESGSGRASLRMSIGR
PVSASKKPVVKINGTAVSVPDNWKGYGQSNRNIFFGMIEVPFDIQLLKNGDNNVDITFSDG
GGHVSSMILQVEKYTVS
00000000000000000000000000000000000000000000000000000000000000000000000000000000
00000000000000000000000000000000000000000000000000000000000000000000000000000000
00000000000000000000000000000000000000000001111100000000000000000000000000000000
00000010000000000000000000000000000000000000000000000000000000000000000000000000
00000000000000000000000000000000000000000000000000000000000000000000000000000000
00000000000000000000000001000000000000000000000000000000000000000000000000000000
0000000000000000000000000000000000000000000000000000000000000000000
>D1GLU5_4gr5_0_566
MSNPFEEYDGGHVVLTDALGRHSLWPAGIAVPAGWSVRHGTDSREGCLAHIEHHWTDLR
PTGPAVERAPAGACVHELFEAQAARAPDAVALLHEADELTYGALNERANRLAHRLVGLG
VAPGTLVGVHLERGFDMVVALLAVLKAGGGYTMLDPQFPVERLALSLEDTGAPLLVTSRPL
SGRLTGTTTLYVEDEAASDAPAGNLATGVGPEDVACVMFTSGSTGRPKGVMSPHRALTGT
YLGQDYAGFGPDEVFLQCSPVSWDAFGLELFGALLFGARCVLQSGQNPDPLEIGELVARHG
VTMLQLSASLFNFLVDEVPEAFEGVRYAITGGEPASVPHVAKARRDHPALRLGNGYGPAES
MGFTTHHAVVAGDLSGTALPIGVPLAGKRAYVLDDDLKPAANGALGELYVAGAGLAHGY
VSRPALTAERFVADPFAGPGGERMYRTGDLARRRADGVLEYVGRADDQVKIRGFRVEPGE
VEARLVGHPAVRQAAVLAQDSRLGDKQLVAYVVAERADAPPDAAELRRHVAEALPAYMV
PVECVPVDELPRTPNGKLDRRALTGSGS
10000000000000000000000000000000000000000000000000000000000001110000000000000000
00000000000000000000000000000000000000000000000000000000000000000000000000000000
00000000000000000000000000111110000000000000000000000000000000000000000000000000

00000000000000000000000000000000000000000000000000000000000000000000000000000000
00000000000000000000000000000000000000000000000000000000000000000000000000000000
00000000000000000000000000000000000000000000000111111111111111111111111111111111
111111111111111111111111111111111111111111111111111111111111111111
>Q41290_1v02_0_564
MALLLASAINHTAHPAGLRSHPNNESFSRHHLCSSPQNISKRRSNLSFRPRAQTISSESAGIH
RLSPWEIPRRDWFPPSFLFGAATSAYQIEGAWNEDGKGPSTWDHFCHNFPEWIVDRSNGD
VAADSYHMYAEDVRLLKEMGMDAYRFSISWPRILPKGTLAGGINEKGVEYYNKLIDLLLEN
GIEPYITIFHWDTPQALVDAYGGFLDEEDYKDYTDFAKVCFEKFGKTVKNWLTFNEPETFCS
VSYGTGVLAPGRCSPGVSCAVPTGNSLSEPYIVAHNLLRAHAETVDIYNKYHKGADGRIGL
ALNVFGRVPYTNTFLDQQAQERSMDKCLGWFLEPVVRGDYPFSMRVSARDRVPYFKEKEQ
EKLVGSYDMIGINYYTSTFSKHIDLSPNNSPVLNTDDAYASQETKGPDGNAIGPPTGNAWIN
MYPKGLHDILMTMKNKYGNPPMYITENGMGDIDKGDLPKPVALEDHTRLDYIQRHLSVL
KQSIDLGADVRGYFAWSLLDNFEWSSGYTERFGIVYVDRENGCERTMKRSARWLQEFNGA
AKKVENNKILTPAGQLN
11111111111111111111111111111111111111111111111111111111111100000000000000000000
00000000000000000000000000000000000000000000000000000000000000000000000000000000
00000000000000000000000000000000000000000000000000000000000000000000000000000000
00000000000000000000000000000000000000000000000000000000000000000000000000000000
00000000000000000000000000000000000000000000000000000000000000000000000000000000
00000000000000000000000000000000000000000000000000000000000000000000000000000000
0000000000000000000000000000000000000000000000011111111111111111
>Q74K42_2yfr_144_707
DVKQVEKKDSVDKTNAEENKDSSVKPAENATKAELKGQVKDIVEESGVDTSKLTNDQINE
LNKINFSKEAKSGTQLTYNDFKKIAKTLIEQDARYAIPFFNASKIKNMPAAKTLDAQSGKVE
DLEIWDSWPVQDAKTGYVSNWNGYQLVIGMMGVPNVNDNHIYLLYNKYGDNDFNHWK
NAGPIFGLGTPVIQQWSGSATLNKDGSIQLYYTKVDTSDNNTNHQKLASATVYLNLEKDQ
DKISIAHVDNDHIVFEGDGYHYQTYDQWKETNKGADNIAMRDAHVIDDDNGNRYLVFE
ASTGTENYQGDDQIYQWLNYGGTNKDNLGDFFQILSNSDIKDRAKWSNAAIGIIKLNDDV
KNPSVAKVYSPLISAPMVSDEIERPDVVKLGNKYYLFAATRLNRGSNDDAWMATNKAVGD
NVAMIGYVSDNLTHGYVPLNESGVVLTASVPANWRTATYSYYAVPVEGRDDQLLITSYITNR
GEVAGKGMHATWAPSFLLQINPDNTTTVLAKMTNQGDWIWDDSSENPDMMGVLEKDAP
NSAALPGEWGKPVDWDLIGGYNLKPHQ
11111111111111111111111111111100000000000000000000000000000000000000000000000000
00000000000000000000000000000000000000000000000000000000000000000000000000000000
00000000000000000000000000000000000000000000000000000000000000000000000000000000
00000000000000000000000000000000000000000000000000000000000000000000000000000000
00000000000000000000000000000000000000000000000000000000000000000000000000000000
00000000000000000000000000000000000000000000000000000000000000000000000000000000
0000000000000000000000000000000000000000000000000000000000000000
>Q8KRB4_2wok_0_561
MTTAARRPAPTTAGAGWDAGVGALVNPSRRRGGTLRLVSSADVDSLDPARTYYVWVWLL
QRLLNRTLMAYPTDPGPAGLVPAPDLAEGPGEVSDGGRTWTYRLRRGLRYDDGTPITSDDV
RHAVQRVFAQDVLPGGPTYLIPLLDDPERPYPGPYRTDEPLRSVLTPDEHTIVFRLTRPFSDF
DHLMAQPCAAPVPRRSDTGADYGRDPRSSGPYRVARHEPDTLLHLERNPHWDRATDPIRP
ALPDRVELTIGLDVDVLDARLIAGEFDINLEGRGLQHAAQRRATADEVLRSHTDNPRTSFL
HFVAMQPHIPPFDNVHVRRAVQYAADKILLQDARGGPVNGGDLTTALFPPTLPAHQDLDL
YPTGPDLRGDLDAARAELAAAGLPDGFRAVIGTQRGKFRLVADAVVESLARVGIELTVKEL
DVATYFSLGAGHPETVREHGLGLLVTDWGADFPTEYGFLAPLVDGRQIKRNGGNWNLPEL
DDPEVNALIDETLHTTDPAARAELWRAVERRVMEHAVLLPLVHDKTLHFRNPWVTNVYV
HPAFGLYDIQAMGLAEED

11111110000000000000000000000000000000000000000000000000000000000000000000000000000
00000000000000000000000000000000000000000000000000000000000000000000000000000000000
00000000000000000000000000000000000000000000000000000000000000000000000000000000000
00000000000000000000000000000000000000000000000000000000000000000000000000000000000
00000000000000000000000000000000000000000000000000000000000000000000000000000000000
00000000000000000000000000000000000000000000000000000000000000000000000000000000000
000000000000000000000000000000000000000000000000000000000000011
>Q2T061_4f4h_0_560
MKTRIALAQLNVTVGDFAGNVAKIVAAAQAAHDAGAHFLIAPELALSGYPPEDLLLRPAF
YAASDAALAELAAQLKPFAGLAVLVGHPLRAPSADGNANRAIERGVPPVDTYNAASLIVG
GEVAGTYRKQDLPNTEVFDEKRYFATDAAPYVFELNGVKFGVVICEDVWHASAAQLAKA
AGAQVLIVPNGSPYHMNKDAVRIDILRARIRETGLPMVYVNLVGGQDELVFDGGSFVLDG
AGELVAKMPQFEEGNAIVEFDGARALPAAIAPALSVEAQVYRALVLGVRDYIGKNGFPGAII
GLSGGVDSALVLAVAVDALGAERVRAVMMPSRYTAGISTTDAADMARRVGVRYDEIAIAP
MFDAFRASLAAEFAGLAEDATEENIQARIRGTLLMALSNKFGSIVLTTGNKSEMAVGYCTL
YGDMAGGFAVIKDIAKTLVYRLCRYRNAAAEYGQPDIVPERILTRAPSAELRENQTDQDSLP
PYDVLDAIMRMYMEEDRPLAEIVAAGYSEADVKRVTRLIKINEYKRRQAPVGIRVTHRAFG
RDWRYPITSRFVESID
00000000000000000000000000000000000000000000000000000000000000000000000000000000000
00000000011111000000000000000000000000000000000000000000000000000000000000000000000
00000000000000000000000000000000000000000000000000000000000000000000000000000000000
00000000000000000000000000000000000000000000000000000000000000000000000000000000000
00000000000000000000000000000000000000000000000000000000000000000000000000000000000
00000000000000000000000000000000000000000000000000001111111111111110000000000000000
00000000000000000000000000000000000000000000000000000000000000
>Q5HCV6_3iii_0_559
MNQHLLGNPKLTVTHVNEVKAGINHIVVDSVQYGNQEMIMEKDGTVEMRDGEKLYINIF
RPNKDGKFPVVMSADTYGKDNKPKITNMGALWPTLGTIPTSSFTPEESPDPGFWVPNDYV
VVKVALRGSDKSKGVLSPWSKREAEDYYEVIEWAANQSWSNGNIGTNGVSYLAVTQWWV
ASLNPPHLKAMIPWEGLNDMYREVAFHGGIPDTGFYRFWTQGIFARWTDNPNIEDLIQAQ
QEHPLFDDFWKQRQVPLSQIKTPLLTCASWSTQGLHNRGSFEGFKQAASEEKWLYVHGRK
EWESYYARENLERQKSFFDFYLKEENNDWKDTPHVIYEVRDQFYKGEFKSASAFPLPNAEY
TPLYLNAENHTLNHAKISSAHVAQYDSEDKQQDVSFKYTFDKDTELVGNMNLKLWVSTK
DSDDMDLFAGIKKLDRRGNEVNFPDFNHIENGQVATGWLRVSHRELDQEKSSIAQPWHK
HETELKLSQDEIVPVEIELLPSGTLFKQGETLEVVVKGSEIVIGNSTPGMKTRYEHEETVNKG
MHMIYTGGKYDSQLIIPIVN
11000000000000000000000000000000000000000000000000000000000000000000000000000000000
11000000000000000000000000000000000000000000000000000000000000000000000000000000000
00000000000000000000000000000000000000000000000000000000000000000000000000000000000
00000000000000000000000000000000000000000000000000000000000000000000000000000000000
00000000000000000000000000000000000000000000000000000000000000000000000000000000000
00000000000000000000000000000000000000000000000000000000000000000000000000000000000
0000000000000000000000000000000000000000000000000000000000000
>A1R4M8_3iuk_0_558
MTTANTPVRPKSAIDAVADAYTEKLIELNPSFATTLGLPGHETEYQDYSPAGAAAHAEATRL
ALEALAGLEPSDDVDAVTLDAMRERLGLELEIHQSGWDAADLNNIASPAQDIRAIFDLMP
TDTVEHWEHIAGRAANVPGAIEGYIASLRAAKDDRKVAAARQIRIVIEQTGRYAAEDGFFA
KMAADASLGDAPLPAEVQDKLDAGTSAARSAYSALGAFLRDELLPVAPEKDAVGRERYSL
ASRSFIGAEVDLEETYAWGVQELERLISEQEKVAGQIKPGASIEEAKSILNNDPARQIKGTDA
LKAWMQELSDRAVSELADVHFDIPDVMKTLECMIAPTDEGGIYYTGPSDDFSRPGRMWWS
VPAGEDTFTTWSETTTVFHEGVPGHHLQVATATYRRELLNNWRRNVCWVSGHGEGWALY

AEQLMLELGYLKDPGDHMGMLDGQRMRAARVVFDIGVHLELPVPERWGTGTWTPEKGF
DFLKANLDISEGQLQFEFTRYLGWPGQAPSYKVGQRLWEQIRAELESREGFDLKSFHSKAL
NIGSVGLDVLRRALL
11111110000000000000000000000000000000000000000000000000000000000000000000000000000
00000000000000000000000000000000000000000000000000000000000000000000000000000000000
00000000000000000000000000000000000000000000000000000000000000000000000000000000000
00000000000000000000000000000000000000000000000000000000000000000000000000000000000
00000000000000000000000000000000000000000000000000000000000000000000000000000000000
00000000000000000000000000000000000000000000000000000000000000000000000000000000000
000000000000000000000000000000000000000000000000000000000000
>A1YYW7_3q3q_0_558
MLKHVAAALLLATAMPVVAQSPAPAAAPAPAARSIAATPPKLIVAISVDQFSADLFSEYRQY
YTGGLKRLTSEGAVFPRGYQSHAATETCPGHSTILTGSRPSRTGIIANNWFDLDAKREDKNL
YCAEDESQPGSSSDKYEASPLHLKVPTLGGRMKAANPATRVVSVAGKDRAAIMMGGATA
DQVWWLGGPQGYVSYKGVAPTPLVTQVNQAFAQRLAQPNPGFELPAQCVSKDFPVQAG
NRTVGTGRFARDAGDYKGFRISPEQDAMTLAFAAAAIENMQLGKQAQTDIISIGLSATDYV
GHTFGTEGTESCIQVDRLDTELGAFFDKLDKDGIDYVVVLTADHGGHDLPERHRMNAMP
MEQRVDMALTPKALNATIAEKAGLPGKKVIWSDGPSGDIYYDKGLTAAQRARVETEALKY
LRAHPQVQTVFTKAEIAATPSPSGPPESWSLIQEARASFYPSRSGDLLLLLKPRVMSIPEQAV
MGSVATHGSPWDTDRRVPILFWRKGMQHFEQPLGVETVDILPSLAALIKLPVPKDQIDGRC
LDLVAGKDDSCAGQ
11111111111111111111111111111100000000000000000000000000000000000000000000000000000
00000000000000000000000000000000000000000000000000000000000000000000000000000000000
00000000000000000000000000000000000000000000000000000000000000000000000000000000000
00000000000000000000000000000000000000000000000000000000000000000000000000000000000
00000000000000000000000000000000000000000000000000000000000000000000000000000000000
00000000000000000000000000000000000000000000000000000000000000000000000000000000000
000000000000000000000000000000000000000000000000000000000111
>P25080_1uwk_0_556
MTDNNKYRDVEIRAPRGNKLTAKSWLTEAPLRMLMNNLDPQVAENPKELVVYGGIGRAA
RNWECYDKIVETLTRLEDDETLLVQSGKPVGVFKTHSNAPRVLIANSNLVPHWANWEHFN
ELDAKGLAMYGQMTAGSWIYIGSQGIVQGTYETFVEAGRQHYGGTVKAKWVLTAGLGG
MGGAQPLAATLAGACSLNIECQQSRIDFRLETRYVDEQATDLDDALVRIAKYTAEGKAISIA
LHGNAAEILPELVKRGVRPDMVTDQTSAHDPLNGYLPAGWTWEQYRDRAQTEPAAVVK
AAKQSMAVHVQAMLDFQKQGVPTFDYGNNIRQMAKEEGVANAFDFPGFVPAYIRPLFCR
GVGPFRWAALSGEAEDIYKTDAKVKELIPDDAHLHRWLDMARERISFQGLPARICWVGLG
LRAKLGLAFNEMVRSGELSAPVVIGRDHLDSGSVSSPNRETEAMRDGSDAVSDWPLLNAL
LNTAGGATWVSLHHGGGVGMGFSQHSGMVIVCDGTDEAAERIARVLTNDPGTGVMRHA
DAGYDIAIDCAKEQGLDLPMITG
11100000000000000000000000000000000000000000000000000000000000000000000000000000000
00000000000000000000000000000000000000000000000000000000000000000000000000000000000
00000000000000000000000000000000000000000000000000000000000000000000000000000000000
00000000000000000000000000000000000000000000000000000000000000000000000000000000000
00000000000000000000000000000000000000000000000000000000000000000000000000000000000
00000000000000000000000000000000000000000000000000000000000000000000000000000000000
0000000000000000000000000000000000000000000000000000000000
>Q13WK7_2w3p_0_555
MSTAETAVAPVDYRTDPSQYKHWKLSFNGPVATLGIDIAEDGGIRDGYKLKLNSYDLGVDI
ELHDAIQRIRFEHPEVRTVVLTSLKDRVFCSGANIFMLGLSTHAWKVNFCKFTNETRNGLE
DSSRHSGLKFLAAVNGACAGGGYELALACDEIYLVDDRSSSVSLPEVPLLGVLPGTGGLTRV
TDKRKVRHDRADIFCTVVEGVRGERAKAWRLVDEVVKPNQFDQAIQARALELAAQSDRP

AHAQGVPLTRIERTDREDGLTYKTLDVTIDRAKRIATFTAKAPQTEPPASIDAIVAAGANW
WPLKFAREFDDAILSMRTNELAVGTWVFRTEGDARHLLAADASLMQHKDHWFVRETIGL
LRRTLARIDVSSRSLFALIEPGSCFAGTFAELAFAADRTYMAALPANEDEEPAITLSEVNFGLY
PMVTHQSRLARRFYEETEPLDAVRSRIGQAIKPVEAERLGLVTASPDDIDWADEIRIALEERA
AMSPDALTGLEANLRFNGPETMETRIFGRLTAWQNWIFNRPNAVGEKGALKVYGKGSKA
QFDVSRV
11111111000000000000000000000000000000000000000000000000000000000000000000000000000
00000000000000000000000000000000000000000000000000000000000000000000000000000000000
00000000000000000000000000000000000000000000000000000000000000000000000000000000000
00000000000000000000000000000000000000000000000000000000000000000000000000000000000
00000000000000000000000000000000000000000000000000000000000000000000000000000000000
00000000000000000000000000000000000000000000000000000000000000000000000000000000000
0000000000000000000000000000000000000000000000000000000000
>P34913_3i28_0_554
MTLRAAVFDLDGVLALPAVFGVLGRTEEALALPRGLLNDAFQKGGPEGATTRLMKGEITLS
QWIPLMEENCRKCSETAKVCLPKNFSIKEIFDKAISARKINRPMLQAALMLRKKGFTTAILT
NTWLDDRAERDGLAQLMCELKMHFDFLIESCQVGMVKPEPQIYKFLLDTLKASPSEVVFL
DDIGANLKPARDLGMVTILVQDTDTALKELEKVTGIQLLNTPAPLPTSCNPSDMSHGYVTV
KPRVRLHFVELGSGPAVCLCHGFPESWYSWRYQIPALAQAGYRVLAMDMKGYGESSAPPEI
EEYCMEVLCKEMVTFLDKLGLSQAVFIGHDWGGMLVWYMALFYPERVRAVASLNTPFIPA
NPNMSPLESIKANPVFDYQLYFQEPGVAEAELEQNLSRTFKSLFRASDESVLSMHKVCEAG
GLFVNSPEEPSLSRMVTEEEIQFYVQQFKKSGFRGPLNWYRNMERNWKWACKSLGRKILIP
ALMVTAEKDFVLVPQMSQHMEDWIPHLKRGHIEDCGHWTQMDKPTEVNQILIKWLDSD
ARNPPVVSKM
11100000000000000000000000000000000000000000000000000000000000000000000000000000000
00000000000000000000000000000000000000000000000000000000000000000000000000000000000
00000000000000000000000000000000000000000000000000000000000000000000000000000000000
00000000000000000000000000000000000000000000000000000000000000000000000000000000000
00000000000000000000000000000000000000000000000000000000000000000000000000000000000
00000000000000000000000000000000000000000000000000000000000000000000000000000000000
00000000000000000000000000000000000000000000000001111111
>Q06135_2w61_0_554
MNKKQNFYAAIIVAIFLCLQLSHGSSGVSFEKTPAIKIVGNKFFDSESGEQFFIKGIAYQLQRS
EEELSNANGAFETSYIDALADPKICLRDIPFLKMLGVNTLRVYAIDPTKSHDICMEALSAEG
MYVLLDLSEPDISINRENPSWDVHIFERYKSVIDAMSSFPNLLGYFAGNEVTNDHTNTFASP
FVKAAIRDAKEYISHSNHRKIPVGYSTNDDAMTRDNLARYFVCGDVKADFYGINMYEWC
GYSTYGTSGYRERTKEFEGYPIPVFFSEFGCNLVRPRPFTEVSALYGNKMSSVWSGGLAYMYF
EEENEYGVVKINDNDGVDILPDFKNLKKEFAKADPKGITEEEYLTAKEPTEVESVECPHIAV
GVWEANEKLPETPDRSKCACLDEILPCEIVPFGAESGKYEEYFSYLCSKVDCSDILANGKTG
EYGEFSDCSVEQKLSLQLSKLYCKIGANDRHCPLNDKNVYFNLESLQPLTSESICKNVFDSIR
NITYNHGDYSKSNPSRSKESLNVKYPSSEERENDGTIAFKTSGFVILLISMIAAGILL
11111111111111111111111111000000000000000000000000000000000000001111110000000000000
00000000000000000000000000000000000000000000000000000000000000000000000000000000000
00000000000000000000000000000000000000000000000000000000000000000000000000000000000
00000000000000000000000000000000000000000000000000000000000000000000000000000000000
00000000000000000000000000011110000000000000000000000000000000000000000000000000000
00000000000000000000000000000000000000000000000000000000000000000000000000000000000
00000000111111111111111111111111111111111111111111111111
>P0C061_1amu_2_555
NSSKSILIHAQNKNGTHEEEQYLFAVNNTKAEYPRDKTIHQLFEEQVSKRPNNVAIVCENE
QLTYHELNVKANQLARIFIEKGIGKDTLVGIMMEKSIDLFIGILAVLKAGGAYVPIDIEYPKER

IQYILDDSQARMLLTQKHLVHLIHNIQFNGQVEIFEEDTIKIREGTNLHVPSKSTDLAYVIYTS
GTTGNPKGTMLEHKGISNLKVFFENSLNVTEKDRIGQFASISFDASVWEMFMALLTGASLYI
ILKDTINDFVKFEQYINQKEITVITLPPTYVVHLDPERILSIQTLITAGSATSPSLVNKWKEKVT
YINAYGPTETTICATTWVATKETIGHSVPIGAPIQNTQIYIVDENLQLKSVGEAGELCIGGEGL
ARGYWKRPELTSQKFVDNPFVPGEKLYKTGDQARWLSDGNIEYLGRIDNQVKIRGHRVEL
EEVESILLKHMYISETAVSVHKDHQEQPYLCAYFVSEKHIPLEQLRQFSSEELPTYMIPSYFIQL
DKMPLTSNGKIDRKQLPEPDLTFGMRVDYEAPRNEIEETLVTIWQDVLG
11111111111111000000000000000000000000000000000000000000000000000000000000000000000
00000000000000000000000000000000000000000000000000000000000000000000000000000000000
00000000000000000000000111100000000000000000000000000000000000000000000000000000000
00000000000000000000000000000000000000000000000000000000000000000000000000000000000
00000000000000000000000000000000000000000000000000000000000000000000000000000000000
00000000000000000000000000000000000000000000000000000000000000000000000000000000000
00000000000000000000000000000011111111111111111111111111
>Q00955_1w96_12_565
KMEYEITNYSERHTELPGHFIGLNTVDKLEESPLRDFVKSHGGHTVISKILIANNGIAAVKEI
RSVRKWAYETFGDDRTVQFVAMATPEDLEANAEYIRMADQYIEVPGGTNNNNYANVDLI
VDIAERADVDAVWAGWGHASENPLLPEKLSQSKRKVIFIGPPGNAMRSLGDKISSTIVAQS
AKVPCIPWSGTGVDTVHVDEKTGLVSVDDDIYQKGCCTSPEDGLQKAKRIGFPVMIKASEG
GGGKGIRQVEREEDFIALYHQAANEIPGSPIFIMKLAGRARHLEVQLLADQYGTNISLFGRD
CSVQRRHQKIIEEAPVTIAKAETFHEMEKAAVRLGKLVGYVSAGTVEYLYSHDDGKFYFLEL
NPRLQVEHPTTEMVSGVNLPAAQLQIAMGIPMHRISDIRTLYGMNPHSASEIDFEFKTQDA
TKKQRRPIPKGHCTACRITSEDPNDGFKPSGGTLHELNFRSSSNVWGYFSVGNNGNIHSFSD
SQFGHIFAFGENRQASRKHMVVALKELSIRGDFRTTVEYLIKLLETEDFEDNTITTGWLDDLI
10000000000000000000000000000000000000000000000000000000000000000000000000000000000
00000000000000000000000000000000000000000000000000000000000000000000000000000000000
00000000000000000000000000000000000000000000000000000000000000000000000000000000000
00000000000000000000000000000000000000000000000000000000000000000000000000000000000
00000000000000000000000000000000000000000000000000000000000000000000000000000000000
00000000000000000000000000000000000000000000000000000000000000000000000000000000000
00000000000000000000000000000000000000000000000000000000
>Q59470_1egm_0_553
MRSKRFEALAKRPVNQDGFVKEWIEEGFIAMESPNDPKPSIKIVNGAVTELDGKPVSDFDLI
DHFIARYGINLNRAEEVMAMDSVKLANMLCDPNVKRSEIVPLTTAMTPAKIVEVVSHMNV
VEMMMAMQKMRARRTPSQQAHVTNVKDNPVQIAADAAEGAWRGFDEQETTVAVARYA
PFNAIALLVGSQVGRPGVLTQCSLEEATELKLGMLGHTCYAETISVYGTEPVFTDGDDTPWS
KGFLASSYASRGLKMRFTSGSGSEVQMGYAEGKSMLYLEARCIYITKAAGVQGLQNGSVSCI
GVPSAVPSGIRAVLAENLICSSLDLECASSNDQTFTHSDMRRTARLLMQFLPGTDFISSGYSA
VPNYDNMFAGSNEDAEDFDDYNVIQRDLKVDGGLRPVREEDVIAIRNKAARALQAVFAG
MGLPPITDEEVEAATYAHGSKDMPERNIVEDIKFAQEIINKNRNGLEVVKALAQGGFTDVA
QDMLNIQKAKLTGDYLHTSAIIVGDGQVLSAVNDVNDYAGPATGYRLQGERWEEIKNIPG
ALDPNEID
00000000000000000000000000000000000000000000000000000000000000000000000000000000000
00000000000000000000000000000000000000000000000000000000000000000000000000000000000
00000000000000000000000000000000000000000000000000000000000000000000000000000000000
00000000000000000000000000000000000000000000000000000000000000000000000000000000000
00000000000000000000000000000000000000000000000000000000000000000000000000000000000
00000000000000000000000000000000000000000000000000000000000000000000000000000000000
00000000000000000000000000000000000000000000000000000111
>O26157_3aii_0_552
MVPVEDLVYRYALLNAVKHRGRANPGAVMGAVMSNEPELRKMAPQVKEAVEAAVERVN

SLSPEEQQQEMERLGLEITERKQKKRKGLRELAGVKGEVVLRFAPNPSGPLHIGHARAAIL
NHEYARKYDGRLILRIEDTDPRRVDPEAYDMIPADLEWLGVEWDETVIQSDRMETYYEYTE
KLIERGGAYVCTCRPEEFRELKNRGEACHCRSLGFRENLQRWREMFEMKEGSAVVRVKTD
LNHPNPAIRDWVSMRIVEAEHPRTGTRYRVYPMMNFSVAVDDHLLGVTHVLRGKDHLAN
REKQEYLYRHLGWEPPEFIHYGRLKMDDVALSTSGAREGILRGEYSGWDDPRLGTLRAIAR
RGIRPEAIRKLMVEIGVKIADSTMSWKKIYGLNRSILEEEARRYFFAADPVKLEVVGLPGPVR
VERPLHPDHPEIGNRVLELRGEVYLPGDDLGEGPLRLIDAVNVIYSGGELRYHSEGIEEAREL
GASMIHWVPAESALEAEVIMPDASRVRGVIEADASELEVDDVVQLERFGFARLDSAGPGM
VFYYAHK
11111111111111111111111111111111111111111111111111111111111111111111111111111111111
11100000000000000000000000000000000000000000000000000000000000000000000000000000000
00000000000000000000000000000000000000000000000000000000000000000000000000000000000
00000000000000000000000000000000000000000001111100000000000000000000000000011111110
00000000000000000000000000000000000000000000000000000000000000000000000000000000000
00000000000000000000000000000000000000000000000000000000000000000000000000000000000
0000000000000000000000000000000000000000000000000000000
>Q8RR56_1t1e_0_551
MSDMEKPWKEEEKREVLAGHARRQAPQAVDKGPVTGDQRISVTVVLRRQRGDELEAHVE
RQAALAPHARVHLEREAFAASHGASLDDFAEIRKFAEAHGLTLDRAHVAAGTAVLSGPVD
AVNQAFGVELRHFDHPDGSYRSYVGDVRVPASIAPLIEAVFGLDTRPVARPHFRLRRRAEGE
FEARSQSAAPTAYTPLDVAQAYQFPEGLDGQGQCIAIIELGGGYDETSLAQYFASLGVSAPQ
VVSVSVDGATNQPTGDPNGPDGEVELDIEVAGALAPGAKIAVYFAPNTDAGFLNAITTAV
HDPTHKPSIVSISWGGPEDSWAPASIAAMNRAFLDAAALGVTVLAAAGDSGSTDGEQDGL
YHVDFPAASPYVLACGGTRLVASAGRIERETVWNDGPDGGSTGGGVSRIFPLPSWQERAN
VPPSANPGAGSGRGVPDVAGNADPATGYEVVIDGETTVIGGTSAVAPLFAALVARINQKLG
KPVGYLNPTLYQLPPEVFHDITEGNNDIANRARIYQAGPGWDPCTGLGSPIGIRLLQALLPS
ASQAQP
11111111111000000000000000000000000000000000000000000000000000000000000000000000000
00000000000000000000000000000000000000000000000000000000000000000000000000000000000
00000000000000000000000000000000000000000000000000000000000000000000000000000000000
00000000000000000000000000000000000000000000000000000000000000000000000000000000000
00000000000000000000000000000000000000000000000000000000000000000000000000000000000
00000000000000000000000000000000000000000000000000000000000000000000000000000000000
000000000000000000000000000000000000000000000001111111
>P00959_1f4l_0_550
MTQVAKKILVTCALPYANGSIHLGHMLEHIQADVWVRYQRMRGHEVNFICADDAHGTPI
MLKAQQLGITPEQMIGEMSQEHQTDFAGFNISYDNYHSTHSEENRQLSELIYSRLKENGFIK
NRTISQLYDPEKGMFLPDRFVKGTCPKCKSPDQYGDNCEVCGATYSPTELIEPKSVVSGATP
VMRDSEHFFFDLPSFSEMLQAWTRSGALQEQVANKMQEWFESGLQQWDISRDAPYFGFEI
PNAPGKYFYVWLDAPIGYMGSFKNLCDKRGDSVSFDEYWKKDSTAELYHFIGKDIVYFHSL
FWPAMLEGSNFRKPSNLFVHGYVTVNGAKMSKSRGTFIKASTWLNHFDADSLRYYYTAKL
SSRIDDIDLNLEDFVQRVNADIVNKVVNLASRNAGFINKRFDGVLASELADPQLYKTFTDA
AEVIGEAWESREFGKAVREIMALADLANRYVDEQAPWVVAKQEGRDADLQAICSMGINLF
RVLMTYLKPVLPKLTERAEAFLNTELTWDGIQQPLLGHKVNPFKALYNRIDMRQVEALVE
ASKEEV
11110000000000000000000000000000000000000000000000000000000000000000000000000000000
00000000000000000000000000000000000000000000000000000000000000000000000000000000000
00000000000000000000000000000000000000000000000000000000000000000000000000000000000
00000000000000000000000000000000000000000000000000000000000000000000000000000000000
00000000000000000000000000000000000000000000000000000000000000000000000000000000000
00000000000000000000000000000000000000000000000000000000000000000000000000000000000

00000000000000000000000000000000000000000000000000011
>Q72IT0_2wdd_23_572
ALEDPRSLYDLPPYGDATLLYFSDLHGQAFPHYFMEPPNLIAPKPLMGRPGYLTGEAILRYY
GVERGTPLAYLLSYVDFVELARTFGPIGGMGALTALIRDQKARVEAEGGKALVLDGGDTW
TNSGLSLLTRGEAVVRWQNLVGVDHMVSHWEWTLGRERVEELLGLFRGEFLSYNIVDDLF
GDPLFPAYRIHRVGPYALAVVGASYPYVKVSHPESFTEGLSFALDERRLQEAVDKARAEGAN
AVVLLSHNGMQLDAALAERIRGIDLILSGHTHDLTPRPWRVGKTWIVAGSAAGKALMRVD
LKLWKGGIANLRVRVLPVLAEHLPKAEDVEAFLKAQLAPHQDHLFTPLAVSETLLYKRDTL
YSTWDQLVGEAVKAIYPEVEVVFSPAVRWGTTILPGQAITWDHLYAYTGFTYPELYLFYLRG
AQIKAVLEDIASNVFTSDPFYQQGGDVSRVFGLRYVLDPDAPTGERVREVEVGGRPLDPNR
RYLAAAYGGRLQRVGEAKPGYEPRPIYEVLAEYLRSVGRVRVRPEPNVKVIGRNYRLPEVTG
11111110000000000000000000000000000000000000000000000000000000000000000000000000000
00000000000000000000000000000000000000000000000000000000000000000000000000000000000
00000000000000000000000000000000000000000000000000000000000000000000000000000000000
00000000000000000000000000000000000000000000000000000000000000000000000000000000000
00000000000000000000000000000000000000000000000000000000000000000000000000000000000
00000000000000000000000000000000000000000000000000000000000000000000000000000000000
0000000000000000000000000000000000000000000000000000
>B8H1H2_3u7v_0_548
MEEAMGMSRFATAVGLALALVCGPLASGAHAADAAMPQLVTKDGRHALMVDGAPFLM
LAAQVNNSSAWPSQMAKVWPAIEKVGANTVQVPIAWEQIEPVEGQFDFSYLDLLLEQARE
RKVRLVLLWFGTWKNSSPSYAPEWVKLDDKRFPRLIKDDGERSYSMSPLAKSTLDADRKAF
VALMTHLKAKDAAQKTVIMVQVENETGTYGSVRDFGPAAQKVFNGPAPATLVKAVGAKP
GTWSQAFGKDADEFFHAWHIGRFVDQVAAGGKAVYPLPMYVNAALRDPIKPGDPKTYSA
GGPTDNVIGVWQAAAPSIDLIAPDIYMKESVKYEAVLGHYARPNNALFVAETGNDPAYAR
YVFSTLGKQGIGWSPFGMDYTGYGNYPLGAAKVDEALIEVFAANYRLLAPMARELARLSF
EGKVWGGAEPDDRAAQTTTMGDWTVSLRYGKWQFGASDWTWLKDRPPGPQGPEGGGL
VAQLSPNEFLVTGRNVRVEFGRATADGKPMMMARVEEGHFENGQWVFDRLWNGDQTDY
GLNFTGGSQILRVKLATY
11111111111111111111111111111111000000000000000000000000000000000000000000000000000
00000000000000000000000000000000000000000000000000000000000000000000000000000000000
00000000000000000000000000000000000000000000000000000000000000000000000000000000000
00000000000000000000000000000000000000000000000000000000000000000000000000000000000
00000000000000000000000000000000000000000000000000000000000000000000000000000000000
00000000000000000000000000000000000000000000000000000000000000000000000000000000000
000000000000000000000000000000000000000000000000000
>Q7KZF4_3bdl_339_887
DKQFVAKVMQVLNADAIVVKLNSGDYKTIHLSSIRPPRLEGENTQDKNKKLRPLYDIPYMF
EAREFLRKKLIGKKVNVTVDYIRPASPATETVPAFSERTCATVTIGGINIAEALVSKGLATVIR
YRQDDDQRSSHYDELLAAEARAIKNGKGLHSKKEVPIHRVADISGDTQKAKQFLPFLQRA
GRSEAVVEYVFSGSRLKLYLPKETCLITFLLAGIECPRGARNLPGLVQEGEPFSEEATLFTKEL
VLQREVEVEVESMDKAGNFIGWLHIDGANLSVLLVEHALSKVHFTAERSSYYKSLLSAEEA
AKQKKEKVWAHYEEQPVEEVMPVLEEKERSASYKPVFVTEITDDLHFYVQDVETGTQLEKL
MENMRNDIASHPPVEGSYAPRRGEFCIAKFVDGEWYRARVEKVESPAKIHVFYIDYGNREV
LPSTRLGTLSPAFSTRVLPAQATEYAFAFIQVPQDDDARTDAVDSVVRDIQNTQCLLNVEHL
SAGCPHVTLQFADSKGDVGLGLVKEGLVMVEVRKEKQFQKVITEYLNAQESAKSA
00000000000000000000000000000000000000000000000000000000000000000000000000000000000
00000000000000000000000000000000000000000000000000000000000000000000000000000000000
00000000000000000000000000000000000000000000000000000000000000000000000000000000000
00000000000000000000000000000000000000000000000000000000000000000000000111111111100
00000000000000000000000000000000000000000000000000000000000000000000000000000000000

00000000000000000000000000000000000000000000000000000000000000000000000000000000
000000000000000000000000000000000000000000111
>Q8DQ62_2bib_25_571
QESSGNKIHFINVQEGGSDAIILESNGHFAMVDTGEDYDFPDGSDSRYPWREGIETSYKHVL
TDRVFRRLKELSVQKLDFILVTHTHSDHIGNVDELLSTYPVDRVYLKKYSDSRITNSERLWD
NLYGYDKVLQTATETGVSVIQNITQGDAHFQFGDMDIQLYNYENETDSSGELKKIWDDNS
NSLISVVKVNGKKIYLGGDLDNVHGAEDKYGPLIGKVDLMKFNHHHDTNKSNTKDFIKN
LSPSLIVQTSDSLPWKNGVDSEYVNWLKERGIERINAASKDYDATVFDIRKDGFVNISTSYKP
IPSFQAGWHKSAYGNWWYQAPDSTGEYAVGWNEIEGEWYYFNQTGILLQNQWKKWNN
HWFYLTDSGASAKNWKKIDGIWYYFNKENQMEIGWVQDKEQWYYLDVDGSMKTGWLQ
YMGQWYYFAPSGEMKMGWVKDKETWYYMDSTGVMKTGEIEVAGQHYYLEDSGAMKQG
WHKKANDWYFYKTDGSRAVGWIKDKDKWYFLKENGQLLVNGKTPEGYTVDSSGAWLVD
VSIEKSATIKTT
00000000000000000000000000000000000000000000000000000000000000000000000000000000
00000000000000000000000000000000000000000000000000000000000000000000000000000000
00000000000000000000000000000000000000000000000000000000000000000000000000000000
00000000000000000000000000000000000000000000000000000000000000000000000000000000
00000000000000000000000000000000000000000000000000000000000000000000000000000000
00000000000000000000000000000000000000000000000000000000000000000000000000000000
0000000000000000000000000000000000000111111
>E3PQS3_3ugf_109_654
FPDNAVPYPWSNAQLSWQRTAFHFQPERSWMSDPDGPIFYKGWYHFFYQYNPDNPVWG
NNTWGHTVSRDLIHWLYLPLALAADQWYDMQGVFSGSATCLPDGRIMMLYTGVTKEMV
EMLSLAYPADLSDPLLVEWVKYPGNPILSAPPGVSPTEFRDASTGWYVSNGTWRIAIGAKY
NTTGIAMVYETKDFKSFKLLEELLHAVPDTGLWECVDLYPVSTTGEKGLETSVNGPKVKHV
LKASIDEQQRDYYAIGTYDLGTNKWTPDNPEEDVGIGLRYDWGKYYASKTFYDPKKQRRV
VWAWTKELDSEVADREKGWANVQTIPRTVLLDQKTGTNVLLWPVEEVESLRLSSKEFSKVK
AGAGSVVPLDVGTATQLDIIAEFEIDKEALEGTIEADMGYNCTTSGGAAERGVLGPFGLLVS
ATENLSEQTPVYFYIAKGTDGNFKTFFCLDESRSSKASDVSKQVKGFTVPVLDGEKFTMRLL
VDHSIVESFAQGGRSCITSRVYPTEAIYGAAKLFLFNNATGASITASLKIWEMNSAFIQPFHR
11111000000000000000000000000000000000000000000000000000000000000000000000000000
00000000000000000000000000000000000000000000000000000000000000000000000000000000
00000000000000000000000000000000000000000000000000000000000000000000000000000000
00000000000000000000000000000000000000000000000000000000000000000000000000000000
00000000000000000000000000000000000000000000000000000111111111000000000000000000
00000000000000000000000000000000000000000000000000000000000000000000000000000000
0000000000000000000000000000000000000000000001
>Q5A5Q7_3c9f_14_559
ASFPHRNLTWNDINFVHTTDTHGWYSGHINQPLYHANWGDFISFTTHLRRIAHSRNQDLL
LIDSGDRHDGNGLSDITSPNGLKSTPIFIKQDYDLLTIGNHELYLWENSKQEYETVVNHFQD
KYVCSNVDIRLDNGSFVPLGSKYKYFTTPIRGIRVLAFGFLFDFKRFNSGTRVTPMAETIHEP
WFQEALKHEVDLIIIVGHTPISHNWGEFYQVHQYLRQFFPDTIIQYFGGHSHIRDFTVFDSLS
TGLQSGRYCETVGWTSVNLDKADLNLPVRQRFSRSYIDFNTDSFKYHTNLDKEFDTAKGKS
VSKLIRETRKELKLDTLIGYVKTNYYVDYVPIDHPKSIFNLLASKILKTLPKSKHEERITIINTG
SIRYDLYKGPYTIDSKFIVSPFENIWVNITVPKSVATKVAAKLNDADYISASRLKPPHQYDLQ
VQDLSTSPHQAHFEMQEKLPKGYVTHDDFGADGDDTLHRAVVNFPVPNVIQSVEINDEV
DSPVNLVFYSFITPNIIWALKELNFSTEQVPTPYSDIYLGTLLNEFVANN
10000000000000000000000000000000000000000000000000000000000000000000000000000000
00000000000000000000000000000000000000000000000000000000000000000000000000000000
00000000000000000000000000000000000000000000000000000000000000000000000000000000
00000000000000000000000000000000000000000000000000000000000000000000000000000000

00000000000000000000000000000000000000000000000000000000000000000000000000000000000
00001100000000000000000001000000000000000000000000000000000000000000000000000000000
000000000000000000000000000000000000000000000000
>Q51504_3oc2_34_578
DLHVIDHDFLKGQGDARSVRHIAIPAHRGLITDRNGEPLAVSTPVTTLWANPKELMTAKER
WPQLAAALGQDTKLFADRIEQNAEREFIYLVRGLTPEQGEGVIALKVPGVYSIEEFRRFYPA
GEVVAHAVGFTDVDDRGREGIELAFDEWLAGVPGKRQVLKDRRGRVIKDVQVTKNAKPG
KTLALSIDLRLQYLAHRELRNALLENGAKAGSLVIMDVKTGEILAMTNQPTYNPNNRRNL
QPAAMRNRAMIDVFEPGSTVKPFSMSAALASGRWKPSDIVDVYPGTLQIGRYTIRDVSRNS
RQLDLTGILIKSSNVGISKIAFDIGAESIYSVMQQVGLGQDTGLGFPGERVGNLPNHRKWPK
AETATLAYGYGLSVTAIQLAHAYAALANDGKSVPLSMTRVDRVPDGVQVISPEVASTVQGM
LQQVVEAQGGVFRAQVPGYHAAGKSGTARKVSVGTKGYRENAYRSLFAGFAPATDPRIAM
VVVIDEPSKAGYFGGLVSAPVFSKVMAGALRLMNVPPDNLPTATEQQQVNAAPAKGGRG
11111111111111100000000000000000000000000000000000000000000000000000000000000000000
00000000000000000000000000000000000000000000000000000000000000000000000000000000000
00000000000000000000000000000000000000000000000000000000000000000000000000000000000
00000000000000000000000000000000000000000000000000000000000000000000000000000000000
00000000000000000000000000000000000000000000000000000000000000000000000000000000000
00000000000000000000000000000000000000000111111111110000000000000000000000000011111
00000000000000000000000000001111111111111111111
>Q51643_4faj_0_544
MKKYKKFCFLGIGLLPLVLASCGTNTATKDSQDATEKKVEQVATLTAGTPVQSLDPATAVD
QTSITLLANVMEGLYRLDEKNQPQPAIAAGQPKVSNNGKTYTIVIRDGAKWSDGTQITASD
FVAAWQRVVDPKTVSPNVELFSAIKNAKEIASGKQAKDTLAVKSIGEKTLEIELVEPTPYFTD
LLSLTAYYPVQQKAIKEYGKDYGVSQKAIVTNGAFNLTNLEGVGTSDKWTISKNKEYWDQ
KDVSMDKINFQVVKEINTGINLYNDGQLDEAPLAGEYAKQYKKDKEYSTTLMANTMFLE
MNQTGENKLLQNKNVRKAINYAIDRESLVKKLLDNGSVASVGVVPKEMAFNPVNKKDFA
NEKLVEFNKKQAEEYWDKAKKEIDLSKNTSLDLLVSDGEFEKKAGEFLQGQLQDSLEGLKV
TVTPIPANVFMERLTKKDFTLSLSGWQADYADPISFLANFETNSPMNHGGYSNKNYDELLK
DSSSKRWQELKKAEKLLINDMGVVPIFQVGTAKLEKSKIKNVLMHSIGAKYDYKKMRIEK
11111111111111111111111111111111111111000000000000000000000000000000000000000000000
00000000000000000000000000000000000000000000000000000000000000000000000000000000000
00000000000000000000000000000000000000000000000000000000000000000000000000000000000
00000000000000000000000000000000000000000000000000000000000000000000000000000000000
00000000000000000000000000000000000000000000000000000000000000000000000000000000000
00000000000000000000000000000000000000000000000000000000000000000000000000000000000
00000000000000000000000000000000000000000000000
>G8GV21_3riq_130_672
AVFRSEADKKFKYSVKLSDYMTLQEAATAAVDSLLIDIDYNFIDGEAVDFGGKVLTIECKAK
FIGDGVLNWNNLGSGSKVISPHMHTKTTPYTVYRFDDNGNWVTNPTTVLASVAQRLDKG
YKPNVNDHDIWASLPDNVKNQVAGATLRVNSANNIIFTHPEATMGGYLFTLCNHILVESPR
NFIAWESGITFENHHTTAWGTGNKVVGGEIKYGSGSAVLFIRNDGGDDHDGGVRDLISYRV
GESGVKTYQNEIGGRSARNYRLVFDNITTIQCYYDGIDVNADTGSPTERVDDYTLAEYPWF
QLPTQHIIRNIITRDCMGIGAWWDGQKNIIDNVVTYEAHKEGMFDRGTNNDITNITVVCA
NKDLTNLNQIVCEGGSRLRGIMVHAYTTQGYAVYAPSSEVSNVSCAGSGTKKILCTYVADIQ
GGNINVQHGENAMTLSMRPAMGGTINPSLVLTADCQVASPGNEASIVKLSAIQDGARVGE
LQLNRLGFKHMSIPVAESQLPESALEFNSSIGFFFGTDDELRILAKKPDGTFVTYSL
11000000000000000000000000000000000000000000000000000000000000000000000000000000000
00000000000000000000000000000000000000000000000000000000000000000000000000000000000
00000000000000000000000000000000000000000000000000000000000000000000000000000000000
00000000000000000000000000000000000000000000000000000000000000000000000000000000000

00000000000000000000000000000000000000000000000000000000000000000000000000000000000
00000000000000000000000000000000000000000000000000000000000000000000000000000000000
0000000000000000000000000000000000000000000
>B2HVG8_3o83_0_541
MKKQLIEFVRWSPERAQHYRNKGYWIDQPLTRILTVGVQSHPHSPAIICGERQLSYIELDRLS
TNLASRLAEKGLGKGDTALVQLPNVAEFYIVFFALLKAGVVVLNALYSHRQYELNAFIKQI
QPKLLIGSRQHEVFSNNQFIDSLHEVNLSPEIILMLNHQATDFGLLDWIETPAETLVDFSSTP
ADEVAFFQLSGGSTGTPKLIPRTHNDYDYSVRASAEICDLNSNTRLLCALPAPHNFMLSSPG
ALGVLHAGGCVVMAPNPEPLNCFSIIQRHQVNMASLVPSAVIMWLEKAAQYKDQIQSLKL
LQVGGASFPESLARQVPEVINCKLQQVFGMAEGLVNYTRLDDSDEQIFTTQGRPISSDDEIKI
VDEQYKEVPEGEIGMLATRGPYTFCGYYQSPEHNSQVFDEDNYYYSGDLVQRTPDGNLRV
VGRIKDQINRGGEKIASEEIEKLILLHPEVMHAALVAIVDEQFGEKSCAFIVSRNPELKAVVL
RRHLMELGIAQYKLPDQIKLIESLPLTAVGKVDKKQLRSILNTSTTS
11000000000000000000000000000000000000000000000000000000000000000000000000000000000
00000000000000000000000000000000000000000000000000000000000000000000000000000000000
00000000000000000000000000000000000000000000000000000000000000000000000000000000000
00000000000000000000000000000000000000000000000000000000000000000000000000000000000
00000000000000000000000000000000000000000000000000000000000000000000000000000000000
00000000000000000000000111111111111111111111111111111111111111111111111111111111111
1111111111111111111111111111111111111111111
>Q47PU3_1w4x_0_541
MAGQTTVDSRRQPPEEVDVLVVGAGFSGLYALYRLRELGRSVHVIETAGDVGGVWYWNRY
PGARCDIESIEYCYSFSEEVLQEWNWTERYASQPEILRYINFVADKFDLRSGITFHTTVTAAAF
DEATNTWTVDTNHGDRIRARYLIMASGQLSVPQLPNFPGLKDFAGNLYHTGNWPHEPVD
FSGQRVGVIGTGSSGIQVSPQIAKQAAELFVFQRTPHFAVPARNAPLDPEFLADLKKRYAEF
REESRNTPGGTHRYQGPKSALEVSDEELVETLERYWQEGGPDILAAYRDILRDRDANERVA
EFIRNKIRNTVRDPEVAERLVPKGYPFGTKRLILEIDYYEMFNRDNVHLVDTLSAPIETITPRG
VRTSEREYELDSLVLATGFDALTGALFKIDIRGVGNVALKEKWAAGPRTYLGLSTAGFPNLF
FIAGPGSPSALSNMLVSIEQHVEWVTDHIAYMFKNGLTRSEAVLEKEDEWVEHVNEIADET
LYPMTASWYTGANVPGKPRVFMLYVGGFHRYRQICDEVAAKGYEGFVLT
11111111100000000000000000000000000000000000000000000000000000000000000000000000000
00000000000000000000000000000000000000000000000000000000000000000000000000000000000
00000000000000000000000000000000000000000000000000000000000000000000000000000000000
00000000000000000000000000000000000000000000000000000000000000000000000000000000000
00000000000000000000000000000000000000000000000000000000000000000000000000000000000
00000000000000000000000000000000000000000000000000000000000000000000000000000000000
0000000000000000000000000000000000000000000
>P30613_4ima_33_573
GPAGYLRRASVAQLTQELGTAFFQQQQLPAAMADTFLEHLCLLDIDSEPVAARSTSIIATIGP
ASRSVERLKEMIKAGMNIARLNFSHGSHEYHAESIANVREAVESFAGSPLSYRPVAIALDTK
GPEIRTGILQGGPESEVELVKGSQVLVTVDPAFRTRGNANTVWVDYPNIVRVVPVGGRIYID
DGLISLVVQKIGPEGLVTQVENGGVLGSRKGVNLPGAQVDLPGLSEQDVRDLRFGVEHGV
DIVFASFVRKASDVAAVRAALGPEGHGIKIISKIENHEGVKRFDEILEVSDGIMVARGDLGIEI
PAEKVFLAQKMMIGRCNLAGKPVVCATQMLESMITKPRPTRAETSDVANAVLDGADCIML
SGETAKGNFPVEAVKMQHAIAREAEAAVYHRQLFEELRRAAPLSRDPTEVTAIGAVEAAFK
CCAAAIIVLTTTGRSAQLLSRYRPRAAVIAVTRSAQAARQVHLCRGVFPLLYREPPEAIWAD
DVDRRVQFGIESGKLRGFLRVGDLVIVVTGWRPGSGYTNIMRVLSIS
11111111111110000000000000000000000000000000000000000000000000000000000000000000000
00000000000000000000000000000000000000000000000011111111000000000000000000001100000
00000000000000000000000000000000000000000000000000000000000000000000000000000000000
00000000000000000000000000000000000000000000000000000000000000000000000000000000000

00000000000000000000000000000000000000000000000000000000000000000000000000000000000
00000000000000000000000000000000000000000000000000000000000000000000000000000000000
0000000000000000000000000000000000000000000
>O75191_4bc2_0_535
MAEHAPRRCCLGWDFSTQQVKVVAVDAELNVFYEESVHFDRDLPEFGTQGGVHVHKDG
LTVTSPVLMWVQALDIILEKMKASGFDFSQVLALSGAGQQHGSIYWKAGAQQALTSLSPD
LRLHQQLQDCFSISDCPVWMDSSTTAQCRQLEAAVGGAQALSCLTGSRAYERFTGNQIAKI
YQQNPEAYSHTERISLVSSFAASLFLGSYSPIDYSDGSGMNLLQIQDKVWSQACLGACAPHL
EEKLSPPVPSCSVVGAISSYYVQRYGFPPGCKVVAFTGDNPASLAGMRLEEGDIAVSLGTSDT
LFLWLQEPMPALEGHIFCNPVDSQHYMALLCFKNGSLMREKIRNESVSRSWSDFSKALQST
EMGNGGNLGFYFDVMEITPEIIGRHRFNTENHKVAAFPGDVEVRALIEGQFMAKRIHAEG
LGYRVMSKTKILATGGASHNREILQVLADVFDAPVYVIDTANSACVGSAYRAFHGLAGGT
DVPFSEVVKLAPNPRLAATPSPGASQVYEALLPQYAKLEQRILSQTRGPPE
11111000000000000000000000000000000000000000000000000000000000000000000000000000000
00000000000000000000000000000000000000000000000000000000000000000000000000000000000
00000000000000000000000000000000000000000000000000000000000000000000000000000000000
00000000000000000000000000000000000000000000000000000000000000000000000000000000000
00000000000000000000000000000000000000000000000000000000000000000000000000000000000
00000000000000000000000000000000000000000000000000000000000000000000000000000000000
00000000000000000000000000000000001111
>P51691_1hdh_0_535
MSKRPNFLVIVADDLGFSDIGAFGGEIATPNLDALAIAGLRLTDFHTASTCSPTRSMLLTGTD
HHIAGIGTMAEALTPELEGKPGYEGHLNERVVALPELLREAGYQTLMAGKWHLGLKPEQT
PHARGFERSFSLLPGAANHYGFEPPYDESTPRILKGTPALYVEDERYLDTLPEGFYSSDAFGD
KLLQYLKERDQSRPFFAYLPFSAPHWPLQAPREIVEKYRGRYDAGPEALRQERLARLKELGL
VEADVEAHPVLALTREWEALEDEERAKSARAMEVYAAMVERMDWNIGRVVDYLRRQGE
LDNTFVLFMSDNGAEGALLEAFPKFGPDLLGFLDRHYDNSLENIGRANSYVWYGPRWAQ
AATAPSRLYKAFTTQGGIRVPALVRYPRLSRQGAISHAFATVMDVTPTLLDLAGVRHPGKR
WRGREIAEPRGRSWLGWLSGETEAAHDENTVTGWELFGMRAIRQGDWKAVYLPAPVGPA
TWQLYDLARDPGEIHDLADSQPGKLAELIEHWKRYVSETGVVEGASPFLVR
11000000000000000000000000000000000000000000000000000000000000000000000000000000000
00000000000000000000000000000000000000000000000000000000000000000000000000000000000
00000000000000000000000000000000000000000000000000000000000000000000000000000000000
00000000000000000000000000000000000000000000000000000000000000000000000000000000000
00000000000000000000000000000000000000000000000000000000000000000000000000000000000
00000000000000000000000000000000000000000000000000000000000000000000000000000000000
00000000000000000000000000000111111111
>Q945K2_1ju2_27_562
LATTSDHDFSYLSFAYDATDLELEGSYDYVIVGGGTSGCPLAATLSEKYKVLVLERGSLPTAY
PNVLTADGFVYNLQQEDDGKTPVERFVSEDGIDNVRGRVLGGTSIINAGVYARANTSIYSAS
GVDWDMDLVNQTYEWVEDTIVYKPNSQSWQSVTKTAFLEAGVHPNHGFSLDHEEGTRIT
GSTFDNKGTRHAADELLNKGNSNNLRVGVHASVEKIIFSNAPGLTATGVIYRDSNGTPHQ
AFVRSKGEVIVSAGTIGTPQLLLLSGVGPESYLSSLNIPVVLSHPYVGQFLHDNPRNFINILPP
NPIEPTIVTVLGISNDFYQCSFSSLPFTTPPFGFFPSASYPLPNSTFAHFASKVAGPLSYGSLTLK
SSSNVRVSPNVKFNYYSNLTDLSHCVSGMKKIGELLSTDALKPYKVEDLPGVEGFNILGIPLP
KDQTDDAAFETFCRESVASYWHYHGGCLVGKVLDGDFRVTGINALRVVDGSTFPYTPASH
PQGFYLMLGRYVGIKILQERSASDLKILDSLKSAASLVL
00000000000000000000000000000000000000000000000000000000000000000000000000000000000
00000000000000000000000000000000000000000000000000000000000000000000000000000000000
00000000000000000000000000000000000000000000000000000000000000000000000000000000000
00000000000000000000000000000000000000000000000000000000000000000000000000000000000

000000000000000000000000000000000000000000000000000000000000000000000000000000000000000000000000
000000000000000000000000000000000000000000000000000000000000000000000000000000000000000000000000
00000000000000000000000111111111111111
>P13482_2jg0_30_564
EETPVTPQPPDILLGPLFNDVQNAKLFPDQKTFADAVPNSDPLMILADYRMQQNQSGFDL
RHFVNVNFTLPKEGEKYVPPEGQSLREHIDGLWPVLTRSTENTEKWDSLLPLPEPYVVPGG
RFREVYYWDSYFTMLGLAESGHWDKVADMVANFAHEIDTYGHIPNGNRSYYLSRSQPPFF
ALMVELLAQHEGDAALKQYLPQMQKEYAYWMDGVENLQAGQQEKRVVKLQDGTLLNR
YWDDRDTPRPESWVEDIATAKSNPNRPATEIYRDLRSAAASGWDFSSRWMDNPQQLNTLR
TTSIVPVDLNSLMFKMEKILARASKAAGDNAMANQYETLANARQKGIEKYLWNDQQGW
YADYDLKSHKVRNQLTAAALFPLYVNAAAKDRANKMATATKTHLLQPGGLNTTSVKSGQ
QWDAPNGWAPLQWVATEGLQNYGQKEVAMDISWHFLTNVQHTYDREKKLVEKYDVSTT
GTGGGGGEYPLQDGFGWTNGVTLKMLDLICPKEQPCDNVPATRPTVKSATTQPSTKEAQP
TP
111111000000000000000000000000000000000000000000000000000000000000000000000000000000011110000000
000000000000000000000000000000000000000000000000000000000000000000000000000000000000000000000000
000000000000000000000000000000000000000000000000000000000000000000000000000000000000000000000000
000000000000000000000000000000000000000000000000000000000000000000000000000000000000000000000000
000000000000000000000000000000000000000000000000000000000000000000000000000000000000000000000000
000000000000000000000000000000000000000000000000000000000000000000000000000000000000000000000000
000000000000000000001111111111111111111
>P16098_2xg9_0_534
MEVNVKGNYVQVYVMLPLDAVSVNNRFEKGDELRAQLRKLVEAGVDGVMVDVWWGLV
EGKGPKAYDWSAYKQLFELVQKAGLKLQAIMSFHQCGGNVGDAVNIPIPQWVRDVGTRD
PDIFYTDGHGTRNIEYLTLGVDNQPLFHGRSAVQMYADYMTSFRENMKDFLDAGVIVDIEV
GLGPAGEMRYPSYPQSHGWSFPGIGEFICYDKYLQADFKAAAAAVGHPEWEFPNDVGQY
NDTPERTQFFRDNGTYLSEKGRFFLAWYSNNLIKHGDRILDEANKVFLGYKVQLAIKISGIH
WWYKVPSHAAELTAGYYNLHDRDGYRTIARMLKRHRASINFTCAEMRDLEQSSQAMSAP
EELVQQVLSAGWREGLNVACENALPRYDPTAYNTILRNARPHGINQSGPPEHKLFGFTYLR
LSNQLVEGQNYVNFKTFVDRMHANLPRDPYVDPMAPLPRSGPEISIEMILQAAQPKLQPFP
FQEHTDLPVGPTGGMGGQAEGPTCGMGGQVKGPTGGMGGQAEDPTSGIGGELPATM
110000000000000000000000000000000000000000000000000000000000000000000000000000000000000000000000
000000000000000000000000000000000000000000000000000000000000000000000000000000000000000000000000
000000000000000000000000000000000000000000000000000000000000000000000000000000000000000000000000
000000000000000000000000000000000000000000000000000000000000000000000000000000000000000000000000
000000000000000000000000000000000000000000000000000000000000000000000000000000000000000000000000
000000000000000000000000000000000000000000000000000000000000000000000000000000000000000111111111
11111111111111111111111111111111111111
>Q8A5P5_3sgg_22_556
AGVVVPKYTPSTENPGGPGEEPGDGLIDPAEPLDRGFMHLKGRELKSLNSISITGLNDGEKV
ILSTLAGLAARVTGDQVYINEGGPSSVWLKQMQNKYGIPVNTYNALAPLVQHYVETGVIK
GYIVYTPYSEGQSHSINVATSLCGLLRGIAVPESLVDKVKAMGVTTELMDVRSYDEKWLYEN
YKDQLDKSLAADMKPEIFHHLRDYITMTNAFAFYDYNARRDWSWRTSILKDLDKGAYCFG
YYDLDEWGMVNNASQLGVSMLPTDQAANLATLSSIYDTTGLKQRPATKEVVTEENVHYV
TFLVSDGDNIAFNLWGQQGYMDHDLHGQFPLGYTISPSLYDLAPAALRWYYENSKEGDYF
VAGPSGSSYIFPSKMSDADLDDYLAKLNEYVDKSGLNICNILDQKIMDNPKVYNKYLAQPN
IDAIFYTGYGEKGDGRIKFSDNGKPVIEQRSVLWEGIDGGSNRGEESTVISQINSRSANPHSA
DGYTFVFVHCWTKNQQSIKTVIDGLNDNVRVVPVDQFVQLVKQNLGPK
111111111111111111111111111111111111111111111000000000000000000000000000000000000000000000000000
000000000000000000000000000000000000000000000000000000000000000000000000000000000000000000000000
000000000000000000000000000000000000000000000000000000000000000000000000000000000000000000000000

00000000000000000000000000000000000000000000000000000000000000000000000000000000000
00000000000000000000000000000000000000000000000000000000000000000000000000000000000
00000000000000000000000000000000000000000000000000000000000000000000000000000000000
0000000000000000000000000000000000000
>A6XH05_2j5c_57_590
RTGGYQPTLWDFSTIQSFDSEYKEEKHLMRAAGMIDQVKMMLQEEVDSIRRLELIDDLRRL
GISCHFEREIVEILNSKYYTNNEIDERDLYSTALRFRLLRQYDFSVSQEVFDCFKNAKGTDFKP
SLVDDTRGLLQLYEASFLSAQGEETLRLARDFATKFLQKRVLVDKDINLLSSIERALELPTHW
RVQMPNARSFIDAYKRRPDMNPTVLELAKLDFNMVQAQFQQELKEASRWWNSTGLVHEL
PFVRDRIVECYYWTTGVVERRQHGYERIMLTKINALVTTIDDVFDIYGTLEELQLFTTAIQRW
DIESMKQLPPYMQICYLALFNFVNEMAYDTLRDKGFDSTPYLRKVWVGLIESYLIEAKWYY
KGHKPSLEEYMKNSWISIGGIPILSHLFFRLTDSIEEEAAESMHKYHDIVRASCTILRLADDM
GTSLDEVERGDVPKSVQCYMNEKNASEEAREHVRSLIDQTWKMMNKEMMTSSFSKYFV
EVSANLARMAQWIYQHESDGFGMQHSLVNKMLRDLLFHRYE
11111111111111111111111111000000000000000000000000000000000000000000000000000000000
00000000000000000000000000000000000000000000000000000000000000000000000000000000000
00110000000000000000000000000000000000000000000000000000000000000000000000000000000
00000000000000000000000000000000000000000000000000000000000000000000000000000000000
00000000000000000000000000000000000000000000000000000000000000000000000000000000000
00000000000000000000011111111100000000000000000000000000000000000000000000000000000
000000000000000001000000000000000000
>Q9UBK8_2qtl_191_724
GALPVASPASSRTDLVKSELLHIESQVELLRFDDSGRKDSEVLKQNAVNSNQSNVVIEDFESS
LTRSVPPLSQASLNIPGLPPEYLQVHLQESLGQEESQVSVTSADPVFQVPISKAVQLTTNDAI
KTTLLVELDISNTDFSYQPGDAFSVICPNSDSEVQSLLQRLQLEDKREHCVLLKIKADTKKK
GATLPQHIPAGCSLQFIFTWCLEIRAIPKKAFLRALVDYTSDSAEKRRLQELCSKQGAADYSR
FVRDACACLLDLLLAFPSCQPPLSLLLEHLPKLQPRPYSCASSSLFHPGKLHFVFNIVEFLSTA
TTEVLRKGVCTGWLALLVASVLQPNIHASHEDSGKALAPKISISPRTTNSFHLPDDPSIPIIMV
GPGTGIAPFIGFLQHREKLQEQHPDGNFGAMWLFFGCRHKDRDYLFRKELRHFLKHGILT
HLKVSFSRDAPVGEEEAPAKYVQDNIQLHGQQVARILLQENGHIYVCGDAKNMAKDVHD
ALVQIISKEVGVEKLEAMKTLATLKEEKRYLQDIWS
11111111111111111111111111111111111111111111111111110000000000000000000000000000000
00000000001111111111111100000000000000000000000000000000000000000000000000000000000
00000000000000000000000000000000000000000000000000000000000000000000000000000000000
00000000000000000000000000000000000000000000000000000000000000000000000000000000000
00000111111111111110000000000000000000000000000000000000000000000000000000000000000
00000000000000000000000000000000000111111000000000000000000000000000000000000000000
000000000000000000000000000000000000
>P11986_1p1j_0_532
MTEDNIAPITSVKVVTDKCTYKDNELLTKYSYENAVVTKTASGRFDVTPTVQDYVFKLDLK
KPEKLGIMLIGLGGNNGSTLVASVLANKHNVEFQTKEGVKQPNYFGSMTQCSTLKLGIDAE
GNDVYAPFNSLLPMVSPNDFVVSGWDINNADLYEAMQRSQVLEYDLQQRLKAKMSLVKP
LPSIYYPDFIAANQDERANNCINLDEKGNVTTRGKWTHLQRIRRDIQNFKEENALDKVIVL
WTANTERYVEVSPGVNDTMENLLQSIKNDHEEIAPSTIFAAASILEGVPYINGSPQNTFVPG
LVQLAEHEGTFIAGDDLKSGQTKLKSVLAQFLVDAGIKPVSIASYNHLGNNDGYNLSAPKQ
FRSKEISKSSVIDDIIASNDILYNDKLGKKVDHCIVIKYMKPVGDSKVAMDEYYSELMLGGH
NRISIHNVCEDSLLATPLIIDLLVMTEFCTRVSYKKVDPVKEDAGKFENFYPVLTFLSYWLKA
PLTRPGFHPVNGLNKQRTALENFLRLLIGLPSQNELRFEERLL
11111111000000000000000000000000000000000000000000000000000000000000000000000000000
00000000000000000000000000000000000000000000000000000000000000000000000000000000000
00000000000000000000000000000000000000000000000000000000000000000000000000000000000

00000000000000000000000000000000000000000000000000000000000000000000000000000000000
00000000000000000000000000000000000000000000000000000000000000000000000000000000000
00000000000000000000000000000000000000000000000000000000000000000000000000000000000
00000000000000000000000000000000000
>Q13WK4_2y52_0_530
MTELLKNHVAGQWIAGTGAGITLTDPVTGVALVRVSSEGLDLARAFSFAREDGGAALRALT
YAQRAARLADIVKLLQAKRGDYYAIATANSGTTRNDSAVDIDGGIFTLSYYAKLGASLGEV
HALRDGSAESLSKDRSFSAQHVLSPTRGVALFINAFNFPSWGLWEKAAPALLSGVPVIVKPA
TATAWLTQRMVADVVDAGILPPGALSIICGSSAGLLDQIRSFDVVSFTGSADTAATLRAHPA
FVQRGARLNVEADSLNSAILCADATPDTPAFDLFIKEVVREMTVKSGQKCTAIRRAFVPEA
ALEPVLEALKAKLAKITVGNPRNDAVRMGSLVSREQYENVLAGIAALREEAVLAYDSSAVP
LIDADANIAACVAPHLFVVNDPDNATLLHDVEVFGPVASVAPYRVTTDTNALPEAHAVAL
ARRGQGSLVASIYSNDDAHLGRLALELADSHGRVHAISPSVQHSQTGHGNVMPMSLHGG
PGRAGGGEELGGLRALAFYHRRSAIQAASAAIGTLTQATHWPAA
00000000000000000000000000000000000000000000000000000000000000000000000000000000000
00000000000000000000000000000000000000000000000000000000000000000000000000000000000
00000000000000000000000000000000000000000000000000000000000000000000000000000000000
00000000000000000000000000000000000000000000000000000000000000000000000000000000000
00000000000000000000000000000000000000000000000000000000000000000000000000000000000
00110000000000000000000000000000000000000000000000000000000000000000000000000000000
000000000000000000000000000000111
>Q8X5X6_2ahu_0_530
MKPVKPPRINGRVPVLSAQEAVNYIPDEATLCVLGAGGGILEATTLITALADKYKQTQTPRN
LSIISPTGLGDRADRGISPLAQEGLVKWALCGHWGQSPRISDLAEQNKIIAYNYPQGVLTQT
LRAAAAHQPGIISDIGIGTFVDPRQQGGKLNEVTKEDLIKLVEFDNKEYLYYKAIAPDIAFIR
ATTCDSEGYATFEDEVMYLDALVIAQAVHNNGIVMMQVQKMVKKATLHPKSVRIPGYL
VDIVVVDPDQSQLYGGAPVNRFISGDFTLDDSTKLSLPLNQRKLVARRALFEMRKGAVGNV
GVGIADGIGLVAREEGCADDFILTVETGPIGGITSQGIAFGANVNTRAILDMTSQFDFYHGG
GLDVCYLSFAEVDQHGNVGVHKFNGKIMGTGGFIDISATSKKIIFCGTLTAGSLKTEIADGK
LNIVQEGRVKKFIRELPEITFSGKIALERGLDVRYITERAVFTLKEDGLHLIEIAPGVDLQKDIL
DKMDFTPVISPELKLMDERLFIDAAMGFVLPEAAH
11100000000000000000000000000000000000000000000000000000000000000000000000000000000
00000000000000000000000000000000000000000000000000000000000000000000000000000000000
00000000000000000000000000000000000000000000000000000000000000000000000000000000000
00000000000000000000000000011111100000000000000000000000000000000000000000000000000
00000000001111100000000000000000000000000000000000000000000000000000000000000000000
00000000000000000000000000000000000000000000000000000000000000000000000000000000000
000000000000000000000000000000011
>Q9RUW8_2nvo_0_530
MKNLLRAINPLNRPQTERLDERQVRNNAGGFVYTVSDESRLTRFLVLGVDGGTFYASAQK
HTVQATDFVRELVQRDAALALRVTLDVVRGQRAPKADPALLVLALIAKTAPNAADRKAA
WDALPEVARTGTMLLHFLAFADALGGWGRLTRRGVANVYETADVDKLALWAVKYKARD
GWSQADALRKAHPKTDDAARNAVLKFMVDGVLPKVDSPALRVIEGHLKATEAQTDAAA
AALMQEYRLPLEAVPTHVRGAEVYRAAMQTNGLTWLLRNLGNLGRVGVLTPNDSATVQ
AVIERLTDPAALKRGRIHPLDALKARLVYAQGQGVRGKGTWLPVPRVVDALEEAFTLAFG
NVQPANTRHLLALDVSGSMTCGDVAGVPGLTPNMAAAAMSLIALRTEPDALTMGFAEQF
RPLGITPRDTLESAMQKAQSMSFGGTDCAQPILWAAQERLDVDTFVVYTDNETWAGQVHP
TVALDQYAQKMGRAPKLIVVGLTATEFSIADPQRRDMLDVVGFDAAAPNVMTAFARGEV
11111111111111111111111111111111111000000000000000000000000000000000000000000000000
00000000000000000000000000000000000000000000000000000000000000000000000000000000000
00000000000000000000000000000000000000000000000000000000000000000000000000000000000

00000000000000000000000000000000000000000000000000000000000000000000000000000000000
00000000000000000000000000000000000000000000000000000000000000000000000000000000000
00000000000000000000000000000000000000000000000000000000000000000000000000000000000
00000000000000000000000000000000
>Q7MPU6_3ufb_0_529
MTIRFMARTKKADQPMTTAQQLGAIVKSSRQIMRKDKGLNGDLDRLPMLTWIMFLKFLD
DLEQMRETEAVLEGKSFQPAIEAPYRWRDWAAIEGGITGDELIAFINNDEAMRPDGTRGIG
LFAYLRSLQGDNGGDRRDVIATVFKGMQNRMINGYLLRDVVDKINGIHFNSSEEMHTLSRL
YETMLREMRDAAGDSGEFYTPRPVVRFMVEVMDPQLGESVLDPACGTGGFLVEAFEHLER
QCKTVEDREVLQESSIFGGEAKSLPYLLVQMNLLLHGLEYPRIDPENSLRFPLREMGDKDRV
DVILTNPPFGGEEEKGILGNFPEDMQTAETAMLFLQLIMRKLKRPGHGSDNGGRAAVVVP
NGTLFSDGISARIKEELLKNFNLHTIVRLPEGVFAPYTDIAGNLLFFDRSGPTDDIWYYQITVP
EGRKKYTKTKPMESHEFDECLNWWSNRIVNQNAWKESASEIIKYSESGQLIDVNLDRKNP
NSLEVLEHEEPMDLIEKITTKEESLLAILEQIKAEISTQGISK
11111111111000000000000000000000000000000000000000000000000000000000000000000000000
00000000000000000000000000000000000000000000000000000000000000000000000000000000000
00000000000000000000000000000000000000000000000000000000000000000000000000000000000
00000000000000000000000000000000000000000000000000000000000000000000000000000000000
00000000000000000000000000000000000000000000000000000000000000000000000000000000000
00000000000000000000000000000000000000000000000000000000000000000000000000000000111
11111111111111111111111111111111
>Q8KI25_2r0c_0_528
MNAPIETDVLILGGGPVGMALALDLAHRQVGHLVVEQTDGTITHPRVGTIGPRSMELFRR
WGVAKQIRTAGWPGDHPLDAAWVTRVGGHEVYRIPLGTADTRATPEHTPEPDAICPQHW
LAPLLAEAVGERLRTRSRLDSFEQRDDHVRATITDLRTGATRAVHARYLVACDGASSPTRKA
LGIDAPPRHRTQVFRNILFRAPELRSLLGERAALFFFLMLSSSLRFPLRALDGRGLYRLTVGV
DDASKSTMDSFELVRRAVAFDTEIEVLSDSEWHLTHRVADSFSAGRVFLTGDAAHTLSPSGG
FGMNTGIGSAADLGWKLAATLRGWAGPGLLATYEEERRPVAITSLEEANVNLRRTMDREL
PPGLHDDGPRGERIRAAVAEKLERSGARREFDAPGIHFGHTYRSSIVCGEPETEVATGGWRP
SARPGARAPHAWLTPTTSTLDLFGRGFVLLSFGTTDGVEAVTRAFADRHVPLETVTCHAPEI
HALYERAHVLVRPDGHVAWRGDHLPAELGGLVDKVRGAA
10000000000000000000000000000000000000000000000000000000000000000000000000000000000
00000000000000000000000000000000000000000000000000000000000000000000000000000000000
00000000000000000000000000000000000000000000000000000000000000000000000000000000000
00000000000000000000000000000000000000000000000000000000000000000000000000000000000
00000000000000000000011111111110000000000000000000000000000000000000000000000000000
01111111110000000000000000000000000000000000000000000000000000000000000000000000000
0000000000000000000000000000000
>P20906_1q6z_0_527
MASVHGTTYELLRRQGIDTVFGNPGSNELPFLKDFPEDFRYILALQEACVVGIADGYAQAS
RKPAFINLHSAAGTGNAMGALSNAWNSHSPLIVTAGQQTRAMIGVEALLTNVDAANLPR
PLVKWSYEPASAAEVPHAMSRAIHMASMAPQGPVYLSVPYDDWDKDADPQSHHLFDRH
VSSSVRLNDQDLDILVKALNSASNPAIVLGPDVDAANANADCVMLAERLKAPVWVAPSA
PRCPFPTRHPCFRGLMPAGIAAISQLLEGHDVVLVIGAPVFRYHQYDPGQYLKPGTRLISVT
CDPLEAARAPMGDAIVADIGAMASALANLVEESSRQLPTAAPEPAKVDQDAGRLHPETVF
DTLNDMAPENAIYLNESTSTTAQMWQRLNMRNPGSYYFCAAGGLGFALPAAIGVQLAEP
ERQVIAVIGDGSANYSISALWTAAQYNIPTIFVIMNNGTYGALRWFAGVLEAENVPGLDVP
GIDFRALAKGYGVQALKADNLEQLKGSLQEALSAKGPVLIEVSTVSPVK
10000000000000000000000000000000000000000000000000000000000000000000000000000000000
00000000000000000000000000000000000000000000000000000000000000000000000000000000000
00000000000000000000000000000000000000000000000000000000000000000000000000000000000

000000000000000000000000000000000000000000000000000000000000000000000000000000000000
00000000000000000000000000000000000000000000000000000000000000000000000000000000000
00000000000000000000000000000000000000000000000000000000000000000000000000000000000
000000000000000000000000000111
>Q9UKM7_1x9d_171_698
PPSQDLKDGTQEEATKRQEAPVDPRPEGDPQRTVISWRGAVIEPEQGTELPSRRAEVPTKPP
LPPARTQGTPVHLNYRQKGVIDVFLHAWKGYRKFAWGHDELKPVSRSFSEWFGLGLTLID
ALDTMWILGLRKEFEEARKWVSKKLHFEKDVDVNLFESTIRILGGLLSAYHLSGDSLFLRKA
EDFGNRLMPAFRTPSKIPYSDVNIGTGVAHPPRWTSDSTVAEVTSIQLEFRELSRLTGDKKFQ
EAVEKVTQHIHGLSGKKDGLVPMFINTHSGLFTHLGVFTLGARADSYYEYLLKQWIQGGK
QETQLLEDYVEAIEGVRTHLLRHSEPSKLTFVGELAHGRFSAKMDHLVCFLPGTLALGVYH
GLPASHMELAQELMETCYQMNRQMETGLSPEIVHFNLYPQPGRRDVEVKPADRHNLLRPE
TVESLFYLYRVTGDRKYQDWGWEILQSFSRFTRVPSGGYSSINNVQDPQKPEPRDKMESFFL
GETLKYLFLLFSDDPNLLSLDAYVFNTEAHPLPIWTPA
11111111111111111111111111111111111111111111111111111111111111111111111110000000000
00000000000000000000000000000000000000000000000000000000000000000000000000000000000
00000000000000000000000000000000000000000000000000000000000000000000000000000000000
00000000000000000000000000000000000000000000000000000000000000000000000000000000000
00000000000000000000000000000000000000000000000000000000000000000000000000000000000
00000000000000000000000000000000000000000000000000000000000000000000000000000000000
000000100000000000000000000011
>P13507_1gcy_21_547
DQAGKSPNAVRYHGGDEIILQGFHWNVVREAPNDWYNILRQQAATIAADGFSAIWMPVP
WRDFSSWSDGSKSGGGEGYFWHDFNKNGRYGSDAQLRQAASALGGAGVKVLYDVVPNH
MNRGYPDKEINLPAGQGFWRNDCADPGNYPNDCDDGDRFIGGDADLNTGHPQVYGMF
RDEFTNLRSQYGAGGFRFDFVRGYAPERVNSWMTDSADNSFCVGELWKGPSEYPNWDWR
NTASWQQIIKDWSDRAKCPVFDFALKERMQNGSIADWKHGLNGNPDPRWREVAVTFVD
NHDTGYSPGQNGGQHHWALQDGLIRQAYAYILTSPGTPVVYWSHMYDWGYGDFIRQLIQ
VRRAAGVRADSAISFHSGYSGLVATVSGSQQTLVVALNSDLGNPGQVASGSFSEAVNASNG
QVRVWRSGTGSGGGEPGALVSVSFRCDNGATQMGDSVYAVGNVSQLGNWSPAAALRLTD
TSGYPTWKGSIALPAGQNEEWKCLIRNEANATQVRQWQGGANNSLTPSEGATTVGRL
00000000000000000000000000000000000000000000000000000000000000000001110000000000000
00000000000000000000000000000000000000000000000000000000000000000000000000000000000
00000000000000000000000000000000000000000000000000000000000000000000000000000000000
00000000000000000000000000000000000000000000000000000000000000000000000000000000000
00000000000000000000000000000000000000000000000000000000000000000000000000000000000
00011111111111111111111111111111111111111111111111111111111111111111111111111111111
11111111111111111111111111111
>P42251_2yeq_56_582
APNFSSYPFTLGVASGDPLSDSVVLWTRLAPDPLNGGGMPKQAVPVKWEVAKDEHFRKIV
RKGTEMAKPSLAHSVHVEADGLEPNKVYYYRFKTGHELSPVGKTKTLPAPGANVPQMTF
AFASCQQYEHGYYTAYKHMAKEKLDLVFHLGDYIYEYGPNEYVSKTGNVRTHNSAEIITLQ
DYRNRHAQYRSDANLKAAHAAFPWVVTWDDHEVENNYANKIPEKGQSVEAFVLRRAA
AYQAYYEHMPLRISSLPNGPDMQLYRHFTYGNLASFNVLDTRQYRDDQANNDGNKPPSD
ESRNPNRTLLGKEQEQWLFNNLGSSTAHWNVLAQQIFFAKWNFGTSASPIYSMDSWDGYP
AQRERVINFIKSKNLNNVVVLTGDVHASWASNLHVDFEKTSSKIFGAEFVGTSITSGGNGA
DKRADTDQILKENPHIQFFNDYRGYVRCTVTPHQWKADYRVMPFVTEPGAAISTRASFVY
QKDQTGLRKVSSTTIQGGVKQSDEVEEDRFFSHNKAHEKQMIKKRAKITN
00000000000000000000000000000000000000000000000000000000000000000000000000000000000
00000000000000000000000000000000000000000000000000000000000000000000000000000000000
00000000000000000000000000000000000000000000000000000000000000000000000000000000000

0000000000000000000000000000000000000000000000000000000000000000000000000000000000
0000000000000000000000000000000000000000000000000000000000000000000000000000000000
0000000000000000000000000000000000000000000000000000000000000000000000000000000000
00000000000000000000000011111
>Q04912_4fww_41_567
VKYVVPSFSAGGLVQAMVTYEGDRNESAVFVAIRNRLHVLGPDLKSVQSLATGPAGDPGC
QTCAACGPGPHGPPGDTDTKVLVLDPALPALVSCGSSLQGRCFLHDLEPQGTAVHLAAPA
CLFSAHHNRPDDCPDCVASPLGTRVTVVEQGQASYFYVASSLDAAVAASFSPRSVSIRRLKA
DASGFAPGFVALSVLPKHLVSYSIEYVHSFHTGAFVYFLTVQPASVTDDPSALHTRLARLSAT
EPELGDYRELVLDCRFAPKRRRRGAPEGGQPYPVLRVAHSAPVGAQLATELSIAEGQEVLFG
VFVTGKDGGPGVGPNSVVCAFPIDLLDTLIDEGVERCCESPVHPGLRRGLDFFQSPSFCPNP
PGLEALSPNTSCRHFPLLVSSSFSRVDLFNGLLGPVQVTALYVTRLDNVTVAHMGTMDGRIL
QVELVRSLNYLLYVSNFSLGDSGQPVQRDVSRLGDHLLFASGDQVFQVPIQGPGCRHFLTC
GRCLRAWHFMGCGWCGNMCGQQKECPGSWQQDHCP
0000000000000000000000000000000000000000000000000000000000000000000000000000000000
0000000000000000000000000000000000000000000000000000000000000000000000000000000000
0000000000000000000000000000000000000000000000000000000000000000000000000000000000
0000000000000011111111110000000000000000000000000000000000000001111000000000000000
0000000000000000000000000000000000000000000000000000000000000000000000000000000000
0000000000000000000000000000000000000000000000000000000000000000000000000000000000
00000000000000000000000000000
>Q5LGM0_3i4g_19_545
CDLERYPLTDLSEETFWNSESNAELALTSLYRGSLTDGVEYNPSDWWSYHGMIMMEHLSD
NAFDRRGENNPFFKISSGNLTADNAFIKRYWETSYKRIGYCNRFLVGIQNSSESEKKTRMIAE
ARFLRATQYFYLASYFKNVPLVENVLTGEEANNVTKTSQADILKWCVTEFTAAAADLPRFS
AIPAGEAGRACKQAALAFLGRTCMLQKDWKSGAKAFHDIMELGDNAINANYQELFYPST
GTSNKENIFYIQYLENYLGTGLPQHALSAKDGGWSLVNPAADLYESYEFKDGTPFSYDDPR
YDPSNLGKDRDPRLDYTIYYNGAIFMGTEYKMSPDYSAAKKEKLDYTSEASRTGFMMRKYF
EESTPINDVQSANGLTPVIRYAEVLLGYLECLVEDNQTITQGILDETINAVRGRASVNMPPVT
EVTPAKLREIVRHERRIELAMEGIRYWDIMRWGIAHEVLSQKIWGAPYPGSTQYATTTKEVD
PTGNYRWYVGKRAFRNPTDYTWPIPQSEQNINPNLRD
1111111111111000000000000000000000000000000000000000000000000000000000000000000000
0000000000000000000000000000000000000000000000000000000000000000000000000000000000
0000000000000000000000000000000000000000000000000000000000000000000000000000000000
0000000000000000000000000000000000000000000000000000000000000000000000000000000000
0000000000000000000000000000000000000000000000000000000000000000000000000000000000
0000000000000000000000000000000000000000000000000000000000000000000000000000000000
00000000000000000000000000001
>Q9I0N4_1yt8_1_526
SQIAVRTFHDIRAALLARRELALLDVREEDPFAQAHPLFAANLPLSRLELEIHARVPRRDTPI
TVYDDGEGLAPVAAQRLLDLGYSDVALLDGGLSGWRNAGGELFRDVNVPSKAFGELVEA
ERHTPSLAAEEVQALLDARAEAVILDARRFDEYQTMSIPGGISVPGAELVLRVAELAPDPRT
RVIVNCAGRTRSIIGTQSLLNAGIPNPVAALRNGTIGWTLAGQQLEHGQTRRFGAISQDTRK
AAAQRARAVADRAGVERLDLAGLAQWQDEHDRTTYLLDVRTPEEYEAGHLPGSRSTPGG
QLVQETDHVASVRGARLVLVDDDGVRANMSASWLAQMGWQVAVLDGLSEADFSERGAW
SAPLPRQPRADTIDPTTLADWLGEPGTRVLDFTASANYAKRHIPGAAWVLRSQLKQALERL
GTAERYVLTCGSSLLARFAVAEVQALSGKPVFLLDGGTSAWVAAGLPTEDGESLLASPRIDR
YRRPYEGTDNPREAMQGYLDWEFGLVEQLGRDGTHGFFVI
1100000000000000000000000000000000000000000000000000000000000000000000000000000000
0000000000000000000000000000000000000000000000000000000000000000000000000000000000
0000000000000000000000000000000000000000000000000000000000000000000000000000000000

00000000000000000000000000000000000000000000000000000000000000000000000000000000000
00000000000000000000000000000000000000000000000000000000000000000000000000000000000
00000000000000000000000000000000000000000000000000000000000000000000000000000000000
0000000000000000000000000000
>Q5F2I3_3fo3_28_552
EPGENLKPVDAMQCFDCHTQIEDMHTVGKHATVNCVHCHDATEHVETASSRRMGERPV
TRMDLEACATCHTAQFNSFVEVRHESHPRLEKATPTSRSPMFDKLIAGHGFAFEHAEPRSH
AFMLVDHFVVDRAYGGRFQFKNWQKVTDGMGAVRGAWTVLTDADPESSDQRRFLSQTAT
AANPVCLNCKTQDHILDWAYMGDEHEAAKWSRTSEVVEFARDLNHPLNCFMCHDPHSA
GPRVVRDGLINAVVDRGLGTYPHDPVKSEQQGMTKVTFQRGREDFRAIGLLDTADSNVMC
AQCHVEYNCNPGYQLSDGSRVGMDDRRANHFFWANVFDYKEAAQEIDFFDFRHATTGA
ALPKLQHPEAETFWGSVHERNGVACADCHMPKVQLENGKVYTSHSQRTPRDMMGQACL
NCHAEWTEDQALYAIDYIKNYTHGKIVKSEYWLAKMIDLFPVAKRAGVSEDVLNQARELH
YDAHLYWEWWTAENSVGFHNPDQARESLMTSISKSKEAVSLLNDAIDAQVASR
11110000000000000000000000000000000000000000000000000000000000000000000000000000000
00000000000000000000000000000000000000000000000000000000000000000000000000000000000
00000000000000000000000000000000000000000000000000000000000000000000000000000000000
00000000000000000000000000000000000000000000000000000000000000000000000000000000000
00000000000000000000000000000000000000000000000000000000000000000000000000000000000
00000000000000000000000000000000000000000000000000000000000000000000000000000000000
000000000000000000000000011
>Q5WWY0_2wzg_0_524
MKARRSNELSKLRMRFFSALNHTSEIDLHTLFDNLKSNLTLGSIEHLQEGSVTYAIIQELLKG
ADAQKKIESFLKGAIKNVIHPGVIKGLTPNEINWNVAKAYPEYYEHEKLPDVTFGGFKVRD
SNEFKFKTNVQTSIWFSIKPELFMPSKQQEALKRRREQYPGCKIRLIYSSSLLNPEANRQMKA
FAKKQNISLIDIDSVKTDSPLYPLIKAELANLGMGGNPAAASDLCRWIPELFNEGFYVDIDLP
VDSSKIVEGHQITGGVPIMLNMGSIISEPIAPHHRRQEAVCMNTDIIAYANDRETQVMMDT
VALHLKNIYDDPYTALKDTPLAQTAFFNRCEEEGKNIFELRKGLQDAFRSDSLLELYVFLGP
AKFKEVFKLKETQIKYIDDHISEFNEHDLLLHLISDNPSEINQHTLDFGRAKVMYMDIAKEH
YSAFYKPLVEEISGPGAIYNALGGASNFTTTHRRSTGPMLPTTPPRVLQVFCDAHDKGPFVS
DNIARWQTNVRELGVLNREGLSWLPSVG
11111000000000000000000000000000000000000000000000000000000000000000000000000000000
00000000000000000000000000000000000000000000000000000000000000000000000000000000000
00000000000000000000000000000000000000000000000000000000000000000000000000000000000
00000000000000000000000000000000000000000000000000000000000000000000000000000000000
00000000000000000000000000000000000000000000000000000000000000000000000000000011111
11100000000000000000000000000000000000000000000000000000000000000000000000000000000
000000000001111111100000011
>Q06801_1x1n_52_575
AVPAVGEDFPIDYADWLPKRDPNDRRRAGILLHPTSFPGPYGIGDLGPQAFKFLDWLHLAG
CSLWQVLPLVPPGKRGNEDGSPYSGQDANCGNTLLISLEELVDDGLLKMEELPEPLPTDRV
NYSTISEIKDPLITKAAKRLLSSEGELKDQLENFRRDPNISSWLEDAAYFAAIDNSVNTISWY
DWPEPLKNRHLAALEEVYQSEKDFIDIFIAQQFLFQRQWKKVRDYARSKGISIMGDMPIYV
GYHSADVWANKKQFLLNRKGFPLIVSGVPPDAFSETGQLWGSPLYDWKAMEKDGFSWW
VRRIQRATDLFDEFRIDHFRGFAGFWAVPSEEKIAILGRWKVGPGKPLFDAILQAVGKINIIA
EDLGVITEDVVQLRKSIEAPGMAVLQFAFGSDAENPHLPHNHEQNQVVYTGTHDNDTIRG
WWDTLPQEEKSNVLKYLSNIEEEEISRGLIEGAVSSVARIAIIPMQDVLGLGSDSRMNIPATQF
GNWSWRIPSSTSFDNLDAEAKKLRDILATYGRL
10000000000000000000000000000000000000000000000000000000000000000000000000000000000
00000000000000000000000000000000000000000000000000000000000000000000000000000000000
00000000000000000000000000000000000000000000000000000000000000000000000000000000000

00000000000000000000000000000000000000000000000000000000000000000000000000000000000
00000000000000000000000000000000000000000000000000000000000000000000000000000000000
00000000000000000000000000000000000000000000000000000000000000000000000000000000000
00000000000000000000000000
>Q1A2D0_3zq9_35_558
VVHGQTAKTITIKVDTSKERKPISPYIYGTNQDLAGDENMAARRLGGNRMTGYNWENNM
SNAGSDWQQSSDNYLCSNGGLTQAECEKPGAVTTSFHDQSLKLGTYSLVTLPMAGYVAKD
GNGSVQESEKAPSARWNQVVNAKNTPFQLQPDLNDNRVYVDEFVHFLVNKYGTASTKA
GVKGYALDNEPALWSHTHPRIHGEKVGAKELVDRSVSLSKAVKAIDAGAEIFGPVLYGFGA
YKDLQTAPDWDSVKGNYSWFVDYYLDQMRLSSQAEGKRLLDVFDVHWYPEAMGGGIRIT
NEVGNDETKKARMQAPRTLWDPTYKEDSWIAQWNSEFLPILPRLKQSVDKYYPGTKLAM
TEYSYGGENDISGGIAMTDVLGILGKNDVYMANYWKLKDGVNNYVSPAYKLYRNYDGKN
STFGDTSVSAQTSDIVNSSVHASVTNASDKELHLVVMNKSMDSAFDAQFDLSGAKTYSSGK
VWGFDKNSSQIKEAAPITQISGNRFTYTVPPLTAYHIVLTTGNYTSPV
11111100000000000000000000000000000000000000000000000000000000000000000000000000000
00000000000000000000000000000000000000000000000000000000000000000000000000000000000
00000000000000000000000000000000000000000000000000000000000000000000000000000000000
00000000000000000000000000000000000000000000000000000000000000000000000000000000000
00000000000000000000000000000000000000000000000000000000000000000000000000000000000
00000000000000000000000000000000000000000000000000000000000000000000000000000000000
00000000000000000000111111
>A6LG40_4fj6_19_540
ADSIYVREQQIPILIDRIDNVLYEMRIPAQKGDVLNEITIQIGDNVDLSDIQAIRLFYSGVEAPS
RKGEHFSPVTYISSHIPGNTRKALESYSVRQDEVTAPLSRTVKLTSKQPMLKGINYFWVSIQM
KPETSLLAKVATTMPNAQINNKPINITWKGKVDERHVGIGVRQAGDDGSAAFRIPGLVTTN
NGTLLGVYDIRYNSSVDLQEKIDIGVSRSTDKGQTWEPMRVAMTFKQTDGLPHGQNGVGD
PSILVDEKTNTIWVVAAWTHGMGNERAWWNSMPGMTPDETAQLMLVKSEDDGKTWSEPI
NITSQVKDPSWYFLLQGPGRGITMQDGTLVFPIQFIDATRVPNAGIMYSKDRGKTWHLHNL
ARTNTTEAQVAEVEPGVLMLNMRDNRGGSRAVATTKDLGKTWTEHPSSRSALQESVCMA
SLIKVNAKDNITGKDLLLFSNPNTTKGRNHITIKASLDGGLTWPTEHQVLLDEAEGWGYSC
LSMIDKETVGIFYESSVAHMTFQAVKLQDLIHQ
10000000000000000000000000000000000000000000000000000000000000000000000000000000000
00000000000000000000000000000000000000000000000000000000000000000000000000000000000
00000000000000000000000000000000000000000000000000000000000000000000000000000000000
00000000000000000000000000000000000000000000000000000000000000000000000000000000000
00000000000000000000000000000000000000000000000000000000000000000000000000000000000
00000000000000000000000000000000000000000000000000000000000000000000000000000000000
000000000000000000000001
>O15550_3avr_879_1400
QIIPSMSVSIYPSSAEVLKACRNLGKNGLSNSSILLDKCPPPRPPSSPYPPLPKDKLNPPTPSIY
LENKRDAFFPPLHQFCTNPNNPVTVIRGLAGALKLDLGLFSTKTLVEANNEHMVEVRTQL
LQPADENWDPTGTKKIWHCESNRSHTTIAKYAQYQASSFQESLREENEKRSHHKDHSDSES
TSSDNSGRRRKGPFKTIKFGTNIDLSDDKKWKLQLHELTKLPAFVRVVSAGNLLSHVGHTIL
GMNTVQLYMKVPGSRTPGHQENNNFCSVNINIGPGDCEWFVVPEGYWGVLNDFCEKNN
LNFLMGSWWPNLEDLYEANVPVYRFIQRPGDLVWINAGTVHWVQAIGWCNNIAWNVGP
LTACQYKLAVERYEWNKLQSVKSIVPMVHLSWNMARNIKVSDPKLFEMIKYCLLRTLKQC
QTLREALIAAGKEIIWHGRTKEEPAHYCSICEVEVFDLLFVTNESNSRKTYIVHCQDCARKT
SGNLENFVVLEQYKMEDLMQVYDQFTLAPPLPSASS
11111100000000000000001111111000000000000000000000000000000000000000000000000000000
00000000000000000000000000000000000000000000000000000000000000000000000000000000000
00111111111111111111111111111110000000000000000000000000000000000000000000000000000

00000000000000000000000000000000000000000000000000000000000000000000000000000000000000000000
00000000000000000000000000000000000000000000000000000000000000000000000000000000000000000000
00000000000000000000000000000000000000000000000000000000000000000000000000000000000000000000
000000000000000000111111
>Q9VRD9_3pc3_0_521
MPQPKPYERPADFIDPGKPSKCKWHLGTAEKSPHIHRGIAHRQQITPNILEVIGCTPLVKLN
NIPASDGIECEMYAKCEFLNPGGSVKDRIGYRMVQDAEEQGLLKPGYTIIEPTSGNTGIGLA
MACAVKGYKCIIVMPEKMSNEKVSALRTLGAKIIRTPTEAAYDSPEGLIYVAQQLQRETPNSI
VLDQYRNAGNPLAHYDGTAAEILWQLDNKVDMIVVSAGTAGTISGIGRKIKEQVPSCQIVG
VDPYGSILARPAELNKTDVQFYEVEGIGYDFPPTVFDDTVVDVWTKIGDSDCFPMSRRLNA
EEGLLCGGSSGGAMHAALEHARKLKKGQRCVVILPDGIRNYMTKFVSDNWMEARNFKEP
VNEHGHWWWSLAIAELELPAPPVILKSDATVGEAIALMKKHRVDQLPVVDQDDGSVLGV
VGQETLITQIVSMNRQQSDPAIKALNKRVIRLNESEILGKLARVLEVDPSVLILGKNPAGKVE
LKALATKLDVTTFIAAGKQKPKANGTTNGGSH
11111100000000000000000000000000000000000000000000000000000000000000000000000000000000000000
00000000000000000000000000000000000000000000000000000000000000000000000000000000000000000000
00000000000000000000000000000000000000000000000000000000000000000000000000000000000000000000
00000000000000000000000000000000000000000000000000000000000000000000000000000000000000000000
00000000000000000000000000000000000000000000000000000000000000000000000000000000000000000000
00000000000000000000000000000000000000000000000000000000000000000000000000000000000000000000
000000000000111111111111
>Q59675_1us2_85_605
VAASEGNVVIEVDMANGWRGNASGSTSHSGITYSADGVTFAALGDGVGAVFDIARPTTLE
DAVIAMVVNVSAEFKASEANLQIFAQLKEDWSKGEWDCLAASSELTADTDLTLTCTIDEDD
DKFNQTARDVQVGIQAKGTPAGTITIKSVTITLAQEAYSANVDHLRDLAPSDFPIGVAVSNT
DSATYNLLTNSREQAVVKKHFNHLTAGNIMKMSYMQPTEGNFNFTNADAFVDWATENN
MTVHGHALVWHSDYQVPNFMKNWAGSAEDFLAALDTHITTIVDHYEAKGNLVSWDVV
NEAIDDNSPANFRTTDSAFYVKSGNSSVYIERAFQTARAADPAVILYYNDYNIEQNNAKTTK
MVDMVKDFQARSIPIDGVGFQMHVCMNYPSIANISAAMKKVVDLGLLVKITELDVAVNQP
HCDAYPANKINPLTEAAQLAQKKRYCDVVKAYLDTVPVNQRGGISVWGTTDANTWLDG
LYREQFEDEKISWPLLFDNNYNDKPALRGFADALIGTQCTNTH
11111111100000000000000000000000000000000000000000000000000000000000000000000000000000000000
00000000000000000000000110000000000000000000000000000000000000000000000000000001110000000000
00000000000000000000000000000000000000000000000000000000000000000000000000000000000000000000
00000000000000000000000000000000000000000000000000000000000000000000000000000000000000000000
00000000000000000000000000000000000000000000000000000000000000000000000000000000000000000000
00000000000000000000000000000000000000000000000000000000000000000000000000000000000000000000
00000000000000000000000
>P51584_2w5f_31_550
ADYEVVHDTFEVNFDGWCNLGVDTYLTAVENEGNNGTRGMMVINRSSASDGAYSEKGFY
LDGGVEYKYSVFVKHNGTGTETFKLSVSYLDSETEEENKEVIATKDVVAGEWTEISAKYKAP
KTAVNITLSITTDSTVDFIFDDVTITRKGMAEANTVYAANAVLKDMYANYFRVGSVLNSGT
VNNSSIKALILREFNSITCENEMKPDATLVQSGSTNTNIRVSLNRAASILNFCAQNNIAVRGH
TLVWHSQTPQWFFKDNFQDNGNWVSQSVMDQRLESYIKNMFAEIQRQYPSLNLYAYDVV
NEAVSDDANRTRYYGGAREPGYGNGRSPWVQIYGDNKFIEKAFTYARKYAPANCKLYYND
YNEYWDHKRDCIASICANLYNKGLLDGVGMQSHINADMNGFSGIQNYKAALQKYINIGC
DVQITELDISTENGKFSLQQQADKYKAVFQAAVDINRTSSKGKVTAVCVWGPNDANTWLG
SQNAPLLFNANNQPKPAYNAVASIIPQSEWGDGNNPA
00000000000000000000000000000000000000000000000000000000000000000000000000000000000000000000
00000000000000000000000000000000000000000000000000000000000000000000000000001111111000000000
00000000000000000000000000000000000000000000000000000000000000000000000000000000000000000000

000000000000000000000000000000000000000000000000000000000000000000000000000000000000
000000000000000000000000000000000000000000000000000000000000000000000000000000000000
000000000000000000000000000000000000000000000000000000000000000000000000000000000000
0000000000000000000000
>Q9ZBA9_1ei5_0_519
MSKFDTSALEAFVRHIPQNYKGPGGVVAVVKDGEVVLQHAWGFADLRTRTPMTLDTRMPI
CSVSKQFTCAVLLDAVGEPELLDDALEAYLDKFEDERPAVRDLCNNQSGLRDYWALSVLCG
ADPEGVFLPAQAQSLLRRLKTTHFEPGSHYSYCNGNFRILADLIEAHTGRTLVDILSERIFAP
AGMKRAELISDTALFDECTGYEGDTVRGFLPATNRIQWMGDAGICASLNDMIAWEQFIDA
TRDDESGLYRRLSGPQTFKDGVAAPYGFGLNLHETGGKRLTGHGGALRGWRCQRWHCA
DERLSTIAMFNFEGGASEVAFKLMNIALGVSSSEVSRVEADSAWFGSWLDDETGLVLSLEDA
GHGRMKARFGTSPEMMDVVSANEARSAVTTIRRDGETIELVRASENLRLSMKRVKGEAKH
DIIGRYHSDELDADLLLVSEGGAIYGAFEGFLGKSDMYPLYSVGSDVWLLPVQRSMDAPSPG
EWKLVFRRDDKGEITGLSVGCWLARGVEYRRVQP
110000000000000000000000000000000000000000000000000000000000000000000000000000000000
000000000000000000000000000000000000000000000000000000000000000000000000000000000000
000000000000000000000000000000000000000000000000000000000000000000000000000000000000
000000000000000000000000000000000000000000000000000000000000000000000000000000000000
000000000000000000000000000000000000000000000000000000000000000000000000000000000000
000000000000000000000000000000000000000000000000000000000000000000000000000000000000
0000000000000000000000
>Q9C1B7_3fot_0_518
MSASSSSALPPLVPALYRWKSTGSSGRQVQRRCVGAEAIVGLEEKNRRALYDLYIATSLRNIA
PASTLLTLQNLKEMFELALLDARFEHPECACTVSWDDEVPAIITYESPESNESARDWARGCI
HVQPTAKSALDLWSEMEEGRAAANDNTPSKSIELFLLSDVSTDSTPIPQDATVEILFHSNHL
FWDGIGCRKFVGDLFRLVGSYIGRSDSREMKKIQWGQEIKNLSPPVVDSLKLDINTLGSEFD
DKCTEYTSALVANYKSRGMKFQPGLALPRCVIHKLSADESIAIVKAVKTRLGPGFTISHLTQ
AAIVLALLDHLKPNDLSDDEVFISPTSVDGRRWLREDIASNFYAMCQTAAVVRIENLKSIAV
SHKDEKELQVRALESACRNIKKSYRQWLENPFLQALGLRVHNFEASYLHAKPIPFEGEANP
LFISDGINERFIPHEIKQTATGENVLSVESIDFVVNQSLPYLAIRLDSWRDASTLNIIYNDANY
TEAEVQKYLQSIVEFMLAFRL
111111110000000000000000000000000000000000000000000000000000000000000000000000000000
000000000000000000000000000000000000000000000000000000000000000000010000000000000000
000000000000000000000000000000000000000000000000000000000000000000000000000000000000
000000000000000000000000000000000000000000000000000000000000000000000000000111000000
000000000000000000000000000000000000000000000000000000000000000000000000000000000000
000000000000000000000000000000000000000000000000000000000000000000000000000000000000
000000000000000000000
>Q1M964_2vqr_0_513
MRKKNVLLIVVDQWRADFVPHVLRADGKIDFLKTPNLDRLCREGVTFRNHVTTCVPCGP
ARASLLTGLYLMNHRAVQNTVPLDQRHLNLGKALRGVGYDPALIGYTTTVPDPRTTSPND
PRFRVLGDLMDGFHPVGAFEPNMEGYFGWVAQNGFDLPEHRPDIWLPEGEDAVAGATDR
PSRIPKEFSDSTFFTERALTYLKGRDGKPFFLHLGYYRPHPPFVASAPYHAMYRPEDMPAPIR
AANPDIEAAQHPLMKFYVDSIRRGSFFQGAEGSGATLDEAELRQMRATYCGLITEVDDCLG
RVFSYLDETGQWDDTLIIFTSDHGEQLGDHHLLGKIGYNDPSFRIPLVIKDAGENARAGAIE
SGFTESIDVMPTILDWLGGKIPHACDGLSLLPFLSEGRPQDWRTELHYEYDFRDVYYSEPQS
FLGLGMNDCSLCVIQDERYKYVHFAALPPLFFDLRHDPNEFTNLADDPAYAALVRDYAQK
ALSWRLKHADRTLTHYRSGPEGLSERSH
100000000000000000000000000000000000000000000000000000000000000000000000000000000000
000000000000000000000000000000000000000000000000000000000000000000000000000000000000
000000000000000000000000000000000000000000000000000000000000000000000000000000000000

000000000000000000000000000000000000000000000000000000000000000000000000000000000000
000000000000000000000000000000000000000000000000000000000000000000000000000000000000
000000000000000000000000000000000000000000000000000000000000000000000000000000000000
0000000000000000
>Q55168_3zq5_0_513
MATTVQLSDQSLRQLETLAIHTAHLIQPHGLVVVLQEPDLTISQISANCTGILGRSPEDLLGR
TLGEVFDSFQIDPIQSRLTAGQISSLNPSKLWARVMGDDFVIFDGVFHRNSDGLLVCELEPAY
TSDNLPFLGFYHMANAALNRLRQQANLRDFYDVIVEEVRRMTGFDRVMLYRFDENNHGD
VIAEDKRDDMEPYLGLHYPESDIPQPARRLFIHNPIRVIPDVYGVAVPLTPAVNPSTNRAVDL
TESILRSAYHCHLTYLKNMGVGASLTISLIKDGHLWGLIACHHQTPKVIPFELRKACEFFGRV
VFSNISAQEDTETFDYRVQLAEHEAVLLDKMTTAADFVEGLTNHPDRLLGLTGSQGAAICF
GEKLILVGETPDEKAVQYLLQWLENREVQDVFFTSSLSQIYPDAVNFKSVASGLLAIPIARHN
FLLWFRPEVLQTVNWGGDPNHAYEATQEDGKIELHPRQSFDLWKEIVRLQSLPWQSVEIQS
ALALKKAIVNLILRQAEE
100000000000000000000000000000000000000000000000000000000000000000000000000000000000
000000000000000000000000000000000000000000000000000000000000000000000000000000000000
000000000000000000000000000000000000000000000000000000000000000000000000000000000000
000000000000000000000000000000000000000000000000000000000000000000000000000000000000
000000000000000000000000000000000000000000000000000000000000000000000000000000000000
000000000000000000000000000000000000000000000000000000000000000000000000000000000000
0000000000000000
>Q9SQT8_2o7s_89_602
VKNPSLICAPVMADSIDKMVIETSKAHELGADLVEIRLDWLKDFNPLEDLKTIIKKSPLPTLF
TYRPKWEGGQYEGDENERRDVLRLAMELGADYIDVELQVASEFIKSIDGKKPGKFKVIVSS
HNYQNTPSVEDLDGLVARIQQTGADIVKIATTAVDIADVARMFHITSKAQVPTIGLVMGER
GLMSRILCSKFGGYLTFGTLDSSKVSAPGQPTIKDLLDLYNFRRIGPDTKVYGIIGKPVSHSKS
PIVHNQAFKSVDFNGVYVHLLVDNLVSFLQAYSSSDFAGFSCTIPHKEAALQCCDEVDPLA
KSIGAVNTILRRKSDGKLLGYNTDCIGSISAIEDGLRSSGDPSSVPSSSSPLASKTVVVIGAGGA
GKALAYGAKEKGAKVVIANRTYERALELAEAIGGKALSLTDLDNYHPEDGMVLANTTSM
GMQPNVEETPISKDALKHYALVFDAVYTPRITRLLREAEESGAITVSGSEMFVRQAYEQFEIF
TGLPAPKELYWQIMSKY
000000000000000000000000000000000000000000000000000000000000000000000000000000000000
000000000000000000000000000000000000000000000000000000000000000000000000000000000000
000000000000000000000000000000000000000000000000000000000000000000000000000000000000
000000000000000000000000000000000000000000000000000000000000000000000000000000000000
000000000000001111111111111111111000000000000000000000000100000000000000000000100000
000001100000000000000000000000000000000000000000000000000000000000000000000000000000
0000000000000000
>Q9XJP3_2vbe_109_622
DPDQFGPDLIEQLAQSGKYSQDNTKGDAMIGVKQPLPKAVLRTQHDKNKEAISILDFGVID
DGVTDNYQAIQNAIDAVASLPSGGELFIPASNQAVGYIVGSTLLIPGGVNIRGVGKASQLRA
KSGLTGSVLRLSYDSDTIGRYLRNIRVTGNNTCNGIDTNITAEDSVIRQVYGWVFDNVMVN
EVETAYLMQGLWHSKFIACQAGTCRVGLHFLGQCVSVSVSSCHFSRGNYSADESFGIRIQPQ
TYAWSSEAVRSEAIILDSETMCIGFKNAVYVHDCLDLHMEQLDLDYCGSTGVVIENVNGGF
SFSNSWIAADADGTEQFTGIYFRTPTSTQSHKIVSGVHINTANKNTAANNQSIAIEQSAIFVF
VSGCTLTGDEWAVNIVDINECVSFDKCIFNKPLRYLRSGGVSVTDCYLAGITEVQKPEGRYN
TYRGCSGVPSVNGIINVPVAVGATSGSAAIPNPGNLTYRVRSLFGDPASSGDKVSVSGVTINV
TRPSPVGVALPSMVEYLAI
111100000000000000000000000000000000000000000000000000000000000000000000000000000000
000000000000000000000000000000000000000000000000000000000000000000000000000000000000
000000000000000000000000000000000000000000000000000000000000000000000000000000000000

00000000000000000000000000000000000000000000000000000000000000000000000000000000000
00000000000000000000000000000000000000000000000000000000000000000000000000000000000
00000000000000000000000000000000000000000000000000000000000000000000000000000000000
0000000000000000
>P0AFL6_1u6z_0_512
MPIHDKSPRPQEFAAVDLGSNSFHMVIARVVDGAMQIIGRLKQRVHLADGLGPDNMLSEE
AMTRGLNCLSLFAERLQGFSPASVCIVGTHTLRQALNATDFLKRAEKVIPYPIEIISGNEEAR
LIFMGVEHTQPEKGRKLVIDIGGGSTELVIGENFEPILVESRRMGCVSFAQLYFPGGVINKENF
QRARMAAAQKLETLTWQFRIQGWNVAMGASGTIKAAHEVLMEMGEKDGIITPERLEKLV
KEVLRHRNFASLSLPGLSEERKTVFVPGLAILCGVFDALAIRELRLSDGALREGVLYEMEGRF
RHQDVRSRTASSLANQYHIDSEQARRVLDTTMQMYEQWREQQPKLAHPQLEALLRWAA
MLHEVGLNINHSGLHRHSAYILQNSDLPGFNQEQQLMMATLVRYHRKAIKLDDLPRFTLF
KKKQFLPLIQLLRLGVLLNNQRQATTTPPTLTLITDDSHWTLRFPHDWFSQNALVLLDLEKE
QEYWEGVAGWRLKIEEESTPEIAA
11111111111000000000000000000000000000000000000000000000000000000000000000000000000
00000000000000000000000000000000000000000000000000000000000000000000000000000000000
00000000000000000000000000000000000000000000000000000000000000000000000000000000000
00000000000000000000000000000000000000000000000000000000000000000000000000000000000
00000000000000000000000000000000000000000000000000000000000000000000000000000000000
00000000000000000000000000000000000000000000000000000000000000000000000000000000000
000000000000011
>P11349_1q16_0_511
MKIRSQVGMVLNLDKCIGCHTCSVTCKNVWTSREGVEYAWFNNVETKPGQGFPTDWEN
QEKYKGGWIRKINGKLQPRMGNRAMLLGKIFANPHLPGIDDYYEPFDFDYQNLHTAPEGS
KSQPIARPRSLITGERMAKIEKGPNWEDDLGGEFDKLAKDKNFDNIQKAMYSQFENTFMM
YLPRLCEHCLNPACVATCPSGAIYKREEDGIVLIDQDKCRGWRMCITGCPYKKIYFNWKSG
KSEKCIFCYPRIEAGQPTVCSETCVGRIRYLGVLLYDADAIERAASTENEKDLYQRQLDVFLD
PNDPKVIEQAIKDGIPLSVIEAAQQSPVYKMAMEWKLALPLHPEYRTLPMVWYVPPLSPIQ
SAADAGELGSNGILPDVESLRIPVQYLANLLTAGDTKPVLRALKRMLAMRHYKRAETVDG
KVDTRALEEVGLTEAQAQEMYRYLAIANYEDRFVVPSSHRELAREAFPEKNGCGFTFGDG
CHGSDTKFNLFNSRRIDAIDVTSKTEPHP
00000000000000000000000000000000000000000000000000000000000000000000000000000000000
00000000000000000000000000000000000000000000000000000000000000000000000000000000000
00000000000000000000000000000000000000000000000000000000000000000000000000000000000
00000000000000000000000000000000000000000000000000000000000000000000000000000000000
00000000000000000000000000000000000000000000000000000000000000000000000000000000000
00000000000000000000000000000000000000000000000000000000000000000000000000000000000
00000000000111
>A7ASX7_3nrr_0_510
MSNSYEGCGDLTIFVAVALNKVIGHKNQIPWPHITHDFRFLRNGTTYIPPEVLSKNPDIQNV
VIFGRKTYESIPKASLPLKNRINVILSRTVKEVPGCLVYEDLSTAIRDLRANVPHNKIFILGGSF
LYKEVLDNGLCDKIYLTRLNKEYPGDTYFPDIPDTFEITAISPTFSTDFVSYDFVIYERKDCKTV
FPDPPFDQLLMTGTDISVPKPKYVACPGVRIRNHEEFQYLDILADVLSHGVLKPNRTGTDAY
SKFGYQMRFDLSRSFPLLTTKKVALRSIIEELLWFIKGSTNGNDLLAKNVRIWELNGRRDFLD
KNGFTDREEHDLGPIYGFQWRHFGAEYLDMHADYTGKGIDQLAEIINRIKTNPNDRRLIVC
SWNVSDLKKMALPPCHCFFQFYVSDNKLSCMMHQRSCDLGLGVPFNIASYSILTAMVAQV
CGLGLGEFVHNLADAHIYVDHVDAVTTQIARIPHPFPRLRLNPDIRNIEDFTIDDIVVEDYV
SHPPIPMAMSA
11000000000000000000000000000000000000000000000000000000000000000000000000000000000
00000000000000000000000000000000000000000000000000000000000000000000000000000000000
00000000000000000000001111110000000000000000000000000000000000000000000000000000000

0000000000000000000000000000000000000000000000000000000000000000000000000000000000000
00000000000000000000000000000000000000000000000000000000000000000000000000000000000
00000000000000000000000000000000000000000000000000000000000000000000000000000000000
0000000000000
>Q9X519_1o98_0_510
MSKKPVALIILDGFALRDETYGNAVAQANKPNFDRYWNEYPHTTLKACGEAVGLPEGQM
GNSEVGHLNIGAGRIVYQSLTRINIAIREGEFDRNETFLAAMNHVKQHGTSLHLFGLLSDG
GVHSHIHHLYALLRLAAKEGVKRVYIHGFLDGRDVGPQTAPQYIKELQEKIKEYGVGEIATL
SGRYYSMDRDKRWDRVEKAYRAMVYGEGPTYRDPLECIEDSYKHGIYDEFVLPSVIVREDG
RPVATIQDNDAIIFYNFRPDRAIQISNTFTNEDFREFDRGPKHPKHLFFVCLTHFSETVKGYV
AFKPTNLDNTIGEVLSQHGLRQLRIAETEKYPHVTFFMSGGREEKFPGEDRILINSPKVPTYD
LKPEMSAYEVTDALLKEIEADKYDAIILNYANPDMVGHSGKLEPTIKAVEAVDECLGKVVD
AILAKGGIAIITADHGNADEVLTPDGKPQTAHTTNPVPVIVTKKGIKLRDGGILGDLAPTML
DLLGLPQPKEMTGKSLIVK
10000000000000000000000000000000000000000000000000000000000000000000000000000000000
00000000000000000000000000000000000000000000000000000000000000000000000000000000000
00000000000000000000000000000000000000000000000000000000000000000000000000000000000
00000000000000000000000000000000000000000000000000000000000000000000000000000000000
00000000000000000000000000000000000000000000000000000000000000000000000000000000000
00000000000000000000000000000000000000000000000000000000000000000000000000000000000
0000000000001
>A6L2F0_4f7a_27_536
DNKINGSFDDTVKEYDFQKYTTNFETIQKGIYFNYDWGEGTTWPWQTFQNLNHDMFSGY
FHDFASKFSDKNTVYALEAGWTASAWNYTYNYIFPVAHKSTLITQDEAKYKHFYGATLILK
VEAMHRITDTYGPIVYSKFGKNETNSVDTQEEAYKAFFDDLDKAVDALDTYLKEGGKEDG
VKSINMCNCPTASRWIKFANSLRLRLAMRVSNVDKTLATSEAQKALENSYGVIESSDENIQI
SGKGYQNPLAGVAGWGETYMGATIASVLNGYEDPRISIYYNPATLAEHTEEYLGVPQGVYA
KDGDPNYYQSYSFINTQTITASTPAVLLTAAETWFLRAEASLRGINPKNESAKQCYEAGVQT
SFSQWGAGDASLYLTSKGKPTDYINYAAGPGKDMKALITTTPNFDDAVNQEEQLEKIITQK
WIACWPEGMEAWAEQRRTGYPKLFKVQTNNSNGTIDTDIMIRRLPFSQDDAKKDPEQYK
NLCTALGGADNGGTRLWWDTGKNNF
11111111111100000000000000000000000000000000000000000000000000000000000000000000000
00000000000000000000000000000000000000000000000000000000000000000000000000000000000
00000000000000000000000000000000000000000000000000000000000000000000000000000000000
00000000000000000000000000000000000000000000000000000000000000000000000000000000000
00000000000000000000000000000000000000000000000000000000000000000000000000000000000
00000000000000000000000000000000000000000000000000000000000000000000000000000000000
000000000000
>B5TYU1_3c3v_20_529
ISFRQQPEENACQFQRLNAQRPDNRIESEGGYIETWNPNNQEFECAGVALSRLVLRRNALR
RPFYSNAPQEIFIQQGRGYFGLIFPGCPSTYEEPAQQGRRYQSQRPPRRLQEEDQSQQQQDS
HQKVHRFNEGDLIAVPTGVAFWLYNDHDTDVVAVSLTDTNNNDNQLDQFPRRFNLAGN
HEQEFLRYQQQSRQSRRRSLPYSPYSPQSQPRQEEREFSPRGQHSRRERAGQEEENEGGNIFS
GFTPEFLAQAFQVDDRQIVQNLRGENESEEQGAIVTVRGGLRILSPDRKRGADEEEEYDED
EYEYDEEDRRRGRGSRGSGNGIEETICTATVKKNIGRNRSPDIYNPQAGSLKTANELNLLILR
WLGLSAEYGNLYRNALFVPHYNTNAHSIIYALRGRAHVQVVDSNGNRVYDEELQEGHVL
VVPQNFAVAGKSQSDNFEYVAFKTDSRPSIANLAGENSVIDNLPEEVVANSYGLPREQARQ
LKNNNPFKFFVPPSQQSPRAVA
00000000000000000000000000000000000000000000000000000000000000000000000000000000000
00000000000000111111111111111111110000000000000000000000000000000000000000000000000
00000000000000000000000001111111111111111111111111111111111111111111111110000000000

00000000000000000000000000000000000000000111111111111111111111111111111111110000000
00000000000000000000000000000000000000000000000000000000000000000000000000000000000
00000000000000000000000000000000000000000000000000000000000000000000000000000000000
000111111111
>Q3MB62_3eo7_0_509
MPEIHQSIAQHYHERTKYDPETIASKSQRLDWAKQPVPFKEYKIGSAIDLKPYLQETPEVFV
NDTNGQWWQRLSRLLFRSYGLTARMPSMGNTVYLRAAPSAGGLYPAEVYVVSRGTPLLSP
GLYNYQCRTHSLIHYWESDVWQSLQEACFWHPALESTQLAIIVTAVFYRSAWRYEDRAYRR
ICLDTGHLLGNIELSAAITDYRPHLIGGFIDEAVNDLLYIDPLQEGAIAVLPLADLLDIQQNIS
PGCTALPSATETNYPQVPDGELLKYFHHHTQISASITGKLNLPTVIQEKSLEDKYNFPFCLKI
STVSAPIYWGENLSDLEITMHKRRSTRAYNGEELTFDELKALLDFTYQPQNYIDQSLDNSPD
YFDLNLIETFIAVCGVQGLEAGCYYYAPKAQELRQIRFKNFRRELHFLCLGQELGRDAAAVI
FHTSDLKSAIAQYGDRVYRYLHMDAGHLGQRLNLAAIQLNLGVSGIGGFFDDQVNEVLGI
PNDEAVIYITTLGRPR
11110000000000000000000000110000000000000000000000000000111110000000000000000000000
00000000000000000000000000000000000000000000000000000000000000000000000000000000000
00000000000000000000000000000000000000000000000000000000000000000000000000000000000
00000000000000000000000000000000000011111111111100000000000000000000000000000000000
00000000000000000000000000000000000000000000000000000000000000000000000000000000000
00000000000000000000000000000000000000000000000000000000000000000000000000000000000
000000000000
>A7M4E1_4eps_25_533
EEEVPGNVRNGIVLNVTDTGIISNEPSTRTEDTGFVTTFTQGDQIGLFAVKDGAILDEINNMP
FTFNGSSWSGKPILYDDRLVGVNFYAYYPYQSEMTGKTDLIGDDFFAPLAAGWELTTEQSD
QKAYAKQDLMTSNATALIGENGNYSLSFQLTHRMSLVVVKLPSTRYIFTDAEGVAMPEETPY
VAMSVDVAFYLDNVEEGTKISPYYDAKKDEYRLLRKPSSENQIIGHYNDKQCTLDTAEKMK
EGKYKRFVVDGGYKEVTHHLQVGDYYYADGSVVSGNEAEPAKDNCIGIVCWVGNPMPSV
LYKDVAGTPYTATNDALLRSHPNCVHGLVMSLYTETGKFSPALTQSIHDWFMTTSFTSSYVS
VTGYYDANENNKNKPLRFLGYNNSEVLDLYYDTFKTDFECFQYQDDCESSFPSPSITTGWY
VPSSGELVALQDKDNSLESKLNTKLIKVSDKTMDISATYWSSTERNNKNMYIVTYSKTAGSA
GTGGVKTNTYTYRFFLGF
11111100000000000000000001111110000000000000000000000000000000000000000000000000000
00000000000000000000000000000000000000000000000000000000000000000000000000000000000
00000000000000000000000000000000000000000000000000000000000000000000000000000000000
00000000000000000000000000000000000000000000000000000000000000000000000000000000000
00000000000000000000000000000000000000000000000000000000000000000000000000000000000
00000000000000000000000000000000000000000000000000000000000000000000000000000000000
00000000000
>P06681_2odp_243_751
KIQIQRSGHLNLYLLLDCSQSVSENDFLIFKESASLMVDRIFSFEINVSVAIITFASEPKVLMSVL
NDNSRDMTEVISSLENANYKDHENGTGTNTYAALNSVYLMMNNQMRLLGMETMAWQE
IRHAIILLTDGKSNMGGSPKTAVDHIREILNINQKRNDYLDIYAIGVGKLDVDWRELNELGS
KKDGERHAFILQDTKALHQVFEHMLDVSKLTDTICGVGNMSANASDQERTPWHVTIKPKS
QETCRGALISDQWVLTAAHCFRDGNDHSLWRVNVGDPKSQWGKEFLIEKAVISPGFDVFA
KKNQGILEFYGDDIALLKLAQKVKMSTHARPICLPCTMEANLALRRPQGSTCRDHENELL
NKQSVPAHFVALNGSKLNINLKMGVEWTSCAEVVSQEKTMFPNLTDVREVVTDQFLCSGT
QEDESPCKGESGGAVFLERRFRFFQVGLVSWGLYNPCLGSADKNSRKRAPRSKVPPPRDFHI
NLFRMQPWLRQHLGDVLNFLPL
00000000000000000000000000000000000000000000000000000000000000000000000000000000000
00000000000000000000000000000000000000000000000000000000000000000000000000000000000
00000000000000000000000000000000000000000000000000000000000000000000000000000111100

00000000000000000111111110000000000000000000000000000000000000000000000000000000000
00000000000000000000000000000000000000000000000000000000000000000000000000000000000
00000000000000000000000000000000000000000000000011111000000000000000000000000000000
00000000011
>Q44124_3hol_38_546
GVSKEEYKDVETAKKEKEQLGELMEPALGYVVKVPVSSFENKKVDISDIEVITNGNLDDVPY
KANSSKYNYPDIKTKDSSLQYVRSGYVIDGEHSGSNEKGYVYYKGNSPAKELPVNQLLTYT
GSWDFTSNANLNNEEGRPNYLNDDYYTKFIGKRVGLVSGDAKPAKHKYTSQFEVDFATKK
MTGKLSDKEKTIYTVNADIRGNRFTGAATASDKNKGKGESYNFFSADSQSLEGGFYGPKAE
EMAGKFVANDKSLFAVFSAKHNGSNVDTVRIIDASKIDLTNFSISELNNFGDASVLIIDGKKI
KLAGSGFTNKHTIEINGKTMVAVACCSNLEYMKFGQLWQQAEGGKPENNSLFLQGERTAT
DKMPKGGNYKYIGTWDAQVSKENNWVATADDDRKAGYRTEFDVDFGNKNLSGKLFDK
NGVNPVFTVDPKIDGNGFTGKAKTSDAGFALDSGSSRYENVKFNDVAVSGGFYGPTAAEL
GGQFHHKSENGSVGAVFGAKQQVKK
11110000000000000000000000000000000000000000000000000000000000000000000000000000000
00000000000000000000000000000000000000000000000000000000000000000000000000000000000
00000000000000000000000000000000000000000000000000000000000000000000000000000000000
00000000000000000000000000000000000000000000000000000000000000000000000000000000000
00000000000000000000000000000000000000000000000000000000000000000000000000000000000
00000000000000000000000000000000000000000000000000000000000000000000000000000000000
00000000000
>P84147_1v0t_0_505
ADSATPHLDAVEQTLRQVSPGLEGDVWERTSGNKLDGSAADPSDWLLQTPGCWGDDKC
ADRVGTKRLLAKMTENIGNATRTVDISTLAPFPNGAFQDAIVAGLKESAAKGNKLKVRILV
GAAPVYHMNVIPSKYRDELTAKLGKAAENITLNVASMTTSKTAFSWNHSKILVVDGQSALT
GGINSWKDDYLDTTHPVSDVDLALTGPAAGSAGRYLDTLWTWTCQNKSNIASVWFAASG
NAGCMPTMHKDTNPKASPATGNVPVIAVGGLGVGIKDVDPKSTFRPDLPTASDTKCVVGL
HDNTNADRDYDTVNPEESALRALVASAKGHIEISQQDLNATCPPLPRYDIRLYDALAAKM
AAGVKVRIVVSDPANRGAVGSGGYSQIKSLSEISDTLRNRLANITGGQQAAKTAMCSNLQL
ATFRSSPNGKWADGHPYAQHHKLVSVDSSTFYIGSKNLYPSWLQDFGYIVESPEAAKQLDA
KLLDPQWKYSQETATVDYARGICNA
11000000000000000000000000000000000000000000000000000000000000000000000000000000000
00000000000000000000000000000000000000011111100000000000000000000000000000000000000
00000000000000000000000000000000000000000000000000000000000000000000000000000000000
00000000000000000000000000000000000000000000000000000000000000000000000000000000000
00000000000000000000000000000000000000000001111110000000000000000000000000000000000
00000000000000000000000000000000000000000000000000000000000000000000000000000000000
00000001
>Q46079_1dbg_0_505
MKMLNKLAGYLLPIMVLLNVAPCLGQVVASNETLYQVVKEVKPGGLVQIADGTYKDVQLI
VSNSGKSGLPITIKALNPGKVFFTGDAKVELRGEHLILEGIWFKDGNRAIQAWKSHGPGLV
AIYGSYNRITACVFDCFDEANSAYITTSLTEDGKVPQHCRIDHCSFTDKITFDQVINLNNTAR
AIKDGSVGGPAMYHRVDHCFFSNPQKPGNAGGGIRIGYYRNDIGRCLVDSNLFMRQDSEA
EIITSKSQENVYYGNTYLNCQGTMNFRHGDHQVAINNFYIGNDQRFGYGGMFVWGSRHVI
ACNYFELSETIKSRGNAALYLNPGAMASEHALAFDMLIANNAFINVNGYAIHFNPLDERR
KEYCAANRLKFETPHQLMLKGNLFFKDKPYVYPFFKDDYFIAGKNSWTGNVALGVEKGIP
VNISANRSAYKPVKIKDIQPIEGIALDLNALISKGITGKPLSWDEVRPYWLKEMPGTYALTAR
LSADRAAKFKAVIKRNKEH
11111111111111111111111110000000000000000000000000000000000000000000000000000000000
00000000000000000000000000000000000000000000000000000000000000000000000000000000000
00000000000000000000000000000000000000000000000000000000000000000000000000000000000

00000000000000000000000000000000000000000000000000000000000000000000000000000000
00000000000000000000000000000000000000000000000000000000000000000000000000000000
00000000000000000000000000000000000000000000000000000000000000000000000000000000
00000000
>Q6F6A2_3umv_0_505
MPPTSVSPPRTAPGPANPSPAHPSRVRVIHPGGGKPGGPVVYWMLRDQRLADNWALLHA
AGLAAASASPLAVAFALFPRPFLLSARRRQLGFLLRGLRRLAADAAARHLPFFLFTGGPAEI
PALVRRLGASTLVADFSPLRPVREALDAVVGDLRREAPGVAVHQVDAHNVVPVWTASAK
MEYSAKTFRGKVSKVMDEYLVEFPELPAVVPWDREQPEGVDWDALIARVCSEAENVPEID
WCEPGEEAAIEALLGSKDGFLTKRIKSYETDRNDPTKPRALSGLSPYLHFGHISAQRCALEA
KKCRHLSPKSVDAFLEELVVRRELADNFCYYQPQYDSLSGAWEWARKTLMDHAADKREHI
YTREQLENAKTHDPLWNASQLEMVHHGKMHGFMRMYWAKKILEWTSGPEEALSTAIYL
NDKYEIDGRDPSGYVGCMWSICGLHDQGWKERPVFGKIRYMNYAGCKRKFDVDAYISYV
KRLAGQSKKRNAEESPNPVVKLSKSQH
11111111111111111100000000000000000000000000000000000000000000000000000000000000
00000000000000000000000000000000000000000000000000000000000000000000000000000000
00000000000000000000000000000000000000000000000000000000000000000000000000000000
00000000000000000000000000000000000000000000000000000000000000000000000000000000
00000000000000000000000000000000000000000000000000000000000000000000000000000000
00000000000000000000000000000000000000000000000000000000000000000000000011111111
11111111
>Q8TWB5_3f8t_0_505
MFRLGTTPRATTYDPDARIGEVASRFGLPTRVLIEIVRTESFQRSLRRVTSGKPVVLDLRELDS
DLASWIATHARLVEPALRELVRTVAPDVEPRVRFRGLPHRFRRVERIRPMDGALISIEGVVRE
VRGAERLEHAIVDTGSELVAVRLHGHRLGPGLRVEILGIVRSATLDALEVHKKDPIPEVHPD
PAELEEFRELADKDPLTTFARAIAPLPGAEEVGKMLALQLFSCVGKNSERLHVLLAGYPVV
CSEILHHVLDHLAPRGVYVDLRRTELTDLTAVLKEDRGWALRAGAAVLADGGILAVDHLE
GAPEPHRWALMEAMDKGTVTVDGIALNARCAVLAAINPGEQWPSDPPIARIDLDQDFLS
HFDLIAFLGVDPRPGEPEEQDTEVPSYTLLRRYLLYAIREHPKPELTEEARKRLEHWYETRRE
EVEERLGMGLPTLPVTRRQLESVERLAKAHARMRLSDDVEPEDVDIAAELVDWYLETAMQ
IPGGDEIRISSLKP
11111111111111111000000000000000000000000000000000000000000000000000000000000000
00000000000000000000000000000000000000000000000000000000000000000000000000000000
00000000000000000000000000000000000000000000000000000000000000000000000000000000
00000000000000000000000000000000000000000000000000000000000000000000000000000000
00000000000000000110000000000000000000000000011111111111110000000000000000000000
00000000000000000000000000000000000000000000000000000000000000000000000000111111
11111111
>B3EY95_4eu8_0_504
MTERIRNVALRSKVCPAETASELIKHGDVVGTSGFTGAGYPKEVPKALAQRMEAAHDRGE
KYQISLITGASTGPQLDGELAKANGVYFRSPFNTDATMRNRINAGETEYFDNHLGQVAGR
AVQGNYGKFNIALVEATAITEDGGIVPTSSVGNSQTFLNLAEKVIIEVNEWQNPMLEGIHDI
WDGNVSGVPTRDIVPIVRADQRVGGPVLRVNPDKIAAIVRTNDRDRNAPFAAPDETAKAI
AGYLLDFFGHEVKQNRLPPSLLPLQSGVGNVANAVLEGLKEGPFENLVGYSEVIQDGMLA
MLDSGRMRIASASSFSLSPEAAEEINNRMDFFRSKIILRQQDVSNSPGIIRRLGCIAMNGMIE
ADIYGNVNSTRVMGSKMMNGIGGSGDFARSSYLSIFLSPSTAKGGKISAIVPMAAHVDHIM
QDAQIFVTEQGLADLRGLSPVQRAREIISKCAHPDYRPMLQDYFDRALKNSFGKHTPHLLT
EALSWHQRFIDTGTMLPS
10000000000000000000000000000000000000000000000000000000000000000000000000000000
00000000000000000000000000000000000000000000000000000000000000000000000000000000
00000000000000000000000000000000000000000000000000000000000000000000000000000000

00000000000000000000000000000000000000000000000000000000000000000000000000000000000
00000000000000000000000000000000000000000000000000000000000000000000000000000000000
00000000000000000000000000000000000000000000000000000000000000000000000000000000000
0000000
>O09496_2x3h_0_504
MAKLTKPKTEGILHKGQSLYEYLDARVLTSKPFGAAGDATTDDTEVIAASLNSQKAVTISDG
VFSSSGINSNYCNLDGRGSGVLSHRSSTGNYLVFNNPRTGRLSNITVESNKATDTTQGQQVS
LAGGSDVTVSDVNFSNVKGTGFSLIAYPNDAPPDGLMIKGIRGSYSGYATNKAAGCVLADS
SVNSLIDNVIAKNYPQFGAVELKGTASYNIVSNVIGADCQHVTYNGTEGPIAPSNNLIKGV
MANNPKYAAVVAGKGSTNLISDVLVDYSTSDARQAHGVTVEGSDNVINNVLMSGCDGTN
SLGQRQTATIARFIGTANNNYASVFPSYSATGVITFESGSTRNFVEVKHPGRRNDLLSSASTID
GAATIDGTSNSNVVHAPALGQYIGSMSGRFEWRIKSMSLPSGVLTSADKYRMLGDGAVSLA
VGGGTSSQVRLFTSDGTSRTVSLTNGNVRLSTSSTGYLQLGADAMTPDSTGTYALGSASRA
WSGGFTQAAFTVTS
11111100000000000000000000000000000000000000000000000000000000000000000000000000000
00000000000000000000000000000000000000000000000000000000000000000000000000000000000
00000000000000000000000000000000000000000000000000000000000000000000000000000000000
00000000000000000000000000000000000000000000000000000000000000000000000000000000000
00000000000000000000000000000000000000000000000000000000000000000000000000000000000
00000000000000000000000000000000000000000000000000000000000000000000000000000000000
0000001
>Q4FVQ2_3hn7_0_504
MHIHILGICGTFMGSLALLARALGHTVTGSDANIYPPMSTQLEQAGVTIEEGYLIAHLQPAP
DLVVVGNAMKRGMDVIEYMLDTGLRYTSGPQFLSEQVLQSRHVIAVAGTHGKTTTTTMLA
WILHYAGIDAGFLIGGVPLVNTTDTNLQQVFAHSSYLGTEKDDSDNSVNTGYFVIEADEYD
SAFFDKRSKFVHYRPRTAILNNLEFDHADIFADLDAIQTQFHHMVRMIPSTGKIIMPAATISL
EDTLAKGVWTPIWRTSVIDSTISSVRREDSPLENSQAENSSDWQAELISADGSQFTVSFNDN
KEATALVNWSMSGLHNVNNALVAIAAAYNIGVSVKTACAALSAFAGIKRRMELIGDVNDI
LVFDDFAHHPTAITTTLDGAKKKLADRRLWAIIEPRSNTMKMGIHQDSLAQSATLADHTLW
YEPTGLEWGLKEVIDNATIANPSIGSQQVLSSVDDIIKHICTHAKAGDAIVIMSNGGFEGIHQ
RLLTALGNIVAIL
00000000000000000000000000000000000000000000000000000000000000000000000000000000000
00000000000000000000000000000000000000000000000000000000000000000000000000000000000
00000000000000000000000000000000000000000001111110000000000000000000000000000000000
00000000000000001111111111111111111000000000000000000000000000000000000000000000000
00000000000000000000000000000000000000000000000000000000000000000000000000000000000
00000000000000000000000000000000000000000000000000000000000000000000000000000000000
0000001
>Q92WS7_3v4c_0_504
MIFTPKGKHLVAGEWLDGAGTFASAPAHGPAHDFAVGTVELVNRACEAAEEAFWTYGYSS
RKERAAFLRAIADEIEARAEAITEIGSQETGLPEARLNGERGRTTGQLRLFADHIEKGDYLDR
RVDAAMPERQPAPRQEIRLVQRPVGPVAVFGASNFPLAFSTAGGDTAAALAAGCPVVVKG
HSAHPGTGEIVAEAVDAAIRKTGVHPGVFSLIQGGSRDVGHALVQHPHIKAVGFTGSLAGG
RALFDLCAARPEPIPFFGELGSVNPMFLLPEALKARAETLGQGWAGSLTMGAGQFCTNPGI
AVVIEGADADRFTTAAVEALAKVAPQTMLTDGIAKAYRDGQARFATRNAVKPLLATESSGR
DASPNLFETTGAQFLADHALGEEVFGPLGLVVRVGSPAEMEELARGFQGQLTATIHMDAG
DLETARRLRPVLERKAGRVLVNGFPTGVEVVDSMVHGGPYPASTNFGATSVGTMSIRRFLR
PVAYQNMPEDLLPEDFLG
00000000000000000000000000000000000000000000000000000000000000000000000000000000000
00000000000000000000000000000000000000000000000000000000000000000000000000000000000
00000000000000000000000000000000000000000000000000000000000000000000000000000000000

00000000000000000000000000000000000000000000000000000000000000000000000000000000000000000000
00000000000000000000000000000000000000000000000000000000000000000000000000000000000000000000
00000000000000000000000000000000000000000000000000000000000000000000000000000000000000000000
0000011
>P32368_3lwt_0_503
MTGPIVYVQNADGIFFKLAEGKGTNDAVIHLANQDQGVRVLGAEEFPVQGEVVKIASLMG
FIKLKLNRYAIIANTVEETGRFNGHVFYRVLQHSIVSTKFNSRIDSEEAEYIKLLELHLKNSTF
YFSYTYDLTNSLQRNEKVGPAASWKTADERFFWNHYLTEDLRNFAHQDPRIDSFIQPVIYG
YAKTVDAVLNATPIVLGLITRRSIFRAGTRYFRRGVDKDGNVGNFNETEQILLAENPESEKIH
VFSFLQTRGSVPIYWAEINNLKYKPNLVLGENSLDATKKHFDQQKELYGDNYLVNLVNQK
GHELPVKEGYESVVHALNDPKIHYVYFDFHHECRKMQWHRVKLLIDHLEKLGLSNEDFF
HKVIDSNGNTVEIVNEQHSVVRTNCMDCLDRTNVVQSVLAQWVLQKEFESADVVATGST
WEDNAPLLTSYQNLWADNADAVSVAYSGTGALKTDFTRTGKRTRLGAFNDFLNSASRYYQ
NNWTDGPRQDSYDLFLGG
00000000000000000000000000000000000000000000000000000000000000000000000000000000000000000000
00000000000000000000000000000000000000000000000000000000000000000000000000000000000000000000
00000000000000000000000000000000000000000000000000000000000000000000000000000000000000000000
00000000000000000000000000000000000000000000000000000000000000000000000000000000000000000000
00000000000000000000000000000000000000000000000000000000000000000000000000000000000000000000
00000000000000000000000000000000000000000000000000111111111111111111111111111111111111111111
111111
>Q70AC7_1rqb_1_504
SPREIEVSEPREVGITELVLRDAHQSLMATRMAMEDMVGACADIDAAGYWSVECWGGAT
YDSCIRFLNEDPWERLRTFRKLMPNSRLQMLLRGQNLLGYRHYNDEVVDRFVDKSAENG
MDVFRVFDAMNDPRNMAHAMAAVKKAGKHAQGTICYTISPVHTVEGYVKLAGQLLDM
GADSIALKDMAALLKPQPAYDIIKAIKDTYGQKTQINLHCHSTTGVTEVSLMKAIEAGVDV
VDTAISSMSLGPGHNPTESVAEMLEGTGYTTNLDYDRLHKIRDHFKAIRPKYKKFESKTLVD
TSIFKSQIPGGMLSNMESQLRAQGAEDKMDEVMAEVPRVRKAAGFPPLVTPSSQIVGTQAV
FNVMMGEYKRMTGEFADIMLGYYGASPADRDPKVVKLAEEQSGKKPITQRPADLLPPEWE
KQSKEAATLKGFNGTDEDVLTYALFPQVAPVFFEHRAEGPHSVALTDAQLKAEAEGDEKSL
AVAGPVTYNVNVGGTVREVTVQQA
10000000000000000000000000000000000000000000000000000000000000000000000000000000000000000000
00000000000000000000000000000000000000000000000000000000000000000000000000000000000000000000
00000000000000000000000000000000000000000000000000000000000000000000000000000000000000000000
00000000000000000000000000000000000000000000000000000000000000000000000000000000000000000000
00000000000000000000000000000000000000000000000000000000000000000000000000000000000000000000
00000000000000000000000000000000000000000000000000000000000000000001111111111111111111111111
111111
>P33590_1uiv_22_523
AAPDEITTAWPVNVGPLNPHLYTPNQMFAQSMVYEPLVKYQADGSVIPWLAKSWTHSED
GKTWTFTLRDDVKFSNGEPFDAEAAAENFRAVLDNRQRHAWLELANQIVDVKALSKTEL
QITLKSAYYPFLQELALPRPFRFIAPSQFKNHETMNGIKAPIGTGPWILQESKLNQYDVFVRN
ENYWGEKPAIKKITFNVIPDPTTRAVAFETGDIDLLYGNEGLLPLDTFARFSQNPAYHTQLSQ
PIETVMLALNTAKAPTNELAVREALNYAVNKKSLIDNALYGTQQVADTLFAPSVPYANLGL
KPSQYDPQKAKALLEKAGWTLPAGKDIREKNGQPLRIELSFIGTDALSKSMAEIIQADMRQI
GADVSLIGEEESSIYARQRDGRFGMIFHRTWGAPYDPHAFLSSMRVPSHADFQAQQGLAD
KPLIDKEIGEVLATHDETQRQALYRDILTRLHDEAVYLPISYISMMVVSKPELGNIPYAPIATEI
PFEQIKPVKP
11100000000000000000000000000000000000000000000000000000000000000000000000000000000000000000
00000000000000000000000000000000000000000000000000000000000000000000000000000000000000000000
00000000000000000000000000000000000000000000000000000000000000000000000000000000000000000000

00000000000000000000000000000000000000000000000000000000000000000000000000000000000
00000000000000000000000000000000000000000000000000000000000000000000000000000000000
00000000000000000000000000000000000000000000000000000000000000000000000000000000000
0011
>P13134_2id4_113_612
APPMDSSLLPVKEAEDKLSINDPLFERQWHLVNPSFPGSDINVLDLWYNNITGAGVVAAIV
DDGLDYENEDLKDNFCAEGSWDFNDNTNLPKPRLSDDYHGTRCAGEIAAKKGNNFCGV
GVGYNAKISGIRILSGDITTEDEAASLIYGLDVNDIYSCSWGPADDGRHLQGPSDLVKKALV
KGVTEGRDSKGAIYVFASGNGGTRGDNCNYDGYTNSIYSITIGAIDHKDLHPPYSEGCSAV
MAVTYSSGSGEYIHSSDINGRCSNSHGGTSAAAPLAAGVYTLLLEANPNLTWRDVQYLSILS
AVGLEKNADGDWRDSAMGKKYSHRYGFGKIDAHKLIEMSKTWENVNAQTWFYLPTLYV
SQSTNSTEETLESVITISEKSLQDANFKRIEHVTVTVDIDTEIRGTTTVDLISPAGIISNLGVVRP
RDVSSEGFKDWTFMSVAHWGENGVGDWKIKVKTTENGHRIDFHSWRLKLFGESIDSSKTE
TFVFGNDKEEVE
11111111000000000000000000000000000000000000000000000000000000000000000000000000000
00000000000000000000000000000000000000000000000000000000000000000000000000000000000
00000000000000000000000000000000000000000000000000000000000000000000000000000000000
00000000000000000000000000000000000000000000000000000000000000000000000000000000000
00000000000000000000000000000000000000000000000000000000000000000000000000000000000
00000000000000000000000000000000000000000000000000000000000000000000000001111111111
11
>Q7NWW7_3hz6_1_500
AFYIATFDIGTTEVKAALADRDGGLHFQRSIALETYGDGNGPVEQDAGDWYDAVQRIASS
WWQSGVDARRVSAIVLSGQMQNFLPLDQDHEPLHRAVLYSDKRPLKEAEEINARHGADN
LWSALENPMTAASILPKLVFWRASFPQAFGRLRHVVLGAKDYVVLRLTGRHATDRTNAST
TGLYRPKDDAWHVELLADYGFSLDLMPRLLEPGEQVGGVSALAARQTGFVSGTPVLCGLG
DAGAATLGVGVLDDEDAYLHLGTTGWLARLTQTDPVGDMPVGTIFRLAGIIAGKTLQVAP
VLNAGNILQWALTLVGHRPGEDCAEYFHMAAAEVQGVTVPDGLLFVPYLHAERCPVELP
APRGALLGVTGATTRAQILLAVLEGAALSLRWCAELLGMEKVGLLKVVGGGARSEAWLR
MIADNLNVSLLVKPDAHLHPLRGLAALAAVELEWSHSIQDFLREADLREPASNILHPQPCD
EGRRRRKFERFKQCVETLGRLD
00000000000000000000000000000000000000000000000000000000000000000000000000000000000
00000000000000000000000000000000000000000000000000000000000000000000000000000000000
00000000000000000000000000000000000000000000000000000000000000000000000000000000000
00000000000000000000000000000000000000000000000000000000000000000000000000000000000
00000000000000000000000000000000000000000000000000000000000000000000000000000000000
00000000000000000000000000000000000000000000000000111000000000000000000000000000000
11
>Q54276_1e15_0_498
MSTRKAVIGYYFIPTNQINNYTETDTSVVPFPVSNITPAKAKQLTHINFSFLDINSNLECAWD
PATNDAKARDVVNRLTALKAHNPSLRIMFSIGGWYYSNDLGVSHANYVNAVKTPASRAKF
AQSCVRIMKDYGFDGVDIDWEYPQAAEVDGFIAALQEIRTLLNQQTITDGRQALPYQLTIA
GAGGAFFLSRYYSKLAQIVAPLDYINLMTYDLAGPWEKVTNHQAALFGDAAGPTFYNALR
EANLGWSWEELTRAFPSPFSLTVDAAVQQHLMMEGVPSAKIVMGVPFYGRAFKGVSGGN
GGQYSSHSTPGEDPYPSTDYWLVGCEECVRDKDPRIASYRQLEQMLQGNYGYQRLWNDKT
KTPYLYHAQNGLFVTYDDAESFKYKAKYIKQQQLGGVMFWHLGQDNRNGDLLAALDRY
FNAADYDDSQLDMGTGLRYTGVGPGNLPIMTAPAYVPGTTYAQGALVSYQGYVWQTKW
GYITSAPGSDSAWLKVGRVA
11000000000000000000000000000000000000000000000000000000000000000000000000000000000
00000000000000000000000000000000000000000000000000000000000000000000000000000000000
00000000000000000000000000000000000000000000000000000000000000000000000000000000000

00000000000000000000000000000000000000000000000000000000000000000000000000000000000
00000000000000000000000000000000000000000000000000000000000000000000000000000000000
00000000000000000000000000000000000000000000000000000000000000000000000000000000000
0
>Q9M426_3kal_0_498
MSQPLTTNSVLVEEAAADGDSSAAAPPLFDYHRIDQKLLQNIVYDALVWSTLNCLLVGDKS
VQRSGRVPGVGLVHLPLSLLPGPFPESHWKQGCELAPIFNELVDRVSLDGKFLQESLSRTKN
ADEFTSRLLDIHSKMLQINKKEDIRMGIVRSDYMIDEKTKSLLQIEMNTISTSFALIGCLMTG
LHKSLLSQYGKFLGLNSNRVPANNAVDQSAEALAKAWSEYNNPRAAILVVVQVEERNMY
EQHYISALLREKHHIRSIRKTLTEIDQEGKILPDGTLSVDGQAISVVYFRAGYTPKDYPSESEW
RARLLMEQSSAIKCPTISYHLVGTKKIQQELAKPGVLERFVENKDHIAKLRACFAGLWSLED
SDIVKKAIENPELFVMKPQREGGGNNIYGDELRETLLKLQEAGSQEDAAYILMQRIFPATSP
AILVRDGNWDTGHVISEAGIFGTYLRNKDKIIINNESGYMVRTKISSSYEGGVLPGFGVVDT
VYLT
11111111111111111111111100000000000000000000000000000000000000000000000000000000000
00000000000000000000000000000000000000000000000000000000000000000000000000000000000
00000000000000000000000000000000000000000000000000000000000000000000000000000000000
00000000000000000000000000000000000000000000000000000000000000000000000000000000000
00000000000000000000000000000000000000000000000000000000000000000000000000000001111
10000000000000000000000000000000000000000000000000000000000000000000000000000000000
0
>Q8A653_3fdh_22_519
SEDTMDRINKDHDHTTSVASRFILADVITSTAFSNASGDINTYASSYIEYEVGVDNQLYYAEV
RENEPSSSSTFNNSWNGIYSSLKNARIIIDQCGEGGRDHGNDVTRGMAEVMAAYNCALIA
DFFGDAPCSQAALVDEKGSPVYMTPKMDTQQEIYTQIISYLDDAIANLQKEDLADVTEQDF
LYAGDADKWLKFAYGLKARYTMRLINRSSNKSADYEKVLDYVSKSFTSADDQAAFDIYDSN
NINPFYGFYNSRAGFGASTSLGTKLLAYNDPRANRAFFTPIVDKKRSQVAANDPSLVPAPN
GSPDQSTSKYGISAFVYAKTAPTLLMSYHELMFLKAEALCRLNRDAEDALKEAVVAGLLNA
ENSISIAIKELGSGLNTNSSEVITETSAGKYFDDVVKAKYAANPLQETMIQKYLAMWGASGE
ATETYNDFRRMKGLNENFITLTNPNNSSKFPLRYPYGNSDTAANPEVKAAYGNGDYVYSEP
VWWAGGSR
11111111111111110000000000000000000000000000000000000000000000000000000000000000000
00000000000000000000000000000000000000000000000000001111111111000000000000000000000
00000000000000000000000000000000000000000000000000000000000000000000000000000000000
00000000000000000000000000000000000000000000000000000000000000000000000000000000000
00000000000000000000000000000000000000000000000000000000000000000000000000000000000
00000000000000000000000000000000000000000000000000000000000000000000000000000000000
>Q8FBB1_3ive_21_518
AKDVTIIYTNDLHAHVEPYKVPWIADGKRDIGGWANITTLVKQEKAKNKATWFFDAGDYF
TGPYISSLTKGKAIIDIMNTMPFDAVTIGNHEFDHGWDNTLLQLSQAKFPIVQGNIFYQNSS
KSFWDKPYTIIEKDGVKIGVIGLHGVFAFNDTVSAATRVGIEARDEIKWLQRYIDELKGKVD
LTVALIHEGVPARQSSMGGTDVRRALDKDIQTASQVKGLDILITGHAHVGTPEPIKVGNTLI
LSTDSGGIDVGKLVLDYKEKPHNFTVKNFELKTIYADEWKPDQQTKQVIDGWNKKLDEVV
QQTVAQSPVELKRAYGESASLGNLAADALLAAAGKNTQLALTNSGGIRNEIPAGAITMGG
VISTFPFPNELVTMELTGKQLRSLMEHGASLSNGVLQVSKGLEMKYDSSKPVGQRVITLTLN
GKPIEDATVYHIATQSFLADGGDGFTAFTEGKARNITGGYYVYHAVVDYFKAGNTITDEQL
NGMRVKDIK
00000000000000000000000000000000000000000000000000000000000000000000000000000000000
00000000000000000000000000000000000000000000000000000000000000000000000000000000000
00000000000000000000000000000000000111000000000000000000000000000000000000000000000
00000000000000000000000000000000000000000000000000000000000000000000000000000000000

00000000000000000000000000000000000000000000000000000000000000000000000000000000000
00000000000000000000000000000000000000000000000000000000000000000000000000000000000
>P33197_2d1c_0_495
MPLITTETGKKMHVLEDGRKLITVIPGDGIGPECVEATLKVLEAAKAPLAYEVREAGASVFR
RGIASGVPQETIESIRKTRVVLKGPLETPVGYGEKSANVTLRKLFETYANVRPVREFPNVPTP
YAGRGIDLVVVRENVEDLYAGIEHMQTPSVAQTLKLISWKGSEKIVRFAFELARAEGRKKVH
CATKSNIMKLAEGTLKRAFEQVAQEYPDIEAVHIIVDNAAHQLVKRPEQFEVIVTTNMNGD
ILSDLTSGLIGGLGFAPSANIGNEVAIFEAVHGSAPKYAGKNVINPTAVLLSAVMMLRYLEEF
ATADLIENALLYTLEEGRVLTGDVVGYDRGAKTTEYTEAIIQNLGKTPRKTQVRGYKPFRLP
QVDGAIAPIVPRSRRVVGVDVFVETNLLPEALGKALEDLAAGTPFRLKMISNRGTQVYPPT
GGLTDLVDHYRCRFLYTGEGEAKDPEILDLVSRVASRFRWMHLEKLQEFDGEPGFTKAQGE
D
10000000000000000000000000000000000000000000000000000000000000000000000000000000000
00000000000000000000000000000000000000000000000000000000000000000000000000000000000
00000000000000000000000000000000000000000000000000000000000000000000000000000000000
00000000000000000000000000000000000000000000000000000000000000000000000000000000000
00000000000000000000000000000000000000000000000000000000000000000000000000000000000
000000000000000000000000000000000000000000000000000000000000000000000000000000000
>Q66GI4_4g24_76_571
ASPSENLSRKAKKKAIQQSPEALLKQKLDMCSKKGDVLEALRLYDEARRNGVQLSQYHYN
VLLYVCSLAEAATESSPNPGLSRGFDIFKQMIVDKVVPNEATFTNGARLAVAKDDPEMAFD
MVKQMKAFGIQPRLRSYGPALFGFCRKGDADKAYEVDAHMVESEVVPEEPELAALLKVS
MDTKNADKVYKTLQRLRDLVRQVSKSTFDMIEEWFKSEVATKTGVKKWDVKKIRDAVVSG
GGGWHGQGWLGTGKWNVKRTEMDENGVCKCCKEKLVCIDINPVETETFAASLTRLACER
EVKANFNQFQEWLERHGPFDAVIDGANMGLVNQRSFSFFQLNNTVQRCQQISPSKRLPLVI
LHKSRVNGGPATYPKNRALLEKWKNAGALYATPPGSNDDWYWLYAAVSCKCLLVTNDEM
RDHLFQLLGNSFFPRWKEKHQVRISVTREDGLKLNMPPPYSIVIQESEDGTWHVPMSVEDD
LQTSRQWLCAKRSKTP
11111111111111111100000000000000000000000000000000000000000000000000000000000000000
00000000000000000000000000000000000000000000000000000000000000000000000000000000000
00000000000000000000000000000000000000000000000000000000000000000000000000000000000
00000000000000000000000000000000000000000000000000000000000000000000000000000000000
00000000000000000000000000000000000000000000000000000000000000000000000000000000000
000000000000000000000000000000000000000000000000000000000000000000000000000000011
>Q8VW87_3vss_36_531
QSGLQDGPEPTIHTQQAYAPEDDFTAKWTRADARQLQRMSDPTAPSRENSMPASVTMPTV
PQDFPDMSNEQVWVWDTWPLTDEDANQYSVNGWEIIFSLVADRNLGFDDRHVFAKIGYF
YRPAGVPAAERPENGGWTYGGLVFKEGVTGQIFEDQSFSHQTQWSGSARVSKNGEIKLFFT
DVAFYRNSDGTNIKPYDPRIALSVGKVKANKKGVTLTGFNKVTDLLQADGTYYQTGAQNE
FFNFRDPFTFEDPAHPGETFMVFEGNSAMQRETATCNEADLGYRQGDPYAETVDDVNASG
ATYQIGNVGLAKAKNKQLTEWEFLPPILSANCVTDQTERPQIYFKDGKSYLFTISHRGTFAA
GLDGPEGVYGFVGDGIRSDYQPLNGGSGLALGNPTNLNFLGGQPFAPDFNQHPGHFQAYS
HYVMPGGLVQSFIDTIGTHDDFVRGGTLAPTVKMDIGVGGDPTKTAVDYSYGSEGLGGWA
DIPANKHLFTNGKF
11000000000000000000000000000000000000000000000000000000000000000000000000000000000
00000000000000000000000000000000000000000000000000000000000000000000000000000000000
00000000000000000000000000000000000000000000000000000000000000000000000000000000000
00000000000000000000000000000000000000000000000000000000000000000000000000000000000
00000000000000000000000000000000000000000000000000000000000000000000000000000000000
000000000000000000000000000000000000000000000000000000000000000000000000000000000
>P46882_2vvm_0_494

MTSRDGYQWTPETGLTQGVPSLGVISPPTNIEDTDKDGPWDVIVIGGGYCGLTATRDLTVA
GFKTLLLEARDRIGGRSWSSNIDGYPYEMGGTWVHWHQSHVWREITRYKMHNALSPSFN
FSRGVNHFQLRTNPTTSTYMTHEAEDELLRSALHKFTNVDGTNGRTVLPFPHDMFYVPEFR
KYDEMSYSERIDQIRDELSLNERSSLEAFILLCSGGTLENSSFGEFLHWWAMSGYTYQGCMD
CLISYKFKDGQSAFARRFWEEAAGTGRLGYVFGCPVRSVVNERDAARVTARDGREFAAKR
LVCTIPLNVLSTIQFSPALSTERISAMQAGHVNMCTKVHAEVDNKDMRSWTGIAYPFNKLC
YAIGDGTTPAGNTHLVCFGTDANHIQPDEDVRETLKAVGQLAPGTFGVKRLVFHNWVKD
EFAKGAWFFSRPGMVSECLQGLREKHRGVVFANSDWALGWRSFIDGAIEEGTRAARVVLE
ELGTKREVKARL
00000000000000000000000000000001111110000000000000000000000000000000000000000000000
00000000000000000000000000000000000000000000000000000000000000000000000000000000000
00000000000000000000000000000000000000000000000000000000000000000000000000000000000
00000000000000000000000000000000000000000000000000000000000000000000000000000000000
00000000000000000000000000000000000000000000000000000000000000000000000000000000000
00000000000000000000000000000000000000000000000000000000000000000000000011111111
>Q5SJM0_3oep_0_493
MVRGFYLRFGEGVSEEANRRALALAEALLRAPPPGLLDAVPAYGVLYLEYDPRRLSRGRLL
RLLKGLPQERAEEGRVVEIPVRYDGEDLPEVASRLGLSLEAVKALHQKPLYRVYALGFTPGF
PFLAEVEPALRLPRKPHPRPRVPAHAVAVAGVQTGIYPLPSPGGWNLLGTSLVAVYDPHRET
PFLLRPGDRVRFLEAEGPTPPEPRPLELLPEEPRLPALLVEEPGLMDLVVDGGRFLGGHLGLA
RSGPLDAPSARLANRLVGNGAGAPLLEFAYKGPVLTALRDLVAAFAGYGFVALLEGEEIPPG
QSFLWPRGKTLRFRPRGPGVRGYLAVAGGLEVRPFLGSASPDLRGRIGRPLWAGDVLGLEA
LRPVRPGRAFPQRPLPEAFRLRLLPGPQFAGEAFRALCSGPFRVARADRVGVELLGPEVPGG
EGLSEPTPLGGVQVPPSGRPLVLLADKGSLGGYAKPALVDPRDLWLLGQARPGVEIHFTSG
00000000000000000000000000000000000000000000000000000000000000000000111111100000000
00000000000000000000000000000000000000000000000000000000000000000000000000000000000
00000000000000000000000000000000000000000000000000000000000000000000000000000000000
00000000000000000000000000000000000000000000000000000000000000000000000000000000000
00000000000000000000000000000000000000000000000000000000000000000000000000000000000
0000000000000000000000000000000000000000000000000000000000000000000000000000001
>Q87NC6_2qma_464_957
IAAGGSHVEAAPQEEWKKHFIHTGELGSAEFASVMSHTTSAMKSVFEQVNAPYSGMDPKA
LEDAINAVDLDNKNAPLKSVIDDVAELVAKNAIFTQHPDCIAHLHTPPLMPAVAAEAMIA
ALNQSMDSWDQASSATYVEQKVVNWLCDKYDLSEKADGIFTSGGTQSNQMGLMLARDW
IADKLSGHSIQKLGLPDYADKLRIVCSKKSHFTVQKSASWMGLGEKAVMTVDANADGTM
DITKLDEVIAQAKAEGLIPFAIVGTAGTTDHGAIDDLDFIADMAVKHDMWMHVDGAYGG
ALILSSHKSRLKGVERAHSISVDFHKLFYQTISCGALLVNDKSNFKFLLHHADYLNREHDEL
PNLVDKSIATTKRFDALKVFMTMQNVGPKALGDMYDHLLAQTLEVADMIRTNDQFELLA
EPSLSTVLFRATHETADLDELNKALRLEALTRGIAVLGETIVDGKTALKFTILNPCLTTSDFES
LLSKINMLAVELV
11111111110000000000000000000000000000000000000000000000000000000000000000000000000
00000000000000000000000000000000000000000000000000000000000000000000000000000000000
00000000000000000000000000000000000000000000000000000000000000000000000000000000000
00000000000000000000000000000000000000000000000000000000000000000000000000000000000
00000000000000111111111111111111110000000000000000000000000000000000000000000000000
0000000000000000000000000000000000000000000000000000000000000000000000000000001
>P13398_3a2p_0_492
MSKVDLWQDATAQAELVRSGEISRTELLEATIAHVQAVNPEINAVIIPLFEKARRESELASGP
FAGVPYLLKDLTVVSQGDINTSSIKGMKESGYRADHDAYFVQRMRAAGFVLLGKTNTPEM
GNQVTTEPEAWGATRNPWNLGRSVGGSSGGSGAAVAAALSPVAHGNDAAGSVRIPASVC
GVVGLKPTRGRISPGPLVTDSDNVAGAAHEGLFARSVRDIAALLDVVSGHRPGDTFCAPTA

SRPYAQGISENPGSLRVGVLTHNPVGDFALDPECAAAARGAAAALAALGHDVNDAYPEA
LGDRSFLKDYSTICDVAIAREIERNGELIGRPLTEDDVEWTSWEMVKRADQVTGRAFAACV
DELRYYAGKVERWWEAGWDLLILPTVTRQTPEIGELMLAKGTDLEGRQSAFISGSLQMLAF
TVPFNVSGQPAISLPIGMSSDGMPIGVQIVAAYGREDLLLQVAAQLEGALPWVARRPQLLN
PSRKIPAA
11000000000000000000000000000000000000000000000000000000000000000000000000000000000
00000000000000000000000000000000000000000000000000000000000000000000000000000000000
00000000000000000000000000000000000000000000000000000000000000000000000000000000000
00000000000000000000000000000000000000000000000000000000000000000000000000000000000
00000000000000000000000000000000000000000000000000000000000000000000000000000000000
000000000000000000000000000000000000000000000000000000000000000000000011111111
>Q9Y600_2jis_0_492
MADSEALPSLAGDPVAVEALLRAVFGVVVDEAIQKGTSVSQKVCEWKEPEELKQLLDLELR
SQGESQKQILERCRAVIRYSVKTGHPRFFNQLFSGLDPHALAGRIITESLNTSQYTYEIAPVFV
LMEEEVLRKLRALVGWSSGDGIFCPGGSISNMYAVNLARYQRYPDCKQRGLRTLPPLALFTS
KECHYSIQKGAAFLGLGTDSVRVVKADERGKMVPEDLERQIGMAEAEGAVPFLVSATSGTT
VLGAFDPLEAIADVCQRHGLWLHVDAAWGGSVLLSQTHRHLLDGIQRADSVAWNPHKL
LAAGLQCSALLLQDTSNLLKRCHGSQASYLFQQDKFYDVALDTGDKVVQCGRRVDCLKL
WLMWKAQGDQGLERRIDQAFVLARYLVEEMKKREGFELVMEPEFVNVCFWFVPPSLRGK
QESPDYHERLSKVAPVLKERMVKEGSMMIGYQPHGTRGNFFRVVVANSALTCADMDFLL
NELERLGQDL
11111100000000000000000000000000000000000000000000000000000000000000000000000000000
00000000000000000000000000000000000000000000000000000000000000000000000000000000000
00000000000000000000000000000000000000000000000000000000000000000000000000000000000
00000000000000000000000000000000000000000000000000000000000000000000000000000000000
00000000000000000000000000000000000000000000000000000000000000000000000000000000000
000000000000000000000000000000000000000000000000000000000000000000000000000000
>A0Q436_2d1g_22_513
DVNNSKPNDYGTLVKIEQKLFNNANTLKTTTPIKHVVIIFQENNSFDRYFGMYPNAKNPEG
EPKFVAKENTPNVNGLTKQLLENNPNTKNPYRLDRNFQPCSQNHEYHQEISSFNGGLMN
KFVEHGGHDNDTYKQNCDGQVMGYYDGNTVTALWNYAQNFALNDNTFGTTFGPSTPG
ALNLVAGANGPAMSPSGNLENIENNYIIDDPNPYYDDCSYGTSKSGDTNTAVAKITDGYNI
GHYLTQKGITWGWFQGGFKPTSYSGKTAICDAMSTNKFGVKSRDYIPHHEPFNYWKETSN
PHHLAPSDDKYIGSNDQANHQYDISEFWKALDQNNMPAVSYLKAPGYQDGHGGYSNPL
DEQEWLVNTINRIQQSKDWDSTAIIIIYDDSDGDYDHVYSPKSQFSDIKGRQGYGPRLPMLVI
SPYAKANYVDHSLLNQASVLKFIEYNWGIGSVSKYSNDKYSNNILNMFDFNKEQKTLKLIL
DPKTGLVMDKLN
11100000000001111100000000000000000000000000000000000000000000000000000000000000000
00000000000000000000000000000000000000000000000000000000000000000000000000000000000
00000000000000000000000000000000000000000000000000000000000000000000000000000000000
00000000000000000000000000000000000000000000000000000000000000000000000000000000000
00000000000000000000000000000000000000000000000000000000000000000000000000000000000
00000000000000000000000000000000000000000000000000000000000000000000000001111
>Q7CG15_3go9_7_498
LMVGGLLLAAASNNVQAEALQPDPAWQQGKLDNGFSWQLLATPQRPSDRIELRLIVNTG
SLSENTQEVGFAHLLPRLALMSSASFTPAQLQSLWQQGIDNERPLPPAITSYDFTLYSLSLPN
NRPDLLKDALAWLSDTAGNLAVSEQTVNAALNTATDPIATFPQNIQEPWWRYRLKGSSLIG
HDPGQPVTQPVDVEKLKQFYQQWYTPDAMTLYVVGNVDSRSIAAQISKAFSELKGKRTAP
AAVATLAPLPPEPVSLMNEQAAQDTLSLMWDTPWHPIQDSMALSRYWRSDLAREALFWH
IKQVLEKNNQKNLKLGFDCRVQYQRAQCAIHLNTPVENLTANMTFVARELAALRANGLS
QAEFDALMTQKNDQLSKLFATYARTDTDILMSQRLRSQQSGVVDIAPEQYQKLRQAFLSGL

TLAELNRELKQQLSQDTTLVLMQPKGEPEVNVKALQEIYNGIMAPQTVAEEEVAPAEAVET
APVMPTTAQ
00000001111111100000000000000000000000000000000000000000000000000000000000000000
00000000000000000000000000000000000000000000000000000000000000000000000000000000
00000000000000000000000000000000000000000000000000000000000000000000000000000000
00000000000000000000000000000000000000000000000000000000000000000000000000000000
00000000000000000000000000000000000000000000000000000000000000000000000000000000
00000000000000000000000000000000000000000000000001111111111111111111111111
>Q9NZM3_3jzy_1200_1691
DTMQPIERKRQGYIHELIQTEERYMADLQLVVEVFQKRMAESGFLTEGEMALIFVNWKELI
MSNTKLLKALRVRKKTGGEKMPVQMIGDILAAELSHMQAYIRFCSCQLNGAALLQQKTDE
DTDFKEFLKKLASDPRCKGMPLSSFLLKPMQRITRYPLLIRSILENTPESHADHSSLKLALER
AEELCSQVNEGVREKENSDRLEWIQAHVQCEGLAEQLIFNSLTNCLGPRKLLHSGKLYKTK
SNKELHGFLFNDFLLLTYMVKQFAVSSGSEKLFSSKSNAQFKMYKTPIFLNEVLVKLPTDPSS
DEPVFHISHIDRVYTLRTDNINERTAWVQKIKAASEQYIDTEKKKREKAYQARSQKTSGIGR
LMVHVIEATELKACKPNGKSNPYCEISMGSQSYTTRTIQDTLNPKWNFNCQFFIKDLYQDV
LCLTLFDRDQFSPDDFLGRTEIPVAKIRTEQESKGPMTRRLLLHEVPTGEVWVRFDLQLFE
11111111111111111111111111111111111111111111111111111111111111111111111111111111
11111111111111111111111111111111111111111111111111111111111111111111111111111111
11111111111111111111111111111111111111111111111111111111111111111111111111111111
11111111111111111111111111111111111111111111111111111111111111111111111111111111
11111111111111111111111111111111110000000000000000000000000000000000000000000000
00000000000000000000000000000000000000000000000000000000000000000000000001
>Q13255_3ks9_27_517
LLAGASSQRSVARMDGDVIIGALFSVHHQPPAEKVPERKCGEIREQYGIQRVEAMFHTLDKI
NADPVLLPNITLGSEIRDSCWHSSVALEQSIEFIRDSLISIRDEKDGINRCLPDGQSLPPGRTKK
PIAGVIGPGSSSVAIQVQNLLQLFDIPQIAYSATSIDLSDKTLYKYFLRVVPSDTLQARAMLDI
VKRYNWTYVSAVHTEGNYGESGMDAFKELAAQEGLCIAHSDKIYSNAGEKSFDRLLRKLR
ERLPKARVVVCFCEGMTVRGLLSAMRRLGVVGEFSLIGSDGWADRDEVIEGYEVEANGGIT
IKLQSPEVRSFDDYFLKLRLDTNTRNPWFPEFWQHRFQCRLPGHLLENPNFKRICTGNESLE
ENYVQDSKMGFVINAIYAMAHGLQNMHHALCPGHVGLCDAMKPIDGSKLLDFLIKSSFIG
VSGEEVWFDEKGDAPGRYDIMNLQYTEANRYDYVHVGTWHEGVLNIDDYKIQMNKSG
11111110000000000000000000000000000000000000000000000000000000000000000000000000
00000000000000001111111111111111111111111000000000000000000000000000000000000000
00000000000000000000000000000000000000000000000000000000000000000000000000000000
00000000000000000000000000000000000000000000000000000000000000000000000000000000
00000000000000000000001111100000000000000000000000000000000000000000000000000000
0000000000000000000000000000000000000000000000000000000000000000000111111
>Q99KQ4_2h3b_0_490
MNAAAEAEFNILLATDSYKVTHYKQYPPNTSKVYSYFECREKKTENSKVRKVKYEETVFYG
LQYILNKYLKGKVVTKEKIQEAKEVYREHFQDDVFNERGWNYILEKYDGHLPIEVKAVPEG
SVIPRGNVLFTVENTDPECYWLTNWIETILVQSWYPITVATNSREQKKILAKYLLETSGNLDG
LEYKLHDFGYRGVSSQETAGIGASAHLVNFKGTDTVAGIALIKKYYGTKDPVPGYSVPAAE
HSTITAWGKDHEKDAFEHIVTQFSSVPVSVVSDSYDIYNACEKIWGEDLRHLIVSRSTEAPLII
RPDSGNPLDTVLKVLDILGKKFPVTENSKGYKLLPPYLRVIQGDGVDINTLQEIVEGMKQK
KWSIENVSFGSGGALLQKLTRDLLNCSFKCSYVVTNGLGVNVFKDPVADPNKRSKKGRLSL
HRTPAGNFVTLEEGKGDLEEYGHDLLHTVFKNGKVTKSYSFDEVRKNAQLNIEQDVAPH
11111110000000000000000000000000000000001111111110000000000000000000000000000000
00000000000000000000000000000000000000000000000000000000000000000000000000000000
00000000000000000000000000000000000000000000000000000000000000000000000000000000
00000000000000000000000000000000000000000000000000000000000000000000000000000000

00000000000000000000000000000000000000000000000000000000000000000000000000000000000
0000000000000000000000000000000000000000000000000000000000000000000011111111
>P0A636_3pny_0_489
MTATETVRVRFCPSPTGTPHVGLVRTALFNWAYARHTGGTFVFRIEDTDAQRDSEESYLALL
DALRWLGLDWDEGPEVGGPYGPYRQSQRAEIYRDVLARLLAAGEAYHAFSTPEEVEARHV
AAGRNPKLGYDNFDRHLTDAQRAAYLAEGRQPVVRLRMPDDDLAWNDLVRGPVTFAAG
SVPDFALTRASGDPLYTLVNPCDDALMKITHVLRGEDLLPSTPRQLALHQALIRIGVAERIPK
FAHLPTVLGEGTKKLSKRDPQSNLFAHRDRGFIPEGLLNYLALLGWSIADDHDLFGLDEMV
AAFDVADVNSSPARFDQKKADALNAEHIRMLDVGDFTVRLRDHLDTHGHHIALDEAAFA
AAAELVQTRIVVLGDAWELLKFFNDDQYVIDPKAAAKELGPDGAAVLDAALAALTSVTD
WTAPLIEAALKDALIEGLALKPRKAFSPIRVAATGTTVSPPLFESLELLGRDRSMQRLRAARQ
LVGHA
00000000000000000000000000000000000000000000000000000000000000000000000000000000000
00000000000000000000000000000000000000000000000000000000000000000000000000000000000
00000000000000000000000000000000000000000000000000000000000000000000000000000000000
00000000000000000000000000000000000000000000000000000000000000000000000000000000000
00000000000000000000000000000000000000000000000000000000000000000000000000000000000
000000000000000000000000000000000000000000000000000000000000000000000011111
>Q0S4Q9_4at2_0_489
MVDATPIRPRSATTVTEWDYEADVVVAGYGIAGVAASIEAARAGADVLVLERTSGWGGAA
ALAGGFIYLGGGTPLQKACGFDDSPENMKTFMMAALGPGADEEKITDYCEGSVEHYNWL
VDCGVPFKESFWGEPGWEPPFDDGLMYSGGENAAPFNEIATPAPRGHVPQMDGKRTGEK
GGGYMLMKPLVETAEKLGVRAEYDMRVQTLVTDDTGRVVGIVAKQYGKEVAVRARRGVV
LATGSFAYNDKMIEAHAPRLIGRPGAAIEEHDGRSILMAQALGADLAHMDATEVAFVCDP
QLIVRGILVNGRGQRYVPEDTYSGRIGQMTLFHQDNQAFLIIDEASYEEGAAATTATPFLRV
QPKWAAETVEELESDMGLPAGALQSTVEVYNKHAAEGSDPLLHKKSEWVKPIGTPVAALD
LRGFTLGFTLGGLRTTVNSEVLHVSGEPIPGLFAAGRCTSGVCAGGYASGTSLGDGSFYGRR
AGISAAKQA
11111100000000000000000000000000000000000000000000000000000000000000000000000000000
00000000000000000000000000000000000000000000000000000000000000000000000000000000000
00000000000000000000000000000000000000000000000000000000000000000000000000000000000
00000000000000000000000000000000000000000000000000000000000000000000000000000000000
00000000000000000000000000000000000000000000000000000000000000000000000000000000000
000000000000000000000000000000000000000000000000000000000000000000000000001
>Q93LY7_2qa1_1_490
DAAVIVVGAGPAGMMLAGELRLAGVEVVVLERLVERTGESRGLGFTARTMEVFDQRGILP
RFGEVETSTQGHFGGLPIDFGVLEGAWQAAKTVPQSVTETHLEQWATGLGADIRRGHEVL
SLTDDGAGVTVEVRGPEGKHTLRAAYLVGCDGGRSSVRKAAGFDFPGTAATMEMYLADIK
GVELQPRMIGETLPGGMVMVGPLPGGITRIIVCERGTPPQRRETPPSWHEVADAWKRLTGD
DIAHAEPVWVSAFGNATRQVTEYRRGRVILAGDSAHIHLPAGGQGMNTSIQDAVNLGWK
LGAVVNGTATEELLDSYHSERHAVGKRLLMNTQAQGLLFLSGPEVQPLRDVLTELIQYGEV
ARHLAGMVSGLEITYDVGTGSHPLLGKRMPALELTTATRETSSTELLHTARGVLLDLADNP
RLRARAAAWSDRVDIVTAVPGEVSATSGLRDTTAVLIRPDGHVAWAAPGSHHDLPMALER
WFGAPLTG
00000000000000000000000000000000000000000000000000000000000000000000000000000000000
00000000000000000000000000000000000000000000000000000000000000000000000000000000000
00000000000000000000000000000000000000000000000000000111110000000000000000000000000
00000000000000000000000000000000000000000000000000000000000000000000000000000000000
00000000000000000000000000000000000000000000000000000000000000000000000000000000000
000000000000000000000000000000000000000000000000000000000000000000000000000
>P00520_1opk_26_514

EALQRPVASDFEPQGLSEAARWNSKENLLAGPSENDPNLFVALYDFVASGDNTLSITKGEK
LRVLGYNHNGEWCEAQTKNGQGWVPSNYITPVNSLEKHSWYHGPVSRNAAEYLLSSGIN
GSFLVRESESSPGQRSISLRYEGRVYHYRINTASDGKLYVSSESRFNTLAELVHHHSTVADGLI
TTLHYPAPKRNKPTIYGVSPNYDKWEMERTDITMKHKLGGGQYGEVYEGVWKKYSLTVAV
KTLKEDTMEVEEFLKEAAVMKEIKHPNLVQLLGVCTREPPFYIITEFMTYGNLLDYLRECNR
QEVSAVVLLYMATQISSAMEYLEKKNFIHRDLAARNCLVGENHLVKVADFGLSRLMTGDT
YTAHAGAKFPIKWTAPESLAYNKFSIKSDVWAFGVLLWEIATYGMSPYPGIDLSQVYELLEK
DYRMERPEGCPEKVYELMRACWQWNPSDRPSFAEIHQAFETMFQESSISDEVEKELGKRGT
1111111111111111111111111111111111111000000000000000000000000000000000000000000000
00000000000000000000000000000000000000000000000000000000000000000000000000000000000
00000000000000000000000000000000000000000000000000000000000000000000000000000000000
00000000000000000000000000000000000000000000000000000000000000000000000000000000000
00000000000000000000000000000000000000000000000000000000000000000000000000000000000
0000000000000000000000000000000000000000000000000000000000000000000000111
>P32171_2cgl_0_488
MTFRNCVAVDLGASSGRVMLARYERECRSLTLREIHRFNNGLHSQNGYVTWDVDSLESAIR
LGLNKVCEEGIRIDSIGIDTWGVDFVLLDQQGQRVGLPVAYRDSRTNGLMAQAQQQLGKR
DIYQRSGIQFLPFNTLYQLRALTEQQPELIPHIAHALLMPDYFSYRLTGKMNWEYTNATTTQ
LVNINSDDWDESLLAWSGANKAWFGRPTHPGNVIGHWICPQGNEIPVVAVASHDTASAVI
ASPLNGSRAAYLSSGTWSLMGFESQTPFTNDTALAANITNEGGAEGRYRVLKNIMGLWLL
QRVLQEQQINDLPALISATQALPACRFIINPNDDRFINPETMCSEIQAACRETAQPIPESDAEL
ARCIFDSLALLYADVLHELAQLRGEDFSQLHIVGGGCQNTLLNQLCADACGIRVIAGPVEA
STLGNIGIQLMTLDELNNVDDFRQVVSTTANLTTFTPNPDSEIAHYVAQIHSTRQTKELCA
10000000000000000000000000000000000000000000000000000000000000000000000000000000000
00000000000000000000000000000000000000000000000000000000000000000000000000000000000
00000000000000000000000000000000000000000000000000000000000000000000000000000000000
00000000000000000000000000000000000000000000000000000000000000000000000000000000000
00000000000000000000000000000000000000000000000000000000000000000000000000000000000
0000000000000000000000000000000000000000000000000000000000000000111111111
>P77967_1np7_0_488
MKHVPPTVLVWFRNDLRLHDHEPLHRALKSGLAITAVYCYDPRQFAQTHQGFAKTGPWR
SNFLQQSVQNLAESLQKVGNKLLVTTGLPEQVIPQIAKQINAKTIYYHREVTQEELDVERNL
VKQLTILGIEAKGYWGSTLCHPEDLPFSIQDLPDLFTKFRKDIEKKKISIRPCFFAPSQLLPSPN
IKLELTAPPPEFFPQINFDHRSVLAFQGGETAGLARLQDYFWHGDRLKDYKETRNGMVGA
DYSSKFSPWLALGCLSPRFIYQEVKRYEQERVSNDSTHWLIFELLWRDFFRFVAQKYGNKLF
NRGGLLNKNFPWQEDQVRFELWRSGQTGYPLVDANMRELNLTGFMSNRGRQNVASFLC
KNLGIDWRWGAEWFESCLIDYDVCSNWGNWNYTAGIGNDARDFRYFNIPKQSQQYDPQ
GTYLRHWLPELKNLPGDKIHQPWLLSATEQKQWGVQLGVDYPRPCVNFHQSVEARRKIE
QMGVIA
00000000000000000000000000000000000000000000000000000000000000000000000000000000000
00000000000000000000000000000000000000000000000000000000000000000000000000000000000
00000000000000000000000000000000000000000000000000000000000000000000000000000000000
00000000000000000000000000000000000000000000000000000000000000000000000000000000000
00000000000000000000000000000000000000000000000000000000000000000000000000000000000
0000000000000000000000000000000000000000000000000000000000000000000111111
>Q8VPD4_2jb2_45_533
AGDLIGKVKGSHSVVVLGGGPAGLCSAFELQKAGYKVTVLEARTRPGGRVWTARGGSEET
DLSGETQKCTFSEGHFYNVGATRIPQSHITLDYCRELGVEIQGFGNQNANTFVNYQSDTSLS
GQSVTYRAAKADTFGYMSELLKKATDQGALDQVLSREDKDALSEFLSDFGDLSDDGRYLG
SSRRGYDSEPGAGLNFGTEKKPFAMQEVIRSGIGRNFSFDFGYDQAMMMFTPVGGMDRIY
YAFQDRIGTDNIVFGAEVTSMKNVSEGVTVEYTAGGSKKSITADYAICTIPPHLVGRLQNNL

PGDVLTALKAAKPSSSGKLGIEYSRRWWETEDRIYGGASNTDKDISQIMFPYDHYNSDRGV
VVAYYSSGKRQEAFESLTHRQRLAKAIAEGSEIHGEKYTRDISSSFSGSWRRTKYSESAWAN
WAGSGGSHGGAATPEYEKLLEPVDKIYFAGDHLSNAIAWQHGALTSARDVVTHIHERVAQ
EA
11000000000000000000000000000000000000000000000000000000000000000000000000000000
00000000000000000000000000000000000000000000000000000000000000000000000000000000
00000000000000000000000000000000000000000000000000000000000000000000000000000000
00000000000000000000000000000000000000000000000000000000000000000000000000000000
00000000000000000000000000000000000000000000000000000000000000000000000000000000
00000000000000001111111000000000000000000000000000000000000000000000001
>O96693_3au8_0_487
MKKYIYIYFFFITITINDLVINNTSKCVSIERRKNNAYINYGIGYNGPDNKITKSRRCKRIKLCK
KDLIDIGAIKKPINVAIFGSTGSIGTNALNIIRECNKIENVFNVKALYVNKSVNELYEQAREFL
PEYLCIHDKSVYEELKELVKNIKDYKPIILCGDEGMKEICSSNSIDKIVIGIDSFQGLYSTMYAI
MNNKIVALANKESIVSAGFFLKKLLNIHKNAKIIPVDSEHSAIFQCLDNNKVLKTKCLQDN
FSKINNINKIFLCSSGGPFQNLTMDELKNVTSENALKHPKWKMGKKITIDSATMMNKGLEV
IETHFLFDVDYNDIEVIVHKECIIHSCVEFIDKSVISQMYYPDMQIPILYSLTWPDRIKTNLKPL
DLAQVSTLTFHKPSLEHFPCIKLAYQAGIKGNFYPTVLNASNEIANNLFLNNKIKYFDISSIIS
QVLESFNSQKVSENSEDLMKQILQIHSWAKDKATDIYNKHNSS
11111111111111111111111111111111111111111111111111111111111111111111111110000000
00000000000000000000000000000000000000000000000000000000000000000000000000000000
00000000000000000000000000000000000000000000000000000000000000000000000000000000
00000000000000000000000000000000000000000000011100000000000000000000000000000000
00000000000000000000000000000000000000000000000000000000000000000000000000000000
0000000000000000000000000000000000000000000000000000000000000000001111
>P36649_3qqx_28_515
AERPTLPIPDLLTTDARNRIQLTIGAGQSTFGGKTATTWGYNGNLLGPAVKLQRGKAVTVDI
YNQLTEETTLHWHGLEVPGEVDGGPQGIIPPGGKRSVTLNVDQPAATCWFHPHQHGKTG
RQVAMGLAGLVVIEDDEILKLMLPKQWGIDDVPVIVQDKKFSADGQIDYQLDVMTAAVG
WFGDTLLTNGAIYPQHAAPRGWLRLRLLNGCNARSLNFATSDNRPLYVIASDGGLLPEPV
KVSELPVLMGERFEVLVEVNDNKPFDLVTLPVSQMGMAIAPFDKPHPVMRIQPIAISASGAL
PDTLSSLPALPSLEGLTVRKLQLSMDPMLDMMGMQMLMEKYGDQAMAGMDHSQMMG
HMGHGNMNHMNHGGKFDFHHANKINGQAFDMNKPMFAAAKGQYERWVISGVGDM
MLHPFHIHGTQFRILSENGKPPAAHRAGWKDTVKVEGNVSEVLVKFNHDAPKEHAYMA
HCHLLEHEDTGMMLGFTV
00000000000000000000000000000000000000000000000000000000000000000000000000000000
00000000000000000000000000000000000000000000000000000000000000000000000000000000
00000000000000000000000000000000000000000000000000000000000000000000000000000000
00000000000000000000000000000000000000000000000000000000000000000000000000000000
00000000000000000000111111111111111111100000000000000000000000000000000000000000
0000000000000000000000000000000000000000000000000000000000000000000000
>P00727_2j9a_32_518
TKGLVLGIYSKEKEEDEPQFTSAGENFNKLVSGKLREILNISGPPLKAGKTRTFYGLHEDFPS
VVVVGLGKKTAGIDEQENWHEGKENIRAAVAAGCRQIQDLEIPSVEVDPCGDAQAAAEG
AVLGLYEYDDLKQKRKVVVSAKLHGSEDQEAWQRGVLFASGQNLARRLMETPANEMTPT
KFAEIVEENLKSASIKTDVFIRPKSWIEEQEMGSFLSVAKGSEEPPVFLEIHYKGSPNASEPPLV
FVGKGITFDSGGISIKAAANMDLMRADMGGAATICSAIVSAAKLDLPINIVGLAPLCENMP
SGKANKPGDVVRARNGKTIQVDNTDAEGRLILADALCYAHTFNPKVIINAATLTGAMDIA
LGSGATGVFTNSSWLWNKLFEASIETGDRVWRMPLFEHYTRQVIDCQLADVNNIGKYRSA
GACTAAAFLKEFVTHPKWAHLDIAGVMTNKDEVPYLRKGMAGRPTRTLIEFLFRFSQDSA
00000000000000000000000000000000000000000000000000000000000000000000000000000000

00000000000000000000000000000000000000000000000000000000000000000000000000000000000
00000000000000000000000000000000000000000000000000000000000000000000000000000000000
00000000000000000000000000000000000000000000000000000000000000000000000000000000000
00000000000000000000000000000000000000000000000000000000000000000000000000000000000
000000000000000000000000000000000000000000000000000000000000000000000011
>Q96242_2rcm_33_517
SGSETPDLTVATRTGSKDLPIRNIPGNYGLPIVGPIKDRWDYFYDQGAEEFFKSRIRKYNSTVY
RVNMPPGAFIAENPQVVALLDGKSFPVLFDVDKVEKKDLFTGTYMPSTELTGGYRILSYLDP
SEPKHEKLKNLLFFLLKSSRNRIFPEFQATYSELFDSLEKELSLKGKADFGGSSDGTAFNFLAR
AFYGTNPADTKLKADAPGLITKWVLFNLHPLLSIGLPRVIEEPLIHTFSLPPALVKSDYQRLYE
FFLESAGEILVEADKLGISREEATHNLLFATCFNTWGGMKILFPNMVKRIGRAGHQVHNRL
AEEIRSVIKSNGGELTMGAIEKMELTKSVVYECLRFEPPVTAQYGRAKKDLVIESHDAAFKV
KAGEMLYGYQPLATRDPKIFDRADEFVPERFVGEEGEKLLRHVLWSNGPETETPTVGNKQC
AGKDFVVLVARLFVIEIFRRYDSFDIEVGTSPLGSSVNFSSLRKASF
11111111111111111100000000000000000000000000000000000000000000000000000000000000000
00000000000000000000000000000000000000000000000000000000000000000000000000000000000
00000000000000000000000000000000000000000000000000000000000000000000000000000000000
00000000000000000000000000000000000000000000000000000000000000000000000000000000000
00000000000000000000000000000000000000000000000000000000000000000000000000000000000
0000000000000000000000000000000000000000000000000000000000000000000011
>P05327_1owl_0_483
MAAPILFWHRRDLRLSDNIGLAAARAQSAQLIGLFCLDPQILQSADMAPARVAYLQGCLQ
ELQQRYQQAGSRLLLLQGDPQHLIPQLAQQLQAEAVYWNQDIEPYGRDRDGQVAAALKT
AGIRAVQLWDQLLHSPDQILSGSGNPYSVYGPFWKNWQAQPKPTPVATPTELVDLSPEQLT
AIAPLLLSELPTLKQLGFDWDGGFPVEPGETAAIARLQEFCDRAIADYDPQRNFPAEAGTSG
LSPALKFGAIGIRQAWRAASAAHALSRSDEARNSIRVWQQELAWREFYQHALYHFPSLAD
GPYRSLWQQFPWENREALFTAWTQAQTGYPIVDAAMRQLTETGWMHNRCWMIVASFLT
KDLIIDWRRGEQFFMQHLVDGDLAANNGGWQWSASSGMDPKPLRIFNPASQAKKFDATA
TYIKRWLPELRHVHPKDLISGEITPIGRRGYPAPIVNHNLRQKQFKALYNQLKAAIAEPEAE
PDS
11000000000000000000000000000000000000000000000000000000000000000000000000000000000
00000000000000000000000000000000000000000000000000000000000000000000000000000000000
00000000000000000000000000000000000000000000000000000000000000000000000000000000000
00000000000000000000000000000000000000000000000000000000000000000000000000000000000
00000000000000000000000000000000000000000000000000000000000000000000000000000000000
000000000000000000000000000000000000000000000000000000000000111111111
>Q9N0F3_1wle_34_517
ATERQDRNLLYEHAREGYSALPLLDMESLCAYPEDAARALDLRKGELRSKDLPGIISTWQEL
RQLREQIRSLEEEKEAVTEAVRALVVNQDNSQVQQDPQYQSLRARGREIRKQLTLLYPKEA
QLEEQFYLRALRLPNQTHPDVPVGDESQARVLHVVGDKPAFSFQPRGHLEIAEKLDIIRQK
RLSHVSGHRSYYLRGAGALLQHGLVNFTLNKLIHRGFTPMTVPDLLRGVVFEGCGMTPNA
KPSQIYNIDPSRFEDLNLAGTAEVGLAGYFMDHSVAFRDLPIRMVCSSTCYRAETDTGKEP
WGLYRVHHFTKVEMFGVTGPGLEQSSELLEEFLSLQMEILTELGLHFRVLDMPTQELGLPAY
RKFDIEAWMPGRGRFGEVTSASNCTDFQSRRLHIMFQTEAGELQFAHTVNATGCAVPRLLI
ALLESYQQKDGSVLVPPALQPYLGTDRITTPTHVPLQYIGPNQPQKPRLPGQPASS
11110000000000000000000000000000000000000000000000000000000000000000000000000000000
00000000000000000000000000000000000000000000000000000000000000000000000000000000000
00000000000000000000000000000000000000000000000000000000000000000000000000000000000
00000000000000000000000000000000000000000000000000000000000000000000000000000000000
00000000000000000000000000000000000000000000000000000000000000000000000000000000000
000000000000000000000000000000000000000000000000000000000011111111111

>Q16647_3b6h_17_499
LLSRRRTRRPGEPPLDLGSIPWLGYALDFGKDAASFLTRMKEKHGDIFTILVGGRYVTVLLDP
HSYDAVVWEPRTRLDFHAYAIFLMERIFDVQLPHYSPSDEKARMKLTLLHRELQALTEAMY
TNLHAVLLGDATEAGSGWHEMGLLDFSYSFLLRAGYLTLYGIEALPRTHESQAQDRVHSA
DVFHTFRQLDRLLPKLARGSLSVGDKDHMCSVKSRLWKLLSPARLARRAHRSKWLESYLL
HLEEMGVSEEMQARALVLQLWATQGNMGPAAFWLLLFLLKNPEALAAVRGELESILWQA
EQPVSQTTTLPQKVLDSTPVLDSVLSESLRLTAAPFITREVVVDLAMPMADGREFNLRRGDR
LLLFPFLSPQRDPEIYTDPEVFKYNRFLNPDGSEKKDFYKDGKRLKNYNMPWGAGHNHCL
GRSYAVNSIKQFVFLVLVHLDLELINADVEIPEFDLSRYGFGLMQPEHDVPVRYRIRP
11111000000000000000000000000000000000000000000000000000000000000000000000000000000
00000000000000000000000000000000000000000000000000000000000000000000000000000000000
00000000000000000000000000000000000000000000000000000000000000000000000000000000000
00000000000000000000000000000000000000000000000000000111111111000000000000000000000
00000000000000000000000000000000000000000000000000000000000000000000000000000000000
00000000000000000000000000000000000000000000000000000000000000000000
>Q1GZG6_3b9t_0_482
MSDHVCQEGCRHHSHGEDSPEIQQEFQEGRRDFMRDFAVGGVLASAASLGISSSAFGQTM
PKTGLTSGHATHYYIPASDKTVSWGFFSKSLKPVVELESGDFATIETLTHHSNDDASLMVKG
DPGAESVFYWDSKRKNVDRRGMGPMDHKLGAGGGMGVHILTGPVAIKGAEPGDVLEVRI
VDVALRPSANPEFKGKTFGSNVAANWGFHYNELIEEPKKREVVTIYELDATGERNWARAF
YNYRWTPQKDPFGVVHPIVDYPGVPVDHSTISKNYNVLKNIRVPVRPHFGTMGLAPKEAD
LVNSVPPSHFGGNIDNWRIGKGATMYYPVSVAGGLFSVGDPHASQGDSEMCGTAIECSLTG
TFQFILHKKADLPGTPLADLQYPLLETQDEWVLHGFSYANYLAELGPDAQNSIFSKSSLDLA
LKDAFRKMRHFLMQTQNLTEDEAVSLMSIGVDFGITQVVDGNWGVHAVVKKGIFPGRDV
11111111111111111111111111111111111111111111111111111111111111100000000000000000000
00000000000000000000000000000000000000000000000000000000000000000000000000000000000
00000000000000000000000000000000000000000000000000000000000000000000000000000000000
00000000000000000000000000000000000000000000000000000000000000000000000000000000000
00000000000000000000000000000000000000000000000000000000000000000000000000000000000
00000000000000000000000000000000000000000000000000000000000000000001
>Q388T4_3gue_2_484
LNPPSAFSGAALACLEKMQASGVEEKCIHIFLIQHALVRKGETGYIPEKSIFPVESLPFLQGIE
TKGENTALLRQAVVLKLNGGLGTGMGLNGPKSLLQVKNGQTFLDFTALQLEHFRQVRNC
NVPFMLMNSFSTSGETKNFLRKYPTLYEVFDSDIELMQNRVPKIRQDNFFPVTYEADPTCE
WVPPGHGDVYTVLYSSGKLDYLLGKGYRYMFISNGDNLGATLDVRLLDYMHEKQLGFLM
EVCRRTESDKKGGHLAYKDVIDETTGQTRRRFVLRESAQCPKEDEDSFQNIAKHCFFNTNN
IWINLMELKKMMDEQLGVLRLPVMRNPKTVNPQDSQSTKVYQLEVAMGAAISLFDRSEAV
VVPRERFAPVKTCSDLLALRSDAYQVTEDQRLVLCEERNGKPPAIDLDGEHYKMIDGFEKL
VKGGVPSLRQCTSLTVRGLVEFGADVSVRGNVVIKNLKEEPLIIGNGRVLDNEVVVVE
11111110000000000000000000000000000000000000000000000000000000000000000000000000000
00000000000000000000000000000000000000000000000000000000000000000000000000000000000
00000000000000000000000000000000000000000000000000000000000000000000000000000000000
00000000000000001111100000000000000000000000000000000000000000000000000000000000000
00000000000000000000000000000000000000000000000000000000000000000000000000000000000
00000000000000000000000000000000000000000000000000000000000000000000
>P14779_3m4v_0_481
MTIKEMPQPKTFGELKNLPLLNTDKPVQALMKIADELGEIFKFEAPGRVTRYLSSQRLIKEA
CDESRFDKNLSQALKFVRDFAGDGLFTSWTHEKNWKKAHNILLPSFSQQAMKGYHAMM
VDIAVQLVQKWERLNADEHIEVPEDMTRLTLDTIGLCGFNYRFNSFYRDQPHPFITSMVRA
LDEAMNKLQRANPDDPAYDENKRQFQEDIKVMNDLVDKIIADRKASGEQSDDLLTHMLN
GKDPETGEPLDDENIRYQIITFLIAGHETTSGLLSFALYFLVKNPHVLQKAAEEAARVLVDPV

PSYKQVKQLKYVGMVLNEALRLWPTAPAFSLYAKEDTVLGGEYPLEKGDELMVLIPQLHR
DKTIWGDDVEEFRPERFENPSAIPQHAFKPFGNGQRACIGQQFALHEATLVLGMMLKHFD
FEDHTNYELDIKETLTLKPEGFVVKAKSKKIPLGGIPSPSTEQSAKKVRKKAENAHNTP
11111000000000000000000000000000000000000000000000000000000000000000000000000000000
00000000000000000000000000000000000000000000000000000000000000000000000000000000000
00000000000000000000000000000000000000000000000000000000000000000000000000000000000
00000000000000000000000000000000000000000000000000000000000000000000000000000000000
00000000000000000000000000000000000000000000000000000000000000000000000000000000000
0000000000000000000000000000000000000000011111111111111111111111111
>Q13325_4j0u_0_481
MSEIRKDTLKAILLELECHFTWNLLKEDIDLFEVEDTIGQQLEFLTTKSRLALYNLLAYVKHL
KGQNKDALECLEQAEEIIQQEHSDKEEVRSLVTWGNYAWVYYHMDQLEEAQKYTGKIGN
VCKKLSSPSNYKLECPETDCEKGWALLKFGGKYYQKAKAAFEKALEVEPDNPEFNIGYAIT
VYRLDDSDREGSVKSFSLGPLRKAVTLNPDNSYIKVFLALKLQDVHAEAEGEKYIEEILDQIS
SQPYVLRYAAKFYRRKNSWNKALELLKKALEVTPTSSFLHHQMGLCYRAQMIQIKKATHN
RPKGKDKLKVDELISSAIFHFKAAMERDSMFAFAYTDLANMYAEGGQYSNAEDIFRKALRL
ENITDDHKHQIHYHYGRFQEFHRKSENTAIHHYLEALKVKDRSPLRTKLTSALKKLSTKRL
CHNALDVQSLSALGFVYKLEGEKRQAAEYYEKAQKIDPENAEFLTALCELRLSI
00000000000000000000000000000000000000000000011000000000000000000000000000000000000
00000000000000000000000000000000000000000000000000000000000000000000000000000000000
00000000000000000000000111111000000000000000000000000000000000000000000000000000000
00000000000000000000000000000000000000000000000000000000000000000000000000000000000
00000000000000000000000000000000000000000000000000000000000000000000000000000000000
0000000000000000000100000000000000000000000000000000000000000000011
>A1W1C2_3zx1_32_512
KNHSINHDLDTSFINFAPKNLKLLDPKQFPQGEILKALPLLKNESKEKNIFRATIEIKENHIEL
IKGKKTLFYTYNGLVPAPKIEVFEGDKLEILVKNKLKEATTIHWHGVPVPPDQDGSPHDPIL
AGEERIYRFEIPQDSAGTYWYHPHPHYTTSKQVFMGLAGAFVIKAKKDALSHLKERDLMIS
DLRLDENAQIPNNNLNDWLNGREGEFVLINGQFKPKIKLATNERIRIYNATAARYLNLRIQ
GAKFILVGTDGGLIEKAIYKEEFFLSPASRVEVLIDAPKDGEFRLESAYYDRDKMMVKEEPNT
LFLANINLKKENVELPKNLKIFKPSEEPKEFKEIIMSEDHMQMHGMMGKSESELKIALASMF
LINGKSYDLKRIDLSSKLGVVEDWIVINKSHMDHPFHIHGTQFELISSKLNGKVQKAEFRAL
RDTINVRPNEELRLRMKQDFKGLRMYHCHILEHEDLGMMGNLEVKE
11111111110000000000000000000000000000000000000000000000000000000000000000000000000
00000000000000000000000000000000000000000000000000000000000000000000000000000000000
00000000000000000000000000000000000000000000000000000000000000000000000000000000000
00000000000000000000000000000000000000000000000000000000000000000000000000000000000
00000000000000000000000000000000000000000000000000000000000000000000000000000000000
000000000000000000000000000000000000000000000000000000000000000000
>A6LIX4_3otn_25_505
QPTGTMTTDSKLTSKESALALTNSAYLKNTVFNKMTPGWGCNTILLLEYMTGKATSENSQS
NYKDFQDLLVSDRSLYIEDWWQDCYAGIANCNLALQKLGEFENLDASLVNGYMAEVKFM
RALYYFYLVRIFGDVPKITTVQSELGELQVSRAPVKEIYDEIIIPDLLEAEQSDLAFSDHTGRVS
MGAVKALLADVYLTYAGYPLQGGKSYYAESAKRSLEVIKSNEYTLFTDYESLRLPSQNNKG
EFIYQVQFSLNKRHNESVRIFLPSRSGISAYDLEYGSLIPTKEFVESFEKGDKRTEEKQYFFTNY
KGHPSKFSPGAAELEFMDLNGYYIYKFFDQVAVDNTAKSDLNWSVYRYTDVLLMYAEAQV
NADGTPNQQSIDIVNQIRGRAGLAPFKQTNASAFLEEVWDQRYFDLCYENKMWFDMLRT
RKIRDDKSGEYVDFIGYKTNWGKVYTETQLLFPIPLSERQANPNLTQNQGY
11111111110000000000000000000000000000000000000000000000000000000000000000000000000
00000000000000000000000000000000000000000000000000000000000000000000000000000000000
00000000000000000000000000000000000000000000000000000000000000000000000000000000000

000000000000000000000000000000000000000000000000000000000000000000000000000000000000
000000000000000000000000000000000000000000000000000000000000000000000000000000000000
000000000000000000000000000000000000000000000000000000000000000000
>O59245_4dwr_0_870
MVVPLKRIDKIRWEIPKFDKRMRVPGRVYADEVLLEKMKNDRTLEQATNVAMLPGIYKYSI
VMPDGHQGYGFPIGGVAAFDVKEGVISPGGIGYDINCGVRLIRTNLTEKEVRPRIKQLVDTL
FKNVPSGVGSQGRIKLHWTQIDDVLVDGAKWAVDNGYGWERDLERLEEGGRMEGADPE
AVSQRAKQRGAPQLGSLGSGNHFLEVQVVDKIFDPEVAKAYGLFEGQVVVMVHTGSRGL
GHQVASDYLRIMERAIRKYRIPWPDRELVSVPFQSEEGQRYFSAMKAAANFAWANRQMIT
HWVRESFQEVFKQDPEGDLGMDIVYDVAHNIGKVEEHEVDGKRVKVIVHRKGATRAFPP
GHEAVPRLYRDVGQPVLIPGSMGTASYILAGTEGAMKETFGSTCHGAGRVLSRKAATRQYR
GDRIRQELLNRGIYVRAASMRVVAEEAPGAYKNVDNVVKVVSEAGIAKLVARMRPIGVAK
G
10000000000000000000000000000000000000000000000000000000000000000000000000000000000
00000000000000000000000000000000000000000000000000000000000000000000000000000000000
00000000000000000000000000000000000000000000000000000000000000000000000000000000000
00000000000000000000000000000000000000000000000000000000000000000000000000000000000
00000000000000000000000000000000000000000000000000000000000000000000000000000000000
000000000000000000000000000000000000000000000000000000000000000000
>P00466_1h1l_2_482
TNATGERNLALIQEVLEVFPETARKERRKHMMVSDPKMKSVGKCIISNRKSQPGVMTVRG
CAYAGSKGVVFGPIKDMAHISHGPAGCGQYSRAERRNYYTGVSGVDSFGTLNFTSDFQERD
IVFGGDKKLSKLIEEMELLFPLTKGITIQSECPVGLIGDDISAVANASSKALDKPVIPVRCEGFR
GVSQSLGHHIANDVVRDWILNNREGQPFETTPYDVAIIGDYNIGGDAWASRILLEEMGLRV
VAQWSGDGTLVEMENTPFVKLNLVHCYRSMNYIARHMEEKHQIPWMEYNFFGPTKIAESL
RKIADQFDDTIRANAEAVIARYEGQMAAIIAKYRPRLEGRKVLLYMGGLRPRHVIGAYEDL
GMEIIAAGYEFAHNDDYDRTLPDLKEGTLLFDDASSYELEAFVKALKPDLIGSGIKEKYIFQK
MGVPFRQMHSWDYSGPYHGYDGFAIFARDMDMTLNNPAWNELTAPWLKSA
00000000000000000000000000000000000000000000000000000000000000000000000000000000000
00000000000000000000000000000000000000000000000000000000000000000000000000000000000
00000000000000000000000000000000000000000000000000000000000000000000000000000000000
00000000000000000000000000000000000000000000000000000000000000000000000000000000000
00000000000000000000000000000000000000000000000000000000000000000000000000000000000
000000000000000000000000000000000000000000000000000000000000000111
>A3DHG6_2y8k_36_515
SPQRGRPRLNAARTTFVGDNGQPLRGPYTSTEWTAAAPYDQIARVKELGFNAVHLYAECF
DPRYPAPGSKAPGYAVNEIDKIVERTRELGLYLVITIGNGANNGNHNAQWARDFWKFYAP
RYAKETHVLYEIHNEPVAWGPPYSSSTANPPGAVDMEIDVYRIIRTYAPETPVLLFSYAVFGG
KGGAAEALKDIRAFNKAVFGNENAVWTNEAVAFHGYAGWQETTIAVEELLKAGYPCFMT
EYAGGAWGSGMGGLDVELTYELERLGVSWLTFQYIPPTGVSDDVTKPEYFSALVENSGLSW
TPDYGNWPAARGVYGNGGLARETATWINNFLTGTTRIEAEDFDWGGNGVSYYDTDSVNV
GGQYRPDEGVDIEKTSDTGGGYNVGWISEGEWLEYTIRVRNPGYYNLSLRVAGISGSRVQVS
FGNQDKTGVWELPATGGFQTWTTATRQVFLGAGLQKLRINALSGGFNLNWIELSPI
00000000000000000000000000000000000000000000000000000000000000000000000000000000000
00000000000000000000000000000000000000000000000000000000000000000000000000000000000
00000000000000000000000000000000000000000000000000000000000000000000000000000000011
00000000000000000000000000000000000000000000000000000000000000000000000000000000000
00000000000000000000000000000000000000000000000000000000000000000000000000000000000
00000000000000000000000000000000000000000000000000000000000000000
>A6KXE4_3jq1_33_512
TEDTFWKDETDFNLALTSCYTPLKNALNGGYYGTRGVMLRIARADEVDFRNDISDVYTVN

RFTNSNTNSLTQGMFYQFYNALYRTNSIMQKLEEKKEQFSTDFQNSVKGECLFIRGFYLFQL
AKEFKDAPLRLTASQSPSTFPLAKSSQADIWAQAKEDLKTAASLLPITNKIGKPTQGAAYAA
LGKIYVVYEENWQEAINVLEPLTQNPYTYKLVEDFNWNFDDTHENNAESIFELLIEDVGGTD
LWGDGENINSTQSNTRPKEYAAAEVGGWYEANPTQQIMDIFWKEKDKDGNFDYRARCSV
AWDYEGCTYYQRPFREVFAQDKWKTYWILKYQNWKTQKDEPAPPKSFINERAIRYADVLL
MLAEAYMNKGALDTSIGYINQIRRRANLNDYSGPITKEGVFEDLVHQRAIEFFVEGERFYDL
RRWGLLEQTLKTCDDTRYKNYQTGKSDNINKFNYFPIPAKELDTNPLCTPSEGW
1110000000000000000000000000000000000000000000000000000000000000000000000000000000000000000000
0000000000000000000000000000000000000000000000000000000000000000000000000000000000000000000000
0000000000000000000000000000000000000000000000000000000000000000000000000000000000000011111011
1111110000000000000000000000000000000000000000000000000000000000000000000000000000000000000000
0000000000000000000000000000000000000000000000000000000000000000000000000000000000000000000000
0000000000000000000000000000000000000000000000000000000000000000000000000000
>O33830_1obb_0_479
MPSVKIGIIGAGSAVFSLRLVSDLCKTPGLSGSTVTLMDIDEERLDAILTIAKKYVEEVGADLK
FEKTMNLDDVIIDADFVINTAMVGGHTYLEKVRQIGEKYGYYRGIDAQEFNMVSDYYTFSN
YNQLKYFVDIARKIEKLSPKAWYLQAANPIFEGTTLVTRTVPIKAVGFCHGHYGVMEIVEKL
GLEEEKVDWQVAGVNHGIWLNRFRYNGGNAYPLLDKWIEEKSKDWKPENPFNDQLSPA
AIDMYRFYGVMPIGDTVRNSSWRYHRDLETKKKWYGEPWGGADSEIGWKWYQDTLGKV
TEITKKVAKFIKENPSVRLSDLGSVLGKDLSEKQFVLEVEKILDPERKSGEQHIPFIDALLNDN
KARFVVNIPNKGIIHGIDDDVVVEVPALVDKNGIHPEKIEPPLPDRVVKYYLRPRIMRMEMA
LEAFLTGDIRIIKELLYRDPRTKSDEQVEKVIEEILALPENEEMRKHYLKR
1000000000000000000000000000000000000000000000000000000000000000000000000000000000000000000000
0000000000000000000000000000000000000000000000000000000000000000000000000000000000000000000000
0000000000000000000000000000000000000000000000000000000000000000000000000000000000000000000000
0000000000000000000000000000000000000000000000000000000000000000000000000000000000000000000000
0000000000000000000000000000000000000000000000000000000000000000000000000000000000000000000000
0000000000000000000000000000000000000000000000000000000000000000000000000000
>P08148_1lml_99_576
VVRDVNWGALRIAVSTEDLTDPAYHCARVGQHVKDHAGAIVTCTAEDILTNEKRDILVKH
LIPQAVQLHTERLKVQQVQGKWKVTDMVGDICGDFKVPQAHITEGFSNTDFVMYVASVPS
EEGVLAWATTCQTFSDGHPAVGVINIPAANIASRYDQLVTRVVTHEMAHALGFSGPFFEDA
RIVANVPNVRGKNFDVPVINSSTAVAKAREQYGCDTLEYLEVEDQGGAGSAGSHIKMRNA
QDELMAPAAAAGYYTALTMAIFQDLGFYQADFSKAEVMPWGQNAGCAFLTNKCMEQSV
TQWPAMFCNESEDAIRCPTSRLSLGACGVTRHPGLPPYWQYFTDPSLAGVSAFMDYCPVV
VPYSDGSCTQRASEAHASLLPFNVFSDAARCIDGAFRPKATDGIVKSYAGLCANVQCDTAT
RTYSVQVHGSNDYTNCTPGLRVELSTVSNAFEGGGYITCPPYVEVCQGNVQAAKDGGN
0000000000000000000000000000000000000000000000000000000000000000000000000000000000000000000000
0000000000000000000000000000000000000000000000000000000000000000000000000000000000000000000000
0000000000000000000000000000000000000000000000000000000000000000000000000000000000000000000000
0000000000000000000000000000000000000000000000000000000000000000000000111100000000000000000000
0000000000000000000000000000000000000000000000000000000000000000000000000000001111110000000000
00000000000000000000000000000000000000000000000000000000000000000000000111
>P0C1B3_2gvy_21_498
ATPADWRSQSIYFLLTDRFARTDGSTTATCNTADQKYCGGTWQGIIDKLDYIQGMGFTAIWI
TPVTAQLPQTTAYGDAYHGYWQQDIYSLNENYGTADDLKALSSALHERGMYLMVDVVAN
HMGYDGAGSSVDYSVFKPFSSQDYFHPFCFIQNYEDQTQVEDCWLGDNTVSLPDLDTTKD
VVKNEWYDWVGSLVSNYSIDGLRIDTVKHVQKDFWPGYNKAAGVYCIGEVLDGDPAYTC
PYQNVMDGVLNYPIYYPLLNAFKSTSGSMDDLYNMINTVKSDCPDSTLLGTFVENHDNPR
FASYTNDIALAKNVAAFIILNDGIPIIYAGQEQHYAGGNDPANREATWLSGYPTDSELYKLIA
SANAIRNYAISKDTGFVTYKNWPIYKDDTTIAMRKGTDGSQIVTILSNKGASGDSYTLSLSG

AGYTAGQQLTEVIGCTTVTVGSDGNVPVPMAGGLPRVLYPTEKLAGSKICSSS
00000000000000000000000000000000000000000000000000000000000000000000000000000000000
00000000000000000000000000000000000000000000000000000000000000000000000000000000000
00000000000000000000000000000000000000000000000000000000000000000000000000000000000
00000000000000000000000000000000000000000000000000000000000000000000000000000000000
00000000000000000000000000000000000000000000000000000000000000000000000000000000000
00000000000000000000000000000000000000000000000000000000000011
>P50579_1b6a_0_477
MAGVEEVAASGSHLNGDLDPDDREEGAASTAEEAAKKKRRKKKKSKGPSAAGEQEPDKE
SGASVDEVARQLERSALEDKERDEDDEDGDGDGDGATGKKKKKKKKKRGPKVQTDPPSV
PICDLYPNGVFPKGQECEYPPTQDGRTAAWRTTSEEKKALDQASEEIWNDFREAAEAHRQ
VRKYVMSWIKPGMTMIEICEKLEDCSRKLIKENGLNAGLAFPTGCSLNNCAAHYTPNAGD
TTVLQYDDICKIDFGTHISGRIIDCAFTVTFNPKYDTLLKAVKDATNTGIKCAGIDVRLCDVG
EAIQEVMESYEVEIDGKTYQVKPIRNLNGHSIGQYRIHAGKTVPIVKGGEATRMEEGEVYAI
ETFGSTGKGVVHDDMECSHYMKNFDVGHVPIRLPRTKHLLNVINENFGTLAFCRRWLDR
LGESKYLMALKNLCDLGIVDPYPPLCDIKGSYTAQFEHTILLRPTCKEVVSRGDDY
11111111111111111111111111111111111111111111111111111111111111111111111111111111111
11111111111111111111111111100000000000000000000000000011111111111110000000000000000
00000000000000000000000000000000000000000000000000000000000000000000000000000000000
00000000000000000000000000000000000000000000000000000000000000000000000000000000000
00000000000000000000000000000000000000000000000000000000000000000000000000000000000
00000000000000000000000000000000000000000000000000000000000000
>Q5CS01_3lij_41_516
DLQATPGMFITSKKGHLSEMYQRVKKLGSGAYGEVLLCRDKVTHVERAIKIIRKTSVSTSSNS
KLLEEVAVLKLLDHPNIMKLYDFFEDKRNYYLVMECYKGGELFDEIIHRMKFNEVDAAVIIK
QVLSGVTYLHKHNIVHRDLKPENLLLESKEKDALIKIVDFGLSAVFENQKKMKERLGTAYYI
APEVLRKKYDEKCDVWSIGVILFILLAGYPPFGGQTDQEILRKVEKGKYTFDSPEWKNVSEG
AKDLIKQMLQFDSQRRISAQQALEHPWIKEMCSKKESGIELPSLANAIENMRKFQNSQKLA
QAALLYMASKLTSQEETKELTDIFRHIDKNGDGQLDRQELIDGYSKLSGEEVAVFDLPQIESE
VDAILGAADFDRNGYIDYSEFVTVAMDRKSLLSKDKLESAFQKFDQDGNGKISVDELASVF
GLDHLESKTWKEMISGIDSNNDGDVDFEEFCKMIQKLCSNNE
00000000000000000000000000001110000000000000000000000011111000000000000000000000000
00000000000000000000000000000000000000000000000000000000000000000000000000000000000
00000000000000000000000000000000000000000000000000000000000000000000000000000000000
00000000000000000000000000000000000011100000000000000000000000000000000000000000000
00000000000000000000000000000000011000000000000000000000000000000000000000000000000
000000000000000000010000000000000000000000000000000000000001
>C1F2K5_3vo0_0_474
MAFARGGLAQTASQTTSSPVRVGLSVDASALGHTIPPDYTGLSYEQAQMANPNYFSGANT
QLAGFLRTLGRQGVLRIGGNTSEYTFWNRHAKPTAADEHLAAGPDKGHHAAAREVITPE
AVNNLSEFLDKTGWKLIYGLNLGKGTPENAADEAAYVMETIGADRLLAFQLGNEPDLFYR
NGIRPASYDFAAYAGDWQRFFTAIRKRVPNAPFAGPDTAYNTKWLVPFADKFKHDVKFISS
HYYAEGPPTDPSMTIERLMKPNPRLLGETAGLKQVEADTGLPFRLTETNSCYQGGKQGVSD
TFAAALWAGDLMYQQAAAGSTGINFHGGGYGWYTPVAGTPEDGFIARPEYYGMLLFAQA
GAGQLLGAKLTDNSAAPLLTAYALRGTDGRTRIALFNKNLDADVEVAISGVASPSGTVLRL
EAPRADDTTDVTFGGAPVGASGSWSPLVQEYVPGHSGQFVLHMRKASGALLEFA
11111111111111111000000000000000000000000000000000000000000000000000000000000000000
00000000000000000000000000000000000000000000000000000000000000000000000000000000000
00000000000000000000000000000000000000000000000000000000000000000000000000000000000
00000000000000000000000000000000000000000000000000000000000000000000000000000000000
00000000000000000000000000000000000000000000000000000000000000000000000000000000000

00000000000000000000000000000000000000000000000000000000000
>P04995_3hl8_0_474
MMNDGKQQSTFLFHDYETFGTHPALDRPAQFAAIRTDSEFNVIGEPEVFYCKPADDYLPQP
GAVLITGITPQEARAKGENEAAFAARIHSLFTVPKTCILGYNNVRFDDEVTRNIFYRNFYDP
YAWSWQHDNSRWDLLDVMRACYALRPEGINWPENDDGLPSFRLEHLTKANGIEHSNAH
DAMADVYATIAMAKLVKTRQPRLFDYLFTHRNKHKLMALIDVPQMKPLVHVSGMFGAW
RGNTSWVAPLAWHPENRNAVIMVDLAGDISPLLELDSDTLRERLYTAKTDLGDNAAVPVK
LVHINKCPVLAQANTLRPEDADRLGINRQHCLDNLKILRENPQVREKVVAIFAEAEPFTPS
DNVDAQLYNGFFSDADRAAMKIVLETEPRNLPALDITFVDKRIEKLLFNYRARNFPGTLDY
AEQQRWLEHRRQVFTPEFLQGYADELQMLVQQYADDKEKVALLKALWQYAEEIV
11111000000000000000000000000000000000000000000000000000000000000000000000000000000
00000000000000000000000000000000000000000000000000000000000000000000000000000000000
00000000000011100000000000000000000000000000000000000000000000000000000000000000000
00000000000000000000000000000000000011111100000000000000000000000000000000000000000
00000000000000000000011111000000000000000000000000000000000000000000000000000000000
00000000000000000000000000000000000000000000000000000000000
>P45066_1p3d_0_474
MKHSHEEIRKIIPEMRRVQQIHFIGIGGAGMSGIAEILLNEGYQISGSDIADGVVTQRLAQAG
AKIYIGHAEEHIEGASVVVVSSAIKDDNPELVTSKQKRIPVIQRAQMLAEIMRFRHGIAVAGT
HGKTTTTAMISMIYTQAKLDPTFVNGGLVKSAGKNAHLGASRYLIAEADESDASFLHLQP
MVSVVTNMEPDHMDTYEGDFEKMKATYVKFLHNLPFYGLAVMCADDPVLMELVPKVGR
QVITYGFSEQADYRIEDYEQTGFQGHYTVICPNNERINVLLNVPGKHNALNATAALAVAKE
EGIANEAILEALADFQGAGRRFDQLGEFIRPNGKVRLVDDYGHHPTEVGVTIKAAREGWG
DKRIVMIFQPHRYSRTRDLFDDFVQVLSQVDALIMLDVYAAGEAPIVGADSKSLCRSIRNLG
KVDPILVSDTSQLGDVLDQIIQDGDLILAQGAGSVSKISRGLAESWKN
11111111110000000000000000000000000000000000000000000000000000000000000000000000000
00000000000000000000000000000000000000000000000000000000000000000000000000000000000
00000000000000000000000000000000000000000000000000000000000000000000000000000000000
00000000000000000000000000000000000000000000000000000000000000000000000000000000000
00000000000000000000000000000000000000000000000000000000000000000000000000000000000
00000000000000000000000000000000000000000000000000000000011
>O28511_2zyr_0_473
MRGLAVLVLLVFAVQVAAAEDFRPVVFVHGLAGSAGQFESQGMRFAANGYPAEYVKTFEY
DTISWALVVETDMLFSGLGSEFGLNISQIIDPETLDKILSKSRERLIDETFSRLDRVIDEALAESG
ADKVDLVGHSMGTFFLVRYVNSSPERAAKVAHLILLDGVWGVDAPEGIPTLAVFGNPKAL
PALGLPEEKVVYNATNVYFNNMTHVQLCTSPETFAVMFEFINGYKPATTDIVPQDGDYVKV
KGKFLAFATNGDVSGWLSIYPIDENGKRLTRLPVKFMRVKGDFEVRLRKGQLYEFQFRKDFS
PIIYHYYRAPFVRDDLWARFLVSKPPLDVELLILPERLSPAAKETSGLLLIRYKEMIGEYDEEIG
GVDEVYVNGVNVCTERICPIERAVNGLWVFDRGADGKSDLDREVVRYSIMPFMSAADLVV
PAEGTISIAVKSRTGGEESFTIPAWSADRHSIIVQFSDYI
11111111111111111111000000000000000000000000000000000000000000000000000000000000000
00000000000000000000000000000000000000000000000000000000000000000000000000000000000
00000000000000000000000000000000000000000000000000000000000000000000000000000000000
00000000000000000000000000000000000000000000000000000000000000000000000000000000000
00000000000000000000000000000000000000000000000000000000000000000000000000000000000
0000000000000000000000000000000000000000000000000000000000
>O85040_1svd_0_472
MAVKKYSAGVKEYRQTYWMPEYTPLDSDILACFKITPQPGVDREEAAAAVAAESSTGTWT
TVWTDLLTDMDYYKGRAYRIEDVPGDDAAFYAFIAYPIDLFEEGSVVNVFTSLVGNVFGFK
AVRGLRLEDVRFPLAYVKTCGGPPHGIQVERDKMNKYGRPLLGCTIKPKLGLSAKNYGRA
VYECLRGGLDFTKDDENINSQPFMRWRDRFLFVQDATETAEAQTGERKGHYLNVTAPTPE

EMYKRAEFAKEIGAPIIMHDYITGGFTANTGLAKWCQDNGVLLHIHRAMHAVIDRNPNH
GIHFRVLTKILRLSGGDHLHTGTVVGKLEGDRASTLGWIDLLRESFIPEDRSRGIFFDQDWGS
MPGVFAVASGGIHVWHMPALVNIFGDDSVLQFGGGTLGHPWGNAAGAAANRVALEACV
EARNQGRDIEKEGKEILTAAAQHSPELKIAMETWKEIKFEFDTVDKLDTQNR
11111111111111100000000000000000000000000000000000000000000000000000000000000000
00000000000000000000000000000000000000000000000000000000000000000000000000000000
00000000000000000000000000000000000000000000000000000000000000000000000000000000
00000000000000000000000000000000000000000000000000000000000000000000000000000000
00000000000000000000000000000000000000000000000000000000000000000000000000000000
00000000000000000000000000000000000000000001111111111111
>Q5LB02_3ejn_17_489
GNLEEMNIDPDNATQTHPKLLLTQICMNAFKRGTDGMYATKKVIQADGESADQYYKWTR
GSFGYYDNLRNVQKMGEEAERVNAPVYTALTKFFRAYYFYELTLRFGDIPYSQALKGEKEEI
YTPEYDAQEDVFAGILQELREADEILANDASVIDGDIIYNGNSTQWRKLINSFRLKVLMTLS
NHTTVGNINIASEFKNIATNSPLMNSLADNGQLVYLDQQGNRYPQFNAQWSGYYMDDTF
IQRMRERRDPRLFIFSAQTNKGKTEGKPIDDFSSYEGGDPAAPYSDAIIKVSEGTISPINDRFR
TDPIVEPTMLMGYAELQQILAEAVVRGWISGNAQTYYEKGIRASFSFYETHAKDYAGYLNE
NAVAQYLKEPLVDFTQASGTEEQIERIIMQKYLVTFYQGNWDSFYEQLRTGYPDFRRPAGTE
IPKRWMYPQGEYDNNGTNVETAITRQFGAGNDKINQATWWQKKS
11111111111111000000000000000000000000000000000000000000000000000000000000000000
00000000000000000000000000000001111110000000000000000000000000000000000000000000
00000000000000000000000000000000000000000000000000000000000000000000000000000000
00000000000000000000000000000000000000000001110000000000000000000000000000000000
00000000000000000000000000000000000000000000000000000000000000000000000000000000
00000000000000000000000000000000000000000000000000000000
>E3Y0J2_3me5_1_471
QENISVTDSYSTGNAAQAMLEKLLQIYDVKMLVAQLNGVGENHWSAAILKRALANDSAW
HRLSEKEFAHLQTLLPKPPEHHPHYAFRFIDLFAGIGGIRRGFESIGGQCVFTSEWNKHAVRT
YKANHYCDPATHHFNEDIRDITLSHQEGVSDEAAAEHIRQHIPEHDVLLAGFPCQPFSLAG
VSKKNSLGRAHGFACDTQGTLFFDVVRIIDARRPAMFVLENVKNLKSHDKGKTFRIIMQTL
DELGYDVADAEDNGPDDPKIIDGKHFLPQHRERIVLVGFRRDLNLKADFTLRDISECFPAQR
VTLAQLLDPMVEAKYILTPVLWKYLYRYAKKHQARGNGFGYGMVYPNNPQSVTRTLSARY
YKDGAEILIDRGWDMATGEKDFDDPLNQQHRPRRLTPRECARLMGFEAPGEAKFRIPVSD
TQAYRQFGNSVVVPVFAAVAKLLEPKIKQAVALRQQEAQHGRRSR
11111111111110000000000000000000000000001111110000000000000000000000000000000000
00000000000000000000000000000000000000000000000000000000000000000000000000000000
00000000000001111111111111111100000000000000000000000000000000000000000000000000
00000000000000000000000000000000000000000000000000000000000000000000000000000000
00000111111111100000000000000011100000000000000000000000000000000000000000000000
000000000000000000000000000000000000000000000000111111
>Q5ZXC3_3pfe_0_470
MFKPQGLYDYICQQWQEEILPSLCDYIKIPNKSPHFDAKWEEHGYMEQAVNHIANWCKSH
APKGMTLEIVRLKNRTPLLFMEIPGQIDDTVLLYGHLDKQPEMSGWSDDLHPWKPVLKNG
LLYGRGGADDGYSAYASLTAIRALEQQGLPYPRCILIIEACEESGSYDLPFYIELLKERIGKPSL
VICLDSGAGNYEQLWMTTSLRGNLVGKLTVELINEGVHSGSASGIVADSFRVARQLISRIEDE
NTGEIKLPQLYCDIPDERIKQAKQCAEILGEQVYSEFPWIDSAKPVIQDKQQLILNRTWRPAL
TVTGADGFPAIADAGNVMRPVTSLKLSMRLPPLVDPEAASVAMEKALTQNPPYNAKVDFK
IQNGGSKGWNAPLLSDWLAKAASEASMTYYDKPAAYMGEGGTIPFMSMLGEQFPKAQFM
ITGVLGPHSNAHGPNEFLHLDMVKKLTSCVSYVLYSFSQKK
00000000000000000000000000000000000000000000000000000000000000000000000000000000
00000000000000000000000000000000000000000000000000000000000000000000000000000000

00000000000000000000000000000000000000000000000000000000000000000000000000000000000
00000000000000000000000000000000000000000000000000000000000000000000000000000000000
00000000000000000000000000000000000000000000000000000000000000000000000000000000000
00000000000000000000000000000000000000000000000000000001
>Q9RS86_2jh3_1_471
RSLVLIGHGSHHHGESARATQQVAEALRGRGLAGHLPYDEVLEGYWQQEPGLRQVLRTVA
YSDVTVVPVFLSEGYVTETVLPRELGLGHQGPVPTGGVVRVLGGRRVRYTRPLGAHPGMA
DAIAAQARDTLPEGTDPADVTLLLLAARPGNAALETHAQALRERGQFAGVEVVLESREAL
TPESHAASAVPLSEWPSRVEAGQAVLVPFLTHLGKHAAERLQQALAQAAERFPQAPPLHV
GGPVGEHPAVAEVVLALAAEGREDERGGDIDQAHAEAWAALRHLAERGGRLGEVLLTPY
GGLFELRHTLDEGRATLDLQTVVTPEGLRDLTARDEAGRWRPIRTWRTLPRGWRAVLSPAD
LRLGLELLYPAVIEESYAHEHRRLHWTPWMSTARRQTGTLARVQRATPDQVDTVAAQVCA
SCLRTRLWAGHTLGQTIFSGVPGGLPCAEACTVLLAAVRDEVGREAMGSGD
00000000000000000000000000000000000000000000000000000000000000000000000000000000000
00000000000000000000000000000000000000000000000000000000000000000000000000000000000
00000000000011111111100000000000000000000000000000000000000000000000000000000000000
00000000000000000000000000000000000000000000000000000000000000000000000000000000000
00000000000000000000000000000000000000000000000000000000000000000000000000000000000
00000000000000000000000000000000000000000000000000111111
>P45494_1lfw_0_469
MDLNFKELAEAKKDAILKDLEELIAIDSSEDLENATEEYPVGKGPVDAMTKFLSFAKRDGF
DTENFANYAGRVNFGAGDKRLGIIGHMDVVPAGEGWTRDPFKMEIDEEGRIYGRGSADD
KGPSLTAYYGMLLLKEAGFKPKKKIDFVLGTNEETNWVGIDYYLKHEPTPDIVFSPDAEYPII
NGEQGIFTLEFSFKNDDTKGDYVLDKFKAGIATNVTPQVTRATISGPDLEAVKLAYESFLAD
KELDGSFEINDESADIVLIGQGAHASAPQVGKNSATFLALFLDQYAFAGRDKNFLHFLAEV
EHEDFYGKKLGIFHHDDLMGDLASSPSMFDYEHAGKASLLNNVRYPQGTDPDTMIKQVL
DKFSGILDVTYNGFEEPHYVPGSDPMVQTLLKVYEKQTGKPGHEVVIGGGTYGRLFERGVA
FGAQPENGPMVMHAANEFMMLDDLILSIAIYAEAIYELTKDEEL
00000000000000000000000000000000000000000000000000000000000000000000000000000000000
00000000000000000000000000000000000000000000000000000000000000000000000000000000000
00000000000000000000000000000000000000000000000000000000000000000000000000000000000
00000000000000000000000000000000000000000000000000000000000000000000000000000000000
00000000000000000000000000000000000000000000000000000000000000000000000000000000000
0000000000000000000000000000000000000000000000000000011
>Q04371_3pt1_0_469
MTIPGRFMTIDKGTFGEYTASTRWPIIIQNAIDDLSKHQETEKSNGTKFEQGEVIKKELKEFR
QEIIDRVPLRPFTEEEIKIANVPLSFNEYLKKHPEVNWGAVEWLFSEVYLYRRVNVLFQRQCE
WAKFDIFNRLKQSTFESSFYGVVELALRYENLLPQLREMKQNPGNEIDDILKVLFKEFIEISL
WGNATDLSLLTNATLEDIKSIQGAKARAASESKIVVNDTEKAWEVLTKARADANSREIRVD
FVLDNSGFELYADLMLAAFLLQSGLATKCIFHAKDIPYMVSDVMLKDFDILVHDLRDREFFP
SGEPSTKESRALDLFAGEMEKFVSSGKIEFREDSFWTTELDYWNLDANETKYHGSILHKDLQ
KSNLVIFKGDLNYRKLTGDRKWPRTTKWETAIGPLATNGITSLSLRTCKADVQVALPEGLD
AKLSQEWEKENPGRGSWWCCSGKWAVICFCSGIHK
00000000000000000000000000000000000000000000000000000000000000000000000000000000000
00000000000000000000000000000000000000000000000000000000000000000000000000000000000
00111110000000000000000000000000000000000000000000000000000000000000000000000000000
00000000000000000000000000000000000000000000000000000000000000000000000000000000000
00000000000000000000000000000000000000000000000000000000000000000000000000000000000
0000000000000000000000000000000000000000000000000000011
>A7RZU9_3re2_0_485
MAGKSIPFPLKDIKSVVEVFRQELRNDQPNLAQLSIVLGFFESASTMKAGDTTSTPSLDEETF

DALHCKFMALLQRDFTFNAGGLPATREIVKNVADLVWGCLAKSYFKDRPHIQNLYSFLTG
NRLDCFGVAFAVVAMCQALGYNDVHLALSEDHAWVVFGKDKIETAEVTWHGKGNEDKR
GKPVIYQDDDRCSWLYLNGNAVHCTRHMEVASIISSINPNINHTTDSIQLSQLQQELLWLLY
DMGHIKTYPMAIANLGDLEEISPTPGRPPAEELFKEAITVAKREYSDHHIYPYTYLGGYYYRK
KKYYEAIASWVDAGYVAGKYNYSKDDEEMYKEFHEIANDLIPNILKDAVAKADPSGTGST
NLTSDPKFFALVLQFYDGICLWEEGSPTPVLHADWAKKLVQSIGKFAPECRSAFNANTDTLS
LRSAKMREMKNLITTSGKLNTSAMKLQLTAQSQVNVPKSRR
11100000000000000000000000000000000000000000000111111100000000000000000000000000000
00000000000000000000000000000000000000000000000000000000000000000000000000000000000
00000000000000000000000000000000000000000000000000000000000000000000000000000000000
00000000000000000000000000000000000000000000000000000000000000000000000000000000000
00000000000000000000000000111111110000000000000000000000000000000000000000000000000
000000000000000000000000000111110000000000001111111111
>Q194T1_3tia_0_468
MNPNQKIITIGSVSLTIATVCFLMQIAILATTVTLHFKQHECDSPASNQVMPCEPIIIERNITEI
VYLNNTTIEKEICPKVVEYRNWSKPQCQITGFAPFSKDNSIRLSAGGDIWVTREPYVSCDPG
KCYQFALGQGTTLDNKHSNDTVHDRIPHRTLLMNELGVPFHLGTRQVCIAWSSSSCHDGK
AWLHVCITGDDKNATASFIYDGRLVDSIGSWSQNILRTQESECVCINGTCTVVMTDGSASG
RADTRILFIEEGKIVHISPLSGSAQHIEECSCYPRYPGVRCICRDNWKGSNRPVVDINMEDYSI
DSSYVCSGLVGDTPRNDDSSSNSNCRNPNNERGTQGVKGWAFDNGNDLWMGRTISKESR
SGYETFKVIGGWSTPNSKSQVNRQVIVDNNNWSGYSGIFSVEGKSCINRCFYVELIRGRPQE
TRVWWTSNSIVVFCGTSGTYGTGSWPDGANINFMPI
11111111111111111111111111111111111111111111111111111111111111111111111111111111100
00000000000000000000000000000000000000000000000000000000000000000000000000000000000
00000000000000000000000000000000000000000000000000000000000000000000000000000000000
00000000000000000000000000000000000000000000000000000000000000000000000000000000000
00000000000000000000000000000000000000000000000000000000000000000000000000000000000
000000000000000000000000000000000000000000000000000000
>Q2G2P5_3rqt_1_469
HSSGKDLNISLPLKTKSIAPYETDVPVKIGAAESLFKTNDQGKIEKALVKSYHQPNDTTLDIE
LKDNIKFQNGQKLTAEKVKSSLENSMKKSDLVKYSLPISSITAKGQKLTIKTNSAYPELVSELA
NPFMAIYDTDAKSDVNQTPVGTGPYQIKDYKQSRKISLSNFKDYWQGKPKLDHITVTYQE
DGNNRVRNLESQKDDLITDVPVNKVQDIENNQNLKVSKESGFRTSLLMYNHTNKKMTKS
VREALDHIIDRQGIADHIYQGYAKPATSPFNDKIPYIKEPKLTKQNIEQAKMLLAKDGYTKE
HPLKIKLITYDGRPELSKIAQVLQSDAKKANIEIDIKSVDDIEGYLKDRSAWDATMYSFGTIP
RGDTGYFFNQAYKKDGAINKGDYNNSNVDDLINQLNHTVDVKERHNISNDIIKLSSRDVP
NSYIAYNDQIVAANSKVKNYKVTPEGIYLIDYRTTIER
11100000000000000000000000000000000000000000000000000000000000000000000000000000000
00000000000000000000000000000000000000000000000000000000000000000000000000000000000
00000000000000000000000000000000000000000000000000000000000000000000000000000000000
00000000000000000000000000000000000000000000000000000000000000000000000000000000000
00000000000000000000000000000000000000000000000000000000000000000000000000000000000
000000000000000000000000000000000000000000000000000001
>Q9M9P3_1z90_0_468
MAATTENLPQLKSAVDGLTEMSESEKSGFISLVSRYLSGEAQHIEWSKIQTPTDEIVVPYEKM
TPVSQDVAETKNLLDKLVVLKLNGGLGTTMGCTGPKSVIEVRDGLTFLDLIVIQIENLNNKY
GCKVPLVLMNSFNTHDDTHKIVEKYTNSNVDIHTFNQSKYPRVVADEFVPWPSKGKTDKE
GWYPPGHGDVFPALMNSGKLDTFLSQGKEYVFVANSDNLGAIVDLTILKHLIQNKNEYCM
EVTPKTLADVKGGTLISYEGKVQLLEIAQVPDEHVNEFKSIEKFKIFNTNNLWVNLKAIKKL
VEADALKMEIIPNPKEVDGVKVLQLETAAGAAIRFFDNAIGVNVPRSRFLPVKASSDLLLVQ
SDLYTLVDGFVTRNKARTNPSNPSIELGPEFKKVATFLSRFKSIPSIVELDSLKVSGDVWFGSSI

VLKGKVTVAAKSGVKLEIPDRAVVENKNINGPEDL
11111000000000000000000000000000000000001111000000000000000000000000000000000000000
00000000000000000000000000000000000000000000000000000000000000000000000000000000000
00000000000000000000000000000000000000000000000000000000000000000000000000000000000
00000000000000000000000000000000000000000000000000000000000000000000000000000000000
00000000000000000000000000000000000000000000000000000000000000000000000000000000000
000000000000000000000000000000000000000000000000000000
>P00350_2zya_0_467
MSKQQIGVVGMAVMGRNLALNIESRGYTVSIFNRSREKTEEVIAENPGKKLVPYYTVKEFVE
SLETPRRILLMVKAGAGTDAAIDSLKPYLDKGDIIIDGGNTFFQDTIRRNRELSAEGFNFIGT
GVSGGEEGALKGPSIMPGGQKEAYELVAPILTKIAAVAEDGEPCVTYIGADGAGHYVKMVH
NGIEYGDMQLIAEAYSLLKGGLNLTNEELAQTFTEWNNGELSSYLIDITKDIFTKKDEDGNY
LVDVILDEAANKGTGKWTSQSALDLGEPLSLITESVFARYISSLKDQRVAASKVLSGPQAQP
AGDKAEFIEKVRRALYLGKIVSYAQGFSQLRAASEEYNWDLNYGEIAKIFRAGCIIRAQFLQ
KITDAYAENPQIANLLLAPYFKQIADDYQQALRDVVAYAVQNGIPVPTFSAAVAYYDSYRA
AVLPANLIQAQRDYFGAHTYKRIDKEGVFHTEWLD
10000000000000000000000000000000000000000000000000000000000000000000000000000000000
00000000000000000000000000000000000000000000000000000000000000000000000000000000000
00000000000000000000000000000000000000000000000000000000000000000000000000000000000
00000000000000000000000000000000000000000000000000000000000000000000000000000000000
00000000000000000000000000000000000000000000000000000000000000000000000000000000000
00000000000000000000000000000000000000000000000000001
>Q8DP07_2wwc_33_500
NETEVAKTSQDTTTASSSSEQNQSSNKTQTSAEVQTNAAAYWDGDYYVKDDGSKAQSEWI
FDNYYKAWFYINSDGRYSQNEWHGNYYLKSGGYMAQNEWIYDSNYKSWFYLKSDGAYA
HQEWQLIGNKWYYFKKWGYMAKSQWQGSYFLNGQGAMIQNEWLYDPAYSAYFYLKSDG
TYANQEWQKVGGKWYYFKKWGYMARNEWQGNYYLTGSGAMATDEVIMDGARYIFAAS
GELKEKKDLNVGWVHRDGKRYFFNNREEQVGTEHAKKIIDISEHNGRINDWKKVIDENK
VDGVIVRLGYSGKEDKELAHNIKELNRLGIPYGVYLYTYAENETDAENDAKQTIELIKKYN
MNLSYPIYYDVENWEYVNKSKRAPSDTDTWVKIINKYMDTMKQAGYQNVYVYSYRSLLQ
TRLKHPDILKHVNWVAAYTNALEWENPYYSGEKGWQYTSSEYMKGIQGRVDVSVWY
11111111111111111111111111111111111110000000000000000000000000000000000000000000000
00000000000000000000000000000000000000000000000000000000000000000000000000000000000
00000000000000000000000000000000000000000000000000000000000000000000000000000000000
00000000000000000000000000000000000000000000000000000000000000000000000000000000000
00000000000000000000000000000000000000000000000000000000000000000000000000000000000
00000000000000000000000000000000000000000000000000000
>O88483_2pnq_71_537
ASTPQKFYLTPPQVNSILKANEYSFKVPEFDGKNVSSILGFDSNRLPANAPIEDRRSATTCLQ
TRGMLLGVFDGHAGCACSQAVSERLFYYIAVSLLPHETLLEIENAVESGRALLPILQWHKHP
NDYFSKEASKLYFNGLRTYWQELIDLNTGESADIDVKEALINAFKRLDNDISLEAQVGDPN
SFLNYLVLRVAFSGATACVAHVDGVDLHVANTGDSRAMLGVQEEDGSWSAVTLSNDHNA
QNERELQRLKLEHPKNEAKSVVKQDRLLGLLMPFRAFGDVKFKWSIDLQKRVIESGPDQL
NDNEYTKFIPPNYHTPPYLTAEPEVTYHRLRPQDKFLVLATDGLWETMHRQDVVRIVGEYL
TGMHHQQPIAVGGYKVTLGQMHGLLTERRAKMSSVFEDQNAATHLIRHAVGNNEFGAV
DHERLSKMLSLPEELARMYRDDITIIVVQFNSHVVGAYQNQEQ
11111111000000000000000000000000000000000000000000000000000000000000000000000000000
00000000000000000000000000011110000000000000000000000000000000000000111111000000000
00000000000000000000000000000000000000000000000000000000000000000000000000000000000
00000000000000000000000000000000000000000000000000000011111111111000000000000000000
00000000000000000000000000000000000000000111111111111111111111111111111100000000000

00111111111111111111111111000000000000000000000000
>P32558_3biq_0_464
MEELNIDFDVFKKRIELLYSKYNEFEGSPNSLLFVLGSSNAENPYQKTTILHNWLLSYEFPAT
LIALVPGKVIIITSSAKAKHLQKAIDLFKDPESKITLELWQRNNKEPELNKKLFDDVIALINSA
GKTVGIPEKDSYQGKFMTEWNPVWEAAVKENEFNVIDISLGLSKVWEVKDVNEQAFLSVS
SKGSDKFMDLLSNEMVRAVDEELKITNAKLSDKIENKIDDVKFLKQLSPDLSALCPPNYKF
NFDLLDWTYSPIIQSGKKFDLRVSARSTNDQLYGNGCILASCGIRYNNYCSNITRTFLIDPSEE
MANNYDFLLTLQKEIVTNILKPGRTPKEVYESVIEYIEKTKPELVPNFTKNIGSLIGLEFRDSN
FILNVKNDYRKIQRGDCFNISFGFNNLKDSQSANNYALQLADTVQIPLDETEPPRFLTNYTK
AKSQISFYFNNEEEDNNKKKSSPATKV
11110000000000000000000000000000000000000000000000000000000000000000000000000000
00000000001100000000000000000000000000000000000000000000000000000000000000000000
00000000000000000000000000000000000000000000000000000000000000000000000000000000
00000000000000000000000000000000000000000000000000000000000000000000000000000000
00000000000000000000000000000000000000000000000000000000000000000000000000000000
0000000000000000000000000000000111111111111111111
>P18477_3uxf_27_490
GAAAPADPNGSTIDPDAATTLTVHKCEQTDTNGVKEGTGNEDPQAECKPVSDVEFTITKL
NVDLTTYDGWKTLADLKGDVVKAGALKSTTVQKITTGANGLASFTDAQTEVGAYLVSETR
TPDKVIPAEDFVVTLPMTNPQDTAKWNYNVHVYPKNTLSGVDKQVTDKPAPGSGRDITYT
ITTSIPKVDYPGGARIKRYEVVDRLDKRIKKEALTPVVKIVGQNEVTLAETTDYTLITAEGKD
HNWATIQLTEEGRRKASEARYNGNGETKLQVTLNAKFDAAVNLEGDLSNTAGLIPNDSPN
FTWDPNNPGTTTDIPGIPTTPVLSKYGKVVLTKTGTDDLADKTKYNGAQFQVYECTKTASG
ATLRDSDPSTQTVDPLTIGGEKTFTTAGQGTVEINYLRANDYVNGAKKDQLTDEDYYCLVE
TKAPEGYNLQADPLPFRVLAEKAEKKAATEVTVTDIPKN
11111100000000000000000000001111110000000000000000000000000000000000000000000000
00000000000000000000000000000000000000000000000000000000000000000000000000000000
00000000000000000000000000000000000000000000000000000000000000000000000000000000
00000000000000000000000000000000000000000000000000000000000000000000000000000000
00000000000000000000000000000000000000000000000000000000000000000000000000000000
000000000000000000000000000000000000000000000001
>A7LZS1_3p2c_25_486
SNRMTEMHVCLADAIQKDNRPEISNRLFRSNAVEKEILRVQKLLKNAKLAWMFTNCFPNT
LDTTVHFRKGSDGKPDTFVYTGDIHAMWLRDSGAQVWPYVQLANSDPELKEMLAGVILR
QFKCINIDPYANAFNDGAIPDGHWMSDLTDMKPELHERKWEIDSLCYPLRLAYHYWKTTG
DASIFNEEWIQAITNVLKTFKEQQRKDGVGPYKFQRKTERALDTVSNDGLGAPVKPVGLIV
SSFRPSDDATTLQFLVPSNFFAVSSLRKAAEILEKVNKKTALSKECKDLAQEVETALKKYAVY
NHPKYGKIYAFEVDGFGNHHLMDDANVPSLLAMPYLGDVNVNDPIYQNTRRFVWSEDN
PYFFKGKAGEGIGGPHIGYDMVWPMSIMMKAFTSQNDAEIKTCIKMLMDTDAGTGFMHE
SFHKDNPKKFTRAWFAWQNTLFGELILKLVNEGKVDLLNSIQ
11111111111111111000000000000000000000000000000000000000000000000000000000000000
00000000000000000000000000000000000000000000000000000000000000000000000000000000
00000000000000000000000000000000000000000000000000000000000000000000000000000000
00000000000000000000000000000000000000000000000000000000000000000000000000000000
00000000000000000000000000000000000000000000000000000000000000000000000000000000
00000000000000000000000000000000000000000000000
>Q8PYK9_2xry_2_463
MNPKRIRALKSGKQGDGPVVYWMSRDQRAEDNWALLFSRAIAKEANVPVVVVFCLTDEF
LEAGIRQYEFMLKGLQELEVSLSRKKIPSFFLRGDPGEKISRFVKDYNAGTLVTDFSPLRIKNQ
WIEKVISGISIPFFEVDAHNVVPCWEASQKHEYAAHTFRPKLYALLPEFLEEFPELEPNSVTPE
LSAGAGMVETLSDVLETGVKALLPERALLKNKDPLFEPWHFEPGEKAAKKVMESFIADRL

DSYGALRNDPTKNMLSNLSPYLHFGQISSQRVVLEVEKAESNPGSKKAFLDEILIWKEISDN
FCYYNPGYDGFESFPSWAKESLNAHRNDVRSHIYTLEEFEAGKTHDPLWNASQMELLSTG
KMHGYMRMYWAKKILEWSESPEKALEIAICLNDRYELDGRDPNGYAGIAWSIGGVHDRA
WGEREVTGKIRYMSYEGCKRKFDVKLYIEKYSAL
00000000000000000000000000000000000000000000000000000000000000000000000000000000000
00000000000000000000000000000000000000000000000000000000000000000000000000000000000
00000000000000000000111111111100000000000000000000000000000000000000000000000000000
00000000000000000000000000000000000000000000000000000000000000000000000000000000000
00000000000000000000000000000000000000000000000000000000000000000000000000000000000
00000000000000000000000000000000000000000000011
>P13489_2q4g_0_460
MSLDIQSLDIQCEELSDARWAELLPLLQQCQVVRLDDCGLTEARCKDISSALRVNPALAEL
NLRSNELGDVGVHCVLQGLQTPSCKIQKLSLQNCCLTGAGCGVLSSTLRTLPTLQELHLSD
NLLGDAGLQLLCEGLLDPQCRLEKLQLEYCSLSAASCEPLASVLRAKPDFKELTVSNNDINE
AGVRVLCQGLKDSPCQLEALKLESCGVTSDNCRDLCGIVASKASLRELALGSNKLGDVGM
AELCPGLLHPSSRLRTLWIWECGITAKGCGDLCRVLRAKESLKELSLAGNELGDEGARLLCE
TLLEPGCQLESLWVKSCSFTAACCSHFSSVLAQNRFLLELQISNNRLEDAGVRELCQGLGQP
GSVLRVLWLADCDVSDSSCSSLAATLLANHSLRELDLSNNCLGDAGILQLVESVRQPGCLL
EQLVLYDIYWSEEMEDRLQALEKDKPSLRVIS
10000000000000000000000000000000000000000000000000000000000000000000000000000000000
00000000000000000000000000000000000000000000000000000000000000000000000000000000000
00000000000000000000000000000000000000000000000000000000000000000000000000000000000
00000000000000000000000000000000000000000000000000000000000000000000000000000000000
00000000000000000000000000000000000000000000000000000000000000000000000000000000000
000000000000000000000000000000000000000000000
>Q4W8M3_1wky_29_489
ANANSGFYVSGTTLYDANGNPFVMRGINHGHAWYKDQATTAIEGIANTGANTVRIVLSD
GGQWTKDDIQTVRNLISLAEDNNLVAVLEVHDATGYDSIASLNRAVDYWIEMRSALIGKED
TVIINIANEWFGSWDGAAWADGYKQAIPRLRNAGLNNTLMIDAAGWGQFPQSIHDYGRE
VFNADPQRNTMFSIHMYEYAGGNASQVRTNIDRVLNQDLALVIGEFGHRHTNGDVDESTI
MSYSEQRGVGWLAWSWKGNGPEWEYLDLSNDWAGNNLTAWGNTIVNGPYGLRETSKLS
TVFTGGGSDGRTSPTTLYDFEESTQGWTGSSLSRGPWTVTEWSSKGNHSLKADIQMSSNSQ
HYLHVIQNRSLQQNSRIQATVKHANWGSVGNGMTARLYVKTGHGYTWYSGSFVPINGSS
GTTLSLDLSNVQNLSQVREIGVQFQSESNSSGQTSIYIDNVIVE
00110000000000000000000000000000000000000000000000000000000000000000000000000000000
00000000000000000000000000000000000000000000000000000000000000000000000000000000000
00000000000000000000000000000000000000000000000000000000000000000000000000000000000
00000000000000000000000000000000000000000000000000001111111111000000000000000000000
00000000000000000000000000000000000000000000000000111111110000000000000000000000000
000000000000000000000000000000000000000000000
>O33853_2osw_30_489
SGSGSGSGTALTPSYLKDDDGRSLILRGFNTASSAKSAPDGMPQFTEADLAREYADMGTNF
VRFLISWRSVEPAPGVYDQQYLDRVEDRVGWYAERGYKVMLDMHQDVYSGAITPEGNSG
NGAGAIGNGAPAWATYMDGLPVEPQPRWELYYIQPGVMRAFDNFWNTTGKHPELVEHY
AKAWRAVADRFADNDAVVAYDLMNEPFGGSLQGPAFEAGPLAAMYQRTTDAIRQVDQD
TWVCVAPQAIGVNQGLPSGLTKIDDPRAGQQRIAYCPHLYPLPLDIGDGHEGLARTLTDVTI
DAWRANTAHTARVLGDVPIILGEFGLDTTLPGARDYIERVYGTAREMGAGVSYWSSDPGP
WGPYLPDGTQTLLVDTLNKPYPRAVAGTPTEWSSTSDRLQLTIEPDAAITAPTEIYLPEAGFP
GDVHVEGADVVGWDRQSRLLTVRTPADSGNVTVTVTPAA
11111111111100000000000000000000000000000000000000000000000000000000000000000000000
00000000000000000000000000000000111111000000000000000000000000000000000000000000000

00000000000000000000000000000000000000000000000000000000000000000000000000000000
00000000000000000000000000000001111000000000000000000000000000000000000000000000
00000000000000000000000000000000000000000000000000000000000000000000000000000000
00000000000000000000000000000000000000000000
>Q60187_3ssa_0_459
MAKEYKTITQIAGPLIFVEKTEPVGYNEIVNIKMGDGTVRRGQVLDSSADIVVVQVFEGTGG
LDKDCGVIFTGETLKLPASVDLLGRILSGSGEPRDGGPRIVPDQLLDINGAAMNPYARLPPK
DFIQTGISTIDGTNTLVRGQKLPIFSASGLPHNEIALQIARQASVPGSESAFAVVFAAMGITNE
EAQYFMSDFEKTGALERAVVFLNLADDPAVERIVTPRMALTAAEYLAYEHGMHVLVILTDI
TNYAEALRQMGAARNEVPGRRGYPGYMYTDLATLYERAGIVKGAKGSVTQIPILSMPGDDI
THPIPDLSGYITEGQIVVARELHRKGIYPPINVLPSLSRLMNSGIGAGKTREDHKAVSDQMYA
GYAEGRDLRGLVAIVGKEALSERDTKFLEFADLFEDKFVRQGRNENRTIEDTLEIGWQILTH
LPENQLGRIDNKYIQKYHPAHRKAK
11111111111000000000000000000000000000000000000000000000011111111000000000000000
00000000000000000000000000000000000000000000000000000000000000000000000000000000
00000000000000000000000000000000000000000000000000000000000000000000000000000000
00000000000000000000000000000000000000000000000000000000000000000000000000000000
00000000000000000000000000000000000000000000000000000000000000000000000000000000
00000000000000000000000000000000000000000000
>Q8GAG1_2bvf_0_458
MVSSKLATPLSIQGEVIYPDDSGFDAIANIWDGRHLQRPSLIARCLSAGDVAKSVRYACDNG
LEISVRSGGHNPNGYATNDGGIVLDLRLMNSIHIDTAGSRARIGGGVISGDLVKEAAKFGLA
AVTGMHPKVGFCGLALNGGVGFLTPKYGLASDNILGATLVTATGDVIYCSDDERPELFWAV
RGAGPNFGVVTEVEVQLYELPRKMLAGFITWAPSVSELAGLLTSLLDALNEMADHIYPSVF
VGVDENRAPSVTVCVGHLGGLDIAERDIARLRGLGRTVSDSIAVRSYDEVVALNAEVGSFE
DGMSNLWIDREIAMPNARFAEAIAGNLDKFVSEPASGGSVKLEIEGMPFGNPKRTPARHRD
AMGVLALAEWSGAAPGSEKYPELARELDAALLRAGVTTSGFGLLNNNSEVTAEMVAEVY
KPEVYCRLAAVKREYDPENRFRHNYNIDPEGS
11110000000000000000000000000000000000000000000000000000000000000000000000000000
00000000000000000000000000000000000000000000000000000000000000000000000000000000
00000000000000000000000000000000000000000000000000000000000000000000000000000000
00000000000000000000000000000000000000000000000000000000000000000000000000000000
00000000000000000000000000000000000000000000000000000000000000000000000000000000
0000000000000000000000000000000000000000011
>Q01415_2a2c_0_457
MATESPATRRVQVAEHPRLLKLKEMFNSKFGSIPKFYVRAPGRVNIIGEHIDYCGYSVLPMA
VEQDVLIAVEPVKTYALQLANTNPLYPDFSTSANNIQIDKTKPLWHNYFLCGLKGIQEHFGL
SNLTGMNCLVDGNIPPSSGLSSSSALVCCAGLVTLTVLGRNLSKVELAEICAKSERYIGTEGG
GMDQSISFLAEEGTAKLIEFSPLRATDVKLPSGAVFVIANSCVEMNKAATSHFNIRVMECRL
AAKLLAKYKSLQWDKVLRLEEVQAKLGISLEEMLLVTEDALHPEPYNPEEICRCLGISLEEL
RTQILSPNTQDVLIFKLYQRAKHVYSEAARVLQFKKICEEAPENMVQLLGELMNQSHMSCR
DMYECSCPELDQLVDICRKFGAQGSRLTGAGWGGCTVSMVPADKLPSFLANVHKAYYQRS
DGSLAPEKQSLFATKPGGGALVLLEA
10000000000000000000000000000000000000000000000000000000000000000000000000000000
00000000000000110000000000000000000000000000000000000000000000000000000000000000
00000000000000000000000000000000000000000000000000000000000000000000000000000000
00000000000000000000000000000000000000000000000000000000000000000000000000000000
00000000000000000000000000000000000000000000000000000000000000000000000000000000
0000000000000001111111110000000000000000000
>Q83DX0_3ij3_0_457
MADCYLTEKKKNFIPIQPMMPDELPDWLDTQDARTQQWVKASGFVGLAGTICSIPESTGA

LQRVLLGVSDYEYSWDFGGLSKVLPPGAFQLNRDDFEDDEYYERALLAFGLGSYQFNAYRK
RSPYLAKLFLPQAHRKRVTDWLTTIYLIRDLINTPAEDMGPSELAQAVKHVAKEFEAKVKIIE
SKDLETEFPAIYAVGRAGSRPPLLIDLKWGDIKAPKVTLVGKGVCFDSGGLDIKTPGGMLLM
KKDMGGAAHALGLARMIMLQQLPVRLRLLIPAVENAIGSRSYRPGDVVQTRARKTIEITNT
DAEGRVVLADALAEAVKEDPDLIIDFSTLTGAARIALGPNLPALFANQDSLAQALIDASLKT
DDPLWRLPLFQPYRNYLKSEVADLTNSSQNRMAGAITAALFLQHFVSDQIPWAHFDIFAW
NLEDLPGRPIGGEAMALRAVFHYLEQQYR
100000000000000000000000000000000000000000000000000000000000000000000000000000000000
000000000000000000000000000000000000000000000000000000000000000000000000000000000000
000000000000000000000000000000000000000000000000000000000000000000000000000000000000
000000000000000000000000000000000000000000000000000000000000000000000000000000000000
000000000000000000000000000000000000000000000000000000000000000000000000000000000000
0000000000000000000000000000000000000000000
>Q8KRB7_2xep_8_465
MMKKADSVPTPAEAALAAQTALAADDSPMGDAARWAMGLLTSSGLPRPEDVAARFIPTF
AAAGNFAETVREWRSKGPFTVRAYHPVAHKGWVVLSAPAGVRYILSLTLDSSGLIRILTLKP
ETVIPDMVTWNDVEETLHTPGVQHSVYAVRLTPDGHEVLHASAPERPMPTGSAYKLYLMR
ALVAEIEKGTVGWDEILTLTPELRSLPTGDMQDLPDGTRVTVRETAHKMIALSDNTGADLV
ADRLGREVVERSLAAAGHHDPSLMRPFLTSHEVFELGWGDPERRAEWVRQDEAGRRELL
EKMAGVMTVRGSDLGATVHQLGIDWHMDAFDVVRVLEGLLQDSGRDTSGTVEEILTAYP
GLLIDEERWRRVYFKAGSSPGVMMFCWLLQDHAGISYVLVLRQSADEQRLIGDGLFLRGIG
AKIIEAEAKLLSSGERRGAGTAAAGDDRASAGEAARR
111111000000000000000000000000000000000000000000000000001111000000000000000000000000
000000000000000000000000000000000000000000000000000000000000000000000000000000000000
000000000000000000000000000000000000000000000000000000000000000000000000000000000000
000000000000000000000000000000000000000000000000000000000000000000000000000000000000
000000000000000000000000000000000000000000000000000000000000000000000000000000000000
0000000000000000000011111111111111111111111
>P24228_2exb_20_476
ANVDEYITQLPAGANLALMVQKVGASAPAIDYHSQQMALPASTQKVITALAALIQLGPDF
RFTTTLETKGNVENGVLKGDLVARFGADPTLKRQDIRNMVATLKKSGVNQIDGNVLIDTSI
FASHDKAPGWPWNDMTQCFSAPPAAAIVDRNCFSVSLYSAPKPGDMAFIRVASYYPVTMF
SQVRTLPRGSAEAQYCELDVVPGDLNRFTLTGCLPQRSEPLPLAFAVQDGASYAGAILKDEL
KQAGITWSGTLLRQTQVNEPGTVVASKQSAPLHDLLKIMLKKSDNMIADTVFRMIGHARF
NVPGTWRAGSDAVRQILRQQAGVDIGNTIIADGSGLSRHNLIAPATMMQVLQYIAQHDNE
LNFISMLPLAGYDGSLQYRAGLHQAGVDGKVSAKTGSLQGVYNLAGFITTASGQRMAFVQ
YLSGYAVEPADQRNRRIPLVRFESRLYKDIYQNN
111000000000000000000000000000000000000000000000000000000000000000000000000000000000
000000000000000000000000000000000000000000000000000000000000000000000000000000000000
000000000000000000000000000000000000000000000000000000000000000000000000000000000000
000000000000000000000000000000000000000000000000000000000000000000000000000000000000
000000000000000000000000000000000000000000000000000000000000000000000000000000000000
000000000000000111111110000000000000000000
>D5DC07_3om2_28_483
AKGNDSKDFNNSYGISHITRDNMVKIPQQQNSDQFKVPAFDESTIKNIASAKGKNASGNTI
DLDVWDSWPLQNADGTVATYHGYQIVFALAGDPKDSNDTSVYLFYKKAGDKSIDSWKNA
GRVFKDSDKFVPNDPHLKNQTQEWSGSGTLTKDGKVRLFYTDYSGKQYGKQTLTTAQVN
MSQPNDNTLKVDGVEDYKSIFDGDGKIYQTVQQFIDEGGYDTGDNHTLRDPHYIEDNGH
KYLVFEANTGTEDGYQGEDSLYNRAYYGGNNPFFQSEKKKLLEGSNKEKASLANGALGIIE
LNDDYTLKKVMKPLITSNTVTDEIERANIFKKDGKWYLFTDSRGSKMTIDGIGQDDVYMLG
YVSNTLTGKYKPLNDTGLVLHMDLDPNDKTFTYSHFAVPQTKGDNVVITSYMTNRGFYED

NHSTFAPSFLVNIDGSKTSVVKDRVLEQGQLTVDED
11111000000000000000000000000000000000000000000000000000000000000000000000000000000
00000000000000000000000000000000000000000000000000000000000000000000000000000000000
00000000000000000000000000000000000000000000000000000000000000000000000000000000000
00000000000000000000000000000000000000000000000000000000000000000000000000000000000
00000000000000000000000000000000000000000000000000000000000000000000000000000000000
0000000000000000000000000000000000000111
>O05783_1lqt_0_455
MRPYYIAIVGSGPSAFFAAASLLKAADTTEDLDMAVDMLEMLPTPWGLVRSGVAPDHPKIK
SISKQFEKTAEDPRFRFFGNVVVGEHVQPGELSERYDAVIYAVGAQSDRMLNIPGEDLPGSIA
AVDFVGWYNAHPHFEQVSPDLSGARAVVIGNGNVALDVARILLTDPDVLARTDIADHALE
SLRPRGIQEVVIVGRRGPLQAAFTTLELRELADLDGVDVVIDPAELDGITDEDAAAVGKVC
KQNIKVLRGYADREPRPGHRRMVFRFLTSPIEIKGKRKVERIVLGRNELVSDGSGRVAAKDT
GEREELPAQLVVRSVGYRGVPTPGLPFDDQSGTIPNVGGRINGSPNEYVVGWIKRGPTGVIG
TNKKDAQDTVDTLIKNLGNAKEGAECKSFPEDHADQVADWLAARQPKLVTSAHWQVID
AFERAAGEPHGRPRVKLASLAELLRIGLG
10000000000000000000000000000000000000000000000000000000000000000000000000000000000
00000000000000000000000000000000000000000000000000000000000000000000000000000000000
00000000000000000000000000000000000000000000000000000000000000000000000000000000000
00000000000000000000000000000000000000000000000000000000000000000000000000000000000
00000000000000000000000000000000000000000000000000000000000000000001000000000000000
0000000000000000000000000000000000000000
>Q5SII2_3m6v_0_455
MLPKAFLSRMAELLGEEFPAFLKALTEGKRTYGLRVNTLKLPPEAFQRISPWPLRPIPWCQE
GFYYPEEARPGPHPFFYAGLYYIQEPSAQAVGVLLDPKPGERVLDLAAAPGGKTTHLAARM
GGKGLLLANEVDGKRVRGLLENVERWGAPLAVTQAPPRALAEAFGTYFHRVLLDAPCSG
EGMFRKDREAARHWGPSAPKRMAEVQKALLAQASRLLGPGGVLVYSTCTFAPEENEGVV
AHFLKAHPEFRLEDARLHPLFAPGVPEWGEGNPELLKTARLWPHRLEGEGHFLARFRKEG
GAWSTPRLERPSPLSQEALRAFRGFLEEAGLTLEGPVLDRAGHLYLLPEGLPTLLGLKAPAP
GLYLGKVQKGRFLPARALALAFGATLPWPEGLPRLALTPEDPRALAFATGEGVAWEGEDH
PLALVVLKTAAGEFPLDFGKAKRGVLRPVGVGL
10000000000000000000000000000000000000000000000000000000000000000000000000000000000
00000000000000000000000000000000000000000000000000000000000000000000000000000000000
00000000000000000000000000000000000000000000000000000000000000000000000000000000000
00000000000000000000000000000000000000000000000000000000000000000000000000000000000
00000000000000000000000000000000000000000000000000000000000000000000000000000000000
0000000000000000000000000000000000000000
>Q5ZXD1_3bhg_0_455
MTLTALNAISPIDGRYVNKTRALSPYFSEFALTYYRLMVEIKWFESLAANDTIPEVPALDNK
ARKFLSDLISNFNESEAEKIKEFEKQTNHDVKAVEYYLQDKFQENEQLKSCVAFIHFACTSE
DINNLAYALMIKQAIAQVIQPTIAEIMGSITLLGKQHADVAMLSRTHGQPATPTTMGKELV
NFVARLKRPQQQLAEVLIPAKFNGAVGNYNAHVAAYPEVDWRKHCANFVTSLGLSFNAY
TTQIEPHDGIAEVSQIMVRINNILLDYTQDIWSYISLGYFKQKTIAEEVGSSTMPHKVNPIDFE
NAEGNLGLSNALFIHFANKLTQSRMQRDLSDSTVLRNLGVAFSYSLIAYHSVAKGNDKLQI
NKSALQKDLSENWEVLAEAIQTVMRRYNEPNAYEQLKELTRGQMIDAENLKKFIKTLSIPE
EAKAELMKLTPETYTGLATQLVKAFS
11000000000000000000000000000000000000000000000000000000000000000000000000000000000
00000000000000000000000000000000000000000000000000000000000000000000000000000000000
00000000000000000000000000000000000000000000000000000000000000000000000000000000000
00000000000000000000000000000000000000011111100000000000000000000000000000000000000
00000000000000000000000000000000000000000000000000000000000000000000000000000000000

00000000000000000000000000000000000000000
>Q6A1F6_2nx9_0_455
MTQAIKRVGVTDVVLRDAHQSLFATRLRIDDMLPIAQQLDQIGYWSLECWGGATFDSCIRF
LGEDPWQRLRLLKQAMPNTPLQMLLRGQNLLGYRHYADDVVDTFVERAVKNGMDVFRV
FDAMNDVRNMQQALQAVKKMGAHAQGTLCYTTSPVHNLQTWVDVAQQLAELGVDSIA
LKDMAGILTPYAAEELVSTLKKQVDVELHLHCHSTAGLADMTLLKAIEAGVDRVDTAISSM
SGTYGHPATESLVATLQGTGYDTGLDIAKLEQIAAYFRDVRKKYHAFEGMMKGSDARILVA
QVPGGMLTNMESQLKQQNALDKLDLVLEEIPRVREELGFLPLVTPTSQIVGTQAVINVVLGE
RYKTITKETSGVLKGEYGKTPAPVNTELQARVLAGAEAITCRPADLIAAEMPTLQDRVLQQ
AKEQHITLAENAIDDVLTIALFDQVGWKFLANRHN
10000000000000000000000000000000000000000000000000000000000000000000000000000000000
00000000000000000000000000000000000000000000000000000000000000000000000000000000000
00000000000000000000000000000000000000000000000000000000000000000000000000000000000
00000000000000000000000000000000000000000000000000000000000000000000000000000000000
00000000000000000000000000000000000000000000000000000000000000000000000000000000000
00000000000000000000000000000000000000011
>Q6SJY7_3ls9_13_468
MILIRGLTRVITFDDQERELEDADILIDGPKIVAVGKDLSDRSVSRTIDGRGMIALPGLINSHQ
HLYEGAMRAIPQLERVTMASWLEGVLTRSAGWWRDGKFGPDVIREVARAVLLESLLGGITT
VADQHLFFPGATADSYIDATIEAATDLGIRFHAARSSMTLGKSEGGFCDDLFVEPVDRVVQ
HCLGLIDQYHEPEPFGMVRIALGPCGVPYDKPELFEAFAQMAADYDVRLHTHFYEPLDAG
MSDHLYGMTPWRFLEKHGWASDRVWLAHAVVPPREEIPEFADAGVAIAHLIAPDLRMGW
GLAPIREYLDAGITVGFGTTGSASNDGGNLLGDLRLAALAHRPADPNEPEKWLSARELLR
MATRGSAECLGRPDLGVLEEGRAADIACWRLDGVDRVGVHDPAIGLIMTGLSDRASLVVV
NGQVLVENERPVLADLERIVANTTALIPKNL
00000000000000000000000000000000000000000000000000000000000000000000000000000000000
00000000000000000000000000000000000000000000000000000000000000000000000000000000000
00000000000000000000000000000000000000000000000000000000000000000000000000000000000
00000000000000000000000000000000000000000000000000000000000000000000000000000000000
00000000000000000000000000000000000000000000000000000000000000000000000000000000000
00000000000000000000000000000000000000111
>Q9V2Z6_1ua4_0_454
MPTWEELYKNAIEKAIKSVPKVKGVLLGYNTNIDAIKYLDSKDLEERIIKAGKEEVIKYSEEL
PDKINTVSQLLGSILWSIRRGKAAELFVESCPVRFYMKRWGWNELRMGGQAGIMANLLGG
VYGVPVIVHVPQLSRLQANLFLDGPIYVPTLENGEVKLIHPKEFSGDEENCIHYIYEFPRGFR
VFEFEAPRENRFIGSADDYNTTLFIREEFRESFSEVIKNVQLAILSGLQALTKENYKEPFEIVKS
NLEVLNEREIPVHLEFAFTPDEKVREEILNVLGMFYSVGLNEVELASIMEILGEKKLAKELLA
HDPVDPIAVTEAMLKLAKKTGVKRIHFHTYGYYLALTEYKGEHVRDALLFAALAAAAKA
MKGNITSLEEIREATSVPVNEKATQVEEKLRAEYGIKEGIGEVEGYQIAFIPTKIVAKPKSTVGI
GDTISSSAFIGEFSFTL
10000000000000000000000000000000000000000000000000000000000000000000000000000000000
00000000000000000000000000000000000000000000000000000000000000000000000000000000000
00000000000000000000000000000000000000000000000000000000000000000000000000000000000
00000000000000000000000000000000000000000000000000000000000000000000000000000000000
00000000000000000000000000000000000000000000000000000000000000000000000000000000000
0000000000000000000000000000000000000000
>P46925_2bju_0_452
MDITVREHDFKHGFIKSNSTFDGLNIDNSKNKKKIQKGFQILYVLLFCSVMCGLFYYVYENV
WLQRDNEMNEILKNSEHLTIGFKVENAHDRILKTIKTHKLKNYIKESVNFLNSGLTKTNYL
GSSNDNIELVDFQNIMFYGDAEVGDNQQPFTFILDTGSANLWVPSVKCTTAGCLTKHLYDS
SKSRTYEKDGTKVEMNYVSGTVSGFFSKDLVTVGNLSLPYKFIEVIDTNGFEPTYTASTFDGIL

GLGWKDLSIGSVDPIVVELKNQNKIENALFTFYLPVHDKHTGFLTIGGIEERFYEGPLTYEKL
NHDLYWQITLDAHVGNIMLEKANCIVDSGTSAITVPTDFLNKMLQNLDVIKVPFLPFYVTL
CNNSKLPTFEFTSENGKYTLEPEYYLQHIEDVGPGLCMLNIIGLDFPVPTFILGDPFMRKYFT
VFDYDNHSVGIALAKKNL
11111111111111111111111111111111111111111111111111111111111111111111111111111111111
11111111111111111111111111111111111111110000000000000000000000000000000000000000000
00000000000000000000000000000000000000000000000000000000000000000000000000000000000
00000000000000000000000000000000000000000000000000000000000000000000000000000000000
00000000000000000000000000000000000000000000000000000000000000000000000000000000000
00000000000000000000000000000000000000
>Q9HU77_3mdw_0_452
MSAIFAERALLPEGWARNVRFEISADGVLAEIRPDANADGAERLGGAVLPGMPNLHSHAF
QRAMAGLAEVAGNPNDSFWTWRELMYRMVARLSPEQIEVIACQLYIEMLKAGYTAVAEFH
YVHHDLDGRSYADPAELSLRISRAASAAGIGLTLLPVLYSHAGFGGQPASEGQRRFINGSEA
YLELLQRLRAPLEAAGHSLGLCFHSLRAVTPQQIATVLAAGHDDLPVHIHIAEQQKEVDDC
QAWSGRRPLQWLYENVAVDQRWCLVHATHADPAEVAAMARSGAVAGLCLSTEANLGDG
IFPATDFLAQGGRLGIGSDSHVSLSVVEELRWLEYGQRLRDRKRNRLYRDDQPMIGRTLYDA
ALAGGAQALGQPIGSLAVGRRADLLVLDGNDPYLASAEGDALLNRWLFAGGDRQVRDV
MVAGRWVVRDGRHAGEERSARAFVQVLGELLD
11000000000000000000000000000000000000000000000000000000000000000000000000000000000
00000000000000000000000000000000000000000000000000000000000000000000000000000000000
00000000000000000000000000000000000000000000000000000000000000000000000000000000000
00000000000000000000000000000000000000000000000000000000000000000000000000000000000
00000000000000000000000000000000000000000000000000000000000000000000000000000000000
00000000000000000000000000000000000011
>B7AM82_4ftd_22_473
DEDYPKSHIEPYDTELLSIKILNAGANGDKVVEGTIDEAKKTINFPRLDVETDFSALSIEAELS
EGAALQSEVMDYSMDAETNEKTQVLRIINHNRYKDYLMKVRKRVPVFGADFEKPTVYNFS
GDNIYSDFATNYTRCASYDGEHVLVVSRPTTPNFHTPHLLKVSDLKRGEIKPIMLDVTGVKG
GTYDYNMGALINGHVYLSSLSGGKVSPFKIYYWETPTSNPEVIANINVGNIPGAGNRHGDN
ASYNIDENGNGFIFFGDNAATEFLKVPISGHKTVDIGNIKVLPSKSDATMVTNVYRVGDTD
QYLWSGIRVPVTLVDESLGEKYKSKIAGEAVAPKVVTFNEERYLLVCTAGQGAASKASIALE
VYDLTKGETIEDALKKFDEGENHNPIYQFKLGGSGNGNALAQTDYYIEKDENGKDAKLCL
FASRTQSGFVICEFPIKQEEMD
11111111111100000000000000000000000000000000000000000000000000000000000000000000000
00000000000000000000000000000000000000000000000000000000000000000000000000000000000
00000000000000000000000000000000000000000000000000000000000000000000000000000000000
00000000000000000000000000000000000000000000000000000000000000000000000000000000000
00000000000000000000000000000000000000000000000000000000000000000000000000000000000
0000000000000000000000000000000000000
>P54318_1bu8_16_467
KEVCYGHLGCFSNDKPWAGMLQRPLKIFPWSPEDIDTRFLLYTNENPNNYQKISATEPDTIK
FSNFQLDRKTRFIVHGFIDKGEDGWLLDMCKKMFQVEKVNCICVDWRRGSRTEYTQASYN
TRVVGAEIAFLVQVLSTEMGYSPENVHLIGHSLGAHVVGEAGRRLEGHVGRITGLDPAEPC
FQGLPEEVRLDPSDAMFVDVIHTDSAPIIPYLGFGMSQKVGHLDFFPNGGKEMPGCQKNIL
STIVDINGIWEGTQNFVACNHLRSYKYYASSILNPDGFLGYPCSSYEKFQQNDCFPCPEEGC
PKMGHYADQFEGKTATVEQTVYLNTGDSGNFTRWRYKVSVTLSGAKKLSGYILVALYGNN
GNSKQYEIFKGSLKPEARHVRDIDVDINVGEIQKVKFLWNNKVINLFRPTLGASQITVQSGV
DGKEYNFCSSDTVREDVLQSLYPC
00000000000000000000000000000000000000000000000000000000000000000000000000000000000
00000000000000000000000000000000000000000000000000000000000000000000000000000000000

00000000000000000000000000000000000000000000000000000000000000000000000000000000000
00000000000000000000000000000000000000000000000000000000000000000000000000000000000
00000000000000000000000000000000000000000000000000000000000000000000000000001111100
0000000000000000000000000000000000000
>Q59310_2vk5_242_693
VEGAVKTEPVDLFHPGFLNSSNYRIPALFKTKEGTLIASIDARRHGGADAPNNDIDTAVRRS
EDGGKTWDEGQIIMDYPDKSSVIDTTLIQDDETGRIFLLVTHFPSKYGFWNAGLGSGFKNID
GKEYLCLYDSSGKEFTVRENVVYDKDGNKTEYTTNALGDLFKNGTKIDNINSSTAPLKAKG
TSYINLVYSDDDGKTWSEPQNINFQVKKDWMKFLGIAPGRGIQIKNGEHKGRIVVPVYYTN
EKGKQSSAVIYSDDSGKNWTIGESPNDNRKLENGKIINSKTLSDDAPQLTECQVVEMPNGQ
LKLFMRNLSGYLNIATSFDGGATWDETVEKDTNVLEPYCQLSVINYSQKVDGKDAVIFSNP
NARSRSNGTVRIGLINQVGTYENGEPKYEFDWKYNKLVKPGYYAYSCLTELSNGNIGLLYEG
TPSEEMSYIEMNLKYLESGANK
00000000000000000000000000000000000000000000000000000000000000000000000000000000000
00000000000000000000000000000000000000000000000000000000000000000000000000000000000
00000000000000000000000000000000000000000000000000000000000000000000000000000000000
00000000000000000000000000000000000000000000000000000000000000000000000000000000000
00000000000000000000000000000000000000000000000000000000000000000000000000000000000
0000000000000000000000000000000000111
>Q9F1X5_2cdu_0_451
MKVIVVGCTHAGTFAVKQTIADHPDADVTAYEMNDNISFLSCGIALYLGKEIKNNDPRGLF
YSSPEELSNLGANVQMRHQVTNVDPETKTIKVKDLITNEEKTEAYDKLIMTTGSKPTVPPIP
GIDSSRVYLCKNYNDAKKLFEEAPKAKTITIIGSGYIGAELAEAYSNQNYNVNLIDGHERVL
YKYFDKEFTDILAKDYEAHGVNLVLGSKVAAFEEVDDEIITKTLDGKEIKSDIAILCIGFRPNT
ELLKGKVAMLDNGAIITDEYMHSSNRDIFAAGDSAAVHYNPTNSNAYIPLATNAVRQGRLV
GLNLTEDKVKDMGTQSSSGLKLYGRTYVSTGINTALAKANNLKVSEVIIADNYRPEFMLST
DEVLMSLVYDPKTRVILGGALSSMHDVSQSANVLSVCIQNKNTIDDLAMVDMLFQPQFDR
PFNYLNILGQAAQAQADKAHK
00000000000000000000000000000000000000000000000000000000000000000000000000000000000
00000000000000000000000000000000000000000000000000000000000000000000000000000000000
00000000000000000000000000000000000000000000000000000000000000000000000000000000000
00000000000000000000000000000000000000000000000000000000000000000000000000000000000
00000000000000000000000000000000000000000000000000000000000000000000000000000000000
0000000000000000000000000000000000001
>O42692_2rkt_0_450
MAFKIQLDTLGQLPGLLSIYTQISLLYPVSDPSQYPTIVSTFEQGLKRFSEAVPWVAGQVKAE
GISEGNTGTSFIVPFEDVPRVVVKDLRDDPSAPTIEGMRKAGYPMAMFDENIIAPRKTLPIGP
GTGPDDPKPVILLQLNFIKGGLILTVNGQHGAMDMVGQDAVIRLLSKACRNDPFTEEEMT
AMNLDRKTIVPYLENYTIGPEVDHQIVKPDVAGGDAVLTPVSASWAFFKFSPKAMSELKDA
ATKTLDASTKFVSTDDALSAFIWKSASRVRLERIDGSAPTEFCRAVDARPAMGVSNNYPGLL
QNMTYHNSTIGEIANESLGATASRLRSELDPASMRQRTRGLATYLHNNPDKSNVSLTADAD
PSTSVMLSSWAKVGLWDYDFGFGLGKPETVRRPIFEPVESLMYFMPKKPDGEFCAALSLRD
EDMDRLKADKEWTKYAQYVG
00000000000000011100000000000000000000000000000000000000000000000000000000000000000
00000000000000000000000000000000000000000000000000000000000000000000000000000000000
00000000000000000000000000000000000000000000000000000011111000000000000000000000000
00011111100000000000000000000000000000000000000000000000000000000000000000000000000
00001100000000000000000000000000000000000000000000000000000000000000000000000000000
000000000000000000000000000000000000
>Q8E186_3lk7_0_450
MKTITTFENKKVLVLGLARSGEAAARLLAKLGAIVTVNDGKPFDENPTAQSLLEEGIKVVC

GSHPLELLDEDFCYMIKNPGIPYNNPMVKKALEKQIPVLTEVELAYLVSESQLIGITGSNGKT
TTTTMIAEVLNAGGQRGLLAGNIGFPASEVVQAANDKDTLVMELSSFQLMGVKEFRPHIA
VITNLMPTHLDYHGSFEDYVAAKWNIQNQMSSSDFLVLNFNQGISKELAKTTKATIVPFSTT
EKVDGAYVQDKQLFYKGENIMSVDDIGVPGSHNVENALATIAVAKLAGISNQVIRETLSNF
GGVKHRLQSLGKVHGISFYNDSKSTNILATQKALSGFDNTKVILIAGGLDRGNEFDELIPDIT
GLKHMVVLGESASRVKRAAQKAGVTYSDALDVRDAVHKAYEVAQQGDVILLSPANASW
DMYKNFEVRGDEFIDTFESLRGE
11100000000000000000000000000000000000000000000000000000000000000000000000000000000
00000000000000000000000000000000000000000000000000000000000000000000000000000000000
00000000000000000000000000000000000000000000000000000000000000000000000000000000000
00000000000000000000000000000000000000000000000000000000000000000000000000000000000
00000000000000000000000000000000000000000000000000000000000000000000000000000000000
000000000000000000000000000000000000
>C8W8H7_4jbf_504_953
IDAPRLQALPTNNHTIAKSAYVQRGAIITSDGVTLAESVKQDDGTYVRNYPHDGMASHTV
GYISTQYGTAGIESSMNETLTGHADHSDWRSALYSMAGINTTGSSVVLTINSQMQAVAEAA
LQGYSGSIVVMDPSTGAVLAKASSPSYTHAELGTIIESGTGSQLVDRTTQALYSPGSSFKTVTL
AAGIDTHKTTLDTTYSAPGTMEIGGGTIHNYANEDMGTIPLREAFARSSNTALAQLGVALG
ADNLVSYARAFGYGTALGQDFSTTPSLMPNPAEMTTWELAWASCGLPVGEHASPAGPQTT
VMQNAVIAAAIANGGVVMNPYIVDRVLSPEGAVVSTTSPKSLGQAVSADTAAQVREAMLG
VVESGTGMGARVPGVKIAGKTGTADVENGNFNSFFIGFAPYDHPTLVVSVVIEGNGENVLG
YGAQVGGRVLAQCLNIQALGAAS
11111111111111111100000000000000000000010001000000000000000000000000000000000000011
11110000000000000000000000000000000000000000000000000000000000000000000001111110000
00000000000000000000000000000000000000000000000000000000000000000000000000000000000
00000000000000000000000000000000000000000000000000000000000000000000000000000000000
00000000000000000000000000000000000000000000000000000000000000000000000000000000000
00000000000000000000000000000001111
>O13702_1ta9_0_449
MIGPRLCAATPRFPLVSLAHRNSKVFALASSNAVAQRWGKRFYAPIETETPHKVGVEFEESK
DRIFTSPQKYVQGRHAFTRSYMYVKKWATKSAVVLADQNVWNICANKIVDSLSQNGMTV
TKLVFGGEASLVELDKLRKQCPDDTQVIIGVGGGKTMDSAKYIAHSMNLPSIICPTTASSDA
ATSSLSVIYTPDGQFQKYSFYPLNPNLIFIDTDVIVRAPVRFLISGIGDALSTWVETESVIRSNST
SFAGGVASIAGRYIARACKDTLEKYALSAILSNTRGVCTEAFENVVEANTLMSGLGFENGGL
AAAHAIHNGMTAIHGPVHRLMHGEKVAYGTLVQVVLEDWPLEDFNNLASFMAKCHLPIT
LEELGIPNVTDEELLMVGRATLRPDESIHNMSKKFNPSQIADAIKAVDSYSQKWQEQTGWT
ERFRLPPSRHSPHLTDIHP
11111111111111111111111111111111111111111111111111111111000000000000000000000000000
00000000000000000000000000000000000000000000000000000000000000000000000000000000000
00000000000000000000000000000000000000000000000000000000000000000000000000000000000
00000000000000000000000000000000000000000000000000000000000000000000000000000000000
00000000000000000000000000000000000000000000000000000000000000000000000000000000000
00000000000000000000000000000000000
>Q6P6M7_3bc8_18_467
RQGCEARRAHEHLIRLLLEQGKCPEDGWDESTLELFLHELAVMDSNNFLGNCGVGEREGR
VASALVARRHYRFIHGIGRSGDISAVQPKAAGSSLLNKITNSLVLNVIKLAGVHSVASCFVVP
MATGMSLTLCFLTLRHKRPKAKYIIWPRIDQKSCFKSMVTAGFEPVVIENVLEGDELRTDLK
AVEAKIQELGPEHILCLHSTTACFAPRVPDRLEELAVICANYDIPHVVNNAYGLQSSKCMHL
IQQGARVGRIDAFVQSLDKNFMVPVGGAIIAGFNEPFIQDISKMYPGRASASPSLDVLITLLS
LGCSGYRKLLKERKEMFVYLSTQLKKLAEAHNERLLQTPHNPISLAMTLKTIDGHHDKAVT
QLGSMLFTRQVSGARAVPLGNVQTVSGHTFRGFMSHADNYPCAYLNAAAAIGMKMQDV

DLFIKRLDKCLNIVRKEQTRA
11110000000000000000000000000000000000000000000000000000000000000000000000000001111
11100000000000000000000000000000000000000000000000000000000000000000000000000000000
00000000000000000000000000000000000000000000000000000000000000000000000000000000000
00000000000000000000000000000000000000000000000000000000000000000000000000000000000
00000000000000000000000000000000000000000000000000000000000000000000000000000000000
00000000000000000000000000000000001
>Q87NY2_3lxq_199_648
HRPLNPAMVAFSNDPLLNDLALNSSYSLLFAVNNMKSEKSAEQFYGKMDNQKMLDLVRA
SSTKIDFDPTLLPTMNSNPATYQGKRKNLVILLQESLGAQFVGSLGGLPLTPNLDELMQEG
WQFTQMYATGTRSVRGIEAVTTGFPPSPSRAVVKLSKSQTGFFTIADLLKEQGYHTQFIYGGE
ANFDNMKTFFFGNGFDQIVEEKNYTNPGFVGSWGVSDEDLYNKADEEFERLSKGDKPFFS
LVFTSSNHSPYEYPEGKIEQYDSEHMTRNNAVKYSDYALGTFFDKAKKSSYWDDTIFIVIAD
HDARVFGANLVPVKHFHIPALIIGKDIQPRKDDRIANNIDMPPTLLSLIGVDAKTPMIGRDL
TKPLAREDERAMMQYDKNFGYLTRDNLVVLSPGEKVSTMEYDFESQTMKPLEVDESVIDR
AKANALFASKAYQNNWYSSKRTN
11111111111111111111111111111111111111100000000000000000000000000000000000000000000
00000000000000000000000000000000000000000000000000000000000000000000000000000000000
00000000000000000000000000000000000000000000000000000000000000000000000000000000000
00000000000000000000000000000000000000000000000000000000000000000000000000000000000
00000000000000000000000000000000000000000000000000000000000000000000000000000000000
00000000000000000000000000000000011
>Q8A0N4_3poh_26_475
DDLEVGKNIDESAYSGIYENNAYLRDGKSNLVSKVVELHGETYATTVKMGLSKTPNTATSA
KVKIDAAYLETYNKAHNTDFALYPQDLVTFANEGILTVNANTKSAEVEMTIRAGEGLQEDK
TYAIPVAISDQSSDITIKDEDAKHCIYLVKDMRNAGDAYKGEGVMQGYLFFEVNDVNPLNT
LSFQLENGKLLWDVVVLFAANINYDAEAGRPRVQCNPNVQYLLDNNETLLQPLRRRGVK
VLLGLLGNHDITGLAQLSEQGAKDFAREVAQYCKAYNLDGVNYDDEYSNSPDLSNPSLTN
PSTAAAARLCYETKQAMPDKLVTVFDWGQMYGVATVDGVDAKEWIDIVVANYGSAAYPI
GQMTKKQCSGISMEFNLGGGGSLSASKAQSMIDGGYGWFMGFAPSPAKYGSVFSRLQGGG
EVLYGSNVAAPTIFYKKNDPTPYKYPDDL
11111111111111100000000000000000000000000000000000000000000000000000000000000000000
00000000000000000000000000000000000000000000000000000000000000000000000000000000000
00000000000000000000000000000000000000000000000000000000000000000000000000000000000
00000000000000000000000000000000000000000000000000000000000000000000000000000000000
00000000000000000000000000000000000000000000000000000000000000000000000000000000000
00000000000000000000000000000000000
>A6LGL1_3lm3_29_476
AADPVVVPANMEPLTIEGNRFVTLCIMIRTTPWEVSRDVKLHPRDEVDWHTLEGVRALRE
AFATNNPNGRLTWGFTMNALEDGRKNYREIRDYVVECQKKYGDEVTYFPGYFPAMYLPR
ERVNREMSEAIEIISKMVGNGYRPQSIMGGFLSADNLRYLAEKENIHVAHAVIWSQHNIDG
GGADGSPSYPFYPSTEHFCKPAQGKSDFIDCVNLDGWTMDFICARRSGQTGHGIDGYNSRR
GVGPIETYKGWGLDLGHREVMHTEAIHFDKGLELNGFGWVANIWEAQMVHEFGKDLIC
DAMKMWVTGTKERWPDTHFVTFGEFGELWRKQYKSNDDWNYRFVERGSGLGDSYNNLE
IKWFMNKEFRLALLRDWHTKNSPAYVIDFTRYDLQAHEPADPSPEKPAKDWSLINKINQK
ALRPQDKPVLIDKLEKEDQDLIRKYYPELLK
11111111111000000000000000000000000000000000000000000000000000000000000000000000000
00000000000000000000000000000000000000000000000000000000000000000000000000000000000
00000000000110000000000000000000000000000000000000000000000000000000000000000000000
00000000000000000000000000000000000000000000000000000000000000000000000000000000000
00000000000000000000000000000000000000000000000000000000000000000000000000000000000

000000000000000000000000000000001
>Q01594_1lk9_38_485
KMTWTMKAAEEAEAVANINCSEHGRAFLDGIISEGSPKCECNTCYTGPDCSEKIQGCSADV
ASGDGLFLEEYWKQHKEASAVLVSPWHRMSYFFNPVSNFISFELEKTIKELHEVVGNAAAK
DRYIVFGVGVTQLIHGLVISLSPNMTATPDAPESKVVAHAPFYPVFREQTKYFNKKGYVWA
GNAANYVNVSNPEQYIEMVTSPNNPEGLLRHAVIKGCKSIYDMVYYWPHYTPIKYKADED
ILLFTMSKFTGHSGSRFGWALIKDESVYNNLLNYMTKNTEGTPRETQLRSLKVLKEVVAMV
KTQKGTMRDLNTFGFKKLRERWVNITALLDQSDRFSYQELPQSEYCNYFRRMRPPSPSYAW
VKCEWEEDKDCYQTFQNGRINTQNGVGFEASSRYVRLSLIKTQDDFDQLMYYLKDMVKA
KRKTPLIKQLFIDQTETASRRPFI
00000000000000000000000000000000000000000000000000000000000000000000000000000000000
00000000000000000000000000000000000000000000000000000000000000000000000000000000000
00000000000000000000000000000000000000000000000000000000000000000000000000000000000
00000000000000000000000000000000000000000000000000000000000000000000000000000000000
00000000000000000000000000000000000000000000000000000000000000000000000000000000000
000000000000111111111111111111111
>F0V3Z3_4a0s_1_447
SSLSRAVLDGASAAEIEAAPVPDTYLALHLRAEDADMFKGVADKDVRKSLRLGEVPMPEL
APDEVLVAVMASSINYNTVWSAMFEPIPTFHFLKQNARQGGWATRHDQPYHVLGSDCSG
VVVRTGIGVRRWKPGDHVIVHPAHVDEQEPATHGDGMLGTEQRAWGFETNFGGLAEYG
VVRASQLLPKPAHLTWEEAAVSPLCAGTAYRMLVSDRGAQMKQGDIVLIWGASGGLGSYA
IQFVKNGGGIPVAVVSSAQKEAAVRALGCDLVINRAELGITDDIADDPRRVVETGRKLAKLV
VEKAGREPDIVFEHTGRVTFGLSVIVARRGGTVVTCGSSSGYLHTFDNRYLWMKLKKIVGS
HGANHEEQQATNRLFESGAVVPAMSAVYPLAEAAEACRVVQTSRQVGKVAVLCMAPEQG
LGVTDPDLRARLGEDRLNPLRGLTATSR
00000000000000000000000000000000000000000000000000000000000000000000000000000000000
00000000000000000000000000000000000000000000000000000000000000000000000000000000000
00000000000000000000000000000000000000000000000000000000000000000000000000000000000
00000000000000000000000000000000000000000000000000000000000000000000000000000000000
00000000000000000000000000000000000000000000000000000000000000000000000000000000000
00000000000000000000000000000011
>Q8G4D5_2p9b_1_447
MLSHNPIVEPFALAHATIVTGDKAGTILRNMTIVVGADGRIEQVAPSIETSIPAEYHYLDGTG
KIVMPGLINAHTHLFSQGKPLNPKLATPKGQRMVATFAHSPLGKPYMAATVKHNATTLLE
SGVTTIRTLGDVGYEVVTLRDQIDAGQILGPRILASGPLMAIPEGHGAPLIALTSGTPEEART
AVAQNLKAGVNAIKIAATGGVTDAQEIGEAGSPQMSVEQMRAICDEAHQYGVIVGAHAQ
SPEGVRRSLLAGVDTIEHGSVLDDELIGMFRHNPNALRGYSALIPTLSAGLPLTLLGQDVTGI
TDIQLENSKNVVGGMVSGARQAHEAGLMIGVGTDTGMTFVPQYATWRELELLVAYAGFSP
AEALHAATAVNASILGVDAETGSLEVGKSADLLVLNANPLDDLRALEHPALVIAAGHPVW
RPGPKRFADIDALLDEAYA
11111000000000000000000000000000000000000000000000000000000000000000000000000001111
11111111111111111111111110000000000000000000000000000000000000000000000000000000000
00000000000000000000000000000000000000000000000111111000000000000000000000000000000
00000000000000000000000000000000000000000000000000000000000000000000000000000000000
00000000000000000000000000000000000000000000000000000000000000000000000000000000000
00000000000000000000000000000000
>O05935_1z01_0_445
MSDQPIIRRRQVKTGISDARANNAKTQSQYQPYKDAAWGFINHWYPALFTHELEEDQVQ
GIQICGVPIVLRRVNGKVFALKDQCLHRGVRLSEKPTCFTKSTISCWYHGFTFDLETGKLVTI
VANPEDKLIGTTGVTTYPVHEVNGMIFVFVREDDFPDEDVPPLAHDLPFRFPERSEQFPHPL
WPSSPSVLDDNAVVHGMHRTGFGNWRIACENGFDNAHILVHKDNTIVHAMDWVLPLGL

LPTSDDCIAVVEDDDGPKGMMQWLFTDKWAPVLENQELGLKVEGLKGRHYRTSVVLPGV
LMVENWPEEHVVQYEWYVPITDDTHEYWEILVRVCPTDEDRKKFQYRYDHMYKPLCLHG
FNDSDLYAREAMQNFYYDGTGWDDEQLVATDISPITWRKLASRWNRGIAKPGRGVAGAVK
DTSLIFKQTADGKRPGYKVEQIKEDH
111111111111111000000000000000000000000000000000000000000000000000000000000000000000000000
000000000000000000000000000000000000000000000000000000000000000000000000000000000000000000
000000000000000000000000000000000000000000000000000000000000000000000000000000000000000000
000000000000000000000000000000000000000000000000000000000000000000000000000000000000000000
000000000000000000000000000000000000000000000000000000000000000000000000000000000000000000
0000000000000000000000000001111
>P03587_3vkw_665_1110
SYTRSEEIESLEQFHMATASSLIHKQMCSIVYTGPLKVQQMKNFIDSLVASLSAAVSNLVKILK
DTAAIDLETRQKFGVLDVASKRWLVKPSAKNHAWGVVETHARKYHVALLEHDEFGIITCD
NWRRVAVSSESVVYSDMAKLRTLRRLLKDGEPHVSSAKVVLVDGVPGCGKTKEILSRVNFE
EDLILVPGRQAAEMIRRRANASGIIVATKDNVRTVDSFLMNYGKGARCQFKRLFIDEGLML
HTGCVNFLVEMSLCDIAYVYGDTQQIPYINRVTGFPYPAHFAKLEVDEVETRRTTLRCPADV
THFLNQRYEGHVMCTSSEKKSVSQEMVSGAASINPVSKPLKGKILTFTQSDKEALLSRGYAD
VHTVHEVQGETYADVSLVRLTPTPVSIIARDSPHVLVSLSRHTKSLKYYTVVMDPLVSIIRDLE
RVSSYLLDMYKV
111111000000000000000000000000000000000000000000000000000000000000000000000000000000000000
000000000000000000000000000000000000000000000000000000000000000000000000000000000000000000
000000000000000000000000000000000000000000000000000000000000000000000000000000000000000000
000000000000000000000000000000000000000000000000000000000000000000000000000000000000000000
000000000000000000000000000000000000000000000000000000000000000000000000000000000000000000
0000000000000000000000000000000
>P0A6E4_1k92_1_446
TTILKHLPVGQRIGIAFSGGLDTSAALLWMRQKGAVPYAYTANLGQPDEEDYDAIPRRAME
YGAENARLIDCRKQLVAEGIAAIQCGAFHNTTGGLTYFNTTPLGRAVTGTMLVAAMKEDG
VNIWGDGSTYKGNDIERFYRYGLLTNAELQIYKPWLDTDFIDELGGRHEMSEFMIACGFDY
KMSVEKAYSTDSNMLGATHEAKDLEYLNSSVKIVNPIMGVKFWDESVKIPAEEVTVRFEQG
HPVALNGKTFSDDVEMMLEANRIGGRHGLGMSDQIENRIIEAKSRGIYEAPGMALLHIAYE
RLLTGIHNEDTIEQYHAHGRQLGRLLYQGRWFDSQALMLRDSLQRWVASQITGEVTLELRR
GNDYSILNTVSENLTYKPERLTMEKGDSVFSPDDRIGQLTMRNLDITDTREKLFGYAKTGLL
SSSAASGVPQVENLENKGQ
000000000000000000000000000000000000000000000000000000000000000000000000000000000000000000
000000000000000000000000000000000000000000000000000000000000000000000000000000000000000000
000000000000000000000000000000000000000000000000000000000000000000000000000000000000000000
000000000000000000000000000000000000000000000000000000000000000000000000000000000000000000
000000000000000000000000000000000000000000000000000000000000000000000000000000000000000000
0000000000000000000000000000011
>P38999_2axq_0_445
MGKNVLLLGSGFVAQPVIDTLAANDDINVTVACRTLANAQALAKPSGSKAISLDVTDDSA
LDKVLADNDVVISLIPYTFHPNVVKSAIRTKTDVVTSSYISPALRELEPEIVKAGITVMNEIGL
DPGIDHLYAVKTIDEVHRAGGKLKSFLSYCGGLPAPEDSDNPLGYKFSWSSRGVLLALRNSA
KYWKDGKIETVSSEDLMATAKPYFIYPGYAFVCYPNRDSTLFKDLYHIPEAETVIRGTLRYQG
FPEFVKALVDMGMLKDDANEIFSKPIAWNEALKQYLGAKSTSKEDLIASIDSKATWKDDED
RERILSGFAWLGLFSDAKITPRGNALDTLCARLEELMQYEDNERDMVVLQHKFGIEWADG
TTETRTSTLVDYGKVGGYSSMAATVGYPVAIATKFVLDGTIKGPGLLAPYSPEINDPIMKELK
DKYGIYLKEKTVA
100000000000000000000000000000000000000000000000000000000000000000000000000000000000000000
000000000000000000000000000000000000000000000000000000000000000000000000000000000000000000

000000000000000000000000000000000000000000000000000000000000000000000000000000000000000000
000000000000000000000000000000000000000000000000000000000000000000000000000000000000000000
000000000000000000000000000000000000000000000000000000000000000000000000000000000000000000
00000000000000000000000000000000000
>A3EYF7_2ykv_0_444
MNEPIGEPGRSPASDTAEKAQAIAAARNTFARDNPVSAGHHERARRSMPGGNTRSILFHRP
FPLVIAQGTGSRFQDVDGHAYVNFLGEYTAGLFGHSHPVIRAAVERALAVGLNLSTQTENE
ALFAEAVCDRFPSIDLVRFTNSGTEANLMALATATAITGRKTVLAFDGGYHGGLLNFASGH
APTNAPYHVVLGVYNDVEGTADLLKRHGHDCAAILVEPMLGAGGCVPAERAFLDLLRAE
ASRCGALLIFDEVMTSRLSGGGAQEMLGISADLTTLGKYIGGGMSFGAFGGRRDLMERFDP
ARDGAFAHAGTFNNNILTMSAGHAALTQIYTRQAASDLSASGDRFRANLNRIAVENQAPL
QFTGLGSLGTIHFSRAPIRSAGDVRAADQQLKELFFFHMLRKGIYLAPRGMYALSLEIADAG
RDAFAEALADFIGEQRALLM
111111111111110000000000000000000000000000000000000000000000000000000000000000000000000000
000000000000000000000000000000000000000000000000000000000000000000000000000000000000000000
000000000000000000000000000000000000000000000000000000000000000000000000000000000000000000
000000000000000000000000000000000000000000000000000000000000000000000000000000000000000000
000000000000000000000000000000000000000000000000000000000000000000000000000000000000000000
0000000000000000000000000000001
>Q13231_1waw_21_465
AKLVCYFTNWAQYRQGEARFLPKDLDPSLCTHLIYAFAGMTNHQLSTTEWNDETLYQEFN
GLKKMNPKLKTLLAIGGWNFGTQKFTDMVATANNRQTFVNSAIRFLRKYSFDGLDLDWE
YPGSQGSPAVDKERFTTLVQDLANAFQQEAQTSGKERLLLSAAVPAGQTYVDAGYEVDKIA
QNLDFVNLMAYDFHGSWEKVTGHNSPLYKRQEESGAAASLNVDAAVQQWLQKGTPASK
LILGMPTYGRSFTLASSSDTRVGAPATGSGTPGPFTKEGGMLAYYEVCSWKGATKQRIQDQ
KVPYIFRDNQWVGFDDVESFKTKVSYLKQKGLGGAMVWALDLDDFAGFSCNQGRYPLIQ
TLRQELSLPYLPSGTPELEVPKPGQPSEPEHGPSPGQDTFCQGKADGLYPNPRERSSFYSCAA
GRLFQQSCPTGLVFSNSCKCCTWN
000000000000000000000000000000000000000000000000000000000000000000000000000000000000000000
000000000000000000000000000000000000000000000000000000000000000000000000000000000000000000
000000000000000000000000000000000000000000000000000000000000000000000000000000000000000000
000000000000000000000000000000000000000000000000000000000000000000000000000000000000000000
000000000000000000000000000000000000111111111111111111111111111111111111111111111111111111
1111111111111111111111111111111111
>Q3ACS3_2h9a_0_444
MGLTGLEIYKHLPKKNCKECGQPTCLAFAMQIAAGKAGLDACPYVSDEAKELLESASAPP
VALIKVGKGEKVLEIGHETVLFRHDKRFEHPCGLAILVEDTLSEGEIKERVEKINKLVFDRVG
QMHSVNLVALKGSSQDAATFAKAVATAREVTDLPFILIGTPEQLAAALETEGANNPLLYAAT
ADNYEQMVELAKKYNVPLTVSAKGLDALAELVQKITALGYKNLILDPQPENISEGLFYQTQ
IRRLAIKKLFRPFGYPTIAFALDENPYQAVMEASVYIAKYAGIIVLNTVEPADILPLITLRLNIY
TDPQKPIAVEPKVYEILNPGPDAPVFITTNFSLTYFCVAGDVEGARIPAYILPVDTDGTSVLTA
WAAGKFTPEKIAQFLKESGIAEKVNHRKAILPGGVAVLSGKLQELSGWEILVGPRESSGINSF
IKQRWNV
111111111111111111111111111111111111111111111111111111111111111000000000000000000000000000
000000000000000000000000000000000000000000000000000000000000000000000000000000000000000000
000000000000000000000000000000000000000000000000000000000000000000000000000000000000000000
000000000000000000000000000000000000000000000000000000000000000000000000000000000000000000
000000000000000000000000000000000000000000000000000000000000000000000000000000000000000000
000000000000000000000000001111
>Q81BF7_2qgm_0_444
MNKKRMIAMVSTALLVTGCAEVGNAQTVAVENSGQSVQKNIVKSIQSQANPLKTIEPSKPF

EDLKPLKKMIGNAQYVGLGENTHGSSEIFTMKFRLVKYLVTEMGFTNFAMEEDWGNGLKL
NEYIQTGKGNPREFLKLLYPTDEIIAMIEWMKDYNADPSNKKKIQFIGLDLKALDQGSFNK
VIDYVRLHRPDLLAEVEENYKELSSFTGSIQEYMKLTPKLKEKFKANAERVARLLKDENEQA
NTEIIPSEYIWAKATASAIEKFTTMLLPNDYPSIIKLHEQYLADHAMWAQETFGGKTMVWA
HNIHIAKGIIDEKLYPYVAGQFLKERLDNNYVTIGSTTTEGNFTLYSEYNPSTGGKITTDTIPQ
DVKSFNYTLGKVPYKMFLLDNRHLKGQAEKWVKAKRPLLSIGGQILPNSSVYFDTSLLEQF
DIIFHIRKTSPSHIK
11111111111111111111111111111111000000000000000000000000000000000000000000000000000
00000000000000000000000000000000000000000000000000000000000000000000000000000000000
00000000000000000000000000000000000000000000000000000000000000000000000001111111111
11000000000000000000000000000000000000000000000000000000000000000000000000000000000
00000000000000000000001111100000000000000000000000000000000000000000000000000000001
111110000000000000000000000000
>A3XHN1_3bg2_0_443
MMNWEHLLSLKRQGDTAKRLRIEQDDTRLGFEVDYDRIIFSAPFRSLQDKTQVIPLSKTDFV
HTRLTHSLEVSVVGRSLGRMVGKKLLEKYPHLEQVYGYKFNDFGAIVAAAALAHDIGNPP
FGHSGEKAIGEFFKNGYGKRYKDSLTAKEYQDLIKFEGNANGFKVLSQSKPGAQGGLRLSY
ATLGAFMKYPKESLPHKPSDHIADKKYGFFQSERALFEDVAQELGLLKRSTTDDVSWSRHP
LAYLVEAADDICYTIIDFEDGINLGLIPEEYALEYMVKLVGQTIDRNKYNALQETSDRVSYLR
ALAIGTLINESVDTFMKYEEEILAGTFDQSLIDKSNYQAQITDIINLSIERIYNSREVIEKEIAGY
EILSTLLEARCRALDNNDTHYNQLIQQLLAPNDHSEKSLYENLIQICAEVSTMTDGKALRN
YKKIKGLDVK
10000000000000000000000000000000000000000000000000000000111000000000000000000000000
00000000000000000000000000000000000000000000000000000000000000000000000000000000000
00000000000000000000000000000001111111111100000000000000000000000000111100000000000
00000000000000000000000011111111111111111110000000000000000000000000000000000000000
00000000000000000000000000000000000000000000000000000000000000000000000111111000000
00000000000000000000000000111
>Q8A045_3k11_21_464
KKAVINDSNTPLHLLQPAYQGTYGDLTPEQVKKDIDRVFAYIDKETPARVVDKNTGKVITDY
TAMGDEAQLERGAFRLASYEWGVTYSALIAAAETTGDKRYTDYVQNRFRFLAEVAPHFKR
VYEEKGKTDSQLLQILTPHALDDAGAVCTAMIKLRLKDESLPVDGLIQNYFDFIINKEYRLA
DGTFARNRPQRNTLWLDDMFMGIPAVAQMSRYDKEAKNKYLAEAVKQFLQFADRMFIPE
KGLYRHGWVESSTDHPAFCWARANGWALLTACELLDVLPEDYPQRPKVMDYFRAHVRGV
TALQSGEGFWHQLLDCNDSYLETSATAIYVYCLAHAINKGWIDAIAYGPVAQLGWHAVAG
KINEEGQVEGTCVGTGMAFDPAFYYYRPVNVYAAHGYGPVLWAGAEMIRLLNTQHPQM
NDSAVQYYQEKQKTTAPIFAVDSE
11110000000000000000000000000000000000000000000000000000000000000000000000000000000
00000000000000000000000000000000000000000000000000000000000000000000000000000000000
00000000000000000000000000000000000000000000000000000000000000000000000000000000000
00000000000000000000000000000000000000000000000000000000000000000000000000000000000
00000000000000000000000000000000000000000000000000000000000000000000000000000000000
00000000000000000000000000001
>A1S420_3iib_24_466
SDDINQKVAEQLAQKAQSSSLGYDIVESLTVEVGPRLAGSEQDKVAVDWAIAKLQSLGFDR
VYKEPVTVPVWRRGIAKASILSPFPQPLVVTALGGSIATPAQGLSATIVRFDTLQDLQNAEAG
SLNDKIAFIDAKTERHRDGKGYGQTASGRSRGAVAAAEKGAVGIIIRSIGTDHDRMAHTGM
MRYEEGVTAIPAAAISNPDADLINAMLKRDKEVVISLELGSERRGETTSYNVIAEVKGSTKA
DEIVLIGAHLDSWDEGTGAIDDGAGVAIVTAAAKHILDLPQKPERTIRVVLYAAEELGLLGG
KTYAKEHEAELEKHYIAAESDFGAGPIYQIDWRVADTAHSPVINAMKVAEPLGVAAGNNK
ASGGPDVSMLPALGVPVASLRQDGSDYFDYHHTPNDTLDKINPEALAQNVAVYAQFAWV

MANSKVELRPLPPKE
00000000000000000000000000000000000000000000000000000000000000000000000000000000000
00000000000000000000000000000000000000000000000000000000000000000000000000000000000
00000000000000000000000000000000000000000000000000000000000000000000000000000000000
00000000000000000000000000000000000000000000000000000000000000000000000000000000000
00000000000000000000000000000000000000000000000000000000000000000000000000000000000
0000000000000000000000000001
>F8UVJ4_3rva_0_442
MSQHKATYQQHIEELQARTREALQREGLDGLVIHSGQGKRLFLDDNHYPFKVNPQFKAW
VPVIDNPNCWLVVNGVDKPTLIFYRPEDFWHKVPPEPNDFWTDSFDIKLLQQADAVEKFL
PYDKSRFAYVGEYIEVAKALGFDNVNPDRVLHYLHYQRAYKTDYELDCMREANKLAVAG
HKAAEQAFREGKSEFDINLAYAAASRQGDNDVPYTSIVALNEHASILHYMQCDTVAPKESR
SFLIDAGANYHGYAADITRTYAQEGVHNSAMFRDLIQAVDKVTLTLVDSLKPGVAYTDIHL
LAHDGIAQILHDTGMVNLTPPEIVEMGITRTFFPHGIGHFLGLQVHDVGGLVNDDRGTPKP
APDDHPFLRCTRMVEARQVFTIEPGLYFIDSLLRDLKATPASKYINWDTIDAYKPFGGIRIED
NIIVHRDKNENMTRDLDLN
10000000000000000000000000000000000000000000000000000000000000000000000000000000000
00000000000000000000000000000000000000000000000000000000000000000000000000000000000
00000000000000000000000000000000000000000000000000000000000000000000000000000000000
00000000000000000000000000000000000000000000000000000000000000000000000000000000000
00000000000000000000000000000000000000000000000000000000000000000000000000000000000
0000000000000000000000000000
>Q5SID2_3g5r_0_442
MERVNVVGAGLAGSEAAWTLLRLGVPVRLFEMRPKRMTPAHGTDRFAEIVCSNSLGGEGE
TNAKGLLQAEMRRAGSLVMEAADLARVPAGGALAVDREEFSGYITERLTGHPLLEVVREE
VREIPPGITVLATGPLTSEALAEALKRRFGDHFLAYYDAASPIVLYESIDLTKCFRAGRYGQSA
DYLNCPMTEEEYRRFHQALLEAQRHTPHDWEKLEFFEACVPVEELARRGYQTLLFGPMKP
VGLVDPRTGKEPFAVVQLRQEDKAGRMWSLVGFQTGLKWPEQKRLIQMIPGLENAEIVRY
GVMHRNTYLNAPRLLGETLEFREAEGLYAAGVLAGVEGYLESAATGFLAGLNAARKALGL
PPVAPPEESMLGGLVRYLATANPEGFQPMYANWGLVPPVEGRMGKKEKRQAMYRRGLEA
FSAWLSGLNPPLPRPEAALV
00000000000000000000000000000000000000000000000000000000000000000000000000000000000
00000000000000000000000000000000000000000000000000000000000000000000000000000000000
00000000000000000000000000000000000000001111111111111111000000000000000000000000000
00000000000000000000000000000000000000000000000000000000000000000000000000000000000
00000000000000000000000000000000000000000000000000000000000000000000000000000000000
0000000000000000000001111111
>Q8Y432_2xmo_0_442
MIRFFKIVTPILLSISLVACSSASGKTEKITAPIEKDRNLSMVVTTDVHYFAPSLTDNGKAFEKY
VAAGDGKQLAYSDEITDAFLADVESKKTDVLIISGDLTNNGEKTSHEELAKKLTQVEKNGT
QVFVVPGNHDINNPWARKFEKDKQLPTDTISPTDFSKIYGDFGYEDAISSDEFSLSYLAAPSS
KVWLLMLDTAIYKTNMQQGNPTTEGGLTAGTLDWIKESSALAKKNGAKLIPVLHHNLTD
HNDVIQKGYTINYNQQVIDALTEGAMDFSLSGHIHTQNIRSAKSTDGKEITDIVTNALSVFP
HKYGNITYSAKNKNFTYQSQKLDMEAWAKAQGSTDENLLNFDQFDYETFYNSGYDKAM
MDLMTDESYDKYNQADKEKMADTMGLNNMYFFAGTAPPKSDGMALWDSAPNSFLKDY
VLSSSNPPKKSNDYYVSP
11111111111111111111111111111111000000000000000000000000000000000000000000000000000
00000000000000000000000000000000000000000000000000000000000000000000000000000000000
00000000000000000000000000000000000000000000000000000000000000000000000000000000000
00010000000000000000000000000000000000000000000000000000000000000000000000000000000
00000000000000000000000000000000000000000000000000000000000000000000000000000000000

0000000000000000000000000000
>Q9QZM2_1g5h_16_458
WLSGYAGPADGTQQPDAPEHAVAREALVDLCRRRHFFSGTPQQLSTAALLSGCHARFGPL
GVELRKNLASQWWSSMVVFREQVFAVDSLHQEPGSSQPRDSAFRLVSPESIREILQDREPSK
EQLVAFLENLLKTSGKLRATLLHGALEHYVNCLDLVNRKLPFGLAQIGVCFHPVSNSNQTP
SSVTRVGEKTEASLVWFTPTRTSSQWLDFWLRHRLLWWRKFAMSPSNFSSADCQDELGRK
GSKLYYSFPWGKEPIETLWNLGDQELLHTYPGNVSTIQGRDGRKNVVPCVLSVSGDVDLGT
LAYLYDSFQLAENSFARKKSLQRKVLKLHPCLAPIKVALDVGKGPTVELRQVCQGLLNELL
ENGISVWPGYSETVHSSLEQLHSKYDEMSVLFSVLVTETTLENGLIQLRSRDTTMKEMMHIS
KLRDFLVKYLASASNV
11111111111111111111111000000000000000000000000000000000000000000000000000000000000
00000000000000000000000000000000000000000000000000000000000000000000000000000000000
00000000000111111111000000000000000000000000000000000000000000000000000000000000000
00000000000000000000000000000000000000000000000000000000000000000001111111111000000
00000000000000000000000000000000000000000000000000000000000000000000000000000000000
0000000000000000000000000000
>O00092_1qwo_23_464
SAGSKSCDTVDLGYQCSPATSHLWGQYSPFFSLEDELSVSSKLPKDCRITLVQVLSRHGARYP
TSSKSKKYKKLVTAIQANATDFKGKFAFLKTYNYTLGADDLTPFGEQQLVNSGIKFYQRYKA
LARSVVPFIRASGSDRVIASGEKFIEGFQQAKLADPGATNRAAPAISVIIPESETFNNTLDHGV
CTKFEASQLGDEVAANFTALFAPDIRARAEKHLPGVTLTDEDVVSLMDMCSFDTVARTSDA
SQLSPFCQLFTHNEWKKYNYLQSLGKYYGYGAGNPLGPAQGIGFTNELIARLTRSPVQDHT
STNSTLVSNPATFPLNATMYVDFSHDNSMVSIFFALGLYNGTEPLSRTSVESAKELDGYSASW
VVPFGARAYFETMQCKSEKEPLVRALINDRVVPLHGCDVDKLGRCKLNDFVKGLSWARSG
GNWGECFS
11111100000000000000000000000000000000000000000000000000000000000000000000000000000
00000000000000000000000000000000000000000000000000000000000000000000000000000000000
00000000000000000000000000000000000000000000000000000000000000000000000000000000000
00000000000000000000000000000000000000000000000000000000000000000000000000000000000
00000000000000000000000000000000000000000000000000000000000000000000000000000000000
000000000000000000000000001
>Q8WZK8_3mm3_56_497
ANDTILPLNNIQGDILVGMKKQKERFVFFQVNDATSFKTALKTYVPERITSAAILISDPSQQP
LAFVNLGFSNTGLQALGITDDLGDAQFPDGQFADAANLGDDLSQWVAPFTGTTIHGVFLI
GSDQDDFLDQFTDDISSTFGSSITQVQALSGSARPGDQAGHEHFGFLDGISQPSVTGWETTV
FPGQAVVPPGIILTGRDGDTGTRPSWALDGSFMAFRHFQQKVPEFNAYTLANAIPANSAGN
LTQQEGAEFLGARMFGRWKSGAPIDLAPTADDPALGADPQRNNNFDYSDTLTDETRCPFG
AHVRKTNPRQDLGGPVDTFHAMRSSIPYGPETSDAELASGVTAQDRGLLFVEYQSIIGNGF
RFQQINWANNANFPFSKPITPGIEPIIGQTTPRTVGGLDPLNQNETFTVPLFVIPKGGEYFFLP
SISALTATIAA
11100000000000000000000000000000000000000000000000000000000000000000000000000000000
00000000000000000000000000000000000000000000000000000000000000000000000000000000000
00000000000000000000000000000000000000000000000000000000000000000000000000000000000
00000000000000000000000000000000000000000000000000000000000000000000000000000000000
00000000000000000000000000000000000000000000000000000000000000000000000000000000000
000000000000000000000000000
>Q9KS51_1yc9_42_483
MWEANELSSTNTFSHQAEMDWPSANWWQRYQDAQLNHLIEEALQHSPSLEMAMARLK
GAQGFARQAGAIRSFDLGLAASATESKVSERYQSATPPDGWNDYGTLTLNFQYDFDFWGK
NRAAVVAATSELAAAEAESVAARLMISTSIANAYAELARLYANQETVHAALQVRNKTVELL
EKRYANGLETLGSVSQAKAVAASVEAELLGIQESIQLQKNALAALVGQGPDRAASIEEPHIT

LTSRYGLPSEAGVGLLGHRADITAARWRAEAAAQQVGIAQAQFYPDVTLSAFIGYQAFGL
DHLFDSGNDAGAIGPAIYLPLFTGGRLEGQLTSAEARYQEAVAQYNGTLVQALYEVADVVT
SSQALQARINKTEQAVQQAEQALHIATNRYQGGLATYLDVLVAEESLLNNQRALVNLQSR
AFSLDLALIHALGGGFETTES
11111111111111111111000000000000000000000000000000000000000000000000000000000000000
00000000000000000000000000000000000000000000000000000000000000000000000000000000000
00000000000000000000000000000000000000000000000000000000000000000000000000000001111
11100000000000000000000000000000000000000000000000000000000000000000000000000000000
00000000000000000000000000000000000000000000000000000000000000000000000000000000000
000000000000000000000001111
>Q65Z69_1y9z_144_584
AETTPWGQTFVGATVLSDSQAGNRTICIIDSGYDRSHNDLNANNVTGTNNSGTGNWYQP
GNNNAHGTHVAGTIAAIANNEGVVGVMPNQNANIHIVKVFNEAGWGYSSSLVAAIDTCV
NSGGANVVTMSLGGSGSTTTERNALNTHYNNGVLLIAAAGNAGDSSYSYPASYDSVMSVA
AVDSNLDHAAFSQYTDQVEISGPGEAILSTVTVGEGRLADITIGGQSYFSNGVVPHNRLTPS
GTSYAPAPINASATGALAECTVNGTSFSCGNMANKICLVERVGNQGSSYPEINSTKACKTA
GAKGIIVYSNSALPGLQNPFLVDANSDITVPSVSVDRATGLALKAKLGQSTTVSNQGNQDY
EYYNGTSMATPHVSGVATLVWSYHPECSASQVRAALNATADDLSVAGRDNQTGYGMINA
VAAKAYLDESCTGPTDPGTG
00000000000000000000000000000000000000000000000000000000000000000000000000000000000
00000000000000000000000000000000000000000000000000000000000000000000000000000000000
00000000000000000000000000000000000000000000000000000000000000000000000000000000000
00000000000000000000000000000000000000000000000000000000000000000000000000000000000
00000000000000000000000000000000000000000000000000000000000000000000000000000000000
00000000000000000000011111
>Q9FYU1_3lx4_56_496
AAPAAEAPLSHVQQALAELAKPKDDPTRKHVCVQVAPAVRVAIAETLGLAPGATTPKQLA
EGLRRLGFDEVFDTLFGADLTIMEEGSELLHRLTEHLEAHPHSDEPLPMFTSCCPGWIAMLE
KSYPDLIPYVSSCKSPQMMLAAMVKSYLAEKKGIAPKDMVMVSIMPCTRKQSEADRDWFC
VDADPTLRQLDHVITTVELGNIFKERGINLAELPEGEWDNPMGVGSGAGVLFGTTGGVME
AALRTAYELFTGTPLPRLSLSEVRGMDGIKETNITMVPAPGSKFEELLKHRAAARAEAAAH
GTPGPLAWDGGAGFTSEDGRGGITLRVAVANGLGNAKKLITKMQAGEAKYDFVEIMACP
AGCVGGGGQPRSTDKAITQKRQAALYNLDEKSTLRRSHENPSIRELYDTYLGEPLGHKAHE
LLHTHYVAGGVEEKDEKK
11111111000000000000000000000000000000000000000000000000000000000000000000000000000
00000000000000001111100000000000000000000000000000000000000000000000000000000000000
00000000000000000011100000000000000000000000000000000000000000000000000000000000000
00000000000000000000000000000000000000000000001111111111100000000000000000000000000
00000000000000000000000000000000000000000000000000000000000000000000000000000000000
00000000000000000001111111
>C7QHQ7_4fx5_0_439
MSQYPDFAFEINQNEFLAAGVREVHAVVTLTATAAAGGAPAAASYGAPASGSENVEVIIID
CSGSMDYPRTKMMAAKEATKVAIDTLTDGAFFAVVAGTEGARVVYPTGGQLLRADYQSRA
AAKEAVGRLHANGGTAMGRWLAQAGRIFDTAPSAIKHAILLTDGKDESETPADLARAIQS
SIGNFTADCRGIGEDWEPKELRKIADALLGTVGIIRDPATLAEDFREMTAKSMGKEVADVAL
RLWAPKGATIRYVKQVSPNLADLSGMRVPGDNPLTGDYPTGAWGAESREYHICVEVEPGN
IGQEKLAGRVQLVAKDAGGATLLGEGKIRAVWTEDTDLSTRINGRVAHYTGQAEMAAAIQ
EGLDAQAAGDLDTATARLGRAMDLAVESGHEDTVKMLRKVTEVDPATSKVRAKAKVDK
GDAMELDVVSTKTVRAKRN
10000000000000000000000000000000001111111111111111000000000000000000000000000000000
00000000000000000000000000000000000000000000000000000000000000000000000000000000000

00000000000000000000000000000000000000000000000000000000000000000000000000000000000
00000000000000000000000000000000000000000000000000000000000000000000000000000000000
00000000000000000000000000000000000000000000000000000000000000000000000000000000000
0000000000000000000000111
>P23874_3dnt_0_439
MPKLVTWMNNQRVGELTKLANGAHTFKYAPEWLASRYARPLSLSLPLQRGNITSDAVFNF
FDNLLPDSPIVRDRIVKRYHAKSRQPFDLLSEIGRDSVGAVTLIPEDETVTHPIMAWEKLTEA
RLEEVLTAYKADIPLGMIREENDFRISVAGAQEKTALLRIGNDWCIPKGITPTTHIIKLPIGEIR
QPNATLDLSQSVDNEYYCLLLAKELGLNVPDAEIIKAGNVRALAVERFDRRWNAERTVLL
RLPQEDMCQTFGLPSSVKYESDGGPGIARIMAFLMGSSEALKDRYDFMKFQVFQWLIGATD
GHAKNFSVFIQAGGSYRLTPFYDIISAFPVLGGTGIHISDLKLAMGLNASKGKKTAIDKIYPR
HFLATAKVLRFPEVQMHEILSDFARMIPAALDNVKTSLPTDFPENVVTAVESNVLRLHGRLS
REYGSK
10000000000000000000000000000000000000000000000000000000000000000000000000000000000
00000000000000000000000000000000000000000000000000000000000000000000000000000000000
00000000000000000011111111111100000000000000000000000000000000000000000000000000000
00000000000000000000000000000000000000000000000000000000000000000000000000000000000
00000000000000000000000000000000000000000000000000000000000000000000000000000000000
0000000000000000000000111
>Q9F1Y6_3v1x_0_439
MPDSGTLGTPPPEQGPTPPTTLPDVPAPVIPSASVTSAASDFLAALHPPVTVPDPAPPPPPAP
AAGNPPDTVTGDSVLQRILRGPTGPGTTSLAPAVRYGRQPGPEAPASAPPAAGRAVPGLYH
HPVPEPDPVRVEEVSRRIKRWAEDEVQLYPEEWEGQFDGFSVGRYMVGCHPDAPTVDHLM
LATRLMVAENAVDDCYCEDHGGSPVGLGGRLLLAHTAIDHFHSTAEYTPTWQASLAADA
PRRAYDSAMGYFVRAATPSQSDRYRHDMARLHLGYLAEGAWAQTGHVPEVWEYLAMRQ
FNNFRPCPTITDTVGGYELPADLHARPDMQRVIALAGNATTIVNDLYSYTKELNSPGRHLN
LPVVIAEREQLCERDAYLKAVEVHNELQHSFEAAAADLAEACPLPPVLRFLRGVAAWVDG
NHDWHRTNTYRYSLPDFW
11111111111111111111111111111111111111111111111111111111111111111111111111111111111
11111111111111111111111111111111000000000000000000000000000000000000000111111000000
00000000000000000000000000000000111111110000000000000000000000000000000000000000000
00000000000000000000000000000000000000000000000000000000000000000000000000000000000
00000000000000000000000000000000000000000000000000000000000000000000000000000000000
0000000000000000000000000
>P53634_3pdf_24_462
DTPANCTYLDLLGTWVFQVGSSGSQRDVNCSVMGPQEKKVVVYLQKLDTAYDDLGNSGH
FTIIYNQGFEIVLNDYKWFAFFKYKEEGSKVTTYCNETMTGWVHDVLGRNWACFTGKKVG
TASENVYVNIAHLKNSQEKYSNRLYKYDHNFVKAINAIQKSWTATTYMEYETLTLGDMIRR
SGGHSRKIPRPKPAPLTAEIQQKILHLPTSWDWRNVHGINFVSPVRNQASCGSCYSFASMG
MLEARIRILTNNSQTPILSPQEVVSCSQYAQGCEGGFPYLIAGKYAQDFGLVEEACFPYTGTD
SPCKMKEDCFRYYSSEYHYVGGFYGGCNEALMKLELVHHGPMAVAFEVYDDFLHYKKGIY
HHTGLRDPFNPFELTNHAVLLVGYGTDSASGMDYWIVKNSWGTGWGENGYFRIRRGTDE
CAIESIAVAATPIPKL
00000000000000000000000000000000000000000000000000000000000000000000000000000000000
00000000000000000000000000000000000111111111111111111111111111111111111111111111111
11111111111111111111111111111111111111100000000000000000000000000000000000000000000
00000000000000000000000000000000000000000000000000000000000000000000000000000000000
00000000000000000000000000000000000111000000000000000000000000000000000000000000000
000000000000000000000000
>Q9X1F8_1mky_0_438
MATVLIVGRPNVGKSTLFNKLVKKKKAIVEDEEGVTRDPVQDTVEWYGKTFKLVDTCGVF

DNPQDIISQKMKEVTLNMIREADLVLFVVDGKRGITKEDESLADFLRKSTVDTILVANKAEN
LREFEREVKPELYSLGFGEPIPVSAEHNINLDTLLETIIKKLEEKGLDLESKPEITDAIKVAIVGR
PNVGKSTLFNAILNKERALVSPIPGTTRDPVDDEVFIDGRKYVFVDTAGLRRKSRVEPRTVE
KYSNYRVVDSIEKADVVVIVLDATQGITRQDQRIAGLVERRGRASVVVFNKWDLVEHREKR
YDEFTKLFREKLYFIDYSPLIFTSADKGWNIDRVIDAINLAYASYTTKVPSSAINSALQKVLAF
TNLPRGLKIFFGLQVDIKPPTFLFFVNSIEKVKNPQKIFLRKLIRDYVFPFEGSPIFLKFKRSR
10000000000000000000000111111111111110000000000000000000000000000000000000000000000
00000000000000000000000000000000000000000000000000000000000000000000000000000000000
00000000000000000000000000000000000000000000001111110000000000000000000011111111111
00000000000000000000000000000000000000000000000000000000000000000000000000000000000
00000000000000000000000000000000000000000000000000000000000000000000000000000000000
000000000000000000000000
>O52582_1yqz_0_437
MPKIVVVGAVAGGATCASQIRRLDKESDIIIFEKDRDMSFANCALPYVIGEVVEDRRYALAYT
PEKFYDRKQITVKTYHEVIAINDERQTVSVLNRKTNEQFEESYDKLILSPGASANSLGFESDIT
FTLRNLEDTDAIDQFIKANQVDKVLVVGAGYVSLEVLENLNERGLHPTLIHRSDKINKLMD
ADMNQPILDELDKREIPYRLNEEINAINGNEITFKSGKVEHYDMIIEGVGTHPNSKFIESSNI
KLDRKGFIPVNDKFETNVPNIYAIGDIATSHYRHVDLPASVPLAWGAHRAASIVAEQIAGN
DTIEFKGFLGNNIVKFFDYTFASVGVKPNELKQFDYKMVEVTQGAHANYYPGNSPLHLRV
YYDTSNRQILRAAAVGKEGADKRIDVLSMAMMNQLTVDELTEFEVAYAPPYSHPKDLINMI
GYKAK
10000000000000000000000000000000000000000000000000000000000000000000000000000000000
00000000000000000000000000000000000000000000000000000000000000000000000000000000000
00000000000000000000000000000000000000000000000000000000000000000000000000000000000
00000000000000000000000000000000000000000000000000000000000000000000000000000000000
00000000000000000000000000000000000000000000000000000000000000000000000000000000000
00000000000000000000000
>P78573_3dje_0_437
MAVTKSSSLLIVGAGTWGTSTALHLARRGYTNVTVLDPYPVPSAISAGNDVNKVISSGQYS
NNKDEIEVNEILAEEAFNGWKNDPLFKPYYHDTGLLMSACSQEGLDRLGVRVRPGEDPNL
VELTRPEQFRKLAPEGVLQGDFPGWKGYFARSGAGWAHARNALVAAAREAQRMGVKFV
TGTPQGRVVTLIFENNDVKGAVTGDGKIWRAERTFLCAGASAGQFLDFKNQLRPTAWTLV
HIALKPEERALYKNIPVIFNIERGFFFEPDEERGEIKICDEHPGYTNMVQSADGTMMSIPFEK
TQIPKEAETRVRALLKETMPQLADRPFSFARICWCADTANREFLIDRHPQYHSLVLGCGASG
RGFKYLPSIGNLIVDAMEGKVPQKIHELIKWNPDIAANRNWRDTLGRFGGPNRVMDFHD
VKEWTNVQYRDISKL
10000000000000000000000000000000000000000000000000000000000000000000000000000000000
00000000000000000000000000000000000000000000000000000000000000000000000000000000000
00000000000000000000000000000000000000000000000000000000000000000000000000000000000
00000000000000000000000000000000000000000000000000000000000000000000000000000000000
00000000000000000000000000000000000000000000000000000000000000000000000000000000000
00000000000000000000000
>Q24QE6_3hxl_0_437
MAAGTFTAQNKVRPGVYINFKSEPQAAGTLGERGIVSMPLILSWGEPGKMITIEAGDDVFP
KLGYSIMDAQLRLINEALKRAKTLLLYRLNAGTKAAVTVGNLTVTAKWGGARGNDITLVI
QENIDDETKFDVSTLVDGAELDKQTVSDIAGLAANDWVIFSGTGALTETAGAPLINGSDGA
VTNQAYIDYLAAVEIFDFNTIALPSTDDALKATFTAFAKRLRDDEGKKIQVVLENYPAADYE
GVISVKNGVVLADGTILTAAQATAWVAGATAGARVNESLTYQGYDEAVDVAPRYTNAQII
AALQAGEFLFTASDNQALVEQDINTLTSFTADKGKQFAKNRVIRVLDGINNDFVRIFSKFYI
GKVSNNADGRNLLKSECINYMNTLQDIDAIKNFDGQTDLTVQSGNDVDAVYIEAYAWPV
DSIEKIYVRVRIK

11111111111111000000000000111100000000000000000000000000000000000000000000000000000
00000000000000000000000000000000000000000000000000000000000000000000000000000000000
00000000000000000000000000000000000000000000000000000000000000000000000000000000000
00000000000000000000000000000000000000000000000000000000000000000000000000000000000
00000000000000000000000000000000011110000000000000000000000000000000000000000110000
00000000000000000000000
>P08563_4b3v_582_1017
EEAFTYLCTAPGCATQTPVPVRLAGVRFESKIVDGGCFAPWDLEATGACICEIPTDVSCEGL
GAWVPTAPCARIWNGTQRACTFWAVNAYSSGGYAQLASYFNPGGSYYKQYHPTACEVEP
AFGHSDAACWGFPTDTVMSVFALASYVQHPHKTVRVKFHTETRTVWQLSVAGVSCNVTT
EHPFCNTPHGQLEVQVPPDPGDLVEYIMNYTGNQQSRWGLGSPNCHGPDWASPVCQRHS
PDCSRLVGATPERPRLRLVDADDPLLRTAPGPGEVWVTPVIGSQARKCGLHIRAGPYGHAT
VEMPEWIHAHTTSDPWHPPGPLGLKFKTVRPVALPRALAPPRNVRVTGCYQCGTPALVEG
LAPGGGNCHLTVNGEDVGAFPPGKFVTAALLNTPPPYQVSCGGESDRASARVIDPAAQSF
TGVVYGTHTTAVSETR
10000000000000000000000000000000000000000000000000000000000000000000000000000000000
00000000000000000000000000000000000000000000000000000000000000000000000000000000000
00000000000000000000000000000000000000000001100000000000000000000000000000000000000
00000000000000000000000000000000000000000000000000000000000000000000000000000000000
00000000000000000000000000000000000000000000000000000000000000000000000000000000000
000000000000000000001
>P35021_1skq_0_434
MSQKPHLNLIVIGHIDHGKSTLVGRLLMDRGFIDEKTVKEAEEAAKKLGKESEKFAFLLDRL
KEERERGVTINLTFMRFETKKYFFTIIDAPGHRDFVKNMITGASQADAAILVVSAKKGEYEA
GMSVEGQTREHIILAKTMGLDQLIVAVNKMDLTEPPYDEKRYKEIVDQVSKFMRSYGFNTN
KVRFVPVVAPAGDNITHRSENMKWYNGPTLEEYLDQLELPPKPVDKPLRIPIQDVYSISGVG
TVPVGRVESGVLKVGDKIVFMPAGKVGEVRSIETHHTKMDKAEPGDNIGFNVRGVEKKDI
KRGDVVGHPNNPPTVADEFTARIIVVWHPTALANGYTPVIHVHTASVACRVSELVSKLDPR
TGQEAEKNPQFLKQGDVAIVKFKPIKPLCVEKYNEFPPLGRFAMRDMGKTVGVGIIVDVKP
AKVEIK
11100000000000000000000000000000000000000000000000000000000000001111111111100000000
00000000000000000000000000000000000000000000000000000000000000000000000000000000000
00000000000000000000000000000000000000000000000000000000000000000000000000000000000
00000000000000000000000000000000000000000000000000000000000000000000000000000000000
00000000000000000000000000000000000000000000000000000000000000000000000000000000000
00000000000000011111
>Q60393_2vxr_862_1296
SNISSNAILSLSYRGGRLIDSSGYGATMNVGSDVIFNDIGNGQFKLNNSENSNITAHQSKFVV
YDSMFDNFSINFWVRTPKYNNNDIQTYLQNEYTIISCIKNDSGWKVSIKGNRIIWTLIDVNA
KSKSIFFEYSIKDNISDYINKWFSITITNDRLGNANIYINGSLKKSEKILNLDRINSSNDIDFKLI
NCTDTTKFVWIKDFNIFGRELNATEVSSLYWIQSSTNTLKDFWGNPLRYDTQYYLFNQGMQ
NIYIKYFSKASMGETAPRTNFNNAAINYQNLYLGLRFIIKKASNSRNINNDNIVREGDYIYL
NIDNISDESYRVYYVLVNSKEIQTQLFLAPINDDPTFYDVLQIKKYYEKTTYNCQILCEKDTKT
FGLFGIGKFVKDYGYVWDTYDNYFCISQWYLRRISENINKLRLGCNWQFIPVDEGWTE
11111000000000000000000000000000000000000000000000000000000000000000000000000000000
00000000000000000000000000000000000000000000000000000000000000000000000000000000000
00000000000000000000000000000000000000000000000000000000000000000000000000000000000
00000000000000000000000000000000000000000000000000000000000000000000000000000000000
00000000000000000000000000000000000000000000000000000000001110000000000000000000000
00000000000000000000
>Q6NF81_3htl_51_485

PERTSIAVHALMGLPTGQPANGTKLDSIGLPKVDGMSFTLYRVNEIDLTTQAGWDAASKIK
LEELYTNGHPTDKVTKVATKKTEGGVAKFDNLTPALYLVVQELNGAEAVVRSQPFLVAAPQ
TNPTGDGWLQDVHVYPKHQALSEPVKTAVDPDATQPGFSVGENVKYRVATKIPEIASNTKF
EGFTVADKLPAELGKPDTNKITVTLGGKPINSTDVSVQTYQVGDRTVLSVQLAGATLQSLD
QHKDQELVVEFEAPVTKQPENGQLDNQAWVLPSNPTAQWDPEESGDAALRGMPSSRVSS
KFGQITIEKSFDGNTPGADRTATFQLHRCEADGSLVKSDPPISLDGKQEFVTGQDGKAVLSG
IHLGTLQLESNVMKYTDAWAGKGTEFCLVETATASGYELLPKPVIVKLEANESTNVLVEQK
VKIDNKKKN
10000000000000001111111111000000000000000000000000000000000000000000000000000000000
00000000000000000000000000000000000000000000000000000000000000000000000000000000000
00000000000000000000000000000000000000000000000000000000000000000000000000000000000
00000000000000000000000000000000000000000000000000000000000000000000000000000000000
00000000000000000000000000000000000000000000000000000000000000000000000000000000000
0000000000000000011
>O35795_3cj7_27_460
PTQDVREPPALKYGIVLDAGSSHTSMFVYKWPADKENDTGIVGQHSSCDVQGGGISSYAN
DPSKAGQSLVRCLEQALRDVPRDRHASTPLYLGATAGMRPFNLTSPEATARVLEAVTQTLT
QYPFDFRGARILSGQDEGVFGWVTANYLLENFIKYGWVGRWIRPRKGTLGAMDLGGASTQ
ITFETTSPSEDPGNEVHLRLYGQHYRVYTHSFLCYGRDQILLRLLASALQIHRFHPCWPKGYS
TQVLLQEVYQSPCTMGQRPRAFNGSAIVSLSGTSNATLCRDLVSRLFNISSCPFSQCSFNGVF
QPPVAGNFIAFSAFYYTVDFLTTVMGLPVGTLKQLEEATEITCNQTWTELQARVPGQKTRL
ADYCAVAMFIHQLLSRGYHFDERSFREVVFQKKAADTAVGWALGYMLNLTNLIPADLPGL
RKGTHF
11111111000000000000000000000000000000000000000000000000000000000000000000000000000
00000000000000000000000000000000000000000000000000000000000000000000000000000000000
00000000000000000000000000000000000000000000000000000000000000000000000000000000000
00000000000011111110000000000000000000000000000000000000000000000000000000000000000
00000000000000000000000000000000000000000000000000000000000000000000000000000000000
0000000000000000000
>Q383X0_3h9u_3_436
YKVRDISLAEWGRRELELAENEMPGLMELRREYGPSKPLKGAKIAGCLHMTMQTAVLIETL
VELGAEVRWASCNIFSTQDHAAAAIAKRGIPVFAWKGETEEEYMWCMKQTLKGFSGDGY
PNMLLDDGGDLTNYVLDECKELDGKIYGVSEETTTGVKNLYKRLQRGKLTIPAMNVNDSV
TKSKFDNLYGCRESLVDGIKRATDVMIAGKTACVCGYGDVGKGCAAALRGFGARVVVTEV
DPINALQAAMEGYQVLLVEDVVEEAHIFVTTTGNDDIITSEHFPRMRDDAIVCNIGHFDTEI
QVAWLKANAKERVEVKPQVDRYTMANGRHIILLAEGRLVNLGCASGHPSFVMSNSFCNQ
VLAQIELWTNRDTGKYPRGAKAQVYFLPKKLDEKVAALHLGKLGAKLTKLTPKQAEYINC
PVDGPFKPDHYRY
00000000000000000000000000000000000000000000000000000000000000000000000000000000000
00000000000000000000000000000000000000000000000000000000100000000000000000000000000
00000000000000000000000000000000000000000000000000000000000000000000000000000000000
00000000000000000000000000000000000000000000000000000000000000000000000000000000000
00000000000000000000000000000000000000000000000000000000000000000000000000000000000
0000000000000000000
>Q4DA54_3lc0_44_477
QKNMVETEPVQGCRDFPPEAMRCRRHLFDVFHATAKTFGFEEYDAPVLESEELYIRKAGEE
ITEQMFNFITKGGHRVALRPEMTPSLARLLLGKGRSLLLPAKWYSIPQCWRYEAITRGRRRE
HYQWNMDIVGVKSVSAEVELVCAACWAMRSLGLSSKDVGIKVNSRKVLQTVVEQAGVTS
DKFAPVCVIVDKMEKIPREEVEAQLAVLGLEPTVVDAITTTLSLKSIDEIAQRVGEEHEAVKE
LRQFFEQVEAYGYGDWVLFDASVVRGLAYYTGIVFEGFDREGKFRALCGGGRYDNLLTTYG
SPTPIPCAGFGFGDCVIVELLQEKRLLPDIPHVVDDVVIPFDESMRPHALAVLRRLRDAGRS

ADIILDKKKVVQAFNYADRVGAVRAVLVAPEEWERGEVQVKMLREGTGKEEGGAERGFAV
PLDRLV
11000000000000000000000000000000000000000000000000000000000000000000000000000000000
00000000000000000000000000000000111110000000000000000000000000000000000000000000000
00000000000000000000000000000000000000000000000000000000000000000000000000000000000
00000000000000000000000000000000000000000000000000000000000000000000000000000000000
00000000000000000000000000000000000000000000000000000000000000000000000000000001111
1111111100000000000
>Q9X9I8_2fn0_0_433
MKISEFLHLALPEEQWLPTISGVLRQFAEEECYVVERPPCWYLGKGCQARLHINADGTQAT
FIDDAGEQKWAVDSIADCARRFMAHPQVKGRRVYGQVGFNFAAHARGIAFNAGEWPLLT
LTVPREELIFEKGNVTVYADSADGCRRLCEWVKEASTTTQNAPLAVDTALNGEAYKQQVA
RAVAEIRRGEYVKVIVSRAIPLPSRIDMPATLLYGRQANTPVRSFMFRQEGREALGFSPELVM
SVTGNKVVTEPLAGTRDRMGNPEHNKAKEAELLHDSKEVLEHILSVKEAIAELEAVCLPGS
VVVEDLMSVRQRGSVQHLGSGVSGQLAENKDAWDAFTVLFPSITASGIPKNAALNAIMQI
EKTPRELYSGAILLLDDTRFDAALVLRSVFQDSQRCWIQAGAGIIAQSTPERELTETREKLASI
APYLMV
00000000000000000000000000000000000000000000000000000000000000000000000000000000000
00000000000000000000000000000000000000000000000000000000011111111111111111111100000
00000000000000000000000000000000000000000000000000000000000000000000000000000000000
00000000000000000000000000000000000000000000000000000000000000000000000000000000000
00000000000000000000000000000000000000000000000000000000000000000000000000000000000
0000000000000000000
>D4GTL2_3oyz_0_432
MTERRHDREFVRTFFTSPTAVEGEDDSAKMLRRAAGLRGMQAPDVWVPDNEDATAPSMR
DEGAENIVEVISEQGAEFPGEIHPRMVWHRDSPETRYQGFQHMLDITDPERGAVEHIHGFV
IPEVGGIDDWKKADEFFTIVEHEHGLDEGSLAMSVIESGEAELAMGDLRDEMGKPTNNLE
RLFLLVDGEVDYTKDMRAMTPTGELPAWPELRHNTSRGASAAGCVAVDGPYDDIRDVEGY
RERMTDNQAKGMLGIWSLTPGQVVEANTSPLPPKTGSWLLDADGEEVELASEDGVEAYD
GDRLSLEATDGGYELRVGGDARELTADELREELLGLTSYVPSMDDIVDSMEEFEAAKEAGR
GAIAMTQSATLRIGGTEIDIEKDRMWDEATYQAAMTPISLFQDVYENRPDQHEELEERYGA
GVVERAMEVGL
11110000000000000000000000000000000000000000000000000000000000000000000000000000000
00000000000000000000000000000000000000000000000000000000000000000000000000000000000
00000000000000000000000000000000000000000000000000000000000000000000000000000000000
00000000000000000000000000000000000111111111111111111111111111111111111111111100000
00000000000000000000000000000000000000011111111000000000000000000000000000000000000
000000000000000001
>Q72DN3_3m1u_31_463
SRPATPPVTPPSREGHVADLDRFPQDLRVYAMKAGADRQLLPFTEQAAQDARWNRRFFAP
WRMTRISVPVKDVAAPFGTDGRPRGYAENLLPWDVTRWGALASGAALDLYPSQAWKGIV
VSNSALREVPTLRPMFTAPTRAGQGYPFDMFQRTAVWMGTPVFVGHATADRAWLYVETAF
AAGWMPAADVARVDDAFMTRYESGSLAAILRDDTSLNGADGTHLATAHIGTVLPLSGAS
QVGRTVLVPVRAPEGHAVVVPVLLTSGEAAQKPVPLTPGNMAELGNRMMGQPYGWGGL
YEDRDCSSTLRDLFTPFGLWLPRNSASQAKAGRYVDIAKLDADDKEARIVAEGVPFMTLLW
LRGHITLYLGLHEGQAAMFHNMWGIRTHRGGVEGRYVLGRAVVTSTRPGLDVPGNDNA
DGLLGRMQGMSILPGGAQ
00000000000000000000000000000000000000000000000000000000000000000000000000000000000
00000000000000000000000000000000000000000000000000000000000000000000000000000000000
00000000000000000000000000000000000000000000000000000000000000000000000000000000000
00000000000000000000000000000000000000000000000000000000000000000000000000000000000

00000000000000000000000000000000000000000000000000000000000000000000000000000000000
000000000000000111
>P08169_1q25_44_475
AAGTQGAEFPELCSYTWEAVDTKNNMLYKINICGNMGVAQCGPSSAVCMHDLKTDSFHS
VGDSLLKTASRSLLEFNTTVNCKQQNHKIQSSITFLCGKTLGTPEFVTATDCVHYFEWRTTA
ACKKNIFKANKEVPCYAFDRELKKHDLNPLIKTSGAYLVDDSDPDTSLFINVCRDIEVLRAS
SPQVRVCPTGAAACLVRGDRAFDVGRPQEGLKLVSNDRLVLSYVKEGAGQPDFCDGHSPA
VTITFVCPSERREGTIPKLTAKSNCRFEIEWVTEYACHRDYLESRSCSLSSAQHDVAVDLQPLS
RVEASDSLFYTSEADEYTYYLSICGGSQAPICNKKDAAVCQVKKADSTQVKVAGRPQNLTL
RYSDGDLTLIYFGGEECSSGFQRMSVINFECNQTAGNNGRGAPVFTGEVDCTYFFTWDTKY
ACV
11110000000000000000000000000000000000000000000000000000000000000000000000000000000
00000000000000000000000000000000000000000000000000000000000000000000000000000000000
00000000000000000000000000000000000000000000000000000000000000000000000000000000000
00000000000000000000000000000000000000000000000000000000000000000000000000000000000
00000000000000000000000000000000000000000000000000000000000000000000000000000000000
00000000000000000
>Q5NIQ1_4eei_0_431
MIKRYDVAEISKIWADENKYAKMLEVELAILEALEDRMVPKGTAAEIRARAQIRPERVDEIE
KVTKHDIIAFCTSIAEQFTAETGKFFHFGVTSSDIIDSALSLQIRDSMSYVIKDLEALCDSLLTK
AEETKEIITMGRSHGMFAEPMSFGQKFLGAYVEFKRRLKDLKDFQKDGLTVQFSGAVGNYC
ILTTEDEKKAADILGLPVEEVSTQVIPRDRIAKLISIHGLIASAIERLAVEIRHLHRSDVFEVYE
GFSKGQKGSSTMPHKKNPISTENLTGMARMLRSHVSIALENCVLWHERDISHSSAERFYLP
DNFGIMVYALRRMKNTIDNLVVQRDIIEDRVRSTSAYLSSFYLHFLVANTPFMREDCYKIVQ
QVAFDLKQGESFSKKLQKVMHDEHNIILDIPEMDFEGIKKTYLKEIDHVFDRSVKA
00000000000000000000000000000000000000000000000000000000000000000000000000000000000
00000000000000000000000000000000000000000000000000000000000000000000000000000000000
00000000000000000000000000000000000000000000000000000000000000000000000000000000000
00000011111111111100000000000000000000000000000000000000000000000000000000000000000
00000000000000000000000000000000000000000000000000111000000000000000000000000000000
00000000000000000
>Q6N7D3_3pfo_0_431
MATETLTKSDAITQSLRAAVDRNFNDQVAFLQRMVQFRSVRGEEAPQQEWLAQQFADRG
YKVDTFSLADVDIASHPKAAPMDTIDPAGSMQVVATADSDGKGRSLILQGHIDVVPEGPVD
LWSDPPYEAKVRDGWMIGRGAQDMKGGVSAMIFALDAIRTAGYAPDARVHVQTVTEEES
TGNGALSTLMRGYRADACLIPEPTGHTLTRAQVGAVWFRLRVRGTPVHVAYSETGTSAILS
AMHLIRAFEEYTKELNAQAVRDPWFGQVKNPIKFNVGIIKGGDWASSTAAWCELDCRLGL
LTGDTPQEAMRGIEKCLADAQATDSFLSENPAELVWSGFQADPAVCEPGGVAEDVLTAAH
KAAFNAPLDARLSTAVNDTRYYSVDYGIPALCYGPYGQGPHAFDERIDLESLRKTTLSIALF
VAEWCGLRKL
11111100000000000000000000000000000000000000000000000000000000000000000000000000000
00000000000000000000000000000000000000000000000000000000000000000000000000000000000
00000000000000000000000000000000000000000000000000000000000000000000000000000000000
00000000000000000000000000000000000000000000000000000000000000000000000000000000000
00000000000000000000000000000000000000000000000000000000000000000000000000000000000
00000000000000000
>Q9Y253_4ecw_0_431
MATGQDRVVALVDMDCFFVQVEQRQNPHLRNKPCAVVQYKSWKGGGIIAVSYEARAFGV
TRSMWADDAKKLCPDLLLAQVRESRGKANLTKYREASVEVMEIMSRFAVIERASIDEAYVD
LTSAVQERLQKLQGQPISADLLPSTYIEGLPQGPTTAEETVQKEGMRKQGLFQWLDSLQIDN
LTSPDLQLTVGAVIVEEMRAAIERETGFQCSAGISHNKVLAKLACGLNKPNRQTLVSHGSV

PQLFSQMPIRKIRSLGGKLGASVIEILGIEYMGELTQFTESQLQSHFGEKNGSWLYAMCRGIE
HDPVKPRQLPKTIGCSKNFPGKTALATREQVQWWLLQLAQELEERLTKDRNDNDRVATQL
VVSIRVQGDKRLSSLRRCCALTRYDAHKMSHDAFTVIKNCNTSGIQTEWSPPLTMLFLCAT
KFSAS
00000000000000000000000000000000000000000000000000000000000000000000000000000000000
00000000000000000000000000000000000000000000000000000000000000000000000111000000000
00000000000000000000000000000000000000000000000000000000000000000000000000000000000
00000000000000000000000000000000000000000000000000000000000000000000000000000000000
00000000000000000000000000000000000000000000000000000000000000000000000000000000000
0000000000000000
>Q1GYA6_3k1t_0_430
MMVPHLTTALTGPLLTLEKRLLDNMPRIEHWFRSQWQEYGAPFYASVDLRNAGFKLAPVD
TNLFPGGFNNLNPDFLPLCIQAAMVAVEKICPDARRLLLIPENHTRNTFYLRNVHALTHILR
QAGLEVRIGSIAPEITAPTFLETHDGHSILLEPVRRKANRLELDNFDSCAILLNNDLSGGIPDI
LQGLEQSLIPPLHAGWATRRKSNHFTAYDRVVEEFAPLIDIDPWLLNPYFDTCGGLDFHARL
GEEQLAEKVDSLLAKIRRKYAEYGVKQEPFVIVKADAGTYGMGIMTVKSADDVRDLNRKQ
RNKMSVVKEGLKVSEVILQEGVYTFEHLKDAVAEPVIYMMDHFVVGGFYRVHTSRGADEN
LNAPGMHFEPLTFETPCSTPDCAGAPDAAPNRFYAYGVVARLALLAATIELQETDPDLLDE
RT
00000000000000000000000000000000000000000000000000000000000000000000000000000000000
00000000000000000000000000000000000000000000000000000000000000000000000000000000000
00000000000000000000000000000000000000000000000000000000000000000000000000000000000
00000000000000000000000000000000000000000000000000000000000000000000000000000000000
00000000000000000000000000000000000000000000000000000000000000000000000000000000000
0000000011111111
>Q6QT42_1uuq_25_455
VAESNSAVAPTANVATSPAHEHFVRVNGGHFELQGKPYVITGVNMWYAAYLGAPNEVGD
RDRLAKELDNLKAIGVNNLRVLAVSEKSEINSAVKPAVTNGFGNYDETLLQGLDYLLVELA
KRDMTVVLYFNNFWQWSGGMTQYMAWIEGEPVQDPNVTNEWEAFMAKSASFYRSEKA
QQEYRKTLEKIITRVNSINGKAYVDDATIMSWQLANEPRPGNSQTTAEEKQIYIDWVHAAA
AYIKTLDAHHLVSSGSEGEMGSVNDMQVFIDAHATPDIDYLTYHMWIRNWSWFDKTKPAE
TWPSAWEKAQNYMRAHIDVAKQLNKPLVLEEFGLDRDMGSYAMDSTTEYRDNYFRGVFE
LMLASLEQGEPSAGYNIWAWNGYGRTTRANYWWQEGDDFMGDPPQEEQGMYGVFDTD
TSTIAIMKEFNARFQPK
11111111111111111111000000000000000000000000000000000000000000000000000000000000000
00000000000000000000000000000000000000000000000000000000000000000000000000000000000
00000000000000000000000000000000000000000000000000000000000000000000000000000000000
00000000000000000000000000000000000000000000000000000000000000000000000000000000000
00000000000000000000000000000000000000000000000000000000000000000000000000000000000
0000000000000001
>P03518_4hj1_690_1118
CSELIQASSRITTCSTEGVNTKCRLSGTALIRAGSVGAEACLMLKGVKEDQTKFLKIKTVSSE
LSCREGQSYWTGSISPKCLSSRRCHLVGECHVNRCLSWRDNETSAEFSFVGESTTMRENKCF
EQCGGWGCGCFNVNPSCLFVHTYLQSVRKEALRVFNCIDWVHKLTLEITDFDGSVSTIDLG
ASSSRFTNWGSVSLSLDAEGISGSNSFSFIESPSKGYAIVDEPFSEIPRQGFLGEIRCNSESSVLS
AHESCLRAPNLISYKPMIDQLECTTNLIDPFVVFERGSLPQTRNDKTFAASKGNRGVQAFSK
GSVQADLTLMFDNFEVDFVGAAVSCDAAFLNLTGCYSCNAGARVCLSITSTGTGSLSAHN
KDGSLHIVLPSENGTKDQCQILHFTVPEVEEEFMYSCDGDERPLLVKGTLIAIDP
00000000000000000000000000000000000000000000000000000000000000000000000000000000000
00000000000000000000000000000000000000000000000000000000000000000000000000000000000
00000000000000000000000000000000000000000000000000000000000000000000000000000000000

00000000000000000000000000000000000000000000000000000000000000000000000000000000000
00000000000000000000000000000000000000000000000000000000000000000000000000000000000
0000000000001
>P22219_3gre_1026_1453
EGDVESIEKFLSTFKILPPLRDYKEFGPIQEIVRSPNMGNLRGKLIATLMENEPNSITSSAVSPG
ETPYLITGSDQGVIKIWNLKEIIVGEVYSSSLTYDCSSTVTQITMIPNFDAFAVSSKDGQIIVLK
VNHYQQESEVKFLNCECIRKINLKNFGKNEYAVRMRAFVNEEKSLLVALTNLSRVIIFDIRTL
ERLQIIENSPRHGAVSSICIDEECCVLILGTTRGIIDIWDIRFNVLIRSWSFGDHAPITHVEVCQ
FYGKNSVIVVGGSSKTFLTIWNFVKGHCQYAFINSDEQPSMEHFLPIEKGLEELNFCGIRSLN
ALSTISVSNDKILLTDEATSSIVMFSLNELSSSKAVISPSRFSDVFIPTQVTANLTMLLRKMKRTS
THSVDDSLYHHDIINSISTCEVDETPLLVACDNSGLIGIFQ
11110000000000010000000000000000000000000000000000000000000000000000000000000000000
00000000000000000000000000000000000000000000000000000000000000000000000000000000000
00000000000000000000000000000000000000000000000000000000000000000000000000000000000
00000000000000000000000000000000000000000000000000000000000000000000000000000000000
00000000000000000000000000001100000000000000000000001111111111111000000000000000000
000000000000
>P25524_3r0d_0_426
MSNNALQTIINARLPGEEGLWQIHLQDGKISAIDAQSGVMPITENSLDAEQGLVIPPFVEPH
IHLDTTQTAGQPNWNQSGTLFEGIERWAERKALLTHDDVKQRAWQTLKWQIANGIQHVR
THVDVSDATLTALKAMLEVKQEVAPWIDLQIVAFPQEGILSYPNGEALLEEALRLGADVVG
AIPHFEFTREYGVESLHKTFALAQKYDRLIDVHCDEIDDEQSRFVETVAALAHHEGMGARV
TASHTTAMHSYNGAYTSRLFRLLKMSGINFVANPLVNIHLQGRFDTYPKRRGITRVKEMLES
GINVCFGHDDVFDPWYPLGTANMLQVLHMGLHVCQLMGYGQINDGLNLITHHSARTLN
LQDYGIAAGNSANLIILPAENGFDALRRQVPVRYSVRGGKVIASTQPAQTTVYLEQPEAIDY
KR
11110000000000000000000000000000000000000000000000000000000000000000000000000000000
00000000000000000000000000000000000000000000000000000000000000000000000000000000000
00000000000000000000000000000000000000000000000000000000000000000000000000000000000
00000000000000000000000000000000000000000000000000000000000000000000000000000000000
00000000000000000000000000000000000000000000000000000000000000000000000000000000000
00000000001
>P37352_1i7o_0_426
MKGTIFAVALNHRSQLDAWQEAFQQSPIKAPPKTAVWFIKPRNTVIGCGEPIPFPQGENLLS
GATVALIVGKTATKVREEDAAEYIAGYALANDVSLPEESFYRPAIKAKCRDGFCPIGETVALS
NVDNLTIYTEINGRPADHWNTSDLQRNAAQLLSALSEFATLNPGDAILLGTPQARVEIQPG
DRVRVLAEGFPPLENPVVDEREVTTRKSFPTLPHPHGTLFALGLNYADHASELEFKPPEEPL
VFLKAPNTLTGDNQTSVRPNNIEYMHYEAELVVVIGKQARNVSEADAMDYVAGYTVCND
YAIRDYLENYYRPNLRVKSRDGLTPMLSTIVPKEAIPDPHNLTLRTFVNGELRQQGTTADLIF
SVPFLIAYLSEFMTLNPGDMIATGTPKGLSDVGDEVVVEVEGVGRLVNRIVSEETAK
00000000000000000000000000000000000000000000000000000000000000000000000000000000000
00000000000000000000000000000000000000000000000000000000000000000000000000000000000
00000000000000000000000000000000000000000000000000000000000000000000011111111000000
00000000000000000000000000000000000000000000000000000000000000000000000000000000000
00000000000000000000000000000000000000000000000000000000000000000000000000000000000
00000000000
>Q4K4B6_3gwb_52_478
ELDGKAPSHRNLNVQTWSTAEGAKVLFVEARELPMFDLRLIFAAGSSQDGNAPGVALLTN
AMLNEGVAGKDVGAIAQGFEGLGADFGNGAYKDMAVASLRSLSAVDKREPALKLFAEVV
GKPTFPADSLARIKNQMLAGFEYQKQNPGKLASLELMKRLYGTHPYAHASDGDAKSIPPIT
LAQLKAFHAKAYAAGNVVIALVGDLSRSDAEAIAAQVSAALPKGPALAKIEQPAEPKASIG

HIEFPSSQTSLMLAQLGIDRDDPDYAAVSLGNQILGGGGFGTRLMSEVREKRGLTYGVYSGF
TPMQARGPFMINLQTRAEMSEGTLKLVQDVFAEYLKNGPTQKELDDAKRELAGSFPLSTAS
NADIVGQLGAMGFYNLPLSYLEDFMRQSQELTVEQVKAAMNKHLNVDKMVIVSAGPTVA
QKPL
11111111111000000000000000000000000000000000000000000000000000000000000000000000000000000000000
00000000000000000000000000000000000000000000000000000000000000000000000000000000000000000000000
00000000000000000000000000000000000000000000000000000000000000000000000000000000000000000000000
00000000000000000000000000000000000000000000000000000000000000000000000000000000000000000000000
00000000000000000000000011100000000000000000000000000000000000000000000000000000000000000000000
000000000000
>D9TQ02_2yjg_0_425
MANIEIPYGKSKLAFDLPDERIQGILRSKAGSYKVNMSEEDIVKRALENPIGTKRLQDLAEG
KKNIVIITSDHTRPVPSRITLPLLLDEIRKKNKSANVKILIATGFHRGTTLQEMKAKFGEDLVE
NEQFVVHDSRNSENMELIGTLPSGGKLEINKLAVEADLLVAEGFIEPHFFAGFSGGRKSILPG
IASVQCILANHCSEFIKNPYARTGVLENNPIHRDMIYAAKKANLAFILNVVIDSSHKIVNAF
AGHSEKAHLKGCEFVSEIATVNAKPADIVITSNGGYPLDQNIYQSVKGMTAGEAACKDGG
VIIIAAECADGHGGEGFYRWFKESKDPQDVMNKILSRGRDETLPDQWEAQILARILINHKVI
MVTDSKNYEYVKDMFMTPAKDLGEALKIAESIVNNDSKINVIPDGVSVIVREK
10000000000000000000000000000111111100000000000000000000000000000000000000000000000000000000000
00000000000000000000000000000000000000000000000000000000000000000000000000000000000000000000000
00000000000000000000000000000000000000000000000000000000000000000000000000000000000000000000000
00000000000000000000000000000000000000000000000000000000000000000000000000000000000000000000000
00000000000000000000000000000000000000000000000000000000000000000000000000000000000000000000000
00000000000
>Q6M8B5_3ppl_0_425
MSSVSLQDFDAERIGLFHEDIKRKFDELKSKNLKLDLTRGKPSSEQLDFADELLALPGKGDF
KAADGTDVRNYGGLDGIVDIRQIWADLLGVPVEQVLAGDASSLNIMFDVISWSYIFGNND
SVQPWSKEETVKWICPVPGYDRHFSITERFGFEMISVPMNEDGPDMDAVEELVKNPQVKG
MWVVPVFSNPTGFTVTEDVAKRLSAMETAAPDFRVVWDNAYAVHTLTDEFPEVIDIVGLG
EAAGNPNRFWAFTSTSKITLAGAGVSFFLTSAENRKWYTGHAGIRGIGPNKVNQLAHARY
FGDAEGVRAVMRKHAASLAPKFNKVLEILDSRLAEYGVAQWTVPAGGYFISLDVVPGTAS
RVAELAKEAGIALTGAGSSYPLRQDPENKNLRLAPSLPPVEELEVAMDGVATCVLLAAAEH
YAN
10000000000000000000000000000000000000000000000000000000000000000000000000000000000000000000000
00000000000000000000000000000000000000000000000000000000000000000000000000000000000000000000000
00000000000000000000000000000000000000000000000000000000000000000000000000000000000000000000000
00000000000000000000000000000000000000000000000000000000000000000000000000000000000000000000000
00000000000000000000000000000000000000000000000000000000000000000000000000000000000000000000000
00000000001
>Q8DP63_2c1i_37_462
FKIYQQKSFEQKIESLKKEKDDQLSEGNQKEHFRQGQAEVIAYYPLQGEKVISSVRELINQD
VKDKLESKDNLVFYYTEQEESGLKGVVNRNVTKQIYDLVAFKIEETEKTSLGKVHLTEDGQP
FTLDQLFSDASKAKEQLIKELTSFIEDKKIEQDQSEQIVKNFSDQDLSAWNFDYKDSQIILYPS
PVVENLEEIALPVSAFFDVIQSSYLLEKDAALYQSYFDKKHQKVVALTFDDGPNPATTPQVL
ETLAKYDIKATFFVLGKNVSGNEDLVKRIKSEGHVVGNHSWSHPILSQLSLDEAKKQITDTE
DVLTKVLGSSSKLMRPPYGAITDDIRNSLDLSFIMWDVDSLDWKSKNEASILTEIQHQVANG
SIVLMHDIHSPTVNALPRVIEYLKNQGYTFVTIPEMLNTRLKAHELYYSRDE
11111111000000000000000000000000000000000000000000000000000000000000000000000000000000000000000
00000000000000001111100000000000000000000000000000000000000000000000000000000111111111111111111
11100000000000000000111111111000000000000000000000000000000000000000000000000000000000000000000
00000000000000000000000000000000000000000000000000000000000000000000000000000000000000000000000

00000000000000000000000000000000000000000000000000000000000000000000000000000000
00000000000
>Q09028_2xu7_0_424
MADKEAAFDDAVEERVINEEYKIWKKNTPFLYDLVMTHALEWPSLTAQWLPDVTRPEGKD
FSIHRLVLGTHTSDEQNHLVIASVQLPNDDAQFDASHYDSEKGEFGGFGSVSGKIEIEIKINH
EGEVNRARYMPQNPCIIATKTPSSDVLVFDYTKHPSKPDPSGECNPDLRLRGHQKEGYGLS
WNPNLSGHLLSASDDHTICLWDISAVPKEGKVVDAKTIFTGHTAVVEDVSWHLLHESLFGS
VADDQKLMIWDTRSNNTSKPSHSVDAHTAEVNCLSFNPYSEFILATGSADKTVALWDLRN
LKLKLHSFESHKDEIFQVQWSPHNETILASSGTDRRLNVWDLSKIGEEQSPEDAEDGPPELL
FIHGGHTAKISDFSWNPNEPWVICSVSEDNIMQVWQMAENIYNDEDPEGSVDPEGQGS
11111111110000000000000000000000000000000000000000000000000000000000000000000000
00000111111111111111111111110000000000000000000000000000000000000000000000000000
00000000000000000000000000000000000000000000000000000000000000000000000000000000
00000000000000000000000000000000000000000000000000000000000000000000000000000000
00000000000000000000001111000000000000000000000000000000000000000000000000011111
1111111111
>Q11U39_3h9m_1_425
SFTPLHTTSEAFIEKALPWLEDRYFHIAYLNPNGYTAYPQGAFRHYLAFGSEAAIHVSDATR
VFETWNEIKKGYTNEWIFVFASYDGKNSVEQLHTSKEAGIAFAAATFFIPEHVWEIQPDGILI
HKGSGSSLVTEIQHAEPSTPVQQSDIFVKQVVSKESYFNAFDELQQIIAQGDAYEINYCIPFTA
KGNISPAATYQRLNKKTPMPFSVYYKFNTEYILSASPERFIKKTGDTIISQPIKGTSKRGKSKA
EDEMLKQQLGTSEKEQSENTMIVDLVRNDLSRTAVAGSVCVPELSGLYTFPNVHQLISTVQS
TIDPACSSIDVIQQAFPMGSMTGAPKVNVMKFIDRIESMARGPFSGTVGYMDPHDNFDFNV
LIRSIFYNSATQELFMEAGSAITSYAKAETEYEECLLKITPMIHILNNQ
00000000000000000000000000000000000000000000000000000000000000000000000000000000
00000000000000000000000000000000000000000000000000000001111110000000000000000000
00000000000000000000000000000000000000000000000000000000000000000000000000111111
11111111111100000000000000000000000000000000000000000000000000000000000000000000
00000000000000000000000000000000000000000000000000000000000000000000000000000000
0000000011
>Q65D11_3vot_0_424
MTKRNKNLAIICQNKHLPFIFEEAERLGLKVTFFYNSAEDFPGNLPAVERCVPLPLFEDEEA
AMDVVRQTFVEFPFDGVMTLFEPALPFTAKAAEALNLPGLPFTTMENCRNKNKTRSILQQ
NGLNTPVFHEFHTLADLENRKLSYPLVVKPVNGFSSQGVVRVDDRKELEEAVRKVEAVNQ
RDLNRFVHGKTGIVAEQFIDGPEFAIETLSIQGNVHVLSIGYKGNSKGPFFEEGVYIAPAQLK
EETRLAIVKEVTGAVSALGIHQGPAHTELRLDKDGTPYVIEVGARIGGSGVSHYIVKESTGIN
FMQLVLQNALKPLESSEFEGEIRPVRTAGNYIIPVQGSGTFEKIDGLEEVKQRQEVKRVFQFM
RRGAKILPYPHFSGYPGFILTSHHSYEECEAFYRELDDELHIIYQNNLTGTIGG
10000000000000000000000000000000000000000000000000000000000000000000000000000000
00000000000000000000000000000000000000000000000000000000000000000000011111000000
00000000000000000000000000000000000000000000000000000000000000000000000000000000
00000000000000000000000000000000000000000000000000000000000000000000000000000000
00000000000000000000000000000000000000000000000000000000000000000000000000000000
0011111111
>Q8PU56_3ka7_0_424
MKTVVIGAGLGGLLSAARLSKAGHEVEVFERLPITGGRFTNLSYKGFQLSSGAFHMLPNGP
GGPLACFLKEVEASVNIVRSEMTTVRVPLKKGNPDYVKGFKDISFNDFPSLLSYKDRMKIAL
LIVSTRKNRPSGSSLQAWIKSQVSDEWLIKFADSFCGWALSLKSDEVPVEEVFEIIENMYRFG
GTGIPEGGCKGIIDALETVISANGGKIHTGQEVSKILIENGKAAGIIADDRIHDADLVISNLG
HAATAVLCSEALSKEADAAYFKMVGTLQPSAGIKICLAADEPLVGHTGVLLTPYTRRINGV
NEVTQADPELAPPGKHLTMCHQYVAPENVKNLESEIEMGLEDLKEIFPGKRYEVLLIQSYH

DEWPVNRAASGTDPGNETPFSGLYVVGDGAKGKGGIEVEGVALGVMSVMEKVLG
00000000000000000000000000000000000000000000000000000000000000000000000000000000000
00000000000000000000000000000000000000000000000000000000000000000000000000000000000
00000000000000000000000000000000000000000000000000000000000000000000000000000000000
00000000000000000000000000000000000000000000000000000000000000000000000000000000000
00000000000000000000000000000000000000000000000000000000000000000000000000000000000
0000000111
>O54874_4aw2_1_423
SGEVRLRQLEQFILDGPAQTNGQCFSVETLLDILICLYDECNNSPLRREKNILEYLEWAKPFT
SKVKQMRLHREDFEILKVIGRGAFGEVAVVKLKNADKVFAMKILNKWEMLKRAETACFRE
ERDVLVNGDSKWITTLHYAFQDDNNLYLVMDYYVGGDLLTLLSKFEDRLPEEMARFYLAE
MVIAIDSVHQLHYVHRDIKPDNILMDMNGHIRLADFGSCLKLMEDGTVQSSVAVGTPDYIS
PEILQAMEDGKGRYGPECDWWSLGVCMYEMLYGETPFYAESLVETYGKIMNHKERFQFPT
QVTDVSENAKDLIRRLICSREHRLGQNGIEDFKKHPFFSGIDWDNIRNCEAPYIPEVSSPTDT
SNFDVDDDCLKNSETMPPPTHTAFSGHHLPFVGFTYTSSCVLSDRSCLRVTAGPTS
00000000000000000000000000000000000000000000000000000000000000000000000000000000000
00000000000000000000000000000000000000000000000000000000000000000000000000000000000
00000000000000000000000000000000000000000000000000000000000000000000000000000000000
00000000000000000000000000000000000000000000000000000000000000000000000000000000000
00000000000000000000000000000000000000000111111111111111111100000000000000000000000
01111111
>P38262_1r5m_112_534
SESNKAGEDGASTVERETQEDDTNSIDSSDDLDGFVKILKEIVKLDNIVSSTWNPLDESILAY
GEKNSVARLARIVETDQEGKKYWKLTIIAELRHPFALSASSGKTTNQVTCLAWSHDGNSIVT
GVENGELRLWNKTGALLNVLNFHRAPIVSVKWNKDGTHIISMDVENVTILWNVISGTVM
QHFELKETGGSSINAENHSGDGSLGVDVEWVDDDKFVIPGPKGAIFVYQITEKTPTGKLIG
HHGPISVLEFNDTNKLLLSASDDGTLRIWHGGNGNSQNCFYGHSQSIVSASWVGDDKVIS
CSMDGSVRLWSLKQNTLLALSIVDGVPIFAGRISQDGQKYAVAFMDGQVNVYDLKKLNSK
SRSLYGNRDGILNPLPIPLYASYQSSQDNDYIFDLSWNCAGNKISVAYSLQEGSVVAI
11111111111111111111111111111111100000000000000000000000000000000000000000000011111
10000000000000000011111100000000000000000000000000000000000000000000000000000000000
00000000000000000000000011111111111111000000000000000000000000000000000000000000000
00000000000000000000000000000000000000000000000000000000000000000000000000000000000
00000000000000000000000000000000111111111111100000000000000000000000000000000000000
00000000
>Q816V9_2i0z_0_422
MHYDVIVIGGGPSGLMAAIGAAEEGANVLLLDKGNKLGRKLAISGGGRCNVTNRLPLDEI
VKHIPGNGRFLYSAFSIFNNEDIITFFENLGVKLKEEDPGRMFPVSNKAQSVVDALLTRLKDL
GVKIRTNTPVETIEYENGQTKAVILQTGEVLETNHVVIAVGGKSVPQTGSTGDGYAWAEKA
GHTITELFPTEVPILSNEPFIRDRSLQGLALRDINLSVLNPKGKAIISHKMDMLFTHFGLSGPA
ALRCSQFVVKALKKFKTNTIQMSIDALPEENSEQLFQRMLKQMKEDPKKGIKNVLKGYVP
ERYFLFLLEKNEIDGSEQAGQVSHEKIRALVKDFKEFTVNVNGTQSIEKAFVTGGGVSVKEI
NPKEMSSKFTNGLYFCGEVLDIHGYTGGYNITSALVTGRIAGTTAGENAKMQY
00000000000000000000000000000000000000000000000000000000000000000000000000000000000
00000000000000000000000000000000000000000000000000000000000000000000000000000000000
00000000000000000000000000000000000000000000000000000000001111000000000000000000000
00000000000000000000000000000000000000000000000000000000000000000000000000000000000
00000000000000000000000000000000000000000000000000000000000000000000000000000000000
00000111
>Q8F0X1_3bws_33_455
SGDFSLLSSPINREKNGTEIVKFSIHPYKGTVIRLGEEILPFKVLEMDKNIALVEMAIPVYKDE

KEIELKLSSPGFQNSSYRIRKPEELNEKLIALDKEGITHRFISRFKTGFQPKSVRFIDNTRLAIPL
LEDEGMDVLDINSGQTVRLSPPEKYKKKLGFVETISIPEHNELWVSQMQANAVHVFDLKTL
AYKATVDLTGKWSKILLYDPIRDLVYCSNWISEDISVIDRKTKLEIRKTDKIGLPRGLLLSKDG
KELYIAQFSASNQESGGGRLGIYSMDKEKLIDTIGPPGNKRHIVSGNTENKIYVSDMCCSKIE
VYDLKEKKVQKSIPVFDKPNTIALSPDGKYLYVSCRGPNHPTEGYLKKGLVLGKVYVIDTTT
DTVKEFWEAGNQPTGLDVSPDNRYLVISDFLDHQIRVYRRDGF
11111111111111110000000000000000000000000000000000000000000000000000000000000000000
00000000000000000000000000000000000000000000000000000000000000000000000000000000000
00000000000000000000000000000000000000000000000000000000000000000000000000000000000
00000000000000000000000000000000000000000000000000000000000000000000000000000000000
00000000000000000000000000000000000000000000000000000000000000000000000000000000000
00000000
>O54828_2pbi_0_421
MTIRHQGQQYRPRMAFLQKIEALVKDMQNPETGVRMHNQRVLVTSVPHAMTGGDVLQ
WITQRLWISNLEAQNLGNFIVKYGYIYPLQDPKNLILKPDSSLYRFQTPYFWPTQQWPAEDT
DYAIYLAKRNIKKKGILEEYEKENYDFLNKKINYKWDFVIMQAKEQYRTGKERNKADRYA
LDCQEKAYWLVHRSPPGMNNVLDYGLDRVTNPNEVKKQTVTAVRKEIMYYQQALMRSTV
KSSVSLGGIVKYSEQFSSNDAIMSGCLPSNPWITDDTQFWDLNAKLVEIPTKMRVERWAFNF
SELIRDPKGRQSFQYFLKKEFSGENLGFWEACEDLKYGDQSKVKEKAEEIYKLFLAPGARR
WINIDGKTMDITVKGLRHPHRYVLDAAQTHIYMLMKKDSYARYLKSPIYKEMLAKAIEPQ
E
11111100000000000000000000000000000000000000000000000000000000000000000000000000000
00000000000000000000000000000000000000000000000000000000000000000000000000000000000
00000000000000000000000000000000000000000000000000000000000000000000000000000000000
00000000000000000000000000000000000000000000000000000000000000000000000000000000000
00000000000000000000000000000000000000000000000000000000000000000000000000000000000
0000001
>O66990_1xrf_0_421
MLKLIVKNGYVIDPSQNLEGEFDILVENGKIKKIDKNILVPEAEIIDAKGLIVCPGFIDIHVHL
RDPGQTYKEDIESGSRCAVAGGFTTIVCMPNTNPPIDNTTVVNYILQKSKSVGLCRVLPTGTI
TKGRKGKEIADFYSLKEAGCVAFTDDGSPVMDSSVMRKALELASQLGVPIMDHCEDDKLA
YGVINEGEVSALLGLSSRAPEAEEIQIARDGILAQRTGGHVHIQHVSTKLSLEIIEFFKEKGVK
ITCEVNPNHLLFTEREVLNSGANARVNPPLRKKEDRLALIEGVKRGIIDCFATDHAPHQTFE
KELVEFAMPGIIGLQTALPSALELYRKGIISLKKLIEMFTINPARIIGVDLGTLKLGSPADITIFD
PNKEWILNEETNLSKSRNTPLWGKVLKGKVIYTIKDGKMVYKD
00000000000000000000000000000000000000000000000000000000000000000000000000000000000
00000000000000000000000000000000000000000000000000000000000000000000000000000000000
00000000000000111111111111111111111111111100000000000000000000000000000000000000000
00000000000001111111111111111111111000000000000000000000000000111111111000000000000
00000000000000000000000000000000000000000000000000000000000000000000000000000000000
0000000
>P08192_1w78_0_421
MIIKRTPQAASPLASWLSYLENLHSKTIDLGLERVSLVAARLGVLKPAPFVFTVAGTNGKGT
TCRTLESILMAAGYKVGVYSSPHLVRYTERVRVQGQELPESAHTASFAEIESARGDISLTYFEY
GTLSALWLFKQAQLDVVILEVGLGGRLDATNIVDADVAVVTSIALDHTDWLGPDRESIGRE
KAGIFRSEKPAIVGEPEMPSTIADVAQEKGALLQRRGVEWNYSVTDHDWAFSDAHGTLEN
LPLPLVPQPNAATALAALRASGLEVSENAIRDGIASAILPGRFQIVSESPRVIFDVAHNPHAA
EYLTGRMKALPKNGRVLAVIGMLHDKDIAGTLAWLKSVVDDWYCAPLEGPRGATAEQLL
EHLGNGKSFDSVAQAWDAAMADAKAEDTVLVCGSFHTVAHVMEVIDARRSGGK
11111000000000000000000000000000000000000000000000000000000000000000000000000000000
00000000000000000000000000000000000000000000000000000000000000000000000000000000000

00000000000000000000000000000000000000000000000000000000000000000000000000000000000
00000000000000000000000000000000000000000000000000000000000000000000000000000000000
00000000000000000000000000000000000000000000000000000000000000000000000000000000000
0000111
>P16640_1q1r_0_421
MNANDNVVIVGTGLAGVEVAFGLRASGWEGNIRLVGDATVIPHHLPPLSKAYLAGKATAE
SLYLRTPDAYAAQNIQLLGGTQVTAINRDRQQVILSDGRALDYDRLVLATGGRPRPLPVASG
AVGKANNFRYLRTLEDAECIRRQLIADNRLVVIGGGYIGLEVAATAIKANMHVTLLDTAAR
VLERVTAPPVSAFYEHLHREAGVDIRTGTQVCGFEMSTDQQKVTAVLCEDGTRLPADLVIA
GIGLIPNCELASAAGLQVDNGIVINEHMQTSDPLIMAVGDCARFHSQLYDRWVRIESVPNA
LEQARKIAAILCGKVPRDEAAPWFWSDQYEIGLKMVGLSEGYDRIIVRGSLAQPDFSVFYLQ
GDRVLAVDTVNRPVEFNQSKQIITDRLPVEPNLLGDESVPLKEIIAAAKAELSSA
10000000000000000000000000000000000000000000000000000000000000000000000000000000000
00000000000000000000000000000000000000000000000000000000000000000000000000000000000
00000000000000000000000000000000000000000000000000000000000000000000000000000000000
00000000000000000000000000000000000000000000000000000000000000000000000000000000000
00000000000000000000000000000000000000000000000000000000000000000000000000000000000
0000000
>A2V759_2ek9_35_455
APAHADHQITKRTDAENMYNTIQFLSQAPRVAGSPEELKAVRYIEQQFKSYGYHVEVQPFQ
FEGYTAPSEVTLKIGTEKKEGEAFTYSPNSDVTAELVYVGLGTTADVAGKDLNGKIALIQRG
NISFADKVRNAAKQGAKAVIIYNNTDGKLNGTLGGSDASFVAAVGITKQEGDALAANLRA
GEKITATVKVAGAEVKTLTSHNVIATKKPDANKKNTNDIIIIGSHHDSVEKAPGANDDASG
VAVTLELARVMSKLKTDTELRFITFGAEENGLIGSKKYAASLSEDEIKRTIGMFQLDMVGSKD
AGDLIMYTIDGKKNRVTDLGAAASSRLSGVLPYGQEGRSDHESFHALGIPAALFIHAPVEP
WYHTPNDTLDKISKEKLDNVADIVGSAVYQAARPGELVIEPIDYPRRNVELQN
11110000000000000000000000000000000000000000000000000000000000000000000000000000000
00000000000000000000000000000000000000000000000000000000000000000000000000000000000
00000000000000000000000000000000000000000000000000000000000000000000000000000000000
00000000000000000000000000000000000000000000000000000000000000000000000000000000000
00000000000000000000000000000000000000000000000000000000000000000000000000000000000
011111
>P14385_2ih2_0_420
MGLPPLLSLPSNSAPRSLGRVETPPEVVDFMVSLAEAPRGGRVLEPACAHGPFLRAFREAH
GTAYRFVGVEIDPKALDLPPWAEGILADFLLWEPGEAFDLILGNPPYGIVGEASKYPIHVFK
AVKDLYKKAFSTWKGKYNLYGAFLEKAVRLLKPGGVLVFVVPATWLVLEDFALLREFLARE
GKTSVYYLGEVFPQKKVSAVVIRFQKSGKGLSLWDTQESESGFTPILWAEYPHWEGEIIRFET
EETRKLEISGMPLGDLFHIRFAARSPEFKKHPAVRKEPGPGLVPVLTGRNLKPGWVDYEKN
HSGLWMPKERAKELRDFYATPHLVVAHTKGTRVVAAWDERAYPWREEFHLLPKEGVRLDP
SSLVQWLNSEAMQKHVRTLYRDFVPHLTLRMLERLPVRREYGFHTSPESARNF
11111111111111111111000000000000000000000000000000000000000000000000000000000000000
00000000000000000000000000000000000000000000000000000000000000000000000000000000000
00000000000000000000000000000000000000000000000000000000000000000000000000000000000
00000000000000000000000000000000000000000000000000000000000000000000000000000000000
00000000000000000000000000000000000000000000000000000000000000000000000000000000011
111111
>P75791_2dbn_0_420
MASTFTSDTLPADHKAAIRQMKHALRAQLGDVQQIFNQLSDDIATRVAEINALKAQGDAV
WPVLSYADIKAGHVTAEQREQIKRRGCAVIKGHFPREQALGWDQSMLDYLDRNRFDEVY
KGPGDNFFGTLSASRPEIYPIYWSQAQMQARQSEEMANAQSFLNRLWTFESDGKQWFNPD
VSVIYPDRIRRRPPGTTSKGLGAHTDSGALERWLLPAYQRVFANVFNGNLAQYDPWHAAH

RTEVEEYTVDNTTKCSVFRTFQGWTALSDMLPGQGLLHVVPIPEAMAYVLLRPLLDDVPED
ELCGVAPGRVLPVSEQWHPLLIEALTSIPKLEAGDSVWWHCDVIHSVAPVENQQGWGNV
MYIPAAPMCEKNLAYAHKVKAALEKGASPGDFPREDYETNWEGRFTLADLNIHGKRALG
MDV
00000000000000000000000000000000000000000000000000000000000000000000000000000000000
00000000000000000000000000000000000011111111111111000000000000000000000000000000000
00000000000000000000000000000000000000000000000000000000000000000000000000000000000
11100000000000000000000000000000000000000000000000000000000000000000000000000000000
00000000000000000000000000000000000000000000000000000000000000000000000000000000000
000001
>Q84HI8_4amo_472_892
RPHPDHELVVCGAPDAAALTGLLTRVRAAATALSRPELTDLAAGLAAAHRGDVPARFAA
AVRDADGLVAALDRALGHLAEGGRRLLDAGRGLFLVVGGPLRVGLLFPGQAAPVHADRG
ALGHLLGDADAGTGSDPDSGVKPAEPVDTAVAQPAIIADSLAGIRWLDRLGARPVGALGH
SLGELAALSWAGALDADDTLALARARGEAMSAATEAPSGMLSLRADLAAARELAAGTGA
VVAVDNGERHVVVAGTRPELDRVAEAARHAGIEATPLAVSHAFHSPLMAPAAEALRRAA
GRLPWRRPERPVASTVTGAWWADEDPVEVLVRQLTGPVRFREALGLLDADLLVEVGPGRM
LSALAEAAGRTAVSLDAGAASAAGMAAGTAALFAAGAVDDATPFFAGRFTRPFDRDRERE
FLVNP
11100000000000000000000000000000000000000000000000000000000000000000000000000000000
00000000000000000000000000000011111111111111111110000000000000000000000000000000000
00000000000000000000000000000000000000000000000000000000000000000000000000000000000
00000000000000000000000000000000000000000000000000000000000000000000000000000000000
00000000000000000000000000000000000000000000000000000000000000000000000011111111111
111111
>Q55793_1t3i_0_419
MVALQIPSLAATVRQDFPILNQEINGHPLVYLDNAATSQKPRAVLEKLMHYYENDNANVH
RGAHQLSVRATDAYEAVRNKVAKFINARSPREIVYTRNATEAINLVAYSWGMNNLKAGDEI
ITTVMEHHSNLVPWQMVAAKTGAVLKFVQLDEQESFDLEHFKTLLSEKTKLVTVVHISNTL
GCVNPAEEIAQLAHQAGAKVLVDACQSAPHYPLDVQLIDCDWLVASGHKMCAPTGIGFL
YGKEEILEAMPPFFGGGEMIAEVFFDHFTTGELPHKFEAGTPAIAEAIALGAAVDYLTDLGM
ENIHNYEVELTHYLWQGLGQIPQLRLYGPNPKHGDRAALASFNVAGLHASDVATMVDQD
GIAIRSGHHCTQPLHRLFDASGSARASLYFYNTKEEIDLFLQSLQATIRFFSDDDFTV
11111100000000000000000000000000000000000000000000000000001100000000000000000000000
00000000000000000000000000000000000000000000000000000000000000000000000000000000000
00000000000000000000000000000000000000000000000000000000000000000000000000000000000
00000000000000000000000000000000000000000000000000000000000000000000000000000000000
00000000000000000000000000000000000000000000000000000000000000000000000000000000001
11111
>Q934G8_1ups_0_419
MFVFMLLLLLPFTISKAKDFPANPIEKAGYKLDFSDEFNGPTLDREKWTDYYLPHWCKDPE
SAKANYRFENGSLVEYITEDQKPWCPEHDGTVRSSAIMSFDKSWIHNFSGTTDNHERNEW
RGYTTKYGYFEIRAKLSNTGGGGHQAWWMVGMQDDTNDWFNSKQTGEIDILETFFSKKD
TWRIAAYGWNDPNFQTSWTISEDKVPSGDPTSEYHIYAMEWTPTALKFYYDNELFKVIYGS
PDYEMGTILNIYTDAGSGAHNDVWPKEWAIDYMRVWKPVDGYKESESLNNYLIRNRQTG
KFLYIEENNDKVSYGDITLKNEKNAKWSKEYRDGYTLLKNNETGEYLNIENQTGYIEHGKV
PKTWWSAQWSEVPVDGYTRFVNRWKPNMSIHTESYEGVLQYGNVPNTYWTSQWQLIPVE
11111111111111110000000000000000000000000000000000000000000000000000000000000000000
00000000000000000000000000000000000000000000000000000000000000000000000000000000000
00000000000000000000000000000000000000000000000000000000000000000000000000000000000
00000000000000000000000000000000000001100000000000000000000000000000000000000000000

00000000000000000000000000000000000000000000000000000000000000000000000000000000000
00000
>Q9C6D2_2pyw_0_419
MSFEEFTPLNEKSLVDYIKSTPALSSKIGADKSDDDLVIKEVGDGNLNFVFIVVGSSGSLVIKQ
ALPYIRCIGESWPMTKERAYFEATTLRKHGNLSPDHVPEVYHFDRTMALIGMRYLEPPHIIL
RKGLIAGIEYPFLADHMSDYMAKTLFFTSLLYHDTTEHRRAVTEFCGNVELCRLTEQVVFSD
PYRVSTFNRWTSPYLDDDAKAVREDSALKLEIAELKSMFCERAQALIHGDLHTGSVMVTQ
DSTQVIDPEFSFYGPMGFDIGAYLGNLILAFFAQDGHATQENDRKEYKQWILRTIEQTWNLF
NKRFIALWDQNKDGPGEAYLADIYNNTEVLKFVQENYMRNLLHDSLGFGAAKMIRRIVG
VAHVEDFESIEEDKRRAICERSALEFAKMLLKERRKFKSIGEVVSAIQQQS
10000000000000000000000000000000000000000000000000000000000000000000000000000000000
00000000000000000000000000000000000000000000000000000000000000000000000000000000000
00000000000000000000000000000000000000000000000000000000000000000000000000000000000
00000000000000000000000000000000000000000000000000000000000000000000000000000000000
00000000000000000000000000000000000000000000000000000000000000000000000000000000000
00001
>Q9H6P5_2a8j_0_419
MTMEKGMSSGEGLPSRSSQVSAGKITAKELETKQSYKEKRGGFVLVHAGAGYHSESKAKEY
KHVCKRACQKAIEKLQAGALATDAVTAALVELEDSPFTNAGMGSNLNLLGEIECDASIMD
GKSLNFGAVGALSGIKNPVSVANRLLCEGQKGKLSAGRIPPCFLVGEGAYRWAVDHGIPSC
PPNIMTTRFSLAAFKRNKRKLELAERVDTDFMQLKKRRQSSEKENDSGTLDTVGAVVVDH
EGNVAAAVSSGGLALKHPGRVGQAALYGCGCWAENTGAHNPYSTAVSTSGCGEHLVRTIL
ARECSHALQAEDAHQALLETMQNKFISSPFLASEDGVLGGVIVLRSCRCSAEPDSSQNKQT
LLVEFLWSHTTESMCVGYMSAQDGKAKTHISRLPPGAVAGQSVAIEGGVCRLESPVN
11111111111111111111111111111111111111110000000000000000000000000000000000000000000
00000000000000000000000000000000000000000000000000000000000000000000000001000000000
00000000000000000111111111111111111111111111111111111111111111111100000000000000000
00000000000000000000000000000000000000000000000000000000000000000000000000000000000
00000000000000000001111111111000000000000000000000000000000000000000000000000000000
01111
>Q9L6H1_2g5d_21_440
QSRSIQTFPQPDTSVINGPDRPAGIPDPAGTTVAGGGAVYTVVPHLSMPHWAAQDFAKSLQ
SFRLGCANLKNRQGWQDVCAQAFQTPVHSFQAKRFFERYFTPWQVAGNGSLAGTVTGYY
EPVLKGDGRRTERARFPIYGIPDDFISVPLPAGLRGGKNLVRIRQTGKNSGTIDNAGGTHTA
DLSRFPITARTTAIKGRFEGSRFLPYHTRNQINGGALDGKAPILGYAEDPVELFFMHIQGSGR
LKTPSGKYIRIGYADKNEHPYVSIGRYMADKGYLKLGQTSMQGIKAYMRQNPQRLAEVLG
QNPSYIFFRELAGSGNEGPVGALGTPLMGEYAGAIDRHYITLGAPLFVATAHPVTRKALNR
LIMAQDTGSAIKGAVRVDYFWGYGDEAGELAGKQKTTGYVWQLLPNGMKPEYRP
11111111111111111100000000000000000000000000000000000000000000000000000000000000000
00000000000000000000000000000000000000000000000000000000000000000000111111110000000
00000111111100000000000000000000000000000000000000000000000000000000000000000000000
00000000000000000000000000000000000000000000000000000000000000000001111100000000000
00000000000000000000000000000000000000000000000000000000000000000000000000000000000
00000
>Q9V3D4_1jnd_20_439
ASNLVCYYDSSSYTREGLGKLLNPDLEIALQFCSHLVYGYAGLRGENLQAYSMNENLDIYK
HQFSEVTSLKRKYPHLKVLLSVGGDHDIDPDHPNKYIDLLEGEKVRQIGFIRSAYDLVKTYG
FDGLDLAYQFPKNKPRKVHGDLGLAWKSIKKLFTGDFIVDPHAALHKEQFTALVRDVKDS
LRADGFLLSLTVLPNVNSTWYFDIPALNGLVDFVNLATFDFLTPARNPEEADYSAPIYHPDG
SKDRLAHLNADFQVEYWLSQGFPSNKINLGVATYGNAWKLTKDSGLEGVPVVPETSGPAP
EGFQSQKPGLLSYAEICGKLSNPQNQFLKGNESPLRRVSDPTKRFGGIAYRPVDGQITEGIW

VSYDDPDSASNKAAYARVKNLGGVALFDLSYDDFRGQCSGDKYPILRAIKYRL
10000000000000000000000000000000000000000000000000000000000000000000000000000000000
00000000000000000000000000000000000000000000000000000000001111111111111111111000000
00000000000000000000000000000000000000000000000000000000000000000000000000000000000
00000000000000000000000000000000000000000000000000000000000000000000000000000000000
00000000000000000000000000000000000000000000000000000000000000000000000000000000000
00000
>P21543_3voc_35_453
AVADDFQASVMGPLAKINDWGSFKKQLQTLKNNGVYAITTDVWWGYVESAGDNQFDWS
YYKTYANAVKEAGLKWVPIISTHKCGGNVGDDCNIPLPSWLSSKGSADEMQFKDESGYAN
SEALSPLWSGTGKQYDELYASFAENFAGYKSIIPKIYLSGGPSGELRYPSYYPAAGWSYPGRG
KFQAYTETAKNAFRTAMNDKYGSLDKINAAWGTKLTSLSQINPPTDGDGFYTNGGYNSAY
GKDFLSWYQSVLEKHLGVIGAAAHKNFDSVFGVRIGAKISGLHWQMNNPAMPHGTEQA
GGYYDYNRLIQKFKDADLDLTFTCLEMSDSGTAPNYSLPSTLVDTVSSIANAKGVRLNGEN
ALPTGGSGFQKIEEKITKFGYHGFTLLRINNLVNNDGSPTGELSGFKQYIISKAKPDNN
00000000000000000000000000000000000000000000000000000000000000000000000000000000000
00000000000000000000000000000000000000000000000000000000000000000000000000000000000
00000000000000000000000000000000000000000000000000000000000000000000000000000000000
00000000000000000000000000000000000000000000000000000000000000000000000000000000000
00000000000000000000000000000000000000000000000000000000000000000000000000000000000
0111
>P35505_1hyo_0_418
MSFIPVAEDSDFPIQNLPYGVFSTQSNPKPRIGVAIGDQILDLSVIKHLFTGPALSKHQHVFDE
TTLNNFMGLGQAAWKEARASLQNLLSASQARLRDDKELRQRAFTSQASATMHLPATIGD
YTDFYSSRQHATNVGIMFRGKENALLPNWLHLPVGYHGRASSIVVSGTPIRRPMGQMRPD
NSKPPVYGACRLLDMELEMAFFVGPGNRFGEPIPISKAHEHIFGMVLMNDWSARDIQQWE
YVPLGPFLGKSFGTTISPWVVPMDALMPFVVPNPKQDPKPLPYLCHSQPYTFDINLSVSLKG
EGMSQAATICRSNFKHMYWTMLQQLTHHSVNGCNLRPGDLLASGTISGSDPESFGSMLEL
SWKGTKAIDVEQGQTRTFLLDGDEVIITGHCQGDGYRVGFGQCAGKVLPALSPA
00000000000000000000000000000000000000000000000000000000000000000000000000000000000
00000000000000000000000000000000000000000000000000000000000000000000000000000000000
00000000000000000000000000000000000000000000000000000000000000000000000000000000000
00000000000000000000000000000000000000000000000000000000000000000000000000000000000
00000000000000000000000000000000000000000000000000000000000000000000000000000000000
0011
>Q81N71_4gb7_0_418
MKLKKSPLLLLIITFIFVITGLGFTYFKHNKTTPSKNNVTKKNWLDDPYLRWSYTHMKEFTLI
NDVKNNPDQIARFPSALQNLDDFAVQRRFGSATPLKELLDDNKTDAFVVVHNGQLVYERY
FNGYNESEPHGMASLAKVFTGAIIQSLAEENRIDLEKTADTYIKELKNTPFGKATLQQLMD
MQVSVEYPTHGYEHPALENQDAQLYLASNILPRDKNYDGPMKIYDMLQEAKETAPPGSVF
SYNNGSTETLAWIIRTITGKSLAENVSERIWSQIGMEENAYYVTDETKIEQASAGLNATARD
MARFGQLLLNNGEYNGKQILPSSITEDIKNVQEGELAIGPGASISYHNQWWIPHNEQGAFE
VLGSYGQTLYIDPKAKMVIVHFSSNATPSNEIHSVYSNMYIDIAHHLEKLPQ
11111111111111111111111111111110000000000000000000000000000000000000000000000000000
00000000000000000000000000000000000000000000000000000000000000000000000000000000000
00000000000000000000000000000000000000000000000000000000000000000000000000000000000
00000000000000000000000000000000000000000000000000000000000000000000000000000000000
00000000000000000000000000000000000000000000000000000000000000000000000000000000000
0000
>P01009_3ne4_0_417
MPSSVSWGILLLAGLCCLVPVSLAEDPQGDAAQKTDTSHHDQDHPTFNKITPNLAEFAFSL

YRQLAHQSNSTNIFFSPVSIATAFAMLSLGTKADTHDEILEGLNFNLTEIPEAQIHEGFQELL
RTLNQPDSQLQLTTGNGLFLSEGLKLVDKFLEDVKKLYHSEAFTVNFGDTEEAKKQINDYV
EKGTQGKIVDLVKELDRDTVFALVNYIFFKGKWERPFEVKDTEEEDFHVDQVTTVKVPMM
KRLGMFNIQHCKKLSSWVLLMKYLGNATAIFFLPDEGKLQHLENELTHDIITKFLENEDRRS
ASLHLPKLSITGTYDLKSVLGQLGITKVFSNGADLSGVTEEAPLKLSKAVHKAVLTIDEKGTE
AAGAMFLEAIPMSIPPEVKFNKPFVFLMIEQNTKSPLFMGKVVNPTQK
11111111111111111111111111111111111111111111111000000000000000000000000000000000000
00000000000000000000000000000000000000000000000000000000000000000000000000000000000
00000000000000000000000000000000000000000000000000000000000000000000000000000000000
00000000000000000000000000000000000000000000000000000000000000000000000000000000000
00000000000000000000000000000000000000000000000000000000000000000000000000000000000
001
>Q8G8Y2_2c81_0_417
MTIPFDHWPEWPQHSDRTRRKIEEVFQSNRWAISGYWTGEESMERKFAKAFADFNGVPYC
VPTTSGSTALMLALEALGIGEGDEVIVPSLTWIATATAVLNVNALPVFVDVEADTYCIDPQLI
KSAITDKTKAIIPVHLFGSMANMDEINEIAQEHNLFVIEDCAQSHGSVWNNQRAGTIGDIG
AFSCQQGKVLTAGEGGIIVTKNPRLFELIQQLRADSRVYCDDSSELMHGDMQLVKKGDIQG
SNYCLSEFQSAILLDQLQELDDKNAIREKNAMFLNDALSKIDGIKVMKRPPQVSRQTYYGY
VFRFDPVKFGGLNADQFCEILREKLNMGTFYLHPPYLPVHKNPLFCPWTKNRYLKSVRKTE
AYWRGLHYPVSERASGQSIVIHHAILLAEPSHLSLLVDAVAELARKFCVTH
11111110000000000000000000000000000000000000000000000000000000000000000000000000000
00000000000000000000000000000000000000000000000000000000000000000000000000000000000
00000000000000000000000000000000000000000000000000000000000000000000000000000000000
00000000000000000000000000000000000000000000000000000000000000000000000000000000000
00000000000000000000000000000000000000000000000000000000000000000000000000000000000
011
>Q9ZBU1_2vft_0_417
MSDITVTNWAGNITYTAKELLRPHSLDALRALVADSARVRVLGSGHSFNEIAEPGDGGVLL
SLAGLPSVVDVDTAARTVRVGGGVRYAELARVVHARGLALPNMASLPHISVAGSVATGTH
GSGVGNGSLASVVREVELVTADGSTVVIARGDERFGGAVTSLGALGVVTSLTLDLEPAYEME
QHVFTELPLAGLDPATFETVMAAAYSVSLFTDWRAPGFRQVWLKRRTDRPLDGFPYAAPA
AEKMHPVPGMPAVNCTEQFGVPGPWHERLPHFRAEFTPSSGAELQSEYLMPREHALAAL
HAMDAIRETLAPVLQTCEIRTVAADAQWLSPAYGRDTVAAHFTWVEDTAAVLPVVRRLEE
ALVPFAARPHWGKVFTVPAGELRALYPRLADFGALAGALDPAGKFTNAFVRGVLAG
11000000000000000000000000000000000000000000000000000000000000000000000000000000000
00000000000000000000000000000000000000000000000000000000000000000000000000000000000
00000000000000000000000000000000000000000000000000000000000000000000000000000000000
00000000000000000000000000000000000000000000000000000000000000000000000000000000000
00000000000000000000000000000000000000000000000000000000000000000000000000000000000
001
>P53196_3vl1_0_416
MTKTITVAHIQYDFKAVLEENDENDDEFYINVDKNLNEIKEHKIVVLGNSRGVDAGKGNT
FEKVGSHLYKARLDGHDFLFNTIIRDGSKMLKRADYTAVDTAKLQMRRFILGTTEGDIKVL
DSNFNLQREIDQAHVSEITKLKFFPSGEALISSSQDMQLKIWSVKDGSNPRTLIGHRATVTDI
AIIDRGRNVLSASLDGTIRLWECGTGTTIHTFNRKENPHDGVNSIALFVGTDRQLHEISTSKK
NNLEFGTYGKYVIAGHVSGVITVHNVFSKEQTIQLPSKFTCSCNSLTVDGNNANYIYAGYE
NGMLAQWDLRSPECPVGEFLINEGTPINNVYFAAGALFVSSGFDTSIKLDIISDPESERPAIEF
ETPTFLVSNDDEVSQFCYVSDDESNGEVLEVGKNNFCALYNLSNP
00000000000000000000000000000000000000000000000000000000000000000000000000000000000
00000000000000000000000000000000000000000000000000000000000000000000000000000000000
00000000000000000000000000000000000000000000000000000000000000000000000000000000000

00000000000000000000000000000000000000000000000000000000000000000000000000000000000
00000000000000000000000000000000000000000000000000000000000000000000000000000000000
01
>Q23229_1mg7_0_416
MQVEANSERRVKILGIDRSENSPVLTYMETEDDPNFRNSKLAAAPHTVHMMDSGFLAINR
QCLVKGKAILAREPKSSNEHMIDDLPKHAHDQHTLSILRDFIDQLKLHNVYEINFYDPLDSS
GKLAVIPMLIALWKCMLASETDICDQEVLKSIMNSVIAKFELQIPCKNAVIDATLSGSREEVH
IIAEDGSLENSNGTTEHFNKKHDLVFVKTDLHPEDFTPQMFPSQAKAKLLRDAFNNEEDED
TFPDILVPAYMTAHSKNRVRQEDYTCLEVEFDSQVALEKLMNEHEQVEGFEVQQGGILVAL
KKDSFFDDELIEKIAIAIATESRQSVSSVSFDLLKLGPGASLVTLANSRRFEPECRVVLQIEVKP
VSPGETSSEGISDEHHYEEYDEDDIMEEEEAPSARQDDTYDEDEE
11111100000000000000000000001111111111000000000000000000000000000000000000000000000
00000000000000000000000000000000000000000000000000000000000000000000000000000000000
00000000000000000000000111110000000000000000000000000000000000000000000000000000000
00000000000000000000000000000000000000000000000000000000000000000000000000000000000
00000000000000000000000000000000000000000011111111111111111111111111111111111111111
11
>Q6WNP1_4doy_0_416
MTLSPEKQHVRPRDAADNDPVAVARGLAEKWRATAVERDRAGGSATAEREDLRASGLLSL
LVPREYGGWGADWPTAIEVVREIAAADGSLGHLFGYHLTNAPMIELIGSQEQEEHLYTQIA
QNNWWTGNASSENNSHVLDWKVSATPTEDGGYVLNGTKHFCSGAKGSDLLFVFGVVQD
DSPQQGAIIAAAIPTSRAGVTPNDDWAAIGMRQTDSGSTDFHNVKVEPDEVLGAPNAFVL
AFIQSERGSLFAPIAQLIFANVYLGIAHGALDAAREYTRTQARPWTPAGIQQATEDPYTIRSY
GEFTIALQGADAAAREAAHLLQTVWDKGDALTPEDRGELMVKVSGVKALATNAALNISS
GVFEVIGARGTHPRYGFDRFWRNVRTHSLHDPVSYKIADVGKHTLNGQYPIPGFTS
11111111111111111000000000000000000000000000000000000000000000000000000000000000000
00000000000000000000000000000000000000000000000000000000000000000000000000000000000
00000000000000000000000000000000000000000000000000000000000000000000000000000000000
00000000000000000000000000000000000000000000000000000000000000000000000000000000000
00000000000000000000000000000000000000000000000000000000000000000000000000000000000
00
>Q9RW45_2imr_1_417
RFSAVSRHHRGASIDPMTFSEATTPDALTPDAHTPRLLTCDVLYTGMGGAQSPGGVVVVGE
TVAAAGHPDELRRQYPHAAEERAGAVIAPPPVNAHTHLDMSAYEFQALPYFQWIPEVVIR
GRHLRGVAAAQAGADTLTRLGAGGVGDIVWAPEVMDALLAREDLSGTLYFEVLNPFPDK
ADEVFAAARTHLERWRRLERPGLRLGLSPHTPFTVSHRLMRLLSDYAAGEGLPLQIHVAEH
PTELEMFRTGGGPLWDNRMPALYPHTLAEVIGREPGPDLTPVRYLDELGVLAARPTLVHM
VNVTPDDIARVARAGCAVVTCPRSNHHLECGTFDWPAFAAAGVEVALGTDSVASGETLNV
REEVTFARQLYPGLDPRVLVRAAVKGGQRVVGGRTPFLRRGETWQEGFRWELSRDL
11111111111111111111111111111111000000000000011100000000000000000000000000000000000
00000000000000000000000000000000000000000000000000000000000000000000000000000000000
00000000000000000000000000000000000000000000000000000000000000000000000000000000000
00000000000000000000000000000000000000000000000000000000000000000000000000000000000
00000000000000000000000000000000000000000000000000000000000001100000000000000000000
00
>P83788_1qz9_0_415
MTTRNDCLALDAQDSLAPLRQQFALPEGVIYLDGNSLGARPVAALARAQAVIAEEWGNG
LIRSWNSAGWRDLSERLGNRLATLIGARDGEVVVTDTTSINLFKVLSAALRVQATRSPERRV
IVTETSNFPTDLYIAEGLADMLQQGYTLRLVDSPEELPQAIDQDTAVVMLTHVNYKTGYMH
DMQALTALSHECGALAIWDLAHSAGAVPVDLHQAGADYAIGCTYKYLNGGPGSQAFVW
VSPQLCDLVPQPLSGWFGHSRQFAMEPRYEPSNGIARYLCGTQPITSLAMVECGLDVFAQT

DMASLRRKSLALTDLFIELVEQRCAAHELTLVTPREHAKRGSHVSFEHPEGYAVIQALIDRG
VIGDYREPRIMRFGFTPLYTTFTEVWDAVQILGEILDRKTWAQAQFQVRHSVT
10000000000000000000000000000000000000000000000000000000000000000000000000000000000
00000000000000000000000000000000000000000000000000000000000000000000000000000000000
00000000000000000000000000000000000000000000000000000000000000000000000000000000000
00000000000000000000000000000000000000000000000000000000000000000000000000000000000
00000000000000000000000000000000000000000000000000000000000000000000000001111111111
1
>Q8ZL86_3g7q_0_415
MTFSLFGDKFTRHSGITRLMEDLNDGLRTPGAIMLGGGNPAHIPAMQDYFQTLLTDMVES
GKAADALCNYDGPQGKTALLNALAVLLRETLGWDIEPQNIALTNGSQSAFFYLFNLFAGR
RADGSTKKVLFPLAPEYIGYADSGLEDDLFVSARPNIELLPEGQFKYHVDFEHLHIGEETGM
ICVSRPTNPTGNVITDEELMKLDRLANQHNIPLVIDNAYGVPFPGIIFSEARPLWNPNIILCM
SLSKLGLPGSRCGIIIANDKTITAIANMNGIISLAPGGMGPAMMCEMIKRNDLLRLSETVIKP
FYYQRVQQTIAIIRRYLSEERCLIHKPEGAIFLWLWFKDLPITTELLYQRLKARGVLMVPGHY
FFPGLDKPWPHTHQCMRMNYVPEPDKIEAGVKILAEEIERAWREG
11111111111111000000000001111100000000000000000000000000000000000000000000000000000
00000000000000000000000000000000000011110000000000000000001111100000000000000000000
00000000000000000000000000000000000000000000000000000000000000000000000000000000000
00000000000000000000000000000000000000000000000000000000000000000000000000000000000
00000000000000000000000000000000000000000000000000000000000000000000000000000000000
0
>O24782_3awq_0_414
MPKTPHTKGPDETLSLLADPYRFISRQCQRLGANAFESRFLLKKTNCLKGAKAAEIFYDTTR
FEREGAMPVAIQKTLLGQGGVQGLDGETHRHRKQMFMGLMTPERVRALAQLFEAEWRR
AVPGWTRKGEIVFYDELHEPLTRAVCAWAGVPLPDDEAGNRAGELRALFDAAGSASPRHL
WSRLARRRVDAWAKRIIEGIRAGSIGSGSGTAAYAIAWHRDRHDDLLSPHVAAVELVNVLR
PTVAIAVYITFVAHALQTCSGIRAALVQQPDYAELFVQEVRRFYPFFPAVVARASQDFEWEG
MAFPEGRQVVLDLYGSNHDAATWADPQEFRPERFRAWDEDSFNFIPQGGGDHYLGHRCP
GEWIVLAIMKVAAHLLVNAMRYDVPDQDLSIDFARLPALPKSGFVMRNVHIGG
11111111000000000000000000000000000000000000000000000000000000000000000000000000000
00000000000000000000000000000000000000000000000000000000000000000000000000000000000
00000000000000000000000000000000000000000000000000000000000000000000000000000000000
00000000000000000000000000000000000000000000000000000000000000000000000000000000000
00000000000000000000000000000000000000000000000000000000000000000000000000000000000
>P0AG80_4aq4_23_437
VTTIPFWHSMEGELGKEVDSLAQRFNAENPDYKIVPTYKGNYEQNLSAGIAAFRTGNAPAI
LQVYEVGTATMMASKAIKPVYDVFKEAGIQFDESQFVPTVSGYYSDSKTGHLLSQPFNSSTP
VLYYNKDAFKKAGLDPEQPPKTWQDLADYAAKLKASGMKCGYASGWQGWIQLENFSAW
NGLPFASKNNGFDGTDAVLEFNKPEQVKHIAMLEEMNKKGDFSYVGRKDESTEKFYNGD
CAMTTASSGSLANIREYAKFNYGVGMMPYDADAKDAPQNAIIGGASLWVMQGKDKETYT
GVAKFLDFLAKPENAAEWHQKTGYLPITKAAYDLTREQGFYEKNPGADTATRQMLNKPPL
PFTKGLRLGNMPQIRVIVDEELESVWTGKKTPQQALDTAVERGNQLLRRFEKSTKS
00000000000000000000000000000000000000000000000000000000000000000000000000000000000
00000000000000000000000000000000000000000000000000000000000000000000000000000000000
00000000000000000000000000000000000000000000000000000000000000000000000000000000000
00000000000000000000000000000000000000000000000000000000000000000000000000000000000
00000000000000000000000000000000000000000000000000000000000000000000000000000000001
>P29723_1o75_19_433
CGSSHHETHYGYATLSYADYWAGELGQSRDVLLAGNAEADRAGDLDAGMFDAVSRATH
GHGAFRQQFQYAVEVLGEKVLSKQETEDSRGRKKWEYETDPSVTKMVRASASFQDLGEDG

EIKFEAVEGAVALADRASSFMVDSEEYKITNVKVHGMKFVPVAVPHELKGIAKEKFHFVED
SRVTENTNGLKTMLTEDSFSARKVSSMESPHDLVVDTVGTGYHSRFGSDAEASVMLKRAD
GSELSHREFIDYVMNFNTVRYDYYGDDASYTNLMASYGTKHSADSWWKTGRVPRISCGIN
YGFDRFKGSGPGYYRLTLIANGYRDVVADVRFLPKYEGNIDIGLKGKVLTIGGADAETLMD
AAVDVFADGQPKLVSDQAVSLGQNVLSADFTPGTEYTVEVRFKEFGSVRAKVVAQ
11111100000000000000000000000000111110000000000000000000000000000000000000000000
00000000000000000000000000000000000000000000000000000000000000000000000000000000
00000000000000000000000000000000000000000000000000000000000000000000000000000000
00000000000000000000000000000000000000000000000000000000000000000000000000000000
00000000000000000000000000000000000000000000000000000000000000000000000000000001
>Q8NVU1_1zjc_0_414
MTNYKEKLQQYAELLVKVGMNVQPKQPVFIRSSVETLELTHLIVEEAYHCGASDVRVVYSD
PTLKRLKFENESVEHFANHEIKSYDVEARMDYVKRGAANLALISEDPDLMDGIDSQKLQAF
QQQNARAFKGYMESVQKNQFPWVVAAFPSKAWAKRVYPELSVEEAYIKFIDEVFDIVRIDG
NDPVENWRQHIANLSVYAQKLQQKNYHALHYVSEGTDLTVGLAKNHIWEDATSYVNGK
EQAFIANIPTEEVFTAPDRNRVDGYVTNKLPLSYNGTIIDQFKLMFKDGEIIDFSAEKGEAVL
KDLINTDEGSRRLGEVALVPDDSPISNRNTIFYNTLFDENAACHLAIGSAYAFNIQGGTEMT
VEEKIASGLNDSNVHVDFMIGSSDLTIYGIFEDGSKELVFENGNWASTF
11000000000000000000000000000000000000000000000000000000000000000000000000000000
00000000000000000000000000000000000000000000000000000000000000000000000000000000
00000000000000000000000000000000000000000000000000000000000000000000000000000000
00000000000000000000000000000000000000000000000000000000000000000000000000000000
00000000000000000000000000000000000000000000000000000000000000000000000000000000
>A1A7Y6_4e9l_594_1007
VADGQQAYTLTLTAVDSEGNPVTGEASRLRLVPQDTNGVTVGAISEIKPGVYSATVSSTRAG
NVVVRAFSEQYQLGTLQQTLKFVAGPLDAAHSSITLNPDKPVVGGTVTAIWTAKDANDNP
VTGLNPDAPSLSGAAAAGSTASGWTDNGDGTWTAQISLGTTAGELDVMPKLNGQDAAA
NAAKVTVVADALSSNQSKVSVAEDHVKAGESTTVTLVAKDAHGNAISGLSLSASLTGTASE
GATVSSWTEKGDGSYVATLTTGGKTGELRVMPLFNGQPAATEAAQLTVIAGEMSSANSTLV
ADNKTPTVKTTTELTFTMKDAYGNPVTGLKPDAPVFSGAASTGSERPSAGNWTEKGNGVY
VSTLTLGSAAGQLSVMPRVNGQNAVAQPLVLNVAGDASKAEIRDMTVKVNNQ
11111111111111111111111111111111111111111111111111111111111111111111111111111111
00000000000000000000000000000000000000000000000000000000000000000000000000000000
00000000000000000000000000000000000000000000000000000000000000000000000000000000
00000000000000000000000000000000000000000000000000000000000000000000000000000000
0000000000000000000000000000000000000000000000000000000000000011111111111111111
>B5L6K6_3ndj_0_413
MSHLADVSPPTACRVCGGGVQEFLDLGRQPLSDRFRKPDELDDEFTYRLAVGRCDSCEMV
QLTEEVPRDLMFHEVYPYHSSGSSVMREHFAMLARDFLATELTGPDPFIVEIGCNDGIMLRT
IQEAGVRHLGFEPSSGVAAKAREKGIRVRTDFFEKATADDVRRTEGPANVIYAANTLCHIPY
VQSVLEGVDALLAPDGVFVFEDPYLGDIVAKTSFDQIYDEHFFLFSATSVQGMAQRCGFELV
DVQRLPVHGGEVRYTLARQGSRTPSAAVAQLLAAEREQELSDMATLRAFAGNVVKIRDEL
TALLHRLRAEGRSVVGYGATAKSATVTNFCGIGPDLVHSVYDTTPDKQNRLTPGAHIPVRP
ASAFSDPYPDYALLFAWNHAEEIMAKEQEFHQAGGRWILYVPEVHIR
11111111110000000000000000000000000000000000000000000000000000000000000000000000
00000000000000000000000000000000000000000000000000000000000000000000000000000000
00000000000000000000000000000000000000000000000000000000000000000000000000000000
00000000000000000000000000000000000000000000000000000000000000000000000000000000
0000000000000000000000000000000000000000000000000000000000000000000000000000000
>Q21PD3_2yhg_519_932
GEGGRYRVIHTTDMGADPDDEQSLVRQLVMANEYDLEGIITTTGCWKKSTSNTAYVDRILN

AYSQAYPNLSKHAEGFPTPAYLDSINVMGQRGYGMGDVGSGKDSAGSNLIIAAVDKDDPR
PVWATCWGGCNTIAQAVWKVQNTRSQAQLDAFISKLRVYDILGQDNAGTWLAKNFPNLI
YIRARSVYSWQPSDSYLDNHIQSHGALGAVYPNRRYATEGDTPAFLHMANPGLNDPSVVS
MGGWGGRFPSKQAGVRGMSCMSGEDAVYDTYYMYTENGESIKRWSTAIHNDFQARMD
WAIESNYSAANHHPVPVVNNDANEAVMYLNASAGSTVSLDASGSSDPDGDSLNYSWSHY
GEADSYSGSVSISNSSSASANVQIPSNAGGKDIHILLTLRDNGSPNLYAYRRVVINVQ
11100000000000000000000000000000000000000000000000000000000000000000000000000000000
00000000000000000000000000000000000000000000000000000000000000000000000000000000000
00000000000000000000000000000000000000000000000000000000000000000000000000000000000
00000000000000000000000000000000000000000000000000000000000000000000000000000000000
0000000000000000000000000000000000000000000000000000000000000000000000000000000000
>Q86QZ1_2q9u_0_413
MSSKPKYVQDQEMIPGVYWVGIVDWMVRIFHGYHTDEGSSYNSYFIDDECPTVIDSVKYPF
AEEWLSRIAACCPLDKIKYVVMNHAEGDHASSLKDHYHKFTNATFVCTKKCQEHLKILYG
MEKATWLIVDDKYTLKIGKRTLKFIPVPLLHWPDSTFTYCPEDKILFSNDGFGQHYATSRRW
ADECDVSHVMHLFKEYTANILGLFSAQMRKALEVASTVEIKYILSAHGVSWRGDAMGLAI
AEYDRWSKGQHCQKKVTVVLDSMYGTTHRMALALLDGARSTGCETVLLEMTSSDITKVA
LHTYDSGAVAFASPTLNNTMMPSVAAALNYVRGLTLIKGKPAFAFGAFGWSNRAVPDIVAE
LRDGCKADVYDEKGITFKFNYTEELLEQAYNAGVDLGKRAIAYCEKNAPKQ
11100000000000000000000000000000000000000000000000000000000000000000000000000000000
00000000000000000000000000000000000000000000000000000000000000000000000000000000000
00000000000000000000000000000000000000000000000000000000000000000000000000000000000
00000000000000000000000000000000000000000000000000000000000000000000000000000000000
0000000000000000000000000000000000000000000000000000000000000000000000000000000011
>A7M066_4irt_18_430
CSQHLVEVGKGYSCTSVNTTVFRNNSLVTHGDEQYISYYDNDGYLVLGKRKLDSEQWTLH
RTQYQGNVKDAHNIISMMIDGEGYIHVSFDHHGHPLNYCRSIAPGSLELGDKMPMTGVDE
GNVTYPEFYPLSGGDLLFVYRSGSSGRGNLVMNRYSLKEHKWTRVQDILIDGENKRNAYW
QLYVDEKGTIHLSWVWRETWHVETNHDICYARSFDNGVTWYKTSGERYELPIKLSNAEYA
CRLPQNCELINQTSMSADAGGNPYIATYWREPNSDVPQYRIVWNDGKMWHQRQITDRQT
PFTLKGGGTKMIPIARPRIVVEGGEVFYIFRDEERGSRVSMAHATDVGTSKWTITDLTDFSVD
AWEPSHDTELWKKQRKLHLFVQHTRQGDGERTAEIEPQMIYVLETNMDTNK
11000000000000000000000000000000000000000000000000000000000000000000000000000000000
00000000000000000000000000000000000000000000000000000000000000000000000000000000000
00000000000000000000000000000000000000000000000000000000000000000000000000000000000
00000000000000000000000000000000000000000000000000000000111100000000000000000000000
000000000000000000000000000000000000000000000000000000001111100000000000000001111
>C7MW73_4jbe_0_412
MTNEVAKAVEECARAAKSAAPSLSGAPDTAIDAALESMADRLLAHRDAVLAANAEDIAK
AEAGGMSAGLLDRLTITESRLTDMADQLRLLAGAPHPQRTVELSTLDGGLRLVERRRPVGV
IGANYEARPNVTVDVASQLVKSRNAGVLRTGSAALKSAQRLLEVVIRPALTDSGIDANVVQ
LVPRPEREAAGALVRLPDLVPLVILRGSGESTRALALEAAQHGVRTLAHADGGGVLYVDEK
ADRDTVRSLVVNSLDRLGVCNRLNLLLIHDAVYEEFWPVVSEALAERGVSPSLPPYDHPIGY
EWALDSEREATVTVARVDGVAEAVRIANEETSGLAAGIATEDARAAEEFFDGYQGTGVFW
NAPTRLLDGFKLLGVPETGINLDKVPGPRGPVTYPDLYVRQYAVLPVER
10000000000000000000000000000000000000000000000000000000000000000000000000000000000
00000000000000000000000000000000000000000000000000000000000000000000000000000000000
00000000000000000000000000000000000000000000000000000000000000000000000000000000000
00000000000000000000000000000000000000000000000000000000000000000000000000000000000
0000000000000000000000000000000000000000000000000000000000000000000000000000000000
>P64012_2c82_0_412

MTNSTDGRADGRLRVVVLGSTGSIGTQALQVIADNPDRFEVVGLAAGGAHLDTLLRQRA
QTGVTNIAVADEHAAQRVGDIPYHGSDAATRLVEQTEADVVLNALVGALGLRPTLAALKT
GARLALANKESLVAGGSLVLRAARPGQIVPVDSEHSALAQCLRGGTPDEVAKLVLTASGGP
FRGWSAADLEHVTPEQAGAHPTWSMGPMNTLNSASLVNKGLEVIETHLLFGIPYDRIDVV
VHPQSIIHSMVTFIDGSTIAQASPPDMKLPISLALGWPRRVSGAAAACDFHTASSWEFEPLD
TDVFPAVELARQAGVAGGCMTAVYNAANEEAAAAFLAGRIGFPAIVGIIADVLHAADQW
AVEPATVDDVLDAQRWARERAQRAVSGMASVAIASTAKPGAAGRHASTLERS
11111111110000000000000000000000000000000000000000000000000000000000000000000000000
00000000000000000000000000000000000000000000000000000000000000000000000000000000000
00000000000000000000000000000000000000000000000000000000000000000000000000000000000
00000000000000000000000000000000000000000000000000000000000000000000000000000000000
000000000000000000000000000000000000000000000000000000000111111111111111111111111
>Q5L1E2_2oel_0_412
MSAVMATYLLHDETDIRKKAEGIALGLTIGTWTDLPALEQEQLRKHKGEVVAIEELGESERV
NAYFGKRLKRAIVKIAYPTVNFSADLPALLVTTFGKLSLDGEVRLLDLEFPDEWKRQFPGPR
FGIDGIRDRVGVHNRPLLMSIFKGMIGRDLAYLTSELKKQALGGVDLVKDDEILFDSELLPFE
KRITEGKAALQEVYEQTGKRTLYAVNLTGKTFALKDKAKRAAELGADVLLFNVFAYGLDVL
QALREDEEIAVPIMAHPAFSGAVTPSEFYGVAPSLWLGKLLRLAGADFVLFPSPYGSVALERE
QALGIARALTDDQEPFARAFPVPSAGIHPGLVPLIIRDFGLDTIVNAGGGIHGHPDGAIGGG
RAFRAAIDAVLAGRPLRAAAAENEALQKAIDRWGVVEVEA
10000000000000000000000000000000000000000000000000000000000000000000000000000000000
00000000000000000000000000000000000000000000000000000000000000000000000000000000000
00000000000000000000000000000000000000000000000000000000000000000000000000000000000
00000000000000000000000000000000000000000000000000000000000000000000000000000000000
000000000000000000000000000000000000000000000000000000000000000000000000000000000
>A0QZY0_3c8z_0_411
MQSWSAPAIPVVPGRGPALRLFDSADRQVRPVTPGPTATMYVCGITPYDATHLGHAATYLT
FDLVHRLWLDAGHTVQYVQNVTDVDDPLFERAERDGIDWRTLGDRETQLFREDMAALRV
LPPHDYVAATDAIAEVVEMVEKLLASGAAYIVEDAEYPDVYFRADATAQFGYESGYDRDT
MLTLFAERGGDPDRPGKSDQLDALLWRAERPGEPSWPSPFGRGRPGWHVECSAIALTRIGT
GLDIQGGGSDLIFPHHEYSAAHAESVTGERRFARHYVHTGMIGWDGHKMSKSRGNLVLVS
QLRAQGVDPSAIRLGLFSGHYREDRFWSNEVLDEANARLARWRSATALPEAPDATDVIAR
VRQYLADDLDTPKALAALDGWCTDALSYGGHDTESPRLVATTVDALLGVDL
00000000000000000000000000000000000000000000000000000000000000000000000000000000000
00000000000000000000000000000000000000000000000000000000000000000000000000000000000
00000000000000000000000000000000000000000000000000000000000000000000000000000000000
00000000000000000000000000000000000111111111111100000000000000000000000000000000000
00000000000000000000000000000000000000000000000000000000000000000000000000000000
>A8W790_2z8d_27_437
DTAGDTKTTDDGGVVNITYMHRLPDSEGMTLVNDIVAKWNKQHPDIQVKATKFDGKASD
MIKKLETDVKSGEAPDLAQVGYAELPEVFTKGLLQDVTQYAEQYKNDFASGPYSLVQVGG
KAYGLPQDTGPLVYFYNKAEFEKLGITEIPQTADEFIAAAKTAAAAGKYIMSYQPDEAGNM
ISGLAGASGGWYKVKGDSWVVNTETDGSKATADFYQQLLDAKAATTNPRWDPSFDASIK
DGSLIGTVAAAWEAPLFMTSSGGTGSGEWQVAQLGDWFGNAGKTGPDGGSAVAVLKNSK
HPKEAMEFLDWFNTQVPDLVSQGLVPAATTEDAETPSEWSTFFGGQDIMKEFKTANNNM
GDFTYMPGFSAVAAKMNETAAKATDGSGKVADIFSDAQTTSVDTLKNFGLSVSE
11111111000000000000000000000000000000000000000000000000000000000000000000000000000
00000000000000000000000000000000000000000000000000000000000000000000000000000000000
00000000000000000000000000000000000000000000000000000000000000000000000000000000000
00000000000000000000000000000000000000000000000000000000000000000000000000000000000
0000000000000000000000000000000000000000000000000000000000000000000000000000001

>B1MFF5_4hr3_0_410
MDFAPSARAAELIAAVREFIDAEVMPVERAVLAHHDELLGARAGTTAELWHVPPELDSLK
AKARAAGLWNLFLPDPELGGGLSNSEYAPLAEQMGRSLFAPTVFNCNAPDSGNMEVLHR
YGSQEQKEVWLEPLLEGDIRSAFCMTEPDVASSDATNMAATAVVEGDEVVINGRKWWSTG
VGHPDCKVIIFMGLTDPNAHRYARHSMVLVPMDTPGITVERMLPTMGFYDEPGGHGVVSF
DNVRLPADAFIAGPGKGFEIAQGRLGPGRVHHAMRLIGLAEVALEHACRRGLDRTAFGKP
LVNLGGNRERIADARIAINQTRLLVLHAAWLLDTVGIMGALSAVSEIKVAAPNMAQQVID
MAIQIHGGGGLSNDFPLAAAWVNARALRLADGPDEVHRGVVARIELAKYAND
11000000000000000000000000000000000000000000000000000000000000000000000000000000000
00000000000000000000000000000000000000000000000000000000000000000000000000000000000
00000000000000000000000000000000000000000000000000000000000000000000000000000000000
00000000000000000000000000000000000000000000000000000000000000000000000000000000000
0000000000000000000000000000000000000000000000000000000000000000000000000000011
>Q7N4T1_4hb9_0_410
MHVGIIGAGIGGTCLAHGLRKHGIKVTIYERNSAASSILPGYGIHINSFGKQALQECLPAEN
WLAFEEASRYIGGQSRFYNERMRLLAVHGGISPMAGKIISEQRLSISRTELKEILNKGLANTI
QWNKTFVRYEHIENGGIKIFFADGSHENVDVLVGADGSNSKVRKQYLPFIERFDVGVSMIIG
RARLTPALTALLPQNFRDGTPNSIVPKSPDWLFISMWRAPVNIHVEASLAEIDNFIVWVYVA
ATDSLPDNITDFSAEALCDLVQSRMISWDPSLHTLVQQSDMENISPLHLRSMPHLLPWKSST
VTLLGDAIHNMTPMTGSGANTALRDALLLTQKLASVASGHEELVKAISDYEQQMRAYANE
IVGISLRSAQNAVIHFSIPPLKQRHLSIRRNKSQSHQHRR
00000000000000000000000000000000000000000000000000000000000000000000000000000000000
00000011111111111111000000000000000000000000000000000000000000000000000000000000000
00000000000000000000000000000000000000000000000000000000000000000000000000000000000
00000000000000000000000000000000000000000000000000000000000000000000000000000000000
0000000000000000000000000000000000000000000000000001111111111111111111111
>P23793_1s9r_0_409
MSVFDSKFKGIHVYSEIGELESVLVHEPGREIDYITPARLDELLFSAILESHDARKEHKQFVAE
LKANDINVVELIDLVAETYDLASQEAKDKLIEEFLEDSEPVLSEEHKVVVVRNFLKAKKTSREL
VEIMMAGITKYDLGIEADHELIVDPMPNLYFTRDPFASVGNGVTIHYMRYKVRQRETLFSRF
VFSNHPKLINTPWYYDPSLKLSIEGGDVFIYNNDTLVVGVSERTDLQTVTLLAKNIVANKEC
EFKRIVAINVPKWTNLMHLDTWLTMLDKDKFLYSPIANDVFKFWDYDLVNGGAEPQPVE
NGLPLEGLLQSIINKKPVLIPIAGEGASQMEIERETHFDGTNYLAIRPGVVIGYSRNEKTNAA
LEAAGIKVLPFHGNQLSLGMGNARCMSMPLSRKDVKW
10000000000000000000000000000000000000000000000000000000000000000000000000000000000
00000000000000000000000000000000000000000000000000000000000000000000000000000000000
00000000000000000000000000000000000000000000000000000000000000000000000000000000000
00000000000000000000000000000000000000000000000000000000000000000000000000000000000
000000000000000000000000000000000000000000000000000000000000000000000000000000
>Q3SAG3_2inv_40_449
ADGQQGAPLNSPNTYDVTTWRIKAHPEVTAQSDIGAVINDIIADIKQRQTSPDARPGAAIII
PPGDYDLHTQVVVDVSYLTIAGFGHGFFSRSILDNSNPTGWQNLQPGASHIRVLTSPSAPQ
AFLVKRAGDPRLSGIVFRDFCLDGVGFTPGKNSYHNGKTGIEVASDNDSFHITGMGFVYLE
HALIVRGADALRVNDNMIAECGNCVELTGAGQATIVSGNHMGAGPDGVTLLAENHEGL
LVTGNNLFPRGRSLIEFTGCNRCSVTSNRLQGFYPGMLRLLNGCKENLITANHIRRTNEGYP
PFIGRGNGLDDLYGVVHIAGDNNLISDNLFAYNVPPANIAPAGAQPTQILIAGGDANVVAL
NHVVSDVASQHVVLDASTTHSKVLDSGTASQITSYSSDTAIRPTP
11111111111000000000000000000000000000000000000000000000000000000000000000000000000
00000000000000000000000000000000000000000000000000000000000000000000000000000000000
00000000000000000000000000000000000000000000000000000000000000000000000000000000000
00000000000000000000000000000000000000000000000000000000000000000000000000000000000

000000000000000000000000000000000000000000000000000000000000000000000000000000
>Q2T1U7_4g67_1_409
SQVSAGTVIYNAGMNPKAHEPASLPVPGPDALAQSEALAASLRAEIASAGGWIPFSRYMER
VLYAPGMGYYSGGAQKFGRRADDGSDFVTAPELSPLFAQTLARPVAQALDASGTRRVMEF
GAGTGKLAAGLLTALAALGVELDEYAIVDLSGELRARQRETLGAQAPGLAARVRWLDALP
ERFEGVVVGNEVLDAMPVRLVAKQARGWCERGVSIDDAGAFVFADRPFARAEEAARLAGI
DADEGYVTETHDAAVAFVRTVCAMLARGAAFFIDYGFPSHEYYHRQRAQGTLMCHYRHR
AHGDPFVYPGLQDITAHVEFSAIHEAGVGAGADLLGYTSQARFLLNAGITDVLAEIDPSDA
QHFLPAANAVQKLISEAEMGELFKVIAFSRGIDGALDAFARGDRSHTL
11111111111111111111110000000000000000000000000000000000000000000000000000000000000
00000000000000000000000000000000000000000000000000000000000000000000000000000000000
00000000000000000000000000000000000000000000000000000000000000000000000000000000000
00000000000000000000000000000000000000000000000000000000000000000000000000000000000
00000000000000000000000000000000000000000000000000000000000000000000000000000
>Q5LQF6_3h5l_30_438
LNSAAQAQSSDPVVIGCPAPLTGIVAADGIEFQRGIQMAADEINAVGGILGRPIELVFADTQS
KGVDVVIQSAQRLIDRDNASALIAGYNLENGTALHDVAADAGVIAMHANTVAVHDEMV
KSDPDRYWGTFQYDPPETLYGGGFLKFLKDIEDNGEFSRPNNKIAIITGPGIYSVNIANAIRD
GAGEYGYDVSLFETVAIPVSDWGPTLAKLRADPPAVIVVTHFYPQDQALFMNQFMTDPTN
SLVYLQYGASLAAFRDIAGDNSVGVTYATVLGTLQDEMGDAFAKAYKERYGDLSSTASGCQ
TYSALYAYSIAAALAGGPGAPYDDVQNKAVADRLRSLIFRGPVGTMRFHADTQSAWSYPTE
TNDPSLGMPHIFSQIFDKAEDGVLIAPAPYKKAGFKMPPWMKG
11111000000000000000000000000000000000000000000000000000000000000000000000000000000
00000000000000000000000000000000000000000000000000000000000000000000000000000000000
00000000000000000000000000000000000000000000000000000000000000000000000000000000000
00000000000000000000000000000000000000000000000000000000000000000000000000000000000
00000000000000000000000000000000000000000000000000000000000000000000000000011
>Q6MHT0_3v39_37_445
KVYLNSMCHMAANSKTQQIQGDDNKDDKFPLASISKVVTTLWAVDRLGPDYRFKTKLHV
TPTANGSYDIHIEGSRDPLFGRNMSYFLISELNRMKITKIEKLTFDENFLLAWLAEEKPMIGG
TTPKYDTVEQQASIVRATLTSSFATAISPGYYTILKTKAARIGVQMSNRPKIDVRTISFVKKAE
FQKNEKSTTMVLMSAPLKTILKRMNNQSNNYIADNLYWNLGGTEAFNAYIAGKMQADTS
DIEFHNGSGNNEGSVAKPVYNEATCEMMIKVLYSLDKSLSAKGYDLSDVMAVAAKDKAST
VGSYGGVMAGSTTAKTGSVNKAKTLMGSVSTKNGEIYFAVLMHTDYDKSRSDWGVASQQI
KNKVSQLINQNGGPKAIKYTEQLPLPFDKYSYLTKANTITTEKK
00000000000000000000000000000000000000000000000000000000000000000000000000000000000
00000000000000000000000000000000000000000000000000000000000000000000000000000000000
00000000000000000000000000000000000000000000000000000000000000000000000000000000000
00000000000000000000000000000000000000000000000000000000000000000000000000000000000
00000000000000000000000000000000000000000000000000000000000000000000011111111
>Q9X2A1_1vl2_0_408
MKEKVVLAYSGGLDTSVILKWLCEKGFDVIAYVANVGQKDDFVAIKEKALKTGASKVYVE
DLRREFVTDYIFTALLGNAMYEGRYLLGTAIARPLIAKRQVEIAEKEGAQYVAHGATGKGN
DQVRFELTYAALNPNLKVISPWKDPEFLAKFKGRTDLINYAMEKGIPIKVSKKRPYSEDENL
MHISHEAGKLEDPAHIPDEDVFTWTVSPKDAPDEETLLEIHFENGIPVKVVNLKDGTEKTD
PLELFEYLNEVGAKNGVGRLDMVENRFIGIKSRGVYETPGATILWIAHRDLEGITMDKEVM
HLRDMLAPKFAELIYNGFWFSPEMEFLLAAFRKAQENVTGKVTVSIYKGNVMPVARYSPYS
LYNPELSSMDVEGGFDATDSKGFINIHALRLKVHQLVKKGYQR
10000000000000000000000000000000000000000000000000000000000000000000000000000000000
00000000000000000000000000000000000000000000000000000000000000000000000000000000000
00000000000000000000000000000000000000000000000000000000000000000000000000000000000

00000000000000000000000000000000000000000000000000000000000000000000000000000000000
00000000000000000000000000000000000000111111110000000000000000000000000000000
>Q720C5_3jzl_0_407
MNNIQAIRKKVETQIDDLQNKTDEIAEFNQAKVLDAFQENKVSDFHFHPSTGYGYDDEGR
DTLERVYATVFKTEAALVRPQIISGTHAISTVLFGILRPDDELLYITGQPYDTLEEIVGIRKQGQ
GSLKDFHIGYSSVPLLENGDVDFPRIAKKMTPKTKMIGIQRSRGYADRPSFTIEKIKEMIVFVK
NINPEVIVFVDNCYGEFVEYQEPPEVGADIIAGSLIKNPGGGLAKTGGYIAGKEALVDLCGY
RLTTPGIGREAGASLYSLLEMYQGFFLAPHVTAQAIKGARFTAAMLAEFGVEADPVWDAP
RTDLIQSVSFHNKEKMVAFAQAIQAASPVNAHVLPIGAYMPGYEDDVIMAAGTFIQGASLE
LTADGPIREPYQLYVQGGLTYEHIKIAVTRAIQKIV
11100000000000000000000000000000000000000000000000000000000000000000000000000000000
00000000000000000000000000000000000000000000000000000000000000000000000000000000000
00000000000000000000000000000000000000000000000000000000000000000000000000000000000
00000000000000000000000000000000000000000000000000000000000000000000000000000000000
000000000000000000000000000000000000000000000000000000000000000000000000000
>Q8A1I2_3hrp_22_429
SDDNKTTGATYDPNQPVTVESFMPVEGKLREKVIVKGSNFGTDKSKVKVYFVDEAAERLST
VIGIDNNTLYCLAPRQLPGGNRIKVIVDGKEVTTDGTFKYEQAQNVSTISGSASKDGNDDG
DLASAKFKYMWGIAAVGNNTVLAYQRDDPRVRLISVDDNKVTTVHPGFKGGKPAVTKDK
QRVYSIGWEGTHTVYVYMKASGWAPTRIGQLGSTFSGKIGAVALDETEEWLYFVDSNKNFG
RFNVKTQEVTLIKQLELSGSLGTNPGPYLIYYFVDSNFYMSDQNLSSVYKITPDGECEWFCG
SATQKTVQDGLREEALFAQPNGMTVDEDGNFYIVDGFKGYCLRKLDILDGYVSTVAGQVD
VASQIDGTPLEATFNYPYDICYDGEGGYWIAEAWGKAIRKYAVE
11111111100000000000000000000000000000000000000000000000000000000000000000000000000
00000000000000000000000000000000000000000000000000000000000000000000000000000000000
00000000000000000000000000000000000000000000000000000000000000000000000000000000000
00000000000000000000000000000000000000000000000000000000000000000000000000000000000
000000000000000000000000000000000000000000000000000000000000000000000000000
>P96559_1iir_0_406
MRVLLATCGSRGDTEPLVALAVRVRDLGADVRMCAPPDCAERLAEVGVPHVPVGPSARAP
IQRAKPLTAEDVRRFTTEAIATQFDEIPAAAEGCAAVVTTGLLAAAIGVRSVAEKLGIPYFYA
FHCPSYVPSPYYPPPPLGEPSTQDTIDIPAQWERNNQSAYQRYGGLLNSHRDAIGLPPVEDIF
TFGYTDHPWVAADPVLAPLQPTDLDAVQTGAWILPDERPLSPELAAFLDAGPPPVYLGFGS
LGAPADAVRVAIDAIRAHGRRVILSRGWADLVLPDDGADCFAIGEVNHQVLFGRVAAVIH
HGGAGTTHVAARAGAPQILLPQMADQPYYAGRVAELGVGVAHDGPIPTFDSLSAALATAL
TPETHARATAVAGTIRTDGAAVAARLLLDAVSREKPTVSA
00000000000000000000000000000000000000000000000000000001111110000000000000000000000
00000000000000000000000000000000000000000000000000000000111111110000000000000000000
00000000000000000000000000000000000000000000000000000000000000000000000000000001110
00000000000000000000000000000000000000000000000000000000000000000000000000000000000
00000000000000000000000000000000000000000000000000000000000000000000111111
>Q7MVG6_3ll7_0_406
MLFDEKEILAITRWAKLYANQSPDRILLGSNDIPPEYRAAVATQIELWPRLRNKLPQWAGISS
LYIPSRLSLEQSSGAVTSSYKSRFIREGTKVVDLTGGLGIDFIALMSKASQGIYIERNDETAVAA
RHNIPLLLNEGKDVNILTGDFKEYLPLIKTFHPDYIYVDPARRSGADKRVYAIADCEPDLIPL
ATELLPFCSSILAKLSPMIDLWDTLQSLLHVQELHVVAAHGEVKELLVRMSLNEATIPPEKVP
IHAINLLLEDTVIPFIFTMEEERSISIPYTDSIDKYVYEPHTALLKAGAFKTVAYRLGLRKLHPN
SHLYTSEAYESAFPGRTFVLEEIIPFSTSVLKQLRKVVPQASISCRNFPLSPIELRQRSKMADGG
EKTLMGTTMADGKKVLLLLRKAE
00000000000000000000000000000000000000000000000000000000000000000000000000000000000
00000000000000000000000000000000000000000000000000000000000000000000000000000000000

00000111110000000000000000000000000000000000000000000000000000000000000000000000000
00000000000000100000000000000000000000000000000000000000000000000000000000000000000
00000000000000000000000000000000000000000000000000000000000000000000000000
>Q8PXX8_4dn7_0_406
MQTEQVSLKKRAESAAEKKAAFGEDFELEKYEEGSKVSKPIEDLQSLDEESKKTLLQVGVIP
SEEGRSGSFLVLDNAVSHSTLKDKNVELMSTHKAMEKYEWLKDYSWKLVQVDADKYTAK
TYLEDADGYFIRVPAGKKTSMPVQTCLMLGSKKAAQTVHNIIIVEEGATLDIITGCTTKKGV
EEGLHLGISEMYIKKGGTLNFTMIHNWAEQIGVRPRTVVSVEEGGTYVSNYICLKPVRSVQT
YPTVRLEGEGAVTRLNTIAIAHPGSELDLGSKAIFNAPGTRAELISRTITIGGRLIARGEMIGN
AKGAKGHLECKGLVLTDKGSQLAIPILEANVDDIELTHEAAVGKIAKDQVEYLMARGLTED
EAVGMIIRGFLDVGIRGIPEELKEEIENTIAQTALGM
10000000000000000000000000000000000000000000000000000000000000000000000000000000000
00000000000000000000000000000000000000000000000000000000000000000000000000000000000
00000000000000000000000000000000000000000000000000000000000000000000000000000000000
00000000000000000000000000000000000000000000000000000000000000000000000000000000000
00000000000000000000000000000000000000000000000000000000000000000000000000
>A9LI60_3g6m_20_425
RATPRMEDLASTDLSTRATGSINAVYFTNWGIYGRNFQPADLQASKILHVLYSFMNLRVDG
TVYSGDTYADLEKHYSDDSWNDIGTNAYGCVKQLYKLKKANRSLKIMLSIGGWTWSTNFP
AAASTEATRATFAKTAVEFMKDWGFDGIDVDWEYPASETDANNMVLLLQRVRQELDSYS
ATYANGYHFQLSIAAPAGPSHYNVLKLAQLGSVLDNINLMAYDYAGSWDSVSGHQTNLYP
STSNPSSTPFSTKAAVDAYIAAGVPASKIILGMPIYGRAFVGTDGPGKPYSTIGEGSWESGIW
DYKVLPKAGATVITDSAAGATYSYDSSSRTMISYDTPDMVRTKVSYAKGLGLGGSMFWEAS
ADKTGSDSLIGTALSSMGSHDSTQNCLSYPNSKFDNIKNSLS
11111111111111111100000000000000000000000000000000000000000000000000000000000000000
00000000000000000000000000000000000000000000000000000000000000000000000000000000000
00000000000000000000000000000000000000000000000000000000000000000000000000000000000
00000000000000000000000000000000000000000000000000000000000000000000000000000000000
0000000000000000000000000000000000000000000000000000000000000000000000000
>Q8RJN8_1xwt_21_425
AFNNNPSSVGAYSSGTYRNLAQEMGKTNIQQKVNSTFDNMFGYNNTQQLYYPYTENGVY
KAHYIKAINPDEGDDIRTEGQSWGMTAAVMLNKQEEFDNLWRFAKAYQKNPDNHPDAK
KQGVYAWKLKLNQNGFVYKVDEGPAPDGEEYFAFALLNASARWGNSGEFNYYNDAITML
NTIKNKLMENQIIRFSPYIDNLTDPSYHIPAFYDYFANNVTNQADKNYWRQVATKSRTLLK
NHFTKVSGSPHWNLPTFLSRLDGSPVIGYIFNGQANPGQWYEFDAWRVIMNVGLDAHLM
GAQAWHKSAVNKALGFLSYAKTNNSKNCYEQVYSYGGAQNRGCAGEGQKAANAVALL
ASTNAGQANEFFNEFWSLSQPTGDYRYYNGSLYMLAMLHVSGNFKFYNNTFN
00000000000000000000000000000000000000000000000000000000000000000000000000000000000
00000000000000000000000000000000000000000000000000000000000000000000000000000000000
00000000000000000000000000000000000000000000000000000000000000000000000000000000000
00000000000000000000000000000000000000000000000000000000000000000000000000000000000
000000000000000000000000000000000000000000000000000000000000000000000001
>Q9FN03_4d9s_0_404
MAEDMAADEVTAPPRKVLIISAGASHSVALLSGDIVCSWGRGEDGQLGHGDAEDRPSPTQ
LSALDGHQIVSVTCGADHTVAYSQSGMEVYSWGWGDFGRLGHGNSSDLFTPLPIKALHGI
RIKQIACGDSHCLAVTMEGEVQSWGRNQNGQLGLGDTEDSLVPQKIQAFEGIRIKMVAAG
AEHTAAVTEDGDLYGWGWGRYGNLGLGDRTDRLVPERVTSTGGEKMSMVACGWRHTIS
VSYSGALYTYGWSKYGQLGHGDLEDHLIPHKLEALSNSFISQISGGWRHTMALTSDGKLYG
WGWNKFGQVGVGNNLDQCSPVQVRFPDDQKVVQVSCGWRHTLAVTERNNVFAWGRG
TNGQLGIGESVDRNFPKIIEALSVDGASGQHIESSNIDPSSGKSWVSPAE
11111111111110000000000000000000000000000000000000000000000000000000000000000000000

00000000000000000000000000000000000000000000000000000000000000000000000000000000
00000000000000000000000000000000000000000000000000000000000000000000000000000000
00000000000000000000000000000000000000000000000000000000000000000000000000000000
0000000000000000000000000000000000000000000000000000000000000111111111
>Q9UZR7_2jjq_0_404
MRGVIRKLNDDGFGVLKGILVPFSAPGDEIIVERVERVKKRRVASQWKLVRSSPLRVGPRCK
AFGKCGGCTLQHLNYDYQLEFKRKKLKRILGFEVEVVPSPKIFGHRNRIDLAITKDGIGFRE
RGKWWKIVDIDECPVFGKTSREAIERLKEFIEEEKISVWNIKKDEGFLRYMVLREGKFTEEV
MVNFVTKEGNLPDPTNYFDFDSIYWSVNRSKSDVSYGDIERFWGKEFIRERLDDVDYLIHP
NSFFQTNSYQAVNLVRKVSELVEGEKILDMYSGVGTFGIYLAKRGFNVKGFDSNEFAIEMAR
RNVEINNVDAEFEVASDREVSVKGFDTVIVDPPRAGLHPRLVKRLNREKPGVIVYVSCNPET
FARDVKMLDYRIDEIVALDMFPHTPHVELVAKLV
00000000000000000000000000000000000000000000000000000001111111111100000000000000
00000000000000000000000000000000000000001100000000000000000000000000000000000000
00000000000000000000000000000000000000000000000000000000000000000000000000000000
00000000000000000000000000000000000000000000000000000000000000000000000000000000
0000000000000000000000000000000000000000000000000000000000000000000000
>Q9UXR8_1gpj_0_403
MEDLVCVGITHKEAEVEELEKARFESDEAVRDIVESFGLSGCVLLQTCNRVEVYASGARDR
AEELGDLIHDDAWVKRGSEAVRHLFRVACGLESMMVGEQEILRQVKKAYDRAARLGTLDE
ALKIVFRRAINLGKRAREETRISEGAVSIGSAAVELAERELGSLHDKTVLVVGAGEMGKTVA
KSLVDRGVRAVLVANRTYERAVELARDLGGEAVRFDELVDHLARSDVVVSATAAPHPVIHV
DDVREALRKRDRRSPILIIDIANPRDVEEGVENIEDVEVRTIDDLRVIARENLERRRKEIPKVE
KLIEEELSTVEEELEKLKERRLVADVAKSLHEIKDRELERALRRLKTGDPENVLQDFAEAYTK
RLINVLTSAIMELPDEYRRAACRALRRASELNG
00000000000000000000000000000000000000000000000000000000000000000000000000000000
00000000000000000000000000000000000000000000000000000000000000000000000000000000
00000000000000000000000000000000000000000000000000000000000000000000000000000000
00000000000000000000000000000000000000000000000000000000000000000000000000000000
000000000000000000000000001111100000000000000000000000000000000000000
>P15363_3e78_0_402
MLKKLKNFILFSSIFSPIAFAISCSNTGVVKQEDVSVSQGQWDKSITFGVSEAWLNKKKGGEK
VNKEVINTFLENFKKEFNKLKNANDKTKNFDDVDFKVTPIQDFTVLLNNLSTDNPELDFGI
NASGKLVEFLKNNPGIITPALETTTNSFVFDKEKDKFYVDGTDSDPLVKIAKEINKIFVETPYA
SWTDENHKWNGNVYQSVYDPTVQANFYRGMIWIKGNDETLAKIKKAWNDKDWNTFRN
FGILHGKDNSFSKFKLEETILKNHFQNKFTTLNEDRSAHPNAYKQKSADTLGTLDDFHIAFS
EEGSFAWTHNKSATKPFETKANEKMEALIVTNPIPYDVGVFRKSVNQLEQNLIVQTFINLA
KNKQDTYGPLLGYNGYKKIDNFQKEIVEVYEKAIK
11111111111111111111111111111111111100000000000000000000000000000000000000000000
00000000000000000000000000000000000000000000000000000000000000000000000000000000
00000000000000000000000000000000000000000000000000000000000000000000000000000000
00000000000000000000000000000000000000000000000000000000000000000000000000000000
00000000000000000000000000000000000000000000000000000000000000000000
>P43593_1vjv_96_498
PEQQVQQFAQLPVGFKNMGNTCYLNATLQALYRVNDLRDMILNYNPSQGVSNSGAQDEE
IHKQIVIEMKRCFENLQNKSFKSVLPIVLLNTLRKCYPQFAERDSQGGFYKQQDAEELFTQL
FHSMSIVFGDKFSEDFRIQFKTTIKDTANDNDITVKENESDSKLQCHISGTTNFMRNGLLEG
LNEKIEKRSDLTGANSIYSVEKKISRLPKFLTVQYVRFFWKRSTNKKSKILRKVVFPFQLDVA
DMLTPEYAAEKVKVRDELRKVEKEKNEKEREIKRRKFDPSSSENVMTPREQYETQVALNES
EKDQWLEEYKKHFPPNLEKGENPSCVYNLIGVITHQGANSESGHYQAFIRDELDENKWYK
FNDDKVSVVEKEKIESLAGGGESDSALILMYKGFGL

11111100000000000000000000000000000000000000000000000000000000000000000000000010000
00000000000000000000111100000000000000000000000000000000000000000000000000000000000
00000000000000000000011111111100000000000000000000000000000000000000000000000000000
00000000000000000000000001111111111111111000000000000000000000000000000000000000000
00000000000000000000000000000000000000000000000000000000000000000000000
>P78563_1zy7_298_740
LHLDQTPSRQPIPSEGLQLHLPQVLADAVSRLVLGKFGDLTDNFSSPHARRKVLAGVVMTT
GTDVKDAKVISVSTGTKCINGEYMSDRGLALNDCHAEIISRRSLLRFLYTQLELYLNNKDDQ
KRSIFQKSERGGFRLKENVQFHLYISTSPCGDARIFSPHEPILEEPADRHPNRKARGQLRTKIE
SGEGTIPVRSNASIQTWDGVLQGERLLTMSCSDKIARWNVVGIQGSLLSIFVEPIYFSSIILGSL
YHGDHLSRAMYQRISNIEDLPPLYTLNKPLLSGISNAEARQPGKAPNFSVNWTVGDSAIEVI
NATTGKDELGRASRLCKHALYCRWMRVHGKVPSHLLRSKITKPNVYHESKLAAKEYQAA
KARLFTAFIKAGLGAWVEKPTEQDQFSLTP
11111110000000000000000000000000000000000000000000000000000000000000000000000000000
00000000000000000000000000000000000000000000000000000000000000000000000000000000111
11111111100000000000000000000000000000000000000000000000000000000000000000000000000
00000000000000000000000000000000000000000000000000000000000000000000000000000000000
00000000000000000000000000000000000000000000000000000000000000000000001
>O08375_2gsd_0_401
MAKVVCVLYDDPINGYPTSYARDDLPRIDKYPDGQTLPTPKAIDFTPGALLGSVSGELGLRK
YLESQGHELVVTSSKDGPDSELEKHLHDAEVIISQPFWPAYLTAERIAKAPKLKLALTAGIGS
DHVDLQAAIDNNITVAEVTYCNSNSVAEHVVMMVLGLVRNYIPSHDWARNGGWNIADC
VARSYDVEGMHVGTVAAGRIGLRVLRLLAPFDMHLHYTDRHRLPEAVEKELNLTWHATR
EDMYGACDVVTLNCPLHPETEHMINDETLKLFKRGAYLVNTARGKLCDRDAIVRALESGR
LAGYAGDVWFPQPAPNDHPWRTMPHNGMTPHISGTSLSAQTRYAAGTREILECYFEGRPI
RDEYLIVQGGGLAGVGAHSYSKGNATGGSEEAAKYEKLDA
10000000000000000000000000000000000000000000000000000000000000000000000000000000000
00000000000000000000000000000000000000000000000000000000000000000000000000000000000
00000000000000000000000000000000000000000000000000000000000000000000000000000000000
00000000000000000000000000000000000000000000000000000000000000000000000000000000000
0000000000000000000000000000000000000000000000000000000000000000000011
>Q2P9J6_3s8m_0_401
MIIHPKVRGFICTTTHPLGCERNVLEQIAATRARGVRNDGPKKVLVIGASSGYGLASRITAAF
GFGADTLGVFFEKPGTASKAGTAGWYNSAAFDKHAKAAGLYSKSINGDAFSDAARAQVIE
LIKTEMGGQVDLVVYSLASPVRKLPGSGEVKRSALKPIGQTYTATAIDTNKDTIIQASIEPASA
QEIEETITVMGGQDWELWIDALEGAGVLADGARSVAFSYIGTEITWPIYWHGALGKAKVD
LDRTAQRLNARLAKHGGGANVAVLKSVVTQASAAIPVMPLYISMVYKIMKEKGLHEGTIE
QLDRLFRERLYRQDGQPAEVDEQNRLRLDDWELRDDVQDACKALWPQVTTENLFELTDY
AGYKHEFLKLFGFGRTDVDYDADVATDVAFDCIELA
11111111111000000000000000000000000000000000000000000000000000000000000000000000000
00000000000000000000000000000000000000000000000000000000000000000000000000000000000
00000000000000000000000000000000000000000000000000000000000000000000000000000000000
00000000000000000000000000000000000000000000000000000000000000000000000000000000000
0000000000000000000000000000000000000000000000000000000000000000000001
>Q9RHZ6_1urs_25_426
CGTSNGGQNTSPSTSSSSAKGEASALPKGQTITVWSWQTGPELQDVKQIAAQWAKAHGD
KVIVVDQSSNPKGFQFYATAARTGKGPDVVFGMPHDNNGVFAEEGLMAPVPSGVLNTGL
YAPNTIDAIKVNGTMYSVPVSVQVAAIYYNKKLVPQPPQTWAEFVKDANAHGFMYDQAN
LYFDYAIIGGYGGYVFKDNNGTLDPNNIGLDTPGAVQAYTLMRDMVSKYHWMTPSTNGSI
AKAEFLAGKIGMYVSGPWDTADIEKAKIDFGVTPWPTLPNGKHATPFLGVITAFVNKESKT
QAADWSLVQALTSAQAQQMYFRDSQQIPALLSVQRSSAVQSSPTFKAFVEQLRYAVPMPNI

PQMQAVWQAMSILQNIIAGKVSPEQGAKDFVQNIQKGIMAQGS
11111111111111111111111111111000000000000000000000000000000000000000000000000000000
00000000000000000000000000000000000000000000000000000000000000000000000000000000000
00000000000000000000000000000000000000000000000000000000000000000000000000000000000
00000000000000000000000000000000000000000000000000000000000000000000000000000000000
0000000000000000000000000000000000000000000000000000000000000000111111
>C4M036_3oc9_0_400
MTYQPVDITTNTIPVTKEHYYRGLELISQGKTALITLAGGQGSRLGFEHPKGMFVLPFEIPKSI
FQMTSERLLRLQELASEYSHQKNVMIHWFLMTNEETIEEINNYFKEHQYFGLSSEQIHCFPQ
GMLPVVDFNGKILYEKKDKPYMAPNGHGGLFKALKDNGILEFMNEKGIKYSVAHNVDNI
LCKDVDPNMIGYMDLLQSEICIKIVKKGFKEEKVGVLVKEQERIKVVEYTELTDELNKQLSN
GEFIYNCGHISINGYSTSFLEKAAEYQLPYHIAKKKVPFVNEQGIVIHPSENNGIKKEMFFFD
VFPLATKVSIFEIQRFIEFSALKNSLNESFDNVNTVKRDWYRLNIYYLKKAGAIVDDSKSPICE
ISFRKSFEEEGLKEFKGKTIQLPFILQ
10000000000000000000000000000000000000000000000000000000000000000000000000000000000
00000000000000000000000000000000000000000000000000000000000000000000000000000000000
00000000000000000000000000000000000000000000000000000000000000000000000000000000000
00000000000000000000000000000000000000000000000000000000000000000000000000000000000
000000000000000000000000000000000000000000000000000000000000000000000
>D0G7Q4_3vrd_30_430
AGRKVVVVGGGTGGATAAKYIKLADPSIEVTLIEPNETYYTCYMSNEVIGGDRELASLRVGY
DGLRAHGIQVVHDSALGIDPDKKLVKTAGGAEFAYDRCVVAPGIDLLYDKIEGYSEALAAK
LPHAWKAGEQTALLRRQLESMDDGGVVIIAPPAPPFRCPPGPYERASQIAHYLKAHKSKSK
VIILDNSQTFSKQAQFTKGWERLYGFGTENALIEWHPGPDAAVVKTDTEAMTVETSFGETF
KAAVINLIPPQRAGKIAQSASLTNDSGWCPVDIRTFESSLQPGIHVIGDACNAAPMPKSAYS
ANSQAKVAAAAVVALLKGEEPGTPSYLNTCYSILAPGYGISIAAVYRPNAEGKAIEAVPDSG
GITPVDAPDWVLEREVQYAHSWYNNIVHDTFG
10000000000000000000000000000000000000000000000000000000000000000000000000000000000
00000000000000000000000000000000000000000000000000000000000000000000000000000000000
00000000000000000000000000000000000000000000000000000000000000000000000000000000000
00000000000000000000000000000000000000000000000000000000000000000000000000000000000
000000000000000000000000000000000000000000000000000000000000000000000
>Q82X47_3b9w_24_424
SAVAPAEINEARLVAQYNYSINILAMLLVGFGFLMVFVRRYGFSATTGTYLVVATGLPLYILLR
ANGIFGHALTPHSVDAVIYAEFAVATGLIAMGAVLGRLRVFQYALLALFIVPVYLLNEWLVL
DNASGLTEGFQDSAGSIAIHAFGAYFGLGVSIALTTAAQRAQPIESDATSDRFSMLGSMVLW
LFWPSFATAIVPFEQMPQTIVNTLLALCGATLATYFLSALFHKGKASIVDMANAALAGGVAI
GSVCNIVGPVGAFVIGLLGGAISVVGFVFIQPMLESKAKTIDTCGVHNLHGLPGLLGGFSAI
LIVPGIAVAQLTGIGITLALALIGGVIAGALIKLTGTTKQAYEDSHEFIHLAGPEDEHKAERLV
LEAKTEIQGLKNRIDAAVLSAKSEG
11111110000000000000000000000000000000000000000000000000000000000000000000000000000
00000000000000000000000000000000000000000000000000000000000000000000000000000000000
00000000000000000000000000000000000000000000000000000000000000000000000000000000000
00000000000000000000000000000000000000000000000000000000000000000000000000000000000
000000000000000000000000000000000000011111111111111111111111111111111
>P04519_1y8z_0_399
MRICIFMARGLEGCGVTKFSLEQRDWFIKNGHEVTLVYAKDKSFTRTSSHDHKSFSIPVILAK
EYDKALKLVNDCDILIINSVPATSVQEATINNYKKLLDNIKPSIRVVVYQHDHSVLSLRRNLG
LEETVRRADVIFSHSDNGDFNKVLMKEWYPETVSLFDDIEEAPTVYNFQPPMDIVKVRSTY
WKDVSEINMNINRWIGRTTTWKGFYQMFDFHEKFLKPAGKSTVMEGLERSPAFIAIKEKGI
PYEYYGNREIDKMNLAPNQPAQILDCYINSEMLERMSKSGFGYQLSKLNQKYLQRSLEYTH

LELGACGTIPVFWKSTGENLKFRVDNTPLTSHDSGIIWFDENDMESTFERIKELSSDRALYDR
EREKAYEFLYQHQDSSFCFKEQFDIITK
00000000000000000000000000000000000000000000000000000000000000000000000000000000000
00000000000000000000000000000000000000000000000000000000000000000000000001111111111
10000000000000000000000000000000000000000000000000000000000000000000000000000000000
00000000000000000000000000000000000000000000000000000000000000000000000000000000000
00000000000000000000000000000000000000000000000000000000000000000000
>Q93J45_3s2j_0_399
MTSLEKARELLREFPVVDGHNDLPWALREQVRYDLDARDIAADQSAHLHTDLARLRSGG
VGAQYWSVYVRSDLPGAVTATLEQIDCVRRLIDRHPGELRAALTAADMEAARAEGRIASL
MGAEGGHSIDNSLATLRALYALGVRYMTLTHNDNNAWADSATDEPGVGGLSAFGREVVR
EMNREGMLVDLSHVAATTMRDALDTSTAPVIFSHSSSRAVCDHPRNIPDDVLERLSANGG
MAMVTFVPKFVLQAAVDWTAEADDNMRAHGFHHLDSSPEAMKVHAAFEERVPRPVATV
STVADHLDHMREVAGVDHLGIGGDYDGTPFTPDGLGDVSGYPNLIAELLDRGWSQSDLA
KLTWKNAVRVLDAAEDVSRGLRAARGPSNATIEQLDGTAAEQPEG
00000000000000000000000000000000000000000000000000000000000000000000000000000000000
00000000000000000000000000000000000000000000000000000000000000000000000000000000000
00000000000000000000000000000000000000000000000000000000000000000000000000000000000
00000000000000000000000000000000000000000000000000000000000000000000000000000000000
00000000000000000000000000000000000000000000000000000000000001111111
>F7P4T2_4dzi_1_399
TALNYRVIDVDNHYYEPLDSFTRHLDKKFKRRGVQMLSDGKRTWAVIGDRVNHFIPNPTF
DPIIVPGCLDLLFRGEIPDGVDPASLMKVERLADHPEYQNRDARIAVMDEQDIETAFMLPTF
GCGVEEALKHDIEATMASVHAFNLWLDEDWGFDRPDHRIIAAPIVSLADPTRAVEEVDFV
LARGAKLVLVRPAPVPGLVKPRSLGDRSHDPVWARLAEAGVPVGFHLSDSGYLHIAAAWG
GKSTFEGFGAKDPLDQVLLDDRAIHDTMASMIVHGVFTRHPKLKAVSIENGSYFVHRLIKR
LKKAANTQPQYFPEDPVEQLRNNVWIAPYYEDDLPELARVIGVDKILFGSDWPHGEGLAS
PVSFTAELKGFSESDIRKIMRDNALDLLGVQVGSAA
10000000000000000000000000000000000000000000000000000000000000000000000000000000000
00000000000000000000000000000000000000000000000000000000000000000000000000000000000
00000000000000000000000000000000000000000000000000000000000000000000000000000111111
11000000000000000000000000000000000000000000000000000000000000000000000000000000000
0000000000000000000000000000000000000000000000000000000000001111111
>P46130_3grh_28_426
QRPSDQTAPGTSSRPILSAKEAQNFDAQHYFASLTPGAAAWNPSPITLPAQPDFVVGPAGT
QGVTHTTIQAAVDAAIIKRTNKRQYIAVMPGEYQGTVYVPAAPGGITLYGTGEKPIDVKIGL
SLDGGMSPADWRHDVNPRGKYMPGKPAWYMYDSCQSKRSDSIGVLCSAVFWSQNNGLQ
LQNLTIENTLGDSVDAGNHPAVALRTDGDQVQINNVNILGRQNTFFVTNSGVQNRLETNR
QPRTLVTNSYIEGDVDIVSGRGAVVFDNTEFRVVNSRTQQEAYVFAPATLSNIYYGFLAVNSR
FNAFGDGVAQLGRSLDVDANTNGQVVIRDSAINEGFNTAKPWADAVISNRPFAGNTGSVD
DNDEIQRNLNDTNYNRMWEYNNRGVGSKVVAEAKK
00000000000000000000000000000000000000000000000000000000000000000000000000000000000
00000000000000000000000000000000000000000000000000000000000000000000000000000000000
00000000000000000000000000000000000000000000000000000000000000000000000000000000000
00000000000000000000000000000000000000000000000000000000000000000000000000000000000
0000000000000000000000000000000000000000000000000000000000001111111
>Q83WF2_3sso_0_398
MTAQTEFDEATVQDVVRLAGGHDSELRELTQKYDPAMISRLLVAEILSRCPPPSNDTPVLVE
LAIVHGSERFRHFLRVVRDSPIRPVGADEGFVGMLVEYELTELLRELFGVTHERPAGVRGTK
LFPYLTDDEEAVEQIGTYLLAAQQGTEAVLAGCGSRKPDLSELSSRYFTPKFGFLHWFTPHY
DRHFRDYRNQQVRVLEIGVGGYKHPEWGGGSLRMWKSFFPRGQIYGLDIMDKSHVDELRI

RTIQGDQNDAEFLDRIARRYGPFDIVIDDGSHINAHVRTSFAALFPHVRPGGLYVIEDMWT
AYWPGFGGQADPQECSGTSLGLLKSLIDAIQHQELPSDPNRSPGYVDRNIVGLHVYHNVAF
VEKGRNDEGGIPTWIPRDFESLVQASSGGAT
11111000000000000000000000000000000000000000000000000000000000000000000000000000000
00000000000000000000000000000000000000000000000000000000000000000000000000000000000
00000000000000000000000000000000000000000000000000000000000000000000000000000000000
00000000000000000000000000000000000000000000000000000000000000000000000000000000000
0000000000000000000000000000000000000000000000000000000000000000000
>Q83XN4_3tm1_0_398
MDPEAGSRARGLKGVHRVSANSGGIGGVPGPHNGLTDVPGVRVGHAGRTGDGWLTGVT
VVLAPPGGAVAAVDVRGGGPGTRETDALDPRNLVQTIDAVVLTGGSAFGLDAAGGVAAW
LEEQGRGFPVGADPSQVVPVVPAAALFDLGRGGTWRARPDAALGRAAVEAAAARPEGDP
VEQGGVGAGTGAVVGGLKGGIGTASVVLDSGATVAALAAVNAAGSAVDPATGVLYGART
GLPGEFAGYGVPDAIGADTHARARARLAEAAEETARRRAGGAATLNTTLAVVATDATLTR
AQAQKLAGTAHDGLARAVRPVHLLSDGDTVFALSTGRRPLLPDRPDATAARAFGVHLEA
GALNEVLAAGADVLTRAVVHAVLAATGVDTPGGVHPSYRELYARP
11111111111111111111111000000000000000000000000000000000000000000000000000000000000
00000000000000000000000000000000000000000000000000000000000000000000000000000000000
00000000000000000000000000000000000000000000000000000000000000000000000000000000000
00000000000000000000000001111111000000000000000000000000000000000000000000000000000
0000000000000000000000000000000000000000000000000000000000000000011
>A6TYH5_3l5a_1_398
YRYKPLLQSIHLPNGIKISNRFVLSPMTVNASTKEGYITKADLAYAARRSNSAGMQVTGAAY
IEPYGKLFEYGFNIDHDACIPGLTNMASTMKQHGSLAIIQLAHAGRFSNQAILNFGKVYGPS
PMTLHSPIEHVVIAMSHEKINSIIQQYRDATLRAIKAGFDGVEISIAQRLLIQTFFSTFSNRRTD
HYGADSLKNRARLCLEVMRAVQEVIDKEAPDNFILGFRATPEETRGSDLGYTIDEFNQLID
WVMDVSNIQYLAIASWGRHIYQNTSRTPGDHFGRPVNQIVYEHLAGRIPLIASGGINSPESA
LDALQHADMVGMSSPFVTEPDFVHKLAEQRPHDINLEFSMADLEDLAIPHAAFKDIVKM
MDYGEGLKKHTRDALRQLEQNYHDSSS
00000000000000000000000000000000000000000000000000000000000000000000000000000000000
00000000000000000000000000000000000000000000000000000000000000000000000000000000000
00000000000000000000000000000000000000000000000000000000000000000000000000000000000
00000000000000000000000000000000000000000000000000000000000000000000000000000000000
000000000000000000000000000000000000000000000000111111111111111111
>P46154_1kol_1_398
SGNRGVVYLGSGKVEVQKIDYPKMQDPRGKKIEHGVILKVVSTNICGSDQHMVRGRTTAQ
VGLVLGHEITGEVIEKGRDVENLQIGDLVSVPFNVACGRCRSCKEMHTGVCLTVNPARAGG
AYGYVDMGDWTGGQAEYLLVPYADFNLLKLPDRDKAMEKIRDLTCLSDILPTGYHGAVTA
GVGPGSTVYVAGAGPVGLAAAASARLLGAAVVIVGDLNPARLAHAKAQGFEIADLSLDTP
LHEQIAALLGEPEVDCAVDAVGFEARGHGHEGAKHEAPATVLNSLMQVTRVAGKIGIPGL
YVTEDPGAVDAAAKIGSLSIRFGLGWAKSHSFHTGQTPVMKYNRALMQAIMWDRINIAEV
VGVQVISLDDAPRGYGEFDAGVPKKFVIDPHKTFSAA
10000000000000000000000000000000000000000000000000000000000000000000000000000000000
00000000000000000000000000000000000000000000000000000000000000000000000000000000000
00000000000000000000000000000000000000000000000000000000000000000000000000000000000
00000000000000000000000000000000000000000000000000000000000000000000000000000000000
000000000000000000000000000000000000000000000000000000000000000001
>Q8R9Q6_3okg_0_397
MTEGPYKLPPGWRWVRLGEVCLPTERRDPTKNPSTYFVYVDISAIDSTVGKIVSPKEILGQH
APSRARKVIRSGDVIFATTRPYLKNIALVPPDLDGQICSTGFCVIRANREFAEPEFLFHLCRSD
FITNQLTASKMRGTSYPAVTDNDVYNTLIPLPPLEEQRRIVAKVEALMERVREVRRLRAEAQ

KDTELLMQTALAEVFPHPGADLPPGWRWVRLGEVCDIIMGQSPPSSTYNFEGNGLPFFQG
KADFGDLHPTPRIWCSAPQKVARPGDVLISVRAPVGSTNVANLACCIGRGLAALRPRDSLE
RFWLLYYLHYLEPELSKMGAGSTFNAITKKDLQNVFIPLPPLEEQRRIVAYLDQIQQQVAAL
KRAQAETEAELKRLEQAILDKAFRGDL
00000000000000000000000000000000000000000000000000000000000000000000000000000000000
00000000000000000000000000000000000000000000000000000000000000000000000000000000000
00000000000000000000000000000000000000000000000000000000000000000000000000000000000
00000000000000000000000000000000000000000000000000000000000000000000000000000011111
11000000000000000000000000000000000000000000000000000000000000000
>Q9HWJ1_2x3n_0_397
MTDNHIDVLINGCGIGGAMLAYLLGRQGHRVVVVEQARRERAINGADLLKPAGIRVVEA
AGLLAEVTRRGGRVRHELEVYHDGELLRYFNYSSVDARGYFILMPCESLRRLVLEKIDGEAT
VEMLFETRIEAVQRDERHAIDQVRLNDGRVLRPRVVVGADGIASYVRRRLLDIDVERRPYPS
PMLVGTFALAPCVAERNRLYVDSQGGLAYFYPIGFDRARLVVSFPREEARELMADTRGESLR
RRLQRFVGDESAEAIAAVTGTSRFKGIPIGYLNLDRYWADNVAMLGDAIHNVHPITGQGM
NLAIEDASALADALDLALRDACALEDALAGYQAERFPVNQAIVSYGHALATSLEDRQRFA
GVFDTALQGSSRTPEALGGERSYQPVRSPAPLG
11100000000000000000000000000000000011100000000000000000000000000000000000000000000
00000000000000000000000000000000000000000000000000000000000000000000000000000000000
00000000000000000000000000000000000000000000000000000000000000000000000000000000000
00000000000000000000000000000000000000000000000000000000000000000000000000000000000
00000000000000000000000000000000000000111111111111111111111111111
>O66036_4dnx_0_396
MIKPVGSDELRPRFVYDPEQHHRLSSEAESLPSVIVSSQAAGNAVMLGAGYFSPLDGFMNL
ADALSSAQSMTLTDGRFFPVPLLCLLESADAIAGATRIALRDPNVEGNPVLAVMDVTAVEQ
VSDAQMALMTEQVYGTSDPKHPGVETFNSQGRTAISGPIQVLNFSYFQTDFPDTFRTAVEIR
HEIQERGWQKIVAFQTRNPMHRAHEELCKMAMEAVEADGVVIHMLLGQLKPGDIPAPVR
DAAIRTMAELYFPPNTVMVTGYGFDMLYAGPREAVLHAYFRQNMGATHFIIGRDHAGVG
DYYGPFDAQTIFDDAVPTDVLAIEIFRADNTAYSKKLGRVVMMRDAPDHTPDDFIQLSGTR
VREMLGQGEAPPPEFSRPEVAQILMDYYRSLPQS
00000000000000000000000000000000000000000000000000000000000000000000000000000000000
00000000000000000000000000000000000000000000000000000000000000000000000000000000000
00000000000000000000000000000000000000000000000000000000000000000000000000000000000
00000000000000000000000000000000000000000000000000000000000000000000000000000000000
0000000000000000000000000000000000000000000000000000000000000001
>O86049_3p24_0_396
MKNVKLLLMLGTAALLAACSNEADSLTTSIDAPVTASIDLQSVSYTDLATQLNDVSDFGKM
IILKDNGFNRQVHVSMDKRTKIQLDNENVRLFNGRDKDSTNFILGDEFAVLRFYRNGESISY
IAYKEAQMMNEIAEFYAAPFKKTRAINEKEAFECIYDSRTRSAGKYPVSVKINVDKAKKILN
LPECDYINDYIKTPQVPHGITESQTRAVPSEPKTVYVICLRENGSTVYPNEVSAQMQDAANS
VYAVHGLKRYVNLHFVLYTTEYACPSGNADEGLDGFTASLKANPKAEGYDDQIYFLIRWGT
WDNNILGISWLNSYNVNTASDFKASGMSTTQLMYPGVMAHELGHILGANHADDPKDLM
YSKYTGYLFHLSEKNMDIIAKNLGWEIADGD
11111111111111111111111111111111000000000000000000000000000000000000000000000000000
00000000000000000000000000000000000000000000000000000000000000000000000000000000000
00000000000000000000000000000000000111111111000000000000000000000000000000000000000
00000000000000000000000000000000000000000000000000000000000000000000000000000000000
0000000000000000000000000000000000000000000000000000000000000000
>P0A2K1_1k8x_0_396
MTTLLNPYFGEFGGMYVPQILMPALNQLEEAFVSAQKDPEFQAQFADLLKNYAGRPTALT
KCQNITAGTRTTLYLKREDLLHGGAHKTNQVLGQALLAKRMGKSEIIAETGAGQHGVASA

LASALLGLKCRIYMGAKDVERQSPNVFRMRLMGAEVIPVHSGSATLKDACNEALRDWSGS
YETAHYMLGTAAGPHPYPTIVREFQRMIGEETKAQILDKEGRLPDAVIACVGGGSNAIGMF
ADFINDTSVGLIGVEPGGHGIETGEHGAPLKHGRVGIYFGMKAPMMQTADGQIEESYSISA
GLDFPSVGPQHAYLNSIGRADYVSITDDEALEAFKTLCRHEGIIPALESSHALAHALKMMRE
QPEKEQLLVVNLSGRGDKDIFTVHDILKARGEI
10000000000000000000000000000000000000000000000000000000000000000000000000000000000
00000000000000000000000000000000000000000000000000000000000000000000000000000000000
00000000000000000000000000000000000000000000000000000000000000000000000000000000000
00000000000000000000000000000000000000000000000000000000000000000000000000000000000
00000000000000000000000000000000000000000000000000000000000011111
>P38698_2qb7_0_396
MSPLRKTVPEFLAHLKSLPISKIASNDVLTICVGNESADMDSIASAITYSYCQYIYNEGTYSEE
KKKGSFIVPIIDIPREDLSLRRDVMYVLEKLKIKEEELFFIEDLKSLKQNVSQGTELNSYLVDN
NDTPKNLKNYIDNVVGIIDHHFDLQKHLDAEPRIVKVSGSCSSLVFNYWYEKLQGDREVV
MNIAPLLMGAILIDTSNMRRKVEESDKLAIERCQAVLSGAVNEVSAQGLEDSSEFYKEIKSR
KNDIKGFSVSDILKKDYKQFNFQGKGHKGLEIGLSSIVKRMSWLFNEHGGEADFVNQCRRF
QAERGLDVLVLLTSWRKAGDSHRELVILGDSNVVRELIERVSDKLQLQLFGGNLDGGVAMF
KQLNVEATRKQVVPYLEEAYSNLEE
11100000000000000000000000000000000000000000000000000000000000000000000000000000000
00000000000000000000000000000000000000000000000000000000000000000000000000000000000
00000000000000000000000000000000000000000000000000000000000000000000000000000000000
00000000000000000000000000000000000000000000000000000000000000000000000000000000000
00000000000000000000000000000000000000000000000000000000000000000
>P72044_3cai_1_397
AYDVARVRGLHPSLGDGWVHFDAPAGMLIPDSVATTVSTAFRRSGASTVGAHPSARRSAA
VLDAAREAVADLVNADPGGVVLGADRAVLLSLLAEASSRAGLGYEVIVSRLDDEANIAP
WLRAAHRYGAKVKWAEVDIETGELPTWQWESLISKSTRLVAVNSASGTLGGVTDLRAMTK
LVHDVGALVVVDHSAAAPYRLLDIRETDADVVTVNAHAWGGPPIGAMVFRDPSVMNSFG
SVSTNPYATGPARLEIGVHQFGLLAGVVASIEYLAALDESARGSRRERLAVSMQSADAYLNR
VFDYLMVSLRSLPLVMLIGRPEAQIPVVSFAVHKVPADRVVQRLADNGILAIANTGSRVLDV
LGVNDVGGAVTVGLAHYSTMAEVDQLVRALASLG
10000000000000000000000000000000000000000000000000000000000000000000000000000000000
00000000000000000000000000000000000000000000000000000000000000000000000000000000000
00000000000000000000000000000000000000000000000000000000000000000000000000000000000
00000000000000000000000000000000000000000000000000000000000000000000000000000000000
00000000000000000000000000000000000000000000000000000000000000000
>Q8SD31_4gbf_374_770
AAGYNANWDDVQLKIDTLPVIVDDTSEYDRMELIQGVTAGFHAYAGFNSWWDCTIVRDD
CVVHPKSPANPYAVIPERLGYAQESWVSHRYGQYWVENGVAKSACIDETKVDEMIPIPVEW
TAPIDGNIPSSIWANKTSLYMLTGKFIFSSTGESAIFEHQDLYRCVKGGTSELLVPAANKPWAI
FTNTEDTYPGEMTVVVNIGPASSADYVYTAYGIPSFISAFNDFVNNTIKPLNHVIDSMSIGCT
HIIMHSIDPLVAPEDYTSESSKVHVMEIIRNGNDTSFMVISPLWFDGRGNDVTANVNSNPIG
GVSGLYTHYTVMYGDGQIAFFGNNDNGQCDVDDHAGPYIQLAAGHNFTVTVNTLNQV
MFWGDSPDNSLLWNGRGTRVKHIEPTPPTGP
11111111111111111111111000000000000000000000000000000000000000000000000000000000000
00000000000000000000000000000000000000000000000000000000000000000000000000000000000
00000000000000000000000000000000000000000000000000000000000000000000000000000000000
00000000000000000000000000000000000000000000000000000000000000000000000000000000000
00000000000000000000000000000000000000000000000000000000000001111
>Q9K6Z0_3od1_0_396
MSKPFMFEKPFGMRDTLPEWYKTKKNICDQMTEEINLWGYDMIETPTLEYYETVGVVSAIL

DQQLFKLLDQQGNTLVLRPDMTAPIARLVASSLKDRAYPLRLAYQSNVYRAQQNEGGKPA
EFEQLGVELIGDGTASADGEVIALMIAALKRAGLSEFKVAIGHVGYVNALLMDVVGNEQR
ADRLRRFLYEKNYVGYREHVKSLNLSTIDKSRLMNLLSLRGGRAAIEEARGLIQTEKGKTAL
AEMTKLYEVLESYGASEYVKFDLTLVLHMSYYTGVVFEGYGNRLGVPLCSGGRYDELLSKF
HRPAQATGFGVRIDLLVEALNGEIISNGHEQTCILFSNERRFEAIELARKKRANGEAVVLQD
LAGVTDVDAMSSNYQDVIYCIGTAGRGGEDA
00000000000000000000000000000000000000000000000000000000000000000000000000000000000
00000000000000000000000000000000110000000000000000000000000000000000000000000000000
00000000000000000000000000000000000000000000000000000000000000000000000000000000000
00000000000000000000000000000000000000000000000000000000000000000000000000001111100
00000000000000000000000000000000000000000000000000000000001111111
>A5F8C0_4dth_699_1094
NAQPNLGRSTKATPDFPTHFPKSSIGIENELAGLVVAMPANSAQKFGYVKSAQGDALFMLT
KDMNQGSYQRPPSLQDGKNYQNWQTHTVELVSYPCEMDDKAAVETRKQAMLWLATHFT
THIDQSNHQPLAPIQSEDGRFVIEITNAKHVIAAGNGISAESQGQTITMTPSGQQATVGVAA
KGFGTSATPELRLLESAPWYQKSLKSQFASLTSAENLDDKELAANVFAYLTSIYLKTAELAKK
FGIYINEWDPMSEQITPNANGLTDPKVKNAWEILPRTKPSKIVEILSKSDAKAVMKHIKPQL
QSRYSESLSKNVFQYFQDGGEVAGHGINNATVGDKHSPELAILFEFRTVPNELQSYLPKTES
TTKSEVKLLDQFDPMKRKTVIQQVESLV
11111111111000000000000000000000000000000000000000000000000000000000000000000000000
00000000000000000000000000000000000000000000000000000000000000000000000000000000000
00000000000000000000000000000000000000000000000000000000000000000000000000000000000
00000000000000000000000000000000000000000000000000000000000000000000000000000000000
00000000000000000000000000000000000011111111111111111111111111111
>P49424_1r7o_28_422
NHAANKSSASRADVKPVTVKLVDSQATMETRSLFAFMQEQRRHSIMFGHQHETTQGLTIT
RTDGTQSDTFNAVGDFAAVYGWDTLSIVAPKAEGDIVAQVKKAYARGGIITVSSHFDNPKT
DTQKGVWPVGTSWDQTPAVVDSLPGGAYNPVLNGYLDQVAEWANNLKDEQGRLIPVIFR
LYHENTGSWFWWGDKQSTPEQYKQLFRYSVEYLRDVKGVRNFLYAYSPNNFWDVTEANY
LERYPGDEWVDVLGFDTYGPVADNADWFRNVVANAALVARMAEARGKIPVISEIGIRAPD
IEAGLYDNQWYRKLISGLKADPDAREIAFLLVWRNAPQGVPGPNGTQVPHYWVPANRPE
NINNGTLEDFQAFYADEFTAFNRDIEQVYQRPTLIVK
11111111111110000000000000000000000000000000000000000000000000000000000000000000000
00000000000000000000000000000000000000000000000000000000000000000000000000000000000
00000000000000000000000000000000000000000000000000000000000000000000000000000000000
00000000000000000000000000000000000000000000000000000000000000000000000000000000000
011111111111111111111111111000000000000000000000000000000000111
>Q92797_3ods_0_394
MASGSGDSVTRRSVASQFFTQEEGPGIDGMTTSERVVDLLNQAALITNDSKITVLKQVQELII
NKDPTLLDNFLDEIIAFQADKSIEVRKFVIGFIEEACKRDIELLLKLIANLNMLLRDENVNVV
KKAILTMTQLYKVALQWMVKSRVISELQEACWDMVSAMAGDIILLLDSDNDGIRTHAIKF
VEGLIVTLSPRMADSEIPRRQEHDISLDRIPRDHPYIQYNVLWEEGKAALEQLLKFMVHPAIS
SINLTTALGSLANIARQRPMFMSEVIQAYETLHANLPPTLAKSQVSSVRKNLKLHLLSVLKH
PASLEFQAQITTLLVDLGTPQAEIARNMPSSKDTRKRPRDDSDSTLKKMKLEPNLGEDDED
KDLEPGPSGTSKASAQISGQSDT
11111111111111111111111111111000000000000000000000000000000000000000000000000000000
00000000000000000000000000000000000000000000000000000000000000000000000000000000000
00000000000000000000000000000000000000000000000000000000000000000000000000000000000
00000000000000000000000000000000000000000000000000000000000000000000000000000000000
000000000000000001111111111111111111111111111111111111111111111
>Q9UR30_3g02_4_397

FAKFPSSASISPNPFTVSIPDEQLDDLKTLVRLSKIAPPTYESLQADGRFGITSEWLTTMREKW
LSEFDWRPFEARLNSFPQFTTEIEGLTIHFAALFSEREDAVPIALLHGWPGSFVEFYPILQLFRE
EYTPETLPFHLVVPSLPGYTFSSGPPLDKDFGLMDNARVVDQLMKDLGFGSGYIIQGGDIGS
FVGRLLGVGFDACKAVHLNLCAMRAPPEGPSIESLSAAEKEGIARMEKFMTDGLAYAMEH
STRPSTIGHVLSSSPIALLAWIGEKYLQWVDKPLPSETILEMVSLYWLTESFPRAIHTYRETTPT
ASAPNGATMLQKELYIHKPFGFSFFPKDLCPVPRSWIATTGNLVFFRDHAEGGHFAALERPR
ELKTDLTAFVEQVWQK
00000000000000000000000000000000000000000000000000000000000000000000000000000000000
00000000000000000000000000000000000000000000000000000000000000000000000000000000000
00000000000000000000000000000000000000000000000000000000000000000000000000000000000
00000000000000000000000000000000000000000000000000000000000000000001111110000000000
00000000000000000000000000000000000000000000000000000000000011
>A7M397_4fs7_21_413
YDFVIDEITYNFTKEHGTVEVSGLREFLNLEPGDRNSSLPLVVNIPPVVTYKDNKYDVVSIGY
AAFQGCRKVTEIKIPSTVREIGEFAFENCSKLEIINIPDSVKMIGRCTFSGCYALKSILLPLMLK
SIGVEAFKGCDFKEITIPEGVTVIGDEAFATCESLEYVSLPDSMETLHNGLFSGCGKLKSIKLP
RNLKIIRDYCFAECILLENMEFPNSLYYLGDFALSKTGVKNIIIPDSFTELGKSVFYGCTDLESIS
IQNNKLRIGGSLFYNCSGLKKVIYGSVIVPEKTFYGCSSLTEVKLLDSVKFIGEEAFESCTSLVSI
DLPYLVEEIGKRSFRGCTSLSNINFPLSLRKIGANAFQGCINLKKVELPKRLEQYRYDFEDTT
KFKWIK
00000000000000000000000000000111111111100000000000000000000000000000000000000000000
00000000000000000000000000000000000000000000000000000000000000000000000000000000000
00000000000000000000000000000000000000000000000000000000000000000000000000000000000
00000000000000000000000000000000000000000000000000000000000000000000000000000000000
0000000000000000000000000000000000000000000000000000000000000
>C7H408_4gt6_27_419
ASDSPMAYTDGSYQFILNADNTATITKYTGNEHRITIPAQVTHGAYIYPVSKIGDRVFCNYKY
VLTSVQIPDTVTEIGSNAFYNCTSLKRVTIQDNKPSCVKKIGRQAFMFCSELTDIPILDSVTEI
DSEAFHHCEELDTVTIPEGVTSVADGMFSYCYSLHTVTLPDSVTAIEERAFTGTALTQIHIPA
KVTRIGTNAFSECFALSTITSDSESYPAIDNVLYEKSANGDYALIRYPSQREDPAFKIPNGVARI
ETHAFDSCAYLASVKMPDSVVSIGTGAFMNCPALQDIEFSSRITELPESVFAGCISLKSIDIPEG
ITQILDDAFAGCEQLERIAIPSSVTKIPESAFSNCTALNNIEYSGSRSQWNAISTDSGLQNLPV
APGSIDVTV
00000000000000000000000000000000000000000000000000000000000000000000000000000000000
00000000000000000000000000000000000000000000000000000000000000000000000000000000000
00000000000000000000000000000000000000000000000000000000000000000000000000000000000
00000000000000000000000000000000000000000000000000000000000000000000000000000000000
0000000000000000000000000000000000000000000000011111111111111
>O48917_1qrr_84_476
KRVMVIGGDGYCGWATALHLSKKNYEVCIVDNLVRRLFDHQLGLESLTPIASIHDRISRWK
ALTGKSIELYVGDICDFEFLAESFKSFEPDSVVHFGEQRSAPYSMIDRSRAVYTQHNNVIGTL
NVLFAIKEFGEECHLVKLGTMGEYGTPNIDIEEGYITITHNGRTDTLPYPKQASSFYHLSKVH
DSHNIAFTCKAWGIRATDLNQGVVYGVKTDETEMHEELRNRLDYDAVFGTALNRFCVQA
AVGHPLTVYGKGGQTRGYLDIRDTVQCVEIAIANPAKAGEFRVFNQFTEQFSVNELASLVT
KAGSKLGLDVKKMTVPNPRVEAEEHYYNAKHTKLMELGLEPHYLSDSLLDSLLNFAVQFK
DRVDTKQIMPSVSWKKIGVKTKSMTT
00000000000000000000000000000000000000000000000000000000000000000000000000000000000
00000000000000000000000000000000000000000000000000000000000000000000000000000000000
00000000000000000000000000000000000000000000000000000000000000000000000000000000000
00000000000000000000000000000000000000000000000000000000000000000000000000000000000
0000000000000000000000000000000000000000000000000000000000111

>P07547_1sg6_0_392
MSNPTKISILGRESIIADFGLWRNYVAKDLISDCSSTTYVLVTDTNIGSIYTPSFEEAFRKAAAE
ITPSPRLLIYNAPPGEVSKSRQTKADIEDWMLSQNPPCGRDTVVIALGGGVIGDLTGFVASTY
MRGVRYVQVPTTLLAMVDSSIGGKTAIDTPLGKNLIGAIWQPTKIYIDLEFLETLPVREFING
MAEVIKTAAISSEEEFTALEENAETILKAVRREVRPGEHRFEGIEEILKARILASARHKAYVVS
ADEREGGLRNLLNWGHSIGHAIEAILTPQILHGECVAIGMVKEAELARHLGILKGVAVSRIV
KCLAAYGLPTSLKDARIRKLTAGKHCSVDQLMFNMALDKKNDGPKKKIVLLSAIGTPYETR
ASVVANEDIRVVLAP
11000000000000000000000000000000000000000000000000000000000000000000000000000000000
00000000000000000000000000000000000000000000000000000000000000000000000000000000000
00000000000000000000000000000000000000000000000000000000000000000000000000000000000
00000001111000000000000000000000000000000000000000000000000000000000000000000000000
0000000000000000000000011111110000000000000000000000000000001
>Q72KX2_2b3f_21_413
KLEIFSWWAGDEGPALEALIRLYKQKYPGVEVINATVTGGAGVNARAVLKTRMLGGDPPD
TFQVHAGMELIGTWVVANRMEDLSALFRQEGWLQAFPKGLIDLISYKGGIWSVPVNIHRS
NVMWYLPAKLKEWGVNPPRTWDEFLATCQTLKQKGLEAPLALGENWTQQHLWESVALA
VLGPDDWNNLWNGKLKFTDPKAVRAWEVFGRVLDCANKDAAGLSWQQAVDRVVQGK
AAFNVMGDWAAGYMTTTLKLKPGTDFAWAPSPGTQGVFMMLSDSFGLPKGAKNRQNAI
NWLRLVGSKEGQDTFNPLKGSIAARLDSDPSKYNAYGQSAMRDWRSNRIVGSLVHGAVAP
ESFMSQFGTVMEIFLQTRNPQAAANAAQAIADQVGLGRLGQ
00000000000000000000000000000000000000000000000000000000000000000000000000000000000
00000000000000000000000000000000000000000000000000000000000000000000000000000000000
00000000000000000000000000000000000000000000000000000000000000000000000000000000000
00000000000000000000000000000000000000000000000000000000000000000000000000000000000
0000000000000000000000000000000000000000000000000000000000011
>Q8PIS1_2gso_39_431
ASASTPHALLLISIDGLRADMLDRGITPNLSHLAREGVRARWMAPSYPSLTFPNHYTLVTGL
RPDHHGIVHNSMRDPTLGGFWLSKSEAVGDARWWGGEPVWVGVENTGQHAATWSWPG
SEAAIKGVRPSQWRHYQKGVRLDTRVDAVRGWLATDGAQRNRLVTLYFEHVDEAGHDH
GPESRQYADAVRAVDAAIGRLLAGMQRDGTRARTNIIVVSDHGMAEVAPGHAISVEDIAP
PQIATAITDGQVIGFEPLPGQQAAAEASVLGAHDHYDCWRKAELPARWQYGSHPRIPSLVC
QMHEGWDALFPDKLAKRAQRGTRGSHGYDPALPSMRAVFLAQGPDLAQGKTLPGFDNV
DVYALMSRLLGIPAAPNDGNPATLLPALRMPPAPDAR
11110000000000000000000000000000000000000000000000000000000000000000000000000000000
00000000000000000000000000000000000000000000000000000000000000000000000000000000000
00000000000000000000000000000000000000000000000000000000000000000000000000000000000
00000000000000000000000000000000000000000000000000000000000000000000000000000000000
0000000000000000000000000000000000000000000000000000001111111
>Q9UQ80_2q8k_1_393
SGEDEQQEQTIAEDLVVTKYKMGGDIANRVLRSLVEASSSGVSVLSLCEKGDAMIMEETGKI
FKKEKEMKKGIAFPTSISVNNCVCHFSPLKSDQDYILKEGDLVKIDLGVHVDGFIANVAHTF
VVDVAQGTQVTGRKADVIKAAHLCAEAALRLVKPGNQNTQVTEAWNKVAHSFNCTPIEG
MLSHQLKQHVIDGEKTIIQNPTDQQKKDHEKAEFEVHEVYAVDVLVSSGEGKAKDAGQRT
TIYKRDPSKQYGLKMKTSRAFFSEVERRFDAMPFTLRAFEDEKKARMGVVECAKHELLQPF
NVLYEKEGEFVAQFKFTVLLMPNGPMRITSGPFEPDLYKSEMEVQDAELKALLQSSASRKTQ
KKKKKKASKTAENATSGETLEENEAGD
11111000000000000000000000000000000000000000000000000000000000000000000000000000000
00000000000000000000000000000000000000000000000000000000000000000000000000000000000
00000000000000000000000000000000000000000000000000000000000000000000000000000000000
00000000000000000000000000000000011000000000000000000000000000000000000000000000000

0000000000000000000000000000011111111111111111111111111111111
>A6LH36_3gyc_27_418
AKSEMAFAGKGEISPRAITMWDFSWLERRWPGAGYEDWDQVLDELSERGYNAIRIDAYPH
LIAENPMKKWLLKEVWNQQDWGSPDMNEVQVQPNLNLFLSKCKERDIKVGLSSWYRLD
VDEVCLKLDTPEKLADCWLTILRSIEEDGLLDTILYVDLCNEWPGDSWAPFFAKTYPNVGW
GNWYKEESLRWMKTSLEKMRQVYPDMPFLYSFDHGDVKKYEEVDCSFLDLYEHHIWMAQ
QNGGEFYKLVGYGYNRFLPDDYKNVVKNAERVYRERPGYWQKLLTDKIELMASVARKNR
RPLVTTECWGLVDYKDWPLLKWDWVKDLCELGTITAARTGMWVGVATSNFCGPQFAGM
WRDVEWHKRLTSIIRSSPLDESLTKNNEVAAKLLKRL
11111111111000000000000000000000000000000000000000000000000000000000000000000000000
00000000000000000000000000000000000000000000000000000000000000000000000000000000000
00000000000000000000000000000000000000000000000000000000000000000000000000000000000
00000000000000000000000000000000000000000000000000000000000000000000000000000000000
000000000000000000000000000000000000000000000000000000000000
>A7UY07_4ghn_29_420
NYFYNDKQIDVENFYIAELPLTPSQFEEDFKEIHQIVMENYSLYQAKHLNMDSLYQACDAR
VRQAQTTTDYGLIVQEYISALQCAHAITCYKRYTANQRVAFIEDFLFVDKPNDYLTEYGFQD
KDRIIAINGLPYKQWIEQNEKYTEASTVPHRRLRTAYDAFRSYADTLRNYTLLRGGDTLTVT
LPLKQRDYFPDNEEQTVESRILQDSIGYLTIKTMMNPVMEDFKAVYPKVKDLPYLIIDVRRN
GGGNSMNGVNICKYFIREAQPHCVSKSYIMQPEADAYKGKIYLLTDTYTLSAAESFTLDMK
ESGNVTLIGEATGGDTGNGPRPFCTKQRTYFRIPTRQPDVSSKGFPMEGIGIPPHHQVSQTV
ADFMKDEDTVLNYAVGLITEKQ
11000000000000000000000000000000000000000000000000000000000000000000000000000000000
00000000000000000000000000000000000000000000000000000000000000000000000000000000000
00000000000000000000000000000000000000000000000000000000000000000000000000000000000
00000000000000000000000000000000000000000000000000000000000000000000000000000000000
000000000000000000000000000000000000000000000000000000000011
>O75151_3pua_59_450
STLKKKRTWHKHGPGQAPDVKPVQNGSQLFIKELRSRTFPSAEDVVARVPGSQLTLGYMEE
HGFTEPILVPKKDGLGLAVPAPTFYVSDVENYVGPERSVDVTDVTKQKDCKMKLKEFVDYY
YSTNRKRVLNVTNLEFSDTRMSSFVEPPDIVKKLSWVENYWPDDALLAKPKVTKYCLICVK
DSYTDFHIDSGGASAWYHVLKGEKTFYLIRPASANISLYERWRSASNHSEMFFADQVDKCY
KCIVKQGQTLFIPSGWIYATLTPVDCLAFAGHFLHSLSVEMQMRAYEVERRLKLGSLTQFPN
FETACWYMGKHLLEAFKGSHKSGKQLPPHLVQGAKILNGAFRSWTKKQALAEHEDELPE
HFKPSQLIKDLAKEIRLSENASKAVRP
11111111111111111111000000000000000000000000000000000000000000000000000000000000000
00000000000000000000000000000000000000000000000000000000000000000000000000000000000
00000000000000000000000000000000000000000000000000000000000000000000000000000000000
00000000000000000000000000000000000000000000000000000000000000000000000000000000000
000000000000000000000000000000000000000000000000000001111111
>P31412_1u7l_0_391
MATALYTANDFILISLPQNAQPVTAPGSKTDSWFNETLIGGRAFVSDFKIPEFKIGSLDTLIVE
SEELSKVDNQIGASIGKIIEILQGLNETSTNAYRTLPINNMPVPEYLENFQWQTRKFKLDKSIK
DLITLISNESSQLDADVRATYANYNSAKTNLAAAERKKTGDLSVRSLHDIVKPEDFVLNSEH
LTTVLVAVPKSLKSDFEKSYETLSKNVVPASASVIAEDAEYVLFNVHLFKKNVQEFTTAARE
KKFIPREFNYSEELIDQLKKEHDSAASLEQSLRVQLVRLAKTAYVDVFINWFHIKALRVYVES
VLRYGLPPHFNIKIIAVPPKNLSKCKSELIDAFGFLGGNAFMKDKKGKINKQDTSLHQYASL
VDTEYEPFVMYIINL
11110000000000000000000000000000000000000000000000000000000000000000000000000000000
00000000000000000000000000000000000000000000000000000000000000000000000000000000000
00000000000000000000000000000000000000000000000000000000000000000000000000000000000

00000000000000000000000000000000000000000000000000000000000000000000000000000000
0000000000000000000000001111111111111111111111110000000000
>P33322_3u28_2_393
KEDFVIKPEAAGASTDTSEWPLLLKNFDKLLVRSGHYTPIPAGSSPLKRDLKSYISSGVINLDK
PSNPSSHEVVAWIKRILRCEKTGHSGTLDPKVTGCLIVCIDRATRLVKSQQGAGKEYVCIVRL
HDALKDEKDLGRSLENLTGALFQRPPLISAVKRQLRVRTIYESNLIEFDNKRNLGVFWASCE
AGTYMRTLCVHLGMLLGVGGHMQELRRVRSGALSENDNMVTLHDVMDAQWVYDNTR
DESYLRSIIQPLETLLVGYKRIVVKDSAVNAVCYGAKLMIPGLLRYEEGIELYDEIVLITTKGEA
IAVAIAQMSTVDLASCDHGVVASVKRCIMERDLYPRRWGLGPVAQKKKQMKADGKLDKY
GRVNENTPEQWKKEYVPLDNAEQ
11000000011111000000000000000000000000000000000000000000000000000000000000000000
00000000000000000000000000000000000000000000000000000000000000000001111100000000
00000000000000000000000000000000000000000000000000000000000000000000000000000000
00000000000000000000000000000000000000000000000000000000000000000000000000000000
0000000000000000111111111111111111111111111111111111111111
>P49903_3fd6_0_391
MSTRESFNPESYELDKSFRLTRFTELKGTGCKVPQDVLQKLLESLQENHFQEDEQFLGAVMP
RLGIGMDTCVIPLRHGGLSLVQTTDYIYPIVDDPYMMGRIACANVLSDLYAMGVTECDNM
LMLLGVSNKMTDRERDKVMPLIIQGFKDAAEEAGTSVTGGQTVLNPWIVLGGVATTVCQP
NEFIMPDNAVPGDVLVLTKPLGTQVAVAVHQWLDIPEKWNKIKLVVTQEDVELAYQEAM
MNMARLNRTAAGLMHTFNAHAATDITGFGILGHAQNLAKQQRNEVSFVIHNLPVLAKM
AAVSKACGNMFGLMHGTCPETSGGLLICLPREQAARFCAEIKSPKYGEGHQAWIIGIVEKG
NRTARIIDKPRIIEVAPQVATQNVNPTPGATS
11111000000000000000000000000000000000001111111111111000000000000000000000000000
00000000000000000000000000000000000000000000000000000000000000000000000000000000
00000000000000000000000000000000000000000000000000000000000000000000000000000000
00000000000000000000000000000000000000000000000000000000000000000000000000000000
0000000000011100000000000000000000000000000011111111111111
>A6ZKP4_3e2v_27_417
TSTATDSPLKYYDIGLNLTDPMFHGIYNGKQYHPADYVKLLERAAQRHVKNALVTGSSIAE
SQSAIELVSSVKDLSPLKLYHTIGVHPCCVNEFADASQGDKASASIDNPSMDEAYNESLYAK
VISNPSFAQGKLKELYDLMNQQAKPHDTSFRSIGEIGLDYDRFHYSSKEMQKVFFEEQLKISC
LNDKLSSYPLFLHMRSACDDFVQILERFVVGFTDEKDTFQLQKLGASSSSGFYKFHPDRKLV
VHSFTGSAIDLQKLLNLSPNIFIGVNGCSLRTEENLAVVKQIPTERLLLETDAPWCEIKRTHA
SFQYLAKYQEVRDFEYPAFKSVKKNKLADKLNAEELYMVKGRNEPCNMEQVAIVVSEVKD
VDLATLIDTTWKTTCKIFGE
11111100000000000000000000000000000000000000000000000000000000000000000000000000
00000000000111111111111111111100000000000000000000000000000000000000000000000000
00000000000000000000000000000000000000000000000000000000000000110000000000000000
00000000000000000000000000000000000000000000000000000000000000000000000000000000
000000000000000000000000000000000000000000000000000000001
>B2I5L9_3gt5_10_400
DFRSADFLRTHISDTMAFYHPRCIDSAGGFFHYFRDDGSIYNATHRHLVSSTRFVFNYAMAY
LQFGTAEYLDAVHHGLSYVRDVHRNPATGGYAWTLCDDRVEDDTNHCYGLAFVMLAYSC
GLKVGIKQAREWMDETWCLLERHFWDAEYGLYKDEADAQWNFTRYRGQNANMHMCE
AMLAAYEASGEQRYLERALVLADRITRRQAAKADGLVWEHYDMRWEVDWDYNRDNPK
HLFRPWGFQPGHQTEWAKLLLILDRYIEVEWLVPVARSLFDVAVARSWDAVRGGLCYGFAP
DGTICDDDKYFWVQAESLAAAALLATRSGDERYWQWYDRLWAYAWQHMVDHRYGAWY
RLLDGDNRKYNDEKSPAGKTDYHTMGACHEVLNVVWTKS
00000000000000000000000000000000000000000000000000000000000000000000000000000000
00000000000000000000000000000000000000000000000000000000000000000000000000000000

00000000000000000000000000000000000000000000000000000000000000000000000000000000000
00000000000000000000000000000000000000000000000000000000000000000000000000000000000
00000000000000000000000000000000000000000000000000000000011
>Q9KLD5_2xwx_23_413
HGYVSAVENGVAEGRVTLCKFAANGTGEKNTHCGAIQYEPQSVEGPDGFPVTGPRDGKIA
SAESALAAALDEQTADRWVKRPIQAGPQTFEWTFTANHVTKDWKYYITKPNWNPNQPLS
RDAFDLNPFCVVEGNMVQPPKRVSHECIVPEREGYQVILAVWDVGDTAASFYNVIDVKFD
GNGPVLPDWNPAGQIIPSMDLSIGDTVYTRVFDNDGENPAYRTELKIDSETLTKANQWSYA
LATKINQTQKQQRAGQLNGDQFVPVYGTNPIYLKEGSGLKSVEIGYQIEAPQPEYSLTVSGL
AKEYEIGEQPIQLDLTLEAQGEMSAELTVYNHHQKPLASWSQAMTDGELKSITLELSEAKA
GHHMLVSRIKDRDGNLQDQQTLDFMLVE
00000000000000000000000000000000000000000000000000000000000000000000000000000000000
00000000000000000000000000000000000000000000000000000000000000000000000000000000000
00000000000001111110000000000000000000000000000000000000000000000000000000000000000
00000000000000000000000000000000000000000000000000000000000000000000000000000000000
00000000000000000000000000000000000000000000000000000000000
>P72178_2xts_40_429
AGTPDPLITEIQPWASEFGEAVDAHPYGLPIHFESHVKRQYVEWLTESPVSSINFTPIHALEG
TITPQGCAFERHHSGAIELSKQDYRLMINGLVEKPLVFTFEDLLRFPRTTTTAFCECAANGG
MEWGGAQLEGCQYTQGMIHNMEYVGVPLSVLLAEAGVKPEGKWLYAEGADASSNGRSF
PMEKVMDDVMLAFFANGEALRKEHGYPARLVVPGWEGNMWVKWVRRLGIYDKAVESRE
ETSKYTDLMPDGRARKWTWVMDAKSVITSPSPQVPIRHGKGPLVISGLAWSGNGRITRVDV
SLDGGKNWTTARITGQALPKALTRFHLDIDWDGSEMLLQSRAVDETGYVQPTKDALRAIR
GRNNVYHNNGIQTWWVKADGEVENVEIA
10000000000000000000000000000000000000000000000000000000000000000000000000000000000
00000000000000000000000000000000000000000000000000000000000000000000000000000000000
00000000000000000000000000000000000000000000000000000000000000000000000000000000000
00000000000000000000000000000000000000000000000000000000000000000000000000000000000
0000000000000000000000000000000000000000000000000000000000
>Q8I384_3sl1_21_410
KNVSIIGSPLAAGQPLGGVQLACDDLRKLGLHNVIDVLGWKYEDIGNIDNGDNEMKQEK
KTNNYINNNDNNNDNNNDNNNDNNNNCYIPNGVIKEKKHDLSNNKMNGYVNHNFY
GNYEENNVISTNDKYKNNCYYDNIRNIKEIGIFSKNLFDTMSNELRKKNFVLNIGGDHGVA
FSSILSSLQMYQNLRVIWIDAHGDINIPETSPSGNYHGMTLAHTLGLFKKKVPYFEWSENLT
YLKPENTAIIGIRDIDAYEKIILKKCNINYYTIFDIEKNGIYNTICTALEKIDPNSNCPIHISLDID
SVDNVFAPGTGTVAKGGLNYREINLLMKILAETKRVVSMDLVEYNPSLDEVDKKVHGDSL
PILDNATKTGKLCLELIARVLGYDIV
00000000000000000000000000000000000000000000000000111111111111111111111111111111111
11111111111111111111111111111111111111111111111110000000000000000000000000000000000
00000000000000000000000000000000000000000000000000000000000000000000000000000000000
00000000000000000000000000000000000000000000000000000000000000000000000000000000000
0000000000000000000000000000000000000000000000000000000000
>Q9RET4_2v2f_263_652
SASNYPAYMDNYLKEVINQVEQETGYNLLTTGMDVYTNVDQEAQKHLWDIYNSDQYVSY
PDDDLQVASTVVDVSNGKVIAQLGARHQASNVSFGTNQAVETNRDWGSAMKPITDYAPA
IEYGVYDSTATMVNDIPYNYPGTSTPVYNWDRAYFGNITLQYALQQSRNVTAVETLNKVGL
DRAKTFLNGLGIDYPSMHYANAISSNTTESNKQYGASSEKMAAAYAAFANGGIYHKPMYI
NKVVFSDGSKKEFSDVGTRAMKETTAYMMTEMMKTVLAYGTGRGAYLPWLAQAGKTGTS
NYTDDEIEKHIKNTGYVAPDEMFVGYTRKYSMAVWTGYSNRLTPIVGDGFLVAAKVYRSMI
TYLSEGSNPEDWNIPEGLYRNGEFVFKNGAR
11110000000000000000000000000000000000000000000000000000000000000000000000000000000

00011111000000000000000000000000000000000000000000000000000000000000000000000000000
00000000000000000000000000000000000000000000000000000000000000000000000000000000000
00000000000000000000000000000000000000000000000000000111111111110000000000000000000
0000000000000000000000000000000000100000000000000000000011
>B9ZZP0_3auk_0_388
MAASRANDAPIVLLHGFTGWGREEMFGFKYWGGVRGDIEQWLNDNGYRTYTLAVGPLSS
NWDRACEAYAQLVGGTVDYGAAHAAKHGHARFGRTYLGLLPELKRGGRIHIIAHSQGGQ
TARMLVSLLENGSQEEREYAKAHNVSLSPLFEGGHHFVLSVTTIATPHDGTTLVNMVDFTD
RFFDLQKAVLEAAAVASNVPYTSQVYDFKLDQWGLRRQPGESFDHYFERLKRSPVWTSTDT
ARYDLSVSGAEKLNQWVQASPNTYYLSFATERTYRGALTGNYYPELGMNAFSAVVCAPFL
GSYRNPTLGIDDRWLENDGIVNTVSMNGPKRGSSDRIVPYDGALKKGVWNDMGTYNVD
HLEIIGVDPNPSFDIRAFYLRLAEQLASLQP
11100000000000000000000000000000000000000000000000000000000000000000000000000000000
00000000000000000000000000000000000000000000000000000000000000000000000000000000000
00000000000000000000000000000000000000000000000000000000000000000000000000000000000
00000000000000000000000000000000000000000000000000000000000000000000000000000000000
00000000000000000000000000000000000000000000000000000000
>Q74P24_4ei7_0_388
MAGNFSEIESQGNISLKFGFLGLGMGGCAIAAECANKETQIKNNKYPYRAILVNTNSQDFN
KIEIKNTGNVRKIQLEGYEQGAARNPQVGEEAFVKHETKIFEAVKQEFEDRDFIWITCGLGG
GTGTGALLKAIEMLYEHDYNFGLLLTLPRDAEALKVLENATSRIRSIAMNQEAFGSIVLIDN
AKLYRKFEEENPSALANEYTSYSNKYIADALHEINLVTSSFTPFSDTHFDASEFAQVINTPGV
LSLAKLELKSNQLDTENPLGYLTQLGNALEKGVLYDTEREELESAKKSALSIVTSPLRAGRLY
NFSFLNQMENFLKERTPYVDERPIAPYVNKHTTKKEEDIVKFYSVVAGLPLPKRVSDIIDEIT
RIKEEREQANSKKSN
11000000000000000000000000000000000000000000000000000000000000000000000000000000000
00000000000000000000000000000000000000000000000000000000000000000000000000000000000
00000000000000000000000000000000000000000000000000000000000000000000000000000000000
00000000000000000000000000000000000000000000000000000000000000000000000000000000000
00000000000000000000000000000000000000000000000000011111
>Q89FH0_1tzz_0_388
MSVRIVDVREITKPISSPIRNAYIDFTKMTTSLVAVVTDVVREGKRVVGYGFNSNGRYGQGGL
IRERFASRILEADPKKLLNEAGDNLDPDKVWAAMMINEKPGGHGERSVAVGTIDMAVWD
AVAKIAGKPLFRLLAERHGVKANPRVFVYAAGGYYYPGKGLSMLRGEMRGYLDRGYNVV
KMKIGGAPIEEDRMRIEAVLEEIGKDAQLAVDANGRFNLETGIAYAKMLRDYPLFWYEEVG
DPLDYALQAALAEFYPGPMATGENLFSHQDARNLLRYGGMRPDRDWLQFDCALSYGLCE
YQRTLEVLKTHGWSPSRCIPHGGHQMSLNIAAGLGLGGNESYPDLFQPYGGFPDGVRVEN
GHITMPDLPGIGFEGKSDLYKEMKALAE
10000000000000000111111111000000000000000000000000000000000000000000000000000000000
00000000000000000000000000000000000000000000000000000000000000000000000000000000000
00000000000000000000000000000000000000000000000000000000000000000000000000000000000
00000000000000000000000000000000000000000000000000000000000000000000000000000000000
00000000000000000000000000000000000000000000000000000000
>Q9KM65_2wka_0_388
MNKPQLPDFIQNKIDHYIENYFDINKNGKHLVLGKQASPDDIILQSNDYLALANHPLIKAR
LAKSLLEEQQSLFMSASFLQNDYDKPMIEKRLAKFTGFDECLLSQSGWNANVGLLQTICQP
NTNVYIDFFAHMSLWEGARYANAQAHPFMHNNCDHLRMLIQRHGPGIIVVDSIYSTLGTI
APLAELVNISKEFGCALLVDESHSLGTHGPNGAGLLAELGLTREVHFMTASLAKTFAYRAG
AIWCNNEVNRCVPFISYPAIFSSTLLPYEAAGLETTLEIIESADNRRQHLDRMARKLRIGLSQ
LGLTIRSESQIIGLETGDERNTEKVRDYLESNGVFGSVFCRPATSKNKNIIRLSLNSDVNDEQI
AKIIEVCSDAVNYGDFYFR

11100000000000000000000000000000000000000000000000000000000000000000000000000000000
00000000000000000000000000000000000000000000000000000000000000000000000000000000000
00000000000000000000000000000000000000000000000000000000000000000000000000000000000
00000000000000000000000000000000000000000000000000000000000000000000000000000000000
00000000000000000000000000000000000000000000000000000000
>Q9X9P9_2olo_0_388
MTESYDVVVVGGGPVGLATAWQVAERGHRVLVLERHTFFNENGGTSGAERHWRLQYTQE
DLFRLTLETLPLWRALESRCERRLIHEIGSLWFGDTDVVTNEGQISGTAAMMDKLSVRYEWL
KATDIERRFGFRGLPRDYEGFLQPDGGTIDVRGTLAALFTLAQAAGATLRAGETVTELVPD
ADGVSVTTDRGTYRAGKVVLACGPYTNDLLEPLGARLAYSVYEMAIAAYRQATPVTEAPF
WFAFQQPTPQDTNLFYGFGHNPWAPGEFVRCGPDFEVDPLDHPSAATGVADRRQMDRLS
GWLRDHLPTVDPDPVRTSTCLAVLPTDPERQFFLGTARDLMTHGEKLVVYGAGWAFKFVP
LFGRICADLAVEDSTAYDISRLAPQSAL
10000000000000000000000000000000000000000000000000000000000000000000000000000000000
00000000000000000000000000000000000000000000000000000000000000000000000000000000000
00000000000000000000000000000000000000000000000000000000000000000000000000000000000
00000000000000000000000000000000000000000000000000000000000000000000000000000000000
00000000000000000000000000000000000000000000000000000000
>A1JMA5_3pe7_0_387
MAKGKQIPLTFDTYQDASTGAQVTRLTPPDVTCHRNYFYQKCFTRDGSKLLFGGAFDGPW
NYYLLDLNTQVATQLTEGRGDNTFGGFLSPDDDALFYVKDGRNLMRVDLATLEENVVYQ
VPAEWVGYGTWVANSDCTKLVGIEIRREDWVPLTDWKKFHEFYFTKPCCRLMRVDLKTGE
STVILQENQWLGHPIYRPYDDSTVAFCHEGPHDLVDARMWLINEDGTNMRKVKTHAEGE
SCTHEFWVPDGSALVYVSYLKGSPDRFIYSADPETLENRQLTSMPACSHLMSNYDGSLMVG
DGSDAPVDVQDDSGYKIENDPFLYVFNMKNGTQHRVARHDTSWKVFEGDRQVTHPHPSF
TPDDKQILFTSDVHGKPALYLATLPESVWK
00000000000000000000000000000000000000000000000000000000000000000000000000000000000
00000000000000000000000000000000000000000000000000000000000000000000000000000000000
00000000000000000000000000000000000000000000000000000000000000000000000000000000000
00000000000000000000000000000000000000000000000000000001111111111110000000000000000
0000000000000000000000000000000000000000000000000000000
>P66799_2ff4_0_387
MAGSATVEKRLDFGLLGPLQMTIDGTPVPSGTPKQRAVLAMLVINRNRPVGVDALITALWE
EWPPSGARASIHSYVSNLRKLLGGAGIDPRVVLAAAPPGYRLSIPDNTCDLGRFVAEKTAG
VHAAAAGRFEQASRHLSAALREWRGPVLDDLRDFQFVEPFATALVEDKVLAHTAKAEAEI
ACGRASAVIAELEALTFEHPYREPLWTQLITAYYLSDRQSDALGAYRRVKTTLADDLGIDPG
PTLRALNERILRQQPLDAKKSAKTTAAGTVTVLDQRTMASGQQAVAYLHDIASGRGYPLQ
AAATRIGRLHDNDIVLDSANVSRHHAVIVDTGTNYVINDLRSSNGVHVQHERIRSAVTLN
DGDHIRICDHEFTFQISAGTHGGT
11111110000000000000000000000000000000000000000000000000000000000000000000000000000
00000000000000000000000000000000000000000000000000000000000000000000000000000000000
00000000000000000000000000000000000000000000000000000000000000000000000000000000000
00000000000000000000000000000000000000000000000000000000000000000000000000000000000
0000000000000000000000000000000000000000000000000000011
>P94182_1ykd_57_444
VTEVEQKLQIVHQTLSMLDSHGFENILQEMLQSITLKTGELLGADRTTIFLLDEEKQELWSIV
AAGEGDRSLEIRIPADKGIAGEVATFKQVVNIPFDFYHDPRSIFAQKQEKITGYRTYTMLALP
LLSEQGRLVAVVQLLNKLKPYSPPDALLAERIDNQGFTSADEQLFQEFAPSIRLILESSRSFYIA
TQKQRAAAAMMKAVKSLSQSSLDLEDTLKRVMDEAKELMNADRSTLWLIDRDRHELWTK
ITQDNGSTKELRVPIGKGFAGIVAASGQKLNIPFDLYDHPDSATAKQIDQQNGYRTCSLLCM
PVFNGDQELIGVTQLVNKKKTGEFPPYNPETWPIAPECFQASFDRNDEEFMEAFNIQAGVA

LQNAQLFATVKQQEQ
00000000000000000000000000000000000000000000000000000000000000000000000000000000000
00000000000000000000000000000000000000000000000000000000000000000000000000000000000
00000000000000000000000000000000000000000000000000000000000000000000000000000000000
00000000000000000000000000000000000000000000000000000000000000000000000000000000000
0000000000000000000000000000000000000000000000000001111
>Q9X5F2_1r0k_0_387
MSQPRTVTVLGATGSIGHSTLDLIERNLDRYQVIALTANRNVKDLADAAKRTNAKRAVIAD
PSLYNDLKEALAGSSVEAAAGADALVEAAMMGADWTMAAIIGCAGLKATLAAIRKGKTV
ALANKESLVSAGGLMIDAVREHGTTLLPVDSEHNAIFQCFPHHNRDYVRRIIITASGGPFRT
TSLAEMATVTPERAVQHPNWSMGAKISIDSATMMNKGLELIEAYHLFQIPLEKFEILVHPQS
VIHSMVEYLDGSILAQIGSPDMRTPIGHTLAWPKRMETPAESLDFTKLRQMDFEAPDYERFP
ALTLAMESIKSGGARPAVMNAANEIAVAAFLDKKIGFLDIAKIVEKTLDHYTPATPSSLEDVF
AIDNEARIQAAALMESLPA
11000000000000000000000000000000000000000000000000000000000000000000000000000000000
00000000000000000000000000000000000000000000000000000000000000000000000000000000000
00000000000000000000000000000000001110000000000000000000000000000000000000000000000
00000000000000000000000000000000000000000000000000000000000000000000000000000000000
0000000000000000000000000000000000000000000000000000001
>D2PU28_4h3v_0_386
MTNLGIGLIGYAFMGAAHSQAWRSAPRFFDLPLHPDLNVLCGRDAEAVRAAAGKLGWST
TETDWRTLLERDDVQLVDVCTPGDSHAEIAIAALEAGKHVLCEKPLANTVAEAEAMAAA
AAKAAAGGIRSMVGFTYRRVPAIALARKLVADGKIGTVRHVRAQYLQDWIADPEAPLSWR
LDKDKAGSGALGDIGAHIVDLTQFITGDRIAEVSGRLETFVKERPKPEAHSGLSGTASAERG
PVTVDDAAVFLATFRGGALGVFEATRFATGRKNAIRIEINGSKGSLAFDFEDMNLLHFYDAT
EDPETAGFRRILATEPVHPYVAGWWPPGHLLGYEHGFTHQVVDLVTAIAEGKDPEPSFADG
LQVQRVLAAVETSSTSRQWQEIPE
00000000000000000000000000000000000000000000000000000000000000000000000000000000000
00000000000000000000000000000000000000000000000000000000000000000000000000000000000
00000000000000000000000000000000000000000000000000000000000000000110000000000000000
00000000000000000000000000000000000000000000000000000000000000000000000000000000000
000000000000000000000000000000000000000000000000000000
>O75936_3o2g_0_386
MACTIQKAEALDGAHLMQILWYDEEESLYPAVWLRDNCPCSDCYLDSAKARKLLVEALDV
NIGIKGLIFDRKKVYITWPDEHYSEFQADWLKKRCFSKQARAKLQRELFFPECQYWGSELQ
LPTLDFEDVLRYDEHAYKWLSTLKKVGIVRLTGASDKPGEVSKLGKRMGFLYLTFYGHTWQ
VQDKIDANNVAYTTGKLSFHTDYPALHHPPGVQLLHCIKQTVTGGDSEIVDGFNVCQKLK
KNNPQAFQILSSTFVDFTDIGVDYCDFSVQSKHKIIELDDKGQVVRINFNNATRDTIFDVPV
ERVQPFYAALKEFVDLMNSKESKFTFKMNPGDVITFDNWRLLHGRRSYEAGTEISRHLEGA
YADWDVVMSRLRILRQRVENGN
00000000000000000000000000000000000000000000000000000000000000000000000000000000000
00000000000000000000000000000000000000000000000000000000000000000000000000000000000
00000000000000000000000000000000000000000000000000000000000000000000000000000000000
00000000000000000000000000000000000000000000000000000000000000000000000000000000000
000000000000000000000000000000000000000000000000000011
>P48582_1zb1_0_386
MKPYLFDLKLKDTEKLDWKKGLSSYLKKSYGSSQWRTFYDEKATSELDHLRNNANGELAP
SSLSEQNLKYYSFLEHLYFRLGSKGSRLKMDFTWYDAEYSSAQKGLKYTQHTLAFEKSCTLF
NIAVIFTQIARENINEDYKNSIANLTKAFSCFEYLSENFLNSPSVDLQSENTRFLANICHAEA
QELFVLKLLNDQISSKQYTLISKLSRATCNLFQKCHDFMKEIDDDVAIYGEPKWKTTVTCKL
HFYKSLSAYYHGLHLEEENRVGEAIAFLDFSMQQLISSLPFKTWLVEFIDFDGFKETLEKKQK

ELIKDNDFIYHESVPAVVQVDSIKALDAIKSPTWEKILEPYMQDVANKCDSLYRGIIPLDVYE
KESIYSEEKATLLR
000000000000000000000000000000000000000000000000000000000000000000000000000000000000
000000000000000000000000000000000000000000000000000000000000000000000000000000000000
000000000000000000000000000000000000000000000000000000000000000000000000000000000000
000000000000000000000000000000000000000000000000000000000000000000000000000000000000
000000000000000000000000000000000111111111111111111
>Q63273_3om1_19_405
VLSSLRMAAILDDQTVCGRGERLALALAREQINGIIEVPAKARVEVDIFELQRDSQYETTDT
MCQILPKGVVSVLGPSSSPASASTVSHICGEKEIPHIKVGPEETPRLQYLRFASVSLYPSNEDVS
LAVSRILKSFNYPSASLICAKAECLLRLEELVRGFLISKETLSVRMLDDSRDPTPLLKEIRDDKV
STIIIDANASISHLVLRKASELGMTSAFYKYILTTMDFPILHLDGIVEDSSNILGFSMFNTSHPF
YPEFVRSLNMSWRENCEASTYPGPALSAALMFDAVHVVVSAVRELNRSQEIGVKPLACTSA
NIWPHGTSLMNYLRMVEYDGLTGRVEFNSKGQRTNYTLRILEKSRQGHREIGVWYSNRTL
AMNATTLDI
100000000000000000000000000000000000000000000000000000000000000000000000000000000000
000000000000000000000000000000000000000000000000000000000000000000000000000000000000
000000000000000000000000000000000000000000000000000000000000000000000000000000000000
000000000000000000000000000000000000000000000000000000000000000000000000000000000000
000000000000000000000000000000000000000011111111111
>P23342_1zov_1_386
STHFDVIVVGAGSMGMAAGYYLAKQGVKTLLVDSFDPPHTNGSHHGDTRIIRHAYGEGRE
YVPFALRAQELWYELEKETHHKIFTQTGVLVYGPKGGSAFVSETMEAANIHSLEHELFEGK
QLTDRWAGVEVPDNYEAIFEPNSGVLFSENCIQAYRELAEAHGATVLTYTPVEDFEVTEDLV
TIKTAKGSYTANKLVVSMGAWNSKLLSKLDVEIPLQPYRQVVGFFECDEAKYSNNAHYPAF
MVEVENGIYYGFPSFGGSGLKIGYHSYGQQIDPDTINREFGAYPEDEANLRKFLEQYMPGA
NGELKKGAVCMYTKTPDEHFVIDLHPKYSNVAIAAGFSGHGFKFSSVVGETLAQLATTGKT
EHDISIFSLNRDALKKEAVK
000000000000000000000000000000000000000000000000000000000000000000000000000000000000
000000000000000000000000000000000000000000000000000000000000000000000000000000000000
000000000000000000000000000000000000000000000000000000000000000000000000000000000000
000000000000000000000000000000000000000000000000000000000000000000000000000000000000
00000000000000000000000000000000000000000000011111
>P32754_3isq_7_392
GAKPERGRFLHFHSVTFWVGNAKQAASFYCSKMGFEPLAYRGLETGSREVVSHVIKQGKIV
FVLSSALNPWNKEMGDHLVKHGDGVKDIAFEVEDCDYIVQKARERGAKIMREPWVEQDK
FGKVKFAVLQTYGDTTHTLVEKMNYIGQFLPGYEAPAFMDPLLPKLPKCSLEMIDHIVGNQ
PDQEMVSASEWYLKNLQFHRFWSVDDTQVHTEYSSLRSIVVANYEESIKMPINEPAPGKKK
SQIQEYVDYNGGAGVQHIALKTEDIITAIRHLRERGLEFLSVPSTYYKQLREKLKTAKIKVKE
NIDALEELKILVDYDEKGYLLQIFTKPVQDRPTLFLEVIQRHNHQGFGAGNFNSLFKAFEEE
QNLRGNLTNMETNGVVPGM
100000000000000000000000000000000000000000000000000000000000000000000000000000000000
000000000000000000000000000000000000000000000000000000000000000000000000000000000000
000000000000000000000000000000000000000000000000000000000000000000000000000000000000
000000000000000000000000000000000000000000000000000000000000000000000000000000000000
00000000000000000000000000000000000000000011111111
>Q0P823_2rij_0_385
MINTKEDFLLLIKQIEQKSGYKKPKAFGIARLDRGQLNKNKILQASFALINYEQNFGSAAIM
LEAFMQRGVEIDFNASEFVQTLKLEDIDFALSCFKPFLEEDGHQNIDLLKIIKDKFKDDEFSF
VCLFEDKEPLSVESIYLKLYLLSTKKVPLRSINLNGAFGLLSNVAWSDDKPIELEYLRANEMR
LKMSNQYPKIDFVDKFPRFLAHIIPEDNTRILESSKVRMGASLAAGTTIMPGASYVNFNAGT

TGACMVEGRISSSAIVGEGSDVGGGASILGVLSGTSGNAISVGKACLLGANSVTGIPLGDNC
IVDAGIAVLEGTKFLLKDAEELAKLNPYFNFDKEIYKGLELKGLNGLHFRQDSISGAMIVAL
NKKAVKLNEALH
00000000000000000000000000000000000000000000000000000000000000000000000000000000000
00000000000000000000000000000000000000000000000000000000000000000000000000000000000
00000000000000000000000000000000000000000000000000000000000000000000000000000000000
00000000000000000000000000000000000000000000000000000000000000000000000000000000000
000000000000000000000000000000000000000000000000111111
>Q0QLE9_2vun_0_385
MSKTIIKNIGKIVSGDIKSPVLQADTIVVEDGLIAAIGGEELMKDAGDATIIDAAGSTVTPGLL
DTHVHVSGGDYAPRQKTMDFISSALHGGVTTMISAGSPHFPGRPKDAAGTKALAITLSKSY
YNARPAGVKVHGGAVILEKGLTEEDFIEMKKEGVWIVGEVGLGTIKNPEDAAPMVEWAH
KHGFKVQMHTGGTSIPGSSTVTADDVIKTKPDVVSHINGGPTAISVQEVDRIMDETDFAMEI
VQCGNPKIADYVARRAAEKGQLGRVIFGNDAPSGTGLIPLGILRNMCQIASMSDIDPEVAV
CMATGNSTAVYGLNTGVIAPGKEADLIIMDTPLGSVAEDAMGAIAAGDIPGISVVLIDGEAV
VTKSRNTPPAKRAAKIL
10000000000000000000000000000000000000000000000000000000000000000000000000000000000
00000000000000000000000000000000000000000000000000000000000000000000000000000000000
00000000000000000000000000000000000000000000000000000000000000000000000000000000000
00000000000000000000000000000000000000000000000000000000000000000000000000000000000
000000000000000000000000000000000000000000000000000000
>Q8WZM4_1n9g_0_385
MYSVLKQSIRPRLLATHNQFRTMITAQAVLYTQHGEPKDVLFTQSFEIDDDNLAPNEVIVKT
LGSPINPSDINQIQGVYPSKPAKTTGFGTAEPAAPCGNEGLFEVIKVGSNVSSLEAGDWVIPS
HVNFGTWRTHALGNDDDFIKLPNPAQSKANGKPNGLTINQGATISVNPLTAYLMLTHYVK
LTPGKDWFIQNGGTSAVGKYASQIGKLLNFNSISVIRDRPNLDEVVASLKELGATQVITEDQ
NNSKEFGPTIKEWIKQSGGEAKLALNCVGGKSSTGIARKLNNNGLMLTYGGMSFQPVTIPT
SLYIFKNFTSAGFWVTELLKNNKELKTSTLNQIIAWYEEGKLTDAKSIETLYDGTKPLHELYQ
DGVANSKDGKQLITY
11111111111111111111110000000000000000000000000000000000000000000000000000000000000
00000000000000000000000000000000000000000000000000000000000000000000000000000000000
00000000000000000000000000000000000000000000000000000000000000000000000000000000000
00000000000000000000000000000000000000000000000000000000000000000000000000000000000
000000000000000000000000000000000000000000000000000000
>B4EEQ5_4b6z_0_383
MTLSITSNFDAGAIDVVSCERPDAIRLRVRGDNRSEFAQWFYYRLTGARGERCVMTFENAA
ECAYPSGWRNYHAVASYDRVDWFRVPTTFDGKTMTIDHTPEFDSIYYAYFEPYSEERHAAFL
GAVQQLPQASVVELGRTVEGRPMSLLTLGTPETDDAPKKKVWIIARQHPGESMAEWFVEG
LVKRLAGWGDWAGDPVARKLYDRVTFYIVPNMNPDGSVHGNLRTNAAGANLNREWMA
PDAERSPEVLAVRDAIHAIGCDMFFDIHGDEDLPYVFVAGSEMLPSFTEQQGKEQAAFIDAF
KVASPDFQTEHGYAASKYKEDALKLASKYIGHQFGCLSLTLEMPFKDNANLPDERVGWNG
ERSAALGAAMLAAILVHVDTFA
10000000000000000000000000000000000000000000000000000000000000000000000000000000000
00000000000000000000000000000000000000000000000000000000000000000000000000000000000
00000000000000000000000000000000000000000000000000000000000000000000000000000000000
00000000000000000000000000000000000000000000000000000000000000000000010000000000000
0000000000000000000000000000000000000000000000000000
>P12998_1bs0_0_383
MSWQEKINAALDARRAADALRRRYPVAQGAGRWLVADDRQYLNFSSNDYLGLSHHPQII
RAWQQGAEQFGIGSGGSGHVSGYSVVHQALEEELAEWLGYSRALLFISGFAANQAVIAAM
MAKEDRIAADRLSHASLLEAASLSPSQLRRFAHNDVTHLARLLASPCPGQQMVVTEGVFS

MDGDSAPLAEIQQVTQQHNGWLMVDDAHGTGVIGEQGRGSCWLQKVKPELLVVTFGKG
FGVSGAAVLCSSTVADYLLQFARHLIYSTSMPPAQAQALRASLAVIRSDEGDARREKLAALI
TRFRAGVQDLPFTLADSCSAIQPLIVGDNSRALQLAEKLRQQGCWVTAIRPPTVPAGTARL
RLTLTAAHEMQDIDRLLEVLHGNG
10000000000000000000000000000000000000000000000000000000000000000000000000000000000
00000000000000000000000000000000000000000000000000000000000000000000000000000000000
00000000000000000000000000000000000000000000000000000000000000000000000000000000000
00000000000000000000000000000000000000000000000000000000000000000000000000000000000
0000000000000000000000000000000000000000000000000000
>Q59KC5_3dra_0_383
MNQLLINKHEKFFNRCLIGLPSTAQSEDSNKLAIIYFCLHGLQLIQKFQFTNQELIYYRNFIIN
QFMIENNQIISFRSTHYFQKTNQKYDCPNLSSTLFALYNLLILKSPYHTIINRKKIMNFLCKCQ
VKDGINKGGFVPTLYYDEENGDYKQYGEPDLRVCYMALLIRHLMKYDANNNNNNREDS
NETDIDLISLQQFILDRININGGFSSTIMDESHLGFTFCAIASLKLLNYPLEKLKSTKEWLIHRQ
VDYPENLYPKDGNGDNYEYYRNIDIGGFNGRENKLSDTCYSWWCTGSLYNIDVNFIKLVDL
NKAEDYLLNKTQNQLFGGFGRDPDSTPDPMHSYLALASLSLWNHEKFALQEINPILTITKES
YQFFKEEIKY
10000000000000000000000000000000000000000000000000000000000000000000000000000000000
00000000000000000000000000000000000000000000000000000000000000000000000000000000000
00000000011111111111110000000000000000000000000000000000000000000000000000000000000
00000000000111111000000000000000000000000000000000000000000000000000000000000000000
0000000000000000000000000000000000000000000000000000
>Q9P8T1_2yha_505_937
VKVARPCRKIEKWTYLELKGSKANEGVPQAMTAFAEFLNRTGIPINPRFSPGMSMSVPGSE
KEFFAKVKELMSSHQFVVVLLPRKDVAIYNMVKRAADITFGVHTVCCVAEKFLSTKGQLGY
FANVGLKVNLKFGGTNHNIKTPIPLLAKGKTMVVGYDVTHPTNLAAGQSPASAPSIVGLVS
TIDQHLGQWPAMVWNNPHGQESMTEQFTDKFKTRLELWRSNPANNRSLPENILIFRDGVS
EGQFQMVIKDELPLVRAACKLVYPAGKLPRITLIVSVKTARSAHYTVLVDEIFRADYGNKAA
DTLEQLTHDMCYLFGRATKAVSICPPAYYADLVCDRARIHQKELFDALDENDSVKTDDFAR
WGNSGAVHPNLRNSMYYI
00000000000000000000000000000000000000000000000000000000000000000000000000000000000
00000000000000000000000000000000000000000000000000000000000000000000000000000000000
00000000000000000000000000000000000000000000000000000000000000000000000000000000000
00000000000000000000000000000001111110000000000000000000000000000000011111111110000
0000000000000000000000000000000000000000000000000000
>Q9ZII9_4de8_97_480
GLTNRLNATSNYSEYSLSVAVLADSEIENVTQLTSVTAPTGTDNENIQKLLADIKSSQNTDLT
VNQSSSYLAAYKSLIAGETKAIVLNSVFENIIELEYPDYASKIKKIYTKGFTKKVEAPKTSKNQ
SFNIYVSGIDTYGPISSVSRSDVNILMTVNRDTKKILLTTTPRDAYVPIADGGNNQKDKLTHA
GIYGVDSSIHTLENLYGVDINYYVRLNFTSFLKMIDLLGGVDVHNDQEFSALHGKFHFPVG
NVHLDSEQALGFVRERYSLADGDRDRGRNQQKVIVAILQKLTSTEALKNYSTIINSLQDSIQ
TNVPLETMINLVNAQLESGGNYKVNSQDLKGTGRMDLPSYAMPDSNLYVMEIDDSSLAVV
KAAIQDVMEGR
11111111111110000000000000000000000000000000000000000000000000000000000000000000000
00000000000000000000000000000000000000000000000000000000000000000000000000000000000
00000000000000000000000000000000000000000000000000000000000000000000000000000000000
00000000000000000000000000000000000000000000000000000000000000000000000000000000000
0000000000000000000000000000000000000000000000000000
>A3ZCM2_3n29_0_381
MFYEKIQTPAYILEEDKLRKNCELLASVGEKSGAKVLLALKGFAFSGAMKIVGEYLKGCTCS
GLWEAKFAKEYMDKEIHTYSPAFKEDEIGEIASLSHHIVFNSLAQFHKFQSKTQKNSLGLRC

NVEFSLAPKELYNPCGRYSRLGIRAKDFENVDLNAIEGLHFHALCEESADALEAVLKVFEEK
FGKWIGQMKWVNFGGGHHITKKGYDVEKLIALCKNFSDKYGVQVYLEPGEAVGWQTGN
LVASVVDIIENEKQIAILDTSSEAHMPDTIIMPYTSEVLNARILATRENEKISDLKENEFAYLLT
GNTCLAGDVMGEYAFDKKLKIGDKIVFLDQIHYTIVKNTTFNGIRLPNLMLLDHKNELQMI
REFSYKDYSLRN
10000000000000000000000000000000000000000000000000000000000000000000000000000000
00000000000000000000000000000000000000000000111111111000000000000000000000000000
00000000000000000000000000000000000000000000000000000000000000000000000000000000
00000000000000000000000000000000000000000000000000000000000000000000000000000000
000000000000000000000000000000000000000000000000
>P17865_2vxy_0_381
MLEFETNIDGLASIKVIGVGGGGNNAVNRMIENEVQGVEYIAVNTDAQALNLSKAEVKM
QIGAKLTRGLGAGANPEVGKKAAEESKEQIEEALKGADMVFVTAGMGGGTGTGAAPVIA
QIAKDLGALTVGVVTRPFTFEGRKRQLQAAGGISAMKEAVDTLIVIPNDRILEIVDKNTPML
EAFREADNVLRQGVQGISDLIATPGLINLDFADVKTIMSNKGSALMGIGIATGENRAAEAA
KKAISSPLLEAAIDGAQGVLMNITGGTNLSLYEVQEAADIVASASDQDVNMIFGSVINENLK
DEIVVTVIATGFIEQEKDVTKPQRPSLNQSIKTHNQSVPKREPKREEPQQQNTVSRHTSQPA
DDTLDIPTFLRNRNKRG
11111111100000000000000000000000000000000000000000000000000000000000000000000000
00000000000000000000000000000000000000000000000000000000000000000000000000000000
00000000000000000000000000000000000000000000000000000000000000000000000000000000
00000000000000000000000000000000000000000000000000000000000000000111111111111111
111111111111111111111111111111111111111111111111
>Q0S811_2rfq_0_381
MQRLDALLPTLRERAQETEDLRRIPDDSMKALQETGFFRLLQPEQWGGYQADPVLFYSAV
RKIASACGSTGWVSSIIGVHNWHLALFSQQAQEDVWGNDTDVRISSSYAPMGAGQVVDG
GYTVNGAWAWSSGCDHASWAVLGGPVIKDGRPVDFVSFLIPREDYRIDDVWNVVGLRGT
GSNTVVVEDVFVPTHRVLSFKAMSNLTAPGLERNTAPVYKMPWGTIHPTTISAPIVGMAYG
AYDAHVEHQGKRVRAAFAGEKAKDDPFAKVRIAEASSDIDAAWRQLSGNVADEYALLVA
GEEVPFELRLRARRDQVRATGRAISSIDKLFESSGATALANGTPLQRFWRDAHAGRVHAAN
DPERAYVMYGTGEFGLPITDTMV
00000000000000000000000000000000000000000000000000000000000000000000000000000000
00000000000000000000000000000000000000000000000000000000000000000000000000000000
00000000000000000000000000000000000000000000000000000000000000000000000000000000
00000000110000000000000000000000000000000000000000000000000000000000000000000000
000000000000000000000000000000000000000000000000
>Q2A1P5_4hy1_0_381
MQNYNAKSIEVLTGLDPVKKRPGMYTNIENPNHLIQEIIDNSVDEVLAGFASKINITLYEDN
SIEVADDGRGMPVDIHPEHKMSGIELIMTKLHSGGKFSNKNYTHSGGLHGVGVSVVNALS
TRLEAEIKRDGNVYHIVFEDGFKTKDLEIIDNVGKKNTGTKIRFWPNKKYFDDIKVNFKAL
KNLLEAKAILCKALTIKYSNEIKKEKLTWHFETGLKGYLDHKLEAETLPAEPFIIDNFSNGDS
YLDAVFCWCEDLSESIKNSYVNLIPTPQDGTHVTGLKNGIYDAIKAYIEKNSLSVKNIKITAN
DSFAQLNYVISVKITNPQFAGQTKEKLSNKDVTNFVATAVKDLLTIWLNQNPDEARQIVENI
SKVAQKRINAD
11111100000000000000000000000000000000000000000000000000000000000000000000000000
00000000000000000111111000000000000000000000000000000000000000000000000000000000
00000000000000000000000000000000000000000000000000000000000000000000000000000000
00000000000000000000000000000000000000000000000111110000000000000000000000000000
000000000000000000000000000000000000000000011111
>Q7Z6Z7_3h1d_3992_4373
DFDVKRKYFRQELERLDEGLRKEDMAVHVRRDHVFEDSYRELHRKSPEEMKNRLYIVFEGE

EGQDAGGLLREWYMIISREMFNPMYALFRTSPGDRVTYTINPSSHCNPNHLSYFKFVGRIVA
KAVYDNRLLECYFTRSFYKHILGKSVRYTDMESEDYHFYQGLVYLLENDVSTLGYDLTFSTE
VQEFGVCEVRDLKPNGANILVTEENKKEYVHLVCQMRMTGAIRKQLAAFLEGFYEIIPKRLI
SIFTEQELELLISGLPTIDIDDLKSNTEYHKYQSNSIQIQWFWRALRSFDQADRAKFLQFVTGT
SKVPLQGFAALEGMNGIQKFQIHRDDRSTDRLPSAHTCFNQLDLPAYESFEKLRHMLLLAI
QECSEGFGLA
00000000000000000000000000000000000000000000000000000000000000000000000000000000
00000000000000000000011111000000000000000000000000000000000000000000000000000000
00000000000000000000000000000000000000000000000000000000000000000000000000000000
00000000000000000000000000000000000000000000000000000000000000000000000000000000
000000000000000000000000000000000000000011111111
>Q83PQ0_3h2z_0_381
MKALHFGAGNIGRGFIGKLLADAGIQLTFADVNQVVLDALNARHSYQVHVVGETEQVDT
VSGVNAVSSIGDDVVDLIAQVDLVTTAVGPVVLERIAPAIAKGLVKRKEQGNESPLNIIACE
NMVRGTTQLKGHVMNALPEDAKAWVEEHVGFVDSAVDRIVPPSASATNDPLEVTVETFSE
WIVDKTQFKGALPNIPGMELTDNLMAFVERKLFTLNTGHAITAYLGKLAGHQTIRDAILDE
KIRAVVKGAMEESGAVLIKRYGFDADKHAAYIQKILGRFENPYLKDDVERVGRQPLRKLSA
GDRLIKPLLGTLEYSLPHKNLIQGIAGAMHFRSEDDPQAQELAALIADKGPQAALAQISGL
DANSEVVSEAVTAYKAMQ
00000000000000000000000000000000000000000000000000000000000000000000000000000000
00000000000000000000000000000000000000000000000000000000000000000000000000000111
11000000000000000000000000000000000000000000000000000000000000000000000000000000
00000000000000000000000000000000000000000000000000000000000000000000000000000000
000000000000000000000000000000000000000000000000
>Q9Y259_3lq3_13_394
VGGCLAKDGLQQSKCPDTTPKRRRASSLSRDAERRAYQWCREYLGGAWRRVQPEELRVYP
VSGGLSNLLFRCSLPDHLPSVGEEPREVLLRLYGAILQGVDSLVLESVMFAILAERSLGPQLY
GVFPEGRLEQYIPSRPLKTQELREPVLSAAIATKMAQFHGMEMPFTKEPHWLFGTMERYLK
QIQDLPPTGLPEMNLLEMYSLKDEMGNLRKLLESTPSPVVFCHNDIQEGNILLLSEPENADS
LMLVDFEYSSYNYRGFDIGNHFCEWVYDYTHEEWPFYKARPTDYPTQEQQLHFIRHYLAE
AKKGETLSQEEQRKLEEDLLVEVSRYALASHFFWGLWSILQASMSTIEFGYLDYAQSRFQFYF
QQKGQLTSVHSSS
11111111111111111111111111110000000000000000000000000000001111100000000000000000
00000000000000000000000000000000000000000000000000000000000000000000000000000000
00000000000000000000000000000000000000000000000000000000000000000000000011100000
00000000000000000000000000000000000000000000000000000000000000000000000000000000
000000000000000000000000000000000000000001111111
>B5CY74_4ak7_21_401
NTQTIAVDDTQNYDERKADSLGIPKGNKLSAAMKRAMKWENHDNKWFFEYKMEPLKGD
LAYEEGVVRRDPSAMLKIGDTYYVWYSKSYGPTQGFAGDIEKDKVFPWDRCDIWYATSKD
GLTWKEQGIAVKRGEKGAYDDRSVFTPEVMEWKGKYYLCYQAVKSPYTVRVKNTIGMAC
ADSPEGLWTKTDKPVLEPSDTGEWEGDEDNRFKVVSKGDFDSHKVHDPCIIPYNGKFYMY
YKGERMGEEITWGGREIKHGVAIAENPMGPYVKSEYNPISNSGHEVCVWPYKGGIASLITT
DGPEKNTLQWSPDGINFEIMSVVKGAPHAIGLNRSADAEKEPTEILRWGLTHIYNSSDYQSI
MRFSTWTLQTHTAKGESKERK
11111111000000000000000000000000000000000000000000000000000000000000000000000000
00000000000000000000000000000000000000000000000000000000000000000000000000000000
00000000000000000000000000000000000000000000000000000000000000000000000000000000
00000000000000000000000000000000000000000000000000000000000000000000000000000000
00000000000000000000000000000000000000000000000
>Q6Q0L3_4ind_0_380

MSVTTLGQSFPANAKVKYYYKLSEKQDLDAFVNSIFVGSYKLKQISYLLYGNTKIVSAPVVP
LGPNASIIIDDELQEGLYLIRIKVYNTNSFSVTVTPFFNNNNTMTYSIGANSEFEIYDIFTKEQG
NIYYIQLPPGLAILEFSLERVFEKGNRINIPKIIHTSGNGYISFRLRKGTYAIKMPYSYNNTTSTT
FTNFQFGTISTSVATIPLVISSIPANGSGSGTFLVYLKITGDYEDVKFSVTYGGGLGVPFTFGLEV
EEINELVENTNFVTQSVTLSGSQVTQSILNVQGSGSHLRLKYASVSGLTTAVTQCQLQATNL
NRSTTYSTVWDFIAGGSSTPPSWDIREINSIQLVANGGSSTSSVTITLILVYEQIAGELS
11111000000000000000000000000000000000000000000000000000000000000000000000000000000
00000000000000000000000000000000000000000000000000000000000000000000000000000000000
00000000000000000000000000000000000000010000000000000000000000000000000000000000000
00000000000000000000000000000000000000000000000000000000000000000000000000000000000
0000000000000000000000000000000000000000000000000
>D6D1V7_4ad4_0_379
MMIFFLSLSLESCSKEDDNNPSNSENNGGNNNLGTELDYDTFCFYYDWYGSEAIDGQYRH
WAHAIAPDPNGGSGQNPGTIPGTQESIASNFYPQLGRYSSSDPNILTKHMDMFVMARTGV
LALTWWNEQDETEAKRIGLILDAADKKKIKVCFHLEPYPSRNVQNLRENIVKLITRYGNHP
AFYRKDGKPLFFIYDSYLIEPSEWEKLLSPGGSITIRNTAYDALMIGLWTSSPTVQRPFILNAH
FDGFYTYFAATGFTYGSTPTNWVSMQKWAKENGKIFIPSVGPGYIDTRIRPWNGSVIRTRTD
GQYYDAMYRKAIEAGVSAISITSFNEWHEGSQIEPAVPYTSSEFTYLDYENREPDYYLTRTAY
WVGKFRESKQ
11111111111111111111111111111100000000000000000000000000000000000000000000000000000
00000000000000000000000000000000000000000000000000000000000000000000000000000000000
00000000000000000000000000000000000000000000000000000000000000000000000000000000000
00000000000000000000000000000000000000000000000000000000000000000000000000000000000
000000000000000000000000000000000000000000000001
>Q09472_3biy_1286_1665
KFSAKRLPSTRLGTFLENRVNDFLRRQNHPESGEVTVRVVHASDKTVEVKPGMKARFVDS
GEMAESFPYRTKALFAFEEIDGVDLCFFGMHVQEYGSDCPPPNQRRVYISYLDSVHFFRPKC
LRTAVYHEILIGYLEYVKKLGYTTGHIWACPPSEGDDYIFHCHPPDQKIPKPKRLQEWYKKM
LDKAVSERIVHDYKDIFKQATEDRLTSAKELPYFEGDFWPNVLEESIKELEQEEEERKREENT
SNESTDVTKGDSKNAKKKNNKKTSKNKSSLSRGNKKKPGMPNVSNDLSQKLYATMEKHK
EVFFVIRLIAGPAANSLPPIVDPDPLIPCDLMDGRDAFLTLARDKHLEFSSLRRAQWSTMCM
LVELHTQSQDRF
00000000000000000000000000000000000000000000000000000000000000000000000000000000000
00000000000000000000000000000000000000000000000000000000000000000000000000000000000
00000000000000000000000000000000000000000000000000000000000000000001111111111111111
11111111111111111111111111111111111111111111100000000000000000000000000000000000000
000000000000000000000000000000000000000000000011
>Q8NFH3_4i79_0_379
MEEIYAKFVSQKISKTRWRPLPPGSLQTAETFATGSWDNEENYISLWSIGDFGNLDSDGGFE
GDHQLLCDIRHHGDVMDLQFFDQERIVAASSTGCVTVFLHHPNNQTLSVNQQWTTAHY
HTGPGSPSYSSAPCTGVVCNNPEIVTVGEDGRINLFRADHKEAVRTIDNADSSTLHAVTFLR
TPEILTVNSIGQLKIWDFRQQGNEPSQILSLTGDRVPLHCVDRHPNQQHVVATGGQDGMLS
IWDVRQGTMPVSLLKAHEAEMWEVHFHPSNPEHLFTCSEDGSLWHWDASTDVPEKSSLF
HQGGRSSTFLSHSISNQANVHQSVISSWLSTDPAKDRIEITSLLPSRSLSVNTLDVLGPCLVCG
TDAEAIYVTRHLFS
11100000000000000000000000000000000000000000000011111111100000000000000000000000
00000000000000000000000000000000000000011111100000000000000000000000000000000000000
00000000000000000000000000000000000000000000000000000000000000000000000000000000000
00000000000000000000000000000000000000000001111111111111111111111111111111111110000
111111000000000000000000000000000000000000000000
>Q9JYL2_1vgy_1_380

TETQSLELAKELISRPSVTPDDRDCQKLLAERLHKIGFAAEELHFGDTKNIWLRRGTKAPVV
CFAGHTDVVPTGPVEKWDSPPFEPAERDGRLYGRGAADMKTSIACFVTACERFVAKHPNH
QGSIALLITSDEEGDALDGTTKVVDVLKARDELIDYCIVGEPTAVDKLGDMIKNGRRGSLSG
NLTVKGKQGHIAYPHLAINPVHTFAPALLELTQEVWDEGNEYFPPTSFQISNINGGTGATN
VIPGELNVKFNFRFSTESTEAGLKQRVHAILDKHGVQYDLQWSCSGQPFLTQAGKLTDVAR
AAIAETCGIEAELSTTGGTSDGRFIKAIAQELIELGPSNATIHQINENVRLNDIPKLSAVYEGIL
ARLLAGNAV
00000000000000000000000000000000000000000000000000000000000000000000000000000000
00000000000000000000000000000000000000000000000000000000000000000000000000000000
00000000000000000000000000000000000000000000000000000000000000000000000000000000
00000000000000000000000000000000000000000000000000000000000000000000000000000000
0000000000000000000000000000000000000000011111
>Q9Y3R4_2f24_0_379
MASLPVLQKESVFQSGAHAYRIPALLYLPGQQSLLAFAEQRASKKDEHAELIVLRRGDYDA
PTHQVQWQAQEVVAQARLDGHRSMNPCPLYDAQTGTLFLFFIAIPGQVTEQQQLQTRAN
VTRLCQVTSTDHGRTWSSPRDLTDAAIGPAYREWSTFAVGPGHCLQLHDRARSLVVPAYAY
RKLHPIQRPIPSAFCFLSHDHGRTWARGHFVAQDTLECQVAEVETGEQRVVTLNARSHLRA
RVQAQSTNDGLDFQESQLVKKLVEPPPQGCQGSVISFPSPRSGPGSPAQWLLYTHPTHSWQ
RADLGAYLNPRPPAPEAWSEPVLLAKGSCAYSDLQSMGTGPDGSPLFGCLYEANDYEEIVFL
MFTLKQAFPAEYLPQ
11000000000000000000000000000000000000001111100000000000000000000000000000000000
00000000000000000000000000000000000000000000000000000000000000000000000000000000
00000000000000000000000000000000000000000000000000000000111100000000000000000000
00000000000000000000000000000000011111000000000000000000000000000000000000000000
0000000000000000000000000000000000000000000111
>Q988D3_3alj_0_378
MANVNKTPGKTRRAEVAGGGFAGLTAAIALKQNGWDVRLHEKSSELRAFGAGIYLWHN
GLRVLEGLGALDDVLQGSHTPPTYETWMHNKSVSKETFNGLPWRIMTRSHLHDALVNRA
RALGVDISVNSEAVAADPVGRLTLQTGEVLEADLIVGADGVGSKVRDSIGFKQDRWVSKD
GLIRLIVPRMKKELGHGEWDNTIDMWNFWPRVQRILYSPCNENELYLGLMAPAADPRGSS
VPIDLEVWVEMFPFLEPCLIEAAKLKTARYDKYETTKLDSWTRGKVALVGDAAHAMCPAL
AQGAGCAMVNAFSLSQDLEEGSSVEDALVAWETRIRPITDRCQALSGDYAANRSLSKGNM
FTPAALEAARYDPLRRVYSWPQ
11111111110000000000000000000000000000000000000000000000000000000000000000000000
00000000000000000000000000000000000000000000000000000000000000000000000000000000
00000000000000000000000000000000000000000000000000000000000000000000000000000000
00000000000000000000000000000000000000000000000000000000000000000000000000000000
000000000000000000000000000000000000000000000
>O88917_4dlq_459_836
PAPSTRRPPAPNLHVSPELFCEPREVRRVQWPATQQGMLVERPCPKGTRGIASFQCLPALGL
WNPRGPDLSNCTSPWVNQVAQKIKSGENAANIASELARHTRGSIYAGDVSSSVKLMEQLL
DILDAQLQALRPIERESAGKNYNKMHKRERTCKDYIKAVVETVDNLLRPEALESWKDMN
ATEQVHTATMLLDVLEEGAFLLADNVREPARFLAAKQNVVLEVTVLSTEGQVQELVFPQE
YASESSIQLSANTIKQNSRNGVVKVVFILYNNLGLFLSTENATVKLAGEAGTGGPGGASLVV
NSQVIAASINKESSRVFLMDPVIFTVAHLEAKNHFNANCSFWNYSERSMLGYWSTQGCRLV
ESNKTHTTCACSHL
11111111111111000000000000000000000000000000000000000000000000000000000000000000
00000000000000000000000000000000000000000000000011111111110000000000000000000000
00000000000000000000000000000000000000000000000000000000000000000000000000000000
00000000000000000000000000000000000000000000000000000000000000000000000000000000
00000000000000000000000000000000000000000000

>Q470B9_3iup_0_377
MHSALQLRSRIKSSGELELSLDSIDTPHPGPDEVLIRIEASPLNPSDLGLLFGAADMSTAKAS
GTAERPIVTARVPEGAMRSMAGRLDASMPVGNEGAGVVVEAGSSPAAQALMGKTVAAIG
GAMYSQYRCIPADQCLVLPEGATPADGASSFVNPLTALGMVETMRLEGHSALVHTAAASN
LGQMLNQICLKDGIKLVNIVRKQEQADLLKAQGAVHVCNAASPTFMQDLTEALVSTGATI
AFDATGGGKLGGQILTCMEAALNKSAREYSRYGSTTHKQVYLYGGLDTSPTEFNRNFGMA
WGMGGWLLFPFLQKIGRERANALKQRVVAELKTTFASHYSKEISLAEVLDLDMIAVYNKR
ATGEKYLINPNKGLAG
10000000000000000000000000000000000000000000000000000000000000000000000000000000000
00000000000000000000000000000000000000000000000000000000000000000000000000000000000
00000000000000000000000000000000000000000000000000000000000000000000000000000000000
00000000000000000000000000000000000000000000000000000000000000000000000000000000000
0000000000000000000000000000000000000000000001
>O59493_2eju_4_380
ELIEVQEGKAKILIPKAESIYDSPVFYNPRMALNRDIVVVLLNILNPKIVLDALSATGIRGIRFA
LETPAEEVWLNDISEDAYELMKRNVMLNFDGELRESKGRAILKGEKTIVINHDDANRLMA
ERHRYFHFIDLDPFGSPMEFLDTALRSAKRRGILGVTATDGAPLCGAHPRACLRKYLAVPLR
GELCHEVGTRILVGVIARYAAKYDLGIDVILAYYKDHYFRAFVKLKDGARKGDETLEKLGYI
YFDDKTGKFELEQGFLPTRPNAYGPVWLGPLKDEKIVSKMVKEAESLSLARKKQALKLLK
MIDQELDIPLFYDTHAIGRRLKIETKKVEEIISALREQGYEATRTHFSPTGIKTSAPYEVFIETIK
RI
10000000000000000111100000000000000000000000000000000000000000000000000000000000000
00000000000000000000000000000000000000000000000000000000000000000000000000000000000
00000000000000000000000000000000000000000000000000000000000000000000000000000000000
00000000000000000000000000000000000000000000000000000000000000000000000000000000000
000000000000000000000000000000000000000000001
>P20507_3b1b_0_376
MARTGALLLVALALAGCAQACIYKFGTSPDSKATVSGDHWDHGLNGENWEGKDGAGN
AWVCKTGRKQSPINVPQYQVLDGKGSKIANGLQTQWSYPDLMSNGTSVQVINNGHTIQV
QWTYNYAGHATIAIPAMHNQTNRIVDVLEMRPNDAADRVTAVPTQFHFHSTSEHLLAGKI
YPLELHIVHQVTEKLEACKGGCFSVTGILFQLDNGPDNELLEPIFANMPSREGTFSNLPAGT
TIKLGELLPSDRDYVTYEGSLTTPPCSEGLLWHVMTQPQRISFGQWNRYRLAVGLKECNSTE
TAADAGHHHHHRRLLHNHAHLEEVPAATSEPKHYFRRVMLAESANPDAYTCKAVAFGQ
NFRNPQYANGRTIKLARYH
11111111111111111111000000000000000000000000000000000000000000000000000000000000000
00000000000000000000000000000000000000000000000000000000000000000000000000000000000
00000000000000000000000000000000000000000000000000000000000000000000000000000000000
00000000000000000000000000000000000000000000000000111111111111111111111111111111111
111111111111000000000000000000000000000000000
>Q64W45_3slr_1_377
YTFKELKRATKQDTVSLPKLKVALLGDTATQLLATAIKGEGILRNYNIELWEAEYNQVERQI
MDPTSDYYQFEPDYTIIFHSTHKLLEKHSLVNSDLQNKLADDRLDFVRLLCEQGIGRVIYYN
YPEIEDTIWGSYATKVQSSFTYQLTKLNYELMNISQAYPNFFICNLAGISAKYGRNFMFDSSV
YVNTEIILSLDALPIISSRTIDIIAAIQGKFKKCLILDLDNTIWGGVVGDDGWENIQVGHGLGI
GKAFTEFQEWVKKLKNRGIIIAVCSKNNEGKAKEPFERNPEMVLKLDDIAVFVANWENKA
DNIRTIQRTLNIGFDSMVFLDDNPFERNMVREHVPGVTVPELPEDPGDYLEYLYTLNLFETA
SFSE
00000000000000000000000000000000000000000000000000000000000000000000000000000000000
00000000000000000000000000000000000000000000000000000000000000000000000000000000000
00000000000000000000000000000000000000000000000000000000000000000000000000000000000
00000000000000000000000000000000000000000000000000000000000000000000000000000000000

00000000000000000000000000000000000000000011
>Q93VR3_2c5e_0_376
MGTTNGTDYGAYTYKELEREQYWPSENLKISITGAGGFIASHIARRLKHEGHYVIASDWKK
NEHMTEDMFCDEFHLVDLRVMENCLKVTEGVDHVFNLAADMGGMGFIQSNHSVIMYN
NTMISFNMIEAARINGIKRFFYASSACIYPEFKQLETTNVSLKESDAWPAEPQDAYGLEKLAT
EELCKHYNKDFGIECRIGRFHNIYGPFGTWKGGREKAPAAFCRKAQTSTDRFEMWGDGLQ
TRSFTFIDECVEGVLRLTKSDFREPVNIGSDEMVSMNEMAEMVLSFEEKKLPIHHIPGPEGVR
GRNSDNNLIKEKLGWAPNMRLKEGLRITYFWIKEQIEKEKAKGSDVSLYGSSKVVGTQAPV
QLGSLRAADGKE
11111111111000000000000000000000000000000000000000000000000000000000000000000000
00000000000000000000000000000000000000000000000000000000000000000000000000000000
00000000000000000000000000000000000000000000000000000000000000000000000000000000
00000000000000000000000000000000000000000000000000000000000000000000000000000000
00000000000000000000000000000000000000000011
>Q97YD5_2i71_0_376
MASIVFSTIGNPKGYQKVTYEIDGEKFESNVSVLALRDLLKVDKTVVILGISVADVYNCKYA
DYRSCKECIIQNSKNDLGISESYVVAPNVYQKFKGKPDHYFTYIYYHSLRILEKEGINEVFIDT
THGINYMGVLAKEAIQLAVSAYAAKSEKEVKVSLYNSDPVGKDVSDTVKLHEIEAIKISPLS
GLKYVTYQILNKDKNFFNKIFSDSVNAIPRFATALDNGLFIYLSEKDSSLHLKRLEDDLSKDP
LLTPSENEINVVYKDMKYALSHALFYVISRFSGNVDLDTLRHYAETYADKVTRAIIENEVDKI
EKYQMGSERKLLGEYMKVEGKGFDKRILYAHGGLPYAGTYVYKEKDKVYVTYGDKIDEIER
QI
00000000000000000000000000000000000000000000000000000000000000000000000000000000
00000000000000000000000000000000000000000000000000000000000000000000000000000000
00000000000000000000000000000000000000000000000000000000000000000000000000000000
00000000000000000000000000000000000000000000000000000000000000000000000000000000
0000111000000000000000000000000000000000000
>Q9VB68_2xxl_0_376
MMIASSLAVLYGIAIVSSMGVQSARADYADDCTTPDGDQGQCMPFSSCRTIEERLTEAQKA
GQKVPADYASYLQKALCGEFNGVRHFCCPSANIQHNSKVMSLFKDENFDCGNFLSQRVSN
GYEVKLSSRPWMALLRYQQFGESRFLCGGAMISERYILTAAHCVHGLQNDLYEIRLGEHRIS
TEEDCRQQGRKKKCAPPVVNVGIEKHLIHEKYDARHIMHDIALLKLNRSVPFQKHIKPICL
PITDELKEKAEQISTYFVTGWGTTENGSSSDVLLQANVPLQPRSACSQAYRRAVPLSQLCVG
GGDLQDSCKGDSGGPLQAPAQYLGEYAPKMVEFGIVSQGVVTCGQISLPGLYTNVGEYVQ
WITDTMASNGL
11111111111111111111111111000000000000000000000000000000000000000000000000000000
00000000000000000000000000000000000000000000000000000000000000000000000000000000
00000000000000000000000000000000000000000000000000000000000000000000000000000000
00000000000000000000000000000000000000000000000000000000000000000000000000000000
0000000000000000000000000000000000000000000
>P81453_3iu0_31_406
DNGAGEETKSYAETYRLTADDVANINALNESAPAASSAGPSFRAPDSDDRVTPPAEPLDRM
PDPYRPSYGRAETVVNNYIRKWQQVYSHRDGRKQQMTEEQREWLSYGCVGVTWVNSGQ
YPTNRLAFASFDEDRFKNELKNGRPRSGETRAEFEGRVAKESFDEEKGFQRAREVASVMNR
ALENAHDESAYLDNLKKELANGNDALRNEDARSPFYSALRNTPSFKERNGGNHDPSRMK
AVIYSKHFWSGQDRSSSADKRKYGDPDAFRPAPGTGLVDMSRDRNIPRSPTSPGEGFVNFD
YGWFGAQTEADADKTVWTHGNHYHAPNGSLGAMHVYESKFRNWSEGYSDFDRGAYVIT
FIPKSWNTAPDKVKQGWP
11111110000000000000000000000000111111111111110000000000000000000000000000000000
00000000000000000000000000000000000000000000000000000000000000000000000000000000
00000000000000000000000000000000000000000000000000000000000000000000000000000000

00000000000000000000000000000000000000000000000000000000000000000000000000000000000
0000000000000000000000000000000000000000000
>Q5ZWI2_4ezi_27_402
ALEHEKLVNYIALGEFSRETAEIALKKMPPLDTLTVHYDLQLYKINYKTQSPDGNLTIASGLV
AMPIHPVGQVGIISYQHGTRFERNDVPSRNNEKNYIYLAAYGNSAGYMTVMPDYLGLGDN
ELTLHPYVQAETLASSSIDMLFAAKELANRLHYPISDKLYLAGYSEGGFSTIVMFEMLAKEYP
DLPVSAVAPGSAPYGWEETMHFVMLEPGPRATAYLAYFFYSLQTYKSYWSGFDEIFAPPYNT
LIPELMDGYHAVDEILQALPQDPLLIFQPKFSNGIISKTDRNTEILKINFNHYDFKPTAPLLLV
GTKGDRDVPYAGAEMAYHSFRKYSDFVWIKSVSDALDHVQAHPFVLKEQVDFFKQFERQE
AMNK
11110000000000000000000000000000000000000000000000000000000000000000000000000000000
00000000000000000000000000000000000000000000000000000000000000000000000000000000000
00000000000000000000000000000000000000000000000000000000000000000000000000000000000
00000000000000000000000000000000000000000000000000000000000000000000000000000000000
0000000000000000000000000000000000000000001
>Q89YQ6_3ina_0_375
MLTAQTKNTQTLMPLTERVNVQADSARINQIIDGCWVAVGTNKPHAIQRDFTNLFDGKPS
YRFELKTEDNTLEGYAKGETKGRAEFSYCYATSDDFRGLPADVYQKAQITKTVYHHGKGA
CPQGSSRDYEFSVYIPSSLDSNVSTIFAQWHGMPDRTLVQTPQGEVKKLTVDEFVELEKTTFF
KKNVGHEKVARLDKQGNPVKDKNGKPVYKAGKPNGWLVEQGGYPPLAFGFSGGLFYIK
ANSDRKWLTDKDDRCNANPGKTPVMKPLTSEYKASTIAYKLPFADFPKDCWITFRVHIDW
TVYGKEAETIVKPGMLDVRMDYQEQGKKVSKHIVDNEKILIGRNDEDGYYFKFGIYRVGDS
TVPVCYNLAGYSER
11111111000000000000000000000000000000000000000000000000000000000000000000000000000
00000000000000000000000000000000000000000000000000000000000000000000000000000000000
00000000000000000000000000000000000000000000000000000000000000000000000000000000000
00000000000000000000000000000000000000000000000000000000000000000000000000000000000
0000000000000000000000000000000000000000000
>O67135_1c3p_0_374
MKKVKLIGTLDYGKYRYPKNHPLKIPRVSLLLRFLDAMNLIDEKELIKSRPATKEELLLFHTE
DYINTLMEAERCQCVPKGAREKYNIGGYENPVSYAMFTGSSLATGSTVQAIEEFLKGNVAF
NPAGGMHHAFKSRANGFCYINDPAVGIEYLRKKGFKRILYIDLDAHHCDGVQEAFYDTDQ
VFVLSLHQSPEYAFPFEKGFLEEIGEGKGKGYNLNIPLPKGLNDNEFLFALEKSLEIVKEVFEP
EVYLLQLGTDPLLEDYLSKFNLSNVAFLKAFNIVREVFGEGVYLGGGGYHPYALARAWTLI
WCELSGREVPEKLNNKAKELLKSIDFEEFDDEVDRSYMLETLKDPWRGGEVRKEVKDTLEK
AKASS
10000000000000000000000000000000000000000000000000000000000000000000000000000000000
00000000000000000000000000000000000000000000000000000000000000000000000000000000000
00000000000000000000000000000000000000000000000000000000000000000000000000000000000
00000000000000000000000000000000000000000000000000000000000000000000000000000000000
000000000000000000000000000000000000000011
>P07332_4e93_447_821
IPEVQKPLHEQLWYHGAIPRAEVAELLVHSGDFLVRESQGKQEYVLSVLWDGLPRHFIIQSL
DNLYRLEGEGFPSIPLLIDHLLSTQQPLTKKSGVVLHRAVPKDKWVLNHEDLVLGEQIGRG
NFGEVFSGRLRADNTLVAVKSCRETLPPDLKAKFLQEARILKQYSHPNIVRLIGVCTQKQPIY
IVMELVQGGDFLTFLRTEGARLRVKTLLQMVGDAAAGMEYLESKCCIHRDLAARNCLVTE
KNVLKISDFGMSREEADGVYAASGGLRQVPVKWTAPEALNYGRYSSESDVWSFGILLWETF
SLGASPYPNLSNQQTREFVEKGGRLPCPELCPDAVFRLMEQCWAYEPGQRPSFSTIYQELQSI
RKRHR
00000000000000000000000000000000000000011000000000000000000000000000000000000000000
00000000000000000000000000000000000000000000000000000000000000000000000000000000000

00000000000000000000000000000000000000000000000000000000000000000000000000000000000
00000000000111111111111110000000000000000000000000000000000000000000000000000000000
0000000000000000000000000000000000000000000
>P60010_1yag_0_374
MDSEVAALVIDNGSGMCKAGFAGDDAPRAVFPSIVGRPRHQGIMVGMGQKDSYVGDEAQ
SKRGILTLRYPIEHGIVTNWDDMEKIWHHTFYNELRVAPEEHPVLLTEAPMNPKSNREKMT
QIMFETFNVPAFYVSIQAVLSLYSSGRTTGIVLDSGDGVTHVVPIYAGFSLPHAILRIDLAGRD
LTDYLMKILSERGYSFSTTAEREIVRDIKEKLCYVALDFEQEMQTAAQSSSIEKSYELPDGQVI
TIGNERFRAPEALFHPSVLGLESAGIDQTTYNSIMKCDVDVRKELYGNIVMSGGTTMFPGIA
ERMQKEITALAPSSMKVKIIAPPERKYSVWIGGSILASLTTFQQMWISKQEYDESGPSIVHHK
CF
11100000000000000000000000000000000000000000000000000000000000000000000000000000000
00000000000000000000000000000000000000000000000000000000000000000000000000000000000
00000000000000000000000000000000000000000000000000000000000000000000000000000000000
00000000000000000000000000000000000000000000000000000000000000000000000000000000000
0000000000000000000000000000000000000000000
>Q5QX87_3llx_0_374
MKSAPDWIAHPDTPYLLIDEAKLKSNINYLKQRVESLGSHLRPHLKTLRTLEAAGYLLDSKS
APATVSTLAEAEAYAKAGYTDLLYAVGIAPAKLKRVAALRQQGINLHILLDNITQAQAVVD
YAAEFGQDFSVFIEIDSDDHRGGIKPSDSKLLTIAKTLGEHFTGLMTHAGGSYACNTEQGLK
NFAKQECDAVRIARNNLETAGIHCAITSVGSTPTAHFGEDFSDISEVRAGVYTTFDLVMKNI
GVCDFSHIAMSVVTTVIGHNKEKNWLLTDSGWMALSRDSGTAGQNRDFGYGQVCKIDGS
VLDGLCVNSTSQEHGVIELSDAYQLEDFPVGHQLRIMPNHACATAAMHPVYHVLMSDGS
HNTWQRITGW
11000000000000000000000000000000000000000000000000000000000000000000000000000000000
00000000000000000000000000000000000000000000000000000000000000000000000000000000000
00000000000000000000000000000000000000000000000000000000000000000000000000000000000
00000000000000000000000000000000000000000000000000000000000000000000000000000000000
0000000000000000000000000000000000000000000
>Q5SKW0_2zul_0_374
MSLTREAYHRLTPLPHPGGRLFIKPGARGYRDPVHDLLQKTVEPFGERALDLNPGVGWGSL
PLEGRMAVERLETSRAAFRCLTASGLQARLALPWEAAAGAYDLVVLALPAGRGTAYVQAS
LVAAARALRMGGRLYLAGDKNKGFERYFKEARALLGYGVVVRREGPYRVALLEKEKEAPP
LPSLWRAFSARILGAEYTFHHLPGVFSAGKVDPASLLLLEALQERLGPEGVRGRQVLDLGA
GYGALTLPLARMGAEVVGVEDDLASVLSLQKGLEANALKAQALHSDVDEALTEEARFDII
VTNPPFHVGGAVILDVAQAFVNVAAARLRPGGVFFLVSNPFLKYEPLLEEKFGAFQTLKVA
EYKVLFAEKRGR
11000000000000000000000000000000000000000000000000000000000000000000000000000000000
00000000000000000000000000000000000000000000000000000000000000000000000000000000000
00000000000000000000000000000000000000000000000000000000000000000000000000000000000
00000000000000000000000000000000000000000000000000000000000000000000000000000000000
0000000000000000000000000000000000000000001
>Q96LA8_4hc4_0_374
MSQPKKRKLESGGGGEGGEGTEEEDGAEREAALERPRRTKRERDQLYYECYSDVSVHEEMI
ADRVRTDAYRLGILRNWAALRGKTVLDVGAGTGILSIFCAQAGARRVYAVEASAIWQQAR
EVVRFNGLEDRVHVLPGPVETVELPEQVDAIVSEWMGYGLLHESMLSSVLHARTKWLKEG
GLLLPASAELFIAPISDQMLEWRLGFWSQVKQHYGVDMSCLEGFATRCLMGHSEIVVQGLS
GEDVLARPQRFAQLELSRAGLEQELEAGVGGRFRCSCYGSAPMHGFAIWFQVTFPGGESEK
PLVLSTSPFHPATHWKQALLYLNEPVQVEQDTDVSGEITLLPSRDNPRRLRVLLRYKVGDQE
EKTKDFAMED
11111111111111111111111111111111111111111111110000000000000000000000000000000000000

00000000000000000000000000000000000000000000000000000000000000000000000000000000
00000000000000000000000000000000000000000000000000000000000000000000000000000000
00000000000000000000000000000000000000000000000011000000000000000000000000000000
00000000000000000000000000000000000000000
>Q9KWY4_2w5v_0_374
MKLKKIVFTLIALGLFSCKTTSVLVKNEPQLKTPKNVILLISDGAGLSQISSTFYFKEGTPNYT
QFKNIGLIKTSSSREDVTDSASGATAFSCGIKTYNAAIGVADDSTAVKSIVEIAALNNIKTGVV
ATSSITHATPASFYAHALNRGLEEEIAMDMTESDLDFFAGGGLNYFTKRKDKKDVLAILKG
NQFTINTTGLTDFSSIASNRKMGFLLADEAMPTMEKGRGNFLSAATDLAIQFLSKDNSAFFI
MSEGSQIDWGGHANNASYLISEINDFDDAIGTALAFAKKDGNTLVIVTSDHETGGFTLAAK
KNKREDGSEYSDYTEIGPTFSTGGHSATLIPVFAYGPGSEEFIGIYENNEIFHKILKVTKWNQ
11111111111111111111111111111000000000000000000000000000000000000000000000000000
00000000000000000000000000000000000000000000000000000000000000000000000000000000
00000000000000000000000000000000000000000000000000000000000000000000000000000000
00000000000000000000000000000000000000000000000000000000000000000000000000000000
00000000000000000000000000000000000000000
>B9WVA1_3op7_0_373
MKLPRFGVEEWLNVHENSAIYDIAGVSISSLTLEELFALSGTNPEDFYKKLQGTKLNYGWIE
GSPAFKKSVSQLYTGVKPEQILQTNGATGANLLVLYSLIEPGDHVISLYPTYQQLYDIPKSLGA
EVDLWQIEEENGWLPDLEKLRQLIRPTTKMICINNANNPTGAVMDRTYLEELVEIASEVGA
YILSDEVYRSFSELDVPSIIEVYDKGIAVNSLSKTYSLPGIRIGWVAANHQVTDILRDYRDYTM
ICAGVFDDLVAQLALAHYQEILERNRHILEENLAILDQWIEEEPLVSYIRPAVVSTSFVKIAVD
MPMEDFCLQLLQEHGVLLVPGNRFERDGYVRLGFACEQETLIKGLEKLSQFLRRFDKEN
00000000000000000000000000000000000000000000000000000000000000000000000000000000
00000000000000000000000000000000000000000000000000000000000000000000000000000000
00000000000000000000000000000000000000000000000000000000000000000000000000000000
00000000000000000000000000000000000000000000000000000000000000000000000000000000
0000000000000000000000000000000000000111
>O77206_1vpr_865_1238
VCEKGFEAGDNKLGGALNAKHVEKYGDNFKNGMHKPEFHEDGLHKPMEVGGKKFESG
FHYLLECHELGGKNASGGYGGPLCEDPYGSEVQAMTEKLLKEADSDRTLCFNNFQDPCPQ
LTKEQVAMCKGFDYGDKTLKLPCGPLPWPAGLPEPGYVPKTNPLHGRWITVSGGQAAFIK
EAIKSGMLGAAEANKIVADTDHHQTGGMYLRINQFGDVCTVDASVAKFARAKRTWKSG
HYFYEPLVSGGNLLGVWVLPEEYRKIGFFWEMESGRCFRIERRAFPVGPYTFMRQATEVGG
KISFVFYVKVSNDPESDPIPLQSRDYTALAGRDNAPTNLGKPYPTLAKDLDYPKKRDGWLE
KNEKEMLRQRNIVSSTF
11000000000000000000000000000000000000000000000000000000000000000000000000000000
00000000000000000000000000000000000000000000000000000000000000000000000000000000
00000000000000000000000000000000000000000000000000000000000000000000000000000000
00000000000000000000000000000000000000000000000000000000000000000000000000000000
0000000000000000000011111111111111111111
>P25740_2iv7_0_373
MIVAFCLYKYFPFGGLQRDFMRIASTVAARGHHVRVYTQSWEGDCPKAFELIQVPVKSHTN
HGRNAEYYAWVQNHLKEHPADRVVGFNKMPGLDVYFAADVCYAEKVAQEKGFLYRLTSR
YRHYAAFERATFEQGKSTKLMMLTDKQIADFQKHYQTEPERFQILPPGIYPDRKYSEQIPNS
REIYRQKNGIKEQQNLLLQVGSDFGRKGVDRSIEALASLPESLRHNTLLFVVGQDKPRKFEA
LAEKLGVRSNVHFFSGRNDVSELMAAADLLLHPAYQEAAGIVLLEAITAGLPVLTTAVCGY
AHYIADANCGTVIAEPFSQEQLNEVLRKALTQSPLRMAWAENARHYADTQDLYSLPEKAA
DIITGGLDG
10000000000000000000000000000000000000000000000000000000000000000000000000000000
00000000000000000000000000000000000000000000000000000000000000000000000000000000

00000000000000000000000000000000000000000000000000000000000000000000000000000000000
00000000000000000000000000000000000000000000000000000000000000000000000000000000000
000000000000000000000000000000000000000111
>P54422_2v36_28_401
KKPPKSYDEYKQVDVGKDGMVATAHPLASEIGADVLKKGGNAIDAAVAIQFALNVTEPM
MSGIGGGGFMMVYDGKTKDTTIIDSRERAPAGATPDMFLDENGKAIPFSERVTKGTAVGVP
GTLKGLEEALDKWGTRSMKQLITPSIKLAEKGFPIDSVLAEAISDYQEKLSRTAAKDVFLPN
GEPLKEGDTLIQKDLAKTFKLIRSKGTDAFYKGKFAKTLSDTVQDFGGSMTEKDLENYDITI
DEPIWGDYQGYQIATTPPPSSGGIFLLQMLKILDHFNLSQYDVRSWEKYQLLAETMHLSYA
DRASYAGDPEFVNVPLKGLLHPDYIKERQQLINLDQVNKKPKAGDPWKYQEGSANYKQV
EQPKDKVEGQ
11111000000000000000000000000000000000000000000000000000000000000000000000000000000
00000000000000000000000000000000000000000000000000000000000000000000000000000000000
00000000000000000000000000000000000000000000000000000000000000000000000000000000000
00000000000000000000000000000000000000000000000000000000000000000000000000000000000
000000000000000000000000000000000001111111
>Q8NSR4_2qr6_0_373
MRDHVEIGIGREARRTYSLDDISVVSSRRTRSSKDVDTTWHIDAYKFDLPFMNHPSDALASP
EFVIEMGKQGGLGVINAEGLWGRHADLDEAIAKVIAAYEEGDQAAATRTLQELHAAPLDT
ELLSERIAQVRDSGEIVAVRVSPQNVREIAPIVIKAGADLLVIQGTLISAEHVNTGGEALNLKE
FIGSLDVPVIAGGVNDYTTALHMMRTGAVGIIVGGGENTNSLALGMEVSMATAIADVAAA
RRDYLDETGGRYVHIIADGSIENSGDVVKAIACGADAVVLGSPLARAEEAAGKGYFWPAV
AAHPRFPRGVVTESVDLDEAAPSLEQILHGPSTMPWGVENFEGGLKRALAKCGYTDLKSF
QKVSLHVN
00000000000000000000000000000000000000000000000000000000000000000000000000000000000
00000000000000000000000000000000000000000000000000000000000000000000000000000000000
00000000000111110000000000000000000000000000000000000000000000000000000000000000000
00000000000000000000000000000000000000000000000000000000000000000000000011110000000
000000000000000000000000000000000000000000
>O34559_1nc5_0_372
MGSMDQSIAVKSPLTYAEALANTIMNTYTVEELPPANRWHYHQGVFLCGVLRLWEATGEK
RYFEYAKAYADLLIDDNGNLLFRRDELDAIQAGLILFPLYEQTKDERYVKAAKRLRSLYGTL
NRTSEGGFWHKDGYPYQMWLDGLYMGGPFALKYANLKQETELFDQVVLQESLMRKHTK
DAKTGLFYHAWDEAKKMPWANEETGCSPEFWARSIGWYVMSLADMIEELPKKHPNRHV
WKNTLQDMIKSICRYQDKETGLWYQIVDKGDRSDNWLESSGSCLYMYAIAKGINKGYLDR
AYETTLLKAYQGLIQHKTETSEDGAFLVKDICVGTSAGFYDYYVSRERSTNDLHGAGAFILA
MTELEPLFRSAGK
11111111110000000000000000000000000000000000000000000000000000000000000000000000000
00000000000000000000000000000000000000000000000000000000000000000000000000000000000
00000000000000000000000000000000000000000000000000000000000000000000000000000000000
00000000000000000000000000000000000000000000000000000000000000000000000000000000000
00000000000000000000000000000000000000000
>Q48031_2odl_68_440
SGLQGMDVVHGTATMQVDGNKTIIRNSVDAIINWKQFNIDQNEMVQFLQENNNSAVFNR
VTSNQISQLKGILDSNGQVFLINPNGITIGKDAIINTNGFTASTLDISNENIKARNFTFEQTKD
KALAEIVNHGLITVGKDGSVNLIGGKVKNEGVISVNGGSISLLAGQKITISDIINPTITYSIAAP
ENEAVNLGDIFAKGGNINVRAATIRNQGKLSADSVSKDKSGNIVLSAKEGEAEIGGVISAQ
NQQAKGGKLMITGDKVTLKTGAVIDLSGKEGGETYLGGDERGEGKNGIQLAKKTSLEKGS
TINVSGKEKGGRAIVWGDIALIDGNINAQGSGDIAKTGGFVETSGHDLFIKDNAIVDAKEW
LLD
10000000000000000000000000000000000000000000000000000000000000000000000000000000000

00000000000000000000000000000000000000000000000000000000000000000000000000000000000
00000000000000000000000000000000000000000000000000000000000000000000000000000000000
00000000000000000000000000000000000000000000000000000000000000000000000000000000000
00000000000000000000000000000000000000000
>O95714_3kci_3949_4320
GSGTIYGWGHNHRGQLGGIEGAKVKVPTPCEALATLRPVQLIGGEQTLFAVTADGKLYATG
YGAGGRLGIGGTESVSTPTLLESIQHVFIKKVAVNSGGKHCLALSSEGEVYSWGEAEDGKLG
HGNRSPCDRPRVIESLRGIEVVDVAAGGAHSACVTAAGDLYTWGKGRYGRLGHSDSEDQL
KPKLVEALQGHRVVDIACGSGDAQTLCLTDDDTVWSWGDGDYGKLGRGGSDGCKVPMK
IDSLTGLGVVKVECGSQFSVALTKSGAVYTWGKGDYHRLGHGSDDHVRRPRQVQGLQGK
KVIAIATGSLHCVCCTEDGEVYTWGDNDEGQLGDGTTNAIQRPRLVAALQGKKVNRVAC
GSAHTLAWSTSKP
11100000000000000000000000000000000000000000000000000000000000000000000000000000000
00000000000000000000000000000000000000000000000000000000000000000000000000000000000
00000000000000000000000000000000000000000000000000000000000000000000000000000000000
00000000000000000000000000000000000000000000000000000000000000000000000000000000000
0000000000000000000000000000000000000111
>P05981_1z8g_45_416
SDQEPLYPVQVSSADARLMVFDKTEGTWRLLCSSRSNARVAGLSCEEMGFLRALTHSELDV
RTAGANGTSGFFCVDEGRLPHTQRLLEVISVCDCPRGRFLAAICQDCGRRKLPVDRIVGGR
DTSLGRWPWQVSLRYDGAHLCGGSLLSGDWVLTAAHCFPERNRVLSRWRVFAGAVAQAS
PHGLQLGVQAVVYHGGYLPFRDPNSEENSNDIALVHLSSPLPLTEYIQPVCLPAAGQALVD
GKICTVTGWGNTQYYGQQAGVLQEARVPIISNDVCNGADFYGNQIKPKMFCAGYPEGGI
DACQGDSGGPFVCEDSISRTPRWRLCGIVSWGTGCALAQKPGVYTKVSDFREWIFQAIKTH
SEASGMVTQL
11100000000000000000000000000000000000000000000000000000000000000000000000000000000
00000000000000000000000000000001110000000000000000000000000000000000000000000000000
00000000000000000000000000000000000000000000000000000000000000000000000000000000000
00000000000000000000000000000000000000000000000000000000000000000000000000000000000
0000000000000000000000000000000000000000
>P42790_1ga6_215_586
AAGTAKGHNPTEFPTIYDASSAPTAANTTVGIITIGGVSQTLQDLQQFTSANGLASVNTQTI
QTGSSNGDYSDDQQGQGEWDLDSQSIVGSAGGAVQQLLFYMADQSASGNTGLTQAFNQ
AVSDNVAKVINVSLGWCEADANADGTLQAEDRIFATAAAQGQTFSVSSGDEGVYECNNR
GYPDGSTYSVSWPASSPNVIAVGGTTLYTTSAGAYSNETVWNEGLDSNGKLWATGGGYSVY
ESKPSWQSVVSGTPGRRLLPDISFDAAQGTGALIYNYGQLQQIGGTSLASPIFVGLWARLQS
ANSNSLGFPAASFYSAISSTPSLVHDVKSGNNGYGGYGYNAGTGWDYPTGWGSLDIAKLS
AYIRSNGFGH
10000000000000000000000000000000000000000000000000000000000000000000000000000000000
00000000000000000000000000000000000000000000000000000000000000000000000000000000000
00000000000000000000000000000000000000000000000000000000000000000000000000000000000
00000000000000000000000000000000000000000000000000000000000000000000000000000000000
00000000000000000000000000000000000011
>P77774_3q7m_20_391
SLFNSEEDVVKMSPLPTVENQFTPTTAWSTSVGSGIGNFYSNLHPALADNVVYAADRAGLV
KALNADDGKEIWSVSLAEKDGWFSKEPALLSGGVTVSGGHVYIGSEKAQVYALNTSDGTV
AWQTKVAGEALSRPVVSDGLVLIHTSNGQLQALNEADGAVKWTVNLDMPSLSLRGESAPT
TAFGAAVVGGDNGRVSAVLMEQGQMIWQQRISQATGSTEIDRLSDVDTTPVVVNGVVFAL
AYNGNLTALDLRSGQIMWKRELGSVNDFIVDGNRIYLVDQNDRVMALTIDGGVTLWTQSD
LLHRLLTSPVLYNGNLVVGDSEGYLHWINVEDGRFVAQQKVDSSGFQTEPVAADGKLLIQA
KDGTVYSITR

1111111110000000000000000000000000000000000000000000000000000000000000000000000001110
00000000000000000000000000000000000000000000000000000000000000000000000000000000000
00011111000000000000000000000000000000000000000011111111110000000000000000000000000
00000000000000000000000000000000000000000000000000000000000000000000000000000000000
0000000000000000000000000000000000000000
>P93324_1fp1_0_371
MGNSYITKEDNQISATSEQTEDSACLSAMVLTTNLVYPAVLNAAIDLNLFEIIAKATPPGAF
MSPSEIASKLPASTQHSDLPNRLDRMLRLLASYSVLTSTTRTIEDGGAERVYGLSMVGKYLVP
DESRGYLASFTTFLCYPALLQVWMNFKEAVVDEDIDLFKNVHGVTKYEFMGKDKKMNQIF
NKSMVDVCATEMKRMLEIYTGFEGISTLVDVGGGSGRNLELIISKYPLIKGINFDLPQVIENA
PPLSGIEHVGGDMFASVPQGDAMILKAVCHNWSDEKCIEFLSNCHKALSPNGKVIIVEFILP
EEPNTSEESKLVSTLDNLMFITVGGRERTEKQYEKLSKLSGFSKFQVACRAFNSLGVMEFYK
11111111111111111000000000000000000000000000000000000000000000000000000000000000000
00000000000000000000000000000000000000000000000000000000000000000000000000001111111
11111110000000000000000000000000000000000000000000000000000000000000000000000000000
00000000000000000000000000000000000000000000000000000000000000000000000000000000000
0000000000000000000000000000000000000000
>Q83BZ9_3tqt_0_371
MAEKLHISVLCGGQSTEHEISIQSAKNIVNTLDAAKYLISVIFIDHVGRWYLIDQPEMFLAHS
PDHLVKEGSARPITIAFGDAAKPWQSLNGDGRRYSADCVFPMVHGTQGEDGALQGLLELL
NLPYVGANVQSSAVCMEKDLTKTVLRAGGIPVVDWHTLSPRDATEGVYQRLLDRWGTSEL
FVKAVSLGSSVATLPVKTETEFTKAVKEVFRYDDRLMVEPRIRGREIECAVLGNGAPKASLPG
EIIPHHDYYSYDAKYLDPNGATTTTSVDLSESVTKQIQQIAIDAFKMVHCSGMARVDFFVTP
NNKVLVNEINTIPGFTNISMYPKMWEASGLPCPNLLDQLIELAIDRHQEQQKLIRCYEVKA
RSL
10000000000000000000000000000000000000000000000000000000000000000000000000000000000
00000001100000000000000000000000000000000000000000000000000000000000000000000000000
00000000000000000000000000000000000000000000000000000000000000000000000000000000000
00111111111111111000000000000000000000000000000000000000000000000000000000000000000
0000000000000000000000000000000000000001
>Q84527_2nv9_0_371
MNSVVNNILKAHPHQTKSFYVSSPKIVEDLIDQWTILFPRVTPHYAVKCNNDEVLLKTMCD
KNVNFDCASSSEIKKVIQIGVSPSRIIFAHTMKTIDDLIFAKDQGVDIATFDSSFELDKIHTYHP
NCKMILRIRCDDPNATVQLGNKFGANEDEIRHLLEYAKQLDIEVIGISFHVGSGSRNPEAYY
RAIKSSKEAFNEAISVGHKPYILDIGGGLHADIDEGELSTYMSDYINDAIKDFFPEDTVTIVAE
PGRFFAEHYSVLATQVIGKRVRDGLYEYFFNESTYGGFSNVIFEKSVPTPQLLRDVPDDEEYV
PSVLYGCTCDGVDVINHNVALPELHIGDWVYFPSWGAYTNVLTTSFNGFGEYDVYYI
00000000000000000000000000000000000000000000000000000000000000000000000000000000000
00000000000000000000000000000000000000000000000000000000000000000000000000000000000
00000000000000000000000000000000000000000000000000000000100000100000000000000000000
00000000000000000000000000000000000000000000000000000000000000000000000000000000000
0000000000000000000000000000000000000000
>Q8E9K5_3eqx_0_371
MEWQAEQAYNHLPPLPLDSKLAELAETLPILKACIPARAALAELKQAGELLPNQGLLINLL
PLLEAQGSSEIENIVTTTDKLFQYAQEDSQADPMTKEALRYRTALYQGFTQLSNRPLCVTTA
LEICSTIKSVQMDVRKVPGTSLTNQATGEVIYTPPAGESVIRDLLSNWEAFLHNQDDVDPLI
KMAMAHYQFEAIHPFIDGNGRTGRVLNILYLIDQQLLSAPILYLSRYIVAHKQDYYRLLLNV
TTQQEWQPWIIFILNAVEQTAKWTTHKIAAARELIAHTTEYVRQQLPKIYSHELVQVIFEQP
YCRIQNLVESGLAKRQTASVYLKQLCDIGVLEEVQSGKEKLFVHPKFVTLMTKDSNQFSRYA
L
00000000000000000000000000000000000000000000000000000000000000000000000000000000000

00000000000000000000000000000000000000000000000000000000000000000000000000000000000
00000000000000000000000000000000000000000000000000000000000000000000000000000000000
00000000000000000000000000000000000000000000000000000000000000000000000000000000000
0000000000000000000000000000000000000011
>G0RV93_3pic_89_459
GAGGATCSALPGSITLRSNAKLNDLFTMFNGDKVTTKDKFSCRQAEMSELIQRYELGTLPG
RPSTLTASFSGNTLTINCGEAGKSISFTVTITYPSSGTAPYPAIIGYGGGSLPAPAGVAMINFNN
DNIAAQVNTGSRGQGKFYDLYGSSHSAGAMTAWAWGVSRVIDALELVPGARIDTTKIGVT
GCSRNGKGAMVAGAFEKRIVLTLPQESGAGGSACWRISDYLKSQGANIQTASEIIGEDPWFS
TTFNSYVNQVPVLPFDHHSLAALIAPRGLFVIDNNIDWLGPQSCFGCMTAAHMAWQALG
VSDHMGYSQIGAHAHCAFPSNQQSQLTAFVQKFLLGQSTNTAIFQSDFSANQSQWIDWTT
PTLS
11111111100000000000000000000000000000000000000000000000000000000000000000000000000
00000000000000000000000000000000000000000000000000000000000000000000000000000000000
00000000000000000000000000000000000000000000000000000000000000000000000000000000000
00000000000000000000000000000000000000000000000000000000000000000000000000000000000
000000000000000000000000000000000000000
>P0C0J0_1dki_27_397
DQNFARNEKEAKDSAITFIQKSAAIKAGARSAEDIKLDKVNLGGELSGSNMYVYNISTGGF
VIVSGDKRSPEILGYSTSGSFDANGKENIASFMESYVEQIKENKKLDTTYAGTAEIKQPVVKS
LLDSKGIHYNQGNPYNLLTPVIEKVKPGEQSFVGQHAATGCVATATAQIMKYHNYPNKGL
KDYTYTLSSNNPYFNHPKNLFAAISTRQYNWNNILPTYSGRESNVQKMAISELMADVGISV
DMDYGPSSGSAGSSRVQRALKENFGYNQSVHQINRSDFSKQDWEAQIDKELSQNQPVYYQ
GVGKVGGHAFVIDGADGRNFYHVNWGWGGVSDGFFRLDALNPSALGTGGGAGGFNGY
QSAVVGIKP
11100000000000000000011111111111111100000000000000000000000000000000000000000000000
00000000000000000000000000000111111000000000000000000000000000000000000000000000000
00000000000000000000000000000000000000000000000000000000000000000000000000000000000
00000000000000000000000000000000000000000000000000000000000000000000000000000000000
000000000000000111111111110000000000000
>P26039_3ivf_0_399
MVALSLKISIGNVVKTMQFEPSTMVYDACRMIRERIPEALAGPPNDFGLFLSDDDPKKGIW
LEAGKALDYYMLRNGDTMEYRKKQRPLKIRMLDGTVKTIMVDDSKTVTDMLMTICARIGI
TNHDEYSLVRELMEEKKDDELNWLDHGRTLREQGVEEHETLLLRRKFFYSDQNVDSRDPV
QLNLLYVQARDDILNGSHPVSFDKACEFAGFQCQIQFGPHNEQKHKAGFLDLKDFLPKEY
VKQKGERKIFQAHKNCGQMSEIEAKVRYVKLARSLKTYGVSFFLVKEKMKGKNKLVPRLL
GITKECVMRVDEKTKEVIQEWSLTNIKRWAASPKSFTLDFGDYQDGYYSVQTTEGEQIAQLI
AGYIDIIL
10000000000000000000000000000000000000000000000000000000000000000000000000000000000
00000000000000000000000000000000000000000000000000111111111100000000000000000000000
00000000000000000000000000000000000000000000000000000000000000000000000000000000000
00000000000000000000000000000000000000000000000000000000000000000000000000000000000
000000000000000000000000000000000000011
>P29320_2qod_576_946
DEKRLHFGNGHLKLPGLRTYVDPHTYEDPTQAVHEFAKELDATNISIDKVVGAGEFGEVCS
GRLKLPSKKEISVAIKTLKVGYTEKQRRDFLGEASIMGQFDHPNIIRLEGVVTKSKPVMIVTE
YMENGSLDSFLRKHDAQFTVIQLVGMLRGIASGMKYLSDMGYVHRDLAARNILINSNLVC
KVSDFGLSRVLEDDPEAAYTTRGGKIPIRWTSPEAIAYRKFTSASDVWSYGIVLWEVMSYGER
PYWEMSNQDVIKAVDEGYRLPPPMDCPAALYQLMLDCWQKDRNNRPKFEQIVSILDKLIR
NPGSLKIITSAAARPSNLLLDQSNVDITTFRTTGDWLNGVWTAHCKEIFTGVEYSSCDTIAKI
S

11111111111111111100000111111111000000000000000000000000000000000000000000000000000
00000000000000000000000000000000000000000000000000000000000000000000000000000000000
00000000000000000000000111111111111111111111000000000000000000000000000000000000000
00000000000000000000000000000000000000000000000000000000000000000001111110000001111
111111111111111111111111111111111111111
>C0JV69_4fsd_0_369
MPCSCASGCQKSKNGGSTPSIRDHVADYYGKTLQSSADLKTSACKLAAAVPESHRKILADI
ADEVLEKFYGCGSTLPADGSLEGATVLDLGCGTGRDVYLASKLVGEHGKVIGVDMLDNQL
EVARKYVEYHAEKFFGSPSRSNVRFLKGFIENLATAEPEGVPDSSVDIVISNCVCNLSTNKLA
LFKEIHRVLRDGGELYFSDVYADRRLSEAAQQDPILYGECLGGALYLEDFRRLVAEAGFRDV
RLVSVGPVDVSDPQLRKLVPDVQFYSCTFRCFKVATLEATREDYGQSATYLGGIGEEFKLDRF
FTFPREKPVRVDRNTAEIIRHSRLHQWFSVSAEQQHMGLFKANDSYALLHAPLSMQVEQLV
11111111111111111111111111111111111111111111111100000000000000000000000000000000000
00000000000000000000000000000000000000000000000000000000000000000000000000000000000
00000000000000000000000000000000000000000000000000000000000000000000000000000000000
00000000000000000000000000000000000000000000000000000000000000000000000000000000000
00000000000000000000000000000000000000
>P00791_3psg_15_384
LVKVPLVRKKSLRQNLIKNGKLKDFLKTHKHNPASKYFPEAAALIGDEPLENYLDTEYFGTI
GIGTPAQDFTVIFDTGSSNLWVPSVYCSSLACSDHNQFNPDDSSTFEATSQELSITYGTGSMT
GILGYDTVQVGGISDTNQIFGLSETEPGSFLYYAPFDGILGLAYPSISASGATPVFDNLWDQGL
VSQDLFSVYLSSNDDSGSVVLLGGIDSSYYTGSLNWVPVSVEGYWQITLDSITMDGETIACSG
GCQAIVDTGTSLLTGPTSAIANIQSDIGASENSDGEMVISCSSIDSLPDIVFTINGVQYPLSPSA
YILQDDDSCTSGFEGMDVPTSSGELWILGDVFIRQYYTVFDRANNKVGLAPVA
00000000000000000000000000000000000000000000000000000000000000000000000000000000000
00000000000000000000000000000000000000000000000000000000000000000000000000000000000
00000000000000000000000000000000001111100000000000000000000000000000000000000000000
00000000000000000000000000000000000000000000000000000000000000000000000000000000000
00000000000000000000000000000000000000
>P06993_1hz4_436_805
HEIKDIREDTMHAEFNALRAQVAINDGNPDEAERLAKLALEELPPGWFYSRIVATSVLGEV
LHCKGELTRSLALMQQTEQMARQHDVWHYALWSLIQQSEILFAQGFLQTAWETQEKAFQ
LINEQHLEQLPMHEFLVRIRAQLLWAWARLDEAEASARSGIEVLSSYQPQQQLQCLAMLIQ
CSLARGDLDNARSQLNRLENLLGNGKYHSDWISNANKVRVIYWQMTGDKAAAANWLR
HTAKPEFANNHFLQGQWRNIARAQILLGEFEPAEIVLEELNENARSLRLMSDLNRNLLLLN
QLYWQAGRKSDAQRVLLDALKLANRTGFISHFVIEGEAMAQQLRQLIQLNTLPELEQHRA
QRILREINQHH
10000000000000000000000000000000000000000000000000000000000000000000000000000000000
00000000000000000000000000000000000000000000000000000000000000000000000000000000000
00000000000000000000000000000000000000000000000000000000000000000000000000000000000
00000000000000000000000000000000000000000000000000000000000000000000000000000000000
00000000000000000000000000000000000111
>P0C796_1pp1_0_369
MPPKRRLVDDADAMEDQDLYEPPASLPKLPGKFLQYTVGGSDPHPGIGHEKDIRQNAVAL
LDQSRRDMFHTVTPSLVFLCLLIPGLHAAFVHGGVPRESYLSTPVTRGEQTVVKTAKFYGEK
TTQRDLTELEISSIFSHCCSLLIGVVIGSSSKIKAGAEQIKKRFKTMMAALNRPSHGETATLLQ
MFNPHEAIDWINGQPWVGSFVLSLLTTDFESPGKEFMDQIKLVASYAQMTTYTTIKEYLAEC
MDATLTIPVVAYEIRDFLEVSAKLKEEHADLFPFLGAIRHPDAIKLAPRSFPNLASAAFYWSK
KENPTMAGYRASTIQPGASVKETQLARYRRREISRGEDGAELSGEISAIMRMIGVTGLN
11111111111111111111111111111100000000000000000000000000000000000000000000000000000
00000000000000000000000000000000000000000000000000000000000000000000000000000000000

000000000000000000000000000000000000000000000000000000000000000000000000000000000000
000000000000000000000000000000000000000000000000000011111111000000000000000000000000
00000000000000000000000000000000000000
>P15776_3cl5_18_387
FDNPPTNVVSHLNGDWFLFGDSRSDCNHVVNTNPRNYSYMDLNPALCDSGKISSKAGNSI
FRSFHFTDFYNYTGEGQQIIFYEGVNFTPYHAFKCTTSGSNDIWMQNKGLFYTQVYKNMA
VYRSLTFVNVPYVYNGSAQSTALCKSGSLVLNNPAYIAREANFGDYYYKVEADFYLSGCDE
YIVPLCIFNGKFLSNTKYYDDSQYYFNKDTGVIYGLNSTETITTGFDFNCHYLVLPSGNYLAI
SNELLLTVPTKAICLNKRKDFTPVQVVDSRWNNARQSDNMTAVACQPPYCYFRNSTTNYV
GVYDINHGDAGFTSILSGLLYDSPCFSQQGVFRYDNVSSVWPLYSYGRCPTAADINTPDVPI
CVYD
000000000000000000000000000000000000000000000000000000000000000000000000000000000000
000000000000000000000000000000000000000000000000000000000000000000000000000000000000
000000000000000000000000000000000000000000000000000000000000000000000000000000000000
000000000000000000000000000000000000000000000000000000000000000000000000000000000000
00000000000000000000000000111111111111
>P26882_1ihg_0_369
MSHPSPQAKPSNPSNPRVFFDVDIGGERVGRIVLELFADIVPKTAENFRALCTGEKGIGPTTG
KPLHFKGCPFHRIIKKFMIQGGDFSNQNGTGGESIYGEKFEDENFHYKHDKEGLLSMANA
GSNTNGSQFFITTVPTPHLDGKHVVFGQVIKGMGVAKILENVEVKGEKPAKLCVIAECGEL
KEGDDWGIFPKDGSGDSHPDFPEDADVDLKDVDKILLISEDLKNIGNTFFKSQNWEMAIKK
YTKVLRYVEGSRAAAEDADGAKLQPVALSCVLNIGACKLKMSDWQGAVDSCLEALEIDPS
NTKALYRRAQGWQGLKEYDQALADLKKAQEIAPEDKAIQAELLKVKQKIKAQKDKEKAA
YAKMFA
100000000000000000000000000000000000000000000000000000000000000000000000000000000000
000000000000000000000000000000000000000000000000000000000000000000000000000000000000
000000000000000000000000000000000000000000000000000000000000000000000000000000000000
000000000000000000000000000000000000000000000000000000000000000000000000000000000000
00000000000000000000000000000000011111
>Q183D5_3qi7_25_394
GKKDKEKIIDDFKVAVVTQPLSENKVQYNMVEEMAKEYEEENKIDKDKDGQTKVKQTIKH
VVLPENFTSNIDSAINKIVKLADDKEVQAIVVSTDQAGLLPALQKVKEKRPEIITISAPMGDD
KNQLSQFVDVNLGVSAEERGKVLAERSKEMGAKAFIHYASTDDLKDVNIAKRLEMIKETC
KNIGLPFVQVNTPNINTEEDKNKVKQFLNEDIEKQVKKYGKDINVFGVNEYMDEVILTKAL
ELKYIVAEQSNPSPIQTYPSVMGLKISEKDAQNYDKINDMISEKAKAFGMSNRLGGYPMPM
DAFLPSLAIYLATEMVKQDLTQEDVCDPDYLEAFTELRFGIGSEFTPLTEVLYNYQSVILSQLI
Y
111111100000000000000000000000000000000000000011111000000000000000000000000000000000
000000000000000000000000000000000000000000000000000000000000000000000000000000000000
000000000000000000000000000000000000000000000000000000000000000000000000000000000000
000000000000000000000000000000000000000000000000000000000000000000000000000000000000
00000000000000000000000000000000000000
>A9CKT8_2pnw_1_369
NTPFSIDEVSFRDLPGWGQDDPRKLFPAMATILSHLRNAKPYRTGALGITAAELVSLLELAE
RGQVNSPEQARQFFETNSVPFRISPAQGKSGFVTAFYEPELEVSATPDDVWRYPIYRRPPELV
DIDNDNRPDGFDPSYAFGKADEEGISYFPDRRAIDEGCLRGRGLEIAWARSKVDLFFVHVQ
GAARLVFPDGAIKRITYAAKAGHVFSPIGRLLLDRGELDPKTISMQTIRQWLADHPDEVDG
VLWHNRSYIFFREADVAGLDMGPIAAAKVPLVAGRALAVDRLIHTFGLPFFIHAPTLTHLD
DGKPFARLMLALDTGSAIVGPARGDIFTGSGFEAGELAGTVRNEADFYILLPRIAAERYRR
111000000000000000000000000000000000000000000000000000000000000000000000000000000000
000011110000000000000000000000000000000000000000000000000000000000000000000000000000

00000000000000000000000000000000000000000000000000000000000000000000000000000000000
00000000000011111110000000000000000000000000000000000000000000000000000000000000000
0000000000000000000000000000000000001
>Q7VU70_3dme_0_368
MSTDIDCIVIGAGVVGLAIARALAAGGHEVLVAEAAEGIGTGTSSRNSEVIHAGIYYPADSL
KARLCVRGKHLLYEYCAARGVPHQRLGKLIVATSDAEASQLDSIARRAGANGVDDLQHID
GAAARRLEPALHCTAALVSPSTGIVDSHALMLAYQGDAESDGAQLVFHTPLIAGRVRPEGG
FELDFGGAEPMTLSCRVLINAAGLHAPGLARRIEGIPRDSIPPEYLCKGSYFTLAGRAPFSRLI
YPVPQHAGLGVHLTLDLGGQAKFGPDTEWIATEDYTLDPRRADVFYAAVRSYWPALPDGA
LAPGYTGIRPKISGPHEPAADFAIAGPASHGVAGLVNLYGIESPGLTASLAIAEETLARLAA
11000000000000000000000000000000000000000000000000000000000000000000000000000000000
00000000000000000000000000000000000000000000000000000000000000000000000000000000000
00000000000000000000000000000000000000000000000000000000000000000000000000000000000
00000000000000000000000000000000000000000000000000000000000000000000000000000000000
0000000000000000000000000000000000001
>A6KZ57_3s9j_26_393
AGKQNEVELVAEKQLAFPLDEQTYYLSKSMFQFEENGKEYLHFENTQKSLYDIVIFDIENQQ
IAKRIPLHKTGPNGLPAVFGSRPSPDSQYILVAQNNISRLSSINSQGEIIRNYNFQTPEGRFTPL
SFGSYYNAPAFIKDSCIFLRQEILKPDMKKEDWPRTHMFASQDLRTGEVKWIPIFYPPIFKEE
YDNIAGGYGFSYDYNYKESRLVCGFFGYDSLMVTDDLKHIRWYNAKSRYLKSMKPKLGNS
MEGINAIIKLNENPRYWHIMYDKYRNVYYRFAEMPYKLAPNESPYETPKGKEFSVIVLNAD
FEIIGETKFPGKKYFYKMSFVGREGLYISENNLENPQFDENKLVFTCFKIKNASPNK
11000000000000000000000000000000000000000000000000000000000000000000000000000000000
00000000000000000000000000000000000000000000000000000000000000000000000000000000000
00000000000000000000000000000000000000000000000000000000000000000000000000000000000
00000000000000000000000000000000000000000000000000000000000000000000000000000000000
000000000000000000000000000000001111
>P78406_3mmy_0_367
MSLFGTTSGFGTSGTSMFGSATTDNHNPMKDIEVTSSPDDSIGCLSFSPPTLPGNFLIAGSWA
NDVRCWEVQDSGQTIPKAQQMHTGPVLDVCWSDDGSKVFTASCDKTAKMWDLSSNQAI
QIAQHDAPVKTIHWIKAPNYSCVMTGSWDKTLKFWDTRSSNPMMVLQLPERCYCADVIY
PMAVVATAERGLIVYQLENQPSEFRRIESPLKHQHRCVAIFKDKQNKPTGFALGSIEGRVAIH
YINPPNPAKDNFTFKCHRSNGTNTSAPQDIYAVNGIAFHPVHGTLATVGSDGRFSFWDKD
ARTKLKTSEQLDQPISACCFNHNGNIFAYASSYDWSKGHEFYNPQKKNYIFLRNAAEELKP
RNKK
11100000000000000011110000000000000000000000000000000000000000000000000000000000000
00000000000000000000000000000000000000000000000000000000000000000000000000000000000
00000000000000000000000000000000000000000000000000000000000000000000000000000000000
00000000000000000000000000000000000000000000000000000000000000000000000000000000000
000000000000000000000000000000000111
>Q5SJ29_2dby_0_367
MLAVGIVGLPNVGKSTLFNALTRANALAANYPFATIDKNVGVVPLEDERLYALQRTFAKGE
RVPPVVPTHVEFVDIAGLVKGAHKGEGLGNQFLAHIREVAAIAHVLRCFPDPDVVHVMGR
VDPLEDAEVVETELLLADLATLERRLERLRKEARADRERLPLLEAAEGLYVHLQEGKPARTF
PPSEAVARFLKETPLLTAKPVIYVANVAEEDLPDGRGNPQVEAVRRKALEEGAEVVVVSARL
EAELAELSGEEARELLAAYGLQESGLQRLARAGYRALDLLTFFTAGEKEVRAWTVRRGTKA
PRAAGEIHSDMERGFIRAEVIPWDKLVEAGGWARAKERGWVRLEGKDYEVQDGDVIYVLF
NA
00000000000000000000000000000000000000000000000000000000000000000000000000000000000
00000000000000000000000000001111111111100000000000000000000000000000000000000000000
00000000000000000000000000000000000000000000000000000000000000000000000000000000000

00000000000000000000000000000000000000000000000000000000000000000000000000000000000
000000000000000000000000000000000011
>Q81LQ0_3h2y_0_367
MTETIKCIGCGVEIQTEDKNEVGYAPASSLEKEQVICQRCFRLKHYNEIQDVSLTDDDFLRIL
NGIGKSDALVVKIVDIFDFNGSWLPGLHRFVGNNKVLLVGNKADLIPKSVKHDKVKHWM
RYSAKQLGLKPEDVFLISAAKGQGIAELADAIEYYRGGKDVYVVGCTNVGKSTFINRMIKEF
SDETENVITTSHFPGTTLDLIDIPLDEESSLYDTPGIINHHQMAHYVGKQSLKLITPTKEIKPM
VFQLNEEQTLFFSGLARFDYVSGGRRAFTCHFSNRLTIHRTKLEKADELYKNHAGDLLSPPT
PEELENMPELVKYEFNIREPKTDVVFSGLGWVTVNEPGAKIVAHVPKGVSVSLRKSLI
11111111111111111111111111111111111111111111111111111100000000000000000000000000000
00000000000000000000000000000000000000000000000000000000000000000000000000000000000
00000000000000000000000000000000111100000000000000000000000000000000000000000000000
00000000000000000000000000000000000000000000000000000000000000000000000000000000000
000000000000000000000000000000000000
>A1S356_3fmc_0_366
MRVDKHEVRVGELAAGQPLSLPVYRFKGKGAGPSVYIQANVHGAEVQGNAVIYQLMKLL
EHYELLGDISLVPLANPLGINQKSGEFTLGRFDPITGVNWNREYLDHGFNIEVWYQEHSHL
DDDTLITAFRATLVEECARRLNNPWGVTTGHRLAVTLQSMAHRADIVLDLHTGPKSCKHL
YCPEYERSAAQYFSIPYTLLIPNSFGGAMDEAAFVPWWTLAEVASSHGRELGVRVSALTLEL
GSQERIDLDDALEDAEGILAYLSHRGVIAETVLPKPMKRYGCFLKNYRKFHAPKAGMVEYL
GKVGVPMKATDPLVNLLRLDLYGTGEELTVLRLPEDGVPILHFASASVHQGTELYKVMTKV
FEL
11000000000000000000000000000000000000000000000000000000000000000000000000000000000
00000000000000000000000000000000000000000000000000000000000000000000000000000000000
00000000000000000000000000000000000000000000000000000000000000000000000000000000000
00000000000000000000000000000000000000000000000000000000000000000000000000000000000
00000000000000000000000000000000000
>Q216E8_3qfw_1_367
TEPRIVATYHIASDAERIEQRALALAIEQSVECPLEAIGDPAIVANIVGRVEDVAELQPGRYAV
RIGLAAATAPAEPGQLLNMLFGNSSIQPDIALADVELPAHYLTAFGGPRVGLAGIRTLTGAQ
SRALTASALKPQGLSPAALASIAHQLALGGVDLIKDDHGLADQAFSPFAERAAAVGKAVRE
ANAARGGRTLYAPNISGTLDDMRRQLGVIRDEGIGAVLVAPMIVGVSNFHAIVKEAAGLVV
VAHPAMAGAAKIAAPLLLGRLFRLFGADATVFPNYGGRFAYSTASCLALAQAARDPFGKL
NACIPTPAGGIMLQRVNELLRFYGQDVMLLIGGSLLASRERLTEQASRFVNKVADYGQR
00000000000000000000000000000000000001111111000000000000000000000000000000000000000
00000000000000000000000000000000000000000000000000000000000000000000000000000000000
00000000000000000000000000000000000000000000000000000000000000000000000000000000000
00000000000000000000000000000000000000000000000000000000000000000000000000000000000
00000001111111110000000000000000000
>Q51658_4fa5_20_386
EQARPADDALAALGAQLFVDPALSRNATQSCATCHDPARAFTDPREGKAGLAVSVGDDG
QSHGDRNTPTLGYAALVPAFHRDANGKYKGGQFWDGRADDLKQQAGQPMLNPVEMA
MPDRAAVAARLRDDPAYRTGFEALFGKGVLDDPERAFDAAAEALAAYQATGEFSPFDSKY
DRVMRGEEKFTPLEEFGYTVFITWNCRLCHMQRKQGVAERETFTNFEYHNIGLPVNETARE
ASGLGADHVDHGLLARPGIEDPAQSGRFKVPSLRNVAVTGPYMHNGVFTDLRTAILFYNK
YTSRRPEAKINPETGAPWGEPEVARNLSLAELQSGLMLDDGRVDALVAFLETLTDRRYEPLL
EESRAAQKD
11111000000000000000000000000000000000000000000000000000000000000000000000000000000
00000000000000000000000000000000000000000000000000000000000000000000000000000000000
00000000000000000000000000000000000000000000000000000000000000000000000000000000000
00000000000000000000000000000000000000000000000000000000000000000000000000000000000

00000000000000000000000000000011111
>Q6ARP5_2pge_1_367
SGMELSYRRSDLIFKRPAGTSRGVLTSKPTWFVRLDIDGHGGQGEVSLIPGLSLDPEEQIGRE
LDLLARRLRAEEPIRLRQFLAERGGADFSDYRSVLTDIAGILDSWQVSTDGRFPALRFALEM
ALLDLLSGGRQEWFASDFTRGEKRIPVNGLIWMGEAAFMQEQIEAKLAEGYGCLKLKIGAI
DFDKECALLAGIRESFSPQQLEIRVDANGAFSPANAPQRLKRLSQFHLHSIEQPIRQHQWSE
MAALCANSPLAIALDEELIGLGAEQRSAMLDAIRPQYIILKPSLLGGFHYAGQWIELARERG
IGFWITSALESNLGLAAIAQWTALYQPTMPQGLGTGQLYTNNLPSNLAVDGGLLGVS
0000000000000000111111100000000000000000000000000000000000000000000000000000000000000000000000
0000000000000000000000000000000000000000000000000000000000000000000000000000000000000000000000
0000000000000000011100000000000000000000000000000000000000000000000000000000000000000000000000
0000000000000000000000000000000000000000000000000000000000000000000000000000000000000000000000
00000000000000000000000000000000001
>Q8TE04_3smp_230_596
KNRPPFPWFGMDIGGTLVKLVYFEPKDITAEEEQEEVENLKSIRKYLTSNTAYGKTGIRDVHL
ELKNLTMCGRKGNLHFIRFPSCAMHRFIQMGSEKNFSSLHTTLCATGGGAFKFEEDFRMIA
DLQLHKLDELDCLIQGLLYVDSVGFNGKPECYYFENPTNPELCQKKPYCLDNPYPMLLVN
MGSGVSILAVYSKDNYKRVTGTSLGGGTFLGLCCLLTGCETFEEALEMAAKGDSTNVDKLV
KDIYGGDYERFGLQGSAVASSFGNMMSKEKRDSISKEDLARATLVTITNNIGSIARMCALNE
NIDRVVFVGNFLRINMVSMKLLAYAMDFWSKGQLKALFLEHEGYFGAVGALLELFKMTD
D
1000000000000000000000000000000000000000000000000000000000000000000000000000000000000000000000
0000000000000000000000000000000000000000000000000000000000000000000000000000000000000000000000
0000000000000000000000000000000000000000000000000000000000000000000000000000000000000000000000
0000000000000000000000000000000000000000000000000000000000000000000000000000000000000000000000
00000000000000000000000000000000111
>P0A988_3q4l_0_365
MKFTVEREHLLKPLQQVSGPLGGRPTLPILGNLLLQVADGTLSLTGTDLEMEMVARVALVQ
PHEPGATTVPARKFFDICRGLPEGAEIAVQLEGERMLVRSGRSRFSLSTLPAADFPNLDDWQ
SEVEFTLPQATMKRLIEATQFSMAHQDVRYYLNGMLFETEGEELRTVATDGHRLAVCSMPI
GQSLPSHSVIVPRKGVIELMRMLDGGDNPLRVQIGSNNIRAHVGDFIFTSKLVDGRFPDYRR
VLPKNPDKHLEAGCDLLKQAFARAAILSNEKFRGVRLYVSENQLKITANNPEQEEAEEILD
VTYSGAEMEIGFNVSYVLDVLNALKCENVRMMLTDSVSSVQIEDAASQSAAYVVMPMRL
0000000000000000000001110000000000000000000000000000000000000000000000000000000000000000000000
0000000000000000000000000000000000000000000000000000000000000000000000000000000000000000000000
0000000000000000000000000000000000000000000000000000000000000000000000000000000000000000000000
0000000000000000000000000000000000000000000000000000000000000000000000000000000000000000000000
0000000000000000000000000000000000
>P0ADR6_4auk_0_365
MNKVVLLCRPGFEKECAAEITDKAGQREIFGFARVKENAGYVIYECYQPDDGDKLIRELPFS
SLIFARQWFVVGELLQHLPPEDRITPIVGMLQGVVEKGGELRVEVADTNESKELLKFCRKFT
VPLRAALRDAGVLANYETPKRPVVHVFFIAPGCCYTGYSYSNNNSPFYMGIPRLKFPADAP
SRSTLKLEEAFHVFIPADEWDERLANGMWAVDLGACPGGWTYQLVKRNMWVYSVDNGP
MAQSLMDTGQVTWLREDGFKFRPTRSNISWMVCDMVEKPAKVAALMAQWLVNGWCRE
TIFNLKLPMKKRYEEVSHNLAYIQAQLDEHGINAQIQARQLYHDREEVTVHVRRIWAAVG
GRRDER
0000000000000000000000000000000000000000000000000000000000000000000000000000000000000000000000
0000000000000000000000000000000000000000000000000000000000000000000000000000000000000000000000
0000000000000000000000000000000000000000000000000000000000000000000000000000000000000000000000
0000000000000000000000000000000000000000000000000000000000000000000000000000000000000000000000
0000000000000000000000000111111111

>P11741_3eoj_0_365
ALFGTKDTTTAHSDYEIILEGGSSSWGQVKGRAKVNVPAAIPLLPTDCNIRIDAKPLDAQKG
VVRFTTKIESVVDSVKNTLNVEVDIANETKDRRIAVGEGSLSVGDFSHSFSFEGQVVNMYYY
RSDAVRRNIPNPIYMQGRQFHDILMKVPLDNNDLVDTWEGFQQSISGGGANFGDWIREFW
FIGPAFAAINEGGQRISPIVVNSSNVEGGEKGPVGVTRWKFSHAGSGVVDSISRWTELFPVE
QLNKPASIEGGFRSDSQGIEVKVDGNLPGVSRDAGGGLRRILNHPLIPLVHHGMVGKFNDF
TVDTQLKIVLPKGYKIRYAAPQFRSQNLEEYRWSGGAYARWVEHVCKGGTGQFEVLYAQ
11111100000000000000000000000000000000000000000000000000000000000000000000000000000
00000000000000000000000000000000000000000000000000000000000000000000000000000000000
00000000000000000000000000000000000000000000000100000000000000000000000000000000000
00000000000000000000000000000000000000000000000000000000000000000000000000000000000
0000000000000000000000000000000000
>Q4UVI8_2f8h_0_365
MTDLLASTLEHLETLVSFDTRNPPRAIAAEGGIFDYLRAQLPGFQVEVIDHGDGAVSLYAVR
GTPKYLFNVHLDTVPDSPHWSADPHVMRRTEDRVIGLGVCDIKGAAAALVAAANAGDG
DAAFLFSSDEEANDPRCIAAFLARGLPYDAVLVAEPTMSEAVLAHRGISSVLMRFAGRAGH
ASGKQDPAASALHQAMRWGGKALDHVESLAHARFGGLTGLRFNIGRVDGGIKANMIAP
AAELRFGFRPLPSMDVDGLLATFAGFADPAAAHFEETFRGPSLPSGDIARAEERRLAARDV
ADALDLPIGNAVDFWTEASLFSAGGYTALVYGPGDIAQAHTADEFVTLAQLQRYVESVNRI
INGSH
00000000000000000000000000000000000000000000000000000000000000000000000000000000000
00000000000000000000000000000000000000000000000000000000000000000000000000000000000
00000000000000111111000000000000000000000000000000000000000000000000000000000000000
00000000000000000000000000000000000000000000000000000000000000000000000000000000000
0000000000000000000000000000000001
>Q949P3_1xfi_1_366
ESDSEMVPFPQLPMPIENNYRACTIPYRFPSDDPKKATPNEISWINVFANSIPSFKKRAESDIT
VPDAPARAEKFAERYAGILEDLKKDPESHGGPPDGILLCRLREQVLRELGFRDIFKKVKDEE
NAKAISLFPQVVSLSDAIEDDGKRLENLVRGIFAGNIFDLGSAQLAEVFSRDGMSFLASCQN
LVPRPWVIDDLENFQAKWINKSWKKAVIFVDNSGADIILGILPFARELLRRGAQVVLAANE
LPSINDITCTELTEILSQLKDENGQLLGVDTSKLLIANSGNDLPVIDLSRVSQELAYLSSDADL
VIVEGMGRGIETNLYAQFKCDSLKIGMVKHLEVAEFLGGRLYDCVFKFNEVQS
11110000000000000000000000000000000000000000000000000000000000000000000000000000000
00000000000000000000000000000000000000000000000000000000000000000000000000000000011
11111111111110000000000000000000000000000000000000000000000000000000000000000000000
00000000000000000000110000000000000000000000000000000000000000000000000000000000000
0000000000000000000000000000000011
>Q9Z4I2_3gd0_35_400
AVPATIPLTITNNSGRAEQIHIYNLGTELSSGRQGWADASGAFHPWPAGGNPPTPAPDASIP
GPAPGRSTTIQIPKFSGRIYFSYGRKMEFRLTTGGLVQPAVQNPTDPNRDILFNWSEYTLNDS
GLWINSTQVDMFSAPYTVGVRRGDGTTLSTGKLRPGGYNGVFNALRGQSGGWANLIQTRS
DGTVLRALSPLYGVETGALPASVMDDYINRVWNKYTGTDLIVTPFADRPDVRYTGRVSGGV
LRFTDGSGAVVTTFQKPDASSVFGCHRLLDAPNDQVRGPISRTLCAGFNRTTLLANPHQPD
RSAAGFYQEPVTNHYARIIHAHMADGKAYGFAFDDVGHHESLVHDGDPRGASLTLDPFD
10000000000000000000000000000000000000000000000000000000000000000000000000000000000
00000000000000000000000000000000000000000000000000000000000000000000000000000000000
00000000000000000000000000000000000000000000000000000000000000000000000000000000000
00000000000000000000000000011100000000000000000000000000000000000000000000000000000
0000000000000000000000000000000000
>P07012_1gqe_0_364
MFEINPVNNRIQDLTERSDVLRGYLDYDAKKERLEEVNAELEQPDVWNEPERAQALGKER

SSLEAVVDTLDQMKQGLEDVSGLLELAVEADDEETFNEAVAELDALEEKLAQLEFRRMFSG
EYDSADCYLDIQAGSGGTEAQDWASMLERMYLRWAESRGFKTEIIEESEGEVAGIKSVTIKIS
GDYAYGWLRTETGVHRLVRKSPFDSGGRRHTSFSSAFVYPEVDDDIDIEINPADLRIDVYRTS
GAGGQHVNRTESAVRITHIPTGIVTQCQNDRSQHKNKDQAMKQMKAKLYELEMQKKNA
EKQAMEDNKSDIGWGSQIRSYVLDDSRIKDLRTGVETRNTQAVLDGSLDQFIEASLKAGL
11100000000000000000000000000000000000000000000000000000000000000000000000000000000
00000000000000000000000000000000000000000000000000000000000000000000000000000000000
00000000000000000000000000000000000000000000000000000000000000000000000000000000000
00000000000000000000000000000000000000000000000000000000000000000000000000000000000
000000000000000000000000000000000
>P10349_1k30_31_395
HSRKFLDVRSEEELLSCIKKETEAGKLPPNVAAGMEELYQNYRNAVIESGNPKADEIVLSNM
TVALDRILLDVEDPFVFSSHHKAIREPFDYYIFGQNYIRPLIDFGNSFVGNLSLFKDIEEKLQQ
GHNVVLISNHQTEADPAIISLLLEKTNPYIAENTIFVAGDRVLADPLCKPFSIGRNLICVYSKK
HMFDIPELTETKRKANTRSLKEMALLLRGGSQLIWIAPSGGRDRPDPSTGEWYPAPFDASSV
DNMRRLIQHSDVPGHLFPLALLCHDIMPPPSQVEIEIGEKRVIAFNGAGLSVAPEISFEEIAAT
HKNPEEVREAYSKALFDSVAMQYNVLKTAISGKQGLGASTADVSLSQPW
00000000000000000000000000000000000000000000000000000000000000000000000000000000000
00000000000000000000000000000000000000000000000000000000000000000000000000000000000
00000000000000000000000000000000000000000000000000000000000000000000000000000000000
00000000000000000000000000000000000000001110000000000000000000000000000000000000000
000000000000000000000000000000000
>P40486_3zuz_142_506
GQMGEGFNWEIEQKMDSSTNNGILKTKYGFDNLYDTVISVSTSNGNDINELDDPEHTDAN
DRVIERLRKENLKFDPEYYVSEYMTHKYGNEEDLEINGIKELLKFTPSIVKQYLQWYKDSTN
PNLVMPIEFTDEEQKQMQDNLPKKSYLVEDIKPLYVTILSVLFSYVFEQIENEGTHTTESAWT
MGKLCPQISFLDQQLKQVNELQDGMKEISKVNKDSSLIKIAIITGIRRALSYPLHRNYDLAM
KAWTFVYYILRGGKRLVIRALLDIHETFRFHDVYYVYDKVLLDDLTAWFISQGSENVIRSLAL
EMRKEQESLSKQDIEFECIASFNEQTGEPEWETLNIREMEILAESEYREQQQNPQ
11111111111111111111100000000000000000000000000000000000000000000000000000000000000
00000000000000000000000000000000000000000000000000000000000000000000000000000000000
00000000000000000000000000000000000011111111111111111000000000000000000000000000000
00000000000000000000000000000000000000000000000000000000000000000000000000000000011
111111100000000000000000000000011
>P54278_1h7s_0_364
MERAESSSTEPAKAIKPIDRKSVHQICSGQVVLSLSTAVKELVENSLDAGATNIDLKLKDYGV
DLIEVSDNGCGVEEENFEGLTLKHHTSKIQEFADLTQVETFGFRGEALSSLCALSDVTISTCH
ASAKVGTRLMFDHNGKIIQKTPYPRPRGTTVSVQQLFSTLPVRHKEFQRNIKKEYAKMVQV
LHAYCIISAGIRVSCTNQLGQGKRQPVVCTGGSPSIKENIGSVFGQKQLQSLIPFVQLPPSDSV
CEEYGLSCSDALHNLFYISGFISQCTHGVGRSSTDRQFFFINRRPCDPAKVCRLVNEVYHMY
NRHQYPFVVLNISVDSECVDINVTPDKRQILLQEEKLLLAVLKTSLIGMFDS
11111111111111111111111111110000000000000000000000000000000000000000000000000000000
00111111111100000000000000000000000000000000000000000000000000000000000000000000000
00000000000000000000000000000000000000000000000000000000000000000000000000000000000
00000000000000000000000000000000000000000000000000000000000000000000000000000000000
000111111000000000000000000000000
>P54997_2de2_0_364
MTSRVDPANPGSELDSAIRDTLTYSNCPVPNALLTASESGFLDAAGIELDVLSGQQGTVHFT
YDQPAYTRFGGEIPPLLSEGLRAPGRTRLLGITPLLGRQGFFVRDDSPITAADLAGRRIGVS
ASAIRILRGQLGDYLELDPWRQTLVALGSWEARALLHTLEHGELGVDDVELVPISSPGVDVP
AEQLEESATVKGADLFPDVARGQAAVLASGDVDALYSWLPWAGELQATGARPVVDLGLD

ERNAYASVWTVSSGLVRQRPGLVQRLVDAAVDAGLWARDHSDAVTSLHAANLGVSTGAV
GQGFGADFQQRLVPRLDHDALALLERTQQFLLTNNLLQEPVALDQWAAPEFLNNSLNRH
R
11111111111111111100000000000000000000000000000000000000000000000000000000000000000
00000000000000000000000000000000000000000000000000000000000000000000000000000000000
00000000000000000000000000000000000000000000000000000000000000000000000000000000000
00000000000000000000000000000000000000000000000000000000000000000000000000000000000
000000000000000000000000000000000
>Q0B6Q2_4gxw_1_365
KGTPGNVPAARTGIEITAAHRAFFHALPKVELHCHLLGAVRHDTFVALAQRSGAPIERAEI
DAFYARGEKPVGVLHVLRALDRYLLTRPDDLRRIAYEYLEDAAAHNVRHAEFFWNPTGTV
RVSGIPYADAQAAIVTGMRDAARDFGIGARLIPSIDREQDPDEAVAIVDWMKANRADEVA
GIGIDYRENDRPPELFWKAYRDARAAGFRTTAHAGEFGMPWRNVETAVDLLHVDRVDHG
YTIVDNPELCARYAERGIVFTVVPTNSYYLRTLPPDQWAERHPMRKMPGLGLKIHPNTDDP
TLHKVNPSEAWELMFSHFGFTIADLKQFMLNGIDGAWVDDDTKAAWRAAWAPEFDMLA
DTLAAD
10000000000000000000000000000000000000000000000000000000000000000000000000000000000
00000000000000000000000000000000000000000000000000000000000000000000000000000000000
00000000000000000000000000000000000000000000000000000000000000000000000000000000000
00000000000000000000000000000000000000000000000000000000000000000000000000000000000
000000000000000000000000000000000
>Q15782_4ay1_25_389
AYKLVCYFTNWSQDRQEPGKFTPENIDPFLCSHLIYSFASIENNKVIIKDKSEVMLYQTINSLK
TKNPKLKILLSIGGYLFGSKGFHPMVDSSTSRLEFINSIILFLRNHNFDGLDVSWIYPDQKENT
HFTVLIHELAEAFQKDFTKSTKERLLLTAGVSAGRQMIDNSYQVEKLAKDLDFINLLSFDFH
GSWEKPLITGHNSPLSKGWQDRGPSSYYNVEYAVGYWIHKGMPSEKVVMGIPTYGHSFTL
ASAETTVGAPASGPGAAGPITESSGFLAYYEICQFLKGAKITRLQDQQVPYAVKGNQWVGY
DDVKSMETKVQFLKNLNLGGAMIWSIDMDDFTGKSCNQGPYPLVQAVKRSLGSL
00000000000000000000000000000000000000000000000000000000000000000000000000000000000
00000000000000000000000000000000000000000000000000000000000000000000000000000000000
00000000000000000000000000000000000000000000000000000000000000000000000000000000000
00000000000000000000000000000000000000000000000000000000000000000000000000000000000
000000000000000000000000000000001
>Q3J1R2_2hzl_0_364
MDRRSFITKAAVGGAAASALAAPALAQSAPKVTWRLASSFPKSLDTIFGGAEVLSKMLSEA
TDGNFQIQVFSAGELVPGLQAADAVTEGTVECCHTVGYYYWGKDPTFALAAAVPFSLSAR
GINAWHYHGGGIDLYNEFLSQHNIVAFPGGNTGVQMGGWFRREINTVADMQGLKMRVG
GFAGKVMERLGVVPQQIAGGDIYPALEKGTIDATEWVGPYDDEKLGFFKVAPYYYYPGWW
EGGPTVHFMFNKSAYEGLTPTYQSLLRTACHAADANMLQLYDWKNPTAIKSLVAQGTQLR
PFSPEILQACFEAANEVYAEMEASNPAFKKIWDSIKAFRSEHYTWAQIAEYNYDTFMMVQQ
NAGKL
11111111111111111111111111110000000000000000000000000000000000000000000000000000000
00000000000000000000000000000000000000000000000000000000000000000000000000000000000
00000000000000000000000000000000000000000000000000000000000000000000000000000000000
00000000000000000000000000000000000000000000000000000000000000000000000000000000000
000000000000000000000000000000000
>Q45135_4ghe_0_364
MSNEIPKPVAPAPDILRCAYAELVVTDLAKSRNFYVDVLGLHVSYEDENQIYLRSFEEFIHHN
LVLTKGPVAALKAMAFRVRTPEDVDKAEAYYQELGCRTERRKDGFVKGIGDALRVEDPLG
FPYEFFFETTHVERLHMRYDLYSAGELVRLDHFNQVTPDVPRGRKYLEDLGFRVTEDIQDDE
GTTYAAWMHRKGTVHDTALTGGNGPRLHHVAFSTHEKHNIIQICDKMGALRISDRIERGP

GRHGVSNAFYLYILDPDNHRIEIYTQDYYTGDPDNPTITWNVHDNQRRDWWGNPVVPSW
YTEASKVLDLDGNVQEIIERTDDSELEVTIGADGFSFTRAGDEDGSYHGQASKGFKLGNQL
11000000000000000000000000000000000000000000000000000000000000000000000000000000000
00000000000000000000000000000000000000000000000000000000000000000000000000000000000
00000000000000000000000000000000000000000000000000000000000000000000000000000000000
00000000000000000000000000000000000000000000000000000000000000000000000000000000000
000000000000000000000000000000111
>Q8X8S5_3g7u_1_365
NVIDLFSGVGGLSLGAARAGFDVKMAVEIDQHAINTHAINFPRSLHVQEDVSLLNAEIIKG
FFKNDMPIDGIIGGPPCQGFSSIGKGNPDDSRNQLYMHFYRLVSELQPLFFLAENVPGIMQE
KYSGIRNKAFNLVSGDYDILDPIKVKASDYGAPTIRTRYFFIGVKKSLKLDISDEVFMPKMIDP
VTVKDALYGLPDIIDANWQSDSESWRTIKKDRKGGFYEKLWGQIPRNVGDTESIAKLKNNII
SGCTGTLHSKIVQERYASLSFGETDKISRSTRLDPNGFCPTLRAGTARDKGSFQAVRPIHPYH
PRVITPREAARLQGFPDWFRFHVTKWHSFRQIGNSVSPIVAEYILKGLYNLLN
00000000000000000000000000000000000000000000000000000000000000000000000000000000001
11111100000000000000000000000000000000000000000000000000000000000000000000000000000
00000000000000000000000000000000000000000000000000000000000000000000000000000000000
00000000000000000000000000000000000000000011111111111110000000000000000000000000000
000000000000000000000000000000000
>P97084_1lc7_0_363
MALFNSAHGGNIREAATVLGISPDQLLDFSANINPLGMPVSVKRALIDNLDCIERYPDADY
FHLHQALARHHQVPASWILAGNGETESIFTVASGLKPRRAMIVTPGFAEYGRALAQSGCEI
RRWSLREADGWQLTDAILEALTPDLDCLFLCTPNNPTGLLPERPLLQAIADRCKSLNINLIL
DEAFIDFIPHETGFIPALKDNPHIWVLRSLTKFYAIPGLRLGYLVNSDDAAMARMRRQQMP
WSVNALAALAGEVALQDSAWQQATWHWLREEGARFYQALCQLPLLTVYPGRANYLLLR
CEREDIDLQRRLLTQRILIRSCANYPGLDSRYYRVAIRSAAQNERLLAALRNVLTGIAPAD
11100000000000000000000000000000000000000000000000000000000000000000000000000000000
00000000000000000000000000000000000000000000000000000000000000000000000000000000000
00000000000000000000000000000000000000000000000000000000000000000000000000000000000
00000000000000000000000000000000000000000000000000000000000000000000000000000000000
0000000000000000000000000000111
>Q3M4J2_3fse_0_363
MTNHNNSGKKKVAILIEQAVEDTEFIIPCNGLKQAGFEVVVLGSRMNEKYKGKRGRLSTQ
ADGTTTEAIASEFDAVVIPGGMAPDKMRRNPNTVRFVQEAMEQGKLVAAVCHGPQVLIEG
DLLRGKQATGFIAISKDMMNAGADYLDEALVVDGNLITSREPGDLAIFTTAILSRLGYGGK
DAALPDEKDRNAEWWKLADAWGGSTKGDIVRGLNTALGGERYSLEALEKYTEKESDVEA
KALFQEMITNKQRHIEYLETYLTRLGEKPSLSANIANQYAKVKTALTGSDDIYQIRSALGDIQ
TGIGDIGNLCAMYTDPIATAIFKEIYKDLVKYEQRLVSLYRTRTNATVQPPKPTTGAAVSM
11111111000000000000000000000000000000000000000000000000000000000000000000000000000
00000000000000000000000000000000000000000000000000000000000000000000000000000000000
00000000000000000000000000000000000000000000000000000000000000000000000000000000000
00000000000000000000000000000000000000000000000000000000000000000000000000000000000
0000000000000000000000000000111
>O30511_2nzw_0_362
MFQPLLDAYVESASIEKMASKSPPPLKIAVANWWGDEEIKEFKNSVLYFILSQRYTITLHQNP
NEFSDLVFGNPLGSARKILSYQNAKRVFYTGENESPNFNLFDYAIGFDELDFNDRYLRMPLY
YDRLHHKAESVNDTTAPYKLKDNSLYALKKPSHCFKEKHPNLCAVVNDESDPLKRGFASF
VASNPNAPIRNAFYDALNSIEPVTGGGSVRNTLGYNVKNKNEFLSQYKFNLCFENTQGYG
YVTEKIIDAYFSHTIPIYWGSPSVAKDFNPKSFVNVHDFKNFDEAIDYIKYLHTHKNAYLDM
LYENPLNTLDGKAYFYQNLSFKKILAFFKTILENDTIYHDNPFIFCRDLNEPLVTI
00000000000000000000000000000000000000000000000000000000000000000000000000000000000

00000000000000000000000000000000000000000000000000000000000000000000000000000000000
00000000000000000000000000000000000000000000000000000000000000000000000000000000000
00000000000000000000000000000000000000000000000000000000000000000000000000000000000
000000000000000001111111111111
>Q313A9_2rb7_0_362
MSSMQHIVELTSDLIRFPSMHSRPEQISRCAGFIMDWCAQNGIHAERMDHDGIPSVMVLPE
KGRAGLLLMAHIDVVDAEDDLFVPRVENDRLYGRGANDDKYAVALGLVMFRDRLNALK
AAGRSQKDMALGLLITGDEEIGGMNGAAKALPLIRADYVVALDGGNPQQVITKEKGIIDIK
LTCTGKAAHGARPWMGVNAVDLLMEDYTRLKTLFAEENEDHWHRTVNLGRIRAGESTN
KVPDVAEGWFNIRVTEHDDPGALIDKIRKTVSGTVSIVRTVPVFLAADSPYTERLLALSGAT
AGKAHGASDARYLGENGLTGVVWGAEGFNTLHSRDECLHIPSLQSIYDPLMQLAREMEE
HAAV
00000000000000000000000000000000000000000000000000000000000000000000000000000000000
00000000000000000000000000000000000000000000000000000000000000000000000000000000000
00000000000000000000000000000000000000000000000000000000000000000000000000000000000
00000000000000000000000000000000000000000000000000000000000000000000000000000000000
000000000000000000000000001111
>Q46E79_4ffr_0_362
MKTICLVGGKLQGFEAAYLSKKAGMKVVLVDKNPQALIRNYADEFYCFDVIKEPEKLLELS
KRVDAVLPVNENLACIEFLNSIKEKFSCPVLFDFEAYRISRDKKKSKDYFKSIGVPTPQDRPSK
PPYFVKPPCESSSVGARIIYDDKDLEGLEPDTLVEEYVEGEVVSLEVVGDGSHFAVVKETLVH
IDETYDCHMVTPLPANPLFRQISHDLAANLPLKGIMDVEAIFGPKGLRVIEIDARFPSQTPTV
VYYSSGINLIELLFRAFTDGVEEIRAIPENKYCIYEHLMFGENGVLIPVGEQVLSMGSDYGKF
YEEPGIEIFLCKGEYPVFTMVFWGKDREETGAKRCKGLSVLKERFGAVL
00000000000000000000000000000000000000000000000000000000000000000000000000000000000
00000000000000000000000000000000000000000000000000000000000000000111100000000000000
00000000000000000000000000000000000000000000000000000000000000000000000000000000000
00000000000000000000000000111100000000000000000000000000000000000000000000000000000
000000000000000000000011111111
>Q9TVW2_1lii_0_362
MAVDSSNSATGPMRVFAIGNPILDLVAEVPSSFLDEFFLKRGDATLATPEQMRIYSTLDQFNP
TSLPGGSALNSVRVVQKLLRKPGSAGYMGAIGDDPRGQVLKELCDKEGLATRFMVAPGQS
TGVCAVLINEKERTLCTHLGACGSFRLPEDWTTFASGALIFYATAYTLTATPKNALEVAGYA
HGIPNAIFTLNLSAPFCVELYKDAMQSLLLHTNILFGNEEEFAHLAKVHNLVAAEKTALSTA
NKEHAVEVCTGALRLLTAGQNTGATKLVVMTRGHNPVIAAEQTADGTVVVHEVGVPVVA
AEKIVDTNGAGDAFVGGFLYALSQGKTVKQCIMCGNACAQDVIQHVGFSLSFTSLPC
11111111110000000000000000000000000000000000000000000000000000000000000000000000000
00000000000000000000000000000000000000000000000000000000000000000000000000000000000
00000000000000000000000000000000000000000000000000000000000000000000000011000000000
00000111111111111111000000000000000000000000000000000000000000000000000000000000000
000000000000000000000000011111
>Q9Y7D0_3gqv_0_362
MGDQPFIPPPQQTALTVNDHDEVTVWNAAPCPMLPRDQVYVRVEAVAINPSDTKMRGQF
ATPWAFLGTDYAGTVVAVGSDVTHIQVGDRVYGAQNEMCPRTPDQGAFSQYTVTRGRVW
AKIPKGLSFEQAAALPAGISTAGLAMKLLGLPLPSPSADQPPTHSKPVYVLVYGGSTATATVT
MQMLRLSGYIPIATCSPHNFDLAKSRGAEEVFDYRAPNLAQTIRTYTKNNLRYALDCITNVE
STTFCFAAIGRAGGHYVSLNPFPEHAATRKMVTTDWTLGPTIFGEGSTWPAPYGRPGSEEER
QFGEDLWRIAGQLVEDGRLVHHPLRVVQGGFDHIKQGMELVRKGELSGEKLVVRLEGP
11100000000000000000000000000000000000000000000000000111110000000000000000000000000
00000000000000000000000000000000000000000000000000000000000000000000000000000000000
00000000000000000000000000000000000000000000000000000000000000000000000000000000000

000000000000000000111000000000000000000000000000000000000000000000000000000000000000
0000000000000000000000000000000
>A7LZL0_3n6z_19_380
ISCSDPNPLDSYTRVPFEKTETDDGNGDGDGDGAGGLFEKGNGTDSKPYMIMNATQIRNM
RSVLKSGMKVYFQLGADIDMAGIDDWQSLNGSGDFPYEIDFDGDSHVIKNFKCSAGDYPSF
FGVLCGDCRNVGFVNASVSSARQGIGIITGYLGLKDKGNGNKTGRIVNCYTTGEVIGSGAA
GGIAGVLANSYDGQESYIKNCYSNATVSDRAASGGKAGGIAGRKVGVGGFIENCYAYGAV
SATKGGVGGILGQIDKSCDIAIKNSAAWSNLTGVDASSTVGRIVGVSASLGSYENCYACESIV
LKVNEKTITASDESSATGTTFHGVAKSAEELGNIIVAWNPNLWKKGTNGYPIFQWSE
11111111100000001111111111111100000000000000000000000000000000000000000000000000000
00000000000000000000000000000000000000000000000000000000000000000000000000000000000
00000000000000000000000000000000000000000000000000000000000000000000000000000000000
00000000000000000000000000000000000000000000000000000000000000000000000000000000000
000000000000000000000000000000
>P52788_3c6k_4_365
RHSTLDFMLGAKADGETILKGLQSIFQEQGMAESVHTWQDHGYLATYTNKNGSFANLRIY
PHGLVLLDLQSYDGDAQGKEEIDSILNKVEERMKELSQDSTGRVKRLPPIVRGGAIDRYWPT
ADGRLVEYDIDEVVYDEDSPYQNIKILHSKQFGNILILSGDVNLAESDLAYTRAIMGSGKED
YTGKDVLILGGGDGGILCEIVKLKPKMVTMVEIDQMVIDGCKKYMRKTCGDVLDNLKGD
CYQVLIEDCIPVLKRYAKEGREFDYVINDLTAVPISTSPEEDSTWEFLRLILDLSMKVLKQDGK
YFTQGNCVNLTEALSLYEEQLGRLYCPVEFSKEIVCVPSYLELWVFYTVWKKAKP
00000000011110000000000000000000000000000000000000000000000000000000000000011100000000
00000000000000111100000000000000000000000000000000000000000000000000000000000000000
00000000000000000000000000000000000000000000000000000000000000000111100000000000000
00000000000000000000000000000000111000000000000000000000000000000000000000000000000
000000000000000000000000000000
>Q6N0W2_3rpw_0_361
MLGLKRFRLPALASAAFIALGAGTAAAQSNVVIMQDPGGGYGDALRKVMYDPFEKETGIK
VVTVQEARSGPRIKAQAEAGKAQWDLTFIFDQETKLLGDCCLADIDYSKLSESAHKTLAA
MPDNLKRKKGVALQVIGVGLVYNKDKFKGDKAPQTWADFWDVKKFPGRRCMPAWPRFT
FEAALMADGVTKDKLYPIDMDRALKKLKEIKPHVVKWWTTAAQPPQLILDGEADMCLAY
TGSMSKLALEGAPIDLTFNQGFVYYDFFSIPKGAPNYDNALKLLSWRLDPKRAAQLTSTFPV
ALPSKVVFDAATDKNIARYWANNPENVAKAIEWSPDFWGAPSPAGNSTNEEYGQEKLNA
MLAQ
11111111111111111111111111100000000000000000000000000000000000000000000000000000000
00000000000000000000000000000000000000000000000000000000000000000000000000000000000
00000000000000000000000000000000000000000000000000000000000000000000000000000000000
00000000000000000000000000000000000000000000000000000000000000000000000000000000000
000000000000000000000000000011
>P95481_2v7k_0_360
MERTLDRVGVFAATHAAVAACDPLQARALVLQLPGLNRNKDVPGIVGLLREFLPVRGLPC
GWGFVEAAAAMRDIGFFLGSLKRHGHEPAEVVPGLEPVLLDLARATNLPPRETLLHVTVW
NPTAADAQRSYTGLPDEAHLLESVRISMAALEAAIALTVELFDVSLRSPEFAQRCDELEAYL
QKMVESIVYAYRFISPQVFYDELRPFYEPIRVGGQSYLGPGAVEMPLFVLEHVLWGSQSDDQ
TYREFKETYLPYVLPAYRAVYARFSGEPALIDRALDEARAVGTRDEHVRAGLTALERVFKVL
LRFRAPHLKLAERAYEVGQSGPEIGSGGYAPSMLGELLTLTYAARSRVRAALDES
11100000000000000000000000000000000000000000000000000000000000000000000000000000000
00000000000000000000000000000000000000000000000000000000000000000000000000000000000
00000000000000000000000000000000000000000000000000000000000000000000000000000000000
00000000000000000000000000000000000000000000000000000000000000000000000000111111111
11100000000000000000000000011

>Q01064_1taz_145_505
VGPTYSTAVLNCLKNLDLWCFDVFSLNQAADDHALRTIVFELLTRHNLISRFKIPTVFLMSF
LDALETGYGKYKNPYHNQIHAADVTQTVHCFLLRTGMVHCLSEIELLAIIFAAAIHDYEHT
GTTNSFHIQTKSECAIVYNDRSVLENHHISSVFRLMQDDEMNIFINLTKDEFVELRALVIEM
VLATDMSCHFQQVKTMKTALQQLERIDKPKALSLLLHAADISHPTKQWLVHSRWTKALM
EEFFRQGDKEAELGLPFSPLCDRTSTLVAQSQIGFIDFIVEPTFSVLTDVAEKSVQPLADEDSKS
KNQPSFQWRQPSLDVEVGDPNPDVVSFRSTWVKRIQENKQKWKERAASGITN
00000000000000000000000000000000000000000000000000000000000000000000000000000000
00000000000000000000000000000000000000000000000000000000000000000000000000000000
00000000000000000000000000000000000000011000000000000000000000000000000000000000
00000000001111110000000000000000000000000000000011111111111111111111111111100000
0000000000000000000000001111
>Q39193_3uc3_0_360
MDRAPVTTGPLDMPIMHDSDRYDFVKDIGSGNFGVARLMRDKLTKELVAVKYIERGDKIDE
NVQREIINHRSLRHPNIVRFKEVILTPTHLAIIMEYASGGELYERICNAGRFSEDEARFFFQQL
LSGVSYCHSMQICHRDLKLENTLLDGSPAPRLKICDFGYSKSSVLHSQPKSTVGTPAYIAPEV
LLRQEYDGKIADVWSCGVTLYVMLVGAYPFEDPEEPRDYRKTIQRILSVKYSIPDDIRISPECC
HLISRIFVADPATRISIPEIKTHSWFLKNLPADLMNESNTGSQFQEPEQPMQSLDTIMQIISEAT
IPAVRNRCLDDFMTDNLDLDDDMDDFDSESEIDIDSSGEIVYAL
11111111110000000000000000000000000000000000000000000000000000000000000000000000
00000000000000000000000000000000000000000000000000000000000000000000000000000001
11111111111111000000000000000000000000000000011110000000000000000000000000000000
00000000000000000000000000000000000111111111111111111100000000000001111111111111
1111111111111111111111111111
>Q4QHJ8_3pg1_0_360
MQAKGEAAMRDLIAELHAMQSPYTVQRFISSGSYGAVCAGVDSEGIPVAIKRVFNTVSDGR
TVNILSDSFLCKRVLREIRLLNHFHHPNILGLRDIFVHFEEPAMHKLYLVTELMRTDLAQVIH
DQRIVISPQHIQYFMYHILLGLHVLHEAGVVHRDLHPGNILLADNNDITICDFNLAREDTA
DANKTHYVTHRWYRAPELVMQFKGFTKLVDMWSAGCVMAEMFNRKALFRGSTFYNQL
NKIVEVVGTPKIEDVVMFSSPSARDYLRNSLSNVPARAWTAVVPTADPVALDLIAKMLEFNP
QRRISTEQALRHPYFESLFDPLDLTEGLSERFHFDESVTDVYDMHKIFTAEVERFND
11110000000000000000000000000000000000000000000000000000000000000000000000000000
00000000000000000000000000000000000000000000000000000000000000000000000000000000
00000000000001111111111111100000000000000000000000000000000000000000000000000000
00000000000000000000000000000000000000000000000000000000000000000000000000000000
0000000000000000000000000001
>Q5SK82_2zzx_0_360
MKRVSRRAFLRRLGVGVAATAAFSPLAVAQARRYRWRIQTAWDAGTVGYSLFQKFTERVK
ELTDGQLEVQPFPAGAVVGTFDMFDAVKTGVLDGMNPFTLYWAGRMPVTAFLSSYALGLD
RPDQWETWFYSLGGLDIARRAFAEQGLFYVGPVQHDLNIIHSKKPIRRFEDFKGVKLRVPG
GMIAEVFAAAGASTVLLPGGEVYPALERGVIDAADFVGPAVNYNLGFHQVAKYIIMGPPET
PAIHQPVDLMDFTINLNRWRSLPKPLQERFIAAVHEYSWIHYAGIQKANLEAWPKYRQAG
VEVIRLSNEDVRKFRRLAIPIWFKWAKMDKYSREAFASQLEYMKGIGYVTDEELKGLSL
11111111111111111111111111111100000000000000000000000000000000000000000000000000
00000000000000000000000000000000000000000000000000000000000000000000000000000000
00000000000000000000000000000000000000000000000000000000000000000000000000000000
00000000000000000000000000000000000000000000000000000000000000000000000000000000
0000000000000000000000000000
>Q8Y6T2_3nvt_0_360
MVNTNLEELRTQVDQLNIDLLELISKRANLVQEIGKIKGTQGSLRFDPLREREMLNTILAAN
EGPFEDSTVQKLFKEIFKAGLELQEEDHSKALLVSRKNKKEDTIVTVKGLPIGNGEPVFVFGP

CSVESYEQVAAVAESIKAKGLKLIRGGAFKPRTSPYDFQGLGLEGLKILKRVSDEYGLGVISEI
VTPADIEVALDYVDVIQIGARNMQNFELLKAAGRVDKPILLKRGLSATIEEFIGAAEYIMSQ
GNGKIILCERGIRTYEKATRNTLDISAVPILKKETHLPVMVDVTHSTGRKDLLLPCAKAALAI
EADGVMAEVHPDPAVALSDSAQQMDIPEFEEFWNAILASNLVPHKIK
11111000000000000000000000000000000000011111000000000000000000000000000000000000000
00000000000000000000000000000000000000000000000000000000000000000000000000000000000
00000000000000000000000000000000000000000000000000000000000000000000000000000000000
00000000000000000000000000000000000000000000000000000000000000000000000000000000000
00000000000000000000000001111
>Q9H777_3zwf_2_362
MDVTFLGTGAAYPSPTRGASAVVLRCEGECWLFDCGEGTQTQLMKSQLKAGRITKIFITHL
HGDHFFGLPGLLCTISLQSGSMVSKQPIEIYGPVGLRDFIWRTMELSHTELVFHYVVHELVPT
ADQCPAEELKEFAHVNRADSPPKEEQGRTILLDSEENSYLLFDDEQFVVKAFRLFHRIPSFGF
SVVEKKRPGKLNAQKLKDLGVPPGPAYGKLKNGISVVLENGVTISPQDVLKKPIVGRKICIL
GDCSGVVGDGGVKLCFEADLLIHEATLDDAQMDKAKEHGHSTPQMAATFAKLCRAKRLV
LTHFSQRYKPVALAREGETDGIAELKKQAESVLDLQEVTLAEDFMVISIPIKK
00000000000000000000000000000000000000000000000000000000000000000000000000000000111
00000000000000000000000000000000000000000000000111111111111111111000000000000000000
00000000000000000000000000011111111111111111111111111111111111111111111111000000000
00000000000000000000000000000000000000000000000000000000000000000000011111111111111
11111111111100000000000000011
>Q9K499_3lg5_0_360
MHAFPHGTTATPTAIAVPPSLRLPVIEAAFPRQLHPYWPKLQETTRTWLLEKRLMPADKVE
EYADGLCYTDLMAGYYLGAPDEVLQAIADYSAWFFVWDDRHDRDIVHGRAGAWRRLRG
LLHTALDSPGDHLHHEDTLVAGFADSVRRLYAFLPATWNARFARHFHTVIEAYDREFHNRT
RGIVPGVEEYLELRRLTFAHWIWTDLLEPSSGCELPDAVRKHPAYRRAALLSQEFAAWYND
LCSLPKEIAGDEVHNLGISLITHHSLTLEEAIGEVRRRVEECITEFLAVERDALRFADELADGT
VRGKELSGAVRANVGNMRNWFSSVYWFHHESGRYMVDSWDDRSTPPYVNNEAAGEK
11111111111110000000000000000000000000000000000000000000000000000000000000000000000
00000000000000000000000000000000000000000000000000000000000000000000000000000000000
00000000000000000000000000000000000000000000000000000000000000000000000000000000000
00000000000000000000000000000000000000000000000000000000000000000000000000000000000
00000000000000000000000111111
>A0QNT0_3p2y_0_359
MTLIGVPRESAEGERRVALVPKVVEKLSARGLEVVVESAAGAGALFSDADYERAGATIGDP
WPADVVVKVNPPTSDEISQLKPGSVLIGFLAPRTQPELASRLRIADVTAFAMESIPRISRAQT
MDALSSQANVAGYKAVLLGASLSTRFVPMLTTAAGTVKPASALVLGVGVAGLQALATAKR
LGAKTTGYDVRPEVAEQVRSVGAQWLDLGIDAAGEGGYARELSEAERAQQQQALEDAIT
KFDIVITTALVPGRPAPRLVTAAAATGMQPGSVVVDLAGETGGNCELTEPGRTIVHHGVTIT
SPLNLPATMPEHASELYAKNVTALLDLLLTDDGVAPDFTDEIVAASCITRTEGDI
00000000000000000000000000000000000000000000000000000000000000000000000000000000000
00000000000000000000000000000000000000000000000000000000000000000000000000000000000
00000000000000000000000000000000000000000000000111111111111100000000000000000000000
00000000000000000000000000000000000000000000000000000000000000000000000000000000000
0000000000000000000000001111
>A7M120_3oyv_24_383
SDDDNPTVDPANIDYTPENASSWHNYMRNVAALLKTDATNLYNAWNSSYKGGESYASLF
KAHSGSPYASALSCVEEIVDKCAEIANEVGTAKIGDPYNLYKAGNTEEALYAVESWYSWHS
RDDYTNNIYSIRNAYYGSLDGNINANSLSTVIAGANSSLDTKIKNAIQKAAKAIQDIPQPFR
NHIPSNETVAAMDACAELESILKNDLKSYIANNSNNINTDAVLNPVVTQYVDAVVVPTYKS
LKEKNDALYNAVIVLADNPSNSAFETACDAWITAREPWEKSEAFLFGPVDEMGLDPNMDS

WPLDQNAIVQILNSQSWSDLEWSEGDDEAAVESAQNVRGFHTLEFLLYKNGEPRKVQ
11111000000000000000000000000000000000000000000000000000000000000000000000000000000
00000000000000000000000000000000000000000000000000000000000000000000000000000000000
00000000000000000000000000000000000000000000000000000000000000000000000000000000000
00000000000000000000000000000000000000000000000000000000000000000000000000000000000
0000000000000000000000000000
>O06543_2gce_0_359
MAGPLSGLRVVELAGIGPGPHAAMILGDLGADVVRIDRPSSVDGISRDAMLRNRRIVTADL
KSDQGLELALKLIAKADVLIEGYRPGVTERLGLGPEECAKVNDRLIYARMTGWGQTGPRSQ
QAGHDINYISLNGILHAIGRGDERPVPPLNLVGDFGGGSMFLLVGILAALWERQSSGKGQV
VDAAMVDGSSVLIQMMWAMRATGMWTDTRGANMLDGGAPYYDTYECADGRYVAVGAI
EPQFYAAMLAGLGLDAAELPPQNDRARWPELRALLTEAFASHDRDHWGAVFANSDACV
TPVLAFGEVHNEPHIIERNTFYEANGGWQPMPAPRFSRTASSQPRPPAATIDIEAVLTDWDG
10000000000000000000000000000000000000011111000000000000000000000000000000000000000
00000000000000000000000000000000000000000000000000000000000000000000000000000000000
00000000000000000000000000000000000000000000000000000000000000000000000000000000000
00000000000000000000000000000000000000000000000000000000000000000000000000000000000
0000000000000000000000000000
>Q81IM3_4hd5_0_359
MRKYAAIALCTSAILAGCNTSNVSQEPKKEKKVQEVAIQKEALQEQGKISYTPITHESTNTSI
HITDLKDSLNEVQYKIWRTADGKERAKSFSSKEKEKQFTIPFDIKEFEGKRGEFQIEATGMKE
DGKTIPLTKSIITFEQKVPVLMYHAIDDYHGQGIKDLFVSPANFEAQMKHLKDNGYTLLTFE
RWGDINKVNKPIFVTFDDGMKNNMNAFRVLQKLKDDTFKPAATEYMIVDNVDVEGALST
SEIKEMVDSGIFSVQSHTATHADLPKITNYEEELKGSKEKLEKITGKPVIAIAYPFGHVDDKV
VTETKKYYQFATTTKPGQFITKGEPDELLKMKRVRIHHTTTVEQFASSIK
11111111111111111111111111111111111111111111000000000000000000000000000000000000000
00000000000000000000000000000000000000000000000000000000000000000000000000000000000
00000000000000000000000000000000000000000000000000000000000000000000000000000000000
00000000000000000000000000000000000000000000000000000000000000000000000000000000000
0000000000000000000000000000
>Q9H3S7_3rau_1_360
EAVPRMPMIWLDLKEAGDFHFQPAVKKFVLKNYGENPEAYNEELKKLELLRQNAVRVPRD
FEGCSVLRKYLGQLHYLQSRVPMGSGQEAAVPVTWTEIFSGKSVAHEDIKYEQACILYNLG
ALHSMLGAMDKRVSEEGMKVSCTHFQCAAGAFAYLREHFPQAYSVDMSRQILTLNVNLM
LGQAQECLLEKSMLDNRKSFLVARISAQVVDYYKEACRALENPDTASLLGRIQKDWKKLV
QMKIYYFAAVAHLHMGKQAEEQQKFGERVAYFQSALDKLNEAIKLAKGQPDTVQDALRF
TMDVIGGKYNSAKKDNDFIYHEAVPALDTLQPVKGAPLVKPLPVNPTDPAVTGPDIFAKLV
11000000000000000000000000000000000000000000000000000000000000000000000000000000000
00000000000000000000000000000000000000000000000000000000000000000000000000000000000
00000000000000000000000000000000000000000000000000000000000000000000000000000000000
00000000000000000000000000000000000000000000000000000000000000000000000000000000000
0000000000000000000000000000
>Q9ZTU2_3p9c_0_359
MGSTAADMAASADEDACMFALQLASSSVLPMTLKNAIELGLLEILVAAGGKSLTPTEVAAK
LPSAANPEAPDMVDRILRLLASYNVVTCLVEEGKDGRLSRSYGAAPVCKFLTPNEDGVSM
AALALMNQDKVLMESWYYLKDAVLDGGIPFNKAYGMSAFEYHGTDPRFNRVFNEGMKN
HSIIITKKLLELYHGFEGLGTLVDVGGGVGATVAAIAAHYPTIKGVNFDLPHVISEAPQFPGV
THVGGDMFKEVPSGDTILMKWILHDWSDQHCATLLKNCYDALPAHGKVVLVQCILPVNP
EANPSSQGVFHVDMIMLAHNPGGRERYEREFQALARGAGFTGVKSTYIYANAWAIEFTK
11111111000000000000000000000000000000000000000000000000000000000000000000000000000
00000000000000000000000000000000000000000000000000000000000000000000000000000000000

00000000000000000000000000000000000000000000000000000000000000000000000000000000000
00000000000000000000000000000000000000000000000000000000000000000000000000000000000
0000000000000000000000000000
>O34453_1m7v_4_362
EILWNEAKAFIAACYQELGKEEEVKDRLADIKSEIDLTGSYVHTKEELEHGAKMAWRNSNR
CIGRLFWNSLNVIDRRDVRTKEEVRDALFHHIETATNNGKIRPTITIFPPEEKGEKQVEIWNH
QLIRYAGYESDGERIGDPASCSLTAACEELGWRGERTDFDLLPLIFRMKGDEQPVWYELPRS
LVIEVPITHPDIEAFSDLELKWYGVPIISDMKLEVGGIHYNAAPFNGWYMGTEIGARNLADE
KRYDKLKKVASVIGIAADYNTDLWKDQALVELNKAVLHSYKKQGVSIVDHHTAASQFKRF
EEQEEEAGRKLTGDWTWLIPPISPAATHIFHRSYDNSIVKPNYFYQDKPYE
00000000000000000000000000000000000000000000000000000000000000000000000000000000000
00000000000000000000000000000000000000000000000000110000000000000000000000000000000
00000000000000000000000000000000000000000000000000000000000000000000000000000000000
00000000000000000000000000000000000000000000000000000000000000000000000000000000000
000000000000000000000000000
>Q1CZR7_3cwv_1_359
LGMHLPGGIVENVRKRPGMYCGDVGEYGLHHLVYFLLDVAYEEARRGECRDVVLEVGGD
GSIALFCTSRTVTAENLVRVATGAGFLGRPPGDGWGWDSMLVVSLALSSRYQVDIWADGR
QWRVMGEHGHPQGEGAAVTPMEPMPVSAERGVRVHFVPDATIFEVLAFDRARLSRRCNE
LAALAPGLRVSFADLQRGERTLWHLPGGVAQWAHVLTEARPQLHPEPVVFDFTWDGLRV
QCALQWCEDEDSTLLSFANAVRTVRHGAHVKGVTQALRGALAKLSGETRGAFPWARVAQ
GLTAIVAVSGPRRQMAFAGPTKELLAIPGLEEAIRKQLQPLFIELLREHPVTPALLARRTSGS
11111100000000000000000000000000000000000000000000000000000000000000000000000000000
00000000000000000000000000000000000000000000000000000000000000000000000000000000000
00000000000000000000000000000000000000000000000000000000000000000000000000000000000
00000000000000000000000000000000000000000000000000000000000000000000000000000000000
000000000000000000000000111
>Q44297_1bg6_0_358
MIESKTYAVLGLGNGGHAFAAYLALKGQSVLAWDIDAQRIKEIQDRGAIIAEGPGLAGTAH
PDLLTSDIGLAVKDADVILIVVPAIHHASIAANIASYISEGQLIILNPGATGGALEFRKILRENG
APEVTIGETSSMLFTCRSERPGQVTVNAIKGAMDFACLPAAKAGWALEQIGSVLPQYVAVE
NVLHTSLTNVNAVMHPLPTLLNAARCESGTPFQYYLEGITPSVGSLAEKVDAERIAIAKAFD
LNVPSVCEWYKESYGQSPATIYEAVQGNPAYRGIAGPINLNTRYFFEDVSTGLVPLSELGRAV
NVPTPLIDAVLDLISSLIDTDFRKEGRTLEKLGLSGLTAAGIRSAVE
11100000000000000000000000000000000000000000000000000000000000000000000000000000000
00000000000000000000000000000000000000000000000000000000000000000000000000000000000
00000000000000000000000000000000000000000000000000000000000000000000000000000000000
00000000001111111000000000000000000000000000000000000000000000000000000000000000000
000000000000000000000000000
>Q9RVE0_2o5v_0_358
MGDVRLSALSTLNYRNLAPGTLNFPEGVTGIYGENGAGKTNLLEAAYLALTGQTDAPRIEQ
LIQAGETEAYVRADLQQGGSLSIQEVGLGRGRRQLKVDGVRARTGDLPRGGAVWIRPEDSE
LVFGPPSGRRAYLDSLLSRLSARYGEQLSRYERTVSQRNAALRGGEEWAMHVWDDVLLKL
GTEIMLFRRRALTRLDELAREANAQLGSRKTLALTLTESTSPETYAADLRGRRAEELARGST
VTGPHRDDLLLTLGDFPASDYASRGEGRTVALALRRAELELLREKFGEDPVLLLDDFTAELD
PHRRQYLLDLAASVPQAIVTGTELAPGAALTLRAQAGRFTPVADEEMQAEGTA
10000000000000000000000000000000000000000000000000000000000000000000000000000000000
00000000000000000000000000000000000000000000000000000000000000000000000000000000000
00000000000000000000000000000000000000000000000000000000000000000000000000000000000
00000000000000000000000000000000000000000000000000000000000000000000000000000000000
000000000000000000000000000

>Q08493_2qym_305_662
TVPRFGVQTDQEEQLAKELEDTNKWGLDVFKVAELSGNRPLTAIIFSIFQERDLLKTFQIPAD
TLATYLLMLEGHYHANVAYHNSLHAADVAQSTHVLLATPALEAVFTDLEILAALFASAIHD
VDHPGVSNQFLINTNSELALMYNDASVLENHHLAVGFKLLQAENCDIFQNLSAKQRLSLR
RMVIDMVLATDMSKHMNLLADLKTMVETKKVTSLGVLLLDNYSDRIQVLQNLVHCADLS
NPTKPLPLYRQWTDRIMAEFFQQGDRERESGLDISPMCDKHTASVEKSQVGFIDYIAHPLW
ETWADLVHPDAQDLLDTLEDNREWYQSKIPRSPSDLTNPERDGPDRFQFELTLE
10000000000000000000000000000000000000000000000000000000000000000000000000000000000
00000000000000000000000000000000000000000000000000111111111111100000000000000000000
00000000000000000000000000000000000000000000000000000000000000000000000000000000000
00000000000000000111111111111111111111111100000000000000000000000000000000000000011
11111111111111111111111111
>Q18014_3zy4_25_382
TDPNGYIVFCPCMGRFGNQVDQFLGVLAFAKALDRTLVLPNFIEFKHPETKMIPFEFLFQVG
TVAKYTRVVTMQEFTKKIMPTVWPPEKRKAFCWTPRQAIYDKSAEPGCHSKEGNPFGPYW
DQIDVSFVGDEYFGDIPGGFDLNQMGSRKKWLEKFPSEEYPVLAFSSAPAPFPSKGKVWSIQ
KYLRWSSRITEQAKKFISANLAKPFVAVHLRNDADWVRVCEHIDTTTNRPLFASEQCLGEG
HHLGTLTKEICSPSKQQILEQIVEKVGSIGAKSVFVASDKDHMIDEINEALKPYEIEAHRQEP
DDMYTSLAIMGRADLFVGNCVSTFSHIVKRERDHAGQSPRPSAFFGIRAV
00000000000000000000000000000000000000000000001110000000000000000000000000000000000
00000000000000000001111000000000000000000000000000000000000000000000000000000000000
00000000000000000000000000000000000000000000000000000000000000000000000000000000000
00000000000000000000000000000000000000000000000000000000000000000000000000000000000
00000000000111000000000111
>Q58194_3daf_0_357
MKIAILGAGCYRTHAAAGITNFMRACEVAKEVGKPEIALTHSSITYGAELLHLVPDVKEVIV
SDPCFAEEPGLVVIDEFDPKEVMEAHLSGNPESIMPKIREVVKAKAKELPKPPKACIHLVHP
EDVGLKVTSDDREAVEGADIVITWLPKGNKQPDIIKKFADAIPEGAIVTHACTIPTTKFAKIF
KDLGREDLNITSYHPGCVPEMKGQVYIAEGYASEEAVNKLYEIGKIARGKAFKMPANLIGP
VCDMCSAVTATVYAGLLAYRDAVTKILGAPADFAQMMADEALTQIHNLMKEKGIANMEE
ALDPAALLGTADSMCFGPLAEILPTALKVLEKHKVVEEEGKTKCEIMSQKE
00000000000000000000000000000000000000000000000000000000000000000000000000000000000
00000000000000000000000000000000000000000000000000000000000000000000000000000000000
00000000000000000000000000000000000000000000000000000000000000000000000000000000000
00000000000000000000000000000000000000000000000000000000000000000000000000000000000
00000000000011111111111111
>Q6WZB0_2wbo_0_357
MTESPTTHHGAAPPDSVATPVRPWSEFRLTPAEAAAAAALAARCAQRYDETDGPEFLLDA
PVIAHELPRRLRTFMARARLDAWPHALVVRGNPVDDAALGSTPVHWRTARTPGSRPLSFL
LMLYAGLLGDVFGWATQQDGRVVTDVLPIKGGEHTLVSSSSRQELGWHTEDAFSPYRADY
VGLLSLRNPDGVATTLAGVPLDDLDERTLDVLFQERFLIRPDDSHLQVNNSTAQQGRVEFE
GIAQAADRPEPVAILTGHRAAPHLRVDGDFSAPAEGDEEAAAALGTLRKLIDASLYELVLD
QGDVAFIDNRRAVHGRRAFQPRYDGRDRWLKRINITRDLHRSRKAWAGDSRVLGQR
11111111111111111111000000000000000000000000000000000000000000000000000000000000000
00000000000000000000000000000000000000000000000000000000000000000000000000000000000
00000000000000000000000000000000000000000000000000000000000000000000000000000000000
00000000000000000000000000000000000000000000000000000000000000000000000000000000000
00000000000000000000000001
>P15802_2x1d_0_356
MLHILCQGTPFEIGYEHGSAAKAVIARSIDFAVDLIRGKTKKTDEELKQVLSQLGRVIEERWP
KYYEEIRGIAKGAERDVSEIVMLNTRTEFAYGLKAARDGCTTAYCQLPNGALQGQNWDFFS

ATKENLIRLTIRQAGLPTIKFITEAGIIGKVGFNSAGVAVNYNALHLQGLRPTGVPSHIALRIA
LESTSPSQAYDRIVEQGGMAASAFIMVGNGHEAFGLEFSPTSIRKQVLDANGRMVHTNHC
LLQHGKNEKELDPLPDSWNRHQRMEFLLDGFDGTKQAFAQLWADEDNYPFSICRAYEEG
KSRGATLFNIIYDHARREATVRLGRPTNPDEMFVMRFDEEDERSALNARL
00000000000000000000000000000000000000000000000000000000000000000000000000000000000
00000000000000011110000000000000000000000000000000000000000000000000000000000000000
00000000000000000000000000000000000000000000000000000000000000000000000000000000000
00000000000000000000000000000000000000000000000000000000000000000000000000000000000
0000000000000000000000001
>P19424_1g0c_227_583
AVKSPSEAGALQLVELNGQLTLAGEDGTPVQLRGMSTHGLQWFGEIVNENAFVALSNDW
GSNMIRLAMYIGENGYATNPEVKDLVYEGIELAFEHDMYVIVDWHVHAPGDPRADVYSG
AYDFFEEIADHYKDHPKNHYIIWELANEPSPNNNGGPGLTNDEKGWEAVKEYAEPIVEML
REKGDNMILVGNPNWSQRPDLSADNPIDAENIMYSVHFYTGSHGASHIGYPEGTPSSERSN
VMANVRYALDNGVAVFATEWGTSQANGDGGPYFDEADVWLNFLNKHNISWANWSLTN
KNEISGAFTPFELGRTDATDLDPGANQVWAPEELSLSGEYVRARIKGIEYTPIDRTKFTKL
00000000000000000000000000000000000000000000000000000000000000000000000000000000000
00000000000000000000000000000000000000000000000000000000000000000000000000000000000
00000000000000000000000000000000000000000000000000000000000000000000000000000000000
00000000000000000000000000000000000000000000000000000000000000000000000000000000000
0000000000000000000001111
>P32179_1ka1_0_356
MALERELLVATQAVRKASLLTKRIQSEVISHKDSTTITKNDNSPVTTGDYAAQTIIINAIKSNF
PDDKVVGEESSSGLSDAFVSGILNEIKANDEVYNKNYKKDDFLFTNDQFPLKSLEDVRQIID
FGNYEGGRKGRFWCLDPIDGTKGFLRGEQFAVCLALIVDGVVQLGCIGCPNLVLSSYGAQD
LKGHESFGYIFRAVRGLGAFYSPSSDAESWTKIHVRHLKDTKDMITLEGVEKGHSSHDEQT
AIKNKLNISKSLHLDSQAKYCLLALGLADVYLRLPIKLSYQEKIWDHAAGNVIVHEAGGIH
TDAMEDVPLDFGNGRTLATKGVIASSGPRELHDLVVSTSCDVIQSRNA
10000000000000000000000000000000000000000000000000000000000000000000000000000000000
00000000000000000000000000000000000000000000000000000000000000000000000000000000000
00000000000000000000000000000000000000000000000000000000000000000000000000000000000
00000000000000000000000000000000000000000000000000000000000000000000000000000000000
0000000000000000000000011
>Q57159_4aqn_0_356
MSDTMVVNGSGGVPAFLFSGSTLSSYRPNFEANSITIALPHYVDLPGRSNFKLMYIMGFPIDT
EMEKDSEYSNKIRQESKISKTEGTVSYEQKITVETGQEKDGVKVYRVMVLEGTIAESIEHLDK
KENEDILNNNRNRIVLADNTVINFDNISQLKEFLRRSVNIVDHDIFSSNGFEGFNPTSHFPSN
PSSDYFNSTGVTFGSGVDLGQRSKQDLLNDGVPQYIADRLDGYYMLRGKEAYDKVRTAPLT
LSDNEAHLLSNIYIDKFSHKIEGLFNDANIGLRFSDLPLRTRTALVSIGYQKGFKLSRTAPTV
WNKVIAKDWNGLVNAFNNIVDGMSDRRKREGALVQKDIDSGLLK
11111111111100000000000000001111100000000000000000000000000000000000000000000000000
00000000000000000000000000000000000000000000000000000000000000000000000000000000000
00000000000000000000000000000000000000000000000000000000000000000000000000000000000
00000000000000000000000000000000000000000000000000000000000000000000000000000000000
0000000000000000000000000
>Q57329_1rkx_0_356
MINNSFWQGKRVFVTGHTGFKGGWLSLWLQTMGATVKGYSLTAPTVPSLFETARVADGM
QSEIGDIRDQNKLLESIREFQPEIVFHMAAQPLVRLSYSEPVETYSTNVMGTVYLLEAIRHVG
GVKAVVNITSDKCYDNKEWIWGYRENEAMGGYDPYSNSKGCAELVTSSYRNSFFNPANYG
QHGTAVATVRAGNVIGGGDWALDRIVPDILRAFEQSQPVIIRNPHAIRPWQHVLEPLSGYL
LLAQKLYTDGAEYAEGWNFGPNDADATPVKNIVEQMVKYWGEGASWQLDGNAHPHEA

HYLKLDCSKAKMQLGWHPRWNLNTTLEYIVGWHKNWLSGTDMHEYSITEINNYMNTK
10000000000000000000000000000000000000000000000000000000000000000000000000000000000
00000000000000000000000000000000000000000000000000000000000000000000000000000000000
00000000000000000000000000000000000000000000000000000000000000000000000000000000000
00000000000000000000000000000000000000000111111100000000000000000000000000000000000
0000000000000000000000000
>Q96MX6_3i2n_0_356
MSAFEKPQIIAHIQKGFNYTVFDCKWVPCSAKFVTMGNFARGTGVIQLYEIQHGDLKLLREI
EKAKPIKCGTFGATSLQQRYLATGDFGGNLHIWNLEAPEMPVYSVKGHKEIINAIDGIGGL
GIGEGAPEIVTGSRDGTVKVWDPRQKDDPVANMEPVQGENKRDCWTVAFGNAYNQEER
VVCAGYDNGDIKLFDLRNMALRWETNIKNGVCSLEFDRKDISMNKLVATSLEGKFHVFDM
RTQHPTKGFASVSEKAHKSTVWQVRHLPQNRELFLTAGGAGGLHLWKYEYPIQRSKKDSE
GIEMGVAGSVSLLQNVTLSTQPISSLDWSPDKRGLCVCSSFDQTVRVLIVTKLNKI
11110000000000000000000000000000000011000000000000000000000000000000000000000000000
00000000000000000000000000000000000000000000000000000000000000000000000000000000000
00000000000000000000000000000000000000000000000000000000000000000000000000000000000
00000000000000000000000000000000000000000110000000000000000000000000000000000000000
0000000000000000000001111
>P07173_3t6d_0_355
MKQLIVNSVATVALASLVAGCFEPPPATTTQTGFRGLSMGEVLHPATVKAKKERDAQYPPA
LAAVKAEGPPVSQVYKNVKVLGNLTEAEFLRTMTAITEWVSPQEGCTYCHDENNLASEAK
YPYVVARRMLEMTRAINTNWTQHVAQTGVTCYTCHRGTPLPPYVRYLEPTLPLNNRETPT
HVERVETRSGYVVRLAKYTAYSALNYDPFTMFLANDKRQVRVVPQTALPLVGVSRGKERRP
LSDAYATFALMMSISDSLGTNCTFCHNAQTFESWGKKSTPQRAIAWWGIRMVRDLNMNYL
APLNASLPASRLGRQGEAPQADCRTCHQGVTKPLFGASRLKDYPELGPIKAAAK
11111111111111111111000000000000000000000000000000000000000000000000000000000000000
00000000000000000000000000000000000000000000000000000000000000000000000000000000000
00000000000000000000000000000000000000000000000000000000000000000000000000000000000
00000000000000000000000000000000000000000000000000000000000000000000000000000000000
000000000000000000000011
>P09394_1ydy_0_355
MKLTLKNLSMAIMMSTIVMGSSAMAADSNEKIVIAHRGASGYLPEHTLPAKAMAYAQGA
DYLEQDLVMTKDDNLVVLHDHYLDRVTDVADRFPDRARKDGRYYAIDFTLDEIKSLKFTEG
FDIENGKKVQTYPGRFPMGKSDFRVHTFEEEIEFVQGLNHSTGKNIGIYPEIKAPWFHHQEG
KDIAAKTLEVLKKYGYTGKDDKVYLQCFDADELKRIKNELEPKMGMELNLVQLIAYTDWN
ETQQKQPDGSWVNYNYDWMFKPGAMKQVAEYADGIGPDYHMLIEETSQPGNIKLTGMV
QDAQQNKLVVHPYTVRSDKLPEYTPDVNQLYDALYNKAGVNGLFTDFPDKAVKFLN
11111111111111111111111111110000000000000000000000000000000000000000000000000000000
00000000000000000000000000000000000000000000000000000000000000000000000000000000000
00000000000000000000000000000000000000000000000000000000000000000000000000000000000
00000000000000000000000000000000000000000000000000000000000000000000000000000000000
000000000000000000000000
>P36203_3hr8_0_355
MPEEKQKKSVLEKALKRIEENFGKGSIMILGDETQVQPVEVIPTGSLAIDIATGVGGYPRGRI
VEIFGQESSGKTTLALHAIAEAQKMGGVAAFIDAEHALDPVYAKNLGVDLKSLLISQPDHG
EQALEIVDELVRSGVVDLIVVDSVAALVPRAEIEGAMGDMQVGLQARLMSQALRKIAGSV
NKSKAVVIFTNQIRMKIGVMFGSPETTTGGLALKFYATMRMEVRRGEPIKEGKDVIGNVISV
KIVKNKVAPPFKTAQTYIIYGKGIDREYELFNIAVNEGIVDRKGSWYYYTTLKGEEVSLGQGS
SNAVQFLKDNPEIAGEIERRIREKYGLLSVEKEEQRKEKKSSGEEAS
11100000000000000000000000000000000000000000000000000000000000000000000000000000000
00000000000000000000000000000000000000000000000000000000000000000000000000000000000

00000000000000000000000000000000000000000000000000000000000000000000000000000000000
00000000000000000000000000000000000000000000000000000000000000000000000000000000000
000000000000011111111111
>Q12053_3hrq_1304_1659
EIKTTTTLHRVVEETTKPLGATLVVETDISRKDVNGLARGHLVDGIPLCTPSFYADIAMQVG
QYSMQRLRAGHPGAGAIDGLVDVSDMVVDKALVPHGKGPQLLRTTLTMEWPPKAAATT
RSAKVKFATYFADGKLDTEHASCTVRFTSDAQLKSLRRSVSEYKTHIRQLHDGHAKGQFMR
YNRKTGYKLMSSMARFNPDYMLLDYLVLNEAENEAASGVDFSLGSSEGTFAAHPAHVDAI
TQVAGFAMNANDNVDIEKQVYVNHGWDSFQIYQPLDNSKSYQVYTKMGQAKENDLVH
GDVVVLDGEQIVAFFRGLTLRSVPRGALRVVLQTTVKKADRQLGFKTMPSPPPPTTTM
00000000000000000000000000000000000000000000000000000000000000000000000011111111000
00000000000000000000000000000000000000000000000000110000000000000000000000000000000
00000000000000000000000000000000000000000000000000000000000000000000000000000000000
00000000000000000000000000000000000000000011000000000000000000000000000000000000000
111111111111111111111111
>Q96323_1gp6_0_355
MVAVERVESLAKSGIISIPKEYIRPKEELESINDVFLEEKKEDGPQVPTIDLKNIESDDEKIREN
CIEELKKASLDWGVMHLINHGIPADLMERVKKAGEEFFSLSVEEKEKYANDQATGKIQGYG
SKLANNASGQLEWEDYFFHLAYPEEKRDLSIWPKTPSDYIEATSEYAKCLRLLATKVFKALS
VGLGLEPDRLEKEVGGLEELLLQMKINYYPKCPQPELALGVEAHTDVSALTFILHNMVPGL
QLFYEGKWVTAKCVPDSIVMHIGDTLEILSNGKYKSILHRGLVNKEKVRISWAVFCEPPKDK
IVLKPLPEMVSVESPAKFPPRTFAQHIEHKLFGKEQEELVSEKND
10000000000000000000000000000000000000000000000000000000000000000000000000000000000
00000000000000000000000000000000000000000000000000000000000000000000000000000000000
00000000000000000000000000000000000000000000000000000000000000000000000000000000000
00000000000000000000000000000000000000000000000000000000000000000000000000000000000
000000000000000000111111
>A6A5N8_3bv6_0_354
MSKVNEITRESWILSTFPEWGTWLNEEIEQTVVEPNTFSMWWLGCTGIWLKSAGNTNLSID
FWCGTGKKTQKNRLMNTQHQMMRMGGVEALQPNLRTSIFPLDPFAIKEIDAVLASHDHA
DHIDVNVAAAVLQNCGEHVKFIGPQACVDLWLGWGVPQERCIVAKVGDVLEIGDVKIRV
LDSFDRTALVTLPKGVSSYDKAILDGMDERAVNYLIETSGGSVYHSGDSHYSNYYAKHGND
YQIDVALLSYGENPRGVTDKMTSSDVLRAAESLDCQVVVPFHHDIWANFQNDPREIEVLW
NMKKDRLQYQFAPFFWQVGGKYTYPTDKGRMHYQHFRGFQDIFKNEPELPYKAFL
10000000000000000000000000000000000000000000000000000000000000000000000000000000000
00000000000000000000000000000000000000000000000000000000000000000000000000000000000
00000000000000000000000000000000000000000000000000000000000000000000000000000000000
00000000000000000000000000000000000000000000000000000000000000000000000000000000000
00000000000000000000000
>A6LGN3_3taw_31_385
AVSETISETAWCLDGFERPTGVNPVIKPLPTKFYCPMREDSVAWEESDTFNPAATIYDGKIVV
MYRAEDNSAQGIGSRTSRLGYATSTDGIHFERDTKPAFYPAKDNQAENECPGGTEDPRIAM
TEDGTYVLLYTQWNRKVPRLAVATSKDLKHWTKFGPAFEKAYNGKFKDEATKSASLVTTLK
GDKQVIAKVNGKYFMYWGEKNVYAATSDNLIDWDPLLDENGELLKLFSPRSGYFDSQLTE
CGPPAILTKDGIVLLYNGKNEPGEKGDTAYPANSYCAGQALFDVNNPTKLIGRLDKPFLQP
TDDFEKSGQYPAGTVFVEGLVYYRNKWYLYYGCADSFVAVAVSDKQLNF
11111100000000000000000000000000000000000000000000000000000000000000000000000000000
00000000000000000000000000000000000000000000000000000000000000000000000000000000000
00000000000000000000000000000000000000000000000000000000000000000000000000000000000
00000000000000000000000000000000000000000000000000000000000000000000000000000000000
00000000000000000000000

>O25743_2qm6_24_378
SAASYPPIKNTKVGLALSSHPLASEIGQKVLEEGGNAIDAAVAIGFALAVVHPAAGNIGGGG
FAVIHLANGENVALDFREKAPLKATKNMFLDKQGNVVPKLSEDGYLAAGVPGTVAGMEA
MLKKYGTKKLSQLIDPAIKLAENGYAISQRQAETLKEARERFLKYSSSKKYFFKKGHLDYQE
GDLFVQKDLAKTLNQIKTLGAKGFYQGQVAELIEKDMKKNGGIITKEDLASYNVKWRKPV
VGSYRGYKIISMSPPSSGGTHLIQILNVMENADLSALGYGASKNIHIAAEAMRQAYADRSVY
MGDADFVSVPVDKLINKAYAKKIFDTIQPDTVTPSSQIKPGMGQLHEGSN
11111110000000000000000000000000000000000000000000000000000000000000000000000000000
00000000000000000000000000000000000000000000000000000000000000000000000000000000000
00000000000000000000000000000000000000000000000000000000000000000000000000000000000
00000000000000000000000000000000000000000000000000000000000000000000000000000000000
00000000000000000000000
>P07740_1luc_0_354
MKFGNFLLTYQPPELSQTEVMKRLVNLGKASEGCGFDTVWLLEHHFTEFGLLGNPYVAAA
HLLGATETLNVGTAAIVLPTAHPVRQAEDVNLLDQMSKGRFRFGICRGLYDKDFRVFGTD
MDNSRALMDCWYDLMKEGFNEGYIAADNEHIKFPKIQLNPSAYTQGGAPVYVVAESASTT
EWAAERGLPMILSWIINTHEKKAQLDLYNEVATEHGYDVTKIDHCLSYITSVDHDSNRAKD
ICRNFLGHWYDSYVNATKIFDDSDQTKGYDFNKGQWRDFVLKGHKDTNRRIDYSYEINPV
GTPEECIAIIQQDIDATGIDNICCGFEANGSEEEIIASMKLFQSDVMPYLKEKQ
00000000000000000000000000000000000000000000000000000000000000000000000000000000000
00000000000000000000000000000000000000000000000000000000000000000000000000000000000
00000000000000000000000000000000000000000000000000000000000000000000000000000000000
00000000000011111111111111111111111111110000000000000000000000000000000000000000000
00000000000000000000000
>P17443_1f0k_0_354
MSGQGKRLMVMAGGTGGHVFPGLAVAHHLMAQGWQVRWLGTADRMEADLVPKHGIEI
DFIRISGLRGKGIKALIAAPLRIFNAWRQARAIMKAYKPDVVLGMGGYVSGPGGLAAWSLG
IPVVLHEQNGIAGLTNKWLAKIATKVMQAFPGAFPNAEVVGNPVRTDVLALPLPQQRLA
GREGPVRVLVVGGSQGARILNQTMPQVAAKLGDSVTIWHQSGKGSQQSVEQAYAEAGQP
QHKVTEFIDDMAAAYAWADVVVCRSGALTVSEIAAAGLPALFVPFQHKDRQQYWNALPL
EKAGAAKIIEQPQLSVDAVANTLAGWSRETLLTMAERARAASIPDATERVANEVSRVARA
11111000000000000000000000000000000000000000000000000000000000000000000000000000000
00000000000000000000000000000000000000000000000000000000000000000000000000000000000
00000000000000000000000000000000000000000000000000000000000000000000000000000000000
00000000000000000000000000000000000000000000000000000000000000000000000000000000000
00000000000000000000000
>Q58361_3gmi_0_354
MLIGEIMDLNLKNFLEDREEIIRDAKRKDEKSFKDFKKIVEEIKERENKDKIVCDFTEYNPLH
KGHKYALEKGKEHGIFISVLPGPLERSGRGIPYFLNRYIRAEMAIRAGADIVVEGPPMGIMGS
GQYMRCLIKMFYSLGAEIIPRGYIPEKTMEKVIDCINKGYHIQVKPYKIICIETGEILGEKLNID
NYVIASMSQMIYKLNREGLKFNPKFVFVKRLEGISGTKIREAIFSGKFEDIKNMLPKTTLSILK
ELYDNGKLNELILKRFEDRILETANEYDLYEYLPSNVAEILEKKRPFNNIEEIKNSLPYGFSRH
FRERILSKLEARIPNETLSKYINNYPAKIKILAVKL
00000000000000000000000000000000000000000000000000000000000000000000000000000000000
00000000000000000000000000000000000000000000000000000000000000000000000000000000000
00000000000000000000000000000000000000000000000000000000000000000000000000000000000
00000000000000000000000000000000000000000000000000000000000000000000000000000000000
00000000000000000000001
>Q7CWZ6_3c9h_4_358
MRICLFLCLCLCMASPALAQVAVFPALSGKTDAQTLVVYSSLDEPLATPMIEGFQKANPDIA
VHYEDMLTGEIYDRIVKETDAGKKTADFAFSSAMDLQVKLSNDGYAQRSDLAMSARWPA

WANWRNTAYALTFEPAVFVYHKPSFTTEKPPATRAEFVDYLERHAKEVHGRIATYDIERSGV
GFLFMSRDQEQFGDIWSVIKAMGAAGVKVYSTSSAILERVSDGRFVLGYNILGSYAADWAS
RHPDVGIVLPKDYTVVMSRIGLVPEAAANPELGRRYLEFFMSKEGQTIMARQLQIPAVSPEV
AGENTANTMQAIHGAQLRPVPVSPGLMVYLDQVKRSRLIERWNEALRSQ
11111111111111000000000000000000000000000000000000000000000000000000000000000000000
00000000000000000000000000000000000000000000000000000000000000000000000000000000000
00000000000000000000000000000000000000000000000000000000000000000000000000000000000
00000000000000000000000000000000000000000000000000000000000000000000000000000000000
00000000000000000000001
>A1RHE8_3rpd_0_353
MKTRLNKTSLNQALLPTSTAGSLPKPLWLAEPETLWSPWKLQGEELITGKHDALRLSLQDQ
QLAGIDIVSDGEQTRQHFVTTFIEHLNGVDFSKRKIVKIRDRYDASVPTVVGPVSRQKSVFVE
DAKFLRKQTTQPIKWALPGPMTMIDTLYDDHYKSREKLAWEFAKILNEEAKELEAAGVDII
QFDEPAFNVFFDEVNDWGIACLERAIEGLKCETAVHICYGYGIKANTDWKKTLGSEWRQY
EEVFPKLQKSNIDIISLECHNSHVPMELLELIRGKKVMVGAIDVATDTIETAEEVADTLRKAL
KFVDADKLYPCTNCGMTPLSHQVTRGKLNALSAGAEIVRKELLALR
11111111100000000000000000000000000000000000000000000000000000000000000000000000000
00000000000000000000000000000000000000000000000000000000000000000000000000000000000
00000000000000000000000000000000000000000000000000000000000000000000000000000000000
00000000000000000000000000000000000000000000000000000000000000000000000000000000000
0000000000000000000000
>A7M0D0_4dqa_26_379
NDLLEPKVYFESKEYNFSVEDEMDVMTFDLVSRLSSATSSQVDVSYSVAEPSVVDEYNAKYG
TNYEMLDVSQVKLSSTTSSISSGKLYADNIEVELSGLEALKAGNSYVLPMRVHSSSVSTLSGT
NIAYFFFSKPLKITKAGNFSNHYISVKFPVGTFFSSFTYEALINVDYFLDNNTIMGTEGVMILR
IGDAGGGITPKDYLEVAGGQNYRVTKPLLTNRWYHVALTYDQPTGKTGIYVNGEKWAGSD
WGIDGFDPNSDMGFYIGRIYGFKWGERPFHGKMSEVRVWSVARTENQLKQNMLGVDPAS
EGLALYYKLDGSETQEGGVIKDATGRINGTTNGITIKTLDAPIAIN
11111000000000000000000000000000000000000000000000000000000000000000000000000000000
00000000000000000000000000000000000000000000000000000000000000000000000000000000000
00000000000000000000000000000000000000000000000000000000000000000000000000000000000
00000000000000000000000000000000000000000000000000000000000000000000000000000000000
0000000000000000000000
>P11655_4h5i_0_353
MKFVTASYNVGYPAYGAKFLNNDTLLVAGGGGEGNNGIPNKLTVLRVDPTKDTEKEQFHI
LSEFALEDNDDSPTAIDASKGIILVGCNENSTKITQGKGNKHLRKFKYDKVNDQLEFLTSVD
FDASTNADDYTKLVYISREGTVAAIASSKVPAIMRIIDPSDLTEKFEIETRGEVKDLHFSTDGK
VVAYITGSSLEVISTVTGSCIARKTDFDKNWSLSKINFIADDTVLIAASLKKGKGIVLTKISIKS
GNTSVLRSKQVTNRFKGITSMDVDMKGELAVLASNDNSIALVKLKDLSMSKIFKQAHSFAI
TEVTISPDSTYVASVSAANTIHIIKLPLNYANYTSMKQKISK
00000000000000000000000000000000000000000000000000000000000000000000000000000000000
00000000000000000000000000000000000000000000000000000000000000000000000000000000000
00000000000000000000000000000000000000000000000000000000000000000000000000000000000
00000000000000000000000000000000000000000000000000000000000000000000000000000000000
0000000000001111111111
>Q3IZQ3_3fdg_0_353
MTETIPVFDGHNDFLLRLLRNPANRETIWLKGDGTGHLDLPRMKEGGFAGGFFAIYVPSPQ
AHDAAHFEAMMDAPPFELPLPPMIRAEQAQPVALAMAGHLLWMERAARGRFKVCRTAA
EVRSCHADGIVSGIMHMEGAEAIGADLDALHLFHSLGLRSLGPVWSRPTVFGHGVPFRFPG
SPDTGEGLTEAGRRLVAECNRLKIMLDLSHLNEKGFDDVARLSDAPLVATHSNAHAVTPST
RNLTDRQLAMIRESRGMVGLNFATSFLREDGRRSAEMGWEPVLRHLDHLIDRLGEDHVG

MGSDFDGATIPQGIADVTGLPALQAAMRAHGYDEPLMRKLCHENWYGLLERSWG
11000000000000000000000000000000000000000000000000000000000011111000000000000000000
00000000000000000000000000000000000000000000000000000000000000000000000000000000000
00000000000000000000000000000000000000000000000000000000000000000000000000000000000
00000000000000000000000000000000000000000000000000000000000000000000000000000000000
000000000000000000000
>Q64V53_2ra8_0_353
MKRVFVFQDFKSQKFWSIDVRGTDVIVNYGKLGTDGQTQVKNFSSAGEAEKAAGKLIAEK
TKKGYVETLEEVAKEMKVEAKKYALSYDEAEEGVNLMDKILKDKKLPSLKQITIGCWGYEG
EDCSDIADGIVENKEKFAHFEGLFWGDIDFEEQEISWIEQVDLSPVLDAMPLLNNLKIKGTN
NLSIGKKPRPNLKSLEIISGGLPDSVVEDILGSDLPNLEKLVLYVGVEDYGFDGDMNVFRPLF
SKDRFPNLKWLGIVDAEEQNVVVEMFLESDILPQLETMDISAGVLTDEGARLLLDHVDKIK
HLKFINMKYNYLSDEMKKELQKSLPMKIDVSDSQEYDDDYSYPMITE
00000000000000000000000000000000000000000000000000000000000000000000000000000000000
00000000000000000000000000000000000000000000000000000000000000000000000000000000000
00000000000000000000000000000000000000000000000000000000000000000000000000000000000
00000000000000000000000000000000000000000000000000000000000000000000000000000000000
0000000000011111100000
>Q6B0I6_3dxt_0_353
METMKSKANCAQNPNCNIMIFHPTKEEFNDFDKYIAYMESQGAHRAGLAKIIPPKEWKAR
ETYDNISEILIATPLQQVASGRAGVFTQYHKKKKAMTVGEYRHLANSKKYQTPPHQNFEDL
ERKYWKNRIYNSPIYGADISGSLFDENTKQWNLGHLGTIQDLLEKECGVVIEGVNTPYLYFG
MWKTTFAWHTEDMDLYSINYLHLGEPKTWYVVPPEHGQRLERLARELFPGSSRGCGAFLR
HKVALISPTVLKENGIPFNRITQEAGEFMVTFPYGYHAGFNHGFNCAEAINFATPRWIDYG
KMASQCSCGEARVTFSMDAFVRILQPERYDLWKRGQDRAVVDHMEPRVPA
11111111110000000000000000000000000000000000000000000000000000000000000000000000000
00000000000000000000000000000000000000000000000000000000000000000000000000000000000
00000000000000000000000000000000000000000000000000000000000000000000000000000000000
00000000000000000000000000000000000000000000000000000000000000000000000000000000000
0000000001111111111111
>A9CI16_2i7g_0_352
MELGLYTFADVNPNPADGRGPEGARRLRELLEEIELADQVGLDVFGLGEHHRPDYVVSSPS
TVLAAAAVKTKNIRLTSAVSVLSSDDPVRVFQQFSTVDLLSNGRAEIMAGRGSFIESYPLFGY
DLEDYDVLFAEKLDLLLALREQEVVTWSGTKHPAINGRGVYPRPLQERLPVWIAVGGTPQS
VARAGAMGLPVALAIIGGEYRRFAPLFDLYHEAARRAGQEKTKLRTSINVHGFIADTTDKA
ADQFYGPQAEVMNRIGRERGWGPTNRAHFDAARGPEGNLFLGEPELVAEKIIKAHGVFK
NDRFLLQMAIGLMPHDQIMRGIELYGTKVAPLVRKELTGSADPVKATA
00000000000000000000000000000000000000000000000000000000000000000000000000000000000
00000000000000000000000000000000000000000000000000000000000000000000000000000000000
00000000000000000000000000000000000000000000000000000000000000000000000000000000000
00000000000000000000000000000000000000000000000000000000000000000000000000000000000
000000000000111111111
>B5RX19_3lop_33_385
ADVSVIQSLPLSGSQAVTGRALNAGARLYFDWLNLNGGINGETIRLVARDDEQKIEQTVRN
VRDMARVDNPVALLTVVGTANVEALMREGVLAEARLPLVGPATGASSMTTDPLVFFPIKAS
YQQEIDKMITALVTIGVTRIGVLYQEDALGKEATTGVERTLKAHALAITAMASYPRNTANV
GPAVDKLLAADVQAIFLGATAEPAAQFVRQYRARGGEAQLLGLSSIDPGILQKVAGLDAVR
GYSLALVMPNPGKSVNPVIREFNRARAAVGAKDVDLSFRAVEGFVAAKVLAEAIRRAGPK
PTREQVRHALTELRDYDVGGGFTVDFTDRSRPGSHYIELGVVGPNGLVIQ
10000000000000000000000000000000000000000000000000000000000000000000000000000000000
00000000000000000000000000000000000000000000000000000000000000000000000000000000000

00000000000000000000000000000000000000000000000000000000000000000000000000000000000
00000000000000000000000000000000000000000000000000000000000000000000000000000000000
00000000000000000000
>B8FRW3_3db2_0_352
MYNPVGVAAIGLGRWAYVMADAYTKSEKLKLVTCYSRTEDKREKFGKRYNCAGDATMEA
LLAREDVEMVIITVPNDKHAEVIEQCARSGKHIYVEKPISVSLDHAQRIDQVIKETGVKFLC
GHSSRRLGALRKMKEMIDTKEIGEVSSIEAVFSNERGLELKKGNWRGEPATAPGGPLTQLGV
HQIDNLQFLLGPVARVFNFGKPMYTEVENITVNQTLLEFEDGKQAYLGTNWACPGVFSINV
YGTKANLFYQLDFSWWSNSDVTDEHSTLIKREFASMSDDPDNRILRDVKVDFESVDHLRVE
VEEVADVIRNGGETEIGAEASLRNLAVVLAAVKSVHEKRPVEIAEIIG
00000000000000000000000000000000000000000000000000000000000000000000000000000000000
00000000000000000000000000000000000000000000000000000000000000000000000000000000000
00000000000000000000000000000000000000000000000000000000000000000000000000000000000
00000000000000000000000000000111111000000000000000000000000000000000000000000000000
00000000000000000000
>P43591_2gaa_0_352
MVDTHKLADDVLQLLDNRIEDNYRVCVILVGSPGSGKSTIAEELCQIINEKYHTFLSEHPNV
IEVNDRLKPMVNLVDSLKTLQPNKVAEMIENQGLFKDHVEDVNFQPVKYSALTSNNEECT
AVVARGGTANAIRIAAVDNPVNVNKLAQDSINIAQIVPMDGFHLSRRCLDLFKDPQTAHK
RRGSPSTFDSNNFLQLCKILAKTSLCKVSSHHKFYSTSSVFEKLSKTFSQTIPDIFVPGFNHAL
KDPTPDQYCISKFTRIVILEGLYLLYDQENWKKIYKTLADTGALLVYKIDIDYEATEERVAKR
HLQSGLVTTIAEGREKFRSNDLLNGRDIDNHLIKVDNIVHIRND
10000000000000000000000000000000000000000000000000000000000000000000000000000000000
00000000000000000000000000000011111000000000000000000001111111111111000000000000000
00000000000000000000000000000000000000000000011111100000000000000000000000000000000
00000000000000000000000000000000000000000000000000000000000000000000000000000000000
00000000000000000000
>P76116_3vgz_0_352
MHLRHLFSSRLRGSLLLGSLLVVSSFSTQAAEEMLRKAVGKGAYEMAYSQQENALWLATSQ
SRKLDKGGVVYRLDPVTLEVTQAIHNDLKPFGATINNTTQTLWFGNTVNSAVTAIDAKTGE
VKGRLVLDDRKRTEEVRPLQPRELVADDATNTVYISGIGKESVIWVVDGGNIKLKTAIQNTG
KMSTGLALDSEGKRLYTTNADGELITIDTADNKILSRKKLLDDGKEHFFINISLDTARQRAFI
TDSKAAEVLVVDTRNGNILAKVAAPESLAVLFNPARNEAYVTHRQAGKVSVIDAKSYKVV
KTFDTPTHPNSLALSADGKTLYVSVKQKSTKQQEATQPDDVIRIAL
11111111111111111111111111111111000000000000000000000000000000000000000000000000000
00000000000000000000000000000000000000000000000000000000000000000000000000000000000
00000000000000000000000000000000000000000000000000000000000000000000000000000000000
00000000000000000000000000000000000000000000000000000000000000000000000000000000000
00000000000000000000
>Q13946_1zkl_129_481
SNSLNILDDDYNGQAKCMLEKVGNWNFDIFLFDRLTNGNSLVSLTFHLFSLHGLIEYFHLD
MMKLRRFLVMIQEDYHSQNPYHNAVHAADVTQAMHCYLKEPKLANSVTPWDILLSLIAA
ATHDLDHPGVNQPFLIKTNHYLATLYKNTSVLENHHWRSAVGLLRESGLFSHLPLESRQQ
METQIGALILATDISRQNEYLSLFRSHLDRGDLCLEDTRHRHLVLQMALKCADICNPCRTW
ELSKQWSEKVTEEFFHQGDIEKKYHLGVSPLCDRHTESIANIQIGFMTYLVEPLFTEWARFSN
TRLSQTMLGHVGLNKASWKGLQREQSSSEDTDAAFELNSQLLPQENRLS
11111111100000000000000000000000000000000000000000000000000000000000000000000000000
00000000000000000000000000000000000000000000000000000000000000000000000000000000000
00000000000000000000000000000000000000000000000000000000000000000000000000000000000
00000000000000000000000000000000000000000000000000000000000000000000000000000111111
11111111111111111111

>Q97VU1_2rau_0_352
MYEEWKIVKREAPILGNDQLIENIWKMKREDSPYDIISLHKVNLIGGGNDAVLILPGTWSSG
EQLVTISWNGVHYTIPDYRKSIVLYLARNGFNVYTIDYRTHYVPPFLKDRQLSFTANWGWST
WISDIKEVVSFIKRDSGQERIYLAGESFGGIAALNYSSLYWKNDIKGLILLDGGPTKHGIRPKF
YTPEVNSIEEMEAKGIYVIPSRGGPNNPIWSYALANPDMPSPDPKYKSISDFLMDSLYVTGSA
NPYDYPYSKKEDMFPILASFDPYWPYRLSLERDLKFDYEGILVPTIAFVSERFGIQIFDSKILPS
NSEIILLKGYGHLDVYTGENSEKDVNSVVLKWLSQQR
10000000000000000000000000000000000000000000000000000000000000000000000000000000000
00000000000000000000000000000000000000000000000000000000000000000000000000000000000
00000000000000000001100000000000000000000000000000000000000000000000000000000000000
00000000000000000000000000000000000000000000000000000000000000000000000000000000000
000000000000000000000
>Q9UM73_4foc_1057_1409
VYRRKHQELQAMQMELQSPEYKLSKLRTSTIMTDYNPNYCFAGKTSSISDLKEVPRKNITLI
RGLGHGAFGEVYEGQVSGMPNDPSPLQVAVKTLPEVCSEQDELDFLMEALIISKFNHQNIV
RCIGVSLQSLPRFILLELMAGGDLKSFLRETRPRPSQPSSLAMLDLLHVARDIACGCQYLEEN
HFIHRDIAARNCLLTCPGPGRVAKIGDFGMARDIYRASYYRKGGCAMLPVKWMPPEAFME
GIFTSKTDTWSFGVLLWEIFSLGYMPYPSKSNQEVLEFVTSGGRMDPPKNCPGPVYRIMTQC
WQHQPEDRPNFAIILERIEYCTQDPDVINTALPIEYGPLVEEEEK
11111111111111111111111111111111111000000000000000000000000000000000000000000001111
11000000000000000000000000000000000000000000000000000000000000000000000000000000000
00000000000000000000000000000000000000000000000000000000111111110000000000000000000
00000000000000000000000000000000000000000000000000000000000000000000000000000000000
000000000000011111111
>A9WC34_4e3e_0_351
MSAKTNPGNFFEDFRLGQTIVHATPRTITEGDVALYTSLYGSRFALTSSTPFAQSLGLERAPID
SLLVFHIVFGKTVPDISLNAIANLGYAGGRFGAVVYPGDTLSTTSKVIGLRQNKDGKTGVVY
VHSVGVNQWDEVVLEYIRWVMVRKRDPNAPAPETVVPDLPDSVPVTDLTVPYTVSAANY
NLAHAGSNYLWDDYEVGEKIDHVDGVTIEEAEHMQATRLYQNTARVHFNLHVEREGRFG
RRIVYGGHIISLARSLSFNGLANALSIAAINSGRHTNPSFAGDTIYAWSEILAKMAIPGRTDIG
ALRVRTVATKDRPCHDFPYRDAEGNYDPAVVLDFDYTVLMPRRG
10000000000000000000000000000000000000000000000000000000000000000000000000000000000
00000000000000000000000000000000000000000000000000000000000000000000000000000000000
00000000000000000000000000000000000000000000000000000000000000000000000000000000000
00000000000000000000000000000000000000000000000000000000000000000000000000000000000
00000000000000000000
>O66740_2zsj_0_351
MNRWQGIIKQYKKYLPVDENTPIVTLYEGNTPLIEADNLARAIGFKGKIYLKYEGLNPTGSF
KDRGMTLAISKAVEAGKRAVICASTGNTSASAAAYAARAGLRAYVLLPKGAVAIGKLSQA
MIYGAKVLAIQGTFDDALNIVRKIGENFPVEIVNSVNPYRIEGQKTAAFEICDTLGEAPDYH
FIPVGNAGNITAYWKGFKIYYEEGKITKLPRMMGWQAEGAAPIVKGYPIKNPQTIATAIKIG
NPYSWKSALKAAQESGGKIDAVSDSEILYAYKLIASTEGVFCEPASAASVAGLIKLVREGFFK
GGEVVTCTLTGNGLKDPDTAIKVCEEPITVPPDFDEVVKVLGF
11000000000000000000000000000000000000000000000000000000000000000000000000000000000
00000000000000000000000000000000000000000000000000000000000000000000000000000000000
00000000000000000000000000000000000000000000000000000000000000000000000000000000000
00000000000000000000000000000000000000000000000000000000000000000000000000000000000
00000000000000000000
>Q43576_3alf_25_376
QNVKGGYWFKDSGLALNNIDSTLFTHLFCAFADLNPQLNQLIISPENQDSFRQFTSTVQRK
NPSVKTFLSIAGGRANSTAYGIMARQPNSRKSFIDSSIRLARQLGFHGLDLDWEYPLSAADM

TNLGTLLNEWRTAINTEARNSGRAALLLTAAVSNSPRVNGLNYPVESLARNLDWINLMAY
DFYGPNWSPSQTNSHAQLFDPVNHVSGSDGINAWIQAGVPTKKLVLGIPFYGYAWRLVNA
NIHGLRAPAAGKSNVGAVDDGSMTYNRIRDYIVESRATTVYNATIVGDYCYSGSNWISYDD
TQTVRNKVNYVKGRGLLGYFAWHVAGDQNWGLSRTASQTWGVSFQEMK
00000000000000000000000000000000000000000000000000000000000000000000000000000000000
00000000000000000000000000000000000000000000000000000000000000000000000000000000000
00000000000000000000000000000000000000000000000000000000000000000000000000000000000
00000000000000000000000000000000000000000000000000000000000000000000000000000000000
00000000000000001111
>Q5AU62_1ti7_0_351
MAQQKKTIAVVNATGRQAASLIRVAAAVGHHVRAQVHSLKGLIAEELQAIPNVTLFQGPL
LNNVPLMDTLFEGAHLAFINTTSQAGDEIAIGKDLADAAKRAGTIQHYIYSSMPDHSLYGP
WPAVPMWAPKFTVENYVRQLGLPSTFVYAGIYNNNFTSLPYPLFQMELMPDGTFEWHAPF
DPDIPLPWLDAEHDVGPALLQIFKDGPQKWNGHRIALTFETLSPVQVCAAFSRALNRRVTY
VQVPKVEIKVNIPVGYREQLEAIEVVFGEHKAPYFPLPEFSRPAAGSPKGLGPANGKGAGA
GMMQGPGGVISQRVTDEARKLWSGWRDMEEYAREVFPIEEEANGLDWML
11000000000000000000000000000000000000000000000000000000000000000000000000000000000
00000000000000000000000000000000000000000000000000000000000000000000000000000000000
00000000000000000000000000000000000000000000000000000000000000000000000000000000000
00000000000000000000000000000000011111111111111111111111111000000000000000000000000
00000000000000000000
>Q5FA19_3fvq_0_351
MTAALHIGHLSKSFQNTPVLNDISLSLDPGEILFIIGASGCGKTTLLRCLAGFEQPDSGEISLS
GKTIFSKNTNLPVRERRLGYLVQEGVLFPHLTVYRNIAYGLGNGKGRTAQERQRIEAMLELT
GISELAGRYPHELSGGQQQRVALARALAPDPELILLDEPFSALDEQLRRQIREDMIAALRAN
GKSAVFVSHDREEALQYADRIAVMKQGRILQTASPHELYRQPADLDAALFIGEGIVFPAALN
ADGTADCRLGRLPVQSGAPAGTRGTLLIRPEQFSLHPHSAPAASIHAVVLKTTPKARHTEIS
LRAGQTVLTLNLPSAPTLSDGISAVLHLDGPALFFPGNTL
10000000000000000000000000000000000000000000000000000000000000000000000000000000000
00000000000000000000000000000000000000000000000000000000000000000000000000000000000
00000000000000000000000000000000000000000000000000000000000000000000000000000000000
00000000000000000000000000000000000000000000000000000000000000000000000000000000000
00000000000000000001
>Q5P5I1_2ivf_0_351
MTYVQDGNKSELRKAKRQLVTVIDLNKCLGCQTCTVACKNIWTKRPGTEHMRWNNVTT
YPGKGYPRDYERKGGGFLRGEPQPGVLPTLIDSGDDFQFNHKEVFYEGKGQTVHFHPTSKS
TGKDPAWGYNWDEDQGGGKWPNPFFFYLARMCNHCTNPACLAACPTGAIYKREDNGIV
LVDQERCKGHRHCVEACPYKAIYFNPVSQTSEKCILCYPRIEKGIANACNRQCPGRVRAFG
YLDDTTSHVHKLVKKWKVALPLHAEYGTGPNIYYVPPMGARGFGEDGEITDKTRIPLDVLE
GLFGPEVKRVLAVLHTERENMRAGRGSELMDLLISKKWSDRFGGFTNDPLTQS
11111111111111100000000000000000000000000000000000000000000000000000000000000000000
00000000000000000000000000000000000000000000000000000000000000000000000000000000000
00000000000000000000000000000000000000000000000000000000000000000000000000000000000
00000000000000000000000000000000000000000000000000000000000000000000000000000000000
00000000000000000000
>Q86YC2_2w18_834_1185
SVEQTETAELPASDSINPGNLQLVSELKNPSGSCSVDVSAMFWERAGCKEPCIITACEDVVSL
WKALDAWQWEKLYTWHFAEVPVLQIVPVPDVYNLVCVALGNLEIREIRALFCSSDDESEK
QVLLKSGNIKAVLGLTKRRLVSSSGTLSDQQVEVMTFAEDGGGKENQFLMPPEETILTFAEV
QGMQEALLGTTIMNNIVIWNLKTGQLLKKMHIDDSYQASVCHKAYSEMGLLFIVLSHPCA
KESESLRSPVFQLIVINPKTTLSVGVMLYCLPPGQAGRFLEGDVKDHCAAAILTSGTIAIWDL

LLGQCTALLPPVSDQHWSFVKWSGTDSHLLAGQKDGNIFVYHYS
11111111111111111110000000000000000000000001111000000000000000000000000000000000000
00000000000000000000000000000000111111000000000000000000000000000000000000000000000
00000000000000000000000000000000000000001110000000000000000000000000000000011111111
11110000000000000000000000000000000000000000000000000000000000000000000110000000000
0000000000000000000
>Q970S6_2eqa_0_351
MTQIIKIDPLNPEIDKIKIAADVIRNGGTVAFPTETVYGLGANAFDGNACLKIFQAKNRPVD
NPLIVHIADFNQLFEVAKDIPDKVLEIAQIVWPGPLTFVLKKTERVPKEVTAGLDTVAVRMP
AHPIALQLIRESGVPIAAPSANLATRPSPTKAEDVIVDLNGRVDVIIDGGHTFFGVESTIINVT
VEPPVLLRPGPFTIEELKKLFGEIVIPEFAQGKKEAEIALAPGMKYKHYAPNTRLLLVENRNIF
KDVVSLLSKKYKVALLIPKELSKEFEGLQQIILGSDENLYEVARNLFDSFRELDKLNVDLGIMI
GFPERGIGFAIMNRARKASGFSIIKAISDVYKYVNI
10000000000000000000000000000000000000000000000000000000000000000000000000000000000
00000000000000000000000000000000000000000000000000000000000000000000000000000000000
00000000000000000000000010000000000000000000000011111111111111111100000000000000000
00000000000000000000000000000000000000000000000000000000000000000000000000000000000
0000000000000000001
>Q9HXY6_3pmo_1_352
MSTLSYTLGQLAAHVGAEVRGDADLPIQGLATLQEAGPAQLSFLANPQYRKYLPESRAGA
VLLTAADADGFAGTALVVANPYLAYASLSHLFDRKPKAAAGIHPTAIVAADAEVDPSASVG
AYAVIESGARIGAGVSIGAHCVIGARSVIGEGGWLAPRVTLYHDVTIGARVSIQSGAVIGGEG
FGFANEKGVWQKIAQIGGVTIGDDVEIGANTTIDRGALSDTLIGNGVKLDNQIMIAHNVQI
GDHTAMAACVGISGSAKIGRHCMLAGGVGLVGHIEICDNVFVTGMTMVTRSITEPGSYSSG
TAMQPAAEWKKSAARIRQLDDMARRLQQLEKRLAAVTSSGDASSDA
00000000000000000000000000000000000000000000000000000000000000000000000000000000000
00000000000000000000000000000000000000000000000000000000000000000000000000000000000
00000000000000000000000000000000000000000000000000000000000000000000000000000000000
00000000000000000000000000000000000000000000000000000000000000000000000000000000000
00000001111111111111
>P17612_3l9m_0_350
MGNAAAAKKGSEQESVKEFLAKAKEDFLKKWESPAQNTAHLDQFERIKTLGTGSFGRVM
LVKHKETGNHYAMKILDKQKVVKLKQIEHTLNEKRILQAVNFPFLVKLEFSFKDNSNLYMV
MEYVPGGEMFSHLRRIGRFSEPHARFYAAQIVLTFEYLHSLDLIYRDLKPENLLIDQQGYIQV
TDFGFAKRVKGRTWTLCGTPEYLAPEIILSKGYNKAVDWWALGVLIYEMAAGYPPFFADQP
IQIYEKIVSGKVRFPSHFSSDLKDLLRNLLQVDLTKRFGNLKNGVNDIKNHKWFATTDWIAI
YQRKVEAPFIPKFKGPGDTSNFDDYEEEEIRVSINEKCGKEFSEF
11111111111000000000000000000000000000000000000000000000000000000000000000000000000
00000000000000000000000000000000000000000000000000000000000000000000000000000000000
00000000000000000000000000000000000000000000000000000000000000000000000000000000000
00000000000000000000000000000000000000000000000000000000000000000000000000000000000
000000000000000000
>Q180C0_3g68_0_350
MTIQDYMLETPVRMREIISNADSLFNEVKRTNLKKIIITGSGTSYHSGVQVQPYLQNLLDIDV
VKMYPFMITEDTFKFDNENTLVVGVSQGGSSYSTYNAMKLAEDKGCKIASMAGCKNALID
EISDYILTVNCGEEKSGAKTKGYYCTKLNLMLLGLQIAREKGIISSEKYNEEINKILDAINRFE
AVYKLSKQWIERNKEKLVNSKEIRIIGHSDIYGDTLEAALKLLETMRIPVTGYEFEEFIHGIYN
AINSDSTIFILDTGKEPRVTKMIDVLSGWTENVFAIGRDVTENDKNLKIDITDNPYYQTFNFI
VPIQLICGEIPTLRGVDPSVPKDTRFHMKLGSKKLNK
00000000000000000000000000000000000000000000000000000000000000000000000000000000000
00000000000000000000000000000000000000000000000000000000000000000000000000000000000

00000000000000000000000000000000000000000000000000000000000000000000000000000000000
00000000000000000000000000000000000000000000000000000000000000000000000000000000000
000000000000000001
>Q8N8N7_2zb7_0_350
MIVQRVVLNSRPGKNGNPVAENFRMEEVYLPDNINEGQVQVRTLYLSVDPYMRCRMNED
TGTDYITPWQLSQVVDGGGIGIIEESKHTNLTKGDFVTSFYWPWQTKVILDGNSLEKVDPQ
LVDGHLSYFLGAIGMPGLTSLIGIQEKGHITAGSNKTMVVSGAAGACGSVAGQIGHFLGCS
RVVGICGTHEKCILLTSELGFDAAINYKKDNVAEQLRESCPAGVDVYFDNVGGNISDTVISQ
MNENSHIILCGQISQYNKDVPYPPPLSPAIEAIQKERNITRERFLVLNYKDKFEPGILQLSQWF
KEGKLKIKETVINGLENMGAAFQSMMTGGNIGKQIVCISEEISL
00000000000000000000000000000000000000000000000000000000000000000000000000000000000
00000000000000000000000000000000000000000000000000000000000000000000000000000000000
00000000000000000000000000000000000000000000000000000000000000000000000000000000000
00000000000000000000000000000000000000000000000000000000000000000000000000000000000
000000000000000001
>Q9FK51_1z84_0_350
MTSPSHASDRGGGDGDSVENQSPELRKDPVTNRWVIFSPARAKRPTDFKSKSPQNPNPKPS
SCPFCIGREQECAPELFRVPDHDPNWKLRVIENLYPALSRNLETQSTQPETGTSRTIVGFGFH
DVVIESPVHSIQLSDIDPVGIGDILIAYKKRINQIAQHDSINYIQVFKNQGASAGASMSHSHS
QMMALPVVPPTVSSRLDGTKDYFEETGKCCLCEAKSKHFVIDESSHFVSVAPFAATYPFEIWI
IPKDHSSHFHHLDDVKAVDLGGLLKLMLQKIAKQLNDPPYNYMIHTSPLKVTESQLPYTH
WFLQIVPQLSGVGGFEIGTGCYINPVFPEDVAKVMREVSLT
11111111111111111111000000000000000000011110000000001111111000000000000000000000000
00000000000000000000000011111111000000000000000000000000000000000000000000000000000
00000000000000000000000000000000000000000000000000000000000000000000000000000000000
00000000000000000000000000000000000000000000000000000000000000000000000000000000000
000000000000000000
>O58107_2cz9_0_349
MIKVKSPGRVNLIGEHTDYTYGYVMPMAINLYTKIEAEKHGEVILYSEHFGEERKFSLNDLR
KENSWIDYVKGIFWVLKESDYEVGGIKGRVSGNLPLGAGLSSSASFEVGILETLDKLYNLKL
DSLSKVLLAKKAENEFVGVPCGILDQFAVVFGREGNVIFLDTHTLDYEYIPFPKDVSILVFYT
GVRRELASSEYAERKHIAEESLKILGKGSSKEVREGELSKLPPLHRKFFGYIVRENARVLEVR
DALKEGNVEEVGKILTTAHWDLAKNYEVSCKELDFFVERALKLGAYGARLTGAGFGGSAI
ALVDKEDAETIGEEILREYLKRFPWKARHFIVEPSDGVGI
00000000000000000000000000000000000000000000000000000000000000000000000000000000000
00000000000000000000000000000000000000000000000000000000000000000000000000000000000
00000000000000000000000011110000000000000000000000000000000000000000000000000000000
00000000000000000000000000000000000000000000000000000000000000000000000000000000000
00000000000000000
>P0AB91_1n8f_0_349
MNYQNDDLRIKEIKELLPPVALLEKFPATENAANTVAHARKAIHKILKGNDDRLLVVIGPC
SIHDPVAAKEYATRLLALREELKDELEIVMRVYFEKPRTTVGWKGLINDPHMDNSFQINDG
LRIARKLLLDINDSGLPAAGEFLDMITPQYLADLMSWGAIGARTTESQVHRELASGLSCPVG
FKNGTDGTIKVAIDAINAAGAPHCFLSVTKWGHSAIVNTSGNGDCHIILRGGKEPNYSAK
HVAEVKEGLNKAGLPAQVMIDFSHANSSKQFKKQMDVCADVCQQIAGGEKAIIGVMVES
HLVEGNQSLESGEPLAYGKSITDACIGWEDTDALLRQLANAVKARRG
11111110000000000000000000000000000000000000000000000000000000000000000000000000000
00000000000000000000000000000000000000000000000000000000000000000000000000000000000
00000000000000000000000000000000000000000000000000000000000000000000000000000000000
00000000000000000000000000000000000000000000000000000000000000000000000000000000000
00000000000000000

>Q08601_4f6p_82_431
EQAKAQLSNGYNNPNVNASNMYGPPQNMSLPPPQTQTIQGTDQPYQYSQCTGRRKALII
GINYIGSKNQLRGCINDAHNIFNFLTNGYGYSSDDIVILTDDQNDLVRVPTRANMIRAMQW
LVKDAQPNDSLFLHYSGHGGQTEDLDGDEEDGMDDVIYPVDFETQGPIIDDEMHDIMVKP
LQQGVRLTALFDSCHSGTVLDLPYTYSTKGIIKEPNIWKDVGQDGLQAAISYATGNRAALIG
SLGSIFKTVKGGMGNNVDRERVRQIKFSAADVVMLSGSKDNQTSADAVEDGQNTGAMSH
AFIKVMTLQPQQSYLSLLQNMRKELAGKYSQKPQLSSSHPIDVNLQFIM
11111111110111100000000000000000000000000000000000000000000000000000000000000000000
00000000000000000000000000000000000000000000000000000000000111111111100000000000000
00000000000000000000000000000000000000000000000000011111111111111111111111111111111
11111111111111111110000000000000111111111111100000000000000000000000000000000000000
000000000000000000
>Q7SIC2_1juh_0_349
DTSSLIVEDAPDHVRPYVIRHYSHARAVTVDTQLYRFYVTGPSSGYAFTLMGTNAPHSDAL
GVLPHIHQKHYENFYCNKGSFQLWAQSGNETQQTRVLSSGDYGSVPRNVTHTFQIQDPDT
EMTGVIVPGGFEDLFYYLGTNATDTTHTPYIPSSSDSSSTTGPDSSTISTLQSFDVYAELSFTPR
TDTVNGTAPANTVWHTGANALASTAGDPYFIANGWGPKYLNSQYGYQIVAPFVTATQAQ
DTNYTLSTISMSTTPSTVTVPTWSFPGACAFQVQEGRVVVQIGDYAATELGSGDVAFIPGGV
EFKYYSEAYFSKVLFVSSGSDGLDQNLVNGGEEWSSVSFPADW
11000000000000000000000000000000000000000000000000000000000000000000000000000000000
00000000000000000000000000000000000000000000000000000000000000000000000111111111111
00000000000000000000000000000000000000000000000000000000000000000000000000000000000
00000000000000000000000000000000000000000000000000000000000000000000000000000000000
000000000000000000
>Q88H32_1u7h_0_349
MTYFIDVPTMSDLVHDIGVAPFIGELAAALRDDFKRWQAFDKSARVASHSEVGVIELMPVA
DKSRYAFKYVNGHPANTARNLHTVMAFGVLADVDSGYPVLLSELTIATALRTAATSLMAA
QALARPNARKMALIGNGAQSEFQALAFHKHLGIEEIVAYDTDPLATAKLIANLKEYSGLTIR
RASSVAEAVKGVDIITTVTADKAYATIITPDMLEPGMHLNAVGGDCPGKTELHADVLRNAR
VFVEYEPQTRIEGEIQQLPADFPVVDLWRVLRGETEGRQSDSQVTVFDSVGFALEDYTVLRY
VLQQAEKRGMGTKIDLVPWVEDDPKDLFSHTRGRAGKRRIRRVA
00000000000000000000000000000000000000000000000000000000000000000000000000000000000
00000000000000000000000000000000000000000000000000000000000000000000000000000000000
00000000000000000000000000000000000000000000000000000000000000000000000000000000000
00000000000000000000000000000000000000000000000000000000000000000000000000000000000
000000000011111111
>Q8IJP2_3s6b_167_516
EKEDHLKTIVKKHLSPENFDPTNRKYWVYDDHLKNFVNFKFTGDVRPWPLSKINHVPSHI
ERPDYAISSIPESELIYKRKSDIYVNNEEEIQRIREACILGRKTLDYAHTLVSPGVTTDEIDRKV
HEFIIKNNAYPSTLNYYKFPKSCCTSVNEIVCHGIPDYRPLKSGDIINIDISVFYKGVHSDLNE
TYFVGDINDVPKEGKELVETCYFSLMEAIKKCKPGMFYKNIGTLIDAYVSKKNFSVVRSYSG
HGVGKLFHSNPTVPHFKKNKAVGIMKPGHVFTIEPMINQGHYSDVLWPDQWTSATSDGK
LSAQFEHTLLITNNGVEILTKRTQDSPPLGFDTKDELYYN
11110000000000000000000000000000000000000000000000000000000000000000000000000000000
00000000000000000000000000000000000000000000000000000000000000000000000000000000000
00000000000000000000000000000000000000000000000000000000000000000000000000000000000
00000000000000000000000000000000000000000000000000000000000000000000000000000000000
000000000000000001
>Q9KUL5_2gu1_62_411
QPKRIHYMVKVGDTLSGIFAQLGVPYSILQKILSVDLDHLQLDMIQPGEELELMMDDMGQ
LSRLIYHMSIVEKAIYTRENDGSFSYDFQEISGEWREILFSGEINGSFSVSARRVGLTSSQVANI

TQVMKDKIDFSRSLRAGDRFDILVKQQYLGEHNTGNSEIKAISFKLAKGDVSAFLAEDGRFY
DRAGNSLERAFNRYPVDKAYRQITSGFNPKRKHPVTGRVVPHNGTDFATPIGAPVYSTGDG
KVIVVRKHPYAGNYLVIEHNSVYKTRYLHLDKILVKKGQLVKRGQKIALAGATGRLTGPHL
HFEVLVRNRPVDAMKADLPIAKSLSSNQKTSFLARVSEFDH
11100000000000000000000000000000000000000000000000000000000000000000000000000000000
00000000000000000000000000000000000000000000000000000000001000000000000000000000000
00000000000000000000000000000000000000000000000000000000000000000000000000000000000
00000000000000000000000000000000000000000000000000000000000000000000000000000001111
111111111111111111
>P15555_1ikg_31_379
ADLPAPDDTGLQAVLHTALSQGAPGAMVRVDDNGTIHQLSEGVADRATGRAITTTDRFRV
GSVTKSFSAVVLLQLVDEGKLDLDASVNTYLPGLLPDDRITVRQVMSHRSGLYDYTNDMFA
QTVPGFESVRNKVFSYQDLITLSLKHGVTNAPGAAYSYSNTNFVVAGMLIEKLTGHSVATEY
QNRIFTPLNLTDTFYVHPDTVIPGTHANGYLTPDEAGGALVDSTEQTVSWAQSAGAVISSTQ
DLDTFFSALMSGQLMSAAQLAQMQQWTTVNSTQGYGLGLRRRDLSCGISVYGHTGTVQG
YYTYAFASKDGKRSVTALANTSNNVNVLNTMARTLESAFCGKPTT
11000000000000000000000000000000000000000000000000000000000000000000000000000000000
00000000000000000000000000000000000000000000000000000000000000000000000000000000000
00000000000000000000000000000000000000000000000000000000000000000000000000000000000
00000000000000000000000000000000000000000000000000000000000000000000000000000000000
00000000000000011
>Q29599_1px5_0_348
MELRHTPARDLDKFIEDHLLPNTCFRTQVKEAIDIVCRFLKERCFQGTADPVRVSKVVKGGS
SGKGTTLRGRSDADLVVFLTKLTSFEDQLRRRGEFIQEIRRQLEACQREQKFKVTFEVQSPRR
ENPRALSFVLSSPQLQQEVEFDVLPAFDALGQWTPGYKPNPEIYVQLIKECKSRGKEGEFST
CFTELQRDFLRNRPTKLKSLIRLVKHWYQTCKKTHGNKLPPQYALELLTVYAWEQGSRKTD
FSTAQGFQTVLELVLKHQKLCIFWEAYYDFTNPVVGRCMLQQLKKPRPVILDPADPTGNVG
GGDTHSWQRLAQEARVWLGYPCCKNLDGSLVGAWTMLQKI
00000000000000000000000000000000011100000000000000000000000000000000000000000000000
00000000000000000000000000000000000000001111100000000000000000000000000000000000000
00000000000000000000000000000000000000000000000000000000000000000000000000000000000
00000000000000000000000000000000000000000000000000000000000000000000000000000000000
00000000000000000
>Q2T5S3_3mcz_0_348
MNASAVETIYESTEDKAALTSVVDLVKLSDQYRQSAILHYAVADKLFDLTQTGRTPAEVAAS
FGMVEGKAAILLHALAALGLLTKEGDAFRNTALTERYLTTTSADYIGPIVEHQYLQWDNW
PRLGEILRSEKPLAFQQESRFAHDTRARDAFNDAMVRLSQPMVDVVSELGVFARARTVIDL
AGGHGTYLAQVLRRHPQLTGQIWDLPTTRDAARKTIHAHDLGGRVEFFEKNLLDARNFE
GGAADVVMLNDCLHYFDAREAREVIGHAAGLVKPGGALLILTMTMNDDRVTPALSADFS
LHMMVNTNHGELHPTPWIAGVVRDAGLAVGERSIGRYTLLIGQRSSGE
11111110000000000000000000000000000000000000000000000000000000000000000000000000000
00000000000000000000000000000000000000000000000000000000000000000000000000000000000
00000000000000000000000000000000000000000000000000000000000000000000000000000000000
00000000000000000000000000000000000000000000000000000000000000000000000000000000000
00000000000000000
>Q57265_2zdr_0_348
MQNNNEFKIGNRSVGYNHEPLIICEIGINHEGSLKTAFEMVDAAYNAGAEVVKHQTHIVE
DEMSDEAKQVIPGNADVSIYEIMERCALNEEDEIKLKEYVESKGMIFISTPFSRAAALRLQR
MDIPAYKIGSGECNNYPLIKLVASFGKPIILSTGMNSIESIKKSVEIIREAGVPYALLHCTNIYPT
PYEDVRLGGMNDLSEAFPDAIIGLSDHTLDNYACLGAVALGGSILERHFTDRMDRPGPDIV
CSMNPDTFKELKQGAHALKLARGGKKDTIIAGEKPTKDFAFASVVADKDIKKGELLSGDN

LWVKRPGNGDFSVNEYETLFGKVAACNIRKGAQIKKTDIE
10000000000000000000000000000000000000000000000000000000000000000000000000000000000
00000000000000000000000000000000000000000000000000000000000000000000000000000000000
00000000000000000000000000000000000000000000000000000000000000000000000000000000000
00000000000000000000000000000000000000000000000000000000000000000000000000000000000
00000000000000000
>Q9NRR6_2xsw_274_622
EGSLLASGALLGADELARYFPDRNVALFVATWNMQGQKELPPSLDEFLLPAEADYAQDLY
VIGVQEGCSDRREWETRLQETLGPHYVLLSSAAHGVLYMSLFIRRDLIWFCSEVECSTVTTRI
VSQIKTKGALGISFTFFGTSFLFITSHFTSGDGKVAERLLDYTRTVQALVLPRNVPDTNPYRSS
AADVTTRFDEVFWFGDFNFRLSGGRTVVDALLCQGLVVDVPALLQHDQLIREMRKGSIFK
GFQEPDIHFLPSYKFDIGKDTYDSTSKQRTPSYTDRVLYRSRHKGDICPVSYSSCPGIKTSDHR
PVYGLFRVKVRPGRDNIPLAAGKFDRELYLLGIKRRIS
11111110000000000000000000000000000000000000000000000000000000000000000000000000000
00000000000000000000000000000000000000000000000000000000000000000000000000000000000
00000000000000000000000000000000000000000000000000000011100000000000000000000000000
00000000000000000000000000000000000000000000000000000000000000000000000000000000000
00000000000000000
>O73984_2cxi_0_347
MPKFDVSKSDLERLIGRSFSIEEWEDLVLYAKCELDDVWEENGKVYFKLDSKDTNRPDLWS
AEGVARQIKWALGIEKGLPKYEVKKSNVTVYVDEKLKDIRPYGVYAIVEGLRLDEDSLSQMI
QLQEKIALTFGRRRREVAIGIFDFDKIKPPIYYKAAEKTEKFAPLGYKEEMTLEEILEKHEKGR
EYGHLIKDKQFYPLLIDSEGNVLSMPPIINSEFTGRVTTDTKNVFIDVTGWKLEKVMLALNV
MVTALAERGGKIRSVRVVYKDFEIETPDLTPKEFEVELDYIRKLSGLELNDGEIKELLEKMMY
EVEISRGRAKLKYPAFRDDIMHARDILEDVLIAYGY
10000000000000000000000000000000000000000000000000000000000000000000000000000000000
00000000000000000000000000000000000000000000000000000000000000000000000000000000000
00000000000000000000000000000000000000000000000000000000000000000000000000000000000
00000000000000000000000000000000000000000000000000000000000000000000000000000000000
0000000000000000
>P06204_3jze_0_347
MTAPSQVLKIRRPDDWHVHLRDGDMLKTVVPYTSEIYGRAIVMPNLASPITTVDAAIAYRQ
RILDAVPAGHDFTPLMTCYLTDSLDADELERGFHEGVFTAAKLYPANATTNSSHGVTSVDA
IMPVLERMEKLGIPLLVHGEVTHADVDIFDREARFIDTVMEPLRQRLTALKVVFEHITTKDA
AQYVRDGNDYLAATITPQHLMFNRNDMLVGGIRPHLYCLPILKRNIHQQALRELVASGFTR
AFLGTDSAPHSRHRKETSCGCAGCFNAPSALGSYAAVFEEMNALAHFEAFCSLNGPQFYG
LPMNTGWVELVRDEQQIPGNIALADDSLVPFLAGETVRWSVKK
11110000000000000000000000000000000000000000000000000000000000000000000000000000000
00000000000000000000000000000000000000000000000000000000000000000000000000000000000
00000000000000000000000000000000000000000000000000000000000000000000000000000000000
00000000000000000000000000000000000000000000000000000000000000000000000000000000000
0000000000000000
>P09148_1guq_0_347
MTQFNPVDHPHRRYNPLTGQWILVSPHRAKRPWQGAQETPAKQVLPAHDPDCFLCAGN
VRVTGDKNPDYTGTYVFTNDFAALMSDTPDAPESHDPLMRCQSARGTSRVICFSPDHSKTL
PELSVAALTEIVKTWQEQTAELGKTYPWVQVFENKGAAMGCSNPHPHGQIWANSFLPNE
AEREDRLQKEYFAEQKSPMLVDYVQRELADGSRTVVETEHWLAVVPYWAAWPFETLLLPK
AHVLRITDLTDAQRSDLALALKKLTSRYDNLFQCSFPYSMGWHGAPFNGEENQHWQLHA
HFYPPLLRSATVRKFMVGYEMLAETQRDLTAEQAAERLRAVSDIHFRESGV
10000000000000000000000000000000000000000000000000000000000000000000000000000000000
00000000000000000000000000000000000000000000000000000000000000000000000000000000000

00000000000000000000000000000000000000000000000000000000000000000000000000000000000
00000000000000000000000000000000000000000000000000000000000000000000000000000000000
000000000000111
>P83703_1ujn_0_347
MQRLEVREPVPYPILVGEGVLKEVPPLAGPAALLFDRRVEGFAQEVAKALGVRHLLGLPGG
EAAKSLEVYGKVLSWLAEKGLPRNATLLVVGGGTLTDLGGFVAATYLRGVAYLAFPTTTLAI
VDASVGGKTGINLPEGKNLVGAFHFPQGVYAELRALKTLPLPTFKEGLVEAFKHGLIAGDE
ALLKVEDLTPQSPRLEAFLARAVAVKVRVTEEDPLEKGKRRLLNLGHTLGHALEAQTRHAL
PHGMAVAYGLLYAALLGRALGGEDLLPPVRRLLLWLSPPPLPPLAFEDLLPYLLRDKKKVSE
SLHWVVPLAPGRLVVRPLPEGLLREAFAAWREELKGLGLLR
00000000000000000000000000000000000000000000000000000000000000000000000000000000000
00000000000000000000000000000000000000000000000000000000000000000000000000000000000
00000000000000000000000000000000000000000000000000000000000000000000000000000000000
00000000000000000000000000000000000000000000000001111111110000000000000000000000000
000000000000000
>Q0TR20_4edp_0_347
MKKKILATLLTGLVLGTSLVGCGKTEGAEAGKKLVVSTWGLNEDVLKETVFEPFAKEHGVE
IVLDIGNNSERLTKMKNNPNSQIDITYLAESFAEQGVEAGIFDKLDYSKIPNASEMNEKAKS
TVEAGYGPAYTLNSIGIVVDPSAGIEINSWEDLWKPELKNKIAIPDITTTNGPAMVEIAAEKA
GVDVKTDNGEAAFKELEALKPNVVKTYSKSSDLANMFSNGEIVAAVASDFAFGTISKAKPE
VINVIPESGTYLNFNTININKNSKNKDLAYEFINYALSKEVQEKTAKALNESPVNKEVKLSEE
ETKNLTYGPVVDNAKVIDFKFVNSVMDQWVNNWNRIMN
11111111111111111111111111111111000000000000000000000000000000000000000000000000000
00000000000000000000000000000000000000000000000000000000000000000000000000000000000
00000000000000000000000000000000000000000000000000000000000000000000000000000000000
00000000000000000000000000000000000000000000000000000000000000000000000000000000000
000000000000000
>Q6BSM0_3io3_0_347
MDLELEPTLESIVQHDSLKWIFVGGKGGVGKTTTSSSVAVQLALAQPNEQFLLISTDPAHNL
SDAFCQKFGKDARKVEGLPNLSCMEIDPEAAMSDLQQQASQYNNDPNDPLKSMMSDMT
GSIPGIDEALSFMEVLKHIKNQKVLEGEDNSNAISYKTIIFDTAPTGHTLRFLQLPSTLEKLLS
KFKDLSGKLGPMLSMMGGGQQQDIFEKLNEVQKNVSEVNEQFTNPELTTFICVCISEFLSLY
ETERMIQELMSYNMDVNSIVVNQLLFAEGDDHSCKRCESRWKMQKKYLDQMGELYEDYH
LVKMPLLGCEIRGVENLKKFSKFLLKPYDPKADSDIVFDLEEK
10000000000000000000000000000000000000000000000000000000000000000000000000000000000
00000001111111111111111111111111111111111111111111111111111111111111111100000000000
00000000111111111111111111111111111111111111111111111000000000000000000000000000000
00000000000000000000000001111100000000000000000000000000000000000000000000000000000
000000000000011
>Q7LLW9_2w2k_0_347
MPRPRVLLLGDPARHLDDLWSDFQQKFEVIPANLTTHDGFKQALREKRYGDFEAIIKLAVE
NGTESYPWNADLISHLPSSLKVFAAAGAGFDWLDLDALNERGVAFANSRGAGDTATSDLA
LYLILSVFRLASYSERAARTGDPETFNRVHLEIGKSAHNPRGHVLGAVGLGAIQKEIARKAV
HGLGMKLVYYDVAPADAETEKALGAERVDSLEELARRSDCVSVSVPYMKLTHHLIDEAFFA
AMKPGSRIVNTARGPVISQDALIAALKSGKLLSAGLDVHEFEPQVSKELIEMKHVTLTTHIG
GVAIETFHEFERLTMTNIDRFLLQGKPLLTPAGKVFAPSSAA
10000000000000000000000000000000000000000000000000000000000000000000000000000000000
00000000000000000000000000000000000000000000000000000000000000000000000000000000000
00000000000000000000000000000000000000000000000000000000000000000000000000000000000
00000000000000000000000000000000000000000000000000000000000000000000000000000000000
000000000000011

>Q98H03_3pi7_0_347
MSPMTIPSEMKALLLVGDGYTKTPSGSALEAMEPYLEQGRIAVPAPGPSQVLIKVNLASINPS
DVAFIKGQYGQPRVKGRPAGFEGVGTIVAGGDEPYAKSLVGKRVAFATGLSNWGSWAEYAV
AEAAACIPLLDTVRDEDGAAMIVNPLTAIAMFDIVKQEGEKAFVMTAGASQLCKLIIGLAK
EEGFRPIVTVRRDEQIALLKDIGAAHVLNEKAPDFEATLREVMKAEQPRIFLDAVTGPLASA
IFNAMPKRARWIIYGRLDPDATVIREPGQLIFQHKHIEGFWLSEWMRQFKERRGPAILEAQK
RFSDGRWSTDVTAVVPLAEAIAWVPAELTKPNGKVFIRP
11100000000000000000000000000000000000000000000000000000000000000000000000000000000
00000000000000000000000000000000000000000000000000000000000000000000000000000000000
00000000000000000000000000000000000000000000000000000000000000000000000000000000000
00000000000000000000000000000000000000000000000000000000000000000000010000000000000
0000000000000000
>B7ADR8_4gpv_20_366
NDEVENLGNNVPGEQAVLTIKLKGDGDNQAQSRAAGPATDTEDAVINNYLVFLFREGGAL
DCAPYEGSSNAAATITTGTTAAKKAYVVANTGALAGGLFATVKTETDLLAVTGSLMDNTD
NASTQTKTNLWMSGESEVKFNGGTNAQVTVSLSFVAAKIQLIVKDNRKNMTGGTITITDD
AAVLLFAGKKGRFFGSAAEKVTQNEFYTGFNQYTGAFDSGVTTSTALSDAVSPGDFTINAG
STVFNHFYTFGNDGTTQPTILAIKSTKTVGGTSSPIFYPILFTNTDARHTIEPGKSYTVTVTLN
GDVAAGGGGGTTDPEEPVVSSSIEVTVTAAQWVTQPVDKEFN
11111110000000000000000000001111111100000000000000000000000000000000000000000000000
00000000000000000000000000000000000000000000000000000000000000000000000000000000000
00000000000000000000000000000000000000000000000000000000000000000000000000000000000
00000000000000000000000000000000000000000000000000000000000000000000000000000000000
000000000001111
>P0A5M2_1k0r_0_346
MNIDMAALHAIEVDRGISVNELLETIKSALLTAYRHTQGHQTDARIEIDRKTGVVRVIARET
DEAGNLISEWDDTPEGFGRIAATTARQVMLQRFRDAENERTYGEFSTREGEIVAGVIQRDSR
ANARGLVVVRIGTETKASEGVIPAAEQVPGESYEHGNRLRCYVVGVTRGAREPLITLSRTHP
NLVRKLFSLEVPEIADGSVEIVAVAREAGHRSKIAVRSNVAGLNAKGACIGPMGQRVRNVM
SELSGEKIDIIDYDDDPARFVANALSPAKVVSVSVIDQTARAARVVVPDFQLSLAIGKEGQN
ARLAARLTGWRIDIRGDAPPPPPGQPEPGVSRGMAHDR
00000000000000000000000000000000000000000000000000000000000000000000000000000000000
00000000000000001111111100000000000000000000000000000000000000000000000000000000000
00000000000000000000000000000000000000000000000000000000000000000000000000000000000
00000000000000000000000000000000000000000000000000000000000000000000000000000000111
111111111111111
>Q585F3_4afv_0_346
MCSLITQLCDAGQLADYVGLGWLNAVSSQPYLVQALGLQPPPRRVDVDAAFRDAKGLHG
HQPWVATPLPGQTVRALFIGINYYGTSAALSGCCNDVKQMLATLQKKGLPINEAVILVDED
NFPGRTDQPTRDNIVRYMAWLVKDAKPGDVLFFHYSGHGTQCKSRGDSDEKYDQCIAPV
DFQKSGCIVDDDIHKLLFSRLPEKVRLTAVFDCCHSGSIMDLPFTYVCSGGEQASGTPHMKR
IREGNDVLGDVMMISGCADEQTSADVKNTATFGTGSTGAGGAATQCITCMLMNNQSLSY
GKLLIETRDMLKRKGFKQVPQLSASKAIDLDQTFSLTEMFSVDRSIQ
11111111111111000000000000000000000000000000000000000000000000000000000000000000000
00000000000000000000000000000000000000000000000000000000000000000000000000000000001
11111000000000000000000000000000000000000000000000000000000000000000000000000000000
00000000000000000111111111100000000000000000000000000000000000000000000000000000000
000000000000000
>Q6EZC2_2iy9_0_346
MLKILWTYILFLLFISASARAEKPWYFDAIGLTETTMSLTDKNTPVVVSVVDSGVAFIGGLSD
SEFAKFSFTQDGSPFPVKKSEALYIHGTAMASLIASRYGIYGVYPHALISSRRVIPDGVQDSWI

RAIESIMSNVFLAPGEEKIINISGGQKGVASASVWTELLSRMGRNNDRLIVAAVGNDGADIR
KLSAQQRIWPAAYHPVSSVNKKQDPVIRVAALAQYRKGETPVLHGGGITGSRFGNNWVDI
AAPGQNITFLRPDAKTGTGSGTSEATAIVSGVLAAMTSCNPRATATELKRTLLESADKYPSL
VDKVTEGRVLNAEKAISMFCKKNYIPVRQGRMSEEL
11111111111111111111110000000000000000000000000000000000000000000000000000000000000
00000000000000000000000000000000000000000000000000000000000000000000000001000000000
00000000000000000000000000000000000000000000000000000000000000000000000000000000000
00000000000000000000000000000000000000000000000000000000000000000000000000000000000
111111111111111
>Q8N4Q0_2x7h_24_370
QGSAIPQAMQKLVVTRLSPNFREAVTLSRDCPVPLPGDGDLLVRNRFVGVNASDINYSAGR
YDPSVKPPFDIGFEGIGEVVALGLSASARYTVGQAVAYMAPGSFAEYTVVPASIATPVPSVKP
EYLTLLVSGTTAYISLKELGGLSEGKKVLVTAAAGGTGQFAMQLSKKAKCHVIGTCSSDEKS
AFLKSLGCDRPINYKTEPVGTVLKQEYPEGVDVVYESVGGAMFDLAVDALATKGRLIVIGFI
SGYQTPTGLSPVKAGTLPAKLLKKSASVQGFFLNHYLSKYQAAMSHLLEMCVSGDLVCEV
DLGDLSPEGRFTGLESIFRAVNYMYMGKNTGKIVVELPH
11110000000000000000000000000000000000000000000000000000000000000000000000000000000
00000000000000000000000000000000000000000000000000000000000000000000000000000000000
00000000000000000000000000000000000000000000000000000000000000000000000000000000000
00000000000000000000000000000000000000000000000000000000000000000000000000000000000
000000000000000
>O87364_3js6_0_345
MSNVYVMALDFGNGFVKGKINDEKFVIPSRIGRKTNENNQLKGFVDNKLDVSEFIINGNN
DEVLLFGNDLDKTTNTGKDTASTNDRYDIKSFKDLVECSIGLLAREVPEEVVNVVIATGMPS
NEIGTDKQAKFEKLLNKSRLIEIDGIAKTINVKGVKIVAQPMGTLLDLNMENGKVFKAFTE
GKYSVLDFGSGTTIIDTYQNMKRVEEESFVINKGTIDFYKRIASHVSKKSEGASITPRMIEKGL
EYKQCKLNQKTVIDFKDEFYKEQDSLIEEVMSNFEITVGNINSIDRIIVTGGGANIHFDSLSH
YYSDVFEKADDSQFSNVRGYEKLGELLKNKVEQESK
10000000000000000000000000000000000000000000000000000000000000000000000000000000000
00000000000000000000000000000000000000000000000000000000000000000000000000000000000
00000000000000000000000000000000000000000000000000000000000000001111111100000000001
11111111111100000000000000000000000000000000000000000000000000000000000000000000000
00000000000111
>Q55835_2voz_0_345
MTTKISRRTFFVGGTALTALVVANLPRRASAQSRTINLYSSRHYNTDDALYDAFGEVNLIEAS
AEELIERIQSEGANSPGDILFTVDAGMLWRAEQAGLFQPVRSGKLNERIPENLRHPDGLWY
GFTQRARVLYYSRDRVNPADLSTYEALADPQWRGKILVRPSSNVYNLSLTASRIAIHGEPETR
RWLQGLVGNFARQPEGNDTAQIRAIAAGIGDVAIANSYYYIRLQKSTDPADQEVVEKVSLF
FPNTGSGERGTHVNVSGAGVLKNAPNRDAAIAFLEYLASDDAQRYFAEGNNEYPVIPGVPI
DPVLAAHGQLKGDPLNVSNLGRYQPDSARLMNEVGWQ
11111111111111111111111111111110000000000000000000000000000000000000000000000000000
00000000000000000000000000000000000000000000000000000000000000000000000000000000000
00000000000000000000000000000000000000000000000000000000000000000000000000000000000
00000000000000000000000000000000000000000000000000000000000000000000000000000000000
00000000000000
>Q723P8_4fmz_34_379
AATLATLPAPINQIFPDADLAEGIRAVLQKASVTDVVTQEELESITKLVVAGEKVASIQGIEYL
TNLEYLNLNGNQITDISPLSNLVKLTNLYIGTNKITDISALQNLTNLRELYLNEDNISDISPLA
NLTKMYSLNLGANHNLSDLSPLSNMTGLNYLTVTESKVKDVTPIANLTDLYSLSLNYNQIE
DISPLASLTSLHYFTAYVNQITDITPVANMTRLNSLKIGNNKITDLSPLANLSQLTWLEIGTN
QISDINAVKDLTKLKMLNVGSNQISDISVLNNLSQLNSLFLNNNQLGNEDMEVIGGLTNLT

TLFLSQNHITDIRPLASLSKMDSADFANQVIKK
00000000000000000000000000000000000000000000000000000000000000000000000000000000000
00000000000000000000000000000000000000000000000000000000000000000000000000000000000
00000000000000000000000000000000000000000000000000000000000000000000000000000000000
00000000000000000000000000000000000000000000000000000000000000000000000000000000000
00000000001111
>Q9NX46_2g4k_17_362
RSLSRFRGCLAGALLGDCVGSFYEAHDTVDLTSVLRHVQSLEPDPGTPGSERTEALYYTDDT
AMARALVQSLLAKEAFDEVDMAHRFAQEYKKDPDRGYGAGVVTVFKKLLNPKCRDVFEP
ARAQFNGKGSYGNGGAMRVAGISLAYSSVQDVQKFARLSAQLTHASSLGYNGAILQALAV
HLALQGESSSEHFLKQLLGHMEDLEGDAQSVLDARELGMEERPYSSRLKKIGELLDQASVT
REEVVSELGNGIAAFESVPTAIYCFLRCMEPDPEIPSAFNSLQRTLIYSISLGGDTDTIATMAG
AIAGAYYGMDQVPESWQQSCEGYEETDILAQSLHRVFQKS
00000000000000000000000000000000000000000000011111111000000000000000000000000000000
00000000000000000000000000000000000000000000000000000000000000000000000000000000000
00000000000000000000000000000000000000000000000000000000000000000000000000000000000
00000000000000000000000000000000000000000000000000000000000000000000000000000000000
00000000000000
>Q9RWH8_3m4a_0_345
MPSRTELLAEEYLRREGHDPQKFRYVHPDGTPYTDADGLARIARLAVPPAYQDVYVSPDAE
NELQAFGRDAAGRLQYRYHPDFVQAGALKKWQRLTRFAGALPTLKVATTADLRASGLPP
RKVMALMTRLLHVARFRVGSDIYARQHKTYGLSTLRQRHVVVDGNTVTFRFKGKHGVSQ
HKATSDRTLAANMQKLLDLPGPWLFQTVDAGGGERRRIHSTELNAYLREVIGPFTAKDFRT
WGGTLLAAEYLAQQGTESSERQAKKVLVDCVKFVADDLGNTPAVTRGSYICPVIFDRYLDG
KVLDDYEPRTERQEAELEGLTRSEGALKRMLESERTLRQRGRALK
10000000011110011111111111111111111111100000000000000000000000000000000010000000000
01110000000000000000000000000000000000000000000000000000000000000000000000000000000
00000000000000000000000000000000000000000001000000000000000000000000000000000000000
00000000000000000000000000000000000000000000000000000000000000000000000000000000000
00000011111111
>P31517_2j0p_0_344
MSKSIYEQYLQAKADNPGKYARDLATLMGISEAELTHSRVSHDAKRLKGDARALLAALEA
VGEVKAITRNTYAVHEQMGRYENQHLNGHAGLILNPRNLDLRLFLNQWASAFTLTEETRH
GVRHSIQFFDHQGDALHKVYVTEQTDMPAWEALLAQFITTEIPELQLEPLSAPEVTEPTATD
EAVDAEWRAMTDVHQFFQLLKRNNLTRQQAFRAVGNDLAYQVDNSSLTQLLNIAQQEQ
NEIMIFVGNRGCVQIFTGMIEKVTPHQDWINVFNQRFTLHLIETTIAESWITRKPTKDGFVTS
LELFAADGTQIAQLYGQRTEGQPEQTQWRDEIARLNNKDIAA
11100000000000000000000000000000000000000000000000000000000000000000000000000000000
00000000000000000000000000000000000000000000000000000000000000000000000000000000000
00011111111110000000000000000000000000000000000000000000000000000000000000000000000
00000000000000000000000000000000000000000000000000000000000000000000000000000000000
0000000001111
>P51016_1nvm_0_344
MTFNPSKKLYISDVTLRDGSHAIRHQYTLDDVRAIARALDKAKVDSIEVAHGDGLQGSSFN
YGFGRHTDLEYIEAVAGEISHAQIATLLLPGIGSVHDLKNAYQAGARVVRVATHCTEADVS
KQHIEYARNLGMDTVGFLMMSHMIPAEKLAEQGKLMESYGATCIYMADSGGAMSMNDIR
DRMRAFKAVLKPETQVGMHAHHNLSLGVANSIVAVEEGCDRVDASLAGMGAGAGNAPL
EVFIAVAERLGWNHGTDLYTLMDAADDIVRPLQDRPVRVDRETLGLGYAGVYSSFLRHAEI
AAAKYNLKTLDILVELGHRRMVGGQEDMIVDVALDLLAAHKENRA
10000000000000000000000000000000000000000000000000000000000000000000000000000000000
00000000000000000000000000000000000000000000000000000000000000000000000000000000000

00000000000000000000000000000000000000000000000000000000000000000000000000000000000
00000000000000000000000000000000000000000000000000000000000000000000000000000000000
0000000001111
>Q833I7_2o0m_0_344
MLKEFKMIEAVAPDMLDVLQERFQILRNIYWMQPIGRRSLSETMGITERVLRTETDVLKQLN
LIEPSKSGMTLTERGLEVYQGLELVMNQLLGMHQIEKEMTQYFGIQRCIVVAGDSDIQKKV
LSDFGDVLTNTLNLLLPNGENTIAVMGGTTMAMVAENMGSLETEKRHNLFVPARGGIGEA
VSVQANSISAVMANKTGGNYRALYVPEQLSRETYNSLLQEPSIQEVLTLISHANCVVHSIGR
ALHMAARRKMSDDEMVMLKQKNAVAESFGYFFDEEGKVVYKIPRIGLQLKNLQEIPYVVA
IAGGKTKAKAIRAYMKNAPKQTWLITDEAAANEILKGVTL
11111111111111111111111111111111111111111111111111111111111111111111111111111111111
11111111111000000000000000000000000000000000000000000000000000000000000000000000000
00000000000000000000000000000000000000000000000000000000000000000000000000000000000
00000000000000000000000000000000000000000000000000000000000000000000000000000000000
0000000001111
>O50325_3b4n_0_343
MFKYVIPLCALTLAAPSFAAQTTLMLSQKSDVNYLGWSTDESKVARQEVYRGTTSNPDLRE
RIAVLDAETRTFKDADTNSGLNYWYWVDVVSENQAQVVSNAVTTAPNAGPLRAAKASSE
CKPGATFENRTVDCGGVTIGTSCPNDSDKQKPLIILKNATVKNLRISASGRADGIHCDSGNC
TIENVIWEDICEDAATNNGKTMTIVGGIAHNAKDGYGGKPDKVLQHNSKNSTTVVKGNF
TLTGEHGKLWRSCGDCSNNGGPRFLTVTSATVNGTIDSIAGVNRNYGDVATISGLKIKNYK
EGKPPVCEEFKGVVKGQGSTEKYGEKWDTTNCKVSRSGVSKL
11111111111111111110000000000000000000000000000000000000000000000000000000000000000
00000000000000000000000011111111110000000000000000000000000000000000000000000000000
00000000000000000000000000000000000000000000000000000000000000000000000000000000000
00000000000000000000000000000000000000000000000000000000000000000000000000000000000
000000000000
>Q13490_3t6p_264_617
SMQTHAARMRTFMYWPSSVPVQPEQLASAGFYYVGRNDDVKCFCCDGGLRCWESGDDP
WVEHAKWFPRCEFLIRMKGQEFVDEIQGRYPHLLEQLLSTPPIIHFGPGESSSEDAVMMNTP
VVKSALEMGFNRDLVKQTVQSKILTTGENYKTVNDIVSALLNAEDEKREEEKEKQAEEMA
SDDLSLIRKNRMALFQQLTCVLPILDNLLKANVINKQEHDIIKQKTQIPLQARELIDTILVKG
NAAANIFKNCLKEIDSTLYKNLFVDKNMKYIPTEDVSGLSLEEQLRRLQEERTCKVCMDKE
VSVVFIPCGHLVVCQECAPSLRKCPICRGIIKGTVRTFLS
00000000000000000000000000000000000000000000000000000000000000000000000000000000000
00000000000000011111111111110000000000000000000000000000000000000000000000000000000
00000000000000000000000000000000000000000000000000000000000000000000000000000000000
00000000000000000000000000000000000000000000000000000000000000000000000000000000000
000000000011
>Q5LKY7_3e3m_1_344
DSRKPGHRPVTMRDVAKAAGVSRMTVSRALKKDSPISSETRERILKVVKDMNYVPDQVAG
SLTTKRSGFVGLLLPSLNNLHFAQTAQSLTDVLEQGGLQLLLGYTAYSPEREEQLVETMLRR
RPEAMVLSYDGHTEQTIRLLQRASIPIVEIWEKPAHPIGHTVGFSNERAAYDMTNALLARGF
RKIVFLGEKDDDWTRGAARRAGFKRAMREAGLNPDQEIRLGAPPLSIEDGVAAAELILQEY
PDTDCIFCVSDMPAFGLLSRLKSIGVAVPEQVSVVGFGNFEVSRFASPEISTVRVDPIAIGRET
GSLILRLLDPKQRSPQTAQHITLPPVLEFRPSLKN
11111111111111111111111111111111111111111111111111111111111111111110000000000000000
00000000000000000000000000000000000000000000000000000000000000000000000000000000000
00000000000000000000000000000000000000000000000000000000000000000000000000000000000
00000000000000000000000000000000000000000000000000000000000000000000011111111000000
000000000000

>Q8Y3G4_3gg9_9_352
MSMKIAVLDDYQDAVRKLDCFSLLQDHEVKVFNNTVKGVGQLAARVADVEAIVLIRERTR
VTRQLLDRLPKLKIISQTGRVSRDAGGHIDLEACTDKGVVVLEGKGSPVAPAELTWALVMA
AQRRIPQYVASLKHGAWQQSGLKSTTMPPNFGIGRVLKGQTLGIFGYGKIGQLVAGYGRAF
GMNVLVWGRENSKERARADGFAVAESKDALFEQSDVLSVHLRLNDETRGIVTVADLTRMK
PTALFVNTSRAELVEENGMVTALNRGRPGMAAIDVFETEPILQGHTLLRMENCICTPHIGY
VERESYEMYFGIAFQNILDILQGNVDSVANPTALAPALIRA
10000000000000000000000000000000000000000000000000000000000000000000000000000000000
00000000000000000000000000000000000000000000000000000000000000000000000000000000000
00000000000000000000000000000000000000000000000000000000000000000000000000000000000
00000000000000000000000000000000000000000000000000000000000000000000000000000000000
000000000001
>O05512_3cbw_19_361
LAKPIEAHTVSPVNPNAQQTTKTVMNWLAHLPNRTENRVLSGAFGGYSHDTFSMAEADR
IRSATGQSPAIYGCDYARGWLETANIEDSIDVSCNGDLMSYWKNGGIPQISLHLANPAFQSG
HFKTPITNDQYKKILDSSTVEGKRLNAMLSKIADGLQELENQGVPVLFRPLHEMNGEWFW
WGLTSYNQKDNERISLYKQLYKKIYHYMTDTRGLDHLIWVYSPDANRDFKTDFYPGASYV
DIVGLDAYFQDAYSINGYDQLTALNKPFAFTEVGPQTANGSFDYSLFINAIKQKYPKTIYFLA
WNDEWSAAVNKGASALYHDSWTLNKGEIWNGDSLTPIVE
11111110000000000000000000000000000000000000000000000000000000000000000000000000000
00000000000000000000000000000000000000000000000000000000000000000000000000000000000
00000000000000000000000000000000000000000000000000000000000000000000000000000000000
00000000000000000000000000000000000000000000000000000000000000000000000000000000000
00000000000
>O14717_1g55_1_390
EPLRVLELYSGVGGMHHALRESCIPAQVVAAIDVNTVANEVYKYNFPHTQLLAKTIEGITLE
EFDRLSFDMILMSPPCQPFTRIGRQGDMTDSRTNSFLHILDILPRLQKLPKYILLENVKGFEVS
STRDLLIQTIENCGFQYQEFLLSPTSLGIPNSRLRYFLIAKLQSEPLPFQAPGQVLMEFPKIEIH
RKNQQDSDLSVKMLKDFLEDDTDVNQYLLPPKSLLRYALLLDIVQPTCRRSVCFTKGYGSYI
EGTGSVLQTAEDVQVENIYKSLTNLSQEEQITKLLILKLRYFTPKEIANLLGFPPEFGFPEKITV
KQRYRLLGNSLNVHVVAKLIKILYE
00000000000000000000000000000000000000000000000000000000000000000000000000000111111
11111111111100000000000000000000000000000000000000000000000000000000000000000000000
00000000000000000000011111111111100000000000000000000000000000000000000000000000000
00000000000000000000000000000000000000000000000000000000000000000000000000000000000
00000000000
>P36551_2aex_110_452
TSLGRPEEEEDELAHRCSSFMAPPVTDLGELRRRPGDMKTKMELLILETQAQVCQALAQV
DGGANFSVDRWERKEGGGGISCVLQDGCVFEKAGVSISVVHGNLSEEAAKQMRSRGKVL
KTKDGKLPFCAMGVSSVIHPKNPHAPTIHFNYRYFEVEEADGNKQWWFGGGCDLTPTYL
NQEDAVHFHRTLKEACDQHGPDLYPKFKKWCDDYFFIAHRGERRGIGGIFFDDLDSPSKEE
VFRFVQSCARAVVPSYIPLVKKHCDDSFTPQEKLWQQLRRGRYVEFNLLYDRGTKFGLFTPG
SRIESILMSLPLTARWEYMHSPSENSKEAEILEVLRHPRDWV
11111111000000000000000000000000000000000000000000000000000000000000000000000000000
00000000000000000000000000000000000000000000000000000000000000000000000000000000000
00000000000000000000000000000000000000000000000000000000000000000000000000000000000
00000000000000000000000000000000000000000000000000000000000000000000000000000000000
00000000000
>Q49410_3myu_25_367
CATKSDNTLIFNISLDHNADTSIEKFFTVFSKKLSGKLNKKINVNFNIVDDSFTKINNIQANK
ADFAFVNSQAIASNNWFGYTPLIQTLTTAFKEDLELDYYEDGNLQKKAEKTNLLFLSPPYKE

WDDIKQKWTGNRYDFLYEPSKLVSFYRSMILITGSASEITAIKKAWNEKNWNQFMKFGIGH
GQTNSASRFELPDLLFRKHFAKNYPGLQNAINSDPDKFAVVRGREIGINKNIKIVFDDANSF
SWTQNIKGSKRPFYTPIDPNDRLEILTYSDPLLYDIGIVSNNLSRIYQKAIGEIFIELAQSSEDLY
GPSIGYNGYKMINDFEKEVVEIIEKTYGK
11111100000000000000000000000000000000000000000000000000000000000000000000000000000
00000000000000000000000000000000000000000000000000000000000000000000000000000000000
00000000000000000000000000000000000000000000000000000000000000000000000000000000000
00000011000000000000000000000000000000000000000000000000000000000000000000000000000
00000000001
>Q58546_2yyy_0_342
MPAKVLINGYGSIGKRVADAVSMQDDMEVIGVTKTKPDFEARLAVEKGYKLFVAIPDNERV
KLFEDAGIPVEGTILDIIEDADIVVDGAPKKIGKQNLENIYKPHKVKAILQGGEKAKDVEDN
FNALWSYNRCYGKDYVRVVSCNTTGLCRILYAINSIADIKKARIVLVRRAADPNDDKTGPV
NAITPNPVTVPSHHGPDVVSVVPEFEGKILTSAVIVPTTLMHMHTLMVEVDGDVSRDDILE
AIKKTPRIITVRAEDGFSSTAKIIEYGRDLGRLRYDINELVVWEESINVLENEIFLMQAVHQESI
VIPENIDCIRAMLQMEEDNFKSIEKTNKAMGIQ
10000000000000000000000000000000000000000000000000000000000000000000000000000000000
00000000000000000000000000000000000000000000000000000000000000000000000000000000000
00000000000000000000000000000000000000000000000000000000000000000000000000000000000
00000000000000000000000000000000000000000000000000000000000000000000000000000000000
00000000000
>Q5LSC5_3fj1_0_342
MTQHITRMRREIDEIPEAVQRLLDHGAQDVARVAAVLRLRDPSFVATVARGSSDHVCTYLS
YAAELLLGLPVASLGPSVASVYDARLRLDRALCLAVSQSGKSPDIVAMTRNAGRDGALCVA
LTNDAASPLAGVSAHTIDIHAGPELSVAATKTFVTSAVAGLMLLADWAEDDGLRAALGNL
PETLAAASRIDWPEMRVAIGARPSLFTLGRGTSLAVSNEAALKFKETCQLHAESYSSAEVLH
GPVSIVEEGFPVLGFAAGDAAEAPLAEIADQIAAKGATVFATTGRVTRARVLEHVRSGHAL
TDPLSLIVSFYSMVEAFASERGIDPDAPRHLNKVTETV
00000000000000000000000000000000000000000000000000000000000000000000000000000000000
00000000000000000000000000000000000000000000000000000000000000000000000000000000000
00000000000000000000000000000000000000000000000000000000000000000000000000000000000
00000000000000000000000000000000000000000000000000000000000000000000000000000000001
11111111111
>Q8ZJX7_2a3n_0_342
MNNQEKNMLGFNQDEYLTSAREIIAARQKAEQVADEIYQAGFSSLFFASVGGSLAPMMAI
NEFAKELTTLPVYVEQAAELIHKGNKRLNKDSVVITLSKSGDTKESVAIAEWCKAQGIRVVA
ITKNADSPLAQAATWHIPMRHKNGVEYEYMLLYWLFFRVLSRNNEFASYDRFASQLEILPA
NLLKAKQKFDPQADAIASRYHNSDYMMWVGGAEMWGEVYLFSMCILEEMQWKRTRPVS
SAEFFHGALELLEKDVPLILVKGEGKCRALDERVERFASKITDNLVVIDPKAYALDGIDDEFR
WIMAPCVVSTLLVDRLAAHFEKYTGHSLDIRRYYRQFDY
11111110000000000000000000000000000000000000000000000000000000000000000000000000000
00000000000000000000000000000000000000000000000000000000000000000000000000000000000
00000000000000000000000000000000000000000000000000000000000000000000000000000000000
00000000000000000000000000000000000000000000000000000000000000000000000000000000000
00000000000
>B4EA43_4gnv_0_341
MKTTPGPVMLDVVGTTLSRDDARRLAHPNTGGVILFARHFQNRAQLTALTDSIRAVREDIL
IAVDHEGGRVQRFRTDGFTVLPAMRRLGELWDRDVLLATKVATAVGYILAAELRACGIDMS
FTPVLDLDYGHSKVIGDRAFHRDPRVVTLLAKSLNHGLSLAGMANCGKHFPGHGFAEAD
SHVALPTDDRTLDAILEQDVAPYDWLGLSLAAVIPAHVIYTQVDKRPAGFSRVWLQDILRG
KLGFTGAIFSDDLSMEAAREGGTLTQAADAALAAGCDMVLVCNQPDAAEVVLNGLKAR

ASAESVRRIKRMRARGKALKWDKLIAQPEYLQAQALLSSALA
00000000000000000000000000000000000000000000000000000000000000000000000000000000000
00000000000000000000000000000000000000000000000000000000000000000000000000000000000
00000000000000000000000000000000000000000000000000000000000000000000000000000000000
00000000000001100000000000000000000000000000000000000000000000000000000000000000000
0000000000
>O14994_2p0a_75_416
GLMEPPGPSTPIVQRPRILLVIDDAHTDWSKYFHGKKVNGEIEIRVEQAEFSELNLAAYVTG
GCMVDMQVVRNGTKVVSRSFKPDFILVRQHAYSMALGEDYRSLVIGLQYGGLPAVNSLYSV
YNFCSKPWVFSQLIKIFHSLGPEKFPLVEQTFFPNHKPMVTAPHFPVVVKLGHAHAGMGKI
KVENQLDFQDITSVVAMAKTYATTEAFIDSKYDIRIQKIGSNYKAYMRTSISGNWKANTGSA
MLEQVAMTERYRLWVDSCSEMFGGLDICAVKAVHSKDGRDYIIEVMDSSMPLIGEHVEEDR
QLMADLVVSKMSQLPMPGGTAPSPLRPWAPQIKSA
11111111111111000000000000000000000000000000000000000000000000000000011111111110000
00000000000000000000000000000000000000000000000000000000000000000000000000000000000
00000000000000000000000000000000000000000000000000000000000000000000000000000000000
00000000000000000000000000000000000000000000000000000000000000000000000001111111111
1111111111
>Q3M5E0_2qe8_0_341
MENLGDRLEVVAELSLAPGNITLTPDGRLFLSLHQFYQPEMQVAELTQDGLIPFPPQSGNAII
TFDTVLGIKSDGNGIVWMLDNGNQSKSVPKLVAWDTLNNQLSRVIYLPPPITLSNSFVNDL
AVDLIHNFVYISDPAPDDKAALIRVDLQTGLAARVLQGYPGIAPEDIDLVIDGVPVQIGQPD
GTVIRPHLGVNGIVLDAENEWLYLSPMHSTSMYRIKSADLSNLQLTDAELGSKIERYSEKPIC
DGISIDKDHNIYVGDLAHSAIGVITSADRAYKLLVTDEKLSWTDSFNFGSDGYLYFDCNQLH
HSAPLNAGENISAPPYYIFRLKPLAAGIVGR
11111000000000000000000000000000000000000000000000000000000000000000000000000000000
00000000000000000000000000000000000000000000000000000000000000000000000000000000000
00000000000000000000000000000000000000000000000000000000000000000000000000000000000
00000000000000000000000000000000000000000000000000000000000000000000000000000000000
0000000000
>Q62884_3i36_874_1215
WRKKRKDAKNNEVSFSQIKPKKSKLIRVENFEAYFKKQQADSNCGFAEEYEDLKLIGISLPK
YAAEIAENRGKNRYNNVLPYDISRVKLSVQTHSTDDYINANYMPGYHSKKDFIATQGPLPN
TLKDFWRMVWEKNVYAIVMLTKCVEQGRTKCEEYWPSKQAQDYGDITVAMTSEVVLPEW
TIRDFVVKNMQSSESHPLRQFHFTSWPDHGVPDTTDLLINFRYLVRDYMKQIPPESPILVHCS
AGVGRTGTFIAIDRLIYQIENENTVDVYGIVYDLRMHRPLMVQTEDQYVFLNQCVLDIIRA
QKDSKVDLIYQNTTAMTIYENLERVSMFGKANGYIA
11111111111111111111110000000000000000000000000000000000000000000000000000000000000
00000000000000000000000000000000000000000000000000000000000000000000000000000000000
00000000000000000000000000000000000000000000000000000000000000000000000000000000000
00000000000000000000000000000000000000000000000000000000000111111111111111111111111
1111111111
>Q6N0R4_2gwg_0_341
MIIDIHGHYTTAPKALEDWRNRQIAGIKDPSVMPKVSELKISDDELQASIIENQLKKMQERG
SDLTVFSPRASFMAHHIGDFNVSSTWAAICNELCYRVSQLFPDNFIGAAMLPQSPGVDPKT
CIPELEKCVKEYGFVAINLNPDPSGGHWTSPPLTDRIWYPIYEKMVELEIPAMIHVSTSCNTC
FHTTGAHYLNADTTAFMQCVAGDLFKDFPELKFVIPHGGGAVPYHWGRFRGLAQEMKKP
LLEDHVLNNIFFDTCVYHQPGIDLLNTVIPVDNVLFASEMIGAVRGIDPRTGFYYDDTKRYI
EASTILTPEEKQQIYEGNARRVYPRLDAALKAKGK
00000000000000000000000000000000000000000000000000000000000000000000000001111100000
00000000000000000000000000000000000000000000000000000000000000000000000000000000000

00000000000000111111111000000000000000000000000000000000000000000000000000000000000
00000000000000000000000000000000000000000000000000000000000000000000000000000000000
0000000000
>Q8I2X3_3tgh_23_364
KCQLRFASLGDWGKDTKGQILNAKYFKQFIKNERVTFIVSPGSNFIDGVKGLNDPAWKNLY
EDVYSEEKGDMYMPFFTVLGTRDWTGNYNAQLLKGQGIYIEKNGETSIEKDADATNYPKW
IMPNYWYHYFTHFTVSSGPSIVKTGHKDLAAAFIFIDTWVLSSNFPYKKIHEKAWNDLKSQ
LSVAKKIADFIIVVGDQPIYSSGYSRGSSYLAYYLLPLLKDAEVDLYISGHDNNMEVIEDNDM
AHITCGSGSMSQGKSGMKNSKSLFFSSDIGFCVHELSNNGIVTKFVSSKKGEVIYTHKLNIKK
KKTLDKVNALQHFAALPNVELTDVPSSGPMGNKD
00000000000000000000000000000000000000000000000000000000000000000000000000000000000
00000000000000011111111111111100000000000000000000000011111111100000000000000000000
00000000000000000000000000000000000000000000000000000000000000000000000000000000000
00000000000000000000000000000000000000000000000000000000000000000000000000000000000
0000000111
>P00557_3tyk_0_340
MKKPELTATSVEKFLIEKFDSVSDLMQLSEGEESRAFSFDVGGRGYVLRVNSCADGFYKDRY
VYRHFASAALPIPEVLDIGEFSESLTYCISRRAQGVTLQDLPETELPAVLQPVAEAMDAIAAA
DLSQTSGFGPFGPQGIGQYTTWRDFICAIADPHVYHWQTVMDDTVSASVAQALDELMLW
AEDCPEVRHLVHADFGSNNVLTDNGRITAVIDWSEAMFGDSQYEVANIFFWRPWLACME
QQTRYFERRHPELAGSPRLRAYMLRIGLDQLYQSLVDGNFDDAAWAQGRCDAIVRSGAGT
VGRTQIARRSAAVWTDGCVEVLADSGNRRPSTRPRAKE
11111100000000000000000000000000000000000000000000000000000000000000000000000000000
00000000000000000000000000000000000000000000000000000000000000000000000000000000000
00000000000000000000000000000000000000000000000000000000000000000000000000000000000
00000000000000000000000000000000000000000000000000000111111111111111111111111111111
111111111
>Q4W8A1_3awf_235_575
SHQQMKASSRRTISQNKRRYRKDGFDLDLTYVTDHVIAMSFPSSGRQSLFRNPIGEVSRFFK
TKHPDKFRIYNLCSERGYDETKFDNHVYRVMIDDHNVPTLVDLLKFIDDAKVWMTSDPDH
VIAIHCKGGKGRTGTLVSSWLLEDGKFDTAKEALEYFGSRRTDFEVGDVFQGVETASQIRYV
GYFEKIKKNYGGQLPPMKKLKVTGVTITAIQGVGRGNGSDLSMQIVSERQEVLLCKFAEGY
NCALQYDATDDCVTCEVKNCPVLAGDIKVRFMSTSKSLPRGYDNCPFYFWFNTSLVEGDH
VTLKREEIDNPHKKKTWKIYRDNFTVKLTFSDAEDI
11111111111111111100000000000000000000000011111100000000000000000000000000000000000
00000000000000000000000000000000000000000000000000000000000000000000000000000000011
11000000000000000000000000000000000000000000000000000000000000000000000000000000000
00000000000000000000000000000000000000000000000000000000000000000000000000000000000
000001111
>Q57VZ2_3bje_0_340
MAASANGSTKGSETDLPIGKDGTTLHLKCKSDELADRIIFVGDPGRVDVISGYFDKDSIRAS
RDHREIRFATGTYKGTPVTVISTGMGVDNIEIVLNEIHALKEYDMERGQWRHRKGDADAPS
AGPFFDPSTMKIIRLGTCGSPAESVPPLALAVTRHAIGMDNTSLYYSAGTRETSKDQQEIRRI
VREQTGLRAIDIYTSMAHPNITKSICAACDAHNAATGSEADKQQYVIGTTATASGFYGCQG
RRVGRFMKHLTVPNMVEELGSLKFNLSNGVEVVTNIEMETSAICYLSDMLGYQAGAACVV
VSKRVGEKKMFLGDQLDAAMKRCIKIILEALVSA
11111111111111000000000000000000000000000000000000000000000000000000000000000000000
00000000000000000000000000000000000000000000000000000000000000000000000000000000000
00000000000000000000000000000000000000000000000000000000000000000000000000000000000
00000000000000000000000000000000000000000000000000000000000000000000000000000000000
000000000

>Q5MJ80_2vqx_0_340
MPTLTARSVIPPYMLRRIIEHGSLPQRDCALHTLNHVQSLLGNKPLRAPGAKTSTGGEVIRD
IYDAENSTQLPGKQVRNEGQASNHDVAVDEAYDYLGVTYDFFWQAFKRNSLDNQGLPLT
GSVHYGKEYQNAFWNGQQMVFGDGDGEIFNRFTIAIDVVGHELAHGVTESEAGLIYFQQ
AGALNESLSDVFGSLVKQFHLKQTADKADWLIGEGLLAKGINGKGLRSMSAPGTAYDDPL
LGKDPQPASMKDYIQTKEDNGGVHLNSGIPNRAFYLAATALGGYAWEKAGYIWYDTLCD
KALPQDADFATFARTTVKHAEQRFDSKVAQKVQQAWHQVGVA
11111000000000000000000000000000000011111111111110000000000000000000000000000000000
00000000000000000000000000000000000000000000000000000000000000000000000000000000000
00000000000000000000000000000000000000000000000000000000000000000000000000000000000
00000000000000000000000000000000000000000000000000000000000000000000000000000000000
000000000
>Q74FR4_3ezu_0_340
MSGDILNDIVAACLELERKASSVFKMFAAHAGSDEARRFWETVADETRHHSAVYERLQER
GGRENLPIIIYKPAETLEELEMIGKSIDEQVERYTEAPSSEAACLLGFRLQLYLLHPAFASLCRL
TRDASGEDLPDIGYGRYLRRFIDGIGSCGLATAETELLGEALFRLWNEARQLAAQSHFDALT
GVMTRAGFFKTVGSLAYAAQRSGSNVGIMLIDLDYFKLVGDNYGHQTGDRILQLVAETITS
HLRRSDVVGRYDGDEFVVYLSPVEPASLRTVAENLRRSIEEESARMVPVTASIGVAQGILGTD
VDGGIEELVRLADECLMQAKYTGKNKVVVK
00000000000000000000000000000000000000000000000000000000000000000000000000000000000
00000000000000000000000000000000000000000000000100000000000000000000000000000000000
00000000000000000000000000000000000000000000000000000000000001111100000000000000000
00000000000000000000000000000000000000000000000000000000000000000000000000000000000
000000000
>Q7WYN3_3l8q_27_367
APTSSIEIVLDKTTASVGEIVTASINIKNITNFSGCQLNMKYDPAVLQPVTSSGVAYTKSTMPG
AGTILNSDFNLRQVADNDLEKGILNFSKAYVSLDDYRTAAAPEQTGTVAVVKFKVLKEETSS
ISFEDTTSVPNAIDGTVLFDWNGDRIQSGYSVIQPAVINLDMIKASYITMGYDKNAAEVGEII
KATVKINKITNFSGYQVNIKYDPTVLQAVNPKTGVAYTNSSLPTSGELLVSEDYGPIVQGVH
KISEGILNLSRSYTALEVYRASESPEETGTLAVVGFKVLQKKATTVVFEDSETMPNGITGTTLF
NWYGNRIQSGYFVIQPGEINSAPIAT
00000000000000000000000000000000000000000000000000000000000000000000000000000000000
00000000000000000000000000000000000000000000000000000000000000000000000000000000000
00000000000000000000000000000000000000000000000000000000000000000000000000000000000
00000000000000000000000000000000000000000000000000000000000000000000000000000000000
000000011
>Q8Y4Z4_3gs9_0_340
MNSDIIVADFWKNNEEILTDFDKDSFCESWTENEMWSIEFKVAQTPKNAHCYSFLDYESSV
YFRGQEFVVKQLSHDAVGKTLSKDIRAPHIYYTCQDGRQDDAITGSFTLEQCLTHIFKTDNR
GFSWEIIDPSNILEKVQQENFGNNNYLTLIDQLLDDYGVVVIPDNRHLVFKPREIYGAKTEN
FIRYKYNTDEASFDIDTLSLKTKIKGYGKVDSNGNNYFSPITYTSPEVEKWGIRWQEPVSDER
YTVAGNMQRRLKLELQDYPATTGSVILKNDYECEKGDYVLFIYEPLGIDYDVQIVAYKKYPF
TIKAPEITLSNNKKSIVSIMAQLAKVLKGAK
11000000000000000000000000000000000001111100000000000000000000000000000000000111000
00000000000000000000000000000000000000000000000000000000000000000000000000000000000
00000000000000000000000000000000000000000000000000000000000000000000000000000000000
00000000000000000000000000011100000000000000000000000000000000000000000000000000000
000000011
>Q99707_2o2k_924_1264
KERRYLPLSQARKSGFQMDWLSEPHPVKPTFIGTQVFEDYDLQKLVDYIDWKPFFDVWQLR
GKYPNRGFPKIFNDKTVGGEARKVYDDAHNMLNTLISQKKLRARGVVGFWPAQSIQDDI

HLYAEAAVPQAAEPIATFYGLRQQAEKDSASTEPYYCLSDFIAPLHSGIRDYLGLFAVACFGV
EELSKAYEDDGDDYSSIMVKALGDRLAEAFAEELHERVRRELWAYCGSEQLDVADLRRLRY
KGIRPAPGYPSQPDHTEKLTMWRLADIEQSTGIRLTESLAMAPASAVSGLYFSNLKSKYFAVG
KISKDQVEDYALRKNISVAEVEKWLGPILGYDTD
10000000000000000000000000000000000000000000000000000000000000000000000000001100000
00000000000000000000000000000000000000000000000000000000000000001110000000000000000
00000000000000000000000000000000000000000000000000000000000000000000000000000000000
00000000000000000000000000000000000000000000000000000000000000000000000000000000000
000000001
>Q9Y376_1upk_0_340
MPFPFGKSHKSPADIVKNLKESMAVLEKQDISDKKAEKATEEVSKNLVAMKEILYGTNEKEP
QTEAVAQLAQELYNSGLLSTLVADLQLIDFEGKKDVAQIFNNILRRQIGTRTPTVEYICTQQN
ILFMLLKGYESPEIALNCGIMLRECIRHEPLAKIILWSEQFYDFFRYVEMSTFDIASDAFATFK
DLLTRHKLLSAEFLEQHYDRFFSEYEKLLHSENYVTKRQSLKLLGELLLDRHNFTIMTKYISK
PENLKLMMNLLRDKSRNIQFEAFHVFKVFVANPNKTQPILDILLKNQAKLIEFLSKFQNDR
TEDEQFNDEKTYLVKQIRDLKRPAQQEA
11111111100000000000000000001110000000000000000000000000111111100000000000000000000
00000000000000000000000000000000000000000000000000000000000000000000000000000000000
00000000000000000000000000000000000000000000000000000000000000000000000000000000000
00000000000000000000000000000000000000000000000000000000000000001000000000000000000
000000011
>P38830_2evf_0_339
MNEMENTDPVLQDDLVSKYERELSTEQEEDTPVILTQLNEDGTTSNYFDKRKLKIAPRSTLQ
FKVGPPFELVRDYCPVVESHTGRTLDLRIIPRIDRGFDHIDEEWVGYKRNYFTLVSTFETANC
DLDTFLKSSFDLLVEDSSVESRLRVQYFAIKIKAKNDDDDTEINLVQHTAKRDKGPQFCPSV
CPLVPSPLPKHQIIREASNVRNITKMKKYDSTFYLHRDHVNYEEYGVDSLLFSYPEDSIQKVA
RYERVQFASSISVKKPSQQNKHFSLHVILGAVVDPDTFHGENPGIPYDELALKNGSKGMFV
YLQEMKTPPLIIRGRSPSNYASSQRITVR
11111111111111111111111111111111000000000000000000000000000000000000000000000000000
00000000000000000000000000000000000000000000000000000000111111000000000000000000000
00000000000000000000000000000000000000000000000000000000000000000000000000000000000
00000000000000000000000000000000000001111110000000000000000000000000000000000000000
00011111
>Q9S427_2z72_21_360
NTATEFDGPYVITPISGQSTAYWICDNRLKTTSIEKLQVNRPEHCGDLPETKLSSEIKQIMPDT
YLGIKKVVALSDVHGQYDVLLTLLKKQKIIDSDGNWAFGEGHMVMTGDIFDRGHQVNEV
LWFMYQLDQQARDAGGMVHLLMGNHEQMVLGGDLRYVHQRYDIATTLINRPYNKLYG
ADTEIGQWLRSKNTIIKINDVLYMHGGISSEWISRELTLDKANALYRANVDASKKSLKADDL
LNFLFFGNGPTWYRGYFSETFTEAELDTILQHFNVNHIVVGHTSQERVLGLFHNKVIAVDSS
IKVGKSGELLLLENNRLIRGLYDGTRETLQENSLNQ
11000000000000000000000000000000000000000000000000000000000000000000000000000000000
00000000000000000000000000000000000000000000000000000000000000000000000000000000000
00000000000000000000000000000000000000000000000000000000000000000000000000000000000
00000000000000000000000000000000000000000000000000000000000000000000000000000000000
00000000
>Q9WYR4_3zsc_27_366
SLNDKPVGFASVPTADLPEGTVGGLGGEIVFVRTAEELEKYTTAEGKYVIVVDGTIVFEPKRE
IKVLSDKTIVGINDAKIVGGGLVIKDAQNVIIRNIHFEGFYMEDDPRGKKYDFDYINVENSH
HIWIDHCTFVNGNDGAVDIKKYSNYITVSWCKFVDHDKVSLVGSSDKEDPEQAGQAYKVT
YHHNYFKNCIQRMPRIRFGMAHVFNNFYSMGLRTGVSGNVFPIYGVASAMGAKVHVEGN
YFMGYGAVMAEAGIAFLPTRIMGPVEGYLTLGEGDAKNEFYYCKEPEVRPVEEGKPALDPR

EYYDYTLDPVQDVPKIVVDGAGAGKLVFEELNTAQ
11100000000000000000000000000000000000000000000000000000000000000000000000000000000
00000000000000000000000000000000000000000000000000000000000000000000000000000000000
00000000000000000000000000000000000000000000000000000000000000000000000000000000000
00000000000000000000000000000000000000000000000000000000000000000000000000000000000
11111111
>P15057_2j8f_0_338
MVKKNDLFVDVSSHNGYDITGILEQMGTTNTIIKISESTTYLNPCLSAQVEQSNPIGFYHFAR
FGGDVAEAEREAQFFLDNVPMQVKYLVLDYEDDPSGDAQANTNACLRFMQMIADAGYK
PIYYSYKPFTHDNVDYQQILAQFPNSLWIAGYGLNDGTANFEYFPSMDGIRWWQYSSNPFD
KNIVLLDDEEDDKPKTAGTWKQDSKGWWFRRNNGSFPYNKWEKIGGVWYYFDSKGYCL
TSEWLKDNEKWYYLKDNGAMATGWVLVGSEWYYMDDSGAMVTGWVKYKNNWYYMT
NERGNMVSNEFIKSGKGWYFMNTNGELADNPSFTKEPDGLITVA
10000000000000000000000000000000000000000000000000000000000000000000000000000000000
00000000000000000000000000000000000000000000000000000000000000000000000000000000000
00000000000000000000000000000000000000000000000000000000000000000000000000000000000
00000000000000000000000000000000000000000000000000000000000000000000000000000000000
0000000
>Q46PQ5_3iuz_0_338
MHPNIATLLSANLGESRTRHLLSLVSVPDGLPSDAEGRATRAEIAQALNMVLFAGILDRVPT
GRAYTDDVAATGGKVVFDHGALRTVKWRDNGALPEGEAAFTRILRPLGYRLNGNYPLDRI
SMTGRSYAHADAPEGIAQFFVSEFHPERFSDAFREAVGRVTGNSADPLTPRAQTLLWQLDR
DGVLTVADGAELIGLLVPCFERQHGVPRLADYETLLRESAEMAWIATEGNAFNHATDRVD
DVFGLSEQQKALGRPMKDKVEVSGSGRVKQTAFRADTVRRQFIGAQGETVERDVPGSFYEF
ITRDRFADAPAASPRVDLGFDAGNAQGIFKMTAAV
00000000000000000000000000000000000000000000000000000000000000000000000000000000000
00000000000000000000000000000000000000000000000000000000000000000000000000000000000
00000000000000000000000000000000000000000000000000000000000000000000000000000000000
00000000000000000000000000000000000000000000000000000000000001111111100000000011111
1111111
>Q4KAG7_3h75_22_360
TSVVFLNPGNSTETFWVSYSQFMQAAARDLGLDLRILYAERDPQNTLQQARELFQGRDKP
DYLMLVNEQYVAPQILRLSQGSGIKLFIVNSPLTLDQRELIGQSRQNYSDWIGSMVGDDEEA
GYRMLKELLHKLGPVPAGHGIELLAFSGLKVTPAAQLRERGLRRALAEHPQVHLRQLVYG
EWNRERAYRQAQQLLKRYPKTQLVWSANDEMALGAMQAARELGRKPGTDLLFSGVNSSP
EALQALIDGKLSVLEAGHFTLGGWALVALHDDALGLDARRLGGPDWQLSLFQALTPAQA
RQLLRLGDQVGTRVDFRGLSAQGKPDSYRYPFGLQLLLR
00000000000000000000000000000000000000000000000000000000000000000000000000000000000
00000000000000011111111111100000000000000000000000000000000000000000000000000000000
00000000000000000000000000000000000000000000000000000000000000000000000000000000000
00000000000000000000000000000000000000000000000000000000000000000000000000000000000
0000000
>Q65HU8_3lma_0_338
MKLTGKQTWEFENPLFVNSSGTAVGPKEKEGPLGHLFDKSYDEMHCNQKNWEMAERKL
MEDAVQSALSKQNLKKEDIDIFLAGDLLNQNVTANYVARHLKIPFLCLFGACSTSMESIAIS
SALIDGGFAKRALAATSSHNATAERQFRYPTEYGGQKPGTATSTVTGSGAVVLSQQPGGIKI
TSATVGRVIDLGITDSQDMGSAMAPAAADTIKQHLEDLGRTPDDYDLILTGDLSGVGSPILK
DLLKEEGINVGTKHNDCGLMIYTPDQQVFAGGSGCACSAVVTFAHIFKEIEAGRLNRVLVV
ATGALLSPTIIQQKESIPCIAHGVVFERAERGNA
00000000000000000000000000000000000000000000000000000000000000000000000000000000000
00000000000000000000000000000000000000000000000000000000000000000011111111111110000

00000000000000000000000000000000000000000000000000000000000000000000000000000000000
00000000000000000000111111100000000000000000000000000000000000000000000000000000000
0011111
>Q8AAM9_4h41_27_365
AADGEGRGELSPKIKAGDLESKLIVPKNNGLKITGTFLDEISHDIPHQNWGEKEWDLDFQH
MKRIGIDTVIMIRSGYRKFMTYPSPYLLKKGCYMPSVDLVDMYLRLAEKYNMKFYFGLYDS
GRYWDTGDLSWEIEDNKYVIDEVWKMYGEKYKSFGGWYISGEISRATKGAIDAFRAMGKQ
CKDISNGLPTFISPWIDGKKAIMGTGKLTREDAVSVQQHEKEWNEIFDGIHEVVDACAFQD
GHIDYDELDAFFTVNKKLADKYGMQCWTNAESFDRDMPIRFLPIKFDKLRMKLEAAKRA
GYDKAITFEFSHFMSPQSAYLQAGHLYDRYREYFEIK
11111111111111111100000000000000000000000000000000000000000000000000000000000000000
00000000000000000000000000000000000000000000000000000000000000000000000000000000000
00000000000000000000000000000000000000110000000000000000000000000000000000000000000
00000000000000000000000000000000000000000000000000000000000000000000000000000000000
0000000
>Q91X46_2z0q_109_447
CVNQVLTAKEIKRQEAIFELSQGEEDLIEDLKLAKKAYHDPMLKLSIMTEQELNQIFGTLDSL
IPLHEELLSQLRDVRKPDGSTEHVGPILVGWLPCLSSYDSYCSNQVAAKALLDHKKQDHRV
QDFLQRCLESPFSRKLDLWNFLDIPRSRLVKYPLLLREILRHTPNDNPDQQHLEEAINIIQGIV
AEINTKTGESECRYYKERLLYLEEGQKDSLIDSSRVLCCHGELKNNRGVKLHVFLFQEVLVIT
RAVTHNEQLCYQLYRQPIPVKDLTLEDLQDGEVRLGGSLRGAFSNNERVKNFFRVSFKNGS
QSQTHSLQANDTFNKQQWLNCIRQAKE
11100000000000000000000000000000000000000000000000000000000000000000000000000000000
00000000000000000000000000000000000000000000111111111111100000000000000000000000000
00000000000000000000000000000000000000000000000000000000000000000000000000000000000
00000000000000000000000000000000000000000110000000000000000000000000000000000000000
0000000
>Q9UVD4_1izc_0_338
MAKSYSEQPELHAKAPYRSAMLTYPGNLRQALKDAMADPSKTLMGVAHGIPSTFVTKVL
AATKPDFVWIDVEHGMFNRLELHDAIHAAQHHSEGRSLVIVRVPKHDEISLSTALDAGAA
GIVIPHVETVEEVREFVKEMYYGPIGRRSFSPWTFSPGIADASLFPNDPYNVATSNNHVCIIP
QIESVKGVENVDAIAAMPEIHGLMFGPGDYMIDAGLDLNGALSGVPHPTFVEAMTKFSTA
AQRNGVPIFGGALSVDMVPSLIEQGYRAIAVQFDVWGLSRLVHGSLAQARASAKQFAGQG
KAATDGTTDETVDGAAEEVANGVSKVKLDEAGDEDKA
10000000000000000000000000000000000000000000000000000000000000000000000000000000000
00000000000000000000000000000000000000000000000000000000000000000000000000000000000
00000000000000000000000000000000000000000000000000000000000000000000000000000000000
00000000000000000000000000000000000000000000000000011111111111111111111111111111111
1111111
>O34313_3gve_36_373
ESAAPQVHLSILATTDIHANMMDYDYYSDKETADFGLARTAQLIQKHREQNPNTLLVDNG
DLIQGNPLGEYAVKYQKDDIISGTKTHPIISVMNALKYDAGTLGNHEFNYGLDFLDGTIKG
ADFPIVNANVKTTSGENRYTPYVINEKTLIDENGNEQKVKVGYIGFVPPQIMTWDKKNLEG
QVQVQDIVESANETIPKMKAEGADVIIALAHTGIEKQAQSSGAENAVFDLATKTKGIDAIIS
GHQHGLFPSAEYAGVAQFNVEKGTINGIPVVMPSSWGKYLGVIDLKLEKADGSWKVADSK
GSIESIAGNVTSRNETVTNTIQQTHQNTLEYVRK
11110000000000000000000000000000000000000000000000000000000000000000000000000000000
00000000000000000000000000000000000000000000000000000000000000000000000000000000000
00000000000000000000000000000000000000000000000000000000000000000000000000000000000
00000000000000000000000000000000000000000000000000000000000000000000000000000000000
000000

>P80357_1yle_0_337
MLVMRPAQAADLPQVQRLAADSPVGVTSLPDDAERLRDKILASEASFAAEVSYNGEESYFF
VLEDSASGELVGCSAIVASAGFSEPFYSFRNETFVHASRSLSIHNKIHVLSLCHDLTGNSLLTS
FYVQRDLVQSVYAELNSRGRLLFMASHPERFADAVVVEIVGYSDEQGESPFWNAVGRNFFD
LNYIEAEKLSGLKSRTFLAELMPHYPIYVPLLPDAAQESMGQVHPRAQITFDILMREGFETD
NYIDIFDGGPTLHARTSGIRSIAQSRVVPVKIGEAPKSGRPYLVTNGQLQDFRAVVLDLDWA
PGKPVALSVEAAEALGVGEGASVRLVAV
00000000000000000000000000000000000000000000000000000000000000000000000000000000000
00000000000000000000000000000000000000000000000000000000000000000000000000000000000
00000000000000000000000000000000011111111110000000000000000000000000000000000000000
00000000000000000000000000000000011000000000000000000000000000000000000000000000000
000000
>Q92LT4_3lzk_0_337
MKLATLKDSTRDGKLVVVSKDLTRCSEVGHIARTLQAALDDWAHAGPRLERVAEGIETGA
QPTMRFHEHDAASPLPRAFQWADGSAYVNHVELVRKARNAEMPASFWTDPLIYQGGSDS
FLGPRDPILMADDAWGIDMEGEAAVIVDDVPMGATLDEAKAAIRLVMLVNDVSLRGLIPG
ELAKGFGFYQSKPSSAFSPVAVTPEELGEAWDGGKLHLPLHVDLNGEPFGRANAGIDMTFD
FPQLIVHAARTRPLSAGTIIGSGTVSNKLEGGPGRPVSEGGAGYSCIAELRMIETIEGGAPKT
QFLKFGDVVRIEMKDRTGHSIFGAIEQKVGKYERG
00000000000000000000000000000000000000000000000000000000000000000000000000000000000
00000000000000000000000000000000000000000000000000000000000000000000000000000000000
00000000000000000000000000000000000000000000000000000000000000000000000000000000000
00000000000000000000000000000000000000000000000000000000000000000000000000000000000
000001
>O08967_2r09_62_398
TTQRNKQIAMGRKKFNMDPKKGIQFLIENDLLQSSPEDVAQFLYKGEGLNKTVIGDYLGER
DDFNIKVLQAFVELHEFADLNLVQALRQFLWSFRLPGEAQKIDRMMEAFASRYCLCNPGV
FQSTDTCYVLSFAIIMLNTSLHNHNVRDKPTAERFITMNRGINEGGDLPEELLRNLYESIKNE
PFKIPEDDGNDLTHTFFNPDREGWLLKLGGRVKTWKRRWFILTDNCLYYFEYTTDKEPRGII
PLENLSIREVEDPRKPNCFELYNPSHKGQVIKACKTEADGRVVEGNHVVYRISAPSPEEKEE
WMKSIKASISRDPFYDMLATRKRRIANKK
00000000000000000000000000000000000000000000000000000000000000000000000000000000000
00000000000000000000000000000000000000000000000000000000000000000000000000000000000
00000000000000000000000000000000000000000000000000000000000000000000000000000000000
00000000000000000000000000000000000000000000000000000000000000000000000000000000000
00111
>P07996_1ux6_833_1169
DNCPLEHNPDQLDSDSDRIGDTCDNNQDIDEDGHQNNLDNCPYVPNANQADHDKDGK
GDACDHDDDNDGIPDDKDNCRLVPNPDQKDSDGDGRGDACKDDFDHDSVPDIDDICPE
NVDISETDFRRFQMIPLDPKGTSQNDPNWVVRHQGKELVQTVNCDPGLAVGYDEFNAVD
FSGTFFINTERDDDYAGFVFGYQSSSRFYVVMWKQVTQSYWDTNPTRAQGYSGLSVKVVN
STTGPGEHLRNALWHTGNTPGQVRTLWHDPRHIGWKDFTAYRWRLSHRPKTGFIRVVMY
EGKKIMADSGPIYDKTYAGGRLGLFVFSQEMVFFSDLKYECRDP
00000000000000000000000000000000000000000000000000000000000000000000000000000000000
00000000000000000000000000000000000000000000000000000000000000001111100000000000000
00000000000000000000000000000000000000000000000000000000000000000000000000000000000
00000000000000000000000000000000000000000000000000000000000000000000000000000000000
00000
>Q5ZRN7_3r2g_0_336
MTDQAITFDDVLLVPSYNHHESRRVVETTSTDRLGKLTLNLPVISANMDTITESNMANFMH
SKGAMGALHRFMTIEENIQEFKKCKGPVFVSVGCTENELQRAEALRDAGADFFCVDVAH

AHAKYVGKTLKSLRQLLGSRCIMAGNVATYAGADYLASCGADIIKAGIGGGSVCSTRIKTG
FGVPMLTCIQDCSRADRSIVADGGIKTSGDIVKALAFGADFVMIGGMLAGSAPTPGEVFQK
DDGSKVKRYRGMASREAQEAFLGQMHEWKTAEGVATEVPFKENPDGIIADIIGGLRSGLTY
AGADSISELQRKLNYVIVTQAGRIESLPHKLLEG
11100000000000000000000000000000000000000000000000000000000000000000000000000000000
00000000000000000000000000000000000000000000000000000000000000000000000000000000000
00000000000000000000000000000000000000000000000000000000000000000000000000000000000
00000000000000000000000000000000000000000000000000000000000000000000000000000111111
11111
>Q96XQ7_2z0m_0_336
MNEKIEQAIREMGFKNFTEVQSKTIPLMLQGKNVVVRAKTGSGKTAAYAIPILELGMKSLV
VTPTRELTRQVASHIRDIGRYMDTKVAEVYGGMPYKAQINRVRNADIVVATPGRLLDLWSK
GVIDLSSFEIVIIDEADLMFEMGFIDDIKIILAQTSNRKITGLFSATIPEEIRKVVKDFITNYEEIE
ACIGLANVEHKFVHVKDDWRSKVQALRENKDKGVIVFVRTRNRVAKLVRLFDNAIELRG
DLPQSVRNRNIDAFREGEYDMLITTDVASRGLDIPLVEKVINFDAPQDLRTYIHRIGRTGRM
GRKGEAITFILNEYWLEKEVKKVSQKA
00000000000000000000000000000000000000000000000000000000000000000000000000000000000
00000000000000000000000000000000000000000000000000000000000000000000000000000000000
00000000000000000000000000000000000000000000000000000000000000000000000000000000000
00000000000000000000000000000000000000000000000000000000000000000000000000000000001
11111
>Q9NFS4_3chk_0_336
MANKNYQMSTGVTAVVQKVVEACQDESKRLDLIEIARSYPPNQLRNMQRTFQAITGTFLD
AFLKKHLSKDFESLVLMLYKPRAQLLCELIRGATKGAGTDEKCLVDVLLTIETHEVREIRQLY
YQLYNDSLGDVVRKDCGDKYMWAKLINAVATGDRIPRDTHELEEDLVLVRKAIETKGVKK
DEVSTWIRIFATYTRADFRQLHKMYSAKYNGDSLRAGVEDEFQGLDEYAFKLAHDFLYDPC
CAAAFSMNVAFAGSGSDSNRLNRITAMHFRECKGCKYYYKKVYGQAFDERCATELKGVY
GDAIKLLWEPVTVPLLSMDDYQGSEQHRPMTLEL
11111111110000000000000000000000000000000000000000000000000000000000000000000000000
00000000000000000000000000000000000000000000000000000000000000000000000000000000000
00000000000000000000000000000000000000000000000000000000000000000000000000000000000
00000000000000000000000000000000000000000000000000000000000000000011111111111111111
11111
>P96398_2bi0_1_336
ASGYGGIRVGGPYFDDLSKGQVFDWAPGVTLSLGLAAAHQSIVGNRLRLALDSDLCAAVT
GMPGPLAHPGLVCDVAIGQSTLATQRVKANLFYRGLRFHRFPAVGDTLYTRTEVVGLRANS
PKPGRAPTGLAGLRMTTIDRTDRLVLDFYRCAMLPASPDWKPGAVPGDDLSRIGADAPAP
AADPTAHWDGAVFRKRVPGPHFDAGIAGAVLHSTADLVSGAPELARLTLNIAATHHDWR
VSGRRLVYGGHTIGLALAQATRLLPNLATVLDWESCDHTAPVHEGDTLYSELHIESAQAHA
DGGVLGLRSLVYAVSDSASEPDRQVLDWRFSALQF
11111100000000000000000000000000000000000000000000000000000000000000000000000000000
00000000000000000000000000000000000000000000000000000000000000000000000000000000000
00000000000000000000000000000000000000000000000000000000000000000000000000000000000
00000000000000000000000000000000000000000000000000000000000000000000000000000000000
0000
>Q57612_2apo_0_335
MILLEKTQEKKINDKEELIVKEEVETNWDYGCNPYERKIEDLIKYGVVVVDKPRGPTSHEVS
TWVKKILNLDKAGHGGTLDPKVTGVLPVALERATKTIPMWHIPPKEYVCLMHLHRDASEE
DILRVFKEFTGRIYQRPPLKAAVKRRLRIRKIHELELLDKDGKDVLFRVKCQSGTYIRKLCEDI
GEALGTSAHMQELRRTKSGCFEEKDAVYLQDLLDAYVFWKEDGDEEELRRVIKPMEYGLR
HLKKVVVKDSAVDAICHGADVYVRGIAKLSKGIGKGETVLVETLKGEAVAVGKALMNTKE

ILNADKGVAVDVERVYMDRGTYPRMWKRKK
1111111111111111000000000000000000000000000000000000000000000000000000000000000000000
0000000000000000000000000000000000000000000000000000000111111111110000000000000000000
00000000000000000000000000000000000000000000000000000000000000000000000000000000000
00000000000000000000000000000000000000000000000000000000000000000000000000000000001
1111
>Q99R58_3nur_0_335
MKSITFEEHYVIEDIQKETMNAISADPKGVPMKVMLEGLEKKTGFTNADELSHHDERIQFM
NNQDVQIQVLSYGNGSPSNLVGQKAIELCQKANDQLANYIAQYPNRFVGFATLPINEPEA
AAREFERCINDLGFKGALIMGRAQDGFLDQDKYDIIFKTAENLDVPIYLHPAPVNSDIYQSY
YKGNYPEVTAATFACFGYGWHIDVGIHAIHLVLSGIFDRYPKLNMIIGHWGEFIPFFLERMD
EALFAEHLNHSVSYYFKNSFYITPSGMLTKPQFDLVKKEVGIDRILYAADYPYIEPEKLGVFL
DELGLTDEEKEKISYTNGAKLLGLSSNN
00000000000000000001111111111111111111111111110000000000000000000000000000000000000
00000000000000000000000000000000000000000000000000000000000000000000000000000000000
00000000000000000000000000000000000000000000000000000000000000000000000000000000000
00000000000000000000000000000000000000000000000000000000000000000000000000000000000
1111
>Q9AIP9_2vov_204_539
GLDTLDRDGDGSTADADCNDFAPTIHPGAAEATLDGVDSNCDGRDSGVAEVVETFKNPG
TYSSPVINFKIASPPGPGTPIYGPPRDFSGYNKSYSLAIGKTSYYDPTTGTKWNDDTITPVSDG
QDIWRGWTHTGKWSFFNGKAGDKITLSVQRDAQEASLKGAHPGFILFWRPEGGPLFWAG
TQDLDEGQTALPADSDTVIGHVIVQHADWTLQGLPPKADHTAPAGVDTELYPMKPDSYT
MYYVDSGYDADKYVASKKLIMHPTAFKGLALNDGTAGAFTKSITLPKTGYYMLYVANVLE
VDDWSVDADGKLTTTGEVWEVPAKGCWVNITISKP
11111111111111111111111111111111111111111111110000000000000000000000000000000000000
00000000000000000000000000000000000000000000000000000000000000000000000000000000000
00000000000000000000000000000000000000000000000000000000000000000000000000000000000
00000000000000000000000000000000000000000000000000000000000000000000000000000000000
0000
>Q9EY50_3gkr_0_335
MPVLNLNDPQAVERYEEFMRQSPYGQVTQDLGWAKVKNNWEPVDVYLEDDQGAIIAAM
SMLLGDTPTDKKFAYASKGPVMDVTDVDLLDRLVDEAVKALDGRAYVLRFDPEVAYSDEF
NTTLQDHGYVTRNRNVADAGMHATIQPRLNMVLDLTKFPDAKTTLDLYPSKTKSKIKRPF
RDGVEVHSGNSATELDEFFKTYTTMAERHGITHRPIEYFQRMQAAFDADTMRIFVAEREGK
LLSTGIALKYGRKIWYMYAGSMDGNTYYAPYAVQSEMIQWALDTNTDLYDLGGIESESTDD
SLYVFKHVFVKDAPREYIGEIDKVLDPEVYAELVKD
10000000000000000000000000000000000000000000000000000000000000000000000000000000000
00000000000000000000000000000000000000000000000000000000000000000000000000000000000
00000000000000000000000000000000000000000000000000000000000000000000000000000000000
00000000000000000000000000000000000000000000000000000000000000000000000000000000000
0000
>Q9KSQ2_1xfk_0_335
MNPNFTTEHTWQGRHDPEDGQAGRRVHHIACPIQVGELANQEPGVALIGFECDAGVERN
KGRTGAKHAPSLIKQALANLAWHHPIPIYDLGNIRCEGDELEQAQQECAQVIQQALPHAR
AIVLGGGHEIAWATFQGLAQHFLATGVKQPRIGIINFDAHFDLRTFESELAPVRPSSGTPFN
QIHHFCQQQGWDFHYACLGVSRASNTPALFERADKLGVWYVEDKAFSPLSLKDHLTQLQ
HFIDDCDYLYLTIDLDVFPAASAPGVSAPAARGVSLEALAPYFDRILHYKNKLMIADIAEYN
PSFDIDQHTARLAARLCWDIANAMAEQVQSIRHP
11111111100000000000000000000000000000000000000000000000000000000000000000000000000
00000000000000000000000000000000000000000000000000000000000000000000000000000000000

00000000000000000000000000000000000000000000000000000000000000000000000000000000000
00000000000000000000000000000000000000000000000000000000000000000000000000000000000
0111
>O57746_3u4g_0_334
MKKVKSLFLLVLGNTEISTIPGISVAGATPELTKITPVADAEYLFYEKPLTIDTIPVTPEGHPTP
AIITKAARELSNFPILVVRGGTYLAPLIPHVHISNVVGRDFRREPALPEVEVIIERAKLLGKELE
KIANEIVIGESTPGGTTTAQAILWALGYEAKTSSASPENPQELKEKVIKEGFKRVGIEKGGLK
DKPLEALKEFGDPMIATVLGLSLGFGGDVVLAGGTQMLAVSALLKSLGEDLSRFMIATTRW
VVEDPSATFIETAREIGIISYVAELDFSKSKFKGLRDYERGYVKEGVGAGGATWLAVKAGYSP
EDVSKKVEELYERLMGLR
00000000000000000000000000000000000000000000000000000000000000000000000000000000000
00000000000000000000000000000000000000000000000000000000000000000000000000001111111
11000000000000000000000000000000000000000000000000000000000000000000000000000000000
00000000000000000000000000000000000000000000000000000000000000000000000000000000000
000
>Q0ZPV7_2o7r_0_334
MSNDHLETTGSSDPNTNLLKYLPIVLNPDRTITRPIQIPSTAASPDPTSSSPVLTKDLALNPLH
NTFVRLFLPRHALYNSAKLPLVVYFHGGGFILFSAASTIFHDFCCEMAVHAGVVIASVDYRL
APEHRLPAAYDDAMEALQWIKDSRDEWLTNFADFSNCFIMGESAGGNIAYHAGLRAAAV
ADELLPLKIKGLVLDEPGFGGSKRTGSELRLANDSRLPTFVLDLIWELSLPMGADRDHEYCN
PTAESEPLYSFDKIRSLGWRVMVVGCHGDPMIDRQMELAERLEKKGVDVVAQFDVGGYH
AVKLEDPEKAKQFFVILKKFVVDSCTTKL
11111111111111111000000000000000000000000000000000000000000000000000000000000000000
00000000000000000000000000000000000000000000000000000000000000000000000000000000000
00000000000000000000000000000000000000000000000000000000000000000000000000000000000
11110000000000000000000000000000000000000000000000000000000000000000000000000001111
111
>Q2A612_3it2_16_350
VGYSSKLIFVSMITRHGDRAPFANIENANYSWGTELSELTPIGMNQEYNLGLQLRKRYIDKF
GLLPEHYVDQSIYVLSSHTNRTVVSAQSLLMGLYPAGTGPLIGDGDPAIKDRFQPIPIMTLSA
DSRLIQFPYEQYLAVLKKYVYNSPEWQNKTKEAAPNFAKWQQILGNRISGLNDVITVGDVL
IVAQAHGKPLPKGLSQEDADQIIALTDWGLAQQFKSQKVSYIMGGKLTNRMIEDLNNAVN
GKSKYKMTYYSGHDLTLLEVMGTLGVPLDTAPGYASNLEMELYKDGDIYTVKLRYNGKYV
KLPIMDKNNSCSLDALNKYMQSINEKFQK
11110000000000000000000000000000000000000000000000000000000000000000000000000000000
00000000000000000000000000000000000000000000000000000000000000000000000000000000000
00000000000000000000000000000000000000000000000000000000000000000000000000000000000
00000000000000000000000000000000000000000000000000000000000000000000000000000000000
011
>Q54324_3m1m_35_369
SSNFSSERIRYAKWFLEHGFNIIPIDPESKKPVLKEWQKYSHEMPSDEEKQRFLKMIEEGYNY
AIPGGQKGLVILDFESKEKLKAWIGESALEELCRKTLCTNTVHGGIHIYVLSNDIPPHKINPL
FEENGKGIIDLQSYNSYVLGLGSCVNHLHCTTDKCPWKEQNYTTCYTLYNELKEISKVDLK
SLLRFLAEKGKRLGITLSKTAKEWLEGKKEEEDTVVEFEELRKELVKRDSGKPVEKIKEEICT
KSPPKLIKEIICENKTYADVNIDRSRGDWHVILYLMKHGVTDPDKILELLPRDSKAKENEKW
NTQKYFVITLSKAWSVVKKYLEA
11110000000000000000000000000000000000000000000000000000000000000000000000000000000
00000000000000000000000000000000000000000000000000000000000000000000000000000000000
00000000000000000000000000000000000000000000000011111111111000000000000000000000000
00000000000000000000000000000000000000000000000000000000000000000000000000000000000
000

>Q6D5A0_2p2s_0_334
MKKIRFAAIGLAHNHIYDMCQQLIDAGAELAGVFESDSDNRAKFTSLFPSVPFAASAEQLIT
DASIDLIACAVIPCDRAELALRTLDAGKDFFTAKPPLTTLEQLDAVQRRVAETGRKFAVYFN
ERINVDSALFAGELVQRGEIGRVIQTMGVGPHRERGARPDWFYQKRQYGGILCDIGIHQIEQ
FLYFTGNTNARVVTSQTANYHHPHHPEFEDFGDAMLLGDNGATGYFRCDWFTPDGLSVW
GDGRLTILGTEGYIEIRKYVDLTRGESNVVYLVNGKGEQRFTPAGSVERAFFPDFLRDCRERT
ENAMSQSHIFKATELSILAQQAANKIA
10000000000000000000000000000000000000000000000000000000000000000000000000000000000
00000000000000000000000000000000000000000000000000000000000000000000000000000000000
00000000000000000000000000000000000000000000000000000000000000000000000000000000000
00000000000000000000000000000000000000000000000000000000000000000000000000000000000
000
>Q7SIC7_1wx1_0_334
MDPEVFAQARLRMDQLTKPPRALGYLEEVALRLAALQGRVKPELGRGAVVVAAADHGVV
AEGVSAYPQEVTRQMVLNFLRGGAAINQFALAADCAVYVLDVGVVGELPDHPGLLKRKV
RPGTANLAQGPAMTPEEAERALLAGREAARRAIAEGATLLAAGDMGIGNTTAAAALTAA
LLGLPPEAVVGRGTGVGEEGLRRKRQAVARALARLHPGMGPLEVAAEVGGLELVAIAGIYL
EGYEAGLPLVLDGFPVTAGALLAWKMAPGLRDHLFAGHLSREPGHRHQLEALGLRPLLDL
DLALGEGTGAVLAMPLLRAAARILHMATFQEAGVSRG
00000000000000000000000000000000000000000000000000000000000000000000000000000000000
00000000000000000000000000000000000000000000000000000000000000000000000000000000000
00000000000000000000001111100000000000000000000000000000000000000000000000000000000
00000000000000000000000000000000000000000000000000000000000000000000000000000000000
000
>Q8DR49_3gon_0_334
MIAVKTCGKLYWAGEYAILEPGQLALIKDIPIYMRAEIAFSDSYRIYSDMFDFAVDLRPNPDY
SLIQETIALMGDFLAVRGQNLRPFSLAIYGKMEREGKKFGLGSSGSVVVLVVKALLALYNLS
VDQNLLFKLTSAVLLKRGDNGSMGDLACIAAEDLVLYQSFDRQKVAAWLEEENLATVLER
DWGFSISQVKPTLECDFLVGWTKEVAVSSHMVQQIKQNINQNFLTSSKETVVSLVEALEQG
KSEKIIEQVEVASKLLEGLSTDIYTPLLRQLKEASQDLQAVAKSSGAGGGDCGIALSFDAQST
KTLKNRWADLGIELLYQERIGHDDKS
00000000000000000000000000000000000000000000000000000000000000000000000000000000000
00000000000000000000000000000000000000000000000000000000000000000000000000000000000
00000000000000000000000000000000000000000000000000000000000000000000000000000000000
00000000000000000000000000000000000000000000000000000000000000000000000000000000111
111
>P15132_3csq_0_333
MVYVSNKYLTMSEMKVNAQYILNYLSSNGWTKQAICGMLGNMQSESTINPGLWQNLDE
GNTSLGFGLVQWTPASNYINWANSQGLPYKDMDSELKRIIWEVNNNAQWINLRDMTFKE
YIKSTKTPRELAMIFLASYERPANPNQPERGDQAEYWYKNLSGGGGGGLQLAQFPMDIINI
SQGENGSFSHKGTLCIDFVGKTEKYPYYAPCDCTCVWRGDASAYLAWTSDKEVMCADGSV
RYITWVNVHESPLPFDVGKKLKKGDLMGHTGIGGNVTGDHWHFNVIDGKEYQGWTKKP
DSCLAGTELHIYDVFAVNNVEIINGNGYDWKTSDWQDG
00000000000000000000000000000000000000000000000000000000000000000000000000000000000
00000000000000000000000000000000000000000000000000000000000000000000000000000111110
00000000000000000000000000000000000000000000000000000000000000000000000000000000000
00000000000000000000001111000000000000000000000000000000000000000000000000000000000
00
>P17707_1jl0_0_333
MEAAHFFEGTEKLLEVWFSRQQPDANQGSGDLRTIPRSEWDILLKDVQCSIISVTKTDKQE
AYVLSESSMFVSKRRFILKTCGTTLLLKALVPLLKLARDYSGFDSIQSFFYSRKNFMKPSHQG

YPHRNFQEEIEFLNAIFPNGAAYCMGRMNSDCWYLYTLDFPESRVISQPDQTLEILMSELDP
AVMDQFYMKDGVTAKDVTRESGIRDLIPGSVIDATMFNPCGYSMNGMKSDGTYWTIHITP
EPEFSYVSFETNLSQTSYDDLIRKVVEVFKPGKFVTTLFVNQSSKCRTVLASPQKIEGFKRLDC
QSAMFNDYNFVFTSFAKKQQQQS
11110000000000000000011111000000000000000000000000000000000000000000000000000000000
0000000000000000000000000000000000000000000000000000000000000000000000000000000001
1000000000000000000000000000000000000000000000000000000000000000000000000000000000
00000000000000000000000000000000000000000000011111100000000000000000000000000000011
11
>P53355_2xuu_0_333
MTVFRQENVDDYYDTGEELGSGQFAVVKKCREKSTGLQYAAKFIKKRRTKSSRRGVSREDIE
REVSILKEIQHPNVITLHEVYENKTDVILILELVAGGELFDFLAEKESLTEEEATEFLKQILNGV
YYLHSLQIAHFDLKPENIMLLDRNVPKPRIKIIDFGLAHKIDFGNEFKNIFGTPEFVAPEIVNY
EPLGLEADMWSIGVITYILLSGASPFLGDTKQETLANVSAVNYEFEDEYFSNTSALAKDFIRR
LLVKDPKKRMTIQDSLQHPWIKPKDTQQALSRKASAVNMEKFKKFAARKKWKQSVRLISL
CQRLSRSFLSRSNMSVARSD
10000000000000000000000000000000000000000000000000000000000000000000000000000000000
00000000000000000000000000000000000000000000000000000000000000000000000000000000000
00000000000000000000000000000000000000000000000000000000000000000000000000000000000
00000000000000000000000000000000000000000000000000000111111111111111111111111111111
11
>P61964_2gnq_0_333
MATEEKKPETEAARAQPTPSSSATQSKPTPVKPNYALKFTLAGHTKAVSSVKFSPNGEWLAS
SSADKLIKIWGAYDGKFEKTISGHKLGISDVAWSSDSNLLVSASDDKTLKIWDVSSGKCLKTL
KGHSNYVFCCNFNPQSNLIVSGSFDESVRIWDVKTGKCLKTLPAHSDPVSAVHFNRDGSLI
VSSSYDGLCRIWDTASGQCLKTLIDDDNPPVSFVKFSPNGKYILAATLDNTLKLWDYSKGKC
LKTYTGHKNEKYCIFANFSVTGGKWIVSGSEDNLVYIWNLQTKEIVQKLQGHTDVVISTAC
HPTENIIASAALENDKTIKLWKSDC
11111111110000000011111111000000000000000000000000000000000000000000000000000000000
00000000000000000000000000000000000000000000000000000000000000000000000000000000000
00000000000000000000000000000000000000000000000000000000000000000000000000000000000
00000000000000000000000000000000000000000000000000000000000000000000000000000000000
00
>Q3M5E3_2qw5_0_333
MTKLPATSDIYISFFMFTTNLQPDNLDYRRIVVAHIKKLQRFGYSGFEFPIAPGLPENYAQDL
ENYTNLRHYLDSEGLENVKISTNVGATRTFDPSSNYPEQRQEALEYLKSRVDITAALGGEIM
MGPIVIPYGVFPTTDFNEPIWSDELQEHLKVRYANAQPILDKLGEYAEIKKVKLAIEPITHWE
TPGPNKLSQLIEFLKGVKSKQVGVVIDSAHEILDGEGPEIFKTQVEYLAQQGRLHYVQVSPP
DRGALHTSWLPWKSFLTPIVKVYDGPIAVEIFNAIPAFTNSLRLTRRKFWIPDEDPPNQYPN
AYDIADEAIKVTRKELKKIGSK
11111000000000000000000000000000000000000000000000000000000000000000000000000000000
00000000000000000000000000000000000000000000000000000000000000000000000000000000000
00000000000000000000000000000000000000000000000000000000000000000000000000000000000
00000000000000000000000000000000000000000000000000000000000000000000000000000000000
11
>Q9HWH2_2ip2_0_333
MNNSNLAAARNLIQVVTGEWKSRCVYVATRLGLADLIESGIDSDETLAAAVGSDAERIHRL
MRLLVAFEIFQGDTRDGYANTPTSHLLRDVEGSFRDMVLFYGEEFHAAWTPACEALLSGTP
GFELAFGEDFYSYLKRCPDAGRRFLLAMKASNLAFHEIPRLLDFRGRSFVDVGGGSGELTK
AILQAEPSARGVMLDREGSLGVARDNLSSLLAGERVSLVGGDMLQEVPSNGDIYLLSRIIGD
LDEAASLRLLGNCREAMAGDGRVVVIERTISASEPSPMSVLWDVHLFMACAGRHRTTEEV

VDLLGRGGFAVERIVDLPMETRMIVAARA
11110000000000000000000000000000000000000000000000000000000000000000000000000000000
00000000000000000000000000000000000000000000000000000000000000000000000000000000000
00000000000000000000000000000000000000000000000000000000000000000000000000000000000
00000000000000000000000000000000000000000000000000000000000000000000000000000000000
00
>Q9UGP5_3hw8_241_574
AQPSSQKATNHNLHITEKLEVLAKAYSVQGDKWRALGYAKAINALKSFHKPVTSYQEACSI
PGIGKRMAEKIIEILESGHLRKLDHISESVPVLELFSNIWGAGTKTAQMWYQQGFRSLEDIRS
QASLTTQQAIGLKHYSDFLERMPREEATEIEQTVQKAAQAFNSGLLCVACGSYRRGKATCG
DVDVLITHPDGRSHRGIFSRLLDSLRQEGFLTDDLVSQEENGQQQKYLGVCRLPGPGRRHR
RLDIIVVPYSEFACALLYFTGSAHFNRSMRALAKTKGMSLSEHALSTAVVRNTHGCKVGPG
RVLPTPTEKDVFRLLGLPYREPAERDW
11111110000000000000000000000000000000000000000000000000000000000000000000000000000
00000000000000000000000000000000000000000000000000000000000000000000000000000000000
00000000000000000000000000000000000000000000000000000000000000000000000000000000000
00000000000000000000000000000000000000000000000000000000000000000000000000000000000
00
>P07617_1vp3_0_332
MDVVSLDKPFMYFEEIDNELDYEPESANEVAKKLPYQGQLKLLLGELFFLSKLQRHGILDG
ATVVYIGSAPGTHIRYLRDHFYNLGVIIKWMLIDGRHHDPILNGLRDVTLVTRFVDEEYLRSI
KKQLHPSKIILISDVRSKRGGNEPSTADLLSNYALQNVMISILNPVASSLKWRCPFPDQWIKD
FYIPHGNKMLQPFAPSYSAEMRLLSIYTGENMRLTRVTKSDAVNYEKKMYYLNKIVRNKVV
VNFDYPNQEYDYFHMYFMLRTVYCNKTFPTTKAKVLFLQQSIFRFLNIPTTSTEKVSHEPIQ
RKISSKNSMSKNRNSKRSVRSNK
00000000000000000000000000000000000000000000000000000000000000000000000000000000000
00000000000000000000000000000000000000000000000000000000001111100000000000000000000
00000000000000000000000000000000000000000000000000000000000000000000000000000000000
00000000000000000000000000000000000000000000000011111111111111111111111111111111111
1
>P13280_1zcv_0_332
MTDQAFVTLTTNDAYAKGALVLGSSLKQHRTSRRLAVLTTPQVSDTMRKALEIVFDEVITV
DILDSGDSAHLTLMKRPELGVTLTKLHCWSLTQYSKCVFMDADTLVLANIDDLFEREELSA
APDPGWPDCFNSGVFVYQPSVETYNQLLHVASEQGSFDGGDQGLLNTFFNSWATTDIRKH
LPFIYNLSSISIYSYLPAFKAFGANAKVVHFLGQTKPWNYTYDTKTKSVRSEGHDPTMTHPQ
FLNVWWDIFTTSVVPLLQQFGLVQDTCSYQHVEDVSGAVSHLSLGETPATTQPFVSSEERKE
RWEQGQADYMGADSFDNIKKKLDTYLQ
00000000000000000000000000000000000000000000000000000000000000000000000000000000000
00000000000000000000000000000000000000000000000000000000000000000000000000000000000
00000000000000000000000000000000000000000000000000000000000000000001111100000000000
00000000000000000111111111111111111111111111111111111111111111111111111111111111111
1
>Q84AQ4_3u52_0_332
MSIEIKTNSVEPIRHTYGHIARRFGDKPATRYQEASYDIEAKTNFHYRPQWDSEHTLNDPTR
TAIRMEDWCAVSDPRQFYYGAYVGNRAKMQESAETSFGFCEKRNLLTRLSEETQKQLLRLL
VPLRHVELGANMNNAKIAGDATATTVSQMHIYTGMDRLGIGQYLSRIALMIDGSTGAALD
ESKAYWMDDEMWQPMRKLVEDTLVVDDWFELTLVQNILIDGMMYPLVYDKMDQWFESQ
GAEDVSMLTEFMRDWYKESLRWTNAMMKAVAGESETNRELLQKWIDHWEPQAYEALKP
LAEASVGIDGLNEARAELSARLKKFELQSRGVSA
11100000000000000000000000000000000000000000000000000000000000000000000000000000000
00000000000000000000000000000000000000000000000000000000000000000000000000000000000

00000000000000000000000000000000000000000000000000000000000000000000000000000000000
00000000000000000000000000000000000000000000000000000000000000000000000000000000001
1
>C4Z2B5_4hn9_23_354
GNSTTSDKGTEEATTSAFDVMSQFNEIGVSYPLTVTDQAGRTVTFEKAPEKIASSYYISTSLLL
ALGLQDKLVGIEAKANTRNIYKLAAPAIVSLPNMGTAKEFNTEACVAATPDVVFLPMKLK
KTADTLESLGIKAVVVNPEDQSLLEECITLVGKITNNAGRAEALNNSIKTFLADNKTNVSGG
NTPSVYLAGNSSVLSTAGSKMYQNTLLTNAGGKNVASELTDTYWANVSYEQILAWNPDYI
VIAADATYTVDDILNDANLAGCNAVKNKNVVKLPNNIEAWDSPVPGSFLGSIYIASVLHPE
KVTKDFYETCVTKFYESFYGFTPAK
11111111000000000000000000000000000000000000000000000000000000000000000000000000000
00000000000000000000000000000000000000000000000000000000000000000000000000000000000
00000000000000000000000000000000000000000000000000000000000000000000000000000000000
00000000000000000000000000000000000000000000000000000000000000000000000000000000001
>P24305_2fgq_19_350
QSSVTLFGIVDTNVAYVNKDAAGDSRYGLGTSGASTSRLGLRGTEDLGGGLKAGFWLEGEI
FGDDGNASGFNFKRRSTVSLSGNFGEVRLGRDLVPTSQKLTSYDLFSATGIGPFMGFRNWA
AGQGADDNGIRANNLISYYTPNFGGFNAGFGYAFDEKQTIGTADSVGRYIGGYVAYDNGP
LSASLGLAQQKTAVGGLATDRDEITLGASYNFGVAKLSGLLQQTKFKRDIGGDIKTNSYML
GASAPVGGVGEVKLQYALYDQKAIDSKAHQITLGYVHNLSKRTALYGNLAFLKNKDASTL
GLQAKGVYAGGVQAGESQTGVQVGIRHAF
11000000000000000000000000000000000000000000000000000000000000000000000000000000000
00000000000000000000000000000000000000000000000000000000000000000000000000000000000
00000000000000000000000000000000000000000000000000000000000000000000000000000000000
00000000000000000000000000000000000000000000000000000000000000000000000000000000000
>P35150_3mfd_26_357
AAIDVSAKSAIIIDGASGRVLYAKDEHQKRRIASITKIMTAVLAIESGKMDQTVTVSANAVRT
EGSAIYLTEGQKVKLKDLVYGLMLRSGNDAAVAIAEHVGGSLDGFVYMMNQKAEQLGMK
NTRFQNPHGLDDHENHYSTAYDMAILTKYAMKLKDYQKISGTKIYKAETMESVWKNKNK
LLTMLYPYSTGGKTGYTKLAKRTLVSTASKDGIDLIAVTINDPNDWDDHMKMFNYVFEHY
QTYLIAKKGDIPKLKGTFYESKAFIKRDITYLLTEEEKENVKINTTLLKPKKAWEKDASKIPDI
VGHMEIMFNDATIAKVPIYYENERHQK
10000000000000000000000000000000000000000000000000000000000000000000000000000000000
00000000000000000000000000000000000000000000000000000000000000000000000000000000000
00000000000000000000000000000000000000000000000000000000000000000000000000000000000
00000000000000000000000000000000000000000000000000000000000000000000000000000011111
>Q5SL04_2ejw_0_331
MEALKIALLGGGTVGSAFYNLVLERAEELSAFGVVPRFLGVLVRDPRKPRAIPQELLRAEPF
DLLEADLVVEAMGGVEAPLRLVLPALEAGIPLITANKALLAEAWESLRPFAEEGLIYHEASV
MAGTPALSFLETLRGSELLELHGILNGTTLYILQEMEKGRTYAEALLEAQRLGYAEADPTLD
VEGIDAAHKLTLLARLLVDPGFPFAEVEAQGIARLTPEVLQKAEARGERVRLVASLFGEGGR
WRAAVAPRRLPQDHPLARARGNALWVRARPLGEAFVTGPGAGGGATASGLFADLLRFLS
GAPGHLPAPRARPPLEEGSPWPGVE
00000000000000000000000000000000000000000000000000000000000000000000000000000000000
00000000000000000000000000000000000000000000000000000000000000000000000000000000000
00000000000000000000000000000000000000000000000000000000000000000000000000000000000
00000000000000000000000000000000000000000000000000000000000000000000000000000000001
>Q6D5T7_3ktc_0_331
MATYNYPEFGAGLWHFANYIDRYAVDGYGPALSTIDQINAAKEVGELSYVDLPYPFTPGVT
LSEVKDALKDAGLKAIGITPEIYLQKWSRGAFTNPDPAARAAAFELMHESAGIVRELGANY
VKVWPGQDGWDYPFQVSHKNLWKLAVDGMRDLAGANPDVKFAIEYKPREPRVKMTWD

SAARTLLGIEDIGLDNVGVLLDFGHALYGGESPADSAQLIIDRGRLFGMDVNDNLRGWDD
DLVVGTVHMTEIFEFFYVLKINNWQGVWQLDQFPFRENHVEAAQLSIRFLKHIYRALDKL
DIPALQAAQEAQNPLQAQRIVQDALLSSITVSE
10000000000000000000000000000000000000000000000000000000000000000000000000000000000
00000000000000000000000000000000000000000000000000000000000000000000000000000000000
00000000000000000000000000000000000000000000000000000000000000000000000000000000000
00000000000000000000000000000000000000000000000000000000000000000000000000000000001
>Q9Z4Z5_2og5_1_332
AANAAGPASRYDVTLDQSDAELVEEIAWKLATQATGRPDDAEWVEAARNAWHAWPATL
RRDLAGFRRDSGPDGAIVLRGLPVDSMGLPPTPRVNGSVQREASLGAAVLLMTACGLGDP
GAFLPEKNGALVQDVVPVPGMEEFQGNAGSTLLTFHNENAFHEHRPDFVMLLCLRADPT
GRAGLRTACVRRVLPLLSDSTVDALWAPEFRTAPPPSFQLSGPEEAPAPVLLGDRSDPDLRV
DLAATEPVTERAAEALRELQAHFDATAVTHRLLPGELAIVDNRVTVHGRTEFTPRYDGTDR
WLQRTFVLTDLRRSRAMRPADGYVLGAAPQPA
11111111000000000000000000000000000000000000000000000000000000000000000000000000000
00000000000000000000000000000000000000000000000000000000000000000000000000000000000
00000000000000000000000000000000000000000000000000111111000000000000000000000000000
00000000000000000000000000000000000000000000000000000000000000000000000000000000111
>H7CHS4_3vpz_1_331
SLHSSAQFDPILVADIGGTNARFALITAFDAAKNEFVIEYNHTFPSADFGSLQNATRHYLSTV
PHIKPVRACLAVAGPIKAGQVHLTNLGWHFSVSEFKQAFSFLQLEVINDFAAFAYAAPYLDS
NQNVVIKAGQADENSNIAVMGPGTGFGAACLVRTAQSSAVLSSEGGHISLAAVTDLDAKL
LIELRKEHPHVSLETVFSGPGIAHLYKAMAAVNGITAKHLDAAQISNLANTGECEVCDATL
NQFCDWLGSAAGDLALAYGALGGLFIGGGILPRMQSRLLESRFVERFSQKGIMSQYNGQVP
VTLVTQDNIPLIGAAACLHNSKQE
11111100000000000000000000000000000000000000000000000000000000000000000000000000000
00000000000000000000000000000000000000000000000000000000000000000000000000000000000
00000000000000000000000000000000000000000000000000000000000000000000000000000000000
0000000000000000000000000000000000000000000000000000000000000000000000000000000111
>O06916_3i1a_0_330
MLKQPIQAQQLIELLKVHYGIDIHTAQFIQGGADTNAFAYQADSESKSYFIKLKYGYHDEIN
LSIIRLLHDSGIKEIIFPIHTLEAKLFQQLKHFKIIAYPFIHAPNGFTQNLTGKQWKQLGKVLR
QIHETSVPISIQQQLRKEIYSPKWREIVRSFYNQIEFDNSDDKLTAAFKSFFNQNSAAIHRLVD
TSEKLSKKIQPDLDKYVLCHSDIHAGNVLVGNEESIYIIDWDEPMLAPKERDLMFIGGGVG
NVWNKPHEIQYFYEGYGEINVDKTILSYYRHERIVEDIAVYGQDLLSRNQNNQSRLESFKYF
KEMFDPNNVVEIAFATEQ
10000000000000000000000000000000000000000000000000000000000000000000000000000000000
00000000000000000000000000000000000000000000000000000000000000000000000000000000000
00000000000000000000000000000000000000000000000000000000000000000000000000000000000
0000000000000000000000000000000000000000000000000000000000000000000000000000000001
>O62305_2bdw_0_542
MMNASTKFSDNYDVKEELGKGAFSVVRRCVHKTTGLEFAAKIINTKKLSARDFQKLEREA
RICRKLQHPNIVRLHDSIQEESFHYLVFDLVTGGELFEDIVAREFYSEADASHCIQQILESIAYC
HSNGIVHRDLKPENLLLASKAKGAAVKLADFGLAIEVNDSEAWHGFAGTPGYLSPEVLKK
DPYSKPVDIWACGVILYILLVGYPPFWDEDQHRLYAQIKAGAYDYPSPEWDTVTPEAKSLID
SMLTVNPKKRITADQALKVPWICNRERVASAIHRQDTVDCLKKFNARRKLKAAILGRNLL
NKKEQGPPSTIKESSESSQTIDDN
11110000000000000000000000000000000000000000000000000000000000000000000000000000000
00000000000000000000000000000000000000000000000000000000000000000000000000000000000
00000000000000000000000000000000000000000000000000000000000000000000000000000000000
0000000000000000000000000000000000000000000000000000000111111111111111111111111111

>O66809_2r75_0_330
MEEFVNPCKIKVIGVGGGGSNAVNRMYEDGIEGVELYAINTDVQHLSTLKVPNKIQIGEKV
TRGLGAGAKPEVGEEAALEDIDKIKEILRDTDMVFISAGLGGGTGTGAAPVIAKTAKEMGI
LTVAVATLPFRFEGPRKMEKALKGLEKLKESSDAYIVIHNDKIKELSNRTLTIKDAFKEVDSVL
SKAVRGITSIVVTPAVINVDFADVRTTLEEGGLSIIGMGEGRGDEKADIAVEKAVTSPLLEGN
TIEGARRLLVTIWTSEDIPYDIVDEVMERIHSKVHPEAEIIFGAVLEPQEQDFIRVAIVATDFPE
EKFQVGEKEVKFKVIKK
11111110000000000000000000000000000000000000000000000000000000000000000000000000000
00000000000000000000000000000000000000000000000000000000000000000000000000000000000
00000000000000000000000000000000000000000000000000000000000000000000000000000000000
0000000000000000000000000000000000000000000000000000000000000000000000000000000001
>P05326_2bu9_0_330
MGSVSKANVPKIDVSPLFGDDQAAKMRVAQQIDAASRDTGFFYAVNHGINVQRLSQKTKE
FHMSITPEEKWDLAIRAYNKEHQDQVRAGYYLSIPGKKAVESFCYLNPNFTPDHPRIQAKT
PTHEVNVWPDETKHPGFQDFAEQYYWDVFGLSSALLKGYALALGKEENFFARHFKPDDTL
ASVVLIRYPYLDPYPEAAIKTAADGTKLSFEWHEDVSLITVLYQSNVQNLQVETAAGYQDIE
ADDTGYLINCGSYMAHLTNNYYKAPIHRVKWVNAERQSLPFFVNLGYDSVIDPFDPREPN
GKSDREPLSYGDYLQNGLVSLINKNGQT
11000000000000000000000000000000000000000000000000000000000000000000000000000000000
00000000000000000000000000000000000000000000000000000000000000000000000000000000000
00000000000000000000000000000000000000000000000000000000000000000000000000000000000
0000000000000000000000000000000000000000000000000000000000000000000000000000000000
>P09871_1elv_357_687
DCGIPESIENGKVEDPESTLFGSVIRYTCEEPYYYMENGGGGEYHCAGNGSWVNEVLGPEL
PKCVPVCGVPREPFEEKQRIIGGSDADIKNFPWQVFFDNPWAGGALINEYWVLTAAHVVE
GNREPTMYVGSTSVQTSRLAKSKMLTPEHVFIHPGWKLLEVPEGRTNFDNDIALVRLKDPV
KMGPTVSPICLPGTSSDYNLMDGDLGLISGWGRTEKRDRAVRLKAARLPVAPLRKCKEVKV
EKPTADAEAYVFTPNMICAGGEKGMDSCKGDSGGAFAVQDPNDKTKFYAAGLVSWGPQC
GTYGLYTRVKNYVDWIMKTMQENSTPRED
00000000000000000000000000000000000011000000000000000000000000000000000000111110000
00000000000000000000000000000000000000000000000000000111111000000000000000000000000
00000000000000000000000000000000000000000000000000000000000000000000000000011111111
1100000000000000000000000000000000000000000000000000000000000000000000000000011111
>Q5ZY11_2im9_25_355
SQTTNIKEQTDTGLAPPAPAITTNSAAIEEQANSSIRKLYHTLNTMPNTSMADRISQISAYFK
GTKYILGSLGEGPNARYDQFPRYRVDGFDCDTYVNTVLSLALANSLESFQECLKHTRYKNG
KRSYINRNHFTSIDWNNYNQKRGLLKDITFSIRNEKKQPVALYANALINKPQWYNHKTIDT
IRLQKQDKNEQEKRLVELKAKGKTLETSLSNVPYIPFTALFSENKPNLHLFSQIPNGAVIEIIR
PNWDLRQQIGTELDISHLGFAIWINNELFFRQASSQYGKVVDVSLIDYLDKARSSPTIKGINI
QVVLPEKPVSNGCQLFDIR
11111111111111111111110000000000000000000011100000000000000000000000000000000000000
00000000000000000000000000000000000000000000000000000000000000000000000000000000000
00000000000000000000000000000000000000000000000000000000000000000000000000000000000
0000000000000000000000000000000000000000000000000000000000000000000000001110000111
>B9PW60_4ir8_0_329
MESPSALPSLDQLLKEQGADQTLTDLILAILDRCGKIASALQGTSVDKVGSVNEFGDEQLTV
DVIAENLLRSWAQSSEGSAVRAVCSEEDIHLQECHKNGEFILCWDPLDGSSIIDCNWAVGSI
VSIWRIGHHGVQWQGADTLIQKTGRQQVASLIVVYGPRTTGVVAVNVDAGGIVKEGTALD
LEMKDNGKFICRGKPIIKPQAKIFSPANLRAAQDLPAYKQLIEFWMEKRYTLRYTGGLVPDV
YQIFVKQQGVFCNPASKAAPAKLRMCFEVLAIALVVEAAGGRTSNGQKSLLDVAIEHMDH
RSALCCGSADEIKRMEETFAALSG

11111000000000000000000000000000000000000000000000000000000000000000000000000000000
00000000000000000000000000000000000000000000000000000000000000000000000000000000000
00000000000000000000000000000000000000000000000000000000000000000000000000000000000
000000000000000000000000000000000000000000000000000000000000000000000000000000000
>P25548_3urm_24_353
QDKGSVGIAMPTKSSARWIDDGNNIVKQLQEAGYKTDLQYADDDIPNQLSQIENMVTKG
VKVLVIASIDGTTLSDVLKQAGEQGIKVIAYDRLIRNSGDVSYYATFDNFQVGVLQATSITDK
LGLKDGKGPFNIELFGGSPDDNNAFFFYDGAMSVLKPYIDSGKLVVKSGQMGMDKVGTLR
WDPATAQARMDNLLSAYYTDAKVDAVLSPYDGLSIGIISSLKGVGYGTKDQPLPVVSGQDA
EVPSVKSIIAGEQYSTIFKDTRELAKVTVNMVNAVMEGKEPEVNDTKTYENGVKVVPSYLL
KPVAVTKENYKQVLVDGGYYKEDQLK
10000000000000000000000000000000000000000000000000000000000000000000000000000000000
00000000000000000000000000000000000000000000000000000000000000000000000000000000000
00000000000000000000000000000000000000000000000000000000000000000000000000000000000
000000000000000000000000000000000000000000000000000000000000000000000000000000000
>P97571_1tlo_26_355
GLGRHENAIKYLGQDYENLRARCLQNGVLFQDDAFPPVSHSLGFKELGPNSSKTYGIKWK
RPTELLSNPQFIVDGATRTDICQGALGDCWLLAAIASLTLNETILHRVVPYGQSFQEGYAGIF
HFQLWQFGEWVDVVVDDLLPTKDGKLVFVHSAQGNEFWSALLEKAYAKVNGSYEALSGG
CTSEAFEDFTGGVTEWYDLQKAPSDLYQIILKALERGSLLGCSINISDIRDLEAITFKNLVRGH
AYSVTDAKQVTYQGQRVNLIRMRNPWGEVEWKGPWSDNSYEWNKVDPYEREQLRVKME
DGEFWMSFRDFIREFTKLEICNLTPD
11111100000000000000000000000000000000000000000000000000000000000000000000000000000
00000000000000000000000000000000000000000000000000000000000000000000000000000000000
00000000000000000000000000000000000000000000000000000000000000000000000000000000000
000000000000000000000000000000000000000000000000000000000000000000000000000000011
>Q6MQC7_3caw_0_329
MIKISYSPYTLKPVQSLNAATAATAREGVLLKVEWNDGLYGFADLHPWPELGDLSLEEQLS
DLRMGRMTTQIEQSIWLARRDALLRKEKKHVFDGGEKIKNNYLLSHFQDLKPGFLDGLKN
EGYNTVKVKMGRDLQKEADMLTHIAASGMRMRLDFNALGSWQTFEKFMVNLPLTVRPLI
EYVEDPFPFDFHAWGEARKLAKIALDNQYDKVPWGKIASAPFDVIVIKPAKTDVDKAVAQ
CQKWNLKLAVTSYMDHPVGVVHAVGVAMELKDKYGDMILESGCLTHRLYQMDSFAAELS
TQGPYLLKNKGTGVGFDKLLEALTWYQLKVR
11000000000000111111111100000000000000000000000000000000000000000000000000000000000
00000000000000000000000000000000000000000000000000000000000000000000000000000000000
00000000000000000000000000000000000000000000000000000000000000000000000000000000000
000000000000000000000000000000000000000000000000000000000000000000000000000000011
>A0R2V4_2ymv_0_328
MSDTRLDVATLANAVQLAARAPSLHNTQPWRLIAEDGELKLFLDPSRVVRSTDRSSREAVM
SCGVLLDHLRVALAAAGWDTEVQRFPNPNDRDHLATLSFRPLQFVTEGHRKRADAILARR
TDRLPMSAYVDWDAFETLLRARLGDGPVHMDTLGEDVREEVAEAAALTESLRLYDAAYHS
ELAWWTTPFATEDGIPQTALISAEESERVAVSRDFPVAPHSSRRPALNNDAATIVVLSTDGYS
REDALDAGEGLSKVLLECTMSGLATCPVTHVTELHTSRDIIGRLIVRDACPQVLVRIGLAPA
LDEVPPPTPRRPVDAFLEVRPRS
10000000000000000000000000000000000000000000000000000000000000000000000000000000000
00000000000000000000000000000000000000000000000000000000000000000000000000000000000
00000000000000000000000000000000000000000000000000000000000000000000000000000000000
00000000000000000000000000000000000000000000000000000000000000000000000000000001
>A6LBC8_4h3w_23_351
YDLSDVNTDDAVMGESWVAPLGMGYVTSDDVVNVEKVPSIREVDGAYVMIYDGEMKIKG
KSLRAASDKVEIASEDITTGDIDGLFDGDFVLALTNPHITLKSNVKNASLDCSLSIEAENTSK

KEATSSDFTLSTVSPNIWIGPLDPKTDAFKFVKNEKLPGIVQIVPQKIHLSLSADSKQWTNAP
ADALSELRYAVELPLTPAPEFSAVSVERIEDAFDEDFVDYIFSDGSARIYGEVTNEMPFDMSIE
MVIMDENNVPVDIQFPAQEVKGQSGEVIFEITKEDMPKMKDARHIDLNLHLTGRDQGEAL
KKGQKTTFNLKLKKEGGISI
11111111111111000000000000000000000000000000000000000000000000000000000000000000000
00000000000000000000000000000000000000000000000000000000000000000000000000000000000
00000000000000000000000000000000000000000000000000000000000000000000000000000000000
00000000000000000000000000000000000000000000000000000000000000000000000000000011
>P11454_3tej_964_1292
AQAPGRAPKAGSETIIAAAFSSLLGCDVQDADADFFALGGHSLLAMKLAAQLSRQVARQV
TPGQVMVASTVAKLATIIDAEEDSTRRMGFETILPLREGNGPTLFCFHPASGFAWQFSVLSRY
LDPQWSIIGIQSPRPNGPMQTAANLDEVCEAHLATLLEQQPHGPYYLLGYSLGGTLAQGIA
ARLRARGEQVAFLGLLDTWPPETQNWQEKEANGLDPEVLAEINREREAFLAAQQGSTSTE
LFTTIEGNYADAVRLLTTAHSVPFDGKATLFVAERTLQEGMSPERAWSPWIAELDIYRQDCA
HVDIISPGTFEKIGPIIRATLNR
11100000000000000000000000000000000000000000000000000000000000000000000000000000000
00000000000000000000000000000000000000000000000000000000000000000000000000000000000
00000000000000000000000000000000000000000000011111000000000000000000000000000000000
00000000000000000000000000000000000000000000000000000000000000000000000000000000
>P54965_2bjf_0_328
MCTGLALETKDGLHLFGRNMDIEYSFNQSIIFIPRNFKCVNKSNKKELTTKYAVLGMGTIFD
DYPTFADGMNEKGLGCAGLNFPVYVSYSKEDIEGKTNIPVYNFLLWVLANFSSVEEVKEAL
KNANIVDIPISENIPNTTLHWMISDITGKSIVVEQTKEKLNVFDNNIGVLTNSPTFDWHVAN
LNQYVGLRYNQVPEFKLGDQSLTALGQGTGLVGLPGDFTPASRFIRVAFLRDAMIKNDKDSI
DLIEFFHILNNVAMVRGSTRTVEEKSDLTQYTSCMCLEKGIYYYNTYENNQINAIDMNKEN
LDGNEIKTYKYNKTLSINHVN
10000000000000000000000000000000000000000000000000000000000000000000000000000000000
00000000000000000000000000000000000000000000000000000000000000000000000000000000000
00000000000000000000000000000000000000000000000000000000000000000000000000000000000
00000000000000000000000000000000000000000000000000000000000000000000000000000000
>Q4Q6K7_2hfs_0_328
MSKPVKSKTTGKNIGYGKVILFGEHFVVHGAEAIVAGISEYTECRLEINPGVPGLQVDDQRP
AIPGYIAQKRDEQIKAHQLVLDHLKVDLSGDGLKMFIGGPLVPSSGIGASASDVVAFSRALS
ELYQLNLTDEEVNLSAFVGEGGYHGTPSGADNTAATYGGLILYRRQNGKSVFKPIAFQQRL
YLVVVGTGINASTAKVVNDVHKMKQQQPVQFKRLYDNYTHIVSQAREALQKGDLQRLGQ
LMNANHDLCRQIDVSCRELESIVQTCRTYGALGAKLSGTGRGGIAVALAASSDQRDAIVKG
LKAKCPEAKFIWRYTVQPSAASNL
00000000000000000000000000000000000000000000000000000000000000000000000000000000000
00000000000000000000000000000000000000000000000000000000000000000000000000000000000
00000000000000000000000000000000000000000000000000000000000000000000000000000000000
00000000000000000000000000000000000000000000000000000000000000000000000000000111
>Q9L5X8_3e3x_280_608
TGLGELKISDTICAQNAVEALPALSVDEPTVTMTFQVNTSPFAGKEGKFVTSRNILERLEKEL
VHNVALRVEQTDDPDKFRVSGRGELHLSILIENMRREGFELAVSRPEVIIKEEDGQLMEPFET
VTIDVMEEHQGGIMENIGLRKGELKDMAPDGKGRVRMDFIMPSRGLIGFQTEFMTLTSGSG
LLYHTFDHYGPHKGGNIGQRVNGVLIANAAGKALTNALFNLQERGRLFIGHGVEVYEGM
VIGIHSRDNDLTVNALKGKQLTNVRASGTDDAQVLTPPIVMTLEQALEFIDDDELVEVTPES
IRIRKKFLTESDRKRASRSAK
11111111111111111111111110000000000000000000000000000000000000000000000000000000000
00000000000000000000000000000000000000000000000000000000000000000000000000000000000
00000000000000000000000000000000000000000000000000000000000000000000000000000000000

00000000000000111111111111110000000000000000000000000000000000000000000000001111
>O32157_3eua_0_327
MSQATAKVNREVQAFLQDLKGKTIDHVFFVACGGSSAIMYPSKYVFDRESKSINSDLYSANE
FIQRNPVQLGEKSLVILCSHSGNTPETVKAAAFARGKGALTIAMTFKPESPLAQEAQYVAQY
DWGDEALAINTNYGVLYQIVFGTLQVLENNTKFEQAIEGLDQLQAVYEKALKQEADNAK
QFAKAHEKESIIYTMASGANYGVAYSYSICILMEMQWIHSHAIHAGEYFHGPFEIIDESVPFII
LLGLDETRPLEERALTFSKKYGKKLTVLDAASYDFTAIDDSVKGYLAPLVLNRVLRSYADEL
AEERNHPLSHRRYMWKVEY
11100000000000000000000000000000000000000000000000000000000000000000000000000000000
00000000000000000000000000000000000000000000000000000000000000000000000000000000000
00000000000000000000000000000000000000000000000000000000000000000000000000000000000
0000000000000000000000000000000000000000000000000000000000000000000000000000000
>O83923_3u65_14_341
CFGLYAKEKVVLKIASIAPARSIWETELKKLSAEWSEITGGLVSMKFYDMSSLGGEREGIRKL
KSSRPGQAAPLDGAVFSCLGLSELAPDSGIYTLSVPFLIQNEKDLERVLHELREDLDRPFRAA
GFRVITWTNAGWLSFYTRAPYASLGQLKKQTIALSSLDSSVLGTCFRICGFDIKDAPNARLA
PLLKAGSIDGFLSVHLFTWATGFYRYISYALDTKICPAVIGMLISDGSWARIPSRYHDAMLQA
ATRVRQRLANNLETLDRECSNNIQKAGVSIVHLTPQEIQEWRTEFAADVKRIQARLPGMLN
MTLYEKIKHLLYSAQR
11111111000000000000000000000000000000000000000000000000000000000000000000000000000
00000000000000000000000000000000000000000000000000000000000000000000000000000000000
00000000000000000000000000000000000000000000000000000000000000000000000000000000000
0000000000000000000000000000000000000000000000000000000000000000000000000000111
>P11353_1txn_0_327
MPAPQDPRNLPIRQQMEALIRRKQAEITQGLESIDTVKFHADTWTRGNDGGGGTSMVIQD
GTTFEKGGVNVSVVYGQLSPAAVSAMKADHKNLRLPEDPKTGLPVTDGVKFFACGLSMVI
HPVNPHAPTTHLNYRYFETWNQDGTPQTWWFGGGADLTPSYLYEEDGQLFHQLHKDAL
DKHDTALYPRFKKWCDEYFYITHRKETRGIGGIFFDDYDERDPQEILKMVEDCFDAFLPSYL
TIVKRRKDMPYTKEEQQWQAIRRGRYVEFNLIYDRGTQFGLRTPGSRVESILMSLPEHASWL
YNHHPAPGSREAKLLEVTTKPREWVK
10000000000000000000000000000000000000000000000000000000000000000000000000000000000
00000000000001111111110000000000000000000000000000000000000000000000000000000000000
00000000000000000000000000000000000000000000000000000000000000000000000000000000000
0000000000000000000001111111111111111111111111111111111111111111111111111111111
>P20132_4h27_0_327
MMSGEPLHVKTPIRDSMALSKMAGTSVYLKMDSAQPSGSFKIRGIGHFCKRWAKQGCAHF
VCSSAGNAGMAAAYAARQLGVPATIVVPSTTPALTIERLKNEGATVKVVGELLDEAFELAK
ALAKNNPGWVYIPPFDDPLIWEGHASIVKELKETLWEKPGAIALSVGGGGLLCGVVQGLQ
EVGWGDVPVIAMETFGAHSFHAATTAGKLVSLPKITSVAKALGVKTVGAQALKLFQEHPIF
SEVISDQEAVAAIEKFVDDEKILVEPACGAALAAVYSHVIQKLQLEGNLRTPLPSLVVIVCGG
SNISLAQLRALKEQLGMTNRLPK
11110000000000000000000000000000000000000000000000000000000000000000000000000000000
00000000000000000000000000000000000000000000000000000000000000000000000000000000000
00000000000000000000000000000000000000000000000000000000000000000000000000000000000
0000000000000000000000000000000000000000000000000000000000000000000000000111111
>Q04081_1rjd_0_327
MERIIQQTDYDALSCKLAAISVGYLPSSGLQRLSVDLSKKYTEWHRSYLITLKKFSRRAFGKV
DKAMRSSFPVMNYGTYLRTVGIDAAILEFLVANEKVQVVNLGCGSDLRMLPLLQMFPHLA
YVDIDYNESVELKNSILRESEILRISLGLSKEDTAKSPFLIDQGRYKLAACDLNDITETTRLLDV
CTKREIPTIVISECLLCYMHNNESQLLINTIMSKFSHGLWISYDPIGGSQPNDRFGAIMQSNL
KESRNLEMPTLMTYNSKEKYASRWSAAPNVIVNDMWEIFNAQIPESERKRLRSLQFLDELE

ELKVMQTHYILMKAQW
10000000000000000000000000000000000000000000000000000000000000000000000000000000000
00000000000000000000000000000000000000000000000000000000000000000000000000000000000
00000000000000000000000000000000000000000000000000000000000000000000000000000000000
0000000000000000000000000000000000000000000000000000000000000000000000000000000
>Q0S9X1_3lm4_2_329
DARFDIAHLARAELFSPKPQETLDFFTKFLGMYVTHREGQSVYLRGYEDPYPWSLKITEAPE
AGMGHAAMRTSSPEALERRAKSLTDGNVDGTWSEDQFGYGKTFEYQSPDGHNLQLLWE
AEKYVAPPELRSKILTRPSKKPLQGIPVKRIDHLNLMSSDVTAVKDSFERHLGFRTTERVVDG
NVEIGAWMSSNLLGHEVACMRDMTGGHGKLHHLAFFYGTGQHNIDAVEMFRDYDIQIEA
GPDKHGITQSQFLYVFEPGGNRIELFGEAGYLHLDPDAETKTWQMSDIDTGLAVGGAKLP
WESYFTYGTPSPLSLDQHIEKYAHFG
00000000000000000000000000000000000000000000000000000000000000000000000000000000000
00000000000000000000000000000000000000000000000000000000000000000000000000000000000
00000000000000000000000000000000000000000000000000000000000000000000000000000000000
0000000000000000000000000000000000000000000000000000000000000000000000000000011
>Q6GJ80_4e4r_0_327
MADLLNVLKDKLSGKNVKIVLPEGEDERVLTAATQLQATDYVTPIVLGDETKVQSLAQKL
NLDISNIELINPATSELKAELVQSFVERRKGKTTEEQAQELLNNVNYFGTMLVYAGKADGLV
SGAAHSTGDTVRPALQIIKTKPGVSRTSGIFFMIKGDEQYIFGDCAINPELDSQGLAEIAVESA
KSALSFGMDPKVAMLSFSTKGSAKSDDVTKVQEAVKLAQQKAEEEKLEAIIDGEFQFDAAI
VPGVAEKKAPGAKLQGDANVFVFPSLEAGNIGYKIAQRLGGYDAVGPVLQGLNSPVNDLS
RGCSIEDVYNLSFITAAQALQ
00000000000000000000000000000000000000000000000000000000000000000000000000000000000
00000000000000000000000000000000000000000000000000000000000000000000000000000000000
00000000000000000000000000000000000000000000000000000000000000000000000000000000000
0000000000000000000000000000000000000000000000000000000000000000000000000000001
>Q9HY91_3bjd_0_327
MNTRNFSLPQLQNLPIEEARIVADALAVHATSRQIDSAASKLAALAEAGLKGDRQAYAAYQ
QLLYVLSLSDDVATAQTRRWLARAIYRVEERFMPAADLSRALSEEDFQKRLEQEIAAQSRER
HPMSQYVFSGSASRAQLQVFLRHQWFRTFRLYRDAADLLVNLTDVDEAAALARYLYGELG
EEDEKGSHPRLLAKLLEAIGLEADFQAVSTMPEEIAYLNNRARAFRHAEVGWGLAVFYITE
LVVPGNHEKLYRALLQAGLSEDQAEYYKVHISLVPPRAKREWQLIARRIPDVQFQNAFLTSL
SQHFRVERAYYDAIWEEMQSVK
10000000000000000000000000000000000000000000000000000000000000000000000000000000000
00000000000000000000000000000000000000000000000000000000000000000000000000000000000
00000000000000000000000000000000000000000000000000000000000000000000000000000000011
0000000000000000000000000000000000000000000000000000000000000000000000000000001
>A9CIT6_4gfi_0_326
MPRYLQATTERFAVAGSFTISRGTRTHADVVTCTIRDGSFTGIGECVPYPRYGESIEGVTADIE
AMADRVAAGLTRQELQQVMKPGAARNAVDCALWDLEAKMSGKRAAEQVLGQPAQPLV
TAYTISLADPDTMAAKTAENAGRPLLKIKTGTADDEARLRAVRAAAPEARIIIDANEGWND
DNIEYYLKLAAELKISLIEQPLPAGKDAMLARIEHPVLICADESVHSTEDLAGLRDRYDAINI
KLDKTGGLTEALVMKAEAERLGFTIMVGCMLGTSLGMAPAVLVAQGTAFADLDGPLLLAE
DRDPGLVYEGSLVYPARPELWG
10000000000000000000000000000000000000000000000000000000000000000000000000000000000
00000000000000000000000000000000000000000000000000000000000000000000000000000000000
00000000000000000000000000000000000000000000000000000000000000000000000000000000000
000000000000000000000000000000000000000000000000000000000000000000000000000000
>D5MP61_3vpl_22_348
LDGVLVPESGILVSVGQDVDSVNDYASALGTIPAGVTNYVGIVNLDGLNSDADAGAGRNN

IAELANAYPTSALVVGVSMNGEVDAVASGRYNANIDTLLNTLAGYDRPVYLRWAYEVDGP
WNGHSPSGIVTSFQYVHDRIIALGHQAKISLVWQVASYCPTPGGQLDQWWPGSEYVDWV
GLSYFAPQDCNWDRVNEAAQFARSKGKPLFLNESTPQRYQVADLTYSADPAKGTNRQSKT
SQQLWDEWFAPYFQFMSDNSDIVKGFTYINADWDSQWRWAAPYNEGYWGDSRVQANAL
IKSNWQQEIAKGQYINHSETLFETLGYGST
0000000000000000000000000000000000000000000000000000000000000000000000000000000000000000000000000000
0000000000000000000000000000000000000000000000000000000000000000000000000000000000000000000000000000
0000000000000000000000000000000000000000000000000000000000000000000000000000000000000000000000000000
0000000000000000000000000000000000000000000000000000000000000000000000000000000000000000000111
>P27448_3fe3_40_366
NSIASCADEQPHIGNYRLLKTIGKGNFAKVKLARHILTGREVAIKIIDKTQLNPTSLQKLFRE
VRIMKILNHPNIVKLFEVIETEKTLYLIMEYASGGEVFDYLVAHGRMKEKEARSKFRQIVSAV
QYCHQKRIVHRDLKAENLLLDADMNIKIADFGFSNEFTVGGKLDTFCGSPPYAAPELFQGK
KYDGPEVDVWSLGVILYTLVSGSLPFDGQNLKELRERVLRGKYRIPFYMSTDCENLLKRFLV
LNPIKRGTLEQIMKDRWINAGHEEDELKPFVEPELDISDQKRIDIMVGMGYSQEEIQESLSK
MKYDEITATYLLLGRK
1111111110000000000000000000000000000000000000000000000000000000000000000000000000000000000000000000
0000000000000000000000000000000000000000000000000000000000000000000000000000000000000000000000000000
0000000000000000000000000000000000000000000000000000000000000000000000000000000000000000000000000000
0000000000000000000000000000000000000000000000000000000000000000000000000000000000000000000001
>P38918_1gve_0_326
MSQARPATVLGAMEMGRRMDVTSSSASVRAFLQRGHTEIDTAFVYANGQSETILGDLGLG
LGRSGCKVKIATKAAPMFGKTLKPADVRFQLETSLKRLQCPRVDLFYLHFPDHGTPIEETLQ
ACHQLHQEGKFVELGLSNYVSWEVAEICTLCKKNGWIMPTVYQGMYNAITRQVETELFPC
LRHFGLRFYAFNPLAGGLLTGRYKYQDKDGKNPESRFFGNPFSQLYMDRYWKEEHFNGIA
LVEKALKTTYGPTAPSMISAAVRWMYHHSQLKGTQGDAVILGMSSLEQLEQNLALVEEGPL
EPAVVDAFDQAWNLVAHECPNYFR
1110000000000000000000000000000000000000000000000000000000000000000000000000000000000000000000000000
0000000000000000000000000000000000000000000000000000000000000000000000000000000000000000000000000000
0000000000000000000000000000000000000000000000000000000000000000000000000000000000000000000000000000
0000000000000000000000000000000000000000000000000000000000000000000000000000000000000000000000
>Q00460_3ge3_0_326
MSFESKKPMRTWSHLAEMRKKPSEYDIVSRKLHYSTNNPDSPWELSPDSPMNLWYKQYRN
ASPLKHDNWDAFTDPDQLVYRTYNLMQDGQESYVQSLFDQFNEREHDQMVREGWEHT
MARCYSPLRYLFHCLQMSSAYVQQMAPASTISNCCILQTADSLRWLTHTAYRTHELSLTYPD
AGLGEHERELWEKEPGWQGLRELMEKQLTAFDWGEAFVSLNLVVKPMIVESIFKPLQQQA
WENNDTLLPLLIDSQLKDAERHSRWSKALVKHALENPDNHAVIEGWIEKWRPLADRAAE
AYLSMLSSDILHAQYLERSTSLRASILTV
1100000000000000000000000000000000000000000000000000000000000000000000000000000000000000000000000000
0000000000000000000000000000000000000000000000000000000000000000000000000000000000000000000000000000
0000000000000000000000000000000000000000000000000000000000000000000000000000000000000000000000000000
0000000000000000000000000000000000000000000000000000000000000000000000001111111111111111111111
>Q5CZ29_3hko_125_451
GSLLELQKKYHLKGAIGQGSYGVVRVAIENQTRAIRAIKIMNKNKIRQINPKDVERIKTEVR
LMKKLHHPNIARLYEVYEDEQYICLVMELCHGGHLLDKLNVFIDDSTGKCAMDVVKTQIC
PCPECNEEAINGSIHGFRESLDFVQREKLISNIMRQIFSALHYLHNQGICHRDIKPENFLFST
NKSFEIKLVDFGLSKEFYKLNNGEYYGMTTKAGTPYFVAPEVLNTTNESYGPKCDAWSAGV
LLHLLLMGAVPFPGVNDADTISQVLNKKLCFENPNYNVLSPLARDLLSNLLNRNVDERFD
AMRALQHPWISQFSDKIYKMS
0000000000000000000000000000000000000000000000000000000000000111000000000000000000000000000000000000
0000000000000000000000000000000000000000000000000000000000000000001100000000000000000000000000000000

00000000000000000000000000000000000000001111111000000000000000000000000000000000
000000000000000000000000000000000000000000000000000000000000000000000000000
>Q97IW1_3k1u_0_326
MENEKILNPIIIQRADPMIYKHNDGYYYFTASVPEYDRIEVRKAKTIEGLRNAEPVDVWRRH
ESGEMSNLIWAPEIHFINGAWYIYFAAAPDKNIEDDTFNHRMFVIQNENENPFTGNWVEK
GRIKTAWESFSLDATIFEHNEKLYYVWAQQDINIKGHSNIYIAEMENPWTLKTKPVMLTKPE
LEWEIKGFWVNEGPAVLKKNGKIFITYSASATDVNYCIGMLTAEENSNLLDKNSWTKSQTP
VFKTSMENHQYGPGHNSFTVSEDGKHDVIVYHARNYTEIKGDPLYDPNRHTRAQIINWRE
DGTPDFGVPEVDSLEIETPVKA
11111000000000000000000000000000000000000000000000000000000000000000000000000000
00000000000000000000000000000000000000000000000000000000000000000000000000000000
00000000000000000000000000000000000000000000000000000000000000000000000000000000
000000000000000000000000000000000000000000000000000000000000000000011111111
>B0LPL6_3tcq_0_325
MRRVTVPTAPPAYADIGYPMSMLPIKSSRAVSGIQQKQEVLPGMDTPSNSMRPVADDNIDH
TSHTPNGVASAFILEATVNVISGPKVLMKQIPIWLPLGIADQKTYSFDSTTAAIMLASYTITHF
GKANNPLVRVNRLGQGIPDHPLRLLRMGNQAFLQEFVLPPVQLPQYFTFDLTALKLVTQPL
PAATWTDETPSNLSGALRPGLSFHPKLRPVLLPGKTGKKGHVSDLTAPDKIQTIVNLMQDF
KIVPIDPAKSIIGIEVPELLVHKLTGKKMSQKNGQPIIPVLLPKYIGLDPISPGDLTMVITPDYD
DCHSPASCSYLSEK
11111111111111111111111111111111111111110000000000000000000000000000000000000000
00000000000000000000000000000000000000000000000000000000000000000000000000000000
00000000000000000000000000011111111110000000000000011111111111000000000000000000
00000000000000000000000000000000000000000011111000000000111111111111111111
>P0ADE8_1vly_0_325
MAFTPFPPRQPTASARLPLTLMTLDDWALATITGADSEKYMQGQVTADVSQMAEDQHLL
AAHCDAKGKMWSNLRLFRDGDGFAWIERRSVREPQLTELKKYAVFSKVTIAPDDERVLLG
VAGFQARAALANLFSELPSKEKQVVKEGATTLLWFEHPAERFLIVTDEATANMLTDKLRGE
AELNNSQQWLALNIEAGFPVIDAANSGQFIPQATNLQALGGISFKKGCYTGQEMVARAKF
RGANKRALWLLAGSASRLPEAGEDLELKMGENWRRTGTVLAAVKLEDGQVVVQVVMN
NDMEPDSIFRVRDDANTLHIEPLPYSLEE
11000000000000000000000000000000000000000000000000000000000000000000000000000000
00000000000000000000000000000000000000000000000000000000000000000000000000000000
00000000000000000000000000000000000000000000000000000000000000000000000000000000
00000000000000000000000000000000000000000000000000000000000000000000000001
>P29068_3v7z_98_423
DFPSYDSGYHNYNEMVNKINTVASNYPNIVKKFSIGKSYEGRELWAVKISDNVGTDENEPE
VLYTALHHAREHLTVEMALYTLDLFTQNYNLDSRITNLVNNREIYIVFNINPDGGEYDISSGS
YKSWRKNRQPNSGSSYVGTDLNRNYGYKWGCCGGSSGSPSSETYRGRSAFSAPETAAMRD
FINSRVVGGKQQIKTLITFHTYSELILYPYGYTYTDVPSDMTQDDFNVFKTMANTMAQTNG
YTPQQASDLYITDGDMTDWAYGQHKIFAFTFEMYPTSYNPGFYPPDEVIGRETSRNKEAVLY
VAEKADCPYSVIGKSCSTK
00000000000000000000000000000000000000000000000000000000000000000000000000000000
00000000000000000000000000000000000000000000000000000000000000000000000000000000
00000000000000000000000000000000000000000000000000000000000000000000000000000000
00000000000000000000000000000000000000000000000000000000000000000000000111
>P32318_3fpz_0_325
MSATSTATSTSASQLHLNSTPVTHCLSDIVKKEDWSDFKFAPIRESTVSRAMTSRYFKDLDKF
AVSDVIIVGAGSSGLSAAYVIAKNRPDLKVCIIESSVAPGGGSWLGGQLFSAMVMRKPAHLF
LQELEIPYEDEGDYVVVKHAALFISTVLSKVLQLPNVKLFNATCVEDLVTRPPTEKGEVTVA
GVVTNWTLVTQAHGTQCCMDPNVIELAGYKNDGTRDLSQKHGVILSTTGHDGPFGAFCA

KRIVDIDQNQKLGGMKGLDMNHAEHDVVIHSGAYAGVDNMYFAGMEVAELDGLNRMG
PTFGAMALSGVHAAEQILKHFAA
11111111111111100000000000000000000000000000000000000000000000000000000000000000000
00000000000000000000000000000000000000000000000000000000000000000000000000000000000
00000000000000000000000000000000000000000000000000000000000000000000000000000000000
00000000000000000000000000000000000000000000000000000000000000000000000000000
>P52839_1q44_0_325
MSSSSSVPAYLGDEDLTQETRALISSLPKEKGWLVSEIYEFQGLWHTQAILQGILICQKRFEAK
DSDIILVTNPKSGTTWLKALVFALLNRHKFPVSSSGNHPLLVTNPHLLVPFLEGVYYESPDFD
FSSLPSPRLMNTHISHLSLPESVKSSSCKIVYCCRNPKDMFVSLWHFGKKLAPEETADYPIEK
AVEAFCEGKFIGGPFWDHILEYWYASRENPNKVLFVTYEELKKQTEVEMKRIAEFLECGFIE
EEVREIVKLCSFESLSNLEVNKEGKLPNGIETKTFFRKGEIGGWRDTLSESLAEEIDRTIEEKF
KGSGLKFSS
11111000000000000000000000000000000000000000000000000000000000000000000000000000000
00000000000000000000000000000000000000000000000000000000000000000000000000000000000
00000000000011111100000000000000000000000000000000000000000000000000000000000000000
0000000000000000011111111111111111111111111111000000000000000000000000000001
>C3SGA2_3a5y_10_334
MSETASWQPSASIPNLLKRAAIMAEIRRFFADRGVLEVETPCMSQATVTDIHLVPFETRFVGP
GHSQGMNLWLMTSPEYHMKRLLVAGCGPVFQLCRSFRNEEMGRYHNPEFTMLEWYRPH
YDMYRLMNEVDDLLQQVLDCPAAESLSYQQAFLRYLEIDPLSADKTQLREVAAKLDLSNV
ADTEEDRDTLLQLLFTFGVEPNIGKEKPTFVYHFPASQASLAQISTEDHRVAERFEVYYKGIE
LANGFHELTDAREQQQRFEQDNRKRAARGLPQHPIDQNLIEALKVGMPDCSGVALGVDR
LVMLALGAETLAEVIAFSVDRA
11100000000000000000000000000000000000000000000000000000000000000000000000000000000
00000000000000000000000000000000000000000000000000000000000000000000000000000000000
00000000000001111110000000000000000000000000000000000000000000000000000000000000000
0000000000000000000000000000000000000000000000000000000000000000000000000000
>P32639_3im2_1838_2162
EATEIISTLSNGLIASHYGVSFFTIQSFVSSLSNTSTLKNMLYVLSTAVEFESVPLRKGDRALLV
KLSKRLPLRFPEHTSSGSVSFKVFLLLQAYFSRLELPVDFQNDLKDILEKVVPLINVVVDILSA
NGYLNATTAMDLAQMLIQGVWDVDNPLRQIPHFNNKILEKCKEINVETVYDIMALEDEER
DEILTLTDSQLAQVAAFVNNYPNVELTYSLNNSDSLISGVKQKITIQLTRDVEPENLQVTSEK
YPFDKLESWWLVLGEVSKKELYAIKKVTLNKETQQYELEFDTPTSGKHNLTIWCVCDSYLD
ADKELSFEINVK
11111000000000000000000000000000000000000000000000000000000000000000000000000000000
00000000000000000000000000000000000000000000000000000000000000000000000000000000000
00000000000000000000000000000000000000000000000000000000000000000000000000000000000
0000000000000000000000000000000000000000000000000000000000000000000000000000
>Q7A8N3_3pt5_1_325
NAIISPDYYYVLTVAGQSNAMAYGEGLPLPDREDAPHPRIKQLARFAHTHPGGPSCHFNDII
PLTHCPHDVQDMQSYHHPLATNHQTQYGTVGQALHIARKLLPFIPDNAGILIVPCCRGGS
AFTAGSEGTYSERHGASHDACRWGTDTPLYQDLVSRTRAALVKNPQNKFLGVCWMQGEF
DLMTSDYASHPQHFNHMVEAFRRDLKQYHSQLNNITDAPWFCGDTTWYWKENFPHAYE
AIYGNYQNNILANIIFVDFQQQGARGLTNAPDEDPDDLSTGYYGSAYRSPENWTTALRSSH
FSSAARRGIISDRFVEAILQFWRER
11000000000000000000000000000000000000000000000000000000000000000000000000000000000
00000000000000000000000000000000000000000000000000000000000000000000000000000000000
00000000000000000000000000000000000000000000000011100000000000000000000000000000000
0000000000000000000000000000000000000000000000000000000000000000000000000000
>Q96WV5_4j8b_2_326

MLTKFESRSSRAKGVAFHPTQPWILTSLHNGRIQLWDYRMGTLLDRFDGHDGPVRGIAFHP
TQPLFVSGGDDYKVNVWNYKSRKLLFSLCGHMDYVRVCTFHHEYPWILSCSDDQTIRIWN
WQSRNCIAILTGHSHYVMCAAFHPSEDLIVSASLDQTVRVWDISGLRMKNAAPVSMSLED
QLAQAHNSISNDLFGSTDAIVKFVLEGHDRGVNWCAFHPTLPLILSAGDDRLVKLWRMTA
SKAWEVDTCRGHFNNVSCCLFHPHQELILSASEDKTIRVWDLNRRTAVQTFRRDNDRFWFI
TVHPKLNLFAAAHDSGVMVFKLE
000000000000000000000000000000000000000000000000000000000000000000000000000000000000000000000
000000000000000000000000000000000000000000000000000000000000000000000000000000000000000000000
001111111111111111111111111111100000000000000000000000000000000000000000000000000000000000000
0000000000000000000000000000000000000000000000000000000000000000000000000000000000000
>O33820_1rm6_0_323
MNILTDFRTHRPATLADAVNALAAEATLPLGAGTDLLPNLRRGLGHPAALVDLTGIDGLAT
ISTLADGSLRIGAGATLEAIAEHDAIRTTWPALAQAAESVAGPTHRAAATLGGNLCQDTRC
TFYNQSEWWRSGNGYCLKYKGDKCHVIVKSDRCYATYHGDVAPALMVLDARAEIVGPAG
KRTVPVAQLFRESGAEHLTLEKGELLAAIEVPPTGAWSAAYSKVRIRDAVDFPLAGVAAAL
QRDGDRIAGLRVAITGSNSAPLMVPVDALLGGNWDDAAAETLAQLVRKTSNVLRTTITGV
KYRRRVLLAISRKVVDQLWEAR
000000000000000000000000000000000000000000000000000000000000000000000000000000000000000000000
000000000000000000000000000000000000000000000000000000000000000000000000000000000000000000000
000000000000000000000000000000000000000000000000000000000000000000000000000000000000000000000
000000000000000000000000000000000000000000000000000000000000000000000000000000000001
>P06010_2wjn_0_323
MADYQTIYTQIQARGPHITVSGEWGDNDRVGKPFYSYWLGKIGDAQIGPIYLGASGIAAFA
FGSTAILIILFNMAAEVHFDPLQFFRQFFWLGLYPPKAQYGMGIPPLHDGGWWLMAGLFM
TLSLGSWWIRVYSRARALGLGTHIAWNFAAAIFFVLCIGCIHPTLVGSWSEGVPFGIWPHID
WLTAFSIRYGNFYYCPWHGFSIGFAYGCGLLFAAHGATILAVARFGGDREIEQITDRGTAVER
AALFWRWTIGFNATIESVHRWGWFFSLMVMVSASVGILLTGTFVDNWYLWCVKHGAAPD
YPAYLPATPDPASLPGAPK
100000000000000000000000000000000000000000000000000000000000000000000000000000000000000000000
000000000000000000000000000000000000000000000000000000000000000000000000000000000000000000000
000000000000000000000000000000000000000000000000000000000000000000000000000000000000000000000
000000000000000000000000000000000000000000000000000000000000000000000000000000000000
>P07739_1luc_0_323
MKFGLFFLNFMNSKRSSDQVIEEMLDTAHYVDQLKFDTLAVYENHFSNNGVVGAPLTVAG
FLLGMTKNAKVASLNHVITTHHPVRVAEEACLLDQMSEGRFAFGFSDCEKSADMRFFNRP
TDSQFQLFSECHKIINDAFTTGYCHPNNDFYSFPKISVNPHAFTEGGPAQFVNATSKEVVEW
AAKLGLPLVFRWDDSNAQRKEYAGLYHEVAQAHGVDVSQVRHKLTLLVNQNVDGEAAR
AEARVYLEEFVRESYSNTDFEQKMGELLSENAIGTYEESTQAARVAIECCGAADLLMSFESM
EDKAQQRAVIDVVNANIVKYHS
000000000000000000000000000000000000000000000000000000000000000000000000000000000000000000000
000000000000000000000000000000000000000000000000000000000000000000000000000000000000000000000
000000000000000000000000000000000000000000000000000000000000000000000000000000000000000000000
000000000000000000000000000000000000000000000000000000000000000000000000000000001111
>P33643_2ist_1_324
AQRVQLTATVSENQLGQRLDQALAEMFPDYSRSRIKEWILDQRVLVNGKVCDKPKEKVLG
GEQVAINAEIEEEARFEPQDIPLDIVYEDEDIIIINKPRDLVVHPGAGNPDGTVLNALLHYYP
PIADVPRAGIVHRLDKDTTGLMVVAKTVPAQTRLVESLQRREITREYEAVAIGHMTAGGTV
DEPISRHPTKRTHMAVHPMGKPAVTHYRIMEHFRVHTRLRLRLETGRTHQIRVHMAHITH
PLVGDPVYGGRPRPPKGASEAFISTLRKFDRQALHATMLRLYHPISGIEMEWHAPIPQDMV
ELIEVMRADFEEHKDEVDW
000000000000000000000000000000000000000000000000000000000000000000000000000000000011100000000

00000000000000000000000000000000000000000000000000000000000000000000000000000000
00000000000000000000000000000000000000000000000000000000000000000000000000000000
0000000000000000000000000000000000000000000000000000000000000000000000000
>P45600_1al3_0_323
MKLQQLRYIVEVVNHNLNVSSTAEGLYTSQPGISKQVRMLEDELGIQIFARSGKHLTQVTPA
GQEIIRIAREVLSKVDAIKSVAGEHTWPDKGSLYVATTHTQARYALPGVIKGFIERYPRVSLH
MHQGSPTQIAEAVSKGNADFAIATEALHLYDDLVMLPCYHWNRSIVVTPEHPLATKGSVSI
EELAQYPLVTYTFGFTGRSELDTAFNRAGLTPRIVFTATDADVIKTYVRLGLGVGVIASMAV
DPVSDPDLVKLDANGIFSHSTTKIGFRRSTFLRSYMYDFIQRFAPHLTRDVVDTAVALRSNED
IEAMFKDIKLPEK
11111111111111111111111111111111111111111111111111111111111111111111111111111111
11110000000000000000000000000000000000000000000000000000000000000000000000000000
00000000000000000000000000000000000000000000000000000000000000000000000000000000
0000000000000000000000000000000000000000000000000000000000000000000000000
>Q05581_1ds1_0_323
MTSVDCTAYGPELRALAARLPRTPRADLYAFLDAAHTAAASLPGALATALDTFNAEGSED
GHLLLRGLPVEADADLPTTPSSTPAPEDRSLLTMEAMLGLVGRRLGLHTGYRELRSGTVYH
DVYPSPGAHHLSSETSETLLEFHTEMAYHRLQPNYVMLACSRADHERTAATLVASVRKALP
LLDERTRARLLDRRMPCCVDVAFRGGVDDPGAIAQVKPLYGDADDPFLGYDRELLAPEDP
ADKEAVAALSKALDEVTEAVYLEPGDLLIVDNFRTTHARTPFSPRWDGKDRWLHRVYIRTD
RNGQLSGGERAGDVVAFTPRG
10000000000000000000000000000000000000000000000000000000000000000000000000000000
00000000000000000000000000000000000000000000000000000000000000000000000000000000
00000000000000000000000000000000000000000000000000000000000000000000000000000000
0000000000000000000000000000000000000000000000000000000000000000000000000
>Q9AQ17_2hhc_0_323
MTKERFVISRRRTGFGDCLWSLASAWSYAQRTGRTLVIDWRGSCYVEQPFSNAFPAFFEPVE
DIAGVPVICDDRVNQLSFPGPFFPRWWNRPSIDCINRPDEQIFRERDELTELFQAREDSEANT
IVCDACLMWRCSEEAERLIFRNIKLRSEIRARIDALYEEHFSGHSIIGVHVRHGNGEDIMEHA
PYWADSELALHQVCMAIRKAKALSYPKPVKVFLCTDSAQVLDQVSGLFPDVFAVPKRFQA
DRAGPLHSAEMGIEGGASALIDMYLLARCATVIRFPPTSAFTRYARLLVPRIIEFDLSNPGHL
TMIDNPYEHFAAS
10000000000000000000000000000000000000000000000000000000000000000000000000000000
00000000000000000000000000000000000000000000000000000000000000000000000000000000
00000000000011111111111100000000000000000000000000000000000000000000000000011111
1111110000000000000000000000000000000000000000000001100000000000111111 1
>O29482_2cyb_0_322
MDITEKLRLITRNAEEVVTEEELRQLIETKEKPRAYVGYEPSGEIHLGHMMTVQKLMDLQE
AGFEIIVLLADIHAYLNEKGTFEEIAEVADYNKKVFIALGLDESRAKFVLGSEYQLSRDYVLD
VLKMARITTLNRARRSMDEVSRRKEDPMVSQMIYPLMQALDIAHLGVDLAVGGIDQRKIH
MLARENLPRLGYSSPVCLHTPILVGLDGQKMSSSKGNYISVRDPPEEVERKIRKAYCPAGVV
EENPILDIAKYHILPRFGKIVVERDAKFGGDVEYASFEELAEDFKSGQLHPLDLKIAVAKYLN
MLLEDARKRLGVSV
00000000000000000000000000000000000000000000000000000000000000000000000000000000
00000000000000000000000000000000000000000000000000000000000000000000000000000000
00000000000000000000000000000000000000000000000000000000000000000000000000000000
000000000000000000000000000000000000000000000000000000000000000000000111
>P0ABS5_1dd9_110_432
HQRQTLYQLMDGLNTFYQQSLQQPVATSARQYLEKRGLSHEVIARFAIGFAPPGWDNVLK
RFGGNPENRQSLIDAGMLVTNDQGRSYDRFRERVMFPIRDKRGRVIGFGGRVLGNDTPKYL
NSPETDIFHKGRQLYGLYEAQQDNAEPNRLLVVEGYMDVVALAQYGINYAVASLGTSTTAD

HIQLLFRATNNVICCYDGDRAGRDAAWRALETALPYMTDGRQLRFMFLPDGEDPDTLVRK
EGKEAFEARMEQAMPLSAFLFNSLMPQVDLSTPDGRARLSTLALPLISQVPGETLRIYLRQE
LGNKLGILDDSQLERLMPK
11110000000000000000000000000000000000000000000000000000000000000000000000000000011
10000000000000000000000000000000000000000000000000000000000000000000000000000000000
00000000001000000000000000000000000000000000000000000000000000000000000000000000000
00000000000000000000000000000000000000000000000000000000000000000000011111
>P70985_1dc1_0_322
MNTQKPFENHLKSVDDLKTTYEEYRAGFIAFALEKNKRSTPYIERARALKVAASVAKTPKD
LLYLEDIQDALLYASGISDKAKKFLTEDDKKESINNLIENFLEPAGEEFIDELIFRYLLFQGDSL
GGTMRNIAGALAQQKLTRAIISALDIANIPYKWLDSRDKKYTNWMDKPEDDYELETFAKGI
SWTINGKHRTLMYNITVPLVKKNVDICLFNCEPEIYTPQKVHQQPEKYLLLGELKGGIDPA
GADEHWKTANTALTRIRNKFSEKGLSPKTIFIGAAIEHSMAEEIWDQLQSGSLTNSANLTKT
EQVGSLCRWIINI
11100000000000000000000000000000000000000000000000000000000000000000000000000000000
00000000000000000000000000000000000000000000000000000000000000000000000000000000000
00000000000000000000000000000000000000000000000000000011111111100000000000000000000
00000000000000000000000000000000000000000000000000000000000000000000000000
>P74902_1r5z_0_322
MADDFAYLNARVRVRRGTLLKESFFQEALDLSFADFLRLLSETVYGGELAGQGLPDVDRAV
LRTQAKLVGDLPRLVTGEAREAVRLLLLRNDLHNLQALLRAKATGRPFEEVLLLPGTLREE
VWRQAYEAQDPAGMAQVLAVPGHPLARALRAVLRETQDLARVEALLAKRFFEDVAKAA
KGLDQPALRDYLALEVDAENLRTAFKLQGSGLAPDAFFLKGGRFVDRVRFARLMEGDYAV
LDELSGTPFSGLSGVRDLKALERGLRCVLLKEAKKGVQDPLGVGLVLAYVKEREWEAVRLR
LLARRAYFGLPRAQVEEEVVCP
11000000000000000000000000000000000000000000000000000000000000000000000000000000000
00000000000000000000000000000000000000000000000000000000000000000000000000000000000
00000000000000000000000000000000000000000000000000000000000000000000000000000000000
00000000000000000000000000000000000000000000000000000000000000000000000001
>Q52349_3h20_0_322
MKNDRTLQAIGRQLKAMGCERFDIGVRDATTGQMMNREWSAAEVLQNTPWLKRMNAQ
GNDVYIRPAEQERHGLVLVDDLSEFDLDDMKAEGREPALVVETSPKNYQAWVKVADAAG
GELRGQIARTLASEYDADPASADSRHYGRLAGFTNRKDKHTTRAGYQPWVLLRESKGKTA
TAGPALVQQAGQQIEQAQRQQEKARRLASLELPERQLSRHRRTALDEYRSEMAGLVKRFG
DDLSKCDFIAAQKLASRGRSAEEIGKAMAEASPALAERKPGHEADYIERTVSKVMGLPSVQ
LARAELARAPAPRQRGMDRGGPDFSM
11110000000000000000000000000000000000000000000000000000000000000000000000000000000
00000000000000000000000000000000000000000000000000000000000000000000001000011100000
00000000000000000000000000000000000000000111111111000000000000000000000000000000000
00000000000000000000000000110000000000000000000000000000001111111111111111
>Q56328_4iil_20_342
RPAVQDERAVRIAVFVPGFRHDSPVYAMLCDGVERAVTQERATGRSIGLDIIEAGPNQALW
REKLAHLAAEQRYRLIVSSNPALPHVLEPILRQFPLQRFLVLDAYAPQEHSLITFRYNQWEQ
AYLAGHLSALVSASAMRFANADKKIGLIAGQSYPVMTQTIIPAFLAGARAVDPAFEVDVRV
VGNWYDAAKSADLARILFHEGVDVMMPICGGANQGVLAAARELGFYVSWFDDNGYAR
APGYVVGSSVMEQERLAYEQTLRCIRGELPSAGAWTLGVKDGYVRFIEEDPLYLQTVPEPIR
VRQSALLRRIQSGELTLPVR
11111110000000000000000000000000000000000000000000000000000000000000000000000000000
00000000000000000000000000000000000000000000000000000000000000000000000000000000000
00000000000000000000000000000000000000000000000000000000000000000000000000000000000
00000000000000000000000000000000000000000000000000000000000000000000000011

>Q86PD7_4fai_31_353
FELVDIPKISYNPSELSEPRFLEYSNLSDKLHLREAIDKILIPRVVGTTNHSIVREYIVQSLRDLD
WDVEVNSFHDHAPIKGKLHFHNIIATLNPNAERYLVLSCHYDSKYMPGVEFLGATDSAVP
CAMLLNLAQVLQEQLKPLKKSKLSLMLLFFDGEEAFEEWGPKDSIYGARHLAKKWHHEG
KLDRIDMLVLLDLLGAPDPAFYSFFENTESWYMRIQSVETRLAKLQLLERYASSGVAQRDPT
RYFQSQAMRSSFIEDDHIPFLRRNVPILHLIPVPFPSVWHTPDDNASVIDYATTDNLALIIRLF
ALEYLLAGTEAK
00000000000000000000000000000000000000000000000000000000000000000000000000000000000
00000000000000000000000000000000000000000000000000000000000000000000000000000000000
00000000000000000000000000000000000000000000000000000000000000000011111111111111000
00000000000000000000000000000000000000000000000000000000000000000000011111
>Q8IXJ6_1j8f_33_355
GEADMDFLRNLFSQTLSLGSQKERLLDELTLEGVARYMQSERCRRVICLVGAGISTSAGIPDF
RSPSTGLYDNLEKYHLPYPEAIFEISYFKKHPEPFFALAKELYPGQFKPTICHYFMRLLKDKGL
LLRCYTQNIDTLERIAGLEQEDLVEAHGTFYTSHCVSASCRHEYPLSWMKEKIFSEVTPKCE
DCQSLVKPDIVFFGESLPARFFSCMQSDFLKVDLLLVMGTSLQVQPFASLISKAPLSTPRLLIN
KEKAGQSDPFLGMIMGLGGGMDFDSKKAYRDVAWLGECDQGCLALAELLGWKKELEDL
VRREHASIDAQS
00000000000011111111111000000000000000000000000000000000000000000000000000000000000
00000000000000000000000000000000000000000000000000000000000000000000000000000000000
00000000000000000000000000000000000000000000000000000000000000000000000000000000000
0000000000000000000000000000000000000000000000000000000000000000000000000
>Q8ZRA1_3ga7_0_322
MKPENKIPVLTRLSDEMTAVVNFQQPGLPPWPADGDIETQRQYYLLERRFWNADAPSMTT
RTCAVPTPYGDVTTRLYSPQPTSQATLYYLHGGGFILGNLDTHDRIMRLLARYTGCTVIGIDY
SLSPQARYPQAIEETVAVCSYFSQHADEYSLNVEKIGFAGDSAGAMLALASALWLRDKHIR
CGNVIAILLWYGLYGLQDSVSRRLFGGAWDGLTREDLDMYEKAYLRNDEDRESPWYCLFN
NDLTRDVPPCFIASAEFDPLIDDSRLLHQTLQAHQQPCEYKMYPGTLHAFLHYSRMMTIAD
DALQDGARFFMARMKTPR
11100000000000000000000111111111100000000000000000000000000000000000000000000000000
00000000000000000000000000000000000000000000000000000000000000000000000000000000000
00000000000000000000000000000000000000000000000000000000000000000000000000000000000
0000000000000000000000000000000000000000000000000000000000000000000000011
>Q9HUL9_3crm_0_322
MSSLPPAIFLMGPTAAGKTDLAMALADALPCELISVDSALIYRGMDIGTAKPSRELLARYPH
RLIDIRDPAESYSAAEFRADALAAMAKATARGRIPLLVGGTMLYYKALLEGLADMPGADPE
VRAAIEAEAQAEGWEALHRQLAEVDPESAARIHPNDPQRLMRALEVYRLGGVSMSDLRR
RQSAEKADFDASGRNQLPYTVAQLAIAPEQRQVLHARIAQRFRQMLEQGFIAEVEALHAR
SDLHAGLPSIRAVGYRQVWDYLDGKLSYAEMTERGIIATRQLAKRQFTWLRSWSHLHWM
DSLAGDNLPRALKYLKTVSILA
11000000000000000000000000000000000000000000000000000000000000000000000000000000000
00000000000000000000000000000011111111111111111111111111111111111111111111111111111
11111111111111111111111111111110000000000000000000000000000000000000000000000000000
0000000000000000000000000000000000000000000000000000000000000000000000000
>Q9ULV8_3vrp_0_322
MALAVAPWGRQWEEARALGRAVRMLQRLEEQCVDPRLSVSPPSLRDLLPRTAQLLREVAH
SRRAAGGGGPGGPGGSGDFLLIYLANLEAKSRQVAALLPPRGRRSANDELFRAGSRLRRQL
AKLAIIFSHMHAELHALFPGGKYCGHMYQLTKAPAHTFWRESCGARCVLPWAEFESLLGT
CHPVEPGCTALALRTTIDLTCSGHVSIFEFDVFTRLFQPWPTLLKNWQLLAVNHPGYMAFL
TYDEVQERLQACRDKPGSYIFRPSCTRLGQWAIGYVSSDGSILQTIPANKPLSQVLLEGQKD
GFYLYPDGKTHNPDLTELG

11111111111000000000000000111100000000000000000000000000000000000000000000000000
00000000000000000111111111111100000000000000000000000000000000000000000000000000
00000000000000000000000000000000000000000000000000000000000000000000000000000000
00000000000000000000000000000000000000000000000000000000000000000000111
>A1YIY3_3p4g_996_1317
NVSQSNSFGFWDGTSTQAEITHSFDHYIGSAFDASNNNVAVTGNVSATLNVLAGDDKVSID
GNVEDVLVAANVAVLDMGTGNDQLYVAGDVLGKIDAGTGNDEIYIKGDVSAAVDAGTG
NDEVYIGGNLSGDLDAGTDNDNIQIGGDVNAALNAGTGNDNLIIGHDVSGIVNMGTDN
DTVEVGRTINASGKVLLDTGDDSLLVSGDLFGEVDGGTGNDTIIIAGKVSGNIQGGTGNDIV
RVQSQVWAEANISLGTGDDVLIVEHELHGTVAGNEGDDSIYLKFYTKEQYNNNSDLRNRV
ANFEHIRVSDGVVKGSPADFADY
11111110000000000000000000000000000000000000000000000000000000000000000000000000
00000000000000000000000000000000000000000000000000000000000000000000000000000000
00000000000000000000000000000000000000000000000000000000000000000000000000000000
0000000000000000000000000000000000000000000000000000000000000000000011
>P37696_1wzz_20_341
DPAPDAVAQQWAIFRAKYLRPSGRVVDTGNGGESHSEGQGYGMLFAASAGDLASFQSMW
MWARTNLQHTNDKLFSWRFLKGHQPPVPDKNNATDGDLLIALALGRAGKRFQRPDYIQD
AMAIYGDVLNLMTMKAGPYVVLMPGAVGFTKKDSVILNLSYYVMPSLLQAFDLTADPRW
RQVMEDGIRLVSAGRFGQWRLPPDWLAVNRATGALSIASGWPPRFSYDAIRVPLYFYWAH
MLAPNVLADFTRFWNNFGANALPGWVDLTTGARSPYNAPPGYLAVAECTGLDSAGELPT
LDHAPDYYSAALTLLVYIARAEETIK
11000000000000000000000000000000000000000000000000000000000000000000000000000000
00000000000000000000000000000000000000000000000000000000000000000000000000000000
00000000000000000000000000000000000000000000000000000000000000000000000000000000
0000000000000000000000000000000000000000000000000000000000000000000001
>Q40316_2p4h_4_325
KGRVCVTGGTGFLGSWIIKSLLENGYSVNTTIRADPERKRDVSFLTNLPGASEKLHFFNADL
SNPDSFAAAIEGCVGIFHTASPIDFAVSEPEEIVTKRTVDGALGILKACVNSKTVKRFIYTSSGS
AVSFNGKDKDVLDESDWSDVDLLRSVKPFGWNYAVSKTLAEKAVLEFGEQNGIDVVTLILP
FIVGRFVCPKLPDSIEKALVLVLGKKEQIGVTRFHMVHVDDVARAHIYLLENSVPGGRYNCS
PFIVPIEEMSQLLSAKYPEYQILTVDELKEIKGARLPDLNTKKLVDAGFDFKYTIEDMFDDAI
QCCKEKGYL
00000000000000000000000000000000001111000000000000000000000000000000000000000000
00111111100000000000000000000000000000000000000000000000000000000000000000000000
00000000000000000000000000000000000000000000000000000000000000000000000000000000
0000000000000000000000000000000000000000000000000000000000000000000000
>Q44048_1f1r_0_321
MTNFVPTPSVPAPDIVRCAYMEIVVTDLAKSREFYVDVLGLHVTEEDENTIYLRSLEEFIHHN
LVLRQGPIAAVAAFAYRVKSPAEVDAAEAYYKELGCRTERRKEGFTKGIGDSVRVEDPLGFP
YEFFYETEHVERLTQRYDLYSAGELVRLDHFNQVTPDVPRGRAYLEDLGFRVSEDIKDSDGV
TYAAWMHRKQTVHDTALTGGNGPRMHHVAFATHEKHNIIQICDKMRPCASATGSNGPRP
APVSNAFYLYILDPDGHRIEIYTQDYYTGDPDNPTITWDVHDNQRRDWWGNPVVPSWYTE
ASLVLDLDGNPQPVIV
11000000000000000000000000000000000000000000000000000000000000000000000000000000
00000000000000000000000000000000000000000000000000000000000000000000000000000000
00000000000000000000000000000000000000000000000000000000000000000000000000000000
0000000000000000000000000000000000000000000000000000000000000000000000
>Q86Y07_2v62_13_334
PFPEGKVLDDMEGNQWVLGKKIGSGGFGLIYLAFPTNKPEKDARHVVKVEYQENGPLFSE
LKFYQRVAKKDCIKKWIERKQLDYLGIPLFYGSGLTEFKGRSYRFMVMERLGIDLQKISGQN

GTFKKSTVLQLGIRMLDVLEYIHENEYVHGDIKAANLLLGYKNPDQVYLADYGLSYRYCP
NGNHKQYQENPRKGHNGTIEFTSLDAHKGVALSRRSDVEILGYCMLRWLCGKLPWEQNL
KDPVAVQTAKTNLLDELPQSVLKWAPSGSSCCEIAQFLVCAHSLAYDEKPNYQALKKILNP
HGIPLGPLDFSTKGQSINVH
10000000000000000000001111100000000000000000000000000100000000000000000000000000000
00000000000000000000000000000000000000000000000000000000000000000000000000000000000
00000000000000000000000000000000000000000000000000000000000000000000000000000000000
00000000000000000000000000000000000000000000000000000000000000000011111
>A9CGZ4_2hmc_0_320
MTASIFSGVIPALMTPCRQDRTPDFDALVRKGKELIADGMSAVVYCGSMGDWPLLTDEQR
MEGVERLVKAGIPVIVGTGAVNTASAVAHAVHAQKVGAKGLMVIPRVLSRGSVIAAQKAH
FKAILSAAPEIPAVIYNSPYYGFATRADLFFALRAEHKNLVGFKEFGGPADMRYAAENITSRD
DEVTLMIGVDTAVVHGFVNCGATGAITGIGNVLPKEVIHLCKLSQAAAKGDADARARALE
LEQALAVLSSFDEGPDLVLYFKYMMVLKGDKEYTLHFNETDALTDSQRGYVEAQFKLFNS
WYADWSKLPGAVQTCKAA
00000000000000000000000000000000000000000000000000000000000000000000000000000000000
00000000000000000000000000000000000000000000000000000000000000000000000000000000000
00000000000000000000000000000000000000000000000000000000000000000000000000000000000
00000000000000000000000000000000000000000000000000000000000011111111111
>P53571_3cls_0_320
MSKILVIAEHRRNDLRPVSLELIGAANGLKKSGEDKVVVAVIGSQADAFVPALSVNGVDEL
VVVKGSSIDFDPDVFEASVSALIAAHNPSVVLLPHSVDSLGYASSLASKTGYGFATDVYIVEY
QGDELVATRGGYNQKVNVEVDFPGKSTVVLTIRPSVFKPLEGAGSPVVSNVDAPSVQSRSQ
NKDYVEVGGGNDIDITTVDFIMSIGRGIGEETNVEQFRELADEAGATLCCSRPIADAGWLP
KSRQVGQSGKVVGSCKLYVAMGISGSIQHMAGMKHVPTIIAVNTDPGASIFTIAKYGIVADI
FDIEEELKAQLAA
10000000000000000000000000000000000000000000000000000000000000000000000000000000000
00000000000000000000000000000000000000000000000000000000000000000000000000000000000
00000000000000000000000000000000000000000000000000000000000000000000000000000000000
00000000000000000000000000000000000000000000000000000000000000000000011
>Q7T291_2nxf_1_321
EDPVFTFGLIADVQYADIEDGENYLRTRRRYYRGSADLLRDAVLQWRRERVQCVVQLGDII
DGHNRRRDASDRALDTVMAELDACSVDVHHVWGNHEFYNFSRPSLLSSRLNSAQRTGTD
TGSDLIGDDIYAYEFSPAPNFRFVLLDAYDLSVIGREEESEKHTHSWRILTQHNHNLQDLNLP
PVSVGLEQRFVKFNGGFSEQQLQWLDAVLTLSDHKQERVLIFSHLPVHPCAADPICLAWN
HEAVLSVLRSHQSVLCFIAGHDHDGGRCTDSSGAQHITLEGVIETPPHSHAFATAYLYEDRM
VMKGRGRVEDLTITYS
10000000000000000000000000000000000000000000000000000000000000000000000000000000000
00000000000000000000000000000000111111000000000000000000000000000000000000000000000
00000000000000000000000000000000000000000000000000000000000000000000000000000000000
00000000000000000000000000000000000000000000000000000000000000000000000
>Q8DJP8_2ozt_1_321
RWQWRIYEEPLQEPLTTAQGVWRSRSGIYLRLEDEQGQVGYGEIAPLPGWGSETLNADIAL
CQQLPGHLTPEIMATIPEALPAAQFGFATAWQSVGRLPYRVRPWPICALLGSGQAALEQWQ
QSWQRGQTTFKWKVGVMSPEEEQAILKALLAALPPGAKLRLDANGSWDRATANRWFAW
LDRHGNGKIEYVEQPLPPDQWQALLSLAQTVTTAIALDESVVSAAEVQRWVDRGWPGFFV
IKTALFGDPDSLSLLLRRGLEPQRLVFSSALEGAIARTAIFHLLETWQPCHALGFGVDRWRS
APLLTTLTAYERLWERLDQ
00000000000000000000000000000000000000000000000000000000000000000000000000000000000
00000000000000000000000000000000000000000000000000000000000000000000000000000000000
00000000000000000000000000000000000000000000000000000000000000000000000000000000000

00000000000000000000000000000000000000000000000000000000000000000000011
>Q97YD2_3qyf_0_320
MEVHVCSVGTSLLKNSLDDDNVRKEIERLGLKDWDRLKFDDDRQNRIKENFDSLRKMLLK
FIRSKGRRASAELDSLFSTFEKLKHNKSEIYVFLYSTNTSNSQLAGEVIRDYLIEEGIRSELVTV
KTISSEENFYEGIVDLFDKVIYRILKFKEQDNEVYINATPGLKPESIFLTLAGLLAGADLIYYKY
QEFNDVVILPSPPITIRPKYLDWLIRFAISGYTLSEKRAEELGIPVRLLEAKMLVERKGEDAYR
LKDWVRKLLGIYLPIGAQNKYYRVIVEGEGERTFDNEVEAYNYMESKRKEGKNVRVEVPD
RVYFLGL
00000000000000000000000000000000000000000000000000000000000000000000000000000000000
00000000000000000000000000000000000000000000000000000000000000000000000000000000000
00000000000000000000000000000000000000000000000000000000000000000000000000000000000
00000000000000000001111111111111111111111111111111111111111111111111111
>A5H1I6_3civ_0_319
MGRIESAFDLGFIRGMTFGFVGQHGTWGTDEARASMRALAEQPFNWVTLAFAGLMEHPG
DPAIAYGPPVTVSDDEIASMAELAHALGLKVCLKPTVNCRDGTWRGEIRFEKEHGPDLESW
EAWFGSYSDMMAHYAHVAKRTGCEMFCVGCEMTTAEPHEAMWRETIARVRTEYDGLVT
YNCNHGREEHVRFWDAVDLISSSAYYPIDRWRDRVPVLREVAEAHEKPLFFMEVGCPSRSG
SGACPWDYRHPGAVCLDEQARFYEAMFAAMPDEPWFKGYMLWEWPWKLYPREAASED
GSYCIYGKPAEDVVARAFSAIANR
11110000000000000000000000000000000000000000000000000000000000000000000000000000000
00000000000000000000000000000000000000000000000000000000000000000000000000000000000
00000000000000000000000000000000000000000000000000000000000000000000000000000000000
0000000000000000000000000000000000000000000000000000000000000000000000
>A9J7N5_3bb7_83_402
NQLRELKQTHTYTVFGYTDGGFAVISADDLAPELLGVSESNFVETDNPSFKWWLKAIDEVIT
NAVKNNKPLSVIKPDPSKYAAEVSTLLTTTWGQQMPYNKLLPNTKKGRLITGCVATATAQA
LNYFKYPVRGIGSHTVHYPANDPSGVAISADFGNTTYDWANMKDDYSGNYTEAEANAVA
TLMLHCGVASEMQYGGPNEGSGAYMTDCAAGLRTYFGFTDAEYITRADYTDEQWMDIVF
SELTKGHPLIYGGVSPGSMGQDAGHAFVIDGYNKAGLVSVNWGWNGDVDGYYKIDLLNP
GNMYSFTAEQDMVRGVYGKP
00000000000000000000000000000000000000000000000000000000000000000000000000000000000
00000000000000000000000000000000000000000000000000000000000000000000000000000000000
00000000000000000000000000000000000000000000000000000000000000000000000000000000000
0000001111111000000000000000000000000000000000000000000000000000000000
>D0VWU8_3ec5_0_319
GSHMKNSVSVDLPGSMKVLVSKSSNADGKYDLIATVDALELSGTSDKNNGSGVLEGVKAD
ASKVKLTISDDLGQTTLEVFKSDGSTLVSKKVTSKDKSSTYELFNEKGELSLKYITRADKSSTY
ELFNEKGELSLKYITRADKSSTYELFNEKGELSEKKITRADKSSTYELFNEKGELSLKYITRAD
GTRLEYTGIKSDGSGKAKEVLKGYVLEGTLTAEKTTLVVKEGTVTLSKNISKSGEVSVELNDT
DSSAATKKTAAWNSGTSTLTITVNSKKTKDLVFTSSNTITVQQYDSNGTSLEGSAVEITKLDEI
KNALK
11110000000000000000000000000000000000000000000000000000000000000000000000000000000
00000000000000000000000000000000000000000000000000000000000000000000000000000000000
00000000000000000000000000000000000000000000000000000000000000000000000000000000000
0000000000000000000000000000000000000000000000000000000000000000000000
>F4Y423_4gbm_1597_1916
SPTSLEIFATKSSPSGNSARPASVSSRLPGIIFILSSPRSGSTLLRVMLAGHSSLFSPPELHLLPFN
TMKERQEQLNLSYLGEGLQKTFMEVKNLDATASQALIKDLESQNLSIQQVYGMLQENIAP
RLLVDKSPTYAMEPTILERGEALFANSKYIYLVRHPYSVIESFVRMRMQKLVGLGEENPYRVA
EQVWAKSNQNILNFLSQLEPERQHQIRYEDLVKKPQQVLSQLCDFLNVPFEPELLQPYQGD
RMTGGVHQKSLSISDPNFLKHNTIDESLADKWKTIQLPYPLKSETQRIASQLSYELPNLVTTP

TNQQPQ
11111111111111111111111110000000000000000000000000000000000000000000000000000000
00000000000000000000000000000000000000000000000000000000000000000000000000000000
00000000000000000000000000000000000000000000000000000000000000000000000000000000
00000000000000000000000000000000000000000000000000000000111111111111
>P26039_3dyj_1973_2292
GTQACITAASAVSGIIADLDTTIMFATAGTLNREGAETFADHREGILKTAKVLVEDTKVLVQ
NAAGSQEKLAQAAQSSVATITRLADVVKLGAASLGAEDPETQVVLINAVKDVAKALGDLIS
ATKAAAGKVGDDPAVWQLKNSAKVMVTNVTSLLKTVKAVEDEATKGTRALEATTEHIRQ
ELAVFCSPEPPAKTSTPEDFIRMTKGITMATAKAVAAGNSCRQEDVIATANLSRRAIADMLR
ACKEAAFHPEVAPDVRLRALHYGRECANGYLELLDHVLLTLQKPNPDLKQQLTGHSKRV
AGSVTELIQAAEAMKGT
10000000000000000000000000000000000000000000000000000000000000000000000000000000
00000000000000000000000000000000000000000000000000000000000000000000000000000000
00000000000000000000000000000000000000000000000000000000000000000000000000000000
00000000000000000000000000000000000000000000000000000000000000000011
>P37595_3c17_1_320
GKAVIAIHGGAGAISRAQMSLQQELRYIEALSAIVETGQKMLEAGESALDVVTEAVRLLEEC
PLFNAGIGAVFTRDETHELDACVMDGNTLKAGAVAGVSHLRNPVLAARLVMEQSPHVM
MIGEGAENFAFARGMERVSPEIFSTSLRYEQLLAARKEGATVLDHSGAPLDEKQKMGTVGA
VALDLDGNLAAATSTGGMTNKLPGRVGDSPLVGAGCYANNASVAVSCTGTGEVFIRALAA
YDIAALMDYGGLSLAEACERVVMEKLPALGGSGGLIAIDHEGNVALPFNTEGMYRAWGYA
GDTPTTGIYREKGDTVATQ
00000000000000000000000000000000000000000000000000000000000000000000000000000000
00000000000000000000000000000000000000000000000000000000000000000000000000111111
00000000000000000000000000000000000000000000000000000000000000000000000000000000
00000000000000000000000000000000000000000000000000000000000001111111
>Q2T3W4_4dz4_0_319
MNETLYGDGAIRRPSVYGSSIENTYAGVLSFMRRNYTRDLDGVDVVVSGVPLDLATTFRSG
ARLGPSAVRAASVQLAELNPYPWGFDPFDDLAVIDYGDCWFDAHHPLSIKPAIVEHARTIL
QSDARMLTLGGDHYITYPLLIAHAQKYGKPLSLIHFDAHCDTWADDAPDSLNHGTMFYK
AVKDGLIDPKASVQVGIRTWNDDYLGINVLDAAWVHEHGARATLERIESIVGGRPAYLTFD
IDCLDPAFAPGTGTPVAGGLSSAQALAIVRGLGGVNLIGADVVEVAPAYDQSEITAIAAAHV
ACDLLCLWRQRKAGAR
11100000000000000000000000000000000000000000000000000000000000000000000000000000
00000000000000000000000000000000000000000000000000000000000000000000000000000000
00000000000000000000000000000000000000000000000000000000000000000000000000000000
00000000000000000000000000000000000000000000000000000000000000000000
>Q43316_4htg_62_381
CVAVEQKTRTAIIRIGTRGSPLALAQAYETREKLKKKHPELVEDGAIHIEIIKTTGDKILSQPLA
DIGGKGLFTKEIDEALINGHIDIAVHSMKDVPTYLPEKTILPCNLPREDVRDAFICLTAATLA
ELPAGSVVGTASLRRKSQILHKYPALHVEENFRGNVQTRLSKLQGGKVQATLLALAGLKRL
SMTENVASILSLDEMLPAVAQGAIGIACRTDDDKMATYLASLNHEETRLAISCERAFLETLD
GSCRTPIAGYASKDEEGNCIFRGLVASPDGTKVLETSRKGPYVYEDMVKMGKDAGQELLSR
AGPGFFGN
11111111100000000000000000000000000000000000000000000000000000000000000000000000
00000000000000000000000000000000000000000000000000000000000000000000000000000000
00000000000000000000000000000000000000000000000000000000000000000000000000000000
00000000000000000000000000000000000000000000000000000000000111111111
>Q83VG5_3v10_30_349
KDYTNEAIFTQFDVNPKGLINNPSQPIEFNLAFSDMNNGQKVKFKPGDFFDLTLPSNDEVS

LRSLRAMGSKMPVLAKDKNGKEITLGELTFNGSHIHFEFMEDVLQLENVTGTINLKSVYDN
AYRGEDDKIAELPTNLGLGSLDKQMITISQPGTPTGVEPSPIFYWKTGTFSTEVHGDMNWW
LNINSPKEAVQSDVKVIDTIGEGHKLVDGSIMVDVEANGELKHISAEAFNKEYGTITVEGQV
LTVMIPKEKAAKTTFTVTYDTRAFDKKLENYKNSSTIEYKDESGNLVTDTPKHYTDTSVVN
MFDDATIGGEMKDK
00000000000000000000000000000000000000000000000000000000000000000000000000000111100
00000000000000000000000000000000000000000000000000000000000000000000000000111000000
00000000000000000000000000000000000000000000000000000000000000000000000000000000000
00000000000000000000000000000000000000000000000000000000000000000000000
>Q8KDY2_3gn6_0_319
MTGLSQSQASPMQIQPGNAAFNPWTDAALDTIRDVNQALTLYAEMRVVPAHHDAFLAAI
DTVSAKLRVLPGFLSLALKQMSGDSTMVKNYPETYKGVLATAYLDGVAAGTQPYFYNLFV
RFADGRAARAAGFEALFETHIHPLLHAMAPRGGDGPELLAYRAVLQSVVAGDRHAIYRGA
EEIRSFLRRPVELPERETVTVENHVMVPEDKHAAWEPQVAILLQVAQDTFEPQDEPSGVGLP
GARDNRYYRKALSTEILRNAHADGGLRAYIMHGVWESVWDHENSHLDPRFLAAAGPVG
AAAVVGPVEPFYLTRRLVVAD
11111111111111111111000000000000000000000000000000000000000000000000000000000000000
00000000000000000000000000000000000000000000000000000000000000000111100000000000000
00000000000000000000000000000000000000000000000000000000000000000000000000000000000
00000000000000000000000000000000000000000000000000000000000000000000000
>Q9XZ16_3shq_0_319
MEVKEVVVIVKWSGKEYPVDLTDQDTVEVLRHEIFRKTQVRPERQKLLNLKYKGKTAADN
VKISALELKPNFKLMMVGSTEADIEDACSLPDNIGEVVDDFDDADEREESVEHSAVYLAKV
QRRVRDYKIKELAPPREGKKLLVLDIDYTLFDHRSPAETGTELMRPYLHEFLTSAYEDYDIVI
WSATSMRWIEEKMRLLGVASNDNYKVMFYLDSTAMISVHVPERGVVDVKPLGVIWALYK
QYNSSNTIMFDDIRRNFLMNPKSGLKIRPFRQAHLNRGTDTELLKLSDYLRKIAHHCPDFN
SLNHRKWEHYHPKKNS
11100000000000000000000000000000000000000000000000000000000000000001100000000001111
11111111000000000000000110000000000000000000000000000000000000000000000000000000000
00000000000000000000000000000000000000000000000000000000000000000000000000000000000
00000000000000000000000000000000000000000000000000000000000000000001111
>A9CHQ7_2rbc_0_318
MVKEPGGKHVLCVGAAVLDTLFRVADMPKGEGKVLPYEVLQIAEGMASSAAYAVHRMGG
RASLWGAVGDDETGTRILRDLSESGIDTSGMTVAPGARSALSTIIIDNRGERLIVPFYDHRLH
EKKRACTPEDIALFDAVLVDVRWPELALDVLTVARALGKPAILDGDVAPVETLEGLAPAAT
HIVFSEPAATRLTGLETVKDMLPVLHARYPQTFIAVTAGPAGCWWTEADDPTVHFQTTMQ
VEAVDTLAAGDIFHGTFALAMAEGMQSRAAVRLSSVAAALKCTVFGGRIGAPTREETEEA
MRQWLERESEPALRAS
11111000000000000000000000000000000000000000000000000000000000000000000000000000000
00000000000000000000000000000000000000000000000000000000000000000000000000000000000
00000000000000000000000000000000000000000000000000000000000000000000000000000000000
0000000000000000000000000000000000000000000000000000000000000011111111
>P14755_3adp_0_318
MASPAAGDVLIVGSGLVGRSWAMLFASGGFRVKLYDIEPRQITGALENIRKEMKSLQQSGSL
KGSLSAEEQLSLISSCTNLAEAVEGVVHIQECVPENLDLKRKIFAQLDSIVDDRVVLSSSSSCL
LPSKLFTGLAHVKQCIVAHPVNPPYYIPLVELVPHPETSPATVDRTHALMRKIGQSPVRVLKE
IDGFVLNRLQYAIISEAWRLVEEGIVSPSDLDLVMSDGLGMRYAFIGPLETMHLNAEGMLSY
CDRYSEGMKRVLKSFGSIPEFSGATVEKVNQAMCKKVPADPEHLAARREWRDECLKRLAK
LKRQMQPQ
11111100000000000000000000000000000000000000000000000000000000000000000000000000000
00000000000000000000000000000000000000000000000000000000000000000000000000000000000

000000000000000000000000000000000000000000000000000000000000000000000000000000000000
0000000000000000000000000000000000000000000000000000000000000000111
>P52333_3lxl_805_1123
AQLYACQDPTIFEERHLKYISQLGKGNFGSVELCRYDPLGDNTGALVAVKQLQHSGPDQQR
DFQREIQILKALHSDFIVKYRGVSYGPGRQSLRLVMEYLPSGCLRDFLQRHRARLDASRLLLY
SSQICKGMEYLGSRRCVHRDLAARNILVESEAHVKIADFGLAKLLPLDKDYYVVREPGQSPI
FWYAPESLSDNIFSRQSDVWSFGVVLYELFTYCDKSCSPSAEFLRMMGCERDVPALCRLLEL
LEEGQRLPAPPACPAEVHELMKLCWAPSPQDRPSFSALGPQLDMLWSGSRGCETHAFTAH
PEGKHHSLSFS
111111100000000000000000000000000000000000000000000000000000000000000000000000000000
000000000000000000000000000000000000000000000000000000000000000000000000000000000000
000000000000000000000000000000000000000000000000000000000000001111000000000000000000
0000000000000000000000000000000000000000000011111111111111111111111
>Q7WUJ1_2pt0_27_345
AKAPEQTVTEPVGSYARAERPQDFEGFVWRLDNDGKEALPRNFRTSADALRAPEKKFHLD
AAYVPSREGMDALHISGSSAFTPAQLKNVAAKLREKTAGPIYDVDLRQESHGYLDGIPVSW
YGERDWANLGKSQHEALADERHRLHAALHKTVYIAPLGKHKLPEGGEVRRVQKVQTEQE
VAEAAGMRYFRIAATDHVWPTPENIDRFLAFYRTLPQDAWLHFHCEAGVGRTTAFMVMT
DMLKNPSVSLKDILYRQHEIGGFYYGEFPIKTKDKDSWKTKYYREKIVMIEQFYRYVQENRA
DGYQTPWSVWLKSHPAKA
111111000000000000000000000000000000000000000000000000000000000000000000000000000000
000000000000000000000000000000000000000000000000000000000000000000000000000000000000
000000000000000000000000000000000000000000000000000000000000000000000000000000000000
0000000000000000000000000000000000000000000000000000000000000000000
>Q91062_1lsh_1305_1623
KYVPQRKPQTSRRHTPASSSSSSSSSSSSSSSSSSSDSDMTVSAESFEKHSKPKVVIVLRAVRAD
GKQQGLQTTLYYGLTSNGLPKAKIVAVELSDLSVWKLCAKFRLSAHMKAKAAIGWGKNC
QQYRAMLEASTGNLQSHPAARVDIKWGRLPSSLQRAKNALLENKAPVIASKLEMEIMPKK
NQKHQVSVILAAMTPRRMNIIVKLPKVTYFQQGILLPFTFPSPRFWDRPEGSQSDSLPAQIAS
AFSGIVQDPVASACELNEQSLTTFNGAFFNYDMPESCYHVLAQECSSRPPFIVLIKLDSERRIS
LELQLDDK
111111111111111111111111111111111111111111111111110000000000000000000000000000000000
000000000000000000000000000000000000000000000000000000000000000000000000000000000000
000000000000000000000000000000000000000000000000000000000001111111111111111111111111
1111111111111111111111111111111111111111111111111111111111111111111
>Q91CD4_1jsd_18_336
DKICIGYQSTNSTETVDTLTETNVPVTHAKELLHTEHNGMLCATNLGHPLILDTCTIEGLIY
GNPSCDLLLGGREWSYIVERPSAVNGMCYPGNVENLEELRSLFSSASSYQRIQIFPDTIWNVS
YSGTSKACSDSFYRSMRWLTQKNNAYPIQDAQYTNNRGKSILFMWGINHPPTDTVQTNLY
TRTDTTTSVTTEDINRTFKPVIGPRPLVNGLHGRIDYYWSVLKPGQTLRVRSNGNLIAPWYG
HILSGESHGRILKTDLNSGNCVVQCQTERGGLNTTLPFHNVSKYAFGNCPKYVGVKSLKLA
VGLRNVPARSS
000000000000000000000000000000000000000000000000000000000000000000000000000000000000
000000000000000000000000000000000000000000000000000000000000000000000000000000000000
000000000000000000000000000000000000000000000000000000000000000000000000000000000000
0000000000000000000000000000000000000000000000000000000000000000011
>Q9V2T0_3vgi_0_318
MSKKKFVIVSILTILLVQAIYFVEKYHTSEDKSTSNTSSTPPQTTLSTTKVLKIRYPDDGEWPG
APIDKDGDGNPEFYIEINLWNILNATGFAEMTYNLTSGVLHYVQQLDNIVLRDRSNWVHG
YPEIFYGNKPWNANYATDGPIPLPSKVSNLTDFYLTISYKLEPKNGLPINFAIESWLTREAWR
TTGINSDEQEVMIWIYYDGLQPAGSKVKEIVVPIIVNGTPVNATFEVWKANIGWEYVAFRIK

TPIKEGTVTIPYGAFISVAANISSLPNYTELYLEDVEIGTEFGTPSTTSAHLEWWITNITLTPLD
RPLIS
1111111111111111111111111111111111111111111111111100000000000000000000000000000000000
0000000000000000000000000000000000000000000000000000000000000000000000000000000000000
0000000000000000000000000000000000000000000000000000000000000000000000000000000000000
00000000000000000000000000000000000000000000000000000000000000000000000
>Q9XYQ1_1z2n_0_318
MTTKQTVSLFIWLPESKQKTLFISTKNHTQFELNNIIFDVTLSTELPDKEPNAIITKRTHPVGK
MADEMRKYEKDHPKVLFLESSAIHDMMSSREEINALLIKNNIPIPNSFSVKSKEEVIQLLQSK
QLILPFIVKPENAQGTFNAHQMKIVLEQEGIDDIHFPCLCQHYINHNNKIVKVFCIGNTLK
WQTRTSLPNVHRCGIKSVDFNNQHLEDILSWPEGVIDKQDIIENSANRFGSKILEDPILLNLT
SEAEMRDLAYKVRCALGVQLCGIDFIKENEQGNPLVVDVNVFPSYGGKVDFDWFVEKVAL
CYTEVAKI
1111000000000000000000000000000000000000000000000000000000000000000000000000000000000
0000000000000000000000000000000000000000000000000000000000000000000000000000000000000
0000000000000000000000000000000000000000000000000000000000000000000000000000000000000
00000000000000000000000000000000000000000000000000000000000000000001111
>B3A043_3h4x_26_343
EPAATTYGTSTSVGVHNAYEKEKYRYFADALDSGAALLELDLWSNALGRSWRVSHSNPLG
NNSNCEGAANASELRTKSRDQDFAGCLSDMRAWHDAHPGHRPILLKIEMKDGFNAKGG
RGPAEFDALIRQKLGDAVYGPGDLTGGHATADEAVRAGGWPSRADLAGKFLFELIPGTVEE
KNPFDKLWTDVEYAGHLKDLAAQGKLAQSTAFPAVHGAAPGDPRERYADPALRPWFVVF
DGDAPTYLNGSIDTSWYDTRHYLLIMTDAHNVPPVIDGTHPTEAEALARVRQLAAAHASF
ATADWYPLPSVLKTVVPRGA
1110000000000000000000000000000000000000000000000000000000000000000000000000000000000
0000000000000000000000000000000000000000000000000000000000000000000000000000000000000
0000000000000000000000000000000000000000000000000000000000000000000000000000000000000
0000000000000000000000000000000000000000000000000000000000000000000000
>P29724_2fqw_35_352
EGGDFVVGMVTDSGDIDDKSFNQQVWEGISRFAQENNAKCKYVTASTDAEYVPSLSAFAD
ENMGLVVACGSFLVEAVIETSARFPKQKFLVIDAVVQDRDNVVSAVFGQNEGSFLVGVAAA
LKAKEAGKSAVGFIVGMELGMMPLFEAGFEAGVKAVDPDIQVVVEVANTFSDPQKGQAL
AAKLYDSGVNVIFQVAGGTGNGVIKEARDRRLNGQDVWVIGVDRDQYMDGVYDGSKSV
VLTSMVKRADVAAERISKMAYDGSFPGGQSIMFGLEDKAVGIPEENPNLSSAVMEKIRSFEE
KIVSKEIVVPVRSARMMN
1100000000000000000000000000000000000000000000000000000000000000000000000000000000000
0000000000000000000000000000000000000000000000000000000000000000000000000000000000000
0000000000000000000000000000000000000000000000000000000000000000000000000000000000000
0000000000000000000000000000000000000000000000000000000000000000000000
>P45299_3ckm_255_572
VSQIGLLLPLSGDGQILGTTIQSGFNDAKGNSTIPVQVFDTSMNSVQDIIAQAKQAGIKTLVG
PLLKQNLDVILADPAQIQGMDVLALNATPNSRAIPQLCYYGLSPEDEAESAANKMWNDG
VRNPLVAMPQNDLGQRVGNAFNVRWQQLAGTDANIRYYNLPADVTYFVQENNSNTTAL
YAVASPTELAEMKGYLTNIVPNLAIYASSRASASATNTNTDFIAQMNGVQFSDIPFFKDTNSP
QYQKLAKSTGGEYQLMRLYAMGADAWLLINQFNELRQVPGYRLSGLTGILSADTNCNVER
DMTWYQYQDGAIVPV
1000000000000000000000000000000000000000000000000000000000000000000000000000000000000
0000000000000000000000000000000000000000000000000000000000000000000000000000000000000
0000000000000000000000000000000000000000000000000000000000000000000000000000000000000
0000000000000000000000000000000000000000000000000000000000000000000000
>P94388_1odt_0_317

MQLFDLPLDQLQTYKPEKTAPKDFSEFWKLSLEELAKVQAEPDLQPVDYPADGVKVYRLTY
KSFGNARITGWYAVPDKEGPHPAIVKYHGYNASYDGEIHEMVNWALHGYATFGMLVRGQ
QSSEDTSISPHGHALGWMTKGILDKDTYYYRGVYLDAVRALEVISSFDEVDETRIGVTGGSQ
GGGLTIAAAALSDIPKAAVADYPYLSNFERAIDVALEQPYLEINSFFRRNGSPETEVQAMKT
LSYFDIMNLADRVKVPVLMSIGLIDKVTPPSTVFAAYNHLETKKELKVYRYFGHEYIPAFQTE
KLAFFKQHLKG
000000000000000000000000000000000000000000000000000000000000000000000000000000000000000000000
000000000000000000000000000000000000000000000000000000000000000000000000000000000000000000000
000000000000000000000000000000000000000000000000000000000000000000000000000000000000000000000
00000000000000000000000000000000000000000000000000000000000000000000000000001
>Q29980_2wy3_23_340
EPHSLRYNLMVLSQDESVQSGFLAEGHLDGQPFLRYDRQKRRAKPQGQWAEDVLGAKTW
DTETEDLTENGQDLRRTLTHIKDQKGGLHSLQEIRVCEIHEDSSTRGSRHFYYDGELFLSQN
LETQESTVPQSSRAQTLAMNVTNFWKEDAMKTKTHYRAMQADCLQKLQRYLKSGVAIRR
TVPPMVNVTCSEVSEGNITVTCRASSFYPRNITLTWRQDGVSLSHNTQQWGDVLPDGNGT
YQTWVATRIRQGEEQRFTCYMEHSGNHGTHPVPSGKVLVLQSQRTDFPYVSAAMPCFVIIII
LCVPCCKKKTSAAEGP
000000000000000000000000000000000000000000000000000000000000000000000000000000000000000000000
000000000000000000000000000000000000000000000000000000000000000000000000000100000000000000000
000000000111111111111111111111111111111111111111111111111111111111111111111111111111111111111
11111111111111111111111111111111111111111111111111111111111111111111111111111
>O06629_3ib8_1_317
HRLRAAEHPRPDYVLLHISDTHLIGGDRRLYGAVDADDRLGELLEQLNQSGLRPDAIVFTG
DLADKGEPAAYRKLRGLVEPFAAQLGAELVWVMGNHDDRAELRKFLLDEAPSMAPLDRV
CMIDGLRIIVLDTSVPGHHHGEIRASQLGWLAEELATPAPDGTILALHHPPIPSVLDMAVTV
ELRDQAALGRVLRGTDVRAILAGHLHYSTNATFVGIPVSVASATCYTQDLTVAAGGTRGRD
GAQGCNLVHVYPDTVVHSVIPLGGGETVGTFVSPGQARRKIAESGIFIEPSRRDSLFKHPPM
VLTSSAPRSPVD
110000000000000000000000000000000000000000000000000000000000000000000000000000000000000000000
000000000000000000000000000000000000000000000000000000000000000000000000000000000000000000000
000000000000000000000000000000000000000000000000000000000000000000000000000000000000000000000
00000000000000000000000000000000000000000000000000000011111111111111111111
>P21853_1h2a_0_316
MKISIGLGKEGVEERLAERGVSRRDFLKFCTAIAVTMGMGPAFAPEVARALMGPRRPSVVYL
HNAECTGCSESVLRAFEPYIDTLILDTLSLDYHETIMAAAGDAAEAALEQAVNSPHGFIAVV
EGGIPTAANGIYGKVANHTMLDICSRILPKAQAVIAYGTCATFGGVQAAKPNPTGAKGVN
DALKHLGVKAINIAGCPPNPYNLVGTIVYYLKNKAAPELDSLNRPTMFFGQTVHEQCPRLP
HFDAGEFAPSFESEEARKGWCLYELGCKGPVTMNNCPKIKFNQTNWPVDAGHPCIGCSEP
DFWDAMTPFYQN
111111111111111111111111111111111111111111111111111111110000000000000000000000000000000000000
000000000000000000000000000000000000000000000000000000000000000000000000000000000000000000000
000000000000000000000000000000000000000000000000000000000000000000000000000000000000000000000
0000000000000000000000000000000000000000000000000000000000000000000000000000
>Q16644_3fhr_32_348
EPKKYAVTDDYQLSKQVLGLGVNGKVLECFHRRTGQKCALKLLYDSPKARQEVDHHWQA
SGGPHIVCILDVYENMHHGKRCLLIIMECMEGGELFSRIQERGDQAFTEREAAEIMRDIGTA
IQFLHSHNIAHRDVKPENLLYTSKEKDAVLKLTDFGFAKETTQNALQTPCYTPYYVAPEVLG
PEKYDKSCDMWSLGVIMYILLCGFPPFYSNTGQAISPGMKRRIRLGQYGFPNPEWSEVSEDA
KQLIRLLLKTDPTERLTITQFMNHPWINQSMVVPQTPLHTARVLQEDKDHWDEVKEEMTS
ALATMRVDYDQV
000000000000000000000000000000000000000000000000000000000000000000000000000000000000000000000

000000000000000000100000000000000000000000000000000000000000000000000000000000001111
11111111111111111100000000000000000000000000011111111111111111111000000000000000000
00000000000000000000000000000000000000000000000000000000000000000000
>Q6TUJ5_4iu5_12_328
KKMSIVLAPFSGGQPHSGVELGPDYLLKQGLQQDMEKLGWDTRLERVFDGKVVEARKAS
DNGDRIGRVKRPRLTAECTEKIYKCVRRVAEQGRFPLTIGGDHSIALGTVAGVLSVHPDAGV
IWVDAHADINTMSGTVSGNLHGCPLSILLGLDRENIPECFSWVPQVLKPNKIAYIGLRAVD
DEEKKILHDLNIAAFSMHHVDRYGIDKVVSMAIEAVSPKGTEPVMVSYDVDTIDPLYVPAT
GTPVRGGLSFREALFLCERIAECGRLVALDVVECNPLLAATESHVNDTISVGCAIARCMMG
ETLLYTPHTSSKL
00000000000000000000000000000000000000000000000000000000000000000000000000000000000
00000000000000000000000000000000000000000000000000000000000000000000000000000000000
00000000000000000000000000000000000000000000000000000000000000000000000000000000000
00000000000000000000000000000000000000000000000000000000000001111111
>Q9L074_2x4l_32_348
GDGKSDGAGDGSGGAAKSGPWSFKDDRGTTVKLDKVPANIVAFTGVAAALFDYGVEVKG
VFGPTTTKDGKPDVQAGDLDVDKVTVLGNEWGKLNVEKYASLAPEVLITTTFDTAGTLWS
VPEESKDKVAKLAPSVAISVFDRQLTQPLQRMWELAESLGADMKAKKVTDAKAAFDKAA
ARLRAAAKAKPEIRVLAGSASPDLFYVSGTNLSVDLEYFKALGVNFVEPSEDAKKATGGWF
ESLSWENVDKYPADVIIMDDRASTIQPADITEGTWKQLPAVKAGQVIARSPEPILSYDKCTPL
LDNLAEAIENAKKVG
11111111111111111110000000000000000000000000000000000000000000000000000000000000000
00000000000000000000000000000000000000000000000000000000000000000000000000000000000
00000000000000000000000000000000000000000000000000000000000000000000000000000000000
00000000000000000000000000000000000000000000000000000000000000000000
>A7LR42_3p02_22_337
DEWTDEQFKQLISFKTQPGGWGVTDVHVRYANSAKYTYNLPVLVSGSTDNTDDRLVSFSLR
DDTLDILNFEKFGNRPELYFRELPQKYYSFPKELTIPAGQSHALLPIEFSLDGLDDSQKWALP
LKVCEDANGTYAVNPRKYYRTAVLRPILFNEFSGRFSGSSLLGTMAGESDIKFSSTEIKLNVV
TDSIVFFYAGQRTEDYEDRINYKVFLQFTGDKVDSKKDLYKMKIWAENEKLKFNSYSTPTYK
VSSEMDATKTYLKHTYIVISDIDFDFVDYTSVPNYEIEYNMKGGLSVSRDLDTRKPDEDQGS
DSKWW
11000000000000000000000000000000000000000000001111000000000000000000000000000000000
00000000000000000000000000000000000000000000000100000000000000000000000000000000000
00000000000000000000000000000000000000000000000000000000000000000000000000000000000
0000000000000000000000000000000000000000000000000000000000000000000
>O05302_3fsy_1_316
STVTGAAGIGLATLAADGSVLDTWFPAPELTESGTSATSRLAVSDVPVELAALIGRDDDRRT
ETIAVRTVIGSLDDVAADPYDAYLRLHLLSHRLVAPHGLNAGGLFGVLTNVVWTNHGPCAI
DGFEAVRARLRRRGPVTVYGVDKFPRMVDYVVPTGVRIADADRVRLGAHLAPGTTVMHE
GFVNYNAGTLGASMVEGRISAGVVVGDGSDVGGGASIMGTLSGGGTHVISIGKRCLLGAN
SGLGISLGDDCVVEAGLYVTAGTRVTMPDSNSVKARELSGSSNLLFRRNSVSGAVEVLARD
GQGIALNEDLHAN
11000000000000000000000000000000000000000000000000000000000000000000000000000000000
00000000000000000000000000000000000000000000000000000000000000000000000000000000000
00000000000000000000000000000000000000000000000000000000000110000000000000000000000
0000000000000000000000000000000000000000000000000000000000001111111
>O28344_4gvs_0_315
MLSVNEIAAEIVEDMLDYEEELRIESKKLENGAIVVDCGVNVPGSYDAGIMYTQVCMGGL
ADVDIVVDTINDVPFAFVTEYTDHPAIACLGSQKAGWQIKVDKYFAMGSGPARALALKPK
KTYERIEYEDDADVAVIALEANQLPDEKVMEFIAKECDVDPENVYALVAPTASIVGSVQISG

RIVETAIFKMNEIGYDPKLIVSGAGRCPISPILENDLKAMGSTNDSMMYYGSVFLTVKKYDEI
LKNVPSCTSRDYGKPFYEIFKAANYDFYKIDPNLFAPAQIAVNDLETGKTYVHGKLNAEVL
FQSYQIVLEE
00000000000000000000000000000000000000000000000000000000000000000000000000000000000
00000000000000000000000000000000000000000000000000000000000000000000000000000000000
00000000000000000000000000000000000000000000000000000000000000000000000000000000000
000000000000000000000000000000000000000000000000000000000000000001
>P39841_1qwr_0_315
MTQSPIFLTPVFKEKIWGGTALRDRFGYSIPSESTGECWAISAHPKGPSTVANGPYKGKTLIEL
WEEHREVFGGVEGDRFPLLTKLLDVKEDTSIKVHPDDYYAGENEEGELGKTECWYIIDCKE
NAEIIYGHTARSKTELVTMINSGDWEGLLRRIKIKPGDFYYVPSGTLHALCKGALVLETQQN
SDATYRVYDYDRLDSNGSPRELHFAKAVNAATVPHVDGYIDESTESRKGITIKTFVQGEYFS
VYKWDINGEAEMAQDESFLICSVIEGSGLLKYEDKTCPLKKGDHFILPAQMPDFTIKGTCTL
IVSHI
10000000000000000000000000000000000000000000000000000000000000000000000000000000000
00000000000000000000000000000000000000000000000000000000000000000000000000000000000
00000000000000000000000000000000000000000000000000000000000000000000000000000000000
000000000000000000000000000000000000000000000000000000000000000000
>P74102_3mg3_0_315
MPFTIDSARGIFPNTLAADVVPATIARFSQLNAEDQLALIWFAYLEMGKTLTIAAPGAASMQ
LAENALKEIQAMGPLQQTQAMCDLANRADTPLCRTYASWSPNIKLGFWYRLGELMEQGF
VAPIPAGYQLSANANAVLATIQGLESGQQITVLRNAVVDMGFTAGKDGKRIAEPVVPPQDT
ASRTKVSIEGVTNATVLNYMDNLNANDFDTLIELFTSDGALQPPFQRPIVGKENVLRFFREE
CQNLKLIPERGVTEPAEDGFTQIKVTGKVQTPWFGGNVGMNIAWRFLLNPEGKIFFVAIDL
LASPKELLNFA
11000000000000000000000000000000000000000000000000000000000000000000000000000000000
00000000000000000000000000000000000000000000000000000000000000000000000000000000011
11100000000000000000000000000000000000000000000000000000000000000000000000000000000
000000000000000000000000000000000000000000000000000000000000001111
>Q41736_1jb9_11_326
SVQQASRSKVSVAPLHLESAKEPPLNTYKPKEPFTATIVSVESLVGPKAPGETCHIVIDHGGN
VPYWEGQSYGVIPPGENPKKPGAPQNVRLYSIASTRYGDNFDGRTGSLCVRRAVYYDPETG
KEDPSKNGVCSNFLCNSKPGDKIQLTGPSGKIMLLPEEDPNATHIMIATGTGVAPFRGYLRR
MFMEDVPNYRFGGLAWLFLGVANSDSLLYDEEFTSYLKQYPDNFRYDKALSREQKNRSGG
KMYVQDKIEEYSDEIFKLLDGGAHIYFCGLKGMMPGIQDTLKKVAERRGESWDQKLAQLK
KNKQWHVEVY
11111000000000000000000000000000000000000000000000000000000000000000000000000000000
00000000000000000000000000000000000000000000000000000000000000000000000000000000000
00000000000000000000000000000000000000000000000000000000000000000000000000011110000
000000000000000000000000000000000000000000000000000000000000000000
>Q47Q21_2opj_1_316
TGRAFAIPLRTRFRGITVREGMLVRGAAGWGEFSPFAEYGPRECARWWAACYEAAELGWP
APVRDTVPVNATVPAVGPEEAARIVASSGCTTAKVKVAERGQSEANDVARVEAVRDALGP
RGRVRIDVNGAWDVDTAVRMIRLLDRFELEYVEQPCATVDELAEVRRRVSVPIAADESIRRA
EDPLRVRDAEAADVVVLKVQPLGGVRAALRLAEECGLPVVVSSAVETSVGLAAGVALAAA
LPELPYACGLATLRLLHADVCDDPLLPVHGVLPVRRVDVSEQRLAEVEIDPAAWQARLAA
ARAAWEQVEREPGP
00000000000000000000000000000000000000000000000000000000000000000000000000000000000
00000000000000001111110000000000000000000000000000000000000000000000000000000000000
00000000011111111111000000000000000000000000000000000000000000000000000000000000000
000000000000000000000000000000000000000000000000000000000001111111

>Q8A8E8_2gvk_0_315
MNPFQNSFGGHIPQDVAGKQGENVIFIVYNLTDSPDTVDKVKDVCANFSAMIRSMRNRFP
DMQFSCTMGFGADAWTRLFPDKGKPKELSTFSEIKGEKYTAVSTPGDLLFHIRAKQMGLCF
EFASILDEKLKGAVVSVDETHGFRYMDGKAIIGFVDGTENPAVDENPYHFAVIGEEDADFA
GGSYVFVQKYIHDMVAWNALPVEQQEKVIGRHKFNDVELSDEEKPGNAHNAVTNIGDDL
KIVRANMPFANTSKGEYGTYFIGYASTFSTTRRMLENMFIGSPAGNTDRLLDFSTAITGTLFF
VPSYDLLGELGE
1111111000000000000000000000000000000000000000000000000000000000000000000000000000
0000000000000000000000000000000000000000000000000000000000000000000000000000000000
0000000000000000000000000000000000000000000000000000000000000000000000000000000000
000000000000000000000000000000000000000000000000000000000000000000
>Q9A310_4hjf_239_554
AVSAEGGLDAPDAAELLRRAELAVEAAAAAGRGGAAAYGRAMETDGLSRLALEADLRG
AIGRGEITPYFQPIVRLSTGALSGFEALARWIHPRRGMLPPDEFLPLIEEMGLMSELGAHMM
HAAAQQLSTWRAAHPAMGNLTVSVNLSTGEIDRPGLVADVAETLRVNRLPRGALKLEVTE
SDIMRDPERAAVILKTLRDAGAGLALDDFGTGFSSLSYLTRLPFDTLKIDRYFVRTMGNNAG
SAKIVRSVVKLGQDLDLEVVAEGVENAEMAHALQSLGCDYGQGFGYAPALSPQEAEVYLN
EAYVDGAAPVKARG
1111111111111111111111111111111111111111111100000000000000000000000000000000000000
0000000000000000000000000000000000000000000000000000000000000000000000000000000000
0000000000000000000000000000000000000000000000000000000000000000000000000000000000
000000000000000000000000000000000000000000000000000000000011111111
>Q9AAP0_3hl1_0_315
MPPVWTLPRLYQHFQGAIDLELWTIPYYLTVLYSIKDPTTVPYRLIQAAVYQEMLHAQLVSN
IANAYGYSPTLSAPEYVGTAVPHIDFDLDTPNPTSIFTPYSAELGPLDLTRVNTMCLIEYPEWR
TQREPDLADDVTDYGSIGEFYDALRVGMEQLRGHVRGNQKQMDEFGPFYQNSPPLTVTES
GDAGFLQALTLVDIIVDQGEGQTQPVETIPTEFQNTADGFQDAWPHFQRFDFIRRMPNWP
GVYTGVTDPPAGSPGAEAQARLIADFAGFLDILNGMFSGGGAPPAFGVQMAKLGGDILSC
WKLGAVPRYS
0000000000000000000000000000000000000000000000000000000000000000000000000000000000
0000000000000000000000000000000000000000000000000000000000000000000000000000000000
0000111111000000000000000000000000000000011111111111111111111000000000000000000000
000000000000000000000000000000000000000000000000000000000000000000
>B3FK34_3r4v_0_314
MPVKVCLIFAGGTGMNVATKLVDLGEAVHCFDTCDKNVVDVHRSVNVTLTKGTRGAGG
NRKVILPLVRPQIPALMDTIPEADFYIVCYSLGGGSGSVLGPLITGQLADRKASFVSFVVGAM
ESTDNLGNDIDTMKTLEAIAVNKHLPIVVNYVPNTQGRSYESINDEIAEKIRKVVLLVNQN
HGRLDVHDVANWVRFTDKHNYLIPQVCELHIETTRKDAENVPEAISQLSLYLDPSKEVAFG
TPIYRKVGIMKVDDLDVTDDQIHFVINSVGVVEIMKTITDSKLEMTRQQSKFTQRNPIIDAD
DNVDEDGMVV
1000000000000000000000000000000000000000000000000011100000000000000000000000000000
0000000000000000000000000000000000000000000000000000000000000000000000000000000000
0000000000000000000000000000000000000000000000000000000000000000000000000000000000
00000000000000000000000000000000000000000000000000000000000000000
>O50082_1ixk_0_314
MKLSPSMLDKLLRLGYSKLFADRYFQLWGERAIRIAEAMEKPLPRCFRVNTLKISVQDLVKR
LNKKGFQFKRVPWAKEGFCLTREPFSITSTPEFLTGLIYIQEASSMYPPVALDPKPGEIVADM
AAAPGGKTSYLAQLMRNDGVIYAFDVDENRLRETRLNLSRLGVLNVILFHSSSLHIGELNV
EFDKILLDAPCTGSGTIHKNPERKWNRTMDDIKFCQGLQMRLLEKGLEVLKPGGILVYSTC
SLEPEENEFVIQWALDNFDVELLPLKYGEPALTNPFGIELSEEIKNARRLYPDVHETSGFFIAK
IRKL

11000000000000000000000000000000000000000000000000000000000000000000000000000000000
00000000000000000000000000000000000000000000000000000000000000000000000000000000000
00000000000000000000000000000000000000111111100000000000000000000000000000000000000
000000000000000000000000000000000000000000000000000000000000000000
>O53831_1ozp_0_314
MTALDWRSALTADEQRSVRALVTATTAVDGVAPVGEQVLRELGQQRTEHLLVAGSRPGGPI
IGYLNLSPPRGAGGAMAELVVHPQSRRRGIGTAMARAALAKTAGRNQFWAHGTLDPARA
TASALGLVGVRELIQMRRPLRDIPEPTIPDGVVIRTYAGTSDDAELLRVNNAAFAGHPEQGG
WTAVQLAERRGEAWFDPDGLILAFGDSPRERPGRLLGFHWTKVHPDHPGLGEVYVLGVD
PAAQRRGLGQMLTSIGIVSLARRLGGRKTLDPAVEPAVLLYVESDNVAAVRTYQSLGFTTYS
VDTAYALAGTDN
11000000000000000000000000000000000000000000000000000000000000000000001110000000000
00000000000000000000000000000000000000000000000000000000000000000000000000000000000
00000000000000000000000000000000000000000011111100000000000000000000000000000000000
000000000000000001111111110000000000000000000000000000000000011111
>P22290_3rrc_210_524
NTVMKNCNYKRKRRERDWDCNTKKDVCIPDRRYQLCMKELTNLVNNTDTNFHRDITFRK
LYLKRKLIYDAAVEGDLLLKLNNYRYNKDFCKDIRWSLGDFGDIIMGTDMEGIGYSKVVEN
NLRSIFGTDEKAQQRRKQWWNESKAQIWTAMMYSVKKRLKGNFIWICKLNVAVNIEPQIY
RWIREWGRDYVSELPTEVQKLKEKCDGKINYTDKKVCKVPPCQNACKSYDQWITRKKNQ
WDVLSNKFISVKNAEKVQTAGIVTPYDILKQELDEFNEVAFENEINKRDGAYIELCVCSVEE
AKKNTQEVVTNVDN
00000000000000000000000000000000000000000001111111111110000000000000000000000000000
00000000000000000000000000000000000000000000000000000000000000000000000000000000000
00000000000000000000000000000000000000000000000000000000000000000000000000000000000
000000000000000000000000000000000000000000000000011111111111111111
>Q12923_1wch_2162_2476
HEDSDKDHSFLTNDELAVLPVVKVLPSGKYTGANLKSVIRVLRGLLDQGIPSKELENLQELK
PLDQCLIGQTKENRRKNRYKNILPYDATRVPLGDEGGYINASFIKIPVGKEEFVYIACQGPLP
TTVGDFWQMIWEQKSTVIAMMTQEVEGEKIKCQRYWPNILGKTTMVSNRLRLALVRMQQ
LKGFVVRAMTLEDIQTREVRHISHLNFTAWPDHDTPSQPDDLLTFISYMRHIHRSGPIITHCS
AGIGRSGTLICIDVVLGLISQDLDFDISDLVRCMRLQRHGMVQTEDQYIFCYQVILYVLTRLQ
AEEEQ
11111110000000000000000000000000000000000000000000000000000000000000000000000000000
00000000000000000000000000000000000000000000000000000000000000000000000000000000000
00000000000000000000000000000000000000000000000000000000000000000000000000000000000
000000000000000000000000000000000000000000000000000000000000000000
>Q4QH86_2b4w_0_314
MKQVKAAFEANKRVYESVLLTFKGVDGYDVYNCSVPFSYKGKTHIYGRVEKRDIWAASHV
RLFEETGKDEFTAVPELSWELEDPYIAKINNEMIFGGTRVRKNGNAILSYYGYFYRGTPDELT
YFTRGPGCMKDIRVLQLQDGRLGVFSRPRVGRKASIGFVILNSIDELGAEVIAKAPPLDILSE
NAWGGVNQAYLLSSGKVGCIGHYSYEDTNEKQQPQSVYVNYAFVLDPQSRAITGAKIIGTK
SCYPPCEPKVPLLADCVFASGIVMRSDGKVDLYSGVGDSHEGRITIDYPFKGHGTIIGDLHFP
MASSL
10000000000000000000000000000000000000000000000000000000000000000000000000000000000
00000000000000000011111000000000000000000000000000000000000000000000001110000000000
00000000000000000011000000000000000000000000001111111000000000000000000000000000000
00000000000000000000000000000000000000000000000000000000000000001111
>Q9KVU4_3q0i_0_314
MSQSLRIVFAGTPDFAARHLAALLSSEHEIIAVYTQPERPAGRGKKLTASPVKTLALEHNVP
VYQPENFKSDESKQQLAALNADLMVVVAYGLLLPKVVLDTPKLGCINVHGSILPRWRGAA

PIQRSIWAGDSETGVTIMQMDVGLDTGDMLKIATLPIEASDTSASMYDKLAELGPQALLEC
LQDIAQGTAVAVKQDDGLANYAHKLSKEEARINWSDAATHIERCIRAFNPWPMSHFEVAE
NSIKVWQARVETRAVTQTPGTIIQADKSGIYVATGQDVLVLESLQIPGKKALPVQDILNARA
DWFSVGSQLS
11100000000000000000000000000000000000111111111100000000000000000000000000000000000000
00000000000000000000000000000000000000000000000000000000000000000000000000000000000
00000000000000000000000000000000000000000000000000000000000000000000000000000000000
00000000000000000000000000000000000000000000000000000000000000000
>P21163_1pgs_40_353
APADNTVNIKTFDKVKNAFGDGLSQSAEGTFTFPADVTTVKTIKMFIKNECPNKTCDEWD
RYANVYVKNKTTGEWYEIGRFITPYWVGTEKLPRGLEIDVTDFKSLLSGNTELKIYTETWLA
KGREYSVDFDIVYGTPDYKYSAVVPVIQYNKSSIDGVPYGKAHTLGLKKNIQLPTNTEKAYL
RTTISGWGHAKPYDAGSRGCAEWCFRTHTIAINNANTFQHQLGALGCSANPINNQSPGN
WAPDRAGWCPGMAVPTRIDVLNNSLTGSTFSYEYKFQSWTNNGTNGDAFYAISSFVIAKSN
TPISAPVVTN
11100000000000000000000000000000000000000000000000000000000000000000000000000000000
00000000000000000000000000000000000000000000000000000000000000000000000000000000000
00000000000000000000000000000000000000000000000000000000000000000000000000000000000
0000000000000000000000000000000000000000000000000000000000000000
>P32989_2h7g_0_313
MRALFYKDGKLFTDNNFLNPVSDNNPAYEVLQHVKIPTHLTDVVVYGQTWEEALTRLIFV
GSDSKGRRQYFYGKMHVQNRNAKRDRIFVRVYNVMKRINCFINKNIKKSSTDSNYQLAVF
MLMETMFFIRFGKMKYLKENETVGLLTLKNKHIEISPDKIVIKFVGKDKVSHEFVVHKSNRL
YKPLLKLTDDSSPEEFLFNKLSERKVYECIKQFGIRIKDLRTYGVNYTFLYNFWTNVKSISPLP
SPKKLIALTIKQTAEVVGHTPSISKRAYMATTILEMVKDKNFLDVVSKTTFDEFLSIVVDHVK
SSTDG
00000000000000000000000000000000000000000000000000000000000000000000000000000000000
00000000000000000000000000000000000000000000000000000000000000000000000000000000000
00000000000000000000000000000000000000000000000000000000000000000000000000000000000
0000000000000000000000000000000000000000000000000000000000000011
>P44741_3m72_14_327
MNITKPFPLPTGYFGIPLGLAALSLAWFHLENLFPAARMVSDVLGIVASAVWILFILMYAYKL
RYYFEEVRAEYHSPVRFSFIALIPITTMLVGDILYRWNPLIAEVLIWIGTIGQLLFSTLRVSELW
QGGVFEQKSTHPSFYLPAVAANFTSASSLALLGYHDLGYLFFGAGMIAWIIFEPVLLQHLRIS
SLEPQFRATMGIVLAPAFVCVSAYLSINHGEVDTLAKILWGYGFLQLFFLLRLFPWIVEKGLN
IGLWAFSFGLASMANSATAFYHGNVLQGVSIFAFVFSNVMIGLLVLMTIYKLTKGQFFLK
11111000000000000000000000000000000000000000000000000000000000000000000000000000000
00000000000000000000000000000000000000000000000000000000000000000000000000000000000
00000000000000000000000000000000000000000000000000000000000000000000000000000000000
0000000000000000000000000000000000000000000000000000000000000111
>P60340_1r3f_0_313
MSRPRRRGRDINGVLLLDKPQGMSSNDALQKVKRIYNANRAGHTGALDPLATGMLPICL
GEATKFSQYLLDSDKRYRVIARLGQRTDTSDADGQIVEERPVTFSAEQLAAALDTFRGDIEQ
IPSMYSALKYQGKKLYEYARQGIEVPREARPITVYELLFIRHEGNELELEIHCSKGTYIRTIIDD
LGEKLGCGAHVIYLRRLAVSKYPVERMVTLEHLRELVEQAEQQDIPAAELLDPLLMPMDSP
ASDYPVVNLPLTSSVYFKNGNPVRTSGAPLEGLVRVTEGENGKFIGMGEIDDEGRVAPRRLV
VEYPA
11111110000000000000000000000000000000000000000000000000000000000000000000000000000
00000000000000000000000000000000000000001111111111111111111111111111100000000000000
00000000000000000000000000000000000000000000000000000000000000000000000000000000000
0000000000000000000000000000000000000000000000000000000000000000

>P70080_3e2t_100_413
GDMENIPWYPKKISDLDKCANRVLMYGSDLDADHPGFKDNVYRKRRKYFADLAMNYKH
GDPIPEIEFTEEEIKTWGTVYRELNKLYPTHACREYLKNLPLLTKYCGYREDNIPQLEDVSRF
LKERTGFTIRPVAGYLSPRDFLAGLAFRVFHCTQYVRHSSDPLYTPEPDTCHELLGHVPLLAE
PSFAQFSQEIGLASLGASDEAVQKLATCYFFTVEFGLCKQEGQLRVYGAGLLSSISELKHSLS
GSAKVKPFDPKVTCKQECLITTFQEVYFVSESFEEAKEKMREFAKTIKRPFGVKYNPYTQSV
QILKD
11110000000000000000000000000000000000000000000000000000000000000000000000000000000
00000000000000000000000000000000000000000000000000000000000000000000000000000000000
00000000000000000000000000000000000000000000000000000000000000000000000000000000000
0000000000000000000000000000000000000000000000000000000000000111
>Q11YA9_3mdq_0_313
MSQRIGVIDMGTNTFHLLITDIVNDRPHTLVNEKSAVGLGKGGITKGFITEEAMDRALDTLK
KFRVILDEHAVVHVIATGTSAVRSGSNKQVLIDRIKKEVNIDVEVIDGAREAELIFRGVQQAV
PMEDHISLAMDIGGGSVEFIIGNKNEILWKQSFEIGGQRLIDRFHVHDPMREDDRVMMHN
YFDEVLVPLEKAINTWRPTQLIGCSGTFDTLAEMNIQHHREKIALEKQTSYLLSLPDFNRLR
KQLVASTRRERLAIAGMIELRADMVVVAICLIEHVLKLVSTNAITVSTYSLKEGVLYTMLDGV
KVGS
11000000000000000000000000000000000000000000000000000000000000000000000000000000000
00000000000000000000000000000000000000000000000000000000000000000000000000000000000
00000000000000000000000000000000000000000000000000000000000000000000000000000000000
0000000000000000000000000000000000000000000000000000000000000000
>Q2FG38_2f9i_0_313
MLDFEKPLFEIRNKIESLKESQDKNDVDLQEEIDMLEASLERETKKIYTNLKPWDRVQIARL
QERPTTLDYIPYIFDSFMELHGDRNFRDDPAMIGGIGFLNGRAVTVIGQQRGKDTKDNIYR
NFGMAHPEGYRKALRLMKQAEKFNRPIFTFIDTKGAYPGKAAEERGQSESIATNLIEMASL
KVPVIAIVIGEGGSGGALGIGIANKVLMLENSTYSVISPEGAAALLWKDSNLAKIAAETMKI
TAHDIKQLGIIDDVISEPLGGAHKDIEQQALAIKSAFVAQLDSLESLSRDEIANDRFEKFRNIG
SYIE
00000000000000000011111111111000000000000000000000000000000000000000000000000000000
00000000000000000000000000000000000000000000000000000000000000000000000000000000000
00000000000000000000000000000000000000000000000000000000000000000000000000000000000
0000000000000000000000000000000000000000000000000000000000000000
>Q7SIG4_3hli_0_313
MEIPVIEPLFTKVTEDIPGAEGPVFDKNGDFYIVAPEVEVNGKPAGEILRIDLKTGKKTVICKP
EVNGYGGIPAGCQCDRDANQLFVADMRLGLLVVQTDGTFEEIAKKDSEGRRMQGCNDCA
FDYEGNLWITAPAGEVAPADYTRSMQEKFGSIYCFTTDGQMIQVDTAFQFPNGIAVRHMN
DGRPYQLIVAETPTKKLWSYDIKGPAKIENKKVWGHIPGTHEGGADGMDFDEDNNLLVAN
WGSSHIEVFGPDGGQPKMRIRCPFEKPSNLHFKPQTKTIFVTEHENNAVWKFEWQRNGKK
QYCETLKFGIF
11000000000000000000000000000000000000000000000000000000000000000000000000000000000
00000000000000000000000000000000000000000000000000000000000000000000000000000000000
00000000000000000000000000000000000000000000000000000000000000000000000000000000000
0000000000000000000000000000000000000000000000000000000000000000
>Q8EED0_3goh_0_313
MEQHQVWAYQTKTHSVTLNSVDIPALAADDILVQNQAIGINPVDWKFIKANPINWSNGH
VPGVDGAGVIVKVGAKVDSKMLGRRVAYHTSLKRHGSFAEFTVLNTDRVMTLPDNLSFER
AAALPCPLLTAWQAFEKIPLTKQREVLIVGFGAVNNLLTQMLNNAGYVVDLVSASLSQAL
AAKRGVRHLYREPSQVTQKYFAIFDAVNSQNAAALVPSLKANGHIICIQDRIPAPIDPAFTRT
ISYHEIALGALHDFGDRQDWQILMQQGEALLTLIAQGKMEIAAPDIFRFEQMIEALDHSEQ
TKLKTVLTLNE

00000000000000000000000000000000000000000000000000000000000000000000000000000000000
00000000000000000000000000000000000000000000000000000000000000000000000000000000000
00000000000000000000000000000000000000111111000000000000000000001111111111000000000
00000000000000000000000000000000000000000000000000000000000000000
>Q8NMJ3_3sno_0_313
MALEPQIKSAPTPVILIVEPYGGSIRQQNPNLPMVFWDDAALTRGDGIFETLLIRDGHACNV
RRHGERFKASAALLGLPEPILEDWEKATQMGIESWYSHPNAGEASCTWTLSRGRSSTGLAS
GWLTITPVSSDKLAQREHGVSVMTSSRGYSIDTGLPGIGKATRGELSKVERTPAPWLTVGAK
TLAYAANMAALRYAKSNGFDDVIFTDGDRVLEGATSTVVSFKGDKIRTPSPGGDILPGTTQA
ALFAHATEKGWRCKEKDLSIDDLFGADSVWLVSSVRGPVRVTRLDGHKLRKPDNEKEIKA
LITKALG
11111111100000000000000000000000000000000000000000000000000000000000000000000000000
00000000000000000010000000000000000000000000000000000000000000000001111111111111111
11111111111111111111111111111111111110000000000000000000000000000000011111100000000
00000000000000000000000000000000000000000000000000000000000000000
>Q8NR27_3gjy_0_313
MARKKNTSDQSRSQAANTPIAGTYEGEYSVIELEADSYTTDGWLISINGVPSSHIVLGQPQA
LEFEYMRWIATGARAFIDAHQDASKLRITHLGGGACTMARYFADVYPQSRNTVVELDAEL
ARLSREWFDIPRAPRVKIRVDDARMVAESFTPASRDVIIRDVFAGAITPQNFTTVEFFEHCHR
GLAPGGLYVANCGDHSDLRGAKSELAGMMEVFEHVAVIADPPMLKGRRYGNIILMGSDTE
FFSSNSTEASAITRELLGGGVPAQYKDESWVRKFASGAQARHDGVSTLQMPSDTPQHPAET
PEHSNTQP
11111111111111111000000000000000000000000000000000000000000000000000000000000000000
00000000000000000000000000000000000000000000000000000000000000000000000000000000000
00000000000000000000000000000000000000000000000000000000000000000000000000000000000
00000000000000000000000000000000000000000000000111111111111111111
>Q9H0W9_1xcr_1_314
ACAEFSFHVPSLEELAGVMQKGLKDNFADVQVSVVDCPDLTKEPFTFPVKGICGKTRIAEV
GGVPYLLPLVNQKKVYDLNKIAKEIKLPGAFILGAGAGPFQTLGFNSEFMPVIQTESEHKPP
VNGSYFAHVNPADGGCLLEKYSEKCHDFQCALLANLFASEGQPGKVIEVKAKRRTGPLNF
VTCMRETLEKHYGNKPIGMGGTFIIQKGKVKSHIMPAEFSSCPLNSDEEVNKWLHFYEMK
APLVCLPVFVSRDPGFDLRLEHTHFFSRHGEGGHYHYDTTPDIVEYLGYFLPAEFLYRIDQPK
ETHSIGRD
10000000000000000000000000000000000000000000000000000000000000000000000000000000000
00000000000000000000000000000000000000000000000000000000000000000000000000000000000
00000000000000000000000000000000000000000000000000000000000000000000000000000000000
00000000000000000000000000000000000000000000000000000000000000000
>Q9HZF5_4f0j_21_334
SQAPVYGERLEGFDYAYPVHYLDFTSQGQPLSMAYLDVAPKKANGRTILLMHGKNFCAGT
WERTIDVLADAGYRVIAVDQVGFCKSSKPAHYQYSFQQLAANTHALLERLGVARASVIGH
SMGGMLATRYALLYPRQVERLVLVNPIGLEDWKALGVPWRSVDDWYRRDLQTSAEGIRQY
QQATYYAGEWRPEFDRWVQMQAGMYRGKGRESVAWNSALTYDMIFTQPVVYELDRLQM
PTLLLIGEKDNTAIGKDAAPAELKARLGNYAQLGKDAARRIPQATLVEFPDLGHTPQIQAP
ERFHQALLEGLQTQP
00000000000000000000000000000000000000000000000000000000000000000000000000000000000
00000000000000000000000000000000000000000000000000000000000000000000000000000000000
00000000000000000000000000000000000000000000000000000000000000000000000000000000000
00000000000000000000000000000000000000000000000000000000000000011
>Q9S1H5_1ko7_0_313
MLTTKSLVERFELEMIAGEAGLNKQIKNTDISRPGLEMAGYFSHYASDRIQLLGTTELSFYNL
LPDEERKGRMRKLCRPETPAIIVTRDLEPPEELIEAAKEHETPLITSKIATTQLMSRLTTFLEHE

LARTTSLHGVLVDVYGVGVLITGDSGIGKSETALELIKRGHRLVADDNVEIREISKDELIGRA
PKLIEHLLEIRGLGIINVMTLFGAGSILTEKRLRLNIHLENWHKEKLYDRVGLNEETLRILDTE
ITKKTIPVRPGRNVAVIIEVAAMNYRLNIMGINTAEEFNDRLNAEILRNGNNGNNGEEK
00000000000000000000000000000000000000000000000000000000000000000000000000000000
00000000000000000000000000000000000000000000000000000000000000000000000000000000
00000000000000000000000000000000000000000000000000000000000000011111111111110000
0000000000000000000000000000000000000000000000001111111111111111
>B1YSS6_4eg0_0_312
MSGIDPKRFGKVAVLFGGESAEREVSLTSGRLVLQGLRDAGIDAHPFDPAERPLSALKDEGF
VRAFNALHGGYGENGQIQGALDFYGIRYTGSGVLGSALGLDKFRTKLVWQQTGVPTPPFE
TVMRGDDYAARATDIVAKLGLPLFVKPASEGSSVAVLKVKTADALPAALSEAATHDKIVIVE
KSIEGGGEYTACIAGDLDLPLIKIVPAGEFYDYHAKYVANDTQYLIPCGLPAEQETELKRIAR
RAFDVLGCTDWGRADFMLDAAGNAYFLEVNTAPGMTDHSLPPKAARSIGIGYSELVVKVL
SLTLND
11100000000000000000000000000000000000000000000000000000000000000000000000000000
00000000000000000000000000000000000000000000000000000000000000000111110000000000
00000000000000000000000000000000000000000000001111111110000000000000000000000000
0000000000000000000000000000000000000000000000000000000000011
>B5XKN1_4es8_19_331
NGVMVVKAAEAEELPDDLMNFKGTWEVSADGSSGRFFSKGATDSYVFHLIPAKDVKKPG
WREHNEVKDSYIKIDKQSIAARYKTSTTAPYSVAFKVNTKSLIKDHDYKITFEQGQIASGITV
DYRIGSAFNKTTDDSFKISDESKYASNVKIEGEEQGFKQREQGDKTISFRTLKEGPMSLVLVS
KVDEKTQGDLDVEFKNFKIIDVTNPSQLDKGVAYVGNKNVQLTLKSDDGRTNFEGDEISLF
NSRGELLQTVTVTKDQQNPISITLSEDQAKSLKNKEKLKVSIKQKQSKKTSKDFFFEVGIDPK
VEAK
11111111111100000000000000000000000000000000000000000000000000000000000000000000
00000000000000000000000000000000000000000000000000000000000000000000000000000000
00000000000000000000000000000000000000000000000000000000000000000000000000000000
00000000000000000000000000000000000000000000000000000000000000
>P09889_1ute_27_339
TAPTPILRFVAVGDWGGVPNAPFHTAREMANAKAIATTVKTLGADFILSLGDNFYFTGVHD
AKDKRFQETFEDVFSDPSLRNVPWHVLAGNHDHLGNVSAQIAYSKISKRWNFPSPYYRLRF
KIPRSNVSVAIFMLDTVTLCGNSDDFVSQQPERPRNLALARTQLAWIKKQLAAAKEDYVLV
AGHYPVWSIAEHGPTHCLVKQLLPLLTTHKVTAYLCGHDHNLQYLQDENGLGFVLSGAG
NFMDPSKKHLRKVPNGYLRFHFGAENSLGGFAYVEITPKEMSVTYIEASGKSLFKTKLPRRA
RSEHQHRRA
11000000000000000000000000000000000000000000000000000000000000000000000000000000
00000000000000000000000000000000000000000000000000000000000000000000000000000000
00000000000000000000000000000000000000000000000000000000000000000000000000000000
0000000000000000000000000000000000000000000000000000111111111
>P11309_2bzh_91_403
MLLSKINSLAHLRAAPCNDLHATKLAPGKEKEPLESQYQVGPLLGSGGFGSVYSGIRVSDN
LPVAIKHVEKDRISDWGELPNGTRVPMEVVLLKKVSSGFSGVIRLLDWFERPDSFVLILERPE
PVQDLFDFITERGALQEELARSFFWQVLEAVRHCHNCGVLHRDIKDENILIDLNRGELKLID
FGSGALLKDTVYTDFDGTRVYSPPEWIRYHRYHGRSAAVWSLGILLYDMVCGDIPFEHDEEI
IRGQVFFRQRVSSECQHLIRWCLALRPSDRPTFEEIQNHPWMQDVLLPQETAEIHLHSLSPG
PSK
11111111111111111111111111111100000000000000000000000000000000000000000000000000
00000000000000000000000000000000000000000000000000000000000000000000000000000000
00000000000000000000000000000000000000000000000000000000000000000000000000000000
0000000000000000000000000000000000000000000000000000001111111

>Q73WB6_3e4w_0_312
MSGGLTPDQAIDAIRGTGGAQPGCRALHAKGTLYRGTFTATRDAVMLSAAPHLDGSTVPA
LIRFSNGSGNPKQRDGAPGVRGMAVKFTLPDGSTTDVSAQTARLLVSSTPEGFIDLLKAMRP
GLTTPLRLATHLLTHPRLLGALPLLREANRIPASYATTEYHGLHAFRWIAADGSARFVRYHL
VPTAAEEYLSASDARGKDPDFLTDELAARLQDGPVRFDFRVQIAGPTDSTVDPSSAWQSTQI
VTVGTVTITGPDTEREHGGDIVVFDPMRVTDGIEPSDDPVLRFRTLVYSASVKLRTGVDRGA
QAPPV
11100000000000000000000000000000000000000000000000000000000000000000000000000000000
00000000000000000000000000000000000000000000000000000000000000000000000000000000000
00000000000000000000000000000000000000000000000000000000000000000000000000000000000
0000000000000000000000000000000000000000000000000000000001111111
>Q87P32_3let_74_386
AITKAVFDNEQGQAQRLQTSSSVEHGQMLFKDANLKTPSDVLNAFAKLDSKMVKSHAAE
LSQLAERAMTEVMLETDSGKNLKALIGDDAVKSLAVRVVKDYGGGVAAAQKNPEVRINQ
MQAVFDMEVMHLKAAQRHIEGLASTDLNQGVYAEGLPEDAFNKAGVTNNVERAAAWII
NASNSKGNDAENITSLLKEYATNGKDLLNMDNLKELHARLVPNVERDYRGPNISGGTLPSS
IGGEGMLKQHIEGFLKENPVADKDLGKHLFAGVIGYHGFTDGNGRMGRMLYAIAELRNDS
FNPLAMNAENSLHGIK
11000000000000000000000000000000000000000000000000000000000000000000000000000000000
00000000000000000000000000000000000000000000000000000000000000000000000000000000000
00000000000000000000000000000000000000000000000000000000000000000000000000000000000
0000000000000000000000000000000000000000000000000000000000000000
>Q9X1N9_1vrm_39_351
RTKDQYYELRDFALGTSVRIVVSSQKINPRTIAEAILEDMKRITYKFSFTDERSVVKKINDHP
NEWVEVDEETYSLIKAACAFAELTDGAFDPTVGRLLELWGFTGNYENLRVPSREEIEEALK
HTGYKNVLFDDKNMRVMVKNGVKIDLGGIAKGYALDRARQIALSFDENATGFVEAGGDV
RIIGPKFGKYPWVIGVKDPRGDDVIDYIYLKSGAVATSGDYERYFVVDGVRYHHILDPSTGYP
ARGVWSVTIIAEDATTADALSTAGFVMAGKDWRKVVLDFPNMGAHLLIVLEGGAIERSET
FKLFERE
11110000000000000000000000000000000000000000000000000000000000000000000000000000000
00000000000000000000000000000000000000000000000000000000000000000000000000000000000
00000000000000000000000000000000000000000000000000000000000000000000000000000000000
0000000000000000000000000000000000000000000000000000000000000000
>A0QS86_3njd_0_311
MTHAIRPVDFDNLKTMTYEVTDRVARITFNRPEKGNAIVADTPLELSALVERADLDPDVHV
ILVSGRGEGFCAGFDLSAYAEGSSSAGGGSPYEGTVLSGKTQALNHLPDEPWDPMVDYQM
MSRFVRGFASLMHCDKPTVVKIHGYCVAGGTDIALHADQVIAAADAKIGYPPMRVWGVP
AAGLWAHRLGDQRAKRLLFTGDCITGAQAAEWGLAVEAPDPADLDARTERLVERIAAMP
VNQLIMAKLACNTALLNQGVATSQMVSTVFDGIARHTPEGHAFVATAREHGFREAVRRRD
EPMGDHGRRASDV
11111100000000000000000000000000000000000000000000000000000000000000000000000111111
11111111000000000000000000000000000000000000000000000000000000000000000000000000000
00000000000000000000000000000000000000000000000000000000000000000000000000000000000
000000000000000000000000000000000000000000000000000000000000000
>P10930_1h6w_84_395
LSYPNATETVYGLTRYSTNDEAIAGVNNESSITPAKFTVALNNAFETRVSTESSNGVIKISSLP
QALAGADDTTAMTPLKTQQLAIKLIAQIAPSETTATESDQGVVQLATVAQVRQGTLREGYA
ISPYTFMNSSSTEEYKGVIKLGTQSEVNSNNASVAVTGATLNGRGSTTSMRGVVKLTTTAGS
QSGGDASSALAWNADVIQQRGGQIIYGTLRIEDTFTIANGGANITGTVRMTGGYIQGNRIV
TQNEIDRTIPVGAIMMWAADSLPSDAWRFCHGGTVSASDCPLYASRIGTRYGGNPSNPGLP
DMR

11111111111111111111111111111111111111111111111111111111111111111111111111111111111
11111111111111111111111111111111111111111111111111111111111111111111111111111100000
00000000000000000000000000000000000000000000000000000000000000000000000000000000000
00000000000000000000000000000000000000000000000000000000000000
>Q12125_2wpv_0_311
MVPAESNAVQAKLAKTLQRFENKIKAGDYYEAHQTLRTIANRYVRSKSYEHAIELISQGAL
SFLKAKQGGSGTDLIFYLLEVYDLAEVKVDDISVARLVRLIAELDPSEPNLKDVITGMNNWS
IKFSEYKFGDPYLHNTIGSKLLEGDFVYEAERYFMLGTHDSMIKYVDLLWDWLCQVDDIED
STVAEFFSRLVFNYLFISNISFAHESKDIFLERFIEKFHPKYEKIDKNGYEIVFFEDYSDLNFLQL
LLITCQTKDKSYFLNLKNHYLDFSQAYKSELEFLGQEYFNIVAPKQTNFLQDMMSGFLGGS
K
11111111111000000000000000000000000000000000000000000000000000000000000000000000000
00000000000000000000000000000000000000000000000000000000000000000000000000000000000
00000000000000000000000000000000000000000000000000000000000000000000000000000000000
00000000000000000000000000000000000000000011111111111111111111
>Q3JR17_3tk8_0_311
MRRRRAAHRRFLPRPRPLNARRNAPIHSSSTSSIATNMSQQLQQIIDNAWENRAELSPKAA
SAEIREAVAHAIEQLDRGALRVAEKIDGAWTVHQWLKKAVLLSFRLEDNAPMPAGGYSQF
YDKVPSKFANYTAEDFAAGGFRVVPPAIARRGSFIAKNVVLMPSYTNIGAYVDEGTMVDT
WATVGSCAQIGKNVHLSGGVGIGGVLEPLQANPVIIEDNCFIGARSEVVEGVIVEENSVISM
GVYLGQSTKIYDRETGEVTYGRIPAGSVVVAGNLPAKDGTHSLYCAVIVKKVDAKTRAKVG
LNELLRGD
11111111111111111111111111111111111111000000000000000000000000000000000000000000000
00000000000000000000000000000000000000000000000000000000000000000000000000000000000
00000000000000000000000000000000000000000000000000000000000000000000000000000000000
00000000000000000000000000000000000000000000001111111111111111
>Q52060_1nvm_0_311
MNQKLKVAIIGSGNIGTDLMIKVLRNAKYLEMGAMVGIDAASDGLARAQRMGVTTTYAG
VEGLIKLPEFADIDFVFDATSASAHVQNEALLRQAKPGIRLIDLTPAAIGPYCVPVVNLEEHL
GKLNVNMVTCGGQATIPMVAAVSRVAKVHYAEIVASISSKSAGPGTRANIDEFTETTSKAIE
VIGGAAKGKAIIIMNPAEPPLIMRDTVYVLSAAADQAAVAASVAEMVQAVQAYVPGYRLK
QQVQFDVIPESAPLNIPGLGRFSGLKTSVFLEVEGAAHYLPAYAGNLDIMTSAALATAERMA
QSMLNA
11000000000000000000000000000000000000000000000000000000000000000000000000000000000
00000000000000000000000000000000000000000000000000000000000000000000000000000000000
00000000000000000000000000000000000000000000000000000000000000000000000000000000000
00000000000000000000000000000000000000000000000000000000000000
>O75962_1nty_1283_1593
EEKRKSARRKEFIMAELIQTEKAYVRDLRECMDTYLWEMTSGVEEIPPGIVNKELIIFGNMQ
EIYEFHNNIFLKELEKYEQLPEDVGHCFVTWADKFQMYVTYCKNKPDSTQLILEHAGSYFD
EIQQRHGLANSISSYLIKPVQRITKYQLLLKELLTCCEEGKGEIKDGLEVMLSVPKRANDAM
HLSMLEGFDENIESQGELILQESFQVWDPKTLIRKGRERHLFLFEMSLVFSKEVKDSSGRSKY
LYKSKLFTSELGVTEHVEGDPCKFALWVGRTPTSDNKIVLKASSIENKQDWIKHIREVIQERT
11111100000000000000000000000000000000000000000000000000000000000000000000000000000
00000000000000000000000000000000000000000000000000000000000000000000000000000000000
00000000000000000000000000000000000000000000000000000000000000000000000000000000000
0000000000000000000000000000000000000000000000000000000000000
>P33652_2wuq_1_311
LNSESLLRELRDALHEGGLTGSFLVRDLYTGEELGIDPDTELPTASLVKLPLALATLERIRLGE
VDGAQQIEVAPGRITTPGPTGLSRFRHPARVAVDDLLYLSTSVSDGTASDALFEITPPAQVEQ
MVREWGFRDLTVRHSMRELSETPAERFESADAHLAHALAISAGTSGRGHRVPQLDVARAN

TGTARAFVDLLEALWAPVLTGPRPGRTSRALPPEPAARLRELMAANLLRHRLAPDFASDAA
TWSSKTGTLLNLRHEVGVVEHADGQVFAVAVLTESQVPADSQPGAEALMAQVARRLRDRL
REW
00000000000000000000000000000000000000000000000000000000000000000000000000000000000
00000000000000000000000000000000000000000000000000000000000000011111111111111111111
11100000000000000000000000000000000000000111111000000000000000000000000000000000000
00000000000000000000000000000000000000000000000000000000000000
>Q05490_2es4_41_351
PAAPSPAPAGAVAGGPAAGVPAAASGAAEAAMPLPAALPGALAGSHAPRLPLAAGGRLA
RTRAVREFFDYCLTAQGELTPAALDALVRREIAAQLDGSPAQAEALGVWRRYRAYFDALA
QLPGDGAVLGDKLDPAAMQLALDQRAALADRTLGEWAEPFFGDEQRRQRHDLERIRIAN
DTTLSPEQKAARLAALDAQLTPDERAQQAALHAQQDAVTKIADLQKAGATPDQMRAQI
AQTLGPEAAARAAQMQQDDEAWQTRYQAYAAERDRIAAQGLAPQDRDARIAQLRQQTF
TAPGEAIRAASLDRGAG
11111111111111111111111111111110000000000000000000000000000000000000000000000000000
00000000000000000000000000000000000000000000000000000000000000000000000000000000000
00000000000000000000000000000000000000000000000000000000000000000000000000000000000
00000000000000000000000000000000000000000000000000000000000000
>Q071N0_3o26_0_310
MPETCPNTVTKRRCAVVTGGNKGIGFEICKQLSSNGIMVVLTCRDVTKGHEAVEKLKNSN
HENVVFHQLDVTDPIATMSSLADFIKTHFGKLDILVNNAGVAGFSVDADRFKAMISDIGED
SEELVKIYEKPEAQELMSETYELAEECLKINYNGVKSVTEVLIPLLQLSDSPRIVNVSSSTGSLK
YVSNETALEILGDGDALTEERIDMVVNMLLKDFKENLIETNGWPSFGAAYTTSKACLNAYT
RVLANKIPKFQVNCVCPGLVKTEMNYGIGNYTAEEGAEHVVRIALFPDDGPSGFFYDCSEL
SAF
11111111111000000000000000000000000000000000000000000000000000000000000000000000000
00000000000000000000000000000000000000000000000000000000000000000000000000000000000
00000000000000000000000000000000000000000000000000000000000000000000000000000000000
00000000000000000000000000000000000000000000000000000000111111
>Q83467_2wsu_392_702
PFVLPLPDGVPTGASIVLEGTLTPSAVFFTLDLVTGPASLALHFNVRLPLEGEKHIVCNSREGS
SNWGEEVRPQEFPFEREKPFVLVIVIQSDTYQITVNGKPLVDFPQRLQGITRASLSGDLVFTRL
TMYPPGDPRPTTLLPPPAAPLDVIPDAYVLNLPTGLTPRTLLTVTGTPTPLAEFFIVNLVYDL
HYDSKNVALHFNVGFTSDSKGHIACNARMNGTWGSEITVSDFPFQRGKPFTLQILTREADF
QVLVDKQPLTQFQYRLKELDQIKYVHMFGHVVQTHLEHQVPDTPVFSTAGVSKVYPQIL
00000000000000000000000000000000000000000000000000000000000000000000000000000000000
00000000000000000000000000000000000000000000000000000000000000000000000000000000000
00000000000000000000000000000000000000000000000000000000000000000000000000000000000
00000000000000000000000000000000000000000000000000111111111111
>Q8DV82_2w3z_0_310
MTVQQHWKKWQAKIFSAKNRPNLLFSLLIIGLVLGVVLVQAKSMQKQHKNHIRSSRISKK
KTTPKPNINPNALKIGSNHNNQAEGYAYSAETVRQMMNNKQASAKQKLVFLTFDDGVDP
NMTPKILDVLAQQHVHATFFLVGCNITDKVKPILQRQITEGHALGIHSFSHVYSLLYPNRVG
NTQQIVSEVTRTQNALKDQLGQNFKTGVWRYPGGHLSWTGLEAADKQLAAQGIQWMD
WNAAVGDAEPLATRPTTVASMLAFLDGSAKIATNPNVQVVLMHDISEKTITLASLPQIIRYY
KDRGYTFAVLK
11111111111111111111111111111111111111111111111111111111111111111111100000000000000
00000000000000001111110000000000000000000000000000000000000000000000000000000000000
00000000000000000000000000000000000000000000000000000000000000000000000000000000000
00000000000000000000000000000000000000000000000000000000000000
>Q9ABM7_3oox_0_310

MSTSAIDPVSFSLYAKDFTRFAQELGASFERYGFAVLSDYDLDQARIDAAVDSAKAFFALPVE
TKKQYAGVKGGARGYIPFGVETAKGADHYDLKEFWHMGRDLPPGHRFRAHMADNVWP
AEIPAFKHDVSWLYNSLDGMGGKVLEAIATYLKLERDFFKPTVQDGNSVLRLLHYPPIPKD
ATGVRAGAHGDINTITLLLGAEEGGLEVLDRDGQWLPINPPPGCLVINIGDMLERLTNNVL
PSTVHRVVNPPPERRGVPRYSTPFFLHFASDYEIKTLQNCVTAENPDRYPESITADEFLQQRL
REIKLA
11000000000000000000000000000000000000000000000000000000000000000000000000000000000
00000000000000000000000000000000000000000000000000000000000000000000000000000000000
00000000000000000100000000000000000000000000000000000000000000000000000000000000000
00000000000000000000000000000000000000000000000000000000000011
>Q9R1T5_2gu2_1_311
TSCVAEEPIKKIAIFGGTHGNELTGVFLVTHWLKNGAEVHRAGLEVKPFITNPRAVEKCTRY
IDCDLNRVFDLENLSKEMSEDLPYEVRRAQEINHLFGPKNSDDAYDVVFDLHNTTSNMGC
TLILEDSRNDFLIQMFHYIKTCMAPLPCSVYLIEHPSLKYATTRSIAKYPVGIEVGPQPHGVLR
ADILDQMRRMLKHALDFIQRFNEGKEFPPCAIDVYKIMEKVDYPRNESGDVAAVIHPNLQ
DQDWKPLHPGDPVFVSLDGKVIPLGGDCTVYPVFVNEAAYYEKKEAFAKTTKLTLNAKSIR
STLH
11000000000000000000000000000000000000000000000000000000000000000000000000000000000
00000000000000000000000000000000000000000000000000000000000000000000000000000000000
00000000000000000000000000000000000000000000000000000000000000000000000000000000000
00000000000000000000000000000000000000000000000000000000000011
>P02906_1sbp_19_328
KDIQLLNVSYDPTRELYEQYNKAFSAHWKQETGDNVVIRQSHGGSGKQATSVINGIEADV
VTLALAYDVDAIAERGRIDKNWIKRLPDNSAPYTSTIVFLVRKGNPKQIHDWNDLIKPGVS
VITPNPKSSGGARWNYLAAWGYALHHNNNDQAKAQDFVKALFKNVEVLDSGARGSTNT
FVERGIGDVLIAWENEALLATNELGKDKFEIVTPSESILAEPTVSVVDKVVEKKDTKAVAEAY
LKYLYSPEGQEIAAKNFYRPRDADVAKKYDDAFPKLKLFTIDEVFGGWAKAQKDHFANGG
TFDQISKR
00000000000000000000000000000000000000000000000000000000000000000000000000000000000
00000000000000000000000000000000000000000000000000000000000000000000000000000000000
00000000000000000000000000000000000000000000000000000000000000000000000000000000000
0000000000000000000000000000000000000000000000000000000000001
>P32396_1ak1_0_309
MSRKKMGLLVMAYGTPYKEEDIERYYTHIRRGRKPEPEMLQDLKDRYEAIGGISPLAQITEQ
QAHNLEQHLNEIQDEITFKAYIGLKHIEPFIEDAVAEMHKDGITEAVSIVLAPHFSTFSVQSY
NKRAKEEAEKLGGLTITSVESWYDEPKFVTYWVDRVKETYASMPEDERENAMLIVSAHSLP
EKIKEFGDPYPDQLHESAKLIAEGAGVSEYAVGWQSEGNTPDPWLGPDVQDLTRDLFEQK
GYQAFVYVPVGFVADHLEVLYDNDYECKVVTDDIGASYYRPEMPNAKPEFIDALATVVLK
KLGR
11000000000000000000000000000000000000000000000000000000000000000000000000000000000
00000000000000000000000000000000000000000000000000000000000000000000000000000000000
00000000000000000000000000000000000000000000000000000000000000000000000000000000000
0000000000000000000000000000000000000000000000000000000000000
>P39172_2ogw_0_309
MLHKKTLLFAALSAALWGGATQAADAAVVASLKPVGFIASAIADGVTETEVLLPDGASEH
DYSLRPSDVKRLQNADLVVWVGPEMEAFMQKPVSKLPGAKQVTIAQLEDVKPLLMKSIH
GDDDDHDHAEKSDEDHHHGDFNMHLWLSPEIARATAVAIHGKLVELMPQSRAKLDANL
KDFEAQLASTETQVGNELAPLKGKGYFVFHDAYGYFEKQFGLTPLGHFTVNPEIQPGAQRL
HEIRTQLVEQKATCVFAEPQFRPAVVESVARGTSVRMGTLDPLGTNIKLGKTSYSEFLSQLA
NQYASCLKGD
11111111111111111111111111000000000000000000000000000000000000000000000000000000000

0000000000000000000000000000000001111111111111111111111110000000000000000000000000000
00000000000000000000000000000000000000000000000000000000000000000000000000000000000
0000000000000000000000000000000000000000000000000000000000011
>P77454_1u60_0_309
MLDANKLQQAVDQAYTQFHSLNGGQNADYIPFLANVPGQLAAVAIVTCDGNVYSAGDS
DYRFALESISKVCTLALALEDVGPQAVQDKIGADPTGLPFNSVIALELHGGKPLSPLVNAGA
IATTSLINAENVEQRWQRILHIQQQLAGEQVALSDEVNQSEQTTNFHNRAIAWLLYSAGYL
YCDAMEACDVYTRQCSTLLNTIELATLGATLAAGGVNPLTHKRVLQADNVPYILAEMMM
EGLYGRSGDWAYRVGLPGKSGVGGGILAVVPGVMGIAAFSPPLDEDGNSVRGQKMVASVA
KQLGYNVFKG
10000000000000000000000000000000000000000000000000000000000000000000000000000000000
00000000000000000000000000000000000000000000000000000000000000000000000000000000000
00000000000000000000000000000000000000000000000000000000000000000000000000000000000
0000000000000000000000000000000000000000000000000000000000000
>Q3ACS0_2h9a_0_309
MAVEVLKEKWNSKVVEVTLGTGDKTVTLGGDSTLPFLTFEGEMPNPPRFALEVFDTPPTD
WPDILVEPFKDVINDPVAWAKKCVEYGADIVALRLVSAHPDGQNRSGAELAEVCKAVADA
IDVPLMIIGCGVEEKDAEIFPVIGEALSGRNCLLSSATKDNYKPIVATCMVHGHSVVASAPLD
INLSKQLNIMIMEMNLAPNRIIMDPLIGALGYGIEYSYSIIERMRLGALTGDKILAMPVVCFI
GQEAWKAKEAKDPEVAEWGDYALRAIHWETVTTVALIQAGGHLFVMRHPKSLAEVKEHL
KRILK
11000000000000000000000000000000000000000000000000000000000000000000000000000000000
00000000000000000000000000000000000000000000000000000000000000000000000000000000000
00000000000000000000000000000000000000000000000000000000000000000000000000000000000
0000000000000000000000000000000000000000000000000000000000001
>Q736M3_3h41_23_332
EEKKDSKAFIDVSAATLWTAPDSLRPIDVPSATNPVDLWKWTKSMTLDEKLWLTNANKLET
QALLGQEVTVVDKKGDWVKVLVHGQPTPRNEEGYPGWMPEKQLTYNQEFADKTNEPFV
LVTKPTAILYINPSEKHKSLEVSYNTRLPLLSEDTISYRVLLPNGQKAWLRKNDGTFYRSQND
IPTPAADDLINTGKMFLGLPYIWAGTSGFGFDCSGFTHTIYKSHGITIPRDSGPQSRNGVAVD
KEHLQKGDLIFFAHDQGKGSVHHVAMYIGDGNMIHSPRAERSVEIIPLNTPGYIEEYAGAR
RYLP
11111000000000000000000000000000000000000000000000000000000000000000000000000000000
00000000000000000000000000000000000000000000000000000000000000000000000000000000000
00000000000000000000000000000000000000000000000000000000000000000000000000000000000
0000000000000000000000000000000000000000000000000000000000000
>Q9CCZ4_2uyo_0_309
MRTHDDTWDIKTSVGTTAVMVAAARAAETDRPDALIRDPYAKLLVTNTGAGALWEAMLD
PSMVAKVEAIDAEAAAMVEHMRSYQAVRTNFFDTYFNNAVIDGIRQFVILASGLDSRAYRL
DWPTGTTVYEIDQPKVLAYKSTTLAEHGVTPTADRREVPIDLRQDWPPALRSAGFDPSART
AWLAEGLLMYLPATAQDGLFTEIGGLSAVGSRIAVETSPLHGDEWREQMQLRFRRVSDALG
FEQAVDVQELIYHDENRAVVADWLNRHGWRATAQSAPDEMRRVGRWGDGVPMADDKD
AFAEFVTAHRL
11111111111110000000000000000000000000000000000000000000011111111111100000000000000
00000000000000000000000000000000000000000000000000000000000000000000000000000000000
00000000000000000000000000000000000000000000000000000000000000000000000000111111111
1000000000000000000000000000000000000000000000000000000000000
>Q9RBY6_2fcu_0_309
MSKKFALTAEQRASFEKNGFIGPFDAYSPEEMKETWKRTRLRLLDRSAAAYQDLDAISGGT
NIANYDRHLDDDFLASHICRPEICDRVESILGPNVLCWRTEFFPKYPGDEGTDWHQADTFA
NASGKPQIIWPENEEFGGTITVWTAFTDANIANGCLQFIPGTQNSMNYDETKRMTYEPDA

NNSVVKDGVRRGFFGYDYRQLQIDENWKPDEASAVPMQMKAGQFIIFWSTLMHASYPHS
GESQEMRMGFASRYVPSFVHVYPDSDHIEEYGGRISLEKYGAVQVIGDETPEYNRLVTHTTR
GKKFEAV
10000000000000000000000000000000000000000000000000000000000000000000000000000000000
00000000000000000000000000000000000000000000000000000000000000000000000000000000000
00000000000000000000000000000000000000000000000000000000000000000000000000000000000
0000000000000000000000000000000000000000000000000000000000000
>Q9RW55_4h6r_0_309
MIDQLYRKAVLTVAERPQVEQLARQKMWNLAERFVAGESIESAIQAVQALERDGIAGNLD
LLGEFIDSPAKCTEFADDVIKLIEAAHAAGIKPYVSIKLSSVGQGKDENGEDLGLTNARRIIA
KAKEYGGFICLDMEDHTRVDVTLEQFRTLVGEFGAEHVGTVLQSYLYRSLGDRASLDDLRP
NIRMVKGAYLEPATVAYPDKADVDQNYRRLVFQHLKAGNYTNVATHDERIIDDVKRFVLA
HGIGKDAFEFQMLYGIRRDLQKQLAAEGYRVRVYLPYGRDWYAYFSRRIAETPRNAAFVVQ
GMLKG
11111111111111111110000000000000000000000000000000000000000000000000000000000000000
00000000000000000000000000000000000000000000000000000000000000000000000000000000000
00000000000000000000000000000000000000000000000000000000000000000000000000000000000
0000000000000000000000000000000000000000000000001111111111111
>A0QVS8_3nwo_0_308
MLSRMPVSSRTVPFGDHETWVQVTTPENAQPHALPLIVLHGGPGMAHNYVANIAALADE
TGRTVIHYDQVGCGNSTHLPDAPADFWTPQLFVDEFHAVCTALGIERYHVLGQSWGGML
GAEIAVRQPSGLVSLAICNSPASMRLWSEAAGDLRAQLPAETRAALDRHEAAGTITHPDYL
QAAAEFYRRHVCRVVPTPQDFADSVAQMEAEPTVYHTMNGPNEFHVVGTLGDWSVIDRL
PDVTAPVLVIAGEHDEATPKTWQPFVDHIPDVRSHVFPGTSHCTHLEKPEEFRAVVAQFLH
QHDLAADARV
11111110000000000000000000000000000000000000000000000000000000000000000000000000000
00000000000000000000000000000000000000000000000000000000000000000000000000000000000
00000000000000000000000000000000000000000000000000000000000000000000000000000000000
000000000000000000000000000000000000000000000000000000000111
>P0AEE5_2ipm_23_331
ADTRIGVTIYKYDDNFMSVVRKAIEQDAKAAPDVQLLMNDSQNDQSKNDQIDVLLAKG
VKALAINLVDPAAAGTVIEKARGQNVPVVFFNKEPSRKALDSYDKAYYVGTDSKESGIIQG
DLIAKHWAANQGWDLNKDGQIQFVLLKGEPGHPDAEARTTYVIKELNDKGIKTEQLQLD
TAMWDTAQAKDKMDAWLSGPNANKIEVVIANNDAMAMGAVEALKAHNKSSIPVFGVD
ALPEALALVKSGALAGTVLNDANNQAKATFDLAKNLADGKGAADGTNWKIDNKVVRV
PYVGVDKDNLAEFSKK
00000000000000000000000000000000000000000000000000000000000000000000000000000000000
00000000000000000000000000000000000000000000000000000000000000000000000000000000000
00000000000000000000000000000000000000000000000000000000000000000000000000000000000
000000000000000000000000000000000000000000000000000000000011
>P15085_2v77_110_418
ARSTDTFNYATYHTLEEIYDFLDLLVAENPHLVSKIQIGNTYEGRPIYVLKFSTGGSKRPAIWI
DTGIHSREWVTQASGVWFAKKITQDYGQDAAFTAILDTLDIFLEIVTNPDGFAFTHSTNRM
WRKTRSHTAGSLCIGVDPNRNWDAGFGLSGASSNPCSETYHGKFANSEVEVKSIVDFVKD
HGNIKAFISIHSYSQLLMYPYGYKTEPVPDQDELDQLSKAAVTALASLYGTKFNYGSIIKAIY
QASGSTIDWTYSQGIKYSFTFELRDTGRYGFLLPASQIIPTAKETWLALLTIMEHTLNHPY
00000000000000000000000000000000000000000000000000000000000000000000000000000000000
00000000000000000000000000000000000000000000000000000000000000000000000000000000000
00000000000000000000000000000000000000000000000000000000000000000000000000000000000
000000000000000000000000000000000000000000000000000000000001
>P29082_2cb2_0_308

MPKPYVAINMAELKNEPKTFEMFASVGPKVCMVTARHPGFVGFQNHIQIGILPFGNRYGG
AKMDMTKESSTVRVLQYTFWKDWKDHEEMHRQNWSYLFRLCYSCASQMIWGPWEPIYEI
IYANMPINTEMTDFTAVVGKKFAEGKPLDIPVISQPYGKRVVAFAEHSVIPGKEKQFEDAIVR
TLEMLKKAPGFLGAMVLKEIGVSGIGSMQFGAKGFHQVLENPGSLEPDPNNVMYSVPEAK
NTPQQYIVHVEWANTDALMFGMGRVLLYPELRQVHDEVLDTLVYGPYIRILNPMMEGTF
WREYLNEQ
10000000000000000000000000000000000000000000000000000000000000000000000000000000000
00000000000000000000000000000000000000000000000000000000000000000000000000000000000
00000000000000000000000000000000000000000000000000000000000000000000000000000000000
00000000000000000000000000000000000000000000000000000000001
>P37487_2haw_0_308
MEKILIFGHQNPDTDTICSAIAYADLKNKLGFNAEPVRLGQVNGETQYALDYFKQESPRLVE
TAANEVNGVILVDHNERQQSIKDIEEVQVLEVIDHHRIANFETAEPLYYRAEPVGCTATILN
KMYKENNVKIEKEIAGLMLSAIISDSLLFKSPTCTDQDVAAAKELAEIAGVDAEEYGLNML
KAGADLSKKTVEELISLDAKEFTLGSKKVEIAQVNTVDIEDVKKRQAELEAVISKVVAEKNL
DLFLLVITDILENDSLALAIGNEAAKVEKAFNVTLENNTALLKGVVSRKKQVVPVLTDAMA
E
00000000000000000000000000000000000000000000000000000000000000000000000000000000000
00000000000000000000000000000000000000000000000000000000000000000000000000000000000
00000000000000000000000000000000000000000000000000000000000000000000000000000000000
00000000000000000000000000000000000000000000000000000000001
>Q7WYF5_3hpy_0_308
MAMTGVLRPGHAQVRVLNLEEGIHFYRNVLGLVETGRDDQGRVYFKCWDERDHSCYIIRE
ADTAGIDFFGFKVLDKATLEKLDADLQAYGLTTTRIPAGEMLETGERVRFELPSGHLIELYAE
KTCVGNGISEVNPAPWNAQREHGIAPIQLDHCLLYGPNIAEVQKIFTEVLGFYLVERVLSPD
GDSDMGIWLSCSHKVHDIAFVEYPEKGKLHHCSFLLESWEQVLRAGDIMSMNEVNVDIGP
TRHGVTRGCTIYAWDPSGNRFETFMGGYHPYPDYEPLSWTYDNFAQGLDYPQRKLHETFM
TVVT
10000000000000000000000000000000000000000000000000000000000000000000000000000000000
00000000000000000000000000000000000000000000000000000000000000000000000000000000000
00000000000000000000000000000000000000000000000000000000000000000000000000000000000
00000000000000000000000000000000000000000000000111111111111
>Q8DI95_2xbg_38_346
IPALDYNPWEAIQLPTTATILDMSFIDRHHGWLVGVNATLMETRDGGQTWEPRTLVLDHS
DYRFNSVSFQGNEGWIVGEPPIMLHTTDGGQSWSQIPLDPKLPGSPRLIKALGNGSAEMIT
NVGAIYRTKDSGKNWQALVQEAIGVMRNLNRSPSGEYVAVSSRGSFYSTWEPGQTAWEPH
NRTTSRRLHNMGFTPDGRLWMIVNGGKIAFSDPDNSENWGELLSPLRRNSVGFLDLAYRT
PNEVWLAGGAGALLCSQDGGQTWQQDVDVKKVPSNFYKILFFSPDQGFILGQKGILLRYV
TDLTAAPA
00000000000000000000000000000000000000000000000000000000000000000000000000000000000
00000000000000000000000000000000000000000000000000000000000000000000000000000000000
00000000000000000000000000000000000000000000000000000000000000000000000000000000000
00000000000000000000000000000000000000000000000000000000011
>Q8U1H5_2dsk_408_716
NANPIPEHFFAPYIDMSLSVHKPLVEYAKLTGTKYFTLAFILYSSVYNGPAWAGSIPLEKFVDE
VRELREIGGEVIIAFGGAVGPYLCQQASTPEQLAEWYIKVIDTYNATYLDFDIEAGIDADKLA
DALLIVQRERPWVKFSFTLPSDPGIGLAGGYGIIETMAKKGVRVDRVNPMTMDYYWTPSN
AENAIKVAENVFRQLKQIYPEKSDEEIWKMIGLTPMIGVNDDKSVFTLEDAQQLVDWAIQH
KIGSLAFWSVDRDHPGPTGEVSPLHRGTNDPDWAFSHVFVKFMEAFGYTFSAQTSEASVPT
00000000000000000000000000000000000000000000000000000000000000000000000000000000000
00000000000000000000000000000000000000000000000000000000000000000000000000000000000

00000000000000000000000000000000000000000000000000000000000000000000000000000000000
000000000000000000000000000000000000000000000000001111111111
>Q95NL6_2jks_54_362
VEVTSRLTVAGSDAKCDFFTPATQDSPPVSGSLTLSKGRMTATFECSATQALSISTIPTNIEQN
VCDPKKTTNGTVCQFGANGSAGTEVTLKDLLETDRIVNWKVNEQREESNKSQKWSLELH
NEDLPLTDKAFVVGCQATSAARGTESGKTAACKLTVNVEARASSLAENNVVTCAYGKGSN
PNPVEVEMSTEKNTLTINCGSDGSLQPTTYAEEYCVADSKDVNRCSTTRFVEIFPKFLKSWW
VTETQKRTSATLTIPQTDLPEADQQFLVGCVPKKTAPEDPKKDKESGTETGAPTSCTVLVTV
KA
11100000000000000011000000000000000000000000000000000000000000000000000000000000000
00000000000000000000000001111110000000000000000000000000000011111000000000000000000
00000000000000000000000000000000000000000000000000000000000000000000000000000000000
000000000000000000000000000000011111111111111100000000000000
>Q9HYL8_3n5l_25_333
DQPVINFGIISTESSQNLKSIWEPFLKDMSQQTGYQVKAFFAPDYAGIIQGMRFDKVDIAWY
GNKAAMEAVDRAHGEIFAQTVAASGAPGYWSLLIANKDSKIDSLEDMLANAKSLTFGNG
DPNSTSGYLVPGYYVFAKNNVDPVKAFKRTLNSSHEVNALAVANKQVDVATFNTEGMERL
ELTQPEKARQLKVIWKSPLIPGDPLVWRNNLSDEQKNKLRDFFFKYGANAEQKKVLADLQ
WSKFQPSDDDQLLPIRQLELFKQRTDVANNANLGAEEKAAKLKALDEELAKLEKRMAERE
QKTAANAG
00000000000000000000000000000000000000000000000000000000000000000000000000000000000
00000000000000000000000000000000000000000000000000000000000000000000000000000000000
00000000000000000000000000000000000000000000000000000000000000000000000000000000000
000000000000000000000000000000000000000000000000000000000111
>A5TY84_3ort_0_307
MTTPSHLSDRYELGEILGFGGMSEVHLARDLRLHRDVAVKVLRADLARDPSFYLRFRREAQ
NAAALNHPAIVAVYDTGEAETPAGPLPYIVMEYVDGVTLRDIVHTEGPMTPKRAIEVIADA
CQALNFSHQNGIIHRDVKPANIMISATNAVKVMDFGIARAIADSGNSVTQTAAVIGTAQYL
SPEQARGDSVDARSDVYSLGCVLYEVLTGEPPFTGDSPVSVAYQHVREDPIPPSARHEGLSA
DLDAVVLKALAKNPENRYQTAAEMRADLVRVHNGEPPEAPKVLTDAERTSLLSSAAGNLS
GPR
00000000000000000000000000000000000000000000000000000000000000000000000000000000000
00000000000000000000000000000000000000000000000000000000000000000000000000000011111
11111111111110000000000000000000000000000000000000000000000000000000000000000000000
00000000000000000000000000000000000000001111111111111111111
>B1MJ53_3r6o_0_307
MNLRIVSFAGADQWQPGVLIDDDTVAPLAPMLARHDIQVSSARELIALLPQISPLISDELAG
GSFEPVPLDSVALGAPIPEPGQVIALGFNYPTHSDTDSPLPKMADPVVFMKSPTSISGPRDAV
IAPRTSHALDYEIEIAVVIGKPGYRIERSQAIKHVAGYMLANDITARDVALPFGFGGSPLQAQ
VVRGKGYPTFCPTGPWLFTTGSDTTFETFDFELRINGELRQSGSTVDMTLGFAEVVETVSATI
ALRAGDIILTGTPGGCGFQFDPPRYLRPGDVIEAHSAKLGKMRLPVHDEKPIESASR
00000000011110000000000000000001111111110000000000000000000011111100000000000000000
00000000000011111111111000000000000000000000000000000000000000000000000000000000000
00000000000001111110000000000000000000000000000000000000000000000000000000000000000
00000000000000000000000000000000000000000000000000000111111
>C8WTE4_3rnl_0_307
MGVARPNFFIVGAAKCGTSSLDRYLSQHPDIYIPPKKEAHFFSIPDFPERFTGPGDEGMNLYT
IRDEDAYMRLFDGVRGERAVGEASVFYLFYPGTAQRMYDAYPDAKILIMLRNPVDRAFSAY
MHLVRDERETLSFRESLAKEEERIRQHYEPLWYYRAVGLYAAQVKRYLDVFGREQVKVILFE
EFARDPVQVVRDCCAFLGVSTDFVPDTSIRHNESGVPKSRSLYNFIAKPNALKEIVKPFIPAA
VRERLGNRAKSMVLGRMEMEPDLREELTAFFAPDVARLEALIHRDLSAWRRPARAAGSQ

11000000000000000000000000000000000000000000000000000000000000000000000000000000000
00000000000000000000000000000000000000000000000000000000000000000000000000000000000
00000000000000000000000000000000000000000000000011111111111111111111111111111111111
1111111111111111000000000000000000000000000000000011111111
>O59072_3a4i_0_307
MDWGRFVEEKVREIRETVGDSKAIIALSGGVDSSTAAVLAHKAIGDRLHAVFVNTGFLRKG
EPEFVVKTFRDEFGMNLHYVDAQDRFFSALKGVTDPEEKRKIIGRVFIEVFEEVAKKIGAEYL
IQGTIAPDWIESQGKIKSHHNVGGLPEKLNLKLIEPLRDLYKDEVRELAKFLGLPEKIYNRM
PFPGPGLAVRVIGEVTPEKIRIVREANAIVEEEVERAGLRPWQAFAVLLGVKTVGVQGDIRA
YKETIAVRIVESIDGMTANAMNVPWEVLQRIAFRITSEIPEVGRVLYDITNKPPATIEFE
00000000000000000000000000000000000000000000000000000000000000000000000000000000000
00000000000000000000000000000000000000000000000011111111111111111111111000000000000
00000000000000000000000000000000000000000000000000000000000000000000000000000000000
0000000000000000000000000000000000000000000000000000000000
>P53667_3s95_329_636
PHRIFRPSDLIHGEVLGKGCFGQAIKVTHRETGEVMVMKELIRFDEETQRTFLKEVKVMRCL
EHPNVLKFIGVLYKDKRLNFITEYIKGGTLRGIIKSMDSQYPWSQRVSFAKDIASGMAYLHS
MNIIHRDLNSHNCLVRENKNVVVADFGLARLMVDEKTQPEGLRSLKKPDRKKRYTVVGN
PYWMAPEMINGRSYDEKVDVFSFGIVLCEIIGRVNADPDYLPRTMDFGLNVRGFLDRYCPP
NCPPSFFPITVRCCDLDPEKRPSFVKLEHWLETLRMHLAGHLPLGPQLEQLDRGFWETYRR
GES
00000000000000000000000000000000000000000000000000000000000000000000000000000000000
00000000000000000000000000000000000000000000000000000000000000000000000000111111111
11111111110000000000000000000000000000000000000000000000000000000000000000000000000
0000000000000000000000000000000000000000000000000000000000
>Q2GA85_3ioy_1_308
KDFAGRTAFVTGGANGVGIGLVRQLLNQGCKVAIADIRQDSIDKALATLEAEGSGPEVMG
VQLDVASREGFKMAADEVEARFGPVSILCNNAGVNLFQPIEESSYDDWDWLLGVNLHGV
VNGVTTFVPRMVERVKAGEQKGGHVVNTASMAAFLAAGSPGIYNTTKFAVRGLSESLHYS
LLKYEIGVSVLCPGLVKSYIYASDDIRPDALKGEMKPVDKTAVERLAGVHEFGMEPDVIGAR
VIEAMKANRLHIFSHPDHKEELREIFDEIIAEYQDYPKDPGYDQRVAFEKFRADSFAEARRQ
SRAAD
00000000000000000000000000000000000000000000000000000000000000000000000000000000000
00000000000000000000000000000000000000000000000000000000000000000000000000000000000
00000000000000000000000000000011111111111111111111111111110000000000000000000000000
0000000000000000000000000000000000000000000000000000000111
>Q4DSA0_2a0m_0_307
MAARTDDPRLLSLFSAQREEDADIVIIGFPYDEGCVRNGGRAGAKKGPAAFRFFLQRLGSV
NNLELNVDASHLKLYDAGDITASTLEEAHEKLESKVFTVLARGAFPFVIGGGNDQSAPNGR
AMLRAFPGDVGVINVDSHLDVRPPLSDGRVHSGTPFRQLLEESSFSGKRFVEFACQGSQCG
ALHAQYVRDHQGHLMWLSEVRKKGAVAALEDAFGLTGKNTFFSFDVDSLKSSDMPGVSC
PAAVGLSAQEAFDMCFLAGKTPTVMMMDMSELNPLVEEYRSPRVAVYMFYHFVLGFATRS
KPKAEN
11110000000000000000000000000000000000000000000000000000000000000000000000000000000
00000000000000000000000000000000000000000000000000000000000000000000000000000000000
00000000000000000000000000000000000000000000000000000000000000000000000000000000000
0000000000000000000000000000000000000000000000000000111111
>Q9HCP0_2cmw_44_351
RVGKKIGCGNFGELRLGKNLYTNEYVAIKLEPIKSRAPQLHLEYRFYKQLGSAGEGLPQVYY
FGPCGKYNAMVLELLGPSLEDLFDLCDRTFTLKTVLMIAIQLLSRMEYVHSKNLIYRDVKPE
NFLIGRQGNKKEHVIHIIDFGLAKEYIDPETKKHIPYREHKSLTGTARYMSINTHLGKEQSRR

DDLEALGHMFMYFLRGSLPWQGLKADTLKERYQKIGDTKRNTPIEALCENFPEEMATYLR
YVRRLDFFEKPDYEYLRTLFTDLFEKKGYTFDYAYDWVGRPIPTPVGSVHVDSGASAITRE
00000000000000000000000000000000000000000000000000000000000000000000000000000000000
00000000000000000000000000000000000000000000000000000000000000000000000000000000000
01000000000000000000000000000000000000000000000000000000000000000000000000000000000
00000000000000000000000000000000000000000000001111111111111
>Q9I1X7_2far_532_839
GARKASAGASRAATAGVRISHPQRLIDPSIQASKLELAEFHARYADLLLRDLRERPVSLVRG
PDGIGGELFFQKHAARLKIPGIVQLDPALDPGHPPLLQIRSAEALVGAVQMGSIEFHTWNAS
LANLERPDRFVLDLDPDPALPWKRMLEATQLSLTLLDELGLRAFLKTSGGKGMHLLVPLER
RHGWDEVKDFAQAISQHLARLMPERFSAVSGPRNRVGKIFVDYLRNSRGASTVAAYSVRA
REGLPVSVPVFREELDSLQGANQWNLRSLPQRLDELAGDDPWADYAGTRQRISAAMRRQL
GRG
11111111110000000000000000000000000000000000000000000000000000000000000000000000000
00000000000000000000000000000000000000000000000000000000000000000000000000000000000
00000000000000000000000000000000000000000000000000000000000000000000000000000000000
00000000000000000000000000000000000000000000000000000000111
>Q9Z1N4_1jp4_0_307
MASSHNVLMRLVASAYSIAQKAGTIVRCVIAEGDLGIVQKTSATDLQTKADRMVQMSICSS
LSRKFPKLTIIGEEDLPPGEVDQELIEDGQSEEILKQPCPSQYSAIKEEDLVVWVDPVDGTKEY
TEGLLDNVTVLIGIAYEGKAIAGIINQPYYNYQAGPDAVLGRTIWGVLGLGAFGFQLKEAP
AGKHIITTTRSHSNKLVTDCIAAMNPDNVLRVGGAGNKIIQLIEGKASAYVFASPGCKKWD
TCAPEVILHAVGGKLTDIHGNPLQYDKEVKHMNSAGVLAALRNYEYYASRVPESVKSALIP
11110000000000000000000000000000000000000000000000000000000000000000000000000011000
00000000000000000000000000000000000000000000000000000000000000000000000000000000000
00000000000000000000000000000000000000000000000000000000000000000000000000000000000
00000000000000000000000000000000000000000000000000000000000
>O14757_2r0u_0_306
MAVPFVEDWDLVQTLGEGAYGEVQLAVNRVTEEAVAVKIVDMKRAVDCPENIKKEICINK
MLNHENVVKFYGHRREGNIQYLFLEYCSGGELFDRIEPDIGMPEPDAQRFFHQLMAGVVY
LHGIGITHRDIKPENLLLDERDNLKISDFGLATVFRYNNRERLLNKMCGTLPYVAPELLKRR
EFHAEPVDVWSCGIVLTAMLAGELPWDQPSDSCQEYSDWKEKKTYLNPWKKIDSAPLALL
HKILVENPSARITIPDIKKDRWYNKPLKKGAKRPRVTSGGVSESPSGFSKHIQSNLDFSPVNS
AS
10000000000000000111000000000000000000000111111000000000000000000000000000000000000
00000000000000000000000000000000000000000000000000000000000000000000000000000000000
00000000000000000000000000000000000000000000000000000000000000000000000000000000000
0000000000000000000000000000011111111111111111111111111111
>Q8DPY6_4h59_34_340
HAPDKIVLDHAFGQTILDKKPERVATIAWGNHDVALALGIVPVGFSKANYGVSADKGVLP
WTEEKIKELNGKANLFDDLDGLNFEAISNSKPDVILAGYSGITKEDYDTLSKIAPVAAYKSKP
WQTLWRDMIKIDSKALGMEKEGDELIKNTEARISKELEKHPEIKGKIKGKKVLFTMINAAD
TSKFWIYTSKDPRANYLTDLGLVFPESLKEFESEDSFAKEISAEEANKINDADVIITYGDDKTL
EALQKDPLLGKINAIKNGAVAVIPDNTPLAASCTPTPLSINYTIEEYLNLLGNACKNAK
10000000000000000000000000000000000000000000000000000000000000000000000000000000000
00000000000000000000000000000000000000000000000000000000000000000000000000000000000
00000000000000000000000000000000000000000000000000000000000000000000000000000000000
0000000000000000000000000000000000000000000000000000000000
>Q9HTZ1_1zl0_0_306
MTSRPSSDQTWQPIDGRVALIAPASAIATDVLEATLRQLEVHGVDYHLGRHVEARYRYLAG
TVEQRLEDLHNAFDMPDITAVWCLRGGYGCGQLLPGLDWGRLQAASPRPLIGFSDISVLLS

AFHRHGLPAIHGPVATGLGLSPLSAPREQQERLASLASVSRLLAGIDHELPVQHLGGHKQR
VEGALIGGNLTALACMAGTLGGLHAPAGSILVLEDVGEPYYRLERSLWQLLESIDARQLGAI
CLGSFTDCPRKEVAHSLERIFGEYAAAIEVPLYHHLPSGHGAQNRAWPYGKTAVLEGNRLR
W
11000000000000000000000000000000000000000000000000000000000000000000000000000000000
00000000000000000000000000000000000000000000000000000000000000000000000000000000000
00000000000000000000000000000000000000000000000000000000000000000000000000000000000
0000000000000000000000000000000000000000000000000000000000
>O06441_1lzk_0_305
MTTFPTLDPELAAALTMLPKVDFADLPNARATYDALIGAMLADLSFDGVSLRELSAPGLDG
DPEVKIRFVTPDNTAGPVPVLLWIHGGGFAIGTAESSDPFCVEVARELGFAVANVEYRLAPE
TTFPGPVNDCYAALLYIHAHAEELGIDPSRIAVGGESAGGGLAAGTVLKARDEGVVPVAFQ
FLEIPELDDRLETVSMTNFVDTPLWHRPNAILSWKYYLGESYSGPEDPDVSIYAAPSRATDLT
GLPPTYLSTMELDPLRDEGIEYALRLLQAGVSVELHSFPGTFHGSALVATAGVRERGAA
10000000000000000000000000000000000000000000000000000000000000000000000000000000000
00000000000000000000000000000000000000000000000000000000000000000000000000000000000
00000000000000000000000000000000000000000000000000000000000000000000000000000000000
000000000000000000000000000000000000000000000000000000000
>P02924_6abp_23_328
ENLKLGFLVKQPEEPWFQTEWKFADKAGKDLGFEVIKIAVPDGEKTLNAIDSLAASGAKGF
VICTPDPKLGSAIVAKARGYDMKVIAVDDQFVNAKGKPMDTVPLVMMAATKIGERQGQE
LYKEMQKRGWDVKESAVMAITANELDTARRRTTGSMDALKAAGFPEKQIYQVPTKSNDIP
GAFDAANSMLVQHPEVKHWLIVGMNDSTVLGGVRATEGQGFKAADIIGIGINGVDAVSEL
SKAQATGFYGSLLPSPDVHGYKSSEMLYNWVAKDVEPPKFTEVTDVVLITRDNFKEELEKK
GLGGK
10000000000000000000000000000000000000000000000000000000000000000000000000000000000
00000000000000000000000000000000000000000000000000000000000000000000000000000000000
00000000000000000000000000000000000000000000000000000000000000000000000000000000000
000000000000000000000000000000000000000000000000000000000
>Q01786_2ckr_160_465
TGTPVERYGKVQVCGTQLCDEHGNPVQLRGMSTHGIQWFDHCLTDSSLDALAYDWKADI
IRLSMYIQEDGYETNPRGFTDRMHQLIDMATARGLYVIVDWHILTPGDPHYNLDRAKTFFA
EIAQRHASKTNVLYEIANEPNGVSWASIKSYAEEVIPVIRQRDPDSVIIVGTRGWSSLGVSEGS
GPAEIAANPVNASNIMYAFHFYAASHRDNYLNALREASELFPVFVTEFGTETYTGDGANDF
QMADRYIDLMAERKIGWTKWNYSDDFRSGAVFQPGTCASGGPWSGSSLKASGQWVRSKL
QS
10000000000000000000000000000000000000000000000000000000000000000000000000000000000
00000000000000000000000000000000000000000000000000000000000000000000000000000000000
00000000000000000000000000000000000000000000000000000000000000000000000000000000000
000000000000000000000000000000000000000000000000000000000
>Q6UDW7_2y8d_2325_2630
GNDYICNKYKNIHDRMKKNNGNFVTDNFVKKSWEISNGVLIPPRRKNLFLYIDPSKICEYK
KDPKLFKDFIYWSAFTEVERLKKAYGGARAKVVHAMKYSFTDIGSIIKGDDMMEKNSSDKI
GKILGDTDGQNEKRKKWWDMNKYHIWESMLCGYREAEGDTETNENCRFPDIESVPQFLR
WFQEWSENFCDRRQKLYDKLNSECISAECTNGSVDNSKCTHACVNYKNYILTKKTEYEIQT
NKYDNEFKNKNSNDKDAPDYLKEKCNDNKCECLNKHIDDKNKTWKNPYETLEDTFKSK
CDCPKP
10000000000000000000000000000000000000000000000000000000000000000000000000000000000
00000000000000000000000000000000000000000000000000000000000000000000000000000000000
00000000000000000000000000000000000000000000000000000000000000000000000000000000000
000000000000000000000000000011111111111111111111100000111

>Q9Y765_3dra_0_305
MTDSKYDYSDITPVDINTEEPQICQILYDEDYKQIMGLLLALMKAEEYSERALHITELGINEL
ASHYTIWIYRFNILKNLPNRNLYDELDWCEEIALDNEKNYQIWNYRQLIIGQIMELNNNDF
DPYREFPILEAMLSSDPKNHHVWSYRKWLVDTFDLHNDAKELSFVDKVIDTDLKNNSAWS
HRFFLLFSKKHLATDNTIDEELNYVKDKIVKCPQNPSTWNYLLGIHERFDRSITQLEEFSLQF
VDLEKDQVTSSFALETLAKIYTQQKKYNESRTVYDLLKSKYDPIRSNFWDYQISKLTSV
1100000000000000000000000000000000000000000000000000000000000000000000000000000000
00000000000000000000000000000000000000000000000000000000000000000000000000000000000
00000000000000000000000000000000000000000000000000000000000000000000000000000000000
0000000000000000000000000000000000000000000000000000000011
>B0RN69_3dr2_0_304
MDSHCRVRPAGPAVPADCDPPRITHAALAARLGDARLLTLYDQATWSEGPAWWEAQRTL
VWSDLVGRRVLGWREDGTVDVLLDATAFTNGNAVDAQQRLVHCEHGRRAITRSDADGQ
AHLLVGRYAGKRLNSPNDLIVARDGAIWFTDPPFGLRKPSQGCPADPELAHHSVYRLPPDG
SPLQRMADLDHPNGLAFSPDEQTLYVSQTPEQGHGSVEITAFAWRDGALHDRRHFASVPD
GLPDGFCVDRGGWLWSSSGTGVCVFDSDGQLLGHIPTPGTASNCTFDQAQQRLFITGGPCL
WMLPLP
11100000000000000000000000000000000000000000000000000000000000000000000000000000000
00000000000000000000000000000000000000000000000000000000000000000000000000000000000
00000000000000000000000000000000000000000001110000000000000000000000000000000000000
00000000000000000000000000000000000000000000000000000000
>Q180U8_3uuw_0_304
MKNIKMGMIGLGSIAQKAYLPILTKSERFEFVGAFTPNKVKREKICSDYRIMPFDSIESLAKK
CDCIFLHSSTETHYEIIKILLNLGVHVYVDKPLASTVSQGEELIELSTKKNLNLMVGFNRRFC
PMYKEIKNNATEIVSINICKHGLNSLRNVRFDSTLIDDYIHVIDTALWLANEDVEISGEDLFL
TDNKNLIFVSHKLKGKNFSINTSMHRDSGTKLEQVEILSKGKIQRVKNLNVLEIEEGGNLTL
KQSGAWVNILKQKGFEDISNHFIDCIENNIKPAINGEECIKAQRLLEKIINSVK
10000000000000000000000000000000000000000000000000000000000000000000000000000000000
00000000000000000000000000000000000000000000000000000000000000000000000000000000000
00000000000000000000000000000000000000000000000000000000000000000000000000000000000
00000000000000000000000000000000000000000000000000000001
>Q46896_3nkd_0_304
MTWLPLNPIPLKDRVSMIFLQYGQIDVIDGAFVLIDKTGIRTHIPVGSVACIMLEPGTRVSHA
AVRLAAQVGTLLVWVGEAGVRVYASGQPGGARSDKLLYQAKLALDEDLRLKVVRKMFEL
RFGEPAPARRSVEQLRGIEGSRVRATYALLAKQYGVTWNGRRYDPKDWEKGDTINQCISAA
TSCLYGVTEAAILAAGYAPAIGFVHTGKPLSFVYDIADIIKFDTVVPKAFEIARRNPGEPDRE
VRLACRDIFRSSKTLAKLIPLIEDVLAAGEIQPPAPPEDAQPVAIPLPVSLGDAGHRSS
11111111000000000000000000000000000000000000000000000000000000000000000000000000000
00000000000000000000000000000000000000000000000000000000000000000000000000000000011
11110000000000000000000000000000000000000000000000000000000000000000000000000000000
00000000000000000000000000000000111111111111111111111111
>Q53654_2f68_29_333
ARDISSTNVTDLTVSPSKIEDGGKTTVKMTFDDKNGKIQNGDMIKVAWPTSGTVKIEGYSKT
VPLTVKGEQVGQAVITPDGATITFNDKVEKLSDVSGFAEFEVQGRNLTQTNTSDDKVATITS
GNKSTNVTVHKSEAGTSSVFYYKTGDMLPEDTTHVRWFLNINNEKSYVSKDITIKDQIQGG
QQLDLSTLNINVTGTHSNYYSGQSAITDFEKAFPGSKITVDNTKNTIDVTIPQGYGSYNSFSI
NYKTKITNEQQKEFVNNSQAWYQEHGKEEVNGKSFNHTVHNINANAGIEGTVKGELK
00000000000000000000000000000000000000000000000000000000000000000000000000000000000
00000000000000000000000000000000000000000000000000000111100000000000000000000000000
00000000000000000000000000000000000000000000000000000000000000000000000000000000000
00000000000000000000000000000000000000000000000000001111

>Q8A1H2_2hqy_0_304
MIPFKDITLADRDTITAFTMKSDRRNCDLSFSNLCSWRFLYDTQFAVIDDFLVFKFWAGEQL
AYMMPVGNGDLKAVLRKLIEDADKEKHNFCMLGVCSNMRADLEAILPERFIFTEDRAYAD
YIYLRSDLATLKGKKFQAKRNHINRFRNTYPDYEYTPITPDRIQECLDLEAEWCKVNNCDQ
QEGTGNERRALIYALHNFEALGLTGGILHVNGKIVAFTFGMPINHETFGVHVEKADTSIDG
AYAMINYEFANRIPEQYIYINREEDLGIEGLRKAKLSYQPVTILEKYMACLKDHPMDMVRW
00000000000000000000000000000000000000000000000000000000000000000000000000000000000
00000000000000000000000000000000000000000000000000000000000000000000000000000000000
00000000000000000000000000000000000000000000000000000000000000000000000000000000000
00000000000000000000000000000000000000000000000001111111
>Q8A3Q9_3kst_25_329
TDDEEKDNNGQGSVSVETNYLPIADPYVMFYNNKYYAYGTGGTTAGEGFACFSSDDLKN
WKREGQALSATDSYGTWGFWAPEVYYVESKKKFYLFYSAEEHICVATSTTPEGPFRQEVKQ
PIWSEKSIDTSLFIDDDGTPYLYFVRFTDGNVIWVAQMTDDLMSIKTETLNQCIKAEVSWELL
QGKVAEGPSLLKKNGVYYLIYSANHYENKGYGVGYATSDTPMGPWVKYSKNPLLQGDAA
TGLVGTGHGAPFQCKDGSWKYIFHAHWSAAEIQPRTSYIKDFAISDQGVVTISGTVIKPRVL
K
11111111111111000000000000000000000000000000000000000000000000000000000000000000000
00000000000000000000000000000000000000000000000000000000000000000000000000000000000
00000000000000000000000000000000000000000000000000000000000000000000000000000000000
00000000000000000000000000000000000000000000000000000000
>Q96P11_2b9e_124_428
GSRPGPASQLPRFVRVNTLKTCSDDVVDYFKRQGFSYQGRASSLDDLRALKGKHFLLDPLM
PELLVFPAQTDLHEHPLYRAGHLILQDRASCLPAMLLDPPPGSHVIDACAAPGNKTSHLAA
LLKNQGKIFAFDLDAKRLASMATLLARAGVSCCELAEEDFLAVSPSDPRYHEVHYILLDPSC
SGSGMPSRQLEEPGAGTPSPVRLHALAGFQQRALCHALTFPSLQRLVYSTCSLCQEENEDV
VRDALQQNPGAFRLAPALPAWPHRGLSTFPGAEHCLRASPETTLSSGFFVAVIERVEVPR
11111111000000000000000000000000000000000000000000000000000000000000000000000000000
00000000000000000000000000000000000000000000000000000000000000000000000000000000000
00000000000000000000111111111111111111000000000000000000000000000000000000000000000
00000000000000000000000000000000000000000000000000001111
>Q9U8X2_3q1x_0_304
MDNYIYSIAHQLYEMYLQDEDAFHSKRDYPHKKVFTELQKLRKIFFPDFFMKHQKITESHIA
SELTKLVDYIKDSVTAYNDELFAHQCVMAILEKLPSIKRTLKTDLIAAYAGDPAAPGLSLIIRC
YPGFQAVIVYRIAHVLYECGERYYCREMMESVHSYTSIDIHPGASIKGHFFIDHGVGVVIGET
AIIGEWCRIYQSVTLGAMHFQEEGGVIKRGTKRHPTVGDYVTIGTGAKVLGNIIVGSHVRIG
ANCWIDRDVDSNQTVYISEHPTHFVKPCTTKGMKNDTEIIAIIPSSPLANSPSI
00000000000000000000000000000000000000000000000000000000000000000000000000000000000
00000000000000000000000000000000000000000000000000000000000000000000000000000000000
00000000000000000000000000000000000000000000000000000000000000000000000000000000000
00000000000000000011111111111111111111111111111111111111
>Q9WXV1_1vhn_1_305
EVKVGLAPMAGYTDSAFRTLAFEWGADFAFSEMVSAKGFLMNSQKTEELLPQPHERNVAV
QIFGSEPNELSEAARILSEKYKWIDLNAGCPVRKVVKKGAGGALLKDLRHFRYIVRELRKSV
SGKFSVKTRLGWEKNEVEEIYRILVEEGVDEVFIHTRTVVQSFTGRAEWKALSVLEKRIPTFV
SGDIFTPEDAKRALEESGCDGLLVARGAIGRPWIFKQIKDFLRSGKYSEPSREEILRTFERHLE
LLIKTKGERKAVVEMRKFLAGYTKDLKGARRFREKVMKIEEVQILKEMFYNFIKEV
10000000000000000000000000000000000000000000000000000000000000000000000000000000000
00000000000000000000000000000000000000000000000000000000000000000000000000000000000
00000000000000000000000000000000000000000000000000000000000000000000000000000000000
00000000000000000000000000000000000000000000000000000000

>Q74AQ6_3hc1_0_303
MDISGRDVETRLTLAREFMSGVDELPTVPDIVLRIAGKLNDPDVAIDEVADLLLQDQVLTAR
VVHLANSPLYSAARPISSIRDAVIYLGLDLLREAIFTCAIVDLFKTGKGPLNRSTLWAHSLGV
ARIAKLIAERTGFLNPVNVYVAGLLHDVGEVFINFFRGKEFSQVVTLVDEEKITFGQAEERLF
GTSHCEVGFALAKRWSLNEFICDTILYHHDIEAVPYKQAAIVAMVAFADEYCTLRRLGFEG
HKPVDSVRTLLENHPSWGVIRRSLGGSDFDEKLIVAELDSSIVEIRAAVDELFLL
1111110000000000000000000000000000000000000000000000000000000000000000000000000000000000000
0000000000000000000000000000000000000000000000000000000000000000000000000000000000000000000
0000000000000000000000000000000000000000000000000000000000000000000000000000000000000000000
000000000000000000000000000000000000000000000000000000000000
>Q7Y442_3shs_0_303
MAFTVSIQSNKRCFLAGDGFTLTATVAGDEPLPSNLTYTWTKDDQPHENNTATLTVADATS
ENAGSYKVTVQDTDTMTSVESEVFLMEEAELIVNITEPQHFYVSSQTDVELHATVKFSGGKT
PADNYELHYSWSKGEDVIDTTQDITIQEFTADKNGVYTVKVWGESEDSAASASTKIMLATM
NVDQDVVESKTVALGNEISLNYVVSEDIVGDSSGMPNLTIKYNWYLQREGQLSPTLIGSEVG
EALEGFSITPDGHLFKESATYDDTAKFWCVAKLYQQIEDETVEVAASTSRKCSMEVVK
1000000000000000000000000000000000000000000000000000000000000000000000000000000000000000000
0000000000000000000000000000000000000000000000000000000000000000000000000000000000000000000
0000000000000000000000000000000000000000000000000000000000000000000000000000000000000000000
000000000000000000000000000000000000000000000000000000000000
>Q9RZ04_1woh_0_303
MSGPAHLPYGGIPTFARAPLVQPDGDWQADVAALGVPFDIALGFRPGARFAPRALREASLR
SVPPFTGLDGKTRLQGVTFADAGDVILPSLEPQLAHDRITEAARQVRGRCRVPVFLGGDHS
VSYPLLRAFADVPDLHVVQLDAHLDFTDTRNDTKWSNSSPFRRACEALPNLVHITTVGLR
GLRFDPEAVAAARARGHTIIPMDDVTADLAGVLAQLPRGQNVYFSVDVDGFDPAVIPGTSS
PEPDGLTYAQGMKILAAAAANNTVVGLDLVELAPNLDPTGRSELLMARLVMETLCEVFDH
V
1100000000000000000000000000000000000000000000000000000000000000000000000000000000000000000
0000000000000000000000000000000000000000000000000000000000000000000000000000000000000000000
0000000000000000000000000000000000000000000000000000000000000000000000000000000000000000000
000000000000000000000000000000000000000000000000000000000000
>O29201_3df7_1_303
KLFLFEFATCGERIEDSTAVEGLAMFKSAFDGFKNYYEITGFVRPEFSCLFTLPVDSMDSMEK
YLEKSDAFLIIAPEDDFLLYTLTKKAEKYCENLGSSSRAIAVTSDKWELYKKLRGEVQVPQTS
LRPLDCKFIIKPRTACAGEGIGFSDEVPDGHIAQEFIEGINLSVSLAVGEDVKCLSVNEQIINN
FRYAGAVVPARISDEVKREVVEEAVRAVECVEGLNGYVGVDIVYSDQPYVIEINARLTTPVV
AFSRAYGASVADLLAGGEVKHVRRQMVRKSKSAEKPYVSVGDYTLEIIDLD
0000000000000000000000000000000000000000000000000000000000000000000000000000000000000000000
0000000000000000000000000000000000000000000000000000000000000000000111100000000000000000000
0000000000000000000000000000000000000000000000000000000000000000000000000000000000000000000
00000000000000000000000000000000000000000000000000000000000
>O50426_1q7t_0_302
MSETPRLLFVHAHPDDESLSNGATIAHYTSRGAQVHVVTCTLGEEGEVIGDRWAQLTADH
ADQLGGYRIGELTAALRALGVSAPIYLGGAGRWRDSGMAGTDQRSQRRFVDADPRQTVG
ALVAIIRELRPHVVVTYDPNGGYGHPDHVHTHTVTTAAVAAAGVGSGTADHPGDPWTVP
KFYWTVLGLSALISGARALVPDDLRPEWVLPRADEIAFGYSDDGIDAVVEADEQARAAKV
AALAAHATQVVVGPTGRAAALSNNLALPILADEHYVLAGGSAGARDERGWETDLLAGL
GFTASGT
0000000000000000000000000000000000000000000000000000000000000000000000000000000000000000000
0000000000000000000000000000000000000000000000000000000000000000000000000000000000000000011
1111000000000000000000000000000000000000000000000000000000000000000000000000000000000000000

000000000000000000000000000000000000000000000000000000
>P07774_2h98_0_302
MELRHLRYFVTVVEEQSISKAAEKLCIAQPPLSRQIQKLEEELGIQLFERGFRPAKVTEAGMF
FYQHAVQILTHTAQASSMAKRIATVSQTLRIGYVSSLLYGLLPEIIYLFRQQNPEIHIELIECGT
KDQINALKQGKIDLGFGRLKITDPAIRRIVLHKEQLKLAIHKHHHLNQFAATGVHLSQIIDE
PMLLYPVSQKPNFATFIQSLFTELGLVPSKLTEIREIQLALGLVAAGEGVCIVPASAMDIGVKN
LLYIPILDDDAYSPISLAVRNMDHSNYIPKILACVQEVFATHHIRPLIE
11111111111111111111111111111111111111111111111111111111111111111111111111111111111
11111000000000000000000000000000000000000000000000000000000000000000000000000000000
00000000000000000000000000000000000000000000000000000000000000000000000000000000000
000000000000000000000000000000000000000000000000000000
>P44862_1nri_0_302
MNDIILKSLSTLITEQRNPNSVDIDRQSTLEIVRLMNEEDKLVPLAIESCLPQISLAVEQIVQAF
QQGGRLIYIGAGTSGRLGVLDASECPPTFGVSTEMVKGIIAGGECAIRHPVEGAEDNTKAVL
NDLQSIHFSKNDVLVGIAASGRTPYVIAGLQYAKSLGALTISIASNPKSEMAEIADIAIETIVG
PEILTGSSRLKSGTAQKMVLNMLTTASMILLGKCYENLMVDVQASNEKLKARAVRIVMQA
TDCNKTLAEQTLLEADQNAKLAIMMILSTLSKSEAKVLLERHQGKLRNALSK
11111110000000000000000000000000000000000000000000000000000000000000000000000000000
00000000000000000000000000000000000000000000000000000000000000000000000000000000000
00000000000000000000000000000000000000000000000000000000000000000000000000000000000
000000011111111111111111111111111111111111111111111111
>Q52QI9_4h6v_0_302
MNRDILRTLSLKGDHNIRVAILDGPVDIAHPCFQGADLTVLPTLAPTAARSDGFMSAHGTH
VASIIFGQPETSVPGIAPQCRGLIVPIFSDDRRRITQLDLARGIERAVNAGAHIINISGGELTDF
GEADGWLENAVSLCRQNNVLLVAAAGNNGCDCLHVPAALPAVLAVGAMDDHGHPLDF
SNWGSTYEQQGILAPGEDILGAKPGGGTERLSGTSFATPIVSGVAALLLSEQVRRGETPDPQ
KVRQLLLQSALPCDDDAPEQARRCLAGRLNVSGAFTLLKGGNMSEELATASFPSVEAS
11111111110000000000000000000000000000011111000000000000000000000000000000000000000
00000000000000000000000000000000000000000000000000000000000000000000000000000000000
00000000000000000000000000000000000000000000000000000000000000000000000000000000000
000000000000000000000000000000000000000111111111111111
>Q5SMG9_2cwc_0_302
MKGLKERQDRRLGAFLGLAVGDALGAQVEGLPKGTFPEVREMKGGGPHRLPPGFWTDDT
SQALCLAESLLQRGFDPKDQMDRYLRWYREGYRSATGVCFGLGHATRRALERYAATGDPY
AGDEAGAGNGPLMRLAPLVLAYENHPDLLSLARRAARTTHGAREALEATEVLAWLLREA
LRGAPKEALLALEPFRGADLHPALRRVVEGGFWEAPEEGPGYAPGTLAAALWAFARGRDF
EEGMRLAVNLGGDADTVGAVYGQLAGAYYGLGAIPGRWLRALHLREEMEALALALYRMS
MASPRE
11110000000000000000000000000000000000000000000000000000000000000000000000000000000
00000000111111111111000000000000000000000000000000000000000000000000000000000000000
00000000000000000000000000000000000000000000000000000000000000000000000000000000000
000000000000000000000000000000000000000000000000000111
>Q976W8_3aio_0_302
MIDPKIKKLLESTIQLPIGKASVEEIRSLFKQFSSLTPREEVGKIEDITIPGSETNIKARVYYPKTQ
GPYGVLVYYHGGGFVLGDIESYDPLCRAITNSCQCVTISVDYRLAPENKFPAAVVDSFDALK
WVYNNSEKFNGKYGIAVGGDSAGGNLAAVTAILSKKENIKLKYQVLIYPAVSFDLITKSLYD
NGEGFFLTREHIDWFGQQYLRSFADLLDFRFSPILADLNDLPPALIITAEHDPLRDQGEAYA
NKLLQSGVQVTSVRFNNVIHGFVSFFPFIEQGRDAIGLIGYVLRKVFYGK
11111111111111111111000000000000000000000000000000000000000000000000000000000000000
00000000000000000000000000000000000000000000000000000000000000000000000000000000000
00000000000000000000000000000000000000000000000000000000000000000000000000000000000

00000000000000000000000000000000000000000000000000000000000000
>Q9BY49_1yxm_0_302
MASWAKGRSYLAPGLLQGQVAIVTGGATGIGKAIVKELLELGSNVVIASRKLERLKSAADE
LQANLPPTKQARVIPIQCNIRNEEEVNNLVKSTLDTFGKINFLVNNGGGQFLSPAEHISSKG
WHAVLETNLTGTFYMCKAVYSSWMKEHGGSIVNIIVPTKAGFPLAVHSGAARAGVYNLTK
SLALEWACSGIRINCVAPGVIYSQTAVENYGSWGQSFFEGSFQKIPAKRIGVPEEVSSVVCFLL
SPAASFITGQSVDVDGGRSLYTHSYEVPDHDNWPKGAGDLSVVKKMKETFKEKAKL
11111100000000000000000000000000000000000000000000000000000000000000000000000000
00000000000000000000000000000000000000000000000000000000000000000000000000000000
00000000000000000000000000000000000000000000000000000000000000000000000000000000
00000000000000000000000000000000000000000000000000000000000000
>O66555_2yv5_0_301
MGKKELKRGLVVDREAQMIGVYLFEDGKTYRGIPRGKVLKKTKINAGDYVWGEVVDPNT
FAIEEVEERKNLLIRPKVANVDRVIIVETLKMPEFNNYLLDNMLVVYEYFKVEPVIVFNKIDL
LNEEEKKELERMDSIYRDAGYDVLKVSAKTGEGIDELVDYLEGFICILAGPSGVGKSSILSRLT
GEELRTQEVSEKTERGRHTTTGVRLIPFGKGSFVGDTPGFSKVEATMFVKPREVRNYFREFL
RYQCKYPDCTHTNEPGCAVKEAVKNGEISCERYKSYLKIIKVYLEEIKELCRED
00000000000000000000000000000000000000000000000000000000000000000000000000000000
00000000000000000000000000000000000000000000000000000000000000000000000000000000
00000000000000000000000000001111111110000000000000000000000000000000000000000000
0000000000000000000000000000000000000000000000000000000000000
>P0AEP3_2e3d_0_301
MAAINTKVKKAVIPVAGLGTRMLPATKAIPKEMLPLVDKPLIQYVVNECIAAGITEIVLVTH
SSKNSIENHFDTSFELEAMLEKRVKRQLLDEVQSICPPHVTIMQVRQGLAKGLGHAVLCAH
PVVGDEPVAVILPDVILDEYESDLSQDNLAEMIRRFDETGHSQIMVEPVADVTAYGVVDCK
GVELAPGESVPMVGVVEKPKADVAPSNLAIVGRYVLSADIWPLLAKTPPGAGDEIQLTDAI
DMLIEKETVEAYHMKGKSHDCGNKLGYMQAFVEYGIRHNTLGTEFKAWLEEEMGIKK
11110000000000000000000000000000000000000000000000000000000000000000000000000000
11110000000000000000000000000000000000000000000000000000000000000000000000000000
00000000000000000000000000000000000000000000000000000000000000000000000000000000
0000000000000000000000000000000000000000000000000000000001111
>P19623_2o07_0_301
MEPGPDGPAASGPAAIREGWFRETCSLWPGQALSLQVEQLLHHRRSRYQDILVFRSKTYGN
VLVLDGVIQCTERDEFSYQEMIANLPLCSHPNPRKVLIIGGGDGGVLREVVKHPSVESVVQC
EIDEDVIQVSKKFLPGMAIGYSSSKLTLHVGDGFEFMKQNQDAFDVIITDSSDPMGPAESLF
KESYYQLMKTALKEDGVLCCQGECQWLHLDLIKEMRQFCQSLFPVVAYAYCTIPTYPSGQI
GFMLCSKNPSTNFQEPVQPLTQQQVAQMQLKYYNSDVHRAAFVLPEFARKALNDVS
11111111111111000000000000000000000000000000000000000000000000000000000000000000
00000000000000000000000000000000000000000000000000000000000000000000000000000000
00000000000000000000000000000000000000000000000000000000000000000000000000000000
0000000000000000000000000000000000000000000000000000000000011
>Q7WBE0_3ocj_0_301
MSPSPLISHRAASADLAGMVRQARQRILLQGNVPGFDVARQIELLHGLAESELGRFLLLYR
GLNAEWTHRLVTHQPGSGALAPLERVFYERLPAVLATRERHGHFRRALQRHLRPGCVVAS
VPCGWMSELLALDYSACPGVQLVGIDYDPEALDGATRLAAGHALAGQITLHRQDAWKLD
TREGYDLLTSNGLNIYEPDDARVTELYRRFWQALKPGGALVTSFLTPPPALSPDSPWDMQAI
DPHDLQLQQLVFTRLIQPRWNALRTHAQTRAQLEEAGFTDLRFEDDRARLFPTVIARKPA
00001110000000000000000000000000000000000000000000000000000000000000000000000000
00000000000000000000000000000000000000000000000000000000000000000000000000000000
00000000000000000000000000000000000000000000000000000000000000000000000000000000
0000000000000000000000000000000000000000000000000000000000000

>Q8KDR9_3kd6_1_302
SLLVIGSLAFDDIETPFGRSDNTLGGSSTYIALSASYFTDEPIRMVGVVGSDFGKEHFDLLHA
KNIDTRGIQVIEDGKTFRWAGRYHYDMNTRDTLDTQLNVFAEFDPHVPQYYRDSKFVCLG
NIDPELQLKVLDQIDDPKLVVCDTMNFWIEGKPEELKKVLARVDVFIVNDSEARLLSGDPN
LVKTARIIREMGPKTLIIKKGEHGALLFTDNGIFAAPAFPLESIYDPTGAGDTFAGGFIGHLAR
CGNTSEAEMRKAVLYGSAMASFCVEQFGPYRYNDLDLLEVDDRYQSFLELSRIE
00000000000000000000000000000000000000000000000000000000000000000000000000000000000
00000000000000000000000000000000000000000000000000000000000000000000000000000000000
00000000000000000000000000000000000000000000000000000000000000000000000000000000000
00000000000000000000000000000000000000000000000000111
>Q8X225_1ml9_29_330
IRSFATHAQLPISIVNREDDAFLNPNFRFIDHSIIGKNVPVADQSFRVGCSCASDEECMYSTC
QCLDEMAPDSDEEADPYTRKKRFAYYSQGAKKGLLRDRVLQSQEPIYECHQGCACSKDCP
NRVVERGRTVPLQIFRTKDRGWGVKCPVNIKRGQFVDRYLGEIITSEEADRRRAESTIARRK
DVYLFALDKFSDPDSLDPLLAGQPLEVDGEYMSGPTRFINHSCDPNMAIFARVGDHADKHI
HDLALFAIKDIPKGTELTFDYVNGLTGLESDAHDPSKISEMTKCLCGTAKCRGYLW
11111111000000000000000000000000000000000000000000000000000000000001111111111
00000000000000000000000000000000000000000000000000000000000000000000000000000000000
00000000000000000000000000000000000000000000000000000000000000000000000000000000000
00000000000000000000111111111111100000000001111111111
>Q8ZWV0_1tzc_0_301
MSQLLQDYLNWENYILRRVDFPTSYVVEGEVVRIEAMPRLYISGMGGSGVVADLIRDFSLT
WNWEVEVIAVKDYFLKARDGLLIAVSYSGNTIETLYTVEYAKRRRIPAVAITTGGRLAQMGV
PTVIVPKASAPRAALPQLLTAALHVVAKVYGIDVKIPEGLEPPNEALIHKLVEEFQKRPTIIA
AESMRGVAYRVKNEFNENAKIEPSVEILPEAHHNWIEGSERAVVALTSPHIPKEHQERVKAT
VEIVGGSIYAVEMHPKGVLSFLRDVGIASVKLAEIRGVNPLATPRIDALKRRLQ
10000000000000000000000000000000000000000000000000000000000000000000000000000000000
00000000000000000000000000000000000000000000000000000000000000000000000000000000000
00000000000000000000000000000000000000000000000000000000000000000000000000000000000
00000000000000000000000000000000000000000000000000000
>A0QAF4_3qlj_0_300
MGVVDGRVVIVTGAGGGIGRAHALAFAAEGARVVVNDIGVGLDGSPASGGSAAQSVVDE
ITAAGGEAVADGSNVADWDQAAGLIQTAVETFGGLDVLVNNAGIVRDRMIANTSEEEFDA
VIAVHLKGHFATMRHAAAYWRGLSKAGKAVDGRIINTSSGAGLQGSVGQGNYSAAKAGI
ATLTLVGAAEMGRYGVTVNAIAPSARTRMTETVFAEMMATQDQDFDAMAPENVSPLVVW
LGSAEARDVTGKVFEVEGGKIRVAEGWAHGPQIDKGARWDPAELGPVVADLLGKARPPVP
VYGA
00000000000000000000000000000000000000000000000000000000000000000000000000000000000
00000000000000000000000000000000000000000000000000000000000000000000000000000000000
00000000000000000000000000000000000000000000011111111111000000000000000000000000000
0000000000000000000000000000000000000000000000000000
>C4ZBG5_3s25_29_329
LNSSYVNGNTAGNLYNAGLFCESDGEVFFSNTNDNGRLYAMNIDGSNIHKLSNDTAMYIN
ADKNYVYYVRNNNQKITSQTFFSYDRNSLCRIKRNGHGSTVLDPDPCIYASLIGNYIYYLHY
DTQTATSLYRIRIDGEEKKKIKNHYLFTCNTSDRYFYYNNPKNGQLYRYDTASQSEALFYDC
NCYKPVVLDDTNVYYMDVNRDNAIVHVNINNPNPVVLTEANIEHYNVYGSLIFYQRGGD
NPALCVVKNDGTGFKELAKGEFCNINVTSQYVYFTDFVSNKEYCTSTQNPDTIKALQP
00000000000000000000000000000000000000000000000000000000000000000000000001111110000
00000000000000000000000000000000000000000000000000000000000000000000000000000000000
00000000000000000000000000000000000000000000000000000000000000000000000000000000000
0000000000000000000000000000000000000000000000000000

>P12255_1rwr_71_371
QGLVPQGQTQVLQGGNKVPVVNIADPNSGGVSHNKFQQFNVANPGVVFNNGLTDGVSRI
GGALTKNPNLTRQASAILAEVTDTSPSRLAGTLEVYGKGADLIIANPNGISVNGLSTLNASN
LTLTTGRPSVNGGRIGLDVQQGTVTIERGGVNATGLGYFDVVARLVKLQGAVSSKQGKPLA
DIAVVAGANRYDHATRRATPIAAGARGAAAGAYAIDGTAAGAMYGKHITLVSSDSGLGVR
QLGSLSSPSAITVSSQGEIALGDATVQRGPLSLKGAGVVSAGKLASGGGAVNVAGGGAV
00000000000000000000000000000000000000000000000000000000000000000000000000000000000
00000000000000000000000000000000000000000000000000000000000000000000000000000000000
00000000000000000000000000000000000000000000000000000000000000000000000000000000000
000000000000000000000000000000000000000000000001111
>P14756_3dbk_197_497
AEAGGPGGNQKIGKYTYGSDYGPLIVNDRCEMDDGNVITVDMNSSTDDSKTTPFRFACPT
NTYKQVNGAYSPLNDAHFFGGVVFKLYRDWFGTSPLTHKLYMKVHYGRSVENAYWDGTA
MLFGDGATMFYPLVSLDVAAHEVSHGFTEQNSGLIYRGQSGGMNEAFSDMAGEAAEFYM
RGKNDFLIGYDIKKGSGALRYMDQPSRDGRSIDNASQYYNGIDVHHSSGVYNRAFYLLAN
SPGWDTRKAFEVFVDANRYYWTATSNYNSGACGVIRSAQNRNYSAADVTRAFSTVGVTCP
SAL
00000000000000000000000000000000000000000000000000000000000000000000000000000000000
00000000000000000000000000000000000000000000000000000000000000000000000000000000000
00000000000000000000000000000000000000000000000000000000000000000000000000000000000
000000000000000000000000000000000000000000000000111
>P20581_2q0i_0_300
MLRLSAPGQLDDDLCLLGDVQVPVFLLRLGEASWALVEGGISRDAELVWADLCRWVADPS
QVHYWLITHKHYDHCGLLPYLCPRLPNVQVLASERTCQAWKSESAVRVVERLNRQLLRAE
QRLPEACAWDALPVRAVADGEWLELGPRHRLQVIEAHGHSDDHVVFYDVRRRRLFCGDA
LGEFDEAEGVWRPLVFDDMEAYLESLERLQRLPTLLQLIPGHGGLLRGRLAADGAESAYTE
CLRLCRRLLWRQSMGESLDELSEELHRAWGGQSVDFLPGELHLGSMRRMLEILSRQALPLD
00000000000000000000000000000000000000000000000000000000000000000000000000000000000
00000000000000000000000000000000000000000000000000000000000000000000000000000000000
00000000000000000000000000000000000000000000000000000000000000000000000000000000000
000000000000000000000000000000000000000000000000111
>Q00511_3pkf_1_301
SAVKAARYGKDNVRVYKVHKDEKTGVQTVYEMTVCVLLEGEIETSYTKADNSVIVATDSIK
NTIYITAKQNPVTPPELFGSILGTHFIEKYNHIHAAHVNIVCHRWTRMDIDGKPHPHSFIRD
SEEKRNVQVDVVEGKGIDIKSSLSGLTVLKSTNSQFWGFLRDEYTTLKETWDRILSTDVDAT
WQWKNFSGLQEVRSHVPKFDATWATAREVTLKTFAEDNSASVQATMYKMAEQILARQQL
IETVEYSLPNKHYFEIDLSWHKGLQNTGKNAEVFAPQSDPNGLIKCTVGRSSLKSKL
00000000000000000000000000000000000000000000000000000000000000000000000000000000000
00000000000000000000000000000000000000000000000000000000000000000000000000000000000
00000000000000000000000000000000000000000000000000000000000000000000000000000000000
000000000000000000000000000000000000000000000111111
>Q24368_1ofc_690_990
ERKANYAVDAYFREALRVSEPKAPKAPRPPKQPIVQDFQFFPPRLFELLDQEIYYFRKTVGYK
VPKNTELGSDATKVQREEQRKIDEAEPLTEEEIQEKENLLSQGFTAWTKRDFNQFIKANEKY
GRDDIDNIAKDVEGKTPEEVIEYNAVFWERCTELQDIERIMGQIERGEGKIQRRLSIKKALD
QKMSRYRAPFHQLRLQYGNNKGKNYTEIEDRFLVCMLHKLGFDKENVYEELRAAIRASPQ
FRFDWFIKSRTALELQRRCNTLITLIERENIELEEKERAEKKKKAPKGSVSAGS
11111100000000001111111100000000000000000000000000000000000000000001111111000000000
00000000000000000000000000000000000000000000000000000000000000000000000000000000000
00000000000000000000000000000000000000000000000000000000000000000000000000000000000
000000000000000000000000000000000000001111111111111

>Q5SM23_1wf3_0_300
MAEKTYSGFVAIVGKPNVGKSTLLNNLLGVKVAPISPRPQTTRKRLRGILTEGRRQIVFVDTP
GLHKPMDALGEFMDQEVYEALADVNAVVWVVDLRHPPTPEDELVARALKPLVGKVPILL
VGNKLDAAKYPEEAMKAYHELLPEAEPRMLSALDERQVAELKADLLALMPEGPFFYPEDY
AKSDQTFGEWVAEILREEAMKRLWHEVPYAVATKVEEVAERENGVLYIKAILYVERPSQKAI
VIGEGGRKIKEIGQATRKQLEALLGKKVYLDLEVKVYPDWRKDPEALRELGYRSSVG
11000000000000000000000000000000000000000000000000000000000000000000000000000000000
00000000000000000000000000000000000000000000000000000000000000000000000000000000000
00000000000000000000000000000000000000000000000000000000000000000000000000000000000
0000000000000000000000000000000000000000000000000111
>Q63K37_3nft_9_309
ALTVRDWPALEALAKTMPADAGARAMTDDDLRAAGVDRRVPEQKLGAAIDEFASLRLPD
RIDGRFVDGRRANLTVFDDARVAVRGHARAQRNLLERLETELLGGTLDTAGDEGGIQPDPI
LQGLVDVIGQGKSDIDAYATIVEGLTKYFQSVADVMSKLQDYISAKDDKNMKIDGGKIKALI
QQVIDHLPTMQLPKGADIARWRKELGDAVSISDSGVVTINPDKLIKMRDSLPPDGTVWDTA
RYQAWNTAFSGQKDNIQNDVQTLVEKYSHQNSNFDNLVKVLSGAISTLTDTAKSYLQI
11111111111111111111111100000000000000000000000000000000000000000000000000000000000
00000000000000000000111111111000000000000000000000000000000000000000000000000000000
00000000000000000000000000000000000000000000000000000000000000000000000000000000000
0000000000000000000000000000000000000000000000111111
>Q7MW40_3bio_0_300
MTDDKKIRAAIVGYGNIGRYALQALREAPDFEIAGIVRRNPAEVPFELQPFRVVSDIEQLESV
DVALVCSPSREVERTALEILKKGICTADSFDIHDGILALRRSLGDAAGKSGAAAVIASGWDP
GSDSVVRTLMQAIVPKGITYTNFGPGMSMGHTVAVKAIDGVKAALSMTIPLGTGVHRRMV
YVELLPGHNLEEVSAAIKADEYFVHDETHVIQVDEVDALIDMGHGVRMVRKGVSGSTQN
QRMSFDMEINNPALTGQVLVCAARAAMRQQPGAYTLQEIPVIDLLPGDREQWIGKLC
11110000000000000000000000000000000000001111000000000000000000000000000000000000000
00000000000000000000000000000000000000000000000000000000000000000000000000000000000
00000000000000000000000000000000000000000000000000000000000000000000000000000000000
0000000000000000000000000000000000000000000000000000
>Q7W3B7_3d0k_0_300
MKPADLTNADRIALELGHAGRNAIPYLDDDRNADRPFTLNTYRPYGYTPDRPVVVVQHG
VLRNGADYRDFWIPAADRHKLLIVAPTFSDEIWPGVESYNNGRAFTAAGNPRHVDGWTY
ALVARVLANIRAAEIADCEQVYLFGHSAGGQFVHRLMSSQPHAPFHAVTAANPGWYTLPT
FEHRFPEGLDGVGLTEDHLARLLAYPMTILAGDQDIATDDPNLPSEPAALRQGPHRYARAR
HYYEAGQRAAAQRGLPFGWQLQVVPGIGHDGQAMSQVCASLWFDGRMPDAAELARLA
GSQSA
10000000000000000000000000000000000000000000000000000000000000000000000000000000000
00000000000000000000000000000000000000000000000000000000000000000000000000000000000
00000000000000000000000000000000000000000011000000000000000000000000000000000000000
0000000000000000000000000000000000000000000000011111
>F7IX06_3vis_0_299
MSVTTPRRETSLLSRALRATAAAATAVVATVALAAPAQAANPYERGPNPTESMLEARSGPFS
VSEERASRFGADGFGGGTIYYPRENNTYGAIAISPGYTGTQSSIAWLGERIASHGFVVIAIDT
NTTLDQPDSRARQLNAALDYMLTDASSAVRNRIDASRLAVMGHSMGGGGTLRLASQRPD
LKAAIPLTPWHLNKSWRDITVPTLIIGAEYDTIASVTLHSKPFYNSIPSPTDKAYLELDGASHF
APNITNKTIGMYSVAWLKRFVDEDTRYTQFLCPGPRTGLLSDVEEYRSTCPF
11111111111111111111111111111111111111111100000000000000000000000000000000000000000
00000000000000000000000000000000000000000000000000000000000000000000000000000000000
00000000000000000000000000000000000000000000000000000000000000000000000000000000000
000000000000000000000000000000000000000000000000000

>P71571_2irx_0_299
MGSASEQRVTLTNADKVLYPATGTTKSDIFDYYAGVAEVMLGHIAGRPATRKRWPNGVDQ
PAFFEKQLALSAPPWLSRATVAHRSGTTTYPIIDSATGLAWIAQQAALEVHVPQWRFVAEP
GSGELNPGPATRLVFDLDPGEGVMMAQLAEVARAVRDLLADIGLVTFPVTSGSKGLHLYTP
LDEPVSSRGATVLAKRVAQRLEQAMPALVTSTMTKSLRAGKVFVDWSQNSGSKTTIAPYSL
RGRTHPTVAAPRTWAELDDPALRQLSYDEVLTRIARDGDLLERLDADAPVADRLTRY
11111100000000000000000000000000000000000000000000000000000000000000000000000000000
00000000000000000000000000000000000000000000000000000000000000000000000000000000000
00000000000000000000000000000000000000000000000000000000000000000000000000000000000
000000000000000000000000000000000000000011111111111
>Q2TDY4_3emr_0_299
MEDLYPSRQNNQPKILKRKDPVIYTDRSKDNQAPITKEQLDSYEKNGFLQIKNFFSEDEVID
MQKAIFELQDSIKDVASDKVIREPESNDIRSIFHVHQDDNYFQDVANDKRILDIVRHLLGSD
VYVHQSRINYKPGFKGKEFDWHSDFETWHVEDGMPRMRAISVSIALSDNYSFNGPLMLIP
GSHNYFVSCVGETPDNNYKESLKKQKLGVPDEESLRELTRIGGGISVPTGKAGSVTLFESNT
MHGSTSNITPYPRNNLFMVYNSVKNRLVEPFSGGEKRPEYIAVREKQPVYSAVN
00000000000000000000000000000000000000000000000000000000000000000000000000000000000
00000000000000000000000000000000000000000000000000000000000000000000000000000000000
00000000000000000000000000000011111111111111111000000000000000000000000000000000000
000000000000000000000000000000000000000000000001111
>Q57556_3v5u_2_301
ILGVGYFLLGLILLYYGSDWFVLGSERIARHFNVSNFVIGATVMAIGTSLPEILTSAYASYMHA
PGISIGNAIGSCICNIGLVLGLSAIISPIIVDKNLQKNILVYLLFVIFAAVIGIDGFSWIDGVVLLI
LFIIYLRWTVKNGSAEIEENNDKNNPSVVFSLVLLIIGLIGVLVGAELFVDGAKKIALALDISD
KVIGFTLVAFGTSLPELMVSLAAAKRNLGGMVLGNVIGSNIADIGGALAVGSLFMHLPAEN
VQMAVLVIMSLLLYLFAKYSKIGRWQGILFLALYIIAIASLRMG
00000000000000000000000000000000000000000000000000000000000000000000000000000000000
00000000000000000000000000000000000000000000000000000000000000011111110000000000000
00000000000000000000000000000000000000000000000000000000000000000000000000000000000
000000000000000000000000000000000000000000000000000
>Q9RLV9_1zwx_35_334
QASDEYPGNFKITSHNVYLFSRNIYPNWGQMHRADLIAQADYMKNNDVVILNEAFDTSA
SHRLLNNLREMYPHQTPVIGRSKHGWDKTEGNYSNFALEDGGVAVVSQWPIVEKSQHIFQ
RGGGADRLSNKGFAYVKIMKNGKPYHIIGTHTPADDSLISKDTSRAIRAEQMQEIQTFIAKK
NIPKDEIIFIGGDLNVNYGTDEYHDMFKLLNVSSPANFNGQMATWDPTTNSMLKESYPKA
APEYLDYIFVENGHARPHSWHNKVLHTKSPQWSVKSWFKTYTYQDFSDHYPVVGFTDNN
11111000000000000000000000000000000000000000000000000000000000000000000000000000000
00000001111100000000000000000000000000000000000000000000000000000000000000000000000
00000000000000000000000000000000000000000000000000000000000000000000000000000000000
000000000000000000000000000000000000000000000000011
>Q9VMC9_4e14_0_299
MYNPRCKDLDRDYFPSYHTTRFQDQPEPNLAVLEHFVRVTKQHGRELTEKQGITVDHLRY
GEGRQLVDVFYSEKTTNQAPLFVFVHGGYWQEMDMSMSCSIVGPLVRRGYRVAVMDYNL
CPQVTLEQLMTQFTHFLNWIFDYTEMTKVSSLTFAGHSAGAHLLAQILMRPNVITAQRSK
MVWALIFLCGVYDLRELSNLESVNPKNILGLNERNIESVSPMLWEYTDVTVWNSTKIYVVA
AEHDSTTFIEQSRHYADVLRKKGYKASFTLFKGYDHFDIIEETAIDDSDVSRFLRNIEIE
00000000000000000000000000000000000000000000000000000000000000000000000000000000000
00000000000000000000000000000000000000000000000000000000000000000000000000000000000
00000000000000000000000000000000000000000000000000000000000000000000000000000000000
000000000000000000000000000000000000000000000000001
>P0A5R0_3ivx_2_300

IPAFHPGELNVYSAPGDVADVSRALRLTGRRVMLVPTMGALHEGHLALVRAAKRVPGSVV
VVSIFVNPMQFGAGEDLDAYPRTPDDDLAQLRAEGVEIAFTPTTAAMYPDGLRTTVQPGPL
AAELEGGPRPTHFAGVLTVVLKLLQIVRPDRVFFGEKDYQQLVLIRQLVADFNLDVAVVGV
PTVREADGLAMSSRNRYLDPAQRAAAVALSAALTAAAHAATAGAQAALDAARAVLDAA
PGVAVDYLELRDIGLGPMPLNGSGRLLVAARLGTTRLLDNIAIEIGTFAGTDRPDGYRA
00000000000000000000000000000000000000000000000000000000000000000000000000000000000
00000000000000000000000000000000000000000000000000000000000000000000000000000000000
00000000000000000000000000000000000000000000000000000000000000000000000000000000000
00000000000000000000000000000000000000011111111111
>P36037_3odt_1_299
GYQLSATLKGHDQDVRDVVAVDDSKVASVSRDGTVRLWSKDDQWLGTVVYTGQGFLNSV
CYDSEKELLLFGGKDTMINGVPLFATSGEDPLYTLIGHQGNVCSLSFQDGVVISGSWDKTAK
VWKEGSLVYNLQAHNASVWDAKVVSFSENKFLTASADKTIKLWQNDKVIKTFSGIHNDVV
RHLAVVDDGHFISCSNDGLIKLVDMHTGDVLRTYEGHESFVYCIKLLPNGDIVSCGEDRTV
RIWSKENGSLKQVITLPAISIWSVDCMSNGDIIVGSSDNLVRIFSQEKSRWASEDEI
00000000000000000000000000000000000000000000000000000000000000000000000000000000000
00000000000000000000000000000000000000000000000000000000000000000000000000000000000
00000000000000000000000000000000000000000000000000000000000000000000000000000000000
00000000000000000000000000000000000000000000001111
>P43629_3vh8_21_319
HMGGQDKPFLSAWPSAVVPRGGHVTLRCHYRHRFNNFMLYKEDRIHIPIFHGRIFQESFN
MSPVTTAHAGNYTCRGSHPHSPTGWSAPSNPVVIMVTGNHRKPSLLAHPGPLVKSGERVIL
QCWSDIMFEHFFLHKEGISKDPSRLVGQIHDGVSKANFSIGPMMLALAGTYRCYGSVTHTP
YQLSAPSDPLDIVVTGPYEKPSLSAQPGPKVQAGESVTLSCSSRSSYDMYHLSREGGAHERR
LPAVRKVNRTFQADFPLGPATHGGTYRCFGSFRHSPYEWSDPSDPLLVSVTGNPS
11111100000000000000000000000000000000000000000000000000000000000000000000000000000
00000000000000000000000000000000000000000000000000000000000000000000000000000000000
00000000000000000000000000000000000000000000000000000000000000000000000000000000000
00000000000011111000000000000000000000000001111111
>P76407_2bon_0_298
MAEFPASLLILNGKSTDNLPLREAIMLLREEGMTIHVRVTWEKGDAARYVEEARKFGVATV
IAGGGDGTINEVSTALIQCEGDDIPALGILPLGTANDFATSVGIPEALDKALKLAIAGDAIAI
DMAQVNKQTCFINMATGGFGTRITTETPEKLKAALGSVSYIIHGLMRMDTLQPDRCEIRGE
NFHWQGDALVIGIGNGRQAGGGQQLCPNALINDGLLQLRIFTGDEILPALVSTLKSDEDNP
NIIEGASSWFDIQAPHDITFNLDGEPLSGQNFHIEILPAALRCRLPPDCPLLR
11110000000000000000000000000000000000000000000000000000000000000000000000000000000
00000000000000000000000000000000000000000000000000000000000000001111111111000000000
00000000000000000000000000000000000000000000000000000000000000000000000000000000000
00000000000000000000000000000000000000000000000000
>Q5KX27_3aal_0_298
MLKIGSHVSMSGKKMLLAASEEAASYGANTFMIYTGAPQNTKRKSIEELNIEAGRQHMQA
HGIEEIVVHAPYIINIGNTTNLDTFSLGVDFLRAEIERTEAIGAKQLVLHPGAHVGAGVEAG
LRQIIRGLNEVLTREQNVQIALETMAGKGSECGRTFEELAYIIDGVAYNDKLSVCFDTCHTH
DAGYDIVNDFDGVLEEFDRIIGLGRLKVLHINDSKNPRGSRKDRHENIGFGHIGFAALNYIV
HHPQLEDIPKILETPYVGEDKNNKKPPYKHEIAMLRAQSFDDQLLEKINAGAE
10000000000000000000000000000000000000000000000000000000000000000000000000000000000
00000000000000000000000000000000000000000000000000000000000000000000000000000000000
00000000000000000000000000000000000000000000000000000000000000000000000000000000000
00000000000000000000000000000000000000000000000000
>Q96NX5_2jam_17_315
NIRKTFIFMEVLGSGAFSEVFLVKQRLTGKLFALKCIKKSPAFRDSSLENEIAVLKKIKHENIVT

LEDIYESTTHYYLVMQLVSGGELFDRILERGVYTEKDASLVIQQVLSAVKYLHENGIVHRDL
KPENLLYLTPEENSKIMITDFGLSKMEQNGIMSTACGTPGYVAPEVLAQKPYSKAVDCWSIG
VITYILLCGYPPFYEETESKLFEKIKEGYYEFESPFWDDISESAKDFICHLLEKDPNERYTCEKA
LSHPWIDGNTALHRDIYPSVSLQIQKNFAKSKWRQAFNAAAVVHH
00000000000000000000000000000000000000011111100000000000000000000000000000000000000
00000000000000000000000000000000000000000000000000000000000000000000000000000000000
00000000000000000000000000000000000000000000000000000000000000000000000000000000000
00000000000000000000000000000000000011111111111111
>Q96Y14_2e2n_0_298
MMIIVGVDAGGTKTKAVAYDCEGNFIGEGSSGPGNYHNVGLTRAIENIKEAVKIAAKGEAD
VVGMGVAGLDSKFDWENFTPLASLIAPKVIIQHDGVIALFAETLGEPGVVVIAGTGSVVEGY
NGKEFLRVGGRGWLLSDDGSAYWVGRKALRKVLKMMDGLENKTILYNKVLKTINVKDLD
ELVMWSYTSSCQIDLVASIAKAVDEAANEGDTVAMDILKQGAELLASQAVYLARKIGTNKV
YLKGGMFRSNIYHKFFTLYLEKEGIISDLGKRSPEIGAVILAYKEVGCDIKKLISD
00000000000000000000000000000000000000000000000000000000000000000000000000000000000
00000000000000000000000000000000000000000000000000000000000000000000000000000000000
00000000000000000000000000000000000000000000000000000000000000000000000000000000000
00000000000000000000000000000000000000000000000001
>Q9AQQ8_3a55_21_319
DSNGNQEINGKEKLSVNDSKLKDFGKTVPVGIDEENGMIKVSFMLTAQFYEIKPTKENEQYI
GMLRQAVKNESPVHIFLKPNSNEIGKVESASPEDVRYFKTILTKEVKGQTNKLASVIPDVATL
NSLFNQIKNQSCGTSTASSPCITFRYPVDGCYARAHKMRQILMNNGYDCEKQFVYGNLKA
STGTCCVAWSYHVAILVSYKNASGVTEKRIIDPSLFSSGPVTDTAWRNACVNTSCGSASVSSY
ANTAGNVYYRSPSNSYLYDNNLINTNCVLTKFSLLSGCSPSPAPDVSSCGF
11111111111111111110000000000000000000000000000000000000000000000000000000000000000
00000000000000000000000000000000000000000000000000000000000000000000000000000000000
00000000000000000000000000000000000000000000000000000000000000000000000000000000000
00000000000000000000000000000000000000000000000000
>Q9NUX5_3kjo_0_298
MSLVPATNYIYTPLNQLKGGTIVNVYGVVKFFKPPYLSKGTDYCSVVTIVDQTNVKLTCLLF
SGNYEALPIIYKNGDIVRFHRLKIQVYKKETQGITSSGFASLTFEGTLGAPIIPRTSSKYFNFTTE
DHKMVEALRVWASTHMSPSWTLLKLCDVQPMQYFDLTCQLLGKAEVDGASFLLKVWDG
TRTPFPSWRVLIQDLVLEGDLSHIHRLQNLTIDILVYDNHVHVARSLKVGSFLRIYSLHTKLQ
SMNSENQTMLSLEFHLHGGTSYGRGIRVLPESNSDVDQLKKDLESANLTA
11111000000000000000000000000000000000000000000000000000000000000000000000000000000
00000000000000000000000000000000000000000000000000000000000000010000000000000000000
00000000000000000000000000000000110000000000000000000000000000000000000000000000000
00000000000000000000000000000000000000000000000000
>Q9WZX6_1n2x_0_298
MRKYSQRHIPVMVREVIEFLKPEDEKIILDCTVGEGGHSRAILEHCPGCRIIGIDVDSEVLRIA
EEKLKEFSDRVSLFKVSYREADFLLKTLGIEKVDGILMDLGVSTYQLKGENRGFTFEREEPLD
MRMDLESEVTAQKVLNELPEEELARIIFEYGEEKRFARRIARKIVENRPLNTTLDLVKAVREA
LPSYEIRRRKRHFATKTFQAIRIYVNRELENLKEFLKKAEDLLNPGGRIVVISFHSLEDRIVKE
TFRNSKKLRILTEKPVRPSEEEIRENPRARSGRLRAAERIEEGGD
11111110000000000000000000000000000000000000000000000000000000000000000000000000000
00000000000000000000000000000000000000000000000000000000000000000000000000000000000
00000000000000000000000000000000000000000000000000000000000000000000000000000000000
00000000000000000000000000000000000000000000011111
>O29535_1sr8_0_297
MLIDPIELYRYPEKWIKDRDAEKKVRSGLYILTEDGYLRRGITTGTTASAAAVAAIASLKEKV
EKVKVSTPAGVDVEVEVEAEKGFARVRKFSGDHEFDVTNGIIFEAEVCETSGIFFGRGVGVK

AGEKAVSRSAKLQILENFIKASREFNFSGGVRISVPDGEEVAKKTGNEKVGIKGGISILGTTGF
VEPWCKKLVETKLKIAMQYHRIAITTGRKAWLYARKKFPEYQPFVFGVHIDEALKHPGEKII
VGFPGLLKIWAGSRDRIEERAREEGVRVVVIEDDMDSWVWDVQGTDH
00000000000000000000000000000000000000000000000000000000000000000000000000000000000
00000000000000000000000000000000000000000000000000000000000000000000000000000000000
00000000000000000000000000000000000000000000000000000000000000000000000000000000000
0000000000000000000000000000000001111111111111111
>O34530_1t9h_0_297
MPEGKIIKALSGFYYVLDESEDSDKVIQCRGRGIFRKNKITPLVGDYVVYQAENDKEGYLME
IKERTNELIRPPICNVDQAVLVFSAVQPSFSTALLDRFLVLVEANDIQPIICITKMDLIEDQDTE
DTIQAYAEDYRNIGYDVYLTSSKDQDSLADIIPHFQDKTTVFAGQSGVGKSSLLNAISPELGL
RTNEISEHLGRGKHTTRHVELIHTSGGLVADTPGFSSLEFTDIEEEELGYTFPDIREKSSSCKFR
GCLHLKEPKCAVKQAVEDGELKQYRYDHYVEFMTEIKDRKPRY
00000000000000000000000000000000000000000000000000000000000000000000000000000000000
00000000000000000000000000000000000000000000000000000000000000000000000000000000000
00000000000000000000111111111111111111000000000000000000000000000000000000000000000
0000000000000000000000000000000000000000000000000
>P0A1J1_1wlg_71_368
GLDVAISQNGFFRLVDSNGSVFYSRNGQFKLDENRNLVNMQGMQLTGYPATGTPPTIQQG
ANPAPITIPNTLMAAKSTTTASMQINLNSTDPVPSKTPFSVSDADSYNKKGTVTVYDSQGN
AHDMNVYFVKTKDNEWAVYTHDSSDPAATAPTTASTTLKFNENGILESGGTVNITTGTING
ATAATFSLSFLNSMQQNTGANNIVATNQNGYKPGDLVSYQINNDGTVVGNYSNEQEQVL
GQIVLANFANNEGLASQGDNVWAATQASGVALLGTAGSGNFGKLTNGALEASNVDLS
00000000000000000000000000000000000000000000000000000000000000000000000000000000000
00000000000000000000000000000000000000000000000000000000000000000000000000000000000
00000000000000000000000000000000000000000000000000000000000000000000000000000000000
0000000000000000000000000000000000000000000011111
>P27037_3q4t_190_487
PLQLLEVKARGRFGCVWKAQLLNEYVAVKIFPIQDKQSWQNEYEVYSLPGMKHENILQFIG
AEKRGTSVDVDLWLITAFHEKGSLSDFLKANVVSWNELCHIAETMARGLAYLHEDIPGLK
DGHKPAISHRDIKSKNVLLKNNLTACIADFGLALKFEAGKSAGDTHGQVGTRRYMAPEVL
EGAINFQRDAFLRIDMYAMGLVLWELASRCTAADGPVDEYMLPFEEEIGQHPSLEDMQEV
VVHKKKRPVLRDYWQKHAGMAMLCETIEECWDHDAEARLSAGCVGERITQMQRLTNI
00000000000000000000000000000000000000000000000000000000000000000000000000000000000
00000000000000000000000000000000000000000000000000000000000000000000000000000000000
00000000000000000000000000000000000000000000000000000000000000000000000000000000000
0000000000000000000000000000000000000000000000111
>P27305_1nzj_10_307
MTDTQYIGRFAPSPSGELHFGSLIAALGSYLQARARQGRWLVRIEDIDPPREVPGAAETILRQ
LEHYGLHWDGDVLWQSQRHDAYREALAWLHEQGLSYYCTCTRARIQSIGGIYDGHCRVL
HHGPDNAAVRIRQQHPVTQFTDQLRGIIHADEKLAREDFIIHRRDGLFAYNLAVVVDDHF
QGVTEIVRGADLIEPTVRQISLYQLFGWKVPDYIHLPLALNPQGAKLSKQNHAPALPKGDP
RPVLIAALQFLGQQAEAHWQDFSVEQILQSAVKNWRLTAVPESAIVNSTFSNASC
11100000000000000000000000000000000000000000000000000000000000000000000000000000000
00000000000000000000000000000000000000000000000000000000000000000000000000000000000
00000000000000000000000000000000000000000000000000000000011111111111110000000000000
0000000000000000000000000000000000000000111111111
>P50053_2hqq_0_297
MEEKQILCVGLVVLDVISLVDKYPKEDSEIRCLSQRWQRGGNASNSCTVLSLLGAPCAFMG
SMAPGHVADFVLDDLRRYSVDLRYTVFQTTGSVPIATVIINEASGSRTILYYDRSLPDVSATDF
EKVDLTQFKWIHIEGRNASEQVKMLQRIDAHNTRQPPEQKIRVSVEVEKPREELFQLFGYG

DVVFVSKDVAKHLGFQSAEEALRGLYGRVRKGAVLVCAWAEEGADALGPDGKLLHSDAF
PPPRVVDTLGAGDTFNASVIFSLSQGRSVQEALRFGCQVAGKKCGLQGFDGIV
11000000000000000000000000000000000000000000000000000000000000000000000000000000000
00000000000000000000000000000000000000000000000000000000000000000000000000000000000
00000000000000000000000000000000000000000000000000000000000000000000000000000000000
0000000000000000000000000000000000000000000000000
>P62190_1sgv_0_297
MSATGPGIVVIDKPAGMTSHDVVGRCRRIFATRRVGHAGTLDPMATGVLVIGIERATKILGL
LTAAPKSYAATIRLGQTTSTEDAEGQVLQSVPAKHLTIEAIDAAMERLRGEIRQVPSSVSAIK
VGGRRAYRLARQGRSVQLEARPIRIDRFELLAARRRDQLIDIDVEIDCSSGTYIRALARDLGD
ALGVGGHVTALRRTRVGRFELDQARSLDDLAERPALSLSLDEACLLMFARRDLTAAEASAA
ANGRSLPAVGIDGVYAACDADGRVIALLRDEGSRTRSVAVLRPATMHPG
00000000000000000000000000000000000000000000000000000000000000000000000000000000000
00000000000000000000000000000001111111111111111111111111111000000000000000000000000
00000000000000000000000000000000000000000000000000000000000000000000000000000000000
0000000000000000000000000000000000000000000001111
>Q02745_2wml_45_342
ELSENFKKLMKYPYRPCTCTRCIEEQRVSAWFDERFNRSMQPLLTAKNAHLEEDTYKWWL
RLQREKQPNNLNDTIRELFQVVPGNVDPLLEKRLVSCRRCAVVGNSGNLKESYYGPQIDSH
DFVLRMNKAPTEGFEADVGSKTTHHFVYPESFRELAQEVSMILVPFKTTDLEWVISATTGR
ISHTYVPVPAKIKVKKEKILIYHPAFIKYVFDRWLQGHGRYPSTGILSVIFSLHICDEVDLYGF
GADSKGNWHHYWENNPSAGAFRKTGVHDGDFESNVTTILASINKIRIFKGR
11111111111110000000000000000000000000000000000000000000000000000000000000000000000
00000000000000000000000000000000000000000000000000000000000000000000000000000000000
00000000000000000000000000000000000000000000000000000000000000000000000000000000000
0000000000011111111100000000000000000000000000000
>Q54528_1q0r_0_297
MSERIVPSGDVELWSDDFGDPADPALLLVMGGNLSALGWPDEFARRLADGGLHVIRYDHR
DTGRSTTRDFAAHPYGFGELAADAVAVLDGWGVDRAHVVGLSMGATITQVIALDHHDRL
SSLTMLLGGGLDIDFDANIERVMRGEPTLDGLPGPQQPFLDALALMNQPAEGRAAEVAKR
VSKWRILSGTGVPFDDAEYARWEERAIDHAGGVLAEPYAHYSLTLPPPSRAAELREVTVPTL
VIQAEHDPIAPAPHGKHLAGLIPTARLAEIPGMGHALPSSVHGPLAEVILAHTRSAA
10000000000000000000000000000000000000000000000000000000000000000000000000000000000
00000000000000000000000000000000000000000000000000000000000000000000000000000000000
00000000000000000000000000000000000000000000000000000000000000000000000000000000000
0000000000000000000000000000000000000000000000000
>Q57280_2oze_0_297
MIQYYYTKKEWGVVMEKEELKILEELRRILSNKNEAIVILNNYFKGGVGKSKLSTMFAYLTD
KLNLKVLMIDKDLQATLTKDLAKTFKVELPRVNFYEGLKNGNLASSIVHLTDNLDLIPGTF
DLMLLPKLTRSWTFENESRLLATLLAPLKSDYDLIIIDTVPTPSVYTNNAIVASDYVMIPLQAE
EESTNNIQNYISYLIDLQEQFNPGLDMIGFVPYLVDTDSATIKSNLEELYKQHKEDNLVFQNII
KRSNKVSTWSKNGITEHKGYDKKVLSMYKNVFFEMLERIIQLENEKE
11111111111111000000000000000000000000000000000000000000000000000000000000000000000
00000000000000000000000000000000000000000000000000000000000000000000000000000000000
00000000000000000000000000000000000000000000000000000000000000000000000000000000000
0000000000000000000000000000000000000000000000000
>Q88J51_3l6d_4_301
SDESFEFDVSVIGLGAMGTIMAQVLLKQGKRVAIWNRSPGKAAALVAAGAHLCESVKAAL
SASPATIFVLLDNHATHEVLGMPGVARALAHRTIVDYTTNAQDEGLALQGLVNQAGGHY
VKGMIVAYPRNVGHRESHSIHTGDREAFEQHRALLEGLAGHTVFLPWDEALAFATVLHAH
AFAAMVTFFEAVGAGDRFGLPVSKTARLLLETSRFFVADALEEAVRRLETQDFKGDQARLD

VHADAFAHIAQSLHAQGVWTPVFDAVCQVVQRAAAMGYGDQDIAATTKSFAREQEPGH
00000000000000000000000000000000000000000000000000000000000000000000000000000000000
00000000000000000000000000000000000000000000000000000000000000000000000000000000000
00000000000000000000000000000000000000000000000000000000000000000000000000000000000
0000000000000000000000000000000000000000000011111
>Q8PDA8_2nw9_0_297
MPVDKNLRDLEPGIHTDLEGRLTYGGYLRLDQLLSAQQPLSEPAHHDEMLFIIQHQTSELW
LKLLAHELRAAIVHLQRDEVWQCRKVLARSKQVLRQLTEQWSVLETLTPSEYMGFRDVLG
PSSGFQSLQYRYIEFLLGNKNPQMLQVFAYDPAGQARLREVLEAPSLYEEFLRYLARFGHAIP
QQYQARDWTAAHVADDTLRPVFERIYENTDRYWREYSLCEDLVDVETQFQLWRFRHMRT
VMRVIGFKRGTGGSSGVGFLQQALALTFFPELFDVRTSVGVDNRPPQGSADAGKR
11111111111111110000000000000000000000000000000000000000000000000000000000000000000
00000000000000000000000000000000000000000000000000000000000000000000000000000000000
00000000000000000000000000000000000000000000000000000000000000000000000000000000000
0000000000000000000000000000000000000000001111111
>A9CII5_2nwh_0_296
MIETETAPGASNPGSWMEEAGGGGFNAARNLSRLGFEVRIIAPRGGDVTGEVVAEAARQA
GVEDTPFTFLDRRTPSYTAILERDGNLVIALADMDLYKLFTPRRLKVRAVREAIIASDFLLCD
ANLPEDTLTALGLIARACEKPLAAIAISPAKAVKLKAALGDIDILFMNEAEARALTGETAEN
VRDWPNILRKAGLSGGVVTRGASEVVAFNGTEKAILHPPLIREVKDVTGAGDAMASGYLA
AIAEGKTIREALRQGAAAAAITVQSSFATSQDLSKDSVEAMLGLVPQAEMLA
00000000000000000000000000000000000000000000000000000000000000000000000000000000000
00000000000000000000000000000000000000000000000000000000000000000000000000000000000
00000000000000111110000000000000000000000000000000000000000000000000000000000000000
000000000000000000000000000000000000000000000001
>B1MHW4_3r9r_0_296
MRPSLSDYQHVASGKVRELYRVDDEHLLFVATDRISAFDFVLDTPIPDKGRILTAMSVFFFGL
LTVPNHLAGPPDDPRIPEEVLGRALLVRRLDMLPVECVARGYLTGSGLLDYQRTGAVCGHV
LPQGLGEASRLDPPLFTPATKADIGEHDMNVDFAAVVGLVGAVRANQLRDETIKIYTRAAA
HALHKGIILADTKFEFGVDIEGNLVLADEVFTPDSSRYWDAAHYQPGVVQDSFDKQFVRN
WLTGPESGWDRASDTPPPPLPDEVAVATRERYIEAYERISGLSFSDWIGPSA
00000000000000000000000000000000000000000000000000000000000000000000000000000000000
00000000000000000000000000000000000000000000000000000000000000000000000000000000000
00000000000000000000000000000000000000000000000000000000000000000000000000000000000
000000000110000000000000000000000000000000000011
>C6KFA4_4glf_0_296
MTSTAVEITVKNAADIAIIGGSGLYQMQALTNKRSVKIETPYGEPSDDIVLGELNGVTVAFLT
RHGQGHRLTPSEVPYRANIYALKTLGVRYIVSVSAVGSLQETLKPLDMVIPDQMIDMTKQR
VSTFFGDGAVAHVSMADPLCPEVADILIRAYDNADIADGQCHAKATYVCIEGPQFSTRAES
HWYRQMQADIIGMTNMPEAKLAREASIAYATLALVTDFDCWHPNEQAVSADYAIQNLMK
NADNAQQVIKQAVALIASEQPKSIAHTALTQALVTPVEAMSAETKTRLAALLP
11111111110000000000000000000000000000000000000000000000000000000000000000000000000
00000000000000000000000000000000000000000000000000000000000000000000000000000000000
00000000000000000000000000000000000000000000000000000000000000000000000000000000000
000000000000000000000000000000000000000000000000
>P09385_1r4p_22_318
REFTIDFSTQQSYVSSLNSIRTEISTPLEHISQGTTSVSVINHTPPGSYFAVDIRGLDVYQARFD
HLRLIIEQNNLYVAGFVNTATNTFYRFSDFTHISVPGVTTVSMTTDSSYTTLQRVAALERSGM
QISRHSLVSSYLALMEFSGNTMTRDASRAVLRFVTVTAEALRFRQIQREFRQALSETAPVYT
MTPGDVDLTLNWGRISNVLPEYRGEDGVRVGRISFNNISAILGTVAVILNCHHQGARSVRA
VNEESQPECQITGDRPVIKINNTLWESNTAAAFLNRKSQFLYTTGK

0000000000000000000000000000000000000000000000000000000000000000000000000000000000000000000000000000
0000000000000000000000000000000000000000000000000000000000000000000000000000000000000000000000000000
0000000000000000000000000000000000000000000000000000000000000000000000000000000000000000000001111111
1111111110000000000000000000000000000000000000000000000000
>P30967_3tk2_0_296
MNDRADFVVPDITTRKNVGLSHDANDFTLPQPLDRYSAEDHATWATLYQRQCKLLPGRA
CDEFMEGLERLEVDADRVPDFNKLNQKLMAATGWKIVAVPGLIPDDVFFEHLANRRFPVT
WWLREPHQLDYLQEPDVFHDLFGHVPLLINPVFADYLEAYGKGGVKAKALGALPMLARL
YWYTVEFGLINTPAGMRIYGAGILSSKSESIYCLDSASPNRVGFDLMRIMNTRYRIDTFQKTY
FVIDSFKQLFDATAPDFAPLYLQLADAQPWGAGDVAPDDLVLNAGDRQGWADTEDV
1111110000000000000000000000000000000000000000000000000000000000000000000000000000000000000000000000
0000000000000000000000000000000000000000000000000000000000000000000000000000000000000000000000000000
0000000000000000000000000000000000000000000000000000000000000000000000000000000000000000000000000000
0000000000000000000000000000000000000000000011111111111111
>Q03161_2cis_0_296
MPIKETDKEVVLTHPADETTSVHILKYGATVYSWKLKSEEQLWLSTAAKLDGSKPVRGGIPL
VFPVFGKNSTDEHLSKLPQHGLARNSTWEFLGQTKENPPTVQFGLKPEIANPELTKLWPM
DYLLILTVELGSDYLKTAIEVENTSSSKELKFNWLFHTYFRIEDIEGTMVSNLAGMKLYDQLL
KESYVDKHPVVTFNQETDVIYQNVSAERAIQIVDKGVQIHTLKRYNLPDTVVWNPWIEKSQ
GMADFEPKTGYQQMICIEPGHVHDFISLAPGKKWNAYQLLCKEELKYQAIQ
1000000000000000000000000000000000000000000000000000000000000000000000000000000000000000000000000000
0000000000000000000000000000000000000000000000000000000000000000000000000000000000000000000000000000
0000000000000000000000000000000000000000000000000000000000000000000000000000000000000000000000000000
0000000000000000000000000000000000000000000000000011111111
>Q2RUN2_3qxb_0_296
MKLGVNLCFAVKRWLEPDRLAGLVRDDLGLEYVQYTYDLTDPWWPDIERDRRAIAYAKAF
RKAGLTIESTFGGLASYTYNHFLAPTLELQSLGYQHLKRAIDMTAAMEVPATGMPFGSYSA
ADALNPARREEIYAIARDMWIELAAYAKRQGLSMLYVEPVPLATEFPSSAADAARLMADLD
GRTEIPVRLLVDWGHALFEPLFGPEADMDHWMDLCQPWIAAYHIQQTDGQLDRHWSFT
QPGVVTPQRLQDFWDKYALTDQTFFAEILYPFEARDEDVLADMIASVKALKAASPAA
0000000000000000000000000000000000000000000000000000000000000000000000000000000000000000000000000000
0000000000000000000000000000000000000000000000000000000000000000000000000000000000000000000000000000
0000000000000000000000000000000000000000000000000000000000000000000000000000000000000000000000000000
0000000000000000000000000000000000000000000000000000000011
>Q83BY6_3tr9_0_296
MMSKEAFFLKQSFHLRLRDDKRIVFSEPAVMGIINVSPNSFYHPHLDLNSALRTAEKMVDE
GADILDIGGEATNPFVDIKTDSPSTQIELDRLLPVIDAIKKRFPQLISVDTSRPRVMREAVNTG
ADMINDQRALQLDDALTTVSALKTPVCLMHFPSETRKPGSTTHFYFLQSVKKELQESIQRC
KKAGISEDRIIIDPGFGQGNYGKNVSENFYLLNKLPEFVAMGLPVLSGWSRKSMIGDVLNQ
PPENRLFGSIAADVLAVYHGASIIRTHDVKATREAIKIATYTRSVDTIEE
1111111111100000000000000000000000000000000000000000000000000000000000000000000000000000000000111111
0000000000000000000000000000000000000000000000000000000000000000000000000000000000000000000000000000
0000000000000000000000000000000000000000000000000000000000000000000000000000000000000000000000000000
0000000000000000000000000000000000000000000000000000001111
>Q8XBR1_3be6_18_314
EPVQVFTDDLGRKVTVPAHPKRIVSLHDLDITIPLIELGVPPVASHGRTRPDGSHFIRSGALLT
GVDFDNSSIAFIGTADIDIEAIVAAKPDLIITEPTRNTPIERLEKIAPTVSIDHLKGGAPEIYRKL
AELTGTQSQLAILERRYQAQINALKATLDSQKITVSVIQANQGKINVMHSYHSLGRVLRDA
GFRFPPLIESIPEGGRMDVSAERLPELDADFVFATWRGDTGGKPQDELAAMEKVMPGWCQ
FLTACRSGRYVLISREEAISNSFASLGLMAAQIQSQIAGRPLPEAK
0000000000000000000000000000000000000000000000000000000000000000000000000000000000000000000000000000

00000000000000000000000000000000000000000000000000000000000000000000000000000000000
00000000000000000000000000000000000000000000000000000000000000000000000000000000000
000000000000000000000000000000000000000000000111
>A9ANE4_4dlm_0_295
MGALRIDSHQHFWRYRAADYPWIGAGMGVLARDYLPDALHPLMHAQALGASIAVQAR
AGRDETAFLLELACDEARIAAVVGWEDLRAPQLAERVAEWRGTKLRGFRHQLQDEADVR
AFVDDADFARGVAWLQANDYVYDVLVFERQLPDVQAFCARHDAHWLVLDHAGKPALA
EFDRDDTALARWRAALRELAALPHVVCKLSGLVTEADWRRGLRASDLRHIEQCLDAALD
AFGPQRLMFGSDWPVCLLAASYDEVASLVERWAESRLSAAERSALWGGTAARCYALPEPA
DARL
11000000000000000000000000000000000000000000000000000000000000000000000000000000000
00000000000000000000000000000000000000000000000000000000000000000000000000000000000
00000000000000000000000000000000000000000000000000000000000000000000000000000000000
00000000000000000000000000000000000000001111111
>Q92SG7_3mz1_0_295
MRAFLRVVETGNFTRASASLNMPKATVTNLIQGLEAHLRTKLLNRTTRRVLVTPDGALYYE
RAARLLSDLDELDGSLSTAQSLPKGRLRVETASAFANLVIIPALPEFHKKYPDIQIDLGVSDRT
IDYLAENVDCAIRAGTLTDQSLIARRITEMKFVACASRDFLERHPVPQHPSDLEKNCYVVGY
FLPKTGQQMPFHFRRGNEEIEVSGRYTVAANESTTYLAAARAGLGVIQAPLFMVREDLRN
GTMVPVLPDWQVEPMPIYLVYPPNRHLSSRLRVFADWVVKVMAQSQNGE
11111111111111111111111111111111111111111111111111111111111111111111111111111111110
00000000000000000000000000000000000000000000000000000000000000000000000000000000000
00000000000000000000000000000000000000000000000000000000000000000000000000000000000
00000000000000000000000000000000000000000000011
>Q9NRN7_2c43_13_308
MEGVRWAFSCGTWLPSRAEWLLAVRSIQPEEKERIGQFVFARDAKAAMAGRLMIRKLVAE
KLNIPWNHIRLQRTAKGKPVLAKDSSNPYPNFNFNISHQGDYAVLAAEPELQVGIDIMKTS
FPGRGSIPEFFHIMKRKFTNKEWETIRSFKDEWTQLDMFYRNWALKESFIKAIGVGLGFELQ
RLEFDLSPLNLDIGQVYKETRLFLDGEEEKEWAFEESKIDEHHFVAVALRKPDGSRHQDVPS
QDDSKPTQRQFTILNFNDLMSSAVPMTPEDPSFWDCFCFTEEIPIRNGTKS
00000000000000000000000000000000000000000000000000000000000000000000000000000000000
01100000000000000000000000000000000000000000000000000000000000000000000000000000000
00000000000000000000000000000000000000000000000000000000000000000000111111111111111
10000000000000000000000000000000000000000001111
>Q9RFD6_3fwy_1_296
SPKDLTIPTGADGEGSVQVHLDEADKITGAKVFAVYGKGGIGKSTTSSNLSAAFSILGKRVL
QIGCDPKHDSTFTLTGSLVPTVIDVLKDVDFHPEELRPEDFVFEGFNGVMCVEAGGPPAGT
GCGGYVVGQTVKLLKQHHLLDDTDVVIFDVLGDVVCGGFAAPLQHADQAVVVTANDFD
SIYAMNRIIAAVQAKSKNYKVRLAGCVANRSRATDEVDRFCKETNFRRLAHMPDLDAIRRS
RLKKKTLFEMDEDQDVLAARAEYIRLAESLWRGLDPIDPHSLPDRDIFELLGFD
11111111111111111111111111110000000000000000000000000000000000000000000000000000000
00000000000000000000000000000000000000000000000000000000000000000000000000000000000
00000000000000000000000000000000000000000000000000000000000000000000000000000000000
00000000000000000000000000000000000000000000000
>O28163_2jhn_0_294
MWRIELKHAVNWELKMKFFVLPELPTPDVVESGVWRRAIVLDGRAVAVMAYPESERTIVVE
GNFENREWEAVRRKLVEYLGLQNPEELYRFMDGDEKLRMLKNRFYGFGRAGLMSMSVFE
GIAKAIIQQQISFVVAEKLAAKIVGRFGDEVEWNGLKFYGFPTQEAILKAGVEGLRECGLSR
RKAELIVEIAKEENLEELKEWGEEEAYEYLTSFKGIGRWTAELVLSMALGKNVFPADDLGVR
RAVSRLYFNGEIQSAEKVREIARERFGRFARDILFYLFLYDRFFSKKTELV
00000000000000000000000000000000000000000000000000000000000000000000000000000000000

00000000000000000000000000000000000000000000000000000000000000000000000000000000000
00000000000000000000000000000000000000000000000000000000000000000000000000000000000
000000000000000000000000000000000000000011000
>O87275_2qc5_0_294
MNFYLEEFNLSIPDSGPYGITSSEDGKVWFTQHKANKISSLDQSGRIKEFEVPTPDAKVMCLI
VSSLGDIWFTENGANKIGKLSKKGGFTEYPLPQPDSGPYGITEGLNGDIWFTQLNGDRIGKL
TADGTIYEYDLPNKGSYPAFITLGSDNALWFTENQNNSIGRITNTGKLEEYPLPTNAAAPVG
ITSGNDGALWFVEIMGNKIGRITTTGEISEYDIPTPNARPHAITAGKNSEIWFTEWGANQIGR
ITNDKTIQEYQLQTENAEPHGITFGKDGSVWFALKCKIGKLNLNE
00000000000000000000000000000000000000000000000000000000000000000000000000000000000
00000000000000000000000000000000000000000000000000000000000000000000000000000000000
00000000000000000000000000000000000000000000000000000000000000000000000000000000000
000000000000000000000000000000000000000000001
>P17063_4evf_0_294
MPKVTDIANELKQAIDAKDEVQIAFIASEYSAESREKIAKAYVASYGKELPDDIKKALKGGS
EESLLMDLFSDRHEVRAQHIRDALSGRNDHMAFFDTVILCTPEDWHETVAAYTRMFKKPL
VEDFMKDVGRKEDWCLLMEKWMAHERVSRPGSPEDEAQRLDQAFDQKNTAYLIDFFGTV
PSAEYRPIAEAFKAQNGKSIEQAIATIYTKTDYYTFYCAHFALLGMHRLAAYLINCACNDK
GDEKRMRRITGMMVDKCLGAKHAYKIYGDMGTDIERCFDKRMAPILRTLWRVK
10000000000000000000000000000000000000000000000000000000000000000000000000000000000
00000000000000000000000000000000000000000000000000000000000000000000000000000000000
00000000000000000000000000000000000000000000000000000000000000000000000000000000000
000000000000000000000000000000000000000000000
>P46844_1gcu_0_294
MDAEPKRKFGVVVVGVGRAGSVRLRDLKDPRSAAFLNLIGFVSRRELGSLDEVRQISLEDA
LRSQEIDVAYICSESSSHEDYIRQFLQAGKHVLVEYPMTLSFAAAQELWELAAQKGRVLHEE
HVELLMEEFEFLRREVLGKELLKGSLRFTASPLEEERFGFPAFSGISRLTWLVSLFGELSLISATL
EERKEDQYMKMTVQLETQNKGLLSWIEEKGPGLKRNRYVNFQFTSGSLEEVPSVGVNKNIF
LKDQDIFVQKLLDQVSAEDLAAEKKRIMHCLGLASDIQKLCHQKK
00000000000000000000000000000000000000000000000000000000000000000000000000000000000
00000000000000000000000000000000000000000000000000000000000000000000000000000000000
00000000000000000000000000000000000000000000000000000000000000000000000000000000000
000000000000000000000000000000000000000000111
>Q2PYN0_2p18_0_294
MRNYCTKTFGSAFSVTVVPTLKDNFSYLINDHTTHTLAAVDVNADYKPILTYIEEHLKQQG
NADVTYTFSTILSTHKHWDHSGGNAKLKAELEAMNSTVPVVVVGGANDSIPAVTKPVREG
DRVQVGDLSVEVIDAPCHTRGHVLYKVQHPQHPNDGVALFTGDTMFIAGIGAFFEGDEKD
MCRAMEKVYHIHKGNDYALDKVTFIFPGHEYTSGFMTFSEKTFPDRASDDLAFIQAQRAK
YAAAVKTGDPSVPSSLAEEKRQNLFLRVADPAFVAKMNQGNAHALMMYLYNACD
00000000000000000000000000000000000000000000000000000000011111111000000000000000000
00000000000001100000000000000000000000000000000000000000000000000000000000000000000
00000000000000000000000000000000000000000000000000000000000000000000000000000000000
000000000000000000000000000000000000000000011
>Q47898_3ljq_45_339
TTNKPIVLSTWNFGLHANVEAWKVLSKGGKALDAVEKGVRLVEDDPTERSVGYGGRPDR
DGRVTLDACIMDENYNIGSVACMEHIKNPISVARAVMEKTPHVMLVGDGALEFALSQGFK
KENLLTAESEKEWKEWLKTSQYKPIVNIENHDTIGMIALDAQGNLSGACTTSGMAYKMHG
RVGDSPIIGAGLFVDNEIGAATATGHGEEVIRTVGTHLVVELMNQGRTPQQACKEAVERIV
KIVNRRGKNLKDIQVGFIALNKKGEYGAYCIQDGFNFAVHDQKGNRLETPGFALK
00000000000000000000000000000000000000000000000000000000000000000000000000000000000
00000000000000000000000000000000000000000000000000000011111111111000000000000000000

00000000000000000000000000000000000000000000000000000000000000000000000000000000000
0000000000000000000000000000000000000000000000
>Q7D6H9_1zel_20_314
VVSPAGADRRIPTWASRVVSGLARDRPVVVTKEDLTQRLTEAGCGRDPDSAIRELRRIGWLV
QLPVKGTWAFIPPGEAAISDPYLPLRSWLARDQNAGFMLAGASAAWHLGYLDRQPDGRIP
IWLPPAKRLPDGLASYVSVVRIPWNAADTALLAPRPALLVRRRLDLVAWATGLPALGPEAL
LVQIATRPASFGPWADLVPHLDDLVADCSDERLERLLSGRPTSAWQRASYLLDSGGEPARGQ
ALLAKRHTEVMPVTRFTTAHSRDRGESVWAPEYQLVDELVVPLRVIGKA
00000000000000000000000000000000000000000000000000000000000000000000000000000000000
00000000000000000000000000000000000000000000000000000000000000000000000000000000000
00000000000000000000000000000000000000000000000000000000000000000000000000000000000
0000000000000000011100000000000000000000000000
>Q8A6W9_2qhp_0_294
MNNIIVGMGEALWDVLPEGKKIGGAPANFAYHVSQFGFDSRVVSAVGNDELGDEIMEVFK
EKQLKNQIERVDYPTGTVQVTLDDEGVPCYEIKEGVAWDNIPFTDELKRLALNTRAVCFGS
LAQRNEVSRATINRFLDTMPDIDGQLKIFDINLRQDFYTKEVLRESFKRCNILKINDEELVTIS
RMFGYPGIDLQDKCWILLAKYNLKMLILTCGINGSYVFTPGVVSFQETPKVPVADTVGAGD
SFTAAFCASILNGKSVPEAHKLAVEVSAYVCTQSGAMPELPVILKDRLL
10000000000000000000000000000000000000000000000000000000000000000000000000000000011
11110000000000000000000000000000000000000000000000000000000000000000000000000000000
00000000000000000000000000000000000000000000000000000000000000000000000000000000000
0000000000000000000000000000000000000000000000
>Q8LNY5_1iyn_0_294
AASDSAQLKSAREDIKELLKTKFCHPIMVRLGWHDAGTYNKNIEEWPQRGGANGSLRFD
VELKHGANAGLVNALNLLKPIKDKYSGVTYADLFQLASATAIEEAGGPKIPMKYGRVDVTE
PEQCPEEGRLPDAGPPSPAQHLRDVFYRMGLNDKEIVALSGAHTLGRSRPDRSGWGKPET
KYTKDGPGAPGGQSWTAQWLKFDNSYFKDIKERRDEDLLVLPTDAALFEDPSFKVYAEKY
AADPEAFFKDYAEAHAKLSNLGAKFGPAEGFSLEGSPAGAAPEKFVAAKYSTGKD
00000000000000000000000000000000000000000000000000000000000000000000000000000000000
00000000000000000000000000000000000000000000000000000000000000000000000000000000000
00000000000000000000000000000000000000000000000000000000000000000000000000000000000
0000000000000000000000000001111111111111111111
>B1WBP0_3rl5_0_293
MAHGIPSQGKVTITVDEYSSNPTQAFTHYNINQSRFQPPHVHMVDPIPYDTPKPAGHTRFV
CISDTHSRTDGIQMPYGDILLHTGDFTELGLPSEVKKFNDWLGNLPYEYKIVIAGNHELTFD
KEFMADLVKQDYYRFPSVSKLKPEDFDNVQSLLTNSIYLQDSEVTVKGFRIYGAPWTPWFN
GWGFNLPRGQSLLDKWNLIPEGTDILMTHGPPLGFRDWVPKELQRVGCVELLNTVQRRVR
PKLHVFGGIHEGYGTMTDGYTTYINASTCTVSFQPTNPPIIFDLPNPQGS
11111111110000000000000000000000000000000000000000000000000000000000000000000000000
00000000000000000000000000000000000000000000000000000000000000000000000000000000000
00000000000000011000000000000000000000000000000000000000000000000000000000000000000
000000000000000000000000000000000000000000111
>O00189_3l81_159_452
QQSKVAPSSAASRPVLSSRSDQSQKNEVFLDVVERLSVLIASNGSLLKVDVQGEIRLKSFLPS
GSEMRIGLTEEFCVGKSELRGYGPGIRVDEVSFHSSVNLDEFESHRILRLQPPQGELTVMRYQ
LSDDLPSPLPFRLFPSVQWDRGSGRLQVYLKLRCDLLSKSQALNVRLHLPLPRGVVSLSQEL
SSPEQKAELAEGALRWDLPRVQGGSQLSGLFQMDVPGPPGPPSHGLSTSASPLGLGPASLSF
ELPRHTCSGLQVRFLRLAFRPCGNANPHKWVRHLSHSDAYVIRI
11111111111111111111111111000000000000000000000000000000000000000000000000000000000
00000000000000000000000000000000000000000000000000000000000000000000000000000000000
00000000000000000000000000000000000000000000000000000000000111111111111110000000000

000000000000000000001111111000000000000000000
>P0AAJ3_1kqf_0_293
MAMETQDIIKRSATNSITPPSQVRDYKAEVAKLIDVSTCIGCKACQVACSEWNDIRDEVGH
CVGVYDNPADLSAKSWTVMRFSETEQNGKLEWLIRKDGCMHCEDPGCLKACPSAGAIIQ
YANGIVDFQSENCIGCGYCIAGCPFNIPRLNKEDNRVYKCTLCVDRVSVGQEPACVKTCPT
GAIHFGTKKEMLELAEQRVAKLKARGYEHAGVYNPEGVGGTHVMYVLHHADQPELYHG
LPKDPKIDTSVSLWKGALKPLAAAGFIATFAGLIFHYIGIGPNKEVDDDEEDHHE
10000000000000000000000000000000000000000000000000000000000000000000000000000000000
00000000000000000000000000000000000000000000000000000000000000000000000000000000000
00000000000000000000000000000000000000000000000000000000000000000000000000000000000
000000000000000000000000000000000000000001111
>P77541_1oqf_1_294
SLHSPGKAFRAALTKENPLQIVGTINANHALLAQRAGYQAIYLSGGGVAAGSLGLPDLGIS
TLDDVLTDIRRITDVCSLPLLVDADIGFGSSAFNVARTVKSMIKAGAAGLHIEDQVGAKRCG
HRPNKAIVSKEEMVDRIRAAVDAKTDPDFVIMARTDALAVEGLDAAIERAQAYVEAGAE
MLFPEAITELAMYRQFADAVQVPILANITEFGATPLFTTDELRSAHVAMALYPLSAFRAMNR
AAEHVYNVLRQEGTQKSVIDTMQTRNELYESINYYQYEEKLDNLFARSQV
00000000000000000000000000000000000000000000000000000000000000000000000000000000000
00000000000000000000000000000000000000000000000000000000000000000000000000000000000
00000000000000000000000000000000000000000000000000000000000000000000000000000000000
000000000000000000000000000000000000000001111
>P96688_2r11_0_293
MSNHSSSIPELSDNGIRYYQTYNESLSLWPVRCKSFYISTRFGQTHVIASGPEDAPPLVLLHG
ALFSSTMWYPNIADWSSKYRTYAVDIIGDKNKSIPENVSGTRTDYANWLLDVFDNLGIEKS
HMIGLSLGGLHTMNFLLRMPERVKSAAILSPAETFLPFHHDFYKYALGLTASNGVETFLNW
MMNDQNVLHPIFVKQFKAGVMWQDGSRNPNPNADGFPYVFTDEELRSARVPILLLLLGEH
EVIYDPHSALHRASSFVPDIEAEVIKNAGHVLSMEQPTYVNERVMRFFNA
11111110000000000000000000000000000000000000000000000000000000000000000000000000000
00000000000000000000000000000000000000000000000000000000000000000000000000000000000
00000000000000000000000000000000000000000000000000000000000000000000000000000000000
000000000000000000000000000000000000000000000
>Q12380_2dyo_0_293
MNDIKQLLWNGELNVLVSIDPSFLMKGSPREIAVLRIRVPRETYLVNYMPLIWNKIKSFLSFD
PLTDSEKYFWFEHNKTPIPWNYPVGVLFDCLAGKSATFTTSFENQVKDVLTFLRIHLVMGDS
LPPTIIPIASSKTQAEKFWFHQWKQVCFILNGSSKAIMSLSVNEARKFWGSVITRNFQDFIEIS
NKISSSRPRHIPLIIQTSRTSGTFRISQPTISMTGVNPTLKDIEGDILDVKEGINGNDVMVICQG
IEIPWHMLLYDLYSKLRSFDGFLYITLVPIKGGDKASSEL
00000000000000000000000000000000000000000000000000000000000000001111000000000000000
00000000000000001111111110000000000000000000000000000000000000000000000000000000000
00000000000000000000000000000000000000000000000000000000000000000000000000001111000
000000000000000000000000000000000000111111111
>Q13546_4itj_0_293
MQPDMSLNVIKMKSSDFLESAELDSGGFGKVSLCFHRTQGLMIMKTVYKGPNCIEHNEAL
LEEAKMMNRLRHSRVVKLLGVIIEEGKYSLVMEYMEKGNLMHVLKAEMSTPLSVKGRIILE
IIEGMCYLHGKGVIHKDLKPENILVDNDFHIKIADLGLASFKMWSKLNNEEHNELREVDGT
AKKNGGTLYYMAPEHLNDVNAKPTEKSDVYSFAVVLWAIFANKEPYENAICEQQLIMCIKS
GNRPDVDDITEYCPREIISLMKLCWEANPEARPTFPGIEEKFRPFYLSQLE
11111100000000000000000000000000000000000000000000000000000000000000000000000000000
00000000000000000000000000000000000000000000000000000000000000000000000000000000000
00000000001111111111110000000000000000000000000000000000000000000000000000000000000
000000000000000000000000000000000000000000000

>Q4L6K9_3huu_1_294
SKLGYIPNQAARTLITNKTLTIGLIQKSSAPEIRQNPFNSDVLNGINQACNVRGYSTRMTVSE
NSGDLYHEVKTMIQSKSVDGFILLYSLKDDPIEHLLNEFKVPYLIVGKSLNYENIIHIDNDNID
AAYQLTQYLYHLGHRHILFLQESGHYAVTEDRSVGFKQYCDDVKISNDCVVIKSMNDLRDF
IKQYCIDASHMPSVIITSDVMLNMQLLNVLYEYQLRIPEDIQTATFNTSFLTENATPSQTSVNI
NPDVLGFTAGNTIIDVLRNETISFREKLISTQIVERVSTTKI
11111111111111111000000000000000000000000000000000000000000000000000000000000000000
00000000000000000000000000000000000000000000000000000000000000000000000000000000000
00000000000000000000000111111110000000000000000000000000000000000000000000000000000
00000000000000000000000111100000000000000000
>Q8ZM09_3r3s_0_293
MSHLYDPTTQYYTGEYPKQKQPAPGVQAKMTPVPDCGEKSYVGSGRLKDRKALVTGGDS
GIGRAAAIAYAREGADVAINYLPAEEEDAQQVKALIEECGRKAVLLPGDLSDESFARSLVHK
AREALGGLDILALVAGKQTAIPEIKDLTSEQFQQTFAVNVFALFWITQEAIPLLPKGASIITTSS
IQAYQPSPHLLDYAATKAAILNYSRGLAKQVAEKGIRVNIVAPGPIWTALQISGGQTQDKIP
QFGQQTPMKRAGQPAELAPVYVYLASQESSYVTAEVHGVCGGEHLG
11000000000000000000000000000000000000000000000000000000000000000000000000000000000
00000000000000000000000000000000000000000000000000000000000000000000000000000000000
00000000000000000000000000000000000000000000000000000000000000000000000000000000000
00000000000000000000000000000000000000000000
>Q92820_1l9x_24_317
RPHGDTAKKPIIGILMQKCRNKVMKNYGRYYIAASYVKYLESAGARVVPVRLDLTEKDYEI
LFKSINGILFPGGSVDLRRSDYAKVAKIFYNLSIQSFDDGDYFPVWGTCLGFEELSLLISGECL
LTATDTVDVAMPLNFTGGQLHSRMFQNFPTELLLSLAVEPLTANFHKWSLSVKNFTMNEK
LKKFFNVLTTNTDGKIEFISTMEGYKYPVYGVQWHPEKAPYEWKNLDGISHAPNAVKTAF
YLAEFFVNEARKNNHHFKSESEEEKALIYQFSPIYTGNISSFQQCYIFD
11111100000000000000000000000000000000000000000000000000000000000000000000000000000
00000000000000000000000000000000000000000000000000000000000000000000000000000000000
00000000000000000000000000000000000000000000000000000000000000000000000000000000000
00000000000000000000000000000000000000000000
>Q99952_2oc3_5_298
DSARSFLERLEARGGREGAVLAGEFSDIQACSAAWKADGVCSTVAGSRPENVRKNRYKDV
LPYDQTRVILSLLQEEGHSDYINGNFIRGVDGSLAYIATQGPLPHTLLDFWRLVWEFGVKVIL
MACREIENGRKRCERYWAQEQEPLQTGLFCITLIKEKWLNEDIMLRTLKVTFQKESRSVYQL
QYMSWPDRGVPSSPDHMLAMVEEARRLQGSGPEPLCVHCSAGCGRTGVLCTVDYVRQLL
LTQMIPPDFSLFDVVLKMRKQRPAAVQTEEQYRFLYHTVAQMFCSTLQNA
00010000001111111100000000000000000000000000000000000000000000000000000000000000000
00000000000000000000000000000000000000000000000000000000000000000000000000000000000
00000000000000000000000000000000000000000000000000000000000000000000000000000000000
00000000000000000000000000000000000000111111
>Q9I1R6_2fef_0_293
MHAFPLTLDNRLAEALPLWRNLARTDRAPRRNIDLADWKADWRELIAALDRFSRSHGYR
QPFAAQGHAALENAWAWGQAAENASTLLLKAIDRGLAGAELRSIYLETAALWLDYSRLLG
AARDSLREQGEVDFETAPALAPRTGQYPFALQLLAMGVLLDAQELIPALVEEVLQFDTDRL
LDYLGAAALGLTSASEETFHPRPFGQLRAFFEEADGSDAQALAPYLQSQYREFFQLSPKAQ
KKTRRLTGPYAWGWWAMEVSALGVLYGWDDGVLRASPHYLGDLVDYARARGDA
11111000000000000000000000000000000000000000000000000000000000000000000000000000000
00000000000000000000000000000000000000000000001111000000000000000000000000000000000
00000000000000000000000000000000000000000000000010000000000000000000000000000000000
00000000000000000000000000000000000000000001
>P55212_2wdp_0_292

MSSASGLRRGHPAGGEENMTETDAFYKREMFDPAEKYKMDHRRRGIALIFNHERFFWHLT
LPERRGTCADRDNLTRRFSDLGFEVKCFNDLKAEELLLKIHEVSTVSHADADCFVCVFLSH
GEGNHIYAYDAKIEIQTLTGLFKGDKCHSLVGKPKIFIIQACRGNQHDVPVIPLDVVDNQTE
KLDTNITEVDAASVYTLPAGADFLMCYSVAEGYYSHRETVNGSWYIQDLCEMLGKYGSSLE
FTELLTLVNRKVSQRRVDFCKDPSAIGKKQVPCFASMLTKKLHFFPKSN
11111111111111111111111111111100000000000000000000000000000000000000000000000000000
00000000000000000000000000000000000000000000000000000000000000000000000000000000001
11111111111111111111111111111111000000000000000000000000000000000000000000000000000
00000000000000000000000000000000000000000000
>P96084_1xrl_0_292
MDQECIENYAKVNGIYIYYKLCKAPEEKAKLMTMHGGPGMSHDYLLSLRDMTKEGITVLF
YDQFGCGRSEEPDQSKFTIDYGVEEAEALRSKLFGNEKVFLMGSSYGGALALAYAVKYQDH
LKGLIVSGGLSSVPLTVKEMNRLIDELPAKYRDAIKKYGSSGSYENPEYQEAVNYFYHQHLL
RSEDWPPEVLKSLEYAERRNVYRIMNGPNEFTITGTIKDWDITDKISAIKIPTLITVGEYDEVT
PNVARVIHEKIAGSELHVFRDCSHLTMWEDREGYNKLLSDFILKHL
11100000000000000000000000000000000000000000000000000000000000000000000000000000000
00000000000000000000000000000000000000000000000000000000000000000000000000000000000
00000000000000000000000000000000000000000000000000000000000000000000000000000000000
00000000000000000000000000000000000000000000
>Q5PXQ6_1tmx_0_292
MSTPVSAEQQAREQDLVERVLRSFDATADPRLKQVMQALTRHLHAFLREVRLTEAEWETGI
GFLTDAGHVTNERRQEFILLSDVLGASMQTIAMNNEAHGDATEATVFGPFFVEGSPRIESG
GDIAGGAAGEPCWVEGTVTDTDGNPVPDARIEVWEADDDGFYDVQYDDDRTAARAHLL
SGPDGGYAFWAITPTPYPIPHDGPVGRMLAATGRSPMRASHLHFMVTAPGRRTLVTHIFVE
GDELLDRDSVFGVKDSLVKSFERQPAGAPTPGGREIDGPWSRVRFDIVLAPA
10000000000000000000000000000000000000000000000000000000000000000000000000000000000
00000000000000000000000000000000000000000000000000000000000000000000000000000000000
00000000000000000000000000000000000000000000000000000000000000000000000000000000000
00000000000000000000000000000000000000000000
>Q5SKD9_2zbt_0_292
MEKGTFQIKTGFAEMFKGGVIMDVTTPEQAVIAEEAGAVAVMALERVPADIRAQGGVARM
SDPKIIKEIMAAVSIPVMAKVRIGHFVEAMILEAIGVDFIDESEVLTPADEEHHIDKWKFKVP
FVCGARNLGEALRRIAEGAAMIRTKGEAGTGNVVEAVRHARTMWKEIRYVQSLREDELM
AYAKEIGAPFELVKWVHDHGRLPVVNFAAGGIATPADAALMMHLGMDGVFVGSGIFKSG
DPRKRARAIVRAVAHYNDPEVLAEVSEDLGEPMVGINLDQLKEEERLAKRGW
00000000000000000000000000000000000000000000000000000000000000000000000000000000000
00000000000000000000000000000000000000000000000000000000000000000000000000000000000
00000000000000000000000000000000000000000000000000000000000000000000000000000000000
00000000000000000000000001111111111111111111
>Q6DNE2_3kg9_957_1249
TAKNLHPLLGEKLNLARIENQHHFQSYLTAESPAYLSQHQVFNKVLFPATGYLEIAAAVGK
NLLTTGEQVVVSDVTIVRGLVIPETDIKTVQTVISTLENNSYKLEIFSTSEGDNQQANQWTLH
AEGKIFLDSTTNTKAKIDLEQYQRECSQVIDIQQHYQQFKSRGIDYGNSFQGIKQLWKGQG
KALGKIALPEEIAGQATDYQLHPALLDAALQILGHAIGNTETDDKAYLPVGIDKLKQYRQTI
TQVWAIVEIPENTLKGSIKLVDNQGSLLAEIEGLRVTATTADALLK
11110000000000000000000000000000000000000000000000000000000000000000000000000000000
00000000000000000000000000000011110000000000000000000000000000000000000000000000000
00000000000000000000000000000000000000000000000000000000000000000000000000000000000
00000000000000000000000000000000000000000000
>Q8UEU8_2ojh_0_292
MRQSTLHTRLSTGPGGSMRSSIEIFNIRTRKMRVVWQTPELFEAPNWSPDGKYLLLNSEGLL

YRLSLAGDPSPEKVDTGFATICNNDHGISPDGALYAISDKVEFGKSAIYLLPSTGGTPRLMTK
NLPSYWHGWSPDGKSFTYCGIRDQVFDIYSMDIDSGVETRLTHGEGRNDGPDYSPDGRWIY
FNSSRTGQMQIWRVRVDGSSVERITDSAYGDWFPHPSPSGDKVVFVSYDADVFDHPRDLD
VRVQLMDMDGGNVETLFDLFGGQGTMNSPNWSPDGDEFAYVRYFPVE
11111111111111100000000000000000000000000000000000000000000000000000000000000000000
00000000000000000000000000000000000000000000000000000000000000000000000000000000000
00000000000000000000000000000000000000000000000000000000000000000000000000000000000
00000000000000000000000000000000000000000001
>Q9P0N9_3qwl_0_292
MTEDSQRNFRSVYYEKVGFRGVEEKKSLEILLKDDRLDTEKLCTFSQRFPLPSMYRALVWKV
LLGILPPHHESHAKVMMYRKEQYLDVLHALKVVRFVSDATPQAEVYLRMYQLESGKLPRS
PSFPLEPDDEVFLAIAKAMEEMVEDSVDCYWITRRFVNQLNTKYRDSLPQLPKAFEQYLNL
EDGRLLTHLRMCSAAPKLPYDLWFKRCFAGCLPESSLQRVWDKVVSGSCKILVFVAVEILLT
FKIKVMALNSAEKITKFLENIPQDSSDAIVSKAIDLWHKHCGTPVHSS
11111111111111111111000000000000000000000000000000000000000000000000000000000000000
00000000000000000000000000000000000000000000000000000000000000000000000000000000000
00000000000000000000000000000000000000000000000000000000000000000000000000000000000
00000000000000000000000000000000000000000001
>P50135_2aot_0_291
MASSMRSLFSDHGKYVESFRRFLNHSTEHQCMQEFMDKKLPGIIGRIGDTKSEIKILSIGGGA
GEIDLQILSKVQAQYPGVCINNEVVEPSAEQIAKYKELVAKTSNLENVKFAWHKETSSEYQS
RMLEKKELQKWDFIHMIQMLYYVKDIPATLKFFHSLLGTNAKMLIIVVSGSSGWDKLWKKY
GSRFPQDDLCQYITSDDLTQMLDNLGLKYECYDLLSTMDISDCFIDGNENGDLLWDFLTET
CNFNATAPPDLRAELGKDLQEPEFSAKKEGKVLFNNTLSFIVIEA
11110000000000000000000000000000000000000000000000000000000000000000000000000000000
00000000000000000000000000000000000000000000000000000000000000000000000000000000000
00000000000000000000000000000000000000000000000000000000000000000000000000000000000
0000000000000000000000000000000000000000000
>Q03132_3el6_2361_2652
HRPADVSALGVRGAEHPLLLAAVDVPGHGGAVFTGRLSTDEQPWLAEHVVGGRTLVPGS
VLVDLALAAGEDVGLPVLEELVLQRPLVLAGAGALLRMSVGAPDESGRRTIDVHAAEDVA
DLADAQWSQHATGTLAQGVAAGPRDTEQWPPEDAVRIPLDDHYDGLAEQGYEYGPSFQ
ALRAAWRKDDSVYAEVSIAADEEGYAFHPVLLDAVAQTLSLGALGEPGGGKLPFAWNTVT
LHASGATSVRVVATPAGADAMALRVTDPAGHLVATVDSLVVRSTGEKWEQPEPRG
11110000000000000000000000000000000000000000000000000000000000000000000000000000000
00000000000000000000000000000000000000000000000000000000000000000000000000000000000
00000000000000000000000000000000000000000000000000000000011110000000000000000000000
0000000000000000000000000000001111111111111
>Q9F1Y5_3vc1_0_291
MTTETTTATATAKIPAPATPYQEDIARYWNNEARPVNLRLGDVDGLYHHHYGIGPVDRAA
LGDPEHSEYEKKVIAELHRLESAQAEFLMDHLGQAGPDDTLVDAGCGRGGSMVMAHRRF
GSRVEGVTLSAAQADFGNRRARELRIDDHVRSRVCNMLDTPFDKGAVTASWNNESTMYV
DLHDLFSEHSRFLKVGGRYVTITGCWNPRYGQPSKWVSQINAHFECNIHSRREYLRAMAD
NRLVPHTIVDLTPDTLPYWELRATSSLVTGIEKAFIESYRDGSFQYVLIAADRV
11111111111111000000000000000000000000000000000000000000000000000000000000000000000
00000000000000000000000000000000000000000000000000000000000000000000000000000000000
00000000000000000000000000000000000000000000000000000000000000000000000000000000000
0000000000000000000000000000000000000000000
>Q9XCU0_1duw_31_322
AALEPTDSGAPSAIVMFPVSAKPNPKGAAMKPAVFNHLAHEKKIANCETCHHTGDPVACS
TCHTTEGKAEGNFVTLDRAMHATNIAKRAKGNTPVSCVSCHEQQTKERRECAGCHAIVT

PKRDQAWCATCHNVTSSMTPEQMQQGIKGKLPPDQNEALAAETVLNHKPVQPLTAMQG
PYKVSIDALADKYEPSNFTHRRHMASLMERIKGDKLAEAFHNKPETLCATCHHRSPLSATP
PKCGSCHTKEIDPANPNRPNLKAAYHLQCMGCHQGMNVGRPKNTDCTTCHKARP
11100000000000000000000000000000000000000000000000000000000000000000000000000000000
00000000000000000000000000000000000000000000000000000000000000000000000000000000000
00000000000000000000000000000000000000000000000000000000000000000000000000000000000
0000000000000000000000000000000000000000000
>A6LIT8_3mz2_25_315
EELEYKGMNTLQISNVDDLISFYQYADDRIPLISGHRGGRGKGYPENSMETFENTLSYTPATF
EIDPRLTKDSVIVLFHDDTLERTSNGTGKVSDYTWEELQNFRLKDPEGNITNYRIPTLEEAIR
WARGKTILILDKKDVPMERTAQLITDMQAEPYVMITVHDGASARFFYEKNPNFMFEAFVK
TKEAVQDYEDNGIPWSHIMAYVGPKITPEVREVIDMLHERGVMCMISTAPSDDKLSTPESR
AEAYRMIIRQGVDIIESDRPIEVAEAISSLIPVSSSKGKFFSTL
11111100000000000000000000000000000000000000000000000000000000000000000000000000000
00000000000000000000000000000000000000000000000000000000000000000000000000000000000
00000000000000000000000000000000000000000000000000000000000000000000000000000000000
00000000000000000000000000000000000000000
>O33245_2jay_0_290
MTWPLPDRLSINSLSGTPAVDLSSFTDFLRRQAPELLPASISGGAPLAGGDAQLPHGTTIVAL
KYPGGVVMAGDRRSTQGNMISGRDVRKVYITDDYTATGIAGTAAVAVEFARLYAVELEHYE
KLEGVPLTFAGKINRLAIMVRGNLAAAMQGLLALPLLAGYDIHASDPQSAGRIVSFDAAG
GWNIEEEGYQAVGSGSLFAKSSMKKLYSQVTDGDSGLRVAVEALYDAADDDSATGGPDLV
RGIFPTAVIIDADGAVDVPESRIAELARAIIESRSGADTFGSDGGEK
11111111111111111111111111111111111111111111111111111111000000000000000000001111111
10000000000000000000000000000000000000000000000000000000000000000000000000000000000
00000000000000000000000000000000000000000000000000000000000000000011111111111111100
00000000000000000000000001111111111111111
>P0ACP1_2iks_43_333
EHNYHPNAVAAGLRAGRTRSIGLVIPDLENTSYTRIANYLERQARQRGYQLLIACSEDQPD
NEMRCIEHLLQRQVDAIIVSTSLPPEHPFYQRWANDPFPIVALDRALDREHFTSVVGADQD
DAEMLAEELRKFPAETVLYLGALPELSVSFLREQGFRTAWKDDPREVHFLYANSYEREAAA
QLFEKWLETHPMPQALFTTSFALLQGVMDVTLRRDGKLPSDLAIATFGDNELLDFLQCPVL
AVAQRHRDVAERVLEIVLASLDEPRKPKPGLTRIKRNLYRRGVLSRS
11111111111111000000000000000000000000000000000000000000000000000000000000000000000
00000000000000000000000000000000000000000000000000000000000000000000000000000000000
00000000000000000000000000000000000000000000000000000000000000000000000000000000000
00000000000000000000000000000000000000000
>P11172_3g3d_189_479
DAETVGRVKRFIQENVFVAANHNGSPLSIKEAPKELSFGARAELPRIHPVASKLLRLMQKKE
TNLCLSADVSLARELLQLADALGPSICMLKTHVDILNDFTLDVMKELITLAKCHEFLIFEDR
KFADIGNTVKKQYEGGIFKIASWADLVNAHVVPGSGVVKGLQEVGLPLHRGCLLIAEMSST
GSLATGDYTRAAVRMAEEHSEFVVGFISGSRVSMKPEFLHLTPGVQLEAGGDNLGQQYNSP
QEVIGKRGSDIIIVGRGIISAADRLEAAEMYRKAAWEAYLSRLGV
11111111111111111111111111111110000000000000000000000000000000000000000000000000000
00000000000000000000000000000000000000000000000000000000000000000000000000000000000
00000000000000000000000000000000000000000000000000000000000000000000000000000000000
00000000000000000000000000000000000000001
>P16442_2rix_63_353
VSLPRMVYPQPKVLTPCRKDVLVVTPWLAPIVWEGTFNIDILNEQFRLQNTTIGLTVFAIKK
YVAFLKLFLETAEKHFMVGHRVHYYVFTDQPAAVPRVTLGTGRQLSVLEVRAYKRWQDVS
MRRMEMISDFCERRFLSEVDYLVCVDVDMEFRDHVGVEILTPLFGTLHPGFYGSSREAFTYE

RRPQSQAYIPKDEGDFYYLGGFFGGSVQEVQRLTRACHQAMMVDQANGIEAVWHDESHL
NKYLLRHKPTKVLSPEYLWDQQLLGWPAVLRKLRFTAVPKNHQAVRNP
00000000000000000000000000000000000000000000000000000000000000000000000000000000000
00000000000000000000000000000001111111100000000000000000000000000000000000000000000
00000000000000000000000000000000000000000000000000000000000000000000000000000000000
000000000000000000000000000000000111111111
>P37565_1vzy_0_290
MDYLVKALAYDGKVRAYAARTTDMVNEGQRRHGTWPTASAALGRTMTASLMLGAMLK
GDDKLTVKIEGGGPIGAIVADANAKGEVRAYVSNPQVHFDLNEQGKLDVRRAVGTNGTLS
VVKDLGLREFFTGQVEIVSGELGDDFTYYLVSSEQVPSSVGVGVLVNPDNTILAAGGFIIQLM
PGTDDETITKIEQRLSQVEPISKLIQKGLTPEEILEEVLGEKPEILETMPVRFHCPCSKERFETAI
LGLGKKEIQDMIEEDGQAEAVCHFCNEKYLFTKEELEGLRDQTTR
00000000000000000000000000000000000000000000000000000000000000000000000000000000000
00000000000000000000000000000000000000000000000000000000000000000000000000000000000
00000000000000000000000000000000000000000000000000000000000000000000000000000000000
000000000000000000000000000000000000000001
>Q5SRE7_2opw_0_290
MACLSPSQLQKFQQDGFLVLEGFLSAEECVAMQQRIGEIVAEMDVPLHCRTEFSTQEEEQL
RAQGSTDYFLSSGDKIRFFFEKGVFDEKGNFLVPPEKSINKIGHALHAHDPVFKSITHSFKVQ
TLARSLGLQMPVVVQSMYIFKQPHFGGEVSPHQDASFLYTEPLGRVLGVWIAVEDATLENG
CLWFIPGSHTSGVSRRMVRAPVGSAPGTSFLGSEPARDNSLFVPTPVQRGALVLIHGEVVHK
SKQNLSDRSRQAYTFHLMEASGTTWSPENWLQPTAELPFPQLYT
10000000000000000000000000000000000000000000000000000000000000000000000000000000000
00000000000000000000000000000000000000000000000000000000000000000000000000000000000
00000000000000000000000000000000000000001111000000000000000000000000000000000000000
000000000000000000000000000000000000000000
>Q73KE9_2y3c_38_328
KSGGGESSEVTPNTPVDKTYTVGSVEFTMKGIAAVNAQLGHNDYSINQPHTVSLSAYLIGET
EVTQELWQAVMGNNPSHFNGSPAVGETQGKRPVENVNWYQAIAFCNKLSIKLNLEPCYT
VNVGGNPVDFAALSFDQIPDSNNADWDKAELDINKKGFRLPTEAEWEWAAKGGTDDKW
SGTNTEAELKNYAWYGSNSGSKTHEVKKKKPNWYGLYDIAGNVAEWCWDWRADIHTGD
SFPQDYPGPASGSGRVLRGGSWAGSADYCAVGERVNISPGVRCSDLGFRLACRP
11111111111110000000000000000000000000000000000000000000000000000000000000000000000
00000000000000000000000000000000000000000000000000000000000000000000000000000000000
00000000000000000000000000000000000000000000000000000000000000000000000000000000000
000000000000000000000000000000000000000000
>Q92N37_2rin_27_317
AEPESCGTVRFSDVGWTDITATTATATTILEALGYETDVKVLSVPVTYTSLKNKDIDVFLGN
WMPTMEADIAPYREDKSVETVRENLAGAKYTLATNAKGAELGIKDFKDIAAHKDELDGKI
YGIEPGNDGNRLIIDMVEKGTFDLKGFEVVESSEQGMLAQVARAEKSGDPIVFLGWEPHPM
NANFKLTYLSGGDDVFGPNYGGATVHTNVRAGYTTECPNVGKLLQNLSFSLQMENEIMG
KILNDGEDPEKAAAAWLKDNPQSIEPWLSGVATKDGGDGLAAVKAALGL
11100000000000000000000000000000000000000000000000000000000000000000000000000000000
00000000000000000000000000000000000000000000000000000000000000000000000000000000000
00000000000000000000000000000000000000000000000000000000000000000000000000000000000
000000000000000000000000000000000000000000
>P07359_1m0z_16_305
HPICEVSKVASHLEVNCDKRNLTALPPDLPKDTTILHLSENLLYTFSLATLMPYTRLTQLNLD
RCELTKLQVDGTLPVLGTLDLSHNQLQSLPLLGQTLPALTVLDVSFNRLTSLPLGALRGLGE
LQELYLKGNELKTLPPGLLTPTPKLEKLSLANNNLTELPAGLLNGLENLDTLLLQENSLYTIP
KGFFGSHLLPFAFLHGNPWLCNCEILYFRRWLQDNAENVYVWKQGVDVKAMTSNVASV

QCDNSDKFPVYKYPGKGCPTLGDEGDTDLYDYYPEEDTEGDKVR
00000000000000000000000000000000000000000000000000000000000000000000000000000000000
00000000000000000000000000000000000000000000000000000000000000000000000000000000000
00000000000000000000000000000000000000000000000000000000000000000000000000000000000
00000000000000000111111111111111111111111
>P36913_1eok_39_328
ATALAGSNGVCIAYYITDGRNPTFKLKDIPDKVDMVILFGLKYWSLQDTTKLPGGTGMMGS
FKSYKDLDTQIRSLQSRGIKVLQNIDDDVSWQSSKPGGFASAAAYGDAIKSIVIDKWKLDGIS
LDIEHSGAKPNPIPTFPGYAATGYNGWYSGSMAATPAFLNVISELTKYFGTTAPNNKQLQIA
SGIDVYAWNKIMENFRNNFNYIQLQSYGANVSRTQLMMNYATGTNKIPASKMVFGAYAEG
GTNQANDVEVAKWTPTQGAKGGMMIYTYNSNVSYANAVRDAVKN
11111110000000000000000000000000000000000000000000000000000000000000000000000000000
00000000000000000000000000000000000000000000000000000000000000000000000000000000000
00000000000000000000000000000000000000000000000000000000000000000000000000000000000
00000000000000000000000000000000000000001
>Q5LE92_3sy6_18_307
EQEDKVSGEKTLAVVSASSDDRPSTRGIINDNTYALGVFRTTANTYAPLYNVKHIYSGGEWG
ADDVIKVDYRNASFFAYYPYHTATGNYAGLAGGTTLTLQAQLFNAGEDICYGAGEASGGG
PVSVYNPFVEFLNMKHAYARLRLTLTRGEKFDKTKKCNIQNITFKSNNANFYLTRSLDIAST
AGATGGSAVAAGYVHNPNVNIATGKSVTYEYMFPPQPLDGSKLTILVTVDGVTRSCDISTLG
SSLDSGKYYGVSLTFTDVGIILSSAVVTVNNFGGQGNTQVDTEL
11111100000000000000000000000000000000000000000000000000000000000000000000000000000
00000000000000000000000000000000000000000000000000000000000000000000000000000000000
00000000000000000000000000000000000000000000000000000000000000000000000000000000000
00000000000000000000000000000111111111111
>O55012_3zyk_0_288
MSGQSLTDRITAAQHSVTGSAVSKTVCKATTHEIMGPKKKHLDYLIQCTNEMNVNIPQLA
DSLFERTTNSSWVVVFKSLITTHHLMVYGNERFIQYLASRNTLFNLSNFLDKSGLQGYDMST
FIRRYSRYLNEKAVSYRQVAFDFTKVKRGADGVMRTMNTEKLLKTVPIIQNQMDALLDFN
VNSNELTNGVINAAFMLLFKDAIRLFAAYNEGIINLLEKYFDMKKNQCKEGLDIYKKFLTR
MTRISEFLKVAEQVGIDRGDIPDLSQAPSSLLDALEQHLASLEGKK
11111111111111111000000000000000000000000000000000000000000000000000000000000000000
00000000000000000000000000000000000000000000000000000000000000000000000000000000000
00000000000000000000000000000000000000000000000000000000000000000000000000000000000
0000000000000000000000000000000000000001
>P13664_1kzq_47_335
SDPPLVANQVVTCPDKKSTAAVILTPTENHFTLKCPKTALTEPPTLAYSPNRQICPAGTTSSCT
SKAVTLSSLIPEAEDSWWTGDSASLDTAGIKLTVPIEKFPVTTQTFVVGCIKGDDAQSCMVT
VTVQARASSVVNNVARCSYGADSTLGPVKLSAEGPTTMTLVCGKDGVKVPQDNNQYCSG
TTLTGCNEKSFKDILPKLTENPWQGNASSDKGATLTIKKEAFPAESKSVIIGCTGGSPEKHHC
TVKLEFAGAAGSAKSAAGTASHVSIFAMVIGLIGSIAACVA
11100000000000000000000000000000000000000000000000000000000000000000000000000000000
00000000000000000000000000000000000000000000000000000000000000000000000000000000000
00000000000000000000000000000000000000000000000000000000000000000000000000000000000
0000000111111111111111111111111111111111
>P19447_4ern_493_781
MELQNNGYIAKVQCAEVWCPMSPEFYREYVAIKTKKRILLYTMNPNKFRACQFLIKFHERR
NDKIIVFADNVFALKEYAIRLNKPYIYGPTSQGERMQILQNFKHNPKINTIFISKVGDTSFDLP
EANVLIQISSHGGSRRQEAQRLGRVLRAKKGMVAEEYNAFFYSLVSQDTQEMAYSTKRQRF
LVDQGYSFKVITKLAGMEEEDLAFSTKEEQQLLQKVLAATDLDAEEEVVAGEFGSRSSQA
SRRFGTMSSMSGADDTVYMEYHSSRSKAPSKHVHPLFKRFRK

1111111100000000000000000000000000000000000000000000000000000000000000000000000000
00000000000000000000000000000000000000000000000000000000000000000000000000000000000
00000000000000000000000000000000000000000000000000000000000000000000000111111111111
111111111111111111111111111111111111111
>P46926_1ne7_0_288
MKLIILEHYSQASEWAAKYIRNRIIQFNPGPEKYFTLGLPTGSTPLGCYKKLIEYYKNGDLSF
KYVKTFNMDEYVGLPRDHPESYHSFMWNNFFKHIDIHPENTHILDGNAVDLQAECDAFEE
KIKAAGGIELFVGGIGPDGHIAFNEPGSSLVSRTRVKTLAMDTILANARFFDGELTKVPTMA
LTVGVGTVMDAREVMILITGAHKAFALYKAIEEGVNHMWTVSAFQQHPRTVFVCDEDAT
LELKVKTVKYFKGLMLVHNKLVDPLYSIKEKETEKSQSSKKPYSD
00000000000000000000000000000000000000000000000000000000000000000000000000000000000
00000000000000000000000000000000000000000000000000000000000000000000000000000000000
00000000000000000000000000000000000000000000000000000000000000000000000000000000000
0000000000000000000000000111111111111111
>Q1W548_4g75_0_288
MVMDLGTTTIVAGQGDFSAYNYGNNGANWPAPSPAQVGYISKGQRDVAYVNGDWEKPL
LALWAQWANWGTTLYGYPSLPFPNYEFIWDIFDLPQADYNQFSLGDDRITAMNRAQNHQ
YPFPNNEGIPSWDRPKIDTFNGGVYTPAAAFAHYLTGKGKKMNFPIERLNIKPNVKAMPQF
IGVLTSSPMGQTTVDFNVPYATAKDSWVAGNTVGEITLRIVGILVKSTSGQWSFRGEIRAYDD
LYDFNPSNHRTETAEGMTRLGREVGQKFKDTTPYPIGIPGAIPVNISG
10000000000000000000000000000000000000000000000000000000000000000000000000000000000
00000000000000000000000000000000000000000000000000000000000000000000000000000000000
00000000000000000000000000000000000000000000000000000000000000000000000000000000000
0000000000000000000000000000000000000000
>Q2RXX6_3dup_1_289
SFLKHVQDCNTHDLSNFVRFVIEGRRVGWVRKALAQRLKAHGRVFDVTRDAVLLSASLRT
PQSRTRAVADVVDRLADEGVVPAPRGELYRVNQSWGEPTLMLLDRAVVPTFGVRAYGVHL
NGYVGAGADLHLWIGRRSPDKSVAPGKLDNMVAGGQPADLSLRQNLIKECAEEADLPEA
LARQAIPVGAITYCMESPAGIKPDTLFLYDLALPEDFRPHNTDGEMADFMLWPAAKVVEA
VRTTEAFKFNVNLTVIDFAIRHGLIDPDNEPDYQEILAGLRGRPRERAAS
00000000000000000000000000000000000000000000000000000000000000000000000000000000000
00000000000000000000000000000000000000000000000000000000000000000000000000000000000
00000000000000000000000000000000000000000000000000000000000000000000000000000000000
0000000000000000000000000000000001111111
>Q741V7_3rd5_0_288
MTGWTAADLPSFAQRTVVITGANSGLGAVTARELARRGATVIMAVRDTRKGEAAARTMA
GQVEVRELDLQDLSSVRRFADGVSGADVLINNAGIMAVPYALTVDGFESQIGTNHLGHFAL
TNLLLPRLTDRVVTVSSMAHWPGRINLEDLNWRSRRYSPWLAYSQSKLANLLFTSELQRRL
TAAGSPLRALAAHPGYSHTNLQGASGRKLGDALMSAATRVVATDADFGARQTLYAASQD
LPGDSFVGPRFGYLGRTQPVGRSRRAKDAGMAAALWALSEQLTKTEFPL
10000000000000000000000000000000000000000000000000000000000000000000000000000000000
00000000000000000000000000000000000000000000000000000000000000000000000000000000000
00000000000000000000000000000000000011111111111111111111000000000000000000000000000
0000000000000000000000000000000000000000
>A0AW19_3gdc_87_374
GGSVLAERAGIDPTAILRDFDRGRTSTLPDGRTLREWDIVAVDKDFEIAPGIIFKGWSYNGRI
PGPTLWAREGDALRIHFTNAGAHPHTIHFHGVHRATMDGTPGIGAGSIAPGQSFTYEFDAT
PFGTHLYHCHQSPLAPHIAKGLYGGFIVEPKEGRPPADDEMVMVMNGYNTDGGDDNEFY
SVNGLPFHFMDFPVKVKQHELVRIHLINVLEYDPINSFHIHGNFFHYYPTGTMLTPSEYTDTI
SQVQGQRGILELRFPYPGKFMFHAHKTEFAELGWMGFFEVSA
11000000000000000000000000000000000000000000000000000000000000000000000000000000000

00000000000000000000000000000000000000000000000000000000000000000000000000000000000
00000000000000000000000000000000000000000000000000000000000000000000000000000000000
00000000000000000000000000000000000000
>C8WX00_3qsg_0_287
MKLGFIGFGEAASAIASGLRQAGAIDMAAYDAASAESWRPRAEELGVSCKASVAEVAGEC
DVIFSLVTAQAALEVAQQAGPHLCEGALYADFTSCSPAVKRAIGDVISRHRPSAQYAAVAVM
SAVKPHGHRVPLVVDGDGARRFQAAFTLYGCRIEVLDGEVGGAALLKMCRSAVLKGLEAL
FLEALAAAEKMGLADRVLASLDASFPEHHLRDLALYLVERNLEHADRRAHELGEVAATLC
SVGVEPLVAEAGYRRLTRVAQVRAALKQRPGDVRAWLRSLANAEDA
00000000000000000000000000000000000000000000000000000000000000000000000000000000000
00000000000000000000000000000000000000000000000000000000000000000000000000000000000
00000000000000000000000000000000000000000000000000000000000000000000000000000000000
00000000000000000000000000000000000111
>Q46845_3c8e_0_287
MTDNTYQPAKVWTWDKSAGGAFANINRPVSGPTHEKTLPVGKHPLQLYSLGTPNGQKVT
IMLEELLALGVTGAEYDAWLIRIGDGDQFSSGFVEVNPNSKIPALRDHTHNPPIRVFESGSIL
LYLAEKFGYFLPQDLAKRTETMNWLFWLQGAAPFLGGGFGHFYHYAPVKIEYAINRFTME
AKRLLDVLDKQLAQHKFVAGDEYTIADMAIWPWFGNVVLGGVYDAAEFLDAGSYKHVQ
RWAKEVGERPAVKRGRIVNRTNGPLNEQLHERHDASDFETNTEDKRQG
11110000000000000000000000000000000000000000000000000000000000000000000000000000000
00000000000000000000000000000000000000000000000000000000000000000000000000000000000
00000000000000000000000000000000000000000000000000000000000000000000000000000000000
00000000000000000000000000000000000000
>Q5M3Z4_3hp7_0_287
MPKERVDVLAYKQGLFETREQAKRGVMAGLVVNVINGERYDKPGEKIDDGTELKLKGEKL
RYVSRGGLKLEKALAVFNLSVEDMITIDIGASTGGFTDVMLQNGAKLVYAVDVGTNQLVW
KLRQDDRVRSMEQYNFRYAEPVDFTEGLPSFASIDVSFISLNLILPALAKILVDGGQVVALVK
PQFEAGREQIGKNGIVRESSIHEKVLETVTAFAVDYGFSVKGLDFSPIQGGHGNIEFLAHLEK
TDSPQNDVPTSIKEVVAQAHKEFKKNEEERSFGVNPKDCPRK
00000000000000000000000000000000000000000000000000000000000000000000000000000000000
00000000000000000000000000000000000000000000000000000000000000000000000000000000000
00000000000000000000000000010000000000000000000000000000000000000000000000000000000
00000000000000000000000001111111111111
>Q8Y7B7_3uf6_0_287
MTKSRFFSDVAETSSFVFAVAGADDEVVLETIRLALKQKLGKFLLFGKKEDKTLTANESVTW
IQTDTAEAAAQGAILAVKNKEADILVKGFIPTATLMHHVLKKENGLRTDQLLSQIAIFDIPT
YHKPLLITDCAMNVAPKTKEKIAITENALAVAHQIGITNPKIALLSAVEEVTAKMPSTLEAQ
EVVQHFGNQISVSGPLALDVAISKEAALHKGITDSSAGEADILIAPNIETGNALYKSLVYFAG
AKVGSAVVGAKVPIVISSRNDSPENKLASFILTVRLVEK
10000000000000000000000000000000000000000000000000000000000000000000000000000000000
00000000000000000000000000000000000000000000000000000000000000000000000000000000000
00000000000000000000000000000000000000000000000000000000000000000000000000000000000
00000000000000000000000000000000000001
>Q99640_3p1a_74_361
HQLQPRRVSFRGEASETLQSPGYDPSRPESFFQQSFQRLSRLGHGSYGEVFKVRSKEDGRLYA
VKRSMSPFRGPKDRARKLAEVGSHEKVGQHPCCVRLEQAWEEGGILYLQTELCGPSLQQH
CEAWGASLPEAQVWGYLRDTLLALAHLHSQGLVHLDVKPANIFLGPRGRCKLGDFGLLVE
LGTAGAGEVQEGDPRYMAPELLQGSYGTAADVFSLGLTILEVACNMELPHGGEGWQQLR
QGYLPPEFTAGLSSELRSVLVMMLEPDPKLRATAEALLALPVLRQP
10000000000000000000000000000000000000000000000000000000000000000000000000000000000
00000000000000000000000000000000000000000000000000000000000000000000000000000000000

00000000000000000011111100000000000000000000000000000000000000000000000000000000000
000000000000000000000000000000000000000
>C7MT25_4ich_0_286
MSESDELRRRVRGLIHSRPGAQREFAAAIGLDESKLSKSLNGTRRFSPHELVRIAEHSGVTVN
WLINGRDDARTVAAVPAPTARSRSAPAGEPQSEARRRILETAWRLIARRGYHNVRIHDIASE
LGTSNATIHYHFPSKKDILLEALRRNVKLAFDRQVAELHTIADARERLVRLVELQLPTPGLLR
DEWSVWLQVWTESTLNPKIRDLYNDAYDRWYQTIAMTIRTGQKQGVFRDQDADELATRL
SALIDGLGIQVLTGKRGCSVDHMRQHLNDFIEHNIVERRP
11111111111111111111111111111111111111111111111111111111111111111111111111111111111
11111111100000000000000000000000000000000000000000000000000000000000000000000000000
00000000000000000000000000000000000000000000000000000000000000000000000000000000000
00000000000000000000000000000000000001
>C7RH50_3r89_0_286
MKIIDKLYEKVSKNGFVCIGLDSSIDYIPENMKAGKSVSEALFSYNKEIIDQTYDVCAIYKLQI
AYYESYGIEGMIAYRDTLSYLREKDLLSIGDVKRSDIAASAKMYAKAHFEGDFETDFITLNPY
MGMDSIEPYEEYIEKGDKGVFVLLRTSNPGAKDFEVLPVDGEEFFYKVGDKMRELNEKYIG
KSGFGPIGLVVGATHSEEVEKIRKRYDKMFFLIPGFGAQKADSMNVYKLLEGLNGGVVNSS
RAILKNWQNYEDGSEKVGYYARKKAIETYEEIKANEVL
00000000000000000000000000000000000000000000000000000000000000000000000000000000000
00000000000000000000000000000000000000000000000000000000000000000000000000000000000
00000000000000000000000000000000000000000000000000000000000000000000000000000000000
00000000000000000000000000000000000001
>P00817_8prk_0_286
MTYTTRQIGAKNTLEYKVYIEKDGKPVSAFHDIPLYADKENNIFNMVVEIPRWTNAKLEITK
EETLNPIIQDTKKGKLRFVRNCFPHHGYIHNYGAFPQTWEDPNVSHPETKAVGDNDPIDVL
EIGETIAYTGQVKQVKALGIMALLDEGETDWKVIAIDINDPLAPKLNDIEDVEKYFPGLLRA
TNEWFRIYKIPDGKPENQFAFSGEAKNKKYALDIIKETHDSWKQLIAGKSSDSKGIDLTNVT
LPDTPTYSKAASDAIPPASPKADAPIDKSIDKWFFISGSV
10000000000000000000000000000000000000000000000000000000000000000000000000000000000
00000000000000000000000000000000000000000000000000000000000000000000000000000000000
00000000000000000000000000000000000000000000000000000000000000000000000000000000000
00000000000000000000000000000000001111
>Q54944_1gvn_0_286
MANIVNFTDKQFENRLNDNLEELIQGKKAVESPTAFLLGGQPGSGKTSLRSAIFEETQGNVI
VIDNDTFKQQHPNFDELVKLYEKDVVKHVTPYSNRMTEAIISRLSDQGYNLVIEGTGRTTDV
PIQTATMLQAKGYETKMYVMAVPKINSYLGTIERYETMYADDPMTARATPKQAHDIVVKN
LPTNLETLHKTGLFSDIRLYNREGVKLYSSLETPSISPKETLEKELNRKVSGKEIQPTLERIEQK
MVLNKHQETPEFKAIQQKLESLQPPTPPIPKTPKLPGI
10000000000000000000000000000000000000000000000000000000000000000000000000000000000
00000000000000000000000000000000000000000000000000000000000000000000000000000000000
00000000000000000000000000000000000000000000000000000000000000000000000000000000000
00000000000000000000000001111111111111
>Q9HUU1_3b8i_0_286
MHRASHHELRAMFRALLDSSRCYHTASVFDPMSARIAADLGFECGILGGSVASLQVLAAP
DFALITLSEFVEQATRIGRVARLPVIADADHGYGNALNVMRTVVELERAGIAALTIEDTLLP
AQFGRKSTDLICVEEGVGKIRAALEARVDPALTIIARTNAELIDVDAVIQRTLAYQEAGADGI
CLVGVRDFAHLEAIAEHLHIPLMLVTYGNPQLRDDARLARLGVRVVVNGHAAYFAAIKAT
YDCLREERGAVASDLTASELSKKYTFPEEYQAWARDYMEVKE
11000000000000000000000000000000000000000000000000000000000000000000000000000000000
00000000000000000000000000000000000000000000000000000000000000000000000000000000000
00000000000000000000000000000000000000000000000000000000000000000000000000000000000

00000000000000000000000000000000000001
>A6LIL3_3kws_26_311
SCSSKPANTAKGEKGSDKTGKDLELKLSFQEGIAPGESLNEKLDFMEKLGVVGFEPGGGL
AGRVNEIKQALNGRNIKVSAICAGFKGFILSTDPAIRKECMDTMKEIIAAAGELGSTGVIIVP
AFNGQVPALPHTMETRDFLCEQFNEMGTFAAQHGTSVIFEPLNRKECFYLRQVADAASLC
RDINNPGVRCMGDFWHMTWEETSDMGAFISGGEYLQHVHVASRKRRSMPGEDGDADNY
INGFKGLKMIGYNNYVSFECGCQGDRNVVVPAAVKLLREQWEQA
11111111111111111111100000000000000000000000000000000000000000000000000000000000000
00000000000000000000000000000000000000000000000000000000000000000000000000000000000
00000000000000000000000000000000000000000000000000000000000000000000000000000000000
0000000000000000000000000000000000000
>O00167_4egc_252_537
SKRSSDPSPAGDNEIERVFVWDLDETIIIFHSLLTGTFASRYGKDTTTSVRIGLMMEEMIFNLA
DTHLFFNDLEDCDQIHVDDVSSDDNGQDLSTYNFSADGFHSSAPGANLCLGSGVHGGVD
WMRKLAFRYRRVKEMYNTYKNNVGGLIGTPKRETWLQLRAELEALTDLWLTHSLKALNLI
NSRPNCVNVLVTTTQLIPALAKVLLYGLGSVFPIENIYSATKTGKESCFERIMQRFGRKAVYV
VIGDGVEEEQGAKKHNMPFWRISCHADLEALRHALELEYL
11111111111111100000000000000000000000000000000000000000000000000000000000000000000
00000000000000000000111111111111111000000000000000000000000000000000000000000000000
00000000000000000000000000000000000000000000000000000000000000000000000000000000000
0000000000000000000000000000000000000
>P51584_1wb4_791_1076
DKFPVAENPSSSFKYESAVQYRPAPDSYLNPCPQAGRIVKETYTGINGTKSLNVYLPYGYDP
NKKYNIFYLMHGGGENENTIFSNDVKLQNILDHAIMNGELEPLIVVTPTFNGGNCTAQNF
YQEFRQNVIPFVESKYSTYAESTTPQGIAASRMHRGFGGFSMGGLTTWYVMVNCLDYVAYF
MPLSGDYWYGNSPQDKANSIAEAINRSGLSKREYFVFAATGSDHIAYANMNPQIEAMKAL
PHFDYTSDFSKGNFYFLVAPGATHWWGYVRHYIYDALPYFFHE
11111111111000000000000000000000000000000000000000000000000000000000000000000000000
00000000000000000000000000000000000000000000000000000000000000000000000000000000000
00000000000000000000000000000000000000000000000000000000000000000000000000000000000
0000000000000000000000000000000000000
>Q5SJM3_1x1o_0_285
MGGVVGEALWQGGLEEALRAWLREDLGQGDLTSLLVVPEDLEGEAVILAKEGGVLAGLW
VAERVFALADPRTAFTPLVAEGARVAEGTEVARVRGPLRGILAGERLALNLLQRLSGIATLTR
AYVEALAGTKAQILDTRKTTPGLRALEKYAVRVGGGRNHRYGLFDGILLKENHVRAAGGV
GEAVRRAKARAPHYLKVEVEVRSLEELEEALEAGADLILLDNFPLEALREAVRRVGGRVPL
EASGNMTLERAKAAAEAGVDYVSVGALTHSAKALDLSLLVVRP
11111111110000000000000000000000000000000000000000000000000000000000000000000000000
00000000000000000000000000000000000000000000000000000000000000000000000000000000000
00000000000000000000000000000000000000000000000000000000000000000000000000000000000
0000000000000000000000000000000000000
>Q6FJS2_2p4f_23_308
EDRYKEKLLQKAKAEGVESIEELKKRLADQIEEKKKELNKIDPLRELEQHLNAGSRIHTNKE
HKTTKMSNKSNEKSGNVLPKDKPYKTLDDYLKLDKIKDLSKQEVEFLWRAKWSNRDDSLV
AVVPYVKTFQGMYKYAVKNPLFVLPLPRENAADGNKADKDSVPVELQYVQWQFAGPNTV
HCLITSLAEYKLHQDFAKPHTTIQFHLDLANDKDMVLMNGQVESDSNVSLQDAQLLLLN
VQRFYGAMGSETSIAKERIQLLEDFNKGSQNFDINKLIQLAQSMEN
11111111111111111111111111111111111111111111111111111111111111111111111111111111100
00000000000000000000000000000000000000000000000000000000000000000000111111111111000
00000000000000000000000000000000000000000000000000000000000000000000000000000000000
0000000000000000000000000000000000000

>Q9WY59_1j5w_0_285
MYLQDVIMKLNDFWASKGCLLEQPYDMEVGAGTFHPATFFGSLRKGPWKVAYVQPSRRPT
DGRYGENPNRLQRYFQYQVIIKPSPENSQELYLESLEYLGINLKEHDIRFVEDNWESPTLGA
WGVGWEVWLDGMEITQFTYFQQIGGISLKDIPLEITYGLERIAMYLQGVDNVYEVQWNEN
VKYGDVFLENEREFSVFNFEEANVGLLFRHFDEYEKEFYRLVEKNLYLPAYDYILKCSHTFN
LLDARGAISVSQRQTYVKRIQAMARKAARVFLEVQANENSPA
00000000000000000000000000000000000000000000000000000000000000000000000000000000000
00000000000000000000000000000000000000000000000000000000000000000000000000000000000
00000000000000000000000000000000000000000000000000000000000000000000000000000000000
000000000000000000000000000000011111
>Q9WYP6_1vk4_0_285
MITFIGHVSKDVNVVDGKREIAYGGGVVMGAITSSLLGVKTKVITKCTREDVSKFSFLRDNG
VEVVFLKSPRTTSIENRYGSDPDTRESFLISAADPFTESDLAFIEGEAVHINPLWYGEFPEDLIP
VLRRKVMFLSADAQGFVRVPENEKLVYRDWEMKEKYLKYLDLFKVDSREAETLTGTNDLR
ESCRIIRSFGAKIILATHASGVIVFDGNFYEASFRSWSLEGRTGRGDTCTAAFLVGFVFKKMSI
EKATKFAAAVTSVKMRHPGPLRREDLEAISGDQYF
00000000000000000000000000000000000000000000000000000000000000000000000000000000111
11000000000000000000000000000000000000000000000000000000000000000000000000000000000
00000000000000000000000000000000000000000000000000000000000000000000000000000000000
000000000000000000000000000000011111
>A3FMN7_2vla_0_284
MNYNPEEQFRCTIIRGKAKNMLDNLLPAYANIIDDICPCDKASFVKDFNNRLIEILGEETTK
KTLDNHRTEIAGKLFGMFYEDDEVIFPSGRTNKYIEDSDQPAFFKDICFKFQFPNGMDKLDK
VIEKVGAKIQIRQFPYILQVLLTADNNNIQLSKDDIAYYVLNSLQVLQGKIKPIEVIEKIIEDRS
NDITKKVRHPGKETSYSMQHIREQLNYLELANLIRIDGNLVKLNYREAENINYIAQFWGNK
PEFNAYKYDFTSEDDKKSFFKDWQQYYSNVNSHKS
00000000000000000000000000000000000000000000000000000000000000000000000000000000000
00000000000000000000000000000000000000000000000000000000000000000000000000000000000
00000000000000000000000000000000000000000000000000000000000000000000000000000000000
00000000000000000000000000000011111
>P22634_2jfn_0_284
MATKLQDGNTPCLAATPSEPRPTVLVFDSGVGGLSVYDEIRHLLPDLHYIYAFDNVAFPYGE
KSEAFIVERVVAIVTAVQERYPLALAVVACNTASTVSLPALREKFDFPVVGVVPAIKPAARLT
ANGIVGLLATRGTVKRSYTHELIARFANECQIEMLGSAEMVELAEAKLHGEDVSLDALKRI
LRPWLRMKEPPDTVVLGCTHFPLLQEELLQVLPEGTRLVDSGAAIARRTAWLLEHEAPDAK
SADANIAFCMAMTPGAEQLLPVLQRYGFETLEKLAVLG
11111111111111111111000000000000000000000000000000000000000000000000000000000000000
00000000000000000000000000000000000000000000000000000000000000000000000000000000000
00000000000000000000000000000000000000000000000000000000000000000000000000000000000
00000000000000000000000000000000000
>Q28MS9_3c24_0_284
MVKDKNDVGPKTVAILGAGGKMGARITRKIHDSAHHLAAIEIAPEGRDRLQGMGIPLTDG
DGWIDEADVVVLALPDNIIEKVAEDIVPRVRPGTIVLILDAAAPYAGVMPERADITYFIGHP
CHPPLFNDETDPAARTDYHGGIAKQAIVCALMQGPEEHYAIGADICETMWSPVTRTHRVT
TEQLAILEPGLSEMVAMPFVETMVHAVDECADRYGIDRQAALDFMIGHLNVEIAMWFGYS
PKVPSDAALRLMEFAKDIVVKEDWREALNPAKVKQAAELIAGK
11110000000000000000000000000000000000000000000000000000000000000000000000000000000
00000000000000000000000000000000000000000000000000000000000000000000000000000000000
00000000000000000000000000000000000000000000000000000000000000000000000000000001110
00000000000000000000000000000000001
>Q5HF73_2f9i_0_284

MFKDFFNRTKKKKYLTVQDSKNNDVPAGIMTKCPKCKKIMYTKELAENLNVCFNCDHHI
ALTAYKRIEAISDEGSFTEFDKGMTSANPLDFPSYLEKIEKDQQKTGLKEAVVTGTAQLDGM
KFGVAVMDSRFRMGSMGSVIGEKICRIIDYCTENRLPFILFSASGGARMQEGIISLMQMGKTS
VSLKRHSDAGLLYISYLTHPTTGGVSASFASVGDINLSEPKALIGFAGRRVIEQTINEKLPDDF
QTAEFLLEHGQLDKVVHRNDMRQTLSEILKIHQEVTK
1111111111111111111111111000000000000000000000000000000000000000000000000000000000
00000000000000000000000000000000000000000000000000000000000000000000000000000000000
00000000000000000000000000000000000000000000000000000000000000000000000000000000000
000000000000000000000000000000000000
>Q5LXE3_3di4_0_284
MTDAPADAPLDADARRAVKPVICYPNDSLPRPDLALYRAARASARKTGEVLVPPREGRCFE
VKAGQFFRISSVEGPQVGDLNLHNLHDLTERFFSGKTRALHGTHVTTGERLWSNLPYLRPM
ATIIEDTLGWYGIDQYGGSVHDVIGTRCDPYTGNLLAGGHYHHCCHSNLTRALADHTGLP
LHEAEMLVHDVLNVFMCTGFTRDTGQYFMKASPVRPGDYLEFFAEIDLLGNLSACPGGDC
SSEHSSDTASCHPLLVEIFAPAEGMLGDWPSPSVNGYDRSHGR
00000000000000000000000000000000000000000000000000000000000000000000000000000000000
00000000000000000000000000000000000000000000000000000000000000000000000000000000000
00000000000000000000000000000000000000000000000000000000000000000000000000000001111
100000000000000000000000000000000000
>Q82IZ1_2rdn_0_284
MTNVTGDYTDCTPLLGDRAALDSFYEEHGYLFLRNVLDRDLVKTVAEQMREGLVALGAA
DPHATLEELTIDSFESVDEVAMHDYVKYDAFWNNPSTIKVFEQVFGEPVFVFLSTTIRYYPSQ
AGSEEPSFHYLTPFHQDGFYIGPNQDFRTFWIPLIRTTRESGGVALADGSHRRGKRDHVLNE
SFRRFGHPVRGIPPTEVSEDEHLLHSPMEPGDILLFHAHMCHKSIPNLSKDPRLMRMSMDT
RVQPAKSHRGFNAMTPWTESAKDASKGIMAKITGTPTDVE
10000000000000000000000000000000000000000000000000000000000000000000000000000000000
00000000000000000000000000000000000000000000000000000000000000000000000000000000000
00000000000000000000000000000000000000000000000000000000000000000000000000000000000
000000000000000000000000000111111111
>Q9RGF1_2p2v_0_284
MTRTRMENELIVSKNMQNIIIAGNGPSLKNINYKRLPREYDVFRCNQFYFEDKYYLGKKIK
AVFFNPGVFLQQYHTAKQLILKNEYEIKNIFCSTFNLPFIESNDFLHQFYNFFPDAKLGYEVI
ENLKEFYAYIKYNEIYFNKRITSGVYMCAIAIALGYKTIYLCGIDFYEGDVIYPFEAMSTNIKTI
FPGIKDFKPSNCHSKEYDIEALKLLKSIYKVNIYALCDDSILANHFPLSININNNFTLENKHN
NSINDILLTDNTPGVSFYKNQLKADNKIMLNFY
11110000000000000000000000000000000000000000000000000000000000000000000000000000000
00000000000000000000000000000000000000000000000000000000000000000000000000000000000
00000000000000000000000000110000000000000000000000000000000000000000000000000000000
000000000000000000000000000000001111
>Q9Y263_3ebb_510_794
MAGVDPFTGNSAYRSAASKTMNIYFPKKEAVTFDQANPTQILGKLKELNGTAPEEKKLTED
DLILLEKILSLICNSSSEKPTVQQLQILWKAINCPEDIVFPALDILRLSIKHPSVNENFCNEKEG
AQFSSHLINLLNPKGKPANQLLALRTFCNCFVGQAGQKLMMSQRESLMSHAIELKSGSNK
NIHIALATLALNYSVCFHKDHNIEGKAQCLSLISTILEVVQDLEATFRLLVALGTLISDDSNA
VQLAKSLGVDSQIKKYSSVSEPAKVSECCRFILNLL
11111111111111111111000000000000000000000000000000000000000000000000000000000000000
00000000000000000000000000000000000000000000000000000000000000000000000000000000000
00000000000000000000000000000000000000000000000000000000000000000000000000000000000
000000000000000000000000000000000000
>P04043_2dpm_0_283
MKIKEIKKVTLQPFTKWTGGKRQLLPVIRELIPKTYNRYFEPFVGGGALFFDLAPKDAVIND

FNAELINCYQQIKDNPQELIEILKVHQEYNSKEYYLDLRSADRDERIDMMSEVQRAARILYM
LRVNFNGLYRVNSKNQFNVPYGRYKNPKIVDEELISAISVYINNNQLEIKVGDFEKAIVDVR
TGDFVYFDPPYIPLSETSAFTSYTHEGFSFADQVRLRDAFKRLSDTGAYVMLSNSSSALVEELY
KDFNIHYVEATRTNGAKSSSRGKISEIIVTNYEK
11111111100000000000000000000000000000000000000000000000000000000000000000000000000
00000000000000000000000000000000000000000000000000000000000000000000000000000000000
00000000000000000000000000000000011111000000000000000000000000000000000000000000000
00000000001111111111110000000000000
>P80561_1xjo_0_283
APDIPLANVKAHLTQLSTIAANNGGNRAHGRPGYKASVDYVKAKLDAAGYTTTLQQFTS
GGATGYNLIANWPGGDPNKVLMAGAHLDSVSSGAGINDNGSGSAAVLETALAVSRAGYQ
PDKHLRFAWWGAEELGLIGSKFYVNNLPSADRSKLAGYLNFDMIGSPNPGYFVYDDDPVIE
KTFKNYFAGLNVPTEIETEGDGRSDHAPFKNVGVPVGGLFTGAGYTKSAAQAQKWGGTA
GQAFDRCYHSSCDSLSNINDTALDRNSDAAAHAIWTLSSGTGEPPT
00000000000000000000000000000000000000000000000000000000000000000000000000000000000
00000000000000000000000000000000000000000000000000000000000000000000000000000000000
00000000000000000000000000000111111000000000000000000000000000000000000000000000000
00000000000000000000000000001111111
>Q8FV25_3eg4_0_283
MTKPDLASLEKTIEKAFDERDGINTATRGEVREAVEQSLILLDRGEVRVAEKQADGNWHV
NQWLKKAVLLSFRLNPMEVIKGGPGQSSWWDKVPSKFDGWTANEFEKAGFRAVPNCIVR
HSAYIAPNAILMPSFVNLGAYVDKGAMIDTWATVGSCAQIGKNVHLSGGVGIGGVLEPMQ
AGPTIIEDNCFIGARSEVVEGCIVREGSVLGMGVFIGKSTKIVDRATGEVFYGEVPPYSVVVA
GTMPGKNVPGENWGPSLYCAVIVKRADEKTRSKTSINELLRD
11111000000000000000000000000000000000000000000000000000000000000000000000000000000
00000000000000000000000000000000000000000000000000000000000000000000000000000000000
00000000000000000000000000000000000000000000000000000000000000000000000000000000000
00000000000000000000000000000000000
>Q8PYS1_3ipi_11_294
NIEEWEEYRYVEAGIKESITLIEDPGLKKMVEHVCHSGGKRIRPIILLLVSEICSGSYSRSLNAA
LAVEMMHSASLIHDDLLDQGLVRRNLPSAPEKFGPSGALLCGDYLIAKSIAFISPYGEKVIQ
DFGKAGMDMAEGEVLDLKLEDESFGENDYFKCIYKKTASLFAISASIGAYTGGAEEELAERF
SHFGNALGTAYQIVDDILEFLEVVEGKESKFTSETLPHIYMKSTSKEEALKKSIDCVKLHVAA
AKETLETFRECPARDKLFQITDYITVDMLENL
00000000000000000000000000000000000000000000000000000000000000000000000000000000000
00000000000000000000000000000000000000000000000000000000000000111111100000000000000
00000000000000000000000000000000000000000000000001111111000000000000000000000000000
00000000000000000000000000000000011
>Q8THK8_3g40_474_757
EWAAKKTNSLTSSSERTWKANLLVPVEDPRELMGTFDFLRDITYPKGSVKLLGLAGNTDKE
NLLSQLPSISEGFQEEGVFSSWTIIDTAEFEENLVVGMEALTGSFFRPSILFLRLPENRDRDEEI
REIIRKASMYRMGVLLFSKHPQAGLGRQNLINLWIENRGLDWDISMELGNMDLALLIAYKL
KSNWKASLSFMTFAPTAIQAQAAENFLQSLAELARIPNVKMQVLRENPIKSSKLPFASLHIFS
LDPNPDLDLARHLMEKAGSSCIFALDSGEENALA
11111111111111111000000000000000000000000000000000000111000000000000000000000000000
00011111000000000000000000000000000000000000000000000000000000000000000000000000011
10000000000000000000000000000000000000000000000000000000000000000000000000000000000
00000000000000000000000000000000000
>Q9X1H5_1vkm_1_284
IIESRIEKGKPVVGMETTVFVHGLPRKEAIELFRRAKEISREKGFQLAVIGILKGKIVAGMSEEE
LEAMMREGADKVGTREIPIVVAEGKNAATTVSATIFLSRRIGIEVVVTGGTGGVHPGRVDVS

QDLTEMSSSRAVLVSSGIKSILDVEATFEMLETLEIPLVGFRTNEFPLFFSRKSGRRVPRIENVEE
VLKIYESMKEMELEKTLMVLNPVPEEYEIPHDEIERLLEKIELEVEGKEVTPFLLKKLVEMTN
GRTLKANLALLEENVKLAGEIAVKLKRS
00000000000000000000000000000000000000000000000000000000000000000000000000000000000
00000000000000000000000000000000000000000000000000000000000000000000000000000000000
00000000000000000000000000000000000000000000000000000000000000000000000000000000000
00000000000000000000000000000000001
>D0E8E2_3qit_1928_2210
MEEKFLEFGGNQICLCSWGSPEHPVVLCIHGILEQGLAWQEVALPLAAQGYRVVAPDLFG
HGRSSHLEMVTSYSSLTFLAQIDRVIQELPDQPLLLVGHSMGAMLATAIASVRPKKIKELILV
ELPLPAEESKKESAVNQLTTCLDYLSSTPQHPIFPDVATAASRLRQAIPSLSEEFSYILAQRITQ
PNQGGVRWSWDAIIRTRSILGLNNLPGGRSQYLEMLKSIQVPTTLVYGDSSKLNRPEDLQQ
QKMTMTQAKRVFLSGGHNLHIDAAAALASLILTS
00000000000000000000000000000000000000000000000000000000000000000000000000000000000
00000000000000000000000000000000000000000000000000000000000000000000000000000000000
00000000000000000000000000000000000000010000000000000000000000000000000000000000000
0000000000000000000000000000000000
>O94556_3zn3_18_300
QLREIRNCLLKCISECSERGLVYAVRWAAEMLNGMNPIEMEHIPFSSTPTGEFDLDPMANE
KLLEVEEKNIYLLAKSYFDCKEFERAAYTLQNCKSSKSIFLRLYSKYLAGEKKSEEENETLLNT
NLTLSSTNREFYYISEVLESLHYQGNKDPYLLYLSGVVYRKRKQDSKAIDFLKSCVLKAPFFW
SAWLELSLSIDSLETLTTVVSQLPSTHIMTKIFYVVYASHELHQVNSSAYEKLAEAEIIFPNSRYL
KTQRALLTYDSRDFDEAESLFENILTNDP
00000000000000000000000000000000000000111111111111111111110000000000000000000000000
00000000000000000000000000000000001111111111111111000000000000000000000000000000000
00000000000000000000000000000000000000000000000000000000000000000000000000000000000
0000000000000000011111111000000000
>B6ZK72_3rga_0_281
MPAETVRKEVALEYCRRVNAGELEGVLQLFAPDALLVDPLGTEPVVGRAALAARLAPALR
GAVHEEPGRPYAAHDGTSVVLPATVTVGAPGAPPQRRGRTRVMGVIEVGEDGLIREMRVM
WGVTDSSWTARPAPDEERRKELAREHCLRINDGDVDGLLKLYSPRIRFEDPVGSWTRTGLE
ALRAHATMAVGSNVRETAGLTVAGQDGRHAAVTVSATMDYLPSGPLLARHHLMTLPAPA
DPHRALIGIEYVMVIGVDADGLIDEMRAYWGATDVSLLDPAA
00000000000000000000000000000000000000000000000000000000000000000000000000000000000
00000000000000000000000000000000000000000000000000000000000000000000000000000000000
00000000000000000000000000000000000000000000000000000000000000000000000000000000000
000000000000000000000000000000011
>O30245_1zao_0_281
MNIAELYGKMGKHSWRIMDAIFKNLWDYEYVPLQLISSHARIGEEKARNILKYLSDLRVVQ
NRQKDYEGSTFTFIGLSLYSLHRLVRSGKVDAIGKLMGEGKESAVFNCYSEKFGECVVKFHK
VGHTSFKKVKEKRDYGDLHFSVLAIRSARNEFRALQKLQGLAVPKVYAWEGNAVLMELID
AKELYRVRVENPDEVLDMILEEVAKFYHRGIVHGDLSQYNVLVSEEGIWIIDFPQSVEVGEE
GWREILERDVRNIITYFSRTYRTEKDINSAIDRILQE
00000000000000000000000000000000000000000000000000000000000000000000000000000000000
00000000000000000000000000000000000000000000111000011111110000000000000000000000000
00000000000000000000000000000000000000000000000000000000000000000000000000000000000
000000000000000000000000000000000
>O76745_1ntf_20_301
GSPPAQLSVHTVSWNSGHERAPTNLEELLGLNSGETPDVIAVAVQGFGFQTDKPQQGPAC
VKNFQSLLTSKGYTKLKNTITETMGLTVYCLEKHLDQNTLKNETIIVTVDDQKKSGGIVTSF
TIYNKRFSFTTSRMSDEDVTSTNTKYAYDTRLDYSKKDDPSDFLFWIGDLNVRVETNATHAK

SLVDQNNIDGLMAFDQLKKAKEQKLFDGWTEPQVTFKPTYKFKPNTDEYDLSATPSWTDR
ALYKSGTGKTIQPLSYNSLTNYKQTEHRPVLAKFRVTL
11000000000000000000000000000000000000000000000000000000000000000000000000000000000
00000000000000000000000000000000000000000000000000000000000000000000000000000000000
00000000000000000000000000000000000000000000000000000000000000000000000000000000000
000000000000000000000000000000000
>Q8PSW8_3lia_31_312
IGTVTSQEEKLAYEKSIEMAGNYANQFDAQMEANQAIARTLACTMAEYGSQDREEAMSIIK
RILNENPQLIGVYLGYEPDAFDGRDKNYINAPGHDSTGRFVPYCNKINGPVIIEPLVHYDSS
DYYQLPKTTGKDTLTEPYFYEGIFMVSYDSPIFKNGEFAGIAGVDVPLEYVDDVASSIRTFDT
GYAFMVSNTGIFLSHPTQKNWIGEKSLSDFDVEEIKNAASDIREGIGGHVEIKDPITGKTVIM
FYEPVKTGDFSFVLVVPKEEMLAGVKDLRDRLL
11111111100000000000000000000000000000000000000000000000000000000000000000000000000
00000000000000000000000000000000000000000000000000000000000000000000000000000000000
00000000000000000000000000000000000000000000000000000000000000000000000000000000000
000000000000000000000001111111111
>Q970U6_3vpb_0_281
MRVVLIVDIVRQEEKLIAKALEENKVQYDIINVAQEPLPFNKALGRYDVAIIRPVSMYRALYS
SAVLEAAGVHTINSSDVINVCGDKILTYSKLYREGIPIPDSIIALSAEAALKAYEQRGFPLIDKP
PIGSWGRLVSLIRDVFEGKTIIEHRELMGNSALKAHIVQEYIQYKGRDIRCIAIGEELLGCYAR
NIPPNEWRANVALGGTPSNIEVDEKLKETVVKAVSIVHGEFVSIDILEHPNKGYVVNELND
VPEFKGFMVATNINVAQKLVEYIKENYSK
00000000000000000000000000000000000000000000000000000000000000000000000000000000000
00000000000000000000000000000000000000000000000000000000000000000000000000000000000
00000000000000000000000000000000000000000000000000000000000000000000000000000000000
000000000000000000000000000000001
>Q9HXK8_2hsi_0_281
MPRTLAFVSTLLLAAFCALPAQADSFIMRLLNKPVPGGVAVVDLGEEGPPPRAFYQGKPVL
VVREEGRRWIAVVGIPLSTKPGPQKLEVRAATGNHEERFSVGSKHYREQRITLKNKRQVNP
LPEDLKRIERELAEQTAAYRRFSPGLPSNLMLDKPVDGPLSSPFGLRRFFNGEERNPHSGLDF
AVPAGTPIKAPAAGKVILIGDYFFNGKTVFVDHGQGFISMFCHLSKIDVKLGQQVPRGGVL
GKVGATGRATGPHMHWNVSLNDARVDPAIFIGAFQP
11111111111111111111111100000000000000000000000000000000000000000000000000000000000
00000000000000000000001111111111111111100000000000000000000000000000000000000000000
01111111111100000000000000000000000000000000000000000000000000000000000000000000000
000000000000000000000000000000001
>A0A011_3vkc_0_280
MNASPQLDHHTELHAAPPLWRPGRVLARLREHQPGPVHIIDPFKVPVTEAVEKAAELTRL
GFAAVLLASTDYESFESHMEPYVAAVKAATPLPVVLHFPPRPGAGFPVVRGADALLLPALL
GSGDDYFVWKSFLETLAAFPGRIPREEWPELLLTVALTFGEDPRTGDLLGTVPVSTASTEEID
RYLHVARAFGFHMVYLYSRNEHVPPEVVRHFRKGLGPDQVLFVSGNVRSGRQVTEYLDSG
ADYVGFAGALEQPDWRSALAEIAGRRPAAPARPGSGR
11111111111111100000000000000000000000000000000000000000000000000000000000000000000
00000000000000000000000000000000000000000000000000000000000000000000000000000000000
00000000000000000000000000000000000000000000000000000000000000000000000000000000000
00000000000000000001111111111111
>B9PVB4_4eiv_5_285
IYKQFTSRTLLNFFEVAALTDGETNESVAAVCKIAAKDPAIVGVSVRPAFVRFIRQELVKSAPE
VAGIKVCAAVNFPEGTGTPDTVSLEAVGALKDGADEIECLIDWRRMNENVADGESRIRLLV
SEVKKVVGPKTLKVVLSGGELQGGDIISRAAVAALEGGADFLQTSSGLGATHATMFTVHLIS
IALREYMVRENERIRVEGINREGAAVRCIGIKIEVGDVHMAETADFLMQMIFENGPRSIVRD

KFRVGGGFNLLKELRDCYESWDSVGVSPDTSP
00000000000000000000000000000000000000000000000000000000000000000000000000000000
00000000000000000000000000000000000000000000000000000000000000000000000000000000
00000000000000000000000000000011111111111111100000000000000000000000000000000000
0000000000000000000000011111111
>O59416_2yzq_4_284
RVKTIMTQNPVTITLPATRNYALELFKKYKVRSFPVVNKEGKLVGIISVKRILVNPDEEQLAM
LVKRDVPVVKENDTLKKAAKLMLEYDYRRVVVVDSKGKPVGILTVGDIIRRYFAKSEKYKG
VEIEPYYQRYVSIVWEGTPLKAALKALLLSNSMALPVVDSEGNLVGIVDETDLLRDSEIVRIM
KSTELAASSEEEWILESHPTLLFEKFELQLPNKPVAEIMTRDVIVATPHMTVHEVALKMAKY
SIEQLPVIRGEGDLIGLIRDFDLLKVLVKSKA
00000000000000000111111111111100000000000000000001111111111000000000000000000000
00000000000000000000000000000000000000000000000000000000000000000000000000000000
00000000000000000011111111111111111111111111111110000000000000000000000000000000
0000000000000000000000000001111
>P78536_2i47_211_491
VKRRADPDPMKNTCKLLVVADHRFYRYMGRGEESTTTNYLIELIDRVDDIYRNTSWDNAG
FKGYGIQIEQIRILKSPQEVKPGEKHYNMAKSYPNEEKDAWDVKMLLEQFSFDIAEEASKVC
LAHLFTYQDFDMGTLGLAYVGSPRANSHGGVCPKAYYSPVGKKNIYLNSGLTSTKNYGKTI
LTKEADLVTTHELGHNFGAEHDPDGLAECAPNEDQGGKYVMYPIAVSGDHENNKMFSN
CSKQSIYKTIESKAQECFQERSNKVCGNSRVDEGEECDPG
11111110000000000000000000000000000000000000000000000000000000000000000000000000
00000000000000000000000000000000000000000000000000000000000000000000000000000000
00000000000000000000000000000000000000000000000000000000000000000000000000000000
0000000000000011111111111111111
>Q834X5_3cjm_32_312
AKESEQKVTIDSAKHEKHTKDKEENNSANTVFFDKINDLLVASVKEFEGTVGISYLDLETGE
QRSVNGQHEFYTASTIKVPLTMLVADTVASGQKKWTDLIPYNAEEDYEEGTGIIAYNIQPEY
PLKTLQEYAITYSDNIAKNMLYDTLGGDAKAKREMYQRYLHKTPSIEEPQFSSEDALVILQK
LYTEKATKPDYQAIYDSMKQSVFHERMETPTTQGKVAHKIGSYDEFIHDMGILETPHPFALA
IFTKGPDNAKSAAFIASVTDKLWQLQVSEYPNQ
11111111111111111111111110000000000000000000000000000000000000000000000000000000
00000000000000000000000000000000000000000000000000000000000000000000000000000000
00000000000000000000000000000000000000000000000000000000000000000000000000000000
0000000000000000000000000000000
>Q9KTK0_3uxv_0_280
MSKYSDAKELASLTLGKKTEYANQYDPSLLQPVPRSLNRNDLHLSATLPFQGCDIWTLYELS
WLNQKGLPQVAIGEVSIPATSANLIESKSFKLYLNSYNQTRFASWDEVQTRLVHDLSACAGE
TVTVNVKSLNEYTAEPIVTMQGECIDDQDIEIANYEFDDALLQGAAQGEEVSEVLHSHLLK
SNCLITNQPDWGSVEIAYHGAKMNREALLRYLVSFREHNEFHEQCVERIFTDIMRYCQPQS
LTVYARYTRRGGLDINPFRSSHQSAPNHNQRMARQ
11111111111111111111100000000000000000000000000000000000000000000000000000000000
00000000000000000000000000000000000000000000000000000000000000000000000000000000
00000000000000000000000000000000000000000000000000000000000000000000000000000000
0000000000000000000000000000000
>E0CX06_3oa3_0_279
MGTLEAQTQGPGSMSSLNNEEWDLLISGKKATLQYPIPLLCYPAPEVVSIAQIIDHTQLSLSA
TGSQIDVLCAEAKEYGFATVCVRPDYVSRAVQYLQGTQVGVTCVIGFHEGTYSTDQKVSEA
KRAMQNGASELDMVMNYPWLSEKRYTDVFQDIRAVRLAAKDAILKVILETSQLTADEIIAG
CVLSSLAGADYVKTSTGFNGPGASIENVSLMSAVCDSLQSETRVKASGGIRTIEDCVKMVRA
GAERLGASAGVKIVNETRLGNRQVDEPMEPTNY

11111111111110000000000000000000000000000000000000000000000000000000000000000000000
00000000000000000000000000000000000000000000000000000000000000000000000000000000000
00000000000000000000000000000000000000000000000000000000000000000000000000000000000
0000000000000000011111111111111
>P16218_2v3g_25_304
NYNSGLKIGAWVGTQPSESAIKSFQELQGRKLDIVHQFINWSTDFSWVRPYADAVYNNGSI
LMITWEPWEYNTVDIKNGKADAYITRMAQDMKAYGKEIWLRPLHEANGDWYPWAIGYSS
RVNTNETYIAAFRHIVDIFRANGATNVKWVFNVNCDNVGNGTSYLGHYPGDNYVDYTSI
DGYNWGTTQSWGSQWQSFDQVFSRAYQALASINKPIIIAEFASAEIGGNKARWITEAYNSIR
TSYNKVIAAVWFHENKETDWRINSSPEALAAYREAIGAG
11110000000000000000000000000000000000000000000000000000000000000000000000000000000
00000000000000000000000000000000000000000000000000000000000000000000000000000000000
00000000000000000000000000000000000000000000000000000000000000000000000000000000000
0000000000000000000000000000111
>P29016_2h26_18_297
EHAFQGPTSFHVIQTSSFTNSTWAQTQGSGWLDDLQIHGWDSDSGTAIFLKPWSKGNFSDK
EVAELEEIFRVYIFGFAREVQDFAGDFQMKYPFEIQGIAGCELHSGGAIVSFLRGALGGLDFL
SVKNASCVPSPEGGSRAQKFCALIIQYQGIMETVRILLYETCPRYLLGVLNAGKADLQRQVK
PEAWLSSGPSPGPGRLQLVCHVSGFYPKPVWVMWMRGEQEQQGTQLGDILPNANWTWY
LRATLDVADGEAAGLSCRVKHSSLEGQDIILYWRNP
11100000000000000000000000000000000000000000000000000000000000000000000000000000000
00000000000000000000000000000000000000000000000000000000000000000000000000000000000
00000000000000000000000000000000000000000000000000000000000000000000000000000000000
0000000000000000000000000000000
>P53078_3nuq_0_279
MTVEYTASDLATYQNEVNEQIAKNKAHLESLTHPGSKVTFPIDQDISATPQNPNLKVFFFDI
DNCLYKSSTRIHDLMQQSILRFFQTHLKLSPEDAHVLNNSYYKEYGLAIRGLVMFHKVNAL
EYNRLVDDSLPLQDILKPDIPLRNMLLRLRQSGKIDKLWLFTNAYKNHAIRCLRLLGIADLF
DGLTYCDYSRTDTLVCKPHVKAFEKAMKESGLARYENAYFIDDSGKNIETGIKLGMKTCIH
LVENEVNEILGQTPEGAIVISDILELPHVVSDLF
00000000000000000000000000000000000000000000111111111000000000000000000000000000000
00000000000000000000000000000000000000000000000000000000000000000000000000000000000
00000000000000000000000000000000000000000000000000000000000000000000000000000000000
0001111100000000000000000000000
>Q38814_1rp0_48_327
AGYDLNAFTFDPIKESIVSREMTRRYMTDMITYAETDVVVVGAGSAGLSAAYEISKNPNVQ
VAIIEQSVSPGGGAWLGGQLFSAMIVRKPAHLFLDEIGVAYDEQDTYVVVKHAALFTSTIMS
KLLARPNVKLFNAVAAEDLIVKGNRVGGVVTNWALVAQNHHTQSCMDPNVMEAKIVVS
SCGHDGPFGATGVKRLKSIGMIDHVPGMKALDMNTAEDAIVRLTREVVPGMIVTGMEVAE
IDGAPRMGPTFGAMMISGQKAGQLALKALGLPNAIDGTL
11000000000000000000000000000000000000000000000000000000000000000000000000000000000
00000000000000000000000000000000000000000000000000000000000000000000000000000000000
00000000000000000000000000000000000000000000000000000000000000000000000000000000000
0000000000000000000000000000000
>Q5HSE9_3npk_1_280
NLKELFIHHLEKNLPKVESFHPFFNEALALMLKAGGKHFRAQLLLSVVQSNKPELLNQAL
DVALALEFIHTYSLIHDDLPAMDNADFRRGIPTLHKSYDETTAILVGDALNTEAFLVLSHAH
LKDEIKIKLIKTLAFNAGLNGMVIGQAIDCFFEDKRLSLNELEFLHTHKTARLIAAALKMGC
EICELNNEESNQIYKLGLKLGLIFQINDDIIDVTTSQEQSGKPTNNDIHKNSFVNLLGLEQAI
KTKENLLNECEQDLEKLNEKLAQMIQNLIIQYL
00000000000000000000000000000000000000000000000000000000000000000000000000000000000

00000000000000000000000000000000000000000000000000000000000000000000000000000000000
0000000000000000000000000000000000000000000000000000111111111111111000000000000000000
0000000000000000000000000000000
>Q742X5_3tjr_0_279
MDGFLSGFDGRAAVVTGGASGIGLATATEFARRGARLVLSDVDQPALEQAVNGLRGQGFD
AHGVVCDVRHLDEMVRLADEAFRLLGGVDVVFSNAGIVVAGPLAQMNHDDWRWVIDID
LWGSIHAVEAFLPRLLEQGTGGHIAFTASFAGLVPNAGLGTYGVAKYGVVGLAETLAREVK
PNGIGVSVLCPMVVETKLVSNSERIRGADYGMSATPEGAFGPLPTQDESVSADDVARLTAD
AILANRLYILPHAAARESIRRRFERIDRTFDEQAAEGWTH
11000000000000000000000000000000000000000000000000000000000000000000000000000000000
00000000000000000000000000000000000000000000000000000000000000000000000000000000000
00000000000000000000000000000000000000000111111111111000000000000000000000000000000
0000000000000000000000000000000
>Q827Q5_3v75_0_279
MSLEPFGARLSRAMDDRGPLCVGIDPHASLLADWGLSDDVAGLERFSRTVVEALGEHVAV
FKPQSAFFERFGSRGVAVLEKTVAEARAAGALVVMDAKRGDIGSTMAAYAEAFLRKDSPLF
SDALTVSPYLGYGSLRPAVELARESGAGLFVLALTSNPEGGEVQHAVRTDGRDVAATMLAH
LAAENAGEEPLGSFGAVVGATLGDLSSYDLDINGPLLAPGIGAQGAAPADLPGVFGAAVR
NVVPNVSRGVLRHGPDVRALRTAADRFAEEIRAAVAAV
00000000000000000000000000000000000000000000000000000000000000000000000000000000000
00000000000000000000000000000000000000000000000000000000000000000000000000000000000
00000000000000000000000000000000000000000000000000000000000000000000000000000000000
0000000000000000000000000000001
>F2QVG4_2w2e_0_278
MPDIENQAADGQAEIKPEDAPYITNAYKPAYARWGFGSDSVRNHFIAMSGEFVGTFLFLWS
AFVIAQIANQAPETPDGGSNPAQLIMISFGFGFGVMVGVFITYRVSGGNLNPAVTLALVLAR
AIPPFRGILMAFTQIVAGMAAAGAASAMTPGEIAFANALGGGASRTRGLFLEAFGTAILCLT
VLMLAVEKHRATWFAPFVIGIALLIAHLICIYYTGAGLNPARSFGPAVAARSFPNYHWIYWL
GPILGAFLAYSIWQMWKWLNYQTTNPGQDSDA
11111111110000000000000000000000000000000000000000000000000000000000000000000000000
00000000000000000000000000000000000000000000000000000000000000000000000000000000000
00000000000000000000000000000000000000000000000000000000000000000000000000000000000
00000000000000000000000111111
>G3XAQ0_3t3w_0_278
GSMVAPSWRRPSRPEQRTEMYIDYDVSDRIATITLNRPEAANAQNPELLDELDAAWTRAAE
DNDVSVIVLRANGKHFSAGHDLRGGGPVPDKLTLEFIYAHESRRYLEYSLRWRNVPKPSIA
AVQGRCISGGLLLCWPCDLIIAAEDALFSDPVVLMDIGGVEYHGHTWELGPRKAKEILFTG
RAMTAEEVAQTGMVNRVVPRDRLDAETRALAGEIAKMPPFALRQAKRAVNQTLDVQGFY
AAIQSVFDIHQTGHGNAMSVSGWPVLVDIEEMKANIK
11111111111111111100000000000000000000000000000000000000000000000000000000000000000
00111110000000000000000000000000000000000000000000000000000000000000000000000000000
00000000000000000000000000000000000000000000000000000000000000000000000000000000000
000000000000000000011111111111
>Q83S74_2ia4_23_301
DAAPAAGSTLDKIAKNGVIVVGHRESSVPFSYYDNQQKVVGYSQDYSNAIVEAVKKKLNK
PDLQVKLIPITSQNRIPLLQNGTFDFECGSTTNNVERQKQAAFSDTIFVVGTRLLTKKGGDIK
DFADLKGKAVVVTSGTTSEVLLNKLNEEQKMNMRIISAKDHGDSFRTLESGRAVAFMMDD
ALLAGERAKAKKPDNWDIVGKPQSQEAYGCMLRKDDPQFKKLMDDTIAQVQTSGEAEK
WFDKWFKNPIPPKNLNMNFELSDEMKALFKEPNDKALN
10000000000000000000000000000000000000000000000000000000000000000000000000000000000
00000000000000000000000000000000000000000000000000000000000000000000000000000000000

000000000000000000000000000000000000000000000000000000000000000000000000000000000000
000000000000000000000000000000
>Q97DC5_3g85_56_334
LRSKNSQSKPTIALYWSSDISVNIISRFLRGLQSKLAKQNYNYNVVICPYKTDCLHLEKGISKE
NSFDAAIIANISNYDLEYLNKASLTLPIILFNRLSNKYSSVNVDNYKMGEKASLLFAKKRYKS
AAAILTESLNDAMDNRNKGFIETCHKNGIKISENHIIAAENSIHGGVDAAKKLMKLKNTPK
ALFCNSDSIALGVISVLNKRQISIPDDIEIVAIGMNDREYTEFSTPPVTIVDIPIEEMAGTCISLV
EKLINRDIENPTSILFDGPLILRNS
11111111000000000000000000000000000000000000000000000000000000000000000000000000000
00000000000000000000000000000000000000000000000000000000000000000000000000000000000
00000000000000000000000000000000000000000000000000000000000000000000000000000000000
000000000000000000000000000001
>Q9HUY3_1w4t_0_278
MTPLTPEQTHAYLHHIGIDDPGPPSLANLDRLIDAHLRRVAFENLDVLLDRPIEIDADKVFA
KVVEGSRGGYCFELNSLFARLLLALGYELELLVARVRWGLPDDAPLTQQSHLMLRLYLAEG
EFLVDVGFGSANPPRALPLPGDEADAGQVHCVRLVDPHAGLYESAVRGRSGWLPLYRFDL
RPQLWIDYIPRNWYTSTHPHSVFRQGLKAAITEGDLRLTLADGLFGQRAGNGETLQRQLR
DVEELLDILQTRFRLRLDPASEVPALARRLAGLISA
00000000000000000000000000000000000000000000000000000000000000000000000000000000000
00000000000000000000000000000000000000000000000000000000000000000000000000000000000
00000000000000000000000000000000000000000000000000000000000000000000000000000000000
000000000000000000000000000011
>Q9PIC1_3ru6_0_278
MKLCVALDLSTKEECLQLAKELKNLDIWLKVGLRAYLRDGFKFIEELKKVDDFKIFLDLKFH
DIPNTMADACEEVSKLGVDMINIHASAGKIAIQEVMTRLSKFSKRPLVLAVSALTSFDEENF
FSIYRQKIEEAVINFSKISYENGLDGMVCSVFESKKIKEHTSSNFLTLTPGIRPFGETNDDQKRV
ANLAMARENLSDYIVVGRPIYKNENPRAVCEKILNKIHRKNISENDIEQNYEVIQQKEWDM
CNHFEEWIKTRPDKEHALKEFYAKCGIKY
00000000000000000000000000000000000000000000000000000000000000000000000000000000000
00000000000000000000000000000000000000000000000000000000000000000000000000000000000
00000000000000111111110000000000000000000000000000000000000001111111111111111111111
111111111111111111111111111111
>B8GY04_2xit_0_277
MAETRVIVVGNEKGGAGKSTIAVHLVTALLYGGAKVAVIDLDLRQRTSARFFENRRAWLD
NKKIELPEPLALNLSDNDVALAERPEEEQVAGFEAAFARAMAECDFILIDTPGGDSAITRMA
HGRADLVVTPMNDSFVDFDMLGTVDPVTLELTKPSLYSLTVWEGRKQRALSGQRQAMDW
VVLRNRLATTEARNRKRLEDRLNALAKRVGFRIGPGLRDRVIYRELFPFGLTIADLSPQVRP
VPVSLQHLAARQELRALMHSLGLSAYSGETMLAAQ
10000000000000000000000000000000000000000000000000000000000000000000000000000000000
00000000000000000000000000000000000000000000000000000000000000000000000000000000000
00000000000000000000000000000000000000000000000000000000000000000000000000000000000
00000000000000000000000011111
>D0VX07_3fdj_0_277
SNAMRLVADSACDIKELRGMVFKAVPLTISTDNEEFCDDGQLDIHRMLDILEKHKGRSYTA
CPGIDAWLEAFGDDDEIFVVTITAGMSGTYNSAMAARAVYLEEHPQAKVRVIDSKSTGPQ
MRIILEQLQQMIEEGKKFEEIDGAIDAYMQKTRLFCSLKSLHNLAQNGRVSKVVASAAEVL
GISVIGTASSHGTLEAIGKCRGDKKLLVKLQALLDDAGYEGGKLRICHVENEALADKIADM
IKQAYGTTDVCVYKAGGLCSYYAERGGIILSCETK
11000000000000000000000000000000000000000000000000000000000000000000000000000000000
00000000000000000000000000000000000000000000000000000000000000000000000000000000000
00000000000000000000000000000000000000000000000000000000000000000000000000000000000

00000000000000000000000000000
>P49638_1oiz_0_277
MAEARSQPSAGPQLNALPDHSPLLQPGLAALRRRAREAGVPLAPLPLTDSFLLRFLRARDF
DLDLAWRLLKNYYKWRAECPEISADLHPRSIIGLLKAGYHGVLRSRDPTGSKVLIYRIAHW
DPKVFTAYDVFRVSLITSELIVQEVETQRNGIKAIFDLEGWQFSHAFQITPSVAKKIAAVLTDS
FPLKVRGIHLINEPVIFHAVFSMIKPFLTEKIKERIHMHGNNYKQSLLQHFPDILPLEYGGEEF
SMEDICQEWTNFIMKSEDYLSSISESIQ
11111111000000000000000000000000000000000000000000000000000000000000000000000000000
00000000000000000000000000000000000000000000000000000000000000000000000000000000000
00000000000000000000000000000000000000000000000000000000000000000000000000000000000
00000000000000000000000000111
>Q15036_4gxb_110_387
QVPTEEVSLEVLLSNGQKVLVNVLTSDQTEDVLEAVAAKLDLPDDLIGYFSLFLVREKEDGA
FSFVRKLQEFELPYVSVTSLRSQEYKIVLRKSYWDSAYDDDVMENRVGLNLLYAQTVSDIER
GWILVTKEQHRQLKSLQEKVSKKEFLRLAQTLRHYGYLRFDACVADFPEKDCPVVVSAGN
SELSLQLRLPGQQLREGSFRVTRMRCWRVTSSVPLPSGSTSSPGRGRGEVRLELAFEYLMSKD
RLQWVTITSPQAIMMSICLQSMVDELMVKKS
10000000000000000000000000000000000000000000000000000000000000000000000000000000000
00000000000000000000000000000000000000000000000000000000000000000000000000000000000
00000000000000000000000001111111000000000000000000000000111111100000000000000000000
00000000000000000000000000000
>Q9TTF5_3p5s_0_277
MADCCTPRKKLCWCVLLFFLVAGITGGAIGIYKWHYSGLNRWHGAGSTADFQKIIQERCD
TYTQTIRPGSRSRNCQAIRQAFMSAFISKDPCKATKEDYNSLINLAPPTVPCGQQVFWSKTK
ELAHEYAKRRRLMTLEDTLLGYLADGLRWCGEPGSSDLNIWSCPDWRKDCRTNYLSVFWE
VLSERFAESACNTVRVVLNGSLENAFDSMSIFGRVEAPNLRPQVELEAWLVHDTGKPPSDS
CSGSSIRKLKSILDGRNVKFRCMDNLSRDQFLQRS
11111111111111111111111111111111111111110000000000000000000000000000000000000000000
00000000000000000000000000000000000000000000000000000000000000000000000000000000000
00000000000000000000000000000000000000000000000000000000000000000000000000000000000
00000000000000000000000000000
>C6KSX0_2ymo_27_303
NLTCDFNDVYKLEFHPNQQTSVTKLCNLTPNVLEKVTIKCGSDKLNYNLYPPTCFEEVYAS
RNMMHLKKIKEFVIGSSMFMRRSLTPNKINEVSFRIPPNMMPEKPIYCFCENKKTITINGSN
GNPSSKKDIINRGIVEIIIPSLNEKVKGCDFTTSESTIFSKGYSINEISNKSSNNQQDIVCTVKA
HANDLIGFKCPSNYSVEPHDCFVSAFNLSGKNENLENKLKLTNIIMDHYNNTFYSRLPSLIS
DNWKFFCVCSKDNEKKLVFTVEASISS
10000000000000000000000000000000000000000000000000000000111111111111111111111111111
11111100000000000000000000000000111111111111111000000000000000000000000000000000000
00000001111100000000000000000000000000000000000000001110000000000000000000000000000
0000000000000000000000000000
>D1AC29_3sii_39_315
RHSRRAIAAETVEILERGRYTAPSGRVVPIADHVAQAVRGTRLYRPEKLAVLLEGLGAASDG
APTRIEVTEETTLAAARRLTGAAGDQVACLNFASAEHPGGGFLSGAHAQEEGLARSSGLYA
SLRAVPQFYAFHHRQRDPLYSDHLIYSPGVPVFRDDAGRLLEEPYRVAFLTSPAPNRRAIGDL
RTVEEIGRVLRGRAAKVLAAARHHGHRRLVLGAWGCGVFGNDPAQVAETFAGLLLDGGP
FAGRFAHVVFAVWDTAPGAPRHAAFARRFGSL
00000000000000000000000000000000000000000000000000000001111111100000000000000000000
00000000000000000000000000000000000000000000000000000000000000000000000000000000000
00000000000000000000000000000000000000000000000000000000000000000000000000000000000
0000000000000000000000000111

>O09008_2j0a_44_320
NPGPLELQLGDIFIAVKTTWAFHRSRLDLLLDTWVSRIRQQTFIFTDSPDERLQERLGPHLVV
TNCSAEHSHPALSCKMAAEFDAFLVSGLRWFCHVDDDNYVNPKALLQLLKTFPQDRDVY
VGKPSLNRPIHASELQSKNRTKLVRFWFATGGAGFCINRQLALKMVPWASGSHFVDTSALI
RLPDDCTVGYIIECKLGGRLQPSPLFHSHLETLQLLGAAQLPEQVTLSYGVFEGKLNVIKLP
GPFSHEEDPSRFRSLHCLLYPDTPWCPLLAAP
1111100000000000000000000000000000000000000000000000000000000000011111110000000000
000000000000000000000000000000000000000000000111111111111111111100000000000000000000
00000000000000000000000000000000000000000000000000000000000000000000000000000000000
0000000000000000000000000111
>P00642_2oxv_0_276
MSNKKQSNRLTEQHKLSQGVIGIFGDYAKAHDLAVGEVSKLVKKALSNEYPQLSFRYRDSI
KKTEINEALKKIDPDLGGTLFVSNSSIKPDGGIVEVKDDYGEWRVVLVAEAKHQGKDIINIR
NGLLVGKRGDQDLMAAGNAIERSHKNISEIANFMLSESHFPYVLFLEGSNFLTENISITRPD
GRVVNLEYNSGILNRLDRLTAANYGMPINSNLCINKFVNHKDKSIMLQAASIYTQGDGRE
WDSKIMFEIMFDISTTSLRVLGRDLFEQLTSK
11111111111111110000000000000000000000000000000000000000000000000000000000000000000
00000000000000000000000000000000000000000000000000000000000000000000000000000000000
00000000000000000000000000000000000000000000000000000000000000000000000000000000000
0000000000000000000000000000
>P01026_1qsj_1009_1285
CGEQNMIGMTPTVIAVHYLDQTEQWEKFGLEKRQEALELIKKGYTQQLAFKQPISAYAAFN
NRPPSTWLTAMWSRSFSLAANLIAIDSQVLCGAVKWLILEKQKPDGVFQEDGPVIHQEMIG
GFRNTKEADVSLTAFVLIALQEARDICEGQVNSLPGSINKAGEYLEASYLNLQRPYTVAIAG
YALALMNKLEEPYLTKFLNTAKDRNRWEEPGQQLYNVEATSYALLALLLLKDFDSVPPVVR
WLNDERYYGGGYGSTQATFMVFQALAQYRADV
00000000000000000000000000000000000000000000000000000000000000000000000000000000000
00000000000000000000000000000000000000000000000000000000000000000000000000000000000
00000000000000000000000000000000000000000000000000000000000000000000000000000000000
0000000000000000000000000001
>P32558_4ioy_674_957
GRTKRLDQIFVRPNPDTKRVPSTVFIHENGIRFQSPLRTDSRIDILFSNIKNLIFQSCKGELIVVI
HIHLKNPILMGKKKIQDVQFYREASDMSVDETGRFRRYGDEDELEQEQEERRKRAALDKEF
KYFADAIAEASNGLLTVENTFRDLGFQGVPNRSAVFCMPTTDCLVQLIEPPFLVINLEEVEICI
LERVQFGLKNFDMVFVYKDFNKPVTHINTVPIESLDFLKQWLTDMDIPYTVSTINLNWATI
MKSLQDDPYQFFLDGGWNFLATGSD
11000000000000000000000000000000000000000000000000000000000000000000000000000000000
00000000111111111111111000000000000000000000000000000000000000000000000000000000000
00000000000000000000000000000000000000000000000000000000000000000000000000000000000
0000000000011111111111111111
>P36161_3kfo_880_1156
KYGHVAWIQQILDGSYADAMNTLKNITVDDSKKGESLSECELHLNVAKLSSLLVEKDNLDI
NTLRKIQYNLDTIDAEKNISNKLKKGEVQICKRFKNGSIREVFNILVEELKSTTVVNLSDLVE
LYSMLDDEESLFIPLRLLSVDGNLLNFEVKKFLNALVWRRIVLLNASNEGDKLLQHIVKRVF
DEELPKNNDFPLPSVDLLCDKSLLTPEYISETYGRFPIDQNAIREEIYEEISQVETLNSDNSLEI
KLHSTIGSVAKEKNYTINYETNTVEY
11111111111111111111111111111111111111111111111111111111111111111000000000000000000
00000000000000000000000000000000000000000000000000000000000000000000000000000000000
00000000000000000000000000000000000000000000000000000000000000000000000000000000000
0000000000000000000000000000
>P39759_2qik_0_276

MNSLLFVYGTLRKHEKNHHLLAQSACINEQARTKGSLFAAKEGPTVVFNDEDEGYIYGEV
YEADELCIHKLDQFFQGYHKQTVFVETDVGIKIALIYFMNKDGCAGFTKISSGDWKEHQMI
SKSKNPIYYFAYGSCMDNARFQKAGVDHYFQDPVGRAVLKGYTTRFTLKREDGSRADMLE
DGGTTEGVLYRIPYSALSYLYKREGVESLTYRPAFVDVEAGGRHYKDCLTFLVLQKEAEIAPP
QHYQIEIERGAELYLSPEFTEKLKRHMNSLPKG
00000000000000000000000000000000110000000000000000000000000000000000000000000000000
00000000000000000011111000000000000000000000000000000000000000000000000000000000000
00000000000000000000000000000000000000000000000000000000000000000000000000000000000
0000000000000000000000000000
>P42574_4ehl_0_276
MENTENSVDSKSIKNLEPKIIHGSESMDSGISLDNSYKMDYPEMGLCIIINNKNFHKSTGMT
SRSGTDVDAANLRETFRNLKYEVRNKNDLTREEIVELMRDVSKEDHSKRSSFVCVLLSHGE
EGIIFGTNGPVDLKKITNFFRGDRCRSLTGKPKLFIIQACRGTELDCGIETDSGVDDDMACH
KIPVEADFLYAYSTAPGYYSWRNSKDGSWFIQSLCAMLKQYADKLEFMHILTRVNRKVATEF
ESFSFDATFHAKKQIPCIVSMLTKELYFYH
11111111111111111111111111111111100000000000000000000000000000000000000000000000000
00000000000000000000000000000000000000000000000000000000000000000000000000000000000
00000001111111111100000000000000000000000000000000000000000000000000000000000000000
0000000000000000000000000001
>Q5KZF0_2p17_0_276
MAIQRRIRRVKTVQMTTNSPIHRSGSVLEPGNWQEYDPFLLLMEDIFERGTFDVHPHRGIET
VTYVISGELEHFDSKAGHSTLGPGDVQWMTAGRGVVHKEDPASGSTVHSLQLWVNLPSAY
KMTEPRYQNLRSKDMPVRKEEGATIRVFSGSSKGVKAPTKNIVPVTMVEMIVEPGTTVVQD
LPGHYNGFLYILEGSGVFGADNIEGKAGQALFFSRHNRGEETELNVTAREKLRLLLYAGEPV
NEPVVAYGPFVMNTPEQIREAIRDYQEGRFGR
00000000000000000000000000000000000000000000000000000000000000000000000000000000000
00000000000000000000000000000000000000000000000000000000000000000000000000000000000
00000000000000000000000000000000000000000000000000000000000000000000000000000000000
1111111111111111111111111111
>Q88RA3_3pvj_0_276
MSLTITPLSPALGAQISGVDISRDISAEERDAIEQALLQHQVLFLRDQPINPEQQARFAARFG
DLHIHPIYPNVPDTPQVLVLDTAVTDVRDNAVWHTDVTFLPTPALGAVLSAKQLPAYGGDT
LWASGIAAFEALSAPLREMLDGLTATHDFTKSFPLERFGTTPQDLARWEATRRNNPPLSHPV
VRTHPVSGRKALFVNEGFTTRINELSELESDALLRLLFAHATRPEFSIRWRWQENDVAFWD
NRVTQHFAVDDYRPNRRVMHRATILGDAPF
10000000000000000000000000000000000000000000000000000000000000000000000000000000000
00000000000000000000000000000000000000000000000000000000000000000000000000000000000
00000000000000000000000000000000000000000000000000000000000000000000000000000000000
0000000000000000000000000000
>Q97U20_3ddj_0_276
MNIETLMIKNPPILSKEDRLGSAFKKINEGGIGRIIVANEKIEGLLTTRDLLSTVESYCKDSCSQ
GDLYHISTTPIIDYMTPNPVTVYNTSDEFTAINIMVTRNFGSLPVVDINDKPVGIVTEREFLLL
YKDLDEIFPVKVFMSTKVQTIYKEVRLDQAVKLMLRRGFRRLPVIDDDNKVVGIVTVVNAI
KQLAKAVDKLDPDYFYGKVVKDVMVTNLVTIDELASVNRAAAEMIVKRIGSLLILNKDNTI
RGIITERDLLIALHHILVMEKFKEKL
00000000000000000000000000000000000000000000000000000000000111000000000000000000000
00000000000000000000000000000000000000000000000000000000000000000000000000000000000
00000000000000000000000000000000000000000000000000000000000000000000000000000000000
0000000000000000000000000001
>A5F384_3gbg_0_275
MIGKKSFQTNVYRMSKFDTYIFNNLYINDYKMFWIDSGIAKLIDKNCLVSYEINSSSIILLKKN

SIQRFSLTSLSDENINVSVITISDSFIRSLKSYILGDLMIRNLYSENKDLLLWNCEHNDIAVLSEV
VNGFREINYSDEFLKVFFSGFFSKVEKKYNSIFITDDLDAMEKISCLVKSDITRNWRWADICG
ELRTNRMILKKELESRGVKFRELINSIRISYSISLMKTGEFKIKQIAYQSGFASVSYFSTVFKSTM
NVAPSEYLFMLTGVAEK
11100000000000000000000000000000000000000000000000000000000000000000000000000000000
00000000000000000111111111100000000000000000000000000000000000000000000000000000000
00000000000000000000000000000000000000000000000000000000000000000000000000000000000
000000000000000000000000111
>P96830_1ywf_0_275
MAVRELPGAWNFRDVADTATALRPGRLFRSSELSRLDDAGRATLRRLGITDVADLRSSREVA
RRGPGRVPDGIDVHLLPFPDLADDDADDSAPHETAFKRLLTNDGSNGESGESSQSINDAAT
RYMTDEYRQFPTRNGAQRALHRVVTLLAAGRPVLTHCFAGKDRTGFVVALVLEAVGLDRD
VIVADYLRSNDSVPQLRARISEMIQQRFDTELAPEVVTFTKARLSDGVLGVRAEYLAAARQT
IDETYGSLGGYLRDAGISQATVNRMRGVLLG
11100000000000000000000000000000000000000000000000000000000000000000000000000000000
00111111111111111111111111111110000000000000000000000000000000000000000000000000000
00000000000000000000000000000000000000000000000000000000000000000000000000000000000
000000000000000000000000001
>Q0TTH0_4gd5_0_275
MFKKRLIAIIGTIFIGATAMVGCNSGGSEAKSTNSVSISGSTSVGPVMEAEAEAFKTKKPDVSI
EINQIGSSAGIKNAMEGVSEIGMASRDLKGEEKQAGLKEVEIAYDGIALITHKNNPVKDLTL
VQIKDIYTGKITNWKELGGNDAPIVVVSREDGSGTRDAFQEIVGFKAEELTVNSQISDGSGN
IKSLVQGNENAIGYISFSYVDDSVSAVKVDGVEATPENVLNKSYKVSRPFLAVYKEENLTESG
KSFIDFILSEEGQDIVAKEHLIKVK
11111111111111111111111111111111100000000000000000000000000000000000000000000000000
00000000000000000000000000000000000000000000000000000000000000000000000000000000000
00000000000000000000000000000000000000000000000000000000000000000000000000000000000
000000000000000000000000000
>Q82L35_3giw_0_275
MGGAALPDNGWPADRIDTESAHSARIYDYIIGGKDYYPADKEAGDAMSREWPALPVHMR
ANRDWMNRAVAHLAKEAGIRQFLDIGTGIPTSPNLHEIAQSVAPESRVVYVDNDPIVLTLS
QGLLASTPEGRTAYVEADMLDPASILDAPELRDTLDLTRPVALTVIAIVHFVLDEDDAVGIVR
RLLEPLPSGSYLAMSIGTAEFAPQEVGRVAREYAARNMPMRLRTHAEAEEFFEGLELVEPGI
VQVHKWHPDAATADGIRDEDIAMYGAVARKP
11111111111111000000000000000000000000000000000000000000000000000000000000000000000
00000000000000000000000000000000000000000000000000000000000000000000000000000000000
00000000000000000000000000000000000000000000000000000000000000000000000000000000000
000000000000000000000000000
>Q82QL3_3gkb_1_276
RNDAYSTLRVSSEHGVARIILDNPPVNVIGATMMRELRTVLTTLADDSSVRVIVFSSADPEFF
LAHVDMRIGEKMDALQELAASAPADVNVFQAVGELIRHQPQVTIVKLAGKARGGGAEFV
AAADMAFAAAETAGLGQIEALMGIIPGGGGTQYLRGRVGRNRALEVVLTADLFDAETAAS
YGWINRALPADELDEYVDRVARNIAALPDGVIEAAKRSLPADDLKEGLLGENDAWAATFS
LPAAQQLISGGLKDGAQTPAGERDLEGLMRSVAR
11100000000000000000000000000000000000000000000000000000000000000000000000000000000
00000000000000000000000000000000000000000000000000000000000000000000000000000000000
00000000000000000000000000000000000000000000000000000000000000000000000000000000000
000000000000000000000000000
>A5ZFJ0_3no2_19_293
SSPQHLLVGGSGWNKIAIINKDTKEIVWEYPLEKGWECNSVAATKAGEILFSYSKGAKMITR
DGRELWNIAAPAGCEMQTARILPDGNALVAWCGHPSTILEVNMKGEVLSKTEFETGIERPH

AQFRQINKNKKGNYLVPLFATSEVREIAPNGQLLNSVKLSGTPFSSAFLDNGDCLVACGDA
HCFVQLNLESNRIVRRVNANDIEGVQLFFVAQLFPLQNGGLYICNWQGHDREAGKGKHP
QLVEIDSEGKVVWQLNDKVKFGMISTICPIRE
10000000000000000000000000000000000000000000000000000000000000000000000000000000000
00000000000000000000000000000000000000000000000000000000000000000000000000000000000
00000000000000000000000000000000000000000000000000000000000000000000000000000000000
00000000000000000000000000
>A6VL88_3c3k_52_326
LRTAKTGMLLVMVSNIANPFCAAVVKGIEKTAEKNGYRILLCNTESDLARSRSCLTLLSGKM
VDGVITMDALSELPELQNIIGAFPWVQCAEYDPLSTVSSVSIDDVAASEYVVDQLVKSGKKR
IALINHDLAYQYAQHRESGYLNRLKFHGLDYSRISYAENLDYMAGKLATFSLLKSAVKPDAI
FAISDVLAAGAIQALTESGLSIPQDVAVVGFDGVDISQITVPALTTVQQPSEQIGMKAVSLLLE
QIHSDVLAKTVHHLLPWKFVRRQSS
11110000000000000000000000000000000000000000000000000000000000000000000000000000000
00000000000000000000000000000000000000000000000000000000000000000000000000000000000
00000000000000000000000000000000000000000000000000000000000000000000000000000000000
00000000000000000000000000
>O04358_3mvg_23_297
IETVEFRVTGTTRRSYSDFLQNLRNRLSSGTSVHDIPLLPAQSGSQQDLLFVRLFDWGNRPIT
LVLNRVNAYVVAYQAQNRFYLLADTPANPQVYGNNPHRLTFIGSYGALQNVAKQNRENI
DLGINPLATAISTLHNWSPPTVETSVARSLIVLIQLVSETARFRAIEQRVTNNIIDQVTPIRYDN
FRPSVGIIDLQTNWQTLSTEVQRAEGGRFLQPVKLQVSVQQTVVISDVEKARTFCGLALLLR
WRPSQASLSSHDFAWRDIRSILDIGA
00000000000000000000000000000000000000000000000000000000000000000000000000000000000
00000000000000000000000000000000000000000000000000000000000000000000000000000000000
00000000000000000000000000000000000000000000000000000000000000000000000000000000000
00111111111111111111111111
>O51169_3tmg_15_289
CDEKKSSKNLKSVKIGYVNWGGETAATNVLKVVFEKMGYNAEIFSVTTSIMYQYLASGKID
GTVSSWVPTADKFYYEKLKTKFVDLGANYEGTIQGFVVPSYVPISSISELKGKGDKFKNKMI
GIDAGAGTQIVTEQALNYYGLSKEYELVPSSESVMLASLDSSIKRNEWILVPLWKPHWAFSR
YDIKFLDDPDLIMGGIESVHTLVRLGLENDDFDAYYVFDHFYWSDDLILPLMDKNDKEPGK
EYRNAVEFVEKNKEIVKTWVPEKYKTLFD
11111111110000000000000000000000000000000000000000000000000000000000000000000000000
00000000000000000000000000000000000000000000000000000000000000000000000000000000000
00000000000000000000000000000000000000000000000000000000000000000000000000000000000
00000000000000000000000000
>P0AFB1_1xnf_19_293
SNTSWRKSEVLAVPLQPTLQQEVILARMEQILASRALTDDERAQLLYERGVLYDSLGLRALA
RNDFSQALAIRPDMPEVFNYLGIYLTQAGNFDAAYEAFDSVLELDPTYNYAHLNRGIALYY
GGRDKLAQDDLLAFYQDDPNDPFRSLWLYLAEQKLDEKQAKEVLKQHFEKSDKEQWGW
NIVEFYLGNISEQTLMERLKADATDNTSLAEHLSETNFYLGKYYLSLGDLDSATALFKLAVA
NNVHNFVEHRYALLELSLLGQDQDDLAESDQQ
11100000000000000000000000000000000000000000000000000000000000000000000000000000000
00000000000000000000000000000000000000000000000000000000000000000000000000000000000
00000000000000000000000000000000000000000000000000000000000000000000000000000000000
00000000000000001111111111
>P83700_1uek_0_274
MERLAPAKVNLGLSVRFRREDGYHELHTLFAPFSLADRLVVEPVSSGLHFQGPYGRENLAY
RAASLYLEAAGQPGGVRILLEKRIPEGAGLGGGSSDAAQVLLALQALYPAEVDLFALARTL
GADVPFFLLGRGAEARGVGERLKPLALPPVPAVVFFPGLRVPTPLVYRAVRPEDFGPDLPVE

AILEALARGEEPPYWNSLEGPAFRLFPELKEVRGRMRALGLRGVLMSGSGSAFFGLAEGPD
HARRAAEALRAWGRAWAGTLGGGDAGSGPA
00000000000000000000000000000000000000000000000000000000000000000000000000000000000
00000000000000000000000000000000000000000000000000000000000000000000000000000000000
00000000000000000000000000000000000000000000000000000000000000000000000000000000000
00000000000000000001111111
>Q58435_3grv_0_274
MFKPKKKLGQCFLIDKNFVNKAVESANLTKDDVVLEIGLGKGILTEELAKNAKKVYVIEID
KSLEPYANKLKELYNNIEIIWGDALKVDLNKLDFNKVVANLPYQISSPITFKLIKRGFDLAVL
MYQYEFAKRMVAKEGTKDYGRLSVAVQSRADVEIVAKVPPSAFYPKPKVYSAIVKIKPNKG
KYHIENENFFDDFLRAIFQHRNKSVRKALIDSSKELNYNKDEMKKILEDFLNTNSEIKNLIN
EKVFKLSVKDIVNLSNEFYRFLQNRGRL
11111111100000000000000000000000000000000000000000000000000000000000000000000000000
00000000000000000000000000000000000000000000000000000000000000000000000000000000000
00000000000000000000000000000000000000000000000000000000000000000000000000000000000
00000000000000000000001111
>Q7BQR6_3oa8_0_274
MRRFAAGCLALALLVLPFVLTGARAAEDESEKEIERYRQMIEDPMANPGFLNVDRGEVLW
SEPRGTRNVSLETCDLGEGPGKLEGAYAHLPRYFADTGKVMDLEQRLLWCMETIQGRDTK
PLVAKPFSGPGRTSDMEDLVAFIANKSDGVKIKVALATPQEKEMYAIGEALFFRRSSINDFSC
STCHGAAGKRIRLQALPQLDVPGKDAQLTMATWPTYRVSQSALRTMQHRMWDCYRQMR
MPAPDYASEAVTALTLYLTKQAEGGELKVPSIKR
11111111111111111111111111111111111111111111000000000000000000000000000000000000000
00000000000000000000000000000000000000000000000000000000000000000000000000000000000
00000000000000000000000000000000000000000000000000000000000000000000000000000000000
00000000000000000000000001
>Q8VPD8_3mq0_0_274
MGQRGQVCQGKCMAEDQQSSQISDTVPALRRAVRILDLVAGSPRDLTAAELTRFLDLPKSS
AHGLLAVMTELDLLARSADGTLRIGPHSLRWANGFLSHLDIVSTFNDHLAQRHDLDPYTV
TLTVREGGEVVYIGCRNSAQPLGHTFRIGMRLPAPFTATGKILLSDLGPGELRMLFSQFPQPL
TSRSVAGLSQLEEELALTRARGYSIDDGQIREGMLCIGAAIRDYSGAASAGIAISLIRSEASDE
KIAYLGEELRTTANALSEKLGYRSQKD
11111111111111111110000000000000000000000000000000000000000000000000000000000000000
00000000000000000000000000000000000000000000000000000000000110000000000000000000000
00000000000000000000000000000000000000000000000000000000000000000000000000000000000
00000000000000000000000111
>A1SBA1_3m1t_0_273
MHSAALLQKVDELPRLPKAIAELLDVVNNEDSTVKAVSEKLSHDPVLSARVLRLANSARFG
CSREVGTIDDAVVRLGMQTLRTLVIASAVVGAVPKVEGFDLADFWGNTFEVAIICQELAKRL
GTLPEEAFTCGILHSIGELLIVNGDPAVAATISAAVADGADRNLMEKELLGYDNAEIGALLA
QSWKFTPHLVKGIQFQNHPKSAEPYSKLAGMLAMAKQIAADWDKIPDDERTSWLAQINIL
AGIKVDLGGLAEKLAKMHGQGMEMGKQLA
00000000000000000000000000000000000000000000000000000001111110000000000000000000000
00000000000000000000000000000000000000000000000000000000000000000000000000000000000
00000000000000000000000000000000000000000000000000000000000000000000000000000000000
0000000000000000000000000
>P06009_2wjn_0_273
MALLSFERKYRVRGGTLIGGDLFDFWVGPYFVGFFGVSAIFFIFLGVSLIGYAASQGPTWDPF
AISINPPDLKYGLGAAPLLEGGFWQAITVCALGAFISWMLREVEISRKLGIGWHVPLAFCVP
IFMFCVLQVFRPLLLGSWGHAFPYGILSHLDWVNNFGYQYLNWHYNPGHMSSVSFLFVN
AMALGLHGGLILSVANPGDGDKVKTAEHENQYFRDVVGYSIGALSIHRLGLFLASNIFLTG

AFGTIASGPFWTRGWPEWWGWWLDIPFWS
10000000000000000000000000000000000000000000000000000000000000000000000000000000000
00000000000000000000000000000000000000000000000000000000000000000000000000000000000
00000000000000000000000000000000000000000000000000000000000000000000000000000000000
0000000000000000000000000
>P16102_2jag_0_273
MSITSVPGVVDAGVLGAQSAAAVRENALLSSSLWVNVALAGIAILVFVYMGRTIRPGRPRLI
WGATLMIPLVSISSYLGLLSGLTVGMIEMPAGHALAGEMVRSQWGRYLTWALSTPMILLAL
GLLADVDLGSLFTVIAADIGMCVTGLAAAMTTSALLFRWAFYAISCAFFVVVLSALVTDWA
ASASSAGTAEIFDTLRVLTVVLWLGYPIVWAVGVEGLALVQSVGVTSWAYSVLDVFAKYVFA
FILLRWVANNERTVAVAGQTLGTMSSDD
11111111111111111111100000000000000000000000000000000000000000000000000000000000000
00000000000000000000000000000000000000000000000000000000000000000000000000000000000
00000000000000000000000000000000000000000000000000000000000000000000000000000000000
0000000000000011111111111
>P32169_2v2b_0_273
MQNITQSWFVQGMIKATTDAWLKGWDERNGGNLTLRLDDADIAPYHDNFHQQPRYIPLS
QPMPLLANTPFIVTGSGKFFRNVQLDPAANLGIVKVDSDGAGYHILWGLTNEAVPTSELPA
HFLSHCERIKATNGKDRVIMHCHATNLIALTYVLENDTAVFTRQLWEGSTECLVVFPDGVGI
LPWMVPGTDEIGQATAQEMQKHSLVLWPFHGVFGSGPTLDETFGLIDTAEKSAQVLVKVYS
MGGMKQTISREELIALGKRFGVTPLASALAL
10000000000000000000000000000000000000000000000000000000000000000000000000000000000
00000000000000000000000000000000000000000000000000000000000000000000000000000000000
00000000000000000000000000000000000000000000000000000000000000000000000000000000000
0000000000000000000000001
>P86927_1xdn_50_323
DQSDFSPYIEIDLPSESRIQSLHKSGLAAQEWVACEKVHGTNFGIYLINQGDHEVVRFAKRS
GIMDPNENFFGYHILIDEFTAQIRILNDLLKQKYGLSRVGRLVLNGELFGAKYKHPLVPKSE
KWCTLPNGKKFPIAGVQIQREPFPQYSPELHFFAFDIKYSVSGAEEDFVLLGYDEFVEFSSKV
PNLLYARALVRGTLDECLAFDVENFMTPLPALLGLGNYPLEGNLAEGVVIRHVRRGDPAVE
KHNVSTIIKLRCSSFMELKHPGKQKE
10000000000000000000000000000000000000000000000000000000000000000000000000000000000
00000000000000000000000000000000000000000000000000000000000000000000000000000000000
00000000000000000000000000000000000000000000000000000000000000000000000000000000000
0000000000000000011111111
>Q12L70_3b2y_0_273
MTSRSPFESFVWQSEIFNCQSNDIDAFYAQLAEEVNRLGLKKNTLGSVDSFAINLYQSASQR
SDLPSLLISSGFHGEEAAGPWGMLHFLRGLQPALFERVNLSLLPLVNPTGFKAGHRFNRFGE
NPNRGFTLENGKPTPNEHTSLEGKLLLEHAQLLCAASRDGILTCHEDVLMNETYVYSFEPT
QTPGRFSLGLRDALGQYFKLAKDGFIDECPVTDGVIFNHFDTSFEAFLVRSGAKLAACSETP
GQEDFDRRVQANSAAMGQFIAHCAPIL
00000000000000000000000000000000000000000000000000000000000000000000000000000000000
00000000000000000000000000000000000000000000000000000111111100000000000000000000000
00000000000000000000000000000000000000000000000000000000000000000000000000000000000
0000000000000000000000001
>Q99V45_1qy6_68_341
VILPNNDRHQITDTTNGHYAPVTYIQVEAPTGTFIASGVVVGKDTLLTNKHVVDATHGDP
HALKAFPSAINQDNYPNGGFTAEQITKYSGEGDLAIVKFSPNEQNKHIGEVVKPATMSNN
AETQVNQNITVTGYPGDKPVATMWESKGKITYLKGEAMQYDLSTTGGNSGSPVFNEKNEV
IGIHWGGVPNEFNGAVFINENVRNFLKQNIEDIHFANDDQPNNPDNPDNPNNPDNPNNP
DNPNNPDEPNNPDNPNNPDNPDNGDNNNSDNPDAA

00000000000000000000000000000000000000000000000000000000000000000000000000000000000
00000000000000000000000000000000000000000000000000000000000000000000000000000000000
00000000000000000000000000000000000000000000000000111111111111111111111111111111111
1111111111111111111111111
>P09914_4hou_6_278
DHQVKDSLEQLRCHFTWELSIDDDEMPDLENRVLDQIEFLDTKYSVGIHNLLAYVKHLKG
QNEEALKSLKEAENLMQEEHDNQANVRSLVTWGNFAWMYYHMGRLAEAQTYLDKVEN
ICKKLSNPFRYRMECPEIDCEEGWALLKCGGKNYERAKACFEKVLEVDPENPESSAGYAISA
YRLDGFKLATKNHKPFSLLPLRQAVRLNPDNGYIKVLLALKLQDEGQEAEGEKYIEEALAN
MSSQTYVFRYAAKFYRRKGSVDKALELLKKALQ
11000000000000000000000000000000000000000000000000000000000000000000000000000011111
10000000000000000000000000000000000000000000000000000000000000000000000000000000000
00000000000000000000000100000000000000000000000000000000000000000000000000000000000
000000000000000000000001
>P15559_1kbq_1_273
VGRRALIVLAHSERTSFNYAMKEAAAAALKKKGWEVVESDLYAMNFNPIISRKDITGKLKD
PANFQYPAESVLAYKEGHLSPDIVAEQKKLEAADLVIFQFPLQWFGVPAILKGWFERVFIGEF
AYTYAAMYDKGPFRSKKAVLSITTGGSGSMYSLQGIHGDMNVILWPIQSGILHFCGFQVLEP
QLTYSIGHTPADARIQILEGWKKRLENIWDETPLYFAPSSLFDLNFQAGFLMKKEVQDEEKN
KKFGLSVGHHLGKSIPTDNQIKARK
10000000000000000000000000000000000000000000000000000000000000000000000000000000000
00000000000000000000000000000000000000000000000000000000000000000000000000000000000
00000000000000000000000000000000000000000000000000000000000000000000000000000000000
000000000000000000000000
>Q2G8F3_3g23_0_272
MTRRIAICAPSTPFTREDSARVIALAAAEFPDLSLSFHEQCFASEGHFAGSDALRLSAFLECA
NDDAFEAVWFVRGGYGANRIAEDALARLGRAASAKQYLGYSDAGTLLAALYAHRIGRSV
HAPMPVDIRRPEGESAVRRTLGWLAGAREGLEPTLGAGAPAVAFNLMTLAMLCGTRLLPD
LSGHVVMIEEVAEHHYAVDRLLFHVTSCLADAGIAGLRLGRVSDVPENDRPFGCSVEEMA
RHWCHRAGIAFLGTADIGHDVDNRIVPFGLA
00000000000000000000000000000000000000000000000000000000000000000000000000000000000
00000000000000000000000000000000000000000000000000000000000000000000000000110000000
00000000000000000000000000000000000000000000000000000000000000000000000000000000000
000000000000000000000011
>Q2S2F8_3ddl_0_272
MLQELPTLTPGQYSLVFNMFSFTVATMTASFVFFVLARNNVAPKYRISMMVSALVVFIAGYH
YFRITSSWEAAYALQNGMYQPTGELFNDAYRYVDWLLTVPLLTVELVLVMGLPKNERGPLA
AKLGFLAALMIVLGYPGEVSENAALFGTRGLWGFLSTIPFVWILYILFTQLGDTIQRQSSRVS
TLLGNARLLLLATWGFYPIAYMIPMAFPEAFPSNTPGTIVALQVGYTIADVLAKAGYGVLIY
NIAKAKSEEEGFNVSEMVEPATASA
11100000000000000000000000000000000000000000000000000000000000000000000000000000000
00000000000000000000000000000000000000000000000000000000000000000000000000000000000
00000000000000000000000000000000000000000000001111100000000000000000000000000000000
000000000000001111111111
>Q47KX2_2qe6_0_272
MSDEQHDSVWPPPGLDFSKPTIARAYDALLGGKDNFEADRALADYACKHIPGLKESAIEN
RKVLVRGVRFLAGEAGISQFLDLGSGLPTVQNTHEVAQSVNPDARVVYVDIDPMVLTHGR
ALLAKDPNTAVFTADVRDPEYILNHPDVRRMIDFSRPAAIMLVGMLHYLSPDVVDRVVGA
YRDALAPGSYLFMTSLVDTGLPAQQKLARITRENLGEGWARTPEEIERQFGDFELVEPGVVY
TALWRPDEPVDPDNLSPGEQLGMAGIGRKKA
11111110000000000000000000000000000000000000000000000000000000000000000000000000000

00000000000000000000000000000000000000000000000000000000000000000000000000000000000
00000000000000000000000000000000000000000000000000000000000000000000000000000000000
000000000000000000000000
>Q6ZZJ1_3dfk_0_272
MPHDPGATRLLAISPHLDDAVLSFGAGLAQAAQDGANVLVYTVFAGAAQPPYSPAAQRM
HTIWGLAPDDDAVLYRRKEDIAALDHLRVAHRHGRFLDSIYRKLPDGRWLTAHVEGRQKL
AVNDHSPDSDHDLVGEVADDIRSIIDEFDPTLVVTCAAIGEHPDHEATRDAALFATHEKNV
PVRLWEDLPYAVFKSGAVELPQGFRLGSADVSSVKPEMRSQKFQAVERYSSQMVLLNGSEN
NLFDRLDEHARQNAPHGGYGETTWPVVRSDDS
11111110000000000000000000000000000000000000000000000000000000000000000000000000000
00000000000000000000000000000000000000000000000000000000000000000000000000000000000
00000000000000000000000000000000000000000000000000000000000000000000000000000000000
000000000000000000000000
>Q83WS2_1wxc_0_272
MTVRKNQATLTADEKRRFVAAVLELKRSGRYDEFVRTHNEFIMSDTDSGERTGHRSPSFLP
WHRRFLLDFEQALQSVDSSVTLPYWDWSADRTVRASLWAPDFLGGTGRSTDGRVMDGPF
AAFTGNWPINVRVDSRTYLRRSLGGSVAELPTRAEVESVLAISAYDLPPYNSASEGFRNHLE
GWRGVNLHNRVHVWVGGQMATGVSPNDPVFWLHHAYVDKLWAEWQRRHPDSAYVPT
GGTPDVVDLNETMKPWNTVRPADLLDHTAYYTFDA
10000000000000000000000000000000000000000000000000000000000000000000000000000000000
00000000000000000000000000000000000000000000000000000000000000000000000000000000000
00000000000000000000000000000000000000000000000000000000000000000000000000000000000
000000000000000000000000
>D1BLB4_4hes_0_271
MEKLIVGKSLEHQLDTVIKELAPAGNISYAVLQFDDEEEPTLIAARGENTVHSSASLIKVLIM
EYVFHLARTEQLDINDTVPLSRTPRVEGGGALQELVGKHSFTYLELCRLMMVLSDNIATNL
LITVLGMENINARAEKLGVDEMELNRMMMDFNALAEGRDNHITAMSLARLYKHIFECRD
RDVYGREMWNILGRQQFRDILPFYWGEGIRFHHKTGSLDRVEHDGGVIETFRGHFCFILLM
SDIDNDRGKELGAQVGRIMKEFVEEALP
11110000000000000000000000000000000000000000000000000000000000000000000000000000000
00000000000000000000000000000000000000000000000000000000000000000000000000000000000
00000000000000000000000000000000000000000000000000000000000000000000000000000000000
00000000000000000000000
>O93646_3bm3_0_271
MVRNLVIDITKKPTQNIPPTNEIIEEAITELNVDELLDRLFEKDESGEVITPSRIAKMLEEKAFE
IYKEYEKQVREAYLSAGYSREKLEQSFQQARFSRGGKAFEIIFTKLLNKFGIRYEHDRVIKIYD
YITEGEKPDFIIPSVRAFLNDPSSAILITVKRKVRERWREAVGEAQILRNKFGDEINFWFVGFD
EEFTIYSAIAMLDNGIDRVYVIDGRYDSLIEEIKRISDPNFNEDKYIQKIRRFSDIFDDIIQFLNK
HGNKKRGKQLTLV
10000000000000000000000000000000000000000000000000000000000000000000000000000000000
00000000000000000000000000000000000000000000000000000000000000000000000000000000000
00000000000000000000000000000000000000000000000000000000000000000000000000000000000
00000000000111111111111
>Q2YQ74_3py5_0_271
MTSPRNCLRFTLLGCGSSPGVPRINGDWGKCDPKNPKNRRRRASLLVERYDAEGNNTVVV
IDTGPDFRMQMIDSGVHMLDAAVYTHPHADHIHGIDDLRTYVVDNGRLMDVYANRLTR
NRLYDTFGYCFETPVGSSYPPILSMHDIAPETPFSIEGAGGAIRFEPFSQVHGDIESLGFRIGSV
VYCTDVSAFPEQSLQYIKDADVLIIGALQYRPHPSHFSLGEALEWIEKLSPKRAILTHMHVPL
DYETVMRETPHHVEPGYDGLRFEVAV
11000000000000000000000000000000000000000000000000000000000000000000000000000000000
00000000000000000000000000000000000000000000000000000000000000000000000000000000000

00000000000000000000000000000000000000000000000000000000000000000000000000000000000
00000000000000000000000
>Q5SI12_3a3u_0_271
MEALRLGFSPCPNDTFIFYALVHGRVESPVPLEPVLEDVETLNRWALEGRLPLTKLSYAAYA
QVRDRYVALRSGGALGRGVGPLVVARGPLQALEGLRVAVPGRHTTAYFLLSLYAQGFVPVE
VRYDRILPMVAQGEVEAGLIIHESRFTYPRYGLVQVVDLGAWWEERTGLPLPLGAILARRDL
GEGLIRALDEAVRRSVAYALAHPEEALDYMRAHAQELSDEVIWAHVHTYVNAFSLDVGEE
GERAVARLFAEAEARGLAAPSPRPLFV
11000000000000000000000000000000000000000000000000000000000000000000000000000000000
00000000000000000000000000000000000000000000000000000000000000000000000000000000000
00000000000000000000000000000000000000000000000000000000000000000000000000000000000
00000000000000000000000
>Q81A57_2qm0_0_271
MNTTVEKQQIITSNTEQWKMYSKLEGKEYQIHISKPKQPAPDSGYPVIYVLDGNAFFQTFHE
AVKIQSVRAEKTGVSPAIIVGVGYPIEGAFSGEERCYDFTPSVISKDAPLKPDGKPWPKTGGA
HNFFTFIEEELKPQIEKNFEIDKGKQTLFGHSLGGLFALHILFTNLNAFQNYFISSPSIWWNN
KSVLEKEENLIIELNNAKFETGVFLTVGSLEREHMVVGANELSERLLQVNHDKLKFKFYEAE
GENHASVVPTSLSKGLRFISYV
10000000000000000000000000000000000000000000000000000000000000000000000000000000000
00000000000000000000000000000000000000000000000000000000000000000000000000000000000
00000000000000000000000000000000000000000000000000000000000000000000000000000000000
00000000000000000000000
>Q8GZD5_1umz_22_293
AALRKPVDVAFGRNYVPTWAFDHIKYFNGGNEIQLHLDKYTGTGFQSKGSYLFGHFSMQ
MKLVPGDSAGTVTAFYLSSQNSEHDEIDFEFLGNRTGQPYILQTNVFTGGKGDREQRIYLWF
DPTKEFHYYSVLWNMYMIVFLVDDVPIRVFKNCKDLGVKFPFNQPMKIYSSLWNADDWAT
RGGLEKTDWSKAPFIASYRSFHIDGCEASVEAKFCATQGARWWDQKEFQDLDAFQYRRLS
WVRQKYTIYNYCTDRSRYPSMPPECKRDRDI
11111000000000000000000000000000000000000000000000000000000000000000000000000000000
00000000000000000000000000000000000000000000000000000000000000000000000000000000000
00000000000000000000000000000000000000000000000000000000000000000000000000000000000
00000000000000000000000
>Q9HC38_3zi1_0_286
MAARRALHFVFKVGNRFQTARFYRDVLGMKVESCSVARLECSGAFSKPYDGKWSKTMVG
FGPEDDHFVAELTYNYGVGDYKLGNDFMGITLASSQAVSNARKLEWPLTEVAEGVFETEAP
GGYKFYLQNRSLPQSDPVLKVTLAVSDLQKSLNYWCNLLGMKIYEKDEEKQRALLGYADN
QCKLELQGVKGGVDHAAAFGRIAFSCPQKELPDLEDLMKRENQKILTPLVSLDTPGKATV
QVVILADPDGHEICFVGDEAFRELSKMDPEGS
00000000000000000000000000000000000000111111111000000000000000000000000000000000000
00000000000000000000000000000000000000000000000000000000000000000000000000000000000
00000000000000000000000000000000000000000000000000000000000000000000000000000000000
00000000000000000000000
>B0VD92_4gki_0_270
MSHIQRETSCSRPRLNSNLDADLYGYRWARDNVGQSGATIYRLYGKPNAPELFLKHGKGSV
ANDVTDEMVRLNWLTAFMPLPTIKHFIRTPDDAWLLTTAIPGKTAFQVLEEYPDSGENIVD
ALAVFLRRLHSIPVCNCPFNSDRVFRLAQAQSRMNNGLVDASDFDDERNGWPVEQVWKE
MHKLLPFSPDSVVTHGDFSLDNLIFDEGKLIGCIDVGRVGIADRYQDLAILWNCLGEFSPSL
QKRLFQKYGIDNPDMNKLQFHLMLDEFF
11000000000000000000000000000000000000000000000000000000000000000000000000000000000
00000000000000000000000000000000000000000000000000000000000000000000000000000000000
00000000000000000000000000000000000000000000000000000000000000000000000000000000000

0000000000000000000000
>D2J2T6_4g9g_0_270
MTINYHELETSHGRIAVRESEGEGAPLLMIHGNSSSGAIFAPQLEGEIGKKWRVIAPDLPGH
GKSTDAIDPDRSYSMEGYADAMTEVMQQLGIADAVVFGWSLGGHIGIEMIARYPEMRGLM
ITGTPPVAREEVGQGFKSGPDMALAGQEIFSERDVESYARSTCGEPFEASLLDIVARTDGRAR
RIMFEKFGSGTGGNQRDIVAEAQLPIAVVNGRDEPFVELDFVSKVKFGNLWEGKTHVIDNA
GHAPFREAPAEFDAYLARFIRDCTQ
10000000000000000000000000000000000000000000000000000000000000000000000000000000000
00000000000000000000000000000000000000000000000000000000000000000000000000000000000
00000000000000000000000000000000000000000000000000000000000000000000000000000000000
0000000000000000000000
>P04067_1edt_42_312
APAPVKQGPTSVAYVEVNNNSMLNVGKYTLADGGGNAFDVAVIFAANINYDTGTKTAYL
HFNENVQRVLDNAVTQIRPLQQQGIKVLLSVLGNHQGAGFANFPSQQAASAFAKQLSDA
VAKYGLDGVDFDDEYAEYGNNGTAQPNDSSFVHLVTALRANMPDKIISLYNIGPAASRLSY
GGVDVSDKFDYAWNPYYGTWQVPGIALPKAQLSPAAVEIGRTSRSTVADLARRTVDEGYG
VYLTYNLDGGDRTADVSAFTRELYGSEAVRTP
11111000000000000000000000000000000000000000000000000000000000000000000000000000000
00000000000000000000000000000000000000000000000000000000000000000000000000000000000
00000000000000000000000000000000000000000000000000000000000000000000000000000000000
0000000000000000000001
>P05820_2xmx_0_270
METLTVHAPSPSTNLPSYGNGAFSLSAPHVPGAGPLLVQVVYSFFQSPNMCLQALTQLEDYI
KKHGASNPLTLQIISTNIGYFCNADRNLVLHPGISVYDAYHFAKPAPSQYDYRSMNMKQMS
GNVTTPIVALAHYLWGNGAERSVNIANIGLKISPMKINQIKDIIKSGVVGTFPVSTKFTHATG
DYNVITGAYLGNITLKTEGTLTISANGSWTYNGVVRSYDDKYDFNASTHRGIIGESLTRLGA
MFSGKEYQILLPGEIHIKESGKR
10000000000000000000000000000000000000000000000000000000000000000000000000000000000
00000000000000000000000000000000000000000000000000000000000000000000000000000000000
00000000000000000000000000000000000000000000000000000000000000000000000000000000000
0000000000000000000000
>P44774_1npy_0_270
MINKDTQLCMSLSGRPSNFGTTFHNYLYDKLGLNFIYKAFTTQDIEHAIKGVRALGIRGCAV
SMPFKETCMPFLDEIHPSAQAIESVNTIVNDNGFLRAYNTDYIAIVKLIEKYHLNKNAKVIV
HGSGGMAKAVVAAFKNSGFEKLKIYARNVKTGQYLAALYGYAYINSLENQQADILVNVTSI
GMKGGKEEMDLAFPKAFIDNASVAFDVVAMPVETPFIRYAQARGKQTISGAAVIVLQAVEQ
FELYTHQRPSDELIAEAAAFARTKF
00000000000000000000000000000000000000000000000000000000000000000000000000000000000
00000000000000000000000000000000000000000000000000000000000000000000000000000000000
00000000000000000000000000000000000000000000000000000000000000000000000000000000000
0000000000000000000001
>P73832_3en0_0_270
MPLSSQPAILIIGGAEDKVHGREILQTFWSRSGGNDAIIGIIPSASREPLLIGERYQTIFSDMGV
KELKVLDIRDRAQGDDSGYRLFVEQCTGIFMTGGDQLRLCGLLADTPLMDRIRQRVHNGEI
SLAGTSAGAAVMGHHMIAGGSSGEWPNRALVDMAVGLGIVPEIVVDQHFHNRNRMARL
LSAISTHPELLGLGIDEDTCAMFERDGSVKVIGQGTVSFVDARDMSYTNAALVGANAPLSL
HNLRLNILVHGEVYHQVKQRAFPRVT
11100000000000000000000000000000000000000000000000000000000000000000000000000000000
00000000000000000000000000000000000000000000000000000000000000000000000000000000000
00000000000000000000000000000000000000000000000000000000000000000000000000000000000
0000000000000000000011

>Q48HR6_3ocr_0_270
MSNVSALPLQPGTTPSGGGSVRDRVSPQEWEVRVKLAAAYRLAALKRWTDHIYTHFSARV
PGPDEHFLINAFGLLFDEITASNLVKVDIDGTIVDDPTGLGINYAGYVIHSAIHAARHDLQA
VLHTHTRDGIAVSAQKDGLLPISQHSIAFSGRVAYHGYEGIALDLSERERLVADLGDKSVMIL
RNHGLLTGGVSVEHAIQQLHALEYACNIQIAAQSAGNAELVFPPREVIAKVEEQAKAIKDG
NGPGVARHWNALIRELERSGTDYRD
11111111111111111100000000000000000000000000000000000000000000000000000000000000000
00000000000000000000000000000000000000000000000000000000000000000000000000000000000
00000000000000000000000000000000000000000000000000000000000000000000000000000110000
0000000000000000000000
>Q5ZXY5_3lul_0_270
MPTRVIEDKGDMTPSFGIDDRIFLGEGLFETIRVNSSKPSFAYMHWERLGNSARQLGIPFEISF
DDWFEHLIQKIQKDNLYHGGIKAILSGGPASRGLAERGQVSQLIFQTFNYSIQKHPVRLISIN
WLRDKANPLYQLKSVNYLEAIIAQRQAIAVGADDALFFNTENHVTETTCANLFLIENNILY
TPRVEDGILPGITRARLISHCQQHKMSVQEISLTKKRIEDADAVFLTNSLQGIRRVLSLDNIIFE
VNHPIIDKLIFLLNQDES
00000000011000000000000000000000000000000000000000000000000000000000000000000000000
00000000000000000000000000000000000000000000000000000000000000000000000000000000000
00000000000000000000000000000000000000000000000000000000000000000000000000000000000
0000000000000000000000
>Q96EP1_2xp0_393_663
VRRSFSDEEGSSEDLLELSDVDSESSDISQPYVVCRQCPEYRRQAAQPPHCPAPEGEPGAPQ
ALGDAPSTSVSLTTAVQDYVCPLQGSHALCTCCFQPMPDRRAEREQDPRVAPQQCAVCLQ
PFCHLYWGCTRTGCYGCLAPFCELNLGDKCLDGVLNNNSYESDILKNYLATRGLTWKNM
LTESLVALQRGVFLLSDYRVTGDTVLCYCCGLRSFRELTYQYRQNIPASELPVAVTSRPDCYW
GRNCRTQVKAHHAMKFNHICEQTRFKN
11111111111111111111111111111100000000000000000000001111111111111111111111111110000
00000000000000000000000000000000000000000000000000000000000000000000000000000000000
00000000000000000000000000000000000000000000000000000000000000000000000000000000000
0000000000000000000001
>Q9PGZ0_3ois_0_270
MQTVLKRRKKSGYGYIPDIADIRDFSYTPEKSVIAALPPKVDLTPPFQVYDQGRIGSCTANAL
AAAIQFERIHDKQSPEFIPSRLFIYYNERKIEGHVNYDSGAMIRDGIKVLHKLGVCPEKEWPY
GDTPADPRTEEFPPGAPASKKPSDQCYKDAQNYKITEYSRVAQDIDHLKACLAVGSPFVFGF
SVYNSWVGNNSLPVRIPLPTKNDTLEGGHAVLCVGYDDEIRHFRIRNSWGNNVGEDGYF
WMPYEYISNTQLADDFWVIKTVRK
11000000000000000000000000000000000000000000000000000000000000000000000000000000000
00000000000000000000000000000000000000000000000000000000000000000000000000000000000
00000000000000000000000000000000000000000000000000000000000000000000000000000000000
0000000000000000000001
>A7V1G8_4i8i_22_291
NGCHANNDTIKVLAIGNSFSQDAVEQYLHELGEAEGITMIIGNMFIGGCSLERHVQNIRNN
APAYAYRKVEKDGEKTETRSMTIEKALADEKWDYISVQQASPLSGIYDSYKASLPELVNYIRE
RIGKETVLMMHQTWAYATNANHTGFKNYDQNQMKMYTSIVDAVKKAANLVGIKKIIPSG
TAIQNARTSFIGDHMNRDGYHLDLTIGRYTAACTWFEALTHRNVTENPYSPEGIDPIHKKA
AQMAAHNAILYPDKVTELTELKKIAD
11111110000000000000000000000000000000000000000000000000000000000000000000000000000
00000000000000000000000000000000000000000000000000000000000000000000000000000000000
00000000000000000000000000000000000000000000000000000000000000000000000000000000000
000000000000000001111
>C7C422_4eyf_0_269

MELPNIMHPVAKLSTALAAALMLSGCMPGEIRPTIGQQMETGDQRFGDLVFRQLAPNVW
QHTSYLDMPGFGAVASNGLIVRDGGRVLVVDTAWTDDQTAQILNWIKQEINLPVALAVVT
HAHQDKMGGMDALHAAGIATYANALSNQLAPQEGMVAAQHSLTFAANGWVEPATAPN
FGPLKVFYPGPGHTSDNITVGIDGTDIAFGGCLIKDSKAKSLGNLGDADTEHYAASARAFG
AAFPKASMIVMSHSAPDSRAAITHTARMADKLR
11111111111111111111111111111000000000000000000000000000000000000000000000000000000
00000000000000000000000000000000000000000000000000000000000000000000000000000000000
00000000000000000000000000000000000000000000000000000000000000000000000000000000000
000000000000000000000
>P24289_1ak0_0_269
WGALGHATVAYVAQHYVSPEAASWAQGILGSSSSSYLASIASWADEYRLTSAGKWSASLHF
IDAEDNPPTNCNVDYERDCGSSGCSISAIANYTQRVSDSSLSSENHAEALRFLVHFIGDMTQ
PLHDEAYAVGGNKINVTFDGYHDNLHSDWDTYMPQKLIGGHALSDAESWAKTLVQNIES
GNYTAQAIGWIKGDNISEPITTATRWASDANALVCTVVMPHGAAALQTGDLYPTYYDSVID
TIELQIAKGGYRLANWINEIHGSEIAK
00000000000000000000000000000000000000000000000000000000000000000000000000000000000
00000000000000000000000000000000000000000000000000000000000000000000000000000000000
00000000000000000000000000000000000000000000000000000000000000000000000000000000000
000000000000000111111
>Q5M586_3b1v_0_269
MTEIALIGNPNSGKTSLFNLITGHNQRVGNWPGVTVERKSGLVKKNKDLEIQDLPGIYSMS
PYSPEEKVARDYLLSQRADSILNVVDATNLERNLYLTTQLIETGIPVTIALNMIDVLDGQGKK
INVDKLSYHLGVPVVATSALKQTGVDQVVKKAAHTTTSTVGDLAFPIYDDRLEAAISQILEV
LGNSVPQRSARFYAIKLFEQDSLVEAELDLSQFQRKEIEDIIRITEEIFTEDAESIVINERYAFIER
VCQMAESHTEDFALTLS
00000000000000000000000000000000000000000000000000000000000000000000000000000000000
00000000000000000000000000000000000000000000000000000000000000000000000000000000000
00000000000000000000000000000000000000000000000000000000000000000000000000000000000
000000000000000111111
>Q5SK18_3aam_0_269
MPRYGFHLSIAGKKGVAGAVEEATALGLTAFQIFAKSPRSWRPRALSPAEVEAFRALREASG
GLPAVIHASYLVNLGAEGELWEKSVASLADDLEKAALLGVEYVVVHPGSGRPERVKEGALK
ALRLAGVRSRPVLLVENTAGGGEKVGARFEELAWLVADTPLQVCLDTCHAYAAGYDVAED
PLGVLDALDRAVGLERVPVVHLNDSVGGLGSRVDHHAHLLQGKIGEGLKRVFLDPRLKDR
VFILETPRGPEEDAWNLRVFRAWLEEA
10000000000000000000000000000000000000000000000000000000000000000000000000000000000
00000000000000000000000000000000000000000000000000000000000000000000000000000000000
00000000000000000000000000000000000000000000000000000000000000000000000000000000000
000000000000000000000
>Q9H008_2x4d_0_269
MAPWGKRLAGVRGVLLDISGVLYDSGAGGGTAIAGSVEAVARLKRSRLKVRFCTNESQKSR
AELVGQLQRLGFDISEQEVTAPAPAACQILKEQGLRPYLLIHDGVRSEFDQIDTSNPNCVVIA
DAGESFSYQNMNNAFQVLMELEKPVLISLGKGRYYKETSGLMLDVGPYMKALEYACGIKA
EVVGKPSPEFFKSALQAIGVEAHQAVMIGDDIVGDVGGAQRCGMRALQVRTGKFRPSDEH
HPEVKADGYVDNLAEAVDLLLQHADK
00000000000000000000000000000000000000000000000000000000000000000000000000000000000
00000000000000000000000000000000000000000000000000000000000000000000000000000000000
00000000000000000000000000000000000000000000000000000000000000000000000000000000000
000000000000000000001
>Q9RP27_1jov_0_269
MKTTLLKTLTPELHLVQHNDIPVLHLKHAVGTAKISLQGAQLISWKPQNAKQDVLWLSEV

EPFKNGNAIRGGVPICYPWFGGVKQPAHGTARIRLWQLSHYYISVHKVRLEFELFSDLNIIE
AKVSMVFTDKCHLTFTHYGEESAQAALHTYFNIGDINQVEVQGLPETCFNSLNQQQENVP
SPRHISENVDCIYSAENMQNQILDKSFNRTIALHHHNASQFVLWNPWHKKTSGMSETGYQ
KMLCLETARIHHLLEFGESLSVEISLKG
00000000000000000000000000000000000000000000000000000000000000000000000000000000000
00000000000000000000000000000000000000000000000000000000000000000000000000000000000
00000000000000000000000000000000000000000000000000000000000000000000000000000000000
000000000000000000001
>B9JYM4_3llc_0_268
MTNVGRPIETHAITVGQGSDARSIAALVRAPAQDERPTCIWLGGYRSDMTGTKALEMDDL
AASLGVGAIRFDYSGHGASGGAFRDGTISRWLEEALAVLDHFKPEKAILVGSSMGGWIALR
LIQELKARHDNPTQVSGMVLIAPAPDFTSDLIEPLLGDRERAELAENGYFEEVSEYSPEPNIF
TRALMEDGRANRVMAGMIDTGCPVHILQGMADPDVPYQHALKLVEHLPADDVVLTLVR
DGDHRLSRPQDIDRMRNAIRAMIEPRP
11111000000000000000000000000000000000000000000000000000000000000000000000000000000
00000000000000000000000000000000000000000000000000000000000000000000000000000000000
00000000000000000000000000000000000000000000000000000000000000000000000000000000000
00000000000000000111
>Q12395_3bq3_0_268
MSNNKIKRKDASPEQEAIESFTSLTKCDPKVSRKYLQRNHWNINYALNDYYDKEIGTFTDE
VSTVAHPPVYPKELTQVFEHYINNNLFDIDSLVKFIEELGYNLEDLATLCLAHLLGYKKLEEP
LKREDFLSTWFMQGCSTISDMQECIKTLDVKLHEDLQYFTQIYNYAFNLILDPNRKDIDTDE
GIQYWKLFFQPEYPVRMEPDLLEAWFRFLRDEGKTTISKDTWRMLLLFFKRYPTIQKIISDYD
ETAAWPFIIDEFYECLQDQQ
11111111111000000000000000000000000000000000000000000001111111100000000000000000000
00000000000000000000000000000000000000000000000000000000000000000000000000000000000
00000000000000000000000000000000000000000000000000000000000000000000000000000000000
00000000000000000000
>Q5LA60_3iee_17_285
ASCSGGDKSKAPVVSTADIENAAEVIKYYNTSLGVLKDMVKEKDVNAVLDYMEQKGKTPA
LSAIVPPAVVSKDSAIVLNPGNCFNEETRRNLKQNYTGLFQARTEFYANFDTYLSYLKKKDV
TNAKKLLDVNYQLSTQMSEYKQNIFDILSPFTEQAELVLLVDNPLKAQIMSVRKMSSTMQSI
LNLYARKHRMDGPRIDLKVAELTKQLDAAKKLPVVNGHEGEMKSYQAFLSQVETFIKQVK
KVREKGEYSDADYDMLTSAFETSII
11111111111110000000000000000000000000000000000000000000000000000000000000000000000
00000000000000000000000000000000000000000000000000000000000000000000000000000000000
00000000000000000000000000000000000000000000000000000000000000000000000000000000000
00000000000000000000
>Q5SH27_3u6u_0_268
MIVVKVGGAEGINYEAVAKDAASLWKEGVKLLLVHGGSAETNKVAEALGHPPRFLTHPGG
QVSRLTDRKTLEIFEMVYCGLVNKRLVELLQKEGANAIGLSGLDGRLFVGRRKTAVKYVEN
GKVKVHRGDYTGTVEEVNKALLDLLLQAGYLPVLTPPALSYENEAINTDGDQIAALLATLY
GAEALVYLSNVPGLLARYPDEASLVREIPVERIEDPEYLALAQGRMKRKVMGAVEAVKGGV
KRVVFADGRVENPIRRALSGEGTVVR
00000000000000000000000000000000000000000000000000000000000000000000000000000000000
00000000000000000000000000000000000011100000000000000000000000000000000000000000000
00000000000000000000000000000000000000000000000000000000000000000000000000000000000
00000000000000000000
>Q8ZK62_2qjv_0_268
MANLLSTCTSESGNIQHISPQNAGWEYVGFDVWQLKAGESITLPSDERERCLVLVAGLASV
KAADSFFYRIGQRMSPFERIPAYSVYLPHHTEAKVTAETDLELAVCSAPGFGELPVRLISPQE

VGVEHRGKGRNQRLVHNILPDSQLADSLLVVEVYTNEGDTSSWPAHKHDTAVEGQETYLE
ETYYHRFNPPQGFCLQRVYTDDRSLDECMAVYNRDVVKVPKGYHPVATIAGYDNYYLNV
MAGPLRKWRFTWEENHAWINSPDYPR
10000000000000000000000000000000000000000000000000000000000000000000000000000000000
00000000000000000000000000000000000000000000000000000000000000000000000000000000000
00000000000000000000000000000000000000000000000000000000000000000000000000000000000
00000000000000011111
>Q9DBL7_2f6r_294_562
MAVNRFRLENGKEELALYQIQLLKDQSHNENEEDKVSSSSFRQRILGNLLQPPNERPELPSG
LYVLGLTGISGSGKSSVAQRLKNLGAYIIDSDHLGHRAYAPGGPAYQPVVEAFGTDILHKDG
TINRKVLGSRVFGNKKQMKILTDIVWPVIAKLAREEMDVAVAKGKTLCVIDAAMLLEAGW
QSMVHEVWTVVIPETEAVRRIVERDGLSEAAAQSRLQSQMSGQQLVEQSNVVLSTLWESH
VTQSQVEKAWNLLQKRLPKAYQTRN
11111111111111100000000001111111111111111100000000000000000000000000000000000000000
00000000000000000000000000000000000000000000000000000000000000000000000000000000000
00000000000000000000000000000000000000000000000000000000000000000000000000000000000
00000000000001111111
>Q9NQ88_3dcy_1_269
ARFALTVVRHGETRFNKEKIIQGQGVDEPLSETGFKQAAAAGIFLNNVKFTHAFSSDLMRT
KQTMHGILERSKFCKDMTVKYDSRLRERKYGVVEGKALSELRAMAKAAREECPVFTPPGG
ETLDQVKMRGIDFFEFLCQLILKEADQKEQFSQGSPSNCLETSLAEIFPLGKNHSSKVNSDSG
IPGLAASVLVVSHGAYMRSLFDYFLTDLKCSLPATLSRSELMSVTPNTGMSLFIINFEEGREVK
PTVQCICMNLQDHLNGLTETR
00000000000000000000000000000000000000000000000000000000000000000000000000000000000
00000000000000000000000000000000000000000000000000000000000000000011111111000000000
00000111111111111100000000000000000000000000000000000000000000000000000000000000000
00000000000000111111
>P10478_1jt2_19_286
LVTISSTSAASLPTMPPSGYDQVRNGVPRGQVVNISYFSTATNSTRPARVYLPPGYSKDKKYS
VLYLLHGIGGSENDWFEGGGRANVIADNLIAEGKIKPLIIVTPNTNAAGPGIADGYENFTK
DLLNSLIPYIESNYSVYTDREHRAIAGLSMGGGQSFNIGLTNLDKFAYIGPISAAPNTYPNER
LFPDGGKAAREKLKLLFIACGTNDSLIGFGQRVHEYCVANNINHVYWLIQGGGHDFNVW
KPGLWNFLQMADEAGLTRDGNT
11111111110000000000000000000000000000000000000000000000000000000000000000000000000
00000000000000000000000000000000000000000000000000000000000000000000000000000000000
00000000000000000000000000000000000000000000000000000000000000000000000000000000000
0000000000000000111
>P15636_1arb_205_472
GVSGSCNIDVVCPEGDGRRDIIRAVGAYSKSGTLACTGSLVNNTANDRKMYFLTAHHCGM
GTASTAASIVVYWNYQNSTCRAPNTPASGANGDGSMSQTQSGSTVKATYATSDFTLLELNN
AANPAFNLFWAGWDRRDQNYPGAIAIHHPNVAEKRISNSTSPTSFVAWGGGAGTTHLNV
QWQPSGGVTEPGSSGSPIYSPEKRVLGQLHGGPSSCSATGTNRSDQYGRVFTSWTGGGAAA
SRLSDWLDPASTGAQFIDGLDSGGGTP
00000000000000000000000000000000000000000000000000000000000000000000000000000000000
00000000000000000000000000000000000000000000000000000000000000000000000000000000000
00000000000000000000000000000000000000000000000000000000000000000000000000000000000
0000000000000011111
>Q04MW8_4i62_0_267
MNKMKKVLMTMFGLVMLPLLFACSNNQSAGIEAIKSKGKLVVALNPDFAPFEYQKVVDG
KNQIVGSDIELAKAIATELGVELELSPMSFDNVLASVQSGKADLAISGVSKTDERSKVFDFST
PYYTAKNKLIVKKSDLATYQSVNDLAQKKVGAQKGSIQETMAKDLLQNSSLVSLPKNGNLI

TDLKSGQVDAVIFEEPVAKGFVENNPDLAIADLNFEKEQDDSYAVAMKKDSKELKEAVDKT
IQKLKESGELDKLIEDAFKASIEK
11111111111111111111111111111100000000000000000000000000000000000000000000000000000
00000000000000000000000000000000000000000000000000000000000000000000000000000000000
00000000000000000000000000000000000000000000000000000010000000000000000000000000000
0000000000000000000
>Q15691_2r8u_0_267
MAVNVYSTSVTSDNLSRHDMLAWINESLQLNLTKIEQLCSGAAYCQFMDMLFPGSIALKK
VKFQAKLEHEYIQNFKILQAGFKRMGVDKIIPVDKLVKGKFQDNFEFVQWFKKFFDANYD
GKDYDPVAARQGQETAVAPSLVAPALNKPKKPLTSSSAAPQRPISTQRTAAAPKAGPGVVR
KNPGVGNGDDEAAELMQQVNVLKLTVEDLEKERDFYFGKLRNIELICQENEGENDPVLQ
RIVDILYATDEGFVIPDEGGPQEEQEEY
00000000000000000000000000000000000000000000000000000000000000000000000000000000000
00000000000000000000000000000000000000000000000001111111111111111111111111111111111
11111111111111111111000011111111111111111111111111111111111111111111111111111111111
111111111111111111
>Q5SLE3_3ab8_0_267
MRILLATDGSPQARGAEALAEWLAYKLSAPLTVLFVVDTRLARIPELLDFGALTVPVPVLRT
ELERALALRGEAVLERVRQSALAAGVAVEAVLEEGVPHEAILRRARAADLLVLGRSGEAHG
DGFGGLGSTADRVLRASPVPVLLAPGEPVELEGALLGYDASESAVRALHALAPLARALGLG
VRVVSVHEDPARAEAWALEAEAYLRDHGVEASALVLGGDAADHLLRLQGPGDLLALGAP
VRRLVFGSTAERVIRNAQGPVLTAR
00000000000000000000000000000000000000000000000011111100000000000000000000000000000
00000000000000000000000000000000000000000000000000000000000000000000000000000000000
00000000000000000000000000000000000000000000000000000000000000000000000000000000000
000000000000000000
>A4HFB4_3css_0_266
MTSFAPTVKICENLSQMSFAAREVILAAIDARVDKSVPVVLALSGGSTPKRLYEELHEKDLA
LLQQHAVQFILGDERLLSEDDEQSNFSMATKALLRDVPSSDVISIDRRAALATSKDEKGGLD
GAWAVAQDYEAKLLNCLPCKQINGTAKSVPVVDIVLLGFGSDGHTASIFPDSVAATDEEHV
VSVSFPSPTMSPKVWRVTLSKTVIQHAKHVVVLAAGKDKNWVVRGVLSESPTDPLPVSRFL
RDCRGSVTLLLDPGAGEGVCA
11100000000000000000000000000000000000000000000000000000000000000000000000000000000
00000000000000000000000000000000000000000000000000000000000000000000000000000000000
00000000000000000000000000000000000000000000000000000000000000000000000000000000000
00000000000000000
>P03962_3gdr_0_266
MSKATYKERAATHPSPVAAKLFNIMHEKQTNLCASLDVRTTKELLELVEALGPKICLLKTH
VDILTDFSMEGTVKPLKALSAKYNFLLFEDRKFADIGNTVKLQYSAGVYRIAEWADITNAH
GVVGPGIVSGLKQAAEEVTKEPRGLLMLAELSCKGSLATGEYTKGTVDIAKSDKDFVIGFIA
QRDMGGRDEGYDWLIMTPGVGLDDKGDALGQQYRTVDDVVSTGSDIIIVGRGLFAKGRD
AKVEGERYRKAGWEAYLRRCGQQN
11000000000000000000000000000000000000000000000000000000000000000000000000000000000
00000000000000000000000000000000000000000000000000000000000000000000000000000000000
00000000000000000000000000000000000000000000000000000000000000000000000000000000000
00000000000000001
>P07386_4fbv_0_266
MAAYLVQNQWGGSQATWNPGGLWLIGARDKQNVVALDIKSDDGGKTLKGTMTYNGEG
PIGFRGTLSSANNYTVENQWGGTSAPWQPGGVWVLGARDKQNIVAVSIKSNDGGKTLTGT
TTYNGEGPIGFKSEVTDGDTYSVENQWGGSAAPWHSGGVWVLGTRGKQNVINVDAKSN
DGGKTLSGTMTYNGEGPIGFRGTLTSPDTYTVENQWGGSTAPWNPGGFWMIGARNGQNV

VALNVASSDGGKTLAGTMIYNGEGPIGFRARLG
10000000000000000000000000000000000000000000000000000000000000000000000000000000000
00000000000000000000000000000000000000000000000000000000000000000000000000000000000
00000000000000000000000000000000000000000000000000000000000000000000000000000000000
000000000000000000
>P16154_3ho6_542_808
SEDNGVDFNKNTALDKNYLLNNKIPSNNVEEAGSKNYVHYIIQLQGDDISYEATCNLFSKN
PKNSIIIQRNMNESAKSYFLSDDGESILELNKYRIPERLKNKEKVKVTFIGHGKDEFNTSEFAR
LSVDSLSNEISSFLDTIKLDISPKNVEVNLLGCNMFSYDFNVEETYPGKLLLSIMDKITSTLPD
VNKNSITIGANQYEVRINSEGRKELLAHSGKWINKEEAIMSDLSSKEYIFFDSIDNKLKAKSK
NIPGLASISEDIKTL
11110000000000000000000000001111100000000000000000000000000000000000000000000000000
00000000000000000000000000000000000000000000000000000000000000000000000000000000000
00000000000000000000000000000000000000000000000000000000000000000000000000000000000
000000000000111111
>P18708_1nsf_477_743
GDFLASLENDIKPAFGTNQEDYASYIMNGIIKWGDPVTRVLDDGELLVQQTKNSDRTPLVS
VLLEGPPHSGKTALAAKIAEESNFPFIKICSPDKMIGFSETAKCQAMKKIFDDAYKSQLSCVV
VDDIERLLDYVPIGPRFSNLVLQALLVLLKKAPPQGRKLLIIGTTSRKDVLQEMEMLNAFSTT
IHVPNIATGEQLLEALELLGNFKDKERTTIAQQVKGKKVWIGIKKLLMLIEMSLQMDPEYR
VRKFLALLREEGASPLDFD
11111111111000000000000000000000000000000000000000000000000000000000000000000000000
00000000000000000000000000000000000000000000000000000000000000000000000000000000000
00000000000000000000000000000000000000000000000000000000000000000000000000000000000
000000000111111111
>Q47XN7_2v27_0_266
MAKGTKYVSKVPDEHGFIEWSTEENLIWQELFTRQIACIKDKACDEYHEGLAKLNLPTDRI
PQLDEVSKVLKVSTGWECYPVPALIGFGEFFRLLSEKKFPVATFIRSREEMDYLQEPDIFHEIF
GHCPLLTNSSFANYTEAYGKMGLNATKEQRVFLARLYWFTIEFGLLDTPKGLRIYGGGVLSS
PGETDYAMNNTDVDRKPFDILDVLRTPYRIDIMQPIYYMLTKVSDLDEIRKFEVDDIMELVA
QAEALGLHEAKFPVKKAS
11100000000000000000000000000000000000000000000000000000000000000000000000000000000
00000000000000000000000000000000000000000000000000000000000000000000000000000000000
00000000000000000000000000000000000000000000000000000000000000000000000000000000000
000000000000000000
>Q5HH39_2xmz_0_266
MTHYKFYEANVETNQVLVFLHGFLSDSRTYHNHIEKFTDNYHVITIDLPGHGEDQSSMDET
WNFDYITTLLDRILDKYKDKSITLFGYSMGGRVALYYAINGHIPISNLILESTSPGIKEEANQL
ERRLVDDARAKVLDIAGIELFVNDWEKLPLFQSQLELPVEIQHQIRQQRLSQSPHKMAKAL
RDYGTGQMPNLWPRLKEIKVPTLILAGEYDEKFVQIAKKMANLIPNSKCKLISATGHTIHVE
DSDEFDTMILGFLKEEQND
00000000000000000000000000000000000000000000000000000000000000000000000000000000000
00000000000000000000000000000000000000000000000000000000000000000000000000000000000
00000000000000000000000000000000000000000000000000000000000000000000000000000000000
000000000000000001
>Q7WT42_1woq_0_266
MAKKDEKSHKNAPLIGIDIGGTGIKGGIVDLKKGKLLGERFRVPTPQPATPESVAEAVALVV
AELSARPEAPAAGSPVGVTFPGIIQHGVVHSAANVDKSWLNTDIDALLTARLGRPVEVIND
ADAAGLAEARYGAGAGVKGTVLVITLGTGIGSAFIFDGKLVPNAELGHLEIDGHDAETKAS
AVARERDGLSWDEYSVLLQRYFSHVEFLFSPELFIVGGGISKRADEYLPNLRLRTPIVPAVLR
NEAGIVGAAIEIALQHKLAK

11111111110000000000000000000000000000000000000000000000000000000000000000000000000
00000000000000000000000000000000000000000000000000000000000000000000000000000000000
00000000000000000000000000000000000000000000000000000000000000000000000000000000000
00000000000001111
>Q9T0I8_2h8g_0_266
MAPHGDGLSDIEEPEVDAQSEILRPISSVVFVIAMQAEALPLVNKFGLSETTDSPLGKGLPW
VLYHGVHKDLRINVVCPGRDAALGIDSVGTVPASLITFASIQALKPDIIINAGTCGGFKVKG
ANIGDVFLVSDVVFHDRRIPIPMFDLYGVGLRQAFSTPNLLKELNLKIGRLSTGDSLDMSTQ
DETLIIANDATLKDMEGAAVAYVADLLKIPVVFLKAVTDLVDGDKPTAEEFLQNLTVVTAA
LEGTATKVINFINGRNLSDL
11111111111111111111100000000000000000000000000000000000000000000000000000000000000
00000000000000000000000000000000000000000000000000000000000000000000000000000000000
00000000000000000000000000000000000000000000000000000000000000000000000000000000000
000000000000000000
>O77448_2r4g_253_518
KGFQFKVIQEKLQGRQFINSDKIKPDHPQTIIKKTLLKEYQSKNFSCQEERDLFLEFTEKIVQ
NFHNINFNYLLKKFCKLPENYQSLKSQVKQIVQSENKANQQSCENLFNSLYDTEISYKQITN
FLRQIIQNCVPNQLLGKKNFKVFLEKLYEFVQMKRFENQKVLDYICFMDVFDVEWFVDLK
NQKFTQKRKYISDKRKILGDLIVFIINKIVIPVLRYNFYITEKHKEGSQIFYYRKPIWKLVSKLTI
VKLEEENLEKVEEKL
11111000000011111111110000000000000000000000000000000000000000000000000000000000000
00000000000000000000000000000000000000000000000000000000000000000000000000000000000
00000000000000000000000000000000000000000000000000000000000000000000000000000000000
00000000000000000
>O81226_2faw_22_287
RPSSRVYIVEVLNEFPHDPYAFTQGLVYAENDTLFESTGLYGRSSVRQVALQTGKVENIHKM
DDSYFGEGLTLLNEKLYQVVWLKNIGFIYDRRTLSNIKNFTHQMKDGWGLATDGKILYGSD
GTSILYEIDPHTFKLIKKHNVKYNGHRVIRLNELEYINGEVWANIWQTDCIARISAKDGTLL
GWILLPNLRKKLIDEGFRDIDVLNGIAWDQENKRIFVTGKLWPKLFEIKLHLVRHRIPDGYIE
RHCLNLRDNTLSLKTDID
11110000000000000000000000000000000000000000000000000000000000000000000000000000000
00000000000000000000000000000000000000000000000000000000000000000000000000000000000
00000000000000000000000000000000000000000000000000000000000000000000000000000000000
00000111111111111
>P34209_3ca8_0_265
MNITPFPTLSPATIDAINVIGQWLAQDDFSGEVPYQADCVILAGNAVMPTIDAACKIARDQ
QIPLLISGGIGHSTTFLYSAIAQHPHYNTIRTTGRAEATILADIAHQFWHIPHEKIWIEDQSTN
CGENARFSIALLNQAVERVHTAIVVQDPTMQRRTMATFRRMTGDNPDAPRWLSYPGFVPQ
LGNNADSVIFINQLQGLWPVERYLSLLTGELPRLRDDSDGYGPRGRDFIVHVDFPAEVIHA
WQTLKHDAVLIEAMESRSLR
11110000000000000000000000000000000000000000000000000000000000000000000000000000000
00000000000000000000000000000000000000000000000000000000000000000000000000000000000
00000000000000000000000000000000000000000000000000000000000000000000000000000000000
00000000000000001
>P44514_4h2k_0_376
MKEKVVSLAQDLIRRPSISPNDEGCQQIIAERLEKLGFQIEWMPFNDTLNLWAKHGTSEPVI
AFAGHTDVVPTGDENQWSSPPFSAEIIDGMLYGRGAADMKGSLAAMIVAAEEYVKANPN
HKGTIALLITSDEEATAKDGTIHVVETLMARDEKITYCMVGEPSSAKNLGDVVKNGRRGKP
FLTKPGKLLDSITSAIEETIGITPKAETGGGTSDGRFIALMGAEVVEFGPLNSTIHKVNECVSV
EDLGKCGEIYHKMLVNLLDS
00000000000000000000000000000000000000000000000000000000000000000000000000000000000

00000000000000000000000000000000000000000000000000000000000000000000000000000000000
00000000000000011111000000000000000000000000011000000000000000000000000000000000000
0000000000000001
>Q07XY2_3lho_0_265
MHTDVNALFAALWQDYIKMTPSAAKIHQLLGHGAPIINDHIALRTFNIAKVNLSVLAKHF
TSIGYVDSGDYKFEQKKLIAKHFEHPDPKQPKVFISELLVEEFSPEVQKSIHGLIDQVDIAATT
ADNFIYSGRHWDVDKATYQALLAESEYAAWVAALGYRANHFTVSINDLPEFERIEDVNQA
LKQAGFVLNSSGGEVKGSPEVLLEQSSTMADKVVVNFTDGDVEIPSCFYEFARRYPMANG
QLYTGFVAASADKIFESTNAMM
00000000000000000000000000000000000000000000000000000000000000000000000000000000000
00000000000000000000000000000000000000000000000000000000000000000000000000000000000
00000000000000000000000000000000000000000000000000000000000000000000000000000000000
0000111111111111
>Q2YQG6_3mc4_0_265
MNAGLDQVDPIWHSIRAEAEEATRNDPVLGAFLYATILNQPSLEEAVMHRIAERLGHPDVS
ADILRQTFDTMLEANPEWSHVLRVDIQAVYDRDPAYSRFMDPVLYLKGFHAIQTHRLAHW
LYKQGRKDFAYYLQSRSSSIFQTDIHPAARLGSGLFLDHATGLVVGETAVVEDNVSILHGVTL
GGTGKSSGDRHPKIRQGVLIGAGAKILGNIQVGQCSKIAAGSVVLKSVPHNVTVAGVPARII
GETGCTEPSRVMDQMLGDGI
10000000000000000000000000000000000000000000000000000000000000000000000000000000000
00000000000000000000000000000000000000000000000000000000000000000000000000000000000
00000000000000000000011110000000000000000000000000000000000000000000000000000000000
0111111111111111
>Q56187_3qvf_0_265
MTPDFLAIKVGGSLFSRKDEPGSLDDDAVTPFARNFARLAETYRGRMVLISGGGAFGHGAI
RDHDSTHAFSLAGLTEATFEVKKRWAEKLRGIGVDAFPLQLAAMCTLRNGIPQLRSEVLRD
VLDHGALPVLAGDALFDEHGKLWAFSSDRVPEVLLPMVEGRLRVVTLTDVDGIVTDGAGG
DTILPEVDARSPEQAYAALWGSSEWDATGAMHTKLDALVTCARRGAECFIMRGDPGSDLE
FLTAPFSSWPAHVRSTRITTTASA
00000000000000000000000000000000000000000000000000000000000000000000000000000000000
00000000000000000000000000000000000000000000000000000000000000000000000000000000000
00000000000000000000000000000000000000000000000000000000000000000000000000000000000
0000000000001111
>Q5FJ32_3hs3_52_317
TLYQKKSKMIGIIIPDLNNRFYAQIIDGIQEVIQKEGYTALISFSTNSDVKKYQNAIINFENNN
VDGIITSAFTIPPNFHLNTPLVMYDSANINDDIVRIVSNNTKGGKESIKLLSKKIEKVLIQHWP
LSLPTIRERIEAMTAEASKLKIDYLLEETPENNPYISAQSALNKSNQFDAIITVNDLYAAEIIKE
AKRRNLKIPDDFQLVGYDNNILCGYTSPTISTIDQNPKLIGQTAAHRLLDLMSGNNSTRNSII
DVLPIKRDST
11110000000000000000000000000000000000000000000000000000000000000000000000000000000
00000000000000000000000000000000000000000000000000000000000000000000000000000000000
00000000000000000000000000000000000000000000000000000000000000000000000000000000000
0000000000000000
>Q6T755_3uj7_0_265
MTLIENLNSDKTFLENNQYTDEGVKVYEFIFGENYISSGGLEATKKILSDIELNENSKVLDIGS
GLGGGCMYINEKYGAHTHGIDICSNIVNMANERVSGNNKIIFEANDILTKEFPENNFDLIYS
RDAILHLSLENKNKLFQKCYKWLKPTGTLLITDYCATEKENWDDEFKEYVKQRKYTLITVE
EYADILTACNFKNVVSKDLSDYWNQLLEVEHKYLHENKEEFLKLFSEKKFISLDDGWSRKIK
DSKRKMQRWGYFKATKN
11111110000000000000000000000000000000000000000000000000000000000000000000000000000
00000000000000000000000000000000000000000000000000000000000000000000000000000000000

00000000000000000000000000000000000000000000000000000000000000000000000000000000000
0000000000000000
>Q8P8M3_2e11_0_265
MHDLRISLVQGSTRWHDPAGNRDYYGALLEPLAGQSDLVILPETFTSGFSNEAIDKAEDMD
GPTVAWIRTQAARLGAAITGSVQLRTEHGVFNRLLWATPDGALQYYDKRHLFRFGNEHLR
YAAGRERLCVEWKGWRINPQVCYDLRFPVFCRNRFDVERPGQLDFDLQLFVANWPSARA
YAWKTLLRARAIENLCFVAAVNRVGVDGNQLHYAGDSAVIDFLGQPQVEIREQEQVVTTTI
SAAALAEHRARFPAMLDGDSFVLGE
00000000000000000000000000000000000000000000000000000000000000000000000000000000000
00000000000000000000000000000000000000000000000000000000000000000000000000000000000
00000000000000000000000000000000000000000000000000000000000000000000000000000000000
0000000000000001
>A6TB83_3khi_0_264
MFKWPWKADDESGNAEMPWEQALAIPVLAHLSSTEQHKLTQMAARFLQQKRLVALQGL
ELTPLHQARIAMLFCLPVLELGIEWLDGFHEVLIYPAPFIVDDEWEDDIGLVHNQRVVQSG
QSWQQGPVVLNWLDIQDSFDASGFNLVVHEVAHKLDTRNGDRASGVPLIPLREVAGWEH
DLHAAMNNIQDEIDLVGESAASIDAYAATDPAECFAVLSEYFFSAPELFAPRFPALWQRFCH
FYRQDPLARRRENGLQDEGDRRIVH
11111111111111000000000000000000000000000000000000000000000000000000000000000000000
00000000000000111111111100000000001111100000000000000000000000000000000000000000000
00000000000000000000000000000000000000000000000000000000000000000000000000000000000
0001111111111111
>O58877_3hpd_0_264
MKFIIEALKRVRERRPLVHNITNFVVMNTTANALLALGASPVMAHAEEELEEMIRLADAVV
INIGTLDSGWRRSMVKATEIANELGKPIVLDPVGAGATKFRTRVSLEILSRGVDVLKGNFGEI
SALLGEEGKTRGVDSLEYGEEEAKKLTMNAAREFNTTVAVTGAVDYVSDGRRTFAVYNGH
ELLGRVTGTGCMVAALTGAFVAVTEPLKATTSALVTFGIAAEKAYEEAKYPGSFHVKLYDW
LYRINENVIRTYAKVREVEL
00000000000000000000000000000000000000000000000000000000000000000000000000000000000
00000000000000000000000000000000000000000000000000111111111000000000000000000000000
00000000000000000000000000000000000000000000000000000000000000000000000000000000000
0000000000000001
>P10746_1jr2_0_264
MKVLLLKDAKEDDCGQDPYIRELGLYGLEATLIPVLSFEFLSLPSFSEKLSHPEDYGGLIFTSP
RAVEAAELCLEQNNKTEVWERSLKEKWNAKSVYVVGNATASLVSKIGLDTEGETCGNAEK
LAEYICSRESSALPLLFPCGNLKREILPKALKDKGIAMESITVYQTVAHPGIQGNLNSYYSQQ
GVPASITFFSPSGLTYSLKHIQELSGDNIDQIKFAAIGPTTARALAAQGLPVSCTAESPTPQAL
ATGIRKALQPHGCC
00000000000000000000000000000000000000000000000000000000000000000000000000000000000
00000000000000000000000000000000000000000000000000000000000000000000000000000000000
00000000000000000000000000000000000000000000000000000000000000000000000000000000000
0000000000011111
>Q03T70_3jy6_63_327
TQSSKLIAVIVANIDDYFSTELFKGISSILESRGYIGVLFDANADIEREKTLLRAIGSRGFDGLIL
QSFSNPQTVQEILHQQMPVVSVDREMDACPWPQVVTDNFEAAKAATTAFRQQGYQHVV
VLTSELELSRTRQERYRGILAAAQDVDVLEVSESSYNHSEVHQRLTQLITQNDQKTVAFALK
ERWLLEFFPNLIISGLIDNQTVTATGFADTDFIRRMEPKLTLITQNPFLMGASSAEIMLRQLA
GEKVAPEKMVIPAKLQ
11110000000000000000000000000000000000000000000000000000000000000000000000000000000
00000000000000000000000000000000000000000000000000000000000000000000000000000000000
00000000000000000000000000000000000000000000000000000000011000000000000000000000000

0000000000000000
>Q6TFC6_3fsb_0_264
MPNNISKSAIIKEGVIIGENVTIEDNVYIDYGCIIRDNVHIKKGSFIGARSILGEYLVDFYNDRI
NKKHPLIIGENALIRTENVIYGDTIIGDNFQTGHKVTIRENTKIGNNVKIGTLSDIQHHVYIG
NYVNIHSNVFVGEKSIIKDFVWLFPHVVLTNDPTPPSNELLGVTIELFAVIAARSVVLPGIHIN
EDALVGAGAVVTKDVPKETVVVGNPAREICSIRKIKNKITGEQVYPWRYTFKRGMPWEETD
YDTWIKNISIEN
11000000000000000000000000000000000000000000000000000000000000000000000000000000000
00000000000000000000000000000000000000000000000000000000000000000000000000000000000
00000000000000000000000000000000000000000000000000000000000000000000000000000000000
0000000000000111
>Q8PVL4_3mgg_1_265
TEYVHGYSEREALRLSEQAETLEKLLHHDTVYPPGAKVLEAGCGIGAQTVILAKNNPDAEI
TSIDISPESLEKARENTEKNGIKNVKFLQANIFSLPFEDSSFDHIFVCFVLEHLQSPEEALKSLK
KVLKPGGTITVIEGDHGSCYFHPEGKKAIEAWNCLIRVQAYMKGNSLVGRQIYPLLQESGFE
KIRVEPRMVYIDSSKPELVDGFILKTIIPMVEGVKEQSLKMQIIKEEEWEKGIEELHKTAEHGG
TFCYTFFKGWGTK
11111111111111000000000000000000000000000000000000000000000000000000000000000000000
00000000000000000000000000000000000000000000000000000000000000000000000000000000000
00000000000000000000000000000000000000000000000000000000000000000000000000000000000
0000000000000000
>Q9TY95_3ch2_563_827
NMFCNKEYCNRLKDENNCISNLQVEDQGNCDTSWIFASKYHLETIRCMKGYEPTKISALYV
ANCYKGEHKDRCDEGSSPMEFLQIIEDYGFLPAESNYPYNYVKVGEQCPKVEDHWMNLW
DNGKILHNKNEPNSLDGKGYTAYESERFHDNMDAFVKIIKTEVMNKGSVIAYIKAENVMG
YEFSGKKVQNLCGDDTADHAVNIVGYGNYVNSEGEKKSYWIVRNSWGPYWGDEGYFKV
DMYGPTHCHFNFIHSVVIFNVDLPMNN
00000000000000000000000000000000000000000000000000000000000000000000000000000000000
00000000000000000000000000000000000000000000111111111000000000000000000000000000000
00000000000000000000000000000000000000000000000000000000000000000000000000000000000
0000000000000000
>Q9WXJ9_4g10_0_264
MAEPQELTIYHIPGCPFSERVEIMLELKGLRMKDVEIDISKPRPDWLLAKTGGTTALPLLDVE
NGESLKESMVILRYLEQRYPEPAVAHPDPFCHAVEGMLAELAGPFSGAGYRMILNREIGKRE
EMRAAVDAEFGKVDAFLKRYATGSDFLFDDRFGWAEVAFTPMFKRLWFLDYYEDYEVPAN
FDRVLRWRAACTAHPAAQYRSKEELLKLYYDYTQGGGNGRIPEGRSISSFSPDVDWRTRPM
PPRDKWGHAATDAELGLTR
10000000000000000000000000000000000000000000000000000000000000000000000000000000000
00000000000000000000000000000000000000000000000000000000000000000000000000000000000
00000000000000000000000000000000000000000000000000000000000000000000000000000000000
0000000000000001
>A1R6X9_3nze_55_318
SPFDTGPELESQIRNQYGVDVHVVPVLDTLNEAETLDRVAMQAARTIGPLVDSNAIIGVAW
GATLSAVSRHLTRKMTHDSIVVQLNGAGNMQTTGITYASDIMRRFGSAYGARVEQFPVPAF
FDHASTKTAMWNERSVQRILDLQARMSIAIFGVGSVDSDYPSHVYAGGYLDEHDLTMLAA
DDVVGDVATVFFRSDGSSDGITLNERSTGPSHEQLRQVRRRICVVSGASKINGLQGALAAG
LATDLILDEASARRLVSFNGH
00000000000000000000000000000000000000000000000000000000000000000000000000000000000
00000000000000000000000000000000000000000000000000000000000000000000000000000000000
00000000000000000000000000000000000000000000000000000000000000000000000000000000000
000000000000111

>P0C2M5_4h1x_27_290
ASKQSASGTIEVISRENGSGTRGAFTEITGILKKDGDKKIDNTAKTAVIQNSTEGVLSAVQGN
ANAIGYISLGSLTKSVKALEIDGVKASRDTVLDGEYPLQRPFNIVWSSNLSKLGQDFISFIHSK
QGQQVVTDNKFIEAKTETTEYTSQHLSGKLSVVGSTSVSSLMEKLAEAYKKENPEVTIDITSN
GSSAGITAVKEKTADIGMVSRELTPEEGKSLTHDAIALDGIAVVVNNDNKASQVSMAELAD
VFSGKLTTWDKIK
11111110000000000000000000000000000000000000000000000000000000000000000000000000000
00000000000000000000000000000000000000000000000000000000000000000000000000000000000
00000000000000000000000000000000000000000000000000000000000000000000000000000000000
000000000000000
>P53570_3cls_0_263
MKILVAVKQTAALEEDFEIREDGMDVDEDFMMYDLNEWDDFSLEEAMKIKESSDTDVEVV
VVSVGPDRVDESLRKCLAKGADRAVRVWDDAAEGSDAIVVGRILTEVIKKEAPDMVFAGV
QSSDQAYASTGISVASYLNWPHAAVVADLQYKPGDNKAVIRRELEGGMLQEVEINCPAVLT
IQLGINKPRYASLRGIKQAATKPIEEVSLADIGLSANDVGAAQSMSRVRRMYIPEKGRATMIE
GTISEQAAKIIQIINEFKGA
00000000000000000000000000000000000000000000000000000000000000000000000000000000000
00000000000000000000000000000000000000000000000000000000000000000000000000000000000
00000000000000000000000000011111111000000000000000000000000000000000000000000000000
000000000000011
>Q0PBW2_2g5g_19_282
AVLQKSSPLQENKDFYILDTHTQKKISFEDMILELLKADVILLGEKHDEVKHKISQVMIFNA
LEGNLSSQNINFDVALEMLASTEQNHLDKAFKNKKTIKANELTNALNWDKVWKWKDYE
QFVNVVFYSKSKILGANLSRSEITSIYNGAQPLKGYVSTTNEVKKQLFDIISLSHKLNPEENKE
LLDKLVEIQQFKDRRMADVLVHHVNKVLLLAGSYHTSKKIGIPLHIQDFKSSKKIVVVNLSY
GEIDLKDSDYVLIYKGEE
11111110000000000000000000000000000000000000000000000000000000000000000000000000000
00000000000000000000000000000000000000000000000000000000000000000000000000000000000
00000000000000000000000000000000000000000000000000000000000000000000000000000000000
000000000000011
>Q71AW2_1zru_0_263
MTIKNFTFFSPNSTEFPVGSNNDGKLYMMLTGMDYRTIRRKDWSSPLNTALNVQYTNTSIIA
GGRYFELLNETVALKGDSVNYIHANIDLTQTANPVSLSAETANNSNGVDINNGSGVLKVCF
DIVTTSGTGVTSTKPIVQTSTLDSISVNDMTVSGSIDVPVQTLTVEAGNGLQLQLTKKNNDL
VIVRFFGSVSNIQKGWNMSGTWVDRPFRPAAVQSLVGHFAGRDTSFHIDINPNGSITWWG
ANIDKTPIATRGNGSYFIK
10000000111111111100000000000000000000000000000000000000000000000000000000000000000
00000000000000000000000000000000000000000000000000000000000000000000000000000000000
00000000000000000000000000000000000000000000000000000000000000000000000000000000000
000000000000000
>Q7SIF6_1g2n_0_263
AAVQELSIERLLEMESLVADPSEEFQFLRVGPDSNVPPKFRAPVSSLCQIGNKQIAALVVWAR
DIPHFSQLEMEDQILLIKGSWNELLLFAIAWRSMEFLTEERDGVDGTGNRTTSPPQLMCLMP
GMTLHRNSALQAGVGQIFDRVLSELSLKMRTLRVDQAEYVALKAIILLNPDVKGLKNRQE
VEVLREKMFLCLDEYCRRSRSSEEGRFAALLLRLPALRSISLKSFEHLFFFHLVADTSIAGYIRD
ALRNHAPPIDTNMM
00000000000000000000000000000000000000000000000000000000000000000000000000000000000
00000000000000000000111111111100000000000000000000000000000000000000000000000000000
00000000000000000000000000000000000000000000000000000000000000000000000000000000000
000000011111111
>Q8PJX9_4foj_425_688

VSIYDPAAADRAEEERIERWVEQLREALVGDGFLLHYQPVLNLQGEPLELYQAFLRLERNG
EMMSPNAFMAIAEEHDLVTEIDRWVVARAIRQLGERQRAGHKTHLLVRIGPNSFSDPQMI
DTIREQLAVYGVPGERLWLQTPESKVFTHLRNAQQFLAAVSAMDCKVGLEQFGSGLDSFQ
LLAHFHPAFLKLDRGITGDIASARDSQEKIREITSRAQPAGILTMAEFVADAQSMSSFFSAGV
DYVQGDFVAPTGPLMNYEFG
11111111100000000000000000000000000000000001100000000000000000000000000000000000000
00000000000000000000000000000000000000000000000000000000000000000000000000000000000
00000000000000000000000000000000000000000000000000000000000000000000000000000000000
000000000000000
>Q9A2S9_3n0r_2_265
LLARLAPHLPYIRRYARALTGDQATGDHYVRVALEALAAGELVLDANLSPRVALYRVFHAI
WLSSGAQLEVGHDQGLHAGDDAAQRLMRIAPRSRQAFLLTALEGFTPTEAAQILDCDFGE
VERLIGDAQAEIDAELATEVLIIEDEPVIAADIEALVRELGHDVTDIAATRGEALEAVTRRTP
GLVLADIQLADGSSGIDAVKDILGRMDVPVIFITAFPERLLTGERPEPTFLITKPFQPETVKAAI
GQALFFHPRRTAKAA
00000000000000000000000000000000000000000000000000000000000000000001111000000000000
00000000000000000000000000000000000000000000000000000000000000000000000000000000000
00000000000000000000000000000000000000000000000000000000000000000000000000000000000
000000000011111
>O34928_1w17_0_262
MKWMCSICCAAVLLAGGAAQAEAVPNEPINWGFKRSVNHQPPDAGKQLNSLIEKYDAFY
LGNTKEKTIYLTFDNGYENGYTPKVLDVLKKHRVTGTFFVTGHFVKDQPQLIKRMSDEGHI
IGNHSFHHPDLTTKTADQIQDELDSVNEEVYKITGKQDNLYLRPPRGVFSEYVLKETKRLGY
QTVFWSVAFVDWKINNQKGKKYAYDHMIKQAHPGAIYLLHTVSRDNAEALDDAITDLKK
QGYTFKSIDDLMFEKEMRLPSL
11111111111111111111111000000000000000000000000000000000000000000000000000000000000
00000000000000000000000000000000000000000000000000000000000000000000000000000000000
00000000000000000000000000000000000000000000000000000000000000000000000000000000000
00000000001111
>P58315_1ojx_0_262
MANLTEKFLRIFARRGKSIILAYDHGIEHGPADFMDNPDSADPEYILRLARDAGFDGVVFQR
GIAEKYYDGSVPLILKLNGKTTLYNGEPVSVANCSVEEAVSLGASAVGYTIYPGSGFEWKMF
EELARIKRDAVKFDLPLVVWSYPRGGKVVNETAPEIVAYAARIALELGADAMKIKYTGDPK
TFSWAVKVAGKVPVLMSGGPKTKTEEDFLKQVEGVLEAGALGIAVGRNVWQRRDALKFA
RALAELVYGGKKLAEPLNV
11000000000000000000000000000000000000000000000000000000000000000000000000000000000
00000000000000000000000000000000000000000000000000000000000000000000000000000000000
00000000000000000000000000000000000000000000000000000000000000000000000000000000000
00001111111111
>Q14790_3h11_216_478
SESQTLDKVYQMKSKPRGYCLIINNHNFAKAREKVPKLHSIRDRNGTHLDAGALTTTFEEL
HFEIKPHDDCTVEQIYEILKIYQLMDHSNMDCFICCILSHGDKGIIYGTDGQEAPIYELTSQFT
GLKCPSLAGKPKVFFIQACQGDNYQKGIPVETDSEEQPYLEMDLSSPQTRYIPDEADFLLGM
ATVNNCVSYRNPAEGTWYIQSLCQSLRERCPRGDDILTILTEVNYEVSNKDDKKNMGKQM
PQPTFTLRKKLVFPSD
11111100000000000000000000000000000000000000000000000000000000000000000000000000000
00000000000000000000000000000000000000000000000000000000000000000001111111111111111
11111100000000000000000000000000000000000000000000000000000000000001111110000000000
00000000000000
>Q5SLS0_2yr0_0_262
MSSALLRAAYAYDRLRAHPPEVAGQIATAMASAVHPKGEEPVFLELGVGTGRIALPLIARGY

RYIALDADAAMLEVFRQKIAGVDRKVQVVQADARAIPLPDESVHGVIVVHLWHLVPDWP
KVLAEAIRVLKPGGALLEGWDQAEASPEWTLQERWRAFAAEEGFPVERGLHAKRLKEVEE
ALRRLGLKPRTREVARWREERTPREALEALSERLYSFTQGLPEPVHARVMERLWAWAEAEL
GDLDRPFPVEKRFLLRVSRLG
111111111111111100000000000000000000000000000000000000000000000000000000000000000000000000000000
000000000000000000000000000000000000000000000000000000000000000000000000000000000000000000000000
000000000000000000000000000000000000000000000000000000000000000000000000000000000000000000000000
00000000000001
>Q7YTB0_3c7t_68_330
SKSRRWVFALRHGERVDLTYGPWVPHCFENDTYVRKDLNLPLKLAHRAGGKGGYVKDTP
LTRLGWFQAQLVGEGMRMAGVSIKHVYASPALRCVETAQGFLDGLRADPSVKIKVEPGLFE
FKNWHMPKGIDFMTPIELCKAGLNVDMTYKPYVEMDASAETMDEFFKRGEVAMQAAVN
DTEKDGGNVIFIGHAITLDQMVGALHRLRDDMEDVQPYEIGRNLLKVPYCALGAMRGKP
WDVVSPPCPPSINSSSGRFDWRILIK
111000000000000000000000000000000000000000000000000000000000000000000000000000000000000000000000
000000000000000000000000000000000000000000000000000000000000000000000000000000000000000000000000
000000000000000000000000000000000000000000000000000000000000000000000000000000000000000000000000
00000000000001
>Q8A3J3_3bzw_22_284
EQASVTNDFSENKQGCIQHPWQGKKVGYIGDSITDPNCYGDNIKKYWDFLKEWLGITPFV
YGISGRQWDDVPRQAEKLKKEHGGEVDAILVFMGTNDYNSSVPIGEWFTEQEEQVLSAHG
EMKKMVTRKKRTPVMTQDTYRGRINIGITQLKKLFPDKQIVLLTPLHRSLANFGDKNVQPD
ESYQNGCGEYIDAYVQAIKEAGNIWGIPVIDFNAVTGMNPMVEEQLIYFYDAGYDRLHPDT
KGQERMARTLMYQLLALPVAF
111111111111111100000000000000000000000000000000000000000000000000000000000000000000000000000000
000000000000000000000000000000000000000000000000000000000000000000000000000000000000000000000000
000000000000000000000000000000000000000000000000000000000000000000000000000000000000000000000000
00000000000000
>Q972R9_2pjz_0_262
MIQLKNVGITLSGKGYERFSLENINLEVNGEKVIILGPNGSGKTTLLRAISGLLPYSGNIFING
MEVRKIRNYIRYSTNLPEAYEIGVTVNDIVYLYEELKGLDRDLFLEMLKALKLGEEILRRKLY
KLSAGQSVLVRTSLALASQPEIVGLDEPFENVDAARRHVISRYIKEYGKEGILVTHELDMLN
LYKEYKAYFLVGNRLQGPISVSELLESSIVEGERNDALLVLDIMDKKVSIVKGDLGMKFGAL
GSLNRIYGIIGA
000000000000000000000000000000000000000000000000000000000000000000000000000000000000000000000000
000000000000000000000000000000000000000000000000000000000000000000000000000000000000000000000000
000000000000000000000000000000000000000000000000000000000000000000000000000000000000000000000000
00000000000011
>Q97SS7_2gnp_55_317
NFDTNMFKLENYVKEKYSLESLEIIPNEFDDTPTILSERISQVAAGVLRNLIDDNMKIGFSWG
KSLSNLVDLIHSKSVRNVHFYPLAGGPSHIHAKYHVNTLIYEMSRKFHGECTFMNATIVQE
NKLLADGILQSRYFENLKNSWKDLDIAVVGIGDFSNKGKHQWLDMLTEDDFKELTKVKTV
GEICCRFFDSKGKEVYENLQERTIAISLEDLKNIPQSLAVAYGDTKVSSILSVLRANLVNHLIT
DKNTILKVLEEDGDL
000000000000000000000000000000000000000000000000000000000000000000000000000000000000000000000000
000000000000000000000000000000000000000000000000000000000000000000000000000000000000000000000000
000000000000000000000000000000000000000000000000000000000000000000000000000000000000000000000000
00000000000001
>O05871_1rwl_402_663
RPSWSPTQASGQTVLPFTGIDFRLSPSGVAVDSAGNVYVTSEGMYGRVVKLATGSTGTTVLP
FNGLYQPQGLAVDGAGTVYVTDFNNRVVTLAAGSNNQTVLPFDGLNYPEGLAVDTQGAV

YVADRGNNRVVKLAAGSKTQTVLPFTGLNDPDGVAVDNSGNVYVTDTDNNRVVKLEAES
NNQVVLPFTDITAPWGIAVDEAGTVYVTEHNTNQVVKLLAGSTTSTVLPFTGLNTPLAVAV
DSDRTVYVADRGNDRVVKLTS
11111111110000000000000000001111000000000000000000000000000000000000000000000000000
00000000000000000000000000000000000000000000000000000000000000000000000000000000000
00000000000000000000000000000000000000000000000000000000000000000000000000000000000
0000000000000
>P39042_1es5_29_290
VTKPTIAAVGGYAMNNGTGTTLYTKAADTRRSTGSTTKIMTAKVVLAQSNLNLDAKVTIQ
KAYSDYVVANKPSQAHLIVGDKVTVRQLLYGLMLPSGCDAAYALADKYGSGSQAAARVKS
FIGKMNTAATNLGLHNTHFDSFDGIGNGANYSTPRHLTKIASSAMKNSTFRTVVKTKAYTA
KTVTKTGSIRTMDTWKNTNGLLSSYSGAIGVKTGSGPEAKYCLVFAATRGGKTVIGTVLAST
SIPARESDATKIMNYGFAL
11000000000000000000000000000000000000000000000000000000000000000000000000000000000
00000000000000000000000000000000000000000000000000000000000000000000000000000000000
00000000000000000000000000000000000000000000000000000000000000000000000000000000000
0000000000000
>P59745_2i5i_0_261
MSNKKLIINADDFGYTPAVTQGIIEAHKRGVVTSTTALPTSPYFLEAMESARISAPTLAIGVH
LTLTLNQAKPILPREMVPSLVDEAGYFWHQSIFEEKVNLEEVYNEWDAQIISFMKSGRRPDH
IDSHHNVHGKNKKLLGVALALARKYQLPLRNASRSIETKDYLELYQDVRTPDEMLYQFYD
KAISTETILQLLDMVVCSEGEVFEINCHPAFIDTILQNQSGYCMPRIREVEILTSQEVKEAIEER
GILLANYESLAM
10000000000000000000000000000000000000000000000000000000000000000000000000000000000
00000000000000000000000000000000000000000000000000000000000000000000000000000000000
00000000000000000000000000000000000000000000000000000000000000000000000000000000000
0000000000000
>Q13LR5_2q0t_0_261
MTQTTPQPDPSRLRDELVRLHGKASPEWDSLVRLDPRFVDAYLKFAGVPQRRNHLDDKTR
AFIALAADACATQLYAPGVARHIERALSFGATREELIEVLELVSTIGIHTSNVGVPVLLEVLEE
EGLRKGAPPLDERRQKLKAEFETNRGYWHPTWEGLLELDPDLFEAYVEFSSVPWRTGVLSP
KIKEFMYCAFDASATHLYVPGLKLHIRNALRYGATAEELMELLEIVSVTGIHGAELGAPLLE
AALKRSGAAAGEPHA
11111000000000000000000000000000000000000000000000000000000000000000000000000000000
00000000000000000000000000000000000000000000000000000000000000000000000000000000000
00000000000000000000000000000000000000000000000000000000000000000000000000000000000
0000011111111
>Q6T3W5_2j6g_0_261
WMTGDFNGSVDIGGSITADDYRQKWEWKVGTGLNGFGNVLNDLTNGGTKLTITVTGNKP
ILLGRTKEAFATPVTGGVDGIPHIAFTDYEGASVVLRKPDGETNKNGLAYFVLPMKNAGGT
KVGSVKVNASYAGVLGRGGVTSADGELLSLFADGLSSIFYGGLPRGSELSAGSAAAARTKLF
GSLSRDDILGQIQRVNANVTSLVDVAGSYRENMEYTDGNVVSAAYALGIANGQTIEATFNQ
AVTTSTQWSAPLNVAITYY
00000000000000000000000000000000000000000000000000000000000000000000000000000000000
00000000000000001110000000000000000000000000000000000000000000000000000000000000000
00000000000000000000000000000000000000000000000000000000000000000000000000000000000
0000000000000
>Q8DNX4_3qfm_0_261
MDMTKIALLSDIHGNTTALEAVLADARQLGVDEYWLLGDILMPGTGRRRILDLLDQLPITA
RVLGNWEDSLWHGVRKELDSTRPSQRYLLRQCQYVLEEISLEEIEVLHNQPLQIHRQFGDLT
VGISHHLPDKNWGRELIHTGKQEEFDRLVTHPPCDIAVYGHIHQQLLRYGTGGQLIVNPGS

IGQPFFLDAQLRKDLRAQYMILEFDDKGLVDMDFRRVDYDVAAELQLAKDLRLPYFEVYY
ESLVNGIHHTHHQEFLRE
11000000000000000000000000000000000000000000000000000000000000000000000000000000000
00000000000000000000000000000000000000000000000000000000000000000000000000000000000
00000000000000000000000000000000000000000000000000000000000000000000000000000000000
000000000011
>Q9KQM4_1r3d_1_262
LSNQLHFAKPTARTPLVVLVHGLLGSGADWQPVLSHLARTQCAALTLDLPGHGTNPERHC
DNFAEAVEMIEQTVQAHVTSEVPVILVGYSLGGRLIMHGLAQGAFSRLNLRGAIIEGGHFG
LQENEEKAARWQHDQQWAQRFSQQPIEHVLSDWYQQAVFSSLNHEQRQTLIAQRSANLG
SSVAHMLLATSLAKQPYLLPALQALKLPIHYVCGEQDSKFQQLAESSGLSYSQVAQAGHNV
HHEQPQAFAKIVQAMIHSIID
00000000000000000000000000000000000000000000000000000000111111000000000000000000000
00000000000000000000000000000000000000000000000000000000000000000000000000000000000
00000000000000000000000000000000000000000000000000000000000000000000000000000000000
0000000000000
>Q9L9F2_2wk1_0_261
MAPIVETAKETNSDSSLYLDLMIKVLAGTVYEDPAHRENFSHRDSTYREEVRNEGRDWPAN
AHTMIGIKRLENIRQCVEDVIGNNVPGDLVETGVWRGGACILMRGILRAHDVRDRTVWV
ADSFQGIPDVGEDGYAGDRKMALHRRNSVLAVSEEEVRRNFRNYDLLDEQVRFLPGWFKD
TLPTAPIDTLAVLRMDGDLYESTWDTLTNLYPKVSVGGYVIVDDYMMCPPCKDAVDEYRA
KFDIADELITIDRDGVYWQRTR
11111111111110000000000000000000000000111111100000000000000000000000000000000000000
00000000000000000000000000000000000000000000000000000000000000000000000000000000000
00000000000000000000000000000000000000000000000000000000000000000000000000000000000
0000000000000
>P0A5N8_3oxh_0_260
MPKRSEYRQGTPNWVDLQTTDQSAAKKFYTSLFGWGYDDNPVPGGGGVYSMATLNGEA
VAAIAPMPPGAPEGMPPIWNTYIAVDDVDAVVDKVVPGGGQVMMPAFDIGDAGRMSFIT
DPTGAAVGLWQANRHIGATLVNETGTLIWNELLTDKPDLALAFYEAVVGLTHSSMEIAAG
QNYRVLKAGDAEVGGCMEPPMPGVPNHWHVYFAVDDADATAAKAAAAGGQVIAEPAD
IPSVGRFAVLSDPQGAIFSVLKPAPQQ
00000000000000000000000000000000000000000111110000000000000000000000011100000000000
00000000000000000000000000000000000000000000000000000000000000000000000000000000000
00000011111110000000000000000000000000000000000000000000000000000000000000000000000
000000000111
>Q01926_3rkg_47_307
KQLLSLKPISASDSLFISCTVFNSKGNIISMSEKFPKWSFLTEHSLFPRDLRKIDNSSIDIIPTIMC
KPNCIVINLLHIKALIERDKVYVFDTTNPSAAAKLSVLMYDLESKLSSTKNNSQFYEHRALE
SIFINVMSALETDFKLHSQICIQILNDLENEVNRLKLRHLLIKSKDLTLFYQKTLLIRDLLDELL
ENDDDLANMYLTVKKSPKDNFSDLEMLIETYYTQCDEYVQQSESLIQDIKSTEEIVNIILDA
NRNSL
00000000000000000000000000000000000000000000000000000000000000000000000000000000000
00000000000000000000000000000000000000000000000000000000000000000000000000000000000
00000000000000000000000000000000000000000000000000000000000000000000000000000000000
000000000111
>Q04066_1vkh_0_260
MSNTVRAISPDITLFNKTLTFQEISQNTREAVIYIHGGAWNDPENTPNDFNQLANTIKSMDT
ESTVCQYSIEYRLSPEITNPRNLYDAVSNITRLVKEKGLTNINMVGHSVGATFIWQILAALKD
PQEKMSEAQLQMLGLLQIVKRVFLLDGIYSLKELLVEYPEYDCFTRLAFPDGIQMYEEEPSRV
MPYVKKALSRFSIDMHLVHSYSDELLTLRQTNCLISCLQDYQLSFKLYLDDLGLHNDVYKN

GKVAKYIFDNIC
01100000000000000000000000000000000000000000000000000000000000000000000000000000000
00000000000000000000000000000000000000000000000000000000000000000000000000000000000
00000000000000000000000000000000000000000000000000000000000000000000000000000000000
000000000000
>Q1M1K6_4e9o_0_260
MPQQLSPINIETKKAISNARLKPLDIHYNESKPTTIQNTGKLVRINFKGGYISGGFLPNEYVLS
SLHIYWGKEDDYGSNHLIDVYKYSGEINLVHWNKKKYSSYEEAKKHDDGLIIISIFLQVLDH
KNVYFQKIVNQLDSIRSANTSAPFDSVFYLDNLLPSKLDYFTYLGTTINHSADAVWIIFPTPI
NIHSDQLSKFRTLLSLSNHEGKPHYITENYRNPYKLNDDTEVYYSGEIIRAATTSPARENYFM
RWLSDLRET
10000000000000000000000000000000000000000000000000000000000000000000000000000000000
00000000000000000000000000000000000000000000000000000000000000000000000000000000000
00000000000000000000000000000000000000111000000000000000000000000000111111111111111
111111111111
>Q1PLH8_3ub1_98_358
MEKQSSLETQAFSFAEEFAWDYFSRYPSDTQDFVRRITKYTTEQLANEMNNGTYSDVIYTSA
FYFEKYSENQVNVSVKARVRVYTPKAGQEQTPQDQLQYDTNLVDYYLEVPIVFDKDMNM
AVDALPVMTAPPEKAYFKNKEFSGTSENDADKTKKITDSVSQFFKAYYEQNQTQIDYFLVD
GADIKGAGQKFSFNKIDRINIYKLSDKEFLAIVDLNVDSFGNAIKQGFNLTVVQEGDKFLVK
TLEPRTSNIDLNNKSNN
11111000000000000000000000000000000000000000000000000000000000000000000000000000000
00000000000000000000000000000000000000000000000000000000000000000000000000000000000
00000000000000000000000000000000000000000000000000000000000000000000000000000000000
000000011111
>Q64105_1sep_0_260
MEADGLGCAVCVLTGASRGFGRALAPQLARLLSPGSVMLVSARSESMLRQLKEELGAQQP
DLKVVLAAADLGTEAGVQRLLSAVRELPRPEGLQRLLLINNAATLGDVSKGFLNVNDLAE
VNNYWALNLTSMLCLTSGTLNAFQDSPGLSKTVVNISSLCALQPYKGWGLYCAGKAARD
MLYQVLAAEEPSVRVLSYAPGPLDNDMQQLARETSKDPELRSKLQKLKSDGALVDCGTSA
QKLLGLLQKDTFQSGAHVDFYD
11000000000000000000000000000000000000000000000000000000000000000000000000000000000
00000000000000000000000000000000000000000000000000000000000000000000000000000000000
00000000000000000000000000000000000000000000000000000000000000000000000000000000000
000000000000
>Q6QPJ4_2j89_0_260
MLQTLSTHLSSTSTSTTTPLLLLSKPFLSPSAKSQLSHSKPFNFPRTLKPISYYKPPMANILSKL
GFGTRSPDPSTMDPTIPQGPDDDLPAPGQQFAQFGAGCFWGVELAFQRVPGVTKTEVGYT
QGLLHNPTYEDVCTGTTNHNEVVRVQYDPKECSFDTLIDVLWARHDPTTLNRQGNDVGT
QYRSGIYYYTPEQEKAAKESLERQQKLLNRKIVTEILPAKKFYRAEEYHQQYLAKGGRFGFM
QSAEKGCNDPIRCYG
11111111111111111111111111111111111111111111111111111111111111111111111111111100000
00000000000000000000000000000000000000000000000000000000000000000000000000000000000
00000000000000000000000000000000000000000000000000000000000000000000000000000000000
000000000000
>Q7X4S4_2jem_0_260
MKNNHLLKSILLWGAVCIIVLAGPLSAFAASSSNPSDKLYFKNKKYYIFNNVWGADQVSG
WWQTIYHNSDSDMGWVWNWPSNTSTVKAYPSIVSGWHWTEGYTAGSGFPTRLSDQKNI
NTKVSYSISANGTYNAAYDIWLHNTNKASWDSAPTDEIMIWLNNTNAGPAGSYVETVSIG
GHSWKVYKGYIDAGGGKGWNVFSFIRTANTQSANLNIRDFTNYLADSKQWLSKTKYVSSV
EFGTEVFGGTGQINISNWDVTVR

11111111111111111111111111111000000000000000000000000000000000000000000000000000000
00000000000000000000000000000000000000000000000000000000000000000000000000000000000
00000000000000000000000000000000000000000000000000000000000000000000000000000000000
000000000000
>Q8IL32_1y6z_384_644
QLPIWKQDEKSLTENDYYSFYKNTFKAYDDPLAYVHFNVEGQISFNSILYIPGSLPWELSKN
MFDEESRGIRLYVKRVFINDKFSESIPRWLTFLRGIVDSENLPLNVGREILQKSKMLSIINKRIV
LKSISMMKGLKETGGDKWTKFLNTFGKYLKIGVVEDKENQEEIASLVEFYSINSGDKKTDLD
SYIENMKEDQKCIYYISGENKKTAQNSPSLEKLKALNYDVLFSLEPIDEFCLSSLTVNKYKGY
EVLDVNKAD
10000000000000000000000000000000000000000000000000000000000000000000000000000000000
00000000000000000000111111111110000000000000000000000000000000000000000000000000000
00000000000000000000000000000000000000000000000000000000000000000000000000000000000
000000000001
>Q8XDG3_4htf_0_260
MQDRNFDDIAEKFSRNIYGTTKGQLRQAILWQDLDRVLAEMGPQKLRVLDAGGGEGQTAI
KMAERGHQVILCDLSAQMIDRAKQAAEAKGVSDNMQFIHCAAQDVASHLETPVDLILFH
AVLEWVADPRSVLQTLWSVLRPGGVLSLMFYNAHGLLMHNMVAGNFDYVQAGMPKKK
KRTLSPDYPRDPTQVYLWLEEAGWQIMGKTGVRVFHDYLREKHQQRDCYEALLELETRYC
RQEPYITLGRYIHVTARKPQSKDKV
00000000000000000000000000000000000000000000000000000000000000000000000000000000000
00000000000000000000000000000000000000000000000000000000000000000000000000000000000
00000000011110000000000000000000000000000000000000000000000000000000000000000000000
000000011111
>Q970L2_3adr_0_260
MPCRGLHSIPAGPVEFPEIATVYVMCGEKLTVMIDAGVSNSIADFSFLDKLDYIVLTHLHID
HIGLLPELLQVYKAKVLVKSGFKKYLTSEDGLKKLNESAEKVLGDLYYVYGGLEKKLDQDK
VIEVEGNEEFDLGGYRMRLIYTPGHARHHMSVLVDDFLFTGDSAGAYFNGVVIPTTPPVIDY
KMYMESLKRQIELKPKVVGFAHGGLVSPKIMEEHLKQMLSKEEIQINVDIGGVAGEILRKQI
EVNLRGLRESKKSI
10000000000000000000000000000000000000000000000000000000000000000000000000000000000
00000000000000000000000000000000000000000000000000000000000000000000000000000000000
00000000000000000000000000000000000000000000000000000000000000000000000000000000000
000000000001
>Q9AAV3_1xfj_0_260
MKTPALPTVQSPLLSSLPGVKHAFFTRQGGVSKGIYDSLNVGRGSQDEPADVEENRARIAR
WFGGGPEDLNVCYQIHSTIAIVADGSWGDARPEGDAVVSKTPGVICGAMAADCAPVLLVD
PEARIVAAAHAGWRGALDGVVQSAVDRMVELGASPANITGVVGPCIGPKSYEVGLEFLHR
FEADCPGSGRFFKPGASEDKRFFDLPAFVLDRLATAGVERREWVGRDTRAEEEWFFSNRRA
FLNNDGDYGRLLSAITLEA
11110000000000000000000000000000000000000000000000000000000000000000000000000000000
00000000000000000000000000000000000000000000000000000000000000000000000000000000000
00000000000000000000000000000000000000000000000000000000000000000000000000000000000
000000000001
>Q9H9E3_3hr0_524_784
QQGKFDTKGIESTDEAKMSFLVTLNNVEVCSENISTLKKTLESDCTKLFSQGIGGEQAQAKF
DSCLSDLAAVSNKFRDLLQEGLTELNSTAIKPQVQPWINSFFSVSHNIEEEEFNDYEANDPW
VQQFILNLEQQMAEFKASLSPVIYDSLTGLMTSLVAVELEKVVLKSTFNRLGGLQFDKELRSL
IAYLTTVTTWTIRDKFARLSQMATILNLERVTEILDYWGPNSGPLTWRLTPAEVRQVLALRID
FRSEDIKRLRL
11111111111000000000000000000000000000000000000000000000000000000000000000000000000

00000000000000000000000000000000000000000000000000000000000000000000000000000000000
00000000000000000000000000000000000000000000000000000000000000000000000000000000000
000000000000
>Q9UHY7_1yns_0_260
MVVLSVPAEVTVILLDIEGTTTPIAFVKDILFPYIEENVKEYLQTHWEEEECQQDVSLLRKQA
EEDAHLDGAVPIPAASGNGVDDLQQMIQAVVDNVCWQMSLDRKTTALKQLQGHMWRA
AFTAGRMKAEFFADVVPAVRKWREAGMKVYIYSSGSVEAQKLLFGHSTEGDILELVDGHF
DTKIGHKVESESYRKIADSIGCSTNNILFLTDVTREASAAEEADVHVAVVVRPGNAGLTDDE
KTYYSLITSFSELYLPSST
11100000000000000000000000000000000000000000000000000000000000000000000000000000000
00000000000000000000000000000000000000000000000000000000000000000000000000000000000
00000000000000000000000000000000000000000000000000000000000000000000000000000000000
000000001111
>D0VWU0_3cxm_0_259
MGTLEAQTQGPGSMMSVSSSRWLAGLITNPEWRDFLAPITADSWRKGAFIRIERFLDEEKE
KGRVILPPAADIFNAFNSCPFRGLKVVLLGQDPYHDLHQAHGLCFSVLPEVPLPPSLRNIYK
ELTTDIAGFQAPKHGYLQSWSEQGMLMLNATLTVEAHKANSHSKTSGWAAFTDAVIQHLS
QHHPNRLVFLLWGGYAQQKKRLIDANRHVVLENVHPSPLSANRGWFGCRCFSACNEALQ
RMSHLPMHWQLPLNAPLH
11111111111111110000000000000000000000000000000000000000000000000000000000000000000
00000000000000000000000000000000000000000000000000000000000000000000000000000000000
00000000000000000000000000000000000000000000000000000000000000000000000000000000000
00000000011
>O67769_2yvt_0_259
MGIMPRKVLAIKNFKERFDLLPKLKGVIAEKQPDILVVVGNILKNEALEKEYERAHLARREP
NRKVIHENEHYIIETLDKFFREIGELGVKTFVVPGKNDAPLKIFLRAAYEAETAYPNIRVLHE
GFAGWRGEFEVIGFGGLLTEHEFEEDFVLKYPRWYVEYILKFVNELKPRRLVTIFYTPPIGEFV
DRTPEDPKHHGSAVVNTIIKSLNPEVAIVGHVGKGHELVGNTIVVNPGEFEEGRYAFLDLTQ
HKIKLEQFS
11100000000000000000000000000000000000000000000000000000000000000000000000000000000
00000000000000000000000000000000000000000000000000000000000000000000000000000000000
00000000000000000000000000000000000000000000000000000000000000000000000000000000000
00000000000
>P06875_1gkf_26_285
EQSSSEIKIVRDEYGMPHIYANDTWHLFYGYGYVVAQDRLFQMEMARRSTQGTVAEVLGK
DFVKFDKDIRRNYWPDAIRAQIAALSPEDMSILQGYADGMNAWIDKVNTNPETLLPKQFN
TFGFTPKRWEPFDVAMIFVGTMANRFSDSTSEIDNLALLTALKDKYGVSQGMAVFNQLKW
LVNPSAPTTIAVQESNYPLKFNQQNSQTAALLPRYDLPAPMLDRPAKGADGALLALTAGK
NRETIAAQFAQGGANGLAGY
11000000000000000000000000000000000000000000000000000000000000000000000000000000000
00000000000000000000000000000000000000000000000000000000000000000000000000000000000
00000000000000000000000000000000000000000111111111111111111111111111111111111111111
11111111111
>P23192_1g60_0_259
MLEINKIHQMNCFDFLDQVENKSVQLAVIDPPYNLSKADWDSFDSHNEFLPFTYRWIDKV
LDKLDKDGSLYIFNTPFNCAFICQYLVSKGMIFQNWITWDKRDGMGSAKRGFSTGQETILFF
SKSKNHTFNYDEVRVPYESTDRIKHASEKGILKNGKRWFPNPNGRLCGEVWHFSSQRHKE
KVNGKTVKLTHITPKPRDLIERIIRASSNPNDLVLDCFMGSGTTAIVAKKLGRNFIGCDMNA
EYVNQANFVLNQLEIN
00000000000000000000000000000000000000000000000000000000000000000000000000000000000
00000000000000000000000000000000000000000000000000000000000000000000000000000000000

0000000000011111111111111110000000000000000000000000000000000000000000000000000000000
00000001111
>P27707_1p62_0_259
MATPPKRSCPSFSASSEGTRIKKISIEGNIAAGKSTFVNILKQLCEDWEVVPEPVARWCNVQS
TQDEFEELTMSQKNGGNVLQMMYEKPERWSFTFQTYACLSRIRAQLASLNGKLKDAEKPV
LFFERSVYSDRYIFASNLYESECMNETEWTIYQDWHDWMNNQFGQSLELDGIIYLQATPETC
LHRIYLRGRNEEQGIPLEYLEKLHYKHESWLLHRTLKTNFDYLQEVPILTLDVNEDFKDKYE
SLVEKVKEFLSTL
11111111111111111000000000000000000000000000000000000000000000011111111111110000000
00000000000000000000000000000000000000000000000000000000000000000000000000000000000
00000000000000000000000000000000000000000000000000000000000000000000000000000000000
00000000000
>P40545_2agk_1_260
TKFIGCIDLHNGEVKQIVGGTLTSKKEDVPKTNFVSQHPSSYYAKLYKDRDVQGCHVIKLGP
NNDDAAREALQESPQFLQVGGGINDTNCLEWLKWASKVIVTSWLFTKEGHFQLKRLERLT
ELCGKDRIVVDLSCRKTQDGRWIVAMNKWQTLTDLELNADTFRELRKYTNEFLIHAADVE
GLCGGIDELLVSKLFEWTKDYDDLKIVYAGGAKSVDDLKLVDELSHGKVDLTFGSSLDIFGG
NLVKFEDCCRWNEKQG
00000000000000001111111111111111111100000000000000000000000000000000000000000000000
00000000000000000000000000000000000000000000000000000000000000000000000000000000000
00000000000001111110000000000000000000000000000000000000000000000000000000000000000
00000000000
>P83772_1j2t_0_259
MSKSVFVGELTWKEYEARVAAGDCVLMLPVGALEQHGHHMCMNVDVLLPTAVCKRVAE
RIGALVMPGLQYGYKSQQKSGGGNHFPGTTSLDGATLTGTVQDIIRELARHGARRLVLMN
GHYENSMFIVEGIDLALRELRYAGIQDFKVVVLSYWDFVKDPAVIQQLYPEGFLGWDIEHG
GVFETSLMLALYPDLVDLDRVVDHPPATFPPYDVFPVDPARTPAPGTLSSAKTASREKGELIL
EVCVQGIADAIREEFPPT
11000000000000000000000000000000000000000000000000000000000000000000000000000000000
00000000000000000000000000000000000000000000000000000000000000000000000000000000000
00000000000000000000000000000000000000000000000000000000000000000000000000000000000
00000000001
>Q3MEY9_3ccf_0_259
MTNLGTAKNFWDATLYQDKHSFVWQYGEDLLQLLNPQPGEFILDLGCGTGQLTEKIAQSG
AEVLGTDNAATMIEKARQNYPHLHFDVADARNFRVDKPLDAVFSNAMLHWVKEPEAAI
ASIHQALKSGGRFVAEFGGKGNIKYILEALYNALETLGIHNPQALNPWYFPSIGEYVNILEK
QGFDVTYAALFNRPTTLAEGEFGMANWIQMFASAFLVGLTPDQQVQLIRKVEATLQDKLY
HQESWTADYRRIRIVSIKAQ
11111111111111111000000000000000000000000000000000000000000000000000000000000000000
00000000000000000000000000000000000000000000000000000000000000000000000000000000000
00000000000000000000000000000000000000000000000000000000000000000000000000000000000
00000000000
>Q5HE50_2ihy_0_259
MLIQLDQIGRMKQGKTILKKISWQIAKGDKWILYGLNGAGKTTLLNILNAYEPATSGTVNL
FGKMPGKVGYSAETVRQHIGFVSHSLLEKFQEGERVIDVVISGAFKSIGVYQDIDDEIRNEA
HQLLKLVGMSAKAQQYIGYLSTGEKQRVMIARALMGQPQVLILDEPAAGLDFIARESLLSIL
DSLSDSYPTLAMIYVTHFIEEITANFSKILLLKDGQSIQQGAVEDILTSENMSRFFQKNVAVQR
WNNRFSMAMLE
00000000000000000000000000000000000000000000000000000000000000000000000000000000000
00000000000000000000000000000000000000000000000000000000000000000000000000000000000
00000000000000000000000000000000000000000000000000000000000000000000000000000000000

00000000001
>Q8L9J9_2apj_0_259
MEGGSITPGEDKPEIQSPIPPNQIFILSGQSNMAGRGGVFKDHHNNRWVWDKILPPECAPN
SSILRLSADLRWEEAHEPLHVDIDTGKVCGVGPGMAFANAVKNRLETDSAVIGLVPCASGG
TAIKEWERGSHLYERMVKRTEESRKCGGEIKAVLWYQGESDVLDIHDAESYGNNMDRLIK
NLRHDLNLPSLPIIQVAIASGGGYIDKVREAQLGLKLSNVVCVDAKGLPLKSDNLHLTTEA
QVQLGLSLAQAYLSNFC
11111111111111111000000000000000000000000000000000000000000000000000000000000000000
00000000000000000000000000000000000000000000000000000000000000000000000000000000000
00000000000000000000000000000000000000000000000000000000000000000000000000000000000
00000000000
>Q9I1L3_1yx1_0_259
MSLHPVSISLSSYGADLVRSRGQASFLPLLAMAGAQRVELREELFAGPPDTEALTAAIQLQG
LECVFSSPLELWREDGQLNPELEPTLRRAEACGAGWLKVSLGLLPEQPDLAALGRRLARH
GLQLLVENDQTPQGGRIEVLERFFRLAERQQLDLAMTFDIGNWRWQEQAADEAALRLGR
YVGYVHCKAVIRNRDGKLVAVPPSAADLQYWQRLLQHFPEGVARAIEYPLQGDDLLSLSRR
HIAALARLGQPQEERQHG
11000000000000000000000000000000000000000000000000000000000000000000000000000000000
00000000000000000000000000000000000000000000000000000000000000000000000000000000000
00000000000000000000000000000000000000000000000000000000000000000000000000000000000
00011111111
>Q9UG22_2xtp_0_259
MDQNEHSHWGPHAKGQCASRSELRIILVGKTGTGKSAAGNSILRKQAFESKLGSQTLTKTC
SKSQGSWGNREIVIIDTPDMFSWKDHCEALYKEVQRCYLLSAPGPHVLLLVTQLGRYTSQD
QQAAQRVKEIFGEDAMGHTIVLFTHKEDLNGGSLMDYMHDSDNKALSKLVAACGGRICA
FNNRAEGSNQDDQVKELMDCIEDLLMEKNGDHYTNGLYSLIQRSKCGPVGSDERVKEFKQ
SLIKYMETQRSYTALAEAN
11111111111111111110000000000000000000000000000000000000000000000000000000000000000
00000000000000000000000000000000000000000000000000000000000000000000000000000000000
00000000000000000000000000000000000000000000000000000000111111111111100000000000000
00000111111
>Q0P9X8_2v25_0_258
MVFRKSLLKLAVFALGACVAFSNANAAEGKLESIKSKGQLIVGVKNDVPHYALLDQATGEI
KGFEVDVAKLLAKSILGDDKKIKLVAVNAKTRGPLLDNGSVDAVIATFTITPERKRIYNFSEP
YYQDAIGLLVLKEKKYKSLADMKGANIGVAQAATTKKAIGEAAKKIGIDVKFSEFPDYPSIK
AALDAKRVDAFSVDKSILLGYVDDKSEILPDSFEPQSYGIVTKKDDPAFAKYVDDFVKEHK
NEIDALAKKWGL
11111111111111111111111111110000000000000000000000000000000000000000000000000000000
00000000000000000000000000000000000000000000000000000000000000000000000000000000000
00000000000000000000000000000000000000000000000000000000000000000000000000000000000
0000000000
>Q5LES9_3kkz_0_258
MSNENKTIHDFELNLICDFFSNMERQGPGSPEVTLKALSFIDNLTEKSLIADIGCGTGGQTM
VLAGHVTGQVTGLDFLSGFIDIFNRNARQSGLQNRVTGIVGSMDDLPFRNEELDLIWSEGA
IYNIGFERGLNEWRKYLKKGGYLAVSECSWFTDERPAEINDFWMDAYPEIDTIPNQVAKIHK
AGYLPVATFILPENCWTDHYFTPKVAAQKIFLTKYAGNKIAEEFSMLQSIEEELYHKYKEYYG
YTFFIAKKIRL
11111000000000000000000000000000000000000000000000000000000000000000000000000000000
00000000000000000000000000000000000000000000000000000000000000000000000000000000000
00000000000000000000000000000000000000000000000000000000000000000000000000000000000
0000000000

>Q5XA29_3qpb_0_258
MQNYSGEVGLQYHLQIRPGDVGRYVIMPGDPKRCAKIAEHFDNAVLVADSREYVTYTGTL
NGEKVSVTSTGIGGPSASIAMEELKLCGADTFIRVGTCGGIELDVKGGDIVIATGAIRMEGTS
KEYAPIEFPAVADLEVTNALVNAAKKLGYTSHAGVVQCKDAFYGQHEPERMPVSYELLNK
WEAWKRLGTKASEMESAALFVAASHLGVRCGSDFLVVGNQERNALGMDNPMAHDTEA
AIQVAVEALRTLIENDKSQ
11111110000000000000000000000000000000000000000000000000000000000000000000000000000
00000000000000000000000000000000000000000000000000000000000000000000000000000000000
00000000000000000000000000000000000000000000000000000000000000000000000000000000000
0000000011
>Q72K29_3zwd_0_258
MIVFTDLDGTLLDERGELGPAREALERLRALGVPVVPVTAKTRKEVEALGLEPPFIVENGGG
LYLPRDWPVRAGRPKGGYRVVSLAWPYRKVRARLREAEALAGRPILGYGDLTAEAVARLT
GLSREAARRAKAREYDETLVLCPEEVEAVLEALEAVGLEWTHGGRFYHAAKGADKGRAV
ARLRALWPDPEEARFAVGLGDSLNDLPLFRAVDLAVYVGRGDPPEGVLATPAPGPEGFRYA
VERYLLPRLSRRGGSGP
00000000000000000000000000000000000000000000000000000000000000000000000000000000000
00000000000000000000000000000000000000000000000000000000000000000000000000000000000
00000000000000000000000000000000000000000000000000000000000000000000000000000000000
0001111111
>Q7A652_2q8p_30_288
SGEFRIVPTTVALTMTLDKLDLPIVGKPTSYKTLPNRYKDVPEIGQPMEPNVEAVKKLKPTH
VLSVSTIKDEMQPFYKQLNMKGYFYDFDSLKGMQKSITQLGDQFNRKAQAKELNDHLNS
VKQKIENKAAKQKKHPKVLILMGVPGSYLVATDKSYIGDLVKIAGGENVIKVKDRQYISSNT
ENLLNINPDIILRLPHGMPEEVKKMFQKEFKQNDIWKHFKAVKNNHVYDLEEVPFGITAN
VDADKAMTQLYDLFYK
10000000000000000000000000000000000000000000000000000000000000000000000000000000000
00000000000000000000000000000000000000000000000000000000000000000000000000000000000
00000000000000000000000000000000000000000000000000000000000000000000000000000000000
0000000000
>Q7BUF9_3flb_0_258
MHRPEAEKWLRRFERAPDARARLVCLPHAGGSASFFFPLAKALAPAVEVLAVQYPGRQDR
RHEPPVDSIGGLTNRLLEVLRPFGDRPLALFGHSMGAIIGYELALRMPEAGLPAPVHLFASG
RRAPSRYRDDDVRGASDERLVAELRKLGGSDAAMLADPELLAMVLPAIRSDYRAVETYRH
EPGRRVDCPVTVFTGDHDPRVSVGEARAWEEHTTGPADLRVLPGGHFFLVDQAAPMIATM
TEKLAGPALTGSTGGNS
11110000000000000000000000000000000000000000000000000000000000000000000000000000000
00000000000000000000000000000000000000000000000000000000000000000000000000000000000
00000000000000000000000000000000000000000000000000000000000000000000000000000000000
1111111111
>Q9MCI8_2xqh_159_417
SDAKASGEFSVAVGNGARATEKASTAVGSWAAADGKQSTALGVGTYAYANASTALGSVAF
VDNTATYGTAAGNRAKVDKDATEGTALGAKATVTNKNSVALGANSVTTRDNEVYIGYKT
GTESDKTYGTRVLGGLSDGTRNSDAATVGQLNRKVGGVYDDVKARITVESEKQKKYTDQK
TSEVNEKVEARTTVGVDSDGKLTRAEGATKTIAVNDGLVALSGRTDRIDYAVGAIDGRVTR
NTQSIEKNSKAIAANTRTL
00000000000000000000000000000000000000000000000000000000000000000000000000000000000
00000000000000000000000000000000000000000000000000000000000000000000000000000000000
00000000000000000000000000000000000000000000000000000000000000000000000000000000000
0000000001
>D2PUG5_4ius_0_257

MGIAARIASAQLQGLVRSRRQVVAGPLVGLLHDEDVWFLSRAVAAEPGVPFDPAEVPWAL
ETIRKAFAAEGRWLSAELVEEANPGLAYVLVEHGMTIVSRPPLLAVEPGDLLVPEFPAGVTA
AVVASAEEQEAANAIAGDAYETDDVASPFQPEPADGGAVLIRMDGVPVATAAWTAIADGV
TEVAGVGTLHSHRRQGLGALATAYATQQAFEVGGATLAWLTPGDDGADRIYRRLGYEPKA
TAVHLGDPGGHLADLR
00000000000000000000000000000000000000000000000000000000000000000000000000000000000
00000000000000000000000000000000000000000000000000000000000000110000000000000000000
00000000000000000000000000000000000000000000000000000000000000000000000000000000000
000000000
>O28471_1ztf_0_257
MKDLKKIESYLDKLRIKEKDGEERKIYAEVLDGRTLKTLYKLSAKGYITAMGGVISTGKEAN
VFYADGVFDGKPVAMAVKIYRIETSEFDKMDEYLYGDERFDMRRISPKEKVFIWTEKEFRNL
ERAKEAGVSVPQPYTYMKNVLLMEFIGEDELPAPTLVELGRELKELDVEGIFNDVVENVKR
LYQEAELVHADLSEYNIMYIDKVYFIDMGQAVTLRHPMAESYLERDVRNIIRFFSKYGVKAD
FEEMLKEVKGE
11110000000000000000000000000000000000000000000000000000000000000000000000000000000
00000000000000000000000000000000000000000000000000000000000000000000000000000000000
00000000000000000000000000000000000000000000000000000000000000000000000000000000000
000000001
>P33183_3ca6_305_562
TSFTRNIVGRDGLCVDVRNGYDTDGTPLQLWPCGTQRNQRWTFDSDDTIRSMGKCMTAN
GLNNGSNIVIFNCSTAAENAIKWEVPIDGSIINPSSGLVMTAPRAASRTILLLEDNIYAASQG
WTVTNNVKPIVASIVGYKEMCLQSNGENNGVWMEDCEATSLQQQWALYGDRTIRVNSTR
GLCVTTNGYNSKDLIIILKCQGLPSQRWFFNSDGAIVNPKSRHVMDVRASNVSLREIIIFPAT
GNPNQQWVTQVLPS
00000000000000000000000000000000000000000000000000000000000000000000000000000000000
00000000000000000000000000000000000000000000000000000000000000000000000000000000000
00000000000000000000000000000000000000000000000000000000000000000000000000000000000
000000001
>Q02790_1q1c_1_258
TAEEMKATESGAQSAPLPMEGVDISPKQDEGVLKVIKREGTGTEMPMIGDRVFVHYTGWL
LDGTKFDSSLDRKDKFSFDLGKGEVIKAWDIAIATMKVGEVCHITCKPEYAYGSAGSPPKIPP
NATLVFEVELFEFKGEDLTEEEDGGIIRRIQTRGEGYAKPNEGAIVEVALEGYYKDKLFDQRE
LRFEIGEGENLDLPYGLERAIQRMEKGEHSIVYLKPSYAFGSVGKEKFQIPPNAELKYELHLK
SFEKAKESW
11111111111111111110000000000000000000000000000000000000000000000000000000000000000
00000000000000000000000000000000000000000000000000000000000000000000000000000000000
00000000000000000000000000000000000000000000000000000000000000000000000000000000000
000000011
>Q2G812_3cjy_0_257
MTEAFPALVRQDDARYAITVGPDLAVGPPGHAYLFGGASMALALDVAAETVGRPVVQGSL
QFVSFTPLGSVLDLTVEVLQSGRTLAQARVAGTVDGRLVFHSGISLGMREGFSARQWALAP
PVPQPDNCPPCTTLPAQDDNARYLEGIEVREAGGPEVPSGRTRLWLRRKDGAPLDAASLA
MFADFLPIALGRATGCSGGGNSLDNSLRITGAAAPGWCLCDMIIPSSASGFAQGQVTLWDQ
SGRLLATGAQSLLLKG
11100000000000000000000000000000000000000000000000000000000000000000000000000000000
00000000000000000000000000000000000000000000000000000000000000000000000000000000000
00000000000000000000000000000000110000000000000000000000000000000000000000000000000
000000000
>Q97S09_2uz0_1_258
AVMKIEYYSQVLDMEWGVNVLYPDANRVEEPECEDIPVLYLLHGMSGNHNSWLKRTNVE

RLLRGTNLIVVMPNTSNGWYTDTQYGFDYYTALAEELPQVLKRFFPNMTSKREKTFIAGLS
MGGYGCFKLALTTNRFSHAASFSGALSFQNFSPESQNLGSPAYWRGVFGEIRDWTTSPYSLE
SLAKKSDKKTKLWAWCGEQDFLYEANNLAVKNLKKLGFDVTYSHSAGTHEWYYWEKQL
EVFLTTLPIDFKLEERLT
00000000000000000000000011111111100000000000000000000000000000000000000000000000000
00000000000000000000000000000000000000000000000000000000000000000000000000000000000
00000000000000000000000000000000000000000000000000000000000000000000000000000000000
000000001
>A9CI91_2odf_0_256
MTVRSRFFTEAEGKAVGVENAAAKGDVLLVCEHASATIPQKYGTLGLSADVLSSHAAWDP
GALAVARLLSEKFHATLVYQRFSRLVYDCNRPPESPSAMPVKSEIYDIPGNFDLDEAERFART
SALYVPFHDRVSEIIAERQAAGRKVVVVTIHSFTPVYHGRFREVEIGILHDNDSRLADAMLA
GAEGASLTVRRNDPYGPEDGVTHTLRLHALPDGLLNVMIEIRNDLIANEGEQAAIAGFLHE
LMGKALSSIEE
11111000000000000000000000000000000000000000000000000000000000000000000000000000000
00000000000000000000000000000000000000000000000000000000000000000000000000000000000
00000000000000000000000000000000000000000000000000000000000000000000000000000000000
00000001
>O67644_3vn5_0_256
MPSLKISPSEAEKIQNYLVSSGFRKINAPYTLWALEGNGVKVYYYKTGSLLIQGKNSEKVLKE
VLNLLEKKKLPGCDESGKGDIFGSLVLCCVCIPEENYLKVSSLNPRDTKRLSDKRVERLYLAL
KPLVKAYCYEIKPEEYNKLYRKFRNLNKMMTHFYKLLIERVKEECGVSEVVVDKYQPSNPF
GEDVIFETEAERNLAVAVASIFARYKFLQSLKEVERELGIKIPKGTSKEVKELAKSLKNPERFIK
LNFNV
10000000000000000000000000000000000000000000000000000000000000000000000000000000000
00000000000000000000000000000000000000000000000000000000000000000000000000000000000
00000000000000000000000000000000000000000000000000000000000000000000000000000000000
00000001
>O75909_2i53_10_266
SVTSANLDHTKPCWYWDKKDLAHTPSQLEGLDPATEARYRREGARFIFDVGTRLGLHYDT
LATGIIYFHRFYMFHSFKQFPRYVTGACCLFLAGKVEETPKKCKDIIKTARSLLNDVQFGQF
GDDPKEEVMVLERILLQTIKFDLQVEHPYQFLLKYAKQLKGDKNKIQKLVQMAWTFVNDS
LCTTLSLQWEPEIIAVAVMYLAGRLCKFEIQEWTSKPMYRRWWEQFVQDVPVDVLEDICHQ
ILDLYSQGKQQMPH
11100000000000000000000000000000000000000000000000000000000000000000000000000000000
00000000000000000000000000000000000000000000000000000000000000000000000000000000000
00000000000000000000000000000000000000000000000000000000000000000000000000000000000
00000000
>P16667_2reu_232_488
KEKSIEDIVFEKFQPYINWSIDKLCEHFSINKGEKGLNYRIASAILNLKGKTTKSKPFPEVEEFE
KSSIVVKTVHFNKKNVNKESMSFGAFKFEELANEEWEDSEGYPSAQWRNFLLETRFLFFVV
KEDEDGVDIFKGIKFFSMPEEDINGPVKRMWDDTVKKLKEGVTLEAVPDKSTKDGWRIKN
NFVDKSDDLICHVRPHTNNRDYRGGSNADKLPKKINWINRPDSDDYSDEWMTKQSFWIN
NDYIKKQVEDLL
11100000000000000000000000000000000000000000001111111111111111111000000000000000000
00000000000000000000000000000000000000000000000000000000000000000000000000000000000
00000000000000000000000000000000000000000000000000000000000000000000000000000000000
00000000
>P52704_3c6y_0_256
MAFAHFVLIHTICHGAWIWHKLKPLLEALGHKVTALDLAASGVDPRQIEEIGSFDEYSEPLL
TFLEALPPGEKVILVGESCGGLNIAIAADKYCEKIAAAVFHNSVLPDTEHCPSYVVDKLMEV

FPDWKDTTYFTYTKDGKEITGLKLGFTLLRENLYTLCGPEEYELAKMLTRKGSLFQNILAKR
PFFTKEGYGSIKKIYVWTDQDEIFLPEFQLWQIENYKPDKVYKVEGGDHKLQLTKTKEIAEIL
QEVADTYN
10000000000000000000000000000000000000000000000000000000000000000000000000000000000
00000000000000000000000000000000000000000000000000000000000000000000000000000000000
00000000000000000000000000000000000000000000000000000000000000000000000000000000000
00000000
>P84786_2b7u_0_256
SQCKAMTVKFTVELDIERLTGQTYTDFIKNLRRSLATWYLHGVPVLPLYNQEADPRGFDLK
LTFRGQVTTVRIHRDDLVLRGYQMQGAGKWLELERPSTQTGHLIEGSELLEFGPSYEELAA
AAQQDILDISYNKNALQDAVSKLAVSTNTRDRARSLIVVSQMFCEATRFVDIANHFAFNLE
SSEPVKLPQWMQNDLEKNWVRFSFMILKSNADPCYKFEPQTIYGKIIKTADELLNFLGIVEQ
HPDTRSPPCAAG
11100000000000000000000000000000000000000000000000000000000000000000000000000000000
00000000000000011100000000000000000000000000000000000000000000000000000000000000000
00000000000000000000000000000000000000000000000000000000000000000000000000000000000
00000000
>Q47112_2axc_59_315
SGHGNGGGNSNSGGGSNSSVAAPMAFGFPALAAPGAGTLGISVSGEALSAAIADIFAALKG
PFKFSAWGIALYGILPSEIAKDDPNMMSKIVTSLPAETVTNVQVSTLPLDQATVSVTKRVTDV
VKDTRQHIAVVAGVPMSVPVVNAKPTRTPGVFHASFPGVPSLTVSTVKGLPVSTTLPRGITE
DKGRTAVPAGFTFGGGSHEAVIRFPKESGQKPVYVSVTDVLTPAQVKQRQDEEKRLQQEW
NDAHPVEVAER
11111111111111100000000000000000000000000000000000000000000001111000000000000000000
00000000000000000000000000000000000000000000000000000000000000000000000000000000000
00000000000000000000000000000000000000000000000000000000000000000000000000000000000
00111111
>A6LIG9_3lmz_27_282
ALPDPVKPKAPKAVNPFHLGMAGYTFVNFDLDTTLKTLERLDIHYLCIKDFHLPLNSTDEQ
IRAFHDKCAAHKVTGYAVGPIYMKSEEEIDRAFDYAKRVGVKLIVGVPNYELLPYVDKKVK
EYDFHYAIHLHGPDIKTYPDATDVWVHTKDLDPRIGMCLDVGHDLRNGCDPVADLKKYH
TRVFDMHIKDVTDSSKAGVGIEIGRGKIDFPALIRMMREVNYTGMCSLEYEKDMKDPFLGI
AESIGYFKAVSDLT
11111000000000000000000000000000000000000000000000000000000000000000000000000000000
00000000000000000000000000000000000000000000000000000000000000000000000000000000000
00000000000000000000000000000000000000000000000000000000000000000000000000000000000
0000000
>D5SQH6_3rht_0_255
MTRVLYCGDTSLETAAGYLAGLMTSWQWEFDYIPSHVGLDVGELLAKQDLVILSDYPAER
MTAQAIDQLVTMVKAGCGLVMLGGWESYHGLGGNWDQTLLAEVLPVDIKSADDRINFD
QPTLAIPAAINSVSHPILQNLPWEDRPPTIGGLNRIAAKAKAQTLLMARVWRPTFSLEHGK
TTWEHADHHPLLVVGEAGTGRVAAFASDVAPHWVGGLVDWGDERVTSQAPGAGAIEVG
NLYSQFFRQMLEWVAKSTR
11000000000000000000000000000000000000000000000000000000000000000000000000000000000
00000000000000000000000000000000000000000000000000000000000000000000000000000000000
00000000000000000000000000000000000000000000000000000000000000000000000000000000000
0000011
>P03419_2ykd_0_255
METYVNKLHEGSTYTAAVQYNVLEKDDDPASLTIWVPMFQSSMPADLLIKELANVNILVK
QISTPKGPSLRVMINSRSAVLAQMPSKFTICANVSLDERSKLAYDVTTPCEIKACSLTCLKSK
NMLTTVKDLTMKTLNPTHDIIALCEFENIVTSKKVIIPTYLRSISVRNKDLNTLENITTTEFKN

AITNAKIIPYSGLLLVITVTDNKGAFKYIKPQSQFIVDLGAYLEKESIYYVTTNWKHTATRFAI
KPMED
00000000000000000000000000000000000000000000000000000000000000000000000000000000000
00000000000000010000000000000000000000000000000000000000000000000000000000000000000
00000000000000000000000000000000000000000000000000000000000000000000000000000000000
0000001
>P60477_4h8e_0_255
MFKKLINKKNTINNYNEELDSSNIPEHIAIIMDGNGRWAKKRKMPRIKGHYEGMQTIKKIT
RIASDIGVKYLTLYAFSTENWSRPESEVNYIMNLPVNFLKTFLPELIEKNVKVETIGFTDKLPK
STIEAINNAKEKTANNTGLKLIFAINYGGRAELVHSIKNMFDELHQQGLNSDIIDETYINNH
LMTKDYPDPELLIRTSGEQRISNFLIWQVSYSEFIFNQKLWPDFDEDELIKCIKIYQSRQRRFG
GLSEE
11111111111111111100000000000000000000000000000000000000000000000000000000000000000
00000000000000000000000000000000000000000000000000000000000000000000000000000000000
00000000000000000000000000000000000000000000000000000000000000000000000000000000000
0000111
>Q7WR29_3hft_0_255
MAGAAHRLMEARMRRYDNKFARISDIDINQPESWRGRIFLTFDIDWAADFVLQDTIDLIEG
AGVCATWFATHSTPLLENIRRNPLFELGVHPNFNPLLAGAHAEGVQEILDRTLELAPGCVS
VRSHSLVQATSILNMFGERRLRYDCNILVPWDAGIVLQPWRHWTGDMVRVPYLWEDDVA
CLYDWEFDSTFDYWYQPDGINVLDFHPIHVYMNTESLRRYEDSREVHRNPVDLIRWRNTS
AGSRTFLQSLLARNI
11111111111111000000000000000000000000000000000000000000000000000000000000000000000
00000000000000000000000000000000000000000000000000000000000000000000000000000000000
00000000000000000000000000000000000000000000000000000000000000000000000000000000000
0000000
>Q8UH15_2p35_0_255
MAWSAQQYLKFEDERTRPARDLLAQVPLERVLNGYDLGCGPGNSTELLTDRYGVNVITGI
DSDDDMLEKAADRLPNTNFGKADLATWKPAQKADLLYANAVFQWVPDHLAVLSQLMD
QLESGGVLAVQMPDNLQEPTHIAMHETADGGPWKDAFSGGGLRRKPLPPPSDYFNALSPK
SSRVDVWHTVYNHPMKDADSIVEWVKGTGLRPYLAAAGEENREAFLADYTRRIAAAYPP
MADGRLLLRFPRLFVVAVKK
11110000000000000000000000000000000000000000000000000000000000000000000000000000000
00000000000000000000000000000000000000000000000000000000000000000000001111110000000
00000000000000000000000000000000000000000000000000000000000000000000000000000000000
0000000
>Q8ZVK6_1xqp_0_255
MAAESQLKRVIETLRRLGIEEVLKLERRDPQYRAVCNVVKRHGETVGSRLAMLNALISYRLT
GKGEEHWEYFGKYFSQLEVIDLCRDFLKYIETSPFLKIGVEARKKRALKACDYVPNLEDLGL
TLRQLSHIVGARREQKTLVFTIKILNYAYMCSRGVNRVLPFDIPIPVDYRVARLTWCAGLIDF
PPEEALRRYEAVQKIWDAVARETGIPPLHLDTLLWLAGRAVLYGENLHGVPKEVIALFQWR
GGCRPPSE
11000000000000000000000000000000000000000000000000000000000000000000000000000000000
00000000000000000000000000000000000000000000000000000000000000000000000000000000000
00000000000000000000000000000000000000000000000000000000000000000000000000000000000
0000011
>Q9Q0U9_3hw3_0_255
MEDFVRQCFNPMIVELAEKAMKEYGEDPKIETNKFAAICTHLEVCFMYSDFHFIDERGESTI
IESGDPNALLKHRFEIIEGRDRTMAWTVVNSICNTTGVEKPKFLPDLYDYKENRFIEIGVTRR
EVHTYYLEKANKIKSEKTHIHIFSFTGEEMATKADYTLDEESRARIKTRLFTIRQEMASRGLW
DSFRQSERGEETVEERFEITGTMCRLADQSLPPNFSSLEKFRAYVDGFEPNGCIEGKLSQMSK

EVNAR
00000000000000000000000000000000000000000000000001111111111111111111111110000000000
00000000000000000000000000000000000000000000000000000000000000000000000000000000000
00000000000000000000000000000001111111111111111111111111111111111111111111111111111
1111111
>A6L7K2_3kog_27_281
SCEKENIGIEVTPVNAKFIITPVVIDATTGTDVTQSAEISFSKGNGTYEGTPELASESININAKY
KGMTGSASVTIPALKAGQFGAKEVTIILSENFFAQEESSNSQIETTKHSGFKNNTSDYWYYIT
VTYTKKEGSEVIKNDYEGDDSEIKNIIDAYNKGVREDKVTLNDVQVLAHSRFSVFVDYMKT
TSVYQIIEKSPKRDGNPVASFTVDSYNTIVSPKNEQIPGHGHAPSHGHGHGHGDDSNAGG
GIIIAD
11111111111000000000000000000000000000000000000000000000000000000000000000000000000
00000000000000000000000000000000000000000000000000000000000000000000000000000000000
00000000000000000000000000000000011000000000000000000000000000000000000001111111111
111111
>O31618_1xm3_0_254
MSMLTIGGKSFQSRLLLGTGKYPSFDIQKEAVAVSESDILTFAVRRMNIFEASQPNFLEQLDLS
KYTLLPNTAGASTAEEAVRIARLAKASGLCDMIKVEVIGCSRSLLPDPVETLKASEQLLEEGF
IVLPYTSDDVVLARKLEELGVHAIMPGASPIGSGQGILNPLNLSFIIEQAKVPVIVDAGIGSPK
DAAYAMELGADGVLLNTAVSGADDPVKMARAMKLAVEAGRLSYEAGRIPLKQYGTASSP
GEGLP
10000000000000000000000000000000000000000000000000000000000000000000000000000000000
00000000000000000000000000000000000000000000000000000000000000000000000000000000000
00000000000000000000000000000000000000000000000000000000000000000000000000000000000
000111
>P08254_1slm_17_271
YPLDGAARGEDTSMNLVQKYLENYYDLKKDVKQFVRRKDSGPVVKKIREMQKFLGLEVT
GKLDSDTLEVMRKPRCGVPDVGHFRTFPGIPKWRKTHLTYRIVNYTPDLPKDAVDSAVEKA
LKVWEEVTPLTFSRLYEGEADIMISFAVREHGDFYPFDGPGNVLAHAYAPGPGINGDAHFD
DDEQWTKDTTGTNLFLVAAHEIGHSLGLFHSANTEALMYPLYHSLTDLTRFRLSQDDINGI
QSLYGPPPDSPET
11111111111111100000000000000011111111100000000000000000000000000000000000000000000
00000000000000000000000000000000000000000000000000000000000000000000000000000000000
00000000000000000000000000000000000000000000000000000000000000000000000000000000000
011111
>P0AAI3_1lv7_140_394
MLTEDQIKTTFADVAGCDEAKEEVAELVEYLREPSRFQKLGGKIPKGVLMVGPPGTGKTLL
AKAIAGEAKVPFFTISGSDFVEMFVGVGASRVRDMFEQAKKAAPCIIFIDEIDAVGRQRGAG
LGGGHDEREQTLNQMLVEMDGFEGNEGIIVIAATNRPDVLDPALLRPGRFDRQVVVGLPD
VRGREQILKVHMRRVPLAPDIDAAIIARGTPGFSGADLANLVNEAALFAARGNKRVVSMV
EFEKAKDKIMMG
00000000000000000000000000000000000001111100000000000000000000000000000000000000000
00000000000000000000000000000000000000000000000000000000000000000000000000000000000
00000000000000000000000000000000000000000000000000000000000000000000000000000000000
000000
>P39621_1qgq_1_255
PKVSVIMTSYNKSDYVAKSISSILSQTFSDFELFIMDDNSNEETLNVIRPFLNDNRVRFYQSDIS
GVKERTEKTRYAALINQAIEMAEGEYITYATDDNIYMPDRLLKMVRELDTHPEKAVIYSASK
TYHLNENRDIVKETVRPAAQVTWNAPCAIDHCSVMHRYSVLEKVKEKFGSYWDESPAFYR
IGDARFFWRVNHFYPFYPLDEELDLNYITDQSIHFQLFELEKNEFVRNLPPQRNCRELRESL
KKLGMG

00000000000000000000000000000000000000000000000000000000000000000000000000000000
00000000000000000000000000000000000000000000000111000000000000000000000000000000
00000000000000000000000000000000000000000000000011111111111110000000000000000000
000000
>P95748_3pfg_0_254
MAHSSATAGPQADYSGEIAELYDLVHQGKGKDYHREAADLAALVRRHSPKAASLLDVAC
GTGMHLRHLADSFGTVEGLELSADMLAIARRRNPDAVLHHGDMRDFSLGRRFSAVTCMF
SSIGHLAGQAELDAALERFAAHVLPDGVVVVEPWWFPENFTPGYVAAGTVEAGGTTVTRV
SHSSREGEATRIEVHYLVAGPDRGITHHEESHRITLFTREQYERAFTAAGLSVEFMPGGPSGR
GLFTGLPGAKGETR
11111111100000000000000000000000000000000000000000000000000000000000000000000000
00000000000000000000000000000000000000000000000000000000000000000000000000000000
00000000000000000000000000000000000000000000000000000000000000000000000000000000
011111
>Q63ZY3_4hbd_577_831
SGSNTEEEIRMELSPDLISACLALEKYLDNPNALTERELKVAYTTVLQEWLRLACRSDAHPE
LVRRHLVTFRAMSARLLDYVVNIADSNGNTALHYSVSHANFPVVQQLLDSGVCKVDKQN
RAGYSPIMLTALATLKTQDDIETVLQLFRLGNINAKASQAGQTALMLAVSHGRVDVVKALL
ACEADVNVQDDDGSTALMCACEHGHKEIAGLLLAVPSCDISLTDRDGSTALMVALDAGQ
SEIASMLYSRMNIK
11111111110000000000000000000000000000000000000000000000000000000000000000000000
00000000000000000000000000000000000000000000000000000000000000000000000000000000
00000000000000000000000000000000000000000000000000000000000000000000000000000000
000000
>A9CI98_3dnh_0_253
MLDVAPPVITPRGTKIEPSAGAPFEAVRVARDVLHTSRTAALATLDPVSGYPYTTATNIGIEP
DGTPFFFAAGLTLHARNMETDARISVTLAPFGKGDALTLPRLTLVGRADRIGPDEVPLAIAR
YIARYPKAKLYLSLPDTRLYRLRTEGVQINGGPARNASNITPADLRTDLSGAEELMAAAESE
ATRLNAIKGEASRLAVLAGAKTGRWKITSIDPDGIDLASASDLARLWFAERVETLKQFEKAL
AQLLK
11100000000011111110000000000000000000000000000000000000000000000000000000000000
00000000000000000000000000000000000000000000000000000000000000000000001111110000
00000000000000000000000000000000000000000000000000000000000000000000000000000000
00000
>P27797_3pow_17_367
EPAVYFKEQFLDGDGWTSRWIESKHKSDFGKFVLSSGKFYGDEEKDKGLQTSQDARFYALS
ASFEPFSNKGQTLVVQFTVKHEQNIDCGGGYVKLFPNSLDQTDMHGDSEYNIMFGPDICG
PGTKKVHVIFNYKGKNVLINKDIRCKDDEFTHLYTLIVRPDNTYEVKIDNSQVESGSLEDD
WDFLPDPSIYAYDNFGVLGLDLWQVKSGTIFDNFLITNDEAYAEEFGNETWGVTKAAEKQ
MKDKQDEEQRLK
00000000000000000000000000000000000000000000000000000000000000000000000000000000
00000000000000000000000000000000000000000000000000000000000000000000000000000000
00000000000000000011110000000000000000000000000000000000000000000000000000000000
00001
>P43555_2a6y_28_281
HPLGDTSDASKLSSDYSLPDLINARKVPNNWQTGEQASLEEGRIVLTSKQNSKGSLWLKQG
FDLKDSFTMEWTFRSVGYSGQTDGGISFWFVQDSNVPRDKQLYNGPVNYDGLQLLVDNN
GPLGPTLRGQLNDGQKPVDKTKIYDQSFASCLMGYQDSSVPSTIRVTYDLEDDNLLKVQVD
NKVCFQTRKVRFPSGSYRIGVTAQNGAVNNNAESFEIFKMQFFNGVIEDSLIPNVNAMGQP
KLITKYIDQQTG
11111110000000000000000000000000000000000000000000000000000000000000000000000000

00000000000000000000000000000000000000000000000000000000000000000000000000000000
00000000000000000000000000000000000000000000000000000000000000011111111110000000
11111
>P53676_4ikn_164_417
IPWRRAGVKYTNNEAYFDVVEEIDAIIDKSGSTVFAEIQGVIDACIKLSGMPDLSLSFMNPRL
LDDVSFHPCIRFKRWESERVLSFIPPDGNFRLISYRVSSQNLVAIPVYVKHNISFKENSSCGRF
DITIGPKQNMGKTIEGITVTVHMPKVVLNMNLTPTQGSYTFDPVTKVLAWDVGKITPQKLP
SLKGLVNLQSGAPKPEENPNLNIQFKIQQLAISGLKVNRLDMYGEKYKPFKGVKYITKAGK
FQVRT
00000000000000000000000000000000000000000000000000000000000000000000000000000000
00000000000000000000000000000001110000000000000000000000000000000000000000000000
00000000000000000000000000000001110000000000000000000000000000000000000000000000
00000
>P96717_3qy7_0_253
MIDIHCHILPAMDDGAGDSADSIEMARAAVRQGIRTIIATPHHNNGVYKNEPAAVREAAD
QLNKRLIKEDIPLHVLPGQEIRIYGEVEQDLAKRQLLSLNDTKYILIEFPFDHVPRYAEQLFYD
LQLKGYIPVIAHPERNREIRENPSLLYHLVEKGAASQITSGSLAGIFGKQLKAFSLRLVEANLI
HFVASDAHNVKTRNFHTQEALYVLEKEFGSELPYMLTENAELLLRNQTIFRQPPQPVKRRK
LFGFF
00000000000000000000000000000000000000000000000000000000000000000000000000000000
00000000000000000000000000000000000000000000000000000000000000000000000000000000
00000000000000000000000000000000000000000000000000000000000000000000000000000011
11111
>Q09065_3cxn_0_253
MDKGKSVKSTEKSVGMPPKTPKTDNNAHVDNEFLILQVNDAVFPIGSYTHSFGLETYIQQK
KVTNKESALEYLKANLSSQFLYTEMLSLKLTYESALQQDLKKILGVEEVIMLSTSPMELRLA
NQKLGNRFIKTLQAMNELDMGEFFNAYAQKTKDPTHATSYGVFAASLGIELKKALRHYLY
AQTSNMVINCVKSVPLSQNDGQKILLSLQSPFNQLIEKTLELDESHLCTASVQNDIKAMQH
ESLYSRLYMS
11111111111111111111111100000000000000000000000000000000000000000000000000000000
00000000000000000000000000000000000000000000000000000000000000000000000000000000
00000000000000000000000000000000000000000000000000000000000000001111111111111111
11111
>Q2IE03_4hg2_0_253
MAAFKDHFTPVADAYRAFRPRYPRALFRWLGEVAPARGDALDCGCGSGQASLGLAEFFER
VHAVDPGEAQIRQALRHPRVTYAVAPAEDTGLPPASVDVAIAAQAMHWFDLDRFWAELR
RVARPGAVFAAVTYGLTRVDPEVDAVVDRLYHGLLARDWPPERVHVESGYRTLPFPFPELE
APPLEIEERWPMDAFLGYLGTWSAVTAHRRRTGADPLAEIAPALRAAWGTPERPLRVTWPI
AIRAGRILPHAGG
11111111111111111110000000000000000000000000000000000000000000000000000000000000
00000000000000000000000000000000000000000000000000000000000000000000000000000000
00000000000000000000000000000000000000000000000000000000000000000000000000000000
00011
>Q53W91_3ayv_0_253
MDVRLAFPLSRAEEALPRLQALGLGAEVYLDPALLEEDALFQSLRRRFSGKLSVHLPFWNL
DLLSPDPEVRGLTLRRLLFGLDRAAELGADRAVFHSGIPHGRTPEEALERALPLAEALGLVV
RRARTLGVRLLLENSHEPHPEALRPVLEAHAGELGFCFDAAHARVFSRTPDPGPWLALAP
EHLHLNDTDGVYDRHWNLGRGVLGHGAWLRPYLDRTMVLEVREDPEASLAFLQALAGE
GRTALDRLLMGER
00000000000000000000000000000000000000000000000000000000000000000000000000000000
00000000000000000000000000000000000000000000000000000000000000000000000000000000

00000000000000000000000000000000000000000000000000000000000000000000000000000111111
11111
>Q56991_3md9_25_278
AERIVTIGGDVTEIAYALGAGDEIVARDSTSQQPQAAQKLPDVGYMRTLNAEGILAMKPTM
LLVSELAQPSLVLTQIASSGVNVVTVPGQTTPESVAMKINAVATALHQTEKGQKLIEDYQQR
LAAVNKTPLPVKVLFVMSHGGLTPMAAGQNTAADAMIRAAGGSNAMQGFSRYRPLSQE
GVIASAPDLLLITTDGVKALGSSENIWKLPGMALTPAGKHKRLLVVDDMALLGFGLETPQV
LAQLREKMEQMQ
00000000000000000000000000000000000000000000000000000000000000000000000000000000000
00000000000000000000000000000000000000000000000000000000000000000000000000000000000
00000000000000000000000000000000000000000000000000000000000000000000000000000000000
00001
>Q5ZXQ9_3c8w_0_253
MDKYLSANSLEGVIDNEFSMPAPRWLNTYPAGPYRFINREFFIIAYETDPDLLQAILPPDMEL
LEPVVKFEFIRMPDSTGFGDYTESGQVVPVRYKGEEGGFTISMFLDCHAPIAGGREIWGFPK
KLAKPKLFVEEDTLIGILKYGSIDIAIATMGYKHRPLDAEKVLESVKKPVFLLKNIPNVDGTP
LVNQLTKTYLTDITVKGAWTGPGSLELHPHALAPISNLYIKKIVSVSHFITDLTLPYGKVVAD
YLA
11110000000000000000000000000000000000000000000000000000000000000000000000000000000
00000000000000000000000000000000000000000000000000000000000000000000000000000000000
00000000000000000000000000000000000000000000000000000000000000000000000000000000000
00000
>Q7MW92_2i6d_0_253
MLSANQIKFLRSLRERKYRLREQAFAVEGPKLVGEMLPFYRCRMLVGTAAMLRAVSTPHDA
EVVELPESFDFKRISTQTTPQPLMAVFDLPAEPEPVVEGLTLLLDGVQDPGNVGTILRTADW
FGIRHVWLGTGSADVFSPKVVQASMGALARVQPTPLKNTVDTLAYFRRQGIPVYGAFLDG
QSLYEAPLPNFTEPAILVLGSEGRGISPEVAAEITDRLTIPASGLSVKGHTESLNVAIATAILCSE
WRRRS
00000000000000000000000000000000000000000000000000000000000000000000000000000000000
00000000000000000000000000000000000000000000000000000000000000000000000000000000000
00000000000000000000000000000000000000000000000000000000000000001111000000000000000
00000
>Q81L49_1rz2_0_253
MSSEKERKKKIFFQRILTVVFLGTFFYSVYELGDIFMDYYENRKVMAEAQNIYEKSPMEEQS
QDGEVRKQFKALQQINQEIVGWITMDDTQINYPIVQAKDNDYYLFRNYKGEDMRAGSIF
MDYRNDVKSQNRNTILYGHRMKDGSMFGSLKKMLDEEFFMSHRKLYYDTLFEGYDLEVFS
VYTTTTDFYYIETDFSSDTEYTSFLEKIQEKSLYKTDTTVTAGDQIVTLSTCDYALDPEAGRLV
VHAKLVKRQ
11111111111111111111111111111111110000000000000000000000000000000000000000000000000
00000000000000000000000000000000000000000000000000000000000000000000000000000000000
00000000000000000000000000000000000000000000000000000000000000000000111111000000000
00000
>Q8G9L3_2boy_0_253
MSTDRTGNIVGKMIAAINAVIKDEKVSYSEYKASTGWLISVGEQNEWPLFLDVFFEHAIESV
AAESNRGSQSSIQGPYFIPGAPELSIPYTMPMRDDESGDTLIFRGEVVDQEGAPLADVLLDM
WQADAAGEYSFINPTLPDYLFRGKIRTDENGRFTLRTIVPAPYEIPKNGPTGALLAAAGWH
AWRPAHLHWIIAKEGYESLTTQLYFENGQWTGSDVANAVKPELLLSLDKIEAQSGPHFETS
YKFTLGKV
10000000000000000000000000000000000000000000000000000000000000000000000000000000000
00000000000000000000000000000000000000000000000000000000000000000000000000000000000
00000000000000000000000000000000000000000000000000000000000000000000000110000000000

00000
>Q8KYA6_3p8a_0_253
MILKFDHIIHYIDQLDRFSFPGDVIKLHSGGYHHKYGTFNKLGYINENYIELLDVENNEKLK
KMAKTIEGGVAFATQIVQEKYEQGFKNICLHTNDIEAVKNKLQSEQVEVVGPIQMERDTHK
DGKVKWQLLYIMNQDDDEIKPPFFIQWEESDSMRTKKLQKYFQKQFSIETVIVKSKNRSQT
VSNWLKWFDMDIVEENDHYTDLILKNDDIYFRIEDGKVSKYHSVIIKDAQATSPYSIFIRGAI
YRFEPLV
00000000000000000000000000000000000000000000000000000000000000000000000000000000000
00000000000000000000000000000000000000000000000000000000000000000000000000000000000
00000000000000000000000000000000000000000000000000000000000000000000000000000000000
00001
>Q8SU65_2vyo_0_253
MLLCLLYFTSSWCSGSDVPDVCTNSGMIAINFVDGPVRGVTDRILNTLDELGVKATFSFTVN
QKAVGNVGQLYRRAVEEGHNVALRVDPSMDEGYQCLSQDALENNVDREIDTIDGLSGTEI
RYAAVPICNGQVNSEMYNILTERGVLPVGYTFCPYDYDDPVGEFESMIEGSDPKHHSFIILM
HDGQEADTSRLENMVKIGKDKGYRFVNMDECLQGYKGAPGDPELSLRGKGVESIGKGFLP
FFLMMLVRLL
11111111111111000000000000000000000000000000000000000000000000000000000000000000000
00000000000000000000000000000000000000000000000000000000000000000000000000000000000
00000000000000000000000000000000000000000000000000000011111111111111111111111111111
11111
>Q927R6_3ds8_37_290
KDQIPIILIHGSGGNASSLDKMADQLMNEYRSSNEALTMTVNSEGKIKFEGKLTKDAKRPII
KFGFEQNQATPDDWSKWLKIAMEDLKSRYGFTQMDGVGHSNGGLALTYYAEDYAGDKT
VPTLRKLVAIGSPFNDLDPNDNGMDLSFKKLPNSTPQMDYFIKNQTEVSPDLEVLAIAGELS
EDNPTDGIVPTISSLATRLFMPGSAKAYIEDIQVGEDAVHQTLHETPKSIEKTYWFLEKFKTD
ETVIQLDYK
10000000000000000000000000000000000000000000000000000000000000000000000000000000000
00000000000000000000000000000000000000000000000000000000000000000000000000000000000
00000000000000000000000000000000000000000000000000000000000000000000000000000000000
00000
>Q9KHL8_4f78_0_253
MMKTIELEKEEIYCGNLLLVNKNYPLRDNNVKGLVPADIRFPNILMKRDVANVLQLIFEKIS
AGNSIVPVSGYRSLEEQTAIYDGSLKDNGEDFTRKYVALPNHSEHQTGLAIDLGLNKKDIDF
IRPDFPYDGICDEFRRAAPDYGFTQRYARDKEEITGISHEPWHFRYVGYPHSKIMQENGFSLE
EYTQFIKAYLEDNKYLFEQAHRAEIEIYYVPAKDDKTLIKIPENCVYQISGNNIDGFVVTIWR
KTDD
00000000000000000000000000000000000000000000000000000000000000000000000000000000000
00000000000000000000000000000000000000000000000000000000000000000000000000000000000
00000000000000000000000000000000000000000000000000000000000000000000000000000000000
01111
>G9W5C4_4dz1_0_252
MLSKKFGLSMIVLGIMSSSAFADSIVEGRTLNVAVSPASPPMLFKSADGKLQGIDLELFSSYC
QSRHCKLNITEYAWDGMLGAVASGQADVAFSGISITDKRKKVIDFSEPYYINSFYLVSMANH
KITLNNLNELNKYSIGYPRGMAYSDLIKNDLEPKGYYSLSKVKLYPTYNETMADLKNGNLD
LAFIEEPVYFTFKNKKKMPIESRYVFKNVDQLGIAFKKGSPVRDDFNLWLKEQGPQKISGIV
DSWMK
11111111111111111111111100000000000000000000000000000000000000000000000000000000000
00000000000000000000000000000000000000000000000000000000000000000000000000000000000
00000000000000000000000000000000000000000000000000000000000000000000000000000000000
0000

>O00294_2fim_289_541
EPREFVLRPAPQGRTVRCRLTRDKKGMDRGMYPSYFLHLDTEKKVFLLAGRKRKRSKTAN
YLISIDPTNLSRGGENFIGKLRSNLLGNRFTVFDNGQNPQRGYSTNVASLRQELAAVIYETN
VLGFRGPRRMTVIIPGMSAENERVPIRPRNASDGLLVRWQNKTLESLIELHNKPPVWNDDS
GSYTLNFQGRVTQASVKNFQIVHADDPDYIVLQFGRVAEDAFTLDYRYPLCALQAFAIALSS
FDGKLACE
10000000000000000000000000111110000000000000000000000000000000001111110000000000000
00000000000000000000000000000000000000000000000000000000000000000000000000000000000
00000000000000000000000000000000000000000000000000000000000000000000000000000000011
1111
>Q60099_1aq6_0_252
MIKAVVFDAYGTLFDVQSVADATERAYPGRGEYITQVWRQKQLEYSWLRALMGRYADFW
GVTREALAYTLGTLGLEPDESFLADMAQAYNRLTPYPDAAQCLAELAPLKRAILSNGAPD
MLQALVANAGLTDSFDAVISVDAKRVFKPHPDSYALVEEVLGVTPAEVLFVSSNGFDVGGA
KNFGFSVARVARLSQEALARELVSGTIAPLTMFKALRMREETYAEAPDFVVPALGDLPRLVR
GMAGAHLAPAV
00000000000000000000000000000000000000000000000000000000000000000000000000000000000
00000000000000000000000000000000000000000000000000000000000000000000000000000000000
00000000000000000000000000000000000000000000000000000000000000000000000000000001111
1111
>Q9WXQ8_3a1s_16_268
VKVALAGCPNVGKTSLFNALTGTKQYVANWPGVTVEKKEGVFTYKGYTINLIDLPGTYSLG
YSSIDEKIARDYLLKGDADLVILVADSVNPEQSLYLLLEILEMEKKVILAMTAIDEAKKTGMK
IDRYELQKHLGIPVVFTSSVTGEGLEELKEKIVEYAQKNTILHRMILDYGEKVESEIKKVENFL
RDKKLRINPRYFALKYLSGDPEFYSEGVKLGLPELSEEERIGYRLLIAKRKREYVENVVKEAF
AD
00000000000000000000000000000000000000000000000000000000000000000000000000000000000
00000000000000000000000000000000000000000000000000000000000000000000000000000000000
00000000000000000000000000000000000000000000000000000000000000000000000000000000000
0001
>G0UVW6_4e40_113_364
AEGEIKVELKDADEVAAACELRAQLAGVSIASGILLRPAVIRNATTEFSRKKSEEILAKGGAA
AERASAAVNRVSGLDKANETAQKVRKAAAVAHHALEHVKEEVEIVAKKANEITELTAGAT
EHAKGAKANGDASVVKVSNLLARAKESEDQYVKKAAEECSNSTNYDVTAKSLAAALDKL
PGVKEDNAVKTTFQSILTSLDNLDKDVKSVEQRAEELETALEKAERQLEKAEKAAEEAETES
SKVETESS
11000000000000000000000000000000000000000000000000000000000000000000000000000000000
00000000000000000000000000000000000000000000000000000000000000000000000000000000000
00000000000000000000000000000000000000000000000000000000000000000000000000000000011
111
>O59000_1wzn_0_251
MYELYTLLAEYYDTIYRRRIERVKAEIDFVEEIFKEDAKREVRRVLDLACGTGIPTLELAERGY
EVVGLDLHEEMLRVARRKAKERNLKIEFLQGDVLEIAFKNEFDAVTMFFSTIMYFDEEDLRK
LFSKVAEALKPGGVFITDFPCWFYGGRDGPVVWNEQKGEEKLVIMDWREVEPAVQKLRFK
RLVQILRPNGEVKAFLVDDELNIYTPREVRLLAEKYFEKVKIYGNLKRELSPNDMRYWIVGI
AKSF
00000000000000000000000000000000000000000000000000000000000000000000000000000000000
00000000000000000000000000000000000000000000000000000000000000001111111000000000000
00000000000000000000000000000000000000000000000000000000000000000000000000000000000
001
>P16692_3p2u_0_251

MSLTLTLTGTGGAQGVPAWGCECAACARARRSPQYRRQPCSGVVKFNDAITLIDAGLHDL
ADRWSPGSFQQFLLTHYHMDHVQGLFPLRWGVGDPIPVYGPPDEQGCDDLFKHPGLLDF
SHTVEPFVVFDLQGLQVTPLPLNHSKLTFGYLLETAHSRVAWLSDTAGLPEKTLKFLRNNQP
QVMVMDCSHPPRADAPRNHCDLNTVLALNQVIRSPRVILTHISHQFDAWLMENALPSGFE
VGFDGMEIGVA
10000000000000000000000000000000000000000000000000000000000000000000000000000000000
00000000000000000000000000000000000000000000000000000000000000000000000000000000000
00000000000000000000000000000000000000000000000000000000000000000000000000000000000
001
>Q8TZE5_3v68_0_251
MIERILEFTAKHEEWIVGENVEDFTNENIAMFLSRVSNTVSSKIPGYLGEKIDVNGLLSIKIEG
SLEEKLKALISPKVSRQIGRLVMEDDKKLKKLLVEVAKAVLTREILKNELPIEFPGGKIEGLKI
QPRYEEDHINFTARYGSWIVVKRMIIDEKTPLLDIARLLASINETAVNKIKDFADVDDKKIVE
YFGGFKKVKKEEEIKEIVQLFREFKGNEFEVRYAAREMLSKLGLKVDVPSKNLEKYLEKAG
10000000000000000000000000000000000000000000000000000000000000000000000000000000000
00000000000000000000000000000000000000000000000000000000000000000000000000000000000
00000000000000000000000000000000000000000000000000000000000000000000000000000000000
111
>Q96T66_1nuu_0_251
MKSRIPVVLLACGSFNPITNMHLRMFEVARDHLHQTGMYQVIQGIISPVNDTYGKKDLAA
SHHRVAMARLALQTSDWIRVDPWESEQAQWMETVKVLRHHHSKLLRSPPQMEGPDHGK
ALFSTPAAVPELKLLCGADVLKTFQTPNLWKDAHIQEIVEKFGLVCVGRVGHDPKGYIAESP
ILRMHQHNIHLAKEPVQNEISATYIRRALGQGQSVKYLIPDAVITYIKDHGLYTKGSTWKGK
STQSTEGKTS
11000000000000000000000000000000000000000000000000000000000000000000000000000000000
00000000000000000000000111111111111111111000000000000000000000000000000000000000000
00000000000000000000000000000000000000000000000000000000000000000000011111111111111
111
>Q9A0M1_2xgr_0_251
MKLSKQKASLLTAVLLLLSLSITTITVDAARVRTYPNVSHANTHYKNTVSSKLLPFTANYQL
QLGELDNLNRATFSHIQLQDRHETKDVRTKINYDPVGWHNYQFPYGDGSKSSWVMNRG
HLVGYQFCGLNDEPRNLVAMTAWLNTGAYSGANDSNPEGMLYYENRLDSWLALHPDFW
LDYKVTPIYSGNEVVPRQIELQYVGIDSSGELLTIRLNSNKESIDENGVTTVILENSAPNINLD
YLNGTATPKN
11111111111111111111111111111111000000000000000000000000000000000000000000000000000
00000000001111111111111111111110000000000000000000000000000000000000000000000000000
00000000000000000000000000000000000000000000000000000000000000000000000000000000000
011
>Q9P2W7_1v83_82_333
LPTIHVVTPTYSRPVQKAELTRMANTLLHVPNLHWLVVEDAPRRTPLTARLLRDTGLNYTH
LHVETPRNYKLRGDARDPRIPRGTMQRNLALRWLRETFPRNSSQPGVVYFADDDNTYSLE
LFEEMRSTRRVSVWPVAFVGGLRYEAPRVNGAGKVVGWKTVFDPHRPFAIDMAGFAVNL
RLILQRSQAYFKLRGVKGGYQESSLLRELVTLNDLEPKAANCTKILVWHTRTEKPVLVNEGK
KGFTDPSVEI
00000000000000000000000000000000000000000000000000000000000000000000000011111110000
00000000000000000000000000000000000000000000000000000000000000000000000000000000000
00000000000000000000000000000000000000000000000000000000000000000000000000000000000
000
>Q9Y5P4_2e3r_346_623
LHWPTSLPSGDAFSSVGTHRFVQKVEEMVQNHMTYSLQDVGGDANWQLVVEEGEMKVY
RREVEENGIVLDPLKATHAVKGVTGHEVCNYFWNVDVRNDWETTIENFHVVETLADNAII

IYQTHKRVWPASQRDVLYLSVIRKIPALTENDPETWIVCNFSVDHDSAPLNNRCVRAKINV
AMICQTLVSPPEGNQEISRDNILCKITYVANVNPGGWAPASVLRAVAKREYPKFLKRFTSYV
QEKTAGKPILF
11111111111111111000000000000000000000001000000000000000000000000000000000000000000
00000000000000000000000000000000000000000000000000000000000000000000000000000000000
00000000000000000000001100000000000000000000000000000000000000000000000000000000000
000
>A6T9H1_3hlx_0_250
MLITDTLSPQAFEEALRAKGDFYHIHHPYHIAMHNGNATREQIQGWVANRFYYQTTIPLK
DAAIMANCPDAQTRRKWVQRILDHDGSHGEDGGIEAWLRLGEAVGLSRDDLLSERHVLP
GVRFAVDAYLNFARRACWQEAACSSLTELFAPQIHQSRLDSWPQHYPWIKEEGYFYFRSRLS
QANRDVEHGLALAKAYCDSAEKQNRMLEILQFKLDILWSMLDAMTMAYALQRPPYHTVT
DKAAWHTTRLV
10000000000000000000000000000000000000000000000000000000000000000000000000000000000
00000000000000000000000000000000000000000000000000000000000000000000000000000000000
00000000000000000000000000000000000000000000000000000000000000000000000000000000000
00
>A7B4J8_3nyy_28_278
VSGFQRLQKPVVSQPDFRRQPVSETMQVYLKQAADPGRDVGLYWMATDFENRRFPGKVS
PSGFQKLYRQWRNQTGWDAYVQSCRAIWNDVKYFPIPQSLDDTEDKISYVDSWMFERNYG
GKRGHEGTDIMAEKNTPGYYPVVSMTDGVVTEKGWLEKGGWRIGITAPTGAYFYYAHLDS
YAELEKGDPVKAGDLLGYMGDSGYGEEGTTGEFPVHLHLGIYLKEGTEEISVNPYPVLRYA
ENARIKCVYSR
00000000000000000000000000000000000000000000000000000000000000000000000000000000000
00000000000000000000000000000000000000000000000000000000000000000000000000000000000
00000000000000000000000000000000000000000000000000000000000000000000000000000000000
01
>C0LU01_3gas_0_250
MLNRIIEHMNAHHVEDMKGLLKKFGQVHHAENVAFKSVDSQGIVIGYNNNQTLRIEFNH
EVKDPKDYKNATIELCQSVEKTHDLKGVEEEVKAFKEGFDSVCLATLHPNGHVVCSYAPL
MSDGKQYYIYVSEVAEHFAGLKNNPHNVEVMFLEDESKAKSAILRKRLRYKTNTRFIERGA
EFDKAFDSFIEKTGGAGGIKTIRAMQDFHLIALDFKEGRFVKGFGQAYDILGDKIAYVGDKG
NPHNFAHKK
10000000000000000000000000000000000000000000000000000000000000000000000000000000000
00000000000000000000000000000000000000000000000000000000000000000000000000000000000
00000000000000000000000000000000000000000000000000000000000000000000000000000000000
11
>O23680_3def_0_250
MGSLVREWVGFQQFPAATQEKLIEFFGKLKQKDMNSMTVLVLGKGGVGKSSTVNSLIGEQ
VVRVSPFQAEGLRPVMVSRTMGGFTINIIDTPGLVEAGYVNHQALELIKGFLVNRTIDVLLY
VDRLDVYRVDELDKQVVIAITQTFGKEIWCKTLLVLTHAQFSPPDELSYETFSSKRSDSLLKTI
RAGSKMRKQEFEDSAIAVVYAENSGRCSKNDKDEKALPNGEAWIPNLVKAITDVATNQRK
AIHVD
11111000000000000000000000000000000000000000000000000000000000000000010000000000000
00000000000000000000000000000000000000000000000000000000000000000000000000000000000
00000000000000000000000000000000000000000000000000000000000000000000000000000000000
01
>P13661_3isg_25_275
STDISTVASPLFEGTEGCFLLYDASTNAEIAQFNKAKCATQMAPDSTFKIALSLMAFDAEIID
QKTIFKWDKTPKGMEIWNSNHTPKTWMQFSVVWVSQEITQKIGLNKIKNYLKDFDYGNQ
DFSGDKERNNGLTEAWLESSLKISPEEQIQFLRKIINHNLPVKNSAIENTIENMYLQDLDNST

KLYGKTGAGFTANRTLQNGWFEGFIISKSGHKYVFVSALTGNLGSNLTSSIKAKKNAITILNT
LNL
00000000000000000000000000000000000000000000000000000000000000000000000000000000000
00000000000000000000000000000000000000000000110000000000000000000000000000000000000
00000000000000000000000000000000000000000000000000000000000000000000000000000000000
00
>Q5YXQ1_2xed_0_250
MGIRRIGLVVPSSNVTVETEMPALLSRHPGAEFSFHSTRMRMHTVSPEGLAAMNAQRERCV
LEIADAAPEVILYACLVAVMVGGPGEHHRVESAVAEQLATGGSQALVRSSAGALVEGLRAL
DAQRVALVTPYMRPLAEKVVAYLEAEGFTISDWRALEVADNTEVGCIPGEQVMAAARSLD
LSEVDALVISCCVQMPSLPLVETAEREFGIPVLSAATAGAYSILRSLDLPVAVPGAGRLLRQDS
AVTAS
00000000000000000000000000000000000000000000000000000000000000000000000000000000000
00000000000000000000000000000000000000000000000000000000000000000000000000000000000
00000000000000000000000000000000000000000000000000000000000000000000000000000000111
11
>Q763K9_3fri_0_250
MNINDALTSILASKKYRALCPDTVRRILTEEWGRHKSPKQTVEAARTRLHGICGAYVTPESL
KAAAAALSAGDVKKALSLHASTKERLAELDTLYDFIFSAETPRRVLDIACGLNPLALYERGI
ASVWGCDIHQGLGDVITPFAREKDWDFTFALQDVLCAPPAEAGDLALIFKLLPLLEREQAG
SAMALLQSLNTPRMAVSFPTRSLGGRGKGMEANYAAWFEGGLPAEFEIEDKKTIGTELIYLI
KKNG
10000000000000000000000000000000000000000000000000000001000000000000000000000000000
00000000000000000000000000000000000000000000000000000000000000000000000000000000000
00000000000000000000000000000000000000000111111110000000000000000000000000000000000
00
>Q8DEG9_4g54_333_583
VPAILVNDEQWLGESAVARMTTRAQGLEELYKLWGYRATRIDTLCEQPSTSIFQCQVRQLN
WQELQQTPHPLLLTLQHEGQRAYVVLLEVNSERVVLLTGEQRLTFTVPQLMSLWRGEVTDL
WPMPLRETLRLGMHGEAIEVLDQLLAKALNDEPLMTTQFNAELMQRVEWFQRWQAMTE
DGIAGQRTLARLQHMVSLSEPWRELSQAEKQGEEQVMRYPEFPSLAPLLRTYPLAETGDVI
NVADQTSPTL
11111111110000000000000000000000000000000000000000000000000000000000000000000000000
00000000000000000000000000000000000000000000000000000000000000000000000000000000000
00000000000000000000000000000000000000000111110000000000000000000000000111111111111
11
>Q9Y5S1_2f37_68_318
PDPNRFDRDRLFNAVSRGVPEDLAGLPEYLSKTSKYLTDSEYTEGSTGKTCLMKAVLNLKD
GVNACILPLLQIDRDSGNPQPLVNAQCTDDYYRGHSALHIAIEKRSLQCVKLLVENGANV
HARACGRFFQKGQGTCFYFGELPLSLAACTKQWDVVSYLLENPHQPASLQATDSQGNTVL
HALVMISDNSAENIALVTSMYDGLLQAGARLCPTVQLEDIRNLQDLTPLKLAAKEGKIEIFR
HILQREFS
10000000000000000000000000000000000000000000000000000000000000000000000000000000000
00000000000000000000000000000000000000000000000000000000000000000000000000000000000
00000000000000000000000000000000000000000000000000000000000000000000000000000000000
01
>A9CHD2_2qv5_148_397
GQLPVVGADGLRPMEQYARPWSGARGTRVAIVVGGLGLSQTGSQKAIRDLPPEVTLGFAA
SGNSLQRWMQDARREGHEILLQIPLEPFGYPGTNPGPDTLLAGDPAKVNIDRLHRSMAKIT
NYTGVMNYLGGRFLAEQSALEPVMRDIGKRGLLFLDDGSSAQSLSGGIAKAISAPQGFADV
LLDGEVTEASILRKLDDLERIARRNGQAIGVASAFDESIAAISKWSREAGGRGIEIVGVSALVS

GQAG
00000000000000000000000000000000000000000000000000000000000000000000000000000000000
00000000000000000000000000000000000000000000000000000000000000000000000000000000000
00000000000000000000000000000000000000000000000000000000000000000000000000000001111
1
>P38446_1zm8_24_273
QVPPLTELSPSISVHLLLGNPSGATPTKLTPDNYLMVKNQYALSYNNSKGTANWVAWQLNS
SWLGNAERQDNFRPDKTLPAGWVRVTPSMYSGSGYDRGHIAPSADRTKTTEDNAATFLM
TNMMPQTPDNNRNTWGNLEDYCRELVSQGKELYIVAGPNGSLGKPLKGKVTVPKSTWKI
VVVLDSPGSGLEGITANTRVIAVNIPNDPELNNDWRAYKVSVDELESLTGYDFLSNVSPNIQ
TSIESKVDN
11111111111000000000000000000000000000000000000000000000000000000000000000000000000
00000000000000000000000000000000000000000000000000000000000000000000000000000000000
00000000000000000000000000000000000000000000000000000000000000000000000000000000000
0
>P72077_2r6z_0_249
MTDILIDDTATEAVRTLIRAFPLVPVSQPPEQGSYLLAEHDTVSLRLVGEKSNVIVDFTSGAA
QYRRTKGGGELIAKAVNHTAHPTVWDATAGLGRDSFVLASLGLTVTAFEQHPAVACLLSD
GIRRALLNPETQDTAARINLHFGNAAEQMPALVKTQGKPDIVYLDPMYPERRKSAAVKKE
MAYFHRLVGEAQDEVVLLHTARQTAKKRVVVKRPRLGEHLAGQAPAYQYTGKSTRFDVYL
PYGADKG
10000000000000000000000000000000000000000000000000000000000000000000000000000000000
00000000000000000000000000000000000000000000000000000000000000000000000000000000000
00000011111111111000000000000000000000000000000000000000000000000000000000000000000
0
>Q0PBL7_3fjm_0_249
MKKIITLFGACALAFSMANADVNLYGPGGPHTALKDIANKYSEKTGVKVNVNFGPQATW
FEKAKKDADILFGASDQSALAIASDFGKDFNVSKIKPLYFREAIILTQKGNPLKIKGLKDLAN
KKVRIVVPEGAGKSNTSGTGVWEDMIGRTQDIKTIQNFRNNIVAFVPNSGSARKLFAQDQA
DAWITWIDWSKSNPDIGTAVAIEKDLVVYRTFNVIAKEGASKETQDFIAYLSSKEAKEIFKKY
GWRE
11111111111111111111000000000000000000000000000000000000000000000000000000000000000
00000000000000000000000000000000000000000000000000000000000000000000000000000000000
00000000000000000000000000000000000000000000000000000000000000000000000000000000000
0
>Q4Q7M2_1xtp_0_249
MPSKEASSRNLPISGRDTNGKTYRSTDEMWKAELTGDLYDPEKGWYGKALEYWRTVPATV
SGVLGGMDHVHDVDIEGSRNFIASLPGHGTSRALDCGAGIGRITKNLLTKLYATTDLLEPVK
HMLEEAKRELAGMPVGKFILASMETATLPPNTYDLIVIQWTAIYLTDADFVKFFKHCQQAL
TPNGYIFFKENCSTGDRFLVDKEDSSLTRSDIHYKRLFNESGVRVVKEAFQEEWPTDLFPLK
MYALK
00111111100000000000000000000000000000000000000000000000000000000000000000000000000
00000000000000000000000000000000000000000000000000000000000000000000000000000000000
00000000000000000000000000000011000000000000000000000000000000000000000000000000000
0
>Q82IF8_3eet_0_249
MTFGEQPAYLRVAGDLRKKIVDGSLPPHTRLPSQARIREEYGVSDTVALEARKVLMAEGLVE
GRSGSGTYVRERPVPRRVARSGYRPDSGATPFRQEQADGAVRGTWESHSEQAEASGAIAER
LDIRPGERVMCTKYVFRDAGEVMMLSTSWEPLAVTGRTPVMLPEEGPVGGMGVVERMAA
IDVIVDNVTEEVGARPGLAEELLTLGGVPGHVVLVIQRTYFASGRPVETADVVVPADRYRVA
YHLPVK

11110000000000000000000000000000000000000000000000000000000000110000000000000000
00001000000000000000000000000000000000000000000000000000000000000000000000000000
00000000000000000000000000000000000000000000000000000000000000000000000000000000
0
>Q8P4B4_2pke_0_249
MTPIAQRDGQAIQLVGFDGDDTLWKSEDYYRTAEADFEAILSGYLDLGDSRMQQHLLAVE
RRNLKIFGYGAKGMTLSMIETAIELTEARIEARDIQRIVEIGRATLQHPVEVIAGVREAVAAIA
ADYAVVLITKGDLFHQEQKIEQSGLSDLFPRIEVVSEKDPQTYARVLSEFDLPAERFVMIGNS
LRSDVEPVLAIGGWGIYTPYAVTWAHEQDHGVAADEPRLREVPDPSGWPAAVRALDAQA
GRQQ
00000000000000000000000000000000000000000000000000000000000000000000000000000000
00000000000000000000000000000000000000000000000000000000000000000000000000000000
00000000000000000000000000000000000000001111110000000000000000000000000000000000
0
>Q8R1S4_2d1l_0_249
MEAVIEKECSALGGLFQTIISDMKGSYPVWEDFINKAGKLQSQLRTTVVAAAAFLDAFQKV
ADMATNTRGGTREIGSALTRMCMRHRSIEAKLRQFSSALIDCLINPLQEQMEEWKKVANQ
LDKDHAKEYKKARQEIKNKSSDTLKLQKKAKKVDAQGRGDIQPQLDSALQDVNDKYLLL
EETEKQAVRKALIEERGRFCTFISMLRPVIEEEISMLGEITHLQTISEDLKSLTMDPHKLPSSSE
QVILD
00000000000000000000000000000000000000000000000000000000000000000000000000000000
00000000000000000000000000000000000000000000000000000000000000000000000000000000
00000000000000000000000000000000000000000000000000000000000000000000000000000111
1
>Q9F5K5_1o9g_0_249
MSAYRHAVERIDSSDLACGVVLHSAPGYPAFPVRLATEIFQRALARLPGDGPVTLWDPCCG
SGYLLTVLGLLHRRSLRQVIASDVDPAPLELAAKNLALLSPAGLTARELERREQSERFGKPSY
LEAAQAARRLRERLTAEGGALPCAIRTADVFDPRALSAVLAGSAPDVVLTDLPYGERTHWE
GQVPAQPVAGLLRSLASALPAHAVIAVTDRSRKIPVAPVKALERLKIGTRSAVLVRAADVLE
AGP
10000000000000000000000000000000000000000000000000000000000000000000000000000000
00000000000000000000000000000000000000000000000000000000000000000000000000000000
00000000000000000000000000000000000000000000000000000000000000000000000000000000
0
>D3YEX6_3pfb_0_248
MATITLERDGLQLVGTREEPFGEIYDMAIIFHGFTANRNTSLLREIANSLRDENIASVRFDFN
GHGDSDGKFENMTVLNEIEDANAILNYVKTDPHVRNIYLVGHSQGGVVASMLAGLYPDLI
KKVVLLAPAATLKGDALEGNTQGVTYNPDHIPDRLPFKDLTLGGFYLRIAQQLPIYEVSAQ
FTKPVCLIHGTDDTVVSPNASKKYDQIYQNSTLHLIEGADHCFSDSYQKNAVNLTTDFLQN
NNAF
00000000000000000000000000000000000000000000000000000000000000000000000000000000
00000000000000000000000000000000000000000000000000000000000000000000000000000000
00000000000000000000000000000000000000000000000000000000000000000000000000011111
>O58059_3bs4_2_250
SWEIEELDREIGKIKKHSLILIHEEDASSRGKDILFYILSRKLKSDNLVGMFSISYPLQLIIRILSRF
GVDVIKYLENHRLAIVDTFGSFHGIKATMPGVWYLEGMLSSETLPIKYAKAVEDHKKVWM
DLNLFEGRELYGFAISMSGYLEVFTPEETLRYLETSAEVRYGHPAYKKYPRGTNFWLWEGVK
DKRVLLSVYRRADYVLKTRSSLGENGIKRELLVIKTPKPIEELVRFEYEFKGNEPKLRR
00000000000000000000000000000000000000000000000000000000000000000000000000000000
00000000001110000000000000000000000000000000000000000000000000000000000000000000
00000000000000000000000000000000000000000000000000000000000011111000000000000000

>O67517_1lfp_0_248
MAGHSHWAQIKHKKAKVDAQRGKLFSKLIREIIVATRLGGPNPEFNPRLRTAIEQAKKAN
MPWENIERAIKKGAGELEGEQFEEVIYEGYAPGGVAVMVLATTDNRNRTTSEVRHVFTKH
GGNLGASGCVSYLFERKGYIEVPAKEVSEEELLEKAIEVGAEDVQPGEEVHIIYTVPEELYEV
KENLEKLGVPIEKAQITWKPISTVQINDEETAQKVIKLLNALEELDDVQQVIANFEIPEEILQ
KVG
11110000000000000000000000000000000000000000000000000000000000000000000000000000000
00000000000000000000000000000000000000000000000000000000000000000000000000000000000
00000000000000000000000000000000000000000000000000000000000000000000000000000000011
>P12282_1jw9_0_248
MAELSDQEMLRYNRQIILRGFDFDGQEALKDSRVLIVGLGGLGCAASQYLASAGVGNLTLL
DFDTVSLSNLQRQTLHSDATVGQPKVESARDALTRINPHIAITPVNALLDDAELAALIAEH
DLVLDCTDNVAVRNQLNAGCFAAKVPLVSGAAIRMEGQITVFTYQDGEPCYRCLSRLFGE
NALTCVEAGVMAPLIGVIGSLQAMEAIKMLAGYGKPASGKIVMYDAMTCQFREMKLMRN
PGCEVCGQ
10000000000000000000000000000000000000000000000000000000000000000000000000000000000
00000000000000000000000000000000000000000000000000000000000000000000000000000000000
00000000000000001111110000000000000000000000000000000000000000000000000000000000001
>P41043_1m0u_0_248
MADEAQAPPAEGAPPAEGEAPPPAEGAEGAVEGGEAAPPAEPAEPIKHSYTLFYFNVKAL
AEPLRYLFAYGNQEYEDVRVTRDEWPALKPTMPMGQMPVLEVDGKRVHQSISMARFLAK
TVGLCGATPWEDLQIDIVVDTINDFRLKIAVVSYEPEDEIKEKKLVTLNAEVIPFYLEKLEQTV
KDNDGHLALGKLTWADVYFAGITDYMNYMVKRDLLEPYPALRGVVDAVNALEPIKAWIE
KRPVTEV
11111111111111111111111111111111111111111111110000000000000000000000000000000000000
00000000000000000000000000000000000000000000000000000000000000000000000000000000000
00000000000000000000000000000000000000000000000000000000000000000000000000000000000
>Q5KY38_1t0b_0_248
MTTPIRVVVWNEFRHEKKDEQVRAIYPEGMHTVIANYLAEAGFDAATAVLDEPEHGLTDE
VLDRCDVLVWWGHIAHDEVKDEVVERVHRRVLEGMGLIVLHSGHFSKIFKKLMGTTCNL
KWREADEKERLWVVAPGHPIVEGIGPYIELEQEEMYGEFFDIPEPDETIFISWFEGGEVFRSG
CTFTRGKGKIFYFRPGHETYPTYHHPDVLKVIANAVRWAAPVNRGEIVFGNVKPLEPIKAK
QGGVTQ
11000000000000000000000000000000000000000000000000000000000000000000000000000000000
00000000000000000000000000000000000000000000000000000000000000000000000000000000000
00000000000000000000000000000000000000000000000000000000000000000000000000001111111
>Q65EX3_3oqh_0_248
MTELIMESKHQLFKTETLTQNCNEILKRRRHVLVGISPFNSRFSEDYIHRLIAWAVREFQSVS
VLLAGKEAANLLEALGTPHGKAERKVRKEVSRNRRFAEKALEAHGGNPEDIHTFSDFANQ
TAYRNLRMEVEAAFFDQTHFRNACLEMSHAAILGRARGTRMDVVEVSADMLELAVEYVI
AELPFFIAAPDILGVEETLLAYHRPWKLGEQISRNEFAVKMRPNQGYLMVSEADERVESKS
MQEERV
11111111110000000000000000000000000000000000000000000000000000000000000000000000000
00000000000000000000000000000000000000000000000000000000000000000000000000000000000
00000000000000000000000000000000000000000000000000000000000000000000011111111111111
>Q6D6E7_2p7h_0_248
MTISRNYDQEIKDTAGHKYAYNFDFDVMHPFMVRAFTPFFRPGNLLELGSFKGDFTSRLQE
HFNDITCVEASEEAISHAQGRLKDGITYIHSRFEDAQLPRRYDNIVLTHVLEHIDDPVALLKR
INDDWLAEGGRLFLVCPNANAVSRQIAVKMGIISHNSAVTEAEFAHGHRCTYALDTLERDA
SRAGLQVTYRSGIFFKALANFQWDQILQTDILSKEYLDGCYQLGQQYPDLCASIFLLCEKGI
NQ

111111111111111111110000000000000000000000000000000000000000000000000000000000000000
000000000000000000000000000000000000000000000000000000000000000000000000000000000000
000000000000000000000000000000000000000000000000000000000000000000000000000000000000
>Q87M69_3ck6_0_248
MGFMIEHWDFSTPMATQETTTAEHIQPNHWYHCERLHPDIRSWLEDNHVPRATVDHLLA
DESRPSFHPLDDDNFMLILRGINMNENASPEDMLSIRILYFQGALISTRKIPSRAIMEIRQALA
EHKGPKSLASLLNQIIEGLNGKIDLYLDTIEETLNEFDVNDESTYNHIAAQKALISIKRFIRPQ
QYAIRDLIESESELVTSRPHQYRFAHNNITRINETIEFYLGEVALFQDEIKHNRDEKTNKNS
000000000000000000000000000000000000000000000000000000000000000000000000000000000000
000000000000000000000000000000000000000000000000000000000000000000000000000000000000
000000000000000000000000000000000000000000000000000000000000000000000000000000011111
>Q9LCY2_3g89_0_248
MFHGKHPGGLSERGRALLLEGGKALGLDLKPHLEAFSRLYALLQEASGKVNLTALRGEEEV
VVKHFLDSLTLLRLPLWQGPLRVLDLGTGAGFPGLPLKIVRPELELVLVDATRKKVAFVERAI
EVLGLKGARALWGRAEVLAREAGHREAYARAVARAVAPLCVLSELLLPFLEVGGAAVAMK
GPRVEEELAPLPPALERLGGRLGEVLALQLPLSGEARHLVVLEKTAPTPPAYPRRPGVPERH
PLC
000000000000000000000000000000000000000000000011111111110000000000000000000000000000
000000000000000000000000000000000000000000000000000000000000000000000000000000000000
000000000000000000000000000000000000000000000000000000000000000000000000000000000000
>Q9UBU9_1jkg_370_618
PPCKGSYFGTENLKSLVLHFLQQYYAIYDSGDRQGLLDAYHDGACCSLSIPFIPQNPARSSLA
EYFKDSRNVKKLKDPTLRFRLLKHTRLNVVAFLNELPKTQHDVNSFVVDISAQTSTLLCFSV
NGVFKEVDGKSRDSLRAFTRTFIAVPASNSGLCIVNDELFVRNASSEEIQRAFAMPAPTPSSS
PVPTLSPEQQEMLQAFSTQSGMNLEWSQKCLQDNNWDYTRSAQAFTHLKAKGEIPEVAF
MK
000000000000000000000000000000000000000000000000000001111110000000000000000000000000
000000000000000000000000000000000000000000000000000000000000000000000000000000000000
000000000000000000011111111111111111111111111111111111111111111111111111111111111111
>A2Q6L5_1u58_21_268
GTEDSSESGLRYAYTLVVDGTANTRRCFGTGHVDGEAFVGYSNNKTHGIGRWVNASHVEE
ENKEFVRQCKELQAELDKMQNNSKVIGVKTVQLDVGCTSKIEKHYAYDGNETEDDTATSA
TERDRDCQKKLTEYRKLVLASAVSPQLEVERRSSGREGGMRLRCFARDYYPADLEIRWWKD
DGGGGALPQTSKQHHDPLPSGNGLYQKHIDVYVDGGLEHVYSCRVKGIATGLELQIVRWK
GYARGAG
111111000000000000000000000000000000000000000000000000000000000000000000000000000000
000000000000000000000000000000111111111111100000000000000000000000000000000000000000
000000000000000000000000000000000000000000000000000000000000000000000000000001111111
>A4SNL2_3mf4_1_248
KQKILIVEDSMTIRRTLIQAIAQQTGLEIDAFDTLEGARHCQGDEYVVALVDLTLPDAPSGE
AVKVLLERGLPVVILTADISEDKREAWLEAGVLDYVMKDSRHSLQYAVGLVHRLYLNQQIE
VLVVDDSRTSRHRTMAQLRKQLLLVHEASHAREALTTLEQNPAIRLVLVDYYMPEIDGISLV
RMLRERYSKQQLAIIGISVSDKRGLSARYLKQGANDFLNQPFEPEELQCRVSHNLEALEQFN
S
000000000000000000000000000000000000000000000000000000000000000000000000000000000000
000000000000000000000000000000000000000000000000000000000000000000000000000000000000
000000000000000000000000000000000000000000000000000000000000000000000000000000000011
>A7V680_4eiu_23_270
IDNYEEPSETIWGEVVDEATGKRVLTDQGSEGIRVRLTELSWGDNVQHNPDFYCMMDGTF
QNTKIFKGEYNVRIDGPFIPLVRENTDGTLLHDGSVNTEISGTTKVKFEVQPFLNVEFVGNP
QVSNGVIKAQVRVTRGVSDEVFREKIQPMGNWKDEYLNVTDIQFFVSYSNTVGYRARDER

WSSSINYEGKSFEGLLGKEVTIQSNGNVPSGRKVFVRAAARINYDTPVGSGTRRWNYSEPM
EVLIP
111111000000000000000000000000000000000000000000000000000000000000000000000000000000000000000
000000000000000000000000000000000000000000000000000000000000000000000000000000000000000000000
00000000000000000000000000000000000000000000000000000000000000000000000000000000000000000000
>E7FH78_3nok_0_247
MSPTNRPVPDAGGSVQEPVRRVAHIIREYPHATNAFTQGLVFHQGHFFESTGHQGTLRQLS
LESAQPVWMERLGNIFAEGLASDGERLYQLTWTEGLLFTWSGMPPQRERTTRYSGEGWGL
CYWNGKLVRSDGGTMLTFHEPDGFALVGAVQVKLRGQPVELINELECANGVIYANIWHSS
DVLEIDPATGTVVGVIDASALTRAVAGQVTNPEAVLNGIAVEPGSGRIFMTGKLWPRLFEVR
LDVVD
111111111111110000000000000000000000000000000000000000000000000000000000000000000000000000000
000000000000000000000000000000000000000000000000000000000000000000000000000000000000000000000
00000000000000000000000000000000000000000000000000000000000000000000000000000000000000000000
>Q06121_3nyz_0_247
MPRYLKGWLKDVVQLSLRRPSFRASRQRPIISLNERILEFNKRNITAIIAEYKRKSPSGLDVER
DPIEYSKFMERYAVGLSILTEEKYFNGSYETLRKIASSVSIPILMKDFIVKESQIDDAYNLGADT
VLLIVKILTERELESLLEYARSYGMEPLIEINDENDLDIALRIGARFIGINSRDLETLEINKENQR
KLISMIPSNVVKVAESGISERNEIEELRKLGVNAFLIGSSLMRNPEKIKEFIL
100000000000000000000000000000000000000000000000000000000000000000000000000000000000000000000
000000000000000000000000000000000000000000000000000000000000000000000000000000000000000000000
00000000000000000000000000000000000000000000000000000000000000000000000000000000000000000000
>Q55891_3i8u_0_247
MAVTDLSLTNSSLMPTLNPMIQQLALAIAASWQSLPLKPYQLPEDLGYVEGRLEGEKLVIEN
RCYQTPQFRKMHLELAKVGKGLDILHCVMFPEPLYGLPLFGCDIVAGPGGVSAAIADLSPT
QSDRQLPAAYQKSLAELGQPEFEQQRELPPWGEIFSEYCLFIRPSNVTEEERFVQRVVDFLQI
HCHQSIVAEPLSEAQTLEHRQGQIHYCQQQQKNDKTRRVLEKAFGEAWAERYMSQVLFD
VIQ
111110000000000000000000000000000000000000000000000000000000000000000000000000000000000000000
000000000000000000000000000000000000000000000000000000000000000000000000000000000000000000000
00000000000000000000000000000000000000000000000000000000000000000000000000000000000000000000
>Q5KUD5_1t0t_0_247
MSEAAQTLDGWYCLHDFRTIDWSAWKTLPNEEREAAISEFLALVDQWETTESEKQGSHAV
YTIVGQKADILFMILRPTLDELHEIETALNKTKLADYLLPAYSYVSVVELSNYLASGSEDPYQI
PEVRRRLYPILPKTNYICFYPMDKRRQGNDNWYMLSMEQRRELMRAHGMTGRKYAGKVT
QIITGSVGLDDFEWGVTLFSDDALQFKKLVYEMRFDEVSARFGEFGSFFVGTRLSVEKVPSFF
HV
111110000000000000000000000000000000000000000000000000000000000000000000000000000000000000000
000000000000000000000000000000000000000000000000000000000000000000000000000000000000000000000
00000000000000000000000000000000000000000000000000000000000000000000000000000000000000000000
>Q9CPT3_2gfh_0_247
MGLSRVRAVFFDLDNTLIDTAGASRRGMLEVIKLLQSKYHYKEEAEIICDKVQVKLSKECFH
PYSTCITDVRTSHWEEAIQETKGGADNRKLAEECYFLWKSTRLQHMILADDVKAMLTELR
KEVRLLLLTNGDRQTQREKIEACACQSYFDAIVIGGEQKEEKPAPSIFYHCCDLLGVQPGDC
VMVGDTLETDIQGGLNAGLKATVWINKSGRVPLTSSPMPHYMVSSVLELPALLQSIDCKVS
MSV
000000000000000000000000000000000000000000000000000000000000000000000000011110000000000000000
000000000000000000000000000000000000000000000000000000000000000000000000000000000000000000000
00000000000000000000000000000000000000000000000000000000000000000000000000000000000000000001
>Q9UGB7_2ibn_37_284
DRVFTTYKLMHTHQTVDFVRSKHAQFGGFSYKKMTVMEAVDLLDGLVDESDPDVDFPNS

FHAFQTAEGIRKAHPDKDWFHLVGLLHDLGKVLALFGEPQWAVVGDTFPVGCRPQASVV
FCDSTFQDNPDLQDPRYSTELGMYQPHCGLDRVLMSWGHDEYMYQVMKFNKFSLPPEAF
YMIRFHSFYPWHTGRDYQQLCSQQDLAMLPWVREFNKFDLYTKCPDLPDVDKLRPYYQG
LIDKYCPGILSW
00000000000000000000000000000000000000000000000000011100000000000000000000000000000
00000000000000000000000000000000000000000000000000000000000000000000000000000000000
000000000000000000000000000000000000000000000000000111111000000000000000000000000
>C2IC25_4evw_0_246
MIVIPMAGMSSRFFKAGYTQPKYMLEAHGQTLFEHSVNSFAAYFASTPFLFIVRNVYDTAVF
VREKATQLGIKQFYIAELHTETRGQAETVTLGLEELAKQGVDYQGSITVFNIDTFRPNFVFP
DISQHSDGYLEVFQGGGDNWSFAKPEHAGSTKVIQTAEKNPISDLCSTGLYHFNRKEDYLE
AYREYVARPSQEWERGELYIAPLYNLLIQKGLNIHYHLIARHEVIFCGVPDEYTDFLRQPQP
00000000000000000000000000000000000000000000000000000000000000000000000000000000000
00000000000000000000000000000000000000000000000000000000000000000000000000000000000
000000000000000000000000000000000000000000000000000000000000000000000000000000111
>P16326_2d4x_39_285
SDDPIAASQAVVLSQAQAQNSQYALARTFATQKVSLEESVLSQVTTAIQTAQEKIVYAGNGT
LSDDDRASLATDLQGIRDQLMNLANSTDGNGRYIFAGYKTEAAPFDQATGGYHGGEKSVT
QQVDSARTMVIGHTGAQIFNSITSNAVPEPDGSDSEKNLFVMLDTAIAALKTPVEGNNVEK
EKAAAAIDKTNRGLKNSLNNVLTVRAELGTQLSELSTLDSLGSDRALGQKLQMSNLVDVD
WNSV
11111111110000000000000000000000000000000000000000000000000000000000000000000000000
00000000000000000000000000000000000000000000000000000000000000000000000000000000000
000000000000000000000000000000000000000000000000000000000011111111111111111111111
>P17989_3axe_24_270
VSAKDFSGAELYTLEEVQYGKFEARMKMAAASGTVSSMFLYQNGSEIADGRPWVEVDIEV
LGKNPGSFQSNIITGKAGAQKTSEKHHAVSPAADQAFHTYGLEWTPNYVRWTVDGQEVR
KTEGGQVSNLTGTQGLRFNLWSSESAAWVGQFDESKLPLFQFINWVKVYKYTPGQGEGGS
DFTLDWTDNFDTFDGSRWGKGDWTFDGNRVDLTDKNIYSRDGMLILALTRKGQESFNGQ
VPRDDEPAP
11000000000000000000000000000000000000000000000000000000000000000000000000000000000
00000000000000000000000000000000000000000000000000000000000000000000000000000000000
000001111111000000000000000000000000000000000000000000000000000000000000000001111
>Q1RAR4_4gek_0_246
MSHRDTLFSAPIARLGDWTFDERVAEVFPDMIQRSVPGYSNIISMIGMLAERFVQPGTQVYD
LGCSLGAATLSVRRNIHHDNCKIIAIDNSPAMIERCRRHIDAYKAPTPVDVIEGDIRDIAIEN
ASMVVLNFTLQFLEPSERQALLDKIYQGLNPGGALVLSEKFSFEDAKVGELLFNMHHDFKR
ANGYSELEISQKRSMLENVMLTDSVETHKARLHKAGFEHSELWFQCFNFGSLVALKAEDA
A
11111111111111000000000000000000000000000000000000000000000000000000000000000000000
00000000000000000000000000000000000000000000000000000000000000000000000000000000000
000000000000000000000000000000000000000000000000000000000000000000000000000000000
>Q45U61_3d3z_23_269
TIDTCSSDSPLSCQTDNEASCCFNSPGGSLLQTQFWDYDPSDGPSGSWTIHGLWPDNCDGT
YQEYCDESREYSNITSILEAQNRTELLSYMKEYWPDYEGADEDESFWEHEWNKHGTCINTI
EPSCYTDYYAQEEVGDFFQQVVDLFKTLDSYTALSDAGITPSEDATYKLSDIEDALAAIHDG
YPPYVGCEDGALSQLYYYFNVKGSAIGGTYVASERLEDSNCKDSGIKYPPKYSSSKKIYGSSL
00000000000000000000000000000000000000000000000000000000000000000000000000000000000
00000000000000000000000000000000000000000000000000000000000000000000000000000000000
000000000000000000000000000000000000000000000000000000000000000000000000111111111
>Q8A611_2ess_0_246

MSEENKIGTYQFVAEPFHVDFNGRLTMGVLGNHLLNCAGFHASDRGFGIATLNEDNYTW
VLSRLAIELDEMPYQYEKFSVQTWVENVYRLFTDRNFAVIDKDGKKIGYARSVWAMINLNT
RKPADLLALHGGSIVDYICDEPCPIEKPSRIKVTSNQPVATLTAKYSDIDINGHVNSIRYIEHIL
DLFPIELYQTKRIRRFEMAYVAESYFGDELSFFCDEVSENEFHVEVKKNGSEVVCRSKVIFE
00000000000000000000000000000000000000000000000000000000000000000000000000000000
00000000000000000000000000000000000000001111000000000000000000000000000000000000
000000000000000000000000000000000000000000000000000000000000000000000000000000
>Q930A6_4g9q_0_246
MMTTSNAGAQQPNVEGRRFSPDQVRSVAPALEQYTQQRLYGDVWQRPGLNRRDRSLVTI
AALIARGEAPALTYYADQALENGVKPSEISETITHLAYYSGWGKAMATVGPVSEAFAKRGIG
QDQLAAVESTPLPLDEEAEAQRATTVGNQFGSVAPGLVQYTTDYLFRDLWLRPDLAPRDRS
LVTIAALISVGQVEQITFHLNKALDNGLSEEQAAEVITHLAFYAGWPNAMSALPVAKAVFE
KRRG
11111111100000000000000000000000000000000000000000000000000000000000000000000000
00000000000000000000000000000000000000000000000000000000000000000000000000000000
000000000000000000000000000000000000000000000000000000000000000000000000000000
>Q9KAF6_1vl5_18_264
YVTSQIHAKGSDLAKLMQIAALKGNEEVLDVATGGGHVANAFAPFVKKVVAFDLTEDILK
VARAFIEGNGHQQVEYVQGDAEQLPFTDERFHIVTCRIAAHHFPNPASFVSEAYRVLKKGG
QLLLVDNSAPENDAFDVFYNYVEKERDYSHHRAWKKSDWLKMLEEAGFELEELHCFHKT
FIFEDWCDRMNVTTEKKQELSDFIKSKPTEYYQKFKIVVEDGRVYSFRGESILMKARKPTVK
NKGSS
11111111100000000000000000000000000000000000000000000000000000000000000000000000
00000000000000000000000000000000000000000000000000000000000000000000000000000000
000000000000000000000000000000000000000000000000000000000000000000000001111111
>Q9RWH9_2boo_0_246
MTDQPDLFGLAPDAPRPIIPANLPEDWQEALLPEFSAPYFHELTDFLRQERKEYTIYPPAPDV
FNALRYTPLGEVKVLILGQDPYHGPNQAHGLSFSVRPGVRVPPSLRNIYKELTEDIPGFVAP
KHGYLRSWAEQGVLLLNAVLTVRAGQANSHQGKGWEHFTDAVIKAVNAKEERVVFILWG
SYARKKKKLITGKNHVVIESGHPSPLSEQYFFGTRPFSKTNEALEKAGRGPVEWQLPATVTE
E
11111111111111110000000000000000000000000000000000000000000000000000000000000000
00000000000000000000000000000000000000000000000000000000000000000000000000000000
000000000000000000000000000000000000000000000000000000000000000000000000000001
>A7LVT2_4ayl_0_245
MRIGILYICTGKYDIFWKDFYLSAERYFMQDQSFIIEYYVFTDSPKLYDEENNKHIHRIKQKN
LGWPDNTLKRFHIFLRIKEQLERETDYLFFFNANLLFTSPIGKEILPPSDSNGLLGTMHPGFY
NKPNSEFTYERRDASTAYIPEGEGRYYYAGGLSGGCTKAYLKLCTTICSWVDRDATNHIIPIW
HDESLINKYFLDNPPAITLSPAYLYPEGWLLPFEPIILIRDKNKPQYGGHELLRRKN
00000000000000000000000000000000000000000000000000000000000000000000000000000000
00000000000000000000000000000000000000000000011111111111111111111100000000000000
00000000000000000000000000000000000000000000000000000000000000000011111111111
>P40165_1jzt_0_245
MSTLKVVSSKLAAEIDKELMGPQIGFTLQQLMELAGFSVAQAVCRQFPLRGKTETEKGKHV
FVIAGPGNNGGDGLVCARHLKLFGYNPVVFYPKRSERTEFYKQLVHQLNFFKVPVLSQDE
GNWLEYLKPEKTLCIVDAIFGFSFKPPMREPFKGIVEELCKVQNIIPIVSVDVPTGWDVDKGP
ISQPSINPAVLVSLTVPKPCSSHIRENQTTHYVGGRFIPRDFANKFGFEPFGYESTDQILKL
11100000000000000000000000000000000000000000000000000000000000000000000000000000
00000000000000000000000000000000000000000000000000000000000000000000000000000000
00000000000000000000000000000000000000000000000000000000000000000000000000000
>P41088_4doi_0_245

MSSSNACASPSPFPAVTKLHVDSVTFVPSVKSPASSNPLFLGGAGVRGLDIQGKFVIFTVIGV
YLEGNAVPSLSVKWKGKTTEELTESIPFFREIVTGAFEKFIKVTMKLPLTGQQYSEKVTENCV
AIWKQLGLYTDCEAKAVEKFLEIFKEETFPPGSSILFALSPTGSLTVAFSKDDSIPETGIAVIENK
LLAEAVLESIIGKNGVSPGTRLSVAERLSQLMMKNKDEKEVSDHSVEEKLAKEN
11111111111111000000000000000000000000000000000000000000000000000000000000000000000
00000000000000000000000000000000000000000000000000000000000000000000000000000000000
0000000000000000000000000000000000000000000000000000000000000000000011111111111
>Q5SJQ0_1wzo_0_245
MKLARFLAKGRVHQGVYREGLLLDEAGEAHRPEDVTWLLPFTPGKILGVALNYADHAEEL
GLSRPEEPALFWKPNTSLLPHKGVVLYPKGARFVHYEVELAVVVGRPMKRVRAKDALDYV
LGYTIANDLVARDYVTNTFRPPIRAKGRDTFLPLGPFLVVEEVEDPQDLWLRAYVNGELRQE
GHTSRMLYSVAELLEFISEFMTLEPYDVLLTGTPKGISQVRPGDVMRLEIEGLGALENPIEEEP
00000000000000000000000000000000000000000000000000000011111111000000000000000000000
00000000000000000000000000000000000000000000000000000000000000000000000000000000000
0000000000000000000000000000000000000000000000000000000000000000000000000000000
>Q8A309_2a2m_0_245
MNDFKNQWLRKRTFAIPASRLTGRLTTLKSDVPAADSLFWKLWNGSLDTAVQVLQTDYFK
GIAAGTLDPNAYGSLMVQDGYYCFRGRDDYATAATCAQDETLREFFKAKAKSYDEYNETY
HQTWHLREASGLIPGTDIKDYADYEAYVAGSLASPYMCVVMLPCEYLWPWIANFLDGYTP
TNSLYRFWIEWNGGTPNGAYQMGNMLEQYRDKIDEDKAVEIFNTAMNYELKVFTSSTILTT
IENGK
11111111000000000000000000000000000000000000000000000000000000000000000000000000000
00000000000000000000000000000000000000000000000000000000000000000000000000000000000
0000000000000000000000000000000000000000000000000000000000000000000000000111111
>Q8RNV5_2oa9_0_245
MSEYLNLLKEAIQNVVDGGWHETKRKGNTGIGKTFEDLLEKEEDNLDAPDFHDIEIKTHET
AAKSLLTLFTKSPTNPRGANTMLRNRYGKKDEYGNNILHQTVSGNRKTNSNSYNYDFKIDI
DWESQVVRLEVFDKQDIMIDNSVYWSFDSLQNQLDKKLKYIAVISAESKIENEKKYYKYNS
ANLFTDLTVQSLCRGIENGDIKVDIRIGAYHSGKKKGKTHDHGTAFRINMEKLLEYGEVKVI
V
00000000000000000000000001110000000000000000000000000000000000000000000000000000000
00000000000000000000000000000000000000000000000000000000000000000000000000000000000
0000000000000000000000000000000000000000000000000000000000000000000000000000000
>P04390_1eon_0_244
MSLRSDLINALYDENQKYDVCGIISAEGKIYPLGSDTKVLSTIFELFSRPIINKIAEKHGYIVEE
PKQQNHYPDFTLYKPSEPNKKIAIDIKTTYTNKENEKIKFTLGGYTSFIRNNTKNIVYPFDQYI
AHWIIGYVYTRVATRKSSLKTYNINELNEIPKPYKGVKVFLQDKWVIAGDLAGSGNTTNIGS
IHAHYKDFVEGKGIFDSEDEFLDYWRNYERTSQLRNDKYNNISEYRNWIYRGRK
10000000000000000000000000000000000000000000000000000000000000000000000000000000000
00000000000000000000000000000000000000000000000000000000001110000000000000000000000
000000000000000000000000000000000000000000000000000000000000000000000000000000
>P04871_2wux_0_244
MPDYSYRPTIGRTYVYDNKYYKNLGAVIKNAKRKKHFAEHEIEEATLDPLDNYLVAEDPFL
GPGKNQKLTLFKEIRNVKPDTMKLVVGWKGKEFYRETWTRFMEDSFPIVNDQEVMDVFLV
VNMRPTRPNRCYKFLAQHALRCDPDYVPHDVIRIVEPSWVGSNNEYRISLAKKGGGCPIM
NLHSEYTNSFEQFIDRVIWENFYKPIVYIGTDSAEEEEILLEVSLVFKVKEFAPDAPLFTGPAY
11000000000000000000000000000001111111111111111100000000000000000000000000000000000
00000000000000000000000000000000000000000000000000000000000000000000000000000000000
000000011111111111110000000000000000000000000000000000000000000000000000000000
>P20849_2uur_23_267
AVKRRPRFPVNSNSNGGNELCPKIRIGQDDLPGFDLISQFQVDKAASRRAIQRVVGSATLQV

AYKLGNNVDFRIPTRNLYPSGLPEEYSFLTTFRMTGSTLKKNWNIWQIQDSSGKEQVGIKIN
GQTQSVVFSYKGLDGSLQTAAFSNLSSLFDSQWHKIMIGVERSSATLFVDCNRIESLPIKPRG
PIDIDGFAVLGKLADNPQVSVPFELQWMLIHCDPLRPRRETCHELPARITPSQTTDER
11111111111111111100000011111000000000000000000000000000000000000000000000000000000
00000000000000000000000000000000000000000000000000000000000000000000000000000000000
0000000000000000000000000000000000000000000000000000000000000000000111111111111
>P68640_1xkp_32_276
QFRGESVQIVSGTLQSIADMAEEVTFVFSERKELSLDKRKLSDSQARVSDVEEQVNQYLSKV
PELEQKQNVSELLSLLSNSPNISLSQLKAYLEGKSEEPSEQFKMLCGLRDALKGRPELAHLSH
LVEQALVSMAEEQGETIVLGARITPEAYRESQSGVNPLQPLRDTYRDAVMGYQGIYAIWSDL
QKRFPNGDIDSVILFLQKALSADLQSQQSGSGREKLGIVISDLQKLKEFGSVSDQVKG
00000000000000000000000001111110000000000000000000000000000000000000000000000000000
00000000000000000000000000000000000000000000000000000000000000000000000000000000000
0000000000000000000000000000000000000000000000000000000000000000000000011111111
>P75914_1m65_0_244
MYPVDLHMHTVASTHAYSTLSDYIAQAKQKGIKLFAITDHGPDMEDAPHHWHFINMRIW
PRVVDGVGILRGIEANIKNVDGEIDCSGKMFDSLDLIIAGFHEPVFAPHDKATNTQAMIATI
ASGNVHIISHPGNPKYEIDVKAVAEAAAKHQVALEINNSSFLHSRKGSEDNCREVAAAVRD
AGGWVALGSDSHTAFTMGEFEECLKILDAVDFPPERILNVSPRRLLNFLESRGMAPIAEFAD
L
10000000000000000000000000000000000000000000000000000000000000000000000000000000000
00000000000000000000000000000000000000000000000000000000000000000000000000000011111
1111110000000000000000000000000000000000000000000000000000000000000000000000000
>Q9HCB6_3q13_189_433
GVTDKPILDCCACGTAKYRLTFYGNWSEKTHPKDYPRRANHWSAIIGGSHSKNYVLWEYG
GYASEGVKQVAELGSPVKMEEEIRQQSDEVLTVIKAKAQWPAWQPLNVRAAPSAEFSVDR
TRHLMSFLTMMGPSPDWNVGLSAEDLCTKECGWVQKVVQDLIPWDAGTDSGVTYESPNK
PTIPQEKIRPLTSLDHPQSPFYDPEGGSITQVARVVIERIARKGEQCNIVPDNVDDIVADLAPE
EK
11000000000000000000000000000000000000000000000000000000000000000000000000000000000
00000000000000000000000000000000000000000000000000000000000000000000000000000000000
0000000000000000000000000000000000000000000000000000000000000000000000001111111
>Q9HLX1_3ll5_0_244
MMILKIGGSVITDKSAYRTARTYAIRSIVKVLSGIEDLVCVVHGGGSFGHIKAMEFGLPGPKN
PRSSIGYSIVHRDMENLDLMVIDAMIEMGMRPISVPISALRYDGRFDYTPLIRYIDAGFVPVS
YGDVYIKDEHSYGIYSGDDIMADMAELLKPDVAVFLTDVDGIYSKDPKRNPDAVLLRDIDT
NITFDRVQNDVTGGIGKKFESMVKMKSSVKNGVYLINGNHPERIGDIGKESFIGTVIR
00000000000000000000000000000000000000000000000000000000000000000000000000000000000
00000000000000000000000000000000000000000000000000000000000000000000000000000000000
0000000000000000000000111111111111000000000000000000000000000000000000000000000
>A6L7P8_3dv9_0_243
MFKEAINNYLHTHGYESIDLKAVLFDMDGVLFDSMPNHAESWHKIMKRFGFGLSREEAYM
HEGRTGASTINIVSRRERGHDATEEEIKAIYQAKTEEFNKCPKAERMPGALEVLTKIKSEGLT
PMVVTGSGQTSLLDRLNHNFPGIFQANLMVTAFDVKYGKPNPEPYLMALKKGGFKPNEA
LVIENAPLGVQAGVAAGIFTIAVNTGPLHDNVLLNEGANLLFHSMPDFNKNWETLQSALK
QD
00000000000000000000000000000000000000000000000000000000000000000000000000000000000
00000000000000000000000000000000000000000000000011100000000000000000000000000000000
000000000000000000000000000000000000000000000000000000000000000000000000000001
>P0A3S3_3owv_30_273
APNSPKTNLSQKKQASEAPSQALAESVLTDAVKSQIKGSLEWNGSGAFIVNGNKTNLDAK

VSSKPYADNKTKTVGKETVPTVANALLSKATRQYKNRKETGNGSTSWTPPGWHQVKNLK
GSYTHAVDRGHLLGYALIGGLDGFDASTSNPKNIAVQTAWANQAQAEYSTGQNYYESKVR
KALDQNKRVRYRVTLYYASNEDLVPSASQIEAKSSDGELEFNVLVPNVQKGLQLDYRTGEV
TVTQ
11111111111100000000000000000000000000000000000000000000000000000000000000000000000
00000000000011111111000000000000000000000000000000000000000000000000000000000000000
000000000000000000000000000000000000000000000000000000000000000000000000000000
>P44627_1vhy_1_244
RIPRIYHPISLENQTQCYLSEDAANHVARVLRMTEGEQLELFDGSNHIYPAKIIESNKKSVKV
EILGRELADKESHLKIHLGQVISRGERMEFTIQKSVELGVNVITPLWSERCGVKLDAERMDK
KIQQWQKIAIAACEQCGRNIVPEIRPLMKLQDWCAENDGALKLNLHPRAHYSIKTLPTIPA
GGVRLLIGSEGGLSAQEIAQTEQQGFTEILLGKRVLRTETASLAAISALQICFGDLGE
10000000000000000000000000000000000000000000000000000000000000000000000000000000000
00001100000000000000000000000000000000000000000000000000000000000000000000000000000
000000000000000000000000000000000000000000000000000000000000000000000000000000
>P46675_2qk1_316_559
SMLPEETILDKLPKDFQERITSSKWKDRVEALEEFWDSVLSQTKKLKSTSQNYSNLLGIYGHI
IQKDANIQAVALAAQSVELICDKLKTPGFSKDYVSLVFTPLLDRTKEKKPSVIEAIRKALLTIC
KYYDPLASSGRNEDMLKDILEHMKHKTPQIRMECTQLFNASMKEEKDGYSTLQRYLKDEV
VPIVIQIVNDTQPAIRTIGFESFAILIKIFGMNTFVKTLEHLDNLKRKKIEETVKTL
00000000000000000000000000000000000000000000000000000000000000000000000000000000000
00000000000000000000000000000000000000000000000000000000000000000000000000000000000
000000000000000000000000000000000000000000000000000000000000000000000000000001
>Q5LAY5_3nqi_23_266
DSGESGPQQWAGVVKVNDRMGYVTFTDAAGTELIPTNTIPVTLNARMAYIYCQVDEGQD
LSTNPKSIKITLLADPTGIDATAITTPKVGESGDVTTNAPVGSLSFVSGYSTVAPFQFSENTIVL
PVLYRVKNVTTTEDIKNELAKHTFTLVCYTDDIKSGDTILKLYLRYKVEDEPAAIAERATRTSS
FKAYEISQILREYTLKSGQTKPAKITIVAQQNEYNNKLEDTSTIEKVYEIEYKTAE
11111100000000000000000000000000000000000000000000000000001111100000000000000000000
00000000000000000000000000000000000000000000000000000000000000000000000000000000000
000000000000000000000000000000000000000000000000000000000000000000000000000000
>Q74D82_3c37_20_263
ATSMTDIKGFNMISIEQEKELGNKFAVEIEKQQQPVNDPEVQRYVDKVGKRLLSGARAVEF
DYVFKVVKDDSVNAFAIPGGRVYVHTGLLKAADNETELAGVLAHEINHAVARHGTRQMT
QEYGYSLVLSLVLGDNPNMLAQLAGQLFGKAGMMSYSREYENQADFLGVETMYKAGYNP
NGLTSFFQKLNAMDGGTQSNVARFFSTHPLTSERIQRVQAEIAKLPPQRYLTDETEFKKIKG
RLK
11111110000000000000000000000000000000000000000000000000000000000000000000000000000
00000000000000000000000000000000000000000000000000001100000000000000000000000000000
000000000000000000000000011111111111111000000000000000000000000000000000000000
>Q7MWD8_3is6_41_284
QRSYERDFPHHDRICIVKTHGTRQSQEGDKPEELDFSQVSGGVAPAIQEEIPGVELATRTTLY
GTSKMILEDNKTYETKTLLAEPAFLDMFGVELIAGVRDSALRDNMTCLISESLARKMGGDV
LGKRLRPAESKSDRAITIGGVFEDLPHNSSIQADMLLPITWMPAESLNNWIGNDRYIAYVRL
RPGVSPESLDEALLEMQKRHQDMEVFRKAGVELHYSLTPFNRLRLEDPTLVNMLRIQQ
11110000000000000000000001100000000000000000000000000000000000000000000000000000000
00000000000000000000000000000000000000000000000000000000000000000000000000000000000
000000000000000000000000000000000000000000000000000000000000000000000000000000
>Q87LT3_2oez_1_244
TTHKFEHPLNEKTRIYLRVESLLRQAHLASGFADNHQYQLFFRALFDMVEIFEQIQLKSELA
KDLEKQRLSYRHWLNVEGVDQEALNSLLNEIDVVHSQLMGAERFGQALKEDRFLSSIRQR

FNLPGGSCCFDLPALHYWLHLPIERKKHDANQWQKSLKPLSDALTLWLKLARETGHFKA
QIARAGFFQSDADEANILRLHIPMKYGVYPMISGHKNRFAIKFMAFENGQACSQDVEFELA
VC
00000000000000000000000000000000000000000000000000000000000000000000000000000000000
00000000000000000000000000000000000000000000010000000000000000000000000000000000000
000000000000000000000000000000000000000000000000000000000000000000000000000000
>Q975N2_1ud9_1_244
HIVYDDVRDLKAIIQALLKLVDEALFDIKPEGIQLVAIDKAHISLIKIELPKEMFKEYDVPEEF
KFGFNTQYMSKLLKAAKRKEEIIIDADSPEVVKLTLSGALNRVFNVNNIEVLPPEVPEVNLE
FDIKATINASGLKNAIGEIAEVADTLLISGNEEKVVVKGEGENKVEVEFSKDTGSLADIEFNK
ESSSAYDVEYLNDIISLTKLSDYVKVAFADQKPMQLEFNMEGGGKVTYLLAPKLS
00000000000000000000000000000000000000000000000000000000000000000000000000000000000
00000000000000000000000000000000111111000000000000000000000000000000000000000000000
000000000000000000000000000000000000000000000000000000000000000000000000000000
>Q9DTZ2_3a0n_105_348
TNVISTLDLNLLTKGGGSWNVDGVNMKKSAVTTFDGKRVVKAVYDKNSGTSANPGVGGF
SFSAVPDGLNKNAITFAWEVFYPKGFDFARGGKHGGTFIGHGAASGYQHSKTGASNRIMW
QEKGGVIDYIYPPSDLKQKIPGLDPEGHGIGFFQDDFKNALKYDVWNRIEIGTKMNTFKNG
IPQLDGESYVIVNGKKEVLKRINWSRSPDLLISRFDWNTFFGGPLPSPKNQVAYFTNFQMKK
YE
10000000000000000000000000000000000000000000000000000000000000000000000000000000000
00000000000000000000000000000000000000000000000000000000000000000000000000000000000
000000000000000000000000000000000000000000000000000000000000000000000000000001
>A0QQ72_3f8m_1_243
TAGAAPRILKHQVVRAELDRMLDGMRIGDPFPAEREIAEQFEVARETVRQALRELLIDGRV
ERRGRTTVVARPKIRQPLGMGSYTEAAKAQGLSAGRILVAWSDLTADEVLAGVLGVDVGA
PVLQLERVLTTDGVRVGLETTKLPAQRYPGLRETFDHEASLYAEIRSRGIAFTRTVDTIDTALP
DAREAALLGADARTPMFLLNRVSYDQDDVAIEQRRSLYRGDRMTFTAVMHAKNSAIVS
11111111000000000000000000000000000000000000000000000000000000000000000000000000000
00000000000000000000000000000000000000000000000000000000000000000000000000000000000
00000000000000000000000000000000000000000000000000000000000000000000000011111
>D1C4A9_3rqz_0_242
MRILIISDVHANLVALEAVLSDAGRVDDIWSLGDIVGYGPRPRECVELVRVLAPNISVIGNH
DWACIGRLSLDEFNPVARFASYWTTMQLQAEHLQYLESLPNRMIDGDWTVVHGSPRHPIW
EYIYNARIAALNFPAFDTPLCFVGHTHVPLYIREDEALSNVAPHHPNDGEVLDVSSGRYIIN
PGAVGQPRDGDPRASYAIFEPDAQRVTFHRVEYRIADTQAQMREAGLPESLVTRLAAGV
00000000000000000000000000000000000000000000000000000000000000000000000001100000000
00000000000000000000000000000000000000000000000000000000000000000000000000000000000
00000000000000000000000000000000000000000000000000000000000000000000000000000
>O25234_2i9i_35_277
ETKTPNDAKNQVQTHERMKTSSEHVTPLDFNYPIHIVQAPQNHHVVGILTPRIQVSDNLKP
YIDKFQDALINQIQTIFEKRGYQVLRFQDEKALNAQDKRKIFSVLDLKGWVGILEDLKMNL
KDPNNPNLDTLVDQSSGSVWFNFYEPESNRVVHDFAVEVGTFQAMTYTYKHNNSGGLNS
SNSIIHEYLEKNKEDAIHKILNRMYAVVMKKAVTELTKENIDKYREAIDRMKGFKSSMPQK
K
11111111111111100000000000000000000000000000000000000000000000000000000000000000000
00000000000000000000000000000000000000000000000000000000000000000000000000000000000
00000000000000000000000000000000000000000000000000000000000000000000001111111
>P38777_1ycd_0_242
MTVQIPKLLFLHGFLQNGKVFSEKSSGIRKLLKKANVQCDYIDAPVLLEKKDLPFEMDDEK
WQATLDADVNRAWFYHSEISHELDISEGLKSVVDHIKANGPYDGIVGFSQGAALSSIITNKI

SELVPDHPQFKVSVVISGYSFTEPDPEHPGELRITEKFRDSFAVKPDMKTKMIFIYGASDQAV
PSVRSKYLYDIYLKAQNGNKEKVLAYEHPGGHMVPNKKDIIRPIVEQITSSLQEASE
11000000000000000000000000000000000000000000000000000000000000000000000000000000000
00000000000000000000000000000000000000000000000000000000000000000000000000000000000
00000000000000000000000000000000000000000000000000000000000000000000000000011
>Q06609_1n0w_96_338
SEIIQITTGSKELDKLLQGGIETGSITEMFGEFRTGKTQICHTLAVTCQLPIDRGGGEGKAMYI
DTEGTFRPERLLAVAERYGLSGSDVLDNVAYARAFNTDHQTQLLYQASAMMVESRYALLIV
DSATALYRTDYSGRGELSARQMHLARFLRMLLRLADEFGVAVVITNQVVAQVDGAAMFA
ADPKKPIGGNIIAHASTTRLYLRKGRGETRICKIYDSPCLPEAEAMFAINADGVGDAKD
10000000000000000000000000000000000000000000000000000000000000000000000000000000000
00000000000000000000000000000000000000000000000000111111000000000000000000000000000
00000111111111111111111111111000000000000000000000000000000000000000000000000
>Q2FVD0_4egd_21_263
GYVIMTKTNGRNAQIKDTFNQTLKLYPTKNLDDFYDKEGFRDQEFKKGDKGTWIVNSEM
VIEPKGKDMETRGMVLYINRNTRTTKGYYFISEMTDDSNGRPKDDEKRYPVKMEHNKIIPT
KPLPNDKLKKEIENFKFFVQYGNFKDINDYKDGDISYNPNVPSYSAKYQLNNDDYNVQQL
RKRYDIPTKQAPKLLLKGDGDLKGSSVGSRSLEFTFVENKEENIYFTDSVQYTPSEDTRYESN
11111111000000000000000000000000000000000000000000000000000000000000000000000000000
00000000000111111000000000000000000000000000000000000000000000000000000000000000000
00000000000000000000000000000000000000000000000000000000000000000000001111111
>Q3IXX1_3onp_0_242
MSIEPVFILVRPQMGENIGAAARAMLNFGLGRLRIVDPRDGWPNPKAVAMASGAGRLLD
HAGLFPTVAEAIRDCDYVFATTARGRELTKPVMTPERAMAHGRALTGEGRRVGILFGPERT
GLENEDVALANAIVTVPVNPEFFSLNLAQCVLLLAYEWRRQHDETPPEVIDMARVDFASGL
EVEKLGDHFEEKLEAAGFFFPPEKAPGMKLNLRNMWARLPLTRADVQTLHGMLRQIAWK
LKQ
11000000000000000000000000000000000000000000000000000000000000000000000000000000000
00000000000000000000000000000000000000000000000000000000000000000000000000000111111
11111111111111111111111111111111111111111111111111111111111111111111111111111
>Q7V6D4_2oc5_0_242
MPTLEMPVAAVLDSTVGSSEALPDFTSDRYKDAYSRINAIVIEGEQEAHDNYIAIGTLLPDH
VEELKRLAKMEMRHKKGFTACGKNLGVEADMDFAREFFAPLRDNFQTALGQGKTPTCLLI
QALLIEAFAISAYHTYIPVSDPFARKITEGVVKDEYTHLNYGEAWLKANLESCREELLEANRE
NLPLIRRMLDQVAGDAAVLQMDKEDLIEDFLIAYQESLTEIGFNTREITRMAAAALVS
11111111111111111110000000000000000000000000000000000000000000000000000000000000000
00000000000000000000000000000000000000000000000000000000000000000000000000000000000
00000000000000000000000000000000000000000000000000000000000000000000000000011
>Q8Z0I2_4ev1_31_273
LSEQQIKEKLDSVPIYLVTNEKGLPLSRPLPNAPNGQKAGGSITGAYMSRQEAQAFINELRN
AKNKDPKMQEIVKSLQVTAVPLGVIYQQLQQTKKDPNRLLFAFKPVDQEIKGAMDLLRQS
GQQVNQFKSVPMFAVRFAPDQGYVPIKVGTGNEQVVPLFLSKQDAQGLLGQVKPKHPKA
DIQVLDIDGVLQTLQDKNDTWLNQVVLVPSPESREYIRTLPKPPNTPAAPNRNNNNSRPGA
K
00000000000000000000000000000000000000000000000000000000000000000000000000000000000
00000000000000000000000000000000000000000000000000000000000000000000000000000000000
00000000000000000000000000000000000000000000000000000000000111111111111111111
>Q9UQR0_2biv_0_242
MGQTVNEDSMDVKKENQEKTPQSSTSSVQRDDFHWEEYLKETGSISAPSECFRQSQIPPVN
DFKVGMKLEARDPRNATSVCIATVIGITGARLRLRLDGSDNRNDFWRLVDSPDIQPVGTCE
KEGDLLQPPLGYQMNTSSWPMFLLKTLNGSEMASATLFKKEPPKPPLNNFKVGMKLEAID

KKNPYLICPATIGDVKGDEVHITFDGWSGAFDYWCKYDSRDIFPAGWCRLTGDVLQPPGTS
11111111111111111111111111111110000000000000000000000000000000000000000000000000000
00000000000000000000000000000000000000000000000000111110000000000000000000000000000
00000000000000000000000000000000000000000000000000000000000000000000000000000
>Q9YDZ4_2hls_0_242
MARYYVLDLSEDFRRELRETLAEMVNPVEVHVFLSKSGCETCEDTLRLMKLFEEESPTRNG
GKLLKLNVYYRESDSDKFSEFKVERVPTVAFLGGEVRWTGIPAGEEIRALVEVIMRLSEDESG
LEDATKEALKSLKGRVHIETIITPSCPYCPYAVLLAHMFAYEAWKQGNPVILSEAVEAYENPD
IADKYGVMSVPSIAINGYLVFVGVPYEEDFLDYVKSAAEGRLTVKGPIRAGEAEEL
10000000000000000000000000000000000000000000000000000000000000000000000000000000000
00000000000000000000000000000000000000000000000000000000000000000000000000000000000
00000000000000000000000000000000000000000000000000000000000000000001111111111
>B1MDI3_3quv_0_241
MKIDVVTIFPEYLQPVRQSLPGKAIDAGLVDVAVHDLRRWTHDVHKSVDDSPYGGGPGMV
MKPTVWGDALDEICTSETLLVVPTPAGYPFTQETAWQWSTEDHLVIACGRYEGIDQRVADD
AATRMRVREVSIGDYVLNGGEAAALVIIEAVLRLVPGVLGNALSAQEDSHSEGMASLLEGP
SYTRPPSWRGMDVPPVLLSGDHAKIAAWRAEQSRQRTIERRPDLLGFDSPTGEHGGDGLS
00000000000000000000000000000000000000000000000000000000000000000000000000000000000
00000000000000000000000000000000000000000000000000000000000000000000000000000000000
1111111110000000000000000000000000000000000000000000000011111111111
>B1YKR8_3d2l_0_241
MAYEQFAYVYDELMQDVPYPEWVAWVLEQVEPGKRIADIGCGTGTATLLLADHYEVTGV
DLSEEMLEIAQEKAMETNRHVDFWVQDMRELELPEPVDAITILCDSLNYLQTEADVKQTF
DSAARLLTDGGKLLFDVHSPYKMETLFNGKTYATHAEQSSYIWFADPGEEPLSVVHELTFFI
EGEDGRYDRVDETHHQRTYPPEQYITWLREAGFRVCAVTGDFKSDAPTETAERIFFVAEKI
11110000000000000000000000000000000000000000000000000000000000000000000000000000000
00000000000000000000000000000000000000000000000000000000000000000000000000000000000
0000000000000000000000000000000000000000000000000000000000000000000000000000
>Q07960_1ow3_197_438
HVKLEQLGIPRQVLKYDDFLKSTQKSPATAPKPMPPRPPLPNQQFGVSLQHLQEKNPEQEPI
PIVLRETVAYLQAHALTTEGIFRRSANTQVVREVQQKYNMGLPVDFDQYNELHLPAVILKT
FLRELPEPLLTFDLYPHVVGFLNIDESQRVPATLQVLQTLPEENYQVLRFLTAFLVQISAHSDQ
NKMTNTNLAVVFGPNLLWAKDAAITLKAINPINTFTKFLLDHQGELFFPSPDPSGL
11111111111111111111111111111111111111000000000000000000000000000000000000000000000
00000000000000000000000000000000000000000000000000000000000000000000000000000000000
00000000000000000000000000000000000000000000000000000000000000000000000011111111
>Q12318_4eq6_0_241
MEVLKNIRIYPLSNFITSTKNYINLPNELRNLISEEQESKLGFLHIIESDFKPSVALQKLVNCTT
GDEKILIIDIVSIWSQQKQRQHGAIYMNSLSCINITGLIVFLELLYDSPMDALRRCQVDNFNF
QLRGIVIDNLSFLNFESDKNYDVINLSKFEKLFKILRKLREFLGCWIITKSFPTDFYNGIENTLV
DKWSIKRKSGVTLYPTKLPDSYMKGMDLIIYREVVDGRPQYRRIAALEE
00000000000000000000000000000000000000000000000000000000000000000000000000000000000
00000000000000000000000000000000000000100000000000000000011111111110000000000000000
000000000000000000000000000111111111111100000000000000000000110000000001111
>Q58WP5_3jqy_44_285
RLKTQDSRLKTQDSRLKTQDSRLKTQDSFSVDDNGSGNVFVCGDLVNSKENKVQFNGNN
NKLIIEDDVECRWLTVIFRGDNNYVRIHKNSKIKGDIVATKGSKVIIGRRTTIGAGFEVVTDK
CNVTIGHDCMIARDVILRASDGHPIFDIHSKKRINWAKDIIISSYVWVGRNVSIMKGVSVGS
GSVIGYGSIVTKDVPSMCAAAGNPAKIIKRNIIWARTDKAELISDDKRCSSYHAKLTQ
11111111111111111111111000000000000000000000000000000000000000000000000000000000000
00000000000000000000000000000000000000000000000000000000000000000000000000000000000

0000000000000000000000000000000000000000000000000000000000000000000000000000000000000
>Q881P0_3ewn_33_274
HPHAMPDMGPDMNKVPWMGDEQIAMLVYPGMTVMDLVGPHCMFGSLMGAKIYIVAKS
LDPVTSDAGLAIVPTATFGTCPRDLTVLFAPGGTDGTLAAASDAETLAFMADRGARAKYIT
SVCSGSLILGAAGLLKGYKATSHWSCRDALAGFGAIPTEARVVRDRNRITGAGVTAGLDFG
LSMVAELRDQTYAECAQLMSEYDPDPPFNAGSMKTAPAHVRTAMIELVAEFTKKADALAG
FPK
000000000000000000000000000000000000000000000000000000000000000000000000000000000000000000000
000000000000000000000000000000000000000000000000000000000000000000000000000000000000000000000
0000000000000000000000000000000000000000000000000000000000000000000000000000000000011
>Q8U3S5_1vk1_0_241
MGVEKVPKYDIPVKKVEYVFIELDKMKPHEQLVQRELEDFIESVTGSGIFWKPMLLAKIPGT
DEYLIVDGHHRWAGLQKLGAKRAPSVILDYFDEGVKVYTWYPAFKGDVNKVIERLKAEGL
EVIEDEKAEEKAEKGEIAFALIGEKSFAIPGGLEEQKKVSKVLDEMDQAKEIELVYYGLKEDA
KADMEKGEIDYVFIRKAPTKEEVMELVKRGEVFSPKTTRHVLPFIPDKIDVKLEDLF
111111111100000000000000000000000000000000000000000000000000000000000000000000000000000000000
000000000000000000000000000000000000000000000000000000000000000000000000000000000000000000000
0000000000000000000000000000000000000000000000000000000000000000000000000000000000000
>Q98F67_3bkw_0_241
MAQNIYDQPDFFAGYSQLGRSIEGLDGAAEWPALRAMLPEVGGLRIVDLGCGFGWFCRW
AHEHGASYVLGLDLSEKMLARARAAGPDTGITYERADLDKLHLPQDSFDLAYSSLALHYV
EDVARLFRTVHQALSPGGHFVFSTEHPIYMAPARPGWAIDAEGRRTWPIDRYLVEGPRKTD
WLAKGVVKHHRTVGTTLNALIRSGFAIEHVEEFCPTDAQITARPELAEELDRPMFLLVSARR
111111111111111111111110000000000000000000000000000000000000000000000000000000000000000000000
000000000000000000000000000000000000000000000000000000000000000000000000000000000000000000000
0000000000000000000000000000000000000000000000000000000000000000000000000000000000000
>Q99YS3_3vzh_0_241
MYRSRDFYVRVSGQRALFTNPATKGGSERSSYSVPTRQALNGIVDAIYYKPTFTNIVTEVKVI
NQIQTELQGVRALLHDYSADLSYVSYLSDVVYLIKFHFVWNEDRKDLNSDRLPAKHEAIME
RSIRKGGRRDVFLGTRECLGLVDDISQEEYETTVSYYNGVNIDLGIMFHSFAYPKDKKTPLKS
YFTKTVMKNGVITFKAQSECDIVNTLSSYAFKAPEEIKSVNDECMEYDAMEKGEN
000000000000000000000011111100000000000000000000000000000000000000000000000000000000111111111
100000000000000000000000000000000000000000000000000000000000000000000000000000000000000000000
0000000000001111000000000000000000000000000000000000011111111111111111111111111111111
>Q9BUP3_2bka_0_241
MAETEALSKLREDFRMQNKSVFILGASGETGRVLLKEILEQGLFSKVTLIGRRKLTFDEEAYK
NVNQEVVDFEKLDDYASAFQGHDVGFCCLGTTRGKAGAEGFVRVDRDYVLKSAELAKAG
GCKHFNLLSSKGADKSSNFLYLQVKGEVEAKVEELKFDRYSVFRPGVLLCDRQESRPGEWL
VRKFFGSLPDSWASGHSVPVVTVVRAMLNNVVRPRDKQMELLENKAIHDLGKAHGSLKP
111100000000000000000000000000000000000000000000000000000000000000000000000000000000000000000
000000000000000000000000000000000000000000000000000000000000000000000000000000000000000000000
0000000000000000000000000000000000000000000000000000000000000000000000000000000111111
>A0RD96_3l8d_1_241
KVGECMTKFNWHESAEKKWDSSAEFWNQNSQEMWDSGSRSTIIPFFEQYVKKEAEVLDV
GCGDGYGTYKLSRTGYKAVGVDISEVMIQKGKERGEGPDLSFIKGDLSSLPFENEQFEAIMA
INSLEWTEEPLRALNEIKRVLKSDGYACIAILGPTAKPRENSYPRLYGKDVVCNTMMPWEFE
QLVKEQGFKVVDGIGVYKRGVNEKMLGQLSTDLQQSLTFLWVFMLKRHKEMKEFLGGK
111111111111111111111111111111000000000000000000000000000000000000000000000000000000000000000
000000000000000000000000000000000000000000000000000000000000000000000000000000000000000000000
000000000000000000000000000000000000000000000000000000000000000000000000000111111111
>A1S6W5_3bos_0_240

MRSNRVTQHPPLQLSLPVHLPDDETFTSYYPAAGNDELIGALKSAASGDGVQAIYLWGPVK
SGRTHLIHAACARANELERRSFYIPLGIHASISTALLEGLEQFDLICIDDVDAVAGHPLWEEAI
FDLYNRVAEQKRGSLIVSASASPMEAGFVLPDLVSRMHWGLTYQLQPMMDDEKLAALQRR
AAMRGLQLPEDVGRFLLNRMARDLRTLFDVLDRLDKASMVHQRKLTIPFVKEMLRL
11111111110000000000000000000000000000000000000000000000000000000000000000000000000
00000000000000000000000000000000000000000000000000000000000000000000000000000000000
00000000000000000000000000000000000000000000000000000000000000000000000000
>C7F6X3_3uyu_20_260
QRDLSVELGVASNFAILAKAGISSVPDSAILGDIGVSPAAATYITGFGLTQDSSTTYATSPQVT
GLIYAADYSTPTPNYLAAAVANAETAYNQAAGFVDPDFLELGAGELRDQTLVPGLYKWTSS
VSVPTDLTFEGNGDATWVFQIAGGLSLADGVAFTLAGGANSTNIAFQVGDDVTVGKGAH
FEGVLLAKRFVTLQTGSSLNGRVLSQTEVALQKATVNSPFVPAPEVVQKRSNARQWL
00000000000000000000000000000000000000000000000000000000000000000000000000000000000
00000000000000000000000000000000000000000000000000000000000000000000000000000000000
00000000000000000000000000000000000000000000000000000000000000000111111111
>O58271_2e8g_0_240
MWDTSKDYRLLVAEKSVELFIRTIEGAKFRGQWDKKRSIQLAKEMIPDIQALRYSYIDPEELV
DTPQMKDLKEKAKGIIEALGGEDWHHKFLSQASREDREKVEEQVARIKFFLNTILNLDRRL
KLGKINDPVIAVDIVVGEVMSVGKHPSADRLLVTNVNIGERAVTVVTNDLTVKEGNRVAVA
LLPPRNFFGIVSEGMFLGAGEGVLKNVKGEIGGLPKGIPLEALNETRNAVEAFLKG
00000000000000000000000000000000000000000000000000000000000000000000000000000000000
00000000000000000000000000000000000000000000000000000000000000000000000000000000000
00000000000000000000000000000000000000000000000000000000000000000000000001
>P20749_1k1a_126_366
MATRADEDGDTPLHIAVVQGNLPAVHRLVNLFQQGGRELDIYNNLRQTPLHLAVITTLPSV
VRLLVTAGASPMALDRHGQTAAHLACEHRSPTCLRALLDSAAPGTLDLEARNYDGLTALH
VAVNTECQETVQLLLERGADIDAVDIKSGRSPLIHAVENNSLSMVQLLLQHGANVNAQMY
SGSSALHSASGRGLLPLVRTLVRSGADSSLKNCHNDTPLMVARSRRVIDILRGKATRPAS
11111100000000000000000000000000000000000000000000000000000000000000000000000000000
00000000000000000000000000000000000000000000000000000000000000000000000000000000000
00000000000000000000000000000000000000000000000000000000000000000001111111
>P38659_3ec3_282_522
PPSKEILTLKQVQEFLKDGDDVVILGVFQGVGDPGYLQYQDAANTLREDYKFHHTFSTEIA
KFLKVSLGKLVLMQPEKFQSKYEPRMHVMDVQGSTEASAIKDYVVKHALPLVGHRKTSND
AKRYSKRPLVVVYYSVDFSFDYRTATQFWRNKVLEVAKDFPEYTFAIADEEDYATEVKDLGL
SESGEDVNAAILDESGKKFAMEPEEFDSDALQEFVMAFKKGKLKPVIKSQPVPKNNKG
00000000000000000000000000000000000000000000000000000000000000000000000000000000000
00000000000000000000000000000000000000000000000000000000000000000000000000000000000
00000000000000000000000000000000000000000000000000000000000000011111111111
>Q30Y26_3cgx_2_242
MSESCILFFVKYPEPGKVKTRLGEVVGNDKAAMLYRHFVQDMLQGLARLHADLHICYVPG
DADLPEKFKAWLGPQHMFAAQQGLDLGERMKHAMQKAFDDGYDRVVLMGSDIPDYPC
ELVQKALNDLQHYDAAIGPAFDGGYYLIGFRKDSFCPDVFDGIRWGEADVYQPTVEKMRR
ARLEVLQLPDWNDVDTVWDLNVLYRTNKNSSFRRSSTYALLRENDALIRQYDIDLPGMAP
VEKE
00000000000000000000000000000000000000000000000000000000000000000000000000000000000
00000000000000000000000000000000000000000000000000000000000000000000000000000000000
00000000000000000000000000000000000000000000000000000000000000001111111111
>Q6VBJ0_4afc_30_270
TSSNDISLASKDPTTFPLGCSPDITTPKKGLSMELYSYDFRKKGSYPCWDAAYLDPNYPRTGY
KSHRLLAKVDGVTGNINFYYHATKGCTPQLGHLPASYNYPKPLTMTNFTMLLYGYFRPKV

TGFHTFTISADDLLFVNFGAGNAFDCCRRDSSADHFGNYQAYAIWGSKTAKDELTVHLDA
GVYYPIRLFYNNREYDGALSFTFKTESNENTVSDFSEYFFSLDDTEEGCPGLISYDSS
11111110000000000000000000000000000000000000000000000000000000000000000000000000000
00000000000000000000000000000000000000000000000000000000000000000000000000000000000
000000000000000000000000000000000000000000000000000000000000000000000001111
>Q9J0W2_3mmg_0_240
SKALLKGVRDFNPISACVCLLENSSDGHSERLFGIGFGPYIIANQHLFRRNNGELTIKTMHG
EFKVKNSTQLQMKPVEGRDIIVIKMAKDFPPFPQKLKFRQPTIKDRVCMVSTNFQQKSVSSL
VSESSHIVHKEDTSFWQHWITTKDGQCGSPLVSIIDGNILGIHSLTHTTNGSNYFVEFPEKFV
ATYLDAADGWCKNWKFNADKISWGSFTLVEDAPEDDFMAKKTVAAIMDDLVRTQ
00000000000000000000000000000000000000000000000000000000000000000000000000000000000
00000000000000000000000000000000000000000000000000000000000000000000000000000000000
000000000000000000000000000000000000000000000000000111111111111111111111111
>B2Z3V8_3smv_0_239
MQLTDFKALTFDCYGTLIDWETGIVNALQPLAKRTGKTFTSDELLEVFGRNESPQQTETPG
ALYQDILRAVYDRIAKEWGLEPDAAEREEFGTSVKNWPAFPDTVEALQYLKKHYKLVILSNI
DRNEFKLSNAKLGVEFDHIITAQDVGSYKPNPNNFTYMIDALAKAGIEKKDILHTAESLYH
DHIPANDAGLVSAWIYRRHGKEGYGATHVPSRMPNVDFRFNSMGEMAEAHKQALKG
00000000000000000000000000000000000000000000000000000000000000000000000000000000000
00000000000000000000000000000000000000000000000000000000000000000000000000000000000
00000000000000000000000000000000000000111111000000000000000000000000000000
>P54302_1zhh_38_277
YFSKQIIWQEVDRTKQQTSALIHNIFDSHFAAIQIHHDSNSKSEVIRDFYTDRDTDVLNFFFL
SIDQSDPSHTPEFRFLTDHKGIIWDDGNAHFYGVNDLILDSLANRVSFSNNWYYINVMTSIG
SRHMLVRRVPILDPSTGEVLGFSFNAVVLDNNFALMEKLKSESNVDNVVLVANSVPLANSLI
GDEPYNVADVLQRKSSDKRLDKLLVIETPIVVNAVTTELCLLTVQDNQSVVTL
11111111111100000000000000000000000000000000000000000000000000000000000000000000000
00000000000000000000000000000000000000000000000000000000000000000000000000000000000
00000000000000000000000000000000111111111111110000000000000000000001111111
>P56106_4a7w_0_239
MQAKIKNKRVLVKFSGEALAGDNQFGIDIHVLDHIAKEIKSLVENDIEVGIVIGGGNIIRGVS
AAQGGIIRRTSGDYMGMLATVINAVAMQEALEHIGLDTRVQSAIEIKEICESYIYRKAIRHLE
KGRVVIFGAGTGNPFFTTDTAATLRAIEIGSDLIIKATKVDGIYDKDPNKFKDAKKLDTLSYN
DALIGDIEVMDDTAISLAKDNKLPIVVCNMFKKGNLLQVIKHQQGVFSMVK
11111000000000000000000000000000000000000000000000000000000111111110000000000000000
00000000000000000000000000000000000000000000000000000000000000000000000000000000000
00000000000000000000000000000000000000000000000000000000000000000000000000
>P84136_1sfs_0_239
MHHHHHHSSGVDLGTENLYFQSNAMARGIWGVDSAQVVTDQLFQCVRTELGYPKFWGR
YLSEVPNVSEGLTRDEIVRIRNYGVKVLPIYNAFREAVGYANGQVAARNAVFHARRLGIPK
NKLLFANIEDFFAVDAAWIAAWVETLYPTGYRPGLYADPTKGDFAAAYCEAVSRNNQVAV
QAVIWSAAPRPGTTKEQKAPRYQPAAPPCSANVWVWQYGRDAEVCPVDTNLADRRLLDF
LY
11111111111111111111111111110000000000000000000000000000000000000000000000000000000
00000000000000000000000000000000000000000000000000000000000000000000000000000000000
00000000000000000000000000000000000000000000000000000000000000000000000000
>Q05115_3ixm_0_239
MQQASTPTIGMIVPPAAGLVPADGARLYPDLPFIASGLGLGSVTPEGYDAVIESVVDHARRL
QKQGAAVVSLMGTSLSFYRGAAFNAALTVAMREATGLPCTTMSTAVLNGLRALGVRRVAL
ATAYIDDVNERLAAFLAEESLVPTGCRSLGITGVEAMARVDTATLVDLCVRAFEAAPDSDGI
LLSCGGLLTLDAIPEVERRLGVPVVSSSPAGFWDAVRLAGGGAKARPGYGRLFDES

11111100000000000000000000000000000000000000000000000000000000000000000000000000000
00000000000000000000000000000000000000000000000000000000000000000000000000000000000
0000000000000000000000000000000000000000000000000000000000000000000000001
>Q12046_3u1l_0_239
MTSWRDKSAKVQVKESELPSSIPAQTGLTFNIWYNKWSQGFAGNTRFVSPFALQPQLHSGK
TRGDNDGQLFFCLFFAKGMCCLGPKCEYLHHIPDEEDIGKLALRTEVLDCFGREKFADYRE
DMGGIGSFRKKNKTLYVGGIDGALNSKHLKPAQIESRIRFVFSRLGDIDRIRYVESKNCGFVK
FKYQANAEFAKEAMSNQTLLLPSDKEWDDRREGTGLLVKWANEDPDPAAQKRLQE
11000000000000000000000000000000000000000000000000000000000000000000000000000000000
00000000000000000000000000000000000000111111111000000000000000000000000000000000000
0000000000000000000000000000000000000000000000000000000000001111111111111
>A1SAQ1_3mw8_0_238
MKLLLTRPEGKNAAMASALDALAIPYLVEPLLSVEAAAVTQAQLDELSRADILIFISTSAVSF
ATPWLKDQWPKATYYAVGDATADALALQGITAERSPADSQATEGLLTLPSLEQVSGKQIVIV
RGKGGREAMADGLRLRGANVSYLEVYQRACPPLDAPASVSRWQSFGIDTIVVTSGEVLEN
LINLVPKDSFAWLRDCHIIVPSARVETQARKKGLRRVTNAGAANQAAVLDALGM
00000000000000000000000000000000000000000000000000000000000000000000000000000000000
00000000000000001110000000000000000000000000000000000000000000000000000000000000000
000000000000000000000000000000000000000000000000000000000000000000000000
>A9CKP4_2fsq_0_238
MHAPLVSKDLDYISTANHDQPPRHLGSRFSAEGEFLPEPGNTVVCHLVEGSQTESAIVSTRQ
RFLDMPEASQLAFTPVSSLHMTVFQGVIESRRALPYWPQTLPLDTPIDAVTDYYRDRLSTFPT
LPAFNMRVTGLRPVGMVMKGATAEDDSIVALWRDTFADFFGYRHPDHDTYEFHITLSYIVS
WFEPECLPRWQAMLDEELEKLRVAAPVIQMRPPAFCEFKDMNHFKELVVFDKR
11111000000000000000000000000000000000000000000000000000000000000000000000000000000
00000000000000000000000000000000000000000000000000000000000000000000000000000000000
000000000000000000000000000000000000000000000000000000000000000000000011
>C8WQT1_3rag_0_238
MTREATIRQILVITDGCSNIGPDPVEAARRAHRHGIVVNVIGIVGRGDAGEQGYQEAHSIAD
AGGGMCRIVQPADISATAQMMTHQTMQMTLQQVVNQELLAVMGKSTEDLPPADRARVM
QVVEKLEDEVALHLVVCLDTSASMRDKIPTVREAVRDLALSLKVRSGPLAVSVIAFPGKGEE
ATRLVQPFSSEVNVAALEAELVARGGTPTGPAIDHAADLLLSHARNVDEDAPRREFG
00000000000000000000000000000000000000000000000000000000000000000000000000000000000
00000000000000000000000000000000000000000000000000000000000000000000000000000000000
000000000000000000000000000000000000000000000000000000000000000111111111
>D1C2D8_3pu9_0_238
MTPMPVTIELAVAKTHKFGTRESGDTVELVERPGGGFSAVLVDGQGSGAGAKRLSLLVAGA
AVRLLNEGVRDGAAARAAHDFLYAMRDGKVSAALDILSVDLASRSVLVTRNSEVPMLLGR
NGEFEQISESGGRIGIYRHTRPRVLEFPAEPGLTVILVSDGIIGAGGRRGQPLEFLATGGRVAG
PETPAQAIADELLEAALVADDGRAGDDMTVVVLRLRNVEEVEPIRRMALTVPLG
11100000000000000000000000000000000000000000000000000000000000000000000000000000000
00000000000000000000000000000000000000000000000000000000000000000000000000000000000
000000000000000000000000000000000000000000000000000000000000000000000000
>E5EVW2_4efp_17_255
ADSDVPNDILEEQLYNSIVVADYDSAVEKSKHLYEEKKSEVITNVVNKLIRNNKMNCMEYA
YQLWLQGSKDIVRDCFPVEFRLIFAENAIKLMYKRDGLALTLSNDVHGNDGRLAFGDGKD
KTSPKVSWKFIALWENNKVYFKILNTERNQYLVLGVGTNPNGDHMAFGVNSVDSFRAQW
YLQPAKYDKDNLFYIYNREYSKALTLSRTLETSGNRMAWGYNGRVIGSPEHYAWGVKAF
11110000000000000000000000000000000000000000000000000000000000000000000000000000000
00000000000000000000000000000000000000000000000000000000000000000000000000000000000
000000000000000000000000000000000000000000000000000000000000000000000000

>P25813_1xdz_0_238
MNIEEFTSGLAEKGISLSPRQLEQFELYYDMLVEWNEKINLTSITEKKEVYLKHFYDSITAAFY
VDFNQVNTICDVGAGAGFPSLPIKICFPHLHVTIVDSLNKRITFLEKLSEALQLENTTFCHDR
AETFGQRKDVRESYDIVTARAVARLSVLSELCLPLVKKNGLFVALKAASAEEELNAGKKAIT
TLGGELENIHSFKLPIEESDRNIMVIRKIKNTPKKYPRKPGTPNKSPIEG
00000000000000000000000000000000000000000000000000000000000000000000000000000000000
00000000000000000000000000000000000000000000000000000000000000000000000000000000000
0000000001000000000000000000000000000000000000000000000000000000000000001
>Q9H4Y5_3q19_0_238
MSGDATRTLGKGSQPPGPVPEGLIRIYSMRFCPYSHRTRLVLKAKDIRHEVVNINLRNKPEW
YYTKHPFGHIPVLETSQCQLIYESVIACEYLDDAYPGRKLFPYDPYERARQKMLLELFCKVP
HLTKECLVALRCGRECTNLKAALRQEFSNLEEILEYQNTTFFGGTCISMIDYLLWPWFERLD
VYGILDCVSHTPALRLWISAMKWDPTVCALLMDKSIFQGFLNLYFQNNPNAFD
11110000000000000000000000000000000000000000000000000000000000000000000000000000000
00000000000000000000000000000000000000000000000000000000000000000000000000000000000
0000000000000000000000000000000000000000000000000000000000000000000000000
>A5W059_3noj_0_237
MNTLIGKTGIVVRNIQRAELDSIDALGRLGVATVHEAQNRKGLLSSKMRPIQQGTSLAGSA
VTVLVAPGDNWMFHVAVEQCRPGDVLVVSPSSPCTDGYFGDLLATSLQARGVRALIVDAG
VRDTQTLRDMGFAVWARAINAQGTVKETLGSVNLPVICGGQLINPGDIVVADDDGVVVVR
RDECESTLVAAAERAGLEEEKRLRLAAGELGLDIYKMRERLEAKGLRYVDNIEDLEG
10000000000000000000000000000000000000000000000000000000000000000000000000000000000
00000000000000000000000000000000000000000000000000000000000000000000000000000000000
000000000000000000000000000000000000000000000000000000000000000000000011
>Q16KU7_4fd7_0_237
MKWTRSVKVPFPSVWHRFQAKDLTSQQLVWYRVQDLPEDRFEDAIRHMCDYFARDELMN
QAKGLAKDLVAMGDVVALWKAMLPDRMSLVCFREGSDEIVGVNILDVASRSDKDNAQFN
SAIFQAIYDTIEYVSHQANIFDRYNVDHYLNAMGLSVDPKYRGRGIATEILRARIPLCRAVGL
KLSATCFTGPNSQTAATRVGFQEDFTITYGELARVDQRFNYPGIEENFCKYMSLRVD
10000000000000000000000000000000000000000000000000000000000000000000000000000000000
00000000000000000000000000000000000000000000000000000000000000000000000000000000000
000000000000000000000000000000000000000000000000000000000000000000000000
>Q6RUA7_2wnk_26_263
PEATSCETEGSSISFTVEKAGHVVRFNCPSTLEEIKPAYEAGDSTKVCTTADCSNEAALKDVL
KSASLAQAEGSGPSGGNDFTLTVDALPEAETSVFFLCQRTGASRSARRLGTAVPSDKCGVHI
LVKAAPQAPVCSAQDHTLELQITAANSDTSFVCGGTFNVIKPANAAKVLQGDSCETEVDL
VSLVPHASRSALEQSGLIKLSVTDLPQQQQKLCYRCEDSSQKACKVLVTVSAS
11100000000000000000000000000000000000000000000000000000000000000000000001110000000
00000000000000000000001111111111000000000000000000000000000000000000000000000000000
000000000000000000000000000000000000000000000000000000000000000000000000
>Q6ZYJ2_2x3m_0_237
MSAFDEFNEGFGLDVSDTPEELAFETESAIEEIESETSPGDQPKGSEPEEIRVWAEEKARKAVE
EGREVTNWADWIMGWRTPNASEKKMEFMYWYTRTYLEEAKDIRPDIADALARGMAGLA
FGRTDWVASMLDPQIMRHIYTDPEVARIYSETRDMLRRVSDYYISLTTMELGKVADIIAEAK
AKGENPEVVAREIAEAVPRLSPKSLYFNLYYIGRSIGDNYVLEVARVLSKMRRR
11111111111111111111111111111111111111111111111111111111100000000000000000000000000
00000000000000000000000000000000000000000000000000000000000000000000000000000000000
000000000000000000000000000000000000000000000000000000000000000000000111
>Q84T68_3hkm_0_237
MEESRADGRNPNQLRPFSCTRNPLDRAHGSARWAQGDTIVLAAVYGPKPGTRKGENPEK
ASIEVVWKPMTGQIGKQEKEYEMTLKRTLQSICLLTVHPNTTTSVILQVVGNDGSLLPCAIN

ACCAALVFAGIPLKHLAVAIGCGVLEDGEVILDTNKAEEQQLKSFAHLVFPNSRKSASSKEP
NQKEEDSERGLITSITHGVMSEEDYFSCIERGLAASSRISDFMRTTLQKQAPGDV
11110000000000000000000000000000000000000000000000000000000000000000000000000000000
00000000000000000000000000000000000000000000000000000000000000000000000000000000000
000000011111111111111111111000000000000000000000000000000000000000111111
>Q88E10_2yfa_45_282
MGDIGQLNKDLTDLRIARLQYMIANGDDTAAANTLAKLDAFSKQQAYLATTFKSPENVKL
LGELGDTISAYKLSLNKMRQGYDATRAARVSMDSSAIRADQAMDALSQEVMARPEADSVR
LAQYQLISKARQQLLQVRIDVRGYIAENSSANEQAALRQLDAALADTDNLKRQLPSEDAR
LQQFENAVLAYRDAVRQFRDAVANITTSRAEMTVQGADIVKRSDALYQIQLERRDIES
00000000000000000000000000000000000000000000000000000000000000000000000000000000000
00000000000000000000000000000000000000000000000000000000000000000000000000000000000
000000000000000000000000000000000000000000000000000000000000000000011111
>Q8GED7_3oqv_1_238
LAGLVPAPDHGMREEILGDRSRLIRQRGEHALIGISAGNSYFSQKNTVMLLQWAGQRFERT
DVVYVDTHIDEMLIADGRSAQEAERSVKRTLKDLRRRLRRSLESVGDHAERFRVRSLSELQE
TPEYRAVRERTDRAFEEDAEFATACEDMVRAVVMNRPGDGVGISAEHLRAGLNYVLAEAP
LFADSPGVFSVPSSVLCYHIDTPITAFLSRRETGFRAAEGQAYVVVRPQELADAA
11111111111111111111111111000000000000000000000000000000000000000000000000000000000
00000000000000000000000000000000000000000000000000000000000000000000000000000000000
000000000000000000000000000000000000000000000000010000000000000000000000
>Q8IUH5_3eu9_50_287
THIDDYSTWDIVKATQYGIYERCRELVEAGYDVRQPDKENVTLLHWAAINNRIDLVKYYIS
KGAIVDQLGGDLNSTPLHWATRQGHLSMVVQLMKYGADPSLIDGEGCSCIHLAAQFGHT
SIVAYLIAKGQDVDMMDQNGMTPLMWAAYRTHSVDPTRLLLTFNVSVNLGDKYHKNTAL
HWAVLAGNTTVISLLLEAGANVDAQNIKGESALDLAKQRKNVWMINHLQEARQAKGYD
N
11000000000000000000000000000000000000000000000000000000000000000000000000000000000
00000000000000000000000000000000000000000000000000000000000000000000000000000000000
000000000000000000000000000000000000000000000000000000000000000001111111
>Q8RNV8_2odh_0_237
MKIWSKEEVVNKLHEIKNKGYLSVPTDMFRTDDGVVGQILERQFGVQENNITLGDLGEFEL
KGMRNRKAKSNLTLFHKKPVAGQTVIQIFNRFGYVKPSSRNPEVMKKKLFTTIKGGRLNNL
GLTLNAKHASEINLYYQDEYLSTWDLNLSKIEKLVLVFAETIGRANSPEEQFHFTKAYMLTEI
NDITSLINDGVLVMDLCIDQDLSKSKGPHDRGPHLRIPISKLDKLYRNIERLL
00000000000000000000000000000000000000000000000000000000000000000000000000000000000
00000000000000011111000000000000000000000000000000000000000000000000000000000000000
000000000000000000000000000000000000000000000000000000000000000000000000
>Q8TE30_1vyb_0_237
MTGSNSHITILTLNINGLNSAIKRHRLASWIKSQDPSVCCIQETHLTCRDTHRLKIKGWRKIY
QANGKQKKAGVAILVSDKTDFKPTKIKRDKEGHYIMVKGSIQQEELTILNIYAPNTGAPRFI
KQVLSDLQRDLDSHTLIMGDFNTPLSTLDRSTRQKVNKDTQELNSALHQADLIDIYRTLHP
KSTEYTFFSAPHHTYSKIDHIVGSKALLSKCKRTEIITNYLSDHSAIKLELR
11000000000000000000000000000000000000000000000000000000000000000000000000000000000
00000000000000000000000000000000000000000000000000000000000000000000000000000000000
000000000000000000000000000000000000000000000000000000000000000000000000
>Q96RI1_3bej_248_485
TTKSCREKTELTPDQQTLLHFIMDSYNKQRMPQEITNKILKEEFSAEENFLILTEMATNHVQ
VLVEFTKKLPGFQTLDHEDQIALLKGSAVEAMFLRSAEIFNKKLPSGHSDLLEERIRNSGISD
EYITPMFSFYKSIGELKMTQEEYALLTAIVILSPDRQYIKDREAVEKLQEPLLDVLQKLCKIHQ
PENPQHFACLLGRLTELRTFNHHHAEMLMSWRVNDHKFTPLLCEIWDVQ

11111111100000000000000000000000000000000000000000000000000000000000000000000000000
00000000000000000000000000000000000000000000000000000000000000000000000000000000000
000000000000000000000000000000000000000000000000000000000000000000000001
>Q9X017_2etj_0_237
MGIDELYKKEFGIVAGVDEAGRGCLAGPVVAAAVVLEKEIEGINDSKQLSPAKRERLLDEIM
EKAAVGIGIASPEEIDLYNIFNATKLAMNRALENLSVKPSFVLVDGKGIELSVPGTCLVKGDQ
KSKLIGAASIVAKVFRDRLMSEFHRMYPQFSFHKHKGYATKEHLNEIRKNGVLPIHRLSFEP
VLELLTDDLLREFFEKGLISENRFERILNLLGARKSVVFRKERTNHNLPLF
00000000000000000000000000000000000000000111111111100000000000000000000000000000000
00000000000000000000000000000000000000000000000000000000000000000000000000000000000
000000000000000000000000000000000000000000000000000000011111111111111111
>O69639_3k6y_160_396
SALLNTSGLPAVLEPFSRTPVIPVASPDPALVNNPVVAATEPSVVKIRSLAPRCQKVLEGTGFV
ISPDRVMTNAHVVAGSNNVTVYAGDKPFEATVVSYDPSVDVAILAVPHLPPPLVFAAEPA
KTGADVVVLGYPGGGNFTATPARIREAIRLSGPDIYGDPEPVTRDVYTIRADVEQGDSGGPL
IDLNGQVLGVVFGAAIDDAETGFVLTAGEVAGQLAKIGATQPVGTGACVS
11111111110000000000000000000000000000000000000000000000000000000000000000000000000
00000000000000000000000000000000000000000000000000000000000000000000000000000000000
00000000000000000000000000000000000000000000000000000000000000000000000
>Q01269_3kbr_24_260
AQESRLDRILESGVLRVATTGDYKPFSYRTEEGGYAGFDVDMAQRLAESLGAKLVVVPTSW
PNLMRDFADDRFDIAMSGISINLERQRQAYFSIPYLRDGKTPITLCSEEARFQTLEQIDQPGV
TAIVNPGGTNEKFARANLKKARILVHPDNVTIFQQIVDGKADLMMTDAIEARLQSRLHPEL
CAVHPQQPFDFAEKAYLLPRDEAFKRYVDQWLHIAEQSGLLRQRMEHWLEYR
11000000000000000000000000000000000000000000000000000000000000000000000000000000000
00000000000000000000000000000000000000000000000000000000000000000000000000000000000
00000000000000000000000001000000000000000000000000000000000000000000111
>Q88AY9_3myx_0_236
MPQPTVLLLARADLSPVGTEFTTGPIDAHDPFDSGRRTAFVDEQGIAAGIVEFGTALSVEAY
PYTEMLVMHRGSVTLTSGTDSVTLSTGESAVIGRGTQVRIDAQPESLWAFCASTQASGPDKS
GITALDRLALLTPSSPPDPSIMISPLPQCRSNNLFEDTASTLRIGVWDSTPYERISRPHKIHELM
NLIEGRVVLSLENGSSLTVNTGDTVFVAQGAPCKWTSTGYVRKFYAVT
11000000000000000000000000000000000000000000000000000000000000000000000000000000000
00000000000000000000000000000000000000000000000000000000000000000000000000000000000
00000000000000000000000000000000000000000000000000000000000000000000000
>Q8XL08_2w1n_764_1000
VHGKLKEAAEVTGSVSLEALEEVQVGENIEVGVGIDELVNAEAFAYDFTLNYDENAFEYVE
AISDDGVFVNAKKIEDGKVRVLVSSLTGEPLPAKEVLAKVVLRAEAKTEGSNLSVTNSSVGD
GEGLVHEIAGTEKTVNIIEGTSPEIVVNPVRDFKASEINKKNVTVTWTEPETTEGLEGYILYK
DGKKVAEIGKDETSYTFKKLNRHTIYNFKIAAKYSNGEVSSKESLTLRTAR
11111111000000000000000000000000000000000000000000000000000000000000000000000000000
00000000000000000000000000000000000000000000000000000000000000000000000000000000000
00000000000000000000000000000000000000000000000000000000000000000000001
>Q97Y88_2wte_0_236
MKSYFVTMGFNETFLLRLLNETSAQKEDSLVIVVPSPIVSGTRAAIESLRAQISRLNYPPPRIYE
IEITDFNLALSKILDIILTLPEPIISDLTMGMRMINTLILLGIIVSRKRFTVYVRDEGGGSRVISFN
DNTIRALMRDYSREEMKLLNVLYETKGTGITELAKMLDKSEKTLINKIAELKKFGILTQKGK
DRKVELNELGLNVIKLNKSVIESSKSSEELVKENKGKEVNIPY
00000000000000000000000000000000000000000000000000000000000000000000000000000000000
00000000000000000000000000000000000000000000000000000000000000000000000000000000000
00000000000000000000000000000000000000000000000001111111111111111111111

>Q9NRY4_3c5h_12_248
TYNISVVGLSGTEKEKGQCGIGKSCLCNRFVRPSADEFHLDHTSVLSTSDFGGRVVNNDHF
LYWGEVSRSLEDCVECKMHIVEQTEFIDDQTFQPHRSTALQPYIKRAAATKLASAEKLMYF
CTDQLGLEQDFEQKQMPDGKLLVDGFLLGIDVSRGMNRNFDDQLKFVSNLYNQLAKTKK
PIVVVLTKCDEGVERYIRDAHTFALSKKNLQVVETSARSNVNVDLAFSTLVQLIDK
00000000000000000000000000000000000000000000000000000000000000000000111111111000000
00000000000000000000000000000000000000000000000000000000000000000000000001111000000
00000000000000000000000000000000000000000000000000000000000000000000000
>Q9RTX8_4eek_0_236
MPDAPFDAVLFDLDGVLVESEGIIAQVWQSVLAERGLHLDLTEIAMYFTGQRFDGVLAYLA
QQHDFVPPPDFLDVLETRFNAAMTGVTAIEGAAETLRALRAAGVPFAIGSNSERGRLHLKL
RVAGLTELAGEHIYDPSWVGGRGKPHPDLYTFAAQQLGILPERCVVIEDSVTGGAAGLAAG
ATLWGLLVPGHPHPDGAAALSRLGAARVLTSHAELRAALAEAGLLTPALTPDLS
11000000000000000000000000000000000000000000000000000000000000000000000000000000000
00000000000000000000000000000000000000000000000000000000000000000000000000000000000
00000000000000000000000000000000000000000000000000000000000000000111111
>A1B060_2r31_0_235
MSEWKARRFWASVGIHKEEGGWAVLLDERPLRTPGKQPLRLPTEALALAIAEEWQAVQEV
IDPNAMPLTRSANSAIEKVAPQFDAVAAMLGDYGGTDLLSYRADAPEALVRAQAEGWDPL
IDWAATELRAPLRITHGVIPVPQDPVVLLKLRAEVASLDPFGLTALHDLVTLPGSLILGLAVIR
GRIDAPTAHALSRIDEEFQAERWGRDEEAEAQAASRLAAMRDSERFWHLTRG
00000000000000000000000000000000000000000000000000000000000000000000000000000000000
00000000000000000000000000000000000000000000000000000000000000000000000000000000000
0000000000000000000000000000000000000000000000000000000000000000000001
>P0A114_1zi9_0_235
MLTEGISIQSYDGHTFGALVGSPAKAPAPVIVIAQEIFGVNAFMRETVSWLVDQGYAAVCPD
LYARQAPGTALDPQDERQREQAYKLWQAFDMEAGVGDLEAAIRYARHQPYSNGKVGLVG
YCLGGALAFLVAAKGYVDRAVGYYGVGLEKQLKKVPEVKHPALFHMGGQDHFVPAPSRQ
LITEGFGANPLLQVHWYEEAGHSFARTSSSGYVASAAALANERRLDFLAPLQSKKP
00000000000000000000000000000000000000000000000000000000000000000000000000000000000
00000000000000000000000000000000000000000000000000000000000000000000000000000000000
0000000000000000000000000000000000000000000000000000000000000000000111
>P16466_3fy3_29_264
NGIVPDAGHQGPDVSAVNGGTQVINIVTPNNEGISHNQYQDFNVGKPGAVFNNALEAGQ
SQLAGHLNANSNLNGQAASLILNEVVSRNPSFLLGQQEVFGIAAEYVLSNPNGITCDGCGF
INTSRSSLVVGNPLFENGQLKGYSTLNNTNLLSLGKNGLNTTGLLDLIAPRIDSRGKITAAEI
SAFTGQNTFSQHFDILSSQKPVSALDSYFFGSMQSGRIRIINTAEGSGVKLAG
00000000000000000000000000000000000000000000000000000000000000000000000000000000000
00000000000000000000000000000000000000000000000000000000000000000000000000000000000
0000000000000000000000000000000000000000000000000000000000000000000011
>Q14353_3orh_0_235
MSAPSATPIFAPGENCSPAWGAAPAAYDAADTHLRILGKPVMERWETPYMHALAAAASSK
GGRVLEVGFGMAIAASKVQEAPIDEHWIIECNDGVFQRLRDWAPRQTHKVIPLKGLWEDV
APTLPDGHFDGILYDTYPLSEETWHTHQFNFIKNHAFRLLKPGGVLTYCNLTSWGELMKSK
YSDITIMFEETQVPALLEAGFRRENIRTEVMALVPPADCRYYAFPQMITPLVTKG
11111100000000000000000000000000000000000000000000000000000000000000000000000000000
00000000000000000000000000000000000000000000000000000000000000000000000000000000000
0000000000000000000000000000000000000000000000000000000000000000000000
>Q7D120_2ozv_0_235
MDAMLLASLVADDRACRIADLGAGAGAAGMAVAARLEKAEVTLYERSQEMAEFARRSLE
LPDNAAFSARIEVLEADVTLRAKARVEAGLPDEHFHHVIMNPPYNDAGDRRTPDALKAE

AHAMTEGLFEDWIRTASAIMVSGGQLSLISRPQSVAEIIAACGSRFGGLEITLIHPRPGEDAV
RMLVTAIKGSRARLTFRAPLIMHETGSHAFTPFVDDLNNGRAAYARNVRAIRTAS
00000000000000000000000000000000000000000000000000000000000000000000000000000000000
00000000000000000000111111111111111111110000000000000000000000000000000000000000000
0000000000000000000000000000000000000000000000000000000000000000011111
>Q81Z49_2j13_24_259
AYTNTPHNWGIPRPKNETVPDAGKLYTDLLQKNGGFYLGDTKKKDIYLTFDNGYENGYTG
KILDVLKEKKVPATFFVTGHYIKTQKDLLLRMKDEGHIIGNHSWSHPDFTAVNDEKLREELT
SVTEEIKKVTGQKEVKYVRPPRGVFSERTLALTKEMGYYNVFWSLAFLDWKVDEQRGWQY
AHNNVMTMIHPGSILLLHAISKDNAEALAKIIDDLREKGYHFKSLDDLVKSNQP
00000000000111111111100000000000000000000000000000000000000000000000000000000000000
00000000000000000000000000000000000000000000000000000000000000000000000000000000000
0000001111111111111111110000000000000000000000000000000000000000000011
>Q8P2Q3_1pg6_0_235
MVDNRMRFTIDQNMQFPLVEIDLEHGGSVYLQQGSMVYHTENVTLNTKLNGKGSGLGKL
VGAIGRSMVSGESMFITQAMSNGDGKLALAPNTPGQIVALELGEKQYRLNDGAFLALDGS
AQYKMERQNIGKALFGGQGGLFVMTTEGLGTLLANSFGSIKKITLDGGTMTIDNAHVVAW
SRELDYDIHLENGFMQSIGTGEGVVNTFRGHGEIYIQSLNLEQFAGTLKRYLPTSSN
11110000000000000000000000000000000000000000000011110000000000000000000000000000000
00000000000000000000000000000000000000000000001111110000000000000000000000000000000
0000000000000000000000000000000000000000000000000000000000000000011111
>Q96T88_2pb7_407_642
KECTIVPSNHYGPIPGIPVGTMWRFRVQVSESGVHRPHVAGIHGRSNDGAYSLVLAGGYED
DVDHGNFFTYTGSGGRDLSGNKRTAEQSCDQKLTNTNRALALNCFAPINDQEGAEAKDW
RSGKPVRVVRNVKGGKNSKYAPAEGNRYDGIYKVVKYWPEKGKSGFLVWRYLLRRDDDE
PGPWTKEGKDRIKKLGLTMQYPEGYLEALANREREKENSKREEEEQQEGGFASPRTG
10000000000000000000000000000000000000000000000000000000000000000000000000001111111
11100000000000000000000000000000000000000000000000000000000000000000000000000000000
0000000000000000000000000000000000000000001111111111111111111111111111
>Q9FBN7_3cbt_0_235
MTDGEAVSAAAGPGAEGPPGPVGHWAPGSHILWRYRENGGPHVHIARPVTVVRDDADLL
AVWLAPGTECVKPVLADGTPVHLEPLATRYTKPRTVQRDQWFGTGVLKLARPGEAWSVW
LFWDPGWRFKNWYVNLERPLTRWEGGVDSEDHFLDISVHPDRTWHWRDEDEFAQALRD
GLMDPASAGRVRRAGRSAVAEIRAWGSPFADGWEHWRPDPAWPVPSLPGDWDRTPAHVS
S
11111111111111111111110000000000000000000000000000000000000000000000000000000000000
00000000000000000000000000000000000000000000000000000000000000000000000000000000000
0000000000000000000000000000000000000000000000000000000000000000001111
>Q9KFY3_4f3m_0_235
MRNEVQFELFGDYALFTDPLTKIGGEKLSYSVPTYQALKGIAESIYWKPTIVFVIDELRVMKPI
QMESKGVRPIEYGGGNTLAHYTYLKDVHYQVKAHFEFNLHRPDLAFDRNEGKHYSILQRS
LKAGGRRDIFLGARECQGYVAPCEFGSGDGFYDGQGKYHLGTMVHGFNYPDETGQHQLD
VRLWSAVMENGYIQFPRPEDCPIVRPVKEMEPKIFNPDNVQSAEQLLHDLGGE
00000000000000000000000000000000000000000000000000000000000000000000000000000000000
00000000000000000000000000000000000000000000000000000000000000000000000000000000000
0000000111100000000000000000000000000000000110000000110000000000000011
>Q9NQV5_3ray_78_313
DSSAMEVEPKKLKGKRDLIVPKSFQQVDFWFCESCQEYFVDECPNHGPPVFVSDTPVPVGI
PDRAALTIPQGMEVVKDTSGESDVRCVNEVIPKGHIFGPYEGQISTQDKSAGFFSWLIVDKN
NRYKSIDGSDETKANWMRYVVISREEREQNLLAFQHSERIYFRACRDIRPGEWLRVWYSED
YMKRLHSMSQETIHRNLARGEKRLQREKSEQVLDNPEDLRGPIHLSVLRQGK

11111111111111111111111111000000000000000000000000000000000000000000000000000000
00000000000000000000000011110000000000000000000000000000000000000000000000000000
0000000000000000000000001111111111111111111111111111111111111111111
>Q9XVJ3_1ooe_0_235
MSSGKVIVYGGKGALGSAILEFFKKNGYTVLNIDLSANDQADSNILVDGNKNWTEQEQSIL
EQTASSLQGSQVDGVFCVAGGWAGGSASSKDFVKNADLMIKQSVWSSAIAAKLATTHLKP
GGLLQLTGAAAAMGPTPSMIGYGMAKAAVHHLTSSLAAKDSGLPDNSAVLTIMPVTLDTP
MNRKWMPNADHSSWTPLSFISEHLLKWTTETSSRPSSGALLKITTENGTSTITPQ
10000000000000000000000000000000000000000000000000000000000000000000000000000000
00000000000000000000000000000000000000000000000000000000000000000000000000000000
0000000000000000000000000000000000000000000000000000000000000000000
>Q02189_2x9o_1_235
FDSFLNELHSDITKRGGSPLPLPEGLEECRSSKSSSVIQSWLWDVPGFRRWRVTRLDAGDSLQ
VFNSVAYPDYNYDHPLMGVDLLWFGARQKLVAVLDFQPLVQDKDYLDRYFSGLKELNQRF
PDLNGEETMRSFDPNQYFSSWLLFCRGGAEQADLSLPKAFSAFLKAYWDLHDNAKSIPSTI
PPEEVKNLQDKYDIYSAERDPAHGLFTSHFGKDWSNRFLHEFLFPASSSHK
00000000000000000000000000000000000000000000000000000000000000000000000000000000
00000000000000000000000000000000000000000011000000000000000000000000000000000000
000000000000000000000000000000000000000000000000000000000000000111
>Q53WA2_1ve2_0_234
MRGKVYLVGAGFGGPEHLTLKALRVLEVAEVVLHDRLVHPGVLALAKGELVPVGKEGYG
GKTPQEAITARLIALAREGRVVARLKGGDPMVFGRGGEEALALRRAGIPFEVVPGVTSAVG
ALSALGLPLTHRGLARSFAVATGHDPALPLPRADTLVLLMPLHTLGGLKERLLERFPPETPL
ALLARVGWPGEAVRLGRVEDLPGLGEGLPSPALLVVGKVVGLYGELLPKDHGL
00000000000000000000000000000000000000000000000000011111100000000000000000000000
00000000000000000000000000000000000000000000000000000000000000000000000000000000
000000000000000000000000000000000000000000000000000000000000000000
>P0A8P1_2cxa_0_233
MRLVQLSRHSIAFPSPEGALREPNGLLALGGDLSPARLLMAYQRGIFPWFSPGDPILWWSPD
PRAVLWPESLHISRSMKRFHKRSPYRVTMNYAFGQVIEGCASDREEGTWITRGVVEAYHRL
HELGHAHSIEVWREDELVGGMYGVAQGTLFCGESMFSRMENASKTALLVFCEEFIGHGGK
LIDCQVLNDHTASLGACEIPRRDYLNYLNQMRLGRLPNNFWVPRCLFSPQE
00000000000000000000000000000000000000000000000000000000000000000000000000000000
00000000000000000000011100000000000000000000000000000000000000000000000000000000
00000000000000000000000000000000000000000000000000000000000000011
>P36068_1xly_5_238
DIKVTPGTCELVEQILALLSRYLSSYIHVLNKFISHLRRVATLRFERTTLIKFVKKLRFYNDCVL
SYNASEFINEGKNELDPEADSFDKVILPIASMFVKCVETFDLLNYYLTQSLQKEILSKTLNED
LTLTAESILAIDDTYNHFVKFSQWMIESLRIGSNLLDLEVVQFAIKCADEDGTNIGETDNIFL
QEILPVNSEEEFQTLSAAWHSILDGKLSALDEEFDVVATKWHD
00000000000000000000000000000000000000000000000000000000000000000000000000000000
00000000000000000000000000000000000000000000000000000000000000000000000000000000
00000000000011111111000000000000000000000000000000000000000000011
>P40011_2c5q_0_233
MSDLQKLQRFSTCDISDGLLNVYNIPTGGYFPNLTAISPPQNSSIVGTAYTVLFAPIDDPRPAV
NYIDSVPPNSILVLALEPHLQSQFHPFIKITQAMYGGLMSTRAQYLKSNGTVVFGRIRDVDE
HRTLNHPVFAYGVGSCAPKAVVKAVGTNVQLKILTSDGVTQTICPGDYIAGDNNGIVRIPV
QETDISKLVTYIEKSIEVDLLVSEDIKNGIPAKQAQNDRRSVLKKYI
10000000000000000000000000000000000000000000000000000000000000000000000000000000
00000000000000000000000000000000000000000000000000000000000000000000000000000000
00000000000000000000000000000000000000000000000000000000000000000

>Q13162_3tjj_37_270
WETEERPRTREEECHFYAGGQVYPGEASRVSVADHSLHLSKAKISKPAPYWEGTAVIDGEFK
ELKLTDYRGKYLVFFFYPLDFTFVCPTEIIAFGDRLEEFRSINTEVVACSVDSQFTHLAWINTP
RRQGGLGPIRIPLLSDLTHQISKDYGVYLEDSGHTLRGLFIIDDKGILRQITLNDLPVGRSVDE
TLRLVQAFQYTDKHGEVCPAGWKPGSETIIPDPAGKLKYFDKLN
11111111111111111111111111111111111111100000000000000000000000000000000000000000000
00000000000000000000000000000000000000000000000000000000000000000000000000000000000
00000000000000000000000000000000000000011111111111111111111111111111
>Q1GJN9_3bfm_0_233
MSETITFPPLMTGEAAGPGQDPFDLACQKAELGVDAGLVVYELGTDVLRAALVLAPEVPL
AKAMAMLPVCGVGFQNALGALAPPEVAVHLDWNGALRINGARCGRLRIAASTDDPDTQ
PDWLVVGLDLPLWPEGDGGETPDETALYAEGCADVAAPRLLESWARHCLHWINRWDEGE
LETIHGEWRGLAHGMGEARTEAGRSGTFLGVDEDFGMLLRDETTTHLIPLTTVLVQD
11100000000000000000000000000000000000000000000000000000000000000000000000000000000
00000000000000000000000000000000000000000000000000000000000000000000000000000000000
00000000000000000000000000000000000000000000000000000000000000000001
>Q3M7N3_2hbw_0_233
MLSNLESSIQSPKSGEYQCLAALNLYDSPECTSLATQAAVGRHLQVTSNQQGAAVEVCLCE
DDYPGWLSLGDLGLLKPATVLYQAKSFSESEIKKLLPGAIAFTQKAMQQSNYYLWGGTVGP
NYDCSGLMQAAFVSVGIWLPRDAYQQEAFTQAITIDELAPGDLVFFGTPVKATHVGLYLGD
GCYIHSSGKAQGRDGIGIDILSEQGDVVSRSYYQQLRGAGRVVKSYKPQRH
11111111111110000000000000000000000000000000000000000000000000000000000000000000000
00000000000000000000000000000000000000000000000000000000000000000000000000000000000
00000000000000000000000000000000000000000000000000000000000000000000
>Q81FP2_2ixd_0_233
MSGLHILAFGAHADDVEIGMAGTIAKYTKQGYEVGICDLTEADLSSNGTIELRKEEAKVAA
RIMGVKTRLNLAMPDRGLYMKEEYIREIVKVIRTYKPKLVFAPYYEDRHPDHANCAKLVEE
AIFSAGIRKYMPELSPHRVESFYNYMINGFHKPNFCIDISEYLSIKVEALEAYESQFSTGSDGV
KTPLTEGYVETVIAREKMFGKEVGVLYAEGFMSKKPVLLHADLLGGCK
10000000000000000000000000000000000000000000000000000000000000000000000000000000000
00000000000000000000000000000000000000000000000000000000000000000000000000000000000
00000000000000000000000000000000000000000000000000000000000000000000
>Q81YJ8_3r7a_0_233
MKNRETDSNVVTLYVTRHGKTILNTNHRAQGWADSPLVEKGVEVATNLGTGLKDIHFMN
AYSSDSGRAIETANLVLKYSEQSKLKLEQRKKLRELNFGIFEGEKLDNMWDAVGKAAGVTS
PEELLKFSIQEVIDLIRAADPTKQAEDWELFSTRIKAEIDKISEEAAKDGGGNVLVVVHGLLI
TTLIEMLDSSKTKLGVENASVTKIVYQDGIYTVESVGDMSYVAKGKESVEI
11100000000000000000000000000000000000000000000000000000000000000000000000000000000
00000000000000000000000000000000000000000000000000000000000000000000000000000000000
00000000000000000000000000000000000000000000000000000000000000000011
>Q97NR6_1sqs_0_233
MNKIFIYAGVRNHNSKTLEYTKRLSSIISSRNNVDISFRTPFNSELEISNSDSEELFKKGIDRQS
NADDGGVIKKELLESDIIIISSPVYLQNVSVDTKNFIERIGGWSHLFRLAGKFVVTLDVAESN
GSDNVSEYLRDIFSYMGGQILHQVSITNSLKDIAEAQLMEATYKIEDVLEGKIKYKTTDYQE
RAYQTLKLILENYDSEHFEKMYWEKKRLFEANSLEEWYYVENIK
00000000000000000000000000000000000000000000000000000000000000000000000000000000000
00000000000000000000000000000000000000000000000000000000000000000000000000000000000
00000000000000000000000000000000000000000000000000000000000000000011
>Q9UBP6_3ckk_31_264
DHTLRYPVKPEEMDWSELYPEFFAPLTQNQSHDDPKDKKEKRAQAQVEFADIGCGYGGLL
VELSPLFPDTLILGLEIRVKVSDYVQDRIRALRAAPAGGFQNIACLRSNAMKHLPNFFYKGQ

LTKMFFLFPDPHFKRTKHKWRIISPTLLAEYAYVLRVGGLVYTITDVLELHDWMCTHFEEHP
LFERVPLEDLSEDPVVGHLGTSTEEGKKVLRNGGKNFPAIFRRIQDPVLQ
11111000000000000000011111111111111111111110000000000000000000000000000000000000000
00000000000000000000000000000000000000000000000001111111111100000000000000000000000
0000000000000000000000000000000000000000000000000000000000000000000
>O66837_2eay_0_232
MFKNLIWLKEVDSTQERLKEWNVSYGTALVADRQTKGRGRLGRKWLSQEGGLYFSFLLNP
KEFENLLQLPLVLGLSVSEALEEITEIPFSLKWPNDVYFQEKKVSGVLCELSKDKLIVGIGINV
NQREIPEEIKDRATTLYEITGKDWDRKEVLLKVLKRISENLKKFKEKSFKEFKGKIESKMLYL
GEEVKLLGEGKITGKLVGLSEKGGALILTEEGIKEILSGEFSLRRS
00000000000000000000000000000000000011111111111000000000000000000000000000000000000
00000000000000000000000000000000000000000000000000000000000000000000000000000000000
000000000000000000000000000000000000000000000000000000000000000000
>Q1GJV4_3siy_0_232
MTSFDRPFEAARPDGENPSAHETLAEGGRLRPEATYTIPARQGRAIRMAQGEALMVINRD
GSQIGDFWAFVEGDCGEYLSMEHLRPTLRRVSPRPGDVLVSNRRRPILTLLEDSSPGVHDTL
VASCDVHRYAQLGHEGYHDNCTDNLRMALGALGLRPTTVPCPLNLWMNTPVVEGGAM
EWRPPVSRRGDHVLFRAELDVVVVISCCPMDLLPINGEEAQPRALDVRLRPRPA
11000000000000000000000000000000000000000000000000000000000000000000000000000000000
00000000000000000000000000000000000000000000000000000000000000000000000000000000000
000000000000000000000000000000000000000000000000000000000000000000
>Q58MU6_2vcl_0_232
MTKNPRNNKPKKILDSSYKSKTIWQNYIDALFETFPQLEISEVWAKWDGGNVTKDGGDAK
LTANIRTGEHFLKAREAHIVDPNSDIYNTILYPKTGADLPCFGMDLMKFSDKKVIIVFDFQH
PREKYLFSVDGLPEDDGKYRFFEMGNHFSKNIFVRYCKPDEVDQYLDTFKLYLTKYKEMID
NNKPVGEDTTVYSDFDTYMTELDPVRGYMKNKFGEGRSEAFVNDFLFSYK
11111111111111111111100000000000000001111100000000000000000000000000000000000000000
00000000000000000000000000000000000000000000000000000000000000000000000000000000000
000000000000000000000000000000000001100000000000000000000000000000
>Q749P2_3lr1_23_255
EERLKMSTTTSTQDSGLLKVLLPPFEKKNNVKVDVIAVGTGQALKLGEAGDVDVVFVHAR
KLEDKFVADGFGVNRKDVMYNDFVIVGPKNDPAGIAKAKTAAEALKLLATKGATFISRGD
KSGTHTKELDLWKSAGVDPKGNWYVEAGQGMGPVITMATERRAYTLTDRGTYNAFKGA
KTDLVILFQGEKGLFNPYGIMAVNPKKFPHVKYDLAMKLIDYVTGPEGLKIISDY
00000000000000000000000000000000000000000000000000000000000000000000000000000000000
00000000000000000000000000000000000000000000000000000000000000000000000000000000000
0000000000000000000000011100000000000000000000000000000000000000000
>Q880F6_3k4i_1_233
SVPFEYTPIAQSVLDECEHLDTASLSDALDSLGIDGGLPGIASQVPGTRCVGIAFTVQYQPVD
ASEGFRGAANYIDQVPSGSVIVSSNSGRHDCTVWGDIMTHFALANGIKGTVIDGVARDIDT
VINCNYPLFSRGRFMQSAKNRTQLKAVQVPLVIDGITIQPGDLMVCDGSGCVVVPQQLAA
EVVLRARAVEQTERRIIEAISSGSTLEQARMTYRYDQPWLSEAEHGGTQ
11100000000000000000000000000000000000000000000001111111111000000000000000000000000
00000000000000000000000000000000000000000000000000000000000000000000000000000000000
0000000000000000000000000000000000000000000000000111111111111111111
>Q8KBS5_2hcf_0_232
MSRTLVLFDIDGTLLKVESMNRRVLADALIEVYGTEGSTGSHDFSGKMDGAIIYEVLSNVGL
ERAEIADKFDKAKETYIALFRERARREDITLLEGVREELLDALSSRSDVLLGLLTGNFEASGRH
KLKLPGIDHYFPFGAFADDALDRNELPHIALERARRMTGANYSPSQIVIIGDTEHDIRCARE
LDARSIAVATGNFTMEELARHKPGTLFKNFAETDEVLASILTPKHS
10000000000000000000000000000000000000011100000000000000000000000000000000000000000

00000000000000000000000000000000000000000000000000000000000000000000000000000000
0000000000000000000000000000000000000000000000000000000000000000001111
>Q9HZJ9_2fbq_0_232
MAQSETVERILDAAEQLFAEKGFAETSLRLITSKAGVNLAAVNYHFGSKKALIQAVFSRFLG
PFCASLEKELDRRQAKPEAQHATLEDLLHLLVSQAMAVKPRSGNDLSIFMRLLGLAFSQSQ
GHLRKYLEEVYGKVFRRYMLLVNEAAPKLPPIELFWRVHFMLGAAAFSMSGIKALRAMAE
TDFGVNTSTEQVMHLMVPFFAAGMRAESGIDDPLLAGAQLRPRNKTPAKA
10000000000000000000000000000000000000000000000000000000000000000000000000000000
00000000000000000000000000000000000000000000000000000000000000000000000000000000
00000000000000000000000000000000000000000000000011111111111111111
>F8QQG5_3u3n_23_254
VNYCRLPCRGDNYHVGCGEPAYAQECGQSPRTRELLKEHRNEILSKINDVRDHVAKGSWG
LPVAARMKVVVWDAELAGLAKRHTKGCVGETHACRNTERFWLPGQLNFKYSGDKLPRIK
ELIDDAVKKGHLQKHNITREIIGNYRENGPNGDVKELALAISDRVTAVGCGLTTWQDGAK
ARALLTCNFSSQNTRGRPVYKIGNSPGEKCIEKDETYKNLCPATEPIDPNKSN
00000000000000000000000000000000000000000000000000000000000000000000000000000000
00000000000000000000000000000000000000000000000000000000000011100000000000000000
0000000000000000000000000000000000000000000000000000000000000000000
>O00584_3t0o_24_255
DKRLRDNHEWKKLIMVQHWPETVCEKIQNDCRDPPDYWTIHGLWPDKSEGCNRSWPFN
LEEIKDLLPEMRAYWPDVIHSFPNRSRFWKHEWEKHGTCAAQVDALNSQKKYFGRSLELY
RELDLNSVLLKLGIKPSINYYQVADFKDALARVYGVIPKIQCLPPSQDEEVQTIGQIELCLTK
QDQQLQNCTEPGEQPSPKQEVWLANGAAESRGLRVCEDGPVFYPPPKKTKH
11111111100000000000000000000000000000000000000000000000000000000000000000000000
00000000000000000000000000000000000000000000000000000000000000000000000000000011
100000000000000000000000011111111111111111111000000000000000000001111
>P13667_3idv_52_283
EDDLEVKEENGVLVLNDANFDNFVADKDTVLLEFYAPWCGHCKQFAPEYEKIANILKDKD
PPIPVAKIDATSASVLASRFDVSGYPTIKILKKGQAVDYEGSRTQEEIVAKVREVSQPDWTPPP
EVTLVLTKENFDEVVNDADIILVEFYAPWCGHCKKLAPEYEKAAKELSKRSPPIPLAKVDAT
AETDLAKRFDVSGYPTLKIFRKGRPYDYNGPREKYGIVDYMIEQSG
10000000000000000000000000000000000000000000000000000000000000000000000000000000
00000000000000000000000000000000000000000000000000000000000000000000000000000000
0000000000000000000000000000000000000000000000000000000000000000000
>Q45563_2cb9_1042_1273
QLSAAGEQHVIQLNQQGGKNLFCFPPISGFGIYFKDLALQLNHKAAVYGFHFIEEDSRIEQY
VSRITEIQPEGPYVLLGYSAGGNLAFEVVQAMEQKGLEVSDFIIVDAYKKDQSITADTENDD
SAAYLPEAVRETVMQKKRCYQEYWAQLINEGRIKSNIHFIEAGIQTETSGAMVLQKWQDA
AEEGYAEYTGYGAHKDMLEGEFAEKNANIILNILDKINSDQKVLPNKH
11000000000000000000000000000000000000000000000000000000000000000000000000000000
00000000000000000000000000000000000011111110000000000000000000000000000000000000
000000000000000000000000000000000000000000000000000011111111111
>Q5SH33_2cul_0_231
MAAYQVLIVGAGFSGAETAFWLAQKGVRVGLLTQSLDAVMMPFLPPKPPFPPGSLLERAYD
PKDERVWAFHARAKYLLEGLRPLHLFQATATGLLLEGNRVVGVRTWEGPPARGEKVVLAV
GSFLGARLFLGGVVEEAGRLSEASYPDLLEDLSRLGFRFVEREGEVPETPSTPGYRVRYLAFH
PEEWEEKTFRLKRLEGLYAVGLCVREGDYARMSEEGKRLAEHLLHELG
10000000000000000000000000000000000000000000000000000000000000000000000000000000
00000000000000000000000000000000000000000000000000000000000000000000000000000000
0011111100000000000000000000000000000000000000000000000000000000001
>Q7MAW7_2gau_0_231

MWKTASDKGLGHLLRDVWSLLNEEERELLDKEIQPFPCKKASTVFSEGDIPNNLFYLYEGKI
KILREGVYGRFHISRIVKPGQFFGMRPYFAEETCSSTAIAVENSKVLAIPVEAIEALLKGNTSF
CRYFLKALAKELGYAERRTVTLTQKHVRGRLAETLLILKENFGFENDGATLSIYLSREELATL
SNMTVSNAIRTLSTFVSERMLALDGKRIKIIDCDRLQKTARSG
11111111100000000000000000000000000000000000000000000000000111110000000000000
00000000000000000000000000000000000000000000000000000000000000000000000000000000000
000000000000000000000000000000000000000000000000000000000000000000
>Q9IFX1_3qsq_414_645
GATPMTFGRSIPEPGEQFRVLLTVGPPMAPNTANSQNWVNKTIVPPENQYTVKIGIDLEHY
TTMQGFTPVESVSWYTADFQPSDEPSPIPGLYARVNNTKKADVYGVQQFKSSHTNNRHQIT
SVFLVRVTTSFQVINYTSYFIRGAESGSNVSNLKIRDQTYHTPLQFTQGKWYLLTSTVMHDG
PTSSGWVWMNQELTNNIAYRVDPGMMYLITPPPAASQLYFELHTVLPQ
11111111111111100000000000000000000000000000000000000000000000000000000000000000000
00000000000000000000000000000000000000000000000000000000000000000000000000000000000
000000000000000000000000000000000000000000000000000000000000000001
>Q9RMI1_3sd9_2_233
SEKNLTLTHFKGPLYIVEDKEYVQENSMVYIGTDGITIIGATWTPETAETLYKEIRKVSPLPINE
VINTNYHTDRAGGNAYWKTLGAKIVATQMTYDLQKSQWGSIVNFTRQGNNKYPNLEKSL
PDTVFPGDFNLQNGSIRAMYLGEAHTKDGIFVYFPAERVLYGNCILKENLGNMSFANRTEY
PKTLEKLKGLIEQGELKVDSIIAGHDTPIHDVGLIDHYLTLLEKAPK
11000000000000000000000000000000000000000000000000000000000000000000000000000000000
00000000000000000000000000000000000000000000000000000000000000000000000000000000000
000000000000000000000000000000000000000000000000000000000000000000
>B2HSG1_3r6h_0_230
MSGPVTYTHDDAIGVIRMDDGKVNVLGPTMQQALNEAIDAADRDNVGALVIAGNHRVF
SGGFDLKVLTSGEAKPAIDMLRGGFELSYRLLSYPKPVVIACTGHAIAMGAFLLCSGDHRVA
AHAYNVQANEVAIGMTIPYAAMEVLKLRLTPSAYQQAAGLAKTFFGETALAAGFIDEISLPE
VVLSRAEEAAREFAGLNQQAHNATKLRARAEALKAIRAGIDGIEAEFGL
11000000000000000000000000000000000000000000000000000000000000000000111000000000000
00000000000000000000000000000000000000000000000000000000000000000000000000000000000
00000000000000000000000000000000000000000000000000000000000000001
>E8X8J1_3zeu_0_230
MRILAIDTATEACSVALWNNGTINAHFELCPREHTQRILPMVQEILAASGASLNEIDALAFG
RGPGSFTGVRIGIGIAQGLALGANLPMIGVSTLATMAQGAWRKTGATRVLAAIDARMGEV
YWAEYQRDAQGVWQGEETEAVLKPERVGERLKQLSGEWATVGTGWSAWPDLAKECGLT
LHDGEVSLPAAEDMLPIASQKLAAGETVAVEHAEPVYLRNEVAWKKLPGKE
00000000000000000000000000000000000000000000000000000000000000000000000000000000000
00000000000000000000000000000000000000000000000000000000000000000000000000000000000
00000000000000000000000000000000000000000000000000000000000000001
>P16083_3g5m_0_230
MAGKKVLIVYAHQEPKSFNGSLKNVAVDELSRQGCTVTVSDLYAMNLEPRATDKDITGTLS
NPEVFNYGVETHEAYKQRSLASDITDEQKKVREADLVIFQFPLYWFSVPAILKGWMDRVLC
QGFAFDIPGFYDSGLLQGKLALLSVTTGGTAEMYTKTGVNGDSRYFLWPLQHGTLHFCGFK
VLAPQISFAPEIASEEERKGMVAAWSQRLQTIWKEEPIPCTAHWHFGQ
10000000000000000000000000000000000000000000000000000000000000000000000000000000000
00000000000000000000000000000000000000000000000000000000000000000000000000000000000
00000000000000000000000000000000000000000000000000000000000000000
>Q6G3B2_3ne8_178_408
SFRVVLDPGHGGIDGGARGVTGILEKDVTLAFARALRDELQKGSHTIVALTRDSDIFLRLSER
VKKAQEFDADLFISIHADTIDVHSLRGATVYTISDEASDAIAKSLAESENKVDLLDGLPKEES
LELTDILLDLTRRETHAFSINFANNVVSNLSKSHINLINNPHRYADFQVLKAPDVPSVLIEIGY

LSNKEDEKLLNNPQWRKQMAASIAYSIRQFAEYRQKIMQPL
00000000000000000000000000000000000000000000000000000000000000000000000000000000000
00000000000000000000000000000000000000000000111111000000000000000000000000000000000
00000000000000000000000000000000000000000000000000000000000000000
>A7X435_3w01_0_229
MYDIKKWRHIFKLDPAKHISDDDLDAICMSQTDAIMIGGTDDVTEDNVIHLMSKIRRYPLP
LVLEISNIESVMPGFDFYFVPTVLNSTDVAFHNGTLLEALKTYGHSIDFEEVIFEGYVVCNAD
SKVAKHTKANTDLTTEDLEAYAQMVNHMYRLPVMYIEYSGIYGDVSKVQAVSEHLTETQL
FYGGGISSEQQATEMAAIADTIIVGDIIYKDIKKALKTVKIKESSK
00000000000000000000000000000000000000000000000000000000000000000000000000000000000
00000000000000000000000000000000000000000000000000000000000000000000000000000000000
0000000000000000000000000000000000000000000000000000000000000011
>A9CFJ9_2ycd_0_229
MSNIETVPASIEMKPNPTITVFERSPDGGRGLARDMPVRWALEEVGQPYHVRRLSFEAMKE
ASHLAYQPFGQIPSYEQGDLILFESGAIVMHIAQHHSGLLPEDQLRRARTVAWMFAALNTIE
PSILNFTTVWLFERNEPWHEARLARTKEQLLKRLDELSAWLGDREWLEGSFSAADILMICV
LRRLESSGILKDYGNLLAYVERGKARPAFKRAFDAQLAVFTAASKN
11111111111110000000000000000000000000000000000000000000000000000000000000000000000
00000000000000000000000000000000000000000000000000000000000000000000000000000000000
0000000000000000000000000000000000000000000000000000000000001111
>O15118_3gki_22_251
QSCVWYGECGIAYGDKRYNCEYSGPPKPLPKDGYDLVQELCPGFFFGNVSLCCDVRQLQTL
KDNLQLPLQFLSRCPSCFYNLLNLFCELTCSPRQSQFLNVTATEDYVDPVTNQTKTNVKEL
QYYVGQSFANAMYNACRDVEAPSSNDKALGLLCGKDADACNATNWIEYMFNKDNGQA
PFTITPVFSDFPVHGMEPMNNATKGCDESVDEVTAPCSCQDCSIVCGPKPQ
00000000000000000000000000000000000000000000000000000000000000000000000000000000000
00000000000000000000000000000000000000000000000000000000000000000000000000000000000
0000000000000000000000000000000000000000000000000000000000011111
>P15374_1uch_0_229
MEGQRWLPLEANPEVTNQFLKQLGLHPNWQFVDVYGMDPELLSMVPRPVCAVLLLFPITE
KYEVFRTEEEEKIKSQGQDVTSSVYFMKQTISNACGTIGLIHAIANNKDKMHFESGSTLKKF
LEESVSMSPEERARYLENYDAIRVTHETSAHEGQTEAPSIDEKVDLHFIALVHVDGHLYELD
GRKPFPINHGETSDETLLEDAIEVCKKFMERDPDELRFNAIALSAA
11110000000000000000000000000000000000000000000000000000000000000000000000000000000
00000000000000000000000000000000000000000000000000000000000011111111111111111111111
0000000000000000000000000000000000000000000000000000000000000000
>P45124_1vio_1_230
RLDKFIAENVGLTRSQATKAIRQSAVKINGEIVKSGSVQISQEDEIYFEDELLTWIEEGQYFML
NKPQGCVCSNDDGDYPTIYQFFDYPLAGKLHSAGRLDVDTTGLVLLTDDGQWSHRITSPK
HHCEKTYLVTLADPVEENYSAACAEGILLRGEKEPTKPAKLEILDDYNVNLTISEGRYHQVK
RMFAALGNKVVGLHRWKIGDVVLDESLEEGEYRPLTQSEIEKLV
00000000000000000000000000000000000000000000000000000000000000000000000000011000000
00000000000000000000000000000000000000000000000000000000000000000000000000000000000
0000000000000000000000000000000000000000000000000000000000000000
>P51580_2bzg_15_244
TEVQKNQVLTLEEWQDKWVNGKTAFHQEQGHQLLKKHLDTFLKGKSGLRVFFPLCGKAV
EMKWFADRGHSVVGVEISELGIQEFFTEQNLSYSEEPITEIPGTKVFKSSSGNISLYCCSIFDLP
RTNIGKFDMIWDRGALVAINPGDRKCYADTMFSLLGKKFQYLLCVLSYDPTKHPGPPFYVP
HAEIERLFGKICNIRCLEKVDAFEERHKSWGIDCLFEKLYLLTEK
10000000000000000000000000000000000000000000000000000000000000000000000000000000000
00000000000000000000000000000000000000000000000000000000000000000000000000000000000

000000000000000000000000000000000000000000000000000000000000000
>Q4QIE0_4f2n_0_229
MFGRAPMKAATATAAVGFSCLCYHTLPHLRYPAELPTLGFNYKDGIQPVMSPRQLELHYSK
HHSAYVDKLNTLGKGYEGKTIEEIILATTGINESKVMFNQAAQHFNHSFFWKCLSPGGKPM
PKTLENAIAKQFGSVDDFMVSFQQAGVNNFGSGWTWLCVDPQTKELLIDSTSNAGCPLTS
GLRPIFTADVWEHAYYKDFENRRADYLKELWQIVDWEFVCHMYERATK
11111111111111111111100000000000000000000000000000000000000000000000000000000000000
00000000000000000000000000000000000000000000000000000000000000000000000000000000000
000000000000000000000000000000000000000000000000000000000000000
>Q6SJB6_3chv_0_229
MDTPANKPCIICVAITGSVPTKADNPAVPITVSEQVESTQEAFEAGAAIAHCHVRNDDGTPS
SDPDRFARLTEGLHTHCPGMIVQFSTGGRSGAGQARGGMLPLKPDMASLSVGSNNFPSRV
YENPPDLVDWLAAQMRSYRVTPEIEAFDLSHILRAIDMHGRGLLYGKLYVQFVMGVKNAM
PADREVFDFYVRMMRTRAPQAEWCAAGIGANQLTVNVMGHRAGGHTRT
11110000000000000000000000000000000000000000000000000000000000000000000000000000000
00000000000000000000000000000000000000000000000000000000000000000000000000000000000
000000000000000000000000000000000000000000000000000000000000000
>Q8NYF3_1wv3_0_229
MHKLIIKYNKQLKMLNLRDGKTYTISEDERADITLKSLGEVIHLEQNNQGTWQANHTSIN
KVLVRKGDLDDITLQLYTEADYASFAYPSIQDTMTIGPNAYDDMVIQSLMNAIIIKDFQSIQE
SQYVRIVHDKNTDVYINYELQEQLTNKAYIGDHIYVEGIWLEVQADGLNVLSQNTVASSLIR
LTQEMPHAQADDYNTYHRSPRIIHREPTDDIKIERPPQPIQKNNT
00000000000000000000000000000000000000000000000000000000000000000000000000000000000
00000000000000000000000000000000000000000000000000000000000000000000000000000000000
000000000000000000001111111111111111111111111111111111111111111
>Q99QX5_3bfv_0_229
MTNTRRSTSSLIVHEQPKSPISEKFRGIRSNIMFANPDSAVQSIVITSEAPGAGKSTIAANLAV
AYAQAGYKTLIVDGDMRKPTQHYIFNLPNNEGLSSLLLNWSTYQDSIISTEIQDLDVLTSGPI
PPNPSELITSRAFANLYDTLLMNYNFVIIDTPPVNTVTDAQLFSKFTGNVVYVVNSENNNK
DEVKKGKELIEATGAKLLGVVLNRMPKDKSASYYAYYGTDES
00000000000000000000000000000000000000000000000000000000000000000000000000000000000
00000000000000000000000000000000000000000000000000000000000000000000000000000000000
000000000000000000000000000000000000000000000000111111111111111
>Q9KZ14_2qib_0_229
MTTGVRRRMGVEERRQQLIGVALDLFSRRSPDEVSIDEIASAAGISRPLVYHYFPGKLSLYEA
ALQRASDDLADRFVEPRQGPLGARLLRVMGRYFDFVDEHGPGFSALMRGGPAVGSTTTNA
LVDSVRQAAYVQILSHLDVTEPPARLELVVRSWISLAESTALLWLDGRRIPRAELETQLVHDF
AALMAVSAAYDEEMGALVRRVLADEPEDGPFGDLVDRLLALSAR
11111111000000000000000000000000000000000000000000000000000000000000000000000000000
00000000000000000000000000000000000000000000000000000000000000000000000000000000000
000000000000000000000000000000000000000000000000000000000000000
>C8WVE1_3qsj_0_228
MTDIRKAATLVVIRDGANKDIEVLVVRRAKTMRFLPGFVAFPGGAADPSDAEMAKRAFGR
PVCAEDDDDPALAVTALRETAEEIGWLLAVRDGEGTKMDTPLAPDEQADLCKGGDALSA
WLSARGLAFDLGLLRRIGRFVTPPTQPVRFDTRFFLCVGQHLGEPRLHGAELDAALWTPAR
DMLTRIQSGELPAVRPTIAVLKALVACPNAEIAMSTLSIGPLPPPAPRA
00000000000000000000000000000000000000000000000000000000000000000000000000000000000
00000000000000000000000000000000000000000000000000000000000000000000000000000000000
00000000000000000000000000000000000000000000000000000011111111
>P06701_2fl7_0_228
MAKTLKDLDGWQVIITDDQGRVIDDNNRRRSRKRGGENVFLKRISDGLSFGKGESVIFNDN

VTETYSVYLIHEIRLNTLNNVVEIWVFSYLRWFELKPKLYYEQFRPDLIKEDHPLEFYKDKFF
NEVNKSELYLTAELSEIWLKDFIAVGQILPESQWNDSSIDKIEDRDFLVRYACEPTAEKFVPIDI
FQIIRRVKEMEPKQSDEYLKRVSVPVSGQKTNRQVMHKMG
11111110000000000000000111111111111100000000000000000000000000000000000000001111100
00000000000000000000000000000000000000000000000000000000000000000000000000011111110
00000000000000000000000000000000000000000000000011111111111111
>P06722_1azo_0_228
MSQPRPLLSPPETEEQLLAQAQQLSGYTLGELAALVGLVTPENLKRDKGWIGVLLEIWLGA
SAGSKPEQDFAALGVELKTIPVDSLGRPLETTFVCVAPLTGNSGVTWETSHVRHKLKRVLWI
PVEGERSIPLAQRRVGSPLLWSPNEEEDRQLREDWEELMDMIVLGQVERITARHGEYLQIRP
KAANAKALTEAIGARGERILTLPRGFYLKKNFTSALLARHFLIQ
11100000000000000000000000000000000000000000000000000000000001111100000000000000000
00000000000000000000000000000000000000000000000000000000000000000000000000000000000
00000000000000000001111111000000000000000000000000000000000000
>P36936_1fye_0_228
MELLLLSNSTLPGKAWLEHALPLIANQLNGRRSAVFIPFAGVTQTWDEYTDKTAEVLAPLG
VNVTGIHRVADPLAAIEKAEIIIVGGGNTFQLLKESRERGLLAPMADRVKRGALYIGWSAG
ANLACPTIRTTNDMPIVDPNGFDALDLFPLQINPHFTNALPEGHKGETREQRIRELLVVAPE
LTVIGLPEGNWIQVSNGQAVLGGPNTTWVFKAGEEAVALEAGHRF
00000000000000000000000000000000000000000000000000000000000000000000000000000000000
00000000000000000000000000000000000000000000000000000000000000000000000000000111111
11100000000000000000000000000000000000000000000000000000000000
>Q0P8G4_2cic_0_228
MTNIEILENMLKLQQKLNDETNGLNWENGYTKEGKLISWRRCIYMECAELIDSFTWKHW
KNISSLTNWENVRIEIVDIWHFILSLLLEEYRDKNNKDFKAIATEVNAVSVFQDFCKEEEYPN
EGDIYGILNDIELIIHKCSGFGFNLGELLSTYFTLAIKCGLNLEILYKTYIGKNVLNIFRQNNGY
KDGSYKKTWNGKEDNEVLAQILEQELDFDTIYKKLEECYKKA
00000000000000000000000000000000000000000000000000000000000000000000000000000000000
00000001111110000000000000000000000000000000000000000000000000000000000000000000000
00000000000000000000000000000000000000000000000000000000000000
>Q5LEB7_3evy_655_883
SSEVVLMKPYEKLVERFNEMAAEFLSYFPTVKSVGNLESELDKRRFVILFRAMLRLRNEVKG
YNEFDAEDLTIEEQRFADYQSKYLDMSNEFAITSEKEDAESILQDIDFELELVHRDIINVMYIL
ALLQDLKPESSSYPKDRKAVLDTMDSNPELRSKIALIDNFIKLHIDGRQSNDLPADMESDLD
KYIATQKAIAIEQVATEEGIDSTLLHEYISEYEYLGKPKNE
11111000000000000000000000000000000000000000000000000000000000000000000000000000000
00000011111111111111111111111111111111111111111111111111111111111111111111111111111
11111111111111111111111111111111111111111111111111111111111111
>Q73BT6_3cc8_0_228
MNSPKNSLYEEKSGHYYNAVNPNLLKHIKKEWKEVLDIGCSSGALGAAIKENGTRVSGIEA
FPEAAEQAKEKLDHVVLGDIETMDMPYEEEQFDCVIFGDVLEHLFDPWAVIEKVKPYIKQN
GVILASIPNVSHISVLAPLLAGNWTYTEYGLLDKTHIRFFTFNEMLRMFLKAGYSISKVDRVY
VDHKMYEPLIEELYGICKKYRLGSGFMAETVVFQYIIEAEKSQL
11111111111111111100000000000000000000000000000000000000000000000000000000000000000
00000000000000000000000000000000000000000000000000000000000000000000000000000000000
00000000000000000000000000000000000000000000000000000000000000
>Q82MH6_3goc_0_228
MTTVRIPAGWPATEEEARAVQDELRGRVILDEPGPPPGTGRVTGVDVAYDDERDVVVAAA
VVLDAATLDVVAEATAVGEVSFPYVPGLLAFREIPTVLAALDALPCPPGLIVCDGYGVAHPR
RFGLASHLGVLTGLPTIGVAKNPFTFSYEDPGAPRGSAAPLLAGADEVGRALRTQSGVKPV
FVSVGHRVDLDHACAHTLALTPKYRIPETTRRADSLCRRALKEATA

10000000000000000000000000000000000000000000000000000000000000000000000000000000000
00000000000000000000000000000000000000000000000000000000000000000000000000000000000
000000000000000000000000000000000000000000000000000000000000000
>Q8CNK1_3no6_0_228
MTFSKELREASRPIIDDIYNDGFIQDLLAGKLSNQAVRQYLRADASYLKEFTNIYAMLIPKMS
SMEDVKFLVEQIEFMLEGEVEAHEVLADFINEPYEEIVKEKVWPPSGDHYIKHMYFNAFAR
ENAAFTIAAMAPCPYVYAVIGKRAMEDPKLNKESVTSKWFQFYSTEMDELVDVFDQLMDR
LTKHCSETEKKEIKENFLQSTIHERHFFNMAYINEKWEYGGNNNE
00000000000000000000000000000000000000000000000000000000000000000000000000000000000
00000000000000000000000000000000000000000000000000000000000000000000000000000000000
000000000000000000000000000000000000000000000000000000000000011
>P08325_2w2r_1_228
SSFKKILGLSSKSHKKSKKMGLPPPYDESCPMETQPSAPLSNDFFGMEDMDLYDKDSLRYEK
FRFMLKMTVRSNKPFRSYDDVTAAVSQWDNSYIGMVGKRPFYKIIAVIGSSHLQATPAVLA
DLNQPEYYATLTGRCFLPHRLGLIPPMFNVQETFRKPFNIGLYKGTLDFTFTVSDDESNEKVP
HVWDYMNPKYQSQIQQEGLKFGLILSKKATGTWVLDQLSPFK
11111111111111111111111111111111111111100000000000001111100000000000000000000000000
00000000000000000000000000000000000000000000000000000000000000000000000000000000000
00000000000000000000000000000000000000000000000000000000000000
>P08572_1li1_1484_1711
SVSIGYLLVKHSQTDQEPMCPVGMNKLWSGYSLLYFEGQEKAHNQDLGLAGSCLARFSTM
PFLYCNPGDVCYYASRNDKSYWLSTTAPLPMMPVAEDEIKPYISRCSVCEAPAIAIAVHSQD
VSIPHCPAGWRSLWIGYSFLMHTAAGDEGGGQSLVSPGSCLEDFRATPFIECNGGRGTCHY
YANKYSFWLTTIPEQSFQGSPSADTLKAGLIRTHISRCQVCMKNL
11100000000000000000000000000000000000000000000000000000000000000000000000000000000
00000000000000000000000000000000000000000000000000000000000000000000000000000000000
00000000000000000000000000000000000000000000000000000000000000
>P73797_1ii5_43_384
LKVGVVGNPPFVFYGEGKNAAFTGISLDVWRAVAESQKWNSEYVRQNSISAGITAVAEGEL
DILIGPISVTPERAAIEGITFTQPYFSSGIGLLIPGSATPLFRSVGDLKNKEVAVVRDTTAVDWA
NFYQADVRETNNLTAAITLLQKKQVEAVMFDRPALIYYTRQNPNLNLEVTEIRVSLEPYGFV
LKENSPLQKTINVEMLNLLYSRVIAEFTERWLGPGIEENQ
00000000000000011111000000000000000000000000000000000000000000000000000000000000000
00000000000000000000000000000000000000000000000000000000000000000000000000000000000
00000000000000000000000000000000000000000000000000000001111111
>Q10404_1w66_2_229
GSIRSKLSAIDVRQLGTVDYRTAWQLQRELADARVAGGADTLLLLEHPAVYTAGRRTETHE
RPIDGTPVVDTDRGGKITWHGPGQLVGYPIIGLAEPLDVVNYVRRLEESLIQVCADLGLHA
GRVDGRSGVWLPGRPARKVAAIGVRVSRATTLHGFALNCDCDLAAFTAIVPCGISDAAVTS
LSAELGRTVTVDEVRATVAAAVCAALDGVLPVGDRVPSHAVPSPL
00000000000000000000000000000000000000000000000000000000000000000000000000000000000
00000000000000000000000000000000000000000000000000000000000000000000000000000000000
00000000000000000000000000000000000000000000000011111111111111
>Q4K977_3noq_0_227
MAVQIGFLLFPEVQQLDLTGPHDVLASLPDVQVHLIWKEPGPVVASSGLVLQATTSFADCP
PLDVICIPGGTGVGALMEDPQALAFIRQQAARARYVTSVCTGSLVLGAAGLLQGKRATTH
WAYHELLAPLGAIPVHERVVRDGNLLTGGGITAGIDFALTLAAELFDAATAQRVQLQLEYA
PAPPFNAGSPDTAPASVVQQARQRAADSLHKRREITLRAAARLAAG
00000000000000000000000000000000000000000000000000000000000000000000000000000000000
00000000000000000000000000000000000000000000000000000000000000000000000000000000000
00000000000000000000000000000000000000000000000000000000000001

>Q60357_1g61_0_227
MTMIIRKYFSGIPTIGVLALTTEEITLLPIFLDKDDVNEVSEVLETKCLQTNIGGSSLVGSLSVA
NKYGLLLPKIVEDEELDRIKNFLKENNLDLNVEIIKSKNTALGNLILTNDKGALISPELKDFK
KDIEDSLNVEVEIGTIAELPTVGSNAVVTNKGCLTHPLVEDDELEFLKSLFKVEYIGKGTANK
GTTSVGACIIANSKGAVVGGDTTGPELLIIEDALGLI
11000000000000000000000000000000000000000000000000000000000000000000000000000000000
00000000000000000000000000000000000000000000000000000000000000000000000000000000000
0000000000000000000000000000000000000000000000000000000000001
>Q7MXD3_3ho7_80_307
IIEEEQQSLTGRLNIAVLPTIAPYLLPRVFPIWKKELAGLEIHVSEMQTSRCLASLLSGEIDMAI
IASKAETEGLEDDLLYYEEFLGYVSRCEPLFEQDVIRTTEVNPHRLWLLDEGHCFRDQLVRF
CQMKGLHERQTAYSGGSMEAFMRLVESGQGITFIPQLTVEQLSPSQKELVRPFGMPRPVREV
RLAVRQDYSRRKLREQLIGLLRSAVPSDMHKLQTGQHLA
11111111100000000000000000000000000000000000000000000000000000000000000000000000000
00000000000000000000000000000000000000000000000000000000000000000000000000000000000
00000000000000000000000000000000000000000000000000000000000000
>Q97QE9_1jyk_1_228
KAIILAAGLGTRLRPMTENTPKALVQVNQKPLIEYQIEFLKEKGINDIIIIVGYLKEQFDYLKE
KYGVRLVFNDKYADYNNFYSLYLVKEELANSYVIDADNYLFKNMFRNDLTRSTYFSVYRED
CTNEWFLVYGDDYKVQDIIVDSKAGRILSGVSFWDAPTAEKIVSFIDKAYVSGEFVDLYWDN
MVKDNIKELDVYVEELEGNSIYEIDSVQDYRKLEEILKNEN
00000000000000000000000000000000000000000000000000000000000000000000000000000000000
00000000000000000000000000000000000000000000000000000000000000000000000000000000000
00000000000000000000000000000000000000000000000000000000000111
>Q9HIW7_1u02_1_228
IFLDYDGTLVPIIMNPEESYADAGLLSLISDLKERFDTYIVTGRSPEEISRFLPLDINMICYHGA
CSKINGQIVYNNGSDRFLGVFDRIYEDTRSWVSDFPGLRIYRKNLAVLYHLGLMGADMKPK
LRSRIEEIARIFGVETYYGKMIIELRVPGVNKGSAIRSVRGERPAIIAGDDATDEAAFEANDD
ALTIKVGEGETHAKFHVADYIEMRKILKFIEMLGVQKKQ
00000000000000000000000000000000000000000000000000000000000000000000000000000000000
00000000000000000000000000000000000000000000000000000000000000000000000000000000000
0000000000000000000000000000000000000000000000000000000000001
>B7UN63_3p42_21_247
AQGMVTIYLPGEQQTLSVGPVENVAQLVTQPQLRDRLWWPGALLTDSAAKAKALKDYQH
VMAQLASWEAEADDDVAATIKSVRQQLLNLNITGRLPVKLDPDFVRVDENSNPPLVGDYT
LYTVQRPVTITLLGAVSGAGQLPWLAGRSVTDYLQDHPRLAGADKNNVMVITPEGETVVA
PVALWNKRHVEPPPGSQLWLGFSAHVLPEKYADLNDQIVSVLTQRVPE
10000000000000000000000000000000000000000000000000000000000000000000000000000000000
00000000000000000000000000000000000000000000000000000000000000000000000000000000000
0000000000000000000000000000000000000000000000000000000000000
>O43809_3bap_0_226
MSVVPPNRSQTGWPRGVTQFGNKYIQQTKPLTLERTINLYPLTNYTFGTKEPLYEKDSSVAA
RFQRMREEFDKIGMRRTVEGVLIVHEHRLPHVLLLQLGTTFFKLPGGELNPGEDEVEGLKR
LMTEILGRQDGVLQDWVIDDCIGNWWRPNFEPPQYPYIPAHITKPKEHKKLFLVQLQEKAL
FAVPKNYKLVAAPLFELYDNAPGYGPIISSLPQLLSRFNFIYN
11111111111111111111000000000000000000000000000000000000000000000000000000000000000
00000000000000000000000000000000000000000000000011110000000000000000000000000000000
0000000000000000000000000000000000000000000000000000000000000
>Q15796_1khx_240_466
MDTGSPAELSPTTLSPVNHSLDLQPVTYSEPAFWCSIAYYELNQRVGETFHASQPSLTVDGFT
DPSNSERFCLGLLSNVNRNATVEMTRRHIGRGVRLYYIGGEVFAECLSDSAIFVQSPNCNQR

YGWHPATVCKIPPGCNLKIFNNQEFAALLAQSVNQGFEAVYQLTRMCTIRMSFVKGWGAE
YRRQTVTSTPCWIELHLNGPLQWLDKVLTQMGSPSVRCSSMS
11111111111111111111111000000000000000000000000000000000000000000000000000000000000
00000000000000000000000000000000000000000000000000000000000000000000000000000000000
0000000000000000000000000000000000000000000000000000000000000
>Q2G7J6_3ec4_0_226
MSEDHPLDRPVWNSLGGPQSELDVASGNLRRLDPAYGPFAAAAPGAEAGLASLLQGDAD
EIWLVEPEPVAPPPGTRVIRVAPLLQMIADGPVPSFDDPGIVALGETDVPEMTALALATEPGP
WASGTWRYGQFYGVRIDGRLAAMAGERMRPAPNLAEVSGVCTWPEYRGRGLAARLIRKV
IAGMAARGEVPYLHSYASNASAIRLYESLGFRARRAMTATLLGKST
11100000000000000000000000000000000000000000000000000000000000000000000000000000000
00000000000000000000000000000000000000000000000000000000000000000000000000000000000
0000000000000000000000000000000000000000000000000000000000000
>Q5LCY3_4i17_18_244
AQTTDPNQLKNEGNDALNAKNYAVAFEKYSEYLKLTNNQDSVTAYNCGVCADNIKKYKE
AADYFDIAIKKNYNLANAYIGKSAAYRDMKNNQEYIATLTEGIKAVPGNATIEKLYAIYYLK
EGQKFQQAGNIEKAEENYKHATDVTSKKWKTDALYSLGVLFYNNGADVLRKATPLASSN
KEKYASEKAKADAAFKKAVDYLGEAVTLSPNRTEIKQMQDQVKAMIK
11000000000000000000000000000000000000000000000000000000000000000000000000000000000
00000000000000000000000000000000000000000000000000000000000000000000000000000000000
0000000000000000000000000000000000000000000000000000000000001
>Q9UIJ7_1zd8_0_226
MGASARLLRAVIMGAPGSGKGTVSSRITTHFELKHLSSGDLLRDNMLRGTEIGVLAKAFID
QGKLIPDDVMTRLALHELKNLTQYSWLLDGFPRTLPQAEALDRAYQIDTVINLNVPFEVIK
QRLTARWIHPASGRVYNIEFNPPKTVGIDDLTGEPLIQREDDKPETVIKRLKAYEDQTKPVLE
YYQKKGVLETFSGTETNKIWPYVVYAFLQTKVPQRSQKASVTP
11111100000000000000000000000000000000000000000000000000000000000000000000000000000
00000000000000000000000000000000000000000000000000000000000000000000000000000000000
0000000000000000000000000000000000000000000000000000111111111
>Q9WZQ4_2yyv_0_226
MVDVVMAPCSPVECRTAVVIDVLRATSTIVTALSNGASGVIPVKTIEEALEKKKEGVLICGER
NAQKPKGFNLGNSPLEYRKEKISGKTIVLTTTNGTQVIEKIRSEEIIAASFLNLSAVVEYLKSK
EDILLVCAGTNGRFSLEDFLLAGAIVKRLKRNDLGDGAHAAERYFESVENTREEIKKHSSH
AKRLISLGFENDIEFCTTEDLFKTVPALVNGVFILKEFP
10000000000000000000000000000000000000000000000000000000000000000000000000000000000
00000000000000000000000000000000000000000000000000000000000000000000000000000000000
0000000000000000000000000000000000000000000000000000000000011
>A0JSJ2_3dtt_0_225
MKIAVLGTGTVGRTMAGALADLGHEVTIGTRDPKATLARAEPDAMGAPPFSQWLPEHPH
VHLAAFADVAAGAELVVNATEGASSIAALTAAGAENLAGKILVDIANPLDFSHGMPPTLN
PVNTDSLGEQIQRTFPEAKVVKTLNTMNASLMVDPGRAAGGDHSVFVSGNDAAAKAEVA
TLLKSLGHQDVIDLGDITTARGAEMLLPVWIRLWGALGTANFNFKIAR
00000000000000000000000000000000000000000011111000000000000000000000000000000000000
00000000000000000000000000000000000000000000000000000000000000000000000000000000000
000000000000000000000000000000000000000000000000000000000000
>B6SEG4_4ex6_1_226
SGAPAAADRGVILDLDGTLADTPAAIATITAEVLAAMGTAVSRGAILSTVGRPLPASLAGLL
GVPVEDPRVAEATEEYGRRFGAHVRAAGPRLLYPGVLEGLDRLSAAGFRLAMATSKVEKA
ARAIAELTGLDTRLTVIAGDDSVERGKPHPDMALHVARGLGIPPERCVVIGDGVPDAEMG
RAAGMTVIGVSYGVSGPDELMRAGADTVVDSFPAAVTAVLDGHP
11111000000000000000000000000000000000000000000000000000000000000000000000000000000

00000000000000000000000000000000000000000000000000000000000000000000000000000000000
000000000000000000000000000000000000000000000000000000000011
>O53827_2fwv_0_225
MSSGAGSDATGAGGVHAAGSGDRAVAAAVERAKATAARNIPAFDDLPVPADTANLREGA
DLNNALLALLPLVGVWRGEGEGRGPDGDYRFGQQIVVSHDGGDYLNWESRSWRLTATGD
YQEPGLREAGFWRFVADPYDPSESQAIELLLAHSAGYVELFYGRPRTQSSWELVTDALARSR
SGVLVGGAKRLYGIVEGGDLAYVEERVDADGGLVPHLSARLSRFVG
11111111111111111111111111000000000000000000000000000000000000000000000000000000000
00000000000000000000000000000000000000000000000001111111111000000000000000000000000
000000000000000000000000000000000000000000000000000000000000
>Q5CYG0_2o8p_6_231
EMDERLLQKYRAQVFEWGGCFDKMFEALKSLIYLSEFENSEFDDEERHLLTLCIKHKISDYR
TMTSQVLQEQTKQLNNDELVKICSEYVFSLRKDIKAFLQSFEDCVDRLVEKSFFSKFFKLKVK
SDISRYKLEFGLCSLEDSKKIHQDAFTLLCEHPDKIEQLPLGFIQNLAYILSEKYGEKKQVFN
MLNSLGKILELQIKEQENMDRKAQITVYLQGIKDYIEK
10000000000000000000000000000000000000000000000000000000000000000000000000000000000
00000000000000000000000000000000000000000000000000000000000000000000000000000000000
000000000000000000000000000000000000000000000000000000011111
>Q82SR2_3buu_31_256
KSAVTAQSILEKADEIRFPQDSFQVNVAIRTAAPDHAEDLYRYQVLSKGNENSIVMITEPASE
RGQAILMKGRDLWVFMPSVSQPIRLSLSQRLTGQVANGDIARANFTGDYHPQLLRNESIDD
EDYYVLELTGIDRSVTYQKVLLWVNQSNFRPYKAEFYSVSGRLLKTSRYENFDNILGEMRPT
RIIMEDALKSGEVSVLDYSDMKLRDLPDKIFTKDYLKRLE
11000000000000000000000000000000000000000000000000000000000000000000000000000000000
00000000000000000000000000000000000000000000000000000000000000000000000000000000000
000000000000000000000000000000000000000000000000000000000000
>Q8ISJ0_2cw2_0_225
MLSVAGHRFASLATTPVLRMGLARCFSSVTGPFQCPPLPYVKNALEPHMSAETLTYHHDK
HHQTYVDTLNSIAAENSTIASKTLEQIIKTETGKPFNQAAQVYNHTFFFNNLAPNGGGEPT
GKIAELITRDFGSFEKFKEDFSAAAVGHFGSGWVWLIADDGKLKIVQGHDAGNPIRESKTP
LMNIDVWEHAYYIDYRNARAQYVKNYWNLVNWDFVNDNVAKAGI
11111111111111111111111111100000000000000000000000000000000000000000000000000000000
00000000000000000000000000000000000000000000000000000000000000000000000000000000000
000000000000000000000000000000000000000000000000000000000000
>Q8LAS7_2zfd_0_225
MSQCVDGIKHLCTSVLGCFDLDLYKQSGGLGDPELLARDTVFSVSEIEALYELFKKISSAVID
DGLINKEEFQLALFKTNKKESLFADRVFDLFDTKHNGILGFEEFARALSVFHPNAPIDDKIH
FSFQLYDLKQQGFIERQEVKQMVVATLAESGMNLKDTVIEDIIDKTFEEADTKHDGKIDKEE
WRSLVLRHPSLLKNMTLQYLKDITTTFPSFVFHSQVEDT
11111111111111111111111111111110000000000000000000000000000000000000000000000000000
00000000000000000000000000000000000000000000000000000000000000000000000000000000000
000000000000000000000000000000000000000000000000111111111111
>Q9Y2Q3_1yzx_0_225
MGPLPRTVELFYDVLSPYSWLGFEILCRYQNIWNINLQLRPSLITGIMKDSGNKPPGLLPRK
GLYMANDLKLLRHHLQIPIHFPKDFLSVMLEKGSLSAMRFLTAVNLEHPEMLEKASRELW
MRVWSRNEDITEPQSILAAAEKAGMSAEQAQGLLEKIATPKVKNQLKETTEAACRYGAFG
LPITVAHVDGQTHMLFGSDRMELLAHLLGEKWMGPIPPAVNARL
11100000000000000000000000000000000000000000000000000000000000000000000000000000000
00000000000000000000000000000000000000000000000000000000000000000000000000000000000
000000000000000000000000000000000000000000000000000000011111
>O14964_4avx_0_224

MGRGSGTFERLLDKATSQLLLETDWESILQICDLIRQGDTQAKYAVNSIKKKVNDKNPHVA
LYALEVMESVVKNCGQTVHDEVANKQTMEELKDLLKRQVEVNVRNKILYLIQAWAHAFR
NEPKYKVVQDTYQIMKVEGHVFPEFKESDAMFAAERAPDWVDAEECHRCRVQFGVMTR
KHHCRACGQIFCGKCSSKYSTIPKFGIEKEVRVCEPCYEQLNRKAEG
11111000000000000000000000000000000000000000000000000000000000000000000000000000
00000000000000000000000000000000000000000000000000000000000000000000000000000000
00000000000000000000000000000000000000000000000000000000000000011
>O48791_4dix_271_495
KSEENISLVYEIDGTEALGSCLRVRPCSNDAPDLSKCTIQWYRSSSDGSKKELISGATKSVYAP
EPFDVGRVLHADIIYDGHSLSLSTVGKIDPAAGLGSYVEALVRKHDVDFNVVVTQMSGEDH
TSESIHLFHVGKMRIKLCKGKTVIAKEYYSSAMQLCGVRGGGNAAAQALYWQAKKGVSFV
IAFESERERNAAIMLARRFACDCNVTLAGPEDRTETGQSP
11111110000000000000000000000000000000000000000000000000000000000000000000000000
00000000000000000000000000000000000000000000000000000000000000000000000000000000
00000000000000000000000000000000000000000000000000000000000111111
>P11350_1q16_0_224
MQFLNMFFFDIYPYIAGAVFLIGSWLRYDYGQYTWRAASSQMLDRKGMNLASNLFHIGIL
GIFVGHFFGMLTPHWMYEAWLPIEVKQKMAMFAGGASGVLCLIGGVLLLKRRLFSPRVRA
TTTGADILILSLLVIQCALGLLTIPFSAQHMDGSEMMKLVGWAQSVVTFHGGASQHLDGVA
FIFRLHLVLGMTLFLLFPFSRLIHIWSVPVEYLTRKYQLVRARH
00000000000000000000000000000000000000000000000000000000000000000000000001000000
00000000000000000000000000000000000000000000000000000000000000000000000000000000
00000000000000000000000000000000000000000000000000000000000000000
>Q2YIN4_3tcv_0_224
MTDLQNWTPRPKPERKIFEGRYVRLEPLNAQKHGDELFAASSVEDAEQRFTWLFETPPATR
AEFEPWLDKASKSDDPLFFAVIDKASGKVAGRQALMRIDPANGVIEIGSIYWGPLISRRPAAT
EAQFLFMQYVFDVLGYRRYEWECHNENGPSRRAAERFGFRFEGIFRQHMVVKGRNRDTA
WFSVLDSEWPALKQAYQAWLAPENFDSAGQQKKTLQEFRDLG
11000000000000000000000000000000000000000000000000000000000000000000000000000000
00000000000000000000000000000000000000000000000000000000000000000000000000000000
00000000000000000000000000000000000000000000000000000000000000000
>Q5NGB5_3of5_0_224
MKKFFIIGTDTEVGKTYISTKLIEVCEHQNIKSLCLKPVASGQSQFSELCEDVESILNAYKHKF
TAAEINLISFNQAVAPHIIAAKTKVDISIENLKQFIEDKYNQDLDILFIEGAGGLLTPYSDHTT
QLDLIKALQIPVLLVSAIKVGCINHTLLTINELNRHNIKLAGWIANCNDSNIKYIDEQINTIEE
LSGYKCSAKISRNADYLDFIDLSKILISPEENE
00000000000000000000000000000000000000000000000000000000000000000000000000000000
00000000000000000000000000000000000000000000000000000000000000000000000000000000
00000000000000000000000000000000000000000000000000000000000111111
>Q85307_1cq3_21_245
QSFSSSSSCTEEENKHHMGIDVIIKVTKQDQTPTNDKICQSVTEVTESEDESEEVVKGDPTTY
YTVVGGGLTMDFGFTKCPKISSISEYSDGNTVNARLSSVSPGQGKDSPAITREEALSMIKDCE
MSINIKCSEEEKDSNIKTHPVLGSNISHKKVSYEDIIGSTIVDTKCVKNLEISVRIGDMCKESSE
LEVKDGFKYVDGSASEDAADDTSLINSAKLIACV
10000000000000000000000000000000000000000000000000000000000000000000000000000000
00000000000000000000000000000000000000000000000000000000000000000000000000000000
00000000000000000000000000000000000000000000000000000000000000000
>Q8K3Y6_3u9g_0_224
MADPGVCCFITKILCAHGGRMTLEELLGEIRLPEAQLYELLETAGPDRFVLLETGGQAGITR
SVVATTRARVCRRKYCQRPCDSLHLCKLNLLGRCHYAQSQRNLCKYSHDVLSEQNFQILK
NHELSGLNQEELACLLVQSDPFFLPEICKSYKGEGRKQTCGQPQPCERLHICEHFTRGNCSY

LNCLRSHNLMDRKVLTIMREHGLSPDVVQNIQDICNNKHAR
11000000000000000000000000000000000000000000000000000011111100000000000000000000000
00000000000000000000000000000000000000000000000000000000000000000000000000000000000
00000000000000000000000000000000000000000000000000000000000
>Q8X7A5_2pof_26_250
TGEESDTLRKIVLEECLPNQQQNQNPSPCAEVKPNAGYVVLKDLNGPLQYLLMPTYRING
TESPLLTDPSTPNFFWLAWQARDFMSKKYGQPVPDRAVSLAINSRTGRTQNHFHIHISCIRP
DVREQLDNNLANISSRWLPLPGGLRGHEYLARRVTESELVQRSPFMMLAEEVPEAREHMG
SYGLAMVRQSDNSFVLLATQRNLLTLNRASAEEIQDHQCEILR
11110000000000000000000000000000000000000000000000000000000000000000000000000000000
00000000000000000000000000000000000000000000000000000000000000000000000000000000000
00000000000000000000000000000000000000000000000000000000001
>Q9JZ41_2vq2_28_252
AEKANQVSNIKTQLAMEYMRGQDYRQATASIEDALKSDPKNELAWLVRAEIYQYLKVND
KAQESFRQALSIKPDSAEINNNYGWFLCGRLNRPAESMAYFDKALADPTYPTPYIANLNKG
ICSAKQGQFGLAEAYLKRSLAAQPQFPPAFKELARTKMLAGQLGDADYYFKKYQSRVEVL
QADDLLLGWKIAKALGNAQAAYEYEAQLQANFPYSEELQTVLTGQ
11100000000000000000000000000000000000000000000000000000000000000000000000000000000
00000000000000000000000000000000000000000000000000000000000000000000000000000000000
00000000000000000000000000000000000000000000000000000000011
>Q9RRB0_2yg9_0_224
MRGGALHQNRRVTLPSVPLPAVLPPLTDHAGAVAHLSRDPVLAQVTSLCGELPVLAPTPDP
FGRLVRSVAGQQLSVKAAQAIYGRLEGLPGGVVPAALLKVSGDDLRGVGLSWAKVRTVQA
AAAAAVSGQIDFAHLSGQPDELVIAELVQLPGIGRWTAEMFLLFALARPDVFSSGDLALRQ
GVERLYPGEDWRDVTARWAPYRSLASRYLWANSARMQAGGAPL
11111111111111111100000000000000000000000000000000000000000000000000000000000000000
00000000000000000000000000000000000000000000000000000000000000000000000000000000000
00000000000000000000000000000000000000000000000000000000000
>P70227_3jrr_0_223
MNEMSSFLHIGDIVSLYAEGSVNGFISTLGLVDDRCVVEPAAGDLDNPPKKFRDCLFKVCP
MNRYSAQKQYWKAKQTKQDKEKIADVVLLQKLQHAAQMEQKQNDTENKKVHGDVVK
YGSVIQLLHMKSNKYLTVNKRLPALLEKNAMRVTLDATGNEGSWLFIQPFWKLRSNGDNV
VVGDKVILNPVNAGQPLHASNYELSDNAGCKEVNSVNCNTSWKINLF
10000000000000000000000000000000000000000000000000000000000000000000000000000011000
00000000000000000000000000000000000000000000000000000000000000000000000000000000000
0000000000000000000000000000000000000000000000000000000000
>Q12396_2a6x_51_274
DELKWNKGYSLPNLLEVTDQQKELSQWTLGDKVKLEEGRFVLTPGKNTKGSLWLKPEYSI
KDAMTIEWTFRSFGFRGSTKGGLAFWLKQGNEGDSTELFGGSSKKFNGLMILLRLDDKLGE
SVTAYLNDGTKDLDIESSPYFASCLFQYQDSMVPSTLRLTYNPLDNHLLKLQMDNRVCFQT
RKVKFMGSSPFRIGTSAINDASKESFEILKMKLYDGVIEDSL
11000000000000000000000000000000000000000000000000000000000000000000000000000000000
00000000000000000000000000000000000000000000000000000000000000000000000000000000000
0000000000000000000000000000000000000000000000000000000111
>Q59471_1egm_0_223
MEINEKLLRQIIEDVLSEMKGSDKPVSFNAPAASAAPQATPPAGDGFLTEVGEARQGTQQD
EVIIAVGPAFGLAQTVNIVGIPHKSILREVIAGIEEEGIKARVIRCFKSSDVAFVAVEGNRLSGS
GISIGIQSKGTTVIHQQGLPPLSNLELFPQAPLLTLETYRQIGKNAARYAKRESPQPVPTLND
QMARPKYQAKSAILHIKETKYVVTGKNPQELRVAL
11111111111111111111111111111111111111111111100000000000000000000000000000000000000
00000000000000000000000000000000000000000000000000000000000000000000000000000000000

000000000000000000000000000000000000000000000000000000001
>Q65TW5_3kg4_1_224
ANRIRLHIWGDYACFTRPEMKVERVSYDVITPSAARGILSAIHWKPAINWVIDKIYVLKPIRF
ESVRRNELGAKISESKVSGAMKRKSVADLYTVIEDDRQQRAATVLKDVAYVIEAHAVMTSK
AGVDENTTKHIEMFKRRALKGQCFQQPCMGVREFPAHFALIDDNDPLPLSQLSESEFNRDL
GWMLHDIDFEHGNTPHFFRAELKNGVIDVPPFYAEEVKR
00000000000000000000000000000000000000000000000000000000000000000000001111111111111
11111111111111111100000000000000000000000000000000000000000000000000000000000000000
000000000000000000000000000111100000000000000000000011111
>Q9HKT8_3dtz_0_223
MTEIYTSVLSYRLLEGKAYSDADTRSLDRMMRSIDEFFSANPGYINFHIYRSYRTDSDVIFWY
SSRNPDLMILAKERVQASMRPIAVSSFSSISIYDESPYNAMNKKLEDSLRLPPLRYFVAYPMSK
TPDWYLLDFDTRKEIMHEHIKMALNHPDEKGIRSYTTYSFGIGDQEFVVLYEIPDIAAWSRV
TEKLREARARKWIIKETPILLGRLVDAGDIAGFLL
10000000000000000000000000000000000000000000000000000000000000000000000000000000000
00000000000000000000000000000000000000000000000000000000000000000000000000000000000
000000000000000000000000000000000000000000000000000000000
>P04786_1ois_140_362
DTIKWVTLKHNGVIFPPPYQPLPSHIKLYYDGKPVDLPPQAEEVAGFFAALLESDHAKNPVF
QKNFFNDFLQVLKESGGPLNGIEIKEFSRCDFTKMFDYFQLQKEQKKQLTSQEKKQIRLERE
KFEEDYKFCELDGRREQVGNFKVEPPDLFRGRGAHPKTGKLKRRVNPEDIVLNLSKDAPVP
PAPEGHKWGEIRHDNTVQWLAMWRENIFNSFKYVRLAA
00000000000000000000000000000000000000000000000000000000000000000000000000000000000
00000000000000000000000000000110000000000000000000000000000000000000000000000000000
00000000000000000000000000000000000000000000000000000000
>P06681_3erb_20_242
APSCPQNVNISGGTFTLSHGWAPGSLLTYSCPQGLYPSPASRLCKSSGQWQTPGATRSLSKA
VCKPVRCPAPVSFENGIYTPRLGSYPVGGNVSFECEDGFILRGSPVRQCRPNGMWDGETAV
CDNGAGHCPNPGISLGAVRTGFRFGHGDKVRYRCSSNLVLTGSSERECQGNGVWSGTEPIC
RQPYSYDFPEDVAPALGTSFSHMLGATNPTQKTKESLGR
11000000000000000000000000000000000000000000000000000111111100000000000000000000000
00000000000000000000000000000000000000000000000000000000000000000000000000000000000
00000000000000000000000000000001111111111111111111111111
>P0A4U6_3la7_0_222
MIVTQDKALANVFRQMATGAFPPVVETFERNKTIFFPGDPAERVYFLLKGAVKLSRVYEAG
EEITVALLRENSVFGVLSLLTGNKSDRFYHAVAFTPVELLSAPIEQVEQALKENPELSMLMLR
GLSSRILQTEMMIETLAHRDMGSRLVSFLLILCRDFGVPCADGITIDLKLSHQAIAEAIGSTRV
TVTRLLGDLREKKMISIHKKKITVHKPVTLSRQFT
11111000000000000000000000000000000000000000000000000000000000000000000000000000000
00000000000000000000000000000000000000000000000000000000000000000000000000000000000
00000000000000000000000000000000000000000000000001111111
>Q8A8D4_3gf6_21_243
DNDAIYYPVGDVDIERGGPALEVGEEDVLVARSFNEEDYVLDTIAQYPNDPTLGKLTFMIDL
KNQQKDQNVADFNGVGKSKLTMSLGYKDGNYPSESQVPIYTSQDVTAKYAVKLRLKGELL
VSGDEWMIDYVYAQLASLFQPYPPANFPEVFMCKGGMKLGTFDSFRRTCTFDITYDRSDLS
FSQLYFNLFINLAGQKRENRVRLRIDKESYFELYEQSEEM
11000000000000000000000000000000000000000000000000000000000000000000000000000000000
00000000000000000000000000000000000000000000000000000000000000000000000000000000000
00000000000000000000000000000000000000000000000000000011
>Q8FBQ3_2fs5_1_223
PVRASIEPLTWENAFFGVNSAIVRITSEAPLLTPDALAPWSRVQAKIAASNTGELDALQQLG

FSLVEGEVDLALPVNNVSDSGAVVAQETDIPALRQLASAAFAQSRFRAPWYAPDASGRFYA
QWIENAVRGTFDHQCLILRAASGDIRGYVSLRELNATDARIGLLAGRGAGAELMQTALNW
AYARGKTTLRVATQMGNTAALKRYIQSGANVESTAYWLYR
10000000000000000000000000000000000000000000000000000000000000000000000000000000000
00000000000000000000000000000000000000000000000000000000000000000000000000000000000
000000000000000000000000000000000000000000000000000000000
>Q9BUD6_3d34_26_248
QPLGGESICSARALAKYSITFTGKWSQTAFPKQYPLFRPPAQWSSLLGAAHSSDYSMWRKN
QYVSNGLRDFAERGEAWALMKEIEAAGEALQSVHEVFSAPAVPSGTGQTSAELEVQRRHSL
VSFVVRIVPSPDWFVGVDSLDLCDGDRWREQAALDLYPYDAGTDSGFTFSSPNFATIPQDTV
TEITSSSPSHPANSFYYPRLKALPPIARVTLVRLRQSPR
11111110000000000000000000000000000000000000000000000000000000000000000000000000000
00000000000000000000000000000000000000000000000000000000000000000000000000000000000
000000000000000000000000000000000000000000000000000000001
>A1TY68_3o14_0_221
MEINADFTKPVVIDTDQLEWRPSPMKGVERRMLDRIGGEVARATSIVRYAPGSRFSAHTHD
GGEEFIVLDGVFQDEHGDYPAGTYVRNPPTTSHVPGSAEGCTIFVKLWQFDPADRTQFSKN
MEAELGAPVEGISTSLLHEDERETVTHRKLEPGANLTSEAAGGIEVLVLDGDVTVNDEVLG
RNAWLRLPEGEALSATAGARGAKIWMKTGHLRFVRTPEV
00000000000000000000000000000000000000000000000000000000000000000000000000000000000
00000000000000000000000000000000000000000000000000000000000000000000000000000000000
0000000000000000000000000000000000000000000000000000001
>A8J7D3_3gze_29_250
GFGELKEEWRGEVVHLSWSPRAFLLKNFLSDEECDYIVEKARPKMVKSSVVDNESGKSVDS
EIRTSTGTWFAKGEDSVISKIEKRVAQVTMIPLENHEGLQVLHYHDGQKYEPHYDYFHDPV
NAGPEHGGQRVVTMLMYLTTVEEGGETVLPNAEQKVTGDGWSECAKRGLAVKPIKGDAL
MFYSLKPDGSNDPASLHGSCPTLKGDKWSATKWIHVAPIGG
11111110000000000000000000000000000000000000000000000000000000000000000000000000000
00000000000000000000000000000000000000000000000000000000000000000000000000000000000
0000000000000000000000000000000000000000000000000000001
>D0ZRV8_4iqn_0_221
MDISLTNLIELVKKVNRNKVPTPMSAEEISRLRVRKYRDPQNTETTELPESLKALLAYDRDLL
SNYNMPVIETLQKSIDNEGVIHSYSPDEEAYYGVGMDSSGIDIEDLMPVWSNDPRLPALIRID
HVGDQAIFIYITERDANGEYPIARMERNEFWLAESSLVEYLYNIISGAKDIGFTEEDLHLPQW
KAQQKMNEQRDAALLDLEDYHEAFWAKLDALVD
00000000000000000000000000000000000000000000000000000000000000000000000000000000000
00000000000000000000000000000000000000000000000000000000000000000000000000000000000
0000000000000000000000000000000000000000000000000000011
>P08294_2jlp_18_239
WTGEDSAEPNSDSAEWIRDMYAKVTEIWQEVMQRRDDDGALHAACQVQPSATLDAAQP
RVTGVVLFRQLAPRAKLDAFFALEGFPTEPNSSSRAIHVHQFGDLSQGCESTGPHYNPLAVP
HPQHPGDFGNFAVRDGSLWRYRAGLAASLAGPHSIVGRAVVVHAGEDDLGRGGNQASV
ENGNAGRRLACCVVGVCGPGLWERQAREHSERKKRRRESECKAA
11111111111111111111111111111111111110000000000000000000000000000000000000000000000
00000000000000000000000000000000000000000000000000000000000000000000000000000000000
0000000000000000000000000000000000000001111111111111111
>P76318_2icu_0_221
MCGRFAQSQTREDYLALLAEDIERDIPYDPEPIGRYNVAPGTKVLLLSERDEHLHLDPVFW
GYAPGWWDKPPLINARVETAATSRMFKPLWQHGRAICFADGWFEWKKEGDKKQPFFIYR
ADGQPIFMAAIGSTPFERGDEAEGFLIVTAADQGLVDIHDRRPLVLSPEAAREWMRQEISG
KEASEIAASGCVPANQFSWHPVSRAVGNVKNQGAELIQPV

1100000000000000000000000000000000000000000000000000000000000000001000000000000000
000000000000000000000000111111000000000000000000000000000000000000000000000000000000
00000000000000000000000000000000000000000000000000000000
>Q3M5Q1_2o2g_0_221
MDRTLTHQPQEYAVSVSVGEVKLKGNLVIPNGATGIVLFAHGSGSSRYSPRNRYVAEVLQQ
AGLATLLIDLLTQEEEEIDLRTRHLRFDIGLLASRLVGATDWLTHNPDTQHLKVGYFGASTG
GGAALVAAAERPETVQAVVSRGGRPDLAPSALPHVKAPTLLIVGGYDLPVIAMNEDALEQ
LQTSKRLVIIPRASHLFEEPGALTAVAQLASEWFMHYLR
11111100000000000000000000000000000000000000000000000000000000000000000000000000000
00000000000000000000000000000000000000000000000000000000000000000000000000000000000
00000000000000000000000000000000000000000000000000000000
>Q5KWH6_3c65_368_589
LGERLGIPAPRRIEAFDNSNIYGADPVSALVVFLDGKPAKKEYRKYKVKTVAGPNDYETMR
EVVRRRYTRVLKEGLPLPDLIIIDGGKGHLSAVRDVLENELGLDVPLAGLAKDEKHRTSELL
AGDPPDVVPLDRQSQEFYLLQRIQDEVHRFAVMFHRKTRQKTMFHSVLDDIPGVGEKRKK
ALLNYFGSVKKMKEATVEELQRANIPRAVAEKIYEKLHE
00000000000000000000000000000000000000000000000000000000000000000000000000000000000
00000000000000000000000000001111111000000000000000000000000000000000000001111111111
11111111111111111111111111111111111111111111111111111111
>Q8DQK6_2a6b_0_221
METQDYAFQPGLTVGELLKSSQKDWQAAINHRFVKELFAGTIENKVLKDYLIQDYHFFDAF
LSMLGACVAHADKLESKLRFAKQLGFLEADEDGYFQKAFKELKVAENDYLEVTLHPVTKA
FQDLMYSAVASSDYAHLLVMLVIAEGLYLDWGSKDLALPEVYIHSEWINLHRGPFFAEWVQ
FLVDELNRVGKNREDLTELQQRWNQAVALELAFFDIGYDV
11000000000000000000000000000000000000000000000000000000000000000000000000000000000
00000000000000000000000000000000000000000000000000000000000000000000000000000000000
00000000000000000000000000000000000000000000000000000001
>Q983D6_3b5e_0_221
MIGDGIENSPLLTDLAFPYRLLGAGKESRECLFLLHGSGVDETTLVPLARRIAPTATLVAARG
RIPQEDGFRWFERIDPTRFEQKSILAETAAFAAFTNEAAKRHGLNLDHATFLGYSNGANLVS
SLMLLHPGIVRLAALLRPMPVLDHVPATDLAGIRTLIIAGAADETYGPFVPALVTLLSRHGA
EVDARIIPSGHDIGDPDAAIVRQWLAGPIAIAQAD
11000000000000000000000000000000000000000000000000000000000000000000000000000000000
00000000000000000000000000000000000000000000000000000000000000000000000000000000000
00000000000000000000000000000000000000000000000001111111
>Q9BV86_2ex4_1_222
TSEVIEDEKQFYSKAKTYWKQIPPTVDGMLGGYGHISSIDINSSRKFLQRFLREGPNKTGTSC
ALDCGAGIGRITKRLLLPLFREVDMVDITEDFLVQAKTYLGEEGKRVRNYFCCGLQDFTPEP
DSYDVIWIQWVIGHLTDQHLAEFLRRCKGSLRPNGIIVIKDNMAQEGVILDDVDSSVCRDL
DVVRRIICSAGLSLLAEERQENLPDEIYHVYSFALR
00000000000000000000000000000000000000000000000000001111000000000000000000000000000
00000000000000000000000000000000000000000000000000000000000000000000000000000000000
00000000000000000000000000000000000000000000000000000000
>E3SET7_3pkw_0_220
MTSIKQLTLYTAELDRMLAFYTNMLGAQHVHEQADAFTIQLGVSQIQFRAAADGTKPFYH
IAINIAANHFQEGKAWLSGFGELLTENDEDQAYFPFFNAYSCYVEDPSGNIIELISRQQAAPV
LDKPFSADQLLSIGEINITTSDVEQAATRLKQAELPVKLDQIEPAGLNFIGDQDLFLLLGPPG
RRWLFSERVAVIYPLQMELDNGVSLAITETGELVI
11000000000000000000000000000000000000000000000000000000000000000000000000000000000
00000000000000000000000000000000000000000000000000000000000000000000000000000000000
0000000000000000000000000000000000000000000000000000000

>P13000_1dts_0_224
MSKRYFVTGTDTEVGKTVASCALLQAAKAAGYRTAGYKPVASGSEKTPEGLRNSDALALQ
RNSSLQLDYATVNPYTFAEPTSPHIISAQEGRPIESLVMSAGLRALEQQADWVLVEGAGGWF
TPLSDTFTFADWVTQEQLPVILVVGVKLGCINHAMLTAQVIQHAGLTLAGWVANDVTPPG
KRHAEYMTTLTRMIPAPLLGEIPWLAEAATGKYINLALL
10000000000000000000000000000000000000000000000000000000000000000000000000000000000
00000000000000000000000000000000000000000000000000000000000000000000000000000000000
0000000000000000000000000000000000000000000000000000000
>P17067_1ekj_107_327
TTSSSDGIPKSEASERIKTGFLHFKKEKYDKNPALYGELAKGQSPPFMVFACSDSRVCPSHVL
DFQPGEAFVVRNVANLVPPYDQAKYAGTGAAIEYAVLHLKVSNIVVIGHSACGGIKGLLSF
PFDGTYSTDFIEEWVKIGLPAKAKVKAQHGDAPFAELCTHCEKEAVNASLGNLLTYPFVRE
GLVNKTLALKGGYYDFVKGSFELWGLEFGLSSTFSV
11111111000000000000000000000000000000000000000000000000000000000000000000000000000
00000000000000000000000000000000000000000000000000000000000000000000000000000000000
0000000000000000000000000000000000000000000000000000000
>P39805_1h99_56_276
EKFKTLLYDIPIECMEVSEEIISYAKLQLGKKLNDSIYVSLTDHINFAIQRNQKGLDIKNALLW
ETKRLYKDEFAIGKEALVMVKNKTGVSLPEDEAGFIALHIVNAELNEEMPNIINITKVMQEI
LSIVKYHFKIEFNEESLHYYRFVTHLKFFAQRLFNGTHMESQDDFLLDTVKEKYHRAYECTK
KIQTYIEREYEHKLTSDELLYLTIHIERVVKQA
00000000000000000000000000000000000000000000000000000000000000000000000000000000000
00000000000000000000000000000000000000000000000000000000000000000000000000000000001
1000000000000000000000000000000000000000000000000000011
>Q04178_2xbu_0_220
MSANDKQYISYNNVHQLCQVSAERIKNFKPDLIIAIGGGGFIPARILRTFLKEPGVPTIRIFAII
LSLYEDLNSVGSEVEEVGVKVSRTQWIDYEQCKLDLVGKNVLIVDEVDDTRTTLHYALSELE
KDAAEQAKAKGIDTEKSPEMKTNFGIFVLHDKQKPKKADLPAEMLNDKNRYFAAKTVPD
KWYAYPWESTDIVFHTRMAIEQGNDIFIPEQEHKQ
11110000000000000000000000000000000000000000000000000000000000000000000001111111111
00000000000000000000000000000000000000000000000000000000000000000000000000000000000
0000000000000000000000000000000000000000000000000000111
>Q2GI86_3l3b_0_220
MALNSAVILAGCGHMDGSEIREAVLVMLELDRHNVNFKCFAPNKNQKQVVDHKKKESV
GEVRNILVESARIARGSVYDIEQIRVEEFDMLVIPGGYGVAKNFSNLFDEDKENDYILPEFKN
AVREFYNAKKPIGAVCISPAVVVALLKDIAKVKVTIGEDSNGLIDKMGGVHVDCPTIKSVKD
DVNRIFSCSAYMRNDSLYNVYLGIQDMISSMVNYLKSK
10000000000000000000000000000000000000000000000000000000000000000000000000000000000
00000000000000000000000000110000000000000000000000000000000000000000000000000000000
0000000000000000000000000000000000000000000000000000011
>Q46SA5_3ihu_0_220
MPIDTPTDASPADGSASDTVFFGIMSGLELGTFVPGQRLVETDLVAHFGVGRNSVREALQRL
AAEGIVDLQRHRGAVIRRLSLQETLDVLDVAERMTGLLARAATRGSGNQPQVQALRASVQ
ALVAAEKAQDGETFSNARRHFYRTLLEMGDNRELRRLFPTIHMPIVHAQHRLASLRQMRL
DDYRRIATAVLAGEPDAAEAAGAAHVKNVRGAILDRQPA
11111111111111000000000000000000000000000000000000000000000000000000000000000000000
00000000000000000000000000000000000000000000000000000000000000000000000000000000000
0000000000000000000000000000000000000000000000000000011
>Q57884_2hf9_0_220
MHLVGVLDIAKDILKANKRLADKNRKLLNKHGVVAFDFMGAIGSGKTLLIEKLIDNLKDK
YKIACIAGDVIAKFDAERMEKHGAKVVPLNTGKECHLDAHLVGHALEDLNLDEIDLLFIE

NVGNLICPADFDLGTHKRIVVISTTEGDDTIEKHPGIMKTADLIVINKIDLADAVGADIKKM
ENDAKRINPDAEVVLLSLKTMEGFDKVLEFIEKSVKEVK
11111111110000000000000000000000000000000000000000000000000000000000000000000000
00000000000000000000000000000000000000000000000000000000000000000000000000000000
0000000000000000000000000000000000000000000000000000000000000
>Q5EAQ1_2i2o_1_221
ENSSKEDYKIQSFDLETQKLLKTALKDPGSVDLEKVSSVIVDQSLKDQVFSREAGRICYTIVQ
AEAKQTNGSVFRRNLLNRLQQEFKAREETRKRSTQEWVCLVSFICNIFDYLKVNNMPMVA
LVHPVYDCLFRLAQSDALKNEEEVDCLVLQLHRIGDQLEKMNVQLMDELFNLLRDGFLLQ
EDLSSMGRLLLLEILEFRAGGWKLSDTAQKYYYSEVTD
11111000000000000000000000000000000000000000000000000000000000000000000000000000
00000000000000000000000000000000000000000000000000000000000000000000000000000000
0000000000000000000000000000000000000000000000000000000011111
>Q6DNF2_2ree_218_438
SLNCFENNYYNLRHPKIEDLRDLIALETLCWSENLQVDNEEIYRRIFKIPQGQFILELEDKIV
GAIYSQRIDNPQLLDNKTCTQVPLLHTESGVVVQLLAVNILPELQNQGLGDRLLEFMLQYC
AQISGVEKVVAVTLCRNYPDYSPMPMAEYIHQKNESGLLVDPLLRFHQIHGAKIEKLLPGY
RPKDWENQTCGVLVSYDIQHRQRFDGATVEKNRQTK
11111110000000000000000000000000000000000000000000000000000000000000000000000000
00000000000000000000000000000000000000000000000000000000000000000000000000000000
0000000000000000000000000000000000000000000000011111111111111
>Q81PQ3_4fet_32_252
FSNQVIQRGASGEDVIELQSRLKYNGFYTGKVDGVFGWGTYWALRNFQEKFGLPVDGLAG
AKTKQMLVKATKYDKSTANKGNSGGTAQENKPSQNKGTNVPNGYSQNDIQLMANAVYG
ESRGEPYLGQVAVAAVILNRVTSASFPNTVSGVIFEPRAFTAVADGQIYLTPNETAKKAVLDA
INGWDPTGNALYYFNPDTATSKWIWTRPQIKKIGKHIFCK
11111111111111111111111111111111111111111111111111111111111111111111111111111111
11111111111111000000000000000000000000000000000000000000000000000000000000000000
0000000000000000000000000000000000000000000000000000000000000
>Q89ZG6_3see_12_232
AGVSISAQNLIKNEKFATEVTNKVTNPSKATAGEWFIMNNEADGVTTIAWEQTGDAKYPN
AMKIDNSGAEKNTSWYKAFLGQRITDGLEKGIYVLTFYAKAKEAGTPVSVYIKQTNEEKND
NGKLNTTFFMRRDYDADAQPNASGAQYNFKIKDADKWTKVVVYYDMGQVVNAISSKKS
NPALEVSDTDDDAAILKDCYVAILGQNKGGVVEISDVTLKKK
11111110000000000000000000000000000000000000000000000000000000000000000000000000
00000000000000000000000000000000000000000000000000000000000000000000000000000000
0000000000000000000000000000000000000000000000000000000000000
>A3DCJ4_2xqo_130_349
PANTQSGILNDGYFPPGTSKHELIARASSLKVSEVKAIIKKQVDEHWDVIRDVCGFKNKEVA
YAFFFGMATRESTFRAATETGSGASHAFGPLQTAETAYANANPNYMPEHNVPEMHQYDFT
EYNFYDVGISVHMGIRHFLHFARLAKEKYSGRDIARHGLMGYNTGWIDGADESWIVRYAD
ETAALGAWYLRNNHMSDDEFTWDTDPRVDRSNPWEIYY
11111111111000000000000000000000000000000000000000000000000000000000000000000000
00000000000000000000000000000000000000000000000000000000000000000000000000000000
000000000000000000000000000000000000000000000000000000000000
>A9CHH7_2axo_1_220
KGVVELFTSQGCASCPPADEALRKMIQKGDVVGLSYHVDYWNYLGWTDSLASKENTERQ
YGYMRALGRNGVYTPQAILNGRDHVKGADVRGIYDRLDAFKREGQGLNVPVSSKFAGDE
VEIDIGAGNGKADVVVAYFTREQTVDVQKGENQGKKMSYWHSVYDVQTVGMWDGSPM
TVKLPASVVAKVKKGGCAVLLQTANASGDPAAIVGASILLGNETQ
00000000000000000000000000000000000000000000000000000000000000000000000000000000

00000000000000000000000000000000000000000000000000000000000000111111100000000000000
000000000000000000000000000000000000000000000000000000
>E0X765_4ibn_0_219
MPETRRSAGSDIPTPLVAVYADESCLGNGREGENPGGAGVLVEYARPGGAGDIVRRDVWVS
EPATTNNRMALRSVIEAFRAIGHKGTRFRVVFTTDSRYIVDGMTRWVHDWAQRGWKRKS
GAIENLALWQEAVQAVNGHAVEWRWVRGHAGHAQNEYANHLAVTAAGGQTQSGGLV
DSGYEEWAARVSTAASRMRLEPFPDAAAFRPSPALPVVAAGRPS
11111111100000000000000000000000000000000000000000000000000000000000000000000000000
00000000000000000000000000000000000000000000000000000000000000000000000000000000000
000000000000000000000000000000000000000000000000000000
>P76458_1k6d_0_219
MKTKLMTLQDATGFFRDGMTIMVGGFMGIGTPSRLVEALLESGVRDLTLIANDTAFVDTGI
GPLIVNGRVRKVIASHIGTNPETGRRMISGEMDVVLVPQGTLIEQIRCGGAGLGGFLTPTGV
GTVVEEGKQTLTLDGKTWLLERPLRADLALIRAHRCDTLGNLTYQLSARNFNPLIALAADI
TLVEPDELVETGELQPDHIVTPGAVIDHIIVSQESK
00000000000000000000000000000000000000000000000000000000000000000000000000000000000
00000000000000000000000000000000000000000000000000000000000000000000000000000000000
000000000000000000000000000000000000000000000000000001
>Q02KA8_4ecj_0_219
MIDLYTAATPNGHKVSIALEEMGLPYRVHALSFDKKEQKAPEFLRINPNGRIPAIVDRDND
DFAVFESGAILIYLAEKTGQLMPADVKGRSRVIQWLMFQMGGVGPMQGQANVFFRYFPEK
LQGAIDRYQHETRRLYEVLDGRLGEAEYLAGDYSIADIATYPWVRIHDWSGVAVDGLDNL
QRWIAAIEARPAVQRGLLVPRREKEGDDAIRTAQSMLTR
00000000000000000000000000000000000000000000000000000000000000000000000000000000000
00000000000000000000000000000000000000000000000000000000000000000000000000000000000
000000000000000000000000000000000000011111111111111111
>Q10337_3l40_658_877
TNAKASKRVYITFTGYDKKPSIDNLKKLDMSITSNPSKCTHLIAPRILRTSKFLCSIPYGPCVV
TMDWINSCLKTHEIVDEEPYLLNDPEKELELGCTLESALKRARAQGPSLLEDYVVYLTSKTV
APENVPAVISIVKSNGGVCSTLNVYNKRLARHLEDGNVVLITCNEDSHIWTNFLDNASQN
KTIFLQNYDWLIKTVLRQEIDVNDRIADEFARAV
00000000000000000000000000000000000000000000000000000000000000000000000000000000000
00000000000000000000000000000000000000000000000000000000000000000000000000000000000
000000000000000000000000000000000000000000000000000011
>Q1AN22_2v2g_0_219
MGITLGEVFPNFEADSTIGKLKFHDWLGNSWGVLFSHPRDFTPVCTTELGRVIQLEGDFKK
RGVKLIALSCDNVADHKEWSEDVKCLSGVKGDMPYPIIADETRELAVKLGMVDPDERTST
GMPLTCRAVFIIGPDKKLKLSILYPATTGRNFSEILRVIDSLQLTAQKKVATPADWQPGDRCM
VVPGVSAEEAKTLFPNMEVKAVPSGKGYLRYTPQPK
10000000000000000000000000000000000000000000000000000000000000000000000000000000000
00000000000000000000000000000000000000000000000000000000000000000000000000000000000
000000000000000000000000000000000000000000000000000000
>Q5LQD5_2imh_1_220
TFSILAHDPETGAIGGAAATGSLCVGGWVLRGDLNAGMSASQGAAPSTFWGEEVLQHLR
DGSHPEDAVNHVTSQDSGRAYRQLAAMDLLGNAAAFTGSENQDIKGSVTFASGIASGNM
LGDNSVLGAMTEAFVASDLTFERRLLAALIAAEGAGSDFRGLLSAAMLVLHPDRPPVTLRI
DYHPDNPIGALEQLYQKATTGDYADWARQVPVLSDKERILD
00000000000000000000000000000000000000000000000000000000000000000000000000000000000
00000000000000000000000000000000000000000000000000000000000000000000000111100000000
000000000000000000000000000000000000000000000000000000
>Q7NRS2_3keb_0_219

MEDFWVQYGDEMLPVIGDFPRKGDYLPSFMLVDDQKHDAALESFSHTPKLIVTLLSVDED
EHAGLLLLRETRRFLDSWPHLKLIVITVDSPSSLARARHEHGLPNIALLSTLRGRDFHKRYG
VLITEYPLSGYTSPAIILADAANVVHYSERLANTRDFFDFDAIEKLLQEGEQQAMAAEREAA
EARQEQDAEREKGTERLLEKAKLIQDQLNRNGDPGR
00000000000000000000000000000000000000000000000000000000000000000000000000000000000
00000000000000000000000000000000000000000000000000000000000000000000000000000000000
00000000001111111111111111111111111111111111111111111
>Q8ZRS0_3qy1_0_219
MKDIDTLISNNALWSKMLVEEDPGFFEKLAQAQKPRFLWIGCSDSRVPAERLTGLEPGELFV
HRNVANLVIHTDLNCLSVVQYAVDVLEVEHIIICGHSGCGGIKAAVENPELGLINNWLLHIR
DIWLKHSSLLGKMPEEQRLDALYELNVMEQVYNLGHSTIMQSAWKRGQNVTIHGWAYSI
NDGLLRDLDVTATNRETLENGYHKGISALSLKYIPHQ
10000000000000000000000000000000000000000000000000000000000000000000000000000000000
00000000000000000000000000000000000000000000000000000000000000000000000000000000000
00000000000000000000000000000000000000000000000000111
>Q9GSV7_3sib_0_219
MQPPVANFCLWNLQPIQGSWMGAACIYQMPPSVRNTWWFPLLNTIPLDQYTRIYQWFMG
VDRDRSGTLEINELMMGQFPGGIRLSPQTALRMMRIFDTDFNGHISFYEFMAMYKFMELAY
NLFVMNDRNRSGTLEPHEILPALQQLGFYINQRTSLLLHRLFARGMAFCDLNCWIAICAFA
AQTRSAYQMIFMNPYYGPMKPFNPMEFGKFLDVVTSLLE
11111000000000001111111111110000000000000000000000000000000000000000000000000000000
00000000000000000000000000000000000000000000000000000000000000000000000000000000000
00000000000000000000000000000000000000000000000000000
>Q9WYT0_1o26_0_219
MKIDILDKGFVELVDVMGNDLSAVRAARVSFDMGLKDEERDRHLIEYLMKHGHETPFEHI
VFTFHVKAPIFVARQWFRHRIASYNELSGRYSKLSYEFYIPSPERLEGYKTTIPPERVTEKISEIV
DKAYRTYLELIESGVPREVARIVLPLNLYTRFFWTVNARSLMNFLNLRADSHAQWEIQQYA
LAIARIFKEKCPWTFEAFLKYAYKGDILKEVQV
00000000000000000000000000000000000000000000000000000000000000000000000000000000000
00000000000000000000000000000000000000000000000000000000000000000000000000000000000
00000000000000000000000000000000000000000000000000011
>A6B7Q2_3guy_1_219
IVITGASSGLGAELAKLYDAEGKATYLTGRSESKLSTVTNCLSNNVGYRACDLASHQEVEQL
FEQLDSIPSTVVHSAGSGYFGLLQEQDPEQIQTLIENNLSSAINVLRELVKRYKDQPVNVVMI
MSTAAQQPKAQESTYCAVKWAVKGLIESVRLELKGKPMKIIAVYPGGMATEFWETSGKSLD
TSSFMSAEDAALMIHGSLANIGNGYVSDITVNR
00000000000000000000000000000000000000000000000000000000000000000000000000000000000
00000000000000000000000000000000000000000000000000000000000000000000000000000000000
0000000000001111111111100000000000000000000000000000
>H1AAP2_3vrk_0_218
MEKSNTDALLENNRLYAGGQATHRPGHPGMQPIQPSRRVAVVACMDARLDVEDLLGLQT
GEAHIIRNAGGVINEDAIRCLIISHHLLNTHEIILVHHTRCGMLAFTDDLLRAGLEGDAAAE
KLIGQATGRAFVSAGKASASPAAFQAFRGPPEPLDAPRSDASTERIAADVRRGLSIILNHPW
LPTAGPDAITVRGFIYDVDTGRLEEVSYPGPMGGFG
11110000000000000000000000000000000000000000000000000000000000000000000000000000000
00000000000000000000000000000000000000000000000000000000000000000000000000000000000
0000000000000000000000000000000000000000000000000111
>O28869_1y7p_0_218
MLRGLRIIAENKIGVLRDLTTIIAEEGGNITFAQTFLIKHGEHEGKALIYFEIEGGDFEKILERV
KTFDYIIEIEEEESFERVFGKRVIILGGGALVSQVAIGAISEADRHNLRGERISVDTMPVVGEEE
IAEAVKAVSRLHRAEVLVLAGGIMGGKITEEVKKLRKSGIRVISLSMFGSVPDVADVVISDPV

MAGTLAVMHISEKAKFDLDRVKGRRI
10000000000000000000000000110000000000000000000000000000000000000000000000000000000
00000000000000000000000000000000000000000000000000000000000000000000000000000000000
00000000000000000000000000000000000000000000000000011
>O29524_3o7b_0_218
MGSAFVFLEASLELIPQKIRGHPAVRADAIRRGKRPEKILLDDSKHHTAMKSLEFREKRGRP
DIVHQCLLLLLDSPLRDFEVYVHTLNGEIIWVNRETRLPRNYNRFVGLMEKLFEERRITAGD
TTLIEFKDVGLRDIVRGRDVLLFREKGGRFEFSELLDGDVAVCIGAFPHGDFFEETLRELGEF
KEVSLGTESYTSLYVTSRVLCEYERVRAHKVG
00000000000000000000000000000000000000000000000000000000000000000000000000000000000
00000000000000000000000000000000000000000000000000000000000000000000000000000000000
00000000000000000000000000000000000000000000000000111
>O60942_2c46_0_218
MAHNKIPPRWLNCPRRGQPVAGRFLPLKTMLGPRYDSQVAEENRFHPSMLSNYLKSLKVK
MGLLVDLTNTSRFYDRNDIEKEGIKYIKLQCKGHGECPTTENTETFIRLCERFNERNPPELIG
VHCTHGFNRTGFLICAFLVEKMDWSIEAAVATFAQARPPGIYKGDYLKELFRRYGDIEEAPP
PPLLPDWCFEDDEDEDEDEDGKKESEPGSSASFG
11100000000000000000000000000000000000000000000000000000000000000000000000000000000
00000000000000000000000000000011111100000000000000000000000000000000000000000000000
00000000000000000000000000000000011111111111111111111
>O66680_3o0a_224_442
GRSEGALIRFYVEIEEPEKFLNCVPEELKETLLKEKRIYIDVFTTRPDTVFGATFVVLAPEHPLV
PVLACIGERLGNACYSDVENFVEKMKKMSTRERTMEEDKEGVFLGVYATNPANGEKIPVW
SANYVLYEYGTGAIMCVPAHDQRDWEFAKKYDLPIKVVVKPEGAWDFEKGAYEGKGTLV
NSDGFDGLDSETAKRKITEWLQDRGLGEKKVSYRL
11100000000000000000000000000000000000000000000000000000000000000000000000000000000
00000000000000000000000000000000000000000000000000000000000000000000000000000000000
00000000000000000000000000000000000000000000000000011
>P01870_2vuo_104_322
CPPPELLGGPSVFIFPPKPKDTLMISRTPEVTCVVVDVSQDDPEVQFTWYINNEQVRTARPPL
REQQFNSTIRVVSTLPITHQDWLRGKEFKCKVHNKALPAPIEKTISKARGQPLEPKVYTMGP
PREELSSRSVSLTCMINGFYPSDISVEWEKNGKAEDNYKTTPAVLDSDGSYFLYNKLSVPTSE
WQRGDVFTCSVMHEALHNHYTQKSISRSPGK
10000000000000000000000000000000000000000000000000000000000000000000000000000000000
00000000000000000000000000000000000000000000000000000000000000000000000000000000000
00000000000000000000000000000000000000000000000000111
>P13726_1jps_32_250
SGTTNTVAAYNLTWKSTNFKTILEWEPKPVNQVYTVQISTKSGDWKSKCFYTTDTECDLTD
EIVKDVKQTYLARVFSYPAGNVESTGSAGEPLYENSPEFTPYLETNLGQPTIQSFEQVGTKVN
VTVEDERTLVRRNNTFLSLRDVFGKDLIYTLYYWKSSSSGKKTAKTNTNEFLIDVDKGENYC
FSVQAVIPSRTVNRKSTDSPVECMGQEKGEFRE
11110000000000000000000000000000000000000000000000000000000000000000000000000000000
11111110000000000000000000000000000000000000000000000000000000000000000000000000000
00000000000000000000000000000000000000000000011111111
>P13901_2cxv_1519_1737
STLEIAGLVRKNLVQFGVGEKNGCVRWVMNALGVKDDWLLVPSHAYKFEKDYEMMEFYF
NRGGTYYSISAGNVVIQSLDVGFQDVVLMKVPTIPKFRDITQHFIKKGDVPRALNRLATLVT
TVNGTPMLISEGPLKMEEKATYVHKKNDGTTVDLTVDQAWRGKGEGLPGMCGGALVSSN
QSIQNAILGIHVAGGNSILVAKLVTQEMFQNIDKKIESQ
00000000000000000000000000000000000000000000000000000000000000000000000000000000000
00000000000000000000000000000000000000000000000000000000000000000000000000000000000

000000000000000000000000000000000000000000001111111
>Q1EM40_2pq7_0_218
MNLTELMERIPHLREILNIVREAFKDYDDPAHDISHTFRVMENASEIASREKCDLQKAIIAA
LLHDIKRPHEALTGVDHAESGAEYASGLLPTMGFDISFVAEVSKAIRSHRYSGGLTPTSLTGK
ILQDADRLDAIGAVAIARVFSYSGKTGTPLHSLQFSPRSSYSGNSRSSINHFHEKILKIRPETFW
TETARKMAEDRYSFVVEFVQRFLAEWGQI
00000000000000000000000000000000000000000000000000000000000000000000000000000000
00000000000000000000000000001111100000000000000000000000000000111111111111111111
111111111111111111000000000000000000000000000000011
>Q5FLP2_3f44_0_218
MDETPIFKIKKLTIAENDRSEYIRYAEKNMHDSIPAEEGTLLIGSGHDDAHGEDNYEIEVFRN
KGAEDLHIAGSHADDFVETVNKIATKQKVIDLHPEVITTKAQRALNSYADNFVMRLIKVEV
KDADAEKFSHAVKKEMTTSMASEPGMEIMMSGTNIDNPNEWYFIEVYANDEAYDIHVKTP
HYKEYIEETDGMVKSRDVKTLVRDTLATQGAIVLD
11000000000000000000000000000000000000000000000000000000000000000000000000000000
00000000000000000000011111100000000000000000000000000000000000000000000000000000
000000000000000000000000000000000000000000000000000
>Q7WLM8_1zn6_0_218
MCSHYQALKDQERMRKYFAAHPSAEVPADMWPRYMGAFIRRPLEWDSGDEAVPEREAAT
GRWGMIPPGTRPEKLAEASKKNTSNARSETAHQLWTFRNAWAKAQHCIIPADAIYEPDWR
SGKAVPTRFTRADGAPLGIAGLWDRYRNAAGEWIDSYTMLTINADDDPLFRDYHQAGKE
KRMVVILPDGAYGDWLTAPATDTRDFLLPYPADRLVAAAVK
10000000000000000000000000000000000000000011111111000000000000000000000000000000
00000000000000000000000000000000000000000000000000000000000000000000000000000000
000000000000000000000000000000000000000000000000000
>Q88RE7_3pr8_1_219
LKLYGFSVSNYYNMVKLALLEKGLTFEEVTFYGGQAPQALEVSPRGKVPVLETEHGFLSETS
VILDYIEQTQGGKALLPADPFGQAKVRELLKEIELYIELPARTCYAESFFGMSVEPLIKEKARA
DLLAGFATLKRNGRFAPYVAGEQLTLADLMFCFSVDLANAVGKKVLNIDFLADFPQAKAL
LQLMGENPHMPRILADKEASMPAFMEMIRSGKR
00000000000000000000000000000000000000000000000000000000000000000000000000000000
00000000000000000000000000000000000000000000000000000000000000000000000000000000
000000000000000000000000000000000000000000000000011
>D0VWT7_3cp7_0_217
QNPADSPHIGKVFFSTNQGDFVCSANIVASANQSTVATAGHCLHDGNGGQFARNFVFAPA
YDYGESEHGVWAAEELVTSAEWANRGDFEHDYAFAVLETKGGTTVQQQVGTASPIAFNQP
RGQYYSAYGYPAAAPFNGQELHSCHGTATNDPMGSSTQGIPCNMTGGSSGGPWFLGNGT
GGAQNSTNSYGYTFLPNVMFGPYFGSGAQQNYNYASTTN
11000000000000000000000000000000000000000000000000000000000000000000000000000000
00000000000000000000000000000000000000000000000000000000000000000000000000000000
00000000000000000000000000000000000000000000000000
>D7PC21_3ou2_0_217
MTTSHGLIESQLSYYRARASEYDATFVPYMDSAAPAALERLRAGNIRGDVLELASGTGYWT
RHLSGLADRVTALDGSAEMIAEAGRHGLDNVEFRQQDLFDWTPDRQWDAVFFAHWLAH
VPDDRFEAFWESVRSAVAPGGVVEFVDVTDHERRLEQQDDSEPEVAVRRTLQDGRSFRIVK
VFRSPAELTERLTALGWSCSVDEVHPGFLYATCRPGPR
11000000000000000000000000000000000000000000000000000000000000000000000000000000
00000000000000000000000000000000000000000000000000000000000000001111111111110000
00000000000000000000000000000000000000000000000000
>P84149_1q40_294_511
SPETMFFQDEDSRNLATNFIANYLKLWDANRSELMILYQNESQFSMQVDSSHPHLIESGNS

GYSGSTDFGYYLNNSRNLTRVSSIKARMAKLSIGQEQIYKSFQQLPKTRHDIIATPELFSMEV
YKFPTLNGIMITLHGSFDEVAQPEVDGSASSAPSGPRGGSRYHSGPKHKRIPLSKKSFDRTFV
VIPGPNGSMIVASDTLLIRPYTSDFPWKVQK
11111111110000000000000000000000000000000000000000000000011111100000000000000000000
00000000000000000000000000000000000000000000000000000000000000000000001111111111000
0000000000000000000000000011000000000000000000000011
>Q8F2Y8_3ubk_0_217
MIKLHGASISNYVNKVKLGILEKGLEYEQIRIAPSQEEDFLKISPMGKIPVLEMDGKFIFESGA
ILEFLDTIFPQTPKLIPEDPWEAARVREISTIIETYLDIPARRIYLPAAKVSPEIVEEVHSTLVKGI
KALQRVVRFSPYIAGNVFTLADCSGFAHLSVLDEELRPFYPNNHPLDLLNGWKEYFVFMKT
KAGPALVEKDKQILKKILARAKTKIG
00000000000000000000000000000000000000000000000000000000000000000000000000000000000
00000000000000000000000000011111000000000000000000000000000000000000000000000000000
0000000000000000000000000000000000000000000000011111
>A3QGX4_3bhn_0_216
MYKVGIVLFDDFTDVDFFLMNDLLGRTSDSWTVRILGTKPEHHSQLGMTVKTDGHVSEVK
EQDVVLITSGYRGIPAALQDENFMSALKLDPSRQLIGSICAGSFVLHELGLLKGKKLTTNPD
AKAVLQGMGGDVQDLPLVIEGNIATAGGCLSLLYLVGWLAERLFDSVKRKQIQNQLIPAGQ
MEIFETLISETIQSAESAYEYRSACESDAESLVV
00000000000000000000000000000000000000000000000000000000000000000000000000000000000
00000000000000000000000000000000000000000000000000000000000000000000000000000000000
000000000000000000000000000000000000000000000000111
>E9Q401_3im6_0_216
MADAGEGEDEIQFLRTDDEVVLQCTATIHKEQQKLCLAAEGFGNRLCFLESTSNSKNVPPD
LSICTFVLEQSLSVRALQEMLANTVEKSEGQVDVEKWKFMMKTAQGGGHRTLLYGHAILL
RHSYSGMYLCCLSTSRSSTDKLAFDVGLQEDTTGEACWWTIHPASKQRSEGEKVRVGDDLI
LVSVSSERYLHLSYGNSSWHVDAAFQQTLWSVAPI
11111111111000000000000000000000000000000000000000000111100000000000000000000000000
00001111111111011111111111000000000000000000000000000111111000000000000000000000000
000000000000000000000000000000000000000000000000000
>O87854_2hxi_0_216
MAGRRRWSTEQILDAAAELLLAGDAETFSVRKLAASLGTDSSSLYRHFRNKTELLRAVADRI
LLSAMDGYRPEGDWKQRLTAVALRLRESFGQQPQLAAVWGRHGSGGTGSRLMMEEVLQA
LRASGLPDDEIPARYHRLVILISSLITAEGGFGAVGAQEHEQGMEQFRVAVLGADPERFPALS
HFAREIRPLGADRGAAFEEILAAHLAHLEAAAP
11110000000000000000000000000000000000000000000000000000000000000000000000000000000
00000000000000000000000000000000000000000000000000000000000000000000011111111111111
000000000000000000000000000000000000000000000000000
>P03661_3dgs_18_234
AETVESCLAKPHTENSFTNVWKDDKTLDRYANYEGCLWNATGVVVCTGDETQCYGTWV
PIGLAIPENEGGGSEGGGSEGGGSEGGGTKPPEYGDTPIPGYTYINPLDGTYPPGTEQNPAN
PNPSLEESQPLNTFMFQNNRFRNRQGALTVYTGTVTQGTDPVKTYYQYTPVSSKAMYDAY
WNGKFRDCAFHSGFNEDPFVCEYQGQSSDLPQPPVNA
11110000000000000000000000000000000000000000000000000000000000000000011111111111111
11111111000000000000000000000000000000000000000000000000000000000000000000000000000
000000000000000000000000000000000000000000000000000
>P0AEK7_1kqf_0_216
MSKSKMIVRTKFIDRACHWTVVICFFLVALSGISFFFPTLQWLTQTFGTPQMGRILHPFFGIAI
FVALMFMFVRFVHHNIPDKKDIPWLLNIVEVLKGNEHKVADVGKYNAGQKMMFWSIMS
MIFVLLVTGVIIWRPYFAQYFPMQVVRYSLLIHAAAGIILIHAILIHMYMAFWVKGSIKGMIE
GKVSRRWAKKHHPRWYREIEKAEAKKESEEGI

10000000000000000000000000000000000000000000000000000000000000000000000000000000000
00000000000000000000000000000000000000000000000000000000000000000000000000000000000
000000000000000000000000000000000000000000000000000
>P75966_2olw_0_216
MRQFIISENTMQKTSFRNHQVKRFSSQRSTRRKPENQPTRVILFNKPYDVLPQFTDEAGRKT
LKEFIPVQGVYAAGRLDRDSEGLLVLTNNGALQARLTQPGKRTGKIYYVQVEGIPTQDALE
ALRNGVTLNDGPTLPAGAELVDEPAWLWPRNPPIRERKSIPTSWLKITLYEGRNRQVRRMT
AHVGFPTLRLIRYAMGDYSLDNLANGEWREVTD
11111111111111111111111111111111111100000000000000000000000000000000000000000000000
00000000000000000000000000000000000000000000000000000000000000000000000000010000000
000000000000000000000000000000000000000000000000000
>Q5SLQ3_2qhs_0_216
MEFLVEDLGLVPYGEAWAYQKRVHREVVAGNRPPTLLLLEHPRVITLGRKATGENLLFPES
WYRENGFELYWVERGGDVTYHGPGQLVGYPIFPVGREVRRFLRQIEEAIVRVAAGYGISAYP
TPGYAGVWVGEDKLCAIGVAVKEGVSFHGFALNVNTDLNDFTVIVPCGLKGKGVTSLEKL
LGRKVPMEEAKARVVAAFAEVFGLRPVEGSVHEA
00000000000000000000000000000000000000000000000000000000000000000000000000000000000
00000000000000000000000000000000000000000000000000000000000000000000000000000000000
000000000000000000000000000000000000000000001111111
>Q7CR15_3lhq_0_216
MARKTKQQALETRQHILDVALRLFSQQGVSATSLAEIANAAGVTRGAIYWHFKNKSDLFS
EIWELSESNIGELEIEYQAKFPDDPLSVLREILVHILEATVTEERRRLLMEIIFHKCEFVGEMVV
VQQAQRSLCLESYDRIEQTLKHCINAKMLPENLLTRRAAILMRSFISGLMENWLFAPQSFDL
KKEARAYVTILLEMYQLCPTLRASTVNGSP
00000000000000000000000000000000000000000000000000000000000000000000000000000000000
00000000000000000000000000000000000000000000000000000000000000000000000000000000000
000000000000000000000000000000000000000000000111111
>Q8DWD0_3mvp_0_216
MSNFEKRNRKMAEKNIRKPKQERSIEKRNKILQVAKDLFSDKTYFNVTTNEIAKKADVSVG
TLYAYFASKEDILTALLKRYNDFFLTTIFADINSQDSLDRFKKNPKEWLNVLINQLLAAEDKIF
HAQIEMLAYAIPQAKALLEEHNNNLKNLTYKCLLYYSDQAANPSFKTLSLVVFDFISALVDE
LLYHEHTQEEAHQIKKTGIDSLDLIIKSYL
11111111111110000000000000000000000000000000000000000000000000000000000000000000000
00000000000000000000000000000000000000000000000000000000000000000000000000000000000
000000000000000000000000000000000000000000000000000
>Q8T8F4_4g2u_19_235
EAGFCCPADLNQTDEARIFLDFHNQVRRDIAGASPLLNLTGAVQMRNVLGPAKNMYRMD
WDCNLEAKAKAMIWPCTTPLPIDTSIPQNLAQWLLFQNSQEEVLTQTPWSWVTASLRNLQ
PDTEANIYNWQIRPLSNIANWQNLKVGCAHKVCKFPTGTNMVVSCAYGGEVLQDNEVV
WDKGPTCMCNAYPNSFCCNNLCDTIAAATLRNQPCKST
00000000000000000000000000000000000000011000000000000000000000000000000000000000000
00000000000000000000000000000000000000000000000000000000000000000000000000000000000
0000000000000000000000000000000000000000000000011
>Q96Q83_2iuw_69_285
RVIDREGVYEISLSPTGVSRVCLYPGFVDVKEADWILEQLCQDVPWKQRTGIREDITYQQPR
LTAWYGELPYTYSRITMEPNPHWHPVLRTLKNRIEENTGHTFNSLLCNLYRNEKDSVDWH
SDDEPSLGRCPIIASLSFGATRTFEMRKKPPPEENGDYTYVERVKIPLDHGTLLIMEGATQAD
WQHRVPKEYHSREPRVNLTFRTVYPDPRGAPW
00000000000000000000000000000000000000000000000000000000000000000000000000000000000
00000000000000000000000000000000000000000000000000000000000000000000001111111100000
000000000000000000000000000000000000000000001111111

>Q98I56_2o2x_0_216
MADKTGTPHPLTEPGVWIERIGGRVFPPHLPALFLDRDGTINVDTDYPSDPAEIVLRPQMLP
AIATANRAGIPVVVVTNQSGIARGYFGWSAFAAVNGRVLELLREEGVFVDMVLACAYHEA
GVGPLAIPDHPMRKPNPGMLVEAGKRLALDLQRSLIVGDKLADMQAGKRAGLAQGWLV
DGEAAVQPGFAIRPLRDSSELGDLLAAIETLGRDNRS
11111110000000000000000000000000000000000000000000000000000000000000000000000000000
00000000000000000000000000000000000000000000000000000000000000000000000000000000000
000000000000000000000000000000000000000000000000001
>Q9C8L2_4dol_70_286
KSAASSVGNAEDYAEETATSVKFKRSVTLPGCSSPLSLLGTGFREKKFAIIGVKVYAAGYYVN
ESILSGLSAWTGRSADEIQRDSSLFVSIFQAQAEKSLQIVLVRDVDGKTFWDALDEAISPRIKS
PSSEDTTALSTFRGIFQNRPLNKGSVILLTWINTSNMLVSVSSGGLPTNVDATIESGNVTSALF
DVFFGDSPVSPTLKSSVANQLAMTLV
11111111110000000000000000000000000000000000000000000000000000000000000000000000000
00000000000000000000000000000000000000000000000000000000000000000000000000000000000
000000000000000000000000000000000000000000000000000
>Q9H2K2_3p0q_945_1161
LNTSGSGTILIDLSPDDKEFQSVEEEMQSTVREHRDGGHAGGIFNRYNILKIQKVCNKKLWE
RYTHRRKEVSEENHNHANERMLFHGSPFVNAIIHKGFDERHAYIGGMFGAGIYFAENSSKS
NQYVYGIGGGTGCPVHKDRSCYICHRQLLFCRVTLGKSFLQFSAMKMAHSPPGHHSVTGR
PSVNGLALAEYVIYRGEQAYPEYLITYQIMRPEG
11111100000000000000000000000000000000000000000000000000000000000000000000000000000
00000000000000000000000000000000000000000000000000000000000000000000000000000000000
001000000000000000000000000000000000000000000000001
>Q9LZX1_1zxu_0_216
MEQPYVVYAYPQGSGPSGAPTPQAGGVVVDPKYCAPYPIDMAIVRKMMSLTDGNFVITDVN
GNLLFKVKEPVFGLHDKRVLLDGSGTPVVTLREKMVSMHDRWQVFRGGSTDQRDLLYTV
KRSSMLQLKTKLDVFLGHNKDEKRCDFRVKGSWLERSCVVYAGESDAIVAQMHRKHTVQ
SVFLGKDNFSVTVYPNVDYAFIASLVVILDDVNREDRAA
11111111111111111111111000000000000000000000011111000000000000000000000000000000000
00000000001111110000000000000000000000111111100000000000010000000000000000000000000
000000001111111100000000000000000000000000000011111
>A0R5U7_2q0q_0_215
MAKRILCFGDSLTWGWVPVEDGAPTERFAPDVRWTGVLAQQLGADFEVIEEGLSARTTNI
DDPTDPRLNGASYLPSCLATHLPLDLVIIMLGTNDTKAYFRRTPLDIALGMSVLVTQVLTSA
GGVGTTYPAPKVLVVSPPPLAPMPHPWFQLIFEGGEQKTTELARVYSALASFMKVPFFDAG
SVISTDGVDGIHFTEANNRDLGVALAEQVRSLL
10000000000000000000000000000000000000000000000000000000000000000000000000000000000
00000000000000000000000000000000000000000000000000000000000000000000000000000000000
00000000000000000000000000000000000000000000000000
>P31489_1p9h_25_240
DDYDGIPNLTAVQISPNADPALGLEYPVRPPVPGAGGLNASAKGIHSIAIGATAEAAKGAA
VAVGAGSIATGVNSVAIGPLSKALGDSAVTYGAASTAQKDGVAIGARASTSDTGVAVGFNS
KADAKNSVAIGHSSHVAANHGYSIAIGDRSKTDRENSVSIGHESLNRQLTHLAAGTKDTDA
VNVAQLKKEIEKTQENTNKRSAELLANANAYAD
11111100000000000000000000111111111100000000000000000000000000000000000000000000000
00000000000000000000000000000000000000000000000000000000000000000000000000000000000
00000000000000000000000000000111111111111111111111
>P96222_3sdg_0_215
MTTSAASQASLPRGRRTARPSGDDRELAILATAENLLEDRPLADISVDDLAKGAGISRPTFYF
YFPSKEAVLLTLLDRVVNQADMALQTLAENPADTDRENMWRTGINVFFETFGSHKAVTRA

GQAARATSVEVAELWSTFMQKWIAYTAAVIDAERDRGAAPRTLPAHELATALNLMNERTL
FASFAGEQPSVPEARVLDTLVHIWVTSIYGENR
11111111111111111111111110000000000000000000000000000000000000000000000000000000000
0000000001111100000000000000000000000000000000000000000000000000000000000000000000000
00000000000000000000000000000000000000000000000011
>Q58WW0_3i4i_27_242
NVFTHFGEGFDYYDSQLWEKADGWGNGGVFNCIWRAYNIELKDGILNLSITDDMPSSSKP
YAGAEYRTRDKFGYGLYQVRMKPAKNPGIVSSFFTYTGPVHGTPWDEIDIEFLGKDTTKVQ
FNYYTNSAGNHEYIYDLRFDASEDFHIYAFNWQPNYIAWLVDGEEVYRAYDDIPVHPGKIM
LNIWPGIGVDEWLGAYDGKTNLTASYDWVAYDPI
10000000000000000000000000000000000000000000000000000000000000000000000000000000000
00000000000000000000000000000000000000000000000000000000000000000000000000000000000
00000000000000000000000000000000000000000000000000
>Q73GA4_3f4r_18_233
VVKQDLSDNQYIQKKPNEITSNELLLPLPNDKLLGDPKAPILMIEYASLTCYHCSLFHRNVF
PKIKEKYIDTGKMLYIFRHFPLDYRGLKAAMLSHCYEKQEDYFNFNKAVFNSIDSWNYYNL
SDLTLLQRIAALSNLKQDAFNQCINDKKIMDKIVNDKSLAINKLGITATPIFFIKLNDDKSYIE
HNKVKHGGYKELKYFTNVIDKLYGKAIVK
11111111111111111110000000000000000000000000000000000000000000000000000000000000000
00000000000000000000000000000000000000000000000000000000000000000000000000000000000
00000000000000000000000000000000000000000000000000
>Q81LZ8_3itq_0_215
MTNNNQIGENKEQTIFDHKGNVIKTEDREIQIISKFEEPLIVVLGNVLSDEECDELIELSKSKL
ARSKVGSSRDVNDIRTSSGAFLDDNELTAKIEKRISSIMNVPASHGEGLHILNYEVDQQYKA
HYDYFAEHSRSAANNRISTLVMYLNDVEEGGETFFPKLNLSVHPRKGMAVYFEYFYQDQSL
NELTLHGGAPVTKGEKWIATQWVRRGTYK
11111111110000000000000000000000000000000000000000000000000000000111111110000000000
00000000000000000000000000000000000000000000000000000000000000000000000000000000000
00000000000000000000000000000000000000000000000000
>A4IKE6_4ano_1_215
SEKKTYLETQLDAVMINDQPYTVIFQRAKLKMQDPLELEVLKEVDPCIVRDIDVSEDEVKVV
IKPPSSFLTFAAIRKTTLLSRIRAAIHLVSKVKHHSARRLIFIVCPENLMFNRALEPFFLHVGVK
ESLPPDEWDDERLLREVKATVLALTEGEYRFDEYLKFHETLKCSPIAKELWQADHLDAVLA
VLEKWVDEEEAKERAKVHIPKKRWNMQ
00000000000000000000000000000000000000000000000000000000000000000000000000000000000
00000000000000000000000000000000000000000000000000000000000000000000000000000000000
0000000000000000000000000000000000000000000111111
>P0AB87_1dzv_0_214
MERNKLARQIIDTCLEMTRLGLNQGTAGNVSVRYQDGMLITPTGIPYEKLTESHIVFIDGNG
KHEEGKLPSSEWRFHMAAYQSRPDANAVVHNHAVHCTAVSILNRSIPAIHYMIAAAGGNS
IPCAPYATFGTRELSEHVALALKNRKATLLQHHGLIACEVNLEKALWLAHEVEVLAQLYLT
TLAITDPVPVLSDEEIAVVLEKFKTYGLRIEE
00000000000000000000000000000000000000000000000000000000000000000000000000000000000
00000000000000000000000000000000000000000000000000000000000000000000000000000000000
0000000000000000000000000000000000000000111111111
>P30014_3v9x_0_214
MSDNAQLTGLCDRFRGFYPVVIDVETAGFNAKTDALLEIAAITLKMDEQGWLMPDTTLHF
HVEPFVGANLQPEALAFNGIDPNDPDRGAVSEYEALHEIFKVVRKGIKASGCNRAIMVAH
NANFDHSFMMAAAERASLKRNPFHPFATFDTAALAGLALGQTVLSKACQTAGMDFDSTQ
AHSALYDTERTAVLFCEIVNRWKRLGGWPLSAAEEV
11110000000000000000000000000000000000000000000000000000000000000000000000000000000

00000000000000000000000000000000000000000000000000000000000000000000000000000000000
0000000000000000000000000000000000000000000000000
>P71119_1wnw_0_214
MTTATAGLAVELKQSTAQAHEKAEHSTFMSDLLEGRLGVAEFTRLQEQAWLFYTALEQAA
DAVRASGFAESLLDPALNRAEVLARDLDKLNDGSEWRSRITASPAVIDYVNRLEEIRDNVD
GPALVAHHYVRYLGDLSGGQVIARMMQRHYGVDPEALGFYHFEGIAKLKVYKDEYREKL
NNLELSDEQRENLLKEATDAFVFNHQVFADLGKGL
11111000000000000000000000000000000000000000000000000000000000000000000000000000000
00000000000000000000000000000000000000000000000000000000000000000000000000000000000
0000000000000000000000000000000000000000000000011
>Q0S4E1_2ov9_1_215
SVGTHPTFENSPSGTVLTSPPDGSAVDRATDAARRVVDALLRTDRGNANLERVAEELNSIA
GHLEEHAPAVAERLIDMWNGEGVTRHDPVTGPENALAPPVVLEGLSDGSVRGTVTLTIPY
QGPPGHVHGGVSALLLDHVLGVANAWGGKAGMTAQLSTRYHRPTPLFEPLTLTGKLMSV
DGRKITTAGDIRTADGQVCVSVEGLFVDKTVPRPR
11110000000000000000000000000000000000000000000000000000000000000000000000000000000
00000000000000000000000000000000000000000000000000000000000000000000000000000000000
0000000000000000000000000000000000000000001111111
>Q2G185_4ann_11_225
QDMLTPLDAEEAAKTKLRLDMREIPKSSIKPEHFHLMYLLEQHSPYFIDAELTELRDSFQIHY
DINDNHTPFDNIKSFTKNEKLRYLLNIKNLEEVNRTRYTFVLAPDELFFTRDGLPIAKTRGL
QNVVDPLPVSEAEFLTRYKALVICAFNEKQSFDALVEGNLELHKGTPFETKVIEAATLDLLT
AFLDEQYQKQEQDYSQNYAYVRKVGHTV
11111111111111111100000000000000000000000000000000000000000000000000000000000000000
00000000000000000000000000000000000000000000000000000000000000000000000000000000000
0000000000000000000000000000111111111111111111111
>Q87RY9_2qsx_81_295
IQHATASLIQTNTDQELLVVDVTPSFASLWLVPNINDFHQRHPNIRVKILTGDGAVKNIHGE
SDLHVRCLPLSTHYEYSQLLCEETLLLIGNTNLPKLSDNQAISHYPFIPQTTRPQLWEQFKQE
NDLECPITYHSVGFEHFYLACEAVRMEKGLALLPDFMAQFSILRGDIQHIGNLKLHSGYGY
YVVIPNFRLTSRKVALFHDWLKDKLTHHT
11111111111111100000000000000000000000000000000000111100000000000000000000000000000
00000000000000011100000000000000000000000000011110000000000000000000000000000000000
0000000000000000000000000000000000000000000000111
>Q89YU3_3l39_0_214
MKNSFFSKFTPKEPKFFPLLKQLSDVLSASSVLLVESMEHDLPTERADYYKQIKDMEREGDR
LTHLIFDELSTTFITPFDREDIHDLASCMDDVIDGINSSAKRIVIYNPRPISESGKELSRLIHEEA
INIGKAMDELETFRKNPKPLRDYCTQLHDIENQADDVYELFITKLFEEEKDCIELIKIKEIMH
ELEKTTDAAEHVGKILKNLIVKYS
11111100000000000000000000000000000000000000000000000000000000000000000000000000000
00000000000000000000000000000000000000000000000000000000000000000000000000000000000
0000000000000000000000000000000000000000000000000
>Q8L8B8_2a33_0_214
MEIKGESMQKSKFRRICVFCGSSQGKKSSYQDAAVDLGNELVSRNIDLVYGGGSIGLMGLVS
QAVHDGGRHVIGIIPKTLMPRELTGETVGEVRAVADMHQRKAEMAKHSDAFIALPGGYGT
LEELLEVITWAQLGIHDKPVGLLNVDGYYNSLLSFIDKAVEEGFISPTAREIIVSAPTAKELVK
KLEEYAPCHERVATKLCWEMERIGYSSEE
11111110000000000000000000000000000000000000000000000000000000000000000000000000011
11100000000000000000000000000000000000000000000000000000000000000000000000000000000
0000000000000000000000001111111111111111111111111
>Q928A2_3mzo_0_214

MGIHQYFQSLSDLENIYRCPGKFKYQEHSVAEHSYKVTSIAQFFGAVEEDAGNEVNWRALY
EKALNHDYSELFIGDIKTPVKYATTELREMLSEVEESMTKNFISREIPATFQPIYRHLLKEGKD
STLEGKILAISDKVDLLYESFGEIQKGNPENIFVEIYSEALATIYEYREMASVKYFLKEILPDML
AEKGIEKTELPQLTTEITTKALRDE
00000000000000000000000000000000000000000000000000000000000000000000000000000000000
00000000000000000000000000000000000000000000000000000000000000000000000000000000000
0000000000000000000000000000000000000000000011111
>Q9HJY3_3ndh_1_215
NIRDRMNDHLKDLFRDRLIIDKVQRRLPYMFQLAELESSRAGKVGMEVGSLRERIISSLLIYK
FGEKNVETDLPITEPEIDVKLFGSPISIKTITGKEPAGVKLIWTVDATKARQFLETWHPRFDLI
LVHINWSSLGGVYYIPDYVQQRIFDEIGKDKYIKLPKQGTNPRGVEISNEALKEIMTDEETMS
IKIEWKKTNVQYNAFKRWVDLWSEG
11111110000000000000000000000000000000000000000000000000000000000000000000000000000
00000000000000000000000000000000000000000000000000000000000000000000000000000000000
0000000000000000000000000000000000000000000000000
>O05891_1gtv_0_213
MLIAIEGVDGAGKRTLVEKLSGAFRAAGRSVATLAFPRYGQSVAADIAAEALHGEHGDLAS
SVYAMATLFALDRAGAVHTIQGLCRGYDVVILDRYVASNAAYSAARLHENAAGKAAAWV
QRIEFARLGLPKPDWQVLLAVSAELAGERSRGRAQRDPGRARDNYERDAELQQRTGAVYA
ELAAQGWGGRWLVVGADVDPGRLAATLAPPDVPS
00000000000000000000000000000000000000000000000000000000000000000000000000000000000
00000000000000000000000000000000000000000000000000000000000000000000000000000000000
000000000000000000000000000000000000000000111111
>O85353_4ea9_1_214
SASLAIGGVVIIGGGGHAKVVIESLRACGETVAAIVDADPTRRAVLGVPVVGDDLALPMLR
EQGLSRLFVAIGDNRLRQKLGRKARDHGFSLVNAIHPSAVVSPSVRLGEGVAVMAGVAINA
DSWIGDLAIINTGAVVDHDCRLGAACHLGPASALAGGVSVGERAFLGVGARVIPGVTIGA
DTIVGAGGVVVRDLPDSVLAIGVPAKIKGDRS
10000000000000000000000000000000000000000011100000000000000000000000000000000000000
00000000000000000000000000000000000000000000000000000000000000000000000000000000000
000000000000000000000000000000000000000000000111
>P09543_1woj_185_398
LPLYFGWFLTKKSSETLRKAGQVFLEELGNHKAFKKELRQFVPGDEPREKMDLVTYFGKRP
PGVLHCTTKFCDYGKAPGAEEYAQQDVLKKSYSKAFTLTISALFVTPKTTGARVELSEQQLQ
LWPSDVDKLSPTDNLPRGSRAHITLGCAADVEAVQTGLDLLEILRQEKGGSRGEEVGELSR
GKLYSLGNGRWMLTLAKNMEVRAIFTGYYG
00000000000000000000000000000000000000000001111100000000000000000000000000000000000
00000000000000000000000000000000000000000000000000000000000000000000000000000000000
000000000000000000000000000000000000000000000000
>P15924_1lm5_2608_2821
FSDTLEESSPIAAIFDTENLEKISITEGIERGIVDSITGQRLLEAQACTGGIIHPTTGQKLSLQDA
VSQGVIDQDMATRLKPAQKAFIGFEGVKGKKKMSAAEAVKEKWLPYEAGQRFLEFQYLTG
GLVDPEVHGRISTEEAIRKGFIDGRAAQRLQDTSSYAKILTCPKTKLKISYKDAINRSMVEDIT
GLRLLEAASVSSKGLPSPYNMSSA
11110000000000000000000000000000000000000000000000000000000000000000000000000000000
00000000001110000000000000000000000000000000000000000000000000000000000000000000000
000000000000000000000000000000000000011111111111
>P36057_1nrj_30_243
GIKQKSYQPSIIIAGPQNSGKTSLLTLLTTDSVRPTVVSQEPLSAADYDGSGVTLVDFPGHVK
LRYKLSDYLKTRAKFVKGLIFMVDSTVDPKKLTTTAEFLVDILSITESSCENGIDILIACNKSEL
FTARPPSKIKDALESEIQKVIERRKKSLNEVERKINEEDYAENTLDVLQSTDGFKFANLEASV

VAFEGSINKRKISQWREWIDEKL
11111000000000000000000000000000000000000000000000000000000000000000000000000000000
00000000000000000000000000000000000000000000000000000000000000000000000000011111111
111111000011110000000000000000000000000000000000
>P71554_3da8_1_214
QEPLRVPPSAPARLVVLASGTGSLLRSLLDAAVGDYPARVVAVGVDRECRAAEIAAEASVPV
FTVRLADHPSRDAWDVAITAATAAHEPDLVVSAGFMRILGPQFLSRFYGRTLNTHPALLPAF
PGTHGVADALAYGVKVTGATVHLVDAGTDTGPILAQQPVPVLDGDDEETLHERIKVTERR
LLVAAVAALATHGVTVVGRTATMGRKVTIG
10000000000000000000000000000000000000000000000000000000000000000000000000000000000
00000000000000000000000000000000000000000000000000000000000000000000000000000000000
000000000000000000000000000000000000000000000000
>Q0WP12_3lcc_0_213
MAEEQQNSDQSNGGNVIPTPEEVATFLHKTVEEGGWEKCWEEEITPWDQGRATPLIVHLV
DTSSLPLGRALVPGCGGGHDVVAMASPERFVVGLDISESALAKANETYGSSPKAEYFSFVKE
DVFTWRPTELFDLIFDYVFFCAIEPEMRPAWAKSMYELLKPDGELITLMYPITDHVGGPPYK
VDVSTFEEVLVPIGFKAVSVEENPHAIPTR
11111111111100000000000000000000000000000000000000000000000000000000000000000000000
00000000000000000000000000000000000000000000000000000000000000000000000000000000000
000000000000000000000000000000000000000000000000
>Q47PI5_4h79_0_213
MTTTETPKTISLTWVGTFVDQRVREIQEGYRLDNPRAVATLARLRRGAGKEIGDTPDLWGLI
LDDRFYADAPPLKEKDMEVAENSAHIALTLYAIHQQSRRDDRMHQRGWGLGEAVRRLMP
SSEIDEPLRKRFVQVGHAVTYKALAQRLREIVTLLRRDAIPLDYGLLADQLYQFRTPQGAQR
VRTAWGRGFHAYRPKTTQNPDSTTTTEKDNS
11111111110000000000000000000000000000000000000000111111100000000000000000000000000
00000000000000000000000000000000000000000000000000000000000000000000000000000000000
000000000000000000000000000011111111111111111111
>Q5LIG1_3hn5_21_234
MKDNYDAPESMLTGRVMYNGEALQLRGNEAVQLQLYQHGYAKHDPINVYVNQDGMYS
ANLFDGEYQMITKSGNGPWTSEGRDTINVTVAGNTVQDVEVTPYYLVRDAQMTLEGNKV
NASFKVEKVAGGGIDRVFFMLSTTQFVNDAEHNVDRYDETDNLDAYDETGKLYTFATRDY
TDNSMFQTALKRGTLFGRICIWPKGSDQGIYSKVIRLK
11111111000000000000000000000000000000000000000000000000000000000000000000000000000
00000000000000000000000000000000000000000000000000000000000000000000000000000000000
000000000000000000000000000000000000000000000000
>Q92882_3ehr_0_213
MSKPPPKPVKPGQVKVFRALYTFEPRTPDELYFEEGDIIYITDMSDTNWWKGTSKGRTGLIPS
NYVAEQAESIDNPLHEAAKRGNLSWLRECLDNRVGVNGLDKAGSTALYWACHGGHKDI
VEMLFTQPNIELNQQNKLGDTALHAAAWKGYADIVQLLLAKGARTDLRNIEKKLAFDMA
TNAACASLLKKKQGTDAVRTLSNAEDYLDDEDSD
11111111111000000000000000000000000000000000000000000000000000000000000000000000000
00000000000000000000000000000000000000000000000000000000000000000000000000000000000
000000000000000000000000111111111111111111111111
>Q9BYX4_3b6e_276_489
NSNMGSDSGTMGSDSDEENVAARASPEPELQLRPYQMEVAQPALEGKNIIICLPTGSGKTR
VAVYIAKDHLDKKKKASEPGKVIVLVNKVLLVEQLFRKEFQPFLKKWYRVIGLSGDTQLKIS
FPEVVKSCDIIISTAQILENSLLNLENGEDAGVQLSDFSLIIIDECHHTNKEAVYNNIMRHYL
MQKLKNNRLKKENKPVIPLPQILGLTAS
11111111111111000000000000000000000000000000000000000000000000000000000000000000000
00000000000000000000000000000000011100000000000000000000000000001111111000000000000

000111111100000000000000000000000000000000000000
>Q9WYS0_3sxy_0_213
MKKIEVDLVRTKVYNLLKEMILNHELKLGEKLNVRELSEKLGISFTPVRDALLQLATEGLVK
VVPRVGFFVTDVDEKFIRETIETRIMMEVFCLENYFDKIAGSEELLEIKGEIDDVEKSAKREIF
DDSDERLHKLFIRASGNELIISLYEKIWDRIDLVRHLNERYVVSNREHKELIERIISGDKEGAIE
KLKEHLKNVEAETIKNLYTYERS
11111111100000000000000000000000000000000000000000000000000000000000000000000000000
00000000000000000000000000000000000000000000000000000000000000000000000000000000000
00000000000000000000000000000000000000000000001111
>O33635_3lat_302_514
VSSQKTSSLPKYTPKVNSSINNYIRKKNMKAPRIEEDYTSYFPKYGYRNGVGRPEGIVVHDT
ANDNSTIDGEIAFMKRNYTNAFVHAFVDGNRIIETAPTDYLSWGAGPYGNQRFINVEIVHT
HDYDSFARSMNNYADYAATQLQYYNLKPDSAENDGRGTVWTHAAISNFLGGTDHADPH
QYLRSHNYSYAELYDLIYEKYLIKTKQVAPWG
11111100000000000000000000000000000000000000000000000000000000000000000000000000000
00000000000000000000000000000000000000000000000000000000000000000000000000000000000
00000000000000000000000000000000000000000000000
>O60609_2gh0_150_362
KLNMLKPDSDLCLKFAMLCTLNDKCDRLRKAYGEACSGPHCQRHVCLRQLLTFFEKAAEP
HAQGLLLCPCAPNDRGCGERRRNTIAPNCALPPVAPNCLELRRLCFSDPLCRSRLVDFQTH
CHPMDILGTCATEQSRCLRAYLGLIGTAMTPNFVSNVNTSVALSCTCRGSGNLQEECEMLE
GFFSHNPCLTEAIAAKMRFHSQLFSQDWPHP
11111110000000000000000000000000000000000000000000000000000000000000000000000000000
00000000000000000000000000000000000000000000000000000000000000000000000000000000000
00000000000000000000000000000000000000000111111
>P19878_1hh8_0_212
MSLVEAISLWNEGVLAADKKDWKGALDAFSAVQDPHSRICFNIGCMYTILKNMTEAEKAF
TRSINRDKHLAVAYFQRGMLYYQTEKYDLAIKDLKEALIQLRGNQLIDYKILGLQFKLFACE
VLYNIAFMYAKKEEWKKAEEQLALATSMKSEPRHSKIDKAMECVWKQKLYEPVVIPVGKL
FRPNERQVAQLAKKDYLGKATVVASVVDQDS
10000000000000000000000000000000000000000000000000000000000000000000000000000000000
00000000000000000000000000000000000000000000000000000000000000000000000000000000000
00000000000000000000000000011111111111111111111
>P23940_3bam_0_212
MEVEKEFITDEAKELLSKDKLIQQAYNEVKTSICSPIWPATSKTFTINNTEKNCNGVVPIKEL
CYTLLEDTYNWYREKPLDILKLEKKKGGPIDVYKEFIENSELKRVGMEFETGNISSAHRSMN
KLLLGLKHGEIDLAIILMPIKQLAYYLTDRVTNFEELEPYFELTEGQPFIFIGFNAEAYNSNVP
LIPKGSDGMSKRSIKKWKDKVENK
00000000000000000000000000000000000000000000000000000000000000000000000000000000000
00000000000000000000000000000000000000000000000000000000000000000000000000000000000
00000000000000000000000000000000000000000001111
>P40465_4eq6_0_212
MEYEDLELITIWPSPTKNKLCQFIKQNLSKEHVVTQLFFIDATSSFPLSQFQKLVPPTLPENVR
IYENIRINTCLDLEELSAITVKLLQILSMNKINAQRGTEDAVTEPLKIILYINGLEVMFRNSQFK
SSPQRSHELLRDTLLKLRVMGNDENENASIRTLLEFPKEQLLDYYLKKNNNTRTSSVRSKRR
RIKNGDSLAEYIWKYYADSLFE
00000000000000000000000000000000000000000000000000000000000000000000000000000000000
00000000000000001111111100000000000000000000000000000000000000000000000000000000000
00000000000011111111111110000000000000000000000
>P43316_1hd5_0_212
ADGRSTRYWDCCKPSCGWAKKAPVNQPVFSCNANFQRITDFDAKSGCEPGGVAYSCADQ

TPWAVNDDFALGFAATSIAGSNEAGWCCACYELTFTSGPVAGKKMVVQSTSTGGDLGSNH
FDLNIPGGGVGIFDGCTPQFGGLPGQRYGGISSRNECDRFPDALKPGCYWRFDWFKNADN
PSFSFRQVQCPAELVARTGCRRNDDGNFPAVQIP
00000000000000000000000000000000000000000000000000000000000000000000000000000000
00000000000000000000000000011111000000000000000000000000000000000000000000000000
0000000000000000000000000000000000000000000000111
>P43615_3uc9_0_212
MVLQYPQNKILVLSDHPHNFLKTQFLQDLFHCSSTGISIVKDQTWENRYYKVHFDLYIDSC
KEIPVWVEEFITPECEPLRNVMAGIILITDIRQTKPQELLHQFMIAAHRNTFVVLANVNEEV
EQDEIDELNEIWSNAFTNVIEFVNWKRSKPTVNHNDYGEKLGLDRIQEIIDTHDWLNCEVQ
PATKIREEIPNEMPLEQIIRNLQSARLKY
10000000000000000000000000000000000000000000000000000000000000000000000000000000
00000000000000000000000000000000000000000000000000000000001111000000000000000000
0000000000000000001111111111111111111111111111111
>Q00805_3c9a_112_443
RSIIGGKHGDRDVRILYQVGDSEEDLPVCAPNAVCSKIDLYETPWIERQCRCPCPSSLGVEDG
HTIADKTRHYKMCQPVHKLPVCTHFRDYTWTLTTAAELNVTEQIVHCRCPRNSVTYLTKR
EPIGNGSPGYRYLFACSPLTRLRCQRKQPCKLFTVRKRQEFLDEVNINSLCQCPKGHRCPSH
HTQSGVIAGESFLEDNIQTYSGYCMAND
11111111100000000000000000000000000000000000000000000000000000000000000000000000
00000000000000000000000000000000000000000000000000000000000000000000000000000000
0000000000000000000000000000000000000000000000000
>Q83WB0_1wck_19_444
AFDPNLVGPTLPPIPPFTLPTGPTGPTGPTGPTGDTGTTGPTGPTGPTGPTGPTGPTGPTGPT
GATGLTGPTGPTGPSGLGLPAGLYAFNSGGISLDLGINDPVPFNTVGSQFGTAISQLDADTFV
ISETGFYKITVIANTATASVLGGLTIQVNGVPVPGTGSSLISLGAPIVIQAITQITTTPSLVEVIVT
GLGLSLALGTSASIIIEKVA
11111111111111111111111111111111111111111111111111111111111111111111111111100000
00000000000000000000000000000000000000000000000000000000000000000000000000000000
0000000000000000000000000000000000000000000000000
>Q97WL0_2qzc_0_212
MSIVGNVENLINGVGELWNKYVKHEFILKMRDGSLPLDIFRYYLIQDGKYVEDMLRALLIA
SSKGPIDKVTKILNLVFSSRDKGLETHGKLYSKLDISRDVIVKTGYNLINYAYTRHLYYYANLD
WNKFLVAWTPCMFGYSIVGDYVIDSPNEVYKTWASFYASTEYKKRIEAILYALDEVSITEDLL
NIFINSVRFEIGFWDASLRKDPTVY
11000000000000000000000000000000000000000000000000000000000000000000000000000110
00000000000000000000000000000000000000000000000000000000000000000000000000000000
0000000000000000000000000000000000000000000000000
>Q9HX10_2a35_0_212
MHSTPKRVLLAGATGLTGEHLLDRILSEPTLAKVIAPARKALAEHPRLDNPVGPLAELLPQL
DGSIDTAFCCLGTTIKEAGSEEAFRAVDFDLPLAVGKRALEMGARHYLVVSALGADAKSSIF
YNRVKGELEQALQEQGWPQLTIARPSLLFGPREEFRLAEILAAPIARILPGKYHGIEACDLAR
ALWRLALEEGKGVRFVESDELRKLGK
11100000000000000000000000000000000000000000000000000000000000000000000000000000
00000000000000000000000000000000000000000000000000000000000000000000000000000000
0000111000000000000000000000000000000000000000000
>Q9VKD7_4gel_40_252
RSKREKASRVHEVIIFNELGEICAAVHMRNSSMGSQKPQVSPCCNTHCSLRNVAKIVEQIDR
AVYSIDLAIYTFTSLFLADSIKRALQRGVIIRIISDGEMVYSKGSQISMLAQLGVPVRVPITTNL
MHNKFCIIDGFERVEEIRLLRKLKFMRPCYSIVISGSVNWTALGLGGNWENCIITADDKLTAT
FQAEFQRMWRAFAKTEGSQIQLK

00000000000000000000000000000000011000000000000000000000000000000000000000000000000
00000000000000000000000000000000000000000000000000000000000000000000000000000000000
00000000000000000000000000000000000000001111111
>A3ER16_3mqz_0_211
MSVQTIERLQDYLLPEWVSIFDIADFSGRMLRIRGDIRPALLRLASRLAELLNESPGPRPWYP
HVASHMRRRVNPPPETWLALGPEKRGYKSYAHSGVFIGGRGLSVRFILKDEAIEERKNLGR
WMSRSGPAFEQWKKKVGDLRDFGPVHDDPMADPPKVEWDPRVFGERLGSLKSASLDIGF
RVTFDTSLAGIVKTIRTFDLLYAEAEKGS
11111000000000000000000000000000000000000000000000000000000000000000000000000000000
00000000000000000000000000000000000000000000000000000000000000000000000000000000000
0000000000000000000000000000000000000000000011
>O59543_1v77_0_211
MVGGGGVKFIEMDIRDKEAYELAKEWFDEVVVSIKFNEEVDKEKLREARKEYGKVAILLSN
PKPSLVRDTVQKFKSYLIYVESNDLRVIRYSIEKGVDAIISPWVNRKDPGIDHVLAKLMVKK
NVALGFSLRPLLYSNPYERANLLRFMMKAWKLVEKYKVRRFLTSSAQEKWDVRYPRDLISL
GVVIGMEIPQAKASISMYPEIILKRLKY
11111100000000000000000000000000000000000000000000000000000000000000000000000000000
00000000000000000000000000000000000000000000000000000000000000000000000000000000000
0000000000000000000000000000000000000000001111
>P13449_2qdy_0_211
MDGVHDLAGVQGFGKVPHTVNADIGPTFHAEWEHLPYSLMFAGVAELGAFSVDEVRYVV
ERMEPRHYMMTPYYERYVIGVATLMVEKGILTQDELESLAGGPFPLSRPSESEGRPAPVETTT
FEVGQRVRVRDEYVPGHIRMPAYCRGRVGTISHRTTEKWPFPDAIGHGRNDAGEEPTYHV
KFAAEELFGSDTDGGSVVVDLFEGYLEPAA
00000000000000000000000000000000000000000000000000000000000000000000000000000000000
00000000000000000000000000000000000000000000000000000000000000000000000000000000000
0000000000000000000000000000000000000000000001
>Q02153_2wz1_407_618
HKRPVPAKRYDNVTILFSGIVGFNAFCSKHASGEGAMKIVNLLNDLYTRFDTLTDSRKNPF
VYKVETVGDKYMTVSGLPEPCIHHARSICHLALDMMEIAGQVQVDGESVQITIGIHTGEVV
TGVIGQRMPRYCLFGNTVNLTSRTETTGEKGKINVSEYTYRCLMSPENSDPQFHLEHRGPVS
MKGKKEPMQVWFLSRKNTGTEETKQDDD
11110000000000000000000000000000100000000000000000000000000000000000000000000000000
00000000000000000000000000000000000000000000000000000000000000000000000000000000000
0000000000000000000000000000000000000111111111
>Q2G9F6_2qtq_0_211
MSSDVQKGDNLETPGARDLLLQTASNIMREGDVVDISLSELSLRSGLNSALVKYYFGNKAG
LLKALLDRDMENIVKSVDALLAKDDMSPEAKLRRHISKCIDTYYDYPYLNRLLMRLVRDSD
EAEAKRIADQYLLPLHRAYNRFIGEGVKAGVFRPINPQLFYFTVTGAADRFFSARLVLKHCF
DQDTLTEQLRDSYREHTVDFIMAGILAH
11111111111111000000000000000000000000000000000000000000000000000000000000000000000
00000000000000000000000000000000000000000000000000000000000000000000000000000000000
0000000000000000000000000000000000000000000000
>Q5LCG5_4jdu_84_295
VKRKGVEVMIALDISNSMLAQDVQPSRLEKAKRLISKLVDGMENDKVGMIVFAGDAFTQL
PITSDYISAKMFLESISPSLISKQGTAIGAAINLAARSFTPQEGVGRAIVVITDGENHEGGAVE
AAKEAAKKGIQVNVLGVGLPDGAPIPIEGSNDFRRDREGNVIVTRLNEAMCQEIAKEGNGI
YIRVDNSNSAQKAINQEINKMAKSDVE
11110000000000000000000000000000000000000000000000000000000000000000000000000000010
00000000000000000000000000000000000000000000000000000000000000000000000000000000000
0000000000000000000000000000000000000000111111

>Q82S11_2in3_0_211
MAMEKPVLWYIADPMCSWCWGFAPVIENIRQEYSAFLTVKIMPGGLRPGTNTPLLPEKRA
QILHHWHSVHITTGQPFTFENALPEGFIYDTEPACRGVVSVSLIEPEKVFPFFAAIQRAFYVG
QEDVAQLAILKKLAVDLGIPESRFTPVFQSDEAKQRTLAGFQRVAQWGISGFPALVVESGTD
RYLITTGYRPIEALRQLLDTWLQQHGV
11100000000000000000000000000000000000000000111100000000000000000000000000000000000
00000000000000000000000000000000000000000000000000000000000000000000000000000000000
0000000000000000000000000000000000000000000001
>Q8E565_3keo_0_211
MIMDKSIPKATAKRLSLYYRIFKRFNTDGIEKASSKQIADALGIDSATVRRDFSYFGELGRRGF
GYDVKKLMNFFAEILNDHSTTNVMLVGCGNIGRALLHYRFHDRNKMQISMAFDLDSNDL
VGKTTEDGIPVYGISTINDHLIDSDIETAILTVPSTEAQEVADILVKAGIKGILSFSPVHLTLPKD
IIVQYVDLTSELQTLLYFMNQQR
11111000000000000000000000000000000000000000000000000000000000000000000000000000000
00000000000000000000000000000000000000000000000000000000000000000000000000000000000
0000000000000000000000000000000000000000000000
>Q8VDF2_3f8j_416_627
IVPANHFGPIPGVPVGTMWRFRVQVSESGVHRPHVAGIHGRSNDGAYSLVLAGGYEDDVD
NGNYFTYTGSGGRDLSGNKRTAGQSSDQKLTNNNRALALNCHSPINEKGAEAEDWRQGK
PVRVVRNMKGGKHSKYAPAEGNRYDGIYKVVKYWPERGKSGFLVWRYLLRRDDTEPEPW
TREGKDRTRQLGLTMQYPEGYLEALANKEKSRKR
00000000000000000000000000000000000000000000000000000000000000000000000000000000000
00000000000000000000000000000000000000000000000000000000000000000000000000000000000
0000000000000000000000000000000000000000111111
>Q948P5_3a68_35_246
AKGSTNHRALTGVIFEPFEEVKKELDLVPTVPQASLARQKYVDESESAVNEQINVEYNVSYV
YHAMFAYFDRDNVALRGLAKFFKESSEEEREHAEKLMEYQNKRGGKVKLQSIVMPLSDFD
HADKGDALHAMELALSLEKLTNEKLLNLHSVATKNGDVQLADFVETEYLGEQVEAIKRIS
EYVAQLRRVGKGHGVWHFDQMLLHEGGDAA
11111111111110000000000000000000000000000000000000000000000000000000000000000000000
00000000000000000000000000000000000000000000000000000000000000000000000000000000000
0000000000000000000000000000000000000000011111
>Q96GD3_2p0k_26_237
HFTWDKYLKETCSVPAPVHCFKQSYTPPSNEFKISMKLEAQDPRNTTSTCIATVVGLTGARL
RLRLDGSDNKNDFWRLVDSAEIQPIGNCEKNGGMLQPPLGFRLNASSWPMFLLKTLNGAE
MAPIRIFHKEPPSPSHNFFKMGMKLEAVDRKNPHFICPATIGEVRGSEVLVTFDGWRGAFD
YWCRFDSRDIFPVGWCSLTGDNLQPPGTK
00000000000000000000000000000000000000000000000000000000000000000000000000000000000
00000000000000000000000000000000000000000000000000000000000000000000000000000000000
0000000000000000000000000000000000000000000111
>Q9RT57_2g40_0_211
MTTIPSTAEAKLEMLTTINRAIAGSRPEALPPYPVPAPLSRAEILHQFEDRILDYGAAYTHVS
AAELPGAIAKALGNARRVIVPAGIPAPWLTVGMDVLRDEPPLSHAELDRADAVLTGCAVAI
SETGTIILDHRADQGRRALSLIPDFHICVVREDQIVQTVREGVEAVAASVREGRPLTWLSGGS
ATSDIELVRVEGVHGPRRLQVIVVG
11111111111111111111111111111111111110000000000000000000000000000000000000000000000
00000000000000000000000000000000000000000000000000000000000000000000000000000000000
0000000000000000000001111111111000000000000000
>A6TH02_3rjv_17_227
ATEPGSQYQQQAEAGDRRAQYYLADTWVSSGDYQKAEYWAQKAAAQGDGDALALLAQ
LKIRNPQQADYPQARQLAEKAVEAGSKSGEIVLARVLVNRQAGATDVAHAITLLQDAARD

SESDAAVDAQMLLGLIYASGVHGPEDDVKASEYFKGSSSLSRTGYAEYWAGMMFQQGEKG
FIEPNKQKALHWLNVSCLEGFDTGCEEFDRISKG
11000000000000000000000000000000000000000000000000000000000000000000000000000000000
00000000000000000000000000000000000000000000000000000000000000000000000000000000000
000000000000000000000000000000000000000000001
>A7KAX9_3iug_366_576
ERVFGCDLGEHLLNSGFEVPQVLQSCTAFIERYGIVDGIYRLSGVASNIQRLRHEFDSEHVPD
LTKEPYVQDIHSVGSLCKLYFRELPNPLLTYQLYEKFSDAVSAATDEERLIKIHDVIQQLPPPH
YRTLEFLMRHLSLLADYCSITNMHAKNLAIVWAPNLLRSKQIESACFSGTAAFMEVRIQSVV
VEFILNHVDVLFSGRISMAMQE
00000000000000000000000000000000000000000000000000000000000000000000000000000000000
00000000000000000000000000000000000000000000000000000000000000000000000000000000000
111111111111111000000000000000000000011111111
>D3DFG8_4ij6_0_210
MVKLILVRHAESEWNPVGRYQGLLDPDLSERGKKQAKLLAQELSREHLDVIYSSPLKRTYL
TALEIAEAKNLEVIKEDRIIEIDHGMWSGMLVEEVMEKYPEDFRRWVEEPHKVEFQGGESL
ASVYNRVKGFLEEVRKRHWNQTVVVVSHTVPMRAMYCALLGVDLSKFWSFGCDNASYS
VIHMEERRNVILKLNITCHLGEFYVEAHKAI
00000000000000000000000000000000000000000000000000000000000000000000000000000000000
00000000000000000000000000000000000000000000000000000000000000000000000000000000000
000000000000000000000000000000000000000001111
>O31803_4hfs_39_249
ADAQVAKAASELPNGIGGRVYLNSTGAVFTAKIVLPETVKNNDSVSTPYIYSGFRATSGTEA
DIGLQYSKQYNVWKPLMKVGSKNEETYIEGKDKFTYNKGFRPGSTVQMTIYKNLSGNTRM
TLWGTNNDGYTGRIITEIQGTNIGTISKWKTLATAAVSYESQRDAIKATFSTSFNNITIDNKAV
TPVVDTQDFAKVSVAGNNVTISVNK
11111111000000000000000000000000000000000000000000000000000000000000000000000000000
00000000000000000000000000000000000000000000000000000000000000000000000000000000000
000000000000000000000000000000000000000000000
>O67577_1zjr_0_210
MVMEYLVLEKRLKRLREVLEKRQKDLIVFADNVKNEHNFSAIVRTCDAVGVLYLYYYHAE
GKKAKINEGITQGSHKWVFIEKVDNPVQKLLEFKNRGFQIVATWLSKESVNFREVDYTKPT
VLVVGNELQGVSPEIVEIADKKIVIPMYGMAQSLNVSVATGIILYEAQRQREEKGMYSRPSLS
EEEIQKILKKWAYEDVIKERKRTLSTS
11111000000000000000000000000000000000000000000000000000000000000000000000000000000
00000000000000000000000000000000000000000000000000000000000000000000000000000000000
000000000000000000000000000000000000111111111
>Q2GAW8_2ras_0_210
MASSGTEHDAMRARLVDVAQAIVEERGGAGLTLSELAARAGISQANLSRYFETREDLMEAI
ADYWFHPMVEIMEDVLASDLPPRRKMYEFFARRFVVMRRKWEADPVKLQTYIEVGNDYF
EQVRSYIDLADHYLGEIIGEAMSDGAFSGLEVDETISLVNQMCAPYCALNTMTTFMERLSE
DKLARIVDAVFDGLSAQDRGARSLTGLRAA
11110000000000000000000000000000000000000000000000000000000000000000000000000000000
00000000000000000000000000000000000000000000000000000000000000000000000000000000000
000000000000000000000000000000000000000000111
>Q58989_1l7p_0_210
MEKKKKLILFDFDSTLVNNETIDEIAREAGVEEEVKKITKEAMEGKLNFEQSLRKRVSLLKD
LPIEKVEKAIKRITPTEGAEETIKELKNRGYVVAVVSGGFDIAVNKIKEKLGLDYAFANRLIVK
DGKLTGDVEGEVLKENAKGEILEKIAKIEGINLEDTVAVGDGANDISMFKKAGLKIAFCAK
PILKEKADICIEKRDLREILKYIK
10000000000000000000000000000000000000000000000000000000000000000000000000000000000

00000000000000000000000000000000000000000000000000000000000000000000000000000000000
000000000000000000000000000000000000000000000
>Q5NEC5_2qwu_0_210
MIMSEMITRQQVTSGETIHVRTDPTACIGSHPNCRLFIDSLTIAGEKLDKNIVAIDGGEDVTK
ADSATAAASVIRLSITPGSINPTISITLGVLIKSNVRTKIEEKVSSILQASATDMKIKLGNSNKK
QEYKTDEAWGIMIDLSNLELYPISAKAFSISIEPTELMGVSKDGMRYHIISIDGLTTSQGSLPVC
CAASTDKGVAKIGYIAAA
11000000000000000000000000000000000000000000000000000000000000000000000000000000000
00000000000000000000000000000000000000000000000000000000000000000000000000000000000
000000000000000000000000000000000000000000011
>Q89399_2p6w_0_210
MTTPCITILSGHFPKETIYARKTKELVEEYCSIHGYNFYYEESEPLETEEHALHFRRSWIIQQA
AEKFPSTEWFLWLDSDVYVNPKNKNKPITSFIDLSDPNILYHTFHEAPWGSYPINTGVKFVH
KDALEIEKIVWSLRNEAPWNTFPYEQKTVYEYVFPRIPGRYIVHDPYTLNCIVKAYPEHVKD
ALFVHMCGTSRAERDEHMEMVAT
11100000000000000000000000000000000000000000000000000000000000000000000000000000000
00000000000000000000000000000000000000000000000000000000000000000000000000000000000
000000000000000000000000000000000000000000011
>Q8N6P7_3dlq_17_227
PEDPSDLLQHVKFQSSNFENILTWDSGPEGTPDTVYSIEYKTYGERDWVAKKGCQRITRKSC
NLTVETGNLTELYYARVTAVSAGGRSATKMTDRFSSLQHTTLKPPDVTCISKVRSIQMIVHPT
PTPIRAGDGHRLTLEDIFHDLFYHLELQVNRTYQMHLGGKQREYEFFGLTPDTEFLGTIMIC
VPTWAKESAPYMCRVKTLPDRTWT
11000000000000000000000000000000000000000000000000000000000000000000000000000000000
00000000000000000000000000000000000000000000000011110000000000000000000111100000000
000000000000000000000000000000000000000000000
>Q8XV73_2qgu_0_210
MFKKLLHSLFAGLTFMAAVAAVPAHAQEADAQTTVKTAVDDVLATIKGDSDLRSGNMQK
VFQLVDQKIVPRADFKRTTQIAMGRFWSQATPEQQQIQDGFKTLLVRTYAGALANVRNQ
TVSYKPFRAAADDTDVVVRSTVNNNGEPVALDYRMEKSANGWKVYDINISGLWLSETYK
NQFAEVISKRGGVSGLVQFLDERNAQLAKGPAK
11111111111111111111111111110000000000000000000000000000000000000000000000000000000
00000000000000000000000000000000000000000000000000000000000000000000000000000000000
000000000000000000000000000000000000000001111
>Q9KS12_3fzy_3439_3649
ALADGKILHNQNVNSWGPITVTPTTDGGETRFDGQIIVQMENDPVVAKAAANLAGKHAE
SSVVVQLDSDGNYRVVYGDPSKLDGKLRWQLVGHGRDHSETNNTRLSGYSADELAVKLA
KFQQSFNQAENINNKPDHISIVGCSLVSDDKQKGFGHQFINAMDANGLRVDVSVRSSELAV
DEAGRKHTKDANGDWVQKAENNKVSLSWDAQG
00000000000000000000000000000000000000000000000000000000000000000000000000000000000
00000000000000000000000000000000000000000000000000000000000000000000000000000000000
000000000000000000000000000000000000000000111
>P53674_1oki_42_251
TVPITSAKAAELPPGNYRLVVFELENFQGRRAEFSGECSNLADRGFDRVRSIIVSAGPWVAFE
QSNFRGEMFILEKGEYPRWNTWSSSYRSDRLMSFRPIKMDAQEHKISLFEGANFKGNTIEIQ
GDDAPSLWVYGFSDRVGSVKVSSGTWVGYQYPGYRGYQYLLEPGDFRHWNEWGAFQPQ
MQSLRRLRDKQWHLEGSFPVLATEPPK
11111111111100000000000000000000000000000000000000000000000000000000000000000000000
00000000000000000000000000000000000000000000000000000000000000000000000000000000000
00000000000000000000000000000011111111111111
>P54656_1kzf_0_209

MLELFDVSYEELQTTRSEELYKLRKKTFSDRLGWEVICSQGMESDEFDGPGTRYILGICEGQL
VCSVRFTSLDRPNMITHTFQHCFSDVTLPAYGTESSRFFVDKARARALLGEHYPISQVLFLA
MVNWAQNNAYGNIYTIVSRAMLKILTRSGWQIKVIKEAFLTEKERIYLLTLPAGQDDKQQL
GGDVVSRTGCPPVAVTTWPLTLPV
00000000000000011111111111000000000000000000000000000000000000000000000000000000
00000000000000000000000000000000000000000000000000000000000000000000000000000000
000000000000000000000000000000000000000000
>Q00517_3nje_27_236
RMFDSVMQTDQATRVQEQRMRELVRAMGALERDLTQAVERPVRDELGDNRGAFLSEGEN
DQIVEFTRGGWRNPLGQARSRLQRVRWSLSGETLERRYWLVLDRAQDSKPRVQQVLDGVT
ALSWRFLDKEHNWQGHWPTDEGSEEERLESLPLAVEMTLEHRHYGKLVRVWRLLDPPLK
QDQPQGQPGGENGENGEGGVPQPPEGMPGAPE
11111111111110000000000000000000000000000000000000000000000000000000000000000000
00000000000000000000000000000000000000000000000000000000000000000000000000000000
000000000000011111111111111111111111111111
>Q14457_4ddp_240_449
LELDDELKSVENQMRYAQTQLDKLKKTNVFNATFHIWHSGQFGTINNFRLGRLPSVPVEW
NEINAAWGQTVLLLHALANKMGLKFQRYRLVPYGNHSYLESLTDKSKELPLYCSGGLRFF
WDNKFDHAMVAFLDCVQQFKEEVEKGETRFCLPYRMDVEKGKIEDTGGSGGSYSIKTQFN
SEEQWTKALKFMLTNLKWGLAWVSSQFYNK
11111100000000000000001111000000000000000000000000000000000000000000000000000000
00000000000000000000000000000000000000000000000000000000000000100000000000000000
000000000000000000000000000000000000000111
>Q1IZR6_3e97_0_209
MREALKVVTERNFQPDELVVEQDAEGEALHLVTTGVVRVSRVSLGGRERVLGDIYAPGVV
GETAVLAHQERSASVRALTPVRTLMLHREHFELILRRHPRVLWNLAEMLARRVTFLNDELI
AFGQNTEAALTHVFANLYRQRLAAGVPQPEVLPLGTQDIMARTSSSRETVSRVLKRLEAHN
ILEVSPRSVTLLDLAALEALSFEGAETD
00000000000000000000000000000000001100000000000000000000000000000000000000000000
00000000000000000000000000000000000000000000000000000000000000000000000000000000
000000000000000000000000000000000001111111
>Q2GHD1_3kzx_0_209
MKQPTAVIFDWYNTLIDTSINIDRTTFYQVLDQMGYKNIDLDSIPNSTIPKYLITLLGKRWKE
ATILYENSLEKSQKSDNFMLNDGAIELLDTLKENNITMAIVSNKNGERLRSEIHHKNLTHYF
DSIIGSGDTGTIKPSPEPVLAALTNINIEPSKEVFFIGDSISDIQSAIEAGCLPIKYGSTNIIKDILS
FKNFYDIRNFICQLINI
00000000000000000000000000000000000000000000000000000000000000000000000000000000
00000000000000000000000000000000000000000000000000000000000000000000000000000000
000000000000000001111100000000000000000001
>Q2RJ67_1y80_0_209
MPTYEELSQAVFEGDEAQVVELTRSLLSGGAEPLEVINKGLIAGMDRVGVLFKNNEMFVPE
VLMSANAMNAGVEVVKQSQQAFDMPSVGKIVLGTVKGDLHDIGKNLVAMMLESGGFTV
YNLGVDIEPGKFVEAVKKYQPDIVGMSALLTTTMMNMKSTIDALIAAGLRDRVKVIVGGA
PLSQDFADEIGADGYAPDAASATELCRQLLE
11111111111111111111111111111111111111111111111111111111111111111111111111111111
10000000000000000000000000000000000000000000000000000000000000000000000000000000
000000000000000000000000000000000000000001
>Q98D70_3bhq_0_209
MKIDGETRSARKDREIIQAATAAFISKGYDGTSMEEIATKAGASKQTVYKHFTDKETLFGEV
VLSTASQVNDIIESVTTLLSEAIFMEGGLQQLARRLIAVLMDEELLKLRRLIIANADRMPQLG
RAWYEKGFERMLASTASCFQKLTNRGLIQTGDPYLAASHLFGMLLWIPMNEAMFTGSNRR

SKAELERHADASVEAFLAVYGVQPK
11111000000000000000000000000000000000000000000000000000000000000000000000000000000
00000000000000000000000000000000000000000000000000000000000000000000000000000000000
00000000000000000000000000000000000000000111
>Q992I2_2vrs_116_325
LQTTVDGNSTAISNLKSDISSNGLAITDLQDRVKSLESTASHGLSFSPPLSVADGVVSLDMDP
YFCSQRVSLTSYSAEAQLMQFRWMARGTNGSSDTIDMTVNAHCHGRRTDYMMSSTGNLT
VTSNVVLLTFDLSDITHIPSDLARLVPSAGFQAASFPVDVSFTRDSATHAYQAYGVYSSSRVFT
ITFPTGGDGTANIRSLTVRTGIDT
11111000000000000000000000000000000000000000000000000000000000000000000000000000000
00000000000000000000000000000000000000000000000000000000000000000000000000000000000
00000000000000000000000000000000000000000000
>Q9CZP0_2z84_5_214
PSTLELLKDVHLGLPVPCHDPARLALLSGHYLYYHYGCDGLDDRGWGCGYRTLQTLCSWP
GGQSSGVPGLPALQGALEAMGDKPPGFRGSRNWIGCVEASLCLEHFGGPQGRLCHLPRGV
GLRGEEERLYSHFTTGGGPVMVGGDADAQSKALLGICEGPGSEVYVLILDPHYWGTPKNR
CELQAAGWVGWQKVKSVFDSNSFYNLCFTR
10000000000000000000000000000000000000000000000000000000000000000000000000000000000
00000000000000000000000000000000000000000000000000000000000000000000000000000000000
00000000000000000000000000000000000000000000
>Q9SIY3_3v85_0_209
MEVEVKLRLLTAAAHLRLTTLLTPYHLKTLHQRNTFFDTPKNDLSLRRAVLRLRFLQNAAV
SAASPSPPRCIVSLKAKPTLANGISRVEEDEEEIEYWIGKECVESPAKLSDIGSRVLKRVKEEY
GFNDFLGFVCLGGFENVRNVYEWRGVKLEVDETKYDFGNCYEIECETEEPERVKTMIEEFL
TEEKIEFSNSDMTKFAVFRSGKLP
00000000000000000000000000000000000000000000000000000000001111110000000000000000000
00000000000000000000000000000000000000000000000000000000000000000000000000000000000
00000000000000000000000000000000000000000000
>O14646_4b4c_1118_1326
PRENIKGFSDAEIRRFIKSYKKFGGPLERLDAIARDAELVDKSETDLRRLGELVHNGCIKALK
DSSSGTERTGGRLGKVKGPTFRISGVQVNAKLVISHEEELIPLHKSIPSDPEERKQYTIPCHTK
AAHFDIDWGKEDDSNLLIGIYEYGYGSWEMIKMDPDLSLTHKILPDDPDKKPQAKQLQTR
ADYLIKLLSRDLAKKEALSGAG
11111000000000000000000000000000000000000000000000000000000011111111111111111000
00000000000000000000000000000000000000000000000000000000000000000000000000000000000
0000000000000000000000000000000000000000000
>P0A5I8_3mh9_27_235
SSGSKPSGGPLPDAKPLVEEATAQTKALKSAHMVLTVNGKIPGLSLKTLSGDLTTNPTAATG
NVKLTLGGSDIDADFVVFDGILYATLTPNQWSDFGPAADIYDPAQVLNPDTGLANVLANFA
DAKAEGRDTINGQNTIRISGKVSAQAVNQIAPPFNATQPVPATVWIQETGDHQLAQAQLD
RGSGNSVQMTLSKWGEKVQVTKPPVS
11111100000000000000000000000000000000000000000000000000000000000000000000000000000
00000000000000000000000000000000000000000000000000000000000000000000000000000000000
0000000000000000000000000000000000000000000
>P12529_3kot_82_290
MLDRFATKQTQEKLKIGVVGTFAIGCLFPLLSDFKRSYPHIDLHISTHNNRVDPAAEGLDYTI
RYGGGAWHDTDAQYLCSALMSPLCSPTLASQIQTPADILKFPLLRSYRRDEWALWMQAAG
EAPPSPTHNVMVFDSSVTMLEAAQGGMGVAIAPVRMFTHLLSSERIVQPFLTQIDLGSYWIT
RLQSRPETPAMREFSRWLTGVLHK
11111111110000000000000000000000000000000000000000000000000000000000000000000000000
00000000000000000000000000000000000000000000000000000000000000000000000000000000000

0000000000000000000000000000000000000000000000001
>P39993_1ku1_557_765
DRKTEFIECTNAFNEKPKKGIPMLIEKGFIASDSDKDIAEFLFNNNNRMNKKTIGLLLCHPD
KVSLLNEYIRLFDFSGLRVDEAIRILLTKFRLPGESQQIERIIEAFSSAYCENQDYDPSKISDNAE
DDISTVQPDADSVFILSYSIIMLNTDLHNPQVKEHMSFEDYSGNLKGCCNHKDFPFWYLDR
IYCSIRDKEIVMPEEHHGNE
00000000000000000000000000000000000000000000000000000000000000000000000000000000
00000000000000000000000000000000000000000000000000000000000000000000000000000000
0000000000000000000000000000000000000000001111111
>Q7CJK2_3rf0_310_518
RHQDIRQRTAKSLADHYNIDREQARRVLETTEQLYTQWLAQNTKLVQPQLEALLKWAAM
LHEVGLSINHSGMHRHSAYILQNTNLPGFNQEQQTLLATLVRMHRKAIKLDELPRLNLFKK
KYYLPLIQLLRLSTLLNNQRQSTTTPESLRLITDDSHWTLRFPHGYLTQNSLVQLDFEREQAY
WDDVVGWKLVIEEEEPDEAAKVAPEE
00000000000000000000000000000000000000000000000000000000000000000000000000000000
00000000000000000000000000000000000000000000000000000000000000000000000000000000
0000000000000000000000000000000000000000111111111
>Q81YN8_2wag_36_244
DRYEIKGVDVASYQGDIDWRELEKQNMKFAFIKATEGSAFVDKYFSKNWTNANKTSMRVG
AYHFFSFDSKGETQAEQFIRNVPKYKQALPPVIDVEFYANKKDNPPKREDVTKELSVMIEML
EKHYGKKVILYATQEAYDLYIKDAYPQCDIWIRSVLTKPSLSDERKWTFWQYTNRGKLSGYN
GKEKYIDLNVFYGNEEEFENYGMKD
00000000000000000000000000000000000000000000000000000000000000000000000000000000
00000000000000000000000000000000000000000000000000000000000000000000000000000000
0000000000000000000000000000000000000000000000011
>Q8KFI1_2r01_0_208
MEGSLSRGVEIMKLRELVARSRSIRRFDEHVAVNDATLRDLVELVCYTPSAANRQLLRFLPVT
GADMSDKVFPCLKWAGYLEDWPGPEPGERPAAALVMLCRNEDLPGAACDSGIAAQTIML
GAAEKELGGCIVAAIDRERLMASLGIPDAWTVLLVIALGKPAETVVIDQIKPGDDIRYWRDK
HGIHHVPKRQVDELLVTAEQLRERG
11111111111100000000000000000000000000000000000000000000000000000000000000000000
00000000000000000000000000000000000000000000000000000000000000000000000000000000
0000000000000000000000000000000000000000000000011
>Q9HVP2_1y0k_0_208
MNEADYLRLLTRQAEQANDFLSNARKWDRERWVCQRFLEALNVPYRQEDFAAPGEQPP
DVLFKGAGFEVFFVLDEGRRLNEEWREELTRRRQAVSLRQLIRREERPQRIAAAELQARLAP
TLRKKAHNYSERGIDHGELDLLAFVNLKRAVPDFNTPFPPPTEYLRQGWRSLSMVGPTFAR
VLFAHSGAPEFLRANLGRSILFDAGVGL
00000000000000000000000000000000000000000000000000000000000000000000000011111111
11111111111111111111100000000000000000000000000000000000000000000000000000000000
0000000000000000000000000000000000000000000000000
>Q9HX08_3zqu_0_208
MSGPERITLAMTGASGAQYGLRLLDCLVQEEREVHFLISKAAQLVMATETDVALPAKPQA
MQAFLTEYCGAAAGQIRVFGQNDWMAPPASGSSAPNAMVICPCSTGTLSAVATGACNNLI
ERAADVALKERRPLVLVPREAPFSSIHLENMLKLSNLGAVILPAAPGFYHQPQSVEDLVDFV
VARILNTLGIPQDMLPRWGEQHLVSDE
00000000000000000000000000000000000000000000000000000000000000000000000000000000
00000000000000000000000000000000000000000000000000000000000000000000000000000000
0000000000000000000000000000000000000000000000011
>Q9NZD2_3s0i_0_208
MALLAEHLLKPLPADKQIETGPFLEAVSHLPPFFDCLGSPVFTPIKADISGNITKIKAVYDTNP

AKFRTLQNILEVEKEMYGAEWPKVGATLALMWLKRGLRFIQVFLQSICDGERDENHPNLIR
VNATKAYEMALKKYHGWIVQKIFQAALYAAPYKSDFLKALSKGQNVTEEECLEKIRLFLVN
YTATIDVIYEMYTQMNAELNYKV
11111110000000000000000000000000000000000000000000000000000000000000000000000000000
00000000000000000000000000000000000000000000000000000000000000000000000000000000000
0010000000000000000000000000000000000000000
>Q9XA31_2iai_0_208
MTTAKRDTYTPETLLSVAVQVFIERGYDGTSMEHLSKAAGISKSSIYHHVTGKEELLRRAVSR
ALDELFGILDEEHARVGTAAERLEYVVRRMVEVLMAELPYVTLLLRVRGNTGTERWALERR
REFDHRVAALLKDAAAEGDVRADVEVRLATRLVFGMINSIVEWYRPEGPDGRSDASGASG
VSGAGEREVVDAVARLVFGGLRKAS
00000111100000000000000000000000000000000000000000000000000000000000000000000000000
00000000000000000000000000000000000000000000000000000000000000000000000000000000000
0000011111111111000000000000000000000000011
>A3DJ37_3jwg_257_464
LKNEETEKKLNLNQQRLGTVVAVLKSVNAKKVIDLGCGEGNLLSLLLKDKSFEQITGVDVS
YSVLERAKDRLKIDRLPEMQRKRISLFQSSLVYRDKRFSGYDAATVIEVIEHLDENRLQAFEK
VLFEFTRPQTVIVSTPNKEYNFHYQNLFEGNLRHRDHRFEWTRKEFETWAVKVAEKYGYSV
RFLQIGEIDDEFGSPTQMGVFTL
11111111110000000000000000000000000000000000000000000000000000000000000000000000000
00000000000000000000000000000000000000000000000000000000000000000111110000000000000
000000000000000000000000000000000000000000
>A9CJQ0_3fd3_93_300
RVTLNIATNADSLGTWFLDAVSKFTGGSDYLVNIAVDDQDHTVEWLRGGRVLAAVTAHDK
PVQGCRVTPLGVLRYHATASPDFMARHFADGVTPAALARAPGLTFNQKDRLQASWIRTAL
GEDVSYPTHWLPSTDGFVKASLAGMGWGLNPVQLVAEHLAAGRLVELMPGTPLDIPLYW
QVNRLAAERLAGLTANMVGTARVVLMPVG
00000000000000000000000000000000000000000000000000000000000000000000000000000000000
00000000000000000000000000000000000000000000000000000000000000000000000000000000000
000000000000000000000000000000000000000001
>P0A608_1pzs_32_239
CSSPQHASTVPGTTPSIWTGSPAPSGLSGHDEESPGAQSLTSTLTAPDGTKVATAKFEFANGY
ATVTIATTGVGKLTPGFHGLHIHQVGKCEPNSVAPTGGAPGNFLSAGGHYHVPGHTGTPA
SGDLASLQVRGDGSAMLVTTTDAFTMDDLLSGAKTAIIIHAGADNFANIPPERYVQVNGTP
GPDETTLTTGDAGKRVACGVIGSG
11111111111111111111111111111111111110000000000000000000000000000000000000000000000
00000000000000000000000000000000000000000000000000000000000000000000000000000000000
000000000000000000000000000000000000000000
>P0A7A5_3lbf_0_207
MVSRRVQALLDQLRAQGIQDEQVLNALAAVPREKFVDEAFEQKAWDNIALPIGQGQTISQ
PYMVARMTELLELTPQSRVLEIGTGSGYQTAILAHLVQHVCSVERIKGLQWQARRRLKNLD
LHNVSTRHGDGWQGWQARAPFDAIIVTAAPPEIPTALMTQLDEGGILVLPVGEEHQYLKR
VRRRGGEFIIDTVEAVRFVPLVKGELA
10000000000000000000000000000000000000000000000000000000000000000000000000000000000
00000000000000000000000000000000000000000000000000000000000000000000000000000000000
000000000000000000000000000000000000000000
>P21367_1yac_0_207
MTKPYVRLDKNDAAVLLVDHQAGLLSLVRDIEPDKFKNNVLALGDLAKYFNLPTILTTSFE
TGPNGPLVPELKAQFPDTPYIARPGNINAWDNEDFVKAVKATGKKQLIIAGVVTEVCVAFP
ALSAIEEGFDVFVVTDASGTFNEITRHSAWDRLSQAGAQLMTWFGVACELHRDWRNDIEG
LATLFSNHIPDYRNLMTSYDTLTKQK

10000000000000000000000000000000000000000000000000000000000000000000000000000000000
00000000000000000000000000000000000000000000000000000000000000000000000000000000000
000000000000000000000000000000000000000111
>Q01494_3kv0_0_207
MAAAAVVQIPAGATFLETFKKSFVDVPIDAEKGNAISTAEFLEAAESLTTMFDVLGSIAFSPV
KTDMLGNVEKIRKRMLAAPLESQNIQDLVRNELKTKSHTATEGLLWLVRGLEFTCIALSKNI
GSTEELADSFRGSYRVTLKPHHSFLVKPIFSAAMSACPYRKDFYAKLGDDEQKVQEELREYL
VALDKIVNILKRFLESKEAKW
11111111111110000000000000000000000000000000000000000000000000000000000000000000000
00000000000000010000000000000000000000000000000000000000000000000000000000000000000
000000000000000000000000000000000000000000
>Q0S7R9_2gfn_1_208
PKIVDHDERRRALADAVLALIAREGISAVTTRAVAEESGWSTGVLNHYFGSRHELLLAALRR
AGDIQGDRYRTILDEEGAGPIEKLRNITASILPLDERRLAMTRVFLFFYAEGAAEETARGEIA
AFLARWRGVVRESVVAAQREGTVSTDLDADAVTVALVALTDGLALQAILDPVVMKAISAE
DAAARCVDAAVRRTTEEAGAVGR
11000000000000000000000000000000000000000000000000000000000000000000000000000000000
00000000000000000000000000000000000000000000000000000000000000000000000000000000000
000000000000000000000000000000001111111111
>Q7BQR5_3oa8_0_207
MRFETLLKRAAQVGALVLLPLAAHAQEASAVDPARVDAVVKTSFTKLPEGWESRLQQDET
QRICSVTRNNPSPEQAAAIMKAEEVRIKFPAGPVLGSWKDGAKVAQNGRGGQFSDPPGTV
SGGNCYACHQLDPKEVSYGTLGPSLVGYGRERNFSAEDAKIAFAKVYDAQASLACSSMPRF
GVNGVLTEQQIKDVVAYLFDPESPVNK
11111111111111111111111111110000000000000000000000000000000000000000000000000000000
00000000000000000000000000000000000000000000000000000000000000000000000000000000000
000000000000000000000000000000000000000000
>Q852V1_3a9l_0_207
MAQTDTYPNIEALENAETVGVAYNIEVKRQNPSMIYFSPHAGGIEVGTTELIYRVVELTGGS
LYLFQGLLPSGNSRLHVTSTHFDEPMAVCMLSKHTDAVSFHGYKDDYNKNTLVGGLNTEL
RNLIVSKLNSKGIAAEVATDRFTATDPDNIVNRCASGKGVQLEISSAQRRAFFQNNDWSKA
NRGNVTQEFLDYAEAIKEAEAEYYG
11100000000000000000000000000000000000000000000000000000000000000000000000000000000
00000000000000000000000000000000000000000000000000000000000000000000000000000000000
000000000000000000000000000000000000000000
>Q8U092_4aaj_0_207
MFVKICGIKSLEELEIVEKHADATGVVVNSNSKRRIPLEKAREIIENSAIPVFLVSTMVGFSEW
AMAIERTGAQYIQVHSNALPQTIDTLKKEFGVFVMKAFRVPTISKNPEEDANRLLSEISRYN
ADMVLLDTGAGSGKLHDLRVSSLVARKIPVIVAGGLNAENVEEVIKVVKPYGVDVSSGVEK
YGIKDPKLVEEFVRRAKNVVW
00000000000000000000000000000000000000000000000000000000000000000000000000000000000
00000000000000000000000000000000000000000000000000011111100000000000000000000000000
000000000000000000000000000000000000000011
>Q929T5_3b49_0_207
MTEKKIDFKKEEKKFYAPKRKPERIFVPEMNFLMVDGKGDPDGEEYQKAVQSLYAIAYTIK
MSKMGETRLDGYSDFVVPPLEGFWWSEGKFDLKDRDAWLWTSILRQPDFVTEEVLEWAKE
VARKKKPDVDTSRVKLVRFEEGECVQMMHVGPFSEEVHTVAEMHQFMETEGLRNDTGAI
RKHHEIYLSDPRKANPEKMKTILRLPVS
11110000000000000000000000000000000000000000000000000000000000000000000000000000000
00000000000000000000000000000000000000000000000000000000000000000000000000000000000
000000000000000000000000000000000000000000

>Q9H8Y8_3rle_0_207
MGSSQSVEIPGGGTEGYHVLRVQENSPGHRAGLEPFFDFIVSINGSRLNKDNDTLKDLLKA
NVEKPVKMLIYSSKTLELRETSVTPSNLWGGQGLLGVSIRFCSFDGANENVWHVLEVESNS
PAALAGLRPHSDYIIGADTVMNESEDLFSLIETHEAKPLKLYVYNTDTDNCREVIITPNSAW
GGEGSLGCGIGYGYLHRIPTRPFE
11111100000000000000000000000000000000000000000000000000000000000000000000000000000
00000000000000000000000000000000000000000000000000000000000000000000000000000000000
000000000000000000000000000000000000000000
>Q9HIG7_2fur_0_207
MAVECIKDKVTRYPERASYSDEDLVAMLDRNFTCTVSFIDGGIPYAIPMMLASEGKTIYLHG
SMKSRIYGILKTGQLIAISLLEINGIVLAKEIKNNSINYVSALIFGRPYEIDDTEKKIEVFRLLTE
KLVKGRRDNSIKPSYEDLNGVFVFAVKPETFSMKARTGPPHDTSTDDIWSGVLPIQHTISEA
GENAPEYVKSLYGKRIFI
11111111111111100000000000000000000000000000000000000000000000000000000000000000000
00000000000000000000000000000000000000000000000000000000000000000000000000000000000
000000000000000000000000000000000000000000
>Q9Q3G5_3ts3_422_629
SIYLPLPQADDQYTPYFVYNFQGERVSTTETGVFCLAAIPAATTSSRYNNQITTPSIGYRNAS
GTGTSFLLDAASWWNILDVTQTGVLFGQPRLGVGVMQTMKTLKQHIKDYTEPAIQKYYPG
TTNLDEQLKQRLNLAEGDPVISMGDTNGRRAALFYRTSDEKYILFFSTTEDPGAQYQNLKM
LYFWNWSYSDTKQQFLDHLRTVQF
00000000000000000000000000000000000000000000000000000000000011111000000000000000000
00000000000000000000000000000000000000000000000000000000000000000000000000000000000
000000000000000000000000000000000000000000
>O29883_1z0c_414_620
DYKLFITEGYEVGRVNGLAVIGESAGIVLPIIAEVTPSMSKSEGRVIATGRLQEIAREAVMNVS
AIIKKYTGRDISNMDVHIQFVGTYEGVEGDSASISIATAVISAIEGIPVDQSVAMTGSLSVKGE
VLPVGGVTQKIEAAIQAGLKKVIIPKDNIDDVLLDAEHEGKIEVIPVSRINEVLEHVLEDGK
KKNRLMSKFKELELAAV
10000000000000000000000000000000000000111000000000000000000000000000000000000000000
00000000000000000000000000000000000000000000000000000000000000000000000000000000000
00000000000000000000000000000000000000111
>P0A6G7_3mt6_0_206
MSYSGERDNFAPHMALVPMVIEQTSRGERSFDIYSRLLKERVIFLTGQVEDHMANLIVAQM
LFLEAENPEKDIYLYINSPGGVITAGMSIYDTMQFIKPDVSTICMGQAASMGAFLLTAGAKG
KRFCLPNSRVMIHQPLGGYQGQATDIEIHAREILKVKGRMNELMALHTGQSLEQIERDTER
DRFLSAPEAVEYGLVDSILTHRN
11111111111111100000000000110000000000000000000000000000000000000000000000000000000
00000000000000000000000000000000000000000000000000000000000000000000000000000000000
00000000000000000000000000000000000000011
>P0A8I5_3dxz_32_238
GQEHALENYWPVMGVEFSEDMLDFPALFGREAPVTLEIGFGMGASLVAMAKDRPEQDFL
GIEVHSPGVGACLASAHEEGLSNLRVMCHDAVEVLHKMIPDNSLRMVQLFFPDPWHKAR
HNKRRIVQVPFAELVKSKLQLGGVFHMATDWEPYAEHMLEVMSSIDGYKNLSESNDYVPR
PASRPVTKFEQRGHRLGHGVWDLMFERVK
11100000000000000000000000000000000000000000000000000000000000000000000000000000000
00000000000000000000000000000000000000000000000000000000000000000000000000000000000
00000000000000000000001111110000000000000
>P13448_2qdy_0_206
MSVTIDHTTENAAPAQAPVSDRAWALFRALDGKGLVPDGYVEGWKKTFEEDFSPRRGAEL
VARAWTDPEFRQLLLTDGTAAVAQYGYLGPQGEYIVAVEDTPTLKNVIVCSLCSCTAWPILG

LPPTWYKSFEYRARVVREPRKVLSEMGTEIASDIEIRVYDTTAETRYMVLPQRPAGTEGWSQ
EQLQEIVTKDCLIGVAIPQVPTV
11111111100000000000000000000000000000000000000000000000000000000000000000000000000
00000000000000000000000000000000000000000000000000000000000000000000000000000000000
00000000000000000000000000000000000000001
>Q15047_3dlm_195_401
GDLIVSMRILGKKRTKTWHKGTLIAIQTVGPGKKYKVKFDNKGKSLLSGNHIAYDYHPPAD
KLYVGSRVVAKYKDGNQVWLYAGIVAETPNVKNKLRFLIFFDDGYASYVTQSELYPICRPLK
KTWEDIEDISCRDFIEEYVTAYPNRPMVLLKSGQLIKTEWEGTWWKSRVEEVDGSLVRILFL
DDKRCEWIYRGSTRLEPMFSMK
00000000000000000000000000000000000000000000000000000000000000000000000000000000000
00000000000000000000000000000000000000000000000000000000000000000000000000000000000
00000000000000000000000000000000000011111
>Q1GJR2_3c8u_0_206
MTLAALCQGVLERLDPRQPGRQLVALSGAPGSGKSTLSNPLAAALSAQGLPAEVVPMDGF
HLDNRLLEPRGLLPRKGAPETFDFEGFQRLCHALKHQERVIYPLFDRARDIAIAGAAEVGP
ECRVAIIEGNYLLFDAPGWRDLTAIWDVSIRLEVPMADLEARLVQRWLDHGLNHDAAVAR
AQGNDLANARAIEAARLPADLTWPQA
00000000000000000000000000000000000000000000000000000000000000000000000000000000000
00000000000000000000000000000000000000000000000000000000000000000000000000000000000
00000000000000000000000000000000000000001
>Q2RWL2_3cwr_0_206
MVEQRNRGRPAVPDAVVRESIVGAAQRLLSSGGAAAMTMEGVASEAGIAKKTLYRFASGR
ADLIGLLVESWIAPIFPGFEADPQDAAAALERIVYDIAQAVLSREAVSLFRMLASDADLRNR
FLPAYNANGIERSRRELARWLDQQASAGRLPLPIPAERVADLLLSAVIAEPLRQITLGLREPLP
AWDIAPRVADAVRLIAPGRER
11111111100000000000000000000000000000000000000000000000000000000000000000000000000
00000000000000000000000000000000000000000000000000000000000000000000000000000000000
00000000000000000000000000000000000001111
>A3DDK4_2vpt_31_236
ASKTIKIMPVGDSCTEGMGGGEMGSYRTELYRLLTQAGLSIDFVGSQRSGPSSLPDKDHEGH
SGWTIPQIASNINNWLNTHNPDVVFLWIGGNDLLLNGNLNATGLSNLIDQIFTVKPNVTLF
VADYYPWPEAIKQYNAVIPGIVQQKANAGKKVYFVKLSEIQFDRNTDISWDGLHLSEIGYK
KIANIWYKYTIDILRALAGETQ
11000000000000000000000000000000000000000000000000000000000000000000000000000000000
00000000000000000000000000000000000000000000000000000000000000000000000000000000000
0000000000000000000000000000000000000111
>A8R7E6_4eby_24_229
CRTSCPLALASYYLENGTTLSVINQNLNSSIAPYDQINFDPILRYNSNIKDKDRIQMGSRVLV
PFPCECQPGDFLGHNFSYSVRQEDTYERVAISNYANLTTMESLQARNPFPATNIPLSATLNVL
VNCSCGDESVSKDFGLFVTYPLRPEDSLSSIARSSGVSADILQRYNPGVNFNSGNGIVYVPGR
DPNGAFPPFKSSKQDGV
00000000000000000000000000000000000000000000000000000000000000000000000000000000000
00000000000000000000000000000000000000000000000000000000000000000000000000000000000
0000000000000000000000000000000000111111
>D0LJL3_4i66_30_235
SAFRFGQLALGDRWDIYPQALSRMSREIDKRTSIEAAREPAAVTLSSPTLHETPFLYLAGDRE
FAIPPEPEVEALRRHLTFGGFLLIDSAEGALGGAFDRSVRRLLQAVFPAPAPGLEIVSGEHVV
FKSFYLLERPLGRLALSPVMEGILRDGRLMVAYVQNDLGGAFARDDFGNFQLACVPDGER
QRELAFRMLVNLVMYALCLD
10000000000111000000000000000000000000000000000000000000000000000000000000000000000

00000000000000000000000000000000000000000000000000000000000000000000000000000000000
0000000000000000000000000000000000000011
>O53573_1ym3_1_206
PNTNPVAAWKALKEGNERFVAGRPQHPSQSVDHRAGLAAGQKPTAVIFGCADSRVAAEII
FDQGLGDMFVVRTAGHVIDSAVLGSIEYAVTVLNVPLIVVLGHDSCGAVNAALAAINDGTL
PGGYVRDVVERVAPSVLLGRRDGLSRVDEFEQRHVHETVAILMARSSAISERIAGGSLAIVG
VTYQLDDGRAVLRDHIGNIGEEV
11000000000000000000000000000011111111110000000000000000000000000000000000000000000
00000000000000000000000000000000000000000000000000000000000000000000000000000000000
0000000000000000000000000000000000000001
>O57770_1vaj_0_205
MVFKIKDEWGEFLVRLARRAIEEYLKTGKEIEPPKDTPPELWEKMGVFVTLNRYNVPPQTA
LRGCIGFPTPIYPLVEATIKAAIYSAVDDPRFPPVKLEEMDNLVVEVSVLTPPELIEGPPEERPR
KIKVGRDGLIVEKGIYSGLLLPQVPVEWGWDEEEFLAETCWKAGLPPDCWLDEDTKVYKF
TAEIFEEEYPRGPIKRKPLV
11000000000000000000000000000000000000000000000000000000000000000000000000000000000
00000000000000000000000000000000000000000000000000000000000000000000000000000000000
0000000000000000000000000000000000000001
>O95630_3rzv_218_423
SDCHTTVRPAKPPVVDRSLKPGALSNSESIPTIDGLRHVVVPGRLCPQFLQLASANTARGVE
TCGILCGKLMRNEFTITHVLIPKQSAGSDYCNTENEEELFLIQDQQGLITLGWIHTHPTQTA
FLSSVDLHTHCSYQMMLPESVAIVCSPKFQETGFFKLTDHGLEEISSCRQKGFHPHSKDPPLF
CSCSHVTVVDRAVTITDLR
11111111111111111111111111100000000000000000000000000000000000000000000000000000000
00000000000000000000000000000000000000000000000000000000000000000000000000000000000
0000000000000000000000000000000000000000
>P03696_1je5_0_205
MAKKIFTSALGTAEPYAYIAKPDYGNEERGFGNPRGVYKVDLTIPNKDPRCQRMVDEIVKC
HEEAYAAAVEEYEANPPAVARGKKPLKPYEGDMPFFDNGDGTTTFKFKCYASFQDKKTKET
KHINLVVVDSKGKKMEDVPIIGGGSKLKVKYSLVPYKWNTAVGASVKLQLESVMLVELATF
GGGEDDWADEVEENGYVASGSAK
00000000000000000000000000111000000000000000000000000000000000000000000000000011111
11000000000000000000000000000000000000000000000000000000000000000000000000000000000
0000000000000000000000000000000000001111
>P10928_2fkk_396_601
DEIIDETDNLYVSQGPGVDISGDVNLTDFDKIGWPNVEAVQSYQRAFNAVSNIFDTIYPIGTI
YENAVNPNNPVTYMGFGSWKLFGQGKVLVGWNEDISDPNFALNNNDLDSGGNPSHTAG
GTGGSTSVTLENANLPATETDEEVLIVDENGSVIVGGCQYDPDESGPIYTKYREAKASTNST
HTPPTSITNIQPYITVYRWIRIA
11111111100000000000000000000000000000000000000000000000000000000000000000000000000
00000000000000000000000000000000000000000000000000000000000000000000000001111111111
1110000000000000000000000000000000000000
>P30043_1he2_0_205
MAVKKIAIFGATGQTGLTTLAQAVQAGYEVTVLVRDSSRLPSEGPRPAHVVVGDVLQAAD
VDKTVAGQDAVIVLLGTRNDLSPTTVMSEGARNIVAAMKAHGVDKVVACTSAFLLWDPT
KVPPRLQAVTDDHIRMHKVLRESGLKYVAVMPPHIGDQPLTGAYTVTLDGRGPSRVISKHD
LGHFMLRCLTTDEYDGHSTYPSHQYQ
00000000000000000000000000000000000000000000000000000000000000000000000000000000000
00000000000000000000000000000000000000000000000000000000000000000000000000000000000
0000000000000000000000000000000000000001
>P48357_3v6o_427_632

IDVNINISCETDGYLTKMTCRWSTSTIQSLAESTLQLRYHRSSLYCSDIPSIHPISEPKDCYLQS
DGFYECIFQPIFLLSGYTMWIRINHSLGSLDSPPTCVLPDSVVKPLPPSSVKAEITINIGLLKIS
WEKPVFPENNLQFQIRYGLSGKEVQWKMYEVYDAKSKSVSLPVPDLCAVYAVQVRCKRLD
GLGYWSNWSNPAYTVV
11000000000000000000000001111110000000000000000000000000000000000000000000000000000
00000000000000000000000000000000000000000000000001111110000000000000000000000000111
0000000000000000000000000000000000000000
>P62381_1ve4_0_205
MRRFALTVALPKGRMFREAYEVLKRAGLDLPEVEGERTLLHGKEGGVALLELRNKDVPIYV
DLGIAEIGVVGKDVLLDSGRDLFEPVDLGFGACRLSLIRRPGDTGPIRRVATKYPNFTARLLK
ERGWAADVVELSGNIELAAVTGLADAVVDVVQTGATLRAAGLVEVEVLAHSTARLVVNR
QALKLKRAVLKPLIQRLRELSGS
00000000000000000000000000000000011111000000000000000000000000000000000000000000000
00000000000000000000000000000000000000000000000000000000000000000000000000000000000
0000000000000000000000000000000000000000
>Q6NS38_3h8r_55_260
SWRHIRAEGLDCSYTVLFGKAEADEIFQELEKEVEYFTGALARVQVFGKWHSVPRKQATYG
DAGLTYTFSGLTLSPKPWIPVLERIRDHVSGVTGQTFNFVLINRYKDGCDHIGEHRDDEREL
APGSPIASVSFGACRDFVFRHKDSRGKSPSRRVAVVRLPLAHGSLLMMNHPTNTHWYHSLP
VRKKVLAPRVNLTFRKILLTKK
00000000000000000000000000000000000000000000000000000000000000000000000000000000000
00000000000000000000000000000000000000000000000000000000000000000000000000000000000
0000000000000000000000000000000000000111
>Q7NX87_3pzj_0_205
MHSPQISPPIAPSTPLLAGWRSAGKAPEAAIRGEAVSLQPLDAPRHGAALFRLFAGDDSHW
EHLPYGPFEDEDAFITWLALTVAQSDTALYVVCAKDSDQALGFLGYRQMVQAHGAIEIGH
VNFSPALRRTRLATEAVFLLLKTAFELGYRRCEWRCDSRNAASAAAARRFGFQFEGTLRQA
MVVKRRNRDTHVFSMLDGEWDALK
11111100000000000000000000000000000000000000000000000000000000000000000000000000000
00000000000000000000000000000000000000000000000000000000000000000000000000000000000
0000000000000000000000000000000000000011
>Q8U192_1xi3_1_206
NLRNKLKLYVITDRRLKPEVESVREALEGGATAIQMRIKNAPTREMYEIGKTLRQLTREYDA
LFFVDDRVDVALAVDADGVQLGPEDMPIEVAKEIAPNLIIGASVYSLEEALEAEKKGADYL
GAGSVFPTKTKEDARVIGLEGLRKIVESVKIPVVAIGGINKDNAREVLKTGVDGIAVISAVM
GAEDVRKATEELRKIVEEVLG
00000000000000000000000000000000000000000000000000000000000000000000000000000000000
00000000000000000000000000000000000000000000000011110000000000000000000000000000000
0000000000000000000000000000000000000000
>Q9BRJ7_3kvh_5_210
VPELKQISRVEAMRLGPGWSHSCHAMLYAANPGQLFGRIPMRFSVLMQMRFDGLLGFPGG
FVDRRFWSLEDGLNRVLGLGLGCLRLTEADYLSSHLTEGPHRVVAHLYARQLTLEQLHAVEI
SAVHSRDHGLEVLGLVRVPLYTQKDRVGGFPNFLSNAFVSTAKCQLLFALKVLNMMPEEKL
VEALAAATEKQKKALEKLLPASS
11100000000000000000000000000000000000000000000000000000000000000000000000000000011
10000000000000111100000000000000000000000000000000000000000000000000000000000000000
0000000000000000000000000000000000001111
>Q9T1C3_3c6a_358_563
GTLIRATTLSRLSFIDVVNDNGFYQFEKPKEGRKYVATLDCSEGRGQDYHALQIIDITEFPYK
QVAVYHSNTTSHFILPDIVFKYLMMYNECPVYIELNSTGVSIAKSLAMDLEYDNIICDSFIDL
GMKQSKRSKAMGCSALKDLIEKDKLIINHKGTIQELRTFSEKGVSWAAEEGFHDDLVMSLV

IFGWLTTQEKFAEYAGKDE
00000000000000000000000000000000000000000000000000000000000000000000000000000000000
00000000000000000000000000000000000000000000000000000000000000000000000000000000000
000111111110000000000000000000000000000
>O06143_1s8n_0_204
MTGPTTDADAAVPRRVLIAEDEALIRMDLAEMLREEGYEIVGEAGDGQEAVELAELHKPD
LVIMDVKMPRRDGIDAASEIASKRIAPIVVLTAFSQRDLVERARDAGAMAYLVKPFSISDLIP
AIELAVSRFREITALEGEVATLSERLETRKLVERAKGLLQTKHGMTEPDAFKWIQRAAMDRR
TTMKRVAEVVLETLGTPKDT
11111111110000000000000000000000000000000000000000000000000000000000000000000000000
00000000000000000000000000000000000000000000000000000000000000000000000000000000000
000000000000000000000000000000000011111
>O07819_2xts_24_228
DKLGLGREALPEEISAWDTAVLPDGQGLRPGSGDVATGDALFADNCASCHGDFAEGLDSW
PVLAGGDGSLTDPRPVKTIGSYWPYLSTVYDYVHRSMPFGSAQTLSVDDTYAITAFLLYSNG
LVEDDFVLTHENFTQVVLPNAEGFYPDDRDQTEYPLFSKEPCMTDCAVGVEITKRAVDLNV
TPEDPDGRPAGSMPDLGAAAAP
10000000000000000000000000000000000000000000000000000000000000000000000000000000000
00000000000000000000000000000000000000000000000000000000000000000000000000000000000
000000000000000000000000000000000000000
>O31560_1rkt_0_204
MSPKVTKEHKDKRQAEILEAAKTVFKRKGFELTTMKDVVEESGFSRGGVYLYFSSTEEMFRR
IIETGLDEGLRKLDKSAEHQSVWASISSYLDELTEGLRDVADTLAPVQFEYLVTAWRNEERR
QYLEKRYDLFVERFSRLLQKGIDQGEFQPVQPLATIAKFFLNMNDGIIQNALYFDEEKADVS
GLAESAKLYLKTVLQADEK
10000000000000000000000000000000000000000000000000000000000000000000000000000000000
00000000000000000000000000000000000000000000000000000000000000000000000000000000000
000000000000000000000000000000000000000
>P04278_1f5f_29_233
LRPVLPTQSAHDPPAVHLSNGPGQEPIAVMTFDLTKITKTSSSFEVRTWDPEGVIFYGDTNPK
DDWFMLGLRDGRPEIQLHNHWAQLTVGAGPRLDDGRWHQVEVKMEGDSVLLEVDGEE
VLRLRQVSGPLTSKRHPIMRIALGGLLFPASNLRLPLVPALDGCLRRDSWLDKQAEISASAPT
SLRSCDVESNPGIFLPPGTQAE
11111111111110000000000000000000000000000000000000000000000000000000000000000000000
00000000000000000000000000000000000000000000000011110000000000000000000000000000000
000000000000000000000011111111111111111
>P18708_1qcs_0_204
MAGRSMQAARCPTDELSLSNCAVVSEKDYQSGQHVIVRTSPNHKYIFTLRTHPSVVPGSVA
FSLPQRKWAGLSIGQEIEVALYSFDKAKQCIGTMTIEIDFLQKKNIDSNPYDTDKMAAEFIQQ
FNNQAFSVGQQLVFSFNDKLFGLLVKDIEAMDPSILKGEPASGKRQKIEVGLVVGNSQVAFE
KAENSSLNLIGKAKTKENR
00000000000000000000000000000000000000000000000000000000000000000000000000000000000
00000000000000000000000000000000000000000000000000000000000000000000000000000011111
110000000000000000000000000000000001111
>P31697_3bwu_36_240
GVALGATRVIYPAGQKQEQLAVTNNDENSTYLIQSWVENADGVKDGRFIVTPPLFAMKGK
KENTLRILDATNNQLPQDRESLFWMNVKAIPSMDKSKLTENTLQLAIISRIKLYYRPAKLAL
PPDQAAEKLRFRRSANSLTLINPTPYYLTVTELNAGTRVLENALVPPMGESTVKLPSDAGSN
ITYRTINDYGALTPKMTGVME
00000000000000000000000000000000000000000000000000000000000000000000000000000000000
00000000000111110000000000000000000000000000000000000000000000000000000000000000000

00000000000000000000000000000000000000
>P58495_3l4e_0_204
MKNLFLTSSFKDVVPLFTEFESNLQGKTVTFIPTASTVEEVTFYVEAGKKALESLGLLVEELDI
ATESLGEITTKLRKNDFIYVTGGNTFFLLQELKRTGADKLILEEIAAGKLYIGESAGAVITSPNI
AYIQTMDSTKKAVNLTNYDALNLVDFSTLPHYNNTPFKEITQKIVTEYAGKSQIYPISNHEAI
FIRGKEVITKRLS
00000000000000000000000000000000000000000000000000000000000000000000000000000000
00000000000000000000000000000000000000000000000000000000000000000000000000000000
00000000000111100000000000000000000000
>P63250_1n9p_40_370
KKRQRFVDKNGRCNVQHGNLGSERAETLMFSEHAVISMRDGKLTLMFRVGNLRNSHMVS
AQIRCKLLKSRQTPEGEFLPLDQLELDVGFSTGADQLFLVSPLTICHVIDAKSPFYDLSQRSM
QTEQFEVVVILEGIVETTGMTCQARTSYTEDEVLWGHRFFPVISLEEGFFKVDYSQFHATFEV
PTPPYSVKEQEEMLLMSSPL
11000000000000000111110000000000000000000000000000000000000000000000000000000000
00000000000000000000000000000000000000000000000000000000000000000000000000000000
00000000000000000000000000000000000001
>Q2CBJ1_2rg4_1_205
AQIKSLFATRLYHAPLSEHGPALDPAEFAASCYSIAEDDDAGQEWCEREGYPGYTSYASLTD
LPWRFPIFADLVKSLDAHVAAFAEDLEFELDGKALRLEDIWINILPEGGVHGSHIHPHSVISG
TTYVAMPEGTSALKLEDPRLPFMMAAPTRRKGAREELRTFRSVAPKVGDVLLWESWLRHE
VPMNMAEEDRISVSFNYAWG
00000000000000000000000000000000000000000000000000000000000000000000000000000000
00000000000000000000000000000000000000000000000000000000000000000000000000000000
00000000000000000000000000000000000001
>Q56230_3kbg_30_234
MHPKDQSVTLLSIIRDYLKLSDKEREAARILANGLVKVDGKTVREKKFAVGFMDVIEINGES
YRVVYNDQGALVLMKETKERASMKLLKVRSKVIAPGNRIQLGTHDGRTFITDDKSKKVGD
VWAVSVPDMKISEIIKMQPGNKAYITAGSHVNQTGTISKIEAKEGSSANLVHFQEGFSTIKDH
VFMIGSSKFSFVLSPEEVIP
11100000000000000000000000000000000000000000000000000000000000000000000000000000
00000000000000000000000000000000000000000000000000000000000000000000000000000000
00110000000000000000000000000011111111
>Q6VAY1_3m4f_0_204
MKFTLTIAGLLAVGSTAAPTTEKRNPGGIDYVQNYNGDVADFQYNEGAGTYTCGWDGST
DFVVGLGWSTGAARDITYSATYNAGGSGSYLAVYGWVNSPQAEYYIVESYGDYNPCSNAE
GLGTLESDGSTYTVCTDTRTNEPSITGTSTFTQYWSVRQSERTSGTVTVGNHFNYWAQHGF
GDSYNFQVMAVEAFSGSGSASVSVS
11111111111111111111111000000000000000000000000000000000000000000000000000000000
00000000000000000000000000000000000000000000000000000000000000000000000000000000
00000000000000000000000000000000000000
>Q8U2E3_1sgw_1_205
KLEIRDLSVGYDKPVLERITMTIEKGNVVNFHGPNGIGKTTLLKTISTYLKPLKGEIIYNGVPI
TKVKGKIFFLPEEIIVPRKISVEDYLKAVASLYGVKVNKNEIMDALESVEVLDLKKKLGELSQ
GTIRRVQLASTLLVNAEIYVLDDPVVAIDEDSKHKVLKSILEILKEKGIVIISSREELSYCDVNE
NLHKYSTKIDKKD
00000000000000000000000000000000000000000000000000000000000000000000000000000000
00000000000000000000000000000000000000000000000000000000000000000000000000000000
00000000000000000000000000000000111111
>Q9M1X2_4doo_73_277
ADESVVEPKTGFSFPASIGDSRRLLGVGLRKKSLLGLKNIDVYAFGVYADCDDVKKLVGDKY

ANLPASEIRGNKSFMDDLMEADIKMTIRLQIVYGKLNIRSVRNAFQESVGNRLKKFGGSDN
DELLQSFTSLFKDEYKIPRNSTIDLTKDPGHVLSVAIEGNHVGSVKSHLLCRSILDLYIGEEPF
DKNAREDFLDNAASLAFD
11000000000000000000000000000000000000000000000000000000000000000000000000000000000
00000000000000000000000000000000000000000000000000000000000000000000000000000000000
000000000000000000000000000000000000000
>Q9X006_1xkr_0_204
MKISERQKDLLKEIGNIGAGNAATAISYMINKKVEISVPNVEIVPISKVIFIAKDPEEIVVGVK
MPVTGDIEGSVLLIMGTTVVKKILEILTGRAPDNLLNLDEFSASALREIGNIMCGTYVSALAD
FLGFKIDTLPPQLVIDMISAIFAEASIEELEDNSEDQIVFVETLLKVEEEEEPLTSYMMMIPKPG
YLVKIFERMGIQE
00000000000000000000000000000000000000000000000000000000000000000000000000000000000
00000000000000000000000000000000000000000000000000000000000000000000000000000000000
000000000000000000000000000000000000001
>A1E8J1_3fan_1779_1982
GAFRTQKPSLNTVNVVGSSMGSGGVFTIDGKIKCVTAAHVLTGNSARVSGVGFNQMLDFD
VKGDFAIADCPNWQGVAPKAQFCEDGWTGRAYWLTSSGVEPGVIGNGFAFCFTACGDSGS
PVITEAGELVGVHTGSNKQGGGIVTRPSGQFCNVKPIKLSELSEFFAGPKVPLGDVKIGSHII
KDTCEVPSDLCALLAAKPELE
11000000000000000000000000000000000000000000000000000000000000000000000000000000000
00000000000000000000000000000000000000000111110000000000000000000000000000000000000
00000000000000000000000000000000011111
>P43320_1ytq_1_204
ASDHQTQAGKPQSLNPKIIIFEQENFQGHSHELNGPCPNLKETGVEKAGSVLVQAGPWVG
YEQANCKGEQFVFEKGEYPRWDSWTSSRRTDSLSSLRPIKVDSQEHKIILYENPNFTGKKME
IIDDDVPSFHAHGYQEKVSSVRVQSGTWVGYQYPGYRGLQYLLEKGDYKDSSDFGAPHPQ
VQSVRRIRDMQWHQRGAFHPSN
11111111111110000000000000000000000000000000000000000000000000000000000000000000000
00000000000000000000000000000000000000000000000000000000000000000000000000000000000
00000000000000000000000000001111111111
>Q0S7V2_2ibd_0_203
MTPPPADDTSGKSGRRTELLDIAATLFAERGLRATTVRDIADAAGILSGSLYHHFDSKESMV
DEILRGFLDDLFGKYREIVASGLDSRATLEALVTTSYEAIDASHSAVAIYQDEVKHLVANERF
TYLSELNTEFRELWMGVLEAGVKDGSFRSDIDVELAFRFLRDTAWVAVRWYRPGGSVTVDT
VAKQYLSIVLDGLASPHN
11111111110000000000000000000000000000000000000000000000000000000000000000000000000
00000000000000000000000000000000000000000000000000000000000000000000000000000000000
00000000000000000000000000000000000011
>Q45723_2rci_34_237
TVPSSDITNFNEIFYVEPQYIAQAIRLTNTFQGAIDPLTLNFNFEKALQIANGLPNAGVTGTI
NQSVIHQTIEVSVMISQIKEIIRSVLGLVINSANFWNSVVSAITNTFTNLEPQVDENWIVWRN
LSATQTSYFYKILFSIQNEDTGRFMAILPIAFEITVDVQKQQLLFITIKDSARYEVKMKALTVV
QALDSYNAPIIDVF
11111110000000000000000000000000000000000000000000000000000000000000000000000000000
00000000000000000000000000000000000000000000000000000000000000000000000000000000000
00000000000000000000000000000111111111
>Q53766_1jm1_46_249
ADNTDGLAGFPRYKVANIQQVQQQIKSSGCAVYFFAYPLTDEPCFLVDLQALTGQQITEIPN
PYYGKYAGPLGQIQTIKGVGPNGTIFAFSDVCVHLGCQLPAQVIVSSESDPGLYAKGADLHC
PCHGSIYALKDGGVVVSGPAPRPLPIVILDYDSSTGDIYAVGTNAPYFSAGIPRTTPQDNLLY
DPRYSYSVPNNPSCSNG

11000000000000000000000000000000000000000000000000000000000000000000000000000000000
00000000000000000000000000000000000000000000000000000000000000000000000000000000000
00000000000000000000000000000000000000
>Q7MUU3_2p3p_60_263
SQEIVAGELERCFLAMPESVLPIVTMEERNDLCRRAGHLSGFTHTASLESSLGGTVTFLLNR
NFIRIQTSTVGEVFMRILPFSDSSSVICVVTTVLHPVADSRIDFYTTEWKPLKTDRFWQQPRIE
DFFLPHTDRQSYAYQAIYASLTPSYMQVSLSEESDTLSIRQTVTETLAEEEKPLAAIFLSPEPLV
YRWQSGRFVRQVR
11110000000000000000000000000000000000000000000000000000000000000000000000000000000
00000000000000000000000000000000000000000000000000000000000000000000000000000000000
00000000000000000000000000000000000000
>Q7SID2_1ire_0_203
MTENILRKSDEEIQKEITARVKALESMLIEQGILTTSMIDRMAEIYENEVGPHLGAKVVVKA
WTDPEFKKRLLADGTEACKELGIGGLQGEDMMWVENTDEVHHVVVCTLCSCYPWPVLG
LPPNWFKEPQYRSRVVREPRQLLKEEFGFEVPPSKEIKVWDSSSEMRFVVLPQRPAGTDGWS
EEELATLVTRESMIGVEPAKAV
10000000000000000000000000000000000000000000000000000000000000000000000000000000000
00000000000000000000000000000000000000000000000000000000000000000000000000000000000
00000000000000000000000000000000000000
>Q7Z4H3_4dmb_0_203
MASVSSATFSGHGARSLLQFLRLVGQLKRVPRTGWVYRNVQRPESVSDHMYRMAVMAMV
IKDDRLNKDRCVRLALVHDMAECIVGDIAPADNIPKEEKHRREEEAMKQITQLLPEDLRKE
LYELWEEYETQSSAEAKFVKQLDQCEMILQASEYEDLEHKPGRLQDFYDSTAGKFNHPEIV
QLVSELEAERSTNIAAAASEPHS
11111111110000000000000000000000000000000000000000000000000000000000000000000000000
00000000000000000000000000000000000000000000000000000000000000000000000000000000000
00000000000000000000000000000000001111
>Q8ZPK0_1s9u_0_203
MTTFLQRDDFAVTARVLGALFYYSPESHETAPLVQALLNDDWQAQWPLDAEALAPVAAM
FKTHSEESLPQAWQRLFIGPYALPSPPWGSVWLDRESVLFGDSTLALRQWMRENGIQFEMQ
QNEPEDHFGSLLLLAAWLAENDRHHECEQLLAWHLFPWSSRFLDVFIDHAGHPFYQALG
QLARLTLAQWQAQLIIPVAVKPLFR
00000000000000000000000000000000000000000000000000000000000000000000000000000000000
00000000000000000000000000000000011111100000000000000000000000000000000000000000000
00000000000000000000000000000000000000
>Q96HA8_3c9q_1_204
EGNGPAAVHYQPASPPRDACVYSSCYCEENIWKLCEYIKNHDQYPLEECYAVFISNERKMIP
IWKQQARPGDGPVIWDYHVVLLHVSSGGQNFIYDLDTVLPFPCLFDTYVEDAFKSDDDIHP
QFRRKFRVIRADSYLKNFASDRSHMKDSSGNWREPPPPYPCIETGDSKMNLNDFISMDPKV
GWGAVYTLSEFTHRFGSKNC
11111100000000000000000000000000000000000000000000000000000000000000000000000000000
00000000000000000000000000000000000000000000000000000000000000000000000000000000000
00000000000000000000000000000000000111
>Q9XVS8_3nv0_217_420
FRNGYYGSDEVRTLVEEFIITYYKIYDGADGQQTRKQLLDAYDTNNSTFTHTVVCLWDPIKF
VMYPDSESYRMYLRTSHNVLNQEYFAANRASRISHGAMDIVVALSRLPATIHLMDTFVVDV
FLVSATLLGFTLHGTFRDGPSAIKPENTEEHDNYFTRTFMVAPRGEGKVAIVSDQLFISSMSK
RRGDQYRMLVETATDIDQ
00000000000000000000000000000000000000000000000000000000000000000000000000000000000
00000000000000000000000000000000000000000000000000000000000000000000000000000000000
00000000000000000000000000000111111111

>C1D7P6_3syy_0_202
MEQRTEEWFAARLGKVTASRVADVMTKTKSGYAASRQNYMAELICQRLTGTQEIRFSNAA
MQRGTELEPHARARYIIETGEIVTEVGLIDHPTIAGFGASPDGLVGDTGLIEIKCPNTWTHIE
TIKTGKPKPEYIKQMQTQMACTGRQWCDFVSYDDRLPDDMQYFCTRIERDDALIAEIETEV
SAFLAELEAEIEYLKRKAA
11110000000000000000000000011111000000000000000000000011100000000000000000000000000
00000000000000000000000000000000000000000000000000000000000000000000000000000000000
000000000000000000000000000000000000
>O54180_2id3_0_202
MPDPAAPGTLRPGGRTARIREAVLLAAGDALAADGFDALDLGEIARRAGVGKTTVYRRW
GTPGGLAADLLADMAEQSLPRADTGALEEDLRANARLVVRTLDDPRQGRLFRALIAASLC
NEQAAEALHRFYAVRVDEWAGCVRDAVARGEVPDGTDPHGVVAAVSAPLYYALLNTGRS
LTEADADRAARAASTAARAGVWVTG
11111111111100000000000000000000000000000000000000000000000000000000000000000000000
00000000000000000000000000000000000000000000000000000000000000000000000000000000000
000000000000000000000000000000000000
>Q0S914_2np5_0_202
MRERRYSSTSPERLAAALFDVAAESGLEGASVREVAKRAGVSIGAVQHHFSTKDEMFAFAL
RTLVDKLLARLSEVERGGDPARALFAAMSQLLPLDEARSREAHVMAAFAVRAATSPSLAEI
RRKTLFTIRTGLSAVLIGIGTPEAETRAALLLATVDGLALDAIGSPALYPPEYLEHALDIQIGM
ILQGADVVPSSSIELAS
11111111100000000000000000000000000000000000000000000000000000000000000000000000000
00000000000000000000000000000000000000000000000000000000000000000000000000000000000
000000000000000000000000000011111111
>Q6N6X2_3e23_0_202
MEPDMTQAFDDDTLRFYRGNATAYAERQPRSATLTKFLGELPAGAKILELGCGAGYQAEA
MLAAGFDVDATDGSPELAAEASRRLGRPVRTMLFHQLDAIDAYDAVWAHACLLHVPRDE
LADVLKLIWRALKPGGLFYASYKSGEGEGRDKLARYYNYPSEEWLRARYAEAGTWASVAV
ESSEGKGFDQELAQFLHVSVRKPE
11111100000000000000000000000000000000000000000000000000000000000000000000000000000
00000000000000000000000000000000000000000000000000000000000000000000000000000000000
000000000000000000000000000000000000
>Q74GS0_3dew_0_202
MTRADCRSRLMEVATELFAQKGFYGVSIRELAQAAGASISMISYHFGGKEGLYAAVLQEQF
ACFGQLDDIRGQAGDPLAVMTAYLRWTIQRHRNNPQLLRFYTSELTNPTPCFAAIVSPAIAS
VIRLLAESIEAGMTRGLFRRDLHAVNSALALAGMVNYFFLSTLATEGLTSHSPDQDEELIRQ
YVAIFTRGIMADGGAAPA
11110000000000000000000000000000000000000000000000000000000000000000000000000000000
00000000000000000000000000000000000000000000000000000000000000000000000000000000000
000000000000000000000000000001111111
>Q814U7_3bpk_0_202
MLSINPNEQTEKDNYKLLTGSIIPRPVAFVTSVTKEGVLNGAPYSYFNIVAANPPLISVSVQR
KAGERKDTSRNAIEKGEFVVHISDESYVAAINETAANLPPNESEIELAKLTPIESEVISVPGVK
EANIRMECVLERAIPLGGTEDSPACDLLIGRVVRFHVAEHLYEKGRIHAEGLKPISRLAGHN
YAKLGEQFELVRPI
00000000000000000000000000000000000000000000000000000000000000000000000000000000000
00000000000000000000000000000000000000000000000000000000000000000000000000000000000
000000000000000000000000000000001111
>Q8NQB1_2qec_0_202
MSPTVLPATQADFPKIVDVLVEAFANDPAFLRWIPQPDPGSAKLRALFELQIEKQYAVAGNI
DVARDSEGEIVGVALWDRPDGNHSAKDQAAMLPRLVSIFGIKAAQVAWTDLSSARFHPKF

PHWYLYTVATSSSARGTGVGSALLNHGIARAGDEAIYLEATSTRAAQLYNRLGFVPLGYIPS
DDDGTPELAMWKPPAMPTV
00000000000000000000000000000000000000000000000000000000000000000000000000000000001
11111111111000000010001111111110000000000000000000000000000000000000000000000000000
0000000000000000000000000000000000000
>Q99418_1r8q_49_251
LEANEGSKTLQRNRKMAMGRKKFNMDPKKGIQFLVENELLQNTPEEIARFLYKGEGLNKT
AIGDYLGEREELNLAVLHAFVDLHEFTDLNLVQALRQFLWSFRLPGEAQKIDRMMEAFAQ
RYCLCNPGVFQSTDTCYVLSFAVIMLNTSLHNPNVRDKPGLERFVAMNRGINEGGDLPEEL
LRNLYDSIRNEPFKIPEDDGND
11111100000000000000000000000000000000000000000000000000000000000000000000000000000
00000000000000000000000000000000000000000000000000000000000000000000000000000000000
0000000000000000000000000000000000111
>Q9HJ63_2gvi_0_202
MEKLNFGIPEWAFEFHGHKCPYMPMGYRAGSYALKIAGLEKEKDHRTYLLSEMSPEDMN
GCFNDGAQAATGCTYGKGLFSLLGYGKLALILYRPGRKAIRVHVRNSFMDELSTRASDFFR
YRKQGYEPSEIPAGAIDPVLEWISSLEDEEIFEYREIDGFTFEPVKKNGAKVRCDVCGEYTYE
ADAKLLNGKPVCKPDYYGKK
00000000000000000000000000000000000000000000000000000000000000000000000000000000000
00000000000000000000000000000000000000000000000000000000000000000000000000000000000
0000000000000000000000000000000000011
>Q9KSX0_4gud_0_202
MTQNVVIIDTGCANISSVKFAIERLGYAVTISRDPQVVLAADKLFLPGVGTASEAMKNLTER
DLIELVKRVEKPLLGICLGMQLLGKLSEEKGQKADEIVQCLGLVDGEVRLLQTGDLPLPHM
GWNTVQVKEGHPLFNGIEPDAYFYFVHSFAMPVGDYTIAQCEYGQPFSAAIQAGNYYGVQ
FHPERSSKAGARLIQNFLEL
10000000000000000000000000000000000000000000000000000000000000000000000000000000000
00000000011110000000000000000000000000000000000000000000000000000000000000000000000
0000000000000000000000000000000000000
>A9CI22_2hly_0_201
MLIKQTDYFRIYRVINSLLISQNADPASASMYFSTFGAFILQQHYKVKAVPKGGLAAYNLGG
TVLLFADHREDGYVTGAGENFHCWVEADGWAIDFMAPAFSEGTDALAVPAKMFQRPLSA
MAASINDLGQSGDFFYRSEPEATARRFADWHKQAMIGDMASVAANWFRKSPKQMAASLS
VTDRDGKARTVPLTGEMLTGAW
00000000000000000000000000000000000000000000000000000000000000000000000011000000000
00000000000000000000000000000000000000000000000000000000000000000000000000000000000
000000000000000000000000000000000000
>B1Y9Q9_4df1_0_201
MAANLPLVVALDTEVLKAIDVAKRLKGAVAGFKVGWDLIFEGGISIVGEIARYGNVIVDLKI
ADVPHVASRVVEKLVNRGACCVIVHGFLHPSLPRGQHVYVLVKMTAPTIYDEMWEKLLNS
VQDVRGFVLPGNQPEVVAQARKRIGCSYRIISPGIGPQGGRPGAAIEAGADFEIVGRYVLED
PARISQWAQYRPTCFETP
11100000000000000000000000000000000000000000000000000000000000000000000000000000000
00000000000000000000000000000000000000000000000000000000000000000000000000000000000
000000000000000000000000000000000000
>P19320_1vca_24_225
FKIETTPESRYLAQIGDSVSLTCSTTGCESPFFSWRTQIDSPLNGKVTNEGTTSTLTMNPVSFG
NEHSYLCTATCESRKLEKGIQVEIYSFPKDPEIHLSGPLEAGKPITVKCSVADVYPFDRLEIDL
LKGDHLMKSQEFLEDADRKSLETKSLEVTFTPVIEDIGKVLVCRAKLHIDEMDSVPTVRQAV
KELQVYISPKNT
00000000000000000000000000000000000000000000000000000000000000000000000000000000000

00000000000000000000000000000000000000000000000000000000000000000000000000000000000
00000000000000000000000000000000111
>P43642_1d02_0_201
MGKSELSGRLNWQALAGLKASGAEQNLYNVFNAVFEGTKYVLYEKPKHLKNLYAQVVLP
DDVIKEIFNPLIDLSTTQWGVSPDFAIENTETHKILFGEIKRQDGWVEGKDPSAGRGNAHER
SCKLFTPGLLKAYRTIGGINDEEILPFWVVFEGDITRDPKRVREITFWYDHYQDNYFMWRPN
ESGEKLVQHFNEKLKKYLD
11000000000000000000000000000000000000000000000000000000000000000000000000000000000
00000000000000000000000000000000000000000000000000000000000000000000000000000000000
00000000000000000000000000000000000
>P95092_3v6g_0_201
MTAGSDRRPRDPAGRRQAIVEAAERVIARQGLGGLSHRRVAAEANVPVGSTTYYFNDLDA
LREAALAHAANASADLLAQWRSDLDKDRDLAATLARLTTVYLADQDRYRTLNELYMAA
AHRPELQRLARLWPDGLLALLEPRIGRRAANAVTVFFDGATLHALITGTPLSTDELTDAIAR
LVADGPEQREVGQSAHAGRTPD
11111111111110000000000000000000000000000000000000000000000000000000000000000000000
00000000000000000000000000000000000000000000000000000000000000000000000000000000000
00000000000000000001111111111111111
>Q082D2_3fge_0_201
MHFSKEHINALETRTRAHFINSLSGFKSANLIGTQDRQGNTNLSIVSSVIHLGANPPLMGMI
IRPHSVPRHTFENIMQTGLYTINHVNQSIYEQAHQTSARYDKDESEFEATGLTPEYLSDFCAP
FVKESRLKYSVKLVEHQHLAINGTEFVIGEIVDVYVDDNAVQTDGFIDLQAIDTVAISGLDC
YYTGDKLARLPYAKK
00000000000000000000000000000000000000000000000000000000000000000111000000000000000
00000000000000000000000000000000000000000000000000000000000000000000000000000000000
00000000000000000000000000000000011
>Q5SIL0_2zcw_0_201
MTQVRETVSFKAGDVILYPGVPGPRDRAYRVLEGLVRLEAVDEEGNALTLRLVRPGGFFGEE
ALFGQERIYFAEAATDVRLEPLPENPDPELLKDLAQHLSQGLAEAYRRIERLATQRLKNRM
AAALLELSETPLAHEEEGKVVLKATHDELAAAVGSVRETVTKVIGELAREGYIRSGYGKIQL
LDLKGLKELAESRGQGR
11111000000000000000000000000000000000000000000000000000000000000000000000000000000
00000000000000000000000000000000000000000000000000000000000000000000000000000000000
00000000000000000000000000000000111
>Q9CED0_2wqf_0_201
MSFIKSLENRRTIYALGRNVQDEEKVIETIKEAVRFSPTAFNSQTGRLLILTGDAQDKLWDEIV
APELKAAMEAQGVPESAWDNTRAKLDGFKAAFGTILFFEDQAVVKNLQEQFALYADNFP
VWSEQGSGIISVNVWTALAELGLGANLQHYNPLIDEAVAKEWNLPESWKLRGQLVFGSIEA
PAGEKTFMDDADRFIVAK
10000000000000000000000000000000000000000000000000000000000000000000000000111111111
11100000000000000000000000000000000000000000000000000000000000000000000000000000000
00000000000000000000000000000000000
>O67413_2e8b_0_200
MRTFTWRKGSLSKVNTCYVLAGGKSKRFGEDKLLYEIKGKKVIERVYETAKSVFKEVYIVAK
DREKFSFLNAPVVLDEFEESASIIGLYTALKHAKEENVFVLSGDLPLMKKETVLYVLENFKEP
VSVAKTEKLHTLVGVYSKKLLEKIEERIKKGDYRIWALLKDVGYNEVEIPEELRYTLLNMNT
KEDLKRILAIENHY
11100000000000000000000000000000000000000000000000000000000000000000000000000000000
00000000000000000000000000000000000000000000000000000000000000000000000000000000000
0000000000000000000000111111111111
>O75146_1r0d_770_970

SLDVRQEELGAVVDKEMAATSAAIEDAVRRIEDMMNQARHASSGVKLEVNERILNSCTDL
MKAIRLLVTTSTSLQKEIVESGRGAATQQEFYAKNSRWTEGLISASKAVGWGATQLVEAAD
KVVLHTGKYEELIVCSHEIAASTAQLVAASKVKANKHSPHLSRLQECSRTVNERAANVVAS
TKSGQEQIEDRDTMDFSGL
11000000000000000000000000000000000000000000000000000000000000000000000000000000000
00000000000000000000000000000000000000000000000000000000000000000000000000000000000
00000000000000000000000000111111111
>P03120_1dto_0_200
METLCQRLNVCQDKILTHYENDSTDLRDHIDYWKHMRLECAIYYKAREMGFKHINHQVV
PTLAVSKNKALQAIELQLTLETIYNSQYSNEKWTLQDVSLEVYLTAPTGCIKKHGYTVEVQF
DGDICNTMHYTNWTHIYICEEASVTVVEGQVDYYGLYYVHEGIRTYFVQFKDDAEKYSKN
KVWEVHAGGQVILCPTSVFS
00000000000000000000000000000000000000000000000000000000000000000000000000000000000
00000000000000000000000000000000000110000000000000000000000000000000000000000000000
00000000000000000000000000000000000
>P84134_1rz3_0_200
MELRDRIDFLCKTILAIKTAGRLVLGIDGLSRSGKTTLANQLSQTLREQGISVCVFHMDDHIV
ERAKRYHTGNEEWFEYYYLQWDVEWLTHQLFRQLKASHQLTLPFYDHETDTHSKRTVYLS
DSDMIMIEGVFLQRKEWRPFFDFVVYLDCPREIRFARENDQVKQNIQKFINRYWKAEDYYL
ETEEPIKRADVVFDMTS
00000000000000000000000000000000000000000000000000000000000000000000000000000000000
00000000000000000000000000000000000000000000000000000000000000000000001111111111111
10000000000000000000000000000000111
>Q5LRV1_3cvo_0_200
MDDQSGDQMRPELTMPPAEAEALRMAYEEAEVILEYGSGGSTVVAAELPGKHVTSVESDR
AWARMMKAWLAANPPAEGTEVNIVWTDIGPTGDWGHPVSDAKWRSYPDYPLAVWRTE
GFRHPDVVLVDGRFRVGCALATAFSITRPVTLLFDDYSQRRWQHQVEEFLGAPLMIGRLAA
FQVEPQPIPPGSLMQLIRTMTSP
11111110000000000000000000000000000000000000000000000000000000000000000000000000000
00000000000000000000000000000000000000000000000000000000000000000000000000000000000
00000000000000000000000000000000000
>Q838Q8_3hra_0_200
MKTYEVGALLEAANQRDTKKVKEILQDTTYQVDEVDTEGNTPLNIAVHNNDIEIAKALID
RGADINLQNSISDSPYLYAGAQGRTEILAYMLKHATPDLNKHNRYGGNALIPAAEKGHIDN
VKLLLEDGREDIDFQNDFGYTALIEAVGLREGNQLYQDIVKLLMENGADQSIKDNSGRTAM
DYANQKGYTEISKILAQYN
11000000000000000000000000000000000000000000000000000000000000000000000000000000000
00000000000000000000000000000000000000000000000000000000000000000000000000000000000
00000000000000000000000000000000000
>Q9P2F6_3msx_350_550
PSSPTSPMPGQLFGISLPNICENDNLPKPVLDMLFFLNQKGPLTKGIFRQSANVKSCRELKEK
LNSGVEVHLDCESIFVIASVLKDFLRNIPGSIFSSDLYDHWVSVMDQGNDEEKINTVQRLLD
QLPRANVVLLRYLFGVLHNIEQHSSSNQMTAFNLAVCVAPSILWPPASSSPELENEFTKKVS
LLIQFLIENCLRIF
11111111100000000000000000000000000000000000000000000000000000000000000000000000000
00000000000000000000000000000000000000000000000000000000000000000000000000000000000
00000000000000000000000000000000000
>Q9WUL5_3bp5_19_219
LFTVTAPKEVYTVDVGSSVSLECDFDRRECTELEGIRASLQKVENDTSLQSERATLLEEQLPL
GKALFHIPSVQVRDSGQYRCLVICGAAWDYKYLTVKVKASYMRIDTRILEVPGTGEVQLTC
QARGYPLAEVSWQNVSVPANTSHIRTPEGLYQVTSVLRLKPQPSRNFSCMFWNAHMKELT

SAIIDPLSRMEPKVPRT
00000000000000000000000000000000000000000000000000000000000000000000000000000000
00000000000000000000000000000000000000000000000000000000000000000000000000000000
0000000000000000000000001111111111
>Q9WZZ4_1vr9_0_200
MKVKKWVTQDFPMVEESATVRECLHRMRQYQTNECIVKDREGHFRGVVNKEDLLDLDL
DSSVFNKVSLPDFFVHEEDNITHALLLFLEHQEPYLPVVDEEMRLKGAVSLHDFLEALIEAL
AMDVPGIRFSVLLEDKPGELRKVVDALALSNINILSVITTRSGDGKREVLIKVDAVDEGTLIK
LFESLGIKIESIEKEEGF
00000000000000000000000000000000000000000000000000000000000000000000000000000000
00000000000000000000000000000000000011111111111111111111111111111111111111111111
1111111111111111111111111111111111
>D3FPI3_3q3w_0_199
MQKFIIHKGIACPLEYANIDTDQIIPKQFLLAVSKQGFGKHLFHDLRYLDDKESVLNMDFNL
NKKEYQNSSILVSFENFGSGSSREHAPWALVDYGIRAIIAPSFADIFKNNALGNGLLTIELAK
DEVLEIVDELKKSQDKNIEISLLEKRVFFKDKIFSFDLDDFHRICLLEGLDNIALTLKHEAQIK
AYEKNSKSFLV
00000000000000000000000000000000000000000000000000000000000000000000000000000000
00000000000000000000000000000000000000000000000000000000000000000000000000000000
000000000000001111111111111111111
>O58461_2p8t_14_213
MVGQVIRKRGAYPEYTVEDVLAVIFLLKEPLGRKQISERLELGEGSVRTLLRKLSHLDIIRSKQ
RGHFLTLKGKEIRDKLLSMFSEPIGVSVDGYPGIAIVVKNPPEFKSIELRDEAIKFDAKGAMIL
TVKDNEIVFPEDFRPLKEMYPEVAKKIVDYEDGDAVIITWAETPAKALKSAIHVAYILKKEEIT
PEILEVVK
11111111111100000000000000000000000000000000000000000000000000110000000000000000
00000000000000000000000000000000000000000000000000000000000000000000000000000000
000000000000000000000000000000001
>P21816_3eln_0_199
MERTELLKPRTLADLIRILHELFAGDEVNVEEVQAVLEAYESNPAEWALYAKFDQYRYTRNL
VDQGNGKFNLMILCWGEGHGSSIHDHTDSHCFLKLLQGNLKETLFDWPDKKSNEMIKKS
ERTLRENQCAYINDSIGLHRVENVSHTEPAVSLHLYSPPFDTCHAFDQRTGHKNKVTMTFH
SKFGIRTPFTTSGSLENN
11100000000000000000000000000000000000000000000000000000000000000000000000000000
00000000000000000000000000000000000000000000000000000000000000000000000000000000
000000000000000000000001111111111
>P23468_2yd7_20_225
ETPPRFTRTPVDQTGVSGGVASFICQATGDPRPKIVWNKKGKKVSNQRFEVIEFDDGSGSVL
RIQPLRTPRDEAIYECVASNNVGEISVSTRLTVLREDQIPRGFPTIDMGPQLKVVERTRTATM
LCAASGNPDPEITWFKDFLPVDTSNNNGRIKQLRSESIGALQIEQSEESDQGKYECVATNSA
GTRYSAPANLYVR
00000000000000000000000000000000000000000000000000000000000000000000000000000000
00000000000000000000000000000000000000000000000000000000000001110000000000000000
000000000000000000000000000000000
>P28147_1qna_0_199
MTDQGLEGSNPVDLSKHPSGIVPTLQNIVSTVNLDCKLDLKAIALQARNAEYNPKRFAAVI
MRIREPKTTALIFASGKMVCTGAKSEDFSKMAARKYARIVQKLGFPAKFKDFKIQNIVGSCD
VKFPIRLEGLAYSHAAFSSYEPELFPGLIYRMKVPKIVLLIFVSGKIVITGAKMRDETYKAFENI
YPVLSEFRKIQQ
11111111110000000000000000000000000000000000000000000000000000000000000000000000
00000000000000000000000000000000000000000000000000000000000000000000000000000000

0000000000000000000000000000000011
>P29421_2qn4_0_199
MVSLRLPLILLSLLAISFSCSAAPPPVYDTEGHELSADGSYYVLPASPGHGGGLTMAPRVLPC
PLLVAQETDERRKGFPVRFTPWGGAAAPEDRTIRVSTDVRIRFNAATICVQSTEWHVGDEPL
TGARRVVTGPLIGPSPSGRENAFRVEKYGGGYKLVSCRDSCQDLGVSRDGARAWLGASQPP
HVVVFKKARPSPPE
11111111111111111111100000000000000000000000000000000000000000000000000000000000000
00000000000000000000000000000000000011111100000000111111000000000000000000000000000
0000000000000000000000000000111111
>P55010_2iu1_231_430
LERTIEERVNILFDFVKKKKEEGVIDSSDKEIVAEAERLDVKAMGPLVLTEVLFNEKIREQIKK
YRRHFLRFCHNNKKAQRYLLHGLECVVAMHQAQLISKIPHILKEMYDADLLEEEVIISWSE
KASKKYVSKELAKEIRVKAEPFIKWLKEAEEESSGGEEEDEDENIEVVYSKAASVPKVETVKS
DNKDDDIDIDAI
00000000000000000000000000000000000000000000000000000000000000000000000000000000000
00000000000000000000000000000000000000000000000000000000000000000000000000000000000
0000000000000011111111111111111111
>Q43295_4fxp_76_275
NSTNIKWHECSVEKVDRQRLLDQKGCVIWVTGLSGSGKSTLACALNQMLYQKGKLCYILD
GDNVRHGLNRDLSFKAEDRAENIRRVGEVAKLFADAGIICIASLISPYRTDRDACRSLLPEG
DFVEVFMDVPLSVCEARDPKGLYKLARAGKIKGFTGIDDPYEPPLNCEISLGREGGTSPIEM
AEKVVGYLDNKGYLQA
11111110000000000000000000000000000000000000000000000000000000000000000000000000000
00000000000000000000000000000000000000000000000000000000000000000000000000000000000
0000000000000000000000000000000000
>Q5SHB9_2fk5_0_199
MRARLYAAFRQVGEDLFAQGLISATAGNFSVRTKGGFLITKSGVQKARLTPEDLLEVPLEGPI
PEGASVESVVHREVYRRTGARALVHAHPRVAVALSFHLSRLRPLDLEGQHYLKEVPVLAPK
TVSATEEAALSVAEALREHRACLLRGHGAFAVGLKEAPEEALLEAYGLMTTLEESAQILLYH
RLWQGAGPALGGGE
10000000000000000000000000000000000000000000000000000000000000000000000000000000000
00000000000000000000000000000000000000000000000000000000000000000000000000000000000
0000000000000000000000000000001111
>Q5SJJ6_2yzy_14_213
SLGLAQSAQEILDRVEKNLSTPWQATVQGRIQGPGGEEELLARVYALPQARLFRVEFLKPGS
LEGNFTVITEKEVWNYLYLTNQLVISPREKAKIQGLGFAPQGLGDLKALSEQVDLRLEGEVR
LPEGMAWKLVGRSKENQGFAAMELYILKADPRPLRFVFLDEKGKVLADLKVVEFKRTNLT
EAQLKRYPKDAQVVRR
11111000000000000000000000001111100000000000000000000000000000000000000000000000000
00000011111111111111111111111100000000000000000000000011100000000000000000000000000
0000000000000000000000000000000000
>Q6P4G7_2nw2_6_208
QNIDQPTEMTATEGAIVQINCTYQTSGFNGLFWYQQHAGEAPTFLSYNVLDGLEEKGRFSS
FLSRSKGYSYLLLKELQMKDSASYLCAVSRRGGGNKLTFGTGTQLKVELNIQNPDPAVYQL
RDSKSSDKSVCLFTDFDSQTNVSQSKDSDVYITDKTVLDMRSMDFKSNSAVAWSNKSDFAC
ANAFNNSIIPEDTFFPS
00000000000000000000000000000000000000000000000000000000000000000000000000000000000
00000000100000000000000000000000000000000011000000000000000000000000000000000000000
0000000000000000000000000000000000
>Q96HS1_3mxo_89_288
DHYKAKATRHIFLIRHSQYHVDGSLEKDRTLTPLGREQAELTGLRLASLGLKFNKIVHSSMT

RAIETTDIISRHLPGVCKVSTDLLREGAPIEPDPPVSHWKPEAVQYYEDGARIEAAFRNYIHR
ADARQEEDSYEIFICHANVIRYIVCRALQFPPEGWLRLSLNNGSITHLVIRPNGRVALRTLGD
TGFMPPDKITRS
00000000000000000000000000000000000000000000000000000000000000000000000000000000000
00000000111111111100000000000000000000000000000000000000000000000000000000000000000
0000000000000000000000000000000000
>Q9KZX2_3g7r_0_199
MSPSTEAAARTPSEARARLLGTATRIFYAEGIHSVGIDRITAEAQVTRATLYRHFSGKDDLILA
YLDQADRGIRAQVTAARGSSPAADGQVRAVARSIVDGIRSPGFRGCAFLNAVAEYPDPAHP
VHRAVLAHRQWFLDTVTELLAQVGDGDGVAAGRHLVMLRDGAMAAGCLFDPELVSETF
LHGVEGVLRDVSEKTSA
00000000000000000000000000000000000000000000000000000000000000000000000000000000000
00000000000000000000000000000000000000000000000000000000000000000000000000000000000
0000000000000000000000000000001111
>A3D7B7_2q9r_0_198
MTKKTGFFKRLKALTLPQKQLFATALCQRMLPNYQLFSEVCEFGDPAVLSTALELLWQSLY
DPKLKFNIDVHLQRLEDNTPEPADFEAYGVYPAMDAVVAISTLLGAIQGKIEEDIVNISKLSS
STVANYIEAISDVDLVDEALDDFVFAHEVMEEEKELQNSLLEIIEENPKITAELVKGLRKDIIE
TGVSNIGISVA
11100000000000000000000000000000000000000000000000000000000000000000000000000000000
00000000000000000000000000000000000000000000000000000000000000000000000000000000000
000000000000000000000000000000000
>P01910_1iak_23_221
EDDIEADHVGSYGITVYQSPGDIGQYTFEFDGDELFYVDLDKKETVWMLPEFAQLRRFEPQ
GGLQNIATGKHNLEILTKRSNSTPATNEAPQATVFPKSPVLLGQPNTLICFVDNIFPPVINIT
WLRNSKSVTDGVYETSFFVNRDYSFHKLSYLTFIPSDDDIYDCKVEHWGLEEPVLKHWEPEI
PAPMSELTETVVC
11100000000000000000000000000000000000000000000000000000000000000000000000000000000
00000000000000000000000000000000000000000000000000000000000000000000000000000000000
000000000000000000011111111111111
>P24766_2vga_20_218
DDKSVCDSDNKEYMGIEVYVEATLDEPLRQTTCESKIHKYGASVSNGGLNISVDLLNCFLN
FHTVGVYTNRDTVYAKFASLDPWTTEPINSMTHDDLVKLTEECIVDIYLKCEVDKTKDFMK
TNGNRLKPRDFKTVPPSNVGSMIELQSDYCVNDVTTYVKIYDECGNIKQHSIPTLRDYFTTK
NGQPRKILKKKFDNC
11111000000000000000000000000000000000000000000000000000000000000000000000000000000
00000000000000000000000000000000000000000000000000000000000000000000000000000000000
000000000000000000000000000000000
>P48060_3q2u_21_219
ANILPDIENEDFIKDCVRIHNKFRSEVKPTASDMLYMTWDPALAQIAKAWASNCQFSHNTR
LKPPHKLHPNFTSLGENIWTGSVPIFSVSSAITNWYDEIQDYDFKTRICKKVCGHYTQVVWA
DSYKVGCAVQFCPKVSGFDALSNGAHFICNYGPGGNYPTWPYKRGATCSACPNNDKCLD
NLCVNRQRDQVKRYYSV
00000000000000000000000000000000000000000000000000000000000000000000000000000000000
00000000000000000000000000000000000000000000000000000000000000000000000000000000000
000000000000000000000000000111111
>Q0SHW9_3on2_0_198
MPVAEQPYHHGSLRRVLLARAESTLEKDGVDGLSLRQLAREAGVSHAAPSKHFRDRQALL
DALAESGFLRLTAALERAVEEAESHARARFAALAGAYVSFALAHRELLALMYGNKHAPGA
ASQVVEAGHASMDLTVRIVTEAQAAGDIGPGDASRIALVAFATFHGIATLAAGGMLDGAP
VDEVVTAASDTFWRGLAQA

11100000000000000000000000000000000000000000000000000000000000000000000000000000000
00000000000000000000000000000000000000000000000000000000000000000000000000000000000
000000000000000000000000000000001
>Q14995_3cqv_380_578
HLVCPLSKSPYVDPHKSGHEIWEEFSMSFTPAVKEVVEFAKRIPGFRDLSQHDQVNLLKAGT
FEVLMVRFASLFDAKERTVTFLSGKKYSVDDLHSMGAGDLLNSMFEFSEKLNALQLSDEEM
SLFTAVVLVSADRSGIENVNSVEALQETLIRALRTLIMKNHPNEASIFTKLLLKLPDLRSLNN
MHSEELLAFKVHP
00000000000000000000000000000000000000000000000000000000000000000000000000000000000
00000000000000000000000000000000000000000000000000000000000000000000000000000000000
000000000000000000000000000000111
>Q1LW01_3lqb_64_262
NALICEDKSCFWKKNANNIVEVPYVVSGEFSINDKSVIANAISIFHAQTCIRFVPRSIQADYL
SIENKDGCYSAIGRTGGKQVVSLNRKGCVYSGIAQHELNHALGFYHEQSRSDRDQYVRIN
WNNISPGMAYNFLKQKTNNQNTPYDYGSLMHYGKTAFAIQPGLETITPIPDENVQIGQRQ
GLSKIDILRINKLYGC
10000000000000000000000000000000000000000000000000000000000000000000000000000000000
00000000000000000000000000000000000000000000000000000000000000000000000000000000000
000000000000000000000000000000000
>Q7L0Q8_2q3h_31_229
GPGEPGGRGRAGGAEGRGVKCVLVGDGAVGKTSLVVSYTTNGYPTEYIPTAFDNFSAVVSV
DGRPVRLQLCDTAGQDEFDKLRPLCYTNTDIFLLCFSVVSPSSFQNVSEKWVPEIRCHCPKA
PIILVGTQSDLREDVKVLIELDKCKEKPVPEEAAKLCAEEIKAASYIECSALTQKNLKEVFDA
AIVAGIQYSDTQQ
11111111111111110000000000000000000000001111111100000000000000000000000000000000000
00000000000000000000000000000000000000000000000000000000000000000000000000000000000
000000000000000000000000000000111
>Q9HSF6_4e19_0_198
MPVVECDIQTARAALADAGASFSDGNSEHELWHADLGDAHAVAYADKLVVQGGSPTDIT
AVVQPDRGGRVHAYFDGASRGNPGPAAVGWVLVSGDGGIVAEGGDTIGRATNNQAEYDA
LIAALEAAADFGFDDIELRGDSQLVEKQLTGAWDTNDPDLRRKRVRARELLTGFDDWSITH
VPRATNERADALANEALDDA
11111111111111111111111111111111111111111111111111111111111111111110000000000000000
00000000000000000000000000000000000000000000000000000000000000000000000000000000000
000000000000000000000000000000000
>Q9NWW6_2qg6_0_198
MKTFIIGISGVTNSGKTTLAKNLQKHLPNCSVISQDDFFKPESEIETDKNGFLQYDVLEALN
MEKMMSAISCWMESARHSVVSTDQESAEEIPILIIEGFLLFNYKPLDTIWNRSYFLTIPYEECK
RRRSTRVYQPPDSPGYFDGHVWPMYLKYRQEMQDITWEVVYLDGTKSEEDLFLQVYEDLI
QELAKQKCLQVTA
10000000000000000000000000000000000000000000000000000000000000000000000000000000001
11111111000000000000000000000000000000000000000000000000000000000000000000000000000
000000000000000000000000001111111
>Q9RWF4_2c2p_0_198
MTAPHDVPDLTGSGEYLVPDVLQPGLTLVLVGTAPSGISARARAYYANPENKFWRTLHAVG
LTPRQLVPQEYATLPQYGLGLTDVAKRHSGVDAALPGEAWRPDELRRKVEHYRPRIVAFTS
KRGASETLGVPTGKLPYGPQPQPLDWPAETELWVLPSTSPLGHNHFRLEPWQALGDRVRE
LRGAAEAGNPSPETPVL
11111100000000000000000000000000000000000000000000000000000000000000000000000000000
00000000000000000000000000000000000000000000000000000000000000000000000000000000000
000000000000000000000001111111111

>Q9X1A2_3e57_0_198
MKSERILVVKTEDFLKEFGEFEGFMRVNFEDFLNFLDQYGFFRERDEAEYDETTKQVIPYVVI
MDGDRVLITKRTTKQSEKRLHNLYSLGIGGHVREGDGATPREAFLKGLEREVNEEVDVSLR
ELEFLGLINSSTTEVSRVHLGALFLGRGKFFSVKEKDLFEWELIKLEELEKFSGVMEGWSKISA
AVLLNLFLTQN
11100000000000000000000000000000000000000000000000000000000000000000000000111111111
00000000000000000000000000000000000000000000000000000000000000000000000000000000000
000000000000000000000000000001111
>A3D092_3hmz_0_197
MHHQRKTHVDNNIAAVELAKAYRLLNHGPTVLVSARSQGIDNVMAAAWCCALDFAPPK
LTVVLDKMTKTREFIEQSGMFVIQVPTVAQLQLTHRVGSQSLADDANKLLNCGVELFEMA
GHDLPFVAGCSAWLACKLIPEPNNQLQYDLFIAEVIGAWADTQVFNHGHWHFDTAPADK
RSLHYIAGGQFYAIGESFNAD
11111110000000000000000000000000000000000000000000000000000000000000000000000000000
00000000000000000000000000000000000000000000000000000000000000000000000000000000000
00000000000000000000000000000000
>A6AYI6_3er6_3_200
TNKKNLRVVALAPTGRYFASIISSLEILETAAEFAEFQGFMTHVVTPNNRPLIGRGGISVQPT
AQWQSFDFTNILIIGSIGDPLESLDNIDPALFDWIRELHLKGSKIVAIDTGIFVVAKAGLLQQN
KAVMHSYFAHLFGELFPEIMLMTEQKALIDGNVYLSSGPYSHSSVMLEIVEEYFGKHTRNLG
NQFLSTIES
11100000000000000000000000000000000000000000000000000000000000000000000000000000000
00000000000000000000000000000000000000000000000000000000000000000000000000000000000
00000000000000000000000000000111
>A9CKF0_2qni_0_197
MHALYITHPQVKIDPAVPVPEWGLSERGAERAREASRLPWAKALRRIVSSAETKAIETAHM
LAETSGAAIEIIEAMHENDRSATGFLPPPEFEKAADWFFAHPEESFQGWERAIDAQARIVEA
VKAVLDRHDARQPIAFVGHGGVGTLLKCHIEGRGISRSKDQPAGGGNLFRFSIAEFSLAAA
SPTCDWTAMETWQG
00000000000000000000000000000000000000000000000000000000000000000000000000000000000
00000000000000000000000000000000000000000000000000000000000000000000000000000011000
00000000000000100011000000000000
>C8ZIW7_3qle_163_360
FNSMFTYFQEPPFPDLLPPPPPPPYQRPLTLVITLEDFLVHSEWSQKHGWRTAKRPGADYFL
GYLSQYYEIVLFSSNYMMYSDKIAEKLDPIHAFVSYNLFKEHCVYKDGVHIKDLSKLNRDLS
KVIIIDTDPNSYKLQPENAIPMEPWNGEADDKLVRLIPFLEYLATQQTKDVRPILNSFEDKK
NLAEEFDHRVKK
11111111111100000000011110000000000000000000000000000000000000000000000000000000000
00000000000000000000000000000000000000000000000000000000000000000000000000000000000
00000000000000000000000000000000
>O57724_2dm9_0_197
MNGAELIIQEINKEAERKIEYILNEARQQAEKIKEEARRNAEAKAEWIIRRAKTQAELEKQR
IIANARLEVRRKRLAIQEEIISSVLEEVKRRLETMSEDEYFESVKALLKEAIKELNEKKVRVMS
NEKTLGLIASRIEEIKSELGDVSIELGETVDTMGGVIVETEDGRIRIDNTFEARMERFEGEIRST
IAKVLFG
11111111111111111111111111111111111111111111111111111111111111111111111111111111000
00000000000000000000000000000000000000000000000000000000000000000000000000000000000
00000000000000000000000000000000
>O69002_1sk7_0_197
MDTLAPESTRQNLRSQRLNLLTNEPHQRLESLVKSKEPFASRDNFARFVAAQYLFQHDLEP
LYRNEALARLFPGLASRARDDAARADLADLGHPVPEGDQSVREADLSLAEALGWLFVSEG

SKLGAAFLFKKAAALELDENFGARHLAEPEGGRAQGWKSFVAILDGIELNEEEERLAAKG
ASDAFNRFGDLLERTFA
11111111111100000000000000000000000000000000000000000000000000000000000000000000000
00000000000000000000000000000000000000000000000000000000000000000000000000000000000
00000000000000000000000000000000
>P13591_3mtr_413_610
YAPKLQGPVAVYTWEGNQVNITCEVFAYPSATISWFRDGQLLPSSNYSNIKIYNTPSASYLEV
TPDSENDFGNYNCTAVNRIGQESLEFILVQADTPSSPSIDQVEPYSSTAQVQFDEPEATGGVPI
LKYKAEWRAVGEEVWHSKWYDAKEASMEGIVTIVGLKPETTYAVRLAALNGKGLGEISAA
SEFKTQPVQGE
00000000000000000000000000000000000000000000000000000000000000000000000000000000000
00000000000000000000000000000000000000000000000000000000000000000000000000000000000
00000000000000000000000000000011
>P40893_3o12_0_197
MVESKNTELSQGTWLNKPKSVFQEAGKVTLETDEKTDFWRETFYGFTRDSGHFLGVETGS
AFTAQVRVQGSYESLYDQAGIMVRIDDGHWLKAGIEISDGHAMLSSVLTNGKSDWSTAVY
GGNARDFWLRVTVEKGVLRIQVSSDKKTWPLVRLAPFPTSDHYLVGPMACTPERGGLKVT
FSEWSLTAPLGKALHDLS
11111100000000000000000000000000000000000000000000000000000000000000000000000000000
00000000000000000000000000000000000000000000000000000000000000000000000000000000000
00000000000000000000000001111111
>P96856_3mnl_1_198
AVLAESELGSEAQRERRKRILDATMAIASKGGYEAVQMRAVADRADVAVGTLYRYFPSKVH
LLVSALGREFSRIDAKTDRSAVAGATPFQRLNFMVGKLNRAMQRNPLLTEAMTRAYVFAD
ASAASEVDQVEKLIDSMFARAMANGEPTEDQYHIARVISDVWLSNLLAWLTRRASATDVS
KRLDLAVRLLIGDQDSA
11111110000000000000000000000000000000000000000000000000000000000000000000000000000
00000000000000000000000000000000000000000000000000000000000000000000000000000000000
00000000000000000000000000000011
>Q03154_1q7l_0_197
MTSKGPEEEHPSVTLFRQYLRIRTVQPKPDYGAAVAFFEETARQLGLGCQKVEVAPGYVVT
VLTWPGTNPTLSSILLNSHTDVVPVFKEHWSHDPFEAFKDSEGYIYARGAQDMKCVSIQYL
EAVRRLKVEGHRFPRTIHMTFVPDEEVGGHQGMELFVQRPEFHALRAGFALDEGIANPTD
AFTVFYSERSPWWVRV
11111100000000000000000000000000000000000000000000000000000000000000000000000000000
00000000000000000000000000000000000000000000000000000000000000000000000000000000000
00000000000000000000000000000000
>Q2W5N3_3bhw_1_198
AEPRYKADIGGGSLKLPESRIIAGLLLEGVTEDQWRHAIEVENVLQRRSPGTAKRQSSLMRN
RLETMGPELWQMVRDGSTQVAIQAVFAAAIKHSTLLGDFLDLVVRDQFRMFRPDLPRKM
WDQYLEQCRNRDPLMPVWQDSTANKLADCVYRILVEVGYITDSKTYRLKSVRISGEVMSYL
RENNEQYVIRCIQVSI
11000000000000000000000000000000000000000000000011110000000000000000000000000000000
00000000000000000000000000000000000000000000000000000000000000000000000000000000000
00000000000000000000000000000001
>Q6NEA9_2gs5_0_197
MFADRLFNAMERNEPAPGMVLVAAPSMESEDFARSVILIIEHSEYATFGVNLASRSDVAVFN
VIPEWVPCVTKPQALYIGGPLNQQSVVGVGVTAQGVDAARVDNLTRLANRLVMVNLGAD
PEEIKPLVSGMRLFAGHAEWAPGQLAQEIENGDWFVAPALPSDVTAPGSVDVWGDVMRR
QPMPLPLYSTFPVNVGEN
00000000000000000000000000000000000000000000000000000000000000000000000000000000000

00000000000000000000000000000000000000000000000000000000000000000000000000000000000
00000000000000000000000000011111
>Q8ST28_3oci_0_197
MDAPDISYEHQETSVPNRSGIIPTLQNVVATVNLSCKLDLKNIALRARNAEYNPKRFAAVIM
RIREPKTTALIFASGKMVITGAKSEKSSRMAAQRYAKIIHKLGFNATFDDFKIQNIVSSCDIKF
SIRLEGLAYAHSNYCSYEPELFPGLIYRMVKPKIVLLIFVSGKIVLTGAKVRDDIYQAFNNIYP
VLIQHRKA
11111111111111111100000000000000000000000000000000000000000000000000000000000000000
00000000000000000000000000000000000000000000000000000000000000000000000000000000000
00000000000000000000000000000000
>Q96GG9_3tdu_61_258
RKKLEQLYNRYKDPQDENKIGIDGIQQFCDDLALDPASISVLIIAWKFRAATQCEFSKQEFM
DGMTELGCDSIEKLKAQIPKMEQELKEPGRFKDFYQFTFNFAKNPGQKGLDLEMAIAYWN
LVLNGRFKFLDLWNKFLLEHHKRSIPKDTWNLLLDFSTMIADDMSNYDEEGAWPVLIDDF
VEFARPQIAGTKSTTV
00000000000000000000000000000000000000000000000000000000000000000000000000000000000
00000000000000000000000000000000000000000000000000000000000000000000000000000000000
00000000000000000000000000111111
>Q9JMX9_1s2x_1_198
ELGFNEAERQKILDSNSSLMRNANEVRDKFIQNYATSLKDSNDPQDFLRRVQELRINMQKN
FISFDAYYNYLNNLVLASYNRCKQEKTFAESTIKNELTLGEFVAEISDNFNNFTCDEVARISD
LVASYLPREYLPPFIDGNMMGVAFQILGIDDFGKKLNEIVQDIGTKYIILSKNKTYLTSLERAK
LITQLKLNLE
00000000000000000000000000000000000000000000000000000000000000000000000000000000000
00000000000000000000000000000000000000000000000000000000000000000000000000000000000
00000000000011111111111111111111
>Q9QVY3_1f6b_0_197
MSFIFDWIYSGFSSVLQFLGLYKKTGKLVFLGLDNAGKTTLLHMLKDDRLGQHVPTLHPTS
EELTIAGMTFTTFDLGGHIQARRVWKNYLPAINGIVFLVDCADHERLLESKEELDSLMTDET
IANVPILILGNKIDRPEAISEERLREMFGLYGQTTGKGSVSLKELNARPLEVFMCSVLKRQGY
GEGFRWMAQYID
11111111111100000000000000000000000000000000000011111100000000000000000000000000000
00000000000000000000000000000000000000000000000000000000000000000000000000000000000
00000000000000000000000000000000
>P04275_3zqk_1477_1673
VGPGLLGVSTLGPKRNSMVLDVAFVLEGSDKIGEADFNRSKEFMEEVIQRMDVGQDSIHVT
VLQYSYMVTVEYPFSEAQSKGDILQRVREIRYQGGNRTNTGLALRYLSDHSFLVSQGDREQ
APNLVYMVTGNPASDEIKRLPGDIQVVPIGVGPNANVQELERIGWPNAPILIQDFETLPREA
PDLVLQRCCSGEG
11111111111111111100000000000000000000000000000000000000000000000000000000000000000
00000000000000000000000000000000000000000000000000000000000000000000000000000000000
0000000000000000000000000000111
>P71715_2r5u_0_196
MAVVDDLAPGMDSSPPSEDYGRQPPQDLAAEQSVLGGMLLSKDAIADVLERLRPGDFYRP
AHQNVYDAILDLYGRGEPADAVTVAAELDRRGLLRRIGGAPYLHTLISTVPTAANAGYYAS
IVAEKALLRRLVEAGTRVVQYGYAGAEGADVAEVVDRAQAEIYDVADRRLSEDFVALEDLL
QPTMDEIDAIASSGG
11111111111111111111110000000000000000000000000000000000000000000000000000000000000
00000000000000000000000000000000000000000000000000000000000000011100000000000000000
1111111111111111111111111111111
>P96275_3e3u_0_196

MAVVPIRIVGDPVLHTATTPVTVAADGSLPADLAQLIATMYDTMDAANGVGLAANQIGCS
LRLFVYDCAADRAMTARRRGVVINPVLETSEIPETMPDPDTDDEGCLSVPGESFPTGRAKW
ARVTGLDADGSPVSIEGTGLFARMLQHETGHLDGFLYLDRLIGRYARNAKRAVKSHGWGV
PGLSWLPGEDPDPFGH
10000000000000000000000000000000000000000000000000000000000000000000000000000000000
00000000000000000000000000000000000000000000000000000000000000000000000000000000000
0000000000000000000000000000000
>Q28N01_3cjd_0_196
MAGKVEARKAALREKLIDLAEAQIEAEGLASLRARELARQADCAVGAIYTHFQDLNALTL
EVNGRTFARLGAAVGAVVADGQDDHPNERLIAMSHAYLAFAREHPKLWRALFDVEMRSD
GPVPQWYGHAMAQLFSYITTPLAKIFPESDDAELDLMTRTLFSSVHGIVLLGLENRISGVPG
EQLKTMIRLLLEQVGR
11111111000000000000000000000000000000000000000000000000000000000000000000000111110
00000000000000000000000000000000000000000000000000000000000000000000000000000000000
0000000000000000000000000000000
>Q6Q0L5_2c0n_0_196
MRTLFFIPSMGSVRLPLIDFLVKNDIEYVILSRRNHVAVQREIALDMFLEMKDYDTLAFLDED
VVPIEIDFQKVEAKFNEGYDVVCGYYYLKTLRGYSVYRKDWEKEIFDGEVNGCGLGFTFIKR
EFLEKIKRPAFLAFKPIESPHWIGEDVYFFSTHKPRTYALSSLKAYHFIDERLALSPDRKLILQN
DHVARIK
00000000000000000000000000000000000000000000000000000000000000000000000000000000000
00000000000000000000000000000000000000000000000000000001111111110000000000000000000
0000000000000000000000000000000
>Q9BKL4_2c0d_19_215
SLVTKKAYNFTAQGLNKNNEIINVDLSSFIGQKYCCLLFYPLNYTFVCPTEIIEFNKHIKDFEN
KNVELLGISVDSVYSHLAWKNMPIEKGGIGNVEFTLVSDINKDISKNYNVLYDNSFALRGLF
IIDKNGCVRHQTVNDLPIGRNVQEVLRTIDSIIHVDTSGEVCPINWKKGQKAFKPTTESLIDY
MNNANKNV
10000000000000000000000000000000000000000000000000000000000000000000000000000000000
00000000000000000000000000000000000000000000000000000000000000000000000000000000000
0000000000111111111111111111111
>Q9HIL9_1ne2_0_196
MGIKNDLEIRLQKLQQQGNFKNYLEQYPTDASTAAYFLIEIYNDGNIGGRSVIDAGTGNGIL
ACGSYLLGAESVTAFDIDPDAIETAKRNCGGVNFMVADVSEISGKYDTWIMNPPFGSVVKH
SDRAFIDKAFETSMWIYSIGNAKARDFLRREFSARGDVFREEKVYITVPRIYRHHSYDRARIE
AVIFGVRNHSF
00000000000000000011111111000000000000000000000000000000000000000000000000000000000
00000000000000000000000000000000000000000000000000000000000000000000000000000000000
0000000000000000000000000000000
>Q9K9M4_2qml_0_196
MKCNDKLAPFEVFDHVVNKKLSFRHVTMDDVDMLHSWMHEEHVIPYWKLNIPLVDYKK
HLQTFLNDDHQTLMVGAINGVPMSYWESYWVKEDIIANYYPFEEHDQGIHLLIGPQEYLG
QGLIYPLLLAIMQQKFQEPDTNTIVAEPDRRNKKMIHVFKKCGFQPVKEVELPDKIGLLMK
CEQNVFEKRWSDWKMNKF
11110000000000000000000000000000000000000000000000000000000000000000000000000000000
00000000000000000000000000000000000000000000000000000000000000000000000000000000000
0000000000000000000000000000000
>F3YMM1_4ezg_29_224
AAEQTGLKASQDNVNIPDSTFKAYLNGLLGQSSTANITEAQMNSLTYITLANINVTDLTGIE
YAHNIKDLTINNIHATNYNPISGLSNLERLRIMGKDVTSDKIPNLSGLTSLTLLDISHSAHDD
SILTKINTLPKVNSIDLSYNGAITDIMPLKTLPELKSLNIQFDGVHDYRGIEDFPKLNQLYAFS

QTIGGKK
11111000000000000000000000000000000000000000000000000000000000000000000000000000000
00000000000000000000000000000000000000000000000000000000000000000000000000000000000
000000000000000000000000111111
>O53223_4hu2_54_249
ADLLVPKLTASVTDGAVGVTVDAPVSVTAADGVLAAVTMVNDNGRPVAGRLSPDGLRWS
TTEQLGYNRRYTLNATALGLGGAATRQLTFQTSSPAHLTMPYVMPGDGEVVGVGEPVAIRF
DENIADRGAAEKAIKITTNPPVEGAFYWLNNREVRWRPEHFWKPGTAVDVAVNTYGVDL
GEGMFGEDNVQTHFTIG
11000000000000000000000000000000000000000000000000000000000000000000000000000000000
00000000000000000000000000000000000000000000000000000000000000000000000000000000000
000000000000000000000000000000
>O76977_2ptm_469_664
MDSSSRQYREKLKQVEEYMQYRKLPSHLRNKILDYYEYRYRGKMFDERHIFREVSESIRQDV
ANYNCRDLVASVPFFVGADSNFVTRVVTLLEFEVFQPADYVIQEGTFGDRMFFIQQGIVDII
MSDGVIATSLSDGSYFGEICLLTRERRVASVKCETYCTLFSLSVQHFNQVLDEFPAMRKTMEE
IAVRRLTRI
10000000000000000000000000000000000000000000000000000000000000000000000000000000000
00000000000000000000000000000000000000000000000000000000000000000000000000000000000
000000000000000000000000000111
>P0A7I7_2bz1_0_195
MQLKRVAEAKLPTPWGDFLMVGFEELATGHDHVALVYGDISGHTPVLARVHSECLTGDAL
FSLRCDCGFQLEAALTQIAEEGRGILLYHRQEGRNIGLLNKIRAYALQDQGYDTVEANHQL
GFAADERDFTLCADMFKLLGVNEVRLLTNNPKKVEILTEAGINIVERVPLIVGRNPNNEHY
LDTKAEKMGHLLNK
00000000000000000000000000000000000000000000000000000000000000000000000000000000000
00000000000000000000000000000000000000000000000000000000000000000000000000000000000
000000001111111111111111111111
>Q6D668_2hyt_0_195
MVRRTRAEMEETRATLLATARKVFSERGYADTSMDDLTAQASLTRGALYHHFGDKKGLLA
AVVEQIDAEMDERLQAISDTAEDDWEGFRCRCRAYLEMALEPEIQRIVLRDARAVLGGASP
DSQRHCVESMQRLIDNLIRQGVVAEADPQALASLIYGSLAEAAFWIAEGEDGNARLAQGV
AALELLLRGLLVKPR
11100000000000000000000000000000000000000000000000000000000000000000000000000000000
00000000000000000000000000000000000000000000000000000000000000000000000000000000000
000000000000000000000000000000
>Q884H9_2oda_0_195
MPLPTFPALLFGLSGCLVDFGAQAATSDTPDDEHAQLTPGAQNALKALRDQGMPCAWID
ELPEALSTPLAAPVNDWMIAAPRPTAGWPQPDACWMALMALNVSQLEGCVLISGDPRLL
QSGLNAGLWTIGLASCGPLCGLSPSQWQALNNAEREQRRAQATLKLYSLGVHSVIDHLGE
LESCLADIALRRSKGEKP
10000000000000000000000000000000000000000000000000000000000000000000000000000000000
00000000000000000000000000000000000000000000000000000000000000000000000000000000000
000000000000000000000000000000
>Q8A510_4f54_14_209
ASCEKDPDMGKLDDNYLVYTNYDKQANFKDFSTFYLADKILVISDSKEPEYLEGEGAEQIL
AAYTENMEAKGYQPAADKESADLGIQVSYIASTYYFTGYTQPEWWWGYPGYWGPSYWG
NWGGWYYPYAVTYSYSTNSFITEMVNLKADEGEGKKLPVVWTSYLTGFETGSKAINRTLAI
EAVNQSFTQSPYLTNK
11111111111100000000000000000000000000000000000000000000000000000000000000000000000
00000000000000000000000000000000000000000000000000000000000000000000000000000000000

000000000000000000000000000000
>Q9HU79_1yll_0_195
MSELRILRAVDYPRMPWKNGAGSTEEIARDGGDGLDGFGWRLSIADVGESGGFSGFAGYQ
RIISVLEGGGMRLRVDGAESAPLRARQAFAFSGDSEVHCTLLDGAIRDFNLIYAPRRHRARL
QWLRVEGELDWHGTASTLLLFAQQDGVAISLQGQPRGQLAAHDCLCAEGLQGLQHWRL
TAHEPAWVCAVELDSL
10000000000000000000000000000000000000000000000000000000000000000000000000000000000
00000000000000000000000000000000000000000000000000000000000000000000000000000000000
000000000000000000000000000000
>Q9JRY6_2r2a_0_195
MAEICLITGTPGSGKTLKMVSMMANDEMFKPDENGIRRKVFTNIKGLKIPHTYIETDAKKL
PKSTDEQLSAHDMYEWIKKPENIGSIVIVDEAQDVWPARSAGSKIPENVQWLNTHRHQGI
DIFVLTQGPKLLDQNLRTLVRKHYHIASNKMGMRTLLEWKICADDPVKMASSAFSSIYTLD
KKVYDLYESAEVHT
00000000000000000000000000000000000000000000000000000000000000000000000000000000000
00000000000000000000000000000000000000000000000000000000000000000000000000000000000
000000000000000000000000011111
>O06201_3ano_0_194
MSDEDRTDRATEDHTIFDRGVGQRDQLQRLWTPYRMNYLAEAPVKRDPNSSASPAQPFTE
IPQLSDEEGLVVARGKLVYAVLNLYPYNPGHLMVVPYRRVSELEDLTDLESAELMAFTQKAI
RVIKNVSRPHGFNVGLNLGTSAGGSLAEHLHVHVVPRWGGDANFITIIGGSKVIPQLLRDT
RRLLATEWARQP
11111111111110000000000000000000000000000000000000000000000000000000000000000000000
00000000000000000000000000000000000000000000000000000000000000000000000000000000000
00000000000000000000000000000
>P38424_1svi_0_194
MKVTKSEIVISAVKPEQYPEGGLPEIALAGRSNVGKSSFINSLINRKNLARTSSKPGKTQTLNF
YIINDELHFVDVPGYGFAKVSKSEREAWGRMIETYITTREELKAVVQIVDLRHAPSNDDVQ
MYEFLKYYGIPVIVIATKADKIPKGKWDKHAKVVRQTLNIDPEDELILFSSETKKGKDEAWG
AIKKMINR
00000000000000000000000000000000000000000000001111111111111000000000000000000000000
00000000000000000000000000000000000000000000000000000000000000000000000000000000000
00000000000000000000000000000
>Q0P9D1_3bsw_0_194
MARTEKIYIYGASGHGLVCEDVAKNMGYKECIFLDDFKGMKFESTLPKYDFFIAIGNNEIRK
KIYQKISENGFKIVNLIHKSALISPSAIVEENAGILIMPYVVINAKAKIEKGVILNTSSVIEHEC
VIGEFSHVSVGAKCAGNVKIGKNCFLGINSCVLPNLSLADDSILGGGATLVKNQDEKGVFV
GVPAKRM
11000000000110000000000000000000000000000000000000000000000000000000000000000000000
00000000000000000000000000000000000000000000000000000000000000000000000000000000000
00000000000000000000000000000
>Q39YF0_3e05_210_404
LAQYPVIGIDDDEFATAKKLITKQEVRAVTLSKLRLQDDLVMWDIGAGSASVSIEASNLMPN
GRIFALERNPQYLGFIRDNLKKFVARNVTLVEAFAPEGLDDLPDPDRVFIGGSGGMLEEIIDA
VDRRLKSEGVIVLNAVTLDTLTKAVEFLEDHGYMVEVACVNVAKTKGLTEYKMFESHNPV
YIITAWKSDE
11000000000000000000000000000000000000000000000000000000000000000000000000000000000
00000000000000000000000000000000000000000000000000000000000000000000000000000000000
00000000000000000000000000011
>Q54413_2cc0_41_235
AACNGYVGLTFDDGPSGSTQSLLNALRQNGLRATMFNQGQYAAQNPSLVRAQVDAGMW

VANHSYTHPHMTQLGQAQMDSEISRTQQAIAGAGGGTPKLFRPPYGETNATLRSVEAKYG
LTEVIWDVDSQDWNNASTDAIVQAVSRLGNGQVILMHDWPANTLAAIPRIAQTLAGKGL
CSGMISPQTGRAVAPDGS
00000000000000000000000000000000000000000000000000000000000000000000000000000000000
00000000000000000000000000000000000000000000000000000000000000000000000000000000000
00000000000000000000000000011
>Q7D028_2gz4_0_194
MLSGRRLDLLDPSPLDVEIADIAHGLARVARWNGQTRGDHAFTVAQHCLIVETIFCRMCP
GATPDEMQMALLHDAPEYVIGDMISPFKSVVGGGYKTVEKRLEAAVHLRFGLPPHASREL
KDRIKKADTVAAFFEATELAGFSTAEAQKFFGLPRGITRDMFDIIPLPSTEAQRLFIARFEAIE
TLRVTRTGGAV
00000000000000000000000000000000000000000000000000000000000000000000000000000000000
00000000000000000000000000000000000000000000000000000000000000000000000000000000000
00000000000000000000000000111
>Q8TLY9_2veb_0_194
MSVEKIPGYTYGETENRAPFNLEDLKLLKEAVMFTAEDEEYIQKAGEVLEDQVEEILDTWY
GFVGSHPHLLYYFTSPDGTPNEKYLAAVRKRFSRWILDTCNRSYDQAWLDYQYEIGLRHHR
TKKNQTDNVESVPNIGYRYLVAFIYPITATMKPFLARKGHTPEEVEKMYQAWFKATTLQVA
LWSYPYVKYGDF
11111000000000000000000000000000000000000000000000000000000000000000000000000000000
00000000000000000000000000000000000000000000000000000000000000000000000000000000000
00000000000000000000000000000
>Q93ZE8_3mal_23_217
SASAAASGKEGVEITYGSAIKLMHEKTKFRLHSHDVPYGSGSGQQSVTGFPGVVDSNSYWI
VKPVPGTTEKQGDAVKSGATIRLQHMKTRKWLHSHLHASPISGNLEVSCFGDDTNSDTGD
HWKLIIEGSGKTWKQDQRVRLQHIDTSGYLHSHDKKYQRIAGGQQEVCGIREKKADNIWL
AAEGVYLPLNESSK
11111111111000000000000000000000000000000000000000000000000000000000000000000000000
00000000000000000000000000000000000000000000000000000000000000000000000000000000000
00000000000000000000000000111
>Q96DE0_3cou_0_194
MAGARRLELGEALALGSGWRHACHALLYAPDPGMLFGRIPLRYAILMQMRFDGRLGFPG
GFVDTQDRSLEDGLNRELREELGEAAAAFRVERTDYRSSHVGSGPRVVAHFYAKRLTLEEL
LAVEAGATRAKDHGLEVLGLVRVPLYTLRDGVGGLPTFLENSFIGSAREQLLEALQDLGLL
QSGSISGLKIPAHH
11000000000000000000000000000000000000000000000000000000000000000000000000000000000
00000000000000000000000000000000000000000000000000000000000000000000000000000000000
00000000000000011111111111111
>Q96T58_1ow1_3469_3663
GLVLPHTEFQPAPKQDSSPHLTSQRPVDMVQLLKKYPIVWQGLLALKNDTAAVQLHFVSG
NNVLAHRSLPLSEGGPPLRIAQRMRLEATQLEGVARRMTVETDYCLLLALPCGRDQEDVV
SQTESLKAAFITYLQAKQAAGIINVPNPGSNQPAYVLQIFPPCEFSESHLSRLAPDLLASISNIS
PHLMIVIASV
11111111111111111111111110000000000000000000000000000000000000111000000000000000000
00000000000000000000000000000000000000000000000000000000000000000000000000000000000
00000000000000000000000000000
>Q9RW27_1w3q_0_194
MSDFYDPRERDPSVSRRPQNRQSDEWIRELLLRGTIARVATLWQGEDGAAFPFITPLAYAYR
PEQGDLVYHTNVVGRLRANAGQGHPATLEVSEIGQFLPSNSPLELSVQYRSVMVFGTARVL
AGEDARAALTTLSERVFPGLKVGETTRPISEDDLKRTSVYSLSIDRWSGKENWAEQAIQEED
WPALGPEWLG

10000000000000000000000000000000000000000000000000000000000000000000000000000000000
00000000000000000000000000000000000000000000000000000000000000000000000000000000000
00000000000000000000000000000
>Q9WY88_3dcz_29_223
KGKIQEADNAAKLSAIKFVLKDPLTGDYLVDEKEIEEIVKKTGIETVVLKEYKEGVVLGPLYE
FVTKDGRNAYVLSGYAPGFGGNVTVVACFIKTEDGFMLNSVRVIDYSQETPGLGAKIGEESI
QRRFFPVPPEGLKNGLRVDKDAGLPKGSPEELKKQGIVKVSDVMTGATITPRAVVTALNLM
YRYLEEVSK
11111110000000000000000000000000000000000000000000000000000000000000000000000000001
00000000000000000000000000001111111111100000000000000000000000000000000000000000000
00111110000000000000000000000
>A1U0I5_3pas_0_193
MRQRDDSKRIAFLEATVREVADHGFSATSVGKIAKAAGLSPATLYIYYEDKEQLLLATFYYVS
DQVIDAALDSFSRGKDLREGLRRQWHTLFRIGLERPELFRYHETFTHSAWMTPEIQARNESR
AANLLNAVDQGKQSGLIKPVPFPLLETFMFRPIYHLVQRCLQGSFEGTDEHIELAFNMAWD
AVADRRNT
00000000000000000000000000000000000000000000000000000000000000000000000000000000000
00000000000000000000000000000000000000000000000000000000000000000000000000000000000
0000000000000000000000000111
>O28741_2i5h_1_194
EKPKVERSEGKEKLEDYAYVLDFMPYGHPDDKRPIHRREPLAQVVGERNFTLLEVSIRKGK
QPLVMDRVYIGKGERDVVYKIKRRLRYEDLTPAAKTELPYVIEHIIKQDEKKYVDFFNKADSI
TTRMHQLELLPGVGKKMMWAIIEERKKRPFESFEDIAQRVKGIQRPEKLIVSRIIYEIKNPQT
KYKLFTA
11111111111111000000000000000000000000000000000000000000000000000000000000000000000
00000000000000000000000000001100000000000000000000000000000000000000000000000000000
0000000000000000000000000000
>O95750_2p23_22_215
RPLAFSDAGPHVHYGWGDPIRLRHLYTSGPHGLSSCFLRIRADGVVDCARGQSAHSLLEIK
AVALRTVAIKGVHSVRYLCMGADGKMQGLLQYSEEDCAFEEEIRPDGYNVYRSEKHRLPVS
LSSAKQRQLYKNRGFLPLSHFLPMLPMVPEEPEDLRGHLESDMFSSPLETDSMDPFGLVTGL
EAVRSPSFEK
11111111111111111000000000000000000000000000000000000000000000000000000000000000000
00000000000000000000000000000000000000000000000000000000000000000000001111111111111
1111111111111111111111111111
>P05455_1zh5_0_193
MAENGDNEKMAALEAKICHQIEYYFGDFNLPRDKFLKEQIKLDEGWVPLEIMIKFNRLNR
LTTDFNVIVEALSKSKAELMEISEDKTKIRRSPSKPLPEVTDEYKNDVKNRSVYIKGFPTDATL
DDIKEWLEDKGQVLNIQMRRTLHKAFKGSIFVVFDSIESAKKFVETPGQKYKETDLLILFKD
DYFAKKNE
11110000000000000000000000000000000000000000000000000000000000000000000000000000000
00000000000000000000000000000000000000000000000000000000000000000000000000000000000
0000000000000000000000011111
>P0A729_4jhc_0_193
MPKLILASTSPWRRALLEKLQISFECAAPEVDETPRSDESPRQLVLRLAQEKAQSLASRYPDH
LIIGSDQVCVLDGEITGKPLTEENARLQLRKASGNIVTFYTGLALFNSANGHLQTEVEPFDV
HFRHLSEAEIDNYVRKEHPLHCAGSFKSEGFGITLFERLEGRDPNTLVGLPLIALCQMLRRE
GKNPLMG
10000000000000000000000000000000000000000000000000000000000000000000000000000000000
00000000000000000000000000000000000000000000000000000000000000000000000000000000000
0000000000000000000000000000

>P16686_2fsu_0_193
MTLETAFMLPVQDAQHSFRRLLKAMSEPGVIVALHQLKRGWQPLNIATTSVLLTLADNDT
PVWLSTPLNNDIVNQSLRFHTNAPLVSQPEQATFAVTDEAISSEQLNALSTGTAVAPEAGAT
LILQVASLSGGRMLRLTGAGIAEERMIAPQLPECILHELTERPHPFPLGIDLILTCGERLLAIPR
TTHVEVC
11111111111000000000000000000000000000000000000000000000000000000000000000000000000
00000000000000000000000000011111111100000000000000000000011110000000000000000000000
0000000000000000000000000000
>P32173_1h4d_0_193
MNLMTTITGVVLAGGKARRMGGVDKGLLELNGKPLWQHVADALMTQLSHVVVNANRH
QEIYQASGLKVIEDSLADYPGPLAGMLSVMQQEAGEWFLFCPCDTPYIPPDLAARLNHQR
KDAPVVWVHDGERDHPTIALVNRAIEPLLLEYLQAGERRVMVFMRLAGGHAVDFSDHKD
AFVNVNTPEELARWQEKR
11100000000000000000000000000000000000000000000000000000000000000000000000000000000
00000000000000000000000000000000000000000000000000000000000000000000000000000000000
0000000000000000000000000111
>Q5KZG6_2ywi_0_193
MEERVLGMPAVESNMFPLGKQAPPFALTNVIDGNVVRLEDVKSDAATVIMFICNHCPFVK
HVQHELVRLANDYMPKGVSFVAINSNDAEQYPEDSPENMKKVAEELGYPFPYLYDETQEV
AKAYDAACTPDFYIFDRDLKCVYRGQLDDSRPNNGIPVTGESIRAALDALLEGRPVPEKQK
PSIGCSIKWKPSA
11110000000000000000000000000000000000000000000000000000000000000000000000000000000
00000000000000000000000000000000000000000000000000000000000000000000000000000000000
0000000000000000000000000000
>Q7UTD8_3b5m_1_194
ILESLVTTLDEQGRINLAPLGPIVLPPQSPGGLPQFLLRPYEGSTTCDNLLASGNAVIHVIDDA
LLIAKTAIGKVDASDLVVPIPGLEDTHVRLKRCHRWFAVRVTQRAGTPPRHELTARCLASGL
VDPFFGFNRAKHAVIEAAVAATRLHLLPPEEIEEELERARIAIEKTGGEPEREALQLIRRHVRE
SSIS
00000000000000000000000000000000000000000000000000000000000000000000000000000000000
00000000000000000000000000000000000000000000000000000000000000000000000000000000000
0000000000000000000000000001
>Q9EVJ6_3frq_0_193
MPRPKLKSDDEVLEAATVVLKRCGPIEFTLSGVAKEVGLSRAALIQRFTNRDTLLVRMMER
GVEQVRHYLNAIPIGAGPQGLWEFLQVLVRSMNTRNDFSVNYLISWYELQVPELRTLAIQR
NRAVVEGIRKRLPPGAPAAAELLLHSVIAGATMQWAVDPDGELADHVLAQIAAILCLMFP
EHDDFQLLQAHA
11110000000000000000000000000000000000000000000000000000000000000000000000000000000
00000000000000000000000000000000000000000000000000000000000000000000000000000000000
0000000000000000000000011111
>Q9FAC4_1v2x_0_193
MRERTEARRRRIEEVLRRRQPDLTVLLENVHKPHNLSAILRTCDAVGVLEAHAVNPTGGVP
TFNETSGGSHKWVYLRVHPDLHEAFRFLKERGFTVYATALREDARDFREVDYTKPTAVLFG
AEKWGVSEEALALADGAIKIPMLGMVQSLNVSVAAAVILFEAQRQRLKAGLYDRPRLDPE
LYQKVLADWLRK
00000000000000000000000000000000000000000000000000000000000000000000000000000000000
00000000000000000000000000000000000000000000000000000000000000000000000000000000000
0000000000000000000000000111
>Q9RV46_1nqz_0_193
MTAPHDPLDDIQADPWALWLSGRTRTALELPHYRRAAVLVALTREADPRVLLTVRSSELPT
HKGQIAFPGGSLDAGETPTQAALREAQEEVALDPAAVTLLGELDDVFTPVGFHVTPVLGRI

APEALDTLRVTPEVAQIITPTLAELRAVPLVRERRTLPDGTEVPLYRYPWRGLDIWGMTARV
LHDLLEQGPG
11100000000000000000001111111111000000000000000000000000111111000000000000000000000
00000000000000000000000000000000000000000000000000000000000000000000000000000000000
0000000000000000000000001111
>O32218_3gha_29_221
NNKTEQGNDAVSGQPSIKGQPVLGKDDAPVTVVEFGDYKCPSCKVFNSDIFPKIQKDFIDK
GDVKFSFVNVMFHGKGSRLAALASEEVWKEDPDSFWDFHEKLFEKQPDTEQEWVTPGLL
GDLAKSTTKIKPETLKENLDKETFASQVEKDSDLNQKMNIQATPTIYVNDKVIKNFADYDEI
KETIEKELKGK
11111100000000000000000000000000000000000000000000000000000000000000000000000000000
00000000000000000000000000000000000000000000000000000000000000000000000000000000000
000000000000000000000000000
>P51965_3bzh_0_192
MSDDDSRASTSSSSSSSSNQQTEKETNTPKKKESKVSMSKNSKLLSTSAKRIQKELADITLDPP
PNCSAGPKGDNIYEWRSTILGPPGSVYEGGVFFLDITFTPEYPFKPPKVTFRTRIYHCNINSQ
GVICLDILKDNWSPALTISKVLLSICSLLTDCNPADPLVGSIATQYMTNRAEHDRMARQWTK
RYAT
11111111111111111111000000011111111111100000000000000000000000000000000000000000000
00000000000000000000000000000000000000000000000000000000000000000000000000000000000
000000000000000000000000000
>P56427_3s0y_64_256
MQPVVVIPPDSGKEEEQMASESSKPASQNTETKATIARKGEGSVLEQIDQGSILKLPSNLLFE
NATSDAINQDMMLYIERIAKIIQKLPKRVHINVRGFTDDTPLVKTRFKSHYELAANRAYRVM
KVLIQYGVNPNQLSFSSYGSTNPIAPNDSLENRMKNNRVEIFFSTDANDLSKIHSILDNEFNP
HKQQE
00000000000000000000000000000000000000000000000000000000000000000000000000000000000
00000000000000000000000000000000000000000000000000000000000000000000000000000000000
000000000000000000000001111
>P94548_1vi0_1_193
KQKRPKYMQIIDAAVEVIAENGYHQSQVSKIAKQAGVADGTIYLYFKNKEDILISLFKEKMG
QFIERMEEDIKEKATAKEKLALVISKHFSLLAGDHNLAIVTQLELRQSNLELRQKINEILKGY
LNILDGILTEGIQSGEIKEGLDVRLARQMIFGTIDETVTTWVMNDQKYDLVALSNSVLELLVS
GIHNK
11110000000000000000000000000000000000000000000000000000000000000000000000000000000
00000000000000000000000000000000000000000000000000000000000000000000000000000000000
000000000000000000000000000
>Q2RIF7_3zt9_0_192
MEKLEVGIYTRAREGEIACGDACLVKRVEGVIFLAVGDGIGHGPEAARAAEIAIASMESSM
NTGLVNIFQLCHRELRGTRGAVAALCRVDRRQGLWQAAIVGNIHVKILSAKGIITPLATPGI
LGYNYPHQLLIAKGSYQEGDLFLIHSDGIQEGAVPLALLANYRLTAEELVRLIGEKYGRRDD
DVAVIVAR
10000000000000000000000000000000000000000000000000000000000000000000000000000000000
00000000000000000000000000000000000000000000000000000000000000000000000000000000000
000000000000000000000000000
>Q5ZXA4_4fgo_51_243
STQAAPLISVEKIQKLAQSYQGDTRKRFTAWGNLIDSLKKKPVKIQLEKVNSFFNQFNYETD
PITGASDDYWKSPVEFIVDGGGDCEDFAIIKYFTLVAVGVPSDQLRITYAASLTLNQAHMVLS
FYPTPESEPLILDSLESKILKASARPDLKPVYSFNAEGLWLAKTRGESSLMGDSKSLGKWDAL
MKRME
11111100000000000000000000000000000000000000000000000000000000000000000000000000000

00000000000000000000000000000000000000000000000000000000000000000000000000000000000
000000000111110000000000000
>Q82Y41_2g84_0_192
MNDALHIGLPPFLVQANNEPRVLAAPEARMGYVLELVRANIAADGGPFAAAVFERDSGLL
IAAGTNRVVPGRCSAAHAEILALSLAQAKLDTHDLSADGLPACELVTSAEPCVMCFGAVI
WSGVRSLVCAARSDDVEAIGFDEGPRPENWMGGLEARGITVTTGLLRDAACALLREYNAC
NGVIYNARCGVHK
00000000000000000000000000000000000000000000000000000000000000000000000000000000000
00000000000000000000000000000000000000000000000000000000000000000000000000000000000
000000000000000000000001111
>Q92913_3hbw_52_244
GSKKRRRRRPEPQLKGIVTKLYSRQGYHLQLQADGTIDGTKDEDSTYTLFNLIPVGLRVVAI
QGVQTKLYLAMNSEGYLYTSELFTPECKFKESVFENYYVTYSSMIYRQQQSGRGWYLGLNK
EGEIMKGNHVKKNKPAAHFLPKPLKVAMYKEPSLHDLTEFSRSGSGTPTKSRSVSGVLNGG
KSMSHNEST
11111111111000000000000000000000000000000000000000000000000000000000000000000000000
00000000000000000000000000000000000000000000000000000000000000000000000000000111111
111111111111111111111111111
>Q9BQ65_4h7w_72_264
TFPHERGNWATHVYVPYEAKEEFLDLLDVLLPHAQTYVPRLVRMKVFHLSLSQSVVLRHH
WILPFVQALKARMTSFHRFFFTANQVKIYTNQEKTRTFIGLEVTSGHAQFLDLVSEVDRVME
EFNLTTFYQDPSFHLSLAWCVGDARLQLEGQCLQELQAIVDGFEDAEVLLRVHTEQVRCKS
GNKFFSMPLK
11111100000000000000000000000000000000000000000000000000000000000000000000000000000
00000000000000000000000000000000000000000000000000000000000000000000000000000000000
000000000000000000000000000
>Q9I2Q9_2gen_0_192
MGRSSRKDEILQAALACFSEHGVDATTIEMIRDRSGASIGSLYHHFGNKERIHGELYLAGIG
QYAALLEAGFARARSAEETVRLLVTSYIDWVVANPDWARFILHSRGRVEAGELGERLRADN
QAHFARIHAALAGYRAEGLFREMPDDCFASVVIGPAHDLARQWLAGRTRVALADCRELL
AQVAWDSVRAA
11111000000000000000000000000000000000000000000000000000000000000000000000000000000
00000000000000000000000000000000000000000000000000000000000000000000000000000000000
000000000000000000000000000
>A9CKG4_2g7s_0_191
MKNPQSKADDILQCARTLIIRGGYNSFSYADISQVVGIRNASIHHHFPSKSDLVCKLVSQYR
QEAEAGIAELEKNISDPLEQLRAYIGYWEGCIADATHPFCVCALLASEIPVLPETVVLEVRA
HFRSLSDWLTAVLERGIAQGRLVLTGTARANAEIFMATVHGAMLSARAHGDAATFGAITRP
MLERITA
11000000000000000000000000000000000000000000000000000000000000000000000000000000000
00000000000000000000000000000000000000000000000000000000000000000000000000000000000
00000000000000000000000000
>A9WDG5_2rhm_0_191
MQTPALIIVTGHPATGKTTLSQALATGLRLPLLSKDAFKEVMFDGLGWSDREWSRRVGATA
IMMLYHTAATILQSGQSLIMESNFRVDLDTERMQNLHTIAPFTPIQIRCVASGDVLVERILSRI
AQGARHPGHCDDRSPADLELVRSRGDIPPLPLGGPLLTVDTTFPEQIDMNAIVQWVRQHL
QSGTAFL
00000000000000000000000000000000000000000000000000000000000000000000000000000000000
00000000000000000000000000000000000000000000000000000000000000000000000000000000000
00000000000000000000000111
>O32079_1oru_0_191

MWKRMTAKAEGLYIADTKSFVTKQMDKLDFDYGGIPGDLHFGLTKKAGAREPMFSRGTEI
FNRRQISIVSIEECNEIALKMGVPRILPEWLGANVAVSGMPDLTSLKEGSRIIFPSGAALLCEG
ENDPCIQPGEVIQSYYPDQPKLASAFVRHALGIRGIVCIVERPGAVYTGDEIEVHSYQRKVKR
KAERV
00000000000000000000000000000000000000000000000000000000000000000000000000000000000
00000000000000000000000000000000000000000000000000000000000000000000000000000000000
00000000000000001111111111
>O94444_2x5n_1_192
VLEATMILIDNSEWMINGDYIPTRFEAQKDTVHMIFNQKINDNPENMCGLMTIGDNSPQV
LSTLTRDYGKFLSAMHDLPVRGNAKFGDGIQIAQLALKHRENKIQRQRIVAFVGSPIVEDEK
NLIRLAKRMKKNNVAIDIIHIGELQNESALQHFIDAANSSDSCHLVSIPPSPQLLSDLVNQSPI
GQGVVA
00000000000000000000000000000000000000000000000000000000000000000000000000000000000
00000000000000000000000000000000000000000000000000000000000000111000000000000000000
00000000000000000000000000
>P04224_1fne_25_216
IKEEHTIIQAEFYLLPDKRGEFMFDFDGDEIFHVDIEKSETIWRLEEFAKFASFEAQGALANIA
VDKANLDVMKERSNNTPDANVAPEVTVLSRSPVNLGEPNILICFIDKFSPPVVNVTWLRNG
RPVTEGVSETVFLPRDDHLFRKFHYLTFLPSTDDFYDCEVDHWGLEEPLRKHWEFEEKTLLP
ETKEN
00000000000000000000000000000000000000000000000000000000000000000000000000000000000
00000000000000000000000000000000000000000000000000000000000000000000000000000000000
00000000000000001111111111
>P43522_1v3y_0_191
MVYPIRLYGDPVLRRKARPVEDFSGIKRLAEDMLETMFEAKGVGLAAPQIGLSQRLFVAVE
YADEPEGEEERPLRELVRRVYVVANPVITYREGLVEGTEGCLSLPGLYSEEVPRAERIRVEYQD
EEGRGRVLELEGYMARVFQHEIDHLDGILFFERLPKPKREAFLEANRAELVRFQKEARALLK
ELSQG
00000000000000000000000000000000000000000000000000000000000000111111111110000000000
00000000000000000000000000000000000000000000000000000000000000000000000000000000000
00000000000000000111111111
>Q15126_3ch4_0_191
MAPLGGAPRLVLLFSGKRKSGKDFVTEALQSRLGADVCAVLRLSGPLKEQYAQEHGLNFQ
RLLDTSTYKEAFRKDMIRWGEEKRQADPGFFCRKIVEGISQPIWLVSDTRRVSDIQWFREAY
GAVTQTVRVVALEQSRQQRGWVFTPGVDDAESECGLDNFGDFDWVIENHGVEQRLEEQL
ENLIEFIRSRL
00000000000000000000000000000000000000000000000000000000000011111110000000000000000
00000000000000000000000000000000000000000000000000000000000000000000000000000000000
00000000000000000000000000
>Q7WJ28_3byq_0_191
MSLIEIRKRTLIVETTYHENGPAPAQPLKLAASCAVIRNPYAGRYEPDLMPFMAELRSLGTLL
ATELVDTLGKDNIEVYSKAAIVGVDGEMEHGAVWHEAGGWAMRSVLGEPKAMVPAVKA
VATAGYRMMVPVHYIHASYVRSHFNSIEIGIQDAPRPREILFALVMGTGARVHARLGGLTKE
AVSVHDGQR
10000000000000000000000000000000000000000000000000000000000000000000000000000000000
00000000000000000000000000000000000000000000000000000000000000000000000000000000000
00000000000000000000000000
>Q81BJ4_2fq4_0_191
MQSKRGRPRNIETQKAILSASYELLLESGFKAVTVDKIAERAKVSKATIYKWWPNKAAVVM
DGFLSAAAARLPVPDTGSALNDILIHATSLANFLISREGTIINELVGEGQFDSKLAEEYRVRYF
QPRRLQAKQLLEKGIKRGELKENLDIELSIDLIYGPIFYRLLVTGEKLDDSYVHDLVINAFEGI

RLR
11111111000000000000000000000000000000000000000000000000000000000000000000000000000
00000000000000000000000000000000000000000000000000000000000000000000000000000000000
00000000000000000000000000
>A2I2W2_3h8t_25_215
DEPNQPSTPEAVTKTVTIDASKYETWQYFSFSKGEVVNVTDYKNDLNWDMALHRYDVRL
NCGESGKGKGGAVFSGKTEMDQATTVPTDGYTVDVLGRITVKYEMGPDGHQMEYEEQGF
SEVITGKKNAQGFASGGWLEFSHGPAGPTYKLSKRVFFVRGADGNIAKVQFTDYQDAELK
KGVITFTYTYPVK
11111111100000000000000000000000000000000000000000000000000000000000000000000000000
00000000000000000000000000000000000000000000000000000000000000000000000000000000000
0000000000000000000000000
>C4M4W4_3ref_0_190
MLAFSDMNTGAGKIENGKKALKIVVVGDGAVGKTCLLLAFSKGEIPTAYVPTVFENFSHV
MKYKNEEFILHLWDTAGQEEYDRLRPLSYADSDVVLLCFAVNNRTSFDNISTKWEPEIKHYI
DTAKTVLVGLKVDLRKDGSDDVTKQEGDDLCQKLGCVAYIEASSVAKIGLNEVFEKSVDCI
FSNKPVPK
11111111111000000000000000000000000000000000000000000000000000000000000000000000000
00000000000000000000000000000000000000000000000000000000000000000000000000000000000
0000000000000000000000000
>O76764_2ccm_0_190
MAAHQLSDFQRNKILRVFNTFYDCNHDGVIEWDDFELAIKKICNLHSWPTDGKKHNEAR
ATLKLIWDGLRKYADENEDEQVTKEEWLKMWAECVKSVEKGESLPEWLTKYMNFMFDV
NDTSGDNIIDKHEYSTVYMSYGIPKSDCDAAFDTLSDGGKTMVTREIFARLWTEYFVSNDR
GAKGNHLFGTLKL
10000000000000000000000000000000000000000000000000000000000000000000000000000000000
00000000000000000000000000000000000000000000000000000000000000000000000000000000000
0000000000000000000000000
>P24931_1lko_0_190
MKSLKGSRTEKNILTAFAGESQARNRYNYFGGQAKKDGFVQISDIFAETADQEREHAKRLF
KFLEGGDLEIVAAFPAGIIADTHANLIASAAGEHHEYTEMYPSFARIAREEGYEEIARVFASIA
VAEEFHEKRFLDFARNIKEGRVFLREQATKWRCRNCGYVHEGTGAPELCPACAHPKAHFE
LLGINW
10000000000000000000000000000000000000000000000000000000000000000000000000000000000
00000000000000000000000000000000000000000000000000000000000000000000000000000000000
0000000000000000000000000
>P50591_1dg6_90_280
MILRTSEETISTVQEKQQNISPLVRERGPQRVAAHITGTRGRSNTLSSPNSKNEKALGRKINS
WESSRSGHSFLSNLHLRNGELVIHEKGFYYIYSQTYFRFQEEIKENTKNDKQMVQYIYKYTSY
PDPILLMKSARNSCWSKDAEYGLYSIYQGGIFELKENDRIFVSVTNEHLIDMDHEASFFGAF
LVG
11111111111111111111111111111000000000001111111111100000000000000000000000000000000
00000000000000000000011000000000000000000000000000000000000000000000000000000000000
0000000000000000000000000
>P63228_3l1v_0_190
MAKSVPAIFLDRDGTINVDHGYVHEIDNFEFIDGVIDAMRELKKMGFALVVVTNQSGIARG
KFTEAQFETLTEWMDWSLADRDVDLDGIYYCPHHPQGSVEEFRQVCDCRKPHPGMLLSA
RDYLHIDMAASYMVGDKLEDMQAAVAANVGTKVLVRTGKPITPEAENAADWVLNSLAD
LPQAIKKQQKPAQ
11100000000000000000000000000000000000000000000000000000000000000000000000000000000
00000000000000000000000000000000000000000000000000000000000000000000000000000000000

0000000000000000000111111
>Q11TQ6_3dn7_0_190
MHTALINHIRKFIFLTDEDAGTLSAFFQLKKVRKKETLLKTGEICRINYFVVKGCLRLFFIDEK
GIEQTTQFAIENWWLSDYMAFQKQQPADFYIQSVENCELLSITYTEQENLFERIPALERYFRL
VYQKSFAAAQLRSKFQHMYSKEEQYHNFSSRFPEFIQRVPQYLLASYLGFTPEYLSEIRKKYIS
00000000000000000000000000000000000000000000000000000000000000000000000000000000000
00000000000000000000000000000000000000000000000000000000000000011111111111111111111
1111111111111111111111111
>Q5N5L5_4dr9_1_191
TAAVAIRVAKKKLAKPPLDLHYLGDRVLRQPAKRVSRIDDELRQTIRQMLQTMYSADGIGL
AAPQVGINKQLIVIDLELEDEQAPPLVLINPKIERTAGDLEQCQEGCLSIPGVYLDVERPEIVE
VSYKDENGRPQRLVADGLLARCIQHEMDHLNGVLFVDRVENRLELNEALDKKGFAVQAV
RPVAAAS
11100000000000000000000000000000000000000000000000000000000000000000000000000000000
00000000000000000000000000000000000000000000000000000000000000000000000000000000000
0000000000000000000001111
>Q5SJF5_2cve_0_190
MSLTLADKVVYEEEIQKSRFIAKAAPVASEEEALAFLAENREPEATHNGHAYKIGLLYRFSD
DGEPSGTAGRPILHAIEAQGLDRVAVLVVRYFGGVKLGAGGLVRAYGGVAAEALRRAPKVP
LVERVGLAFLVPFAEVGRVYALLEARALKAEETYTPEGVRFALLLPKPEREGFLRALLDATR
GQVALE
10000000000000000000000000000000000000000000000000000000000000000000000000000000000
00000000000000000000000000000000000000000000000000000000000000000000000000000000000
0000000000000000000000000
>Q5SWZ9_4ggj_30_220
LAGRRPRREVLFFPSQVTCTEALLQAPGLPPGPSGCPCSLPHSESSLSRLLRALLAARSSLELC
LFAFSSPQLGRAVQLLHQRGVRVRVITDCDYMALNGSQIGLLRKAGIQVRHDQDLGYMHH
KFAIVDKKVLITGSLNWTTQAIQNNRENVLIMEDTEYVRLFLEEFERIWEEFDPTKYSFFPQK
HRGH
11110000000000000000000000001111111000000000000000000000000000000000000000000000000
00000000000001110000000000000000000000000000000000000000000000000000000000000000000
0000000000000111111111111
>Q81LX0_2jcb_1_191
REEKLRLRKQIIEHMNSLSKERYTTLSEQIVFSLYEQKEWAEAKTIGITLSMENEVNTYPIIEKA
WKEGKRVVVPKCNKETRTMSFRQISNFDQLETVYMNLREPIPALTEEVNADEIDLQIVPGV
AYTERGERIGYGGGYYDRYLVHYKGKTLSLAYSFQMVEHIPVEPFDKNVEKIITEKGTMVKN
GLV
00000000000000000000000000000000000000000000000000000000000000000000000000000000000
00000000000000000000000000000000000000000000000000000000000000000000000000000000000
0000000000000000000000111
>Q9KY22_3ibz_0_190
MGVSLSKGGNVSLTKEAPGLTAVIVGLGWDIRTTTGTDFDLDASALLLNSGGKVASDAHFI
FFNNLKSPDGSVEHTGDNITGEGEGDDEQIKINLATVPADIEKIVFPVSIYDAENRQQSFGQ
VRNAFIRVVNQAGEAEIARYDLSEDASTETAMVFGELYRHGAEWKFRAIGQGYASGLRGIA
QDFGVNV
11111111111100001100000000000000000000000000000000000000000000000000000000000000000
00000000000000000000000000000000000000000000000000000000000000000000000000000000000
0000000000000000000000000
>A6TE93_3dza_30_219
ATDSATAAPAAAATTQVQKEAADVLQVAVQGANAMRDIQFARLALFHGQPDSAKKLTDD
AAALLAADDASWAKFVKTDAKAKMIADRYVIINASIALSEDYVATPEKESAIQSANEKLAK

GDQKGAIDTLRLAGIGVIENQYLMPLNQTRKAVAQSQELLKAGKYYEANLVLKGAEEGIV
VDSEMLVAGN
11111110000000000000000000000000000000000000000000000000000000000000000000000000000
00000000000000000000000000000000000000000000000000000000000000000000000000000000000
000000000000000000000111
>O57939_2cxx_26_215
MATIIFAGRSNVGKSTLIYRLTGKKVRRGKRPGVTRKIIEIEWKNHKIIDMPGFGFMMGLPKE
VQERIKDEIVHFIEDNAKNIDVAVLVVDGKAAPEIIKRWEKRGEIPIDVEFYQFLRELDIPTIV
AVNKLDKIKNVQEVINFLAEKFEVPLSEIDKVFIPISAKFGDNIERLKNRIFEVIRERQGRRV
10000000000000000000000000000001111100000000000000000000000000000000000000000000000
00000000000000000000000000000000000000000000000000000000000000000000000000000000000
000000000000000000011111
>O60573_2jgb_44_233
KAVVPGPAEHPLQYNYTFWYSRRTPGRPTSSQSYEQNIKQIGTFASVEQFWRFYSHMVRPG
DLTGHSDFHLFKEGIKPMWEDDANKNGGKWIIRLRKGLASRCWENLILAMLGEQFMVGE
EICGAVVSVRFQEDIISIWNKTASDQATTARIRDTLRRVLNLPPNTIMEYKTHTDSIKMPGRL
GPQRLLF
00000000000000000000000000000000000000000000000000000000000000000000000000000000000
00000000000000000000000000000000000000000000000000000000000000000000000000000000000
000000000000000111000000
>O80992_3kl1_0_189
MSSSPAVKGLTDEEQKTLEPVIKTYHQFEPDPTTCTSLITQRIHAPASVVWPLIRRFDNPERYK
HFVKRCRLISGDGDVGSVREVTVISGLPASTSTERLEFVDDDHRVLSFRVVGGEHRLKNYKS
VTSVNEFLNQDSGKVYTVVLESYTVDIPEGNTEEDTKMFVDTVVKLNLQKLGVAATSAPM
HDDE
11111111000000000000000000000000000000000000000000000000000000000000000000000000000
00000000000000000000000000000000000000000000000000001000000000000000000000000000000
000000000000000000001111
>O95147_2wgp_1_190
SSRGHSTLPRTLMAPRMISEGDIGGIAQITSSLFLGRGSVASNRHLLQARGITCIVNATIEIPNF
NWPQFEYVKVPLADMPHAPIGLYFDTVADKIHSVSRKHGATLVHCAAGVSRSATLCIAYL
MKFHNVCLLEAYNWVKARRPVIRPNVGFWRQLIDYERQLFGKSTVKMVQTPYGIVPDVYE
KESRH
11111111111111111111110000000000000000000000000000000000000000000000000000000000000
00000000000000000000000000000000000000000000000000000000000000000000000000000000000
000000000000000000000000
>P14598_1uec_150_339
DITGPIILQTYRAIANYEKTSGSEMALSTGDVVEVVEKSESGWWFCQMKAKRGWIPASFLEP
LDSPDETEDPEPNYAGEPYVAIKAYTAVEGDEVSLLEGEAVEVIHKLLDGWWVIRKDDVTG
YFPSMYLQKSGQDVSQAQRQIKRGAPPRRSSIRNAHSIHQRSRKRLSQDAYRRNSVRFLQQ
RRRQAR
11111000000000000011100000000000000000000000000000000000000000000000000000000000000
00000000000000000000000000000000000000000000000000000000000000000000000000000000000
000000000000000000001111
>Q2FZ56_4a0z_0_189
MRGETLKLKKDKRREAIRQQIDSNPFITDHELSDLFQVSIQTIRLDRTYLNIPELRKRIKLVAE
KNYDQISSIEEQEFIGDLIQVNPNVKAQSILDITSDSVFHKTGIARGHVLFAQANSLCVALIK
QPTVLTHESSIQFIEKVKLNDTVRAEARVVNQTAKHYYVEVKSYVKHTLVFKGNFKMFYDK
RG
11111100000000000000000000000000000000000000000000000000000000000000000000000000000
00000000000000000000000000000000000000000000000000000000000000000000000000000000000

000000000000000000000001
>Q2LQ23_3d00_0_189
MTARNILSYSYEEYVEKITAFHGYPAPGVLIGGFMVDLAVKNLPEGILYDAICETRTCLPDAV
QLLTPCTFGNGWLTVLPMGLFAVSLYDKFTGEGVRVFLDVEKMGPWQEIRNWFLKLKTKK
EQDSERLFKEIREAGPDILELRNVKLKPGFLEKKHKGKIVLCPQCREAYPAQDGELCLSCQG
GSPYL
00000000000000000000000000000000000000000000000000000000000000000000000000000000000
00000000000000000000000000000000000011111100000000000000000000000000000000000000000
000000000000000000000000
>Q4QL22_3bge_250_439
GDRFYDLISALHKSVRGSAPDAALYWYARILTAGGDPLYVARRLLAIASEDVGNADPRAMQ
VALAAWDCFTRVGAYEGERAIAQAIIYLAVAPKSNAVYTAFNTAKQQAKDLPDYDVPPHL
RNAPTNLMKELGYGAEYRYAHDEPNAYAAGENYFPPELKDTQYYFPTNRGMEIQIKEKLE
RLREQDKST
00000000000000000000000000000000000000000000000000000000000000000000000000000000000
00000000000000000000000000000000000000000000000011111111111111111100000000000000000
000000000000000000111111
>Q4W5X6_2d5m_0_189
MKKSLGARTLAYPTPLFLVGTYDRDSRPNIMAAAWAGICCSQPPSIAVSLRKATYTYRSITER
GAFTISIPSRAYVRHADYAGIYSGENEDKFASLGLTPVPGEHVDAPYVGEFPMAIELKLIHQI
EIGLHTQFIGEIMDVKVDESCLRDDGLPDINKVDPVIFAPVSREYYAVGEFLAKAFSAGKGL
RS
00000000000000000000000000000000000000000000000000000000000000000000000000000000000
00000000000000000000000000000000000000000000111000000000000000000000000000000000000
000000000000000000001111
>Q4WCU5_2vxk_0_189
MTNATIAPTTTAAPVTKSVDAPTADENTPLFSPSLISPDVLAVLPADYTIRPLCRSDYKRGYL
DVLRVLTTVGDINEEQWNSRYEWIRARSDEYYLLVVCDGEGRIVGTGSLVVERKFIHSLGMV
GHIEDIAVEKGQQGKKLGLRIIQALDYVAEKVGCYKTILDCSEANEGFYIKCGFKRAGLEM
AHYY
11111111111111111111111110000000000000000000000000000000000000000000000000000000000
00000000000000000000000000000000000000000000000000000000000000000000000000000000000
000000000000000000000000
>Q97RK1_2fi1_0_189
MKGMKYHDYIWDLGGTLLDNYETSTAAFVETLALYGITQDHDSVYQALKVSTPFAIETFAP
NLENFLEKYKENEARELEHPILFEGVSDLLEDISNQGGRHFLVSHRNDQVLEILEKTSIAAYF
TEVVTSSSGFKRKPNPESMLYLREKYQISSGLVIGDRPIDIEAGQAAGLDTHLFTSIVNLRQVL
DI
11100000000000000000000000000000000000000000000000000000000000000000000000000000000
00000000000000000000000000000000000000000000000000000000000000000000000000000000000
000000000000000000000000
>Q9ZL26_3gox_0_189
MLKNDDFVIAKNQLGNIVPNSVGVIRAVNGKSAMVLFIGLNELKRVDFSELEAIDIYRTGK
GYDKKICNICHILKNTDGFEINQTDAKGRKTTRPSCRECRKNIDGVKLSSTEKKKMDEIAPP
KGSVFTCPICEKRSIVGVTANLVHDHNHDTGWGREWICDSCNTGLGRFKDNPKFLEKVIEY
LKKYEK
00000000000000000000000000000000000000000000000000000000000000000000000000000000000
00000000000000000000000000000000000000000000000000000000000000000000000000000000000
000000000000000000000001
>E5Q9D7_3nfw_0_188
MVTAEAIDQRTFRRVLGQFCTGVTIITTVHEGNPVGFACQSFAALSLDPPLVLFCPTKVSRS

WKAIEASGRFCVNILHEKQQHVSARFGSREPDKFAGIDWRPSDLGSPIIDGSLAHIDCTVHD
VHDGGDHFVVFGKVHGLSEVPERKPRPLLFYRGEYTGIEPEKNTPAQWRDDLEAFLTATTA
DTWL
10000000000000000000000000000000000000000000000000000000000000000000000000000000
00000000000000000000000000000000000000000000000000000000000000000000000000000000
0000000000000000111111
>P53990_3frr_0_188
MLGSGFKAERLRVNLRLVINRLKLLEKKKTELAQKARKEIADYLAAGKDERARIRVEHIIRE
DYLVEAMEILELYCDLLLARFGLIQSMKELDSGLAESVSTLIWAAPRLQSEVAELKIVADQLC
AKYSKEYGKLCRTNQIGTVNDRLMHKLSVEAPPKILVERYLIEIAKNYNVPYEPDSVVMAE
APP
10000000000000000000000000000000000000000000000000000000000000000000000000000000
00000000000000000000000000000000000000000000000000000000000000000000000000000000
00000000000000000000011
>P83194_1qy4_0_188
MYKEPFGVKVDFETGIIEGAKKSVRRLSDMEGYFVDERAWKELVEKEDPVVYEVYAVEQEE
KEGDLNFATTVLYPGKVGKEFFFTKGHFHAKLDRAEVYVALKGKGGMLLQTPEGDAKWIS
MEPGTVVYVPPYWAHRTVNIGDEPFIFLAIYPADAGHDYGTIAEKGFSKIVIEENGEVKVVD
NPRWKK
00000000000000000000000000000000000000000000000000000000000000000000000000000000
00000000000000000000000000000000000000000000000000000000000000000000000000000000
00000000000000000000011
>P96671_1uxo_1_189
TKQVYIIHGYRASSTNHWFPWLKKRLLADGVQADILNMPNPLQPRLEDWLDTLSLYQHTL
HENTYLVAHSLGCPAILRFLEHLQLRKQLGGIILVSGFAKSLPTLQMLDEFTQGSFDHQKIIES
AKHRAVIASKDDQIVPFSFSKDLAQQIDAALYEVQHGGHFLEDEGFTSLPIVYDVLTSYFSKE
TR
00000000000000000000000000000000000000000000000000000000000000000000000000000000
00000000000000000000000000000000000000000000000000000000000000000000000000000000
00000000000000000000111
>Q0S8Y3_2guh_1_189
TSDPGTAAPVKRTAEQSRSLIVDAAGRAFATRPYREITLKDIAEDAGVSAPLIIKYFGSKEQLF
DALVDFRAAAEIVFSGPLDGLGERMVSMFARPLEPYKPLSLNILFMSGPSEESSRKLRANYSA
QMIDALAERLPGRDARLRAELVMSMLTGLAVMRRKMMQEHATGTPEEVVAHYAPLVQEL
LDG
11111111111000000000000000000000000000000000000000000000000000000000000000000000
00000000000000000000000000000000000000000000000000000000000000000000000000000000
00000000000000000000000
>Q15475_4egc_0_188
MSMLPSFGFTQEQVACVCEVLQQGGNLERLGRFLWSLPACDHLHKNESVLKAKAVVAFH
RGNFRELYKILESHQFSPHNHPKLQQLWLKAHYVEAEKLRGRPLGAVGKYRVRRKFPLPRT
IWDGEETSYCFKEKSRGVLREWYAHNPYPSPREKRELAEATGLTTTQVSNWFKNRRQRDR
AAEAKEREN
00000000000000000000000000000000000000111111000000000000000000000000000000000000
00000000000000000000000000000000000000111111111100000000000000000000000000000000
0000000000000000001111
>Q5LDN3_3ek3_0_188
MKTNEVLETIKARRSVRAYDRKQIPADDLNAILEAGAYAPSGMHYETWHFTAVCNTVKLE
ELNERIKGAFAKSDDKHLRERGHSETYCCYYHAPTLVIVSNEPKQWWAGMDCACAIENMF
LAATSLGIASCWINQLGTTCDDPEVRAYLTSLGVPENHKVYGCVALGYKAEGALLKEKTVK
AGTITIVE

11000000000000000000000000000000000000000000000000000000000000000000000000000000000
00000000000000000000000000000000000000000000000000000000000000000000000000000000000
00000000000000000000000
>Q81YZ2_3hmc_0_188
MGHIIDISKWNGDINWSIAKQHIDFIIARVQDGSNYVDPLYKGYVQAMKQHGIPFGNYAFC
RFVSIADAKKEAQDFWNRGDKSATVWVADVEVKTMNDMRAGTQAFIDELYRLGAKKVG
LYVGHHMYTPFGMANVKSDFVWIPRYGGNKPAYPCDIWQYTETGNVPGIGKCDLNSLIGN
KSLSWFTESA
00000000000000000000000000000000000000000000000000000000000000000000000000000000000
00000000000000000000000000000000000000000000000000000000000000000000000000000000000
00000000000000000000011
>Q978J0_2rfm_0_188
MDKNGEIVEKIKDEKSINQNLDFLRNYRDSYNRTPLMVACMLGMENAIDKLVENFDKLED
KDIEGSTALIWAVKNNRLGIAEKLLSKGSNVNTKDFSGKTPLMWSIIFGYSEMSYFLLEHGA
NVNDRNLEGETPLIVASKYGRSEIVKKLLELGADISARDLTGLTAEASARIFGRQEVIKIFTEV
RRA
11111000000000000000000000000000000000000000000000000000000000000000000000000000000
00000000000000000000000000000000000000000000000000000000000000000000000000000000000
00000000000000000000000
>Q9RVG4_2ou6_0_188
MPTFNPELHAQTLNSERAYFVQPDADPAFTPHIGALVEMLTYARLTTLQAVEGLPEDQLWA
TAPGFANSIGTLLAHIAAVERVYHVLSFQGRDVTPEDDGAAYWGLTMGKEGTAPARLPTLD
ELRAELADARAETLRVFAAKDDAWLAEPLGPGWANQHWAWFHVMEDEVNHRGQLRLL
RQVLAPEEGG
10000000000000000000000000000000000000000000000000000000000000000000000000000000000
00000000000000000000000000000000000000000000000000000000000000000000000000000000000
00000000000000000011111
>A2ASQ1_3pve_1509_1700
LENAGSRPFLADFNGFSYLELKGLHTFERDLGEKMALEMVFLARGPSGLLLYNGQKTDGK
GDFVSLALHNRHLEFRYDLGKGAAIIRSKEPIALGTWVRVFLERNGRKGALQVGDGPRVLG
ESPVPHTMLNLKEPLYVGGAPDFSKLARGAAVASGFDGAIQLVSLRGHQLLTQEHVLRAVD
VAPFAG
11111000000000000000001111110000000000000000000000000000000000000000000000000000000
00000000000000000000000000000000000000000000000000000000000000000000000000000000000
0000000000000000000000
>C4ZCS9_3hyn_0_187
MSYQNANYSAFYVSEPFSESNLGANSTHDFVYYNMLRMWKGEDNSFPFNDAHDKTYNV
RDGSDWEKTLKPRLHTRLDNSKNIILFLSSITANSRALREEMNYGIGTKGLPVIVIYPDYDKK
SDIVDSNGNFKKQIKDLWDKLPAFRDNMSSVATLHIPCTKSVIISALNNEDFMVNTMADAE
KYYYKP
11000000000000000000000000000000000000000000000000000000000000000000000000000000000
00000000000000000000000000000000000000000000000000000000000000000000000000000000000
0000000000000000000000
>D7WGJ0_3vbm_0_187
MNSFYSQEELKKIGFLSVGKNVLISKKASIYNPGVISIGNNVRIDDFCILSGKVTIGSYSHIAAY
TALYGGEVGIEMYDFANISSRTIVYAAIDDFSGNALMGPTIPNQYKNVKTGKVILKKHVIIG
AHSIIFPNVVIGEGVAVGAMSMVKESLDDWYIYVGVPVRKIKARKRKIVELENEFLKSMNS
00000000000000000000000000000000000000000000000000000000000000000000000000000000000
00000000000000000000000000000000000000000000000000000000000000000000000000000000000
0000000000000000000011
>O25743_2qm6_379_566

TTHYSVADRWGNAVSVTYTINASYGSAASIDGAGFLLNNEMDDFSIKPGNPNLYGLVGGD
ANAIEANKRPLSSMSPTIVLKNNKVFLVVGSPGGSRIITTVLQVISNVIDYNMNISEAVSAPRF
HMQWLPDELRIEKFGMPADVKDNLTKMGYQIVTKPVMGDVNAIQVLPKTKGSVFYGSTD
PRKEF
00000000000000000000000000000000000000000000000000000000000000000000000000000000000
00000000000000000000000000000000000000000000000000000000000000000000000000000000000
0000000000000000000011
>P52007_1k4n_0_187
MANWQSIDELQDIASDLPRFIHALDELSRRLGLNITPLTADHISLRCHQNATAERWRRGFEQ
CGELLSENMINGRPICLFKLHEPVQVAHWQFSIVELPWPGEKRYPHEGWEHIEIVLPGDPET
LNARALALLSDEGLSLPGISVKTSSPKGEHERLPNPTLAVTDGKTTIKFHPWSIEEIVASEQSA
00000000000000000000000000000000000000000000000000000000000000000000000000000000000
00000000000000000000000000000000000000000000000000000000000000000111111100000000000
0000000000000000000011
>P95128_3p9n_0_187
MTRIIGGVAGGRRIAVPPRGTRPTTDRVRESLFNIVTARRDLTGLAVLDLYAGSGALGLEALS
RGAASVLFVESDQRSAAVIARNIEALGLSGATLRRGAVAAVVAAGTTSPVDLVLADPPYNV
DSADVDAILAALGTNGWTREGTVAVVERATTCAPLTWPEGWRRWPQRVYGDTRLELAER
LFANV
00000000000000000000111000000000000000000000000000000000000000000000000000000000000
00000000000000000000000000000000000000000000000000000000000000000000000000000000000
0000000000000000001111
>Q03555_1ihc_0_200
MATEGMILTNHDHQIRVGVLTVSDSCFRNLAEDRSGINLKDLVQDPSLLGGTISAYKIVPDEI
EEIKETLIDWCDEKELNLILTTGGTGFAPRDVTPEATKEVIEREAPGMALAMLMGSLNVTPL
GMLSRPVCGIRGKTLIINLPGSKKGSQECFQFILPALPHAIDLLRDAIVKVKEVHDELEDLPS
11111111111100000000000000000000000000000000000000000000000000000000000000000000000
00000000000000000000000000000000000000000000000000000000000000000000000000000000000
0000000000000001111111
>Q5SKQ0_2cvb_0_187
MLQYPELPLESPLIDAELPDPRGGRYRLSQFHEPLLAVVFMCNHCPYVKGSIGELVALAERY
RGKVAFVGINANDYEKYPEDAPEKMAAFAEEHGIFFPYLLDETQEVAKAYRALRTPEVFLF
DERRLLRYHGRVNDNPKDPSKVQSHDLEAAIEALLRGEEPPLKEAPAIGCTIKWRPGNEPE
VRIG
10000000000000000000000000000000000000000000000000000000000000000000000000000000000
00000000000000000000000000000000000000000000000000000000000000000000000000000000000
0000000000000000000000
>Q6PH17_4hkf_0_187
MDFPYDLNALFPERISVLDSNLSAGRKAHGRPDPLPQVTTVIDELGKASSKAQQLPAPITSA
AKLQANRHHLYLLKDGEQNGGRGVIVGFLKVGYKKLFLLDQRGAHLETEPLCVLDFYVTE
TLQRHGYGSELFDFMLKHKQVEPAQMAYDRPSPKFLSFLEKRYDLRNSVPQVNNFVVFAG
FFQSRS
11100000000000000000001111111111100000000000000000000000000000000000000000000000000
00000000000000000000000000000000000000000000000000000000000000000000000000000000000
0000000000000000001111
>Q8NQ97_4af5_0_187
MSVAAGDKPTNSRQEILEGARRCFAEHGYEGATVRRLEEATGKSRGAIFHHFGDKENLFLA
LAREDAARMAEVVSENGLVEVMRGMLEDPERYDWMSVRLEISKQLRTDPVFRAKWIDHQ
SVLDEAVRVRLSRNVDKGQMRTDVPIEVLHTFLETVLDGFISRLATGASTEGLSEVLDLVEG
TVRKRD
11111111111100000000000000000000000000000000000000000000000000000000000000000000000

0000000000000000000000000000000000000000000000000000000000000000000000000000000000000
0000000000000000000011
>Q8NX77_2ap3_20_207
TTDKKEIKAYLKQVDKIKDDEEPIKTVGKKIAELDEKKKKLTEDVNSKDTAVRGKAVKDLIK
NADDRLKEFEKEEDAIKKSEQDFKKAKSHVDNIDNDVKRKEVKQLDDVLKEKYKLHSDY
AKAYKKAVNSEKTLFKYLNQNDATQQGVNEKSKAIEQNYKKLKEVSDKYTKVLNKVGKE
KQDVDQFK
00000000000000000000000000000000000000000000000000000000000000000000000000000000000
00000111100000000000000000000000000000000000000000000000000000000000000000000000000
0000000000000000000111
>Q9AJW7_3lw8_0_187
MLGTSFNNFGISLSHKRYFSGKVDEIIRCTMGKRIVKISSTKINTSILSSVSEQIGENITDWKND
EKKVYVSRVVNQCIDKFCAEHSRKIGDNLRKQIFKQVEKDYRISLDINAAQSSINHLVSGSSY
FKKKMDELCEGMNRSVKNDTTSNVANLISDQFFEKNVQYIDLKKLRGNMSDYITNLESPF
11111110000000000000000000000000000000000000000000000000000000000000000000000000000
00000000000000000000000000000000000000000000000000000000000000000000000000000000000
0000000000000000000000
>Q9KF54_3p9x_0_187
MKRVAIFASGSGTNAEAIIQSQKAGQLPCEVALLITDKPGAKVVERVKVHEIPVCALDPKTY
PSKEAYEIEVVQQLKEKQIDFVVLAGYMRLVGPTLLGAYEGRIVNIHPSLLPAFPGLHAIEQA
IRANVKVTGVTIHYVDEGMDTGPIIAQEAVSIEEEDTLETLTTKIQAVEHRLYPATLHKLLSK
10000000000000000000000000000000000000000000000000000000000000000000000000000000000
00000000000000000000000000000000000000000000000000000000000000000000000000000000000
0000000000000000000000
>Q9VSL8_3fyq_1980_2167
SRGTQACINAAHTVSGIIGDLDTTIMFATAGTLHSDGDGSFADHREHILQTAKALVEDTKVL
VTGAAGTQDQLANAAQNAVSTITQLAEAVKRGACSLGSTQPDSQVMVINAVKDVASALG
DLINCTKLASGKSINDPSMQDLKESARVMVLNVSSLLKTVKAVEDEHTRGTRAMEATVEAI
SQEIRA
11000000000000000000000000000000011100000000000000000000000000000000000000000000000
00000000000000000000000000000000000000000000000011111110000000000000000000000000000
0000000000000000000000
>D1C9F1_3sho_95_281
DRQQLPVQVFTNDIENLNQTLNQTQPEAIEAAVEAICRADHVIVVGMGFSAAVAVFLGHG
LNSLGIRTTVLTEGGSTLTITLANLRPTDLMIGVSVWRYLRDTVAALAGAAERGVPTMALTD
SSVSPPARIADHVLVAATRGVGHSLSPVGLIAVVNLLLAEIAVREPERALAVLREVDRLYREQ
GM
00000000000000000000000000000000000000000000000000000000000000000000000000000000000
00000000000000000000000000000000000000000000000000000000000000000000000000000000000
000000000000000000011
>O43615_2cw9_265_451
DESDNAFIRASRALTDKVTDLLGGLFSKTEMSEVLTEILRVDPAFDKDRFLKQCENDIIPNVL
EAMISGELDILKDWCYEATYSQLAHPIQQAKALGLQFHSRILDIDNVDLAMGKMMEQGPV
LIITFQAQLVMVVRNPKGEVVEGDPDKVLRMLYVWALCRDQDELNPYAAWRLLDISASSTE
QIL
11110000000000000000000000000000000000000000000000000000000000000000000000000000000
00000000000000000000000000000000000000000000000000000000000000000000000000000000000
000000000000000000001
>Q28PN4_3h0n_0_186
MNLDSYERTGLRVSLDLVNIATPGSRRGTPHTGGCVIEDLHDLLKDDPASVAQLGDDHVE
GFVELARLLHTAIDALSNGQVATAATALNHLLRKHPATPELAQDPDGTWRLHHHPLDAEL

VPMWTAICAEGLAREIGHQNVRRFGICNAHRCDRVYFDTSRNGTRQYCSLACQNRVKAA
AFRERRAT
00000000000000000000000000000000000000000000000000000000000000000000000000000000000
00000000000000000000000000000000000000000000000000000000000000000000000000000000000
000000000000000000111
>Q3BQL2_4fc9_473_659
SATASELLLTAALERIEDTAQAMLSTVIDEERNPFLEGAPSYLPGKRPTDVTTFGQVPALRDM
LAESRDLEFLQRVSDMAGPSPRIEDPSEEGLARHYTNVSNWKAQKSAHLGIVDHLGQFVY
HEGSPLDVATLAKAVQMWKTRELIVHAHPQDRARFPELAVHIPEQVSDDSDSEQQTSPEPS
GHQ
00000000000000000000000000000000000000000000000000000000000000000000000000000000000
00000000000000000000000000000000000000000000000000000000000000000000000000000000000
011111111111111111111
>Q57840_1go3_0_186
MYKILEIADVVKVPPEEFGKDLKETVKKILMEKYEGRLDKDVGFVLSIVDVKDIGEGKVVH
GDGSAYHPVVFETLVYIPEMYELIEGEVVDVVEFGSFVRLGPLDGLIHVSQIMDDYVSYDPK
REAIIGKETGKVLEIGDYVRARIVAISLKAERKRGSKIALTMRQPYLGKLEWIEEEKAKKQNQ
E
00000000000000000000000000000000000000000000000000000000000000000000000000000000000
00000000000000000000000000000000000000000000000000000000000000000000001111100000000
000000000000000000111
>Q9CIV9_2iu5_0_186
MSAFFLNMKKSIITQKIIAKAFKDLMQSNAYHQISVSDIMQTAKIRRQTFYNYFQNQEELLS
WIFENDFAELINDNSDYYGWQNELLLLLRYLDENQIFYQKIFVIDKNFEHFFLIQWENLLDK
VIFDQEKKSDYHWSDLEKSFICRYNAAAICAITRESIIRGNSLEKLYSQIVNLLLAQIKIFES
11111110000000000000000000000000000000000000000000000000000000000000000000000000000
00000000000000000000000000000000000000000000000000000000000000000000000000000000000
000000000000000000000
>Q9I2B5_2h1t_0_186
MSRDRLYTWAGLWRSPSSSWEALRLEDDQAESQLRAPDERSGLPYQLDYRLRWDADWHL
REAVFHVESETGVRKLHLLADGRGHWQDGDGEALPAFDGCLDIDIWPSPFTNTFPIRRLGL
ADGQRAEIRALYIEAPALEPRSMRQAYTRLDASHYLYENLEGSAFKAVLLVDEQGLVIDYPG
LFQRL
10000000000000000000000000000000000000000000000000000000000000000000000000000000000
00000000000000000000000000000000000000000000000000000000000000000000000000000000000
000000000000000000000
>O75626_3dal_37_222
KMDMEDADMTLWTEAEFEEKCTYIVNDHPWDSGADGGTSVQAEASLPRNLLFKYATNSE
EVIGVMSKEYIPKGTRFGPLIGEIYTNDTVPKNANRKYFWRIYSRGELHHFIDGFNEEKSNW
MRYVNPAHSPREQNLAACQNGMNIYFYTIKPIPANQELLVWYCRDFAERLHYPYPGELTM
MNLTQ
11111100000000000000000000000001111111100000000000000000000000000000000000000000000
00000001110000000000000000000000000000000000000000000000000000000000000000000000000
00000000000000000000
>O75884_2qs9_0_185
MASPSKAVIVPGNGGGDVTTHGWYGWVKKELEKIPGFQCLAKNMPDPITARESIWLPFME
TELHCDEKTIIIGHSSGAIAAMRYAETHRVYAIVLVSAYTSDLGDENERASGYFTRPWQWEKI
KANCPYIVQFGSTDDPFLPWKEQQEVADRLETKLHKFTDCGHFQNTEFHELITVVKSLLKV
PA
11000000000000000000000000000000000000000000000000000000000000000000000000000000000
00000000000000000000000000000000000000000000000000000000000000000000000000000000000

0000000000000000000
>P03007_1j53_0_185
MSTAITRQIVLDTETTGMNQIGAHYEGHKIIEIGAVEVVNRRLTGNNFHVYLKPDRLVDPEA
FGVHGIADEFLLDKPTFAEVADEFMDYIRGAELVIHNAAFDIGFMDYEFSLLKRDIPKTNTF
CKVTDSLAVARKMFPGKRNSLDALCARYEIDNSKRTLHGALLDAQILAEVYLAMTGGQTS
MA
11111100000000000000000000000000000000000000000000000000000000000000000000000000000
00000000000000000000000000000000000000000000000000000000000000000000000000000000000
0000000000000111111
>P08603_3sw0_1045_1230
TSCVNPPTVQNAYIVSRQMSKYPSGERVRYQCRSPYEMFGDEEVMCLNGNWTEPPQCKDS
TGKCGPPPPIDNGDITSFPLSVYAPASSVEYQCQNLYQLEGNKRITCRNGQWSEPPKCLHPC
VISREIMENYNIALRWTAKQKLYSRTGESVEFVCKRGYRLSSRSHTLRTTCWDGKLEYPTCA
KR
00000000000000000000000000000000000000000000000000000000000000000000000000000000000
00000000000000000000000000000000000000000000000000000000000000000000000000000000000
0000000000000000001
>P43221_1jfu_35_220
SRAPTGDPACRAAVATAQKIAPLAHGEVAALTMASAPLKLPDLAFEDADGKPKKLSDFRG
KTLLVNLWATWCVPCRKEMPALDELQGKLSGPNFEVVAINIDTRDPEKPKTFLKEANLTRL
GYFNDQKAKVFQDLKAIGRALGMPTSVLVDPQGCEIATIAGPAEWASEDALKLIRAATGKA
AAAL
11100000000000000000000000000000000000000000000000000000000000000000000000000000000
00000000000000000000000000000000000000000000000000000000000000000000000000000000000
0000000000000111111
>P51665_2o95_0_185
MPELAVQKVVVHPLVLLSVVDHFNRIGKVGNQKRVVGVLLGSWQKKVLDVSNSFAVPFDE
DDKDDSVWFLDHDYLENMYGMFKKVNARERIVGWYHTGPKLHKNDIAINELMKRYCPN
SVLVIIDVKPKDLGLPTEAYISVEEVHDDGTPTSKTFEHVTSEIGAEEAEEVGVEHLLRDIKDT
TVGT
00000000000000000000000000000000000000000000000000000000000000000000000000000000000
00000000000000000000000000000000000000000000000100000000000001111000000000000000000
0000000000000000011
>P95010_2v7s_33_218
TMDHNPDTSRRLTGEQKIQLIDSMRNKGSYEAARERLTATARIIADRVSAAIPGQTWKFDD
DPNIQQSDRNGALCDKLTADIARRPIANSVMFGATFSAEDFKIAANIVREEAAKYGATTESS
LFNESAKRDYDVQGNGYEFRLLQIKFATLNITGDCFLLQKVLDLPAGQLPPEPPIWPTTSTP
H
11111000000000000000000000000000000000000000000000000000000000000000000000000000000
00000000000000000000000000000000000000000000000000000000000000000000000000000000000
0000000111111111111
>Q3BYJ5_2oix_334_519
ATSWLLDGHLRAYTDDLARRLRGEPNAHLLHFADSQVVTMLSSADPDQQARAQRLLAGD
DIPPIVFLPINQPNAHWSLLVVDRRNKDAVAAYHYDSMAQKDPQQRYLADMAAYHLGLD
YQQTHEMPIAIQSDGYSCGDHVLTGIEVLAHRVLDGTFDYAGGRDLTDIEPDRGLIRDRLA
QAEQAPA
10000000000000000000000000000000000000000000000000000000000000000000000000000000000
00000000000000000000000000000000000000000000000110000000000000000000000000000000000
0000000000000011111
>Q3M655_3ot2_0_185
MVQTPSKPITLDEFLKLPETEPASEYIEGKIIQKPMPQGKHSAIQSECVSVINSVVKPQRIARA

FLELRCTFGDHSTVPDISVFIWSRIPREENGEIANIFLIAPDWTIEILSPDQSQTKVTKNILHCL
KHGTQMGWLIDPDEQTVFVYRPQQETEVFDEPDALVPVPSFASELHLSIKDLFSWLL
11111110000000000000000000000000000000000000000000000000000000000000000000000000000
00000000000000000000000000000000000000000000000000000000000000000000000000000000000
00000000000000000000
>Q749F5_2opl_0_185
MSQTTVVNGVNVDQLMATIEQIKAKPEIAQFKFRATNQWMGGTHNQATIKDFYGACAED
DTRKPMVFDLDEPPVLLGENRGANPVEYLLVALSGCLTTSLVAHAAARGIALRGVKSRYEG
DIDLRGFLGLSEEVPVGYREIRVFFSIDADLTDGQKEELIRMAQKYSPVYNTVAKPVPVAVLL
DRG
11100000000000000000000000000000000000000000000000000000000000000000000000000000000
00000000000000000000000000000000000000000000000000000000000000000000000000000000000
00000000000000000000
>Q7W980_3d3s_0_185
MRKDETSNTSPDISVAQPASALRYHLRPPRRNDGAAIHQLVSECPPLDLNSLYAYLLLCEHH
AHTCVVAESPGGRIDGFVSAYLLPTRPDVLFVWQVAVHSRARGHRLGRAMLGHILERQEC
RHVRHLETTVGPDNQASRRTFAGLAGERGAHVSEQPFFDRQAFGGADHDDEMLLRIGPFT
HPPH
11111111111111111111110000000000000000000000000000000000000000000000000000000000000
00000000000000000000000000000000000000000000000000000000000000000000000000000000001
11111000000000011111
>Q9I0Q8_3owc_0_185
MPTPGTGSVPELQLVPFQLGHFPILQRWFATEKELVQWAGPALRHPLSLEQMHEDLAESRR
RPPLRLLWSACRDDQVIGHCQLLFDRRNGVVRLARIVLAPSARGQGLGLPMLEALLAEAF
ADADIERVELNVYDWNAAARHLYRRAGFREEGLRRSATRVGRERWNVVLMGLLRQEWA
AGGAGND
11111111110000000000000000000000000000000000000000000000000000000000000000000000000
00000000000000000000000000000000000000000000000000000000000000000000000000000000000
00000000000000001111
>P05231_1alu_27_211
APVPPGEDSKDVAAPHRQPLTSSERIDKQIRYILDGISALRKETCNKSNMCESSKEALAENN
LNLPKMAEKDGCFQSGFNEETCLVKIITGLLEFEVYLEYLQNRFESSEEQARAVQMSTKVLI
QFLQKKAKNLDAITTPDPTTNASLLTKLQAQNQWLQDMTTHLILRSFKEFLQSSLRALRQ
M
11111111111111111110000000000000000000000000000111111110000000000000000000000000000
00000000000000000000000000000000000000000000000000000000000000000000000000000000000
00000000000000000000
>P54422_3a75_402_586
TTHFTVADRWGNVVSYTTTIEQLFGTGIMVPDYGVILNNELTDFDAIPGGANEVQPNKRPL
SSMTPTILFKDDKPVLTVGSPGGATIISSVLQTILYHIEYGMELKAAVEEPRIYTNSMSSYRYED
GVPKDVLSKLNGMGHKFGTSPVDIGNVQSISIDHENGTFKGVADSSRNGAAIGINLKRK
00000000000000000000000000000000000000000000000000000000000000000000000000000000000
00000000000000000000000000000000000000000000000000000000000000000000000000000000000
00000000000000000001
>P77847_1pmh_35_219
ESSVNPVVLDFEDGTVMSFGEAWGDSLKCIKKVSVSQDLQRPGNKYALRLDVEFNPNNGW
DQGDLGTWFGGVVEGQFDFTGYKSVEFEMFIPYDEFSKSQGGFPYKVVINDGWKELGSEFN
ITANAGKKVKINGKDYTVIHKAFAIPEDFRTKKRAQLVFQFAGQNSNYKGPIYLDNVRIRPE
DA
11000000000000000000000000000000000000000000000000000000000000000000000000000000000
00000000000000000000000000000000000000000000000000000000000000000000000000000000000

000000000000000000
>Q1H3S7_3e4v_0_184
MRNELPPENAYRILESGPIVLVSTRGADGRANLMTMGFHMMMQHEPPLVGAIIGPWDYSH
QALSETGECVLAVPTVDLAETVVDIGNCSGDALDKFGHFGLTPVPAQTVDAPLVRQCWAN
LECRVVDDGWARRYNLWVLEVQRIWIDTARKETRLIHHQGDGRFSVDGDTLDLGERMTK
WRHLMG
11000000000000000000000000000000000000000000000000000000000000000000000000000000000
00000000000000000000000000000000000000000000000000000000000000000000000000000000000
000000000000001111
>Q8LTE1_3rlk_144_328
SKPDPVIPDPPIDPPPGTGKYTCPFAIWSLEEVYEPPTKNRPWPIYNAVELQPREFDVALKDL
LGNTKWRDWDSRLSYTTFRGCRGNGYIDLDATYLATDQAMRDQKYDIREGKKPGAFGNIE
RFIYLKSINAYCSLSDIAAYHADGVIVGFWRDPSSGGAIPFDFTKFDKTKCPIQAVIVVPRA
11000000000000000000000000000000000000000000000000000000000000000000000000000000000
00000000000000000000000000000000000000000000000000000000000000000000000000000000000
000000000000000000
>Q91H74_2fom_1475_1659
AGVLWDVPSPPPVGKAELEDGAYRIKQKGILGYSQIGAGVYKEGTFHTMWHVTRGAVLM
HKGKRIEPSWADVKKDLISYGGGWKLEGEWKEGEEVQVLALEPGKNPRAVQTKPGLFKT
NTGTIGAVSLDFSPGTSGSPIVDKKGKVVGLYGNGVVTRSGAYVSAIAQTEKSIEDNPEIEDD
IFRK
11111111111111111000000000000000000000000000000000000000000000000000000000000000000
00000000000000000000000000000000000000000000000000000000000000000000000000000000000
011111111111111111
>O69756_3ell_0_183
MSISISYSTTYSGWTVADYLADWSAYFGDVNHRPGQVVDGSNTGGFNPGPFDGSQYALKST
ASDAAFIAGGDLHYTLFSNPSHTLWGKLDSIALGDTLTGGASSGGYALDSQEVSFSNLGLDS
PIAQGRDGTVHKVVYGLMSGDSSALQGQIDALLKAVDPSLSINSTFDQLAAAGVAHATPA
A
10000000000000000000000000000000000000000000000000000000000000000000000000000000000
00000000000000000000000000000000000000000000000000000000000000000000000000000000000
000000000000000000
>P01033_3v96_23_206
CTCVPPHPQTAFCNSDLVIRAKFVGTPEVNQTTLYQRYEIKMTKMYKGFQALGDAADIRFV
YTPAMESVCGYFHRSHNRSEEFLIAGKLQDGLLHITTCSFVAPWNSLSLAQRRGFTKTYTVG
CEECTVFPCLSIPCKLQSGTHCLWTDQLLQGSEKGFQSRHLACLPREPGLCTWQSLRSQIA
00000000000000000000000011111000000000000000000000000111111100000000000000000000000
00000000000000000000000000000000000000000000000000000000000000000000000000000000000
000000000000111111
>P07612_1ypy_1_184
GAAASIQTTVNTLSERISSKLEQEANASAQTKCDIEIGNFYIRQNHGCNLTVKNMCSADAD
AQLDAVLSAATETYSGLTPEQKAYVPAMFTAALNIQTSVNTVVRDFENYVKQTCNSSAVVD
NKLKIQNVIIDECYGAPGSPTNLEFINTGSSKGNCAIKALMQLTTKATTQIAPKQVAGTGVQ
11000000000000000000000000000000000000000000000000000000000000000000000000000000000
00000000000000000000000000000000000000000000000000000000000000000000000000000000000
000000000000000000
>P19478_2o6d_20_203
GGGGEHQHGEEMMAAVPAPDAEGAAGFDEFPIGEDRDVGPLHVGGVYFQPVEMHPAPG
AQPSKEEADCHIEADIHANEAGKDLGYGVGDFVPYLRVVAFLQKHGSEKVQKVMFAPMN
AGDGPHYGANVKFEEGLGTYKVRFEIAAPSHDEYSLHIDEQTGVSGRFWSEPLVAEWDDFE
WKGPQW

11111111111111111111111111000000000000000000000000000000000000000000000000000000000
00000000000000000000000000000000000000000000000000000000000000000000000000000000000
000000000000000000
>Q13395_2ha8_1437_1620
NSRVSDLDLELLFQDRAARLGKSISRLIVVASLIDKPTNLGGLCRTCEVFGASVLVVGSLQCIS
DKQFQHLSVSAEQWLPLVEVKPPQLIDYLQQKKTEGYTIIGVEQTAKSLDLTQYCFPEKSLLL
LGNEREGIPANLIQQLDVCVEIPQQGIIRSLNVHVSGALLIWEYTRQQLLSHGDTKP
11111111111111111110000000000000000000000000000000000000000000000000000000000000000
00000000000000000000000000000000000000000000000000000000000000000000000000000000000
000000000000011111
>Q47510_3r96_337_520
RKYDVSLTPSGIKVNDEITLLYPALKYAEELYLLINQNKINFIKSMAWPAFVNNISDSVSFIEQS
MIDNQNEKALILFIKYKTKIAGVVSFNIIDHANKTAYIGYWLGANFQGKGIVTNAINKLIQE
YGDSGVIKRFVIKCIVDNKKSNATALRCGFTLEGVLQKAEILNGVSYDQNIYSKVIG
11100000000000000000000000000000000000000000000000000000000000000000000000000000000
00000000000000000000000000000000000000000000000000000000000000000000000000000000000
000000000000000001
>Q5SHW9_1wkc_0_183
MTKAELRRRARAAWRRLDLKALSRAVGAALLPWLRERGFRHILLYHPLPHELNLLPLMEA
YPARYYLPKVAGKGLTVHPFGPLAPGPFGLLEPTTPPEDPRVLDLVVVPGLAFDREGYRLGH
GQGFYDRFLKEVRAATVGVVPQALLFPALPRDPWDVPVDHLATEAGVEAVKRPAPGPGGL
LD
00000000000000000000000000000000000000000000000000000000000000000000000000000000000
01111111000000000000000000000000000000000000000000000000000000000000000000000000000
000000000111111111
>Q6FF54_2ew0_0_183
MTKQYLTHRCLIAPPEMADDFFANTVIYLARHDDEGAQGLIINRPSGIQVRELLNDLDIEA
DHVQPHEVLQGGPLRPEAGFVLHTGQPVWHSSIAVGENLCITTSKDILDAIAHNEGVGRY
QIALGYASWTKNQLEGEISRGDWLICDADMDLIFNLPYDERWDAAYKKLGVDRIWLSSEIG
HA
11110000000000000000000000000000000000000000000000000000000000000000000000000000000
00000000000000000000000000000000000000000000000000000000000000000000000000000000000
000000000000011111
>Q81NK7_3pp9_0_183
MSLLIRELETNDLDNFPEIDDSFIVNARLMLSLSKVNRRIEYTVEDVPSYEKSYLQNDNEELV
YNEYINKPNQIIYIALLHNQIIGFIVLKKNWNNYAYIEDITVDKKYRTLGVGKRLIAQAKQW
AKEGNMPGIMLETQNNNVAACKFYEKCGFVIGGFDFLVYKGLNMTSDEVAIYWYLHFDS
00000000000000000000000000000000000001000000000000000000111110000000000000000000000
00000000000000000000000000000000000000000000000000000000000000000000000000000000000
000000000000000001
>Q8X778_2if6_18_201
WQPQTGDIIFQISRSSQSKAIQLATHSDYSHTGMLVMRNKKPYVFEAVGPVKYTPLKQWIA
HGEKGKYVVRRVEGGLSVEQQQKLAQTAKRYLGKPYDFSFSWSDDRQYCSEVVWKVYQN
ALGMRVGEQQKLKEFDLSNPLVQAKLKERYGKNIPLEETVVSPQAVFDAPQLTTVAKEWPL
FSW
00000000000000000000000000000000000000000000000000000000000000000000000000000000000
00000000000000000000000000000000000000000000000000000000000000000000000000000000000
000000000000000011
>Q9CPU0_2za0_0_183
MAEPQPASSGLTDETAFSCCSDPDPSTKDFLLQQTMLRIKDPKKSLDFYTRVLGLTLLQKLD
FPAMKFSLYFLAYEDKNDIPKDKSEKTAWTFSRKATLELTHNWGTEDDETQSYHNGNSDPR

GFGHIGIAVPDVYSACKRFEELGVKFVKKPDDGKMKGLAFIQDPDGYWIEILNPNKIATII
11100000000000000000000000000000000000000000000000000000000000000000000000000000000
00000000000000000000000000000000000000000000000000000000000000000000000000000000000
00000000000000000
>Q9RGN5_3i57_1694_1877
QKVHVQYIDGETDQMLRQDDLDGYTDETIPYSTAEGIKKFEGDGYELFKDNFPAGEKFDN
DDTNDQFYTVIFKHHRENVDPNHSSADGTKGTKTLTETVHYKYANGTKAAEDQTAQVTF
TRNGVLDDVTGIVAWGKWNEASQSYKALTSPTIAGYAPSEAVVKRSSNSDAEQGPTLTVIYT
ADA
00000000000000000000000000000000000000000000000000000000000000000000000000000000000
00000000000000000000000000000000000000000000000000000000000000000000000000000000000
00000000000000001
>Q9WYN2_2qqe_0_183
MSGKLTVITGPMYSGKTTELLSFVEIYKLGKKKVAVFKPKIDSRYHSTMIVSHSGNGVEAHVI
ERPEEMRKYIEEDTRGVFIDEVQFFNPSLFEVVKDLLDRGIDVFCAGLDLTHKQNPFETTALL
LSLADTVIKKKAVCHRCGEYNATLTLKVAGGEEEIDVGGQEKYIAVCRDCYNTLKKRV
10000000000000000000000000000000000000001111111111111100000000000000000000000000000
00000000000000000000000000000000000000000000000000000000000000000000000001000000000
00000000000000111
>A9CKP1_2i6h_0_182
MKNDTAALAADIVDFWKKAGPDKWFDKDAAFDNHFHDRFRDAHFAAARRELDGWLE
GAESSLALMLLLDQFPRNCFRGTAHMYATDPLARFFADEAIRRGHDQAVSEDLRVFFYLPFS
HAEDIAAQQRACDLNQPLGGLYLHHAEEHRDIVERFGRFPHRNGILLRETTPEERQYLEEG
GFSG
00000000000000000000000000000000000000000000000000000000000000000000000000000000000
00000000000000000000000000000000000000000000000000000000000000000000000000000000000
0000000000001111
>C7NDE2_3t8k_0_182
MSEYRTVSAAAMLGTYEDFLELFEKGYEDKESVLKSNILYDVLRNNNDEARYKISMFLINK
GADIKSRTKEGTTLFFPLFQGGGNDITGTTELCKIFLEKGADITALYKPYKIVVFKNIFNYFVD
ENEMIPLYKLIFSQSGLQLLIKDKWGLTALEFVKRCQKPIALKMMEDYIKKYNLKENS
00000000000000000000000000000000000000000000000000000000000000000000000000000000000
00000000000000000000000000000000000000000000000000000000000000000000000000000000000
0000000000000011
>C8XA43_4hbz_0_182
MAEPTAPSDRSDPAGARTLVLMRHAAAGSAVRDHDRPLTPDGVRAATAAGQWLRGHLP
AVDVVVCSTAARTRQTLAATGISAQVRYRDELYGGGVDEILAEVAAVPADASTVLVVGHAP
TIPATGWELVRQSLLNRDADPSSGAGDELRHFAAGTFAVLSTTGAWADLAQAGAELQLVQ
HPVA
11111111111100000000000000000000000000000000000000000000000000000000000000000000000
00000000000000000000000000000000000000000000000000000000000000000000000000000000000
0000000000000000
>P03748_4a0t_370_552
GHVLQLESASDKAHYILSKDGNRNNWYIGRGSDNNNDCTFHSYVHGTTLTLKQDYAVVN
KHFHVGQAVVATDGNIQGTKWGGKWLDAYLRDSFVAKSKAWTQVWSGSAGGGVSVTVS
QDLRFRNIWIKCANNSWNFFRTGPDGIYFIASDGGWLRFQIHSNGLGFKNIADSRSVPNAI
MVENE
10000000000000000000000000000000000000000000000000000000000000000000000000000000000
00000000000000000000000000000000000000000000000000000000000000000000000000000000000
0000000000000000
>P05938_2hq8_0_182

PEVTASERAYHLRKMKTRMKRVDVTGDGFISREDYELIAVRIAKIAKLSAEKAEETRQEFLRV
ADQLGLAPGVRISVEEAAVNATDSLLKMKAEEKAMAVIQSLIMYDCIDTDKDGYVSLPEFK
AFLQAVGPDITDDKAITCFNTLDFNKNGQISRDEFLVTVNDFLFGLEETALANAFYGDL
11000000000000000000000000000000000000000000000000000000000000000000000000000000000
00000000000000000000000000000000000000000000000000000000000000000000000000000000000
00000000000000000
>P18902_1kt4_0_182
ERDCRVSSFRVKENFDKARFAGTWYAMAKKDPEGLFLQDNIVAEFSVDENGHMSATAKGR
VRLLNNWDVCADMVGTFTDTEDPAKFKMKYWGVASFLQKGNDDHWIIDTDYETFAVQY
SCRLLNLDGTCADSYSFVFARDPSGFSPEVQKIVRQRQEELCLARQYRLIPHNGYCDGKSER
NIL
00000000000000000000000000000000000000000000000000000000000000000000000000000000000
00000000000000000000000000000000000000000000000000000000000000000000000000000000000
00000000011111111
>P22629_4dne_0_182
MRKIVVAAIAVSLTTVSITASASADPSKDSKAQVSAAEAGITGTWYNQLGSTFIVTAGADGA
LTGTYESAVGNAESRYVLTGRYDSAPATDGSGTALGWTVAWKNNYRNAHSATTWSGQYV
GGAEARINTQWLLTSGTTEANAWKSTLVGHDTFTKVKPSAASIDAAKKAGVNNGNPLDA
VQQ
11111111111111111111111111111111111100000000000000000000000000000000000000000000000
00000000000000000000000000000000000000000000000000000000000000000000000000001111111
11111111111111111
>P69503_2dy0_0_182
MTATAQQLEYLKNSIKSIQDYPKPGILFRDVTSLLEDPKAYALSIDLLVERYKNAGITKVVGTE
ARGFLFGAPVALGLGVGFVPVRKPGKLPRETISETYDLEYGTDQLEIHVDAIKPGDKVLVVD
DLLATGGTIEATVKLIRRLGGEVADAAFIINLFDLGGEQRLEKQGITSYSLVPFPGH
10000000000000000000000000000000000000000000000000000000000000000000000000000000000
00000000000000000000000000000000000000000000000000000000000000000000000000000000000
00000000000000000
>Q4FPZ7_2i9w_0_182
MLFSIQTCPCQINPALNAVSTPLLYQDCCQPYHDGLYNQEVEDSDAIRADTAEHLMRTRYS
AFVLVKPEYIVKTTLPAQQDLLDIKAIENWAKETDWAGLEVVAHTPKLSKRHAQVEFKAYF
KTPDGLQAHHELSTFVKIKNKANSDASWYFLDPTVSMSVTQKQPCICGSGEKFKRCCGMYI
00000000000000000000000000000000000000011111100000000000000000000000000000000000000
00000000000000000000000000000000000000000000000000000000000000000000000000000000000
00000000000000000
>Q4QG08_1y1x_51_233
MPTSTGVYAPSARHMNDNQELMEWFRAVDTDGSGAISVPELNAALSSAGVPFSLATTEKL
LHMYDKNHSGEITFDEFKDLHHFILSMREGFRKRDSSGDGRLDSNEVRAALLSSGYQVSEQ
TFQALMRKFDRQRRGSLGFDDYVELSIFVCRVRNVFAFYDRERTGQVTFTFDTFIGGSVSIL
10000000000000000000000000000000000000000000000000000000000000000000000000000000000
00000000000000000000000000000000000000000000000000000000000000000000000000000000000
00000000000000000
>Q7WRZ5_3efy_99_281
REKGIVEPSCGVTANAIMKLFLDKDGFSYCFENEQTLSLEQLQERLSCMPECKSFVLRVNDG
ALGHAYIVDIPKGENSCRPAFLYQSDLGEGVTRKLRFEDWMTHKALTPILLDDICNYFSCMS
QNKTDLEQIATLFDIDGNVKMLRKENIQYQKHDNFSFQLFEYDTDNIEKNIEIIKSLCS
11111110000000000000000000000000000000000000000000000000000000000000000000000000000
00000000000000000000000000000000000000000000000000000000000000000000000000000000000
00000000000000000
>Q80J95_4ash_994_1176

APVSIWSRVVQFGTGWGFWVSGHVFITAKHVAPPKGTEIFGRKPGDFTVTSSGDFLKYYFTS
AVRPDIPAMVLENGCQEGVVASVLVKRASGEMLALAVRMGSQAAIKIGSAVVHGQTGMLL
TGSNAKAQDLGTIPGDCGCPYVYKKGNTWVVIGVHVAATRSGNTVIAATHGEPTLEALEF
Q
00000000000000000000000000000000000000000000000000000000000000000000000000000000000
00000000000000000000000000000000000000001111110000000000000000000000000000000000000
00000000000000000
>Q8NT71_3lwa_24_206
SDSTAGTDAVAVGGTFQFHSPDGKMEIFYDEADRQQLPDIGGDSLMEEGTQINLSDFENQV
VILNAWGQWCAPCRSESDDLQIIHEELQAAGNGDTPGGTVLGINVRDYSRDIAQDFVTDN
GLDYPSIYDPPFMTAASLGGVPASVIPTTIVLDKQHRPAAVFLREVTSKDVLDVALPLVDEA
11111111111100000000000000000000000000000000000000000000000000000000000000000000000
00000000011100000000000000000000000000000000000000000000000000000000000000000000000
00000000000000001
>Q9RUG7_2vli_1_183
TPMRSPIIWINGPFGVGKTHTAHTLHERLPGSFVFEPEEMGQALRKLTPGFSGDPQEHPMW
IPLMLDALQYASREAAGPLIVPVSISDTARHRRLMSGLKDRGLSVHHFTLIAPLNVVLERLR
RDGQPQVNVGTVEDRLNELRGEQFQTHIDTAGLGTQQVAEQIAAQVGLTLAPPPQGALH
W
11100000000000000000000000000000000000000000000000000000000000000000000000000000000
00000000000000000000000000000000000000000000000000000000000000000000000000000000000
00000000001111111
>Q9S3Q1_2r2c_107_289
RTEAGDAYYSKFANNTPLPALMVSRKKFGSSYVYDKSYKTWDVPIAEQMEQKAKINIFAVA
EYTDTQKIKVTVKGKILEGNTLPKSMVQVYLLEDKLIAPQVDGNTTVENYEHNHVLRGAV
NGIWGEEFVNLKDYLYTYAVEPLSGMSFVAENYSIVAFVYDVQTFEVYDVVHVKINPQSDG
K
11111111111111111111111111111111111000000000011111100000000000000000000000000000000
00000000000001111111111111111100000000000000000000000000000000000000000000000000000
00000000000000111
>P02763_3kq0_19_200
IPLCANLVPVPITNATLDQITGKWFYIASAFRNEEYNKSVQEIQATFFYFTPNKTEDTIFLREY
QTRQDQCIYNTTYLNVQRENGTISRYVGGQEHFAHLLILRDTKTYMLAFDVNDEKNWGLS
VYADKPETTKEQLGEFYEALDCLRIPKSDVVYTDWKKDKCEPLEKQHEKERKQEEGES
00000000000000000000000000000000000000000000000000000000000000000000000000000000000
00000000000000000000000000000000000000000000000000000000000000000000000000000000000
0000000011111111
>P05198_1kl9_1_182
PGLSCRFYQHKFPEVEDVVMVNVRSIAEMGAYVSLLEYNNIEGMILLSELSRRRIRSINKLIRI
GRNECVVVIRVDKEKGYIDLSKRRVSPEEAIKCEDKFTKSKTVYSILRHVAEVLEYTKDEQLE
SLFQRTAWVFDDKYKRPGYGAYDAFKHAVSDPSILDSLDLNEDEREVLINNINRR
11000000000000000000000000000000000000000000000000111111111111000000000000000000000
00000000000000000000000000000000000000000000000000000000000000000000000000000000000
0000000000000000
>P07360_2ova_20_201
QKPQRPRRPASPISTIQPKANFDAQQFAGTWLLVAVGSACRFLQEQGHRAEATTLHVAPQG
TAMAVSTFRKLDGICWQVRQLYGDTGVLGRFLLQARDARGAVHVVVAETDYQSFAVLYLE
RAGQLSVKLYARSLPVSDSVLSGFEQRVQEAHLTEDQIFYFPKYGFCEAADQFHVLDEVRR
11111111000000000000000000000000000000000111111000000000000000000000000000000000000
00000000000000000000000000000000000000000000000000000000000000000000000000000000000
0000000000000011

>P44882_1izm_0_181
MLISHSDLNQQLKSAGIGFNATELHGFLSGLLCGGLKDQSWLPLLYQFSNDNHAYPTGLVQ
PVTELYEQISQTLSDVEGFTFELGLTEDENVFTQADSLSDWANQFLLGIGLAQPELAKEKGEI
GEAVDDLQDICQLGYDEDDNEEELAEALEEIIEYVRTIAMLFYSHFNEGEIESKPVLH
10000000000000000000000000000000000000000000000000000000000000000000000000000000000
00000000000000000000000000000000000000000000000000000000000000000000000000000000000
0000011111111111
>Q03164_3lqh_1565_1783
GNFCPLCDKCYDDDDYESKMMQCGKCDRWVHSKCENLSDEMYEILSNLPESVAYTCVNC
TERHPAEWRLALEKELQISLKQVLTALLNSRTTSHLLRYRQQQPLDLEGVKRKMDQGNYTS
VLEFSDDIVKIIQAAINSDGGQPEIKKANSMVKSFFIRQMERVFPWFSVKKSRFWEPNKVSS
00000000000000000000000000000000000000000000000000000000000000000000000000000000000
00000000000000001100000000000000000000000000000000000000000000000000000000000000000
0000000000111111
>Q06418_1rhf_40_221
AGLKLMGAPVKLTVSQGQPVKLNCSVEGMEEPDIQWVKDGAVVQNLDQLYIPVSEQHWI
GFLSLKSVERSDAGRYWCQVEDGGETEISQPVWLTVEGVPFFTVEPKDLAVPPNAPFQLSCE
AVGPPEPVTIVWWRGTTKIGGPAPSPSVLNVTGVTQSTMFSCEAHNLKGLASSRTATVHLQ
11111100000000000000000000000000000000000000000000000000000000000000000000000000000
00000000000000000000000000000000000000000000000000000000000000000000000000000000000
0000000000000000
>Q4A3Y2_2zf9_29_210
GPAAGQAYDAGNLDVASSPVKPTLSITKKTLTAAEAPNAKVTMELSVEGAADKYAATGLH
IQFDPKLKLIPDEDGALATAGRAARLLELKKAEADTDNSFFTATGSSTNNGKDGVLWSFVL
QVPADAQPGDKYDVQVAYQSRTTNEDLFTNVKKDEEGLLMQAWTFTQGIEQGYIQVESTT
S
00000000000000000000000000000000000000000000000000000000000000000000000000000000000
00000000000000000000000000000000000000000000000000000000000000000000000000000000000
0000000000000011
>Q5F6Z8_3k5j_0_181
MDYNQTVLSHLQKFWKHHDIKGFTWTLGRIVEELPDFQVFQVIPNHEDEPWVYVSSGIGQ
FLGQEFFIISPFETPEHIETLAMLASASMHYPDQFQLGKTVNIGRPWVEQSSFRHFLISLPYPY
GQELEYMDNVRFFWLLPITQTERLFLNTHSVEELETKFDEAGIDYLDINRASTVWQAG
00000000000000000000000000000000000000000000000000000000000000000000000000000000000
00000000000000000000000000000000000000000000000000000000000000000000000000000000000
0000000000000001
>Q5M2Z6_3mti_0_181
MIKRPIHMSHDFLAEVLDDESIVVDATMGNGNDTAFLAGLSKKVYAFDVQEQALGKTSQR
LSDLGIENTELILDGHENLDHYVREPIRAAIFNLGYLPSADKSVITKPHTTLEAIEKILDRLEV
GGRLAIMIYYGHDGGDMEKDAVLEYVIGLDQRVFTAMLYQPLNQINTPPFLVMLEKLQ
00000000000000000000000000000000000000000000000000000000000000000000000000000000000
00000000000000000000111000000000000000000000000000000000000000000000000000000000000
0000000000000000
>Q8B0H2_1lg7_47_228
VDEMDTHDPNQLRYEKFFFTVKMTVRSNRPFRTYSDVAAAVSHWDHMYIGMAGKRPFYK
ILAFLGSSNLKATPAVLADRGQPEYHAHCEGRAYLPHRMGKTPPMLNVPEHFRRPFNIGLY
KGTVELTMTIYDDESLEAAPMIWDHFNSSKFSDFREKALMFGLIVEKKASGAWVLDSVSHF
K
11111111110000000000000000000000000000000000000000000000000000000000000000111111000
00000000000000000000000000000000000000000000000000000000000000000000000000000000000
0000000000000011

>Q8DYC0_2pc1_0_181
MQIRLAFPNEIDQIMLLIEEARAEIAKTGSDQWQKEDGYPNRNDIIDDILNGYAWVGIEDG
MLATYAAVIDGHEEVYDAIYEGKWLHDNHRYLTFHRIAISNQFRGRGLAQTFLQGLIEGHK
GPDFRCDTHEKNVTMQHILNKLGYQYCGKVPLDGVRLAYQKIKEKGETSIYREIDERNPM
00000000000000000000000000000000000000000000000000000000000000000000000000000000000
00000000000000000000000000000000000000000000000000000000000000000000000000000000000
0111111111111111
>Q8GME2_4hx6_0_181
MSPIIAPPAELVDPKDRVQLRRVFGDFPTGVTVVTVGGSEPRGMTANSFTSVSLSPPLVLICV
GKDAVMHQRLTALPTFAVSVLEAGQEKAARHFADHSRPPGVDQFDTVDWVLGEESGAPLI
AGAVAHLECAIHRLYEGGDHTIFLGEVITATRWPAREGMLFSGGRFRRFAPDADEGRAA
00000000000000000000000000000000000000000000000000000000000000000000000000000000000
00000000000000000000000000000000000000000000000000000000000000000000000000000000000
0000000001111111
>O28839_2qh9_0_180
MKKWRFLGIDDSFDDRKCCVVGCVTCGGYVEGFLYTEIDIDGLDATDKLISMVRRSKFREQI
KCIFLPGITLGGFNLVDIQRVYRETKIPVVVVMRRKPDMEEFDSAMRNLENYELRRKIVEVA
GEIHRIGDIYIQTAGLTPSEAEKLVKASLIKGNMPEPVRISHLVASAIIHGESRGKA
00000000000000000000000000000000000000000000000000000000000000000000000000000000000
00000000000000000000000000000000000000000000000000000000000000000000000000000000000
000000000111111
>P37957_1t2n_31_211
AEHNPVVMVHGIGGASFNFAGIKSYLVSQGWSRDKLYAVDFWDKTGTNYNNGPVLSRFV
QKVLDETGAKKVDIVAHSMGGANTLYYIKNLDGGNKVANVVTLGGANRLTTGKALPGTD
PNQKILYTSIYSSADMIVMNYLSRLDGARNVQIHGVGHIGLLYSSQVNSLIKEGLNGGGQNT
N
11000000000000000000000000000000000000000000000000000000000000000000000000000000000
00000000000000000000000000000000000000000000000000000000000000000000000000000000000
000000000000000
>P80029_1s2p_0_180
DKIPDFVVPGKCASVDRNKLWAEQTPNRNSYAGVWYQFALTNNPYQLIEKCVRNEYSFDG
KQFVIKSTGIAYDGNLLKRNGKLYPNPFGEPHLSIDYENSFAAPLVILETDYSNYACLYSCIDY
NFGYHSDFSFIFSRSANLADQYVKKCEAAFKNINVDTTRFVKTVQGSSCPYDTQKTL
10000000000000000000000000000000000000000000000000000000000000000000000000000000000
00000000000000000000000000000000000000000000000000000000000000000000000000000000000
000000000000000
>P82186_1wc2_0_180
NQKCSGNPRRYNGKSCASTTNYHDSHKGACGCGPASGDAQFGWNAGSFVAAASQMYFD
SGNKGWCGQHCGQCIKLTTTGGYVPGQGGPVREGLSKTFMITNLCPNIYPNQDWCNQGS
QYGGHNKYGYELHLDLENGRSQVTGMGWNNPETTWEVVNCDSEHNHDHRTPSNSMYG
QCQCAHQ
00000000000000000000000000000000000000000000000000000000000000000000000000000000000
00000000000000000000000000000000000000000000000000000000000000000000000000000000000
000000000000001
>P84148_1q42_0_180
MNQDPTQQLEPFLKRFLASLDLLYTQPTSQPFFPNVESYATQLGSNLKRSSAIIVNGQPIIPSPQ
EDCKLQFQKKWLQTPLSSHQLTSYDGHLIPGTGTFVVHFSAKVRFDQSGRNRLGESADLFQ
ENNSIVSKTNQRPIWGSWFGVDVNLVVDENVMQDGEIINSMDYRFTYVPNDSIIKV
11000000000000000000000000000000000000000000000000000000000000000000000000000000000
00000000000000000000000000000000000000111111111111110000000000000000000000000000000
000000000011111

>P96726_1rli_0_180
MKIAVINGGTRSGGNTDVLAEKAVQGFDAEHIYLQKYPIQPIEDLRHAQGGFRPVQDDYDS
IIERILQCHILIFATPIYWFGMSGTLKLFIDRWSQTLRDPRFPDFKQQMSVKQAYVIAVGGDN
PKIKGLPLIQQFEHIFHFMGMSFKGYVLGEGNRPGDILRDHQALSAASRLLKRSDAI
10000000000000000000000000000000000000111111111100000000000000000000000000000000000
00000000000000000000000000000000000000000000000000000000000000000000000000000000000
000000000000011
>Q5MYR6_1xiy_59_239
KENDLIPNVKVMIDVRNMNNISDTDGSPNDFTSIDTHELFNNKKILLISLPGAFTPTCSTKMI
PGYEEEYDYFIKENNFDDIYCITNNDIYVLKSWFKSMDIKKIKYISDGNSSFTESMNMLVDKS
NFFMGMRPWRFVAIVENNILVKMFQEKDKQHNIQTDPYDISTVNNVKEFLKNNQL
00000000000000000000000000000000000000000000000000000000000000000000000000000000000
00000000000000000000000000000000000000000000000000000000000000000000000000000000000
000000000000011
>Q86US8_2hwx_1238_1418
MELEIRPLFLVPDTNGFIDHLASLARLLESRKYILVVPLIVINELDGLAKGQETDHRAGGYAR
VVQEKARKSIEFLEQRFESRDSCLRALTSRGNELESIAFRSEDITGQLGNNDDLILSCCLHYC
KDKAKDFMPASKEEPIRLLREVVLLTDDRNLRVKALTRNVPVRDIPAFLTWAQVG
00000000000000000000000000000000000000000000000000011111000000000000000000000000000
00000000000000000000000000011000000000000000000000011111100000000000000000000000000
000000000000011
>Q87RG9_3c85_348_528
APLNRLGHKIYQHSGKWLQETAAEKLNQRDQLINPGHAQVLILGMGRIGTGAYDELRARY
GKISLGIEIREEAAQQHRSEGRNVISGDATDPDFWERILDTGHVKLVLLAMPHHQGNQTAL
EQLQRRNYKGQIAAIAEYPDQLEGLLESGVDAAFNIYSEAGSGFARHVCKQLEPQFTSIK
11111111111111111111111111111110000000000000000000000000000000000000000000000000000
00000000000000000000000000000000000000000000000000000000000000000000000000000000000
000000000000001
>Q96AZ6_1wlj_0_180
MAGSREVVAMDCEMVGLGPHRESGLARCSLVNVHGAVLYDKFIRPEGEITDYRTRVSGVTP
QHMVGATPFAVARLEILQLLKGKLVVGHDLKHDFQALKEDMSGYTIYDTSTDRLLWREAK
LDHCRRVSLRVLSERLLHKSIQNSLLGHSSVEDARATMELYQISQRIRARRGLPRLAVSD
11111000000000000000000000000000000000000000000000000000000000000000000000000000000
00000000000000000000000000000000000000011111000000000000000000000000000000000000000
000000000000111
>A7M2I6_4e6f_24_203
DSAKEKKDDTRYLVGAVPEVDGKVVFSKEFQIPGMSQAQIYDTMTKWMDERLKENKNIDS
RIVFSDEAKGTIAGVGEEWIVFSSSALSLDRTLVNYQITVTCKPGNCLVELEKIRFTYRETEKY
KAEEWITDKYALNKAKTKLVRGLAKWRRKTVDFADDMFMDVAVAFGAPDTRPKTEK
11111000000000000000000000000000000000000000000000000000000000000000000000000000000
00000000000000000000000000000000000000000000000000000000000000000000000000000000000
00000000000000
>P48329_3sid_0_179
MGLNYNQEDFMGLDRFFQDAVSHNNTDANAASSIEVEMYECDCMYPTFAEIARRSGQPEI
GAMFDAIAKEEGMHAQLLTKLYSELEVKDSAETLEAKRLVSTIESQIDAVASDSRGLRRALE
TALEVETIESQKTYPAFAKLAAEQGNMEVATAFEAIVKSETKHANWVKRALENLLEVA
11100000000000000000000000000000000000000000000000000000000000000000000000000000000
00000000000000000000000000000000000000000000000000000000000000000000000000000000000
00000000000000
>P49051_3pyw_30_209
GKTFPDVPADHWGIDSINYLVEKGAVKGNDKGMFEPGKELTRAEAATMMAQILNLPIDKD

AKPSFADSQGQWYTPFIAAVEKAGVIKGTGNGFEPNGKIDRVSMASLLVEAYKLDTKVNGT
PATKFKDLETLNWGKEKANILVELGISVGTGDQWEPKKTVTKAEAAQFIAKTDKQFGTE
100000000000000000000000000000000000000000000000000000000000000000000000000000000000
000000000000000000000000000000000000000000000000000000000000000000000000000000000000
00000000000001
>Q3JXA5_3rqi_0_179
MSDKNFLVIDDNEVFAGTLARGLERRGYAVRQAHNKDEALKLAGAEKFEFITVDLHLGND
SGLSLIAPLCDLQPDARILVLTGYASIATAVQAVKDGADNYLAKPANVESILAALQTNASEV
QAEEALENPVVLSVDRLEWEHIQRVLAENNNNISATARALNMHRRTLQRKLAKKPVRQ
110000000000000000000000000000000000000000000000000000000000000000000000000000000000
000000000000000000000000000000000000000000000000000011100000000000000000000000000000
00000000011111
>Q4Q7A6_1y63_0_179
MEQPKGINILITGTPGTGKTSMAEMIAAELDGFQHLEVGKLVKENHFYTEYDTELDTHIIEE
KDEDRLLDFMEPIMVSRGNHVVDYHSSELFPERWFHMVVVLHTSTEVLFERLTKRQYSEAK
RAENMEAEIQCICEEEARDAYEDDIVLVRENDTLEQMAATVEEIRERVEVLKVERGL
100000000000000000000000000000000000000000000000001111100000000000000000000000000000
000000000000000000000000000000000000000000000000000000000000000000000000000000000000
00000000011111
>Q57930_2z8u_3_182
MEPEIKIVNVVVSTKIGDNIDLEEVAMILENAEYEPEQFPGLVCRLSVPKVALLIFRSGKVNC
TGAKSKEEAEIAIKKIIKELKDAGIDVIENPEIKIQNMVATADLGIEPNLDDIALMVEGTEYEP
EQFPGLVYRLDDPKVVVLIFGSGKVVITGLKSEEDAKRALKKILDTIKEVQEL
110000000000000000000000000000000000000000000000000000000000000000000000000000000000
000000000000000000000000000000000000000000000000000000000000000000000000000000000000
00000000000111
>Q8XNK4_2vnr_26_205
EVYALEESRDVYLSDLDWLNATHGDDTKSKIVQKNHPFTPGNNNQSTKISLKMEDGSISEF
EKGLGTIAGSPSTITYDISGAGVTKFFSYLGIDRSANPINEQYAKVDKIEVVVDGKVIYSTINQ
FPNGLTYETPAIKVDLNIPENAKRLQLKSYAGEKTWGDEVVYADAKFTAKGDFVN
111111100000000000000000000000000000000000000000000000000000000000000000000000000000
000000000000000000000000000000000000000000000000000000000000000000000000000000000000
00000000000000
>Q967M2_1l1q_0_179
MTMSVADAHALIKTIPDFPTKGIAFKDLSDILSTPAALDAVRKEVTAHYKDVPITKVVGIESR
GFILGGIVANSLGVGFVALRKAGKLPGDVCKCTFDMEYQKGVTIEVQKRQLGPHDVVLLH
DDVLATGGTLLAAIELCETAGVKPENIYINVLYEIEALKGREKVGQKCTRLFSVIRE
100000000000000000000000000000000000000000000000000000000000000000000000000000000000
000000000000000000000000000000000000000000000000000000000000000000000000000000000000
00000000000000
>Q96G74_3tmp_171_350
GAGYNSEDEYEAAAARIEAMDPATVEQQEHWFEKALRDKKGFIIKQMKEDGACLFRAVA
DQVYGDQDMHEVVRKHCMDYLMKNADYFSNYVTEDFTTYINRKRKNNCHGNHIEMQA
MAEMYNRPVEVYQYSTGTSAVEPINTFHGIHQNEDEPIRVSYHRNIHYNSVVNPNKATIGV
GLG
000000000000000000000000000000000000000000000000000000000000000000000000000000000000
000000000000000000000000000000000000000000000000000000000000000000000000000000000000
00001111111111
>Q9Y221_1sqw_0_179
MRPLTEEETRVMFEKIAKYIGENLQLLVDRPDGTYCFRLHNDRVYYVSEKIMKLAANISGD
KLVSLGTCFGKFTKTHKFRLHVTALDYLAPYAKYKVWIKPGAEQSFLYGNHVLKSGLGRITE

NTSQYQGVVVYSMADIPLGFGVAAKSTQDCRKVDPMAIVVFHQADIGEYVRHEETLT
00000000000000000000000000000000000000000000000000000000000000000000000000000000000
00000000000000000000000000000000000000000000000000000000000000000000000000000000000
00000000001111
>A3FQ69_2q2g_147_325
APRSHEVPLLVTLEELYLGKRKKIKVTRKRFIEHKVRNEENIVEVEIKPGWKDGTKLTYSGEG
DQESPGTSPGDLVLIIQTKTHPRFTRDDCHLIMKVTIPLVRALTGFTCPVTTLDNRNLQIPIKE
IVNPKTRKIVPNEGMPIKNQPGQKGDLILEFDICFPKSLTPEQKKLIKEALD
11000000000000000000000000000000000000000000000000000000000000000000000000000000000
00000000000000000000000000000000000000000000000000000000000000000000000000000000000
0000000000001
>A6L094_3can_123_301
GGGVTFCGGEPLLHPEFLIDILKRCGQQGIHRAVDTTLLARKETVDEVMRNCELLLIDLKSM
DSTVHQTFCDVPNELILKNIRRVAEADFPYYIRIPLIEGVNADEKNIKLSAEFLASLPRHPEIIN
LLPYHDIGKGKHAKLGSIYNPKGYKMQTPSEEVQQQCIQILTDYGLKATIGG
00000000000000000000000000000000000000000000000000000000000000000000000000000000000
00000000000000000000000000000000000000000000000000111111111111111111000000000000000
0000000000000
>B6JPK4_3ub7_29_207
KVMQKDVLAQLMEHLETGQYKKREKTLAYMTKILEQGIHEYYKNFDNATARKMALDYFK
RINDDKGMIYMVVVDKNGVVLFDPVNPKTVGQSGLDAQSVDGVYYVRGYLEAAKKGGG
YTYYKMPKYDGGVPEKKFAYSHYDEVSQMVIAATSYYTDINTENQAIKEGVNKVFNENTT
RL
11111110000000000000000000000000000000000000000000000000000000000000000000000000000
00000000000000000000000000000000000000000000000000000000000000000000000000000000000
0001111111111
>O00762_1i7k_0_178
MASQNRDPAATSVAAARKGAEPSGGAARGPVGKRLQQELMTLMMSGDKGISAFPESDNL
FKWVGTIHGAAGTVYEDLRYKLSLEFPSGYPYNAPTVKFLTPCYHPNVDTQGNICLDILKE
KWSALYDVRTILLSIQSLLGEPNIDSPLNTHAAELWKNPTAFKKYLQETYSKQVTSQEP
11111111111111111111111111111100000000000000000000000000000000000000000000000000000
00000000000000000000000000000000000000000000000000000000000000000000000000000000000
0000000001111
>O67157_2eh3_0_178
MGTKERILEVSKELFFEKGYQGTSVEEIVKRANLSKGAFYFHFKSKEELITEIIERTHKKIISLFE
ENKEKTPEELLEMFLEVLYREKKVVYIFLFDLLCSEKFRNIYFEKIEDAKRRFEKFLEKHFPSK
AEILSEIILGFLRQLILHYVIKEERELPFLKEKLREGLKLIFEGVKKCG
10000000000000000000000000000000000000000000000000000000000000000000000000000000000
00000000000000000000000000000000000000000000000000000000000000000000000000000000000
0000001111111
>P29394_1zav_0_178
MLTRQQKELIVKEMSEIFKKTSLILFADFLGFTVADLTELRSRLREKYGDGARFRVVKNTLLN
LALKNAEYEGYEEFLKGPTAVLYVTEGDPVEAVKIIYNFYKDKKADLSRLKGGFLEGKKFTA
EEVENIAKLPSKEELYAMLVGRVKAPITGLVFALSGILRNLVYVLNAIKEKKSE
00000000000000000000000000000000000000000000000000000000000000000000000000000000000
00000000000000000000000000000000000000000000000000000000000000000000000000000000000
0000000000011
>P61914_1iaz_35_213
SADVAGAVIDGASLSFDILKTVLEALGNVKRKIAVGVDNESGKTWTALNTYFRSGTSDIVLP
HKVPHGKALLYNGQKDRGPVATGAVGVLAYLMSDGNTLAVLFSVPYDYNWYSNWWNVR
IYKGKRRADQRMYEELYYNLSPFRGDNGWHTRNLGYGLKSRGFMNSSGHAILEIHVSKA

11110000000000000000000000000000000000000000000000000000000000000000000000000000000
00000000000000000000000000000000000000000000000000000000000000000000000000000000000
0000000000000
>Q7CH76_3n0z_0_178
MSEHFVGKYEVELKFRVMDLTTLHEQLVAQKATAFTLNNHEKDIYLDANGQDLADQQIS
MVLREMNPSGIRLWIVKGPGAERCEASNIEDVSKVQSMLATLGYHPAFTIEKQRSIYFVGKF
HITVDHLTGLGDFAEIAIMTDDATELDKLKAECRDFANTFGLQVDQQEPRSYRQLLGF
11100000000000000000000000000000000000000000000000000000000000000000000000000000000
00000000000000000000000000000000000000000000000000000000000000000000000000000000000
0000000000000
>Q7ZVT3_2y3w_0_178
MTELLFNKRLQVLVKSKDTDERRSVIRVSIELQLPSSPVHRKDLVVRLTDDTDLYFLYNLIISE
EDFQSLKVQQGLLIDFTSFPQKFIDLLEQCICEQDKESPRFLLQLSSSSSAFDHSPSNLNIVETN
AFKHLTHLSLKLLPGSDTDIKKYLASCLSSVKEEKQQLQQKLRKTEEDLT
00000000000000011111000000000000001111110000000000000000000000000000000000000000000
00000000000000000000000000001111100000000000000000000000000000000000000000000000000
0000000000111
>Q96ES7_3mev_114_292
RRGVLMTLLQQSAMTLPLWIGKPGDKPPPLCGAIPASGDYVARPGDKVAARVKAVDGDEQ
WILAEVVSYSHATNKYEVDDIDEEGKERHTLSRRRVIPLPQWKANPETDPEALFQKEQLVL
ALYPQTTCFYRALIHAPPQRPQDDYSVLFEDTSYADGYSPPLNVAQRYVVACKEPKKK
00000000000000000000000000000000000000000000000000000000000000000000000000000000000
00000000000000000000000000000000000000000000000000000000000000000000000000000000000
0000000000011
>Q9KQN0_1y9q_1_179
TDVMFKSQIANQLKNLRKSRGLSLDATAQLTGVSKAMLGQIERGESSPTIATLWKIASGLEA
SFSAFFANDPQLLSSERSFPDDLNMKIHTLFPYAADTGLEIFEITLLDHHQQMSSPHALGVIE
YIHVLEGIMKVFFDEQWHELQQGEHIRFFSDQPHGYAAVTEKAVFQNIVAYPRR
00000000000000000000000000000000000000000000000000000000000000000000000000000000000
00000000000000000000000000000000000000000000000000000000000000000000000000000000000
0000000000001
>Q9YC08_2cxh_1_179
TTSRRPSPRIRSFVKDLSATIPGAFRFTRGHYSMEELAREAIIRGADRIVVVGERRGNPGIIRVY
AVEGPERPDNIVSFIVKGVSLSRERRWGLPSLRGGEVLVARPLDSGVAVEFADAFVIAFHARL
KPPEAAGYVEAVIESLDARTVAVTFRYGGAPVGPMLRLGKPAEMVKRGRRV
00000000000000000000000000000000000000000000000000000000000000000000000000000000000
00000000000000000000000000000000000000000000000000000000000000000000000000000000000
0000000011111
>F9ULL2_2w2b_0_177
MTKTFKTLDDFLGTHFIYTYDNGWEYEWYAKNDHTVDYRIHGGMVAGRWVTDQKADIV
MLTEGIYKISWTEPTGTDVALDFMPNEKKLHGTIFFPKWVEEHPEITVTYQNEHIDLMEQSR
EKYATYPKLVVPEFANITYMGDAGQNNEDVISEAPYKEMPNDIRNGKYFDQNYHRLNK
10000000000000000000000000000000000000000000000000000000000000000000000000000000000
00000000000000000000000000000000000000000000000000000000000000000000000000000000000
000000000000
>O31562_1rxq_0_177
MTSVNLSYPIGEYKPRESISKEQKDKWIQVLEEVPAKLKQAVEVMTDSQLDTPYRDGGWTV
RQVVHHLADSHMNSYIRFKLSLTEETPAIRPYDEKAWSELKDSKTADPSGSLALLQELHGR
WTALLRTLTDQQFKRGFYHPDTKEIITLENALGLYVWHSHHHIAHITELSRRMGWS
11110000000000000000000000000000000000000000000000000000000000000000000000000000000
00000000000000000000000000000000000000000000000000000000000000000000000000000000000

000000000000
>O67322_1ye8_0_177
MKIIITGEPGVGKTTLVKKIVERLGKRAIGFWTEEVRDPETKKRTGFRIITTEGKKKIFSSKFFT
SKKLVGSYGVNVQYFEELAIPILERAYREAKKDRRKVIIIDEIGKMELFSKKFRDLVRQIMHDP
NVNVVATIPIRDVHPLVKEIRRLPGAVLIELTPENRDVILEDILSLLER
00000000000000000000000000000000000001111110000000000000000000000000000000000000000
00000000000000000000000000000000000000000000000000000000000000000000000000000000000
000000000000
>P52800_1iko_29_206
SIVLEPIYWNSSNSKFLPGQGLVLYPQIGDKLDIICPKVDSKTVGQYEYYKVYMVDKDQADR
CTIKKENTPLLNCARPDQDVKFTIKFQEFSPNLWGLEFQKNKDYYIISTSNGSLEGLDNQEG
GVCQTRAMKILMKVGQDASSAGSARNHGPTRRPELEAGTNGRSSTTSPFVKPNP
00000000000000000000000000000000000000000000000000000000000000000000000000000000000
00000000000000000000000000000000000000000000000000000000011111111111111111111111111
111111111111
>P53193_3uo3_4_181
LVQRRFTSTFYELFPKTFPKKLPIWTIDQSRLRKEYRQLQAQHHPDMAQQGSEQSSTLNQAY
HTLKDPLRRSQYMLKLLRNIDLTQEQTSNEVTTSDPQLLLKVLDIHDELSQMDDEAGVKLL
EKQNKERIQDIEAQLGQCYNDKDYAAAVKLTVELKYWYNLAKAFKDWAPGKQLEM
11100000000000000000000000000000000000000000000000000000000000000000000000000000000
00000000000000000000000000000000000000000000000000000000000000000000000000000000000
000000000001
>P83815_1wub_0_177
MKWNLDPSHTSIDFKVRHMGIASVRGSLKVLSGSVETDEAGRPIQVEAVIDAASIATGEPQR
DGHLRSADFLHAEQYPEIRFVSTQIEPLGGNRYRIQGNLTIRDITKPVTLEAEVSAPIKDPWG
MQRVAASASGQINRKDWNLTWNQVLELGALLVGEEVKFNLEVEAVAPAPVAAQ
00000000000000000000000000000000000000000000000000000000000000000000000000000000000
00000000000000000000000000000000000000000000000000000000000000000000000000000000000
000000000011
>Q722P7_2oh1_0_177
MNQNKITAGGLEFLVRFAAPTDRLKINDLMIDTARWLKESGSTQWSDILHGFDVHNIEQRI
ELGEVALFETEAGALAGAMIIRKTPSDWDTDLWEDLAIDKAYYLHRIMVSRAFSGISLSKQM
IYFAEKLGIEMSVPFIRLDCIESNETLNQMYVRYGFQFSGKKNGFYLYQKELSQK
11000000000000000000000000000000000000000000000000000000000000000000000000000000000
00000000000000000000000000000000000000000000000000000000000000000000000000000000000
000000000011
>Q8DTX1_3d4e_28_205
EDKKGGTKPSNEAALTKTENLDFRLSFNKIKVTTDQNHFSGGTSIEQLKQWFGDPNKSEQR
NAGNITLDSYTWVKDGAVINAQLYKNSTVARSISNFSFSREAKIGKEDYDELKIGESYKKVV
EKLGEPDVLSQSMSSDKEEMQTVWSSGIKTKSSSATIELYFENGLLKNKTQKDLE
11111111111111111000000000000000000000000000000000000000000000000000000000000000000
00000000000000000000000000000000000000000000000000000000000000000000000000000000000
000000000000
>Q9KQV9_2fck_0_177
MTPDFQIVTQRLQLRLITADEAEELVQCIRQSQTLHQWVDWCHALFSQQEAEQFIQATRLN
WVKAEAYGFGVFERQTQTLVGMVAINEFYHTFNMASLGYWIGDRYQRQGYGKEALTALIL
FCFERLELTRLEIVCDPENVPSQALALRCGANREQLAPNRFLYAGEPKAGIVFSLIP
00000000000000000000000000000000000000000111100000000000000000000000000000000000000
00000000000000000000000000000000000000000000000000000000000000000000000000000000000
000000000000
>A3TPB6_4eun_0_176

MTGEPTRHVVVMGVSGSGKTTIAHGVADETGLEFAEADAFHSPENIATMQRGIPLTDEDR
WPWLRSLAEWMDARADAGVSTIITCSALKRTYRDVLREGPPSVDFLHLDGPAEVIKGRMS
KREGHFMPASLLQSQLATLEALEPDESGIVLDLRQPPEQLIERALTWLDIAPAVATH
11110000000000000000000000000000000000000000000000000000000000000000000000000000000
00000000000000000000000000000000000000000000000000000000000000000000000000000000000
00000000000
>Q03E11_3jtw_0_176
MARKVILFIAMSIDNYIADDQGAVDWLEKNVHGTESDDSYEKMYSKIDTVIMGRTTYEQVT
QKLSPEKYVYADRQTYIVTSHLGEDTDKIKYWKQSPVELVKRIQKEKGKDVWIVGGAKLID
PLVQANLIDTYILTTVPIFLGSGIRLFDRLEEQVPVRLIDVYQKNELVYSIYQRG
10000000000000000000000000000000000000000000000000000000000000000000000000000000000
00000000000000000000000000000000000000000000000000000000000000000000000000000000000
00000000000
>Q47591_2r5o_254_430
ISSASGESQMSLDEIEDVYHTRPGYRPEEYRWGQGGAKIIDYHIQSAGVDFPPSLTGNQQTD
FLMKVVFEYDFDCVVPGILIKTLDGLFLYGTNSFLASEGRENISVSRGDVRVFKFSLPVDLNS
GDYLLSFGISAGNPQTDMTPLDRRYDSIILHVTKSMDFWGVIDLKSSFTSYQ
11111111100000000000000000000000000000000000000000000000000000000000000000000000000
00000000000000000000000000000000000000000000000000000000000000000000000000000000000
00000000001
>Q53WB3_2p6o_0_176
MELWLVRHGETLWNREGRLLGWTDLPLTAEGEAQARRLKGALPSLPAFSSDLLRARRTAEL
AGFSPRLYPELREIHFGALEGALWETLDPRYKEALLRFQGFHPPGGESLSAFQERVFRFLEGL
KAPAVLFTHGGVVRAVLRALGEDGLVPPGSAVAVDWPRRVLVRLALDGEEATG
00000000000000000000000000000000000000000000000000000000000000000000000000000000000
00000000000000000000000000000000000000000000000000000000000000000000000000000000000
00000111111
>Q82KE3_2qng_0_176
MNGLNKGIRKVELAVKWDPSPPGDPATDLDIVAATFLAGDAYGKPAYVVHFDSRSPDGTIY
LNRDSKDGKGFGWDEVMTLELNRLDSRYARVVVGVVIQQRDAHRTFVGVLNPGLRMREG
YTVLAEDDFGGVLGSTAATVGEFVRDDSGEWTFHPGIHGYDSDPATFARVMGGRQDS
11111000000000000000000000000000000000000000000000000000000000000000000000000000000
00000000000000000000000000000000000000000000000000000000000000000000000000000000000
00000000011
>Q87J40_3h05_0_176
MKKIAIFGSAFNPPSLGHKSVIESLSHFDLVLLEPSIAHAWGKNMLDYPIRCKLVDAFIKDM
GLSNVQRSDLEQALYQPGQSVTTYALLEKIQEIYPTADITFVIGPDNFFKFAKFYKAEEITERW
TVMACPEKVKIRSTDIRNALIEGKDISTYTTPTVSELLLNEGLYRETLSGK
00000000000000000000000000000000000001111110000000000000000000000000000000000111100
00000000000000000000000000000000000000000000000000000000000000000000000000000000000
00000000000
>Q8I4V8_2fbn_127_303
AKKSIYDYTDEEKVQSAFDIKEEGNEFFKKNEINEAIVKYKEALDFFIHTEEWDDQILLDKK
KNIEISCNLNLATCYNKNKDYPKAIDHASKVLKIDKNNVKALYKLGVANMYFGFLEEAKE
NLYKAASLNPNNLDIRNSYELCVNKLKEARKKDKLTFGGMFDKGPLYEEKKNSAN
00000000000000000000000000000000000000000000000000000000000000000000000000000000000
00000000000000000000000000000000000000000000000000000000000000000000000111111111111
11111111111
>Q8NMG3_2zoy_0_176
MRTSKKEMILRTAIDYIGEYSLETLSYDSLAEATGLSKSGLIYHFPSRHALLLGMHELLADDW
DKELRDITRDPEDPLERLRAVVVTLAENVSRPELLLLIDAPSHPDFLNAWRTVNHQWIPDT

DDLENDAHKRAVYLVQLAADGLFVHDYIHDDVLSKSKRQAMLETILELIPSQT
00000000000000000000000000000000000000000000000000000000000000000000000000000000000
00000000000000000000000000000000000000000000000000000000000000000000000000000000000
00000000011
>Q8YXY7_3w2z_220_396
AVSKVMEKILRVSNIDKIFQTTTQEIRQLLKCDRVAVYRFNPDWSGEFVAESVGSGWVKLVG
PDIKTVWEDTHLQETQGGRYRHQESFVVNDIYEAGHFSCHLEILEQFEIKAYIIVPVFAAEKL
WGLLAAYQNSGTREWVEWESSFLTQVGLQFGIAISHAEYLEQTRLQSEQMIR
00000000000000000000000000000000000000000000000000000000000000000000000000000000000
00000000000000000000000000000000000000000000000000000000000000000000000000000000000
00111111111
>Q9Y9D8_2yzk_9_185
LAKVLKKRGAVLRGDFVLSSGRRSSVYIDMRRLLGDESSYSVALDLLLEVGGQDLARSSAVI
GVATGGLPWAAMLALRLSKPLGYVRPERKGHGTLSQVEGDPPKGRVVVVDDVATTGTSIA
KSIEVLRSNGYTVGTALVLVDRGEGAGELLARMGVRLVSVATLKTILEKLGWGGE
00000000000000000000000000000000000000000000000000000000000000000000000000000000000
00000000000000000000000000000000000000000000000000000000000000000000000000000000000
00000000001
>P53370_3h95_140_315
SHQVGVAGAVFDESTRKILVVQDRNKLKNMWKFPGGLSEPEEDIGDTAVREVFEETGIKSEF
RSVLSIRQQHTNPGAFGKSDMYIICRLKPYSFTINFCQEECLRCEWMDLNDLAKTENTTPITS
RVARLLLYGYREGFDKIDLTVEELPAVYTGLFYKLYHKELPENYKTMKGID
110000000000000000000000000000000000000000000000000000000000000000000011111111100
00000000000000000000000000000000000000000000000000000000000000000000000000000000000
0000001111
>P54962_3ebk_6_181
ATDTLANEDCFRHESLVPNLDYERFRGSWIIAAGTSEALTQYKCWIDRFSYDDALVSKYTDS
QGKNRTTIRGRTKFEGNKFTIDYNDKGKAFSAPYSVLATDYENYAIVEGCPAAANGHVIYV
QIRFSVRRFHPKLGDKEMIQHYTLDQVNQHKKAIEEDLKHFNLKYEDLHSTCH
11111100000000000000000000000000000000000000000000000000000000000000000000000000000
00000000000000000000000000000000000000000000000000000000000000111110000000000000000
0000000000
>P87252_1khi_0_175
MGYYDDDAHGHVEADAAPRATTGTGTGSASQTVTIPCHHIRLGDILILQGRPCQVIRISTSA
ATGQHRYLGVDLFTKQLHEESSFVSNPAPSVVVQTMLGPVFKQYRVLDMQDGSIVAMTET
GDVKQNLPVIDQSSLWNRLQKAFESGRGSVRVLVVSDHGREMAVDMKVVHGSRL
11111111111111111111111111000000000000000000000000000000000000000000000000000000000
00000000000000000000000000000000000000000000000000000000000000000000000000000000000
0000000111
>Q30WV8_2r7h_0_175
MPQTLKPDTPAGTPAAGAVAFRRQVLPQDALLVRRVVESTGFFTPEEADVAQELVDEHLM
HGAACGYHFVFATEDDDMAGYACYGPTPATEGTYDLYWIAVAPHRQHSGLGRALLAEVV
HDVRLTGGRLLFAETSGIRKYAPTRRFYERAGFSAEAVLKAFYRAGDDKIIYRLEVA
11111111111111111000000000000000000000000000000000000000000000000000000000000000000
00000000000000000000000000000000000000000000000000000000000000000000000000000000000
0000000000
>Q73EI2_4jg2_3_178
QNRTKVVTSVNTRLSYFHGWEPVSINGGAEKYSVSVLIPKTDKETINAINAAVDAAIEEGIA
KFGGKKPNKAAIKLPLRDGDVERDDEAYKGHYFVNANSKTPPQIVDKAVRPILDRNEVYS
GCYARVSLNFYAFNSNGNKGVACGLGNIQKIRDGEPLGGRTNAADDFTTIEDDD
00000000000000000000000000000000000000000000000000000000000000000000000000000000000

00000000000000000000000000000000000000000000000000000000000000000000000000000000000
0000000111
>Q91CD4_1jsd_338_513
GLFGAIAGFIEGGWPGLVAGWYGFQHSNDQGVGMAADRDSTQKAIDKITSKVNNIVDKM
NKQYGIIDHEFSEIETRLNMINNKIDDQIQDIWTYNAELLVLLENQKTLDEHDANVNNLYN
KVKRALGSNAMEDGKGCFELYHKCDDQCMETIRNGTYNRRKYKEESKLERQKIEGI
00000000000000000000000000000000000000000000000000000000000000000000000000000000000
00000000000000000000000000000000000000000000000000000000000000000000000000000111111
1111111111
>Q9EYL5_3bck_23_198
SATTSSKNGKPLVVVYGDYKCPYCKELDEKVMPKLRKNYIDNHKVEYQFVNLAFLGKDSIV
GSRASHAVLMYAPKSFLDFQKQLFAAQQDENKEWLTKELLDKHIKQLHLDKETENKIIKD
YKTKDSKSWKAAEKDKKIAKDNHIKTTPTAFINGEKVEDPYDYESYEKLLKDKIK
11111111000000000000000000000000000000000000000000000000000000000000000000000000000
00000000000000000000000000000000000000000000000000000000000000000000000000000000000
0000001111
>Q9RIK9_1oh4_504_679
NEARYVLAEEVDFSSPEEVKNWWNSGTWQAEFGSPDIEWNGEVGNGALQLNVKLPGKSD
WEEVRVARKFERLSECEILEYDIYIPNVEGLKGRLRPYAVLNPGWVKIGLDMNNANVESAEI
ITFGGKEYRRFHVRIEFDRTAGVKELHIGVVGDHLRYDGPIFIDNVRLYKRTGGM
11000000000000000000000000000000000000000000000000000000000000000000000000000000000
00000000000000000000000000000000000000000000000000000000000000000000000000000000000
0000000000
>Q9RVD0_3bt5_21_196
LRPPLPPATPQEVQFVQHMLQHHAQALDLAAPMLERSQQRTVRSLALDIQLSQREQMRQ
MEAMLGRWGQPPGEPISPEHARMMGMASEAEVAGLSTLPVEQAERQFLRLMIRHHQGAV
AMTLPMLDAAARPEVERLARQIVVTQRGEIRTMEGVLGRLDGEVPAAPMRPVEHGHGH
11111000000000000000000000000000000000000000000000000000000000000000000000000000000
00000000000000000000000000000000000000000001100000000000000000000000000000011111111
1111111111
>Q9X1F0_1vkf_0_175
MFKGIIAALWDMDSIGEIEPDVVFLLKSDILNLKFHLKILKDRGKTVFVDMDFVNGLGEGEE
AILFVKKAGADGIITIKPKNYVVAKKNGIPAVLRFFALDSKAVERGIEQIETLGVDVVEVLPG
AVAPKVARKIPGRTVIAAGLVETEEEAREILKHVSAISTSSRILWKMKFLP
00000000000000000000000000000000000000000000000000000000000000000000000000000000000
00000000000000000000000000000000000000000000000000000000000000000000000000000000000
0000000111
>O31851_1xtm_21_195
KPPDPPNRVPEKKVVETSAFGHHVQLVNREGKAVGFIEIKESDDEGLDIHISANSLRPGASL
GFHIYEKGSCVRPDFESAGGPFNPLNKEHGFNNPMGHHAGDLPNLEVGADGKVDVIMN
APDTSLKKGSKLNILDEDGSAFIIHEQADDYLTNPSGNSGARIVCGALLGNNEKQ
11111111111111111000000000000000000000000000000000000000000000000000000000000000000
00000000000000000000000000000000000000000000000000000000000000000000000000000000000
000011111
>P15465_1wba_0_174
ADDPVYDAEGNKLVNRGKYTIVSFSDGAGIDVVATGNENPEDPLSIVKSTRNIMYATSISSED
KTPPQPRNILENMRLKINFATDPHKGDVWSVVDFQPDGQQLKLAGRYPNQVKGAFTIQK
GSNTPRTYKLLFCPVGSPCKNIGISTDPEGKKRLVVSYQSDPLVVKFHRHEPE
10000000000000000000000000000000000000000000000000000000000000000000000000000000000
00000000000000000000000000000000000000000000000000000000000000000000000000000000000
000000111

>Q5SHW7_1vch_0_174
METYPITVGGVTRHVPLIEPLPGRRIPLVEFLGDPEFTRAAAEALRPLVPKEAEILFTTETSPIP
LTHVLAEALGLPYVVARRRRRPYMEDPIIQEVQTLTLGVGEVLWLDRRFAEKLLNQRVVLV
SDVVASGETMRAMEKMVLRAGGHVVARLAVFRQGTPGLAVDTVAELPVL
00000000000000000000000000000000000000000000000000000000000000000000000000000000000
00000000000000001111100000000000000000000000000000000000000000000000000000000000000
000000000
>Q8A8A4_3cne_0_174
MAKKVAVLAVNPVNGCGLFQYLEAFFENGISYKVFAVSDTKEIKTNSGMVLIVDDVIANLK
GHEDEFDALVFSCGDAVPVFQQYANQPYNVDLMEVIKTFGEKGKMMIGHCAGAMMFDF
TGITKGKKVAVHPLAKPAIQNGIATDEKSEIDGNFFTAQDENTIWTMLPKVIEALK
10000000000000000000000000000000000000000000000000000000000000000000000000000000000
00000000000000000000000000000000000000000000000000000000000000000000000000000000000
000000000
>Q8TXW1_2x7m_0_174
MKLCLVAFDGRIPMLSSIVDRFEEHVSEYLGEVKVKKKRAKLPEHAYSKVRGQYLARALLD
TLRGMKGEYDRVLGLTSEDLYAPGLNFVFGQARCPGREAVVSVARLLDPDPELYLERVVKE
LTHELGHTFGLGHCPDRNCVMSFSSSLLEVDRKSPNFCRRCTELLQRNLKRGG
00000000000000000000000000000000000000000000000000000000000000000000000000000000000
00000000000000000000000000000000000000000000000000000000000000000000000000000000000
000000011
>A1L259_2o74_0_173
MDINVVNALAYEDFVKLFGNVVEKCPLISAAIWSYRPFKDLADIEARISEFIHSLPDSGKEGI
LRCHPDLAGRDLQSGTLTPESQEEQSQAGMTTLDSAEIVHMYRLNSEYKERFGFPFVICARL
NNKADIVRQLSERLKNRRTAELECAIEEVKKICSLRLHSIVLSDIQTKL
10000000000000000000000000000000000000000000000000000000000000000000000000000000000
00000000000000000000000000000000000000000000000000000000000000000000000000000000000
11111111
>B7AHW8_4gdz_22_195
QESSTQRRADRKAQRDAERARLKAEQQAADAVSYDDAVAALKAQQFVLEANQVMFRNG
QTAFVTSNTNFVLVNQGRGTVQVAFNTVYPGPNGIGGVTVDGTVSDIKTSTDKRGNINCSF
SIQGIGISAQIFLTLTNGDNNATVTINPNFNSNTMTLSGSLLPLNQSNIFKGRSW
11111111111111111111111110000000000000000000000000000000000000000000000000000000000
00000000000000000000000000000000000000000000000000000000000000000000000000000000000
00000000
>D5U596_4hcj_0_173
MGKTNNILYVMSGQNFQDEEYFESKKIFESAGYKTKVSSTFIGTAQGKLGGMTNIDLLFSEV
DAVEFDAVVFVGGIGCITLWDDWRTQGLAKLFLDNQKIVAGIGSGVVIMANAKILEEINVT
CLSADESHVRHGNANIMSENVVVSGNIVTANGPTSSKDFANAVVGVLNSLS
10000000000000000000000000000000000000000000000000000000000000000000000000000000000
00000000000000000000000000000000000000000000000000000000000000000000000000000000000
00000000
>O74023_1mgt_0_173
MLSVEKFRVGERVVWIGVIFSGRVQGIAFAFDRGTLMKRIHDLAEHLGKRGVSISLDVQPSD
YPEKVFKVLIGELDNASFLRELSFEGVTPFEKKVYEWLTKNVKRGSVITYGDLAKALNTSPR
AVGGAMKRNPYPIVVPCHRVVAHDGIGYYSSGIEEKKFLLEIEGVKEWTS
00000000000000000000000000000000000000000000000000000000000000000000000000000000000
00000000000000000000000000000000000000000000000000000000000000000000000000000000000
00011111
>P35833_1bgc_21_194
TPLGPARSLPQSFLLKCLEQVRKIQADGAELQERLCAAHKLCHPEELMLLRHSLGIPQAPLS

SCSSQSLQLTSCLNQLHGGLFLYQGLLQALAGISPELAPTLDTLQLDVTDFATNIWLQMEDL
GAAPAVQPTQGAMPTFTSAFQRRAGGVLVASQLHRFLELAYRGLRYLAEP
11111110000000000000000000000000000000000000000000000000000000000000000000000000
00000000000000000000000000000000000000000000111111100000000000000000000000000000
00000011
>P51513_2anr_48_248
QYFLKVLIPSYAAGSIIGKGGQTIVQLQKETGATIKLSKSKDFYPGTTERVCLIQGTVEALNAV
HGFIAEKIREMPQNVAKTEPVSILQPQTTVNPDRANQVKIIVPNSTAGLIIGKGGATVKAVM
EQSGAWVQLSQKPDGINLQERVVTVSGEPEQNRKAVELIIQKIQEDPQ
00000000000000000000000000000000000000000000000000000000000000000000000000011111
11111111111100000000000000000000000000000000000000000111100000000000000000000000
00000000
>P81297_1cv8_214_387
YNEQYVNKLENFKIRETQGNNGWCAGYTMSALLNATYNTNKYHAEAVMRFLHPNLQGQ
QFQFTGLTPREMIYFEQTQGRSPQLLNRMTTYNEVDNLTKNNKGIAILGSRVESRNGMHA
GHAMAVVGNAKLNNGQEVIIIWNPWDNGFMTQDAKNNVIPVSNGDHYQWYSSIYGY
10000000000000000000000000000000000000000000000000000000000000000000000000000000
00000000000000000000000000000000000000000000000000000000000000000000000000000000
00000000
>Q02192_1ywm_51_224
SIVAASTIPGSAATLNTSITKNIQNGNAYIDLYDVKLGKIDPLQLIVLEQGFTAKYVFRQGTKY
YGDVSQLQSTGRASLTYNIFGEDGLPHVKTDGQIDIVSVALTIYDSTTLRDKIEEVRTNANDP
KWTEESRTEVLTGLDTIKTDIDNNPKTQTDIDSKIVEVNELEKLLVL
11111000000000000000000000000000000000000000000000000000000000000000000000000000
00000000000000000000000000000000000000000000000000000000000000000000000000000000
00000000
>Q13418_3ixe_0_173
MDDIFTQCREGNAVAVRLWLDNTENDLNQGDDHGFSPLHWACREGRSAVVEMLIMRGA
RINVMNRGDDTPLHLAASHGHRDIVQKLLQYKADINAVNEHGNVPLHYACFWGQDQVA
EDLVANGALVSICNKYGEMPVDKAKAPLRELLRERAEKMGQNLNRIPYKDTFWKGTTR
00000000000000000000000000000000000000000000000000000000000000000000000000000000
00000000000000000000000000000000000000000000000000000000000000000000000000000000
11111111
>Q184N1_3kmi_143_316
PTNIHKIHEVQKKLQEEVSIVLIDIADIIVNPKKENGYSRDLYTLNSLIDSSISETYDNINNTLL
SDTRFFLEHMDIIKSQRDILENLYSYVSQLNSTPPQAHILSAFIHKIGYTEFEAETGNLLLEELK
RLMISMKNQPLPVDRTEFENRAILFLCLTELKQFLVNRKHAQML
11000000000000000000000000000000000000000000000000000000000000000000000000000000
00000000000000000000000000000000000000000000000000000000000000000000000000000000
00000000
>Q64XJ4_3nrh_0_173
MKKESQVIFDKNVIEFVTVAAEFCAFLERAESMKRSTFVDTTLKILPLLYLKASMLPKCEMIG
DESPETYVTEEIYEVLRINLASILAEKDDYLEVFLPDMAYSDEPIKKNISEDLADIYQDIKDFIF
VFQLGLNETMNDSLAICQENFGLLWGQKLVNTMRALHDVKYSPKAR
11111000000000000000000000000000000000000000000000000000000000000000000000000000
00000000000011111111111100000000000000000000000000000000000000000000000000000000
00000000
>B4EA21_4grd_0_172
MSEIQTAHTHSAPLVGVLMGSSSDWDVMKHAVAILQEFGVPYEAKVVSAHRMPDEMFDY
AEKARERGLRAIIAGAGGAAHLPGMLAAKTTVPVLGVPVASKYLKGVDSLHSIVQMPKGV
PVATFAIGEAGAANAALFAVSILSGNSVDYANRLAAFRVRQNEAAHAMVLPPLE

11111000000000000000000000000000000000000000000000000000000000000000000000000000000
00000000000000000000000000000000000000000000000000000000000000000000000000000000000
0000000
>O28840_1ynb_0_172
MCIIKPMDDVVKFIHEVGSLKLTPRSGWLKLGIRLPESVAEHSFRAAIIAFILALKSGESVEKA
CKAATAALFHDLHEARTMDLHKIARRYVSCDEEGAREEQLSWMESKPDFSDVEVYVSDA
DKLELAFQGVEYSQQVSYAIRFAENVELKTDAAKEIYRVLMERKNPVWWR
11111100000000000000000000000000000000000000000000000000000000000000000000000000000
00000000000000000000000000000000000000000000000000000000000000000000000000000000000
0000000
>O30273_1ytl_1_173
AKALEQPFDVANIPGPKMATLLEKGKPVANMIKKAKRPLLIVGPDMTDEMFERVKKFVEK
DITVVATGSAITRFIDAGLGEKVNYAVLHELTQFLLDPDWKGFDGQGNYDLVLMLGSIYYH
GSQMLAAIKNFAPHIRALAIDRYYHPNADMSFGNLWKKEEDYLKLLDEILAE
11111111111111110000000000000000000000000000000000000000000000000000000000000000000
00000000000000000000000000000000000000000000000000000000000000000000000000000000000
0000000
>P0CL52_1q5z_497_669
KAGTTDNDNSQTDKTGPFSGLKFKQNSFLSTVPSVTNMHSMHFDARETFLGVIRKALEPDT
STPFPVRRAFDGLRAEILPNDTIKSAALKAQCSDIDKHPELKAKMETLKEVITHHPQKEKLA
EIALQFAREAGLTRLKGETDYVLSNVLDGLIGDGSWRAGPAYESYLNKPG
11111111111111110000000000000000000000000000000000000000000000000000000000000000000
00000000000000000000000000000000000000000000000000000000000000000000000000000011111
1111111
>P16883_2bzv_214_386
RIAISNSNRTRSVPSLTTIWSISPTPNCSIYETQDANLFLCLTKNGAHVLGTITIKGLKGALRE
MHDNALSLKLPFDNQGNLLNCALESSTWRYQETNAVASNALTFMPNSTVYPRNKTAHPG
NMLIQISPNITFSVVYNEINSGYAFTFKWSAEPGKPFHPPTAVFCYITEQ
11111111111111000000000000000000000000000000000000000000000000000000000000000000000
00000000000000000000000000000000011111111111100000000000000000000000000000000000000
0000000
>P24665_3trs_109_281
QSEEYCASAWVGIDGDTCETAILQTGVDFCYEDGQTSYDAWYEWYPDYAYDFSDITISEGDS
IKVTVEATSKSSGSATVENLTTGQSVTHTFSGNVEGDLCETNAEWIVEDFESGDSLVAFADF
GSVTFTNAEATSGGSTVGPSDATVMDIEQDGSVLTETSVSGDSVTVTYV
11000000000000000000000000000000000000000000000000000000000000000000000000000000000
00000000000000000000000000000000000000000000000000000000000000000000000000000000000
0000000
>P25084_3ix4_0_172
MALVDGFLELERSSGKLEWSAILQKMASDLGFSKILFGLLPKDSQDYENAFIVGNYPAAWR
EHYDRAGYARVDPTVSHCTQSVLPIFWEPSIYQTRKQHEFFEEASAAGLVYGLTMPLHGAR
GELGALSLSVEAENRAEANRFMESVLPTLWMLKDYALQSGAGLAFEHPVSK
11000000000000000000000000000000000000000000000000000000000000000000000000000000000
00000000000000000000000000000000000000000000000000000000000000000000000000000000000
0011111
>P32125_1np6_1_173
AGKTMIPLLAFAAWSGTGKTTLLKKLIPALCARGIRPGLIKHTHHDMDVDKPGKDSYELRK
AGAAQTIVASQQRWALMTETPDEEELDLQFLASRMDTSKLDLILVEGFKHEEIAKIVLFRDG
AGHRPEELVIDRHVIAVASDVPLNLDVALLDINDVEGLADFVVEWMQKQN
11110000000000000000000000000000000000000000000000000000000000000000000000000000000
00000000000000000000000000000000000000000000000000000000000000000000000000000000000

0000000
>Q186H8_4exr_32_204
QDNTKKEANASNNALKDEKNNENLMEQDFKVPYTDAINIFKDKYKDADIVDLSLERDLN
KFVYTVEGVDDNNEYKMKIDANTKDVLEDKTEKLDSEDLNGVARKEKLDLNDIMTPQQA
MEIALKEQNGIVKEWSLDKDLDVTFYKIRIDKDKNEYDIKVDSKKGTVLKVEKED
11111111111111111111000000000000000000000000000000000000000000000000000000000000000
00000000000000000000000000000000000000000000000000000000000000000000000000000000000
0000001
>Q283U5_4dq5_47_219
LQALLPKAQSVGNSRVRFTTAEVDSAVARISQKIGVPASYYQFLIPIENFVVAGGFETTVSGSF
RGLGQFNRQTWDGLRRLGRNLPAFEEGSAQLNASLYAIGFLYLENKRAYEASFKGRVFTHEI
AYLYHNQGAPAAEQYLTSGRLVYPKQSEAAVAAVAAARNQHVKESWA
00001111111110000000000000000000000000000000000000000000000000000000000000000000000
00000000000000000000000000000000000000000000000000000000000000000000000000000000000
0000000
>Q30V07_3ejk_0_172
MDIMLNTADISAAAILLPVEGAQLSELRQIPAEGGPVLHMLRLDSPQFSQFGEIYFSEVLPRR
VKAWKRHSLMTQLFAVPVGCIHVVLYDGREKSPTSGRLAQVTLGRPDNYRLLRIPPQVWY
GFAATGDTPALVANCTDIPHRQGESERAPQDAPFIPFSWAGADLSGTPVM
11111111111110000000000000000000000000000000000000000000000000000000000000000000000
00000000000000000000000000000000000000000000000000000000000000000000000000000000000
0000111
>Q59472_1egm_0_172
MNTDAIESMVRDVLSRMNSLQGEAPAAAPAAGGASRSARVSDYPLANKHPEWVKTATNK
TLDDFTLENVLSNKVTAQDMRITPETLRLQASIAKDAGRDRLAMNFERAAELTAVPDDRIL
EIYNALRPYRSTKEELLAIADDLESRYQAKICAAFVREAATLYVERKKLKGDD
11111111111111111111111111111111111100000000000000000000000000000000000000000000000
00000000000000000000000000000000000000000000000000000000000000000000000000000000000
0000000
>Q5LVY3_3iht_0_172
MREMQIMSQPEQSRLDLFIDRMVSQRACLEHAIAQTAGLSGPVYELGLGNGRTYHHLRQH
VQGREIYVFERAVASHPDSTPPEAQLILGDIRETLPATLERFGATASLVHADLGGHNREKND
RFARLISPLIEPHLAQGGLMVSSDRMYFEGLEELPLPPGAVVGRCFIYRRG
11111111110000000000000000000000000000000000000000000000000000000000000000000000000
00000000000000000000000000000000000000000000000000000000000000000000000000000000000
0000000
>Q7Z2W4_2x5y_723_895
SSKKYKLSEIHHLHPEYVRVSEHFKASMKNFKIEKIKKIENSELLDKFTWKKSQMKEEGKLL
FYATSRAYVESICSNNFDSFLHETHENKYGKGIYFAKDAIYSHKNCPYDAKNVVMFVAQVL
VGKFTEGNITYTSPPPQFDSCVDTRSNPSVFVIFQKDQVYPQYVIEYTED
11000000000000000000000000000000000000000000000000000000000000000000000000000000000
00000000000000000000000000000000000000000000000000000000000000000000000000000000000
0000000
>Q87T87_2qhk_30_202
NSSKSLEAELVRDRQELIDARKKELKAYMMMGVTAIKPLYDSDVNGSNKQAAKEILKAMR
FESDGYFFAYDSQGINTLHAIKPSLEGKNLYDLKDENGVAVIAGLIDASQKGDGFLYFSWHK
PTINAQAPKLGYAEYLQKWDWVLGTGIYIDDIDQQVAMQRELRTQELNQHT
11111110000000000000000000000000000000000000000000000000000000000000000000000000000
00000000000000000000000000000000000000000000000000000000000000000000000011111111111
1111111
>Q8II92_3t64_0_172

MHLKIVCLSDEVREMYKNHKTHHEGDSGLDLFIVKDEVLKPKSTTFVKLGIKAIALQYKSN
YYYKCEKSENKKKDDDKSNIVNTSFLLFPRSSISKTPLRLANSIGLIDAGYRGEIIAALDNTSD
QEYHIKKNDKLVQLVSFTGEPLSFELVEELDETSRGEGGFGSTSNNKY
00000000000000000000011000000000000000000000000000000000000000000111111111111110000
00000000000000000000000000000000000000000000000000000000000000000000000000000000000
0111111
>Q8YHT7_3cb0_0_172
MQTVNNIISSVSTVESKAYRDAMSHYAGAVQIVTTAGAAGRRGLTLTAACSVSDNPPTILIC
LQKIHEENRIFIENGVFAINTLAGPHQQLADAFSGRIGLTQDERFELAAWEILATGAPVLKG
ALAAFDCRVVSVQDHSTHHVLFGEVVGLSSHAEEEALIYLNRRYHKLEL
11111111110000000000000000000000000000000000000000000000000000000000000000000000000
00000000000000000000000000000000000000000000000000000000000000000000000000000000000
0000000
>Q96FJ0_2znv_263_435
EGLRCVVLPEDLCHKFLQLAESNTVRGIETCGILCGKLTHNEFTITHVIVPKQSAGPDYCDM
ENVEELFNVQDQHDLLTLGWIHTHPTQTAFLSSVDLHTHCSYQLMLPEAIAIVCSPKHKDT
GIFRLTNAGMLEVSACKKKGFHPHTKEPRLFSICKHVLVKDIKIIVLDLR
10000000000000000000000000000000000000000000000000000000000000000000000000000000000
00000000000000000000000000000000000000000000000000000000000000000000000000000000000
0000000
>A5K2B2_3lx3_0_171
MNPHPVEPRDQIAELLVESPLFSFNCAHFIAFKGFRETLHGHNYNVSLRLRGNIQGDGYVI
DFSILKEKVRKVCKQLDHHFILPMYSDVLNIQEVNDNFKITCEDNSEYSFPKRDCVQIPIKHS
STEEIGLYILNQLIEEIDLPFLKTRSVNYMEVTVSESPSQKATVHRNI
11111111100000000000000000000000000000000000000000000000000000000000000000000000000
00000000000000000000000000000000000000000000000000000000000000000000000000000000000
000000
>O75629_1xhn_48_219
LPPREDAARVARFVTHVSDWGALATISTLEAVRGRPFADVLSLSDGPPGAGSGVPYFYLSPL
QLSVSNLQENPYATLTMTLAQTNFCKKHGFDPQSPLCVHIMLSGTVTKVNETEMDIAKHSL
FIRHPEMKTWPSSHNWFFAKLNITNIWVLDYFGGPKIVTPEEYYNVTVQ
00000000000000000000000000000000000000000000000000000000000000000000000000000000000
00000000000000000000000000000000000000000000000000000000000000000000000000000000000
000011
>O95989_2fvv_0_171
MMKLKSNQTRTYDGDGYKKRAACLCFRSESEEEVLLVSSSRHPDRWIVPGGGMEPEEEPSV
AAVREVCEEAGVKGTLGRLVGIFENQERKHRTYVYVLIVTEVLEDWEDSVNIGRKREWFKI
EDAIKVLQYHKPVQASYFETLRQGYSANNGTPVVATTYSVSAQSSMSGIR
11111110000000000000000000000000000000000000000000000000000000000000000000000000000
00000000000000000000000000000000000000000000000000000000000111111111111111111111111
111111
>P06734_2h2r_149_320
MELQVSSGFVCNTCPEKWINFQRKCYYFGKGTKQWVHARYACDDMEGQLVSIHSPEEQD
FLTKHASHTGSWIGLRNLDLKGEFIWVDGSHVDYSNWAPGEPTSRSQGEDCVMMRGSGR
WNDAFCDRKLGAWVCDRLATCTPPASEGSAESMGPDSRPDPDGRLPTPSAPLHS
11111111000000000000000000000000000000000000000000000000000000000000000000000000000
00000000000000000000000000000000000000000000000000000000000011111111111111111111111
111111
>P21340_1tiq_0_171
MSVKMKKCSREDLQTLQQLSIETFNDTFKEQNSPENMKAYLESAFNTEQLEKELSNMSSQF
FFIYFDHEIAGYVKVNIDDAQSEEMGAESLEIERIYIKNSFQKHGLGKHLLNKAIEIALERNK

KNIWLGVWEKNENAIAFYKKMGFVQTGAHSFYMGDEEQTDLIMAKTLI
10000000000000000000000000000000000000000000000000000000000000000000000000000000000
00000000000000000000000000000000000000000000000000000000000000000000000000000000000
000000
>P56221_1idp_0_171
MGSQVQKSDEITFSDYLGLMTCVYEWADSYDSKDWDRLRKVIAPTLRIDYRSFLDKLWEA
MPAEEFVGMVSSKQVLGDPTLRTQHFIGGTRWEKVSEDEVIGYHQLRVPHQRYKDTTMKE
VTMKGHAHSANLHWYKKIDGVWKFAGLKPDIRWGEFDFDRIFEDGRETFGDK
11111111000000000000000000000000000000000000000000000000000000000000000000000000000
00000000000000000000000000000000000000000000000000000000000000000000000011111111111
111111
>Q00780_1o91_572_743
AVMPTPSPQGEYLPDMGLGIDGVKPPHAYAGKKGKHGGPAYEMPAFTAELTVPFPPVGAP
VKFDKLLYNGRQNYNPQTGIFTCEVPGVYYFAYHVHCKGGNVWVALFKNNEPMMYTYD
EYKKGFLDQASGSAVLLLRPGDQVFLQMPSEQAAGLYAGQYVHSSFSGYLLYPM
11111111111111111111111111111111111111111100000000000000000000000000000000000000000
00000000000000000000000000000000000000000000000000000000000000000000000000000000000
000000
>Q50927_2je3_26_197
AGVAEFNDKGELLLPKNYREWVMVGTQVTPNELNDGKAPFTEIRTVYYVDPESYAHWKKT
GEFRDGTVTVKELVSVGDRKGPGSGNGYFMGDYIGLEASVKDSQRFANEPGNWAFYIFYVP
DTPLVAAAKNLPTAECAACHKENAKTDMVFTQFYPVLRAAKATGESGVVAPK
00000000000000000000000000000011111111110000000000000000000000000000000000000000011
10000000000000000000000000000000000000000000000000000000000000000000000000000000000
000011
>Q66CF3_3q62_0_171
MVDKRESYTKEDLEASGRGELFGAGGPPLPAGNMLMMDRIVKMIEDGGSHNKGYVEAEL
DINPDLWFFGCHFIGDPVMPGCLGLDAMWQLVGFYLGWLGGEGKGRALGVGEVKFTGQ
VLPDAKKVTYRINFKRVIMRKLIMGVADGEVLVDGKVIYTATDLKVGLFKDTNAF
00000000000000000000000000000000000000000000000000000000000000000000000000000000000
00000000000000000000000000000000000000000000000000000000000000000000000000000000000
000111
>Q90941_1w4s_931_1102
SSGSAGLSSLHRTYSQDCSFKNSMYHVGDYVYVEPAEANLQPHIVCIERLWEDSAGEKWLY
GCWFYRPNETFHLATRKFLEKEVFKSDYYNKVPVSKILGKCVVMFVKEYFKLCPENFRDED
VYVCESRYSAKTKSFKKIKLWTMPVSSVRFVPRDVPLPVVRVASVFANTD
11111111111111111111111000000000000000000000000000000000000000000000000000000000000
00000000000000000000000000000000000000000000000000000000000000000000000000000000000
000111
>Q96AC1_4f7h_327_498
SIMTSENHLNNSDKEVDEVDAALSDLEITLEGGKTSTILGDITSIPELADYIKVFKPKKLTLKG
YKQYWCTFKDTSISCYKSKEESSGTPAHQMNLRGCEVTPDVNISGQKFNIKLLIPVAEGMNE
IWLRCDNEKQYAHWMAACRLASKGKTMADSSYNLEVQNILSFLKMQ
11111111111111111111111111111111111111000000000000000000000000000000000000000000000
00000000000000000000000000000000000000000000000000000000000000000000000000000000000
000000
>Q9I6V6_4i3m_0_171
MGLFNAHAVAQQRADRIATLLQSFADGQLDTAVGEAPAPGYERLYDSLRALQRQLREQRA
ELQQVESLEAGLAEMSRQHEAGWIDQTIPAERLEGRAARIAKGVNELVAAHIAVKMKVVS
VVTAYGQGNFEPLMDRLPGKKAQITEAIDGVRERLRGAAEATSAQLATAAYN
11111100000000000000000000000000000000000000000000000000000000000000000000000000000

00000000000000000000000000000000000000000000000000000000000000000000000001111111111
111111
>A0JWG4_2r1i_0_170
MDAGRGAEHDEAVTSDDSASVEVPRRATPADAATVAQMLHDFNTEFGAPTPGTDELASR
LSHLLAGEDVVVLLAGEPPTGLAVLSFRPNVWYPGPVAILDELYVRPGRRGHRLGSALLAA
SCGLVRSRGGALLEINVDGEDTDARRFYEARGFTNTEPNGTEPMLYYYREL
11111111111111100000000000000000000000000000000000000000000000000000000000000000000
00000000000000000000000000000000000000000000000000000000000000000000000000000000000
00000
>A4XEC0_3es1_0_170
MSEALSDSGLPPVQRVVTGHDAHGRAVFKSEDVTPTRMIPSGDASFLLVWTTATVPADNN
DETDGRQREAGLTLDGGSVIRVVDMLPGKESPMHRTNSIDYGIVLEGEIELELDDGAKRTV
RQGGIIVQRGTNHLWRNTTDKPCRIAFILIEAPAYLHNGQPLPEDKPDHK
11000000000000000000000000000000000000000000000000000000000000000000000000000000000
00000000000000000000000000000000000000000000000000000000000000000000000000000000001
11111
>A9CHU9_2fsr_0_170
MNHSIPTLRTERLTLRPLAMADFPAYRDFMASPRSTGVGGPYDLPSTWGVFCHDLANWHF
FGHGALMIDLGETGECIGQIGINHGPLFPEKELGWLLYEGHEGRGYAAEAAVALRDWAFET
LNLPTLVSYVSPQNRKSAAVAERIGGTLDPLAPRSDPEDLVYRYHQVKTA
11100000000000000000000000000000000000000000000000000000000000000000000000000000000
00000000000000000000000000000000000000000000000000000000000000000000000000000000000
01111
>F9UMB1_3dmn_596_766
SYRSTQQITDFTKEILVNGEAVTAFDRQGDLPNVVVTPNFEAGVDQVVDQLAMNDSERDT
TAIIGKSLAECEALTKALKARGEQVTLIQTENQRLAPGVIVVPSFLAKGLEFDAVIVWNANQ
ENYQREDERQLLYTICSRAMHELTLVAVGSLSPLLARVNHALYTLNEAK
00000000000000000011111111000000000000000000000000000000000000000000000000000000000
00000000010000000000000000000000000000000000000000000000000000000000000000000000000
00011
>O14936_1kgd_738_908
RKTLVLLGAHGVGRRHIKNTLITKHPDRFAYPIPHTTRPPKKDEENGKNYYFVSHDQMMQ
DISNNEYLEYGSHEDAMYGTKLETIRKIHEQGLIAILDVEPQALKVLRTAEFAPFVVFIAAPTI
TPGLNEDESLQRLQKESDILQRTYAHYFDLTIINNEIDETIRHLEEA
00000000000000000000000000000000000000000000011100000000000000000000000000000000000
00000000000000000000000000000000000000000000000000000000000000000000000000000000000
00000
>O66665_1r4v_0_170
MQEKYNFGKVSSQHKNYSKIETMLRPKGFDKLDHYFRTELDIDLTDETIELLLNSVKAAFG
KLFYGAEQRARWNGRDFIALADLNITKALEEHIKNFQKIEQDMGVDELLEYIAFIPPVEMN
VGEDLKSEYRNIMGGLLLMHADVIKKATGERKPSREAMEFVAQIVDKVF
11111111111111111111000000000000000000000000000000000000000000000000000000000000000
00000000000000000000000000000000000000000000000000000000000000000000000000000000000
00000
>P0A910_1qjp_21_191
APKDNTWYTGAKLGWSQYHDTGFINNNGPTHENQLGAGAFGGYQVNPYVGFEMGYDW
LGRMPYKGSVENGAYKAQGVQLTAKLGYPITDDLDIYTRLGGMVWRADTKSNVYGKNH
DTGVSPVFAGGVEYAITPEIATRLEYQWTNNIGDAHTIGTRPDNGMLSLGVSYRFG
00000000000000000111111111111100000000000000000000000000000011111110000000000000000
00000000000000000000000000000000000000000000000000000000000000111111111111110000000
00000

>P20344_3qc7_109_279
LVISRSKEISIEVDDIKDAGDTGKRLLKINTPSGARNIIIENEDAKALINGETTNTNKKNLQDL
LFSDGNVKAFLQATTTDENKTALQQLLVSNADVLGLLSGNPTSDNKINLRTMIGAGVPYSL
PAATTTTIGGVKKGAAVTASTATDVATAVKDLNSLITVLKNAGIIS
1111111100000000000000000000000000000000000000000000000000000000000000000000000000
00000000000000000000000000000000000000000000000000000000000000000000000000000000000
00000
>P33064_2p4d_0_170
MDKKSLYKYLLLRSTGDMRRAKSPTIMTRVTNNVYLGNYKNAMNAPSSEVKFKYVLNLT
MDKYTLPNSNINIIHIPLVDDTTTDISKYFDDVTAFLSKCDQRNEPVLVHCVAGVNRSGAMI
LAYLMSKNKESSPMLYFLYVVYHSMRDLRGAFVENPSFKRQIIEKYVIDKN
11110000000000000000000000000000000000000000000000000000000000000000000000000000000
00000000000000000000000000000000000000000000000000000000000000000000000000000000000
01111
>P35269_1f3u_1_171
AALGPSSQNVTEYVVRVPKNTTKKYNIMAFNAADKVNFATWNQARLERDLSNKKIYQEE
EMPESGAGSEFNRKLREEARRKKYGIVLKEFRPEDQPWLLRVNGKSGRKFKGIKKGGVTEN
TSYYIFTQCPDGAFEAFPVHNWYNFTPLARHRTLTAEEAEEEWERRNKVLN
11100000000000000000000000000000000000000000000000000000000001111111110000000000000
00000000000000000000000000000000000000000000000000000000000000000000000000000000000
01111
>P39432_1u14_0_170
MHQVISATTNPAKIQAILQAFEEIFGEGSCHITPVAVESGVPEQPFGSEETRAGARNRVDNA
RRLHPQADFWVAIEAGIDDDATFSWVVIDNGVQRGEARSATLPLPAVILDRVRQGEALGPV
MSQYTGIDEIGRKEGAIGVFTAGKLTRSSVYYQAVILALSPFHNAVYR
00000000000000000000000000000000000000000000000000000000000000000000000000000000000
00000000000000000000000000000000000000000000000000000000000000000000000000000000000
00111
>Q16611_2imt_15_185
EPALPSASEEQVAQDTEEVFRSYVFYRHQQEQEAEGVAAPADPEMVTLPLQPSSTMGQVGR
QLAIIGDDINRRYDSEFQTMLQHLQPTAENAYEYFTKIATSLFESGINWGRVVALLGFGYRL
ALHVYQHGLTGFLGQVTRFVVDFMLHHCIARWIAQRGGWVAALNLGNG
11111000000000000000000000000000000000000000000000000000000000000000000000000000000
00000000000000000000000000000000000000000000000000000000000000000000000000000000000
00111
>Q6PXS3_2wfo_86_256
ELPSLCMLNNSFYYMKGGANIFLIRVSDVSVLMKEYDVSVYEPEDLGNCLNKSDSSWAIHW
FSIALGHDWLMDPPMLCRNKTKKEGSNIQFNISKADESRVYGKKIRNGMRHLFRGFYDPC
EEGKVCYVTINQCGDPSSFEYCGTNYLSKCQFDHVNTLHFLVRSKTHLNF
00000000000000000000000000000000000000000000000000000000000000000000000000000000000
00000000000000000000000000000000000000000000000000000000000000000000000111111111111
11111
>Q9RY71_2o5f_0_170
MGGVSDERLDLVNERDEVVGQILRTDPALRWERVRVVNAFLRNSQGQLWIPRRSPSKSLFP
NALDVSVGGAVQSGETYEEAFRREAREELNVEIDALSWRPLASFSPFQTTLSSFMCVYELRS
DATPIFNPNDISGGEWLTPEHLLARIAAGEAAKGDLAELVRRCYREEE
11111100000000000000000000000000000000000000000000000000000000000000000000000000000
00000000000000000000000000000000000000000000000000000000000000000000000000000000000
00111
>B1YMF4_3nl9_0_169
MKQPNYYQDVKQFHQTFHHPGADQPTAIPLDRGVKRATWTAEEAVVEFLHQSSQNETEF

LAAIETFKAGLDQAVKKSLKETYPVTEVERLVGQGDALTDALYFIMGSFVEAGLEPGPLFEIV
QQANMAKLGPDGQPIFRESDQKVMKPDGWLPPEPQLEAEVVRQMKEKA
1000000000000000000000000000000000000000000000000000000000000000000000000000000000
00000000000000000000000000000000000000000000000000000000000000000000000000000000000
0000
>D2Q3N2_4h89_0_169
MSPEALQVRDAEDADWPAILPFFREIVSAGETYAYDPELTDEQARSLWMTPSGAPQSRTTV
AVDADGTVLGSANMYPNRPGPGAHVASASFMVAAAARGRGVGRALCQDMIDWAGREG
FRAIQFNAVVETNTVAVKLWQSLGFRVIGTVPEAFHHPTHGYVGLHVMHRPL
10000000000000000000000000000000000000000000000000000100000000000000000000000000000
00000000000000000000000000000000000000000000000000000000000000000000000000000000000
0000
>P0AEZ9_1mkz_0_169
MSQVSTEFIPTRIAILTVSNRRGEEDDTSGHYLRDSAQEAGHHVVDKAIVKENRYAIRAQVS
AWIASDDVQVVLITGGTGLTEGDQAPEALLPLFDREVEGFGEVFRMLSFEEIGTSTLQSRAV
AGVANKTLIFAMPGSTKACRTAWENIIAPQLDARTRPCNFHPHLKK
10000000000000000000000000000000000000000000000000000000000000000000000000000000000
00000000000000000000000000000000000000000000000000000000000000000000000000000000000
0000
>Q06505_3nfq_140_309
RRTRRDEDDLEQYLDEKILRLKDEMNIAAQLDIDTLNKRIETGDTSLIAMQKVKLLPKVVSV
LSKANLADTILDNNLLQSVRIWLEPLPDGSLPSFEIQKSLFAALNDLPVKTEHLKESGLGRV
VIFYTKSKRVEAQLARLAEKLIAEWTRPIIGASDNYRDKRIMQLEF
11111110000000000000000000000000000000000000000000000000000000000000000000000000000
00000000000000000000000000000000000000000000000000000000000000000000001111111111111
1111
>Q82XK1_1xfs_0_169
MSTTPIDAELDLMLKRELAVPVNLVWRGLTEPELLKKWFVPKPWSISDCRVDLRPGGEFYT
VMQDPEGNKFPNSGCFLEVTDEKRLIWTSALVKNYRPAVPATTSDKECAHIVMTAVIELQPT
SSGTRYTACAMHNTPGQRKLHEEMGFHEGWGTTITQLEELLKQEKAY
11100000000000000000000000000000000000000000000000000000000000000000000000000000000
00000000000000000000111111111100000000000000000000000000000000000000000000000000000
0000
>Q837C3_2nrk_0_169
MRVIVTEYQPAWVEQFEEEAQALKQILKENCLKVEHIGSTSVPNLAAKPIIDFLVIVEEIEKV
DLLQWEFERIGYEYMGEFGLSGRRYLRKGPIKRTHHVHIYQFDNTQEILRHLAFRNYLREN
PAIATTYGTLKKQLAQAHPDSIDKYMDGKDAFIKKIEKEALKKYWE
11100000000000000000000000000000000000000000000000000000000000000000000000000000000
00000000000000000000000000000000000000000000000000000000000000000001100000000000000
0000
>Q8VQD3_1yj7_20_189
KEQLYTGLTEKEANQMQALLLSNDVNVSKEMDKSGNMTLSVEKEDFVRAITILNNNGFPK
KKFADIEVIFPPSQLVASPSQENAKINYLKEQDIERLLSKIPGVIDCSVSLNVNNNESQPSSAA
VLVISSPEVNLAPSVIQIKNLVKNSVDDLKLENISVVIKSSSGQDG
00000000000000000000000000000000000000000000000000000000000000000000000111111000000
00000000000000000000000000000011111100000000000000000000000000000000000000000000000
1111
>Q9I194_3l91_23_192
DMPRPTGLAADIRWTAYGVPHIRAKDERGLGYGIGYAYARDNACLLAEEIVTARGERARYF
GSEGKSSAELDNLPSDIFYAWLNQPEALQAFWQAQTPAVRQLLEGYAAGFNRFLREADGK
TTSCLGQPWLRAIATDDLLRLTRRLLVEGGVGQFADALVAAAPPGAEKV

11111000000000000000000000000000000000000000000000000000000000000000000000000000000
00000000000000000000000000000000000000000000000000000000000000000000000000000000000
0001
>Q9KAD1_2rbd_0_169
MGILSGNPQDEPLHYGEVFSTWTYLSTNNGLINGYRSFINHTGDEDLKNLIDEAIQAMQDE
NHQLEELLRSNGVGLPPAPPDRPAARLDDIPVGARFNDPEISATISMDVAKGLVTCSQIIGQS
IREDVALMFSQFHMAKVQFGGKMLKLNKNKGWLIPPPLHSDRPIKE
11111100000000000000000000000000000000000000000000000000000000000000000000000000000
00000000000000000000000000000000000000000000000000000000000000000000000000000000001
1111
>Q9U6M1_2xse_391_560
TNLMVSTAVEKKKYLDSEFLLHCISAQLLDMWKQARARWLELVGKEWAHMLALNPERKD
FLWKNQSEMNSAFFDLCEVGKQVMLGLLGKEVALPKEEQAFWIMYAVHLSAACAEELHM
PEVAMSLRKLNVKLKDFNFGGTRYFKDMPPEEKKRRMERKQRIEEARRHGMP
11100000000000000000000000000000000000000000000000000000000000000000000000000000000
00000000000000000000000000000000000000000000000000000001111110000000000000000000000
0000
>Q9UM13_1jhj_2_171
TPNKTPPGADPKQLERTGTVREIGSQAVWSLSSCKPGFGVDQLRDDNLETYWQSDGSQPH
LVNIQFRRKTTVKTLCIYADYKSDESYTPSKISVRVGNNFHNLQEIRQLELVEPSGWIHVPLT
DNHKKPTRTFMIQIAVLANHQNGRDTHMRQIKIYTPVEESSIGKFPR
00000000000000000000000000000000000000000000000000000000000000000000000000000000000
00000000000000000000000000000000000000000000000000000000000000000000000000000111111
1111
>Q9YE85_3ajv_0_169
MGKGEGEVAGCKAAARLGVEGVFVEECFDGSYCRNLERIGYLRKGRLEPLEAAYQASRGM
LCMGETRGWAAAVEVIAGLGLSLDTALVYFDLRRKGRKPLVGVRRGTLVYEHGGRVYEVL
VLSEGYPLKIGSLVEWSRGASMDNHSPIVAIVDRTGLITYYEARAVRSIQ
11000000000000000000000000000000000000000000000000000000000000000000000000000000000
00000000000000000000000000000000000000000000000000000000000000000000000000000000000
0000
>A0JVT3_3kop_0_168
MARYINITLEKRGVTCKALLLDDVAPRTSKAVWDALPQSSQVFHGKYARNEIYNLVPAFAP
KEPGAENTTVTPIPGDVCYFTFTSNDLKTPSHGYEADSGTDEVQTIVDLAVFYGRNNLLLN
GDTGWVPGNVFATIVEGLDEMAAACQDIWMGGARDETLTFSRAEDTA
00000000000000000000000000000000000000000000000000000000000000000000000000000000000
00000000000001111111000000000000000000000000000000000000000000000000000000000000000
111
>A9CJ26_2a9s_0_168
MSLFPGDIEELARRIITDFTPLGLMVSTAESCTGGLIAGALTEIAGSSAVVDRGFVTYTNDAK
RDMLGVGTETLTTFGAVSRQTALQMAHGALYRSRANFAVAVTGIAGPGGGSAEKPVGLVH
LATKARNGNVLHHEMRYGDIGRTEIRLATVRTALEMLIALNQAGSV
00000000000000000000000000000000000000000000000000000000000000000000000000000000000
00000000000000000000000000000000000000000000000000000000000000000000000000000000000
011
>B1YEL3_3jx9_0_168
MLKILATQFNGKLQTLTKQEDELFDVVRLLAQALVGQGKVYLDAYGEFEGLYPMLSDGPD
QMKRVTKIKDHKTLHAVDRVLIFTPDTERSDLLASLARYDAWHTPYSIITLGDVTETLERSIA
PLALKFDKGLLPAEDGSRHGLPSLALGAFLLTHILTQLQEMTEEWE
00000000000000000000000000000000000000000000000000000000000000000000000000000000000
00000000000000000000000000000000000000000000000000000000000000000000000000000000000

001
>B9V973_3h6q_0_168
MGFEDGFYTILHLAEGQHPNSKIPGGMYASSKDGKDVPVTAEPLGPQSKIRWWIARDPQA
GDDMYTITEFRIDNSIPGQWSRSPVETEVPVYLYDRIKAEETGYTCAWRIQPADHGADGVY
HIVGNVRIGSTDWADLREEYGEPQVYMKPVPVIPNVYIPRWFILGYEE
10000000000000000000000000000000000000000000000000000000000000000000000000000000000
00000000000000000000000000000000000000000000000000000000000000000000000000000000000
000
>O05594_3eo5_193_361
RNRIKKVTERLPLPPNARRVEDPEMNMSREVVEDPGVPGTQDVTFAVAEVNGVETGRLPV
ANVVVTPAHEAVVRVGTKPGTEVPPVIDGSIWDAIAGCEAGGNWAINTGNGYYGGVQFD
QGTWEANGGLRYAPRADLATREEQIAVAEVTRLRQGWGAWPVCAARAGAR
00000000000000000000000000000000000000000000000000000000000000000000000000000000000
00001100000000000000000000000000000000000000000000000000000000000000000000000000000
000
>O29428_1i0s_0_168
MDVEAFYKISYGLYIVTSESNGRKCGQIANTVFQLTSKPVQIAVCLNKENDTHNAVKESGAF
GVSVLELETPMEFIGRFGFRKSSEFEKFDGVEYKTGKTGVPLVTQHAVAVIEAKVVKECDVG
THTLFVGEAVDAEVLKDAEVLTYADYHLMKKGKTPRTATVYFESK
00000000000000000000000000000000000000000000000000000000000000000000000000000000000
00000000000000000000000000000000000000000000000000000000000000000000000000000000000
001
>O31691_3rio_1_169
NGSFTVKKVLNNNVLIASHHKYSEVVLIGKGIGFGKKQDDVIEDKGYDKMFILKDEKEQK
QFKKLLDYVDEKLVDISNDVIYHISNRTNHSLNEHIHIALTDHIAFAIKRQQQGFDMKNPFL
METQSLYPEEYQIAKEVIDMINEKAGLCLPEGEIGFIALHIHSALTN
10000000000000000000000000000000000000000000000000000000000000000000000000000000000
00000000000000000000000000000000000000000000000000000000000000000000000000000000000
000
>P00423_2y69_0_168
MLATRVFSLIGRRAISTSVCVRAHGSVVKSEDYALPSYVDRRDYPLPDVAHVKNLSASQKAL
KEKEKASWSSLSIDEKVELYRLKFKESFAEMNRSTNEWKTVVGAAMFFIGFTALLLIWEKHY
VYGPIPHTFEEEWVAKQTKRMLDMKVAPIQGFSAKWDYDKNEWKK
11111111111111111111111110000000000000000000000000000000000000000000000000000000000
00000000000000000000000000000000000000000000000000000000000000000000000000000000000
000
>P25522_2gj8_215_383
GMKVVIAGRPNAGKSSLLNALAGREAAIVTDIAGTTRDVLREHIHIDGMPLHIIDTAGLRE
ASDEVERIGIERAWQEIEQADRVLFMVDGTTTDAVDPAEIWPEFIARLPAKLPITVVRNKADI
TGETLGMSEVNGHALIRLSARTGEGVDVLRNHLKQSMGFDTNMEG
00000000000000000000000000000000000000000000000000000000000000000000000000000000000
00000000000000000000000000000000000000000000000000000000000000000000000000000011111
111
>Q03DN6_3ha2_0_168
MQTLIIVAHPELARSNTQPFFKAAIENFSNVTWHPLVADFNVEQEQSLLLQNDRIILEFPLY
WYSAPALLKQWMDTVMTTKFATGHQYALEGKELGIVVSTGDNGNAFQAGAAEKFTISEL
MRPFEAFANKTKMMYLPILAVHQFLYLEPDAQQRLLVAYQQYATNVGA
00000000000000000000000000000000000000000000000000000000000000000000000000000000000
00000000000000000000000000000000000000000000000000000000000000000000000000000000000
001
>Q23972_1oxj_595_763

VGMSGIGLWLKSLRLHKYIELFKNMTYEEMLLITEDFLQSVGVTKGASHKLALCIDKLKER
ANILNRVEQELLSGQMELSTAVEELTNIVLTPMKPLESPGPPEENIGLRFLKVIDIVTNTLQQ
DPYAVQDDETLGVLMWILDRSIHNEAFMNHASQLKDLKFKLSKMK
00000000000000000000000000000000000000000000000000000000000000000000000000000000000
00000000000000000000000000000000000000000000000000000000000000000000000000000000000
001
>Q53VY0_2zca_0_168
MSPGERFLDWLKRLQGQKAWTAARAAFRRSLAFPPGAYPRAMPYVEPFLAKGDWRQEER
EAHYLVAALYALKDGDHQVGRTLARALWEKAQGSASVEKRFLALLEADRDQIAFRLRQAV
ALVEGGIDFARLLDDLLRWFSPERHVQARWAREYYGAGASEEEKKKEVEA
00000000000000000000000000000000000000000000000000000000000000000000000000000000000
00000000000000000000000000000000000000000000000000000000000000000000000000001111111
111
>Q6N4M9_2gyq_0_168
MGFFSRDIQTMEDLLLHGLRDIYYAEQQITKALPKMIEQATNRDLSQGLTSHLEETQKQIER
LDQVFKKLGQKPSGVNCPAIDGLIKEADETAGEIADKTVLDAAIVANAQAVEHYEIARYGT
LIAWAEELGHDDIVRFLTTNLNEEKAANTKLNTVALRKGVNRKAAS
00000000000000000000000000000000000000000000000000000000000000000000000000000000000
00000000000000000000000000000000000000000000000000000000000000000000000000000111111
100
>Q7W867_3ocm_299_467
SDESDLPSAMPAVPAFGVEERNMVSGVLTLAERSIRSIMTPRTDVSWVNIDDDAATIRQQLT
AAPHSFFPVCRGSLDEVVGIGRAKDLVADLITEGRVRRNRLRDPIIVHESIGILRLMDTLKRS
RGQLVLVADEFGAIEGLVTPIDVFEAIAGEFPDEDELPDIVAES
11111111111111000000000000000000000000000000000000000000000000000000000000000000000
00000000000000000000000000000000000000000000000000000000000000000000000000000000111
111
>Q96F10_2bei_1_169
ASVRIREAKEGDCGDILRLIRELAEFEKLSDQVKISEEALRADGFGDNPFYHCLVAEILPAPG
KLLGPCVVGYGIYYFIYSTWKGRTIYLEDIYVMPEYRGQGIGSKIIKKVAEVALDKGCSQFRL
AVLDWNQRAMDLYKALGAQDLTEAEGWHFFCFQGEATRKLAGK
00000000000000000000000000000000000000000000000000000000000111111100000000000000000
00000000000000000000000000000000000000000000000000000000000000000000000000000000000
000
>Q99ZN4_3fn5_80_248
SVLQAQMAAQQLPVIGGIAIPELGINLPIFKGLGNTELIYGAGTMKEEQVMGGENNYSLAS
HHIFGITGSSQMLFSPLERAQNGMSIYLTDKEKIYEYIIKDVFTVAPERVDVIDDTAGLKEVTL
VTCTDIEATERIIVKGELKTEYDFDKAPADVLKAFNHSYNQVST
11111100000000000000000000000000000000000000000000000000000000000000000000000000000
00000000000000000000000000000000000000000000000000000000000000000000000000000000000
000
>Q9FA38_1y60_1_169
AKITKVQVGEALVGDGNEVAHIDLIIGPRGSPAETAFCNGLVNNKHGFTSLLAVIAPNLPCK
PNTLMFNKVTINDARQAVQMFGPAQHGVAMAVQDAVAEGIIPADEADDLYVLVGVFIHW
EAADDAKIQKYNYEATKLSIQRAVNGEPKASVVTEQRKSATHPFAANA
00000000000000000000000000000000000000000000000000000000000000000000000000000000000
00000000000000000000000000000000000000000000000000000000000000000000000000000000000
001
>Q9JIR4_2q3x_1446_1614
GPAQLVGRQTLATPAMGDIQIGMEDKKGQLEVEVIRARSLTQKPGSKSTPAPYVKVYLLEN
GACIAKKKTRIARKTLDPLYQQSLVFDESPQGKVLQVIVWGDYGRMDHKCFMGVAQILLE

ELDLSSMVIGWYKLFPPSSLVDPTLAPLTRRASQSSLESSSGPPCIRS
00000000000000000000000000000000000000000000111000000000000000000000000000000000000
00000000000000000000000000000000000000000000000000000000000000001111111111111111111
111
>Q9JYL1_2p0n_0_168
MNPFETKSVTFAEPIEMLYACHGKVRRFCGQVAMLSDYIAENGCNQIVLQTIRQIAQYFNV
AAPLHHEDEEENFFPLLLQYAPQAQESVDELLRQHIGLHDNWAAVSAEFAKLEADNAYVP
DEEAFKRFVAGYDVHLAIEEPLFDMGNTFIPKEKLTEIGEIMAARRRK
11111111000000000000000000000000000000000000000000000000000000000000000000000000000
00000000000000000000000000000000000000000000000000000000000000000000000000000000000
000
>P53930_3rls_8_175
IKTLSVSRPIIYGNTAKKMGSVKPPNAPAEHTHLWTIFVRGPQNEDISYFIKKVVFKLHDTYP
NPVRSIEAPPFELTETGWGEFDINIKVYFVEEANEKVLNFYHRLRLHPYANPVPNSDNGNE
QNTTDHNSKDAEVSSVYFDEIVFNEPNEEFFKILMSRPGNLLPS
00000000000000000000000000000000000000000000000000000000000000000000000000000000000
00000000000000000000000000000111111111111111111111000000000000000000000000000000000
00
>P83826_1v8c_0_167
PKVNLYATFRDLTGKSQLELPGATVGEVLENLVRAYPALKEELFEGEGLAERVSVFLEGRDV
RYLQGLSTPLSPGATLDLFPPVAGGGFERTFGAFPPWLLERYLEEWGGTREGEGVYRLPGAV
VRFREVEPLKVGSLSIPQLRVEVEGEEAERWFERIAFAASRGGG
00000000000000000000000000000000000000000000000000000000000000000000000000000000000
00000000000000000000000000000000000000000000000000000000000000000000000000000000001
11
>Q04636_3f5r_0_167
MSTDFDRIYLNQSKFSGRFRIADSGLGWKISTSGGSAANQARKPFLLPATELSTVQWSRGCR
GYDLKINTKNQGVIQLDGFSQDDYNLIKNDFHRRFNIQVEQREHSLRGWNWGKTDLARN
EMVFALNGKPTFEIPYARINNTNLTSKNEVGIEFNIQDEEYQPAGDE
00000000000000000000000000000000000000000000000000000000000000000000000000000000000
00000000000000000000000000001111111111111111111111111111111111111111111111111111111
11
>Q81Q99_3fbu_0_167
MFIKAERLLIRKFEFKDWEAVHEYTSDSDVMKYIPEGVFTEEDTRNFVNKNMGENAKNFP
VILIGENILVGHIVFHKYFGEHTYEIGWVFNPKYFNKGYASEAAQATLKYGFKEMKLHRIIA
TCQPENTPSYRVMEKIGMRREGYFKKCIPHGNEWWDEYYYAILEEE
00000000000000000000000000000000000000000000000000000100000000000000000000000000000
00000000000000000000000000000000000000000000000000000000000000000000000000000000000
00
>Q8CK01_2qf9_37_204
AADSADAGFARDMSVHHQQAVEMSYIVRDRTDDEEVRRLAYDIAQTQANQRGMMIGWL
DLWALPKVSSDPPMTWMGMGDAPSAGEGSLMPGMATDAEMKKLGTLDGKQAEVYYLQ
LMTEHHRGGVHMAKGCVERCTVGVEKRLARGMVESQESEIRLMADLLAERGAK
00000000000000000000000000000000000000000000000000000000000000000000000000000011111
11111000000000000000000000000000000000000000000000000000000000000000000000000000000
00
>Q94JU3_3chm_1_168
DIEQKQAEIIDQLVKRASTCKSEALGPLIIEATSHPSLFAFSEILALPNVAQLEGTTDSVYLDLL
RLFAHGTWGDYKCNATRLPHLSPDQILKLKQLTVLTLAESNKVLPYDTLMVELDVSNVREL
EDFLINECMYAGIVRGKLDQLKRCFEVPFAAGRDLRPGQLGN
11000000000000000000000000000000000000000000000000000000000000000000000000000000000

00000000000000000000000000000000000000000000000000000000000000000000000000000000111
11
>Q9CQT7_2wp7_0_167
MEPPNLYPVKLYVYDLSKGLARRLSPIMLGKQLEGIWHTSIVVHKDEFFFGSSGISSCTPGGT
LLGPPDSVVDVGNTEVTEEIFLEYLSSLGESLFRGEAYNLFEHNCNTFSNEVAQFLTGRKIPSY
ITDLPSEVLSTPFGQALRPFLDSIQIQPPGGNSVGRPNGQS
11100000000000000000000000000000000000000000000000000000000000000000000000000000000
00000000000000000000000000000000000000000000000000000000000000000000000000000001111
11
>B6DMH6_4iwj_62_228
SEELLDLFNRQVTQEFTASQVYLSASIWFDQNDWEGMAAYMLAESAEEREHGLGFVDFAN
KRNIPIELQAVPAPVSCAEWSSPEDVWQSILELEQANTRSLLNLAEAASTCHDFAVMAFLNP
FHLQQVNEEDKIGSILAKVTDENRTPGLLRSLDVVSFLGPCLFRS
00000000000000000000000000000000000000000000000000000000000000000000000000000000000
00000000000000000000000000000000000000000000000000000000000000000000000000001111111
1
>O14508_2c9w_31_197
QAARLAKALRELGQTGWYWGSMTVNEAKEKLKEAPEGTFLIRDSSHSDYLLTISVKTSAGP
TNLRIEYQDGKFRLDSIICVKSKLKQFDSVVHLIDYYVQMCKDKRTGPEAPRNGTVHLYLTK
PLYTSAPSLQHLCRLTINKCTGAIWGLPLPTRLKDYLEEYKFQV
00000000000000000000000000000000000000000000000000000000000000000000000000000000000
00000000000000000000011111111111110000000000000000000000000000000000000000000000000
0
>O30192_2f9f_171_337
PVETSKFKFKCYGDFWLSVNRIYPEKRIELQLEVFKKLQDEKLYIVGWFSKGDHAERYARKI
MKIAPDNVKFLGSVSEEELIDLYSRCKGLLCTAKDEDFGLTPIEAMASGKPVIAVNEGGFKET
VINEKTGYLVNADVNEIIDAMKKVSKNPDKFKKDCFRRAKEF
10000000000000000000000000000000000000000000000000000000000000000000000000000000000
00000000000000000000000000000000000000000000000000000000000000000000000000000000000
0
>O30463_2w3w_0_166
MTRAEVGLVWAQSTSGVIGRGGDIPWSVPEDLTRFKEVTMGHTVIMGRRTWESLPAKVRP
LPGRRNVVVSRRPDFVAEGARVAGSLEAALAYAGSDPAPWVIGGAQIYLLALPHATRCEVT
EIEIDLRRDDDDALAPALDDSWVGETGEWLASRSGLRYRFHSYRRD
11000000000000000000000000000000000000000000000000000000000000000000000000000000000
00000000000000000000000000000000000000000000000000000000000000000000000000000000000
0
>O35274_3egg_416_582
DEEDGEPPYEPESGCVEIPGLSEEEDPAPSRKIHFSTAPIQVFSTYSNEDYDRRNEDVDPMAA
SAEYELEKRVERLELFPVELEKDSEGLGISIIGMGAGADMGLEKLGIFVKTVTEGGAAHRDG
RIQVNDLLVEVDGTSLVGVTQSFAASVLRNTKGRVRFMIGRE
11111110000000000000000000000000000000000000000000000000000000000000000000000000000
00000000000000000000000000000000000000000000000000000000000000000000000000000000000
0
>O53513_4hec_1_167
RYFYDTEFIEDGHTIELISIGVVAEDGREYYAVSTEFDPERAGSWVRTHVLPKLPPPASQLWRS
RQQIRLDLEEFLRIDGTDSIELWAWVGAYDHVALCQLWGPMTALPPTVPRFTRELRQLWED
RGCPRMPPRPRDVHDALVDARDQLRRFRLITSTDDAGRGAAR
00000000000000000000000000000000000000000000000000000000000000000000000000000000000
00000000000000000000000000000000000000000000000000000000000000000000000000000111111
1

>P16218_1v0a_654_820
AVGEKMLDDFEGVLNWGSYSGEGAKVSTKIVSGKTGNGMEVSYTGTTDGYWGTVYSLPD
GDWSKWLKISFDIKSVDGSANEIRFMIAEKSINGVGDGEHWVYSITPDSSWKTIEIPFSSFRRR
LDYQPPGQDMSGTLDLDNIDSIHFMYANNKSGKFVVDNIKLIGA
00000000000000000000000000000000000000000000000000000000000000000000000000011100000
00000000000000000000000000000000000000000000000000000000000000000000000000000000000
0
>P74750_1zde_769_935
KFAEYCLSFGTEILTVEYGPLPIGKIVSEEINCSVYSVDPEGRVYTQAIAQWHDRGEQEVLEY
ELEDGSVIRATSDHRFLTTDYQLLAIEEIFARQLDLLTLENIKQTEEALDNHRLPFPLLDAGTI
KMVKVIGRRSLGVQRIFDIGLPQDHNFLLANGAIAANCFN
00000000000000000000000000000000000000000000000000000000000000000000000000000000000
00000000000000000000111111111110000000000000000000000000000000000000000000000000000
0
>P76483_1ryl_0_166
MGMIGYFAEIDSEKINQLLESTEKPLMDNIHDTLSGLRRLDIDKRWDFLHFGLTGTSAFDPA
KNDPLSRAVLGEHSLEDGIDGFLGLTWNQELAATIDRLESLDRNELRKQFSIKRLNEMEIYP
GVTFSEELEGQLFASIMLDMEKLISAYRRMLRQGNHALTVIVG
11000000000000000000011111000000000000000000000000000000000000000000000000000001100
00000000000000000000000000000000000000000000000000000000000000000000000000000000000
1
>P96578_2y0o_0_166
MGITKEEVNSYYQKAGIVLTDEEVDQIQLMDYGLGKERKVGLQLFVYVNTDRYCSKELVLF
PGQTCPEHRHPPVDGQEGKQETFRCRYGKVYLYVEGEKTPLPKVLPPQEDREHYTVWHEIE
LEPGGQYTIPPNTKHWFQAGEEGAVVTEMSSTSTDKHDIFTDPRI
10000000000000000000000000000000000000000000000000000000000000000000000000000000000
00000000000000000000000000000000000000000000000000000000000000000000000000000000000
0
>Q07688_2r25_0_166
MSTIPSEIINWTILNEIISMDDDDSDFSKGLIIQFIDQAQTTFAQMQRQLDGEKNLTELDNLG
HFLKGSSAALGLQRIAWVCERIQNLGRKMEHFFPNKTELVNTLSDKSIINGINIDEDDEEIKI
QVDDKDENSIYLILIAKALNQSRLEFKLARIELSKYYNTNL
10000000000000000000000000000000000000000000000000000000000000000000000000000000000
00000000000000000000000000000000000000000000000000000000000000000000000000000000000
0
>Q09734_1jvw_29_195
SGDAASHEERMNNYRKRVGRLFMEQKAAQPDAVKLPSGLVFQRIARGSGKRAPAIDDKCE
VHYTGRLRDGTVFDSSRERGKPTTFRPNEVIKGWTEALQLMREGDRWRLFIPYDLAYGVTG
GGGMIPPYSPLEFDVELISIKDGGKGRTAEEVDEILRKAEEDREDM
11100000000000000000000000000000000000000000000000000000000000000000000000000000000
00000000000000000000000000000000000000000000000000000000000000000000000000000000111
1
>Q2G2F3_4ae5_0_166
MKKLYTSYGTYGFLHQIKINNPTHQLFQFSASDTSVIFEETDGETVLKSPSIYEVIKEIGEFSEH
HFYCAIFIPSTEDHAYQLEKKLISVDDNFRNFGGFKSYRLLRPAKGTTYKIYFGFADRHAYED
FKQSDAFNDHFSKDALSHYFGSSGQHSSYFERYLYPIKE
00000000000000000000000000000000000000000000000000000000000000000000000000000000000
00000000000000000000000000000000000000000000000000000000000000000011111000000000000
1
>Q37979_2vo9_0_166
MALTEAWLIEKANRKLNAGGMYKITSDKTRNVIKKMAKEGIYLCVAQGYRSTAEQNALYA

QGRTKPGAIVTNAKGGQSNHNYGVAVDLCLYTNDGKDVIWESTTSRWKKVVAAMKAEG
FKWGGDWKSFKDYPHFELCDAVSGEKIPAATQNTNTNSNRYEGKVIDSA
00000000000000000000000000000000000000000000000000000000000000000000000000000000000
00000000000000000000000000000000000000000000000000000000000000000111111111111111111
1
>Q6NDF6_3okx_0_166
MDATDDIRAGELASDWSGSPDAGVVFIGRIHTPWNRLKECPRHGRADGPVCRIEVFETWL
PALAGIDDGTLLEVFYWLHRSRRDLLLQCPRNDGDARGTFSIRSPLRPNPIGTSIARVDRRD
GANLFIRGLDCLDGTPLVDLKPDRAEFMPLAPPKPGDFQVGEPRR
11000000000000000000000000000000000000000000000000000000000000000000000000000000000
00000000000000000000000000000000000000000000000000000000000000000000000000000000011
1
>Q6XQ58_2i8u_0_166
MMFLRQEDFATVVRSTPLVSLDFIVENSRGEFLLGKRTNRPAQGYWFVPGGRVQKDETLEA
AFERLTMAELGLRLPITAGQFYGVWQHFYDDNFSGTDFTTHYVVLGFRFRVAEEELLLPDE
QHDDYRWLTPDALLASENVHANSRVYFNNDPRAIIGLNKKEVKNV
00000000000000000000000000000000000000000000000000000000000000000000000000000000000
00000000000000000000000000000000000000000000000000000000000000000001111111111111111
1
>Q7DDI9_2ikb_0_166
MSDKFNQFINRVLSHEGGYANHPKDPGGETNWGITKRTAQANGYNGSMRAMTREQAISI
YRKAFWERYRADQMPEAVAFQFFDACVNHGYGNAARMLQRAAGVPDDGVIGAVSLKAI
NSLPENDLLLRFNAERLVFYTKLGTFTSFGKGWVRRVAQNLIHASADNTD
00000000000000000000000000000000000000000000000000000000000000000000000000000000000
00000000000000000000000000000000000000000000000000000000000000000000000000000000011
1
>Q8WVN8_1zuo_196_362
GAVSGSVQASDRLMKELRDIYRSQSYKTGIYSVELINDSLYDWHVKLQKVDPDSPLHSDLQI
LKEKEGIEYILLNFSFKDNFPFDPPFVRVVLPVLSGGYVLGGGALCMELLTKQGWSSAYSIES
VIMQINATLVKGKARVQFGANKNQYNLARAQQSYNSIVQIHE
11110000000000000000000000000000000000000000000000000000000000000000000000000000000
00000000000000000000000000000000000000000000000000000000000000000000000000000000000
1
>Q929G0_3fj2_0_166
MKKVFITTGTEHYLRQLMENYIGENVTLLQNFSQSLLYQESTGEKLFQEGKEYRVLQSSGSL
KGFGIVVFEYIQLRDEEIPIFLQMYQHASLHFSETPGLQSTKLTKAMNTNQFLIVSFWDSEVF
FQEWKKTPLHKEITSIMKKNNTQVGFSHEDIYHYPEFSHDAK
00000000000000000000000000000000000000000000000000000000000000000000000000000000000
00000000000000000000000000000000000000000000000000000000000000000000000000000000111
1
>Q980R9_2x7b_0_166
MELAEKDKGRDFTLRNARMDDIDQIIKINRLTLPENYPYYFFVEHLKEYGLAFFVAIVDNSV
VGYIMPRIEWGFSNIKQLPSLVRKGHVVSIAVLEEYRRKGIATTLLEASMKSMKNDYNAEEI
YLEVRVSNYPAIALYEKLNFKKVKVLKGYYADGEDAYLMARPL
11111111110000000000000000000000000000000000000000000000000000000000000000000000000
00000000000000000000000000000000000000000000000000000000000000000000000000000000000
1
>Q9I0R8_3hvw_167_333
IDEPTGLYNRLRLQEDVSLRLQRDGALTVIAADLLPLALLNTIIRTLGYPFSNDLMLEARDRI
RAELPDFTLYKISPTRFGLLLPRQQQEETESVCLRLLRAFESPVVCRGIPIKANVGLGVLPLAD
DTLDGDQDWLRLVVSAADDARDRGVGWARYNPPLDQAQQR

11111110000000000000000000000000000000000000000000000000000000000000000000000000000
00000000000000000000000000000000000000000000000000000000000000000000000000000000000
1
>Q9I4A4_1z6n_0_166
MASYAELFDIGEDFAAFVGHGLATEQGAVARFRQKLESNGLPSALTERLQRIERRYRLLVAG
EMWCPDCQINLAALDFAQRLQPNIELAIISKGRAEDDLRQRLALERIAIPLVLVLDEEFNLL
GRFVERPQAVLDGGPQALAAYKAGDYLEHAIGDVLAIIEGAAR
00000000000000000000000000000000000000000000000000000000000000000000000000000000000
00000000000000000000000000000000000000000000000000000000000000000000000000000000000
1
>Q9Y3C8_2z6p_0_166
MADEATRRVVSEIPVLKTNAGPRDRELWVQRLKEEYQSLIRYVENNKNADNDWFRLESNK
EGTRWFGKCWYIHDLLKYEFDIEFDIPITYPTTAPEIAVPELDGKTAKMYRGGKICLTDHFKP
LWARNVPKFGLAHLMALGLGPWLAVEIPDLIQKGVIQHKEKCNQ
00000000000000000000000000000000000000000000000000000000000000000000000000000000000
00000000000000000000000000000000000000000000000000000000000000000000000000000000111
1
>Q9ZLI1_3bvf_0_166
MLSKDIIKLLNEQVNKEMNSSNLYMSMSSWCYTHSLDGAGLFLFDHAAEEYEHAKKLIIFL
NENNVPVQLTSISAPEHKFEGLTQIFQKAYEHEQHISESINNIVDHAIKSKDHATFNFLQWY
VAEQHEEEVLFKDILDKIELIGNENHGLYLADQYVKGIAKSRKS
00000000000000000000000000000000000000000000000000000000000000000000000000000000000
00000000000000000000000000000000000000000000000000000000000000000000000000000000000
1
>A0JVA7_2qck_0_165
MTSDNAAFEGTFKEMFRRHAAGVAIITVNYNGTPYGFTATSVASLSAQPPRFTFNMARSSSS
WPAIANTTHIGVHMLGLDNQELADRFARTKNRFEGDHWELGPYEVPILKDVAGWLIGKIQ
MRLSFENNAVVVVEVVEGQVGEDGTPLLYHSGAYSQPVPLDYEI
11111110000000000000000000000000000000000000000000000000000000000000000000000000000
00000000000000000000000000000000000000000000000000000000000000000000000000000000000
>O06337_2chc_2_167
PVDEQWIEILRIQALCARYCLTIDTQDGEGWAGCFTEDGAFEFDGWVIRGRPALREYADAH
ARVVRGRHLTTDLLYEVDGDVATGRSASVVTLATAAGYKILGSGEYQDRLIKQDGQWRIAY
RRLRNDRLVSDPSVAVNVADADVAAVVGHLLAAARRLGTQMSDT
11100000000000000000000000000000000000000000000000000000000000000000000000000000000
00000000000000000000000000000000000000000000000000000000000000000000000000000000011
>O07746_2fp1_33_198
DGTSQLAELVDAAAERLEVADPVAAFKWRAQLPIEDSGRVEQQLAKLGEDARSQHIDPDY
VTRVFDDQIRATEAIEYSRFSDWKLNPASAPPEPPDLSASRSAIDSLNNRMLSQIWSHWSLLS
APSCAAQLDRAKRDIVRSRHLDSLYQRALTTATQSYCQALPPA
10000000000000000000000000000000000000000000000000000000000000000000000000000000000
00000000000000000000000000000000000000000000000000000000000000000000000000000000000
>O25175_3qhp_200_365
TPFKIAMVGRYSNEKNQSVLIKAVALSKYKQDIVLLLKGKGPDEKKIKLLAQKLGVKAEFGF
VNSNELLEILKTCTLYVHAANVESEAIACLEAISVGIVPVIANSPLSATRQFALDERSLFEPNN
AKDLSAKIDWWLENKLERERMQNEYAKSALNYTLENSVIQ
00000000000000000000000000000000000000000000000000000000000000000000000000000000000
00000000000000000000000000000000000000000000000000000000000000000000000000001111111
>P00778_3pro_33_198
ADQVDPQLKFAMQRDLGIFPTQLPQYLQTEKLARTQAAAIEREFGAQFAGSWIERNEDGSF
KLVAATSGARKSSTLGGVEVRNVRYSLKQLQSAMEQLDAGANARVKGVSKPLDGVQSWY

VDPRSNAVVVKVDDGATEAGVDFVALSGADSAQVRIESSPGKLQTT
11000000000000000000000000000000000000000000000000000000000000000000000000000000000
00000000000000000000011111111000000000000000000000000000000000000000000000000000111
>P19921_1n62_0_165
MAKAHIELTINGHPVEALVEPRTLLIHFIREQQNLTGAHIGCDTSHCGACTVDLDGMSVKS
CTMFAVQANGASITTIEGMAAPDGTLSALQEGFRMMHGLQCGYCTPGMIMRSHRLLQEN
PSPTEAEIRFGIGGNLCRCTGYQNIVKAIQYAAAKINGVPFEEAAE
10000000000000000000000000000000000000000000000000000000000000000000000000000000000
00000000000000000000000000000000000000000000000000000000000000000000000000000000111
>P47709_2cm6_518_683
GSARGMALYEEEQVERIGDIEERGKILVSLMYSTQQGGLIVGIIRCVHLAAMDANGYSDPFV
KLWLKPDMGKKAKHKTQIKKKTLNPEFNEEFFYDIKHSDLAKKSLDISVWDYDIGKSNDYI
GGCQLGISAKGERLKHWYECLKNKDKKIERWHQLQNENHVSSD
11111000000000000111000000000000000000000000000000000000000000000000000000000000000
00000000000000000000000000000000000000000000000000000000000000000000000000000111111
>Q12904_1euj_146_311
SKPIDVSRLDLRIGCIITARKHPDADSLYVEEVDVGEIAPRTVVSGLVNHVPLEQMQNRMVI
LLCNLKPAKMRGVLSQAMVMCASSPEKIEILAPPNGSVPGDRITFDAFPGEPDKELNPKKKI
WEQIQPDLHTNDECVATYKGVPFEVKGKGVCRAQTMSNSGIK
11000000000000000000000000000000000000000000000000000000000000000000000000000000000
00000000000000000000000000000000000000000000000000000000000000000000000000000000000
>Q28QQ5_3cje_0_165
MALTPDQMPDRAEVVFTCNGKAVGKMRNELDVAMVKPFEERFALATDEGAFHGGDASA
PPPLALFIAGLTGCVMTQIRAFAKRLKVTVTDLDVECRVVWDWAKAGPVYETGPKSFEIDII
LHSPDPIEAQQALIEAAKKGCFLEQTLGQANTIRHRLKVGDTFIDA
11111111110000000000000000000000000000000000000011111000000000000000000000000000000
00000000000000000000000000000000000000000000000000000000000000000000000000000000000
>Q60773_1blx_0_165
MLLEEVCVGDRLSGARARGDVQEVRRLLHRELVHPDALNRFGKTALQVMMFGSPAVALE
LLKQGASPNVQDASGTSPVHDAARTGFLDTLKVLVEHGADVNALDSTGSLPIHLAIREGHS
SVVSFLAPESDLHHRDASGLTPLELARQRGAQNLMDILQGHMMIPM
11111000000000000000000000000000000000000000000000000000000000000000000000000000000
00000000000000000000000000000000000000000000000000000000000000000000000000000000001
>Q6D2T7_2ph0_0_165
MTMTLNELLATNPDGTLEDIAGKYNTSLFAVVEALPTAQCTLATGDRFDQVWDTIATWGE
VTLISHTADAILEFKSELPTGTHRHGYFNLRGKNGLSGHIRATSCQHIAFIERKFMGMDTAS
VVFFNANGAAMFKIFLGRDSHRQLLSAQVDAFRALASELQPEQV
10000000000000000000000000000000000000000000000000000000000000000000000000000000000
00000000000000000000000000000000000000000000000000000000000000000000000000000000111
>Q82X29_2auw_0_165
MNEYFFPKLTAVEALAPYRLRTTWSTGEVLEVDVGDILRKIPDLAPILDPEAFARVHIAEWE
GSVEWFDTEFGRDNVYAWAKEQAGEVSHEMFGDWMHRNNLSLTTAAEALGISRRMVSYY
RTAHKIIPRTIWLACLGWEATRPETKTLPRTLPAAYAKGVSASLS
00000000000000000000000000000000000000000000000000000000000000000000000000000000000
00000000000000000000000000000000000000000000000000000000000000000000000011111111111
>Q8EAP9_2ox6_0_165
MSKNIGLNAIEMSYLRQSLSLSAAQVGQLTNHSEAEVLAWENAETQAPELAQKKLLDIDDI
IEMQVLNTTDGIEALFKKEPKRHLAFVVYPTQAIYTQYNPEFLSSLPLTELYNTAAWRIKKEC
KLVLEVDVSLINLNVEAYKAYREQNGLSESRESRAKWAATQL
11110000000000000000000000000000000000000000000000000000000000000000000000000000000
00000000000000000000000000000000000000000000000000000000000000000000000000000000000

>Q91MB1_4eqk_317_482
RISSSFSFGGFTFKRTSGSSIKREEELLTGNLQTLKIRVHEGYEEFTMVGKRATAILRKATRRLV
QLIVSGRDEQSVAEAIIVAMVFSQEDCMIKAVRGDLNFVNRANQRLNPMHQLLRHFQKDA
KVLFQNWGIEHIDNVMGMIGVLPDMTPSTEMSMRGIRVSKM
11100000000000000000000000000000000000000000000000000000000000000000000000000000000
00000000000000000000000000000000000000000000000000000000000000000000000000000000000
>Q9HD36_4b4s_1_166
ADPLRERTELLLADYLGYCAREPGTPEPAPSTPEAAVLRSAAARLRQIHRSFFSAYLGYPGN
RFELVALMADSVLSDSPGPTWGRVVTLVTFAGTLLERGPLVTARWKKWGFQPRLKEQEGD
VARDCQRLVALLSSRLMGQHRAWLQAQGGWDGFCHFFRTPFPLA
10000000000000000000000000000000000000000000000000000000000000000000000000000000000
00000000000000000000001111111111111110000000000000000000000000000000000000000000011
>Q9LV66_1xy7_0_165
MAQEDVTAVATNGAGPVETHLVFTEFKQMLLVEAQKVGDAVTFYKSAFGAIESGHSLYPKR
KLDQELPHVLSSELNLAGSSFVVCDVSSLPGFSTAKSEGSGVTFLLGTKDAEAAVAKAVDAG
AVKVEVTEAEVELGFKGKVTDPFGVTWIFAEKKTVITDENKEV
11111111111111111110000000000000000000000000000000000000111111111110000000000000000
00000000000000000000000000000000000000000000000000000000000000000000000111111111111
>Q9V219_2p38_0_165
MSGELRVRRASSWELDLILKEAEKYGELLHEFFCVVEGKYRDVYAVNEEVWKIIEDINMRPY
SLGTFVGTIRVDENLVEKFYPNLEFFSLIKLEKNYVILGPKASFLFTTGKDAPKEAVREIKWQ
GSKRVVVLNDLGDIIGIGLINPKSDRRFIKNLKDVGEFLRR
11110000000000000000000000000000000000000000000000000000000000000000000000000000000
00000000000000000000000000000000000000000000000000000000000000000000000000001111111
>O00170_4apo_165_329
DPWAMTDEEKAKAVPLIHQEGNRLYREGHVKEAAAKYYDAIACLKNLQMKEQPGSPEWI
QLDQQITPLLLNYCQCKLVVEEYYEVLDHCSSILNKYDDNVKAYFKRGKAHAAVWNAQE
AQADFAKVLELDPALAPVVSRELQALEARIRQKDEEDKARFRGIFSH
11111100000000000000000000000000000000000000000000000000000000000000000000000000000
0000000000000000000000000000000000000000000000000000000000000000000000000000011111
>O52396_1nyk_45_209
TPEKEPLKPGDILVYAQGGGEPKPIRLEELKPGDPFVLAYPMDPKTKVVKSGEAKNTLLVAR
FDPEELAPEVAQHAAEGVVAYSAVCTHLGCIVSQWVADEEAALCPCHGGVYDLRHGAQV
IAGPPPKPVPQLPVRVEDGVLVAAGEFLGPVGVQASAGAYTWRV
00000000000000000000000000000000000000000000000000000000000000000000000000000000000
0000000000000000000000000000000000000000000000000000000000000000000000000111111111
>P19250_3a57_24_188
FELPSVPFPAPGSDEILFVVRDTTFNTNAPVNVEVSDFWTNRNVKRKPYKDVYGQSVFTTSG
TKWLTSYMTVNINDKDYTMAAVSGYKHGHSAVFVKSDQVQLQHSYDSVANFVGEDEDSI
PSKMYLDETPEYFVNVEAYESGSGNILVMCISNKESFFECKHQQ
11111111111000000000000000000000000000000000000000000000000000000000000000000000000
0000000000000000000000000000000000000000000000000000000000000000000000000000000000
>P38431_2ful_240_404
VNSELTQLDEYGEWILEQAGEDKENLPSDVELYKKAAELDVLNDPKIGCVLAQCLFDEDIV
NEIAEHNAFFTKILVTPEYEKNFMGGIERFLGLEHKDLIPLLPKILVQLYNNDIISEEEIMRFGT
KSSKKFVPKEVSKKVRRAAKPFITWLETAESDDDEEDDE
00000000000000000000000000000000000000000000000000000000000000000000000000000000000
0000000000000000000000000000000000000000000000000000000000000000000000001111111000
>Q03723_3g9b_23_187
ATASHNIDDILQLKDDTGVITVTADNYPLLSRGVPGYFNILYITMRGTNSNGMSCQLCHDFE
KTYHAVADVIRSQAPQSLNLFFTVDVNEVPQLVKDLKLQNVPHLVVYPPAESNKQSQFEW

KTSPFYQYSLVPENAENTLQFGDFLAKILNISITVPQAFNVQE
11111100000000000000000000000000000000001111110000000000000000000000000000000000000
0000000000000000000000000000000000000000000000000000000000000000000000000000101111
>Q14677_1xgw_0_164
MLNMWKVRELVDKATNVVMNYSEIESKVREATNDDPWGPSGQLMGEIAKATFMYEQFPE
LMNMLWSRMLKDNKKNWRRVYKSLLLLAYLIRNGSERVVTSAREHIYDLRSLENYHFVDE
HGKDQGINIRQKVKELVEFAQDDDRLREERKKAKKNKDKYVGVSSD
11111111111111111000000000000000000000000000000000000000000000000000000000000000000
0000000000000000000000000000000000000000000000000000000000000000000000111111111111
>Q16832_2wuh_25_189
NPAICRYPLGMSGGQIPDEDITASSQWSESTAAKYGRLDSEEGDGAWCPEIPVEPDDLKEFL
QIDLHTLHFITLVGTQGRHAGGHGIEFAPMYKINYSRDGTRWISWRNRHGKQVLDGNSNP
YDIFLKDLEPPIVARFVRFIPVTDHSMNVCMRVELYGCVWLDG
00000000000000000000000000000000000000000000000000000000000000000000000000000000000
0000000000000000000000000000000000000000000000000000000000000000000000000000000001
>Q57764_1n2m_0_164
MNAEINPLHAYFKLPNTVSLVAGSSEGETPLNAFDGALLNAGIGNVNLIRISSIMPPEAEIVP
LPKLPMGALVPTAYGYIISDVPGETISAAISVAIPKDKSLCGLIMEYEGKCSKKEAEKTVREM
AKIGFEMRGWELDRIESIAVEHTVEKLGCAFAAAALWYK
11111000000000000000000000000000000000000000000000000000000000000000000000000000000
0000000000000000000000000000000000000000000000000000000000000000000000000000000000
>Q5F809_3ay2_18_182
SQEPAAPAAEATPAGEAPASEAPAAEAAPADAAEAPAAGNCAATVESNDNMQFNTKDIQ
VSKACKEFTITLKHTGTQPKASMGHNLVIAKAEDMDGVFKDGVGAADTDYVKPDDARVV
AHTKLIGGGEESSLTLDPAKLADGDYKFACTFPGHGALMNGKVTLVD
11111111111111111111111111111111111111111100000000000000000000000000000000000000000
0000000000000000000000000000000000000000000000000000000000000000000000000000000000
>Q6ZYJ9_2vxz_0_164
MPIGHSREVLVRLRDILALLADGCKTTSLIQQRLGLSHGRAKALIYVLEKEGRVTRVAFGNV
ALVCLSMDQYRQLVDGMIREVERLVTTNKLKFISPPRLHDLIIKDPQARKFFSSIIPIAHRTAII
LSFLNHLLKMIYGEPYVKTDETVYLTANRKVHIEPPAK
11110000000000000000000000000000000000000000000000000000000000000000000000000000000
0000000000000000000000000000000000000000000000000000000000000000000000000011111111
>Q8RV61_2iz6_0_164
MSGRKPIIGVMGPGKADTAENQLVMANELGKQIATHGWILLTGGRSLGVMHEAMKGAKE
AGGTTIGVLPGPDTSEISDAVDIPIVTGLGSARDNINALSSNVLVAVGMGPGTAAEVALALK
AKKPVVLLGTQPEAEKFFTSLDAGLVHVAADVAGAIAAVKQLLA
11000000000000000000000000000000000000000000000000000000000000000000111110000000000
0000000000000000000000000000000000000000000000000000000000000000000000000000000000
>Q8Y4N7_1y9i_1_165
VEKQSALESKARSWLIERGVEIDDIAELVLFLQQKYHPGLELEICRQNVEHVLRKREVQNAV
LTGIQLDVMAEKGELVQPLQNIISADEGLYGVDEILALSIVNVYGSIGFTNYGYIDKVKPGIL
AKLNEHDGIAVHTFLDDIVGAIAAAAASRLAHSYHDDIVN
11000000000000000000000000000000000000000000000000000000000000000000000000000000000
0000000000000000000000000000000000000000000000000000000000000000000000000000001111
>Q9V535_1rk8_0_164
MADVLDIDNAEEFEVDEDGDQGIVRLKEKAKHRKGRGFGSDSNTREAIHSYERVRNEDDD
ELEPGPQRSVEGWILFVTSIHEEAQEDEIQEKFCDYGEIKNIHLNLDRRTGFSKGYALVEYET
HKQALAAKEALNGAEIMGQTIQVDWCFVKGPKRVKKSEKRRR
11111111111111111111111111111111111111111111111111111111111111110000010000000000000
0000000000000000000000000000000000000000000000000000000000000000000000011111111111

>O60566_2wvi_56_219
QQKRAFEYEIRFYTGNDPLDVWDRYISWTEQNYPQGGKESNMSTLLERAVEALQGEKRYY
SDPRFLNLWLKLGRLCNEPLDMYSYLHNQGIGVSLAQFYISWAEEYEARENFRKADAIFQE
GIQQKAEPLERLQSQHRQFQARVSRQTLLALEKEEEEEVFESS
00000000000000000000000000000000000111100000000000000000000000000000000000000000000
000000000000000000000000000000000000000000000000000000000000000000000000000000000
>P0C6U6_2vri_1257_1420
KPFAVYKNVKFYLGDISHLVNCVSFDFVVNAANENLLHGGGVARAIDILTEGQLQSLSKDYI
SSNGPLKVGAGVMLECEKFNVFNVVGPRTGKHEHSLLVEAYNSILFENGIPLMPLLSCGIFG
VRIENSLKALFSCDINKPLQVFVYSSNEEQAVLKFLDGLD
00000000000000000000000000000000000000000000000000000000000000000000000000000000000
000000000000000000000000000000000000000000000000000000000000000000000000000000001
>P49687_3kep_441_604
LNKQDGENTLQHEKSSSFGYWCSPSPEQLERLSLKQLAAVSNFVIGRRGYGCITFQHDVDLT
AFTKSFREELFGKIVIFRSSKTVEVYPDEATKPMIGHGLNVPAIITLENVYPVDKKTKKPMKD
TTKFAEFQVFDRKLRSMREMNYISYNPFGGTWTFKVNHF
11111111111111111000000000000000000000000000000000000000000000000000000000000000000
000000000000000000000000000000000000000000000000000000000000000000000000000000000
>Q06697_3v46_229_392
SSGGPRKDPIILIPSAASSILTVANIKQFLLESKYVNPRNLPSVPNGLVNIEKNFERISRPIRFIIV
DNTRMFTKPEYWDRVVAIFTTGHTWQFNNYQWNSPQELFQRCKGYYFHFAGDSVPQHV
QQWNVEKVELDKNKRFKDVEVVRYFWHSLEKELISRGYR
11110000000000000000000000000000000000000000000000000000000000000000000000000000000
000000000000000000000000000000000000000000000000000000000000000000000000000000000
>Q08753_1dly_0_163
MMRTVQLRTLRPCIRAQQQPVRPSTSATAAAATAPAPARKCPSSLFAKLGGREAVEAAVDK
FYNKIVADPTVSTYFSNTDMKVQRSKQFAFLAYALGGASEWKGKDMRTAHKDLVPHLSDV
HFQAVARHLSDTLTELGVPPEDITDAMAVVASTRTEVLNMPQQ
11111111111111111111111111111111111111111100000000000000000000000000000000000000000
000000000000000000000000000000000000000000000000000000000000000000000000000000000
>Q4Q067_2ar1_0_163
MSRKRVRAEDIHYWLLKSEPHKFSIDDLAKQKTSPWDGVRNYAARNNMRAMSVGDKVLF
YHSNTKEPGVAGLAEVVRLAYDDFTALDKTSEYFDPKATKEKNPWKMVDVKFVARWDTV
LTLHELKSRRELQKMALFTQSRLSVQPVSASEYAYILRMNEEQQRK
11111100000000000000000000000000000000000000000000000000000000000000000000000000000
000000000000000000000000000000000000000000000000000000000000000000000000000000001
>Q9AGB1_1f1m_1_164
KGPNLTEISKKITESNAVVLAVKEVETLLTSIDELAKAIGKKIKNDVSLDNEADHNGSLMSG
AYLISTLITKKISAIKDSGELKAEIEKAKKCSEEFTAKLKGEHTDLGKEGVTDDNAKKAILKT
NNDKTKGADELEKLFESVKNLSKAAKEMLTNSVKELTSP
11000000000000000000000000000000000000000000000000000000000000000000000000000000000
000000000000000000000000000000000000000000000000000000000000000000000000000000000
>B7UTD4_3vhj_2_164
KNNLGVAVIGSKQYAVNLLWGSSQDTETTNQALNKSLTLMSSKLYSVIGRFQGEQFAVGDK
NIGHKRGQVTLLSAIDFDGSSFCGLFPADNELWLVIGVDKDGMVHFDKSFHSKDDAKKFFF
DHVAYGYPWDRTYSPSDVGVGESRSISELSLIKGKKLKEKG
00000000000000000000000000000000000000000000000000000000000000000000000000000000000
00000000000000000000000000000000000000000000000000000000000000000000000000000001
>P05656_4azz_514_676
TTPFMSNMTGWTTVNGTWADTIEGKQGRSDGDSFILSSASGSDFTYESDITIKDGNGRGAG
ALMFRSDKDAKNGYLANVDAKHDLVKFFKFENGAASVIAEYKTPIDVNKKYHLKTEAEG

DRFKIYLDDRLVIDAHDSVFSEGQFGLNVWDATAVFQNVTKES
10000000000000000000000000000000000000000000000000000000000000000000000000000000000
00000000000000000000000000000000000000000000000000000000000000000000000000000000
>P0ABL1_2ozy_25_187
ATPAAQASDDRYEVTQQRNPDAACLDCHKPDTEGMHGKHASVINPNNKLPVTCTNCHG
QPSPQHREGVKDVMRFNEPMYKVGEQNSVCMSCHLPEQLQKAFWPHDVHVTKVACASC
HSLHPQQDTMQTLSDKGRIKICVDCHSDQRTNPNFNPASVPLLKEQP
11111111111111111000000000000000000000000000000000000000000000000000000000000000000
00000000000000000000000000000000000000000000000000000000000000000000000000000111
>P0CL19_3sdw_0_162
MAATPLPPLRLAIACDDAGVSYKEALKAHLSDNPLVSSITDVGVTSTTDKTAYPHVAIQAA
QLIKDGKVDRALMICGTGLGVAISANKVPGIRAVTAHDTFSVERAILSNDAQVLCFGQRVIG
IELAKRLAGEWLTYRFDQKSASAQKVQAISDYEKKFVEVN
11111000000000000000000000000000000000000000000000000000000000000000000000000000000
00000000000000000000000000000000000000000000000000000000000000000000000000000000
>Q2RPE2_3fcn_0_162
MGMEHKTYEADLFVWCQQQADGLRALSRSRRDLPDDLDLEHIAEEIEDMGRSELREATSL
VRQICVRVIMAMSAPEAPDRARWRSEVVSWHNLLLDTITPGMIDRIDIGVIWRRAVSEAKA
ALIEINVAPQAGLSFQAPLPADHFLDEDFDYDATVARLGPTA
11100000000000000000000000000000000000000000000000000000000000000000000000000000000
00000000000000000000000000000000000000000000000000000000000000000000000000000011
>Q62E64_3e99_0_162
MKTIDLADIQAFLYRESRLLDDKAWDAWLDCYRADAVFWMPSWDDADALVTDPQREISLI
YYPNRQGLEDRVFRIKTERSSATVPDTRTSHNIANVERESADGDVHTVRFNWHTLSYRYKT
VSSYFGMSRYAIDFSGDAPKIVSKYVVLKNDYINQLIDIYHI
00000000000000000000000000000000000001111111111100000000000000000000000000000000000
00000000000000000000000000000000000000000000000000000000000000000000011100000000
>Q9NQR1_1zkk_230_392
SSRKSKAELQSEERKRIDELIESGKEEGMKIDLIDGKGRGVIATKQFSRGDFVVEYHGDLIEIT
DAKKREALYAQDPSTGCYMYYFQYLSKTYCVDATRETNRLGRLINHSKCGNCQTKLHDID
GVPHLILIASRDIAAGEELLYDYGDRSKASIEAHPWLKH
11000000000000000000000000000000000000000000000000000000000000000000000000000000000
00000000000000000000000000000000000000000000000000000000000000000000000000000000
>Q9NRW4_1wrm_0_162
MGNGMNKILPGLYIGNFKDARDAEQLSKNKVTHILSVHDSARPMLEGVKYLCIPAADSPS
QNLTRHFKESIKFIHECRLRGESCLVHCLAGVSRSVTLVIAYIMTVTDFGWEDALHTVRAGR
SCANPNVGFQRQLQEFEKHEVHQYRQWLKEEYGESPLQDAE
00000000000000000000000000000000000000000000000000000000000000000000000000000000000
00000000000000000000000000000000000000000000000000000000000000000000000011111111
>Q9X9L0_1j3w_0_162
MVEPSLVLYGAPYERAVEVLEETLRETGARYALLIDRKGFVLAHKEALWAPKPPPLDTLATL
VAGNAAATQALAKLLGEARFQEEVHQGERMGLYVDEAGEHALLVLVFDETAPLGKVKLH
GKRAAEALARIAEEALANPPRLALDTEYREGAEALLDDLLRN
11111000000000000000000000000000000000000000000000000000000000000000000000000000000
00000000000000000000000000000000000000000000000000000001111111111111111111111111
>Q9Z214_1i2h_0_162
MGEQPIFSTRAHVFQIDPNTKKNWVPTSKHAVTVSYFYDSTRNVYRIISLDGSKAIINSTITP
NMTFTKTSQKFGQWADSRANTVYGLGFSSEHHLSKFAEKFQEFKEAARLAKEKSQEKMEL
TSTPSQESAGGDLQSPLTPESINGTDDERTPDVTQNSEPR
00000000000000000000000000000000000000000000000000000000000000000000000000000000000
00000000000000000000000000000000001100000000011111000000000011111111111111111111

>A4XDU4_3eby_0_161
MSVAAEVAQVAQSAIDDFNAAYGLCLDDDRLEQWPTLFVDDCLYQVIARENVDNGLPAA
VMYCDSKGMLADRVVALRKANVFPEHFNRHLIGRAVITGVEGDQVSAEASYVVFQTRND
GETRIYNAGKYVDRFDLSGGTVRLKSRTCIYDTLRIATLLATPI
11111000000000000000000000000000000000000000000000000000000000000000000000000000111
1000000000000000000000000000000000000000000000000000000000000000000000000000000
>O35430_4dbb_452_642
DPEDLIDGIIFAANYLGSTQLLSDKTPSKNVRMMQAQEAVSRIKPEGESQPMTEVDLFISTQR
IKVLNADTQEPMMDHPLRTISYIADIGNIVVLMARRRMPRSQYKMICHVFESEDAQLIAQSI
GQAFSVAYQEFLRANGINPEDLSQKEYSDLLNTQDMY
11000000000000000000000000000000000000000000000011111000000000000000000000000000000
0000000000000000000000000000000000000000000000000000011000000000000000000000111
>O73909_2wry_105_266
APAFRYTRSQSFDIFDINQKCFVLESPTQLVALHLQGPSSSQKVRLNIALYRPRGPRGSAGTG
QMPVALGIKGYKLYMSCVMSGTEPTLQLEEADVMRDIDSVELTRFIFYRLDSPTEGTTRFESA
AFPGWFICTSLQPRQPVGITNQPDQVNIATYKLSGR
11000000000000000000000000000000000000000000000001111100000000000000000000000000000
0000000000000000000000000000000000000000000000000000000000000000000000000000000
>O88188_3m7o_0_161
MNGVAAALLVWILTSPSSSDHGSENGWPKHTACNSGGLEVVYQSCDPLQDFGLSIDQCSK
QIQSNLNIRFGIILRQDIRKLFLDITLMAKGSSILNYSYPLCEEDQPKFSFCGRRKGEQIYYAGP
VNNPGLDVPQGEYQLLLELYNENRATVACANATVTSS
11111111111111111111111100000000000000000000000000000000000000000000000000000000000
0000000000000000001111110000000000000000000000000000000000000000000000000000000
>P04893_3p9a_0_161
MAAPKGNRFWEARSSHGRNPKFESPEALWAACCEYFEWVEANPLWEMKAFSYQGEVIQE
PIAKMRAMTITGLTLFIDVTLETWRTYRLREDLSEVVTRAEQVIYDQKFSGAAADLLNANII
ARDLGLKEQSQVEDVTPDKGDRDKRRSRIKELFNRGTGRDS
11111100000000000000000000000000000000000000000000000000000000000000000000000000000
0000000000000000000000000000000000000000000000000000000001111111111111111111111
>P0DJO8_3fdq_0_161
MPKSEIRKLLQEIKKQVDNPGNSSTTEIKKMASEAGIDEQTAEEIYHLLTEFYQAVEEHGGIE
KYMHSNISWLKIELELLSACYQIAILEDMKVLDISEMLSLNDLRIFPKTPSQLQNTYYKLKKE
LIQVEDIPKNKPGRKRKTQKNTKKEKTNIFGKVVPA
11100000000000000000000000000000000000000000000000000000000000000000000000000000000
0000000000000000000000000000000000000000000000000000000000000111111111111111111
>P42305_2hjv_206_367
AAGLTTRNIEHAVIQVREENKFSLLKDVLMTENPDSCIIFCRTKEHVNQLTDELDDLGYPCD
KIHGGMIQEDRFDVMNEFKRGEYRYLVATDVAARGIDIENISLVINYDLPLEKESYVHRTGR
TGRAGNKGKAISFVTAFEKRFLADIEEYIGFEIQKIEA
11110000000000000000000000000000000000000000000000000000000000000000000000000000000
0000000000000000000000000000000000000000000000000000000000000000000000000000000
>P45774_4dq9_32_193
SVKDEAKISAQSFYQRLLLLNEEAILSGQDFGVRIDVDTRRLTFLQLTADKGWQKWQNDK
MTNQTTLKEGLQLDFELGGGAWQKDDRLFNPGSLFDEEMFADEKKEQKQEPAPQLFVLSS
GEVTPFTLSIFPKGQEPDEQWRVTAQENGTLRLLAPGESDEE
00000000000000000000000000000000000000000000000000000000000000000000000000000000000
0000000000000000000111111000000000000000000000000000000000000000000000000000011
>Q42831_2oif_0_161
MSAAEGAVVFSEEKEALVLKSWAIMKKDSANLGLRFFLKIFEIAPSARQMFPFLRDSDVPLE
TNPKLKTHAVSVFVMTCEAAAQLRKAGKITVRETTLKRLGGTHLKYGVADGHFEVTRFAL

LETIKEALPADMWGPEMRNAWGEAYDQLVAAIKQEMKPAE
11111111000000000000000000000000000000000000000000000000000000000000000000000000000
0000000000000000000000000000000000000000000000000000000000000000000000000000011
>Q57997_1mjh_0_161
MSVMYKKILYPTDFSETAEIALKHVKAFKTLKAEEVILLHVIDEREIKKRDIFSLLLGVAGLN
KSVEEFENELKNKLTEEAKNKMENIKKELEDVGFKVKDIIVVGIPHEEIVKIAEDEGVDIIIM
GSHGKTNLKEILLGSVTENVIKKSNKPVLVVKRKNS
11000000000000000000000000000000011111111111111110000000000000000000000000000000000
0000000000000000000000000000000000000000000000000000000000000000000000000000000
>Q58855_2oso_0_161
MAFMEKIFPDILEAIRNEEIIKESKKIPMPYFGLFALVIFDKVKELGSETSLYEIGEEFGKMLSPK
NIEELKKIFKLMNFGDLEIDENKILLKNPPYKIKLSNPPYQWVSKEEPIHDFIAGILAGCLEEI
FKKKFVVNEVECVSQGKDKCVFEVKEVDELNK
00000000000000000000000000000000110000000000000000000000000000000000000000000000000
0000000000000000000000000000000000000000000000000000000000000000000000000001111
>Q59250_1gpr_0_161
MIAEPLQNEIGEEVFVSPITGEIHPITDVPDQVFSGKMMGDGFAILPSEGIVVSPVRGKILNVF
PTKHAIGLQSDGGREILIHFGIDTVSLKGEGFTSFVSEGDRVEPGQKLLEVDLDAVKPNVPSL
MTPIVFTNLAEGETVSIKASGSVNREQEDIVKIEK
11100000000000000000000000000000000000000000000000000000000000000000000000000000000
0000000000000000000000000000000000000000000000000000000000000000000000000000001
>Q5MWV9_3d9x_374_535
KALRGMISEKGGWNLTVNNDNNTVVSSGGALDLSSGSKNLKIVKDGKKNNVTFDVARDL
TLKSIKLDGVTLNETGLFIANGPQITASGINAGSQKITGVAEGTDANDAVNFGQLKKIETEV
KEQVAASGFVKQDSDTKYLTIGKDTDGDTINIANNKSDKRT
11111111110000000000000000000000000000000000000000000000000000000000000000000000000
0000000000000000000000000000000000000000011111111111111111111111111111111111111
>Q747F6_2w31_0_161
MLTMQEIKAHYRFTDEDAELLGSLFPLAETNKERLADQFYDYLLGIPETAEFLKEDLVLQKL
KQTHQDWFVSLFAGSYDNRYIHNLQKIGHAHVRVGLNAHYVNVAMNVVRQFTLSIIQDN
FPDPEERRQRREAVEKILDINLDIMSASYREEEMRKFFVSH
00000000000000000000000000000000000000000000000000000000000000000000000000000000000
0000000000000000000000000000000000000000000000000000000000000000000001111111111
>Q74EF5_3eea_7_168
LIEGSERLSGLTDVDEVIKDLSRLLRKLVKTRWIAVYFFDRERRDFAPARSTGLPASFLPVFRE
MPLAPDKIPLLKSMLRKRQHLMLTDPGSSDLLTPKLRKLLRNLCVLAVPMVVRTQVIGAVF
MARTRDNPPFSDAETAIIRDLVSHAALVVSHMQLFDE
11111000000000000000000000000000000110000000000000000000000000000000000000000000000
0000000000000000000000000000000000000000000000000000000000000000000000000000000
>Q74Y93_3n4j_0_161
MLNIVLFEPEIPPNTGNIIRLCANTGCQLHLIKPLGFTWDDKRLRRAGLDYHEFADIKHHH
DYQAFLDSEKLDSTQPARLFALTTKGTPAHSAVSYQANDYLLFGPETRGLPAYILDALPAQQ
KIRIPMQADSRSMNLSNAVSVVVYEAWRQLGYPGALLKE
00000000000000000000000000000000000000000000000000000000000000000000000000000000000
[truncated: 355,647 more chars]
